# Does prenatal alcohol exposure cause a metabolic syndrome? (Non-)evidence from a mouse model of fetal alcohol spectrum disorder

**DOI:** 10.1371/journal.pone.0199213

**Published:** 2018-06-28

**Authors:** Robyn M. Amos-Kroohs, David W. Nelson, Timothy A. Hacker, Chi-Liang Eric Yen, Susan M. Smith

**Affiliations:** 1 UNC Nutritional Research Institute and Department of Nutrition, University of North Carolina-Chapel Hill, Kannapolis, North Carolina, United States of America; 2 Department of Nutritional Sciences, University of Wisconsin-Madison, Madison, WI, United States of America; 3 Cardiovascular Research Center, Department of Medicine, University of Wisconsin School of Medicine and Public Health, Madison, Wisconsin, United States of America; University of Kentucky, UNITED STATES

## Abstract

Although prenatal alcohol exposure (PAE) reduces offspring growth, it may increase obesity risk at adolescence. Animal models of PAE display glucose intolerance and increased adiposity, suggesting that PAE causes metabolic reprogramming. We tested this hypothesis in a mouse model of binge PAE, wherein pregnant C57Bl/6J females received 3 g/kg alcohol (ETOH) daily from gestational day 12.5 to 17.5; maltodextrin (MD) and medium chain triglycerides (MCT) served as isocaloric nutritional controls, and sham (H2O) treatment controlled for gavage stress. Our comprehensive assessment quantified body composition, energy expenditure, glucose tolerance, and cardiovascular function in offspring at age 17 weeks. Although ETOH pups were initially lighter than all other groups, they did not have a unique obesogenic phenotype. Instead, a similar obesogenic phenotype emerged in all three caloric groups (MCT, MD, ETOH), such that caloric groups had greater post-weaning weight gain (both sexes), reduced gonadal fat weight (males), and reduced glucose clearance (males) compared against H2O offspring. PAE did not affect body composition, respiratory exchange ratio, metabolic adaption to high-fat or low-fat diet, eating behavior, and blood pressure, and ETOH values did not differ from those obtained from isocaloric controls. Exposure to a higher alcohol dose (4.5 g/kg) or a high-fat (60%) diet did not exacerbate differences in body composition or glucose tolerance. “PAE-specific” effects on postnatal growth, glucose tolerance, adiposity, or hypertension only emerged when PAE offspring were compared just against H2O controls, or against MD controls. We conclude that prior reports of obesity and glucose intolerance in adult PAE offspring reflect the contribution of added gestational calories, and not alcohol’s pharmacologic action. Results suggest that the increased adiposity risk in FASD is not caused by metabolic reprogramming, and instead originates from behavioral, medication, and/or dietary practices. This study highlights the importance of appropriate dietary controls in nutritional studies of PAE.

## Introduction

Prenatal alcohol exposure (PAE) is a leading cause of neurodevelopmental disability and affects 2.5% - 4.5% of children [1]. PAE may increase chronic disease risk in later life [2]. PAE is associated with small-for-gestational age (SGA) [3–6], and because SGA itself significantly increases risk for childhood obesity [7], this has prompted suggestions that PAE may be an independent risk factor for obesity and metabolic syndrome in later life. Evidence in support of this hypothesis is contradictory. Heavy prenatal exposure is associated with persistent reductions in growth and BMI [4–6, 8], whereas more moderate exposures are associated with catch-up growth [4, 6, 9–12]; however, it is unknown if this catch-up growth represents a gain of lean mass or instead the accrual of adipose tissue, and both would increase weight and BMI in previously underweight individuals. Several studies document reduced fat mass in children who experienced PAE [4, 8], while others report an elevated incidence of overweight and obesity at adolescence and especially in females [12–15]. Although data are lacking for adults with FASD, increased adiposity elevates risk for cardiovascular disease, type-two diabetes, and other obesity-associated chronic health problems which may signify an underlying metabolic disorder.

Animal models support the hypothesis that PAE may cause metabolic dysregulation in the offspring. In a guinea pig model, the “catch-up” growth of PAE offspring represents excessive visceral fat deposition [16]. A rat PAE model shows similar catch-up growth in both sexes [17–19] and greater adiposity in response to high-fat diet challenge as compared with water-gavage [20] or carbohydrate-fed controls [21, 22]. Glucose metabolism is altered and PAE offspring have elevated gluconeogenesis, altered lipid profiles, and impairments of glucose handling, insulin signaling, and GLUT4 expression that reduce glucose clearance [17– 20, 22–28]. These findings suggest that PAE may alter the offspring’s metabolic programming and that this phenotype may be unmasked by a caloric-rich dietary environment [29–31].

Increases in obesity risk may also be linked to the neurobehavioral deficits associated with FASD. Recent studies document poor nutrition, and disordered feeding and mealtime behaviors in children with PAE [12, 13, 32, 33]. Deficits in self-regulation can affect eating behavior and food choice [34–36]. Children with PAE are hyperphagic and seem to prefer calorie-dense foods [12, 32, 33], which can increase obesity risk. These children are also frequently medicated with drugs that alter weight and appetite, including anti-psychotics and ADD/ADHD-associated stimulants. Thus, factors related to self-control and eating behavior, as well as metabolic reprogramming, could influence obesity and chronic disease risk in individuals with PAE.

Although PAE research typically emphasizes alcohol’s pharmacologic actions, alcohol is also a caloric source and provides seven kilocalories/gram. Thus, gestational models of alcohol exposure may be comparable with gestational models of caloric overconsumption. Although some studies control for alcohol’s caloric impact through isocaloric substitution of carbohydrate, alcohol and carbohydrate have very different metabolic consequences. Unlike carbohydrate, alcohol does not elicit an insulin response and instead is metabolized similar to fat through oxidation to acetate, which is converted to ketones or enters the TCA Cycle as acetyl-CoA. Studies that assess the metabolic consequences of PAE inconsistently control for its caloric contribution. Some studies ignore calories and use water gavage (e.g., [17–20, 25–28]), whereas others use carbohydrate [16, 21, 24] or incompletely-described isocaloric liquid diets [22, 23]. This variability may partially account for inconsistencies within the literature regarding obesity risk and metabolic defects alterations in response to PAE.

Here, we address these inconsistencies and test the hypothesis that PAE causes metabolic dysregulation in the offspring consistent with fetal metabolic reprogramming. We utilize indirect calorimetry, whole body imaging, and glucose tolerance testing to comprehensively interrogate how PAE affects offspring metabolism, obesity risk, and feeding behavior. We address this in a mouse model of FASD that targets alcohol exposure to the post-morphogenetic period of fetal growth that was previously shown to be vulnerable to metabolic reprogramming [27, 30, 37]. Additionally, we address a potential experimental design confound by including multiple macronutrient isocaloric controls, and thereby isolate the pharmacological effects of alcohol from its caloric contribution.

## Materials and methods

### Mouse model of chronic binge PAE

**Fig 1:**
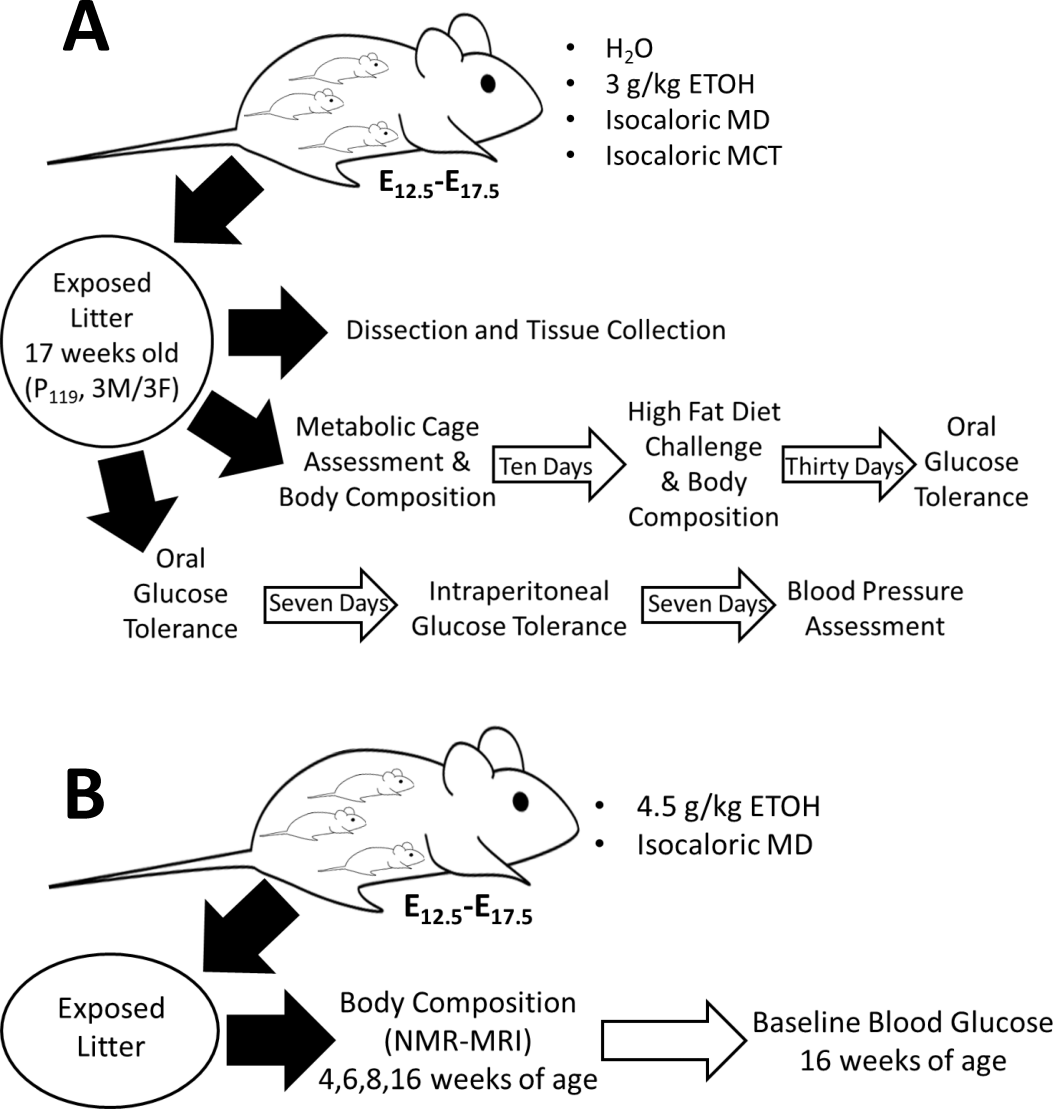
Experimental design for metabolic assessment in alcohol-exposed offspring. (A) Primary study design. Prenatal gavage treatment of timed pregnancies began at E12.5 and continued daily through E17.5 using three controls (H2O, isocaloric MCT, or isocaloric MD) or a 3 g/kg ETOH dose. Offspring developed without interference until seventeen weeks of age, when male/female offspring pairs within each litter were randomized into one of three assessment arms: (1) Tissue collection at 17 weeks (n = 8–10 mice per treatment*sex group); (2) Body composition and metabolic cage assessment, followed by a thirty-day high fat (60%) diet challenge and oral glucose tolerance testing (n = 10–12 mice per treatment*sex group); (3) oral and intraperitoneal glucose tolerance testing, followed by blood pressure assessment (n = 8–10 mice per treatment*sex group). (B) A separate group of pregnant dams were gavaged with either 4.5 g/kg dose or isocaloric MD control as in (A). Body composition of offspring was assessed at 4, 6, 8, and 16 weeks of age. Fasting blood glucose was evaluated at 16 weeks.

All experiments were approved by the University of Wisconsin-Madison IACUC. C57Bl/6J mice (Jackson Lab, Bar Harbor, ME) were pair-housed in a temperature-controlled room with a 12-h light/dark cycle. A total of 60 dams and 5 breeder males were used to generate up to 12 offspring in each sex*treatment group for each investigated outcome. Thirteen-week-old female and male C57Bl/6J mice were mated overnight; the morning of plug detection was denoted embryonic day (E) E0.5. Plugged dams were randomized by weight to the four different treatment groups in cohorts of twenty-four, assigning every fourth pregnant dam to PAE treatment. Beginning on E12.5, pregnant dams were gavaged with 3.0 g/kg ETOH (prepared with 200 proof, USP grade, 7.0 kcal/g; Decon Labs) administered daily as two half-doses of 1.5 g/kg ETOH (10 ml/kg body weight) given two hours apart on E12.5 through E17.5. This dosing period targeted fetal growth and organ maturation and was chosen to avoid ETOH’s negative effects on early organogenesis, and because exposure during this period was previously shown to alter the offspring’s obesity risk and glucose metabolism [27, 30, 37]. Control dams received isocaloric maltodextrin (MD, Lo-Dex 10, 93.5% α(1,4) glucose_4-20_ polymer, 4.0 kcal/g; Teklad, Madison WI), isocaloric medium chain triglycerides (MCT, 60/40 C8:C10 oil, 8.3 kcal/g; Jedwards International, Braintree MA), or equivalent volume of water as a negative caloric control (H2O). Water [17–20] and carbohydrate [16, 21–24] were chosen for comparative purposes against studies employing those controls, and MCT was chosen as a closer metabolic equivalent to alcohol, due to its rapid and direct metabolism that is independent of lipoprotein. Dams and offspring consumed breeder chow (#8626, Teklad; 20.6 kcal% protein, 10.4 kcal% fat, 43.4 kcal% carbohydrate), and all dams, regardless of treatment, received 1mg peanut butter daily from E12.5 to postnatal (P) 7 as a nutritional and cage enrichment. Offspring were group-housed except during the indirect calorimetry assessment, when they were single-housed. All four groups are referred to as ‘prenatal treatment’ in the text, and ‘added-calorie’ groups refer to MCT, MD, and ETOH groups only. At 17 weeks of age, litters were randomized into three testing arms; each arm contained a sex-matched pair from the same litter (Fig 1A). The first sex-matched pair from each litter was dissected to quantify body organ size; data are presented as a percentage of body weight for comparison purposes among groups. The second sex-matched pair in each litter underwent body composition analysis and metabolic assessment in response to diet challenge. The third sex-matched pair in each litter was evaluated for glucose metabolism and cardiovascular function.

### Assessment of whole body metabolism and diet challenge

Body composition of offspring pair two was assessed at age 17 weeks using dual-energy x-ray absorptiometry (DXA), followed by whole body metabolic assessment using indirect calorimetry (TSE Systems, Inc., Germany) [38, 39]. Animals were individually housed in the environmental chambers, during which time we quantified their metabolic adaptation to sequential consumption of *ad libitum* chow (#8626), low-fat diet (10% calories from fat, TD.06416, Teklad; S1 Table), and high-fat diet (60% calories from fat, TD.06414, Teklad), each for three consecutive days. Oxygen consumption, carbon dioxide production, and food and water ingestion were continuously quantified, and cyclic feeding behavior, energy expenditure (EE), and respiratory exchange ratio (RER) were calculated from these parameters. Before starting each new diet, body composition was re-evaluated using NMR (EchoMRI 4-in-1-1100 Analyzer; Houston, TX). At the end of the 10-day metabolic testing period, animals consumed the high-fat diet for an additional 28 days, after which we quantified body composition using NMR, and oral glucose tolerance responses as described below.

### Oral and intraperitoneal glucose tolerance testing

We evaluated glucose metabolism in offspring pair three using a protocol adapted from [40]. At age 17 weeks, we performed an oral glucose tolerance test (OGTT, 200 μL of 10% glucose in H2O), followed one week later by an intraperitoneal-injected glucose tolerance test (IPGTT, 10 μl/g of body weight, 10% glucose in PBS). For both, animals were fasted five hours, and then blood glucose was quantified using a hand-held glucometer (OneTouch Ultra, LifeScan, Inc., Milpitas, CA) immediately before and at defined intervals after glucose administration. Blood insulin was quantified at time zero and fifteen minutes post-glucose challenge using an established ELISA (Crystal Chem, Inc., Downers Grove, IL).

### Heart rate and blood pressure evaluation

After glucose tolerance testing, mice in group three underwent arterial pressure monitoring to quantify heart rate and blood pressure [41]. Mice were anesthetized with urethane (1000mg/kg, IP) and placed on a heated pad. The right common carotid artery was isolated and a 1.2 Fr high-fidelity pressure catheter (Science Inc, Transonic) was inserted and advanced to the aorta until the pressure stabilized, and then was advanced to the left ventricle. The pressure tracing was recorded and analyzed on commercially available software (Notocord, Croissy Sur Seine, France).

### High-Dose alcohol experiment

To investigate a potential dose-dependent effect of PAE, a separate cohort of pregnant C57Bl/6J dams was gavaged with 4.5 g/kg ETOH administered daily as two half-doses of 2.25 g/kg ETOH (10 ml/kg body weight) given four hours apart on E12.5 to E17.5 (Fig 1B). Isocaloric MD was the only control in this experiment and nutrient enrichment was not given. Body composition of these offspring was assessed at 4, 6, 8 and 16 weeks of age using NMR. At age 18 weeks, mice were fasted for five hours and blood glucose was quantified.

### Statistical analysis

Data were analyzed using mixed linear factorial analysis of variance in a randomized block design (ANOVA; Proc Mixed, SAS v9.4, SAS Institute, Cary, NC). A randomized block design was selected to account for litter effects because variance is generally lower within than between litters. Metabolic and glucose tolerance analyses had at least ten adult animals per sex*treatment group, while other adult analyses had at least eight. Weights and weight gain had greater than twenty per sex*treatment group. Between-subject factors were treatment (H_2_O vs. MCT vs. MD vs. ETOH). Significant interactions were further analyzed using slice-effect ANOVAs with *a priori* hypotheses allowing planned comparisons between ETOH and MD groups. Significance was *p* ≤ 0.05. Data analyzed by mixed models are presented as least square means ± SEM for purposes of inference [42].

## Results

### PAE does not affect gestational weight gain

**Fig 2:**
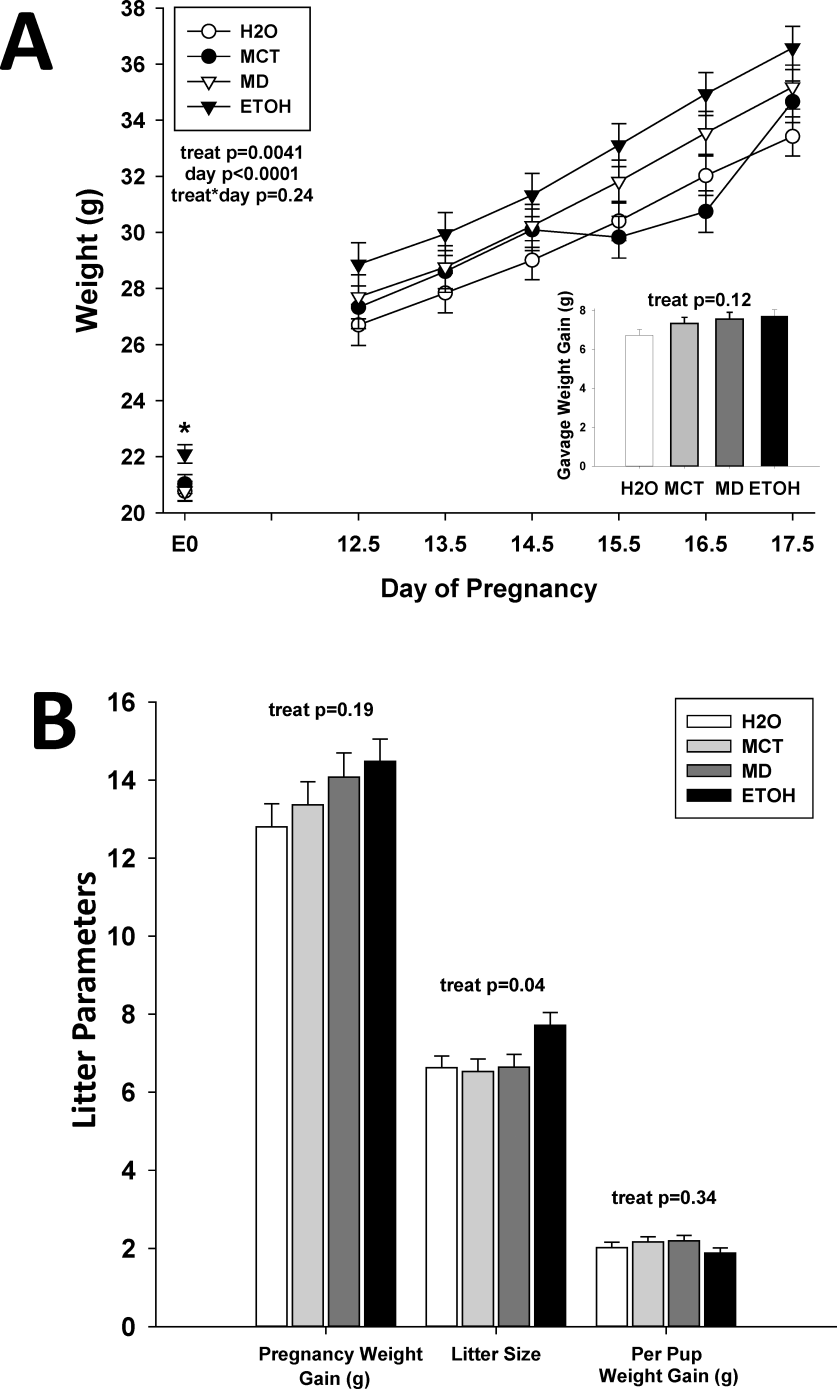
Gestational alcohol exposure does not adversely affect maternal growth and litter size. (A) Maternal weight during the gavage period. Due to a partial randomization of treatment, ETOH dams were initially significantly heavier than other dams at E0.5 (p<0.05) and during the dosing period (p<0.01). However, treatment did not affect weight gain during the gavage period (inset, p>0.1), suggesting a lack of effect by alcohol. (B) Litter parameters at E17.5. Total maternal weight gain from E0.5 to E17.5 did not significantly differ across treatment groups, although ETOH dams gained the most weight. This weight gain likely represented the significant increase in ETOH litter size, because weight gain per pup was not affected by treatment. Values are mean ± SEM of 12 dams per treatment group. * p<0.05 vs. H2O, using mixed linear factorial analysis of variance, followed by slice-effect ANOVAs with *a priori* hypotheses allowing for planned comparisons.

ETOH dams weighed more than MD females (22.10 ± 0.33 g vs. 20.77 ± 0.35 g, p<0.01) at E0.5, and were significantly heavier than MCT and H2O females (Fig 2A, F(3,48) = 3.65, p <0.05). This reflected that pregnant mice were generated sequentially with ETOH treatment assigned to every fourth pregnancy. Blood alcohol concentration in the ETOH group averaged 110 mg/dL two hours after the second half-dose on E12.5. Although ETOH females remained heavier (F(3,83.1) = 4.60, p <0.01) than MCT and H2O dams, but not MD dams, treatment did not affect maternal weight gain during the gavage period (Fig 2A, p = 0.24). Added-calorie groups (MCT, MD, ETOH) showed a non-significant trend to greater weight gain during the gavage period (Fig 2A inset, p = 0.12) and over the entire pregnancy (Fig 2B, p = 0.19) than did H2O dams. ETOH dams had larger litter sizes compared with other treatment groups (Fig 2B, F(3,55) = 2.93, p <0.05), but per-pup weight gain was unaffected by treatment (Fig 2B, p = 0.34), suggesting that PAE did not reduce fetal growth.

### PAE alters offspring growth and body weight

**Fig 3:**
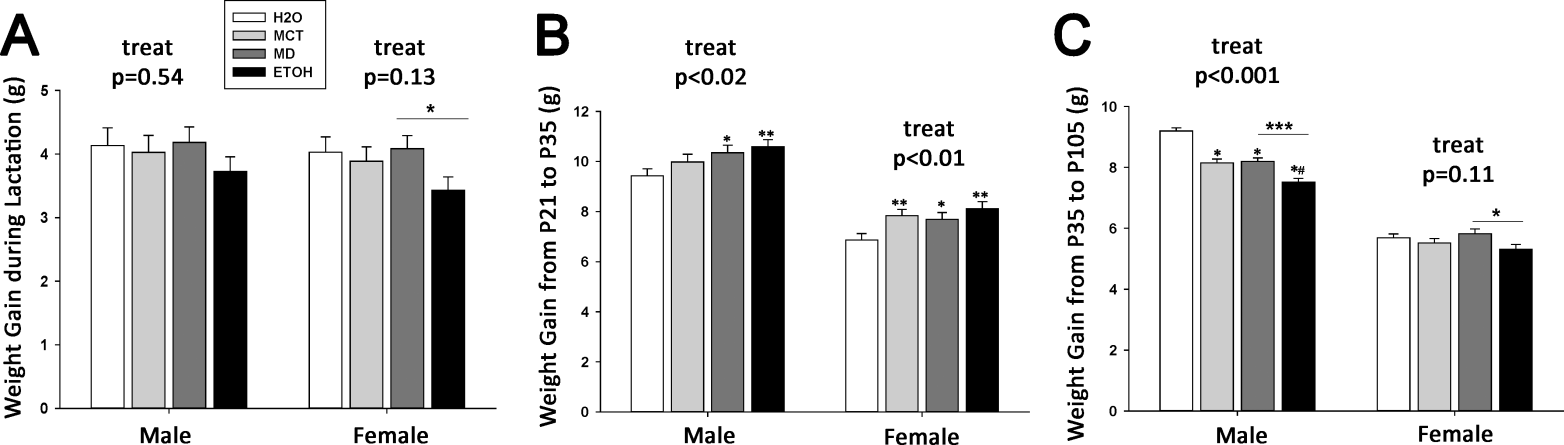
Prenatal treatment differentially affects offspring weight and weight gain over the lifespan. (A) During the lactation period, ETOH offspring gained less weight than did other groups, and this decreased weight gain was significant in female ETOH offspring in comparison with MD controls (p<0.05). (B) During the juvenile period (P21-P35), both male and female ETOH offspring gained significantly more weight in comparison to other groups (M: p<0.02; F: p<0.01). (C) During adolescence and adulthood (P35-P105), ETOH offspring gained less weight than other groups (M: p<0.0001 vs all other groups; F: p<0.05 vs. MD), despite their greater weight gain during the juvenile period. Values are mean ± SEM of more than 20 per sex*treatment group. * p<0.05 vs. H2O, ** p<0.01 vs. H2O, *** p<0.001 vs. H2O, # p<0.05 vs. isocaloric control (MD or MCT, as indicated by bar), using mixed linear factorial analysis of variance, followed by slice-effect ANOVAs with *a priori* hypotheses allowing for planned comparisons.

During the lactation period (P7-P21), the average daily weights of male offspring were unaffected by treatment (p = 0.48) and, although a significant treatment x day interaction (Part A in S1 Fig, F(39,2217) = 2.92, p <0.0001) was indicated, post hoc analyses were not significant. In contrast, prenatal treatment affected lactational weight gain in females (insert, Part B in S1 Fig, F(3,167) = 2.65, p <0.05), and ETOH pups were significantly smaller than MCT and H2O females, and similar in size to MD females. ETOH females were significantly smaller by P12 and remained so until weaning (Part B in S1 Fig, F(39,2040) = 2.65, p <0.0001). Treatment did not affect total weight gain in males (Fig 3A, p = 0.54), whereas ETOH females gained significantly less weight than did MD females (Fig 3A, 3.43 ± 0.21 g vs. 4.08 ± 0.21 g, F(3,167) = 2.65, p<0.05); other comparisons were not significant (p = 0.13).

Although prenatal treatment did not affect absolute body weight in males (p = 0.17; Part C in S1 Fig) or females (p = 0.22; Part D in S1 Fig), it did affect post-weaning growth trajectory. During the juvenile period (P21-P35), ETOH and MD males gained significantly more weight than did H2O controls (Fig 3B, F(3,137) = 3.14, p <0.05), whereas all added-calorie groups gained more weight than H2O controls in females (Fig 3B, F(3,137) = 4.05, p <0.01). This growth pattern shifted as the animals approached adulthood (P36-P105). The added-calorie males gained less weight than did H2O controls (Fig 3C, F(3,1270) = 37.77, p <0.0001), and the ETOH females gained less weight than did MD females (5.32 ± 0.16 g vs. 5.82 ± 0.16 g, p<0.05); other growth comparisons within female offspring did not differ (Fig 3C, p = 0.11). Although prenatal treatment affected growth trajectory, by age 17 weeks when metabolic assessment began, body weights did not significantly differ between treatment groups for either sex.

### PAE does not affect adult body composition

**Fig 4:**
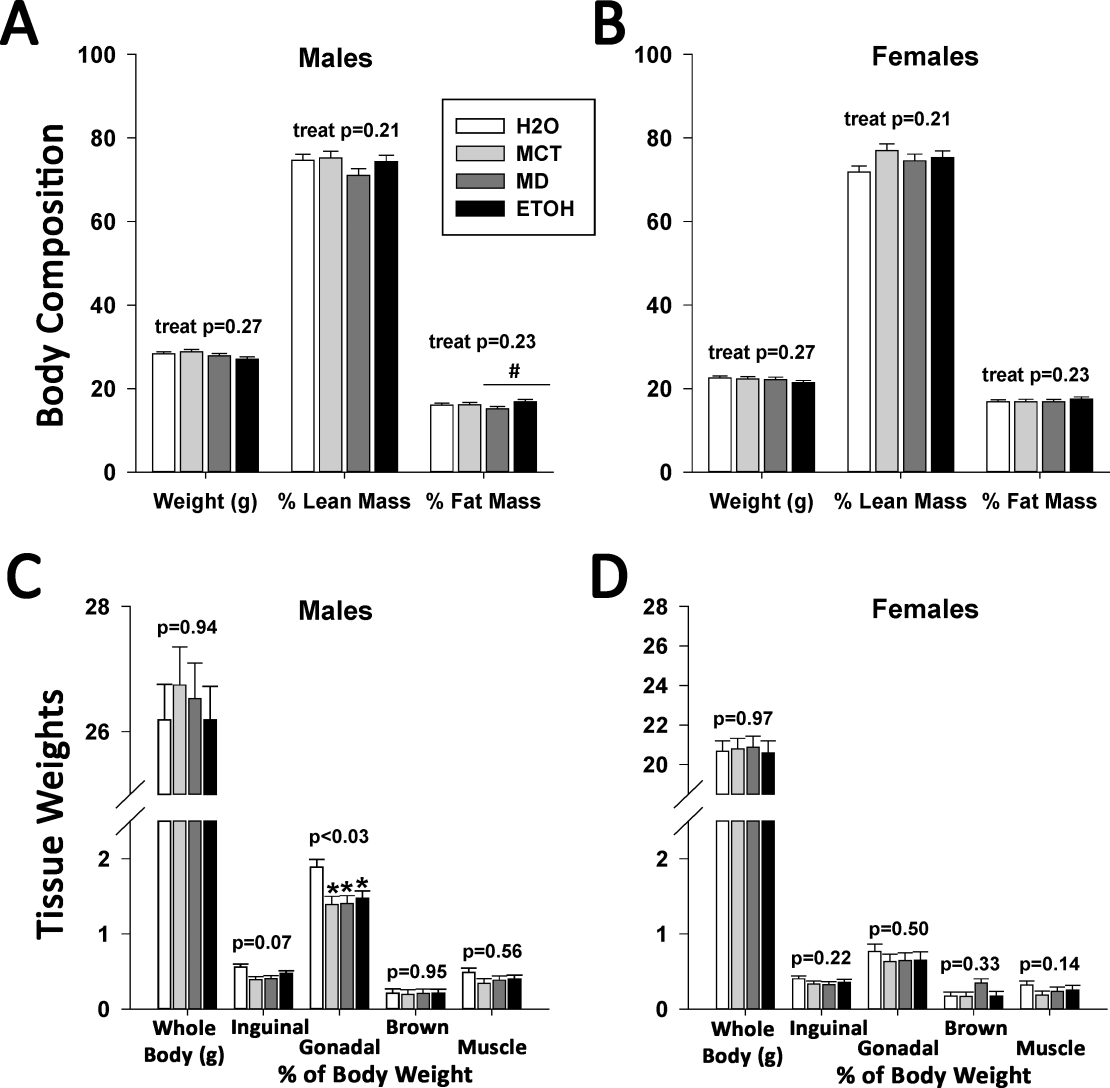
Isocaloric treatment, but not PAE, alters fat mass in adult male but not female mice. (A) Body composition (% lean mass, % fat mass) was quantified in control and alcohol-treated offspring using DXA. At age 17 weeks, male offspring had similar body weights and lean mass content, expressed as a percentage of body mass, regardless of prenatal treatment. However, ETOH males had significantly greater fat mass compared with MD males. (B) At age 17 weeks, female offspring had similar body weights, lean mass content, and fat mass content, expressed as a percentage of body mass, regardless of prenatal treatment. (C) In a separate cohort of males dissected for tissue collection at age 17 weeks, prenatal treatment did not affect total body weight, brown fat mass, and gastrocnemius mass, the latter two expressed as a percentage of body mass. However, male offspring in all three added-calorie groups (MCT, MD, ETOH) had a significantly decreased inguinal fat pad mass in comparison with H2O males (p<0.05). (D) In females, there were no ETOH or treatment effects upon body weight, or fat pad and gastrocnemius mass as a percentage of body weight. Values are mean ± SEM of 10–12 offspring per sex*treatment group. * p<0.05 vs. H2O, # p<0.05 vs. MD, using mixed linear factorial analysis of variance, followed by slice-effect ANOVAs with *a priori* hypotheses allowing for planned comparisons.

We used DXA to evaluate how PAE affected growth quality and the type of weight accrued during the growth progression. At age 17 weeks, bone mineral density (BMD) and bone mineral content (BMC) were unaffected by treatment in male (BMD p = 0.09, BMC p = 0.98; Part A in S2 Fig) and female offspring (BMD p = 0.08, BMC p = 0.75; Part B in S2 Fig). Although male ETOH and MD offspring had similar body weights, ETOH males had a greater fat mass percentage (16.82 ± 0.53% vs. 15.12 ± 0.55%, p<0.05, Fig 4A). There were no other treatment effects in these males with respect to body weight (p = 0.27), percent lean mass (p = 0.21), and percent fat mass (p = 0.23). For females, prenatal treatment did not affect body weight (Fig 4B, p = 0.27), percent lean mass (p = 0.18), or percent fat mass (p = 0.17). This suggested that PAE did not alter adult body composition when caloric influences were controlled for.

We complemented DXA analysis with direct measures of tissue adiposity. In males, both the gonadal (Fig 4C, F(3,28) = 3.34, p <0.05) and inguinal fat pads (p<0.07) from the added-calorie groups weighed less than those from the H2O group. Brown fat (p = 0.95) and gastrocnemius muscle (p = 0.56) weight did not differ. In females (Fig 4D), we found no significant differences in gonadal fat pad (p = 0.50), inguinal fat pad (p = 0.22), brown fat (p = 0.33), or gastrocnemius muscle (p = 0.14) weights.

We found a similar lack of treatment effects upon other organ weights (S2 Table). Male added-calorie groups had a trend to decreased brain mass (p<0.06), normalized to body weight, as compared to H2O males. This was not seen in females (p = 0.22). Splenic weights in both males (F(36,1551) = 2.60, p <0.001) and females (F(36,1551) = 2.60, p <0.001) differed significantly. The spleens of MCT males were significantly larger than MD and H2O male spleens. In females, it was the MD spleens that were significantly larger than both H2O and ETOH groups. Other organ weight comparisons, including kidney, did not differ.

### PAE affects resting heart rate but is not hypertensive

PAE is reported to be hypertensive at adulthood [43, 44], and we used arterial catheterization to quantify treatment-related effects on cardiac function. Heart rate in ETOH males was significantly increased in comparison with MD males (Part A in S3 Fig, p<0.05) and showed a trend to increase in ETOH vs MD females (p<0.09). Aortic systolic and diastolic blood pressure did not differ in either male (systolic: p = 0.30, diastolic: p = 0.63) or female offspring (systolic: p = 0.74, diastolic: p = 0.65), regardless of prenatal treatment (Part B in S3 Fig). With respect to cardiac performance, we found no significant treatment effects upon dP/dt_max_, the maximum rate of pressure change during left ventricular contraction (males: p = 0.11, females: p = 0.87; S3 Table). However, in males (F(3,26) = 4.40, p <0.01), but not females (p = 0.61), there was a significant difference in dP/dt_min_, which is the minimum rate of pressure change in the left ventricle, and MCT males had a significantly decreased rate of pressure change at ventricle relaxation in comparison with other treatments. There were no treatment effects upon absolute left ventricular pressures at maximum contraction (males: p = 0.41, females p = 0.72) and at peak relaxation (males: p = 0.22, females p = 0.48). We also calculated Tau, the isovolumic relaxation constant that reflects how quickly pressure decreases during the ventricle relaxation process, and again we found no treatment differences (males: p = 0.27, females: p = 0.40). Finally, prenatal treatment did not affect left ventricular ejection time (males: p = 0.41, females: p = 0.25). Thus, PAE in this exposure model did not affect cardiac function, nor was it hypertensive.

### PAE does not affect metabolic rate or food intake

**Fig 5:**
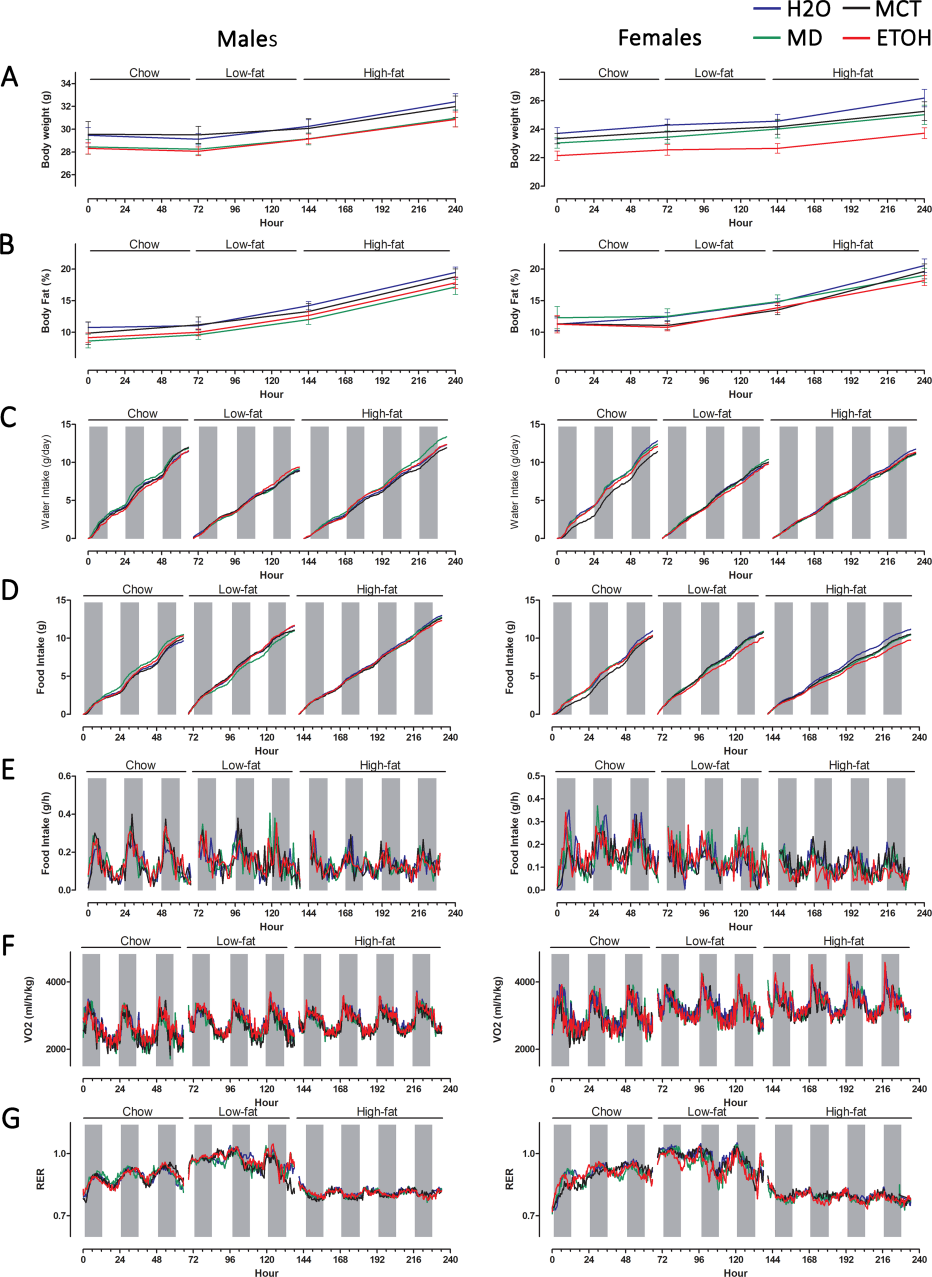
PAE does not alter whole body metabolism in adult male and female mice. Male and female offspring were evaluated for food intake and whole body metabolism using environmental chambers. The individual’s ability to adapt its metabolism and food intake to diet composition was assessed by sequential feeding of chow diet (3 days), low fat diet (10%, 3 days), and high fat diet (60%, 4 days). Body composition was assessed at the beginning and end of each diet challenge using NMR. (A) Body weight at the beginning and end of each diet challenge did not differ by treatment in males (left panel) and was reduced in ETOH females only during chow feeding. (B) The percentage of body size as fat mass increased on the low-fat and high-fat diets, but was not affected by PAE or other treatments in male and female offspring. (C) Cumulative water intake (measured hourly) was not affected by prenatal treatment in either sex. (D) Cumulative food intake increased on the low-fat and high-fat diets compared with chow diet, but did not differ with respect to prenatal treatment. (E) Hourly food intake showed similar patterns across the dark/light cycle in males, and was significantly affected by prenatal treatment in females, as detailed in the text. (F) Oxygen consumption (VO2, ml/h/kg) showed diurnal variation consistent with food consumption pattern and activity, and was unaffected by prenatal treatment in either sex. (G) The calculated respiratory exchange ratio (RER) showed diurnal variation consistent with the pattern of food intake and, as expected, was increased with the low-fat diet and decreased with the high-fat diet. RER was not affected by PAE or other prenatal treatments in either sex. Shaded bars represent the dark cycle when mice are more active. Values are mean ± SEM of 8–10 offspring per sex*treatment group; SEMs are omitted from panels C-G for clarity. Blue, H2O; Green, MD; Black, MCT; Red, ETOH.

To determine if the altered growth trajectory and elevated adiposity in ETOH offspring observed here and elsewhere [16 – 22] reflected a metabolic alteration, we quantified energy expenditure and metabolic adaptation to diet. Diet composition did not differentially affect body weight in male groups (p = 0.49) and all treatment groups had similar weight gains after feeding chow, low-fat, and high-fat diets (Fig 5A; S4 Table). In females, ETOH offspring (Fig 5A) initially weighed less than H2O and MCT females (treatment main effect: F(3,39) = 3.26, p <0.05), and this difference disappeared after feeding the experimental diets (S4 Table). The low-fat and high-fat diets did not further alter body composition, and PAE males (p = 0.99) and females (p = 0.34) accrued similar percentages of lean and fat mass across the testing period, as compared with controls (Fig 5B; S4 Table).

FASD affects feeding behavior and children with PAE have an increased incidence of hyperphagia and disordered eating [12, 32]. In this mouse model, PAE did not affect total food consumption (Fig 5D) or water intake (Fig 5C) in response to chow, low-fat, and high-fat feeding (S4 Table). However, PAE affected the pattern of eating in females, but not in males (Fig 5E, S4 Table), and a treatment effect was found with both chow (F(3,40) = 2.76, p <0.05) and high-fat diets (F(3,40) = 3.92, p <0.02). During the light cycle, chow consumption was significantly greater in both the ETOH (0.15 ± 0.01 grams/hour) and MCT females (0.14 ± 0.01 grams) compared with MD females (0.10 ± 0.01 grams/hour; p<0.02 vs. ETOH, p<0.03 vs. MCT); this difference disappeared during the dark cycle (p = 0.62). This flipped for the high-fat diet, and ETOH females ate less food than did H2O (p<0.01) and MCT (p<0.003), but not MD (p<0.08) controls during the dark cycle; there were no differences during the light cycle (p = 0.34). However, these changes were not associated with changes in body composition, as per above.

With respect to whole body metabolism, respiratory exchange ratio (RER) is the ratio of CO2 production to O2 consumption and indicates metabolic fuel choice. Normally, RER approaches 1.0 when consuming a low-fat (high-carbohydrate) diet and 0.7 when consuming a high-fat diet; thus, dietary challenge assesses metabolic adaptation to fuel choice. When fed a chow diet, PAE did not alter energy expenditure and there was no treatment-specific effect upon oxygen consumption (VO2; Fig 5F) or RER (Fig 5G) in either the light or dark cycles (S4 Table). This suggested that PAE did not affect metabolic rate. When fed a low-fat diet, PAE animals responded normally and RER increased similarly in both males and females across the diurnal cycle. With the high-fat diet, PAE animals again adapted normally and there was no effect of prenatal treatment or sex upon VO2 and RER. These data suggested that PAE did not affect metabolic rate or energy expenditure in the adult offspring.

### PAE does not worsen adiposity under high-fat diet intake

**Fig 6:**
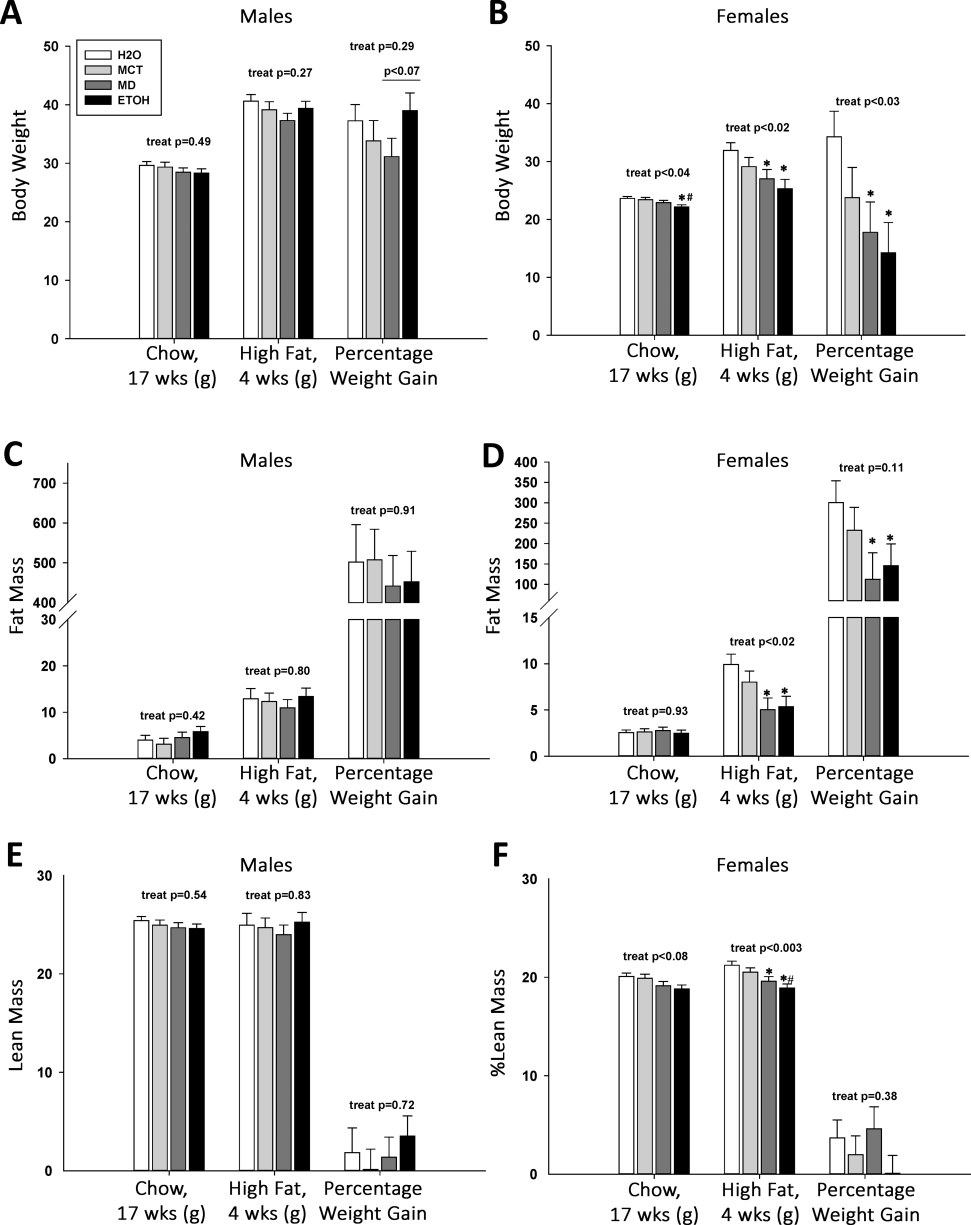
High-fat diet feeding does not unmask a unique adiposity phenotype in PAE offspring. Body composition was assessed by NMR in adult control and PAE mice, before and after they consumed a high-fat diet for 4 weeks. (A) High-fat diet intake caused significant weight gain in all male offspring, regardless of prenatal treatment. The weight gain percentage in ETOH males was higher than in MD males, but this was only a trend (p<0.07). (B) Chow-fed ETOH females weighed significantly less than all other groups at age 17 weeks. Consumption of a high-fat diet for four weeks increased female body weight regardless of prenatal treatment. However, ETOH and MD females weighed significantly less than their H2O counterparts (p<0.03), and their weight gain percentage was also decreased compared with H2O controls (p<0.03). (C) Fat mass significantly increased in males fed a high-fat diet, and this gain, as a percentage of weight, was not differentially affected by prenatal treatment. (D) Fat mass increased in females fed a high-fat diet, and this gain, as a percentage of body weight, was significantly less in MD and ETOH females (vs. H2O females, p<0.05). (E) Lean mass in male offspring was unaffected by high-fat diet or prenatal treatment. (F) Lean mass in female offspring was unaffected by high-fat diet or prenatal treatment. Values are mean ± SEM of 8–10 offspring per sex*treatment group. * p<0.05 vs. H2O, # p<0.05 vs. MCT, using mixed linear factorial analysis of variance, followed by slice-effect ANOVAs with *a priori* hypotheses allowing for planned comparisons.

We hypothesized that, as reported previously [20, 22], extended high-fat feeding would exacerbate the subtle obesity difference between ETOH and MD males and unmask a similar phenotype in females. In males, all treatment groups accrued similar gains in weight (p = 0.27; Fig 6A) and fat mass (p = 0.80; Fig 6C) in response to four weeks of *ad libitum* high-fat diet consumption. However, ETOH male mice appeared to gain a larger percentage of their body weight on high fat feeding, especially in comparison with MD (p<0.07), although not against other groups (H2O: p = 0.67; MCT: p = 0.27). Lean mass was unaffected by diet challenge (p = 0.83; Fig 6E).

In contrast, prenatal treatment affected how females responded to the high-fat diet. At the onset of high-fat feeding, treatment affected body weight (Fig 6B, F(3,40) = 3.20, p <0.05), and ETOH females were smaller than H2O (p<0.01) and MCT (p<0.03), but not MD (p = 0.17) females. Prenatal treatment affected response to high-fat feeding with respect to body weight (F(3,40) = 3.81, p <0.02) and percentage weight gain (F(3,40) = 3.43, p <0.03), such that ETOH (p<0.002) and MD females (p<0.02) remained significantly lighter than H2O females and gained less weight compared with H2O (ETOH, p<0.01; MD, p<0.02). Although body fat mass was equivalent (p = 0.93) between groups prior to diet challenge, at the end of high-fat feeding the ETOH and MD females had a lower percentage body fat than did H2O (Fig 6D, F(3,29) = 3.96, p <0.05), suggesting they had gained less fat mass per body weight (overall: p = 0.11). A trend (F(3,39) = 2.45, p <0.08) suggested that ETOH females also had less lean mass than H2O (p<0.02) prior to diet challenge, and this difference was significant after high-fat feeding (Fig 6F, F(3,29) = 6.03, p <0.003). Although ETOH had less lean mass percentage compared with H2O (p<0.0001) and MCT females (p<0.01), as did MD compared with H2O (p<0.02), lean mass gain did not differ (p = 0.38) and these differences instead reflected that the ETOH and MD females gained less weight on the high-fat diet, consistent with their reduced food intake (Fig 5D and 5E). Thus, in response to high-fat diet, PAE did not further increase adiposity risk for males and lessened that risk (as did MD) for females.

### Prenatal calories but not PAE worsens glucose tolerance in male offspring

**Fig 7:**
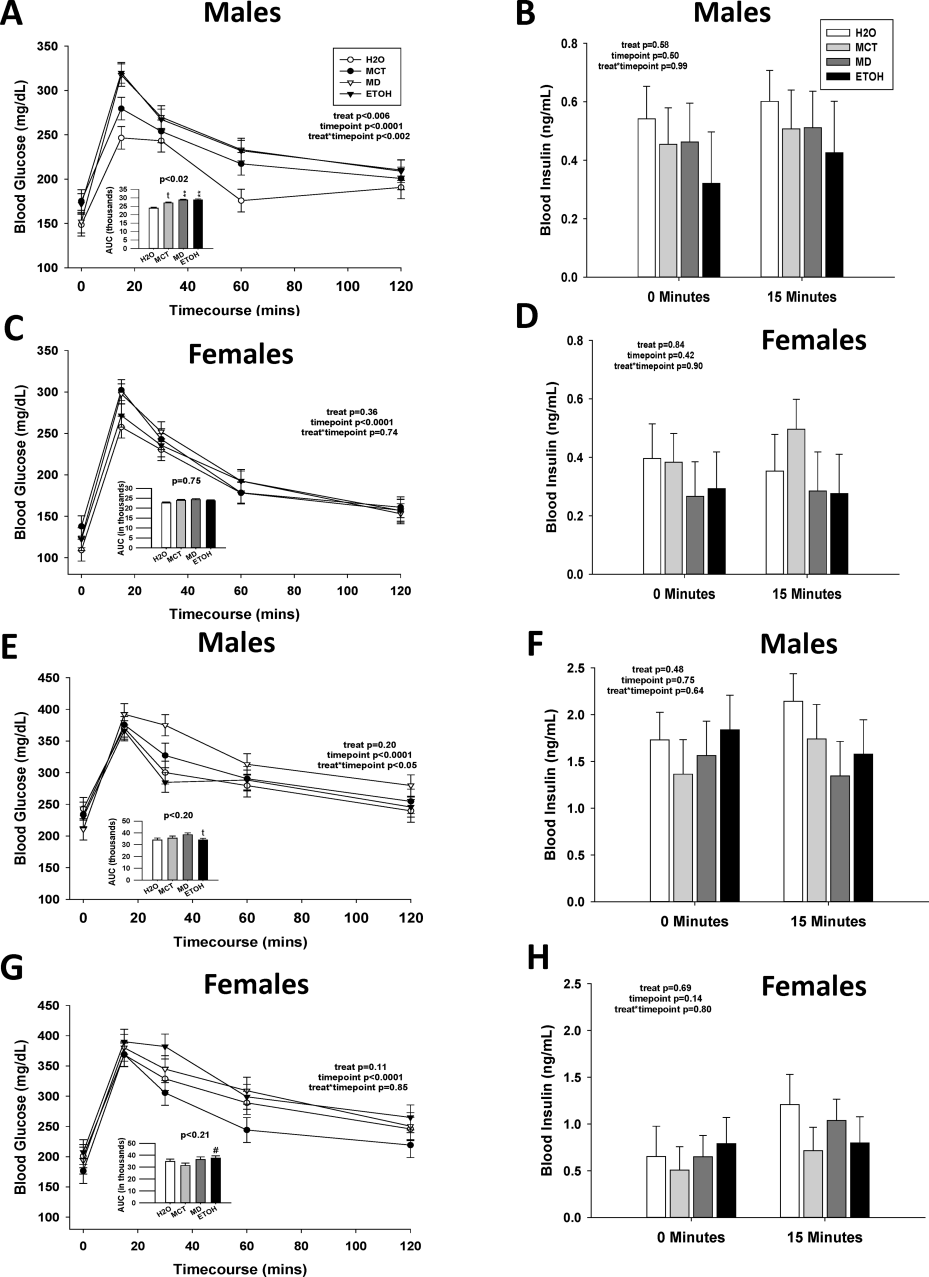
Prenatal calories, but not PAE, differentially impairs oral glucose tolerance in offspring. An oral glucose tolerance test (OGTT) was performed in 17-week-old PAE and control offspring (A-D), and again after they consumed a high-fat diet for four weeks. Blood glucose concentrations and insulin levels were assessed at times following an intragastric glucose bolus. (A) Male offspring receiving added gestational calories (MCT, MD, and ETOH) had higher blood glucose levels in response to oral glucose challenge as compared with H2O males. Males receiving PAE or isocaloric MD had significantly greater area-under-the-curve (AUC) values (inset) as compared with H2O-control males, and their AUC values did not differ from each other, suggesting that glucose clearance was reduced in those isocaloric groups. Males that received isocaloric MCT had a trend to elevated AUC compared with those that received H2O. (B) In these same males, PAE did not affect blood insulin levels as compared with H2O and isocaloric MD or MCT controls at either baseline fast or at 15 minutes post-glucose bolus. (C) PAE did not affect glucose clearance in female offspring as compared with H2O or isocaloric MD or MCT controls, nor did PAE alter the AUC for glucose clearance (inset). (D) PAE did not affect blood insulin levels in female offspring as compared with H2O and isocaloric controls at baseline fast and at fifteen minutes post-glucose bolus. (E) Consumption of a high-fat diet for four weeks elevated fasting blood glucose in PAE and control males. PAE did not further affect blood glucose or AUC values (inset) during the OGTT, as compared with controls. (F) In these males, high-fat diet consumption both elevated fasting insulin levels and their insulin levels at fifteen minutes post-glucose bolus. These levels were not further affected by PAE or prenatal treatment. (G) Consumption of a high-fat diet for four weeks elevated fasting blood glucose levels in PAE and control females. PAE did not further affect blood glucose or AUC values (inset) during the OGTT, as compared with controls. (H) In these same females, high-fat diet feeding elevated both fasting insulin and blood insulin levels at fifteen minutes post glucose bolus in all treatment groups. These values were not further affected by PAE or prenatal treatment. Values are mean ± SEM of 8–12 offspring per sex*treatment group. * p<0.05 vs. H2O, ** p < 0.01 vs. H2O, # p<0.05 vs. MCT, *t* p<0.1 vs. H2O, using mixed linear factorial analysis of variance, followed by slice-effect ANOVAs with *a priori* hypotheses allowing for planned comparisons.

Animal models of PAE exhibit impaired glucose tolerance [17–20, 22–27], and such changes can presage later adiposity changes. We found that prenatal treatment differentially affected glucose clearance in a sex-dependent manner. For 17-week-old males, treatment did not affect fasting blood glucose (p = 0.17) or blood insulin (p = 0.58), and there was no effect of PAE (S5 Table; Fig 7A and 7B). We investigated glucose tolerance using an oral glucose tolerance test (OGTT), which is more physiological, and an intraperitoneal glucose tolerance test (IPGTT), which by-passes intestinal influences. In males subjected to OGTT, a main effect of treatment (Fig 7A, F(3,55.5) = 4.60, p <0.006), and a significant treatment x time interaction (F(3,55.5) = 4.60, p <0.002), revealed significantly elevated blood glucose in all three caloric groups, compared with H2O, at 15 and 60 minutes after oral challenge. Accompanying this was a significant main treatment effect (F(3,38) = 3.93, p <0.05) on glucose clearance, and ETOH, MD, and MCT males had increased areas-under-the-curve (AUC) compared with H2O, although only MD and ETOH were significant (inset, Fig 7A). Although blood insulin levels did not significantly differ with treatment during the OGTT (Fig 7B; p = 0.58), levels tended to be lower in ETOH offspring than in H2O controls. Responses to IPGTT were similar to those of OGTT (Part A in S4 Fig). There was a significant effect of treatment (F(3,55.5) = 2.74, p = 0.05), such that MD males maintained significantly higher blood glucose concentrations compared with H2O (Part A in S4 Fig); male MCT and ETOH blood glucose levels were also elevated compared with H2O, but not to significance.

In contrast, PAE and prenatal calories did not affect glucose handling in females. At age 17 weeks, fasting glucose (p = 0.14) and insulin (p = 0.84) were unaffected by treatment (S5 Table; Fig 5A and 5B). Females did not significantly differ in their responses during OGTT (Fig 7C; treatment, p = 0.36; treatment x time, p = 0.74). AUC measures did not differ between groups (p = 0.75), and blood insulin levels at 15 minutes post-glucose were similar (Fig 7D, treatment x time, p = 0.90). For the IPGTT, PAE again did not affect glucose clearance (treatment, p = 0.85; treatment x time, p = 0.19) or AUC (p = 0.61, Part B in S4 Fig).

We again considered whether high-fat feeding might exacerbate the mild effect of PAE (and MD) upon glucose tolerance [20, 22]. As anticipated, the high-fat diet increased fasting glucose levels in both sexes, and there was no additional effect of prenatal treatment (S5 Table). In the high-fat-fed males, although there was no main effect of treatment in the OGTT (p = 0.20), a significant treatment x time interaction (Fig 7E, F(3,26) = 3.81, p = 0.05) found higher blood glucose levels in MD males at 30 minutes and a trend to increase at 60 and 120 minutes. For the females, high-fat diet did not unmask an effect of PAE, and prenatal treatment did not affect glucose clearance in the OGTT (Fig 7G, treatment, p = 0.11; treatment x time, p = 0.85; AUC, p = 0.21). Prenatal treatment also did not affect insulin levels at fasting and 15 minutes after glucose challenge in males (Fig 7F, p = 0.48) and females (Fig 7H, p = 0.80) but, as expected, insulin was elevated in response to high-fat feeding.

### High-Dose PAE does not affect offspring body composition and glucose metabolism

**Fig 8:**
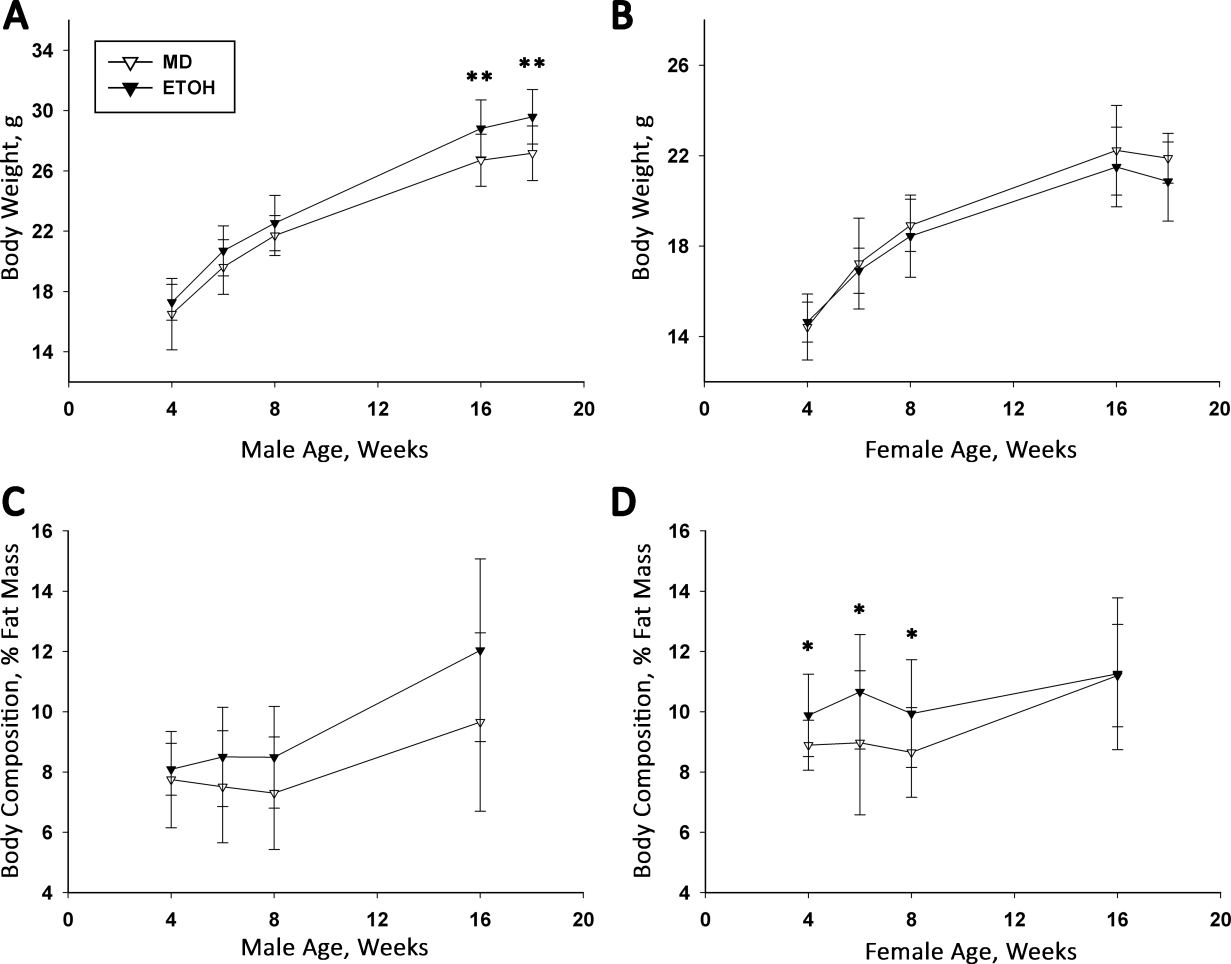
High-Dose PAE does not cause an adiposity phenotype in male and female offspring. Pregnant dams were exposed to 4.5 g/kg alcohol or isocaloric MD daily from E12.5–17.5 and body weight and percentage fat mass was followed thereafter. (A) PAE and MD male offspring do not differ in body weight until age 16 and 18 weeks, when PAE males are significantly heavier. (B) Body weights of PAE and MD female offspring do not significantly differ through age 18 weeks. (C) The percentage fat mass of PAE and MD male offspring does not differ by age, as assessed using NMR. (D) PAE female mice have significantly greater percentage fat mass at ages 4, 6, and 8 weeks as compared with MD controls. Values are mean ± SEM of 12–16 offspring per sex*treatment group, representing 4 MD litters and 5 PAE litters. * p<0.05 and ** p<0.01 vs. MD, using mixed linear factorial analysis of variance, followed by slice-effect ANOVAs with *a priori* hypotheses allowing for planned comparisons.

We hypothesized that alcohol’s impact upon the offspring’s metabolism might be dose-dependent, and that a higher alcohol dose would elicit a greater metabolic dysregulation. We therefore exposed a separate cohort of offspring to a higher alcohol dose, 4.5 g/kg ETOH, or isocaloric MD, from E12.5 to E17.5; a similar exposure (4 g/kg/d) during this gestational window impairs offspring body growth and glucose tolerance in rat [17, 19, 20, 27, 28]. We found that PAE did not affect maternal body weight at E17.5 (MD 34.01 ± 2.04 g, ETOH 30.82 ± 2.42 g; p>0.07), weight gain during the dosing period (MD 7.66 ± 1.17 g, ETOH 6.26 ± 1.00 g; p>0.09), mean litter size (MD 7.2 ± 1.0, ETOH 6.0 ± 1.6; p = 0.21), or pup weight (MD 1.07 ± 0.21 g, ETOH 1.07 ± 0.12 g; p = 0.98). Compared with MD controls, PAE did not affect postnatal growth (males, p = 0.12; females p = 0.62) or growth trajectory (treatment x week interaction, males, p = 0.86; females, p = 0.66; Fig 8A and 8B). However, at age 16 and 18 weeks, the ETOH males were significantly larger than their MD counterparts (MD 26.71 ± 0.52 g, ETOH 28.82 ± 0.52 g, F(1,22) = 8.19, p<0.01). Although their percentage fat mass was similar through age 16 weeks (treatment x week interaction, p = 0.61), values trended to be higher in ETOH males at ages 8 weeks (MD 7.52 ± 0.30%, ETOH 8.36 ± 0.32%, p<0.07) and 16 weeks (MD 9.66 ± 0.90% vs. ETOH 12.04 ± 0.87%, F(1,21) = 3.61, p<0.07; Fig 8C). For females, ETOH offspring had a slightly higher fat mass percentage from ages 4 through 8 weeks (at 8 weeks, F(1,33.9) = 9.23, p<0.002; Fig 8D), and this difference disappeared by age 16 weeks (weight: p = 0.30; fat mass percent: p = 0.94). At 18 weeks old, PAE did not affect fasting blood glucose in males (MD 169.9 ± 37.8 mg/dL, ETOH 166.7 ± 51.3 mg/dL; p = 0.87) or females (MD 142.6 ± 7.7 mg/dL, ETOH 141.2 ± 21.1 mg/dL; p = 0.86). Thus, a higher alcohol dose modestly affected offspring body weight, body composition, and baseline glucose, but these values did not appreciably differ from those at 3.0 g/kg alcohol.

## Discussion

Although multiple reports suggest PAE may increase central adiposity and glucose intolerance in later life, our comprehensive analysis failed to identify a causative role for PAE in promoting metabolic syndrome in this mouse model. We modeled reports in which PAE affected metabolism and the mice generated here shared characteristics of those FASD animal models including postnatal growth restriction [21, 45, 46], increased adiposity relative to MD controls [16, 20, 22], and impaired glucose tolerance compared with H2O controls [17, 19, 20, 25–28]. Yet the magnitude of change in our PAE offspring was modest at best, and we did not find significant alterations in RER, metabolic adaptation to fuel, glucose tolerance, and cardiac function. Instead, our use of multiple caloric controls, intended to isolate the effects of PAE, identified prenatal calories itself–whether in the form of alcohol, maltodextrin, or MCT–as a significant influence upon the offspring’s metabolic responses at adulthood.

We considered whether aspects of the study design may have interfered with the ability to detect a PAE-dependent metabolic phenotype. The C57BL/6J strain used here is vulnerable to both PAE [45–49] and diet-induced obesity [50]. The maternal BACs achieved here, ~110 mg/dl, are typical for murine PAE studies [45–48] and meet or exceed those associated with neurodevelopmental deficits in mouse [47, 48], and glucose intolerance in rat [17, 19, 20, 23, 25–28]. Exposure to a higher ETOH dose (4.5 g/kg) did not worsen the modest adiposity and glucose tolerance responses observed here. Moreover, chronic maternal BACs as low as 30 mg/dl are reported to cause persistent metabolic changes in rat offspring, even in the absence of fetal growth restriction [23], also suggesting our dose was not too low. Alternately, perhaps the noncaloric-driven phenotype emerges under low alcohol exposure. It is also possible our exposure period of E12.5 –E17.5 may have missed a critical window during early embryogenesis [37, 46], as we did not target the periconceptual window that alters placenta development and body growth as described by others [22, 45, 46]. However, for the rat, the developmental periods of E8 –E14, E15 –E21, and E11 –E20 are said to share similar vulnerability to PAE-induced metabolic dysfunction as compared to water gavage [27, 28], and our model replicates that report.

We also considered whether these PAE offspring had a latent metabolic phenotype that would be ‘unmasked’ by dietary challenge, as per [20, 22]. However, as assessed using indirect calorimetry, the metabolism of PAE offspring adjusted identically to that of controls when they consumed low-fat (10% of calories) and high-fat (60% of calories) diets. There were no ETOH-specific changes in glucose tolerance or adiposity after extended high-fat feeding, and, prior to high-fat feeding, there were no trends that suggested a propensity for deterioration in later life. The techniques employed here to evaluate the animals–including indirect calorimetry, diet challenge, NMR, DEXA, and arterial catheterization–are ‘gold standard’ methods to interrogate energy expenditure, body composition, and cardiac function and are unlikely to miss a ‘subtle’ phenotype [50]. It is also possible that age 17–22 weeks was ‘too young’ to exhibit metabolic dysfunction; however, this age (13–20 weeks) displays metabolic dysfunction in rat models of PAE [17, 19, 26, 27], and we saw no evidence of earlier physiological changes (e.g. energy expenditure, food intake, fasting glucose), under control or high-fat diet, that would predispose the animal to obesity in later life. Taken together, these results suggest that alcohol did not alter metabolism, glucose handling, or cardiac function in this mouse model of PAE.

Insight into a potential explanation emerged from our use of multiple controls that were designed to isolate alcohol’s effects from those of gavage stress (H2O) and calories (MD and MCT). Comparison of ETOH offspring with those receiving H2O, as per [17–20, 25–28], revealed PAE phenotypes consistent with those studies including reduced lactational weight gain (in females), increased blood pressure (males), reduced lean mass (females), and elevated fasting glucose, reduced glucose clearance, and elevated insulin (both sexes). A similar comparison of ETOH offspring to those receiving isocaloric carbohydrate, as in [16, 21–24], revealed increased adiposity (males), elevated heart rate (both sexes), increased adiposity in response to high-fat diet (males), and hyperphagia (females). These differences largely disappeared when ETOH was instead compared against both the MCT and MD controls. Moreover, the offspring phenotypes observed in the three caloric groups versus H2O are also observed in studies of maternal obesity [51–53] and include increased gestational and post-weaning weight gain, decreased inguinal fat size, and glucose intolerance, with a greater impact on males. In our model, ETOH, MCT, and MD provided an additional 3% calories (0.42 kilocalories) daily with respect to the mouse requirement [54]. The similarity of the ETOH, MCT, and MD outcomes compared with H2O suggests that it was the extra gestational calories that influenced these metabolism-related endpoints, and raises the possibility that alcohol’s action with respect to the metabolic phenotype was not pharmacologic but caloric. This possibility highlights the critical importance of selecting appropriate controls when investigating agents, such as alcohol, that also have a nutritional impact.

That PAE did not additionally affect obesity risk in this calorie-controlled PAE model questions whether it increases that risk for humans. The clinical literature is inconsistent on this point and the growth effects of PAE are complex and dose-dependent. Whereas heavy exposures are associated with small-for-gestational age, reduced postnatal growth, and lower BMI [3, 5, 6, 8], intermediate exposures are associated with normal or catch-up growth in periadolescence [5, 6, 9–11]. The few studies that assess growth quality report dose-dependent reductions in body fat content as measured by bioimpedence [4] or triceps skin fold [8]. Reports of increased body adiposity in PAE are either anecdotal [12–15, 32] or involve cohorts in which the subjects’ ethnicity or economic status independently elevate obesity risk [5, 8, 13, 32, 55]. Thus, although reduced perinatal growth is an independent risk factor for obesity [7], clinical findings and our animal model both suggest that PAE may not be a primary cause of metabolic syndrome, and adiposity in those with FASD is likely secondary to external factors. Obesity is the complex product of multiple influences, including neurobehavioral influences upon appetite, reward, and appetitive learning, and external factors such as medication use, home environment, and food security history. It may be worth noting that body assessments in the older literature included more children who remained with their birth parents, and a differential home environment compared with that in foster or adoptive care may be a previously misunderstood confound affecting food access and eating patterns.

Our study also informs observations that children diagnosed with FASD have an increased incidence of hyperphagia [12, 32] and a preference for calorically-dense snack foods high in fat and sugar content [12, 13]. Thus, it was unexpected that food intake in these PAE mice did not differ from controls when offered a standard chow or when offered sugar- and fat-rich diets; in fact, ETOH females gained the least weight among groups during high-fat feeding, partly because they consumed less food than controls. However, eating behaviors in humans are more complex than those of laboratory animals and, as with obesity risk, they are shaped by multiple external and internal influences. Those with special relevance for FASD include the behavioral medications that are commonly prescribed, past experiences of food insecurity, and deficits in executive function and reward systems [56, 57]; these factors are also implicated in the mechanisms underlying food addiction and obesity [35, 36]. Taken together, our results suggest the increased adiposity and hyperphagia described in FASD might not be a consequence of dysregulated metabolism or appetitive control, and instead may reflect alternate influences upon the individual.

Our study has additional limitations. Most studies that describe metabolic dysregulation by PAE use rats and ours is one of the few using mouse [45, 46]. We employed a gavage route to standardize the exposure and model episodic binge drinking, but data suggest that gavage and liquid diet delivery of alcohol generate similar results [16–28]. The PAE offspring studied here did not exhibit prenatal growth restriction, which has been implicated in the DOHaD hypothesis [7, 30, 37, 46]; however, glucose intolerance due to PAE is independent of birth weight [19, 22, 23, 27]. Alcohol exposures prior to E12.5, and particularly the periconceptual period, may have greater vulnerability to *in utero* growth retardation and epigenetic reprogramming, yet others report that the preimplantation, organogenesis, and *in utero* growth periods are similarly vulnerable [18, 22, 27, 46]. Another limitation is the partial randomization of dams in our study design. Due to concerns about litter survival in our initial pilot studies, larger dams were occasionally assigned to be dosed with ETOH. However, most outcomes were similar among exposed offspring, regardless of dam size and treatment; moreover, ETOH offspring retained the hallmarks of a PAE animal model with respect to postnatal growth restriction [21, 45, 46] and increased male adiposity [16, 20, 22], suggesting that increased weight in dams did not affect the outcomes of this study. Thus, our experimental design is consistent with the alcohol doses and exposure timing used in previous reports that document increased obesity [16, 20, 22] and impaired glucose tolerance [17–20, 25–27] after PAE dosing. Finally, although we did not assess expression-level changes, this would only be informative in the presence of a phenotype, and no such phenotype emerged even when provoked.

In summary, this study failed to identify a specific effect of PAE upon the offspring’s metabolic rate, glucose tolerance, and cardiac function at adulthood. These results are at odds with a literature, mostly in rats, that show impaired glucose handling [17–20, 23–28], hypertension [43, 44], and visceral adiposity [16, 20, 22]. Instead, alcohol’s impact was minimized in the comparison of ETOH against MCT, and data suggest the probable effector in this model reflects the caloric or metabolic contribution of alcohol, rather than its pharmacologic action per se. This conclusion is consistent with reports that children with FASD have comparatively lower BMI and adiposity [5, 6, 8, 11]. We conclude that the addition of calories above the recommended intake during pregnancy are associated with changes in metabolism and glucose handling that may be incompatible with long-term positive health. The source of the added calories as fat, sugar, or alcohol generated similar outcomes as compared with the water gavage control, suggesting that future studies investigating facets of metabolic syndrome after PAE should control for the caloric and metabolic properties of alcohol.

## Supporting information

S1 Fig Growth curves of male and female offspring in response to PAE. (A) Body weight of male offspring over the lactation period was significantly differentiated by prenatal treatment (treat*day effect = p<0.001) although posthoc analyses by day were not significant. (B) Weight of female offspring over the lactation period was significantly differentiated by prenatal treatment (treat*day effect = p<0.001) although posthoc analyses by day were not significant. Average weight during this timespan (inset) revealed that female ETOH offspring were significantly smaller (p<0.05) than both female H2O and isocaloric (MCT and MD) control offspring. (C) After weaning (P21-P105), prenatal treatment did not affect absolute body weight in male offspring. Although there was a significant treatment x week interaction (F(36,1551) = 2.60, p <0.0001), no significant differences emerged during post-hoc analyses. (D) After weaning (P21-P105), absolute body weight of female offspring was not significantly affected by prenatal treatment. Values are mean ± SEM of > 20 mice per sex*treatment group. * p<0.05 vs. H2O, # p<0.05 vs. MCT control, using mixed linear factorial analysis of variance, followed by slice-effect ANOVAs with *a priori* hypotheses allowing for planned comparisons.
(PDF)

S2 Fig Prenatal treatment does not alter bone mineral density or content in PAE mice. Bone mineral density (BMD) and bone mineral content (BMC) were assessed in control and alcohol-treated offspring at age 17 weeks. (A) In males, neither ETOH nor isocaloric prenatal treatment affected BMD and BMC, as compared with H2O controls. (B) In females, neither ETOH nor isocaloric prenatal treatment affected BMD and BMC, as compared with H2O controls. Values are mean ± SEM of 10–12 offspring per sex*treatment group. * p<0.05 vs. H2O, # p<0.05 vs. MD control, using mixed linear factorial analysis of variance, followed by slice-effect ANOVAs with *a priori* hypotheses allowing for planned comparisons.
(PDF)

S3 Fig PAE Increases heart rate but not blood pressure in adult male offspring. Heart rate and blood pressure were assessed using aortic catheterization in anesthetized mice at 19 weeks of age. (A) Resting heart rate was elevated in adult ETOH offspring, but only to significance in males (p<0.05). (B) Aortic systolic pressure was not significantly different among male and female offspring, regardless of prenatal treatment. (C) Aortic diastolic pressure was not significantly different among male and female offspring, regardless of prenatal treatment. Values are mean ± SEM of 8–10 offspring per sex*treatment group. * p<0.05 vs. MD, using mixed linear factorial analysis of variance, followed by slice-effect ANOVAs with *a priori* hypotheses allowing for planned comparisons.
(PDF)

S4 Fig PAE does not affect glucose clearance in response to intraperitoneal glucose tolerance testing (IPGTT). IPGTT was performed at age 17 weeks in a cohort distinct from that subjected to OGTT. Blood glucose was assessed at times after intraperitoneal glucose administration. (A) Fasting glucose was unaffected by PAE or prenatal treatment, but a significant treatment effect revealed that MD males maintained a significantly higher blood glucose level in comparison with other treatment groups (p<0.05) throughout IPGTT. Net glucose clearance, reflected in the area-under-the-curve (AUC) was unaffected by PAE or prenatal treatment (inset). (B) PAE and prenatal treatment did not affect fasting glucose in female offspring, and did not affect blood glucose levels or glucose clearance (inset) in response to intraperitoneal glucose challenge. Values are mean ± SD of 8–10 offspring per sex*treatment group.
(PDF)

S1 Table Composition of experimental diets in metabolic cages. (DOCX)

S2 Table PAE does not affect organ weight parameters, normalized to body weight, in offspring at 17 weeks of age. (DOCX)

S3 Table PAE does not affect left ventricular performance in offspring at 17 weeks of age. (DOCX)

S4 Table Statistical analyses of the metabolic chamber data presented in Fig 5. (DOCX)

S5 Table Blood glucose changes in response to OGTT in PAE offspring at age 17 weeks and after 4 weeks of high-fat diet challenge. (DOCX)

S1 Dataset Zipped primary data files for interpretation of results. (ZIP)
