# Supplementary figures and images for: Does prenatal alcohol exposure cause a metabolic syndrome? (Non-)evidence from a mouse model of fetal alcohol spectrum disorder
Source: PLoS One. 2018 Jun 28;13(6):e0199213. doi: 10.1371/journal.pone.0199213 (PMC6023152; doi:10.1371/journal.pone.0199213)

## Supplemental Figure 1

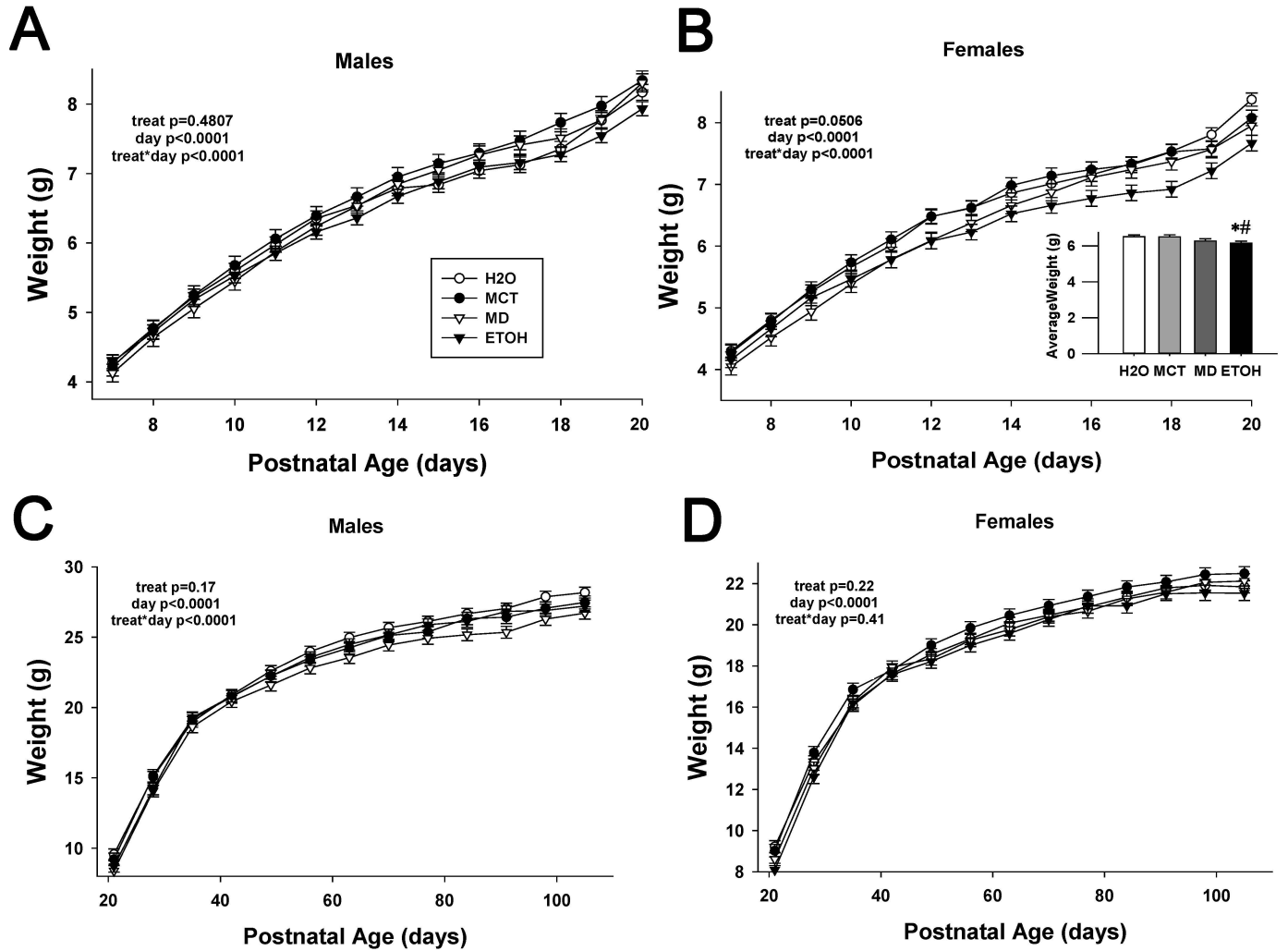

Supplement: S1 Fig — (A) Body weight of male offspring over the lactation period was significantly differentiated by prenatal treatment (treat*day effect = p<0.001) although posthoc analyses by day were not significant. (B) Weight of female offspring over the lactation period was significantly differentiated by prenatal treatment (treat*day effect = p<0.001) although posthoc analyses by day were not significant. Average weight during this timespan (inset) revealed that female ETOH offspring were significantly smaller (p<0.05) than both female H2O and isocaloric (MCT and MD) control offspring. (C) After weaning (P21-P105), prenatal treatment did not affect absolute body weight in male offspring. Although there was a significant treatment x week interaction (F(36,1551) = 2.60, p <0.0001), no significant differences emerged during post-hoc analyses. (D) After weaning (P21-P105), absolute body weight of female offspring was not significantly affected by prenatal treatment. Values are mean ± SEM of > 20 mice per sex*treatment group. * p<0.05 vs. H2O, # p<0.05 vs. MCT control, using mixed linear factorial analysis of variance, followed by slice-effect ANOVAs with a priori hypotheses allowing for planned comparisons. (PDF) [file pone.0199213.s001.pdf]

## Supplemental Figure 2

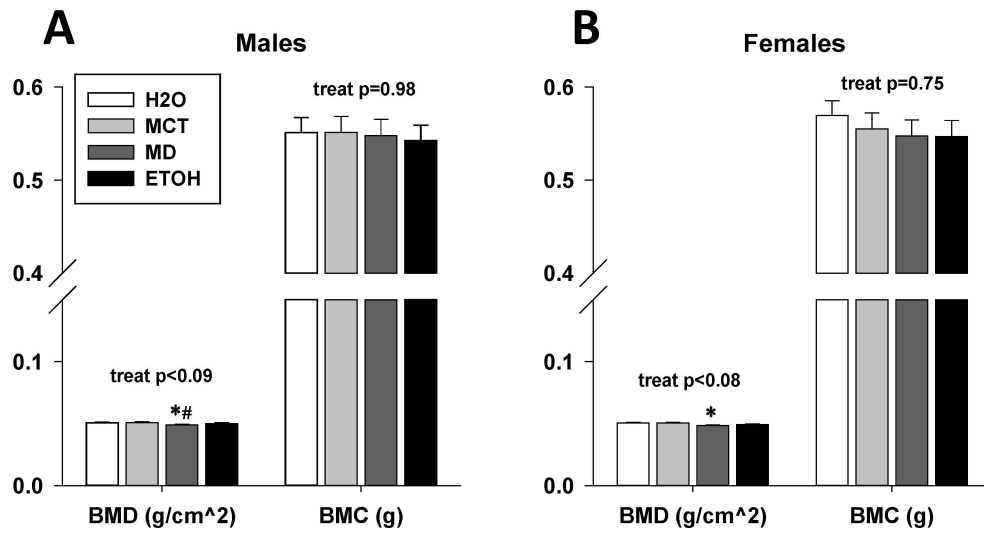

Supplement: S2 Fig — Bone mineral density (BMD) and bone mineral content (BMC) were assessed in control and alcohol-treated offspring at age 17 weeks. (A) In males, neither ETOH nor isocaloric prenatal treatment affected BMD and BMC, as compared with H2O controls. (B) In females, neither ETOH nor isocaloric prenatal treatment affected BMD and BMC, as compared with H2O controls. Values are mean ± SEM of 10–12 offspring per sex*treatment group. * p<0.05 vs. H2O, # p<0.05 vs. MD control, using mixed linear factorial analysis of variance, followed by slice-effect ANOVAs with a priori hypotheses allowing for planned comparisons. (PDF) [file pone.0199213.s002.pdf]

# Supplemental Figure 3

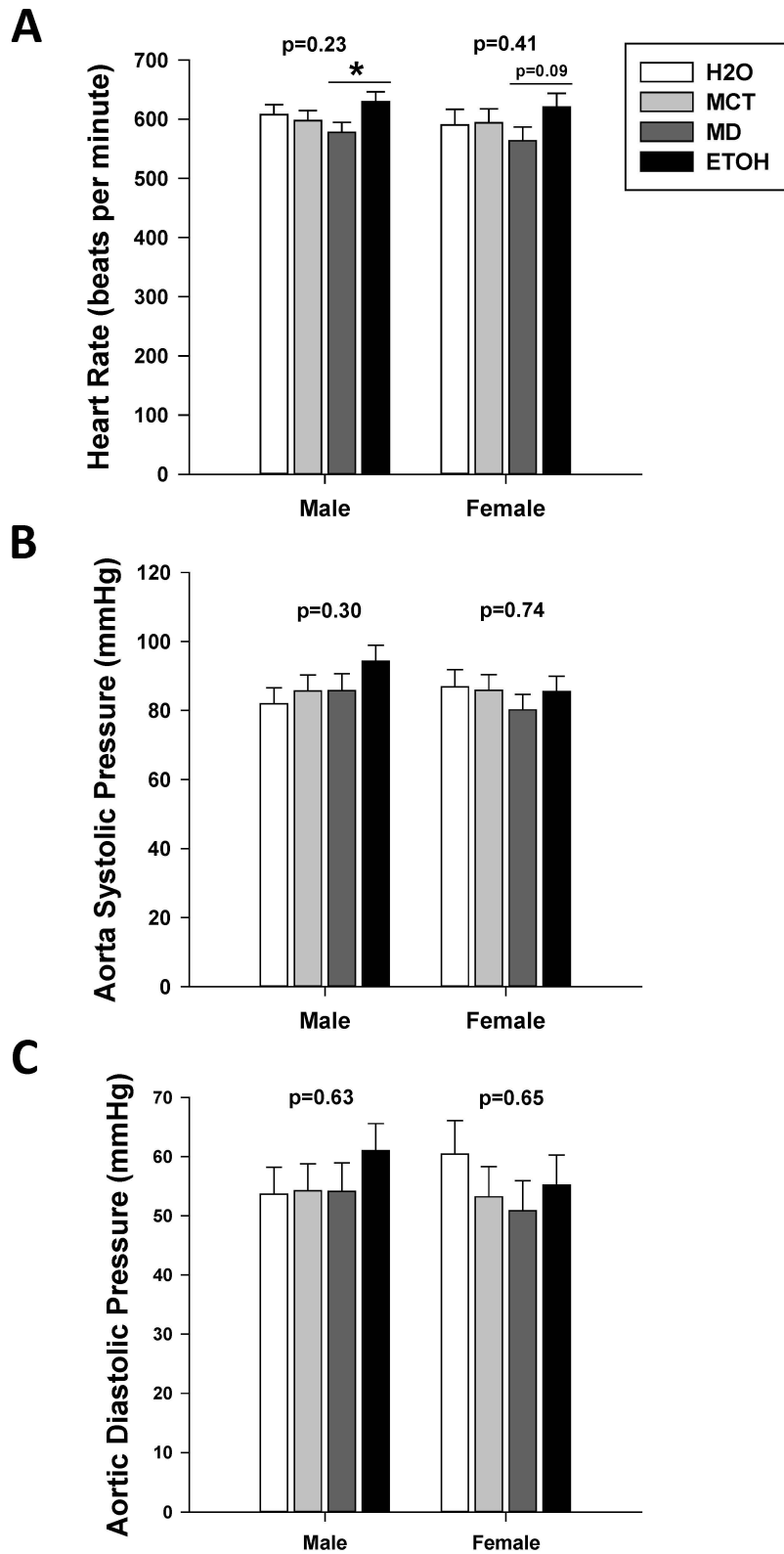

Supplement: S3 Fig — Heart rate and blood pressure were assessed using aortic catheterization in anesthetized mice at 19 weeks of age. (A) Resting heart rate was elevated in adult ETOH offspring, but only to significance in males (p<0.05). (B) Aortic systolic pressure was not significantly different among male and female offspring, regardless of prenatal treatment. (C) Aortic diastolic pressure was not significantly different among male and female offspring, regardless of prenatal treatment. Values are mean ± SEM of 8–10 offspring per sex*treatment group. * p<0.05 vs. MD, using mixed linear factorial analysis of variance, followed by slice-effect ANOVAs with a priori hypotheses allowing for planned comparisons. (PDF) [file pone.0199213.s003.pdf]

Supplemental Figure 4

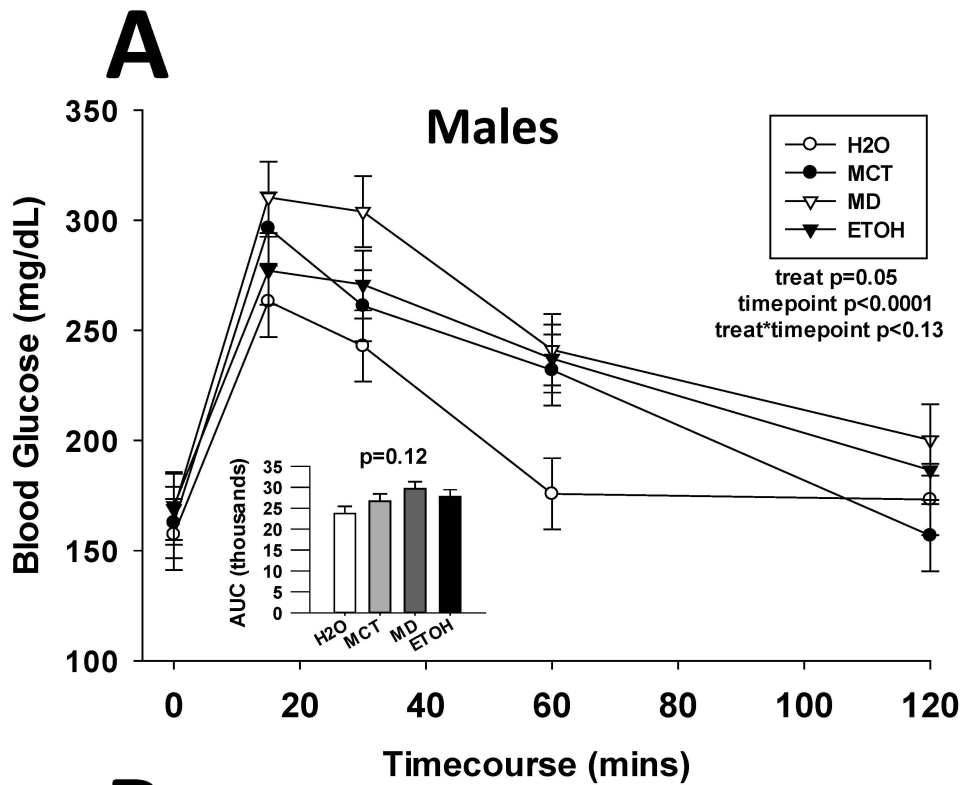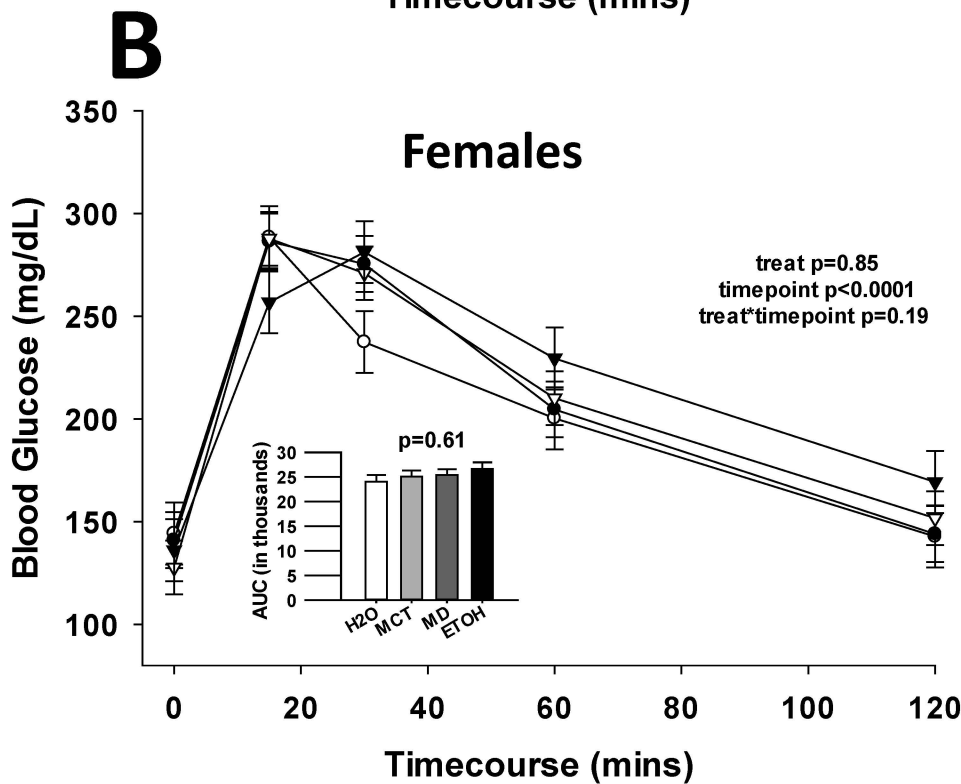

Supplement: S4 Fig — IPGTT was performed at age 17 weeks in a cohort distinct from that subjected to OGTT. Blood glucose was assessed at times after intraperitoneal glucose administration. (A) Fasting glucose was unaffected by PAE or prenatal treatment, but a significant treatment effect revealed that MD males maintained a significantly higher blood glucose level in comparison with other treatment groups (p<0.05) throughout IPGTT. Net glucose clearance, reflected in the area-under-the-curve (AUC) was unaffected by PAE or prenatal treatment (inset). (B) PAE and prenatal treatment did not affect fasting glucose in female offspring, and did not affect blood glucose levels or glucose clearance (inset) in response to intraperitoneal glucose challenge. Values are mean ± SD of 8–10 offspring per sex*treatment group. (PDF) [file pone.0199213.s004.pdf]
