# Supplementary material for: Does prenatal alcohol exposure cause a metabolic syndrome? (Non-)evidence from a mouse model of fetal alcohol spectrum disorder
Source: PLoS One. 2018 Jun 28;13(6):e0199213. doi: 10.1371/journal.pone.0199213 (PMC6023152; doi:10.1371/journal.pone.0199213)
Supplement: S1 Table — (DOCX) [file pone.0199213.s005.docx]

**S1 Table**

|  | Low Fat Diet  (TD.06416)^1^ | High Fat Diet  (TD.06414)^2^ |
| --- | --- | --- |
|  |  |  |
| Ingredient, *g/kg diet* |  |  |
| Casein | 210.0 | 265.0 |
| L-Cystine | 3.0 | 4.0 |
| Corn Starch | 280.0 | - |
| Maltodextrin | 50.0 | 160.0 |
| Sucrose | 325.0 | 90.0 |
| Lard | 20.0 | 310.0 |
| Soybean Oil | 20.0 | 30.0 |
| Cellulose | 37.15 | 65.5 |
| Mineral Mix, AIN-93G-MX | 35.0 | 48.0 |
| Vitamin Mix, AIN-93G-VX | 15.0 | 21.0 |
| Choline Bitartrate | 2.75 | 3.0 |
| Food Coloring | 0.1 (yellow) | 0.1 (blue) |
|  |  |  |

^1^ TD.06414 (Harlan-Teklad, Madison, WI). Provides 20.1% kcal from protein, 69.8% kcal from carbohydrate, 10.2% kcal from fat, and 3.7 kcal/g.

^2^ TD.06416 (Harlan-Teklad, Madison, WI). Provides 18.4% kcal from protein, 21.3% kcal from carbohydrate, 60.3% kcal from fat, and 5.1 kcal/g.

^*^ American Institute of Nutrition. Report of the American Institute of Nutrition ad hoc committee on standards for nutritional studies. J Nutr 1977;107:1340
