# Supplementary material for: Does prenatal alcohol exposure cause a metabolic syndrome? (Non-)evidence from a mouse model of fetal alcohol spectrum disorder
Source: PLoS One. 2018 Jun 28;13(6):e0199213. doi: 10.1371/journal.pone.0199213 (PMC6023152; doi:10.1371/journal.pone.0199213)
Supplement: S2 Table — (DOCX) [file pone.0199213.s006.docx]

|  | **Male Offspring** | | | |  | **Female Offspring** | | | |  |
| --- | --- | --- | --- | --- | --- | --- | --- | --- | --- | --- |
| **Measured Tissue  (% Body Weight)** | **H2O (± SE)** | **MCT (± SE)** | **MD (± SE)** | **ETOH (± SE)** | **P value** | **H2O (± SE)** | **MCT (± SE)** | **MD (± SE)** | **ETOH (± SE)** | **P value** |
| **Major Organs** |  |  |  |  |  |  |  |  |  |  |
| Brain | 1.80 ± 0.06 | 1.90 ± 0.11 | 1.74 ± 0.07 | 1.64 ± 0.04 | *0.73* | 2.06 ± 0.09 | 2.13 ± 0.06 | 2.18 ± 0.03 | 2.17 ± 0.04 | *0.50* |
| Heart | 0.54 ± 0.03 | 0.62 ± 0.03 | 0.54 ± 0.03 | 0.53 ± 0.03 | *0.13* | 0.60 ± 0.03 | 0.68 ± 0.03 | 0.65 ± 0.03 | 0.63 ± 0.03 | *0.08* |
| Liver | 4.15 ± 0.18 | 4.18 ± 0.19 | 4.60 ± 0.18 | 4.71 ± 0.17 | *0.07* | 4.25 ± 0.16 | 4.24 ± 0.16 | 4.62 ± 0.17 | 4.77 ± 0.18 | *0.29* |
| Pancreas | 1.57 ± 0.12 | 1.57 ± 0.12 | 1.51 ± 0.12 | 1.58 ± 0.11 | *0.97* | 1.57 ± 0.10 | 1.54 ± 0.10 | 1.66 ± 0.18 | 1.58 ± 0.12 | *0.88* |
| **Fat, Muscle, and Bone** |  |  |  |  |  |  |  |  |  |  |
| Inguinal Fat | 0.56 ± 0.04 | 0.39 ± 0.05 | 0.40 ± 0.05 | 0.47 ± 0.04 | *0.07* | 0.40 ± 0.03 | 0.33 ± 0.03 | 0.32 ± 0.03 | 0.35 ± 0.03 | *0.22* |
| Reproductive Fat | 1.89 ± 0.12 | 1.39 ± 0.14* | 1.40 ± 0.13* | 1.47 ± 0.12* | *0.03* | 0.76 ± 0.07 | 0.63 ± 0.07 | 0.64 ± 0.07 | 0.65 ± 0.08 | *0.5* |
| Brown Fat | 0.21 ± 0.02 | 0.20 ± 0.02 | 0.21 ± 0.02 | 0.21 ± 0.02 | *0.95* | 0.17 ± 0.02 | 0.17 ± 0.02 | 0.17 ± 0.02 | 0.17 ± 0.02 | *0.92* |
| Gastrocnemius | 0.49 ± 0.07 | 0.34 ± 0.08 | 0.38 ± 0.07 | 0.40 ± 0.02 | *0.56* | 0.32 ± 0.04 | 0.19 ± 0.04 | 0.23 ± 0.04 | 0.25 ± 0.04 | *0.15* |
| Tibia Length (mm) | 66.9 ± 2.7 | 64.8 ± 3.1 | 64.8 ± 2.7 | 67.0 ± 2.5 | *0.90* | 86.5 ± 1.8 | 84.1 ± 1.9 | 84.3 ± 2.1 | 91.2 ± 2.2# | *0.08* |
| **Immune and Stress** |  |  |  |  |  |  |  |  |  |  |
| Adrenals | 0.02 ± 0.002 | 0.02 ± 0.002 | 0.02 ± 0.002 | 0.02 ± 0.002 | *0.86* | 0.04 ± 0.002 | 0.04 ± 0.002 | 0.33 ± 0.002 | 0.03 ± 0.002 | *0.13* |
| Spleen | 0.25 ± 0.008 | 0.29 ± 0.009* | 0.25 ± 0.008# | 0.27 ± 0.008 | *0.01* | 0.35 ± 0.01 | 0.37 ± 0.01 | 0.41 ± 0.01* | 0.35 ± 0.01ǂ | *0.01* |
| Thymus | 0.16 ± 0.06 | 0.12 ± 0.06 | 0.26 ± 0.06 | 0.15 ± 0.05 | *0.39* | 0.22 ± 0.01 | 0.20 ± 0.01 | 0.21 ± 0.01 | 0.19 ± 0.01 | *0.43* |

**S2 Table**

Values are mean ± SEM of 8-10 offspring per sex*treatment group, assessed at 17 weeks of age subsequent to the indicated prenatal exposure. All organ weights are normalized to body weight. * p<0.05 vs. H2O, # p<0.05 vs. MCT, ǂ p<0.05 vs. MD, using mixed linear factorial analysis of variance, followed by slice-effect ANOVAs with *a priori* hypotheses allowing for planned comparisons.
