# Supplementary material for: Does prenatal alcohol exposure cause a metabolic syndrome? (Non-)evidence from a mouse model of fetal alcohol spectrum disorder
Source: PLoS One. 2018 Jun 28;13(6):e0199213. doi: 10.1371/journal.pone.0199213 (PMC6023152; doi:10.1371/journal.pone.0199213)
Supplement: S3 Table — (DOCX) [file pone.0199213.s007.docx]

|  | **Male Offspring** | | | |  | **Female Offspring** | | | |  |
| --- | --- | --- | --- | --- | --- | --- | --- | --- | --- | --- |
|  | **H2O (± SE)** | **MCT (± SE)** | **MD (± SE)** | **ETOH (± SE)** | ***P* value** | **H2O (± SE)** | **MCT (± SE)** | **MD (± SE)** | **ETOH (± SE)** | ***P* value** |
| dP/dt Max (thous) | 8.4 ± 0.84 | 10.7 ± 0.79 | 8.3 ± 0.97 | 10.4 ± 0.90 | *0.11* | 10.9 ± 1.60 | 9.4 ± 1.41 | 9.3 ± 1.50 | 9.3 ± 1.50 | *0.87* |
| dP/dt Min (thous) | -6.0 ± 0.39 | -7.8 ± 0.37* | -6.4 ± 0.45# | -6.5 ± 0.42# | *0.01* | -7.1 ± 0.88 | -7.3 ± 0.78 | -5.9 ± 0.83 | -7.0 ± 0.83 | *0.61* |
| Pressure at dP/dt Max | 45.6 ± 4.13 | 55.1 ± 3.90 | 52.8 ± 4.77 | 51.2 ± 4.42 | *0.41* | 53.8 ± 5.96 | 56.7 ± 5.26 | 48.2 ± 5.58 | 51.2 ± 5.60 | *0.72* |
| Pressure at dP/dt Min | 38.7 ± 4.26 | 44.0 ± 4.01 | 52.8 ± 4.92 | 44.2 ± 4.55 | *0.22* | 42.4 ± 4.63 | 47.8 ± 4.08 | 39.4 ± 4.32 | 47.0 ± 4.33 | *0.48* |
| Weiss Tau | 4.73 ± 4.31 | 9.33 ± 4.61 | 18.05 ± 4.98 | 8.69 ± 4.61 | *0.27* | 4.39 ± 4.83 | 6.99 ± 4.52 | 15.15 ± 4.52 | 10.89 ± 4.52 | *0.40* |
| Ejection Time | 37.8 ± 1.26 | 35.6 ± 1.18 | 37.0 ± 1.45 | 34.9 ± 1.34 | *0.41* | 40.9 ± 2.22 | 36.0 ± 1.96 | 40.5 ± 2.1 | 36.9 ± 2.07 | *0.25* |

**S3 Table**

Left ventricular performance was assessed in anesthetized, 17-week-old mice using cardiac catheterization, following the indicated prenatal exposure. Values are mean ± SEM of 8-10 offspring per sex*treatment group. * p<0.05 vs. H2O, # p<0.05 vs. MCT, ǂ p<0.05 vs. MD, using mixed linear factorial analysis of variance, followed by slice-effect ANOVAs with *a priori* hypotheses allowing for planned comparisons.
