# Supplementary material for: Does prenatal alcohol exposure cause a metabolic syndrome? (Non-)evidence from a mouse model of fetal alcohol spectrum disorder
Source: PLoS One. 2018 Jun 28;13(6):e0199213. doi: 10.1371/journal.pone.0199213 (PMC6023152; doi:10.1371/journal.pone.0199213)
Supplement: S4 Table — (DOCX) [file pone.0199213.s008.docx]

**S4 Table**

**P-Values, Males___ __ P-Values, Females_____**

**Chow Low-Fat High-Fat Chow Low-Fat High-Fat**

Body weight, g 0.51 0.92 0.87 0.44 0.32 0.58

% body mass gain -- -- 0.99 -- -- 0.34

% fat mass gain 0.55 0.43 0.49 0.96 0.87 0.72

Food consumption 0.35 0.07 0.55 0.73 0.77 0.52

Water intake 0.95 0.49 0.66 0.32 0.96 0.55

Feeding behavior

Light cycle 0.30 0.76 0.51 <0.05 0.26 0.34

Dark cycle 0.72 0.18 0.77 0.62 0.28 <0.02

VO2

Light cycle 0.47 0.71 0.79 0.79 0.91 0.78

Dark cycle 0.81 0.63 0.70 0.57 0.98 0.77

RER

Light cycle 0.77 0.51 0.73 0.91 0.23 0.77

Dark cycle 0.98 0.27 0.76 0.66 0.31 0.54

________________________________________________________________________________

Presented are *p* values from mixed linear factorial analysis of variance, followed by slice-effect ANOVAs with *a priori* hypotheses allowing for planned comparisons, as detailed in Methods. Mice at age 17 weeks were housed in environmental chambers and their body weights, food and water intake, oxygen consumption, and calculated respiratory exchange ratio (RER) were calculated at three-day intervals following three-day feedings of chow, low-fat, and high-fat diets. The analyzed data are presented in Figure 5 and represent 8-12 offspring per sex*treatment group.
