# Supplementary material for: Does prenatal alcohol exposure cause a metabolic syndrome? (Non-)evidence from a mouse model of fetal alcohol spectrum disorder
Source: PLoS One. 2018 Jun 28;13(6):e0199213. doi: 10.1371/journal.pone.0199213 (PMC6023152; doi:10.1371/journal.pone.0199213)
Supplement: S5 Table — (DOCX) [file pone.0199213.s009.docx]

| Oral Glucose Tolerance | **Male Offspring** | | | |  | **Female Offspring** | | | |  |
| --- | --- | --- | --- | --- | --- | --- | --- | --- | --- | --- |
|  | **H2O (± SE)** | **MCT (± SE)** | **MD (± SE)** | **ETOH (± SE)** | **P value** | **H2O (± SE)** | **MCT (± SE)** | **MD (± SE)** | **ETOH (± SE)** | **P value** |
| **Pre-Challenge (Chow-Fed)** |  |  |  |  |  |  |  |  |  |  |
| Body Weight (g) | 26.8 ± 0.48 | 26.2 ± 0.48 | 26.2 ± 0.48 | 25.7 ± 0.43 | *0.43* | 20.7 ± 0.35 | 21.3 ± 0.33 | 20.3 ± 1.60 | 21.3 ± 0.37 | *0.09* |
| Fasting Glu (mg/dl) | 149 ± 10.4 | 175 ± 10.4 | 152 ± 10.4 | 172 ± 9.5 | *0.17* | 109 ± 8.6 | 138 ± 8.3 | 124 ± 7.9 | 122 ± 9.1 | *0.14* |
| % Glu Change | 175 ± 14.0 | 164 ± 14.0 | 212 ± 14.0 | 192 ± 12.8 | *0.10* | 239 ± 19.5 | 229 ± 18.6 | 252 ± 17.8 | 228 ± 20.5 | *0.79* |
| **28 Day High-Fat** |  |  |  |  |  |  |  |  |  |  |
| Body Weight (g) | 43.2 ± 1.56 | 40.4 ± 1.68 | 36.4 ± 1.45* | 38.2 ± 1.37* | *0.02* | 33.4 ± 1.39 | 31.3 ± 1.48 | 27.1 ± 1.60* | 25.1 ± 1.39*# | *0.001* |
| Fasting Glu (mg/dl) | 243 ± 14.0 | 234 ± 15.1 | 211 ± 13.0 | 230 ± 12.3 | *0.39* | 201 ± 10.7 | 176 ± 5.56 | 194 ± 12.4 | 208 ± 11.4 | *0.27* |
| % Glu Change | 155 ± 12.4 | 162 ± 13.4 | 191 ± 11.6 | 162 ± 10.9 | *0.16* | 185 ± 14.2 | 210 ± 15.2 | 201 ± 16.4 | 190 ± 15.2 | *0.64* |

**S5 Table**

Mice were subjected to OGTT immediately after metabolic phenotyping in environmental chambers, at age 18 weeks, and were tested again at age 22 weeks after they consumed a high-fat diet for 4 weeks. Values are mean ± SEM of 8-12 offspring per sex*treatment group. * p<0.05 vs. H2O, # p<0.05 vs. MCT, using mixed linear factorial analysis of variance, followed by slice-effect ANOVAs with *a priori* hypotheses allowing for planned comparisons.
