# Supplementary material for: Does prenatal alcohol exposure cause a metabolic syndrome? (Non-)evidence from a mouse model of fetal alcohol spectrum disorder
Source: PLoS One. 2018 Jun 28;13(6):e0199213. doi: 10.1371/journal.pone.0199213 (PMC6023152; doi:10.1371/journal.pone.0199213)
Supplement: S1 Dataset — (ZIP) [file pone.0199213.s010.zip › New folder/Arterial BP.pdf]

			Aortic				LV								
SUBJID	SEX	Treat	Systolic	Diastolic	Mean	HR	Systolic	Diastolic	dPdt Max	dPdt Min	Pressure dPdt Max	Pressure dPdt Min	Weiss Tau	Ejection Time ms	
6.3	F	ETOH	89.34	64.82	76.30	689.96	95.90	-1.09	11342.08	-9105.67	60.08	40.86	3.05	31.24	
9.7	F	ETOH	49.93	30.92	38.61	577.09	46.26	6.82	2069.01	-1765.52	20.13	25.42	20.84	52.29	
10.8	F	ETOH	91.54	61.31	75.81	705.00	95.22	0.43	12319.75	-9336.21	58.54	78.25	4.51	29.63	
17.7	F	ETOH	83.03	53.96	67.88	615.10	85.62	-2.06	8895.10	-7440.07	48.85	41.29	38.42	38.42	
18.6	F	ETOH	86.20	59.40	72.72	558.01	93.60	1.58	9952.94	-6006.10	58.16	51.89	6.56	39.67	
28.5	F	ETOH	94.51403	23.95001	52.76047	626.0967	98.74701	-3.325838	11090.7196	-8736.539595	60.58132694	44.74499936	2.684707273	34.42290749	
42.4	F	ETOH	91.43917	68.14484	79.83146	597.8018	87.30545	0.6203493	9534.593299	-7212.628048	52.2535667	44.98894004	5.157537288	35.02403846	
53.6	F	ETOH	92.52986	69.98157	81.36034	538.3238	82.84091	2.1461438	8935.870677	-6394.709732	50.84045876	48.30147907	5.973245629	34.77380952	
71.8	F	ETOH	93.38378	67.47886	79.68576	645.3078									
82.1	F	ETOH	82.7081	51.9564	65.89793	649.4175									
6.2	M	ETOH	102.47	77.51	89.63	673.69	.	.	.	.	.	.	.	.	
9.1	M	ETOH	85.44	48.23	66.10	641.26	94.22	0.23	11973.99	-7610.76	58.10	49.83	3.72	34.13	
10.3	M	ETOH	108.64	81.47	94.12	695.70	92.70	0.03	8558.71	-7707.87	50.95	48.80	4.61	36.67	
11.2	M	ETOH	122.40	98.23	111.39	591.09	.	.	.	.	.	.	.	.	
16.6	M	ETOH	117.48	81.23	99.31	654.70	.	.	.	.	.	.	.	.	
17.3	M	ETOH	78.42	49.30	61.08	605.89	86.00	0.75	9163.63	-5761.43	45.35	41.74	37.61	37.97	
18.5	M	ETOH	93.69	56.48	74.16	540.31	104.97	-2.32	13999.55	-6575.76	64.25	74.64	5.12	34.65	
41.7	M	ETOH	71.28026	35.38802	50.43214	662.7018	75.57527	-4.233445	8490.17653	-5243.682443	39.79199538	28.54377585	3.289072993	30.70658683	
42.2	M	ETOH	82.30172	37.66109	56.0749	683.8579	83.22017	-2.973809	8197.270551	-7857.78096	44.58768775	32.82825251	2.865271823	32.23423423	
71.4	M	ETOH	80.71816	44.75031	60.56158	543.2778	93.30151	-2.584506	12164.12664	-4867.747366	55.65480633	32.83175495	3.620445206	38.09634551	
27.2	F	H2O	113.18	82.61	98.14	648.94	124.61	1.99	18188.82	-9355.36	80.90	73.71	5.08	39.41	
37.6	F	H2O	91.37418	62.33473	76.62753	589.8809	87.26142	-0.538206	12335.22618	-8368.267662	60.96124572	30.91791982	3.891810338	53.236	
50.5	F	H2O	77.44749	55.9635	64.67584	448.2795	78.4766	6.4416598	5045.014925	-4989.731418	40.04350203	41.47560813	7.618877512	47.55172414	
63.6	F	H2O	93.08804	59.29283	76.37101	615.9067	100.7656	0.1622249	15087.16011	-7041.174396	65.48455643	45.39892825	3.825390942	39.90759076	
69.1	F	H2O	82.90736	59.3224	71.2142	608.6564									
79.4	F	H2O	81.95025	58.93025	67.80698	665.7967	88.32615	-4.354542	8390.839798	-7191.114387	35.94783177	32.35659442	2.096833595	34.80188679	
79.6	F	H2O	72.46756	48.14501	57.33978	599.4229	79.52007	0.8371677	7290.254335	-6442.249884	39.5259026	34.18417927	4.69890268	34.46153846	
83.5	F	H2O	81.95968	56.64192	67.96669	546.0384	87.62604	-1.361074	9731.700497	-6006.987656	53.99880055	39.10949818	3.52735965	37.22622951	
12.3	M	H2O	55.98	32.28	42.68	582.27	59.72	3.78	3637.58	-3670.89	26.42	31.48	9.12	39.72	
23.4	M	H2O	94.54	58.91	76.01	592.67	106.18	5.16	12427.27	-7199.32	68.15	53.96	6.19	40.40	
46.4	M	H2O	107.3471	76.8698	91.67198	631.771									
50.3	M	H2O	70.00755	40.72095	53.64597	616.9908	83.15774	-2.675638	7051.145089	-7040.25228	44.987194	40.4534775	3.141708638	40.38697318	
62.4	M	H2O	75.22016	51.53634	62.43106	660.2632	74.79182	0.8539104	8159.842697	-5368.381307	36.86919508	28.64741219	3.904138011	32.0509915	
69.8	M	H2O	71.70392	46.28607	56.56339	662.6382	80.93243	-0.273936	6882.690175	-6025.054396	43.14917437	34.62499214	3.753920946	35.56666667	
74.2	M	H2O	85.67695	58.29095	70.53745	602.8355	83.95556	-3.919428	9747.233952	-5984.180863	47.46864046	42.31826765	2.710046139	33.22222222	
76.1	M	H2O	79.5073	52.01931	65.48273	505.3341	89.25748	-0.991438	8300.545927	-6465.715533	49.7612074	42.39508493	4.041550738	45.71308017	
79.2	M	H2O	89.50939	56.61984	69.40133	612.3397									No LV Measures
83.2	M	H2O	90.25346	63.23205	76.03117	606.5942	81.44782	-1.624629	10623.57963	-6249.438213	47.75296639	35.64497069	5.014296277	35.13333333	
13.6	F	MCT	73.63	47.09	57.56	605.14	76.77	4.71	7395.16	-5005.78	42.45	34.66	6.66	37.73	
14.3	F	MCT	90.45	60.07	72.97	724.82	97.52	8.00	11758.92	-7710.22	64.01	45.87	5.94	31.82	
20.5	F	MCT	82.95	51.83	65.89	502.14	.	.	.	.	.	.	.	.	No LV measures
21.7	F	MCT	113.95	82.05	98.58	641.74	121.89	-2.06	15702.63	-12241.25	80.70	62.18	3.02	37.33	
32.5	F	MCT	94.31431	68.89188	82.45173	644.7427	106.3288	5.1127112	14580.43013	-10751.44752	75.90340791	57.20439889	6.52022389	34.59878419	
40.3	F	MCT	73.87792	6.129723	32.68327	435.4278	72.4718	46.545995	2123.800622	-1460.617086	54.1031758	63.39581184	308.7523037	39.75	
44.8	F	MCT	91.84731	67.15159	79.85178	639.4679	97.96185	10.748891	10923.99455	-7706.951701	66.09156745	52.47602093	21.68281856	35.76802508	
45.5	F	MCT	85.75333	52.23712	66.48173	635.6045	92.68505	2.5291891	8537.791817	-7710.796883	49.38687108	39.13277702	5.121850877	33.92887029	
77.6	F	MCT	70.5324	49.48933	58.99581	588.1609	81.80203	1.2292148	6468.696866	-6428.876471	37.18139588	35.22864637	4.35767071	38.82403433	No LV Measures'
78.7	F	MCT	81.32373	47.14226	62.4244	521.4416	77.04273	-6.301863	6967.180227	-7034.761623	40.40941715	39.76634449	2.633624461	34.57044674	
13.2	M	MCT	82.56	56.73	68.94	670.03	100.67	-13.72	11388.42	-9598.70	43.42	55.79	.	31.20	
14.1	M	MCT	105.08	70.65	86.21	626.60	97.60	-1.45	12452.29	-7489.94	57.70	41.26	3.99	32.22	
20.3	M	MCT	88.75	62.02	75.38	545.71	95.30	1.43	8628.62	-7694.05	54.74	46.89	39.44	39.44	
21.3	M	MCT	95.68	59.47	78.30	636.20	102.56	2.55	14558.13	-7174.05	70.03	45.49	5.18	39.01	
32.1	M	MCT	77.74856	36.58516	54.12139	547.4095	81.15074	-7.930241	8600.603791	-7347.877283	42.22709733	33.39448684	#N/A	36.44405594	No LV
44.1	M	MCT	85.44484	56.22129	68.58265	615.2363	95.56084	2.53589	11384.96542	-8271.14251	64.03187687	52.70214108	5.017517102	35.00330033	
45.1	M	MCT	92.25898	54.69895	71.83678	628.5115	93.43101	-4.685785	10448.59107	-8513.367018	53.49822827	46.17020649	4.089607244	35.28350515	
77.3	M	MCT	81.70165	51.26496	64.48377	620.5663	84.6743	1.806545	7121.004099	-7356.552865	49.83847285	37.90615644	4.760603876	35.89454545	
78.6	M	MCT	73.00379	48.53616	60.15357	546.6098	92.13471	-4.155884	11822.53988	-7059.16533	60.55026698	36.14411004	2.865633782	35.88562092	
84.3	M	MCT	74.56606	46.01106	58.37614	538.7695									
1.4	F	MD	54.76	30.98	41.19	558.08	.	.	.	.	.	.	.	.	
2.4	F	MD	62.41	41.42	51.32	534.69	62.55	0.29	4868.04	-4192.31	27.60	31.16	5.04	36.94	
19.4	F	MD	84.16	57.82	69.46	591.88	91.08	4.78	9239.80	-5297.27	52.51	46.27	42.86	42.86	
24.4	F	MD	103.85	73.80	89.65	597.37	112.39	-0.23	13887.46	-6843.21	67.99	48.31	3.89	40.13	
25.7	F	MD	71.52	40.27	61.65	409.85	72.51	2.33	3577.39	-3381.36	30.70	41.65	54.21	54.21	
26.4	F	MD	111.54	80.33	96.13	698.51	109.29	-1.05	18056.00	-8924.43	76.57	45.06	3.53	37.16	
34.3	F	MD	84.55963	43.23957	60.69018	671.5183	86.96978	-1.834713	9659.086715	-7559.741746	48.42664739	33.78466799	3.153835734	33.66666667	
38.6	F	MD	81.38338	50.38139	65.7018	546.9467									
43.5	F	MD	74.74639	38.55498	54.35345	533.2145	73.43792	-3.63658	6406.535037	-5613.514187	35.84301692	33.28516568	4.733892954	41.40160643	
52.5	F	MD	72.99632	51.55946	61.08101	492.0527	82.23747	-0.189627	8893.364567	-5154.244321	46.13636386	35.76425974	3.821005358	37.38513514	
1.3	M	MD	75.00	45.18	57.34	644.25	79.19	2.22	8150.13	-4742.27	38.01	40.04	5.95	35.46	
2.1	M	MD	65.91	44.99	52.75	535.28	.	.	.	.	.	.	.	.	
19.2	M	MD	84.16	61.19	72.21	485.88	84.54	2.27	5036.38	-5935.66	47.99	46.75	43.11	43.11	
26.3	M	MD	86.73	60.62	71.46	604.19	97.85	4.71	9787.12	-7112.56	53.88	50.88	36.74	36.74	
34.1	M	MD	96.30177	49.83326	70.94166	584.8951									
38.2	M	MD	89.05271	49.19967	65.98542	539.0346	99.14525	-1.366821	10585.65887	-7691.402383	54.32696326	60.68869686	5.701207636	33.38202247	
43.2	M	MD	118.3264	76.53132	94.41085	657.6555	122.0168	31.29543	9743.541316	-7737.42994	84.29263448	82.06845123	12.83204311	33.28220859	
43.3	M	MD	82.63582	50.80023	66.35828	497.2762									
52.4	M	MD	73.8285	48.92038	60.02674	650.0585	74.54312	-2.099311	6308.334537	-5089.73076	38.37506274	36.39250246	4.021279422	39.97288136	
