# Supplementary material for: Does prenatal alcohol exposure cause a metabolic syndrome? (Non-)evidence from a mouse model of fetal alcohol spectrum disorder
Source: PLoS One. 2018 Jun 28;13(6):e0199213. doi: 10.1371/journal.pone.0199213 (PMC6023152; doi:10.1371/journal.pone.0199213)
Supplement: S1 Dataset — (ZIP) [file pone.0199213.s010.zip › New folder/Body growth.pdf]

Litter	Treat	Sex	Subj #	P7	P8	P9	P10	P11	P12	P13	P14	P15	P16	P17	P18	P19	P20	P21	Week 4	Week 5	Week 6	Week 7	Week 8	Week 9	Week 10	Week 11	Week 12	Week 13	Week 14	Week 15	Week 16	Week 17
1	MD	M	1	4.55	5.18	5.9	6.34	6.6	6.7	7.54	7.42	7.48	7.65	7.64	7.72	7.92	8.23	8.44	15.08	21.18	22.88	23.54	24.82	25.03	25.63	.	.	27.26		27.99		
1	MD	M	2	4.29	4.9	5.33	5.81	6.28	6.4	7.03	7.01	7.27	7.52	7.5	7.59	7.97	8.37	8.88	14.3	19.69	21.51	22.1	23.89	23.66	24.32	.	.	25.59		26.69		
1	MD	M	3	4.4	4.95	5.63	6.05	6.49	7	6.99	7.02	7.31	7.36	7.43	7.78	8.16	8.6	8.88	15.48	20.58	21.89	22.79	23.98	24.51	24.68	.	.	25.79		27.48		
1	MD	F	4	4.33	5.06	5.59	5.98	6.38	6.8	6.93	6.98	7.16	7.33	7.38	7.47	7.65	7.2	8.39	13.39	17.59	19.93	19.19	20.23	20.01	21.51	.	.	23.2		23.52		
1	MD	F	5	3.85	4.36	4.72	4.93	5.21	5.4	5.46	5.63	5.93	6.12	6.2	6.18	6.3	6.56	6.63	.	.	.	.	.	.	.	.	.	.		.		
1	MD	F	6	4.49	5.02	5.69	5.98	6.58	6.7	6.87	7	7.24	7.32	7	7.58	7.96	8.2	8.45	13.5	16.88	18.35	18.73	19.32	20.25	21.94	.	.	.		.		
1	MD	F	7	4.04	4.53	5.11	5.68	6.03	6.2	6.52	6.6	6.82	7.11	7.17	7.3	7.4	7.56	7.85	12.77	16.2	17.53	17.74	19.19	19.28	20.36	.	.	22.08		22.38		
2	MD	M	1	3.85	4.25	4.57	5.05	5.1	4.9	5.09	5.16	5.42	5.63	5.8	5.89	6.05	6.51	6.9	10.3	15.74	20.32	21.78	22.69	23.29	24.42	.	.	25.18		26.08		
2	MD	M	2	4.34	4.88	5.56	6.04	6.08	5.9	6.14	6.46	6.6	7.02	7.13	7.23	7.48	8	8.7	13.65	19.16	20.88	22.25	23.31	23.68	24.55	.	.	26.78		25.88		
2	MD	F	3	4.17	4.82	5.47	6.06	6.23	6	6.35	6.65	7.01	7.38	7.45	7.62	7.84	8.12	8.69	11.77	15.58	17.73	18.74	19.18	19.8	21.2	.	.	23.38		22.96		
2	MD	F	4	3.99	4.73	5.33	5.76	5.99	5.9	6.17	6.46	6.92	7.28	7.28	7.44	7.8	7.97	8.69	13.18	16.35	17.94	16.68	20.08	19.55	20.62	.	.	22.32		21.43		
2	MD	F	5	4.16	4.81	5.48	6.03	6.03	5.9	6.19	6.42	6.65	7.06	7.18	7.32	7.58	7.8	8.28	12.28	15.46	16.85	17.8	19.13	19.78	21.06	.	.	21.85		22.08		
6	ETOH	M	1	4.1	4.6	5.1	5.46	5.8	5.8	5.84	5.7	6.07	6.21	6.5	6.68	6.95	7.5	7.88	12.6	18.58	20.17	21.43	23.18	23.56	24.68	.	.	25.28		25.87		
6	ETOH	M	2	4.12	4.49	5	5.33	5.24	5.4	5.5	5.56	5.69	5.97	6.18	6.31	6.68	7.22	7.54	11.67	17.9	19.98	21.08	22.4	23.49	24.06	.	.	25.88		26.16		
6	ETOH	F	3	4.45	5.03	5.49	5.87	6.14	6.3	6.2	6.07	6.16	6.18	6.39	6.53	.	7.51	7.98	13	16.5	17.68	18.02	19.75	19.77	20.97	.	.	22.88		22.59		
6	ETOH	F	4	3.9	4.35	4.81	5.07	5.33	5.5	5.63	5.74	5.79	5.9	6.07	6.07	6.35	6.79	7	10.79	14.86	17.08	17.68	18.3	17.82	19.14	.	.	20.78		20.88		
6	ETOH	F	5	4.1	4.64	5.16	5.58	5.78	6	6.1	6.08	6.1	6.11	6.24	6.53	6.84	7.31	7.48	.	.	.	.	.	.	.	.	.	.		.		
6	ETOH	F	6	4.28	4.74	5.12	5.55	5.47	5.7	5.76	6.16	5.82	5.81	5.92	6.18	6.65	7	7.49	11.61	14.98	16.58	16.89	19.11	18.93	19.87	.	.	20.52		20.79		
6	ETOH	F	7	3.34	3.77	4.13	4.4	4.43	4.4	4.53	4.61	4.85	5.2	5.46	5.5	5.77	6.23	6.34	.	.	.	.	.	.	.	.	.	.		.		
9	ETOH	M	1	4.1	4.48	4.9	5.27	5.4	5.76	6.3	6.31	6.57	6.73	6.79	6.96	7.47	8.28	8.53	14	19.48	21.39	22.69	24.23	24.95	25.83	.	.	26.96		27.76		
9	ETOH	M	2	4.23	4.67	5.15	5.5	5.56	6.05	6.37	6.85	7.15	7.16	7.39	7.27	7.57	8.2	8.53	14.31	20.12	21.23	22.51	23.38	24.28	24.78	.	.	26.38		26.8		
9	ETOH	M	3	4.08	4.52	4.92	5.26	5.4	6.06	6.27	6.64	7.03	7.04	7.24	7.33	7.71	8.24	8.88	13.66	17.98	20	21.09	22.86	23.38	24.68	.	.	25.49		25.88		
9	ETOH	M	4	4.22	4.82	5.14	5.57	5.6	5.89	6.09	6.52	6.99	7.06	7.22	7.18	7.4	8.62	8.49	13.18	19.54	20.74	22.08	23.38	24.06	25.06	.	.	26.18		26.49		
9	ETOH	M	5	4.12	4.59	4.98	5.26	5.3	5.68	6.19	6.46	6.91	6.88	6.95	7.11	7.53	8.04	8.37	.	.	.	.	.	.	.	.	.	.		.		
9	ETOH	F	6	4.37	4.93	5.5	5.63	5.9	6.32	6.61	6.92	7.26	7.37	7.3	7.28	7.61	8.25	8.48	12.78	16.6	17.75	19.31	20.28	20.88	21.76	.	.	22.49		22.78		
9	ETOH	F	7	4.18	4.6	4.98	5.18	5.4	5.78	6.31	6.61	6.93	7.14	6.96	7.21	7.42	7.98	8.1	12.38	16.02	18.12	18.89	19.88	19.998	20.75	.	.	21.44		21.78		
9	ETOH	F	8	3.92	4.45	4.78	5.01	5.2	5.52	6.03	6.32	6.68	6.76	6.76	6.82	7.05	7.7	7.96	12.08	16.01	17.01	17.85	19.02	18.98	19.7	.	.	20.18		20.66		
10	ETOH	M	1	4.49	5.13	5.45	5.68	5.7	5.99	6.07	6.15	6.18	6.25	6.37	6.56	7.02	7.46	7.68	12.2	19.92	20.89	21.38	22.88	24.08	24.79	.	.	26.48		26.88		
10	ETOH	M	2	4.3	4.8	5.18	5.3	5.3	5.48	5.7	5.93	6	5.97	6.04	6.24	6.66	7.13	7.74	12.98	18.45	19.3	20.58	21.61	22.52	23.64	.	.	25.09		26.14		
10	ETOH	M	3	4.43	4.87	5.36	5.58	5.8	5.98	5.66	6.31	6.34	6.54	6.68	6.57	6.83	7.25	7.98	12.73	20.02	21.52	22.49	23.38	24.13	24.58	.	.	25.88		26.2		
10	ETOH	M	4	4.28	4.76	5.09	5.38	5.4	5.6	6.06	5.77	5.85	5.86	4.88	5.84	6.33	6.72	7.28	11.65	17.25	19.18	21.18	20.89	22.09	22.52	.	.	23.98		25.2		
10	ETOH	M	5	4.42	4.9	5.28	5.6	5.7	5.7	5.8	6.12	6.2	6.32	6.37	6.48	6.78	7.12	7.79	.	.	.	.	.	.	.	.	.	.		.		
10	ETOH	F	6	4.48	4.9	5.3	5.63	5.8	5.89	6.11	6.28	6.33	6.44	6.43	6.6	7.23	7.82	8.18	12.42	15.94	17.6	18.19	18.5	20.28	20.83	.	.	22.18		21.78		
10	ETOH	F	7	4.05	4.44	4.95	5.11	5.3	5.36	5.52	5.68	5.77	6.07	5.88	5.99	6.08	6.28	6.68	10.09	14.13	15.68	16.09	16.82	17.8	18.78	.	.	19.62		19.48		
10	ETOH	F	8	4.43	4.89	5.24	5.36	5.4	5.56	5.78	5.81	5.78	5.8	5.86	6.08	6.28	6.98	7.48	11.6	15.28	16.58	17.48	18.4	18.52	20.22	.	.	21.26		19.98		
11	ETOH	F	1	4.1	4.64	5.08	5.32	5.3	5.63	5.71	5.84	5.88	6.02	5.87	5.87	6.11	6.3	6.59	10.09	13.55	14.89	16.18	16.6	16.79	17.55	.	.	18.38		19.86		
11	ETOH	M	2	4.25	4.75	5.32	5.66	5.7	5.86	6.06	6.47	6.34	6.55	6.33	6.28	6.38	6.58	6.76	9.78	15.46	19.69	20.17	22.36	23.72	24.26	.	.	25.74		26.59		
11	ETOH	M	3	3.48	3.89	4.38	4.63	4.6	5	5.06	5.18	5.6	5.46	5.34	5.34	5.35	5.33	5.56	.	.	.	.	.	.	.	.	.	.		.		
11	ETOH	M	4	3.47	3.77	4.19	4.28	4.3	4.32	4.38	4.65	4.68	7.4	4.71	4.72	4.8	4.9	5.03	.	.	.	.	.	.	.	.	.	.		.		
11	ETOH	M	5	3.98	4.44	5.08	5.19	5.2	5.6	5.59	5.85	6.08	6.08	6.05	6.02	6.18	6.6	7.18	11.07	17.36	20.28	21.73	23.98	24.2	25.52	.	.	27.08		22.78		
11	ETOH	M	6	4.14	4.62	5.06	5.32	5.4	5.57	5.64	5.85	5.82	6.12	6	6.16	6.38	7	7.28	11.29	17.58	18.68	19.82	20.33	20.94	21.99	.	.	23.22		23.39		
11	ETOH	M	7	5.22	4.7	5.3	5.56	5.6	5.78	5.92	6.15	6.18	6.18	6.19	6.28	6.75	6.98	7.11	.	.	.	.	.	.	.	.	.	.		.		
11	ETOH	M	8	3.68	4.09	4.53	4.88	5	5.06	5.3	5.31	5.43	5.53	5.62	5.68	5.99	6.44	6.64	11.38	17.18	19.08	20.39	21.88	22.79	23.85	.	.	25.42		26.4		
12	H2O	M	1	.	5.62	6.18	6.79	6.88	7.3	7.29	7.65	7.78	7.82	7.98	8.36	8.63	9	9.43	15.06	.	19.9	22.36	22.89	23.33	24.23	.	.	25.56		26.29		
12	H2O	M	2	.	4.93	5.51	6.2	6.21	6.6	6.5	6.76	6.94	6.96	7.13	7.5	7.87	8.04	8.52	13.9	.	18.38	21.09	21.65	22.46	23.09	.	.	24.22		24.87		
12	H2O	M	3	.	5.22	5.93	6.33	6.64	6.8	6.81	6.97	7.18	7.31	7.58	7.93	8.18	8.59	8.98	14.68	.	19.96	22.89	23.88	23.82	24.64	.	.	25.89		27.02		
12	H2O	F	4	.	5.22	5.83	6.37	6.47	6.9	6.87	7.21	7.43	7.36	7.49	7.77	8.09	8.3	8.48	13.57	.	16.9	19.87	20.19	21.21	21.68	.	.	22.49		22.39		
12	H2O	F	5	.	5.6	5.94	6.6	6.74	7	7.13	7.34	7.66	7.6	7.88	8.22	8.36	8.59	8.78	13.49	.	16.45	19.56	20.72	20.66	21.9	.	.	21.83		22.76		
13	MCT	M	1	4	4.62	5.29	5.65	6.28	6.65	6.6	6.83	7.06	7.36	7.75	7.62	8	8.47	8.8	16.83	20.81	22.49	23.28	.	24.78	25	.	26.08	.				
13	MCT	M	2	4.39	4.87	5.44	5.94	6.45	6.74	6.81	6.99	7.26	7.38	7.74	7.84	8.12	8.48	8.88	15.68	20.2	22.13	23.89	.	25.3	26.18	.	27.18	.				
13	MCT	F	3	4.05	4.6	5.04	5.53	6	6.38	6.36	6.63	6.89	6.88	6.99	7.15	7.6	7.73	8.16	13.6	17.19	19.41	19.23	.	20.28	20.19	.	20.88	.				22.46
13	MCT	F	4	4.07	4.71	5.06	5.44	5.98	6.36	6.35	6.57	6.64	7	7.08	7.13	1.17	7.49	7.99	.	.	.	.	.	.	.	.	.	.				
13	MCT	F	5	4.47	5.02	5.52	6.04	6.42	6.92	7.04	7.26	7.44	7.58	8	8.1	8.27	8.6	9.28	13.7	17.28	17.02	17.39	.	18.92	19.04	.	20.88	.				22.38
13	MCT	F	6	4.65	5.17	5.73	6.28	6.82	7.28	7.28	7.53	7.69	7.83	8.13	8.38	8.64	8.99	9.18	14.98	18.06	18.97	19.03	.	19.98	20.68	.	21.38	.				
13	MCT	F	7	4.36	4.8	5.4	5.8	6.3	6.68	6.61	6.82	6.83	7.08	7.29	7.48	7.84	8.38	8.72	13.48	15.86	20.69	20.09	.	22.08	22.17	.	22.88	.				21.68
14	MD	M	1	4.22	4.76	5.3	5.85	6.25	6.46	6.48	6.8	7.07	7.38	7.35	7.66	8.18	8.23	8.46	15.31	19.77	21.58	22.81	.	24.34	25.5	.	26.68	.				29.88
14	MD	F	2	4.12	4.61	5.32	6.08	6.31	6.73	6.96	7.46	7.72	8.19	8.39	8.59	8.38	8.78	9.08	14.08	17.23	19.78	19.98	.	20.8	21.36	.	21.52	.				23.35
14	MD	F	3	4.3	4.74	5.45	6.02	6.39	6.6	6.88	7.34	7.47	7.85	8.08	8.19	8.45	8.63	8.88	14.08	16.66	19.37	19.64	.	20.09	21.49	.	21.08	.				
14	MD	F	4	3.91	4.27	4.66	5.15	5.49	5.7	5.66	5.86	6.15	6.53	6.78	6.93	6.98	6.98	6.94	.	.	.	.	.	.	.	.	.	.				
14	MD	F	5	3.33	3.65	4.02	4.46	4.73	5.02	5.28	5.72	5.92	6.26	6.48	6.73	6.81	6.88	6.96	.	.	.	.	.	.	.	.	.	.				
14	MD	F	6	3.61	3.98	4.47	4.93	5.29	5.6	5.78	6.23	6.3	6.58	6.78	6.98	6.99	7.08	7.11	11.38	14.55	17.08	17.02	.	18.48	18.12	.	18.68	.				21.61
16	ETOH	M	1	4.4	4.8	5.3	5.6	6.01	6.47	6.63	6.96	7.24	7.46	7.64	7.89	8.08	8.33	8.48	.	.	.	.	.	.	.	.	.	.				
16	ETOH	M	2	4.46	4.65	5.12	5.5	5.83	6.28	6.38	6.62	6.98	7.09	7.21	7.5	7.95	8.6	8.98	14.79	19.21	20.41	21.81	22.85	23.62	23.62	24.06	24.49	25.18				25.98
16	ETOH	M	3	4.62	5.1	5.48	5.78	6.2	6.82	6.91	7.29	7.68	7.88	8.08	8.28	8.48	9.4	9.81	.	.	.	.	.	.	.	.	.	.				
16	ETOH	M	4	4.63	5.02	5.55	5.79	6.18	6.73	6.74	7.28	7.53	7.7	7.82	7.91	8.03	8.65	9.09	16.02	21.12	23.03	24.69	26.09	26.48	27.09	27.6	28.03	28.2				29.78
16	ETOH	M	5	4.6	5.03	5.42	5.79	6.21	6.8	6.94	7.18	7.19	7.43	7.71	7.98	8.28	8.65	9.08	15.72	20.98	22.48	23.86	25.58	26.25	27.23	27.98	28.83	29.04				30.87
16	ETOH	M	6	4.55	4.96	5.3	5.47	5.79	6.36	6.41	6.86	7.28	7.45	7.76	7.9	8.18	8.89	9.31	16.88	21.34	23.08	24.03	24.96	25.78	26.14	26.59	27.29	27.8				
16	ETOH	M	7	4.37	4.69	5.31	5.62	6	6.45	6.66	7.05	7.4	7.55	7.9	8.15	8.32	8.6	8.76	.	.	.	.	.	.	.	.	.	.				
17	ETOH	M	1	4.47	5.09	5.57	6	6.48	6.89	7.06	7.48	7.98	8.29	8.62	8.44	8.38	8.12	8.68	.	.	.	.	.	.	.	.	.	.				
17	ETOH	M	2	4.54	5	5.45	5.83	6.28	6.73	6.85	7.15	7.47	7.66	7.82	7.94	8.29	8.52	9.3	14.38	19.78	21.28	22.36	23.9	24.43	25.28	25.79	26.88	27.78				29.49
17	ETOH	M	3	4.83	5.22	5.7	6.13	6.53	7.05	7.37	7.62	8.08	8.03	8.17	8.4	8.66	7.86	9.34	15.3	21.35	22.06	22.99	23.68	24.09	25.18	25.18	26.08	26.45				28.2
17	ETOH	M	4	4.8	5.2	5.72	6.12	6.69	7.6	7.19	7.56	7.8	8.04	8.49	8.57	8.68	8.87	9.4	15.18	20.68	21.68	21.88	23.78	24.13	24.37	25.68	26.28	26.66				28.46
17	ETOH	M	5	4.68	5.17	5.82	6.24	6.74	7.46	7.79	8.38	8.53	8.42	8.8	8.98	9.06	9	9.78	.	.	.	.	.	.	.	.	.	.				
17	ETOH	M	6	4.41	4.8	5.46	5.78	6.23	6.72	6.67	7.15	7.47	7.68	7.86	7.89	7.99	7.13	8.68	.	.	.	.	.	.	.	.	.	.				
17	ETOH	F	7	4.5	4.84	5.27	5.53	5.78	6.01	6.13	6.4	6.72	6.9	7.1	7.22	7.23	7.24	7.88	12.27	16.68	19.28	18.58	19.69	20.35	21.28	21.58	21.53	22.08				23.1
17	ETOH	F	8	3.6	3.93	4.27	4.49	4.64	4.97	5.17	5.57	6.13	6.21	6.66	6.83	6.79	6.55	6.78	10.32	15.13	17.87	18.08	18.61	19.32	20.32	20.59	20.92	21.34				21.29
18	ETOH	M	1	4.42	4.94	5.48	5.97	6.62	7.17	7.39	8.09	8.47	8.82	8.88	8.69	8.79	8.75	8.68	14.5	19.52	21.64	24.12	26.07	26.88	28.02	29.3	29.38	30.48				32.39
18	ETOH	M	2	4.48	4.87	5.54	6.06	6.78	7.44	7.84	8.47	8.68	9.08	9.1	9.08	9.46	9.93	10.29	16.78	20.89	22.69	24.67	26.26	26.79	27.88	29.26	29.65	30.23				31.68
18	ETOH	M	3	4.38	4.62	5.25	5.84	6.5	7.02	7.42	8.2	8.38	8.71	8.75	8.56	8.66	9.05	9.38	15.58	20.56	21.89	23.16	24.63	25.28	26.75	27.16	27.88	29.08				32.08
18	ETOH	M	4	4.2	4.28	4.76	5.28	5.75	6.31	6.86	7.42	7.66	7.74	7.74	7.6	8.06	9.08	8.98	.	.	.	.	.	.	.	.	.	.				
18	ETOH	M	5	4.36	4.75	5.4	5.88	6.56	7.13	7.55	8.15	8.48	9.19	9.28	8.89	9.08	9.39	9.79	16.79	21.64	22.5	24.2	25.17	25.67	25.88	26.34	26.88	27.73				
18	ETOH	F	6	4.26	4.69	5.38	5.9	6.63	7.23	7.62	8.19	.	.	7.39	7.59	8.03	8.31	8.7	14.8	18.62	19.78	19.7	20.35	20.88	21.89	22.38	22.45	23.09				23.79
18	ETOH	F	7	4.01	4.39	5.05	5.52	6.29	6.63	6.89	7.49	7.48	8.12	7.96	7.89	8.07	8.43	8.68	13.69	16.19	17	17.93	18.75	18.98	19.9	21.28	21.3	20.59				21.42
19	MD	M	1	4.93	5.17	5.75	6.32	6.83	7.5	7.66	8.13	8.48	8.52	8.6	8.62	8.6	9.33	10.18	15.13	20.13	22.61	23.68	25.56	26.8	27.09	28.19	28.61	28.8				28.18
19	MD	M	2	4.46	4.89	5.48	5.87	6.44	6.82	6.97	7.36	7.49	7.68	8	8.02	8.22	9.22	10.27	16.88	20.47	20.8	22.37	23.73	24.76	25.36	26.11	25.79	26.48				27.85
19	MD	M	3	4.94	5.52	6.04	6.62	7.18	7.62	7.74	8.18	8.38	8.7	8.88	8.82	9.08	9.46	10.6	17.09	20.62	21.27	22.32	23.68	24.18	24.9	25.48	25.32	25.52				30.68
19	MD	F	4	3.98	4.37	4.86	5.25	6.01	6.74	6.82	7.23	7.44	7.68	7.69	7.49	7.56	8.09	8.73	13.9	16.98	17.56	18.52	19.48	19.92	20.52	20.58	20.77	21.18				22.26
19	MD	F	5	4.82	5.43	6.13	6.58	7.12	7.6	7.69	7.99	8.17	8.4	8.27	8.28	8.48	8.95	9.52	14.15	17.9	19.83	20.64	21.48	22.6	23.54	23.86	24.29	24.08				25.9
20	MCT	M	1	4.38	4.88	5.42	5.98	6.48	6.56	7.02	7.24	7.48	7.62	7.68	7.98	8.25	8.31	9.12	13.69	17.17	20.88	22.79	23.94	25.09	26.24	26.38	27.43	27.48				29.73
20	MCT	M	2	4.47	5	5.55	5.88	6.36	6.45	6.93	7.38	7.66	7.93	8.06	8.28	8.4	8.72	9.56	14.08	17.8	20.02	21.46	22.82	23.81	24.55	25.68	25.79	26.79				28.3
20	MCT	M	3	4.7	5.15	5.79	6.2	6.78	6.97	7.38	7.88	8.05	8.37	8.49	8.86	8.99	9.59	10.62	16.58	19.54	21.98	22.88	24.05	25.08	26.29	27.09	27.3	28.08				29.82
20	MCT	F	4	4.47	4.95	5.52	6.13	6.68	6.75	7.19	7.58	7.82	7.99	8.17	8.28	8.38	8.59	9.15	12.48	15.3	17.16	19	18.63	18.68	20.01	19.68	20.51	19.98				22.08
20	MCT	F	5	4.4	4.9	5.54	6.09	6.45	6.58	7.08	7.54	7.58	8.02	8.32	8.28	8.48	8.45	9.12	12.48	15.8	17.92	18.49	19.29	19.72	20.15	20.54	20.98	20.57				22.19
21	MCT	M	1	4.4	4.88	5.2	5.56	5.82	5.74	6	6.25	6.43	6.68	6.68	6.76	7.32	7.78	8.4	13.18	18.27	19.78	21.34	22.46	23.08	24.47	24.36	26.18	26.08				28.79
21	MCT	M	2	3.98	4.45	4.93	5.48	5.74	5.74	6.22	6.8	6.32	6.46	7.02	7.18	7.18	7.48	8.02	11.78	16.88	19.58	20.78	22.06	22.98	23.7	22.68	24.38	24.48				27.19
21	MCT	M	3	4.09	4.55	4.88	5.58	5.76	5.69	6.3	6.55	6.78	6.85	6.92	7.13	7.52	7.68	8.06	12.38	17.75	20.38	21.48	23.37	23.09	24.64	24.09	26.28	25.79				
21	MCT	M	4	4.12	4.47	4.9	5.44	5.68	5.74	6	6.36	6.38	6.68	6.71	6.88	7.2	7.42	8.13	13.05	18.32	20.3	21.01	22.13	22.38	23.42	23	24.78	25.26				27.14
21	MCT	F	5	3.89	4.35	4.67	5.24	5.44	5.4	5.88	6.23	6.37	6.42	6.47	6.69	6.79	6.94	7.56	12.43	15.9	18.55	18.79	18.9	19.48	20.02	20.89	20.7	20.38				22.18
21	MCT	F	6	4.23	4.64	5	5.48	5.7	5.67	6.16	6.47	6.48	6.68	6.71	6.88	7.29	7.38	7.95	12.48	15.21	16.6	17.34	18.21	18.97	19.68	20.08	20.45	20.88				20.98
21	MCT	F	7	3.94	4.3	4.86	5.3	5.55	5.66	6.01	6.46	6.6	6.56	6.7	6.78	7.18	7.2	7.67	12.06	15.32	17.28	17.26	18.56	18.35	18.82	19.63	19.63	19.57				
21	MCT	F	8	3.98	4.37	4.77	5.39	5.52	5.47	5.9	6.8	6.75	6.98	6.54	6.76	6.44	6.95	7.5	12.46	15.58	16.98	17.43	18.24	18.69	19.39	20.18	20.27	20.81				21.48
23	H2O	F	1	4.73	5.25	5.64	5.78	5.9	6.1	6.16	6.34	6.33	6.43	6.45	7.38	7.88	.	8.95	12.14	16.06	18.26	18.6	19.52	20.07	19.8	20.59	21.49	21.67				
23	H2O	M	2	4.54	5.03	5.58	5.83	6.07	6.4	6.59	6.66	6.68	6.52	6.44	6.42	7.71	.	9.12	14.66	19.95	21.98	22.98	23.78	24.13	25.53	24.88	26.68	26.88				29.4
23	H2O	M	3	4.8	5.32	5.8	6.08	6.2	6.8	6.82	6.86	6.88	6.85	6.73	7.63	8.28	.	9.7	15.08	19.6	21.4	22.27	23.23	23.61	24.78	24.66	25.69	25.89				26.89
23	H2O	M	4	4.86	5.32	5.77	5.9	6.3	6.5	6.76	6.84	6.79	6.76	6.82	7.9	8.58	.	9.98	15.42	19.65	20.98	22.49	23.38	23.68	24.78	24.78	25.13	25.79				
23	H2O	M	5	4.38	4.86	5.35	5.52	5.89	6.2	6.48	6.66	6.57	6.5	6.5	6.78	7.52	.	8.75	.	.	.	.	.	.	.	.	.	.				
24	MD	F	1	5.23	5.81	6.47	7.14	7.57	7.67	8.01	8.47	8.48	8.68	8.88	8.96	9.3	9.82	10.9	.	.	.	.	.	.	.	.	.	.				
24	MD	F	2	4.88	5.43	5.95	6.69	7.06	7.22	7.7	8.1	8.48	8.99	9.01	9.08	9.49	9.68	11.1	13.94	15.22	17.48	18.4	19.08	19.55	19.66	21.39	24.46	21.83				23.58
24	MD	F	3	5.2	5.7	6.18	6.83	7.34	7.58	7.96	8.34	8.28	8.54	8.68	9.02	9.24	9.88	10.5	14.41	17.28	19.08	18.82	19.58	19.98	19.91	20.08	20.96	20.88				21.68
24	MD	F	4	5.08	5.6	6.2	7.01	7.47	7.56	7.99	8.42	8.58	9.08	9.17	9.48	9.88	10.38	11.42	15.18	18.63	19.38	19.46	20.43	19.99	21.26	21.49	22.08	22.23				
24	MD	M	5	4.7	5.26	5.84	6.38	6.82	6.98	7.48	7.88	8.23	8.28	8.3	8.23	8.58	9.09	10.38	10.88	13.38	13.88	13.97	12.82	13.14	14.89	15.43	15.29	16.08				14.95
25	MD	M	1	3.8	4.24	4.57	4.98	5.24	5.44	5.83	6.2	6.34	6.5	6.57	6.68	7.15	7.58	8.57	15.46	18.69	20.57	21.62	22.75	23.15	24.42	24.46	24.79	24.63				26.56
25	MD	F	2	3.88	4.3	4.68	5.18	5.48	5.75	6.22	6.51	6.78	6.97	7.16	7.27	7.27	7.7	8.47	.	.	.	.	.	.	.	.	.	.				
25	MD	F	3	3.94	4.32	4.64	5.14	5.33	5.5	5.86	6.2	6.44	6.48	6.59	6.79	7.05	7.38	8.26	12.29	15.88	17.53	17.84	18.88	19.68	20.19	20.58	21.38	21.07				22.18
25	MD	F	4	3.88	4.28	4.68	5.2	5.6	5.77	6.25	6.38	6.68	6.64	6.74	6.92	7.12	7.36	8.62	11.91	14.47	16.52	16.38	17.29	18.09	17.89	18.66	19.32	19.77				20.68
25	MD	F	5	3.86	4.24	4.58	5.16	5.48	5.74	6.11	6.4	6.67	6.87	7.17	7.38	7.82	8.08	9.29	.	.	.	.	.	.	.	.	.	.				
25	MD	F	6	3.86	4.3	4.72	5.19	5.55	5.88	6.2	6.56	6.78	7.14	7.18	7.24	7.3	7.69	8.11	11.08	14.4	17.05	18.44	19.53	19.28	20	20.88	20.89	20.53				22.38
25	MD	F	7	3.91	4.37	4.75	5.25	5.65	5.85	6.2	6.67	6.8	6.88	6.98	7.22	7.58	7.79	8.85	13.01	15.95	18.88	18.42	19.8	19.35	20.08	21.56	21.08	21.18				23.26
26	MD	M	1	3.43	3.86	.	4.29	5.07	5.4	5.35	.	5.9	6.14	.	7.11	7.21	7.5	7.85	11.89	16.93	.	21.33	22.32	.	23.28	.						25.08
26	MD	M	2	4.68	5.23	.	5.8	6.7	7.1	7.09	.	7.85	7.88	.	8.79	8.89	9.26	9.94	15.58	19.19	.	21.83	22.98	.	25.16	.						28.49
26	MD	M	3	4.18	4.82	.	5.45	6.23	6.9	6.72	.	7.48	7.4	.	8.24	8.38	8.89	9.47	14.48	19.08	.	21.75	22.49	.	23.51	.						26.68
26	MD	F	4	4.46	5.08	.	5.67	6.45	6.9	7.93	.	7.32	7.44	.	8.05	7.88	8.58	8.78	12.38	14.59	.	17.62	18.78	.	19.98	.						
26	MD	F	5	4.33	5.05	.	5.5	6.45	6.8	6.84	.	7.43	7.5	.	8.22	8.46	8.89	9.34	14.59	17.28	.	17.99	19.43	.	20.26	.						21.03
26	MD	F	6	4.46	5.12	.	5.62	6.52	6.9	6.8	.	7.62	7.6	.	8.21	8.58	9.2	9.83	14.7	17.23	.	18.58	19.69	.	20.63	.						22.26
27	H2O	F	1	5.01	5.74	.	6.27	6.96	7.4	7.33	.	7.59	7.87	.	8.13	8.76	9.78	10.41	14.16	16.68	.	18.84	19.18	.	19.78	.						21.79
27	H2O	F	2	5.6	6.25	.	7.02	7.64	8.2	7.89	.	8.3	8.38	.	8.82	9.29	10	10.88	14.89	17.58	.	19.13	19.76	.	20.85	.						
27	H2O	F	3	4.66	5.31	.	6.01	6.61	6.9	6.75	.	7.15	7.42	.	7.89	8.38	8.78	9.38	13.49	16.08	.	17.88	18.88	.	19.67	.						22.18
27	H2O	F	4	5.38	5.92	.	6.69	7.33	7.6	7.43	.	7.52	7.74	.	8.7	8.88	9.62	9.99	14.76	17.59	.	17.88	18.54	.	19.38	.						21.52
27	H2O	M	5	5.46	6.11	.	6.86	7.24	7.7	7.63	.	7.95	8.33	.	8.97	9.39	10.56	11.26	17.78	20.66	.	23.32	24.01	.	25.94	.						29.86
28	ETOH	M	1	4.3	4.73	5.26	5.7	6.18	6.34	6.28	6.85	7.16	7.23	7.02	7.06	7.32	8.12	8.43	14.78	19.88	19.89	.	22.7	23.68	24.08	24.91	25.97	25.76	25.4	26.09	26.79	26.06
28	ETOH	F	2	4.21	4.58	5.08	5.49	5.9	6.46	5.99	7.08	7.23	7.2	7.08	7.18	7.32	7.88	8.1	12.38	16.38	17.88	.	18.89	20.06	20.52	21.59	21.75	22.48	22.22	21.98	22.97	22.86
28	ETOH	F	3	4.15	4.55	5.02	5.38	5.9	6.35	6.12	6.81	7.06	7.09	7.11	6.99	7.26	8.08	8.39	13.28	17.12	18.08	.	20.04	20.48	20.89	22.12	22.18	22.89	22.36	22.1	22.38	22.71
28	ETOH	F	4	3.94	4.26	4.83	5.2	5.54	6	5.51	6.4	6.61	6.89	6.68	6.48	6.6	7.57	7.73	.	.	.	.	.	.	.	.	.	.	.	.	.	.
28	ETOH	F	5	4.35	4.67	5.18	5.57	5.28	5.52	5.8	6.35	6.68	6.88	6.81	6.77	6.89	7.59	8.04	13.01	16.43	17.08	.	19.02	19.37	19.61	19.88	20.38	21.18	19.99	21.6	21.78	20.9
28	ETOH	F	6	4.16	4.65	5.2	5.56	6.17	6.43	6.22	7.09	7.34	7.44	7.37	7.29	7.76	8.51	8.98	.	.	.	.	.	.	.	.	.	.	.	.	.	.
28	ETOH	F	7	4.18	4.56	5.13	5.55	5.24	5.89	5.93	6.58	6.79	7.09	6.99	6.89	7.14	7.86	8.39	13.48	17.24	18.16	.	19.09	19.41	19.68	20.29	21.44	21.39	20.58	21.4	21.79	21.58
32	MCT	M	1	3.81	5.04	5.51	5.92	6.26	5.48	6.52	6.78	7.06	6.98	7.07	7.17	7.15	7.7	8.23	16.33	19.67	20.46	21.9	23.29	23.76	24.78	24.89	25.47	26.09	26.49	25.59	26.8	.
32	MCT	M	2	3.5	4.56	5.09	5.54	6.11	6.69	6.29	6.64	6.78	6.69	6.93	7	6.98	7.54	8.08	17.08	20.28	21	22.08	22.98	23.28	24.14	24.62	25.38	25.88	26.12	25.59	25.46	
32	MCT	M	3	3.8	4.84	5.32	5.72	6.12	6.7	6.59	6.81	6.9	6.89	7	7.09	7.26	7.88	8.38	17.17	21.28	22.46	23.38	24.88	24.58	26.42	26.3	27.16	27.96	27.83	27.27	27.08	
32	MCT	M	4	3.43	44.4	4.91	5.28	5.69	6.15	5.9	6.18	6.33	6.34	6.51	6.48	6.58	7.2	7.88	16.43	19.96	20.68	21.68	22.38	22.76	23.87	24.59	24.38	25.08	25.08	24.68	24.83	
32	MCT	F	5	3.42	4.47	5.06	5.37	5.78	5.82	6.1	6.42	6.62	6.59	6.63	6.7	6.6	6.8	7.2	14.66	17.04	17.23	18.48	19.16	19.68	20.79	20.32	21.06	21.09	21.05	21.59	21.15	
32	MCT	F	6	3.6	4.79	5.33	5.8	6.22	6.48	6.76	6.88	6.87	6.88	6.96	7.02	7.08	7.63	7.92	15.69	18.4	18.68	19.54	20.18	20.88	21.25	21.47	21.58	22.5	22.29	21.58	21.85	
32	MCT	F	7	3.52	4.6	5.02	5.37	5.77	6.08	6.12	6.46	6.49	6.68	6.65	6.67	6.58	7.09	7.48	14.28	16.29	17.56	18.28	19.09	19.23	20.47	20.48	20.95	21.89	21.57	21.58	21.93	
34	MD	M	1	2.92	2.98	3.1	3.37	3.76	4.23	4.47	4.76	5.01	5.39	5.62	5.68	5.78	.	6.42	10.8	16.54	19.01	20.02	20.71	21.87	22.3	22.78	23.16	23.66	23.8	23.83	24.32	
34	MD	M	2	3.11	3.26	3.32	3.66	3.91	4.36	4.56	4.86	5.09	5.28	5.52	5.56	5.72	.	6.48	10.52	16.03	18.72	19.98	20.58	21.66	22.54	23.06	23.18	23.48	23.93	24.27	24.43	
34	MD	F	3	3.03	3.04	3.13	3.53	3.67	4.16	4.38	4.7	4.76	4.84	5.4	5.44	5.58	.	6.42	10.24	14.76	16.06	16.58	17.08	17.88	18.83	17.14	19.91	19.69	20.32	20.38	20.52	
34	MD	F	4	2.76	2.88	2.99	3.38	3.47	3.9	4.1	4.44	4.61	5.21	5.12	5.24	5.37	.	5.98	.	.	.	.	.	.	.	.	.	.	.	.	.	
34	MD	F	5	2.88	3.02	3.2	3.41	3.72	4.16	4.39	4.58	4.76	5.03	5.38	5.37	5.5	.	6.17	9.64	13.35	14.54	15.99	16.53	17.33	18.6	18.06	18.69	18.82	19.38	19.48	19.9	
34	MD	F	6	3.08	3.06	3.18	3.62	3.78	4.21	4.43	4.7	4.88	5.09	5.36	5.44	5.51	.	6.47	10.03	14.73	16.45	17.03	17.6	18.54	19.09	19.59	20.08	20.08	20.08	20.62	20.68	
34	MD	F	7	2.84	2.98	3.09	3.17	3.57	4	4.22	4.58	4.7	4.98	5.35	5.38	5.51	.	6.04	9.49	13.76	16.64	16.94	17.29	17.73	18.58	18.83	19.8	19.8	20.28	20.18	20.19	
37	H2O	M	1	4	4.48	5.12	5.02	5.4	5.8	6.17	6.48	6.79	7.03	7.03	7.14	.	7.99	8.08	14.87	20.45	21.08	22.45	24.06	25.71	26.98	26.08	26.44	27.32	27.68	27.74	28.37	.
37	H2O	M	2	4	4.6	5.17	5.34	5.72	6.1	6.17	6.48	6.58	7.04	6.99	7.13	.	7.68	7.88	13.98	19.69	21.05	22.37	23.57	24.44	25.88	25.59	26	26.06	26.48	26.87	27.41	27.28
37	H2O	M	3	4.4	4.96	5.49	5.72	6.18	6.5	6.82	7.14	7.14	7.38	7.46	7.49	.	8.35	8.68	15.14	20.32	21.86	22.98	24.24	25.69	26.49	26.68	26.98	27.83	28.68	28.88	29.58	29.98
37	H2O	M	4	4.23	4.75	5.19	5.49	5.78	6.2	6.37	6.67	6.78	7.01	7.09	7.16	.	7.92	8.14	15.62	21.53	22.94	24.69	25.78	26.7	27.86	27.98	28.28	28.36	28.98	29.28	30.3	30.38
37	H2O	F	5	4.16	4.66	5.27	5.55	5.89	6.2	6.38	6.72	6.91	7.26	7.39	7.23	.	8.28	8.48	14.03	18.63	18.41	19.29	19.16	20.64	20.81	20.89	21.38	20.83	21.68	22.18	22.21	22.25
37	H2O	F	6	4.19	4.82	5.28	5.52	5.94	6.4	6.56	6.98	7.08	7.23	7.28	7.28	.	7.8	7.96	12.87	18.08	19.36	19.58	21.13	21.16	22.69	22.38	22.78	22.58	23.48	23.99	23.22	23.31
37	H2O	F	7	4.41	4.9	5.44	5.75	6.1	6.5	6.68	7.02	7.14	7.34	7.38	7.41	.	8.19	8.27	12.49	17.35	18.06	19.16	19.62	20.78	21.53	21.59	21.43	22.16	22.38	2.51	22.46	23
38	MD	M	1	3.41	3.92	4.46	4.79	5.22	5.6	5.88	6.1	6.29	6.6	6.67	6.68	.	7.28	7.44	12.69	19.06	19.96	21.18	22.24	23.32	24.08	24.1	24.34	25.24	24.98	25.78	26.78	.
38	MD	M	2	4.32	4.87	5.36	5.78	6.2	6.6	6.76	7.08	7.18	7.42	7.49	7.6	.	8.86	9.25	15.22	19.85	20.76	21.69	23.89	23.66	25.28	25.57	25.28	25.81	25.58	25.99	26.32	26.3
38	MD	M	3	4.2	4.76	5.38	5.58	6.06	6.4	6.54	6.78	6.98	7.32	7.33	7.4	.	8.68	8.79	14.42	19.94	20.35	21.89	23.53	24.34	25.9	25.78	26.16	26.36	26.29	26.92	27.78	27.58
38	MD	M	4	3.7	4.29	4.78	5.04	5.4	5.9	6	6.28	6.48	6.63	6.92	6.98	.	7.98	8.12	14.32	20.98	22.08	23.73	25.15	25.53	26.5	27.08	27.68	27.87	27.88	28.18	28.78	28.7
38	MD	F	5	3.82	4.38	4.8	5.09	5.59	6.1	6.3	6.58	6.78	6.97	6.98	7	.	7.58	7.84	12.48	16.46	17.09	17.8	19.28	19.66	20.48	20.73	20.88	21.6	21.28	21.87	21.68	22.12
38	MD	F	6	4.3	4.88	5.4	5.68	6.14	6.5	6.71	7.03	7.22	7.37	7.51	7.58	.	8.9	8.89	13.98	17.47	17.74	17.69	19.89	19.77	19.58	20.78	20.8	21.52	21.62	21.48	21.53	21.78
38	MD	F	7	3.89	4.5	4.92	5.28	5.68	6.1	6.26	6.43	6.68	6.89	6.98	6.92	.	8.02	8.03	12.68	16.93	17.3	17.67	18.13	19.35	19.61	20.68	20.99	22.29	21.42	22.08	21.76	22.56
41	ETOH	F	1	4.12	4.34	4.73	5.18	5.7	5.67	6.06	6.18	6.45	6.36	6.49	.	6.58	6.68	.	13.5	.	17.64	17.62	18.6	19.14	19.94	20.34	20.23	21.68	21.6	21.59	21.78	22.06
41	ETOH	M	2	3.8	4.06	4.48	4.8	5.4	5.39	5.7	5.92	6.2	6.33	6.33	.	6.48	6.53	.	.	.	.	.	.	.	.	.	.	.	.	.	.	.
41	ETOH	M	3	3.88	4.24	4.49	4.88	5.6	5.58	5.85	6	6.28	6.23	6.4	.	6.62	6.68	.	13.84	.	20.18	21.29	23.28	24.48	25.23	25.16	25.82	26.25	26.58	26.75	28.16	27.38
41	ETOH	M	4	3.87	4.31	4.58	4.98	5.6	5.7	6.05	6.27	6.59	6.68	6.67	.	6.79	7.01	.	.	.	.	.	.	.	.	.	.	.	.	.	.	.
41	ETOH	M	5	3.77	3.98	4.13	4.54	5.2	5.08	5.36	5.54	5.79	5.88	5.94	.	6	6.08	.	14.02	.	20.52	21.39	22.88	24.68	25.08	25.08	25.99	25.78	25.78	26.92	27.55	.
41	ETOH	M	6	4.05	4.48	4.78	5.2	5.7	5.88	6.18	6.37	6.66	6.78	6.82	.	7	7.12	.	14.75	.	20.97	22.68	24.69	26.07	26.84	27.6	28.08	28.38	28.38	28.69	29.68	28.96
41	ETOH	M	7	4.05	4.48	4.88	5.22	5.9	5.8	5.05	6.36	6.58	6.58	6.72	.	6.77	6.89	.	13.68	.	21.28	22.76	24.06	25.09	26.07	26.4	26.86	27.28	27.28	27.23	27.8	27.56
41	ETOH	M	8	3.48	3.93	4.16	4.6	5.2	5.22	5.62	5.82	6.02	6.08	6.1	.	5.98	5.98	.	.	.	.	.	.	.	.	.	.	.	.	.	.	.
42	ETOH	M	1	4.11	4.8	.	5.82	6.34	6.62	7.52	7.58	7.99	.	8.1	8.29	8.62	9.08	9.6	16.21	21.15	22.04	23.67	24.2	25.22	25.92	26.63	26.3	27.43	27.28	27.68	28.53	.
42	ETOH	M	2	3.93	4.48	.	5.58	6.14	6.33	7.01	7.23	7.2	.	7.73	7.78	8.16	8.53	8.93	15.04	20.54	21.89	23.86	24.38	25.78	26.38	27.28	26.98	27.71	27.28	27.99	28.52	.
42	ETOH	F	3	3.83	4.46	.	5.48	5.84	626	6.88	7.08	7.26	.	7.32	7.42	7.58	7.78	8.28	12.59	16.71	18.78	19.98	20.3	21.49	22.1	22.72	22.78	23.28	22.69	22.68	23.04	23.68
42	ETOH	F	4	3.79	4.42	.	5.55	6.12	6.36	7.17	7.16	7.38	.	7.7	7.62	7.79	8.38	8.67	13.68	16.65	17.78	19.08	19.48	20.16	20.78	21.57	2138	21.58	22.5	21.98	22.57	22.56
42	ETOH	F	5	3.64	4.29	.	5.32	5.83	6.22	6.99	7.08	7.36	.	7.59	7.59	7.74	8.18	8.37	12.72	16.07	17.39	18.98	18.83	20.18	20.69	21.5	20.82	21.27	20.67	21.28	21.75	21.62
42	ETOH	F	6	4.09	4.71	.	5.78	6.15	6.5	7.3	7.42	7.42	.	7.78	7.67	7.74	8.33	8.99	13.58	17.28	18.38	19	19.86	20.84	21.71	22.12	21.6	22.37	22.88	22.1	23.05	23.19
43	MD	M	1	3.76	4.17	.	4.98	5.28	5.63	6.08	6.2	6.29	.	6.48	6.54	6.78	6.76	6.76	12.42	18.52	20.56	21.87	22.96	23.96	24.95	25.12	25.46	25.33	25.48	25.88	26.27	27.68
43	MD	M	2	3.68	4.08	.	4.83	5.3	5.6	5.96	6.18	6.24	.	6.28	6.56	6.8	7.12	7.12	12.88	18.85	20.41	22	22.81	24	25.03	25.04	24.89	26.09	25.84	26.08	26.18	.
43	MD	M	3	3.5	3.92	.	4.74	5.09	5.62	5.87	6.11	6.24	.	6.49	6.58	6.68	6.79	6.79	11.69	17.14	19.22	21.48	22.77	24.18	25.03	25.92	25.73	26.09	25.68	26.88	27.08	27.68
43	MD	M	4	3.74	4.22	.	4.99	5.26	5.38	6.01	6.13	6.26	.	6.42	6.5	6.68	6.71	6.71	12.88	18.48	20.48	22	23.5	25.19	25.35	26.92	26.68	27.73	27.62	28.05	28.43	.

43	MD	F	5	3.79	4.22	.	5.05	5.39	5.79	6.05	6.17	6.34	.	6.37	6.5	6.58	6.78	6.78	12.39	15.87	17.53	19.28	19.93	20.02	21.38	20.86	20.9	22.28	23.6	22.46	21.96	22.88
43	MD	F	6	3.82	4.24	.	5.02	5.35	5.63	6.02	6.2	6.38	.	6.53	6.68	6.99	7.54	7.54	13.44	16.7	17.58	18.42	18.7	19.79	19.8	19.78	20.28	20.5	20.66	21.59	20.61	21.68
43	MD	F	7	3.85	4.2	.	4.88	5.33	5.62	5.96	6.08	6.22	.	6.22	6.35	6.7	7.03	7.03	12.03	15.72	16.98	17.98	18.68	19.71	19.46	19.73	19.52	20.18	20.13	20.78	20.63	21.28
45	MCT	M	1	4.18	.	4.96	5.23	5.66	6.11	6.43	6.51	.	7.09	6.69	6.8	6.82	6.93	7.66	13.48	19.62	21.22	21.88	22.61	24.1	24.99	25.54	25.8	25.58	25.96	26.69	26.75	.
45	MCT	M	2	4.31	.	5.02	5.44	5.71	6.1	6.13	6.5	.	6.68	6.55	6.61	6.72	6.98	7.73	13.56	19.45	21.28	22.03	23.49	23.19	23.91	24.78	25.08	25.03	24.98	25.55	25.98	.
45	MCT	F	3	4.29	.	5.06	5.44	5.82	6.42	6.49	6.68	.	6.88	6.77	6.83	7.19	7.08	8.02	12.41	15.84	19.03	18.61	19.56	20.68	20.72	20.98	21.58	21.82	22.09	22.18	23.08	.
45	MCT	F	4	4.28	.	4.99	5.34	5.67	6.2	6.17	6.5	.	6.53	6.42	6.5	6.74	6.93	7.52	12.48	16.38	18.39	19.48	19.84	20.81	21.31	21.68	21.99	22.58	23.08	22.68	23.53	.
45	MCT	F	5	4.08	.	4.76	5.04	5.28	5.88	6.05	6.13	.	6.23	6.16	6.3	6.24	6.41	7.08	11.78	15.58	17.57	18.78	19.07	20.67	21.28	21.52	22.46	22.88	22.56	22.78	22.68	.
45	MCT	F	6	4.04	.	4.92	5.28	5.64	6.22	6.38	6.58	.	6.7	6.6	6.52	6.7	6.89	7.38	12.17	15.63	18.08	17.79	18.98	19.46	19.59	20.58	20.6	20.59	21.01	21.48	21.62	.
45	MCT	F	7	3.9	.	4.68	4.88	5.16	5.72	5.81	5.88	.	5.98	5.83	6.03	6.04	6.18	6.86	.	.	.	.	.	.	.	.	.	.	.	.	.	.
45	MCT	F	8	4.13	.	4.93	5.33	5.62	6.12	6.2	6.48	.	6.53	6.49	6.58	6.61	6.88	7.48	.	.	.	.	.	.	.	.	.	.	.	.	.	.
46	H2O	M	1	3.78	4.08	4.93	5.23	5.82	6	6.44	6.4	6.58	6.59	6.63	6.83	7.18	7.1	7.75	14.79	18.83	20.8	22.4	23.88	24.69	25.2	25.48	24.98	24.93	26.28	26.28		
46	H2O	M	2	3.99	4.55	5.26	5.65	6.13	6.37	6.78	6.8	7.01	7.09	7.17	7.3	7.69	7.8	8.52	15.2	19.19	21.14	22.68	23.49	24.58	25.06	25.55	25.86	25.98	26.88	27.39		
46	H2O	M	3	4.07	4.5	5.26	5.62	6.19	6.38	6.86	6.79	6.95	6.96	6.87	6.89	7.18	7.5	7.41	14.3	18.64	20.66	21.68	22.86	24.08	24.22	24	24.08	24.23	25.48	.		
46	H2O	M	4	3.71	4.13	4.84	5.23	5.7	6.06	6.29	6.31	6.55	6.57	6.56	6.46	6.58	7	8.2	14.21	19.07	21.34	22.45	23.82	25.34	25.5	25.08	24.98	25.42	26.78	26.95		
46	H2O	F	5	3.6	4.13	4.73	5.07	5.54	5.85	6.19	6.34	6.55	6.51	6.52	6.6	6.88	6.7	7.42	12.48	15.58	17.53	18.09	18.98	19.67	19.48	19.4	21.19	20.91	21.03	21.28		
46	H2O	F	6	4.12	4.48	5.14	5.41	6.08	6.37	6.67	6.65	6.83	6.96	7.02	7.16	7.33	7.6	8.05	13.9	16.69	18.06	18.58	19.86	20.15	19.99	19.96	20.28	20.59	21.6	21.68		
46	H2O	F	7	3.85	4.18	5.04	5.25	5.76	6.09	6.46	6.47	6.77	6.7	6.84	6.93	7.28	7.3	8.12	13.86	16.55	17.74	18.08	18.99	19.2	19.79	19.91	20.2	20.74	20.97	21.68		
47	MCT	M	1	4.41	5.12	5.56	6.03	6.35	6.82	6.98	7.54	7.73	7.77	7.66	7.48	7.6	7.83	8.31	13.08	17.25	18.82	19.08	20.22	20.77	21.88	21.88	22.6	22.58	22.7	.		
47	MCT	F	2	4.69	5.48	5.84	6.48	6.61	7.33	7.46	7.86	8.18	8.18	8.13	8.13	8.2	8.48	8.61	15.54	19.08	2.159	23.14	24.67	25.78	25.89	26.28	27.18	27.15	.	.		
47	MCT	F	3	4.58	5.32	5.81	6.51	6.84	7.15	4.22	7.71	7.94	8.03	8.03	7.86	8.1	8.49	9.14	15.84	20.68	22.73	24.79	25.96	27	27.49	28.31	28.6	29.07	28.8	.		
47	MCT	F	4	4.62	5.46	5.68	6.1	6.52	6.98	6.98	7.34	7.48	7.45	7.27	7.32	7.6	8.24	8.68	14.74	18.69	20.26	21.69	23.42	23.99	24.58	25.19	25.79	26.5	26.76	.		
40	MCT	F	1	4.55	4.78	5.29	5.49	5.9	6.2	6.38	6.5	6.58	6.43	6.52	7.1	7.73	8.43	8.56	13.68	17.18	18.48	19.33	20.45	.	21.93	21.85	21.83	22.66	23.08	23.46	23.18	
40	MCT	F	2	4.57	5.13	5.58	5.6	5.95	6.35	6.49	6.63	6.74	6.79	6.9	7	7.3	7.97	9.12	12.19	15.88	17.89	18.88	20.12	.	20.29	21.08	21.08	22.1	22.76	22.44	23.05	
40	MCT	F	3	4.5	5.2	5.63	5.82	6.25	6.48	6.74	6.87	6.93	7	6.98	7.3	8.03	8.61	9.3	14	17.32	18.08	19.09	20.58	.	21.19	21.58	21.78	21.88	22.78	22.99	23.09	
40	MCT	F	4	4.35	4.88	5.32	5.53	5.96	6.35	6.49	6.57	6.72	6.62	6.5	7.1	7.61	8.32	9.22	14.48	17.28	17.89	18.78	19.44	.	20.08	20.68	20.98	20.97	21.34	21.38	21.5	
40	MCT	F	5	4.62	5.06	5.46	5.68	5.93	6.26	6.46	6.6	6.65	6.68	6.58	7	7.54	8.31	8.96	.	.	.	.	.	.	.	.	.	.	.	.	.	
40	MCT	M	6	4.69	5.16	5.54	5.76	6.25	6.46	6.61	6.8	6.78	6.54	6.66	7.2	7.82	8.51	9.41	.	.	.	.	.	.	.	.	.	.	.	.	.	
44	H2O	M	1	4.22	4.75	5	5.32	5.59	5.89	6.16	6.16	5.61	5.44	5.19	5.5	5.9	6.54	5.73	12.69	16.89	.	22.48	24.52	25.75	26.61	27.62	28.28	29.18	29.93	29.83	30.01	
44	H2O	M	2	4.48	4.92	5.2	5.48	5.83	6.19	6.38	6.59	6.04	5.72	5.55	5.5	6.12	6.7	7.37	12.69	17.62	.	22.68	2506	26.55	26.73	27.08	28.23	28.45	29.88	30.15	29.98	
44	H2O	M	3	4.07	4.41	4.82	5.1	5.45	5.75	5.98	6.05	5.59	5.24	5.1	4.9	6.26	5.21	5.5	.	.	.	.	.	.	.	.	.	.	.	.	.	
44	H2O	M	4	4.28	4.75	4.92	5.12	5.49	5.77	5.94	6.02	5.56	5.28	5.04	4.8	4.56	5.13	5.6	.	.	.	.	.	.	.	.	.	.	.	.	.	
44	H2O	M	5	4.38	4.73	5.11	5.4	5.8	6.04	6.26	6.32	5.82	5.39	5.13	5.5	6.13	6.77	7.37	12.34	17.35	.	22.88	25.2	26.66	27.46	27.8	28.73	29.58	30	30.29	30.61	
44	H2O	F	6	4.05	4.5	4.92	5.12	5.56	5.91	6.15	6.32	579	5.43	5.29	5.6	6.12	6.73	7.4	11.68	15.08	.	18.3	19.48	20.49	20.24	20.81	21.49	21.28	22	21.36	21.72	
44	H2O	F	8	4.47	5.02	5.23	5.49	5.86	6.14	6.38	6.33	5.89	5.56	5.3	5.7	5.03	6.75	7.32	9.38	14.26	.	17.13	18.13	20.08	19.62	20.42	20.56	21.18	21.98	21.68	21.46	
50	H2O	M	1	.	4.27	4.58	4.83	5.18	5.56	5.7	5.92	6.08	6.23	6.09	6.23	6.77	7.29	.	.	20	22.29	23.24	24.9	25.89	25.83	26.38	26.62	26.49	27.96	27.68	28.24	
50	H2O	M	2	.	4.37	4.76	4.9	5.4	5.52	5.86	6.15	6.3	6.44	6.48	6.58	6.91	7.53	.	.	18.34	21.09	22.74	24.45	25.42	25.48	26.36	26.8	26.98	27.89	28.17	29.06	
50	H2O	M	3	.	4.46	4.88	5.09	5.46	5.76	6.01	6.21	6.3	6.49	6.44	6.17	6.89	6.94	.	.	20.12	21.89	22.94	24.04	24.46	24.39	24.68	24.92	25.38	26.38	25.88	26.19	
50	H2O	F	4	.	4.09	4.47	4.93	5.15	5.44	5.65	6.01	6.21	6.41	6.38	6.8	6.58	7.44	.	.	15.76	17.38	17.68	18.74	19.76	19.68	19.68	20.68	20.83	20.69	20.89	21.18	
50	H2O	F	5	.	4.39	4.7	4.92	5.24	5.56	5.98	6.26	6.53	6.58	659	6.37	6.35	7.22	.	.	14.88	18.28	18.88	20.16	21.08	21.03	21.68	21.54	23.09	23.39	22.89	23.93	
50	H2O	F	6	.	4.18	4.55	4.79	5.15	5.47	5.81	6.04	6.25	6.35	6.28	6.21	6.3	6.88	.	.	16.58	17.88	18.78	19.46	20.22	20.33	20.49	21.48	21.77	21.39	21.83	21.99	
50	H2O	F	7	.	4.36	4.73	4.98	5.46	5.8	6.05	6.38	6.53	6.61	6.59	6.4	7.13	8.02	.	.	16.02	18.18	19.05	20.08	20.8	21.38	21.2	21.5	22.16	22.54	22.98	23.22	
50	H2O	F	8	.	3.94	4.33	4.57	4.97	5.25	5.6	5.93	6.05	6.21	6.26	6.58	6.16	7.86	.	.	.	.	.	.	.	.	.	.	.	.	.	.	
52	MD	M	1	3.91	4.25	4.57	5.12	5.53	6.13	6.57	6.78	6.88	6.96	7	7.28	7.48	8.25	8.42	14.23	18.28	21	22.3	24.43	25.02	25.28	25.88	26.4	26.09	26.59	27.18	27.43	
52	MD	M	2	4.2	4.46	4.71	5.13	5.57	6.14	6.57	6.75	6.98	7.12	7.2	5.52	7.82	8.23	8.8	16.2	20.33	22.49	23.37	25.2	25.89	26.68	27.45	27.09	27.38	27.58	28.18	28.78	
52	MD	M	3	4.42	4.89	5.06	5.48	6.1	6.58	7	7.07	7.24	7.28	7.3	7.6	8.08	8.32	8.98	16.29	20.48	22.88	23.58	24.26	25.7	25.9	26.28	25.85	27	27.09	27.58	28.06	
52	MD	M	4	4.27	4.6	4.8	5.2	5.67	6.18	6.7	6.91	6.96	6.94	7	7.16	7.49	7.9	8.43	14.78	18.84	21.3	22.41	23.84	24.28	24.68	24.74	25.04	25.34	25.98	26.82	26.78	
52	MD	F	5	4.38	4.86	4.98	5.56	5.95	6.41	6.86	7.16	7.33	7.36	7.4	7.58	7.99	7.95	8.94	13.53	16.47	18.68	19.03	19.56	20.68	20.59	20.16	21.38	21.68	23.08	22.89	22.1	
52	MD	F	6	4.09	4.35	4.65	5.03	5.53	6.1	6.4	6.61	6.72	6.81	6.9	6.9	7.32	7.71	8.23	12.68	16.28	17.81	18.55	19.94	21.08	20.61	20.43	20.78	20.78	22.35	21.37	21.37	
53	ETOH	M	1	4.37	4.84	5.17	5.46	6.02	6.46	6.08	6.29	6.38	6.38	6.81	6.84	6.91	7.63	7.96	.	19.08	20.53	22.48	22.54	24.99	24.89	25.56	25.38	25.78	26.29	27.09		
53	ETOH	M	2	4.74	5.21	5.74	6.14	6.61	7	6.59	6.79	6.93	7.08	7.17	7.08	7.2	8.37	9.12	.	19.79	20.28	22.6	22.35	26.2	24.98	26.28	26.36	26.64	27.58	28.58		
53	ETOH	F	3	3.99	4.32	4.84	5.1	5.65	6.04	5.8	5.88	5.91	6	6.07	6.12	6.75	6.9	7.31	.	.	.	.	.	.	.	.	.	.	.	.		
53	ETOH	F	4	4.42	4.81	5.23	5.52	6.13	6.51	6.15	6.3	6.18	6.18	6.48	6.45	7.02	7.61	8.12	12.76	16.68	18.01	18.89	19.34	20.46	19.86	21.26	20.88	21.68	22.7	22.38		
53	ETOH	F	5	4.49	4.8	5.3	5.59	6.15	6.67	6.25	6.33	6.49	6.6	6.82	6.71	7.17	7.63	7.98	12.08	15.88	17.38	18.49	18.92	19.45	19.46	19.78	18.98	20	20.78	20.68		
53	ETOH	F	6	4.2	4.7	5.12	5.47	6.06	6.44	6.15	6.18	6.31	6.45	6.4	6.5	7.78	7.23	7.67	11.96	18.29	19.24	19	19.88	20.13	19.93	20.9	20.77	20.88	21.39	21.39		
53	ETOH	F	7	4.28	4.74	5.16	5.6	6.2	6.54	6.28	6.33	6.57	6.48	6.65	6.74	6.63	7.3	7.95	.	.	.	.	.	.	.							
62	H2O	F	1	4.14	4.65	5.24	5.78	6.08	6.4	6.67	7.1	.	7.76	7.79	7.62	8.06	8.52	9.94	13.39	17.38	.	19.18	19.45	19.76	20.4	21.24	21.49	21.46	21.45	22.89	22.43	22.61
62	H2O	M	2	4.03	4.8	5.06	5.48	5.8	6	6.55	7.07	.	7.78	7.78	7.58	7.86	8.65	10.43	.	.	.	.	.	.	.	.	.	.	.	.	.	.
62	H2O	M	3	4.27	4.9	5.57	6.23	6.6	7	7.14	7.42	.	8.37	8.46	8.23	8.68	9.2	11.03	.	.	.	.	.	.	.	.	.	.	.	.	.	.
62	H2O	M	4	4.4	4.98	5.32	5.88	6.23	7	7.22	7.59	.	8.38	8.4	8.55	8.26	9.48	10.5	17.36	21.8	.	24.93	24.65	24.02	26.52	26.68	27.48	28.58	28.02	27.19	28.78	.
62	H2O	M	5	4.16	4.88	5.34	6.11	6.44	6.4	7.34	7.77	.	8.61	8.72	8.68	8.97	9.49	11.62	17.76	21.82	.	24.74	25.03	26.28	27.16	27.21	28.3	29.69	25.98	27.19	28.78	.
62	H2O	M	6	4.09	4.73	5.26	5.85	6.26	6.5	6.88	7.58	.	8.32	8.32	8.25	8.77	9.68	10.95	16.1	20.98	.	23.78	23.19	23.48	24.59	24.73	25.42	26.29	25.7	29.48	26.48	.
62	H2O	M	7	4.1	4.75	5.05	5.7	6.47	7	6.94	7.3	.	8.08	8.08	7.93	8.25	8.81	10.92	15.38	20.18	.	22.48	22.24	22.58	24.21	24.54	25.04	25.75	29.73	27.03	25.33	.
63	H2O	M	1	3.53	3.76	4.37	4.87	5.1	5.44	5.39	.	6.08	6.25	6.87	6.29	6.38	.	7.45	13.4	13.4	.	20.38	23.68	24.44	24.05	26.66	26.48	27.23	27.72	29.25	29.52	.
63	H2O	M	2	3.43	3.8	4.3	4.75	5.3	4.97	5.65	.	6.48	6.77	6.71	6.88	7.1	.	8.55	11.68	11.68	.	18.49	21.88	22.25	22.95	23.94	24.4	24.77	24.89	26.25	26.54	.
63	H2O	M	3	3.51	4.01	4.54	5.17	5.3	5.64	6.01	.	6.78	6.8	6.97	6.85	7.18	.	8.43	12.96	12.96	.	19.79	23.55	23.94	24.66	25.96	26.2	26.29	26.59	26.54	28.44	28.99
63	H2O	F	4	3.48	3.9	4.66	5.07	5.4	5.93	6.33	.	6.99	7.09	7.3	7.28	7.42	.	8.65	.	.	.	.	.	.	.	.	.	.	.	.	.	.
63	H2O	F	5	3.42	3.79	4.39	4.98	5.3	5.57	5.8	.	6.46	6.53	6.9	6.6	6.85	.	8.01	10.52	15.99	.	17.42	17.52	18.21	20.26	19.18	19.23	19.62	20.43	20.78	21.04	20.53
63	H2O	F	6	3.38	3.89	4.52	4.92	5.3	5.65	6.14	.	6.98	7.02	7.05	6.86	7.04	.	7.82	9.77	15.88	.	18.08	18.3	19.06	19.64	20.91	20.98	20.91	21.37	21.7	21.74	.
63	H2O	F	7	3.33	3.78	4.43	4.96	5.1	5.28	5.7	.	6.03	6.11	7.24	6.4	6.5	.	7.74	10.24	16.45	.	17.21	17.51	18.02	18.34	19.63	19.48	19.71	19.47	20.22	20.15	22.7
64	MD	M	1	4.91	5.72	6.3	6.78	7.39	7.91	.	8.98	9.41	9.7	9.76	9.98	.	10.46	.	15.88	19.04	.	21.58	23.04	23.63	25.82	26.13	26.09	27.48	29.15	29.18	29.12	.
64	MD	M	2	4.85	5.84	6.32	7.03	7.51	7.94	.	9.14	9.38	9.85	10	10.02	.	10.97	.	17.98	21.25	.	23.19	24.71	25.25	27.28	27.08	26.79	27.29	28.73	29.08	29.93	30.7
64	MD	M	3	5	5.96	6.47	7.12	7.59	8.3	.	9.33	9.53	9.79	9.94	10.24	.	10.84	.	17.14	20.2	.	22.18	24.3	24.27	26.19	26.23	27.09	28.09	29.54	29.77	29.89	.
64	MD	F	4	4.88	5.67	6.26	6.82	7.36	7.78	.	8.7	9.19	9.45	9.78	9.86	.	10.5	.	15.09	16.48	.	19.39	19.23	19.9	20.43	20.89	21.88	22.58	23.29	23.38	23.69	23.76
64	MD	F	5	4.68	5.45	5.85	6.28	6.8	7.34	.	8.39	8.72	8.98	9.18	9.08	.	9.86	.	15.58	17.88	.	19.48	19.01	20.35	20.92	21.08	21.79	21.78	22.39	22.48	23.01	.
65	MCT	M	1	3.99	4.26	4.81	5.16	5.3	5.64	6.12	.	6.87	6.88	7.38	7.58	7.78	.	9.34	13.68	19.73	.	21.73	22.29	23.14	23.41	23.56	24.47	24.69	24.78	25.52	25.98	26.02
65	MCT	M	2	3.66	4	4.61	4.99	5	5.52	5.96	.	6.71	6.23	6.84	7.03	7.4	.	8.12	13.46	18.83	.	21.12	21.46	21.56	22.67	23.49	24.28	24.88	24.39	24.98	25.03	27.13
65	MCT	M	3	3.74	4.03	4.78	5.09	5.3	5.74	6.15	.	6.97	6.82	7.32	7.6	7.7	.	8.92	14.08	20.09	.	21.95	22.41	23.86	24.21	25.33	25.68	26.36	26.2	26.65	27.38	28.01
65	MCT	M	4	3.65	4.11	4.87	5.23	5.3	5.78	6.22	.	7.3	7.06	7.47	7.98	8.24	.	9.33	13.68	18.69	.	20.45	21.27	21.93	23.22	23.02	23.86	25.1	24.84	26.03	25.87	25.86
65	MCT	F	5	3.7	4.1	4.87	5.08	5	5.59	5.05	.	6.9	6.58	6.9	7.26	7.68	.	8.61	12.02	16.27	.	17.64	18.48	18.97	19.3	20.31	19.68	19.36	19.9	20.14	20.56	20.29
65	MCT	F	6	3.78	4.37	4.83	5.21	5.3	5.77	6.16	.	7.05	6.91	7.39	7.59	7.63	.	9.08	12.09	15.23	.	16.96	17.56	17.73	15.54	19.22	19.68	19.55	20.29	20.61	20.38	20.45
65	MCT	F	7	3.93	4.28	4.99	5.4	5.7	6.1	6.51	.	7.24	6.2	7.53	7.8	8.05	.	9.35	13.16	17.68	.	18.56	19.42	19.91	21.35	21.13	22.06	22.06	21.94	22.24	21.78	22.58
68	MCT	M	1	4.55	4.64	5.09	5.48	.	6.4	6.28	6.64	6.63	6.74	.	9.42	8.06	8.78	9.39	14.28	18.47	19.15	2067	21.57	22.3	22.68	23.13	23.38	23.28	23.99	24.84	.	.
68	MCT	M	2	5.56	6.11	6.62	7.17	.	9	8.56	8.89	9.12	9.69	.	9.78	10.2	11.19	11.84	18.87	23.38	23.93	26.48	27.1	27.87	29.97	30.78	32.28	32.28	33.51	34.98	.	.
68	MCT	F	3	5.54	6.17	6.79	7.48	.	8.2	9	9.48	9.38	10	.	9.83	9.58	10.28	10.92	15.38	18.03	19.58	20.64	20.48	21.28	21.68	21.72	23.08	22.08	22.89	23.68	.	.
68	MCT	F	4	4.99	5.5	6.12	6.66	.	8.8	8.27	8.54	8.62	9.1	.	9.86	10.08	10.88	11.27	14.62	16.68	17.93	18.23	19.52	19.7	20.76	21.04	24.3	22.89	24.08	23.78	.	.
68	MCT	F	5	5.21	5.71	6.33	6.97	.	8.3	8.42	8.76	8.82	9.36	.	9.56	9.88	10.28	10.89	14.87	17.3	18.66	19.03	19.91	2034	21.74	21.8	21.94	22.62	23.68	22.89	.	.
69	H2O	F	1	4.25	4.72	5.04	5.65	.	7	6.39	6.69	6.92	7.12	.	7.48	7.68	8.22	8.74	13.37	17.57	17.71	18.64	19.65	20.39	20.89	21.38	22.49	22.15	23	23.28	.	.
69	H2O	F	2	3.8	4.16	4.49	4.93	.	6.2	5.96	6.13	6.43	6.71	.	6.99	7.36	7.61	8.13	12.89	17.33	18.39	19.09	19.29	20.64	20.97	22.18	22.47	22.48	23.08	23.22	.	.
69	H2O	F	3	4.01	4.3	4.62	5.05	.	6.8	6.04	6.24	6.42	6.59	.	6.98	7.15	7.7	8.18	12.18	16.77	17.45	18.48	20.86	18.81	19.41	19.87	20.78	20.69	20.8	21.39	.	.
69	H2O	M	4	4.17	4.66	4.99	5.33	.	6.4	6.14	6.59	6.68	6.99	.	7.39	7.48	8.08	8.65	13.46	19.3	20.65	21.48	22.67	22.13	24.89	25.43	26.27	26.2	26.73	27.12	.	.
69	H2O	M	5	4.14	4.58	4.87	5.46	.	6.9	6.46	6.76	6.97	7.2	.	7.88	8.09	8.58	9.19	14.81	20.55	21.3	22.9	23.32	24.22	25.58	26.27	26.76	27.48	27.69	27.88	.	.
69	H2O	M	6	4.2	4.41	4.84	5.3	.	6.6	6.22	6.36	6.68	7.05	.	7.56	7.69	8.02	.	.	.	.	.	.	.	.	.	.	.	.	.	.	.
69	H2O	M	7	4.08	4.44	4.84	5.2	.	6.3	5.98	6.38	6.58	6.75	.	7.29	7.35	7.93	8.5	11.88	16.98	18.88	20.2	21.18	23.82	22.78	23.92	24.99	25.18	25.13	25.38	.	.
69	H2O	M	8	4.2	4.56	4.91	5.36	.	7.4	6.26	6.53	6.82	7.02	.	7.48	7.73	8.22	8.89	13.68	19.18	19.85	20.86	21.87	22.9	23.9	24.48	24.98	25.16	25.68	25.42	.	.
71	ETOH	M	1	4.76	.	5.8	6.22	6.47	6.7	6.95	7.32	7.4	7.87	7.9	8.03	8.78	9.33	.	.	.	.	.	.	.	.	.	.	.	.	.	.	.
71	ETOH	M	2	4.72	.	5.78	6.11	6.29	6.7	6.66	7.23	7.23	7.72	7.73	7.89	8.69	9.16	9.98	15.43	20.89	21.2	22.46	23	23.8	25.48	26.87	26.03	26.05	27.18	.	.	.
71	ETOH	M	3	4.68	.	6.1	6	6.33	6.7	6.99	7.35	7.53	7.95	8.13	8.18	8.89	9.25	9.85	16.01	20.57	20.84	21.98	23.22	23.92	24.28	25.77	25.68	25.72	26.78	.	.	.
71	ETOH	M	4	4.68	.	5.99	6.36	6.63	6.7	7.18	7.75	7.76	8.26	8.33	8.6	9.07	9.97	10.85	17.8	21.35	22.03	23.57	24.17	24.45	25.62	25.65	25.03	26.81	27.36	.	.	.
71	ETOH	M	5	4.8	.	5.76	6.42	6.65	6.9	7.25	7.58	7.66	8.2	8.42	8.66	9.28	9.9	10.74	17.5	21.13	21.34	23.01	24.36	25.03	25.78	26.62	26.01	26.93	27.38	.	.	.
71	ETOH	F	6	4.53	.	5.65	6.01	6.34	6.9	6.83	7.19	7.27	7.65	7.74	7.87	8.39	9.1	9.8	14.91	18.15	18.48	18.81	19.13	19.44	20.48	21.34	21.29	22.53	22.88	.	.	.
71	ETOH	F	7	4.5	.	5.73	6.12	6.53	6.6	6.93	7.33	7.58	8.08	8.11	8.2	8.37	9.03	9.72	14.58	17.7	17.85	19.14	19.41	19.98	21.34	21.38	21.94	23.33	23.42	.	.	.
71	ETOH	F	8	4.57	.	5.83	6.23	6.51	6.8	7.05	7.4	7.68	7.98	7.98	8.04	8.48	9.12	9.98	14.63	17.78	17.71	18.21	19.37	19.97	21.09	21.84	21.48	22.08	22.75	.	.	.
74	H2O	M	1	4.03	4.37	4.85	5.44	5.88	6.02		7.08	7.08	7.38	7.38	7.59	8.47	9.08	9.43	14	17.96	20.37	21.04	22.57	24.68	24.78	25.88	26.39	27.24	27.68	28.32	28.08	.
74	H2O	M	2	4.1	4.38	4.97	5.48	5.9	6.08		6.97	7.27	7.32	7.47	7.81	8.61	9.1	9.67	14.33	18.08	19.94	20.83	21.84	24.43	24.18	24.86	25.78	25.85	27.09	27.09	27.73	.
74	H2O	F	3	4.08	4.45	4.94	5.39	5.8	6.11		6.91	7.29	7.43	7.36	7.62	8.37	8.82	9.3	12.76	14.25	16.78	17.34	17.93	18.98	19.36	20.6	20.49	21.5	19.36	22.21	21.6	.
74	H2O	M	4	3.9	4.51	5.05	5.53	5.95	6.18		6.78	6.98	7.13	7.3	7.61	8.31	8.81	9.36	13.28	16.84	17.94	21.05	22.14	24.68	25.08	25.75	26.38	27.31	28.11	27.89	28.22	.
74	H2O	F	5	4.13	4.5	5.03	5.58	5.98	6.13		6.98	7.17	7.48	7.53	7.78	8.09	9.15	9.6	12.98	14.85	17.38	18.55	17.62	18.89	18.87	20.88	20.29	21.66	19.68	22.25	21.69	.
74	H2O	F	6	4.07	4.43	4.9	5.66	5.92	6.18		6.86	6.96	7.23	7.25	7.32	8.03	8.63	8.99	12.58	4.5	17.63	18.03	19.02	19.88	20.33	21.78	21.71	22.47	19.83	22.61	22.98	.
74	H2O	F	7	3.98	4.48	4.87	5.5	5.8	6.08		6.98	7.1	7.34	7.36	7.64	8.59	8.87	9.38	12.84	15.08	18.47	18.11	19.5	20.36	20.69	22.18	22.08	23.52	20.38	23.48	23.54	.
75	MD	F	1	3.8	4.5	4.83	5.23	5.69	5.99	6.34	6.5	6.87	7.11	7.49	7.55	8.11	8.36	8.55	13.12	16.05	17.43	17.43	18.67	19.58	20.09	20.98	21.54	22.44	23.38	23.06	23.14	
75	MD	M	2	4.21	4.8	5.18	5.37	5.74	6.05	6.4	6.6	6.89	7.07	7.58	7.71	7.76	8.17	8.71	14.55	17.47	19.74	21.66	23.26	24.19	24.68	25.38	25.45	24.34	28.41	28.65	29.12	
75	MD	M	3	4.1	4.78	5.15	5.5	5.94	6.4	6.6	7.1	7.36	7.71	7.83	7.93	8.28	8.83	9.38	.	.	.	.	.	.	.	.	.	.	.	.	.	
75	MD	M	4	3.89	4.66	5.04	5.18	5.44	5.82	6.25	6.5	7	7.22	7.58	7.87	8.3	8.74	9.24	.	.	.	.	.	.	.	.	.	.	.	.	.	
75	MD	M	5	3.96	4.54	4.9	5.13	5.46	5.7	6.09	6.4	6.71	6.94	7.43	7.42	7.82	8.82	9.19	15.87	19.2	21.83	22.28	23.41	24.13	24.9	25.38	26.66	24.36	27.84	28.02	28.74	
75	MD	M	6	3.87	4.38	4.77	4.97	5.3	5.68	6.02	6.4	6.62	7	7.41	7.48	7.67	8.24	8.8	15.47	19.1	20.64	21.82	22.53	23.79	24.63	24.78	25.5	23.79	27.32	27.53	27.84	
75	MD	M	7	3.98	4.43	4.82	5.04	5.38	5.62	5.99	6.5	6.51	7	7.26	7.33	7.39	8.15	8.75	15.45	18.79	20.73	22.56	23.6	25.09	25.88	26.28	27.52	25.3	28.76	29.88	30.02	
76	H2O	M	1	4.59	4.95	5.41	6.11	6.51	6.96	7	7.2	7.42	7.43	7.59	7.68	8.31	9.01	9.99	16.74	20.25	21.41	23.44	24.11	25.62	26.72	27.82	28.35	21.48	28.97	29.64	30.96	
76	H2O	M	2	4.68	5.16	5.62	6.3	6.79	7.19	7.4	7.6	7.68	8.06	8.28	8.66	9.43	9.68	10.59	16.63	20.73	22.1	24.23	24.7	26.49	27.84	27.26	28.04	29	29.35	30.27	30.97	
76	H2O	F	3	4.87	5.42	5.88	6.33	6.67	7.7	8	8.3	8.32	8.38	8.51	8.94	9.42	9.91	10.56	14.52	16.81	18.22	18.89	19.88	20.88	21.9	22.58	22.56	22.48	25.03	24.38	24.45	
76	H2O	F	4	4.68	5.09	5.63	6.22	6.55	7.09	7.4	7.42	7.55	7.98	8.05	8.26	8.78	9.32	9.98	14.12	17.48	17.09	18.99	19.58	20.38	20.71	20.53	21.71	22.68	22.88	22.96	23.53	
76	H2O	F	5	4.59	5.15	5.51	6.22	6.37	6.85	6.9	7.21	7.24	7.4	7.53	8.18	8.53	9.1	9.88	14.67	16.66	17.16	18.58	18.23	19.48	20.39	20.03	21.49	21.52	21.89	22.18	22.57	
76	H2O	F	6	4.42	4.92	5.42	5.89	6.37	6.66	6.9	7.3	7.38	7.4	7.47	7.68	8.18	8.82	.	.	.	.	.	.	.	.	.	.	.	.	.	.	
76	H2O	F	7	4.5	5.08	5.65	6.09	6.69	7.13	7.3	7.72	7.87	8.02	8.19	8.38	8.86	9.43	10.12	15.27	17.46	16.63	19.18	19.48	20.86	22.12	21.7	22.4	22.49	23.07	22.79	23.21	
77	MCT	M	1	5.1	5.46	5.89	6.53	6.79	7.28	7.1	7.68	7.69	8.01	8.12	8.63	9.19	9.67	10.44	16.65	19.57	21.43	24.28	25.79	27.06	28.36	28.23	29.11	26.93	30.06	30.78	30.92	
77	MCT	M	2	5.05	5.51	5.96	6.54	6.97	7.52	8	8.2	8.33	8.72	8.85	9.06	9.68	10.21	10.88	16.4	20.61	22.08	23.68	24.64	25.98	26.22	26.03	27.59	25.45	28.85	28.8	28.99	
77	MCT	M	3	5.45	5.82	6.16	6.87	7.12	7.85	8.3	8.44	8.63	9.15	9.21	9.38	9.88	10.74	11.43	18.58	20.69	21.84	23.68	24.61	26.49	27.18	27.48	28.82	25.81	29.64	29.98	30.7	
77	MCT	F	4	4.94	5.39	5.84	6.55	6.83	7.66	7.8	7.69	8.21	8.41	8.65	9.15	9.48	10.23	10.76	15.67	16.78	17.17	19.05	19.43	19.98	20.23	20.23	21.03	21.81	22.03	22.59	22.24	
77	MCT	F	5	4.65	4.85	5.24	5.75	5.97	6.43	6.5	6.73	6.9	7.11	7.48	7.43	7.63	8.2	8.82	13.48	16.5	17.63	19.08	19.38	20.18	20.83	21.09	21.42	21.4	22.56	22.78	23.04	
77	MCT	F	6	5.22	5.58	6.08	6.81	7.1	7.48	8.1	8.32	8.49	8.88	8.9	9.22	9.87	10.36	10.96	15.46	17.61	18.22	19.58	20.28	20.89	21.43	21.09	22.34	22.52	23.01	23.14	23.67	
78	MCT	M	1	4.04	4.49	4.91	5.24	5.81	6.02	6.39	6.6	6.81	7.09	7.14	7.39	8.02	8.32	8.58	15.72	19.07	20.51	22.14	24.36	25.12	26.64	26.45	27.54	28.34	28.51	28.76	29.66	
78	MCT	M	2	3.93	4.48	4.91	5.08	5.48	5.87	6.29	6.4	6.58	6.88	7.23	7.43	8.11	8.46	9.44	16.24	18.45	20.8	22.37	23.68	25.28	26.16	26.69	28.34	28.76	28.87	29.25	29.93	
78	MCT	M	3	4.05	4.78	5.02	5.28	5.71	6	6.32	6.5	6.79	6.94	7.24	7.49	8.12	8.27	9.42	.	.	.	.	.	.	.	.	.	.	.	.	.	
78	MCT	M	4	4.2	4.77	5.18	5.62	5.97	6.36	6.75	7	7.26	7.45	7.62	7.8	8.68	8.84	9.68	.	.	.	.	.	.	.	.	.	.	.	.	.	
78	MCT	M	5	3.89	4.42	4.81	5.1	5.54	6.01	6.35	6.4	6.9	7	7.14	7.25	7.51	7.68	9.07	14.89	18.48	20.09	22.08	23.89	25.16	25.5	26.44	28.15	27.83	28.02	28.58	29.78	
78	MCT	M	6	4.31	4.88	5.25	5.5	5.67	6.32	6.73	7	7.07	7.36	7.57	7.79	8.18	8.2	9.15	13.76	18.34	20.45	23.69	24.84	27.28	27.48	27.87	29.26	29.77	30.36	30.34	31.18	
78	MCT	F	7	3.93	4.47	4.9	5.21	5.59	5.9	6.22	6.2	6.35	6.7	6.86	7.28	8.06	8.38	8.8	13.57	17.04	19.42	1862	20.5	21.38	21.59	21.78	22.04	23.14	23.29	23.08	23.44	
78	MCT	F	8	4.25	4.78	5.26	5.68	5.97	6.25	6.74	6.9	7.08	7.15	7.24	7.14	7.74	7.99	9.36	14.13	17.31	18.13	19.06	20.08	21.61	21.99	21.95	22.1	22.92	23.79	23.66	23.96	
79	H2O	M	1	4.48	4.9	5.19	5.42	6.05	5.9	6.32	6.78	7.07	7.55	7.62	7.79	8.35	9.05	9.65	18.69	20.84	23.26	24.98	27.77	28.98	28.98	28.88	29.53	30.02	31.6	.	.	
79	H2O	M	2	4.4	4.89	5.09	5.62	6.01	6.2	6.38	6.72	7.06	7.19	7.29	7.69	8.2	8.94	9.6	18.79	20.29	22.82	24.83	27.26	28.7	28.7	28.89	29.59	29.88	31.24	.	.	
79	H2O	M	3	4.18	4.67	5.02	5.35	5.85	6.1	6.43	6.93	7.18	7.42	7.48	7.59	7.64	8.42	9.04	6.72	.	.	.	.	.	.	.	.	.	.	.	.	
79	H2O	F	4	4.18	4.61	5.07	5.61	6.15	6.3	6.56	6.96	7.18	7.41	7.63	7.9	8.14	8.57	9.06	14.38	16.46	17.72	18.28	18.98	19.98	19.98	20.27	21.03	21.76	22.59	.	.	
79	H2O	F	5	4.4	4.84	5.21	5.62	6.12	6.3	6.59	7.02	7.35	7.47	7.68	7.82	7.84	8.38	8.9	14.24	15.48	17.56	17.87	19.49	19.88	19.88	19.65	20.58	21.57	21.72	.	.	
79	H2O	F	6	4.58	5.05	5.4	5.93	6.36	6.7	6.76	7.22	7.58	7.72	7.81	7.92	8.23	9.2	9.94	15.82	17.07	18.46	18.74	19.82	20.34	20.34	20.18	20.98	20.96	22.18	.	.	
79	H2O	F	7	4.3	4.85	5.26	5.71	6.02	6.4	6.76	7.12	7.42	7.73	7.93	7.8	8.1	9.11	9.68	15.03	15.7	17.81	18.58	19.98	20.79	20.79	19.89	20.85	21.8	22.95	.	.	
81	H2O	F	1	.	4.9	5.3	5.77	6.2	6.68	6.76	7.2	7.15	7.34	7.83	8.28	8.92	.	11.43	13.35	15.6	17.25	19.01	20.63	21.28	21.22	21.99	22.81	23.96	.	.	.	
81	H2O	F	2	4.48	4.91	5.3	5.55	5.86	6.8	6.62	7.02	6.86	7.11	7.81	8.33	8.78	.	11.17	13.77	15.94	17.52	18.83	20.49	22.02	22.4	22.48	22.86	23.82	.	.	.	
81	H2O	M	3	4.7	5.14	5.43	5.61	5.82	6.28	6.73	6.9	6.8	6.98	7.07	7.74	8.48	.	12.47	14.98	18.67	21.03	22.46	24.38	25.52	25.68	26.89	26.89	28.74	.	.	.	
81	H2O	M	4	4.68	5.03	5.49	5.67	5.79	6.34	6.71	6.9	6.85	7.07	7.54	7.98	8.78	.	12.15	15.04	18.84	20.7	22.42	24.69	25.68	26.08	27.38	27.86	29.56	.	.	.	
81	H2O	M	5	4.54	4.79	5.03	5.31	5.65	6.07	6.6	6.7	6.6	6.8	7.28	7.72	8.13	.	11.54	14.2	17.76	20.91	21.53	24.33	24.48	25.39	26.09	27.08	28.48	.	.	.	
81	H2O	M	6	4.51	5.01	5.38	5.6	5.91	6.62	6.78	7.2	7.06	7.38	7.89	8.49	9.08	.	12.93	15.81	19.13	21.53	22.84	25.08	25.64	25.77	26.23	26.78	28.08	.	.	.	
82	ETOH	F	1	4.12	.	5.2	5.6	5.8	6.01	6.36	6.74	6.9	7.03	7.14	7.33	7.73	8.08	8.56	14.02	15.28	18.03	18.67	19.49	20.53	20.3	21.18	22.09	22.75	.	.	.	
82	ETOH	F	2	4.3	.	5.11	5.65	5.9	6.02	6.47	6.64	6.82	7.07	7.27	7.41	7.71	8.14	8.57	13.25	14.67	17.53	17.63	18.77	20.01	19.96	20.89	21.18	21.96	.	.	.	
82	ETOH	M	3	4.22	.	4.75	5.07	6	5.18	5.38	5.54	5.65	5.95	6.14	6.47	6.97	7.53	7.95	.	.	.	.	.	.	.	.	.	.	.	.	.	
82	ETOH	M	4	4.45	.	5.22	5.7	5.1	6.01	6.39	6.52	6.6	6.75	6.93	7.26	7.49	8.19	8.52	11.53	14.56	19.06	21.58	24.79	25.35	26.02	27.38	27.18	29.36	.	.	.	
82	ETOH	M	5	3.55	.	4.27	4.64	4.8	5.08	5.59	5.76	5.92	6.13	6.25	6.48	6.58	7.04	7.42	.	.	.	.	.	.	.	.	.	.	.	.	.	
82	ETOH	M	6	4.52	.	5.26	5.81	6.1	6.25	6.65	6.78	6.98	7.27	7.39	7.63	8.3	8.72	9.35	15.98	18	19.95	21.96	24.07	24.7	25.08	26.68	27	29.04	.	.	.	
82	ETOH	M	7	4.18	.	5.04	5.45	5.7	5.9	6.52	6.29	6.47	6.78	6.93	7.13	7.7	8.15	8.83	14.04	16.35	18.53	20.56	22.38	23.69	23.69	24.83	25.09	26.7	.	.	.	
82	ETOH	M	8	4.26	.	5.08	5.54	5.8	5.99	6.38	6.52	6.54	6.76	7.07	7.25	7.96	8.38	9.18	14.63	17.28	19.62	21.65	24.15	24.87	25.02	26.1	26.28	29.04	.	.	.	
83	H2O	M	1	5.41	6.06	6.42	6.53	6.94	7.69	7.3	7.2	7.41	7.55	8.23	9.18	.	.	10.94	16.15	18.06	19.21	21.54	23.28	24.61	.	.	27.23	27.23	27.98	28.87	29.38	
83	H2O	M	2	5.2	5.91	6.32	6.36	6.36	7.04	7.3	7.38	7.43	7.57	8.22	8.99	.	.	11.43	17.04	20.38	21.92	24.08	25.31	26.13	.	.	27.66	27.91	28.48	29.33	29.63	
83	H2O	F	3	5.36	5.75	6.19	6.39	6.39	7	7.3	7.11	7.41	7.76	7.86	8.7	.	.	10.37	14.97	16.7	17.32	19.78	19.24	20.67	.	.	21.75	22.91	22.98	23.47	24.17	
83	H2O	F	4	5.49	5.94	6.49	6.75	6.75	7.58	7.6	7.37	7.68	7.84	8.75	9.03	.	.	.	.	.	.	.	.	.	.	.	.	.	.	.	.	
83	H2O	F	5	5.06	5.52	5.88	6.15	6.15	7.48	7.2	6.98	7.28	7.39	7.86	8.66	.	.	11.23	14.82	16.56	17.63	18.84	18.77	20.08	.	.	21.5	21.85	21.72	22.59	23.64	
83	H2O	F	6	4.81	5.16	5.67	5.99	5.99	6.91	6.7	6.67	6.89	7.04	7.14	8.07	.	.	10.33	14.95	16.63	17.64	18.8	19.92	19.98	.	.	21.68	22.34	22.28	23.09	23.78	
83	H2O	F	7	4.94	5.21	5.7	5.96	5.96	6.96	6.8	6.64	6.9	7.14	7.53	7.98	.	.	10.37	13.45	15.53	16.61	17.78	17.93	18.36	.	.	19.92	20.64	21.12	21.16	21.98	
84	MCT	M	1	3.9	4.55	5.04	5.58	6.07	6.58	6.9	7.01	7.19	7.22	7.43	7.81	.	.	10.98	16.76	19.03	20.87	23.12	24.46	25.84	26.22	.	27.31	27.65	28.34	28.82	29.48	
84	MCT	M	2	3.87	4.78	5.25	5.69	6.17	6.39	7	7.17	7.4	7.77	8.04	8.45	.	.	11.21	16.95	18.23	19.35	21.43	23.38	24.89	24.95	.	25.93	26.85	27.53	28.07	28.76	
84	MCT	M	3	3.98	4.88	5.26	5.89	6.34	6.68	7.1	7.34	7.63	7.99	8.07	8.46	.	.	12.01	17.78	19.45	20.5	23.05	24.32	25.52	25.88	.	26.46	27.03	27.6	28.92	29.13	
84	MCT	F	4	3.97	4.78	5.15	5.73	6.12	6.52	6.9	7.25	7.46	7.63	7.89	8.3	.	.	10.92	15.71	17.65	18.61	18.68	19.48	20.39	21.09	.	22.44	22.53	22.15	22.86	23.96	
84	MCT	F	5	3.73	4.76	5.22	5.7	6.12	6.61	6.8	7.09	7.28	7.52	7.61	7.9	.	.	10.64	14.01	16..49	18.04	18.18	19.23	20.27	20.46	.	20.25	21.37	21.08	21.87	22.51	
84	MCT	F	6	3.81	4.42	4.75	5.22	5.63	5.68	6.3	6.62	6.91	7.09	7.5	7.58	.	.	9.72	13.19	15.56	16.75	17.36	18	19.12	19.25	.	20.55	20.45	20.32	20.3	20.87	
84	MCT	F	7	3.74	4.47	4.86	5.32	5.92	6.09	6.5	6.88	7.19	7.43	7.61	7.87	.	.	10.36	14.32	16.65	17.14	18.2	18.93	18.68	20.09	.	20.71	21.27	21.08	21.34	22.47	
