# Supplementary material for: Does prenatal alcohol exposure cause a metabolic syndrome? (Non-)evidence from a mouse model of fetal alcohol spectrum disorder
Source: PLoS One. 2018 Jun 28;13(6):e0199213. doi: 10.1371/journal.pone.0199213 (PMC6023152; doi:10.1371/journal.pone.0199213)
Supplement: S1 Dataset — (ZIP) [file pone.0199213.s010.zip › New folder/BW-NMR.pdf]

Blk		3	3	3	3	4	4	5	7	7
Box		Box-3	Box-4	Box-5	Box-6	Box-2	Box-3	Box-4	Box-3	Box-4
ID		6.4	9.6	10.6	11.1	17.8	18.7	28.7	41	42
Sex		F	F	F	F	F	F	F	F	F
Exposure		EtOH	EtOH	EtOH	EtOH	EtOH	EtOH	EtOH	EtOH	EtOH
Tx		FE	FE	FE	FE	FE	FE	FE	FE	FE
BW (g)										
	Day									
Chow	0	22.92	23.12	22.21	19.9	22.23	23.07	21.34	22.43	23.05
10%	3	24.07	23.09	23.24	19.74	22.75	22.75	22.42	22.4	23.6
60%	6	24.28	23.96	21.04	20.99	22.59	23.44	22.6	22.47	22.96
End	10	24.65	23.98	25.45	21.36	22.42	24.55	24.16	24.11	23.75
4wk									25.98	29.2
Lean (g)										
	Day									
Chow	0	19.47	19.98	18.63	16.35	19.98	19.03	18.07	18.95	19.54
10%	3	20.17	19.63	18.98	16.25	18.95	18.74	18.74	19.55	19.79
60%	6	19.54	19.53	18.23	16.52	18.57	18.56	18.57	18.74	18.29
End	10	19.03	19.15	19.29	16.1	17.6	18.44	18.75	18.55	18.01
4 wk	28	19.51	20.8	18.58	16.08	18.3	18.25	18.77	19.97	19.87
Fat (g)										
	Day									
Chow	0	1.76	1.78	2.28	2.34	4.66	4.45	1.74	1.92	2.27
10%	3	2.5	2.05	2.95	2.2	2.21	2.77	2.43	1.63	2.77
60%	6	3.38	2.95	3.55	3.28	2.58	3.63	2.52	2.22	3.36
End	10	4.45	3.59	5.55	4.07	3.13	4.88	4.19	4.12	4.29
4 wk	28	5.21	6.19	5.68	2.84	2.72	5.25	6.52	5.09	8.7
Total (g)										
	Day									
Chow	0	21.23	21.76	20.91	18.69	24.64	23.48	19.81	20.87	21.81
10%	3	22.67	21.68	21.93	18.45	21.16	21.51	21.17	21.18	22.56
60%	6	22.92	22.48	21.78	19.8	21.15	22.19	21.09	20.96	21.65
End	10	23.48	22.74	24.84	20.17	20.73	23.32	22.94	22.67	22.3
4 wk	28	24.72	26.99	24.26	18.92	21.02	23.5	25.29	25.06	28.57
Fat (%)										
	Day									
Chow	0	8%	8%	11%	13%	19%	19%	9%	9%	10%
10%	3	11%	9%	13%	12%	10%	13%	11%	8%	12%
60%	6	15%	13%	16%	17%	12%	16%	12%	11%	16%
End	10	19%	16%	22%	20%	15%	21%	18%	18%	19%
4 wk	28	21%	23%	23%	15%	13%	22%	26%	20%	30%
Rectal (C)										
	Day									
Chow	0	.	.	.	.			36.9	37.4	37.5
10%	3	36.4	37.6	37.2	36.9	37.7	35.9	36.1	37.6	36.6
60%	6	37	38.2	37.9	38.1	37.4	37	37	38.3	36.9
End	10	37.4	37.3	37.7	37.8	38.3	37.3	37.4	43.3	43.1

8	3	4	4	7	7	8	10	10	10
Box-4	Box-7	Box-5	Box-8	Box-1	Box-7	Box-2	Box-4	Box-5	Box-6
53.5	12.4	23.1	27.7	37	46	50.6	69	63	62
F	F	F	F	F	F	F	F	F	F
EtOH	H2O	H2O	H2O	H2O	H2O	H2O	H2O	H2O	H2O
FE	FH	FH	FH	FH	FH	FH	FH	FH	FH

21.16	24.03	23.33	22.55	22.28	21.95	22.95	23.55	22.01	24.04
21.58	24.11	24.65	22.43	23.47	22.99	23	24.60	23.09	23.83
22.3	24.76	25.41	23.66	22.67	22.16	23.02	23.70	22.58	25.30
22.84	27.08	24.79	25.31	23.67	23.49	24.2	25.56	24.32	26.68
24.43				31.44	23.08	30.41	.	.	.

18.15	20.3	19.29	19.01	19.11	19.03	19.46	19.96	18.57	19.96
18.71	19.91	20.19	19.13	19.9	19.58	19.41	19.4	18.23	19.58
18.22	19.8	19.67	19.03	18.6	18.38	18.91	19.05	17.97	20.22
18.3	20.44	18.6	18.99	17.99	18.46	19.71	19.86	18.2	19.77
18.87	21.2	20.17	19.75	20.14	19.39	19.76	.	.	.

1.46	2.68	5.55	2.05	1.91	1.55	1.86	2.22	2.3	2.87
1.75	2.73	3.01	1.96	2.37	2.2	2.2	3.19	3.53	3.69
2.34	3.45	4.44	3.14	2.81	2.4	2.7	3.19	3.53	3.69
3.03	5.64	4.7	4.86	4.43	3.71	2.77	4.42	4.92	5.78
3.77	10.59	5.99	7.41	10.64	2.74	9.04	.	.	.

19.61	22.98	24.84	21.06	21.02	20.58	21.32	22.18	20.87	22.83
20.46	22.64	23.2	21.09	22.27	21.78	21.61	22.59	21.76	23.27
20.56	23.25	24.11	22.17	21.41	20.78	21.61	22.24	21.5	23.91
21.33	26.08	23.3	23.85	22.42	22.17	22.48	24.28	23.12	25.55
22.64	31.79	26.16	27.16	30.78	22.13	28.8	#VALUE!	#VALUE!	#VALUE!

7%	12%	22%	10%	9%	8%	9%	10%	11%	13%
9%	12%	13%	9%	11%	10%	10%	14%	16%	16%
11%	15%	18%	14%	13%	12%	12%	14%	16%	15%
14%	22%	20%	20%	20%	17%	12%	18%	21%	23%
17%	33%	23%	27%	35%	12%	31%	#VALUE!	#VALUE!	#VALUE!

40.7	.			37.8	38.2	41.2	.	.	.
37.6	36.7	37.4	37.4	35.4	37.2	36.6	36.9	37.3	35.8
38.2	37.9	37	37.8	36.2	38.2	36.7	37.6	36.9	36.6
37.8	37.7	36.3	36.9	41.3	44.5	37.3	37.8	37.5	36.7

12	12	12	12	3	3	4	4	5	7	7
Box-1	Box-2	Box-5	Box-6	Box-1	Box-2	Box-6	Box-7	Box-6	Box-2	Box-5
74	76	81	83	1.7	2.3	25.3	26.6	34.6	38	43
F	F	F	F	F	F	F	F	F	F	F
H_2O	H_2O	H_2O	H_2O	Malto	Malto	Malto	Malto	MD	Malto	Malto
FH	FH	FH	FH	FM	FM	FM	FM	FM	FM	FM
24.3	26.6	25.7	25	22.23	24.39	23.03	23.46	21.26	22.68	.
23.4	27	26.6	26.6	23.5	24.7	23.17	23.84	21.7	22.53	.
26.2	27.1	25.5	27.4	25.42	24.82	23.25	23.95	21.72	23.22	.
28.58	29.99	27.17	29.75	25.12	26.08	25.1	25.74	21.43	25.14	.
36.30	36.71	35.64	39.39						25.11	23.77
20.60	23.22	22.27	21.54	18.67	20.53	16.1	20.44	18.31	19.3 .	
19.66	22.57	22.58	22.42	19.56	20.31	19.3	19.79	18.68	19.08	.
14.72	21.89	20.9	21.73	19.65	19.71	18.87	19.22	18.25	18.88	.
20.21	22.27	21.28	22.16	18.86	19.98	18.93	19.36	17.78	19.44	.
21.46	23.00	22.48	23.45	19.2	20.46	20.12	19.47	19.09	19.44	19.46
3.19	2.73	2.27	2.33	2.17	2.43	4.07	5.64	1.45	1.87 .	
3.41	3.11	3.09	3.08	2.59	2.82	2.39	2.73	1.78	2.3	.
3.07	3.68	3.64	4.47	4.32	3.45	2.99	3.44	2	2.91	.
7.68	6.54	5.07	7.18	4.86	4.66	4.43	4.7	2.4	4.28	.
13.5	11.9	11.89	14.53	4.81	5.9	6.7	5.47	4.55	4.39	3.31
23.79	25.95	24.54	23.87	20.84	22.96	20.17	26.08	19.76	21.17	.
23.07	25.68	25.67	25.5	22.15	23.13	21.69	22.52	20.46	21.38	.
17.79	25.57	24.54	26.2	23.97	23.16	21.86	22.66	20.25	21.79	.
27.89	28.81	26.35	29.34	23.72	24.64	23.36	24.06	20.18	23.72	.
34.96	34.9	34.37	37.98	24.01	26.36	26.82	24.94	23.64	23.83	.
13%	11%	9%	10%	10%	11%	20%	22%	7%	9%	.
15%	12%	12%	12%	12%	12%	11%	12%	9%	11%	.
17%	14%	15%	17%	18%	15%	14%	15%	10%	13%	..
28%	23%	19%	24%	20%	19%	19%	20%	12%	18%	.
39%	34%	35%	38%	20%	22%	25%	22%	19%	18%	.
35.5	35.8	35.8	36.1	.	.			37.1	37.6	.
35.4	36	36.4	37.4	35.6	36.3	36.9	37.4	37.7	37.2	.
36.8	36.9	36.7	36	37	36.9	37.8	37.2	37.6	37	.
36.1	37	37.6	37	36.4	37.5	37.6	38.2	38.2	45.2	.

8	10	10	4	4	5	7	7	8	10	10
Box-3	Box-1	Box-3	Box-1	Box-4	Box-5	Box-6	Box-8	Box-1	Box-2	Box-7
52.6	64	75	13.3	21.5	32.6	45	47	44.6	68	65
F	F	F	F	F	F	F	F	F	F	F
MD	Malto	Malto	MCT	MCT	MCT	MCT	MCT	MCT	MCT	MCT
FM	FM	FM	FT	FT	FT	FT	FT	FT	FT	FT
22.78	24.91	22.67	22.71	22.49	22.69	22.58	23.6	22.68	23.93	21.37
22.34	25.93	23.42	23.75	23.18	23.18	22.82	23.55	23.27	23.76	20.28
21.79	27.69	24.27	24.04	23.21	23.65	22.91	23.27	23.75	24.18	21.56
22.89	28.69	24.98	25.22	22.63	24.62	23.72	23.97	23.42	26.65	23.19
26.79	.	.				24.22	27.17	26.27	.	.
19.08	20.45	19.44	18.86	19.15	19.69	18.95	20.34	18.9	20.13	17.89
18.83	20.46	19.43	19.37	19.39	19.53	20.24	20.03	19.62	20.62	14.78
17.88	20.84	19.69	19.09	18.82	19.68	19.19	19.04	19.83	20.56	17.39
18.06	20.9	19.01	18.8	18.11	19.55	19.02	18.87	17.03	20.6	17.29
19.37	.	.	20.38	18.95	19.58	19.63	20.02	19.36	.	.
1.94	3.41	1.96	4.86	3.59	1.54	1.79	2.22	2.03	2.32	2.63
2.06	5.42	3.36	2.75	2.16	2.17	1.52	2.4	2.17	2.33	2.82
2.58	5.42	3.36	3.63	2.69	2.38	2.4	3.02	2.59	2.33	2.82
3.99	6.87	4.89	5.21	3.05	3.67	3.35	3.8	4.51	4.55	4.76
5.78	.	.	8.42	1.77	3.54	3.61	6.1	5.37	.	.
21.02	23.86	21.4	23.72	22.74	21.23	20.74	22.56	20.93	22.45	20.52
20.89	25.88	22.79	22.12	21.55	21.7	21.76	22.43	21.79	22.95	17.6
20.46	26.26	23.05	22.72	21.51	22.06	21.59	22.06	22.42	22.89	20.21
22.05	27.77	23.9	24.01	21.16	23.22	22.37	22.67	21.54	25.15	22.05
25.15	#VALUE!	#VALUE!	28.8	20.72	23.12	23.24	26.12	24.73	#VALUE!	#VALUE!
9%	14%	9%	20%	16%	7%	9%	10%	10%	10%	13%
10%	21%	15%	12%	10%	10%	7%	11%	10%	10%	16%
13%	21%	15%	16%	13%	11%	11%	14%	12%	10%	14%
18%	25%	20%	22%	14%	16%	15%	17%	21%	18%	22%
23%	#VALUE!	#VALUE!	29%	9%	15%	16%	23%	22%	#VALUE!	#VALUE!
40.2	.	.			36.9	37.5	37.8	41.5	.	.
36.7	35.8	36	37.2	36.4	36.9	37.4	37.5	35.8	35.2	37.1
37.4	36.2	37.6	36.2	37.4	36.4	37.8	37.4	36.8	35.6	37
37	36.4	37.4	37.1	37.6	37.4	44.1	42.7	37.4	36.4	37.8

12	12	12	1	1	1	1	2	2	5	6
Box-3	Box-4	Box-7	Box-1	Box-3	Box-4	Box-5	Box-2	Box-3	Box-1	Box-2
77	78	84	6.1	9.4	10.1	11.6	16.4	17.4	28.1	41
F	F	F	M	M	M	M	M	M	M	M
MCT	MCT	MCT	EtOH	EtOH	EtOH	EtOH	EtOH	EtOH	EtOH	EtOH
FT	FT	FT	ME	ME	ME	ME	ME	ME	ME	ME
24.7	25.1	25	26.92	27.53	28.28	24.99	29.54	28.77	27.61	27.06
25.4	26.2	26.7	26.95	27.76	27.99	25.13	29.33	28.31	27.71	26.89
25.4	26.1	27.9	27.97	28.88	28.65	26.42	30.87	30.56	28.23	27.81
27.51	28.08	28.92	28.98	29.74	30.95	27.28	33.76	32.63	29.54	28.49
34.60	37.44	38.00								
21.27	21.47	21.25	23.61	23.97	24.43	21.55	25.21	24.09	23.89	23.79
21.83	22.09	22.37	23.51	24	24.05	21.47	25.25	24.3	24.11	23.35
20.77	20.82	21.67	23.39	23.5	23.95	21.28	25.79	25	23.92	23.7
21.01	21.36	20.70	23.51	23.27	24.21	21.34	26.27	25.33	23.98	22.64
21.67	22.17	21.71	23.69	25.17	25.78	22.03	26.01	25.92	23.84	23.77
2.53	1.99	2.64	1.85	1.97	2.21	1.82	2.16	2.58	2.58	1.96
2.58	2.98	3.2	1.85	2.39	2.54	2.31	2.59	2.66	2.56	2.21
3.5	3.91	4.81	2.8	3.23	3.43	3.52	3.73	3.81	3.05	2.71
6.12	5.66	7.74	4.01	5.15	5.4	4.47	6.08	6.15	4.31	3.63
11.77	13.76	15.15	9.2	12.5	15.22	11.5	12.92	16.5	10.79	8.07
23.8	23.46	23.89	25.46	25.94	26.64	23.37	27.37	26.67	26.47	25.75
24.41	25.07	25.57	25.36	26.39	26.59	23.78	27.84	26.96	26.67	25.56
24.27	24.73	26.48	26.19	26.73	27.38	24.8	29.52	28.81	26.97	26.41
27.13	27.02	28.44	27.52	28.42	29.61	25.81	32.35	31.48	28.29	26.27
33.44	35.93	36.86	32.89	37.67	41	33.53	38.93	42.42	34.63	31.84
11%	8%	11%	7%	8%	8%	8%	8%	10%	10%	8%
11%	12%	13%	7%	9%	10%	10%	9%	10%	10%	9%
14%	16%	18%	11%	12%	13%	14%	13%	13%	11%	10%
23%	21%	27%	15%	18%	18%	17%	19%	20%	15%	14%
35%	38%	41%	28%	33%	37%	34%	33%	39%	31%	25%
35.6	35.7	36.7	.	.	.	.	.	.	35.3	35.5
35.6	36.6	36.8	35.2	35.9	36.1	35.5	36	37	35.1	37
36.7	36.9	36.9	35.4	35.9	36.7	36.4	37	36.9	36.1	35.7
37.4	37.3	38	37	36.3	37.5	37	36.3	37.1	36	36.3

6	8	9	11		1	2	2	6	6	8
Box-3	Box-8	Box-7	Box-5		Box-6	Box-6	Box-8	Box-6	Box-8	Box-5
42	53.2	71	82		12.1	23.3	27.5	46	37	50.1
M	M	M	M		M	M	M	M	M	M
EtOH	EtOH	EtoH	EtOH		H2O	H2O	H2O	H2O	H2O	H2O
ME	ME	ME	ME		MH	MH	MH	MH	MH	MH
27.98	29.64	30.79	30.8		28.32	26.91	30.53	25.22	28.13	28.4
28.58	28.81	30.29	29.1		28.92	28.1	30.32	25.68	28.79	28.81
29.21	29.07	31.51	30.7		29.52	28.31	31.98	26.91	29.04	28.61
31.48	29.81	34.70	32.89		31.78	30.33	34.31	27.61	30	30.46
	35.26	43.96	.							37.45
23.67	26.68	25.67	25.72		24.1	23.34	25.72	21.89	24.8	24.56
23.93	25.25	25.14	24.74		24.67	23.83	25.95	22.13	24.61	25.3
23.37	24.95	25.78	24.63		24.06	23.75	25.99	22.48	24.18	24.16
23.56	24.64	25.96	24.53		24.31	24.03	26.33	22.15	23.4	24.27
26.1	25.06	.	.		25.74	24.36	25.54	22.79	30.53	24.11
3.06	1.34	3.98	4.07		2.67	1.82	2.51	2.06	2.14	2.42
3.55	2.06	3.78	3.44		2.79	2.74	3.01	2.27	2.96	2.49
4.3	2.52	4.49	4.45		3.54	3.37	4.11	3.13	3.74	2.9
5.97	3.64	7.87	6.79		5.63	5.3	6.22	4.01	4.42	4.62
13.46	8.14	.	.		16.39	11.53	14.3	11.62	7.99	11.68
26.73	28.02	29.65	29.79		26.77	25.16	28.23	23.95	26.94	26.98
27.48	27.31	28.92	28.18		27.46	26.57	28.96	24.4	27.57	27.79
27.67	27.47	30.27	29.08		27.6	27.12	30.1	25.61	27.92	27.06
29.53	28.28	33.83	31.32		29.94	29.33	32.55	26.16	27.82	28.89
39.56	33.2	#VALUE!	#VALUE!		42.13	35.89	39.84	34.41	38.52	35.79
11%	5%	13%	14%		10%	7%	9%	9%	8%	9%
13%	8%	13%	12%		10%	10%	10%	9%	11%	9%
16%	9%	15%	15%		13%	12%	14%	12%	13%	11%
20%	13%	23%	22%		19%	18%	19%	15%	16%	16%
34%	25%	#VALUE!	#VALUE!		39%	32%	36%	34%	21%	33%
36.2	41.6	38.3	35.6		.	.	.	35.8	35.2	39.9
37.7	37.6	37.3	35.4		35.4	35.8	36.3	38.1	38.4	35.9
37	37.1	36	36.9		37.2	36.2	37.2	37.6	37.9	37.1
36.7	37.7	36.7	36.4		37.6	37.1	37	36.8	36.3	36.7

9	9	9	11	11	11	11	1	1	1	2
Box-1	Box-2	Box-6	Box-1	Box-2	Box-4	Box-6	Box-2	Box-7	Box-8	Box-4
62	63	69	74	76	81	83	5.5	1.1	2.2	19.1
M	M	M	M	M	M	M	M	M	M	M
H2O	H2O	H2O	H2O	H2O	H2O	H2O	Malto	Malto	Malto	Malto
MH	MH	MH	MH	MH	MH	MH	MM	MM	MM	MM
28.58	29.08	29.93	31.4	35.1	31.1	30.5	28.74	29.67	27.42	29.69
28.85	27.98	29.05	29.6	33.6	30	29	28.38	28.42	27.15	29.83
29.85	29.31	30.51	31.2	35.5	31.7	30.9	28.18	29.92	27.4	30.88
32.83	31.11	32.75	34.31	37.72	34.12	33.96	29.47	31.3	29.57	33.86
38.92	38.01	37.92	.	.	.	.				
24.6	25.21	26.27	26.37	27.36	25.61	25.77	25.12	25.26	23.47	25.29
24.47	23.93	24.76	25.08	26.78	24.96	24.95	24.71	24.74	23.41	25.42
25.01	24.21	24.83	25.28	27.11	24.83	25.12	24.03	24.44	23.01	25.15
25.31	24.74	25.41	25.45	26.62	25.22	25.57	23.78	24.51	23.62	25.85
.	.	.	.	.	.	.	25.4	25.18	25.03	26
2.81	2.93	2.97	4.07	6.32	4.26	3.67	1.11	2.59	2.61	2.46
3.23	2.72	2.87	3.39	5.39	3.84	2.95	1.94	2.29	2.45	3.04
3.99	3.8	3.93	4.92	6.78	5.24	4.27	2.22	3.52	2.78	4.02
6.71	5.6	6.14	7.31	9.22	7.41	6.67	3.25	5.1	4.71	6.48
.	.	.	.	.	.	.	8.22	9.93	11.61	11.79
27.41	28.14	29.24	30.44	33.68	29.87	29.44	26.23	27.85	26.08	27.75
27.7	26.65	27.63	28.47	32.17	28.8	27.9	26.65	27.03	25.86	28.46
29	28.01	28.76	30.2	33.89	30.07	29.39	26.25	27.96	25.79	29.17
32.02	30.34	31.55	32.76	35.84	32.63	32.24	27.03	29.61	28.33	32.33
#VALUE!	#VALUE!	#VALUE!	#VALUE!	#VALUE!	#VALUE!	#VALUE!	33.62	35.11	36.64	37.79
10%	10%	10%	13%	19%	14%	12%	4%	9%	10%	9%
12%	10%	10%	12%	17%	13%	11%	7%	8%	9%	11%
14%	14%	14%	16%	20%	17%	15%	8%	13%	11%	14%
21%	18%	19%	22%	26%	23%	21%	12%	17%	17%	20%
#VALUE!	#VALUE!	#VALUE!	#VALUE!	#VALUE!	#VALUE!	#VALUE!	24%	28%	32%	31%
37.3	35.4	36.2	37.3	35.3	35.4	35.4	.	.	.	.
37.1	35.5	37	34.6	35.2	35.2	34.8	35.9	34.9	36.2	36.5
35.1	35.9	36.5	36.4	35.8	37.1	36.6	35.8	37	37.4	37.1
36.3	37	36.7	36.2	36	36.6	37.4	36.5	37.8	37.5	37.4

2	5	6	6	8	9	9	2	2
Box-7	Box-3	Box-1	Box-4	Box-7	Box-3	Box-8	Box-1	Box-5
25.1	34.2	38	43	52.2	64	75	13.7	20.2
M	M	M	M	M	M	M	M	M
Malto	MD	MD	MD	MD	MD	Malto	MCT	MCT
MM	MM	MM	MM	MM	MM	MM	MT	MT
28.82	24.25	26.42	28.07	28.87	32.86	28.17	29	27.64
27.76	24.69	27.14	28.9	29.59	31.06	27.93	29.54	27.51
29.14	25.95	27.98	29.99	30.44	32.00	28.88	31.16	27.57
31.26	26.54	28.87	29.57	33.3	34.96	31.91	32.67	31.06
				42.11	38.84	39.73		
23.69	21.92	23.13	25.58	24.67	26.76	24.88	25.06	22.73
23.47	22.17	23.56	25.59	25.28	25.54	23.77	25.67	23.13
24.04	22.53	23.91	25.37	25.58	26.21	23.95	26.02	22.42
23.91	22.32	23.24	24.37	25.92	26.12	24.46	25.85	23.29
24.4	21.55	24.28	26.13	25.07	.	.	26.12	22.69
2.7	1.01	2.39	1.08	2.98	5.1	2.56	1.72	3.14
2.94	1.59	2.39	1.95	3.18	4.41	2.79	2.42	3.09
3.77	2.02	2.98	3.04	3.91	4.79	3.99	3.95	3.78
6.12	3.02	3.68	3.52	6.41	7.87	6.69	5.73	6.65
15.22	5.87	11.74	11.39	15.3	.	.	13.22	11.6
26.39	22.93	25.52	26.66	27.65	31.86	27.44	26.78	25.87
26.41	23.76	25.95	27.54	28.46	29.95	26.56	28.09	26.22
27.81	24.55	26.89	28.41	29.49	31	27.94	29.97	26.2
30.03	25.34	26.92	27.89	32.33	33.99	31.15	31.58	29.94
39.62	27.42	36.02	37.52	40.37	#VALUE!	#VALUE!	39.34	34.29
10%	4%	9%	4%	11%	16%	9%	6%	12%
11%	7%	9%	7%	11%	15%	11%	9%	12%
14%	8%	11%	11%	13%	15%	14%	13%	14%
20%	12%	14%	13%	20%	23%	21%	18%	22%
38%	21%	33%	30%	38%	#VALUE!	#VALUE!	34%	34%
.	35.7	36	37.2	38.7	36	37.7	.	.
36.3	36.2	36.3	37.8	37.5	36.1	37.1	36.3	36.5
36.9	37.1	35.5	37.3	36.3	36.6	35.4	36.9	35.3
36.8	36.7	36.2	37	36.9	36.9	36.4	35.8	36.3

5	6	6	8	9	9	11	11
Box-2	Box-5	Box-7	Box-6	Box-4	Box-5	Box-3	Box-7
32.3	45	47	44.5	65	68	78	84
M	M	M	M	M	M	M	M
MCT	MCT	MCT	MCT	MCT	MCT	MCT	MCT
MT	MT	MT	MT	MT	MT	MT	MT
29.01	25.37	26.96	31.51	26.94	37.22	29.1	32.9
28.75	26.79	27.95	30.85	27.53	34.80	30.1	31.2
30.26	27.72	28.93	30.22	27.51	36.36	28.7	32.3
30.72	27.3	29.76	33.05	30.48	38.32	32.27	34.26
			41.37	35.18	45.51	.	.
26.22	22.33	24.08	28.56	23.81	27.95	25.40	27.05
25.6	22.62	24.35	27.34	23.57	26.46	25.67	25.89
26.17	23	24.37	26.13	23.43	26.96	24.29	25.79
25.06	22.09	23.05	26.25	23.86	27.28	24.83	25.29
25.27	23.67	25.98	25.83	.	.	.	.
1.78	1.62	1.51	1.57	2.5	8.37	2.53	4.84
2.1	2.47	2.12	2.55	2.55	6.97	3.2	4.24
2.66	3.31	3.38	2.7	3.07	8.13	3.23	5.07
4.05	3.62	4.36	5.22	5.58	9.76	5.71	7.38
12.18	6.7	10.08	13.59	.	.	.	.
28	23.95	25.59	30.13	26.31	36.32	27.93	31.89
27.7	25.09	26.47	29.89	26.12	33.43	28.87	30.13
28.83	26.31	27.75	28.83	26.5	35.09	27.52	30.86
29.11	25.71	27.41	31.47	29.44	37.04	30.54	32.67
37.45	30.37	36.06	39.42	#VALUE!	#VALUE!	#VALUE!	#VALUE!
6%	7%	6%	5%	10%	23%	9%	15%
8%	10%	8%	9%	10%	21%	11%	14%
9%	13%	12%	9%	12%	23%	12%	16%
14%	14%	16%	17%	19%	26%	19%	23%
33%	22%	28%	34%	#VALUE!	#VALUE!	#VALUE!	#VALUE!
37.3	35.3	38.2	38.4	37.6	37.8	36.1	35.4
35.7	37.9	37.6	36.5	37.8	37.2	35.7	36.5
36.6	37.2	38	36.6	37.2	36.7	35.5	35.8
36.5	36.4	37.5	36.8	37.8	36.9	36.6	36.4
