# Supplementary material for: Does prenatal alcohol exposure cause a metabolic syndrome? (Non-)evidence from a mouse model of fetal alcohol spectrum disorder
Source: PLoS One. 2018 Jun 28;13(6):e0199213. doi: 10.1371/journal.pone.0199213 (PMC6023152; doi:10.1371/journal.pone.0199213)
Supplement: S1 Dataset — (ZIP) [file pone.0199213.s010.zip › New folder/DEXA.pdf]

I don'	LITTER	SNO	SEX	TREAT	DEXA Weight	BMD (g/cm^2)	BMC (g)	Lean (g)	Fat (g)	Fat (%) PIXI	Lean (%) PIXI	Fat (%) BW	Lean (%) BW
6.4	6	4	FEMALE	ETOH	20.9	0.0466	0.601	14.9	3.6	19.46	80.54	17.22	71.29
9.6	9	6	FEMALE	ETOH	23.2	0.0481	0.672	17.1	3.8	18.18	81.82	16.38	73.71
10.6	10	6	FEMALE	ETOH	22.1	0.0479	0.572	15.9	3.8	19.29	80.71	17.19	71.95
11.1	11	1	FEMALE	ETOH	18.2	0.0427	0.472	13.3	3.1	18.90	81.10	17.03	73.08
17.8	17	8	FEMALE	ETOH	21.52	0.0521	0.514	15.1	3.5	18.82	81.18	16.26	70.17
18.7	18	7	FEMALE	ETOH	21.73	0.0491	0.578	20.5	4.5	18.00	82.00	20.71	94.34
r10.6	10	6	FEMALE	ETOH	22.1	0.0531	0.577	16.8	3.8	18.45	81.55	17.19	76.02
r11.1	11	1	FEMALE	ETOH	18.2	0.0507	0.509	13.2	3.8	22.35	77.65	20.88	72.53
r6.4	6	4	FEMALE	ETOH	20.9	0.0488	0.503	16.5	3.4	17.09	82.91	16.27	78.95
r9.6	9	6	FEMALE	ETOH	23.2	0.0515	0.550	17.7	3.7	17.29	82.71	15.95	76.29
28.7	28	7	FEMALE	ETOH	21.2	0.0522	0.494	15.6	4.2	21.21	78.79	19.81	73.58
41.1	41	1	FEMALE	ETOH	20.8	0.0489	0.487	15.9	3.5	18.04	81.96	16.83	76.44
42.5	42	5	FEMALE	ETOH	20.8	0.0521	0.521	15.8	3.7	18.97	81.03	17.79	75.96
53.5	53	5	FEMALE	ETOH	20.7	0.0493	0.510	16.1	3.1	16.15	83.85	14.98	77.78
71.7	71	7	FEMALE	ETOH	23.9	0.05	0.593	16.8	4.2	20.2	80.00	17.57	70.29
6.1	6	1	MALE	ETOH	25.1	0.0483	0.594	19.1	3.9	16.96	83.04	15.54	76.10
9.4	9	4	MALE	ETOH	26.1	0.0480	0.612	19.5	4.1	17.37	82.63	15.71	74.71
10.1	10	1	MALE	ETOH	26.6	0.0492	0.643	19.9	4.1	17.08	82.92	15.41	74.81
11.6	11	6	MALE	ETOH	23.2	0.0454	0.488	17.3	3.5	16.83	83.17	15.09	74.57
16.4	16	4	MALE	ETOH	29.32	0.0503	0.600	18.2	4.3	19.11	80.89	14.67	62.07
17.4	17	4	MALE	ETOH	27.88	0.0507	0.463	20.2	5.5	21.40	78.60	19.73	72.45
28.1	28	1	MALE	ETOH	25.5	0.0516	0.509	19.9	4.6	18.78	81.22	18.04	78.04
41.5	41	5	MALE	ETOH	26.8	0.0495	0.469	21.1	4.7	18.22	81.78	17.54	78.73
42.1	42	1	MALE	ETOH	28.6	0.0510	0.491	21.5	5.6	20.66	79.34	19.58	75.17
53.2	53	2	MALE	ETOH	28.4	0.0503	0.609	22.7	4.0	14.98	85.02	14.08	79.93
71.5	71	5	MALE	ETOH	28.9	0.05	0.531	21.1	5.5	20.6	79.32	19.03	73.01
82.6	82	6	MALE	ETOH	28.1	0.05	0.501	20.2	4.9	19.5	80.48	17.44	71.89
12.4	12	4	FEMALE	H2O	22.4	0.0491	0.605	16.4	4.1	20.00	80.00	18.30	73.21
23.1	23	1	FEMALE	H2O	22.58	0.0519	0.520	16.6	3.3	16.58	83.42	14.61	73.52
r12.4	12	4	FEMALE	H2O	22.4	0.0513	0.587	17.5	3.7	17.45	82.55	16.52	78.13
37.5	37	5	FEMALE	H2O	23	0.0519	0.502	16.6	4.0	19.42	80.58	17.39	72.17
46.6	46	6	FEMALE	H2O	21	0.0495	0.504	16.0	3.6	18.37	81.63	17.14	76.19
44.6	44	6	FEMALE	H2O	22.2	0.0517	0.477	17.8	3.5	16.43	83.57	15.77	80.18
50.6	50	6	FEMALE	H2O	21.4	0.0517	0.537	16.4	3.3	16.75	83.25	15.42	76.64
62.1	62	1	FEMALE	H2O	23.2	0.05	0.584	16.8	3.8	18.6	81.55	16.38	72.41
63.5	63	5	FEMALE	H2O	21.1	0.05	0.642	13.3	4.8	26.8	73.48	22.75	63.03
69.2	69	2	FEMALE	H2O	22.8	0.05	0.664	15.3	4.2	21.6	78.46	18.42	67.11
74.3	74	3	FEMALE	H2O	21.6	0.05	0.528	15.5	3.7	19.5	80.73	17.13	71.76
76.3	76	3	FEMALE	H2O	23.7	0.05	0.611	17.3	3.7	17.5	82.38	15.61	73.00
79.4	79	4	FEMALE	H2O	22.6	0.05	0.542	15.9	3.5	18.2	81.96	15.49	70.35
81.1	81	1	FEMALE	H2O	23.6	0.05	0.615	16.8	3.4	16.6	83.17	14.41	71.19
83.6	83	6	FEMALE	H2O	23.3	0.05	0.548	17	3.5	17.3	82.93	15.02	72.96
12.1	12	1	MALE	H2O	25.6	0.0505	0.584	18.5	4.3	18.86	81.14	16.80	72.27
23.3	23	3	MALE	H2O	27.5	0.0478	0.562	18.8	4.0	17.54	82.46	14.55	68.36

37.1	37	1	MALE	H2O	28.7	0.0538	0.514	22.0	5.1	18.82	81.18	17.77	76.66
46.3	46	3	MALE	H2O	25.1	0.0507	0.500	19.1	4.6	19.41	80.59	18.33	76.10
44.5	44	5	MALE	H2O	30.7	0.0538	0.600	24.6	4.2	14.58	85.42	13.68	80.13
50.1	50	1	MALE	H2O	27.7	0.0525	0.515	21.5	4.3	16.67	83.33	15.52	77.62
62.6	62	6	MALE	H2O	27.7	0.05	0.512	21	4.3	17	83.00	15.52	75.81
63.3	63	3	MALE	H2O	28.6	0.05	0.516	21.8	4.1	15.9	84.17	14.34	76.22
69.4	69	4	MALE	H2O	28.2	0.05	0.527	21.1	4.3	16.8	83.07	15.25	74.82
74.1	74	1	MALE	H2O	28.6	0.05	0.564	20.9	5	19.2	80.69	17.48	73.08
76.2	76	2	MALE	H2O	30.6	0.05	0.601	23.4	4.4	15.9	84.17	14.38	76.47
79.2	79	2	MALE	H2O	31.2	0.05	0.674	23.6	4.2	15.1	84.89	13.46	75.64
81.6	81	6	MALE	H2O	28.8	0.05	0.541	21.2	5.2	19.6	80.30	18.06	73.61
83.1	83	1	MALE	H2O	29.2	0.05	0.554	21.4	4.8	18.3	81.68	16.44	73.29
13.3	13	3	FEMALE	MCT	22.38	0.0483	0.558	21.9	4.1	15.77	84.23	18.32	97.86
14.6	14	6	FEMALE	MCT	20.11	0.0511	0.519	16.7	3.5	17.33	82.67	17.40	83.04
32.6	32	6	FEMALE	MCT	21.7	0.0531	0.532	16.4	3.6	18.00	82.00	16.59	75.58
45.3	45	3	FEMALE	MCT	22.9	0.0520	0.563	17.6	4.0	18.52	81.48	17.47	76.86
47.1	47	1	FEMALE	MCT	22	0.0498	0.533	16.8	3.7	18.05	81.95	16.82	76.36
65.6	65	6	FEMALE	MCT	20.9	0.05	0.588	14.7	3.7	20.2	79.89	17.70	70.33
68.6	68	6	FEMALE	MCT	24.4	0.05	0.695	17.2	4	18.9	81.13	16.39	70.49
77.4	77	4	FEMALE	MCT	21.9	0.05	0.527	15.7	3.9	19.7	80.10	17.81	71.69
78.8	78	8	FEMALE	MCT	23.1	0.05	0.583	16.7	3.6	17.9	82.27	15.58	72.29
84.4	84	4	FEMALE	MCT	23.6	0.05	0.529	16.9	3.7	18	82.04	15.68	71.61
13.1	13	1	MALE	MCT	28.48	0.0513	0.606	21.5	4.6	17.62	82.38	16.15	75.49
20.2	20	2	MALE	MCT	26.77	0.0498	0.577	20.0	4.2	17.36	82.64	15.69	74.71
32.3	32	3	MALE	MCT	26.8	0.0524	0.562	20.9	4.3	17.06	82.94	16.04	77.99
45.2	45	2	MALE	MCT	25.4	0.0503	0.487	19.3	4.3	18.22	81.78	16.93	75.98
47.2	47	2	MALE	MCT	26.8	0.0505	0.561	20.7	4.3	17.20	82.80	16.04	77.24
65.4	65	4	MALE	MCT	26.7	0.05	0.506	20	3.7	15.8	84.39	13.86	74.91
68.2	68	2	MALE	MCT	36.2	0.05	0.486	25	8.3	24.9	75.08	22.93	69.06
77.3	77	3	MALE	MCT	30	0.05	0.58	22	4.8	17.9	82.09	16.00	73.33
78.1	78	1	MALE	MCT	29.3	0.05	0.574	21.8	4.1	16	84.17	13.99	74.40
84.1	84	1	MALE	MCT	29.3	0.05	0.523	21.6	4.6	17.6	82.44	15.70	73.72
1.7	1	7	FEMALE	MD	22.4	0.0454	0.591	16.6	3.7	18.23	81.77	16.52	74.11
2.3	2	3	FEMALE	MD	22.9	0.0469	0.659	16.6	3.8	18.63	81.37	16.59	72.49
5.2	5	2	FEMALE	MD	20.1	0.0440	0.584	14.1	4.1	22.53	77.47	20.40	70.15
19.5	19	5	FEMALE	MD	26.09	0.0500	0.584	20.3	4.4	17.81	82.19	16.86	77.81
25.3	25	3	FEMALE	MD	22.7	0.0467	0.446	16.8	3.2	16.00	84.00	14.10	74.01
r1.7	1	7	FEMALE	MD	22.4	0.0507	0.506	16.9	3.9	18.75	81.25	17.41	75.45
r2.3	2	3	FEMALE	MD	22.9	0.0535	0.552	18.1	3.7	16.97	83.03	16.16	79.04
r5.2	5	2	FEMALE	MD	20.1	0.0506	0.529	16.0	3.5	17.95	82.05	17.41	79.60
34.6	34	6	FEMALE	MD	19.7	0.0496	0.477	15.1	3.3	17.93	82.07	16.75	76.65
38.5	38	5	FEMALE	MD	20.7	0.0511	0.507	16.1	3.7	18.69	81.31	17.87	77.78
43.6	43	6	FEMALE	MD	20.2	0.0476	0.495	15.2	3.7	19.58	80.42	18.32	75.25
52.6	52	6	FEMALE	MD	21.9	0.0513	0.551	17.2	3.4	16.50	83.50	15.53	78.54
64.5	64	5	FEMALE	MD	24.7	0.05	0.6	17.5	4	18.6	81.40	16.19	70.85

75.1	75	1	FEMALE	MD	22.4	0.05	0.526	16.1	3.5	17.9	82.14	15.63	71.88
1.1	1	1	MALE	MD	27.7	0.0481	0.540	19.2	3.6	15.79	84.21	13.00	69.31
2.2	2	2	MALE	MD	27.2	0.0461	0.611	20.6	4.0	16.26	83.74	14.71	75.74
5.5	5	5	MALE	MD	28.5	0.0467	0.699	21.6	4.1	15.95	84.05	14.39	75.79
19.1	19	1	MALE	MD	29.69	0.0478	0.550	15.8	3.8	19.39	80.61	12.80	53.22
25.1	25	1	MALE	MD	26.09	0.0521	0.586	16.0	3.5	17.95	82.05	13.42	61.33
34.2	34	2	MALE	MD	23.2	0.0497	0.463	18.3	4.0	17.94	82.06	17.24	78.88
38.1	38	1	MALE	MD	30.1	0.0492	0.461	19.7	4.8	19.59	80.41	15.95	65.45
43.4	43	4	MALE	MD	28.2	0.0489	0.512	22.3	4.2	15.85	84.15	14.89	79.08
52.2	52	2	MALE	MD	28.6	0.0498	0.515	21.9	4.9	18.28	81.72	17.13	76.57
64.2	64	2	MALE	MD	29.8	0.05	0.534	21.5	4.9	18.6	81.44	16.44	72.15
75.6	75	6	MALE	MD	27	0.05	0.555	20	4.4	17.9	81.97	16.30	74.07
