# Supplementary material for: Does prenatal alcohol exposure cause a metabolic syndrome? (Non-)evidence from a mouse model of fetal alcohol spectrum disorder
Source: PLoS One. 2018 Jun 28;13(6):e0199213. doi: 10.1371/journal.pone.0199213 (PMC6023152; doi:10.1371/journal.pone.0199213)
Supplement: S1 Dataset — (ZIP) [file pone.0199213.s010.zip › New folder/Feeding behavior.pdf]

| | | | | | | | | | | | | | | | | | | |
|---|---|---|---|---|---|---|---|---|---|---|---|---|---|---|---|---|---|---|
| Blk | | 3 | 3 | 3 | 3 | 4 | 4 | 5 | 7 | 7 | 8 | 3 | 4 | 4 | 7 | 7 | 8 | 10 |
| Box | | Box-3 | Box-4 | Box-5 | Box-6 | Box-2 | Box-3 | Box-4 | Box-3 | Box-4 | Box-4 | Box-7 | Box-5 | Box-8 | Box-1 | Box-7 | Box-2 | Box-6 |
| ID | | 6.4 | 9.6 | 10.6 | 11.1 | 17.8 | 18.7 | 28.7 | 41 | 42 | 53.5 | 12.4 | 23.1 | 27.7 | 37 | 46 | 50.6 | 62 |
| Sex | | F | F | F | F | F | F | F | F | F | F | F | F | F | F | F | F | F |
| Exposure | | EtOH | EtOH | EtOH | EtOH | EtOH | EtOH | EtOH | EtOH | EtOH | EtOH | H2O | H2O | H2O | H2O | H2O | H2O | H2O |
| Tx | | FE | FE | FE | FE | FE | FE | FE | FE | FE | FE | FH | FH | FH | FH | FH | FH | FH |
| 18:00 | 0 | 0 | 0 | 0 | 0.154545 | 0 | 0 | 0.018182 | 0 | 0 | 0 | 0 | 0 | 0 | 0 | 0.0090909 | 0 | 0 |
| | 1.1 | 0 | 0 | 0.136364 | 0.190909 | 0 | 0.054545 | 0.209091 | 0.118182 | 0.172727 | 0.190909 | 0 | 0 | 0 | 0 | 0.0090909 | 0 | 0 |
| | 2.2 | 0 | 0 | 0.154545 | 0.154545 | 0 | 0 | 0.254545 | 0.218182 | 0 | 0.118182 | 0 | 0 | 0 | 0 | 0 | 0 | 0 |
| | 3.3 | 0 | 0 | 0.263636 | 0.227273 | 0 | 0.327273 | 0.154545 | 0.309091 | 0.1 | 0.463636 | 0.027273 | 0 | 0.063636 | 0 | 0 | 0 | 0 |
| | 4.4 | 0.063636 | 0.081818 | 0.290909 | 0.218182 | 0 | 0.118182 | 0.136364 | 0.1 | 0.609091 | 0.127273 | 0.436364 | 0.509091 | 0.427273 | 0 | 0.3545455 | 0.018182 | 0 |
| | 5.5 | 0.8 | 0.572727 | 0.645455 | 0.263636 | 0 | 0.290909 | 0.336364 | 0.109091 | 0.2 | 0.190909 | 0.1 | 0.4 | 0.463636 | 0.309091 | 0.3818182 | 0.5 | 0 |
| | 6.6 | 0.227273 | 0.1 | 0.081818 | 0 | 0 | 0.236364 | 0.690909 | 0.118182 | 0.427273 | 0.4 | 0.381818 | 0.563636 | 0.254545 | 0.381818 | 0.4363636 | 0 | 0 |
| | 7.7 | 0.190909 | 0.490909 | 0.254545 | 0.281818 | 0 | 0.254545 | 0 | 0.081818 | 0.063636 | 0.081818 | 0.263636 | 0 | 0.627273 | 0.572727 | 0.2090909 | 0.772727 | 0 |
| | 8.8 | 0.327273 | 0.227273 | 0.336364 | 0 | 0 | 0.209091 | 0 | 0.018182 | 0 | 0.127273 | 0.372727 | 0.472727 | 0 | 0.436364 | 0.4090909 | 0.118182 | 0 |
| | 9.9 | 0.018182 | 0.090909 | 0.163636 | 0 | 0 | 0 | 0.145455 | 0.154545 | 0 | 0 | 0.145455 | 0.109091 | 0.009091 | 0.1 | 0.2363636 | 0.036364 | 0 |
| | 11 | 0 | 0.445455 | 0.190909 | 0 | 0 | 0.118182 | 0.227273 | 0 | 0 | 0.054545 | 0 | 0 | 0.2 | 0.009091 | 0 | 0.518182 | 0 |
| | 12.1 | 0 | 0.027273 | 0.227273 | 0 | 0.181818 | 0.227273 | 0 | 0.1 | 0 | 0.054545 | 0.354545 | 0.409091 | 0.181818 | 0 | 0.2363636 | 0.054545 | 0 |
| | 13.2 | 0 | 0.309091 | 0.181818 | 0.354545 | 0.472727 | 0.109091 | 0.545455 | 0.009091 | 0.045455 | 0.009091 | 0 | 0.536364 | 0.109091 | 0.663636 | 0.6363636 | 0.336364 | 0 |
| | 14.3 | 0.227273 | 0.127273 | 0 | 0 | 0.5 | 0.336364 | 0.072727 | 0.354545 | 0 | 0.427273 | 0 | 0.145455 | 0.118182 | 0 | 0 | 0.018182 | 0 |
| | 15.4 | 0.090909 | 0.036364 | 0.190909 | 0.309091 | 0 | 0 | 0.272727 | 0.054545 | 0 | 0 | 0.109091 | 0.109091 | 0.136364 | 0.436364 | 0.2090909 | 0 | 0 |
| | 16.5 | 0.245455 | 0.227273 | 0.1 | 0 | 0 | 0.263636 | 0 | 0 | 0 | 0.327273 | 0.145455 | 0.236364 | 0.190909 | 0 | 0.2181818 | 0 | 0 |
| | 17.6 | 0.181818 | 0.118182 | 0.136364 | 0 | 0.027273 | 0 | 0.254545 | 0.063636 | 0.090909 | 0 | 0.009091 | 0.254545 | 0 | 0.245455 | 0 | 0.063636 | 0 |
| | 18.7 | 0.363636 | 0 | 0.136364 | 0.290909 | 0.654545 | 0.181818 | 0.063636 | 0 | 0.063636 | 0 | 0 | 0 | 0.181818 | 0.272727 | 0.2636364 | 0.145455 | 0 |
| | 19.8 | 0 | 0 | 0 | 0.045455 | 0.227273 | 0 | 0.309091 | 0 | 0.018182 | 0.218182 | 0.172727 | 0 | 0.163636 | 0 | 0.0181818 | 0.145455 | 0 |
| | 20.9 | 0.563636 | 0 | 0.136364 | 0 | 0.290909 | 0 | 0 | 0 | 0.336364 | 0.309091 | 0.254545 | 0.109091 | 0.1 | 0.236364 | 0.2818182 | 0.118182 | 0 |
| | 22 | 0 | 0.090909 | 0.254545 | 0.281818 | 0.145455 | 0.281818 | 0.245455 | 0.009091 | 0 | 0 | 0 | 0.218182 | 0.309091 | 0.027273 | 0.2 | 0 | 0 |
| | 23.1 | 0.1 | 0.045455 | 0.227273 | 0 | 0.172727 | 0.3 | 0.209091 | 0.245455 | 0.172727 | 0.254545 | 0.145455 | 0.227273 | 0 | 0.154545 | 0 | 0.372727 | 0 |
| | 24.2 | 0.036364 | 0.109091 | 0.036364 | 0.263636 | 0.036364 | 0 | 0.254545 | 0.172727 | 0.245455 | 0.209091 | 0.045455 | 0.1 | 0 | 0 | 0.2181818 | 0.145455 | 0 |
| | 25.3 | 0.209091 | 0.154545 | 0.527273 | 0.290909 | 0.436364 | 0.272727 | 0.218182 | 0.136364 | 0.272727 | 0.290909 | 0.054545 | 0.209091 | 0.272727 | 0.327273 | 0.1727273 | 0.354545 | 0 |
| | 26.4 | 0.118182 | 0.509091 | 0.2 | 0 | 0.118182 | 0 | 0.481818 | 0.218182 | 0.5 | 0.290909 | 0.018182 | 0.2 | 0.2 | 0.254545 | 0 | 0.3 | 0 |
| | 27.5 | 0.390909 | 0.127273 | 0.245455 | 0.309091 | 0 | 0.254545 | 0.263636 | 0.418182 | 0.236364 | 0.254545 | 0.263636 | 0.045455 | 0.5 | 0.327273 | 0.2909091 | 0.027273 | 0 |
| | 28.6 | 0.445455 | 0.027273 | 0.218182 | 0 | 0 | 0 | 0.263636 | 0.236364 | 0.272727 | 0.345455 | 0.227273 | 0.4 | 0.145455 | 0.145455 | 0.1545455 | 0.381818 | 0 |
| | 29.7 | 0.354545 | 0.518182 | 0.209091 | 0.227273 | 0.063636 | 0.018182 | 0.327273 | 0.345455 | 0.345455 | 0.181818 | 0.2 | 0 | 0.081818 | 0.090909 | 0.2181818 | 0.272727 | 0 |
| | 30.8 | 0.354545 | 0.218182 | 0.372727 | 0.227273 | 0 | 0.218182 | 0.209091 | 0 | 0 | 0.3 | 0.245455 | 0.136364 | 0.009091 | 0.3 | 0.3454545 | 0.618182 | 0 |
| | 31.9 | 0.081818 | 0.454545 | 0.127273 | 0.218182 | 0.2 | 0.063636 | 0.1 | 0.436364 | 0.409091 | 0.227273 | 0.254545 | 0.1 | 0.236364 | 0.481818 | 0 | 0.263636 | 0.7 |
| | 33 | 0.209091 | 0 | 0.018182 | 0 | 0 | 0 | 0.272727 | 0.290909 | 0.163636 | 0.145455 | 0.227273 | 0.445455 | 0.318182 | 0.009091 | 0.1363636 | 0.072727 | 0.472727 |
| | 34.1 | 0.036364 | 0.290909 | 0.327273 | 0 | 0.309091 | 0.172727 | 0 | 0 | 0.009091 | 0 | 0.109091 | 0.127273 | 0 | 0 | 0.2454545 | 0 | 0.181818 |
| | 35.2 | 0.081818 | 0 | 0.081818 | 0.209091 | 0.009091 | 0.290909 | 0.145455 | 0.209091 | 0.009091 | 0.318182 | 0.245455 | 0.009091 | 0.109091 | 0.163636 | 0.2 | 0.218182 | 0.118182 |
| | 36.3 | 0 | 0.090909 | 0.245455 | 0 | 0.154545 | 0.236364 | 0 | 0.009091 | 0.036364 | 0.281818 | 0 | 0.227273 | 0.2 | 0 | 0.0818182 | 0.027273 | 0.418182 |
| | 37.4 | 0.327273 | 0.190909 | 0.236364 | 0 | 0.454545 | 0 | 0.172727 | 0 | 0.354545 | 0 | 0.190909 | 0.463636 | 0.172727 | 0.354545 | 0.0272727 | 0 | 0.3 |
| | 38.5 | 0 | 0.263636 | 0.136364 | 0 | 0.336364 | 0.363636 | 0.218182 | 0 | 0.309091 | 0.163636 | 0.036364 | 0.645455 | 0.263636 | 0.145455 | 0.2363636 | 0.227273 | 0.1 |
| | 39.6 | 0.227273 | 0 | 0.109091 | 0.263636 | 0.381818 | 0.336364 | 0.127273 | 0.245455 | 0 | 0.063636 | 0.336364 | 0.145455 | 0.118182 | 0.063636 | 0.2272727 | 0.154545 | 0.290909 |
| | 40.7 | 0.218182 | 0.163636 | 0 | 0.190909 | 0.045455 | 0.263636 | 0 | 0 | 0.390909 | 0.027273 | 0.054545 | 0 | 0.236364 | 0.227273 | 0.2818182 | 0.227273 | 0.281818 |
| | 41.8 | 0.154545 | 0.118182 | 0.218182 | 0 | 0.109091 | 0 | 0.381818 | 0.109091 | 0 | 0.181818 | 0.081818 | 0.254545 | 0.127273 | 0.218182 | 0.1818182 | 0.190909 | 0.163636 |
| | 42.9 | 0.254545 | 0.2 | 0 | 0.254545 | 0.245455 | 0.018182 | | 0 | 0.390909 | 0 | 0 | 0.409091 | 0.236364 | 0.154545 | 0.2545455 | 0.145455 | 0 |
| | 44 | 0.109091 | 0 | 0.090909 | 0 | 0.172727 | 0 | | 0.218182 | 0 | 0.072727 | 0.336364 | 0.172727 | 0.218182 | 0 | 0.2272727 | 0 | 0 |
| | 45.1 | 0.118182 | 0.054545 | 0.327273 | 0 | 0.1 | 0 | | 0 | 0.345455 | 0.209091 | 0.263636 | 0 | 0.081818 | 0.309091 | 0.0363636 | 0.2 | 0.190909 |
| | 46.2 | 0.263636 | 0.218182 | 0.154545 | 0.3 | 0 | 0.327273 | | 0.263636 | 0 | 0.218182 | 0 | 0 | 0.227273 | 0.263636 | 0.4727273 | 0 | 0 |
| | 47.3 | 0.145455 | 0.045455 | 0.2 | 0.318182 | 0.481818 | 0.254545 | | 0.009091 | 0.563636 | 0.027273 | 0.209091 | 0.036364 | 0 | 0.254545 | 0 | 0.390909 | 0 |
| | 48.4 | 0.009091 | 0.218182 | 0.4 | 0.327273 | 0 | 0 | | 0.227273 | 0.018182 | 0.336364 | 0.3 | 0.318182 | 0.118182 | 0 | 0.0181818 | 0.245455 | 0.072727 |
| | 49.5 | 0.554545 | 0.236364 | 0.318182 | 0 | 0.418182 | 0.290909 | | 0.290909 | 0.481818 | 0.181818 | 0.3 | 0 | 0 | 0.381818 | 0.3636364 | 0.2 | 0.227273 |
| | 50.6 | 0.209091 | 0.336364 | 0.363636 | 0.209091 | 0.027273 | 0 | | 0.290909 | 0.136364 | 0.372727 | 0.027273 | 0.3 | 0.109091 | 0.263636 | 0.2636364 | 0.563636 | 0.354545 |
| | 51.7 | 0.354545 | 0.290909 | 0.254545 | 0.290909 | 0 | 0 | | 0 | 0.563636 | 0.327273 | 0.381818 | 0.090909 | 0.172727 | 0.490909 | 0.3090909 | 0.1 | 0.5 |
| | 52.8 | 0.309091 | 0.1 | 0.163636 | 0 | 0.545455 | 0.190909 | | 0.318182 | 0 | 0.409091 | 0.136364 | 0.318182 | 0 | 0 | 0.2454545 | 0.263636 | 0 |
| | 53.9 | 0.545455 | 0.454545 | 0.254545 | 0.363636 | | | | 0.436364 | 0 | 0 | 0.054545 | | | 0.472727 | 0.3272727 | 0.163636 | 0.290909 |
| | 55 | 0.236364 | 0.036364 | 0.2 | 0 | | | | 0.345455 | 0.427273 | 0.218182 | 0.190909 | | | 0 | 0.2636364 | 0.327273 | 0.336364 |
| | 56.1 | 0 | 0.127273 | 0.036364 | 0 | | | | 0.381818 | 0 | 0.254545 | 0.227273 | | | 0.281818 | 0 | 0.154545 | 0.6 |
| | 57.2 | 0 | 0.236364 | 0.118182 | 0 | | | | 0 | 0.263636 | 0.018182 | 0.363636 | | | 0.009091 | 0.1272727 | 0 | 0.209091 |
| | 58.3 | 0.254545 | 0.109091 | 0.163636 | 0.309091 | | | | 0 | 0.036364 | 0 | 0 | | | 0 | 0 | 0.318182 | 0 |
| | 59.4 | 0.109091 | 0.345455 | 0.054545 | 0 | | | | 0.354545 | 0 | 0.254545 | 0.018182 | | | 0.272727 | 0.3 | 0 | 0.181818 |
| | 60.5 | 0.009091 | 0.054545 | 0.163636 | 0 | | | | 0 | 0.227273 | 0.2 | 0.290909 | | | 0.236364 | 0.1909091 | 0.336364 | 0.281818 |
| | 61.6 | 0 | 0.1 | 0.227273 | 0.318182 | | | | 0 | 0.072727 | 0 | 0 | | | 0.036364 | 0 | 0.127273 | 0.281818 |
| | 62.7 | 0.136364 | 0.154545 | 0.118182 | 0 | | | | 0.409091 | 0.154545 | 0.236364 | 0.109091 | | | 0.290909 | 0.2545455 | 0.063636 | 0 |
| | 63.8 | 0.209091 | 0 | 0 | 0 | | | | 0 | 0.363636 | 0 | 0.2 | | | 0 | 0.2818182 | 0.118182 | 0.318182 |
| | 64.9 | 0 | 0.045455 | 0.145455 | 0.163636 | | | | 0 | 0 | 0.127273 | 0 | | | 0.263636 | 0 | 0.072727 | 0.027273 |
| | 66 | 0.109091 | 0.081818 | 0.127273 | 0 | | | | 0.072727 | 0.254545 | 0.209091 | 0.218182 | | | 0 | 0.2090909 | 0 | 0.3 |
| | | | | | | | | | | | | | | | | | | 0 |
| 18:00 | 68.1 | 0.2 | 0.263636 | 0.036364 | 0.363636 | 0.327273 | 0.472727 | 0.2 | 0.136364 | 0.463636 | | 0.236364 | 0.227273 | 0.190909 | 0.263636 | 0.1454545 | 0.090909 | 0.181818 |
| | 69.2 | 0.436364 | 0.018182 | 0.281818 | 0.3 | 0.027273 | 0.009091 | 0.009091 | 0.272727 | 0 | | 0.236364 | 0.245455 | 0.218182 | 0.045455 | 0.2454545 | 0.190909 | 0.272727 |
| | 70.3 | 0.309091 | 0.372727 | 0.327273 | 0.218182 | 0.072727 | 0 | 0.090909 | 0.481818 | 0.472727 | | 0.309091 | 0.218182 | 0.081818 | 0.018182 | 0.0727273 | 0.127273 | 0.063636 |
| | 71.4 | 0 | 0.090909 | 0.172727 | 0.009091 | 0.490909 | 0 | 0.154545 | 0 | 0.009091 | | 0 | 0.063636 | 0.3 | 0.3 | 0.2636364 | 0.263636 | 0.4 |
| | 72.5 | 0.054545 | 0.4 | 0.254545 | 0.145455 | 0.1 | 0.218182 | 0.272727 | 0.127273 | 0 | | 0.463636 | 0.290909 | 0.236364 | 0.054545 | 0.2909091 | 0.263636 | 0 |
| | 73.6 | 0.354545 | 0.136364 | 0 | 0.172727 | 0.427273 | 0.309091 | 0.145455 | 0.063636 | 0.363636 | | 0 | 0.236364 | 0 | 0.127273 | 0.3 | 0.290909 | 0.454545 |
| | 74.7 | 0.072727 | 0 | 0 | 0 | 0 | 0.172727 | 0.327273 | 0.154545 | 0 | | 0.027273 | 0.009091 | 0.2 | 0.1 | 0 | 0.345455 | 0 |
| | 75.8 | 0.372727 | 0.145455 | 0.2 | 0.181818 | 0.1 | 0.127273 | 0.172727 | 0.163636 | 0.363636 | | 0.1 | 0.136364 | 0 | 0.272727 | 0.2363636 | 0.218182 | 0.572727 |
| | 76.9 | 0 | 0.045455 | 0 | 0.354545 | 0.181818 | 0 | 0.2 | 0 | 0 | | 0.409091 | 0 | 0.1 | 0.163636 | 0.4818182 | 0 | 0.254545 |
| | 78 | 0.027273 | 0.009091 | 0 | 0 | 0.109091 | 0.363636 | 0 | 0.109091 | 0 | | 0.090909 | 0 | 0.290909 | 0 | 0 | 0.090909 | 0 |
| | 79.1 | 0 | 0 | 0.072727 | 0 | 0.009091 | 0 | 0.263636 | 0.063636 | 0 | | 0 | 0 | 0 | 0 | 0 | 0.054545 | 0 |
| | 80.2 | 0.172727 | 0.1 | 0 | 0 | 0.027273 | 0 | 0 | 0.190909 | 0 | | 0.1 | 0.063636 | 0.354545 | 0.081818 | 0.0090909 | 0 | 0.163636 |
| | 81.3 | 0.209091 | 0.254545 | 0.172727 | 0.181818 | 0.327273 | 0.3 | 0.381818 | 0.227273 | 0.518182 | | 0.263636 | 0.1 | 0.145455 | 0.081818 | 0.1727273 | 0.118182 | 0 |
| | 82.4 | 0.172727 | 0.218182 | 0.172727 | 0.009091 | 0.081818 | 0 | 0.209091 | 0.190909 | 0 | | 0.045455 | 0.245455 | 0.318182 | 0.436364 | 0.0909091 | 0.109091 | 0.672727 |
| | 83.5 | 0 | 0.045455 | 0.118182 | 0.154545 | 0.218182 | 0.363636 | 0 | 0.036364 | 0.536364 | | 0 | 0.218182 | 0 | 0.136364 | 0 | 0.2 | 0 |
| | 84.6 | 0.027273 | 0.318182 | 0.081818 | 0.163636 | 0.1 | 0 | 0.245455 | 0.236364 | 0 | | 0.154545 | 0.509091 | 0.1 | 0.245455 | 0.0545455 | 0.063636 | 0 |
| | 85.7 | 0.054545 | 0.036364 | 0.290909 | 0.027273 | 0.172727 | 0.254545 | 0.045455 | 0.018182 | 0 | | 0.009091 | 0.436364 | 0.272727 | 0.3 | 0 | 0.263636 | 0 |
| | 86.8 | 0.063636 | 0.109091 | 0.409091 | 0.136364 | 0.381818 | 0.027273 | 0 | 0 | 0.527273 | | 0.172727 | 0 | 0.272727 | 0 | 0.2181818 | 0.109091 | 0.290909 |
| | 87.9 | 0.254545 | 0.263636 | 0.009091 | 0 | 0.027273 | 0.372727 | 0.045455 | 0.154545 | 0 | | 0 | 0.454545 | 0 | 0.036364 | 0.0636364 | 0 | 0 |
| | 89 | 0.209091 | 0.027273 | 0.227273 | 0.345455 | 0.372727 | 0 | 0.072727 | 0.2 | 0 | | 0.427273 | 0.127273 | 0.236364 | 0.136364 | 0.2636364 | 0.527273 | 0.4 |
| | 90.1 | 0.218182 | 0.254545 | 0.181818 | 0.036364 | 0.354545 | 0.5 | 0.3 | 0.5 | 0 | | 0.545455 | 0.290909 | 0.463636 | 0 | 0 | 0.418182 | 0 |
| | 91.2 | 0.418182 | 0.445455 | 0.072727 | 0.245455 | 0.081818 | 0 | 0.218182 | 0 | 0.245455 | | 0 | 0.172727 | 0 | 0.290909 | 0.5363636 | 0 | 0.345455 |
| | 92.3 | 0.272727 | 0.027273 | 0.336364 | 0.336364 | 0.045455 | 0.390909 | 0.009091 | 0.563636 | 0 | | 0 | 0.190909 | 0.509091 | 0 | 0 | 0.354545 | 0 |
| | 93.4 | 0 | 0.436364 | 0.263636 | 0.263636 | 0 | 0 | 0.2 | 0 | 0.054545 | | 0.154545 | 0.390909 | 0 | 0.254545 | 0 | 0.281818 | 0.6 |
| | 94.5 | 0.236364 | 0.163636 | 0 | 0.390909 | 0.045455 | 0 | 0.445455 | 0 | 0.290909 | | 0.163636 | 0.354545 | 0 | 0 | 0.4181818 | 0.454545 | 0.327273 |
| | 95.6 | 0.090909 | 0.236364 | 0.154545 | 0.009091 | 0.263636 | 0.345455 | 0 | 0.127273 | 0 | | 0.318182 | 0.309091 | 0.145455 | 0.563636 | 0 | 0.336364 | 0 |
| | 96.7 | 0.081818 | 0.318182 | 0.227273 | 0.272727 | 0 | 0 | 0.036364 | 0.118182 | 0.009091 | | 0.445455 | 0 | 0.272727 | 0.054545 | 0.2818182 | 0.290909 | 0.172727 |
| | 97.8 | 0 | 0.3 | 0.081818 | 0 | 0.363636 | 0 | 0.536364 | 0 | 0 | | 0.263636 | 0.081818 | 0.018182 | 0 | 0.1090909 | 0 | 0 |
| | 98.9 | 0.081818 | 0.081818 | 0.127273 | 0 | 0.009091 | 0 | 0 | 0.145455 | 0.736364 | | 0 | 0.372727 | 0.536364 | 0.472727 | 0 | 0.363636 | 0.218182 |
| | 100 | 0 | 0.090909 | 0.054545 | 0.263636 | 0.163636 | 0.4 | 0.063636 | 0 | 0 | | 0.018182 | 0 | 0 | #VALUE! | 0.1909091 | 0 | 0.318182 |
| | 101.1 | 0.063636 | 0.054545 | 0 | 0.227273 | 0.054545 | 0 | 0 | 0.418182 | 0 | | 0.281818 | 0 | 0 | #VALUE! | 0 | 0.3 | 0.436364 |
| | 102.2 | 0 | 0.154545 | 0.3 | 0 | 0.109091 | 0 | 0 | 0 | 0 | | 0 | 0 | 0.4 | #VALUE! | 0 | 0 | 0 |
| | 103.3 | 0.090909 | 0 | 0.109091 | 0.463636 | 0.027273 | 0.209091 | 0 | 0.272727 | 0 | | 0.254545 | 0 | 0 | #VALUE! | 0.2 | 0 | 0.481818 |
| | 104.4 | 0.090909 | 0.154545 | 0 | 0.018182 | 0.227273 | 0.072727 | 0 | 0 | 0 | | 0.027273 | 0.309091 | 0.345455 | #VALUE! | 0 | 0.390909 | 0 |
| | 105.5 | 0.190909 | 0.254545 | 0.081818 | 0 | 0.109091 | 0 | 0.054545 | 0 | 0.3 | | 0.318182 | 0.281818 | 0 | #VALUE! | 0.3454545 | 0 | 0 |
| | 106.6 | 0.236364 | 0 | 0 | 0.254545 | 0.072727 | 0.2 | 0 | 0.345455 | 0 | | 0 | 0.009091 | 0.227273 | #VALUE! | 0 | 0.172727 | 0 |
| | 107.7 | 0.090909 | 0.354545 | 0 | 0.2 | 0.081818 | 0 | 0.063636 | 0 | 0 | | 0 | 0.054545 | 0 | #VALUE! | 0 | 0.190909 | 0.236364 |
| | 108.8 | 0.163636 | 0.018182 | 0.245455 | 0 | 0.081818 | 0.618182 | 0.118182 | 0.009091 | 0 | | 0.009091 | 0.218182 | 0.527273 | #VALUE! | 0.1636364 | 0 | 0 |
| | 109.9 | 0.218182 | 0.054545 | 0 | 0 | 0.109091 | 0.009091 | 0 | 0.4 | 0.890909 | | 0.154545 | 0.118182 | 0 | #VALUE! | 0 | 0.290909 | 0.163636 |

| | | | | | | | | | | | | | | | | | | |
|---|---|---|---|---|---|---|---|---|---|---|---|---|---|---|---|---|---|---|
| | 111 | 0 | 0 | 0.281818 | 0 | 0.072727 | 0 | 0 | 0 | 0 | | 0.209091 | 0.1 | 0 | #VALUE! | 0 | 0.009091 | 0.227273 |
| | 112.1 | 0.136364 | 0.363636 | 0 | 0.136364 | 0.172727 | 0.581818 | | 0.009091 | 0 | | 0.5 | 0.109091 | 0.518182 | #VALUE! | 0.4909091 | 0.154545 | 0.118182 |
| | 113.2 | 0.427273 | 0.272727 | 0.181818 | 0.109091 | 0.081818 | 0 | | 0.072727 | 0 | | 0 | 0 | 0 | #VALUE! | 0 | 0.490909 | 0 |
| | 114.3 | 0.118182 | 0.281818 | 0.2 | 0.045455 | 0.509091 | 0.318182 | | 0.618182 | 0 | | 0.427273 | 0.272727 | 0 | #VALUE! | 0.4727273 | 0 | 0.818182 |
| | 115.4 | 0.118182 | 0.345455 | 0.409091 | 0.227273 | 0.027273 | 0.045455 | | 0 | 0 | | 0 | 0.345455 | 0.154545 | #VALUE! | 0 | 0.009091 | 0 |
| | 116.5 | 0.181818 | 0 | 0.090909 | 0.236364 | 0.027273 | 0 | | 0.009091 | 0.818182 | | 0.036364 | 0.418182 | 0.136364 | #VALUE! | 0.2272727 | 0.536364 | 0 |
| | 117.6 | 0.354545 | 0.036364 | 0.4 | 0 | 0.227273 | 0.272727 | | 0.172727 | 0 | | 0.318182 | 0.9 | 0.163636 | 0.109091 | 0 | 0 | 0.1 |
| | 118.7 | 0 | 0.209091 | 0 | 0.581818 | 0.227273 | 0 | | 0.018182 | 0 | | 0 | 0.218182 | 0 | 0 | 0.2090909 | 0.063636 | 0.509091 |
| | 119.8 | 0.445455 | 0.136364 | 0.154545 | 0 | 0.063636 | 0.463636 | | 0.263636 | 0 | | 0 | 0.118182 | 0.327273 | 0.663636 | 0 | 0.190909 | 0.009091 |
| | 120.9 | 0.309091 | 0.027273 | 0.454545 | 0.409091 | 0.2 | 0.163636 | | 0 | 0 | | 0.1 | 0 | 0 | 0 | 0 | 0.281818 | 0 |
| | 122 | 0 | 0.081818 | 0 | 0 | 0.018182 | 0 | | 0.009091 | 0 | | 0 | 0 | 0.472727 | 0.836364 | 0.2090909 | 0 | 0.490909 |
| | 123.1 | 0.354545 | 0.118182 | 0.345455 | 0.190909 | 0.027273 | 0 | | 0.445455 | 0 | | 0.7 | 0.063636 | 0 | 0 | 0 | 0 | 0.027273 |
| | 124.2 | 0.218182 | 0 | 0.009091 | 0.254545 | 0.018182 | 0.118182 | | 0 | 0 | | 0 | 0.018182 | 0 | 0 | 0 | 0 | 0.172727 |
| | 125.3 | 0 | 0.263636 | 0 | 0.4 | 0.081818 | 0 | | 0 | 0 | | 0.227273 | 0.1 | 0 | 0 | 0.0727273 | 0 | 0.009091 |
| | 126.4 | 0.172727 | 0.309091 | 0.163636 | 0.145455 | 0.009091 | 0.054545 | | 0.190909 | 0 | | 0 | 0.009091 | 0.327273 | 0 | 0.0363636 | 0 | 0.554545 |
| | 127.5 | 0 | 0 | 0 | 0 | 0.054545 | 0 | | 0.381818 | 0 | | 0.027273 | 0 | 0 | 0 | 0.0272727 | 0 | 0 |
| | 128.6 | 0 | 0.018182 | 0.045455 | 0 | 0 | 0 | | 0 | 0.009091 | | 0 | 0.145455 | 0.072727 | 0 | 0.1454545 | 0.209091 | 0.454545 |
| | 129.7 | 0.527273 | 0.227273 | 0.209091 | 0 | 0.018182 | 0.418182 | | 0 | 0 | | 0.218182 | 0.009091 | 0.081818 | 0 | 0 | 0 | 0 |
| | 130.8 | 0 | 0.309091 | 0 | 0.209091 | 0.236364 | 0 | | 0.172727 | 0.136364 | | 0 | 0.372727 | 0.072727 | 0 | 0.0090909 | 0 | 0.490909 |
| | 131.9 | 0.081818 | 0 | 0.009091 | 0 | 0 | 0.209091 | | 0.209091 | 0.872727 | | 0 | 0.090909 | 0.1 | 0 | 0.1090909 | 0.290909 | 0 |
| | 133 | 0.090909 | 0.027273 | 0 | 0.009091 | 0.3 | 0 | | 0 | 0 | | 0.3 | 0 | 0.227273 | 0 | 0.1454545 | 0.018182 | 0 |
| | 134.1 | | | | | | | | 0.009091 | 0.045455 | | | | | 0 | 0 | | 0.072727 |
| 2:22 | 140.84 | 0.026667 | 0.026667 | 0 | 0.046667 | 0.227273 | 0.272727 | 0.236364 | 0.263636 | 0.236364 | 0.027273 | 0.073333 | 0.209091 | 0.236364 | 0.309091 | 0.1545455 | 0 | 0.2 |
| | 141.94 | 0.096667 | 0.01 | 0.076667 | 0.076667 | 0.036364 | 0.1 | 0.290909 | 0.263636 | 0.018182 | 0.209091 | 0 | 0.136364 | 0.009091 | 0.372727 | 0.1363636 | 0.109091 | 0.354545 |
| | 143.04 | 0.096667 | 0.033333 | 0.123333 | 0.053333 | 0 | 0.045455 | 0.254545 | 0 | 0.290909 | 0.090909 | 0.05 | 0.054545 | 0.081818 | 0.163636 | 0.1727273 | 0.163636 | 0.381818 |
| | 144.14 | 0.03 | 0.04 | 0 | 0 | 0.227273 | 0 | 0 | 0.272727 | 0.127273 | 0.063636 | 0.036667 | 0.254545 | 0.045455 | 0.318182 | 0.2636364 | 0.145455 | 0.154545 |
| | 145.24 | 0.05 | 0.063333 | 0 | 0.07 | 0.072727 | 0.218182 | 0.190909 | 0.136364 | 0 | 0.127273 | 0.063333 | 0.018182 | 0 | 0.190909 | 0 | 0.290909 | 0.172727 |
| | 146.34 | 0.073333 | 0.073333 | 0.056667 | 0 | 0.018182 | 0.118182 | 0 | 0.2 | 0.218182 | 0.172727 | 0.07 | 0.236364 | 0.127273 | 0 | 0 | 0.290909 | 0.009091 |
| | 147.44 | 0.04 | 0.07 | 0.05 | 0.016667 | 0.090909 | 0 | 0 | 0 | 0.009091 | 0.109091 | 0.026667 | 0.163636 | 0 | 0.463636 | 0.0181818 | 0.236364 | 0.009091 |
| | 148.54 | 0.006667 | 0 | 0.07 | 0.036667 | 0 | 0.127273 | 0 | 0 | 0.181818 | 0 | 0.003333 | 0 | 0.154545 | 0 | 0 | 0.054545 | 0.090909 |
| | 149.64 | 0.036667 | 0.04 | 0 | 0 | 0.109091 | 0 | 0.009091 | 0.172727 | 0 | 0.272727 | 0.046667 | 0.172727 | 0 | 0 | 0.1545455 | 0 | 0 |
| | 150.74 | 0.003333 | 0 | 0.036667 | 0.096667 | 0.163636 | 0 | 0.018182 | 0 | 0 | 0.054545 | 0 | 0 | 0.081818 | 0 | 0.0090909 | 0.090909 | 0.009091 |
| 18:00 | 151.84 | 0.016667 | 0 | 0.06 | 0.003333 | 0 | 0.063636 | 0.181818 | 0.218182 | 0.254545 | 0.081818 | 0 | 0.018182 | 0 | 0 | 0 | 0.2 | 0 |
| | 152.94 | 0.036667 | 0.016667 | 0.003333 | 0.036667 | 0.109091 | 0.063636 | 0 | 0.027273 | 0 | 0.027273 | 0.12 | 0.081818 | 0.027273 | 0 | 0.0272727 | 0.009091 | 0.009091 |
| | 154.04 | 0.053333 | 0.08 | 0.013333 | 0 | 0.036364 | 0.009091 | 0.363636 | 0 | 0.1 | 0.227273 | 0.056667 | 0 | 0 | 0.036364 | 0 | 0 | 0.354545 |
| | 155.14 | 0.016667 | 0.006667 | 0.026667 | 0.016667 | 0.281818 | 0.245455 | 0 | 0.290909 | 0 | 0 | 0.053333 | 0.045455 | 0.218182 | 0.054545 | 0.4272727 | 0.190909 | 0 |
| | 156.24 | 0.013333 | 0.043333 | 0.036667 | 0.033333 | 0.009091 | 0.172727 | 0.190909 | 0 | 0.436364 | 0.127273 | 0.07 | 0.081818 | 0.072727 | 0.009091 | 0.1363636 | 0 | 0.254545 |
| | 157.34 | 0.026667 | 0.02 | 0.006667 | 0 | 0.118182 | 0.190909 | 0.245455 | 0 | 0 | 0.136364 | 0 | 0.009091 | 0 | 0.063636 | 0.2545455 | 0.236364 | 0 |
| | 158.44 | 0.023333 | 0.053333 | 0.04 | 0 | 0 | 0.1 | 0 | 0.109091 | 0.172727 | 0.145455 | 0.046667 | 0.063636 | 0.109091 | 0.081818 | 0 | 0.063636 | 0.136364 |
| | 159.54 | 0.046667 | 0 | 0.06 | 0.026667 | 0.263636 | 0 | 0.054545 | 0 | 0.263636 | 0 | 0.066667 | 0.3 | 0.245455 | 0.181818 | 0.1181818 | 0.118182 | 0.127273 |
| | 160.64 | 0.07 | 0.066667 | 0.053333 | 0.02 | 0 | 0.218182 | 0.236364 | 0.290909 | 0 | 0.163636 | 0 | 0 | 0.236364 | 0.227273 | 0 | 0 | 0.172727 |
| | 161.74 | 0.036667 | 0.023333 | 0.053333 | 0.066667 | 0.181818 | 0.336364 | 0.063636 | 0.318182 | 0.227273 | 0.227273 | 0.116667 | 0.190909 | 0.172727 | 0 | 0.1545455 | 0.463636 | 0.263636 |
| | 162.84 | 0.113333 | 0.056667 | 0.1 | 0 | 0.336364 | 0 | 0.154545 | 0.009091 | 0 | 0.245455 | 0.096667 | 0.190909 | 0.136364 | 0.2 | 0.3909091 | 0 | 0.209091 |
| | 163.94 | 0.003333 | 0.046667 | 0.07 | 0.04 | 0.163636 | 0.372727 | 0.218182 | 0.354545 | 0.172727 | 0.090909 | 0.136667 | 0.118182 | 0.054545 | 0.327273 | 0.2636364 | 0.445455 | 0 |
| | 165.04 | 0.13 | 0.036667 | 0.066667 | 0 | 0.018182 | 0 | 0 | 0.009091 | 0 | 0.109091 | 0.063333 | 0 | 0.236364 | 0 | 0.0272727 | 0 | 0.190909 |
| | 166.14 | 0.02 | 0 | 0.05 | 0.08 | 0 | 0.127273 | 0.209091 | 0 | 0.027273 | 0.009091 | 0.003333 | 0.372727 | 0.227273 | 0.027273 | 0.2181818 | 0 | 0.281818 |
| | 167.24 | 0.06 | 0.05 | 0.056667 | 0.053333 | 0 | 0 | 0.036364 | 0 | 0.381818 | 0.118182 | 0.036667 | 0.009091 | 0.018182 | 0.018182 | 0.0272727 | 0.318182 | 0.009091 |
| | 168.34 | 0.056667 | 0.033333 | 0.003333 | 0.006667 | 0.163636 | 0.163636 | 0.154545 | 0.227273 | 0 | 0.109091 | 0.036667 | 0.090909 | 0.027273 | 0.4 | 0.2636364 | 0.009091 | 0.436364 |
| | 169.44 | 0 | 0.05 | 0.073333 | 0.033333 | 0 | 0.236364 | 0.390909 | 0 | 0 | 0.245455 | 0 | 0 | 0.136364 | 0.209091 | 0.3181818 | 0 | 0 |
| | 170.54 | 0.026667 | 0.023333 | 0.03 | 0.04 | 0.254545 | 0 | 0 | 0 | 0.009091 | 0.1 | 0.06 | 0.154545 | 0.127273 | 0.027273 | 0 | 0.245455 | 0 |
| | 171.64 | 0.033333 | 0.02 | 0.016667 | 0.063333 | 0.290909 | 0.145455 | 0.227273 | 0 | 0 | 0.118182 | 0.073333 | 0 | 0 | 0 | 0.0090909 | 0 | 0.145455 |
| | 172.74 | 0.033333 | 0.02 | 0 | 0 | 0 | 0 | 0 | 0 | 0 | 0.009091 | 0.09 | 0 | 0.063636 | 0 | 0 | 0.336364 | 0.018182 |
| | 173.84 | 0.04 | 0.063333 | 0.016667 | 0.023333 | 0.009091 | 0 | 0.290909 | 0.190909 | 0 | 0.218182 | 0.003333 | 0 | 0 | 0 | 0 | 0.027273 | 0.018182 |
| | 174.94 | 0.04 | 0.023333 | 0.043333 | 0.023333 | 0.190909 | 0.318182 | 0 | 0 | 0 | 0.136364 | 0 | 0 | 0.090909 | 0 | 0.1727273 | 0.118182 | 0.336364 |
| | 176.04 | 0.023333 | 0.01 | 0.02 | 0.04 | 0.009091 | 0 | 0.163636 | 0 | 0.318182 | 0 | 0.056667 | 0 | 0.072727 | 0.118182 | 0.0818182 | 0 | 0 |
| | 177.14 | 0 | 0.006667 | 0.033333 | 0 | 0.036364 | 0 | 0 | 0 | 0.163636 | 0.118182 | 0.066667 | 0.081818 | 0.027273 | 0.009091 | 0 | 0.209091 | 0 |
| | 178.24 | 0.003333 | 0.043333 | 0.056667 | 0.03 | 0.172727 | 0.218182 | 0.190909 | 0.354545 | 0 | 0.109091 | 0.05 | 0 | 0.181818 | 0 | 0.1818182 | 0.018182 | 0 |
| | 179.34 | 0.05 | 0.05 | 0.006667 | 0.01 | 0.072727 | 0 | 0 | 0.172727 | 0 | 0.081818 | 0.003333 | 0.145455 | 0.3 | 0 | 0.1454545 | 0.1 | 0.145455 |
| | 180.44 | 0.026667 | 0 | 0.07 | 0 | 0.027273 | 0.227273 | 0.163636 | 0.027273 | 0 | 0.063636 | 0.033333 | 0.118182 | 0.127273 | 0.127273 | 0.0090909 | 0.163636 | 0 |
| | 181.54 | 0.036667 | 0.016667 | 0.026667 | 0.026667 | 0.154545 | 0 | 0.118182 | 0.218182 | 0.290909 | 0.072727 | 0.036667 | 0.163636 | 0 | 0.136364 | 0.1272727 | 0 | 0 |
| | 182.64 | 0.033333 | 0 | 0.083333 | 0.03 | 0.163636 | 0.172727 | 0.2 | 0 | 0 | 0.063636 | 0 | 0.063636 | 0.009091 | 0 | 0 | 0 | 0.181818 |
| | 183.74 | 0.04 | 0.076667 | 0 | 0.08 | 0.009091 | 0.072727 | 0 | 0.154545 | 0.163636 | 0.136364 | 0.07 | 0.345455 | 0.245455 | 0.154545 | 0.2090909 | 0.109091 | 0.209091 |
| | 184.84 | 0.033333 | 0.03 | 0.093333 | 0 | 0.327273 | 0.245455 | 0.072727 | 0 | 0.009091 | 0.072727 | 0.076667 | 0.054545 | 0.272727 | 0.363636 | 0 | 0.2 | 0.254545 |
| | 185.94 | 0.04 | 0.103333 | 0.11 | 0.013333 | 0.009091 | 0.1 | 0.309091 | 0.2 | 0 | 0.118182 | 0.033333 | 0.054545 | 0.136364 | 0 | 0.2181818 | 0.045455 | 0 |
| | 187.04 | 0.09 | 0.016667 | 0.056667 | 0.083333 | 0.190909 | 0.154545 | 0 | 0.272727 | 0.481818 | 0.309091 | 0.123333 | 0.236364 | 0.127273 | 0.345455 | 0.3363636 | 0.081818 | 0.063636 |
| | 188.14 | 0.093333 | 0.066667 | 0.113333 | 0.053333 | 0.181818 | 0.272727 | 0.272727 | 0.272727 | 0 | 0.172727 | 0 | 0.290909 | 0.163636 | 0.009091 | 0 | 0.163636 | 0.390909 |
| | 189.24 | 0.036667 | 0.023333 | 0.066667 | 0.01 | 0.009091 | 0.209091 | 0 | 0.018182 | 0.009091 | 0.009091 | 0.163333 | 0.063636 | 0.018182 | 0.354545 | 0.1727273 | 0.181818 | 0 |
| | 190.34 | 0.03 | 0.063333 | 0.063333 | 0.073333 | 0.154545 | 0.090909 | 0.009091 | 0 | 0 | 0.018182 | 0 | 0.027273 | 0.154545 | 0 | 0.0090909 | 0 | 0 |
| | 191.44 | 0.08 | 0.073333 | 0.02 | 0.05 | 0.063636 | 0.045455 | 0.009091 | 0 | 0 | 0.081818 | 0.11 | 0.236364 | 0.154545 | 0.127273 | 0.2454545 | 0.109091 | 0.627273 |
| | 192.54 | 0.05 | 0.02 | 0.086667 | 0.07 | 0.027273 | 0 | 0.309091 | 0.390909 | 0.436364 | 0.327273 | 0.123333 | 0.154545 | 0 | 0.045455 | 0.1727273 | 0.163636 | 0.281818 |
| | 193.64 | 0.013333 | 0.036667 | 0.066667 | 0 | 0.2 | 0.1 | 0 | 0.318182 | 0 | 0.190909 | 0.08 | 0.209091 | 0.327273 | 0.318182 | 0 | 0 | 0 |
| | 194.74 | 0.04 | 0 | 0.01 | 0.046667 | 0 | 0.236364 | 0.090909 | 0 | 0.009091 | 0 | 0 | 0.036364 | 0.136364 | 0.281818 | 0.3181818 | 0.072727 | 0.290909 |
| | 195.84 | 0 | 0 | 0.03 | 0.003333 | 0.181818 | 0.045455 | 0.381818 | 0.009091 | 0 | 0.009091 | 0.006667 | 0 | 0 | 0.009091 | 0 | 0.009091 | 0 |
| | 196.94 | 0.013333 | 0.07 | 0.033333 | 0.026667 | 0.072727 | 0.054545 | 0 | 0 | 0 | 0 | 0 | 0.209091 | 0.245455 | 0 | 0 | 0.154545 | 0.009091 |
| | 198.04 | 0.016667 | 0 | 0.033333 | 0.08 | 0.063636 | 0 | 0.327273 | 0.145455 | 0.009091 | 0.072727 | 0.11 | 0.063636 | 0 | 0.327273 | 0.2727273 | 0.090909 | 0.309091 |
| | 199.14 | 0 | 0.003333 | 0.06 | 0 | 0 | 0.081818 | 0.009091 | 0.163636 | 0 | 0.018182 | 0.046667 | 0 | 0.172727 | 0 | 0.2545455 | 0.009091 | 0 |
| | 200.24 | 0.013333 | 0.016667 | 0.06 | 0.01 | 0 | 0.154545 | 0.018182 | 0 | 0 | 0.1 | 0.02 | 0.009091 | 0 | 0.009091 | 0.1363636 | 0.036364 | 0.1 |
| | 201.34 | 0 | 0.063333 | 0.003333 | 0.003333 | 0.054545 | 0.081818 | 0 | 0 | 0 | 0.036364 | 0 | 0.181818 | 0.036364 | 0 | 0 | 0 | 0.245455 |
| | 202.44 | 0.04 | 0.086667 | 0.066667 | 0.036667 | 0.109091 | 0 | 0.245455 | 0.190909 | 0.290909 | 0.263636 | 0.036667 | 0.145455 | 0.045455 | 0.427273 | 0 | 0.163636 | 0 |
| | 203.54 | 0.04 | 0.043333 | 0.036667 | 0.03 | 0.118182 | 0.163636 | 0 | 0.2 | 0.009091 | 0.063636 | 0.053333 | 0.009091 | 0.263636 | 0 | 0.3090909 | 0.045455 | 0.163636 |
| | 204.64 | 0.02 | 0.03 | 0 | 0 | 0 | 0.227273 | 0.172727 | 0 | 0.4 | 0.009091 | 0.003333 | 0.027273 | 0.172727 | 0 | 0 | 0.127273 | 0 |
| | 205.74 | 0.043333 | 0.02 | 0.05 | 0.043333 | 0.081818 | 0 | 0 | 0.109091 | 0 | 0.1 | 0.026667 | 0.1 | 0 | 0 | 0 | 0 | 0 |
| | 206.84 | 0.02 | 0.053333 | 0.06 | 0.086667 | 0.036364 | 0.018182 | 0 | 0.118182 | 0 | 0.009091 | 0.03 | 0.081818 | 0.072727 | 0.272727 | 0.0181818 | 0.190909 | 0 |
| | 207.94 | 0.05 | 0.013333 | 0 | 0 | 0.027273 | 0.136364 | | 0 | 0 | 0.181818 | 0 | 0.1 | 0.172727 | 0 | 0.1363636 | 0 | 0.254545 |
| | 209.04 | 0 | 0.08 | 0.02 | 0.02 | 0.090909 | 0.127273 | | 0.209091 | 0.3 | 0.027273 | 0.043333 | 0 | 0.209091 | 0 | 0.2454545 | 0 | 0 |
| | 210.14 | 0.026667 | 0.003333 | 0.08 | 0.023333 | 0.118182 | 0 | | 0.318182 | 0 | 0.281818 | 0.083333 | 0.118182 | 0.309091 | 0.090909 | 0 | 0.118182 | 0.418182 |
| | 211.24 | 0.07 | 0.073333 | 0.123333 | 0.1 | 0.045455 | 0.109091 | | 0.009091 | 0.2 | 0.081818 | 0.106667 | 0.245455 | 0.272727 | 0.254545 | 0.0181818 | 0.163636 | 0.290909 |
| | 212.34 | 0.07 | 0.09 | 0.023333 | 0.003333 | 0 | 0.181818 | | 0.009091 | 0.009091 | 0.063636 | 0.126667 | 0.236364 | 0.154545 | 0.1 | 0.2363636 | 0.172727 | 0.081818 |
| | 213.44 | 0.1 | 0 | 0.13 | 0.08 | 0.063636 | 0 | | 0.445455 | 0 | 0.009091 | 0.09 | 0.045455 | 0 | 0.018182 | 0.0818182 | 0.118182 | 0.345455 |
| | 214.54 | 0.033333 | 0.056667 | 0.04 | 0.06 | 0.236364 | 0.154545 | | 0 | 0 | 0.154545 | 0.023333 | 0.081818 | 0.090909 | 0.009091 | 0 | 0.190909 | 0.009091 |
| | 215.64 | 0.066667 | 0.1 | 0.04 | 0.03 | 0.2 | 0.081818 | | 0 | 0.454545 | 0.109091 | 0.023333 | 0.027273 | 0.127273 | 0.218182 | 0.1090909 | 0.054545 | 0.336364 |
| | 216.74 | 0.046667 | 0.006667 | 0.01 | 0.026667 | 0.163636 | 0 | | 0.427273 | 0 | 0.027273 | 0.096667 | 0.190909 | 0.163636 | 0.181818 | 0.1181818 | 0.181818 | 0.009091 |
| | 217.84 | 0.053333 | 0.066667 | 0.083333 | 0.096667 | 0 | 0.154545 | | 0 | 0 | 0.218182 | 0.073333 | 0 | 0.009091 | 0.009091 | 0 | 0.063636 | 0.127273 |
| | 218.94 | 0.04 | 0.026667 | 0.076667 | 0.003333 | 0 | 0.009091 | | 0.381818 | 0 | 0.109091 | 0 | 0 | 0.154545 | 0 | 0.1272727 | 0.163636 | 0.172727 |
| | 220.04 | 0.033333 | 0 | 0.05 | 0.04 | 0.163636 | 0.163636 | | 0 | 0 | 0.027273 | 0.096667 | 0.009091 | 0 | 0.109091 | 0 | 0.181818 | 0 |
| | 221.14 | 0.046667 | 0.076667 | 0.06 | 0.033333 | 0 | 0 | | 0.327273 | 0 | 0.118182 | 0.046667 | 0 | 0.181818 | 0 | 0.1 | 0.154545 | 0.181818 |
| | 222.24 | 0.013333 | 0 | 0.026667 | 0.03 | 0.009091 | 0 | | 0.009091 | 0 | 0.072727 | 0.013333 | 0.018182 | 0.009091 | 0.009091 | 0.1 | 0.027273 | 0.009091 |
| | 223.34 | 0.026667 | 0 | 0.056667 | 0 | 0 | 0.127273 | | 0.009091 | 0.345455 | 0.045455 | 0.056667 | 0 | 0.136364 | 0 | 0 | 0.072727 | 0 |
| | 224.44 | 0.02 | 0 | 0.03 | 0.03 | 0.072727 | 0.118182 | | 0.018182 | 0.027273 | 0.054545 | 0.003333 | 0 | 0.109091 | 0.072727 | 0.0181818 | 0.118182 | 0.245455 |
| | 225.54 | 0.02 | 0.026667 | 0.043333 | 0.043333 | 0.009091 | 0.018182 | | 0.345455 | 0 | 0.263636 | 0.016667 | 0.054545 | 0.081818 | 0 | 0.1454545 | 0.236364 | 0.145455 |
| | 226.64 | 0.016667 | 0.036667 | 0.02 | 0.01 | 0.045455 | 0.1 | | 0.290909 | 0 | 0.027273 | 0.046667 | 0 | 0.145455 | 0 | 0.1545455 | 0.045455 | 0.1 |
| | 227.74 | 0.023333 | 0.036667 | 0.033333 | 0.04 | 0.136364 | 0.118182 | | 0 | 0.5 | 0.109091 | 0.03 | 0 | 0.127273 | 0 | 0 | 0.018182 | 0.136364 |
| | 228.84 | 0.016667 | 0 | 0.023333 | 0 | 0.090909 | 0 | | 0 | 0 | 0.045455 | 0 | 0 | 0.027273 | 0 | 0.0090909 | 0 | 0.009091 |
| | 229.94 | 0.043333 | 0 | 0.036667 | 0.01 | 0 | 0.081818 | | 0 | 0 | 0.145455 | 0.003333 | 0 | 0.190909 | 0 | 0 | 0.2 | 0 |
| | | | | | | | | | 0.009091 | 0 | | | | | 0 | 0.1636364 | | |

| 10 | 10 | 12 | 12 | 12 | 12 | 3 | 3 | 4 | 4 | 5 | 7 | 7 | 8 | 10 | 10 | 4 | 4 | 5 | 8 |
|---|---|---|---|---|---|---|---|---|---|---|---|---|---|---|---|---|---|---|---|
| Box-5 | Box-4 | Box-1 | Box-2 | Box-5 | Box-6 | Box-1 | Box-2 | Box-6 | Box-7 | Box-6 | Box-2 | Box-5 | Box-3 | Box-1 | Box-3 | Box-1 | Box-4 | Box-5 | Box-1 |
| 63 | 69 | 74 | 76 | 81 | 83 | 1.7 | 2.3 | 25.3 | 26.6 | 34.6 | 38 | 43.6 | 52.6 | 64 | 75 | 13.3 | 21.5 | 32.6 | 44.6 |
| F | F | F | F | F | F | F | F | F | F | F | F | F | F | F | F | F | F | F | F |
| H2O | H2O | H2O | H2O | H2O | H2O | Malto | Malto | Malto | Malto | Malto | Malto | Malto | Malto | Malto | Malto | MCT | MCT | MCT | MCT |
| FH | FH | FH | FH | FH | FH | FM | FM | FM | FM | FM | FM | FM | FM | FM | FM | FT | FT | FT | FT |
| 0 | 0 | 0 | 0 | 0 | 0.009091 | 0.063636 | 0 | 0 | 0.009091 | 0 | 0 | 0 | 0 | 0 | 0 | 0 | 0 | 0.072727 | 0.009091 |
| 0 | 0 | 0 | 0 | 0 | 0 | 0.063636 | 0.118182 | 0 | 0.027273 | 0.090909 | 0 | 0 | 0 | 0 | 0 | 0 | 0 | 0.2 | 0.009091 |
| 0 | 0 | 0 | 0 | 0 | 0.018182 | 0.290909 | 0.645455 | 0 | 0 | 0.4 | 0.018182 | 0 | 0 | 0 | 0 | 0 | 0 | 0.181818 | 0 |
| 0.154545 | 0 | 0 | 0 | 0 | 0 | 0.054545 | 0.536364 | 0 | 0.109091 | 0.190909 | 0.254545 | 0 | 0 | 0 | 0 | 0 | 0 | 0.281818 | 0 |
| 0.363636 | 0 | 0 | 0.163636 | 0.181818 | 0 | 0.209091 | 0.127273 | 0.009091 | 0.154545 | 0.936364 | 0.381818 | 0 | 0 | 0 | 0 | 0 | 0.045455 | 0.336364 | 0 |
| 0.3 | 0 | 0.009091 | 0.236364 | 0.490909 | 0 | 0 | 0.154545 | 0 | 0.3 | 0.018182 | 0.4 | 0 | 0 | 0.063636 | 0 | 0 | 0.245455 | 0.454545 | 0 |
| 0.554545 | 0 | 0.336364 | 0.318182 | 0.572727 | 0.372727 | 0.309091 | 0.727273 | 0.309091 | 0.381818 | 0.218182 | 0.090909 | 0 | 0 | 0.736364 | 0 | 0.309091 | 0.4 | 0.218182 | 0 |
| 0.518182 | 0 | 0.309091 | 0.218182 | 0.581818 | 0.5 | 0.336364 | 0.127273 | 0.181818 | 0.218182 | 0.472727 | 0.136364 | 0 | 0.218182 | 0.1 | 0.390909 | 0.536364 | 0.327273 | 0.063636 | 0 |
| 0.009091 | 0 | 0.309091 | 0.009091 | 0.427273 | 0.445455 | 0.418182 | 0.236364 | 0.245455 | 0.027273 | 0.118182 | 0.218182 | 0 | 0 | 0.718182 | 0.6 | 0 | 0.463636 | 0.245455 | 0 |
| 0 | 0.118182 | 0.145455 | 0 | 0 | 0 | 0 | 0.236364 | 0 | 0.363636 | 0.018182 | 0.263636 | 0 | 0.327273 | 0 | 0 | 0.118182 | 0.190909 | 0.363636 | 0 |
| 0.227273 | 0.227273 | 0 | 0.1 | 0.390909 | 0 | 0.009091 | 0.127273 | 0.263636 | 0.045455 | 0.190909 | 0 | 0 | 0.345455 | 0.345455 | 0 | 0.281818 | 0.063636 | 0.236364 | 0 |
| 0.245455 | 0.036364 | 0.381818 | 0.027273 | 0.409091 | 0.745455 | 0.009091 | 0.409091 | 0.2 | 0.145455 | 0.172727 | 0 | 0 | 0.009091 | 0 | 0.354545 | 0.154545 | 0.1 | 0 | 0 |
| 0.290909 | 0 | 0.009091 | 0 | 0.127273 | 0.072727 | 0 | 0 | 0.236364 | 0.118182 | 0.309091 | 0.336364 | 0 | 0 | 0.236364 | 0 | 0.509091 | 0.354545 | 0.072727 | 0 |
| 0.1 | 0.009091 | 0 | 0 | 0.281818 | 0.427273 | 0 | 0 | 0.263636 | 0 | 0.236364 | 0.154545 | 0 | 0 | 0 | 0.190909 | 0 | 0.227273 | 0.281818 | 0 |
| 0.209091 | 0.072727 | 0.209091 | 0.290909 | 0 | 0.054545 | 0 | 0.018182 | 0 | 0.509091 | 0 | 0.081818 | 0 | 0 | 0 | 0 | 0.272727 | 0 | 0 | 0 |
| 0 | 0.172727 | 0 | 0.072727 | 0 | 0.254545 | 0 | 0 | 0.572727 | 0 | 0.272727 | 0.172727 | 0 | 0 | 0 | 0.354545 | 0 | 0.309091 | 0.163636 | 0 |
| 0 | 0 | 0.090909 | 0.127273 | 0.090909 | 0 | 0 | 0.118182 | 0.009091 | 0.145455 | 0 | 0.136364 | 0 | 0 | 0 | 0 | 0 | 0.054545 | 0.072727 | 0 |
| 0 | 0 | 0 | 0.318182 | 0.009091 | 0 | 0 | 0 | 0.245455 | 0.027273 | 0.163636 | 0.281818 | 0 | 0 | 0 | 0 | 0 | 0.327273 | 0.181818 | 0 |
| 0 | 0 | 0.009091 | 0.118182 | 0.145455 | 0.254545 | 0 | 0 | 0 | 0.127273 | 0 | 0 | 0 | 0 | 0 | 0.2 | 0 | 0.063636 | 0.409091 | 0 |
| 0 | 0.1 | 0 | 0.036364 | 0.090909 | 0 | 0 | 0 | 0.345455 | 0.272727 | 0.163636 | 0.2 | 0 | 0 | 0 | 0.154545 | 0.063636 | 0.1 | 0 | 0 |
| 0.009091 | 0.109091 | 0.181818 | 0.136364 | 0.272727 | 0.172727 | 0.645455 | 0.190909 | 0 | 0.063636 | 0.027273 | 0 | 0 | 0.154545 | 0 | 0.2 | 0.136364 | 0.009091 | 0 | 0 |
| 0 | 0 | 0 | 0 | 0 | 0 | 0 | 0 | 0.218182 | 0.109091 | 0.181818 | 0.218182 | 0 | 0.2 | 0 | 0.181818 | 0.136364 | 0.190909 | 0.254545 | 0.490909 |
| 0.172727 | 0.009091 | 0.218182 | 0.172727 | 0.045455 | 0 | 0.072727 | 0.372727 | 0 | 0.172727 | 0.063636 | 0.281818 | 0 | 0.009091 | 0 | 0 | 0.263636 | 0 | 0.027273 | 0.118182 |
| 0.218182 | 0.154545 | 0 | 0.045455 | 0.163636 | 0.663636 | 0.318182 | 0 | 0.3 | 0.154545 | 0.3 | 0 | 0 | 0.436364 | 0 | 0.272727 | 0.190909 | 0.327273 | 0.2 | 0.563636 |
| 0.172727 | 0.009091 | 0.190909 | 0.272727 | 0.154545 | 0 | 0.245455 | 0.490909 | 0.563636 | 0.218182 | 0.227273 | 0.318182 | 0.3 | 0.445455 | 0.663636 | 0.227273 | 0.209091 | 0.263636 | 0.3 | 0.354545 |
| 0.381818 | 0.154545 | 0.172727 | 0.309091 | 0.327273 | 0.2 | 0.318182 | 0.290909 | 0.309091 | 0 | 0.281818 | 0.3 | 0.072727 | 0.636364 | 0.090909 | 0.290909 | 0.209091 | 0.190909 | 0.190909 | 0.390909 |
| 0.290909 | 0.236364 | 0.181818 | 0.554545 | 0.290909 | 0.7 | 0.227273 | 0.090909 | 0 | 0 | 0.272727 | 0.2 | 0.318182 | 0.336364 | 0.218182 | 0.3 | 0.4 | 0.445455 | 0.354545 | 0.218182 |
| 0.181818 | 0.3 | 0.409091 | 0.2 | 0.390909 | 0.227273 | 0.363636 | 0.418182 | 0.372727 | 0.209091 | 0.245455 | 0.427273 | 0.136364 | 0.245455 | 0.318182 | 0.3 | 0.290909 | 0.027273 | 0.290909 | 0.418182 |
| 0.463636 | 0.345455 | 0.281818 | 0.481818 | 0.7 | 0.272727 | 0.145455 | 0.190909 | 0 | 0.054545 | 0.2 | 0.154545 | 0.1 | 0 | 0.563636 | 0 | 0.1 | 0.018182 | 0.227273 | 0.463636 |
| 0.227273 | 0.345455 | 0.354545 | 0.2 | 0.627273 | 0.5 | 0.154545 | 0.454545 | 0.136364 | 0.009091 | 0.418182 | 0.009091 | 0.027273 | 0.027273 | 0.6 | 0.272727 | 0.1 | 0 | 0.154545 | 0 |
| 0.236364 | 0.372727 | 0.109091 | 0.245455 | 0.354545 | 0.318182 | 0.4 | 0 | 0 | 0 | 0.263636 | 0.263636 | 0.181818 | 0.345455 | 0.381818 | 0.354545 | 0.227273 | 0.018182 | 0.2 | 0.090909 |
| 0.045455 | 0 | 0.3 | 0.209091 | 0.127273 | 0.281818 | 0.345455 | 0.254545 | 0 | 0.309091 | 0.072727 | 0.281818 | 0.127273 | 0 | 0.009091 | 0 | 0.045455 | 0.263636 | 0.381818 | 0.054545 |
| 0.172727 | 0 | 0 | 0.336364 | 0.363636 | 0 | 0 | 0.3 | 0.136364 | 0.009091 | 0.136364 | 0 | 0 | 0.327273 | 0 | 0 | 0.336364 | 0.154545 | 0.018182 | 0.190909 |
| 0.236364 | 0 | 0.154545 | 0 | 0.254545 | 0 | 0 | 0.027273 | 0.336364 | 0.245455 | 0.2 | 0.336364 | 0 | 0.2 | 0.3 | 0.309091 | 0.190909 | 0 | 0.227273 | 0.463636 |
| 0.218182 | 0.545455 | 0.109091 | 0.145455 | 0.327273 | 0.336364 | 0 | 0.109091 | 0.309091 | 0.5 | 0.009091 | 0 | 0.018182 | 0 | 0.227273 | 0 | 0.581818 | 0.272727 | 0.218182 | 0.009091 |
| 0.054545 | 0.036364 | 0 | 0 | 0.072727 | 0 | 0.045455 | 0 | 0.009091 | 0.236364 | 0.227273 | 0.281818 | 0.236364 | 0 | 0.336364 | 0.227273 | 0.336364 | 0.218182 | 0 | 0 |
| 0.018182 | 0 | 0.218182 | 0 | 0 | 0.263636 | 0 | 0.036364 | 0.372727 | 0.218182 | 0.063636 | 0 | 0 | 0.036364 | 0 | 0.163636 | 0.336364 | 0.136364 | 0.072727 | 0.209091 |
| 0 | 0 | 0 | 0.036364 | 0.3 | 0 | 0.109091 | 0.154545 | 0.009091 | 0.127273 | 0.018182 | 0.536364 | 0.027273 | 0 | 0.118182 | 0.109091 | 0.136364 | 0.154545 | 0 | 0.218182 |
| 0.018182 | 0 | 0.245455 | 0 | 0 | 0 | 0.190909 | 0 | 0.190909 | 0.018182 | 0.054545 | 0.036364 | 0.127273 | 0.127273 | 0 | 0.018182 | 0.209091 | 0.245455 | 0.072727 | 0 |
| 0 | 0.545455 | 0 | 0.009091 | 0 | 0 | 0 | 0.118182 | 0.436364 | 0 | | 0 | 0 | 0 | 0.009091 | 0.309091 | 0.072727 | 0.090909 | | 0.254545 |
| 0.009091 | 0 | 0.036364 | 0.381818 | 0 | 0 | 0 | 0.2 | 0 | 0.3 | | 0 | 0 | 0.063636 | 0 | 0.109091 | 0.263636 | 0.018182 | | 0.027273 |
| 0 | 0.118182 | 0.281818 | 0 | 0.145455 | 0.090909 | 0.309091 | 0 | 0.1 | 0.072727 | | 0.127273 | 0.036364 | 0 | 0 | 0 | 0.281818 | 0.281818 | | 0.254545 |
| 0.209091 | 0.009091 | 0 | 0.363636 | 0.281818 | 0 | 0.245455 | 0.309091 | 0.127273 | 0.009091 | | 0 | 0.009091 | 0.118182 | 0.018182 | 0.281818 | 0 | 0 | | 0.1 |
| 0.281818 | 0.327273 | 0 | 0.236364 | 0 | 0.363636 | 0.263636 | 0.272727 | 0.154545 | 0.345455 | | 0.236364 | 0.127273 | 0.118182 | 0 | 0 | 0.272727 | 0.336364 | | 0.2 |
| 0 | 0.236364 | 0.136364 | 0.027273 | 0.336364 | 0.109091 | 0 | 0.018182 | 0 | 0.345455 | | 0.181818 | 0.027273 | 0.127273 | 0 | 0.3 | 0.009091 | 0.1 | | 0.509091 |
| 0.354545 | 0 | 0.027273 | 0.2 | 0.3 | 0.154545 | 0.690909 | 0.309091 | 0.245455 | 0.081818 | | 0.154545 | 0.036364 | 0.354545 | 0.454545 | 0 | 0.254545 | 0.3 | | 0.427273 |
| 0.236364 | 0.336364 | 0.336364 | 0.318182 | 0.272727 | 0.954545 | 0 | 0.190909 | 0 | 0.118182 | | 0.209091 | 0.118182 | 0.390909 | 0.290909 | 0.363636 | 0.136364 | 0.045455 | | 0.436364 |
| 0.445455 | 0.318182 | 0.209091 | 0.463636 | 0.418182 | 0.409091 | 0.581818 | 0.218182 | 0.254545 | 0.263636 | | 0.363636 | 0.127273 | 0.390909 | 0.045455 | 0.281818 | 0.245455 | 0.345455 | | 0.2 |
| 0.236364 | 0.363636 | 0.227273 | 0.363636 | 0 | 0.009091 | 0 | 0.354545 | 0 | 0.218182 | | 0.136364 | 0.081818 | 0.390909 | 0.227273 | 0.354545 | 0.236364 | 0.018182 | | 0.145455 |
| 0.309091 | 0.263636 | 0.1 | 0.218182 | 0.154545 | 0.118182 | 0.163636 | 0.072727 | | | | 0.018182 | 0.154545 | 0.272727 | 0.3 | 0.318182 | | | | 0.136364 |
| 0.381818 | 0.145455 | 0.245455 | 0.181818 | 0.781818 | 0.881818 | 0.427273 | 0.2 | | | | 0.290909 | 0 | 0.181818 | 0.427273 | 0 | | | | 0.390909 |
| 0.009091 | 0.018182 | 0.390909 | 0.090909 | 0.363636 | 0.436364 | 0.172727 | 0.109091 | | | | 0 | 0.018182 | 0.2 | 0.481818 | 0.372727 | | | | 0 |
| 0.263636 | 0.363636 | 0 | 0.209091 | 0.063636 | 0 | 0.090909 | 0.2 | | | | 0 | 0.136364 | 0 | 0.5 | 0 | | | | 0.490909 |
| 0 | 0.418182 | 0 | 0 | 0.3 | 0.027273 | 0.190909 | 0.145455 | | | | 0.272727 | 0.072727 | 0.309091 | 0.009091 | 0.281818 | | | | 0 |
| 0.181818 | 0.127273 | 0 | 0.209091 | 0 | 0.4 | 0 | 0.218182 | | | | 0 | 0 | 0.409091 | 0 | 0.109091 | | | | 0.172727 |
| 0.227273 | 0 | 0 | 0.081818 | 0.018182 | 0.545455 | 0 | 0 | | | | 0 | 0.154545 | 0.172727 | 0.290909 | 0.354545 | | | | 0.163636 |
| 0 | 0.009091 | 0.281818 | 0.309091 | 0.345455 | 0 | 0.181818 | 0.154545 | | | | 0.345455 | 0 | 0 | 0 | 0.018182 | | | | 0.081818 |
| 0.254545 | 0.354545 | 0 | 0 | 0.290909 | 0 | 0 | 0.118182 | | | | 0 | 0.063636 | 0 | 0.409091 | 0 | | | | 0.263636 |
| 0 | 0.290909 | 0.227273 | 0.181818 | 0 | 0 | 0 | 0.009091 | | | | 0.309091 | 0.054545 | 0 | 0.109091 | 0 | | | | 0.009091 |
| 0 | 0 | 0.172727 | 0.145455 | 0 | 0.236364 | 0 | 0.218182 | | | | 0 | 0.090909 | 0 | 0.090909 | 0.218182 | | | | 0.181818 |
| 0.418182 | 0.018182 | 0.009091 | 0.290909 | 0.445455 | 0 | 0.181818 | 0 | | | | 0 | 0 | 0 | 0.045455 | 0 | | | | 0.063636 |
| 0 | 0.436364 | | | | | | | | | | | | | 0.2 | 0.209091 | | | | |
| 0 | 0.318182 | 0.066667 | 0.086667 | 0.213333 | 0.136667 | 0.372727 | 0.290909 | 0.181818 | 0.3 | 0.163636 | 0.436364 | 0.009091 | 0.190909 | 0.381818 | 0.472727 | 0 | 0.572727 | 0 | 0.281818 |
| 0.154545 | 0 | 0.006667 | 0.123333 | 0 | 0 | 0.318182 | 0.163636 | 0 | 0.354545 | 0 | 0 | 0.145455 | 0.381818 | 0.336364 | 0.354545 | 0.327273 | 0 | 0.381818 | 0.009091 |
| 0.309091 | 0.036364 | 0.08 | 0 | 0.143333 | 0.146667 | 0.290909 | 0.290909 | 0.445455 | 0 | 0.427273 | 0.172727 | 0.081818 | 0.209091 | 0.318182 | 0 | 0.418182 | 0 | 0 | 0.427273 |
| 0.545455 | 0.545455 | 0.006667 | 0.04 | 0 | 0.13 | 0.172727 | 0.009091 | 0.363636 | 0.145455 | 0.118182 | 0.318182 | 0.072727 | 0.190909 | 0.318182 | 0 | 0 | 0.063636 | 0.263636 | 0.163636 |
| 0.263636 | 0 | 0.083333 | 0.166667 | 0.016667 | 0.25 | 0.154545 | 0.309091 | 0.145455 | 0 | 0.218182 | 0.190909 | 0.018182 | 0 | 0.145455 | 0.009091 | 0.136364 | 0.109091 | 0.063636 | 0.090909 |
| 0.154545 | 0 | 0.11 | 0.04 | 0.093333 | 0.086667 | 0.309091 | 0.172727 | 0.227273 | 0.309091 | 0.236364 | 0 | 0.409091 | 0.327273 | 0.163636 | 0.272727 | 0.409091 | 0.118182 | 0.209091 | 0.154545 |
| 0.054545 | 0.054545 | 0 | 0.073333 | 0.16 | 0.093333 | 0.018182 | 0 | 0.090909 | 0 | 0.118182 | 0.218182 | 0.427273 | 0.1 | 0.154545 | 0 | 0 | 0.381818 | 0.145455 | 0 |
| 0 | 0 | 0.053333 | 0.08 | 0.16 | 0.07 | 0.1 | 0.418182 | 0.054545 | 0.309091 | 0.245455 | 0.409091 | 0.381818 | 0.281818 | 0.081818 | 0.290909 | 0.218182 | 0 | 0.163636 | 0.309091 |
| 0.181818 | 0.445455 | 0.13 | 0.073333 | 0 | 0.12 | 0.145455 | 0.009091 | 0.318182 | 0 | 0.363636 | 0 | 0 | 0.018182 | 0.036364 | 0.4 | 0.054545 | 0 | 0.209091 | 0 |
| 0 | 0 | 0 | 0 | 0.146667 | 0.143333 | 0 | 0.190909 | 0.181818 | 0.245455 | 0.063636 | 0.390909 | 0 | 0.472727 | 0.236364 | 0 | 0.136364 | 0.245455 | 0 | 0.154545 |
| 0 | 0 | 0 | 0.003333 | 0 | 0 | 0 | 0.354545 | 0 | 0.172727 | 0.027273 | 0 | 0.027273 | 0 | 0.272727 | 0 | 0.027273 | 0.027273 | 0.218182 | 0.190909 |
| 0.1 | 0.427273 | 0.083333 | 0 | 0.023333 | 0.103333 | 0.027273 | 0 | 0.072727 | 0.072727 | 0.218182 | 0.409091 | 0.009091 | 0.354545 | 0.163636 | 0.3 | 0.463636 | 0.4 | 0.209091 | 0.081818 |
| 0.236364 | 0 | 0 | 0.026667 | 0.083333 | 0 | 0.109091 | 0.009091 | 0.090909 | 0.309091 | 0.118182 | 0 | 0.263636 | 0 | 0.009091 | 0 | 0.018182 | 0.018182 | 0.281818 | 0 |
| 0.063636 | 0.136364 | 0.12 | 0.046667 | 0.163333 | 0.033333 | 0.072727 | 0.009091 | 0.118182 | 0 | 0.209091 | 0.018182 | 0 | 0.081818 | 0.027273 | 0.481818 | 0.127273 | 0.327273 | 0.572727 | 0.181818 |
| 0.172727 | 0.3 | 0 | 0.073333 | 0 | 0 | 0.118182 | 0.2 | 0.027273 | 0.054545 | 0.181818 | 0 | 0.136364 | 0.018182 | 0.190909 | 0 | 0.309091 | 0.036364 | 0 | 0.045455 |
| 0.263636 | 0 | 0.05 | 0.01 | 0.026667 | 0.06 | 0.145455 | 0.036364 | 0 | 0.163636 | 0 | 0 | 0.018182 | 0.063636 | 0.127273 | 0.318182 | 0.227273 | 0.418182 | 0 | 0 |
| 0 | 0 | 0.116667 | 0.026667 | 0.096667 | 0.04 | 0.218182 | 0.054545 | 0 | 0.1 | 0.072727 | 0 | 0.254545 | 0 | 0.036364 | 0 | 0.436364 | 0 | 0 | 0.036364 |
| 0.172727 | 0.245455 | 0 | 0.12 | 0.003333 | 0 | 0.381818 | 0.218182 | 0.227273 | 0.236364 | 0.036364 | 0.2 | 0.018182 | 0 | 0.081818 | 0.418182 | 0 | 0.036364 | 0 | 0.154545 |
| 0.372727 | 0 | 0.1 | 0 | 0.06 | 0.026667 | 0.072727 | 0.2 | 0.245455 | 0.272727 | 0.145455 | 0 | 0.009091 | 0.081818 | 0.227273 | 0 | 0.018182 | 0 | 0.163636 | 0 |
| 0.209091 | 0.363636 | 0.113333 | 0.13 | 0.136667 | 0.006667 | 0.218182 | 0 | 0 | 0 | 0.054545 | 0.372727 | 0.218182 | 0.2 | 0.327273 | 0 | 0.027273 | 0.436364 | 0 | 0.227273 |
| 0.218182 | 0.172727 | 0.026667 | 0.1 | 0 | 0.05 | 0.390909 | 0.290909 | 0.227273 | 0.381818 | 0 | 0 | 0.272727 | 0 | 0.609091 | 0.318182 | 0.081818 | 0 | 0.009091 | 0.245455 |
| 0.1 | 0.136364 | 0 | 0.056667 | 0.18 | 0.063333 | 0.018182 | 0.372727 | 0.354545 | 0.163636 | 0.072727 | 0.536364 | 0.136364 | 0.354545 | 0 | 0.018182 | 0.272727 | 0.309091 | 0.072727 | 0.190909 |
| 0.190909 | 0.127273 | 0.17 | 0.176667 | 0.166667 | 0.05 | 0.272727 | 0.463636 | 0 | 0 | 0.109091 | 0 | 0.345455 | 0.190909 | 0.218182 | 0 | 0.2 | 0.172727 | 0.381818 | 0.145455 |
| 0 | 0 | 0.046667 | 0 | 0.006667 | 0.166667 | 0.363636 | 0.018182 | 0 | 0 | 0.354545 | 0.3 | 0.763636 | 0.163636 | 0.109091 | 0.345455 | 0.181818 | 0.018182 | 0.336364 | 0 |
| 0.409091 | 0.809091 | 0.046667 | 0.046667 | 0.003333 | 0.15 | 0.318182 | 0 | 0.327273 | 0.336364 | 0.209091 | 0.409091 | 0 | 0.1 | 0.509091 | 0.354545 | 0.4 | 0.254545 | 0 | 0.445455 |
| 0.263636 | 0 | 0.093333 | 0.11 | 0.01 | 0.083333 | 0.4 | 0.354545 | 0 | 0.327273 | 0 | 0.118182 | 0 | 0.163636 | 0.254545 | 0 | 0.272727 | 0.290909 | 0.136364 | 0.009091 |
| 0.018182 | 0 | 0.073333 | 0 | 0 | 0.11 | 0.027273 | 0.527273 | 0.554545 | 0.490909 | 0.190909 | 0 | 0.118182 | 0.245455 | 0.236364 | 0.272727 | 0 | 0.063636 | 0.445455 | 0.036364 |
| 0.172727 | 0.009091 | 0.123333 | 0.103333 | 0.176667 | 0.133333 | 0.018182 | 0.009091 | 0 | 0 | 0.381818 | 0.045455 | 0.009091 | 0.036364 | 0.254545 | 0.254545 | 0.145455 | 0.190909 | 0 | 0.063636 |
| 0.118182 | 0.263636 | 0 | 0.113333 | 0.173333 | 0.103333 | 0 | 0 | . | 0.454545 | 0.281818 | 0 | 0.145455 | 0.281818 | 0 | 0 | 0.036364 | 0.245455 | 0.036364 | 0.345455 |
| 0.145455 | 0.009091 | 0.096667 | 0.033333 | 0 | 0.076667 | 0.372727 | 0 | . | 0 | 0.027273 | 0.227273 | 0.354545 | 0 | 0.109091 | 0.2 | 0.036364 | 0.063636 | 0.463636 | 0.145455 |
| 0.009091 | 0 | 0 | 0 | 0 | 0 | 0 | 0.2 | . | 0 | 0 | 0.118182 | 0.009091 | 0 | 0.036364 | 0.172727 | 0 | 0 | 0.463636 | 0.227273 |
| 0.081818 | 0.490909 | 0.043333 | 0 | 0 | 0.06 | 0.136364 | 0 | . | 0 | 0.481818 | 0 | 0.036364 | 0.072727 | 0 | 0 | 0 | 0 | 0 | 0.109091 |
| 0.018182 | 0 | 0.076667 | 0.023333 | 0 | 0.006667 | 0 | 0 | . | 0.218182 | 0 | 0 | 0.118182 | 0.318182 | 0 | 0.118182 | 0.245455 | 0 | 0.345455 | 0 |
| 0.009091 | 0.045455 | 0 | 0 | 0 | 0.01 | 0.218182 | 0.472727 | . | 0 | 0.1 | 0 | 0.209091 | 0.009091 | 0.090909 | 0 | 0.145455 | 0.118182 | 0.081818 | 0.027273 |
| 0.136364 | 0.436364 | 0 | 0 | 0.07 | 0.04 | 0 | 0 | . | 0.454545 | 0.009091 | 0.309091 | 0.281818 | 0.309091 | 0 | 0 | 0.254545 | 0 | 0 | 0.154545 |
| 0.090909 | 0 | 0.066667 | 0.033333 | 0.1 | 0.053333 | 0.463636 | 0 | . | 0 | 0 | 0 | 0.009091 | 0.018182 | 0.181818 | 0.118182 | 0.354545 | 0.209091 | 0.127273 | 0.181818 |
| 0.172727 | 0.172727 | 0.123333 | 0.04 | 0.003333 | 0.086667 | 0 | 0.345455 | . | 0 | 0 | 0.227273 | 0 | 0.309091 | 0.118182 | 0.290909 | 0 | 0.181818 | 0.009091 | 0.009091 |
| 0.081818 | 0 | 0.003333 | 0.106667 | 0.08 | 0.05 | 0 | 0.136364 | . | 0.063636 | 0.045455 | 0.281818 | 0.3 | 0.109091 | 0 | 0.154545 | 0.145455 | 0.372727 | 0.109091 | 0.318182 |
| 0.336364 | 0.009091 | 0.196667 | 0.106667 | 0.006667 | 0.083333 | 0 | 0.218182 | . | 0.009091 | 0 | 0 | 0.136364 | 0.090909 | 0.318182 | 0.145455 | 0.018182 | 0.063636 | 0.090909 | 0 |

| | | | | | | | | | | | | | | | | | | | |
|---|---|---|---|---|---|---|---|---|---|---|---|---|---|---|---|---|---|---|---|
| 0 | 0.618182 | 0.076667 | 0.08 | 0 | 0.156667 | 0.136364 | 0 | . | 0.227273 | 0 | 0 | 0.009091 | 0 | 0.236364 | 0 | 0 | 0 | 0.127273 | 0.390909 |
| 0.281818 | 0 | 0 | 0.116667 | 0 | 0.013333 | 0.581818 | 0.554545 | . | 0.354545 | | 0.236364 | 0.018182 | 0.018182 | 0 | 0.181818 | 0 | 0.309091 | | 0 |
| 0 | 0.009091 | 0.2 | 0.126667 | 0.016667 | 0.063333 | 0.345455 | 0.018182 | . | 0 | | 0 | 0.209091 | 0 | 0.372727 | 0.354545 | 0.072727 | 0.027273 | | 0 |
| 0.354545 | 0.372727 | 0.086667 | 0.183333 | 0.2 | 0.116667 | 0 | 0.418182 | . | 0.381818 | | 0.109091 | 0.645455 | 0.154545 | 0.463636 | 0.018182 | 0.172727 | 0.036364 | | 0.072727 |
| 0.009091 | 0 | 0 | 0.106667 | 0 | 0.116667 | 0.227273 | 0 | . | 0 | | 0.709091 | 0 | 0.281818 | 0 | 0.263636 | 0.236364 | 0.036364 | | 0.227273 |
| 0.363636 | 0 | 0.086667 | 0 | 0 | 0 | 0.045455 | 0 | . | 0.2 | | 0 | 0.309091 | 0.018182 | 0.490909 | 0 | 0.263636 | 0.518182 | | 0.081818 |
| 0.027273 | 0 | 0.013333 | 0.106667 | 0.086667 | 0.01 | 0.090909 | 0 | . | 0.354545 | | 0.009091 | 0.327273 | 0.436364 | 0.272727 | 0.436364 | 0.145455 | 0.018182 | | 0.227273 |
| 0.381818 | 0.045455 | 0.1 | 0.03 | 0.013333 | 0.14 | 0.454545 | 0.172727 | . | 0.4 | | 0 | 0.118182 | 0.009091 | 0.345455 | 0.372727 | 0.254545 | 0 | | 0.018182 |
| 0 | 0.354545 | 0.09 | 0.176667 | 0.003333 | 0.146667 | 0.4 | 0.2 | . | 0 | | 0.463636 | 0.227273 | 0.4 | 0.472727 | 0.045455 | 0.263636 | 0.045455 | | 0.109091 |
| 0.418182 | 0 | 0.06 | 0 | 0.133333 | 0.15 | 0.009091 | 0 | . | 0.027273 | | 0 | 0.372727 | 0 | 0.3 | 0.354545 | 0 | 0.463636 | | 0.1 |
| 0.145455 | 0 | 0.04 | 0 | 0 | 0 | 0.409091 | 0.254545 | . | 0.263636 | | 0 | 0.381818 | 0 | 0 | 0 | 0.163636 | 0.054545 | | 0.081818 |
| 0.045455 | 0 | 0.13 | 0 | 0.18 | 0.183333 | 0 | 0.009091 | . | 0.090909 | | 0 | 0 | 0.227273 | 0 | 0 | 0.354545 | 0 | | 0.527273 |
| 0 | 0 | 0 | 0 | 0.09 | 0.246667 | 0.509091 | 0.027273 | . | 0 | | 0 | 0 | 0.427273 | 0.390909 | 0 | 0 | 0.409091 | | 0.009091 |
| 0 | 0.236364 | 0.013333 | 0 | 0 | 0.08 | 0.072727 | 0 | . | 0 | | 0 | 0.409091 | 0 | 0.254545 | 0 | 0 | 0.027273 | | 0.036364 |
| 0.054545 | 0 | 0.1 | 0 | 0 | 0 | 0.263636 | 0 | . | 0 | | 0.218182 | 0.018182 | 0 | 0.009091 | 0 | 0 | 0 | | 0.018182 |
| 0 | 0 | 0.026667 | 0 | 0 | 0 | 0.018182 | 0.236364 | . | 0 | | 0 | 0.363636 | 0.063636 | 0 | 0 | 0.009091 | 0 | | 0.145455 |
| 0 | 0 | 0.096667 | 0 | 0 | 0 | 0.090909 | 0 | . | 0.154545 | | 0.1 | 0 | 0.072727 | 0.3 | 0.245455 | 0 | 0.209091 | | 0 |
| 0.027273 | 0.109091 | 0.006667 | 0.15 | 0.033333 | 0.16 | 0.190909 | 0 | . | 0 | | 0.118182 | 0.445455 | 0.554545 | 0.090909 | 0.254545 | 0.327273 | 0.127273 | | 0.527273 |
| 0 | 0 | 0.073333 | 0 | 0.126667 | 0.003333 | 0.518182 | 0.190909 | . | 0 | | 0.281818 | 0 | 0 | 0 | 0.109091 | 0.136364 | 0.063636 | | 0 |
| 0.018182 | 0 | 0.06 | 0.1 | 0 | 0 | 0.027273 | 0 | . | 0.363636 | | 0.018182 | 0.036364 | 0 | 0.336364 | 0.236364 | 0 | 0.009091 | | 0.409091 |
| 0.009091 | 0.327273 | 0.04 | 0.003333 | 0 | 0 | 0 | 0.1 | . | 0 | | 0 | 0.027273 | 0 | 0 | 0.036364 | 0.472727 | 0.254545 | | 0.145455 |
| 0.072727 | 0 | 0.113333 | 0.006667 | 0.083333 | 0 | | | | | | 0.381818 | 0.063636 | | 0 | 0.190909 | | | | |
| 0 | 0.1 | 0.245455 | 0.236364 | 0.081818 | 0.263636 | 0.053333 | 0.06 | 0.145455 | 0.027273 | 0.2 | 0.190909 | 0.327273 | 0.009091 | 0.118182 | 0.127273 | 0.190909 | 0.018182 | 0.190909 | 0.154545 |
| 0.254545 | 0 | 0.254545 | 0.2 | 0.263636 | 0.072727 | 0.056667 | 0.023333 | 0.445455 | 0.3 | 0 | 0.163636 | 0.209091 | 0.2 | 0.381818 | 0.009091 | 0.009091 | 0.018182 | 0.245455 | 0.018182 |
| 0 | 0 | 0.109091 | 0.281818 | 0.409091 | 0.390909 | 0 | 0.11 | 0.109091 | 0 | 0.290909 | 0.127273 | 0.090909 | 0.2 | 0.009091 | 0.354545 | 0.181818 | 0 | 0.272727 | 0.154545 |
| 0.527273 | 0.318182 | 0.209091 | 0.172727 | 0 | 0.2 | 0.11 | 0.066667 | 0.181818 | 0.154545 | 0.009091 | 0.281818 | 0.527273 | 0.290909 | 0.327273 | 0.018182 | 0 | 0 | 0 | 0.127273 |
| 0 | 0.281818 | 0.045455 | 0.154545 | 0 | 0 | 0.05 | 0.043333 | 0.118182 | 0.236364 | 0 | 0 | 0 | 0 | 0.181818 | 0.1 | 0.172727 | 0.118182 | 0.2 | 0 |
| 0.136364 | 0 | 0.2 | 0.245455 | 0.327273 | 0.381818 | 0.01 | 0.05 | 0.254545 | 0 | 0.245455 | 0.118182 | 0 | 0.127273 | 0.154545 | 0.063636 | 0.218182 | 0.1 | 0 | 0.290909 |
| 0 | 0 | 0.018182 | 0 | 0 | 0.072727 | 0.036667 | 0 | 0.1 | 0.318182 | 0.145455 | 0.036364 | 0.218182 | 0.172727 | 0 | 0.136364 | 0 | 0.218182 | 0.209091 | 0.072727 |
| 0.154545 | 0 | 0.090909 | 0.372727 | 0.354545 | 0 | 0.093333 | 0 | 0.127273 | 0.027273 | 0.009091 | 0 | 0.027273 | 0 | 0.118182 | 0.027273 | 0.163636 | 0.027273 | 0 | 0.027273 |
| 0.054545 | 0 | 0 | 0.109091 | 0.054545 | 0.4 | 0 | 0 | 0.036364 | 0 | 0.118182 | 0.027273 | 0.018182 | 0.072727 | 0 | 0.254545 | 0.227273 | 0 | 0.418182 | 0.118182 |
| 0.1 | 0.009091 | 0.054545 | 0.109091 | 0.372727 | 0.145455 | 0.046667 | 0.026667 | 0.036364 | 0 | 0.018182 | 0 | 0.063636 | 0.136364 | 0.081818 | 0 | 0 | 0.054545 | 0 | 0.145455 |
| 0.172727 | 0.127273 | 0.090909 | 0 | 0.063636 | 0.009091 | 0.006667 | 0.033333 | 0 | 0.118182 | 0.218182 | 0.081818 | 0 | 0.054545 | 0.009091 | 0.063636 | 0 | 0 | 0 | 0.154545 |
| 0.054545 | 0.336364 | 0.127273 | 0.009091 | 0 | 0 | 0 | 0 | 0.136364 | 0 | 0.209091 | 0.045455 | 0.1 | 0.145455 | 0 | 0.254545 | 0.118182 | 0.345455 | 0 | 0.1 |
| 0 | 0 | 0.427273 | 0.163636 | 0 | 0.2 | 0.023333 | 0.04 | 0.345455 | 0.036364 | 0.009091 | 0.045455 | 0.154545 | 0.345455 | 0.063636 | 0.154545 | 0.063636 | 0.027273 | 0.109091 | 0 |
| 0 | 0.172727 | 0.136364 | 0.227273 | 0.090909 | 0.072727 | 0 | 0.023333 | 0 | 0.209091 | 0.090909 | 0.163636 | 0.009091 | 0.009091 | 0.109091 | 0.009091 | 0.290909 | 0 | 0 | 0.372727 |
| 0.172727 | 0.109091 | 0 | 0.172727 | 0.118182 | 0 | 0.066667 | 0.066667 | 0.154545 | 0.209091 | 0.118182 | 0 | 0.081818 | 0.1 | 0 | 0.009091 | 0 | 0.163636 | 0.027273 | 0.081818 |
| 0.090909 | 0 | 0.236364 | 0.236364 | 0.127273 | 0.227273 | 0 | 0.043333 | 0 | 0.154545 | 0 | 0.427273 | 0.072727 | 0.118182 | 0.136364 | 0.309091 | 0.109091 | 0.172727 | 0.109091 | 0.045455 |
| 0 | 0 | 0.072727 | 0 | 0 | 0 | 0.036667 | 0.06 | 0.090909 | 0.009091 | 0 | 0.1 | 0.063636 | 0 | 0.081818 | 0 | 0.172727 | 0.027273 | 0.1 | 0.245455 |
| 0.127273 | 0.190909 | 0.154545 | 0.381818 | 0 | 0.227273 | 0.003333 | 0.01 | 0.1 | 0.2 | 0.118182 | 0 | 0 | 0.027273 | 0.145455 | 0.127273 | 0.054545 | 0 | 0.018182 | 0.109091 |
| 0 | 0.336364 | 0.118182 | 0.090909 | 0.354545 | 0.236364 | 0 | 0.03 | 0.181818 | 0.172727 | 0.1 | 0.227273 | 0.254545 | 0.281818 | 0 | 0.136364 | 0.136364 | 0.145455 | 0 | 0.190909 |
| 0.3 | 0.127273 | 0.263636 | 0.181818 | 0.318182 | 0.336364 | 0.09 | 0.06 | 0.3 | 0.245455 | 0.054545 | 0.227273 | 0.181818 | 0.009091 | 0.163636 | 0.009091 | 0.190909 | 0.036364 | 0.209091 | 0.418182 |
| 0.227273 | 0 | 0.3 | 0.254545 | 0 | 0.145455 | 0.036667 | 0.126667 | 0 | 0.309091 | 0.127273 | 0.245455 | 0.209091 | 0.190909 | 0.327273 | 0.281818 | 0.336364 | 0.227273 | 0.154545 | 0.181818 |
| 0 | 0.263636 | 0.145455 | 0 | 0.327273 | 0.127273 | 0.073333 | 0.113333 | 0.145455 | 0.154545 | 0.118182 | 0 | 0.145455 | 0.145455 | 0.4 | 0.018182 | 0.009091 | 0 | 0.290909 | 0.036364 |
| 0.163636 | 0 | 0.109091 | 0.236364 | 0.209091 | 0.236364 | 0.053333 | 0.03 | 0.136364 | 0.018182 | 0.109091 | 0.354545 | 0.236364 | 0.127273 | 0.2 | 0.218182 | 0.136364 | 0.327273 | 0.145455 | 0.009091 |
| 0.190909 | 0 | 0.281818 | 0.027273 | 0.2 | 0.263636 | 0.083333 | 0 | 0.481818 | 0 | 0.018182 | 0.072727 | 0.281818 | 0.009091 | 0.018182 | 0.218182 | 0.236364 | 0.063636 | 0.254545 | 0.063636 |
| 0.145455 | 0.363636 | 0.009091 | 0.281818 | 0.227273 | 0.327273 | 0 | 0.086667 | 0 | 0.245455 | 0.109091 | 0.181818 | 0 | 0.245455 | 0.372727 | 0.163636 | 0 | 0 | 0.063636 | 0.2 |
| 0.190909 | 0 | 0 | 0.327273 | 0 | 0.1 | 0.026667 | 0 | 0.3 | 0.254545 | 0.145455 | 0.227273 | 0.136364 | 0.027273 | 0.172727 | 0.009091 | 0.190909 | 0.127273 | 0.018182 | 0.163636 |
| 0.1 | 0 | 0.245455 | 0 | 0.127273 | 0.081818 | 0.036667 | 0.07 | 0.009091 | 0 | 0 | 0.009091 | 0.127273 | 0.127273 | 0.381818 | 0.281818 | 0.136364 | 0.190909 | 0.245455 | 0 |
| 0.027273 | 0.590909 | 0.2 | 0.127273 | 0.572727 | 0.281818 | 0.003333 | 0.003333 | 0.118182 | 0.154545 | 0 | 0.490909 | 0.009091 | 0.209091 | 0 | 0 | 0.136364 | 0.036364 | 0.336364 | 0.327273 |
| 0 | 0 | 0 | 0.3 | 0 | 0.054545 | 0.016667 | 0.026667 | 0.163636 | 0 | 0 | 0.009091 | 0.2 | 0.018182 | 0.2 | 0.145455 | 0.218182 | 0.018182 | 0.009091 | 0 |
| 0 | 0.263636 | 0.218182 | 0.063636 | 0 | 0.018182 | 0.023333 | 0.056667 | 0.063636 | 0.309091 | 0.127273 | 0 | 0 | 0.309091 | 0.009091 | 0.227273 | 0.027273 | 0 | 0 | 0.354545 |
| 0.136364 | 0 | 0.118182 | 0.209091 | 0.009091 | 0.381818 | 0 | 0.043333 | 0.009091 | 0.018182 | 0 | 0.127273 | 0.054545 | 0.145455 | 0 | 0.109091 | 0 | 0.290909 | 0.372727 | 0 |
| 0 | 0 | 0.127273 | 0.272727 | 0.109091 | 0.209091 | 0.016667 | 0 | 0.090909 | 0.254545 | 0 | 0.145455 | 0.163636 | 0.136364 | 0.1 | 0.063636 | 0 | 0 | 0 | 0.236364 |
| 0 | 0.209091 | 0 | 0.054545 | 0 | 0.118182 | 0.003333 | 0.043333 | 0.027273 | 0 | 0.154545 | 0.009091 | 0 | 0.018182 | 0.127273 | 0.081818 | 0.127273 | 0.109091 | 0.045455 | 0.009091 |
| 0.263636 | 0 | 0.118182 | 0.172727 | 0.363636 | 0.181818 | 0.003333 | 0.023333 | 0.163636 | 0.190909 | 0 | 0 | 0.163636 | 0.109091 | 0.163636 | 0.145455 | 0.172727 | 0.045455 | 0 | 0.036364 |
| 0 | 0.009091 | 0.027273 | 0 | 0.045455 | 0.036364 | 0.023333 | 0.08 | 0.081818 | 0.063636 | 0.145455 | 0.145455 | 0 | 0.081818 | 0.009091 | 0.190909 | 0.072727 | 0 | 0 | 0 |
| 0.254545 | 0 | 0 | 0.163636 | 0.027273 | 0.054545 | 0.003333 | 0 | 0.1 | 0.081818 | 0 | 0.109091 | 0.127273 | 0.118182 | 0 | 0.090909 | 0.218182 | 0.1 | 0.245455 | 0.3 |
| 0.136364 | 0.390909 | 0.2 | 0 | 0.1 | 0.081818 | 0.016667 | 0.02 | 0.145455 | 0.018182 | 0.072727 | 0.018182 | 0.072727 | 0.009091 | 0.272727 | 0.172727 | 0.136364 | 0.1 | 0 | 0.081818 |
| 0.009091 | 0.254545 | 0.118182 | 0.154545 | 0.181818 | 0 | 0.04 | 0.033333 | 0.027273 | 0 | 0.145455 | 0.127273 | 0.127273 | 0.154545 | 0 | 0.009091 | 0 | 0.009091 | 0 | 0.027273 |
| 0.154545 | 0.009091 | 0.1 | 0.236364 | 0 | 0.118182 | 0.05 | 0.013333 | 0.109091 | 0.2 | 0.209091 | 0 | 0 | 0.090909 | 0.190909 | 0.090909 | 0.109091 | 0.1 | 0.181818 | 0.063636 |
| 0.081818 | 0.272727 | 0.063636 | 0.063636 | 0 | 0.154545 | 0.023333 | 0.023333 | 0.1 | 0.172727 | 0.154545 | 0.018182 | 0.090909 | 0 | 0 | 0.290909 | 0.136364 | 0 | 0 | 0 |
| 0.163636 | 0 | 0.045455 | 0.009091 | 0.318182 | 0.145455 | 0.02 | 0.12 | 0.063636 | 0.209091 | 0.136364 | 0.136364 | 0.018182 | 0.090909 | 0.036364 | 0 | 0.1 | 0.236364 | 0.136364 | 0.109091 |
| 0.127273 | 0.363636 | 0.236364 | 0.163636 | 0.009091 | 0.172727 | 0.123333 | 0.003333 | 0.181818 | 0.145455 | 0 | 0.209091 | 0.163636 | 0.236364 | 0.309091 | 0.281818 | 0 | 0.209091 | 0.190909 | 0.072727 |
| 0 | 0.018182 | 0.354545 | 0.2 | 0.145455 | 0.1 | 0 | 0.106667 | 0.172727 | 0.281818 | 0.136364 | 0.3 | 0.190909 | 0.009091 | 0.245455 | 0 | 0.190909 | 0.090909 | 0 | 0.409091 |
| 0.172727 | 0.381818 | 0.172727 | 0.309091 | 0.245455 | 0 | 0.013333 | 0 | 0.118182 | 0 | 0.236364 | 0 | 0.145455 | 0.190909 | 0.163636 | 0.345455 | 0.136364 | 0 | 0.190909 | 0.327273 |
| 0.063636 | 0 | 0.054545 | 0.136364 | 0.336364 | 0.3 | 0.05 | 0.063333 | 0.072727 | 0.272727 | 0 | 0.145455 | 0.172727 | 0.009091 | 0.1 | 0.063636 | 0.327273 | 0 | 0.372727 | 0.018182 |
| 0.136364 | 0 | 0.418182 | 0.327273 | 0.145455 | 0.418182 | 0.083333 | 0.07 | 0.145455 | 0.045455 | 0.009091 | 0.045455 | 0.009091 | 0.009091 | 0.118182 | 0.090909 | 0.009091 | 0 | 0 | 0.145455 |
| 0.027273 | 0 | 0 | 0.227273 | 0.345455 | 0.272727 | 0.033333 | 0 | 0.181818 | 0 | 0.063636 | 0.118182 | 0.209091 | 0.109091 | 0.181818 | 0.127273 | 0.372727 | 0.3 | 0.009091 | 0 |
| 0.154545 | 0.154545 | 0.354545 | 0.190909 | 0 | 0.1 | 0.046667 | 0.073333 | 0.118182 | 0.172727 | 0.045455 | 0.227273 | 0.090909 | 0 | 0.1 | 0.027273 | 0 | 0.009091 | 0 | 0 |
| 0.1 | 0.009091 | 0 | 0.190909 | 0 | 0.1 | 0 | 0.083333 | 0.036364 | 0.3 | 0.154545 | 0.236364 | 0.427273 | 0.1 | 0.163636 | 0.127273 | 0.309091 | 0.045455 | 0.2 | 0.281818 |
| 0.145455 | 0.318182 | 0 | 0.181818 | 0.072727 | 0.263636 | 0.063333 | 0.013333 | 0.209091 | 0 | 0.009091 | 0.236364 | 0.009091 | 0.009091 | 0.063636 | 0.127273 | 0.154545 | 0.163636 | 0.2 | 0 |
| 0.118182 | 0 | 0.209091 | 0.172727 | 0.109091 | 0.054545 | 0.086667 | 0.083333 | 0.127273 | 0.281818 | 0.209091 | 0.181818 | 0.136364 | 0.036364 | 0.009091 | 0.054545 | 0.136364 | 0.072727 | 0.018182 | 0 |
| 0.036364 | 0.281818 | 0 | 0.136364 | 0.118182 | 0.145455 | 0.003333 | 0 | 0.009091 | 0 | 0.145455 | 0 | 0.209091 | 0 | 0 | 0.118182 | 0 | 0 | 0.163636 | 0.336364 |
| 0.172727 | 0.1 | 0.045455 | 0.1 | 0 | 0.218182 | 0 | 0.066667 | 0.054545 | 0.072727 | 0.163636 | 0.272727 | 0.009091 | 0.236364 | 0.045455 | 0 | 0.136364 | 0 | 0 | 0.090909 |
| 0.145455 | 0.2 | 0.118182 | 0.036364 | 0.009091 | 0.081818 | 0.036667 | 0 | 0.118182 | 0.045455 | 0.109091 | 0.081818 | 0 | 0 | 0.127273 | 0.109091 | 0.018182 | 0.1 | 0.036364 | 0.009091 |
| 0.081818 | 0.018182 | 0.063636 | 0 | 0.009091 | 0.009091 | 0 | 0.073333 | 0 | 0.109091 | 0 | 0.245455 | 0.045455 | 0.009091 | 0 | 0.154545 | 0 | 0.054545 | 0 | 0 |
| 0.018182 | 0 | 0.172727 | 0.363636 | 0.127273 | 0.136364 | 0.063333 | 0.05 | 0.081818 | 0.181818 | 0.054545 | 0 | 0.090909 | 0.045455 | 0 | 0.145455 | 0.254545 | 0.081818 | 0 | 0 |
| 0.154545 | 0.2 | 0.018182 | 0 | 0.063636 | 0 | 0 | 0 | 0 | 0 | 0.145455 | 0.018182 | 0 | 0.127273 | 0 | 0.036364 | 0.245455 | 0 | 0.145455 | 0.154545 |
| 0.063636 | 0 | 0.172727 | 0.190909 | 0 | 0.136364 | 0.043333 | 0.093333 | 0.172727 | 0.027273 | 0.027273 | 0.136364 | 0.163636 | 0.018182 | 0.163636 | 0.1 | 0 | 0.054545 | 0.109091 | 0 |
| 0.1 | 0.045455 | 0 | 0.054545 | 0.009091 | 0.009091 | 0 | 0.013333 | 0 | 0.2 | 0 | 0 | 0.090909 | 0 | 0 | 0.118182 | 0.018182 | 0.018182 | 0 | 0 |
| 0.127273 | 0 | 0.090909 | 0.118182 | 0.018182 | 0.018182 | 0 | 0.01 | 0.072727 | 0.009091 | 0.109091 | 0 | 0.009091 | 0.163636 | 0.118182 | 0.090909 | 0.218182 | 0.236364 | 0.209091 | 0.327273 |
| 0.118182 | 0 | 0.118182 | 0.045455 | 0.009091 | 0.009091 | 0.06 | 0.003333 | 0 | 0 | 0.2 | 0.127273 | 0.209091 | 0.018182 | 0.1 | 0.018182 | 0 | 0.209091 | 0 | 0.018182 |
| 0.190909 | 0.163636 | 0.118182 | 0.109091 | 0.063636 | 0.127273 | 0.04 | 0.04 | 0.136364 | 0.145455 | | 0.018182 | 0 | 0.145455 | 0 | 0.263636 | 0.136364 | 0.127273 | | 0 |
| 0.2 | 0 | 0.3 | 0.181818 | 0.072727 | 0.1 | 0.033333 | 0 | 0.118182 | 0 | | 0.2 | 0.072727 | 0.036364 | 0.254545 | 0 | 0.009091 | 0.027273 | | 0.1 |
| 0.027273 | 0.227273 | 0 | 0.090909 | 0.009091 | 0 | 0.086667 | 0.086667 | 0.281818 | 0.209091 | | 0 | 0.181818 | 0.127273 | 0.036364 | 0.163636 | 0.145455 | 0.127273 | | 0 |
| 0.290909 | 0.3 | 0.427273 | 0.136364 | 0.118182 | 0.118182 | 0 | 0.076667 | 0 | 0 | | 0.309091 | 0.018182 | 0.227273 | 0.190909 | 0.054545 | 0.145455 | 0.127273 | | 0.336364 |
| 0.072727 | 0 | 0.090909 | 0.227273 | 0.309091 | 0.272727 | 0.073333 | 0.046667 | 0.109091 | 0.218182 | | 0.109091 | 0.263636 | 0.172727 | 0.181818 | 0.1 | 0.045455 | 0.1 | | 0.027273 |
| 0.009091 | 0 | 0.145455 | 0.054545 | 0.2 | 0.254545 | 0.01 | 0.01 | 0.009091 | 0 | | 0.181818 | 0.090909 | 0 | 0.2 | 0 | 0.018182 | 0.081818 | | 0.027273 |
| 0.145455 | 0.054545 | 0.181818 | 0.127273 | 0.290909 | 0.163636 | 0.07 | 0.063333 | 0.390909 | 0.190909 | | 0.018182 | 0.327273 | 0.309091 | 0.236364 | 0.109091 | 0.209091 | 0 | | 0.236364 |
| 0 | 0.290909 | 0.145455 | 0.163636 | 0.2 | 0.063636 | 0.066667 | 0.006667 | 0.263636 | 0.118182 | | 0.336364 | 0 | 0 | 0.145455 | 0.145455 | 0.272727 | 0.181818 | | 0.027273 |
| 0.145455 | 0.027273 | 0.254545 | 0.072727 | 0.009091 | 0.245455 | 0 | 0.093333 | 0.172727 | 0.163636 | | 0.190909 | 0.081818 | 0.263636 | 0.181818 | 0.109091 | 0 | 0.009091 | | 0 |
| 0.145455 | 0.309091 | 0.218182 | 0.009091 | 0.290909 | 0.136364 | 0.063333 | 0.046667 | 0.2 | 0.072727 | | 0.136364 | 0.354545 | 0.236364 | 0.227273 | 0.081818 | 0.154545 | 0.181818 | | 0.090909 |
| 0.209091 | 0.009091 | 0.136364 | 0.045455 | 0 | 0.036364 | 0.083333 | 0.076667 | 0.090909 | 0 | | 0 | 0 | 0.072727 | 0.163636 | 0.118182 | 0.1 | 0.090909 | | 0 |
| 0 | 0.009091 | 0.127273 | 0.2 | 0.236364 | 0.118182 | 0 | 0 | 0 | 0.163636 | | 0 | 0 | 0.072727 | 0.254545 | 0.072727 | 0.181818 | 0 | | 0 |
| 0.063636 | 0 | 0.136364 | 0.072727 | 0 | 0.063636 | 0 | 0.03 | 0.063636 | 0 | | 0.154545 | 0.318182 | 0 | 0.054545 | 0.009091 | 0 | 0.172727 | | 0 |
| 0 | 0 | 0.163636 | 0.027273 | 0.109091 | 0.181818 | 0.086667 | 0.056667 | 0.109091 | 0.054545 | | 0.136364 | 0.3 | 0.063636 | 0.118182 | 0 | 0 | 0.045455 | | 0 |
| 0.181818 | 0 | 0.036364 | 0.072727 | 0 | 0.018182 | 0 | 0.003333 | 0 | 0 | | 0 | 0 | 0 | 0.009091 | 0 | 0.036364 | 0 | | 0.009091 |
| 0 | 0.145455 | 0.081818 | 0 | 0 | 0.127273 | 0.063333 | 0.01 | 0.081818 | 0.2 | | 0.227273 | 0 | 0.027273 | 0.027273 | 0 | 0.045455 | 0.036364 | | 0.063636 |
| 0.127273 | 0 | 0.118182 | 0.118182 | 0.227273 | 0.027273 | 0 | 0.03 | 0.118182 | 0 | | 0.018182 | 0.154545 | 0.1 | 0.118182 | 0.081818 | 0.009091 | 0.027273 | | 0 |
| 0 | 0.145455 | 0.109091 | 0 | 0 | 0.109091 | 0.063333 | 0.05 | 0.1 | 0.009091 | | 0.209091 | 0.109091 | 0.118182 | 0 | 0.054545 | 0.090909 | 0.063636 | | 0.063636 |
| 0.190909 | 0.054545 | 0 | 0.172727 | 0 | 0.236364 | 0 | 0 | 0.272727 | 0 | | 0.2 | 0.145455 | 0.118182 | 0.109091 | 0 | 0.063636 | 0.290909 | | 0 |
| 0 | 0 | 0.045455 | 0.218182 | 0.145455 | 0.009091 | 0 | 0.046667 | 0.145455 | 0.245455 | | 0 | 0 | 0.009091 | 0.172727 | 0.154545 | 0 | 0 | | 0 |
| 0.1 | 0.172727 | 0.109091 | 0.054545 | 0.136364 | 0.018182 | 0.036667 | 0 | 0.063636 | 0.136364 | | 0.009091 | 0 | 0.090909 | 0.072727 | 0.154545 | 0.009091 | 0 | | 0.045455 |
| | | 0.163636 | 0.009091 | 0.027273 | 0.145455 | | | | | | 0.154545 | 0.109091 | | | | | | | |

| 7 | 7 | 10 | 10 | 12 | 12 | 12 | 1 | 1 | 1 | 1 | 2 | 2 | 5 | 6 | 6 | 8 | 9 | 11 | | 1 | 2 | 2 |
|---|---|---|---|---|---|---|---|---|---|---|---|---|---|---|---|---|---|---|---|---|---|---|
| Box-6 | Box-8 | Box-7 | Box-2 | Box-3 | Box-4 | Box-7 | Box-1 | Box-3 | Box-4 | Box-5 | Box-2 | Box-3 | Box-1 | Box-2 | Box-3 | Box-8 | Box-7 | Box-5 | | Box-6 | Box-6 | Box-8 |
| 45 | 47 | 65 | 68 | 77 | 78 | 84 | 6.1 | 9.4 | 10.1 | 11.6 | 16.4 | 17.4 | 28.1 | 41 | 42 | 53.2 | 71 | 82 | | 12.1 | 23.3 | 27.5 |
| F | F | F | F | F | F | F | M | M | M | M | M | M | M | M | M | M | M | M | | M | M | M |
| MCT | MCT | MCT | MCT | MCT | MCT | MCT | EtOH | EtOH | EtOH | EtOH | EtOH | EtOH | EtOH | EtOH | EtOH | EtOH | EtoH | EtOH | | H2O | H2O | H2O |
| FT | FT | FT | FT | FT | FT | FT | ME | ME | ME | ME | ME | ME | ME | ME | ME | ME | ME | ME | | MH | MH | MH |
| 0 | 0 | 0 | 0 | 0 | 0 | 0 | 0.054545 | 0.145455 | 0.045455 | 0.090909 | 0.190909 | 0 | 0.063636 | 0.163636 | 0.218182 | 0 | 0 | 0 | | 0 | 0.1 | 0 |
| 0 | 0.009091 | 0 | 0 | 0 | 0 | 0 | 0.227273 | 0.236364 | 0.163636 | 0.181818 | 0.072727 | 0 | 0.318182 | 0.236364 | 0.2 | 0 | 0 | 0 | | 0.309091 | 0 | 0.054545 |
| 0.081818 | 0 | 0 | 0 | 0.009091 | 0 | 0 | 0.163636 | 0.290909 | 0.2 | 0.190909 | 0 | 0 | 0.209091 | 0.372727 | 0.336364 | 0 | 0 | 0 | | 0.118182 | 0.354545 | 0.163636 |
| 0.181818 | 0 | 0 | 0 | 0.145455 | 0 | 0 | 0.381818 | 0.572727 | 0.4 | 0.472727 | 0.081818 | 0.027273 | 0.290909 | 0.209091 | 0.309091 | 0.263636 | 0 | 0 | | 0.218182 | 0.227273 | 0.054545 |
| 0.354545 | 0.009091 | 0 | 0.1 | 0.109091 | 0.045455 | 0 | 0.236364 | 0.363636 | 0.318182 | 0.472727 | 0 | 0 | 0.227273 | 0.427273 | 0.327273 | 0.418182 | 0 | 0.036364 | | 0.118182 | 0.281818 | 0 |
| 0.354545 | 0 | 0 | 0.127273 | 0.090909 | 0.1 | 0 | 0.263636 | 0.145455 | 0.618182 | 0.254545 | 0 | 0.127273 | 0.281818 | 0.390909 | 0.554545 | 0.3 | 0.06 | 0.236364 | | 0.336364 | 0.3 | 0.227273 |
| 0.090909 | 0.1 | 0 | 0.254545 | 0 | 0.563636 | 0.072727 | 0.227273 | 0.345455 | 0.081818 | 0.136364 | 0 | 0 | 0.263636 | 0.172727 | 0.027273 | 0.218182 | 0.03 | 0.109091 | | 0.481818 | 0.372727 | 0 |
| 0 | 0.145455 | 0 | 0.2 | 0.190909 | 0.472727 | 0.209091 | 0.145455 | 0.181818 | 0.409091 | 0.345455 | 0 | 0 | 0.272727 | 0.163636 | 0.263636 | 0.327273 | 0.183333 | 0.309091 | | 0.081818 | 0.163636 | 0.5 |
| 0 | 0.5 | 0 | 0.209091 | 0 | 0.136364 | 0 | 0.345455 | 0.009091 | 0.245455 | 0.063636 | 0 | 0 | 0 | 0.254545 | 0.145455 | 0.154545 | 0.063333 | 0.018182 | | 0.390909 | 0.272727 | 0 |
| 0.345455 | 0.245455 | 0 | 0.318182 | 0 | 0.027273 | 0.063636 | 0.136364 | 0.327273 | 0.190909 | 0.072727 | 0 | 0 | 0.109091 | 0 | 0.154545 | 0.290909 | 0.03 | 0.027273 | | 0.190909 | 0.118182 | 0.090909 |
| 0 | 0 | 0 | 0 | 0.072727 | 0.427273 | 0 | 0 | 0.027273 | 0.045455 | 0.418182 | 0 | 0 | 0 | 0.190909 | 0.109091 | 0 | 0.05 | 0 | | 0.463636 | 0.018182 | 0.454545 |
| 0 | 0.172727 | 0 | 0.145455 | 0 | 0.3 | 0 | 0.327273 | 0.090909 | 0.072727 | 0 | 0 | 0.163636 | 0.209091 | 0.018182 | 0.290909 | 0.372727 | 0.053333 | 0.263636 | | 0.263636 | 0.381818 | 0 |
| 0.190909 | 0.127273 | 0 | 0.227273 | 0.136364 | 0 | 0 | 0.018182 | 0 | 0.163636 | 0.172727 | 0.090909 | 0.209091 | 0.181818 | 0.263636 | 0.063636 | 0 | 0.073333 | 0.081818 | | 0.154545 | 0.272727 | 0.4 |
| 0.218182 | 0.136364 | 0 | 0.109091 | 0 | 0 | 0 | 0.181818 | 0.009091 | 0.118182 | 0.127273 | 0.445455 | 0 | 0.136364 | 0.045455 | 0 | 0.081818 | 0.013333 | 0.054545 | | 0 | 0 | 0 |
| 0.209091 | 0 | 0 | 0.027273 | 0 | 0.627273 | 0 | 0 | 0.254545 | 0.154545 | 0.145455 | 0.309091 | 0.2 | 0.045455 | 0.2 | 0.218182 | 0 | 0 | 0 | | 0.209091 | 0.1 | 0 |
| 0.318182 | 0 | 0 | 0.163636 | 0.018182 | 0 | 0 | 0.163636 | 0.054545 | 0.090909 | 0.136364 | 0 | 0 | 0 | 0 | 0.118182 | 0 | 0.05 | 0.018182 | | 0.190909 | 0.227273 | 0 |
| 0 | 0.5 | 0 | 0 | 0.036364 | 0 | 0 | 0 | 0.009091 | 0.209091 | 0.072727 | 0.027273 | 0.1 | 0.145455 | 0 | 0 | 0.127273 | 0 | 0 | | 0.063636 | 0 | 0.281818 |
| 0 | 0 | 0 | 0.263636 | 0 | 0.181818 | 0 | 0.218182 | 0.172727 | 0 | 0.045455 | 0.018182 | 0 | 0.036364 | 0.163636 | 0.218182 | 0.172727 | 0.036667 | 0 | | 0.045455 | 0.354545 | 0 |
| 0.154545 | 0.109091 | 0.009091 | 0 | 0 | 0.372727 | 0.463636 | 0 | 0.063636 | 0.109091 | 0 | 0 | 0 | 0.218182 | 0.081818 | 0.036364 | 0.009091 | 0.116667 | 0 | | 0.2 | 0 | 0.3 |
| 0.263636 | 0.1 | 0 | 0.163636 | 0 | 0 | 0.127273 | 0.1 | 0.072727 | 0.036364 | 0.1 | 0 | 0.254545 | 0.154545 | 0 | 0 | 0.109091 | 0 | 0.090909 | | 0 | 0 | 0 |
| 0.163636 | 0.218182 | 0.009091 | 0.045455 | 0.009091 | 0 | 0 | 0 | 0.072727 | 0.190909 | 0.181818 | 0 | 0 | 0 | 0.072727 | 0.081818 | 0 | 0.12 | 0 | | 0.018182 | 0.136364 | 0 |
| 0.154545 | 0.236364 | 0 | 0.209091 | 0.018182 | 0 | 0.636364 | 0.2 | 0.109091 | 0.2 | 0.109091 | 0.072727 | 0.263636 | 0.290909 | 0 | 0.272727 | 0.154545 | 0.006667 | 0 | | 0.136364 | 0.127273 | 0.427273 |
| 0.245455 | 0.054545 | 0 | 0.118182 | 0.236364 | 0.518182 | 0 | 0 | 0 | 0.245455 | 0.063636 | 0.390909 | 0 | 0 | 0.2 | 0.472727 | 0 | 0.046667 | 0 | | 0.2 | 0.290909 | 0 |
| 0.445455 | 0.045455 | 0 | 0.018182 | 0.009091 | 0.136364 | 0.209091 | 0.190909 | 0.381818 | 0.127273 | 0.281818 | 0 | 0.236364 | 0.263636 | 0.318182 | 0 | 0.409091 | 0.086667 | 0.027273 | | 0 | 0 | 0.4 |
| 0.2 | 0.218182 | 0 | 0.172727 | 0.1 | 0.372727 | 0.563636 | 0.490909 | 0.5 | 0.290909 | 0.218182 | 0.290909 | 0.290909 | 0.372727 | 0.436364 | 0.172727 | 0.072727 | 0.006667 | 0.345455 | | 0.254545 | 0.436364 | 0.409091 |
| 0.381818 | 0.227273 | 0 | 0.145455 | 0.236364 | 0.445455 | 0.354545 | 0.109091 | 0.327273 | 0.572727 | 0.509091 | 0.245455 | 0 | 0.163636 | 0.481818 | 0.481818 | 0.572727 | 0.033333 | 0.236364 | | 0.263636 | 0.318182 | 0.018182 |
| 0.263636 | 0.127273 | 0 | 0.2 | 0.154545 | 0.009091 | 0.6 | 0.372727 | 0.2 | 0.409091 | 0.245455 | 0.536364 | 0.281818 | 0.363636 | 0.263636 | 0.2 | 0.372727 | 0.09 | 0.209091 | | 0.290909 | 0.318182 | 0.218182 |
| 0 | 0.527273 | 0 | 0.109091 | 0.472727 | 0.281818 | 0.1 | 0.145455 | 0.281818 | 0.118182 | 0.054545 | 0.418182 | 0.172727 | 0.172727 | 0.309091 | 0.027273 | 0.218182 | 0.023333 | 0.190909 | | 0.163636 | 0.345455 | 0.318182 |
| 0.309091 | 0.136364 | 0 | 0.190909 | 0.581818 | 0.518182 | 0.290909 | 0.254545 | 0 | 0.236364 | 0.336364 | 0.309091 | 0.218182 | 0.181818 | 0.190909 | 0.381818 | 0.109091 | 0.01 | 0.527273 | | 0.245455 | 0 | 0 |
| 0.290909 | 0.227273 | 0 | 0 | 0.318182 | 0.290909 | 0.336364 | 0.254545 | 0.472727 | 0.245455 | 0.209091 | 0.218182 | 0.236364 | 0.209091 | 0.245455 | 0 | 0.409091 | 0.16 | 0.245455 | | 0.127273 | 0.345455 | 0.418182 |
| 0 | 0.181818 | 0 | 0.5 | 0.381818 | 0.127273 | 0.181818 | 0.281818 | 0.063636 | 0.181818 | 0.372727 | 0 | 0.2 | 0.163636 | 0.009091 | 0.2 | 0 | 0.02 | 0.3 | | 0.081818 | 0.245455 | 0 |
| 0 | 0.309091 | 0 | 0.009091 | 0 | 0.136364 | 0 | 0.209091 | 0.172727 | 0.218182 | 0 | 0.281818 | 0.109091 | 0.181818 | 0.236364 | 0.254545 | 0.318182 | 0.033333 | 0 | | 0.136364 | 0.345455 | 0 |
| 0.509091 | 0.554545 | 0 | 0.127273 | 0.209091 | 0.081818 | 0.309091 | 0.3 | 0.190909 | 0.181818 | 0.172727 | 0.227273 | 0 | 0.318182 | 0.154545 | 0.318182 | 0 | 0.153333 | 0.118182 | | 0.136364 | 0 | 0.3 |
| 0 | 0 | 0 | 0 | 0.218182 | 0.190909 | 0.227273 | 0.127273 | 0.372727 | 0.1 | 0.354545 | 0.327273 | 0.118182 | 0.1 | 0 | 0.063636 | 0 | 0.093333 | 0 | | 0.390909 | 0.190909 | 0.172727 |
| 0.363636 | 0 | 0 | 0.372727 | 0.327273 | 0.327273 | 0 | 0 | 0 | 0.045455 | 0.1 | 0 | 0.172727 | 0.127273 | 0.127273 | 0 | 0.654545 | 0 | 0.190909 | | 0.190909 | 0.1 | 0 |
| 0 | 0.445455 | 0 | 0.181818 | 0 | 0.290909 | 0 | 0.018182 | 0.227273 | 0.172727 | 0.118182 | 0.536364 | 0.336364 | 0 | 0 | 0.154545 | 0 | 0.1 | 0.263636 | | 0.236364 | 0.290909 | 0 |
| 0.054545 | 0.190909 | 0 | 0 | 0.190909 | 0 | 0.172727 | 0.109091 | 0 | 0 | 0 | 0.118182 | 0.372727 | 0 | 0.218182 | 0.045455 | 0.309091 | 0.063333 | 0 | | 0.172727 | 0 | 0.227273 |
| 0.036364 | 0 | 0.009091 | 0.2 | 0 | 0.418182 | 0.127273 | 0 | 0.009091 | 0.072727 | 0.036364 | 0 | 0 | 0.127273 | 0 | 0 | 0 | 0 | 0 | | 0 | 0.009091 | 0 |
| 0.290909 | 0.163636 | 0.018182 | 0.245455 | 0.154545 | 0.354545 | 0 | 0.063636 | 0.1 | 0.054545 | 0.136364 | 0.054545 | 0 | 0.009091 | | | 0.345455 | 0.096667 | 0 | | 0.109091 | 0.2 | 0.045455 |
| 0.163636 | 0.263636 | 0.609091 | 0 | 0.227273 | 0 | 0.2 | 0 | 0.118182 | 0.1 | 0.136364 | 0.263636 | 0.263636 | | | | 0 | 0 | 0.118182 | | 0.236364 | 0.018182 | 0 |
| 0 | 0.136364 | 0.154545 | 0.327273 | 0 | 0.409091 | 0 | 0 | 0.181818 | 0.036364 | 0 | 0.081818 | 0 | | | | 0.163636 | 0.093333 | 0 | | 0 | 0.309091 | 0.345455 |
| 0.290909 | 0.281818 | 0.372727 | 0 | 0 | 0.054545 | 0.090909 | 0.090909 | 0.009091 | 0.054545 | 0.190909 | 0.154545 | 0 | | | | 0.154545 | 0.06 | 0.145455 | | 0.3 | 0.018182 | 0 |
| 0 | 0.218182 | 0 | 0.354545 | 0.245455 | 0 | 0.190909 | 0 | 0.263636 | 0.254545 | 0.127273 | 0.3 | 0.345455 | | | | 0.045455 | 0.056667 | 0 | | 0.190909 | 0.318182 | 0.463636 |
| 0.236364 | 0.090909 | 0.145455 | 0 | 0.363636 | 0.254545 | 0.272727 | 0.109091 | 0.136364 | 0.154545 | 0.118182 | 0 | 0 | | | | 0 | 0.076667 | 0.263636 | | 0.163636 | 0 | 0 |
| 0.254545 | 0.209091 | 0 | 0.427273 | 0 | 0.236364 | 0.3 | 0.2 | 0.027273 | 0.018182 | 0.263636 | 0.318182 | 0.063636 | | | | 0.381818 | 0 | 0.190909 | | 0 | 0.318182 | 0.4 |
| 0.318182 | 0.118182 | 0.581818 | 0.263636 | 0.245455 | 0.172727 | 0.3 | 0.418182 | 0.409091 | 0.327273 | 0.254545 | 0.172727 | 0.445455 | | | | 0.245455 | 0.16 | 0.272727 | | 0.327273 | 0 | 0.390909 |
| 0.227273 | 0.218182 | 0 | 0 | 0.363636 | 0.363636 | 0.4 | 0.172727 | 0.281818 | 0.245455 | 0.536364 | 0.5 | 0.463636 | | | | 0.354545 | 0.17 | 0.309091 | | 0.181818 | 0.409091 | 0.018182 |
| 0.381818 | 0.227273 | 0.463636 | 0.036364 | 0.345455 | 0.327273 | 0.709091 | 0.236364 | 0.727273 | 0.454545 | 0.263636 | 0.236364 | 0 | | | | 0.3 | 0.033333 | 0.272727 | | 0.245455 | 0.290909 | 0.554545 |
| 0 | 0.163636 | 0 | 0.4 | 0.190909 | 0.163636 | 0.509091 | 0.536364 | 0 | 0.245455 | 0.163636 | 0.7 | 0.218182 | | | | 0.190909 | 0 | 0.363636 | | 0.3 | 0.209091 | 0 |
| 0.309091 | 0.245455 | 0.263636 | 0.227273 | 0.781818 | 0.390909 | 0.009091 | 0.372727 | 0.154545 | 0.081818 | 0.4 | 0 | 0.272727 | | | | 0.236364 | 0.063333 | 0.145455 | | 0.027273 | 0.245455 | 0.345455 |
| 0 | 0 | 0.181818 | 0.436364 | 0.318182 | 0.290909 | 0.163636 | 0.127273 | 0.118182 | 0.581818 | 0.036364 | 0.227273 | 0.181818 | | | | 0.536364 | 0.013333 | 0.045455 | | 0.209091 | 0.1 | 0 |
| 0.354545 | 0.427273 | 0.218182 | 0 | 0.054545 | 0.118182 | 0.590909 | 0.245455 | 0.172727 | 0.009091 | 0.163636 | 0.227273 | 0.090909 | | | | 0.2 | 0.106667 | 0.2 | | 0.254545 | 0.027273 | 0.4 |
| 0 | 0.018182 | 0 | 0 | 0.727273 | 0.327273 | 0.236364 | 0.018182 | 0 | 0.181818 | 0.027273 | 0 | 0 | | | | 0 | 0 | 0.263636 | | 0.045455 | 0.336364 | 0 |
| 0 | 0.181818 | 0 | 0 | 0 | 0.181818 | 0.190909 | 0.309091 | 0.309091 | 0.281818 | 0.272727 | 0 | 0.190909 | | | | 0.109091 | 0 | 0.081818 | | 0.263636 | 0 | 0.318182 |
| 0.309091 | 0.245455 | 0 | 0.181818 | 0.109091 | 0.354545 | 0.145455 | 0 | 0.290909 | 0.045455 | 0.327273 | 0.109091 | 0.027273 | | | | 0.3 | 0 | 0.081818 | | 0.309091 | 0.327273 | 0.009091 |
| 0 | 0.118182 | 0.2 | 0 | 0 | 0.381818 | 0.172727 | 0.2 | 0.118182 | 0.145455 | 0 | 0.172727 | 0.254545 | | | | 0.227273 | 0.056667 | 0.009091 | | 0.127273 | 0 | 0.036364 |
| 0 | 0.227273 | 0 | 0.209091 | 0.172727 | 0 | 0.263636 | 0 | 0.290909 | 0 | 0.081818 | 0.3 | 0.390909 | | | | 0.281818 | 0.11 | 0.227273 | | 0 | 0 | 0 |
| 0.190909 | 0.118182 | 0.218182 | 0.027273 | 0.163636 | 0.109091 | 0.090909 | 0 | 0.072727 | 0.318182 | 0.109091 | 0.063636 | 0.009091 | | | | 0.209091 | 0 | 0 | | 0.127273 | 0.254545 | 0.290909 |
| 0 | 0.109091 | 0.181818 | 0.136364 | 0.018182 | 0.290909 | 0.2 | 0.054545 | 0 | 0.018182 | 0 | 0.227273 | 0.090909 | | | | 0.027273 | 0.14 | 0.081818 | | 0.163636 | 0 | 0 |
| 0 | 0 | 0.090909 | 0 | 0 | 0.154545 | 0 | 0.018182 | 0 | 0.118182 | 0 | 0 | 0.209091 | | | | 0.090909 | 0.083333 | 0 | | 0.136364 | 0 | 0 |
| 0.154545 | 0.263636 | 0 | 0.163636 | 0.354545 | 0 | 0.2 | 0 | 0.009091 | 0.009091 | 0.1 | 0 | 0 | | | | 0.063636 | 0.066667 | | | 0.181818 | 0.309091 | 0.372727 |
| | | 0.109091 | 0 | | | | 0.027273 | 0 | 0.036364 | 0.172727 | | | | | | | 0 | | | 0.090909 | | |
| 0.145455 | 0 | 0.163636 | 0.209091 | 0 | 0.12 | 0.126667 | 0.1 | 0 | 0.345455 | 0.236364 | 0 | 0.363636 | 0.345455 | 0.181818 | 0.263636 | 0 | 0.054545 | 0.172727 | | 0.127273 | 0.263636 | 0.4 |
| 0.154545 | 0.309091 | 0.063636 | 0.072727 | 0 | 0 | 0.036667 | 0.109091 | 0.345455 | 0.345455 | 0.163636 | 0.418182 | 0.427273 | 0.1 | 0.263636 | 0.681818 | 0.090909 | 0.309091 | 0.227273 | | 0.354545 | 0.390909 | 0 |
| 0.272727 | 0.090909 | 0.263636 | 0.290909 | 0.073333 | 0.173333 | 0.116667 | 0.190909 | 0.409091 | 0.245455 | 0.454545 | 0 | 0.027273 | 0.045455 | 0.272727 | 0.372727 | 0.409091 | 0.572727 | 0.1 | | 0.209091 | 0.281818 | 0.218182 |
| 0.345455 | 0.463636 | 0.218182 | 0.318182 | 0.203333 | 0.046667 | 0.11 | 0.345455 | 0 | 0.136364 | 0.109091 | 0.254545 | 0.318182 | 0.254545 | 0.163636 | 0 | 0.236364 | 0.027273 | 0.290909 | | 0.145455 | 0 | 0.009091 |
| 0 | 0 | 0.009091 | 0 | 0.15 | 0 | 0.05 | 0.481818 | 0.4 | 0.263636 | 0.372727 | 0.354545 | 0.036364 | 0.218182 | 0.254545 | 0.136364 | 0.481818 | 0.4 | 0.345455 | | 0.109091 | 0.190909 | 0.472727 |
| 0.181818 | 0.209091 | 0.145455 | 0.109091 | 0.166667 | 0.093333 | 0.096667 | 0.154545 | 0 | 0.018182 | 0 | 0.318182 | 0.063636 | 0.236364 | 0.418182 | 0 | 0.236364 | 0.027273 | 0.263636 | | 0.118182 | 0.236364 | 0 |
| 0.436364 | 0.227273 | 0.081818 | 0.172727 | 0.15 | 0 | 0.143333 | 0.245455 | 0.345455 | 0.054545 | 0.054545 | 0 | 0.081818 | 0.090909 | 0 | 0.418182 | 0.236364 | 0.163636 | 0.063636 | | 0.172727 | 0 | 0 |
| 0.009091 | 0.045455 | 0.227273 | 0 | 0.116667 | 0.04 | 0.09 | 0.218182 | 0.081818 | 0.063636 | 0.145455 | 0.345455 | 0.045455 | 0.236364 | 0.190909 | 0.063636 | 0.372727 | 0.145455 | 0.109091 | | 0.490909 | 0.009091 | 0.490909 |
| 0 | 0.045455 | 0.009091 | 0.163636 | 0.123333 | 0.166667 | 0 | 0.127273 | 0 | 0.154545 | 0.109091 | 0 | 0 | 0.009091 | 0 | 0.090909 | 0 | 0.272727 | 0 | | 0.172727 | 0.1 | 0.363636 |
| 0 | 0.136364 | 0 | 0 | 0 | 0 | 0.086667 | 0.190909 | 0.118182 | 0.027273 | 0.118182 | 0.290909 | 0.236364 | 0.009091 | 0.418182 | 0.590909 | 0.063636 | 0 | 0.245455 | | 0 | 0.181818 | 0.227273 |
| 0.181818 | 0.209091 | 0.109091 | 0.1 | 0 | 0.126667 | 0.106667 | 0.209091 | 0.590909 | 0 | 0.190909 | 0 | 0.245455 | 0.036364 | 0 | 0.009091 | 0.145455 | 0.218182 | 0.209091 | | 0.018182 | 0.127273 | 0.036364 |
| 0.136364 | 0.036364 | 0 | 0.218182 | 0 | 0.043333 | 0 | 0.145455 | 0 | 0.527273 | 0.190909 | 0.318182 | 0.3 | 0.154545 | 0.309091 | 0.036364 | 0 | 0 | 0.254545 | | 0.454545 | 0.327273 | 0 |
| 0.2 | 0.172727 | 0.345455 | 0.018182 | 0.053333 | 0.103333 | 0.006667 | 0.009091 | 0.090909 | 0.190909 | 0.172727 | 0.363636 | 0 | 0.045455 | 0.018182 | 0 | 0.081818 | 0.3 | 0.281818 | | 0.281818 | 0.109091 | 0 |
| 0 | 0.045455 | 0 | 0.090909 | 0.03 | 0 | 0.123333 | 0.136364 | 0.163636 | 0.218182 | 0.081818 | 0.245455 | 0.2 | 0.036364 | 0 | 0.227273 | 0.227273 | 0 | 0.127273 | | 0.090909 | 0.127273 | 0 |
| 0.018182 | 0.3 | 0.327273 | 0.245455 | 0.163333 | 0 | 0.063333 | 0.045455 | 0.090909 | 0.018182 | 0.154545 | 0.2 | 0.081818 | 0.263636 | 0 | 0.036364 | 0.418182 | 0.290909 | 0.2 | | 0.127273 | 0 | 0.563636 |
| 0.154545 | 0 | 0 | 0.154545 | 0 | 0.123333 | 0.073333 | 0.163636 | 0.227273 | 0.245455 | 0.145455 | 0 | 0.236364 | 0.118182 | 0 | 0.181818 | 0.009091 | 0.354545 | 0.045455 | | 0.172727 | 0.318182 | 0 |
| 0 | 0.263636 | 0.218182 | 0 | 0.13 | 0.066667 | 0.086667 | 0 | 0.009091 | 0.190909 | 0.081818 | 0.190909 | 0.109091 | 0.109091 | 0.281818 | 0.236364 | 0.009091 | 0.2 | 0.127273 | | 0.127273 | 0.263636 | 0 |
| 0 | 0.281818 | 0 | 0.136364 | 0 | 0.07 | 0.07 | 0.1 | 0.018182 | 0.109091 | 0.036364 | 0.090909 | 0.081818 | 0 | 0.036364 | 0 | 0.009091 | 0.036364 | 0.227273 | | 0.136364 | 0 | 0.672727 |
| 0.181818 | 0.063636 | 0.209091 | 0 | 0.113333 | 0.07 | 0 | 0.027273 | 0 | 0.027273 | 0.009091 | 0.190909 | 0.236364 | 0.2 | 0 | 0.027273 | 0.036364 | 0 | 0 | | 0.190909 | 0.290909 | 0 |
| 0.345455 | 0.445455 | 0.009091 | 0.427273 | 0 | 0.04 | 0.08 | 0.1 | 0.1 | 0.163636 | 0.181818 | 0.245455 | 0.381818 | 0.172727 | 0 | 0.318182 | 0.018182 | 0.063636 | 0.045455 | | 0.136364 | 0.254545 | 0.154545 |
| 0.472727 | 0 | 0.272727 | 0.309091 | 0.113333 | 0.05 | 0 | 0 | 0.209091 | 0.336364 | 0.327273 | 0.527273 | 0.018182 | 0.2 | 0.245455 | 0.118182 | 0.036364 | 0.118182 | 0.054545 | | 0.072727 | 0.3 | 0 |
| 0 | 0.127273 | 0.272727 | 0.336364 | 0 | 0.133333 | 0.136667 | 0.218182 | 0.254545 | 0.1 | 0.318182 | 0 | 0.627273 | 0.345455 | 0 | 0.009091 | 0.036364 | 0.045455 | 0.136364 | | 0.136364 | 0 | 0.5 |
| 0.518182 | 0 | 0 | 0.254545 | 0.16 | 0 | 0.116667 | 0.2 | 0.018182 | 0.154545 | 0.181818 | 0 | 0 | 0.227273 | 0.272727 | 0.472727 | 0.072727 | 0.118182 | 0.263636 | | 0.2 | 0.672727 | 0.463636 |
| 0.118182 | 0.190909 | 0.527273 | 0 | 0.056667 | 0.016667 | 0 | 0.372727 | 0.227273 | 0.136364 | 0.227273 | 0.436364 | 0.345455 | 0.3 | 0.481818 | 0 | 0.081818 | 0.154545 | 0.009091 | | 0.136364 | 0 | 0 |
| 0 | 0.272727 | 0 | 0.3 | 0 | 0.14 | 0.026667 | 0.327273 | 0.054545 | 0.427273 | 0.290909 | 0.409091 | 0 | 0.5 | 0.327273 | 0.427273 | 0.127273 | 0.109091 | 0.154545 | | 0.4 | 0.018182 | 0.427273 |
| 0.418182 | 0.154545 | 0.372727 | 0.154545 | 0.176667 | 0.063333 | 0.15 | 0.154545 | 0.481818 | 0.209091 | 0.009091 | 0.490909 | 0.136364 | 0.009091 | 0 | 0.390909 | 0.045455 | 0.172727 | 0.381818 | | 0.018182 | 0.354545 | 0 |
| 0 | 0.081818 | 0 | 0.036364 | 0.15 | 0.096667 | 0.093333 | 0.318182 | 0.190909 | 0.281818 | 0.390909 | 0 | 0.109091 | 0.009091 | 0.218182 | 0.390909 | 0.018182 | 0.227273 | 0.127273 | | 0.2 | 0.272727 | 0 |
| 0 | 0.063636 | 0.427273 | 0.109091 | 0.136667 | 0.06 | 0.116667 | 0.154545 | 0.209091 | 0.154545 | 0.027273 | 0 | 0 | 0 | 0.254545 | 0 | 0.109091 | 0.145455 | 0.436364 | | 0.081818 | 0 | 0.418182 |
| 0.363636 | 0.209091 | 0 | 0 | 0 | 0.123333 | 0 | 0.263636 | 0.209091 | 0.209091 | 0.072727 | 0.309091 | 0.127273 | 0.281818 | 0 | 0 | 0.109091 | 0.290909 | 0.290909 | | 0.109091 | 0.209091 | 0 |
| 0.009091 | 0.109091 | 0 | 0 | 0.14 | 0.043333 | 0.083333 | 0.081818 | 0 | 0 | 0.063636 | 0.1 | 0.172727 | 0.363636 | 0.172727 | 0.4 | 0.090909 | 0.045455 | 0.118182 | | 0.172727 | 0.372727 | 0 |
| 0 | 0 | 0 | 0 | 0 | 0 | 0.24 | 0.045455 | 0 | 0.027273 | 0 | 0.3 | 0.154545 | 0.309091 | 0.072727 | 0 | 0.027273 | 0.163636 | 0.018182 | | 0 | 0 | 0.681818 |
| 0 | 0.190909 | 0 | 0 | 0 | 0.023333 | 0 | 0.054545 | 0.718182 | 0.3 | 0.072727 | 0 | 0.109091 | 0.4 | 0.290909 | 0 | 0.054545 | 0.145455 | 0.245455 | | 0.009091 | 0.172727 | 0 |
| 0.136364 | 0.072727 | 0.5 | 0 | 0 | 0 | 0.136667 | 0.245455 | 0 | 0.263636 | 0.209091 | 0.163636 | 0.118182 | 0 | 0.345455 | 0.163636 | 0 | 0.218182 | 0.054545 | | 0.272727 | 0 | 0.236364 |
| 0.2 | 0.127273 | 0.027273 | 0.054545 | 0 | 0.053333 | 0.113333 | 0.045455 | 0 | 0 | 0.172727 | 0.290909 | 0.3 | 0.254545 | 0 | 0.209091 | 0.154545 | 0.509091 | 0.418182 | | 0.145455 | 0.254545 | 0 |
| 0.027273 | 0.236364 | 0.009091 | 0.154545 | 0.096667 | 0.05 | 0 | 0.245455 | 0.527273 | 0 | 0.227273 | 0.009091 | 0.381818 | 0 | 0.272727 | 0 | 0 | 0.6 | 0 | | 0.036364 | 0 | 0 |
| 0.1 | 0 | 0 | 0.009091 | 0 | 0 | 0.076667 | 0.090909 | 0.009091 | 0.345455 | 0.136364 | 0.054545 | 0.218182 | 0 | 0.009091 | 0.045455 | 0.072727 | 0 | 0.345455 | | 0.363636 | 0 | 0 |
| 0.036364 | 0.336364 | 0.263636 | 0.154545 | 0.09 | 0.023333 | 0.073333 | 0.063636 | 0.081818 | 0.163636 | 0.081818 | 0 | 0 | 0.018182 | 0.427273 | 0 | 0.227273 | 0 | 0.181818 | | 0.036364 | 0.036364 | 0.063636 |
| 0 | 0 | 0 | 0 | 0.023333 | 0.123333 | 0.003333 | 0.163636 | 0.181818 | 0.281818 | 0.281818 | 0 | 0.272727 | 0 | 0 | 0.145455 | 0.181818 | 0 | 0.072727 | | 0.263636 | 0 | 0 |
| 0.181818 | 0.263636 | 0 | 0.227273 | 0.06 | 0 | 0.05 | 0.054545 | 0.336364 | 0.063636 | 0.109091 | 0 | 0.054545 | 0 | 0.009091 | 0.027273 | 0.254545 | 0.072727 | 0 | | 0.127273 | 0 | 0 |

| | | | | | | | | | | | | | | | | | | | | | |
|---|---|---|---|---|---|---|---|---|---|---|---|---|---|---|---|---|---|---|---|---|---|
| 0 | 0 | 0.390909 | 0 | 0 | 0 | 0 | 0.218182 | 0.009091 | 0 | 0.145455 | 0 | 0.2 | 0.009091 | | | 0.009091 | 0.127273 | 0.181818 | 0 | 0.027273 | 0 |
| 0.109091 | 0.354545 | 0.009091 | 0.009091 | 0 | 0.063333 | 0 | 0.172727 | 0.009091 | 0.063636 | 0.090909 | 0.118182 | 0 | | | | 0 | 0.036364 | 0.118182 | 0.227273 | 0.009091 | 0.218182 |
| 0 | 0.318182 | 0 | 0.263636 | 0.236667 | 0 | 0.146667 | 0.054545 | 0.381818 | 0.163636 | 0.263636 | 0.445455 | 0.618182 | | | | 0.5 | 0.009091 | 0.009091 | 0.1 | 0.5 | 0.618182 |
| 0.327273 | 0.372727 | 0.472727 | 0 | 0 | 0.21 | 0 | 0.336364 | 0 | 0.290909 | 0.345455 | 0.4 | 0.4 | | | | 0 | 0.190909 | 0 | 0.272727 | 0.427273 | 0.736364 |
| 0.563636 | 0 | 0.009091 | 0.545455 | 0.23 | 0 | 0.09 | 0 | 0 | 0.181818 | 0 | 0 | 0.209091 | | | | 0 | 0.3 | 0.481818 | 0.227273 | 0.481818 | 0 |
| 0 | 0.281818 | 0.518182 | 0.336364 | 0 | 0.163333 | 0 | 0.163636 | 0.036364 | 0.363636 | 0.181818 | 0.472727 | 0 | | | | 0 | 0.009091 | 0 | 0.081818 | 0 | 0 |
| 0.327273 | 0.009091 | 0 | 0.554545 | 0.163333 | 0 | 0.283333 | 0.363636 | 0.109091 | 0.172727 | 0.4 | 0.445455 | 0.018182 | | | | 0.054545 | 0.363636 | 0 | 0.4 | 0.454545 | 0.054545 |
| 0 | 0 | 0.745455 | 0 | 0.026667 | 0.126667 | 0 | 0.372727 | 0.345455 | 0.481818 | 0.290909 | 0 | 0.472727 | | | | 0.463636 | 0.245455 | 0.518182 | 0.290909 | 0.036364 | 0.672727 |
| 0 | 0 | 0 | 0.509091 | 0 | 0 | 0.15 | 0.263636 | 0 | 0.1 | 0 | 0.381818 | 0 | | | | 0 | 0.236364 | 0.327273 | 0 | 0.027273 | 0.227273 |
| 0.463636 | 0.327273 | 0 | 0.009091 | 0.2 | 0 | 0.216667 | 0.190909 | 0.2 | 0.063636 | 0 | 0.118182 | 0 | | | | 0 | 0.045455 | 0.018182 | 0.172727 | 0.081818 | 0 |
| 0 | 0 | 0.2 | 0 | 0 | 0.19 | 0 | 0.009091 | 0.009091 | 0.054545 | 0.190909 | 0 | 0 | | | | 0.127273 | 0.454545 | 0 | 0.063636 | 0 | 0 |
| 0 | 0.181818 | 0.009091 | 0 | 0 | 0.02 | 0 | 0.2 | 0.027273 | 0.018182 | 0 | 0.372727 | 0.381818 | | | | 0.263636 | 0.036364 | 0.227273 | 0.127273 | 0.227273 | 0 |
| 0.163636 | 0.227273 | 0.154545 | 0 | 0 | 0.083333 | 0.14 | 0.009091 | 0.263636 | 0.145455 | 0.218182 | 0.154545 | 0.018182 | | | | 0.1 | 0.209091 | 0 | 0.227273 | 0 | 0 |
| 0 | 0.027273 | 0 | 0 | 0.156667 | 0 | 0 | 0.072727 | 0.127273 | 0.063636 | 0.045455 | 0 | 0.045455 | | | | 0.127273 | 0.763636 | 0.3 | 0 | 0.172727 | 0.1 |
| 0 | 0 | 0 | 0 | 0.003333 | 0 | 0 | 0.072727 | 0 | 0 | 0.3 | 0 | 0.363636 | | | | 0.009091 | 0.009091 | 0.036364 | 0.045455 | 0 | 0.454545 |
| 0.090909 | 0 | 0.172727 | 0 | 0 | 0 | 0.086667 | 0.036364 | 0.3 | 0.109091 | 0.009091 | 0.290909 | 0.009091 | | | | 0.254545 | 0.254545 | 0.009091 | 0.009091 | 0.081818 | 0 |
| 0 | 0.136364 | 0.181818 | 0.281818 | 0.05 | 0 | 0.003333 | 0.127273 | 0.1 | 0.009091 | 0.154545 | 0.027273 | 0.127273 | | | | 0.554545 | 0.018182 | 0.045455 | 0.245455 | 0 | 0 |
| 0 | 0 | 0.018182 | 0 | 0.17 | 0.01 | 0.086667 | 0 | 0.254545 | 0.218182 | 0.1 | 0.163636 | 0.190909 | | | | 0.009091 | 0.127273 | 0.236364 | 0.118182 | 0 | 0.072727 |
| 0.418182 | 0.072727 | 0.136364 | 0 | 0 | 0 | 0.123333 | 0.227273 | 0 | 0 | 0.227273 | 0.263636 | 0.1 | | | | 0 | 0.027273 | 0.172727 | 0.036364 | 0.172727 | 0.490909 |
| 0 | 0.109091 | 0 | 0 | 0.003333 | 0.036667 | 0 | 0.154545 | 0 | 0.127273 | 0.136364 | 0 | 0.263636 | | | | 0.009091 | 0.081818 | 0.009091 | 0.081818 | 0.127273 | 0 |
| 0.009091 | 0 | 0.009091 | 0.036364 | 0.043333 | 0.083333 | 0 | 0 | 0.163636 | 0.245455 | 0 | 0.090909 | 0 | | | | 0.109091 | 0.163636 | 0.090909 | 0.109091 | 0 | 0 |
| 0 | 0.163636 | 0.018182 | 0 | 0 | 0.05 | 0.153333 | 0.090909 | 0.045455 | 0.063636 | 0.145455 | | | | | | 0 | 0.027273 | 0 | 0.163636 | | |

| | | | | | | | | | | | | | | | | | | | | | |
|---|---|---|---|---|---|---|---|---|---|---|---|---|---|---|---|---|---|---|---|---|---|
| 0.227273 | 0.290909 | 0.290909 | 0.163636 | 0.136364 | 0.209091 | 0.027273 | 0.236364 | 0.154545 | 0.163636 | 0.090909 | 0.227273 | 0.136364 | 0.190909 | 0.172727 | 0.172727 | 0 | 0.263636 | 0.154545 | 0.354545 | 0.290909 | 0.372727 |
| 0.363636 | 0.136364 | 0.181818 | 0.3 | 0.254545 | 0 | 0.209091 | 0.127273 | 0.136364 | 0.154545 | 0.163636 | 0.209091 | 0.263636 | 0.236364 | 0.090909 | 0.327273 | 0.118182 | 0.345455 | 0.2 | 0 | 0.2 | 0.2 |
| 0.009091 | 0.154545 | 0.209091 | 0.136364 | 0.245455 | 0.381818 | 0.190909 | 0.254545 | 0.336364 | 0.545455 | 0.254545 | 0.209091 | 0.263636 | 0.418182 | 0.290909 | 0.3 | 0.454545 | 0.172727 | 0.236364 | 0.190909 | 0.209091 | 0.345455 |
| 0.190909 | 0.2 | 0.2 | 0.245455 | 0 | 0.145455 | 0 | 0.290909 | 0.190909 | 0.145455 | 0.136364 | 0.245455 | 0.236364 | 0 | 0.227273 | 0.236364 | 0.009091 | 0.127273 | 0.1 | 0.290909 | 0 | 0.436364 |
| 0 | 0.009091 | 0.272727 | 0.209091 | 0.490909 | 0 | 0.190909 | 0 | 0.218182 | 0 | 0 | 0 | 0.009091 | 0.2 | 0.154545 | 0.181818 | 0.154545 | 0.027273 | 0.190909 | 0.163636 | 0.281818 | 0 |
| 0.109091 | 0.145455 | 0.009091 | 0 | 0 | 0.063636 | 0.009091 | 0.054545 | 0.118182 | 0.227273 | 0.145455 | 0.245455 | 0.163636 | 0 | 0.236364 | 0 | 0.154545 | 0.281818 | 0 | 0.145455 | 0.272727 | 0.072727 |
| 0 | 0.090909 | 0.118182 | 0.227273 | 0.418182 | 0.027273 | 0.454545 | 0.127273 | 0.227273 | 0.036364 | 0.263636 | 0.118182 | 0.290909 | 0.163636 | 0.027273 | 0.418182 | 0.263636 | 0.009091 | 0.109091 | 0.190909 | 0 | 0.236364 |
| 0.2 | 0.154545 | 0.009091 | 0.018182 | 0 | 0.163636 | 0 | 0.227273 | 0.090909 | 0.1 | 0.145455 | 0.1 | 0.009091 | 0.127273 | 0.218182 | 0 | 0.009091 | 0.263636 | 0.2 | 0.145455 | 0 | 0.136364 |
| 0 | 0.027273 | 0 | 0.2 | 0 | 0 | 0 | 0 | 0 | 0.172727 | 0.045455 | 0 | 0.136364 | 0.181818 | 0.063636 | 0.136364 | 0 | 0.045455 | 0 | 0 | 0.054545 | 0.254545 |
| 0 | 0.045455 | 0.118182 | 0.109091 | 0 | 0.309091 | 0.245455 | 0.163636 | 0 | 0 | 0.109091 | 0.281818 | 0.054545 | 0 | 0.1 | 0.009091 | 0 | 0.118182 | 0.190909 | 0.272727 | 0.245455 | 0 |
| 0 | 0.009091 | 0 | 0 | 0 | 0 | 0 | 0.136364 | 0.2 | 0.1 | 0.136364 | 0.045455 | 0.1 | 0 | 0 | 0.118182 | 0 | 0.145455 | 0.127273 | 0 | 0 | 0 |
| 0.118182 | 0.236364 | 0.236364 | 0 | 0.136364 | 0.318182 | 0.090909 | 0.109091 | 0.2 | 0.227273 | 0.081818 | 0.081818 | 0.181818 | 0.036364 | 0 | 0.109091 | 0.118182 | 0.145455 | 0.109091 | 0.236364 | 0.009091 | 0.263636 |
| 0 | 0 | 0.090909 | 0.109091 | 0 | 0 | 0 | 0.2 | 0 | 0.372727 | 0 | 0.163636 | 0.2 | 0.109091 | 0.081818 | 0.136364 | 0.072727 | 0.245455 | 0.081818 | 0.354545 | 0.281818 | 0.009091 |
| 0.090909 | 0.318182 | 0.154545 | 0.145455 | 0.081818 | 0.118182 | 0.036364 | 0.181818 | 0.227273 | 0.2 | 0 | 0.009091 | 0.009091 | 0.190909 | 0.190909 | 0.090909 | 0.081818 | 0.163636 | 0.118182 | 0.063636 | 0 | 0 |
| 0.072727 | 0 | 0 | 0 | 0 | 0 | 0.2 | 0.054545 | 0.136364 | 0 | 0.118182 | 0.109091 | 0.218182 | 0 | 0.1 | 0.1 | 0 | 0.109091 | 0 | 0.109091 | 0.281818 | 0.072727 |
| 0.263636 | 0 | 0 | 0.218182 | 0.181818 | 0.009091 | 0 | 0.072727 | 0.009091 | 0.154545 | 0.136364 | 0 | 0.009091 | 0.118182 | 0.063636 | 0.045455 | 0.372727 | 0.009091 | 0.090909 | 0.127273 | 0 | 0.009091 |
| 0 | 0.009091 | 0.063636 | 0 | 0 | 0.027273 | 0 | 0.036364 | 0.145455 | 0 | 0.045455 | 0.109091 | 0.009091 | 0.136364 | 0.036364 | 0.090909 | 0.127273 | 0.054545 | 0 | 0 | 0.236364 | 0 |
| 0.054545 | 0 | 0.227273 | 0.118182 | 0.309091 | 0.3 | 0.236364 | 0 | 0.118182 | 0.045455 | 0.1 | 0.154545 | 0.145455 | 0.118182 | 0.081818 | 0.136364 | 0 | 0.4 | 0.190909 | 0.027273 | 0.009091 | 0 |
| 0.163636 | 0.127273 | 0 | 0.1 | 0 | 0.081818 | 0 | 0.109091 | 0.090909 | 0 | 0.045455 | 0.3 | 0.118182 | 0.190909 | 0.072727 | 0.018182 | 0.136364 | 0 | 0.081818 | 0.081818 | 0.272727 | 0.3 |
| 0 | 0.154545 | 0.518182 | 0 | 0.009091 | 0.181818 | 0.227273 | 0.136364 | 0.454545 | 0 | 0.072727 | 0 | 0.118182 | 0.154545 | 0.154545 | 0.336364 | 0.018182 | 0.063636 | 0 | 0.290909 | 0 | 0 |
| 0.227273 | 0.227273 | 0.018182 | 0.545455 | 0.5 | 0 | 0.172727 | 0.1 | 0 | 0.072727 | 0.227273 | 0.327273 | 0.190909 | 0.181818 | 0.336364 | 0.118182 | 0.136364 | 0 | 0.1 | 0.190909 | 0.418182 | 0.318182 |
| 0.381818 | 0.090909 | 0.181818 | 0.218182 | 0.009091 | 0.363636 | 0.227273 | 0.254545 | 0.181818 | 0.290909 | 0.163636 | 0 | 0.190909 | 0.063636 | 0 | 0.309091 | 0.318182 | 0.3 | 0.218182 | 0.009091 | 0.009091 | 0.009091 |
| 0.290909 | 0.363636 | 0.209091 | 0 | 0 | 0 | 0.045455 | 0.036364 | 0.2 | 0.181818 | 0.072727 | 0.263636 | 0.154545 | 0.209091 | 0 | 0.254545 | 0 | 0.172727 | 0.181818 | 0.027273 | 0.345455 | 0.2 |
| 0.181818 | 0.018182 | 0.063636 | 0 | 0.454545 | 0.309091 | 0.254545 | 0.445455 | 0.009091 | 0.109091 | 0.136364 | 0.254545 | 0.290909 | 0.027273 | 0.090909 | 0.418182 | 0.209091 | 0.272727 | 0.181818 | 0.154545 | 0.018182 | 0.272727 |
| 0.009091 | 0 | 0.145455 | 0.372727 | 0.236364 | 0.1 | 0.081818 | 0.081818 | 0.236364 | 0.254545 | 0.209091 | 0.136364 | 0.190909 | 0.281818 | 0.054545 | 0 | 0.145455 | 0.4 | 0.163636 | 0.290909 | 0.381818 | 0.009091 |
| 0.263636 | 0.118182 | 0.072727 | 0 | 0 | 0.090909 | 0.172727 | 0.2 | 0.054545 | 0.263636 | 0.081818 | 0 | 0.045455 | 0.072727 | 0.2 | 0.254545 | 0.218182 | 0.009091 | 0.263636 | 0.436364 | 0 | 0.354545 |
| 0.109091 | 0.181818 | 0 | 0.181818 | 0.2 | 0.009091 | 0.209091 | 0.163636 | 0.236364 | 0.181818 | 0.236364 | 0.209091 | 0 | 0.154545 | 0.190909 | 0.063636 | 0 | 0.009091 | 0.090909 | 0 | 0.009091 | 0 |
| 0.181818 | 0.236364 | 0.309091 | 0.118182 | 0.272727 | 0.418182 | 0 | 0.127273 | 0 | 0.063636 | 0 | 0.236364 | 0.181818 | 0.145455 | 0.063636 | 0.172727 | 0 | 0.163636 | 0.345455 | 0.163636 | 0.163636 | 0 |
| 0 | 0.090909 | 0 | 0.327273 | 0 | 0.081818 | 0.209091 | 0.118182 | 0.127273 | 0.181818 | 0.172727 | 0 | 0.363636 | 0.081818 | 0.227273 | 0 | 0.127273 | 0.090909 | 0.072727 | 0.154545 | 0.154545 | 0.245455 |
| 0 | 0.145455 | 0.127273 | 0.172727 | 0.163636 | 0 | 0.027273 | 0.045455 | 0.054545 | 0.1 | 0.063636 | 0.163636 | 0 | 0.018182 | 0.2 | 0.145455 | 0.272727 | 0.127273 | 0.127273 | 0.172727 | 0.045455 | 0 |
| 0.209091 | 0.109091 | 0.009091 | 0.118182 | 0 | 0.172727 | 0 | 0.072727 | 0.054545 | 0.072727 | 0.181818 | 0.027273 | 0.127273 | 0.145455 | 0 | 0.036364 | 0.072727 | 0.263636 | 0.063636 | 0 | 0.236364 | 0.136364 |
| 0 | 0.036364 | 0.090909 | 0.063636 | 0.1 | 0.136364 | 0.4 | 0 | 0.172727 | 0.181818 | 0.136364 | 0 | 0.172727 | 0.054545 | 0.045455 | 0.027273 | 0 | 0.118182 | 0.318182 | 0.009091 | 0 | 0 |
| 0.2 | 0.009091 | 0 | 0 | 0 | 0.018182 | 0 | 0.1 | 0.163636 | 0.072727 | 0.127273 | 0.190909 | 0.181818 | 0.227273 | 0.063636 | 0.236364 | 0.245455 | 0.245455 | 0.209091 | 0.127273 | 0 | 0.136364 |
| 0.009091 | 0.036364 | 0.172727 | 0.045455 | 0.009091 | 0.118182 | 0.127273 | 0.127273 | 0.163636 | 0.154545 | 0.245455 | 0.081818 | 0.163636 | 0.181818 | 0.063636 | 0.354545 | 0.163636 | 0.1 | 0.218182 | 0.081818 | 0.2 | 0.054545 |
| 0 | 0.154545 | 0.136364 | 0.145455 | 0.336364 | 0.190909 | 0.009091 | 0.154545 | 0.436364 | 0.209091 | 0.009091 | 0.218182 | 0 | 0.190909 | 0.281818 | 0 | 0.009091 | 0.236364 | 0.172727 | 0.063636 | 0.090909 | 0.327273 |
| 0.109091 | 0 | 0.063636 | 0.090909 | 0 | 0.327273 | 0.154545 | 0.2 | 0.009091 | 0.054545 | 0.1 | 0.318182 | 0.190909 | 0.145455 | 0.045455 | 0.145455 | 0.072727 | 0 | 0.236364 | 0 | 0.118182 | 0.190909 |
| 0.109091 | 0.154545 | 0 | 0.072727 | 0.136364 | 0 | 0.009091 | 0.072727 | 0.1 | 0.045455 | 0.118182 | 0 | 0.009091 | 0.190909 | 0.045455 | 0 | 0.145455 | 0.127273 | 0.036364 | 0.172727 | 0.209091 | 0.154545 |
| 0 | 0 | 0.090909 | 0.027273 | 0 | 0.172727 | 0.218182 | 0.009091 | 0.118182 | 0.054545 | 0.072727 | 0.145455 | 0.090909 | 0 | 0 | 0.009091 | 0 | 0.127273 | 0.109091 | 0.090909 | 0.218182 | 0 |
| 0.090909 | 0.109091 | 0.036364 | 0.190909 | 0.136364 | 0.154545 | 0.009091 | 0.054545 | 0.109091 | 0.081818 | 0.081818 | 0.218182 | 0.245455 | 0.145455 | 0.018182 | 0.1 | 0 | 0.027273 | 0 | 0.081818 | 0.109091 | 0.154545 |
| 0.109091 | 0 | 0.2 | 0.090909 | 0.227273 | 0 | 0.127273 | 0.009091 | 0.072727 | 0.781818 | 0.090909 | 0 | 0 | 0.109091 | 0.054545 | 0.290909 | 0.118182 | 0.118182 | 0.027273 | 0.127273 | 0.254545 | 0.209091 |
| 0 | 0.127273 | 0.263636 | 0 | 0.036364 | 0.081818 | 0 | 0.109091 | 0.172727 | 0.172727 | 0.1 | 0.127273 | 0.090909 | 0.009091 | 0.090909 | 0.109091 | 0.218182 | 0.018182 | 0.081818 | 0.054545 | 0 | 0.236364 |
| 0.318182 | 0.2 | 0 | 0.272727 | 0.290909 | 0.163636 | 0.236364 | 0 | 0.327273 | 0.245455 | 0.081818 | 0.218182 | 0.145455 | 0.4 | 0.145455 | 0 | 0 | 0.154545 | 0.118182 | 0.127273 | 0.272727 | 0.009091 |
| 0.118182 | 0.009091 | 0.245455 | 0 | 0.290909 | 0.009091 | 0.163636 | 0.2 | 0.018182 | 0.072727 | 0.072727 | 0.063636 | 0.381818 | 0 | 0.154545 | 0.463636 | 0.272727 | 0.163636 | 0.163636 | 0.109091 | 0 | 0.109091 |
| 0.136364 | 0.381818 | 0 | 0.218182 | 0 | 0.236364 | 0 | 0.109091 | 0.118182 | 0.236364 | 0.336364 | 0.327273 | 0.181818 | 0.190909 | 0.118182 | 0.172727 | 0.163636 | 0.209091 | 0.218182 | 0.345455 | 0.190909 | 0.218182 |
| 0 | 0.009091 | 0.445455 | 0.245455 | 0.136364 | 0.109091 | 0.427273 | 0.136364 | 0.118182 | 0 | 0 | 0.218182 | 0.154545 | 0.190909 | 0.218182 | 0.209091 | 0 | 0.190909 | 0.027273 | 0.118182 | 0.072727 | 0.336364 |
| 0.236364 | 0.018182 | 0.118182 | 0.254545 | 0.018182 | 0.172727 | 0.281818 | 0.254545 | 0.036364 | 0.327273 | 0.309091 | 0.345455 | 0.281818 | 0.018182 | 0.2 | 0.4 | 0.254545 | 0.3 | 0.272727 | 0.118182 | 0.245455 | 0.263636 |
| 0 | 0.218182 | 0 | 0.009091 | 0.227273 | 0.018182 | 0.209091 | 0.281818 | 0.090909 | 0.190909 | 0.063636 | 0.009091 | 0.118182 | 0.163636 | 0.063636 | 0.045455 | 0.145455 | 0.418182 | 0.090909 | 0.290909 | 0.172727 | 0 |
| 0.281818 | 0.127273 | 0.036364 | 0.136364 | 0.372727 | 0.309091 | 0.009091 | 0.1 | 0.227273 | 0.109091 | 0.2 | 0.281818 | 0.072727 | 0.236364 | 0.218182 | 0.236364 | 0.018182 | 0.190909 | 0.218182 | 0.036364 | 0.136364 | 0.345455 |
| 0.172727 | 0.072727 | 0 | 0.036364 | 0.145455 | 0.009091 | 0.181818 | 0.163636 | 0 | 0.136364 | 0.063636 | 0.109091 | 0.190909 | 0 | 0.036364 | 0.118182 | 0.172727 | 0.163636 | 0.136364 | 0.245455 | 0.109091 | 0 |
| 0.009091 | 0.009091 | 0 | 0.036364 | 0.081818 | 0.336364 | 0.163636 | 0.2 | 0.227273 | 0.254545 | 0.181818 | 0.009091 | 0.072727 | 0.154545 | 0.190909 | 0 | 0 | 0.118182 | 0.009091 | 0 | 0.109091 | 0.390909 |
| 0 | 0.090909 | 0 | 0.009091 | 0 | 0.090909 | 0.127273 | 0.118182 | 0.027273 | 0.127273 | 0.018182 | 0.109091 | 0.154545 | 0.090909 | 0.036364 | 0.190909 | 0.263636 | 0.072727 | 0.2 | 0.009091 | 0.090909 | 0.009091 |
| 0.381818 | 0 | 0 | 0.009091 | 0.127273 | 0 | 0.109091 | 0.1 | 0.136364 | 0.045455 | 0.172727 | 0.190909 | 0.090909 | 0.1 | 0.045455 | 0 | 0 | 0.163636 | 0.036364 | 0.190909 | 0.063636 | 0.081818 |
| 0 | 0.1 | 0 | 0 | 0 | 0.054545 | 0.1 | 0.009091 | 0.072727 | 0.154545 | 0.172727 | 0.181818 | 0.081818 | 0 | 0.081818 | 0.236364 | 0.054545 | 0.109091 | 0.236364 | 0.072727 | 0.172727 | 0.072727 |
| 0 | 0.209091 | 0 | 0.318182 | 0.118182 | 0.072727 | 0.209091 | 0.154545 | 0.127273 | 0 | 0.090909 | 0 | 0.054545 | 0.090909 | 0.190909 | 0.1 | 0.345455 | 0.127273 | 0.1 | 0.145455 | 0.027273 | 0 |
| 0.127273 | 0 | 0 | 0.009091 | 0 | 0.009091 | 0.281818 | 0.036364 | 0.109091 | 0.109091 | 0.145455 | 0.272727 | 0.172727 | 0 | 0.045455 | 0.145455 | 0 | 0.427273 | 0.209091 | 0.081818 | 0.045455 | 0.227273 |
| 0.009091 | 0 | 0.027273 | 0 | 0.163636 | 0.327273 | 0.009091 | 0.172727 | 0.145455 | 0.290909 | 0.036364 | 0.018182 | 0.109091 | 0.390909 | 0.118182 | 0.163636 | 0 | 0 | 0.045455 | 0.127273 | 0.154545 | 0 |
| 0.127273 | 0 | 0.109091 | 0.009091 | 0 | 0 | 0.009091 | 0 | 0.009091 | 0.036364 | 0.090909 | 0.236364 | 0 | 0 | 0.145455 | 0.063636 | 0.236364 | 0.136364 | 0.218182 | 0.2 | 0.363636 | 0.363636 |
| 0 | 0.209091 | 0 | 0 | 0.2 | 0.181818 | 0.136364 | 0.181818 | 0.363636 | 0.227273 | 0.027273 | 0.090909 | 0.236364 | 0.172727 | 0.290909 | 0 | 0.127273 | 0.172727 | 0.009091 | 0 | 0.145455 | 0 |
| 0 | 0 | 0.309091 | 0.127273 | 0.018182 | 0.118182 | 0 | 0 | 0.027273 | 0.181818 | 0.054545 | 0.2 | 0.163636 | 0 | 0 | 0.2 | 0 | 0.009091 | 0.236364 | 0.163636 | 0.127273 | 0.290909 |
| 0.254545 | 0.118182 | 0.036364 | 0.1 | 0.154545 | 0 | 0.1 | 0.154545 | 0.118182 | 0.1 | 0.109091 | 0.118182 | 0.2 | 0.127273 | 0.063636 | 0.136364 | 0.181818 | 0.118182 | 0.127273 | 0 | 0.1 | 0.118182 |
| 0 | 0 | 0.018182 | 0 | 0 | 0.118182 | 0.054545 | 0 | 0 | 0.072727 | 0.127273 | 0.163636 | 0.018182 | 0 | | | 0 | 0.009091 | 0 | 0.118182 | 0.145455 | 0.136364 |
| 0.009091 | 0.072727 | 0 | 0.245455 | 0.181818 | 0.018182 | 0.227273 | 0 | 0.136364 | 0.081818 | 0.090909 | 0.163636 | 0.172727 | | | | 0.227273 | 0.090909 | 0.045455 | 0 | 0.081818 | 0 |
| 0.009091 | 0.163636 | 0.181818 | 0.263636 | 0.181818 | 0.172727 | 0.009091 | 0.072727 | 0.318182 | 0.218182 | 0.063636 | 0 | 0 | | | | 0.009091 | 0.081818 | 0.245455 | 0.181818 | 0.145455 | 0.236364 |
| 0.254545 | 0.227273 | 0.218182 | 0.145455 | 0.145455 | 0.1 | 0.272727 | 0.045455 | 0.009091 | 0 | 0.054545 | 0.036364 | 0.118182 | | | | 0.181818 | 0.072727 | 0.063636 | 0.227273 | 0.1 | 0.163636 |
| 0.009091 | 0.063636 | 0 | 0.072727 | 0.018182 | 0.354545 | 0 | 0.118182 | 0 | 0.118182 | 0.145455 | 0.218182 | 0.327273 | | | | 0.372727 | 0.236364 | 0.127273 | 0.145455 | 0.154545 | 0 |
| 0.4 | 0.127273 | 0.254545 | 0.154545 | 0.272727 | 0.190909 | 0.272727 | 0.063636 | 0.081818 | 0.081818 | 0.136364 | 0.218182 | 0.154545 | | | | 0 | 0.036364 | 0.145455 | 0.209091 | 0.245455 | 0.409091 |
| 0.009091 | 0.136364 | 0 | 0.190909 | 0.090909 | 0 | 0.2 | 0.209091 | 0.063636 | 0.272727 | 0.154545 | 0.409091 | 0.081818 | | | | 0.145455 | 0.172727 | 0 | 0 | 0.172727 | 0.254545 |
| 0.009091 | 0.027273 | 0.327273 | 0.181818 | 0.3 | 0.072727 | 0.263636 | 0.245455 | 0.172727 | 0.190909 | 0.236364 | 0.1 | 0.190909 | | | | 0.027273 | 0.227273 | 0.436364 | 0.318182 | 0.145455 | 0.163636 |
| 0.290909 | 0.127273 | 0.227273 | 0.309091 | 0.327273 | 0.127273 | 0.190909 | 0.172727 | 0.036364 | 0.227273 | 0.054545 | 0 | 0.181818 | | | | 0.136364 | 0.272727 | 0.218182 | 0.172727 | 0.227273 | 0 |
| 0.027273 | 0.209091 | 0.245455 | 0.163636 | 0.127273 | 0.118182 | 0.2 | 0.136364 | 0.081818 | 0.127273 | 0.109091 | 0.163636 | 0.163636 | | | | 0.218182 | 0.154545 | 0.145455 | 0 | 0 | 0.172727 |
| 0.163636 | 0.163636 | 0 | 0.009091 | 0.336364 | 0.218182 | 0.018182 | 0.127273 | 0.054545 | 0.190909 | 0.063636 | 0.263636 | 0.127273 | | | | 0 | 0.136364 | 0.245455 | 0.054545 | 0.018182 | 0.381818 |
| 0.172727 | 0.127273 | 0 | 0 | 0 | 0.254545 | 0.090909 | 0.218182 | 0 | 0.072727 | 0.090909 | 0.145455 | 0.009091 | | | | 0.109091 | 0.209091 | 0.1 | 0.3 | 0.163636 | 0.227273 |
| 0.090909 | 0.054545 | 0.209091 | 0.236364 | 0.327273 | 0.036364 | 0 | 0.081818 | 0.027273 | 0.209091 | 0.127273 | 0.118182 | 0.236364 | | | | 0.072727 | 0.236364 | 0.090909 | 0 | 0.072727 | 0 |
| 0 | 0.018182 | 0 | 0 | 0 | 0 | 0.154545 | 0 | 0.327273 | 0.009091 | 0.045455 | 0.245455 | 0 | | | | 0 | 0.009091 | 0.190909 | 0.336364 | 0.118182 | 0.136364 |
| 0.2 | 0.163636 | 0.236364 | 0.254545 | 0.381818 | 0.009091 | 0.136364 | 0.063636 | 0.054545 | 0.045455 | 0.054545 | 0.154545 | 0.145455 | | | | 0 | 0.172727 | 0.154545 | 0.227273 | 0 | 0.227273 |
| 0.054545 | 0.136364 | 0 | 0.109091 | 0 | 0.090909 | 0 | 0.172727 | 0.081818 | 0.363636 | 0.1 | 0.118182 | 0.1 | | | | 0.163636 | 0.090909 | 0.245455 | 0 | 0.163636 | 0 |
| 0 | 0 | 0.263636 | 0.072727 | 0.018182 | 0.136364 | 0.090909 | 0.036364 | 0 | 0.018182 | 0.109091 | 0.118182 | 0.081818 | | | | 0 | 0.345455 | 0 | 0.272727 | 0.318182 | 0.090909 |
| 0 | 0.227273 | 0.027273 | 0.118182 | 0.127273 | 0.181818 | 0 | 0.181818 | 0.072727 | 0.218182 | 0.154545 | 0.263636 | 0.309091 | | | | 0.018182 | 0.272727 | 0.254545 | 0.090909 | 0.054545 | 0.163636 |
| 0.172727 | 0.072727 | 0 | 0 | 0 | 0 | 0.109091 | 0 | 0 | 0.063636 | 0.009091 | 0.118182 | 0 | | | | 0.3 | 0 | 0.209091 | 0 | 0.027273 | 0.163636 |
| 0 | 0.136364 | 0.163636 | 0.2 | 0.145455 | 0 | 0.145455 | 0.218182 | 0.163636 | 0.190909 | 0.045455 | 0.290909 | 0.136364 | | | | 0 | 0.190909 | 0.009091 | 0 | 0.118182 | 0.1 |
| 0.027273 | 0 | 0.009091 | 0.009091 | 0.009091 | 0 | 0.009091 | 0.009091 | 0.227273 | 0.009091 | 0.063636 | 0 | 0.054545 | | | | 0 | 0.072727 | 0.1 | 0.145455 | 0.027273 | 0 |
| 0 | 0.136364 | 0.009091 | 0.081818 | 0.2 | 0.127273 | 0.181818 | 0 | 0.009091 | 0.154545 | 0.154545 | 0 | 0.063636 | | | | 0.127273 | 0.054545 | 0.072727 | 0 | 0.181818 | 0.254545 |
| 0.045455 | 0.109091 | | | 0.009091 | 0 | 0.109091 | 0.109091 | 0.009091 | 0.009091 | 0.090909 | 0.072727 | 0.154545 | | | | 0.136364 | | 0 | 0.145455 | 0.127273 | 0.136364 |

| 6 | 6 | 8 | 9 | 9 | 9 | 11 | 11 | 11 | 11 | 1 | 1 | 1 | 2 | 2 | 5 | 6 | 6 | 8 | 9 |
|---|---|---|---|---|---|---|---|---|---|---|---|---|---|---|---|---|---|---|---|
| Box-8 | Box-6 | Box-5 | Box-1 | Box-2 | Box-6 | Box-1 | Box-2 | Box-4 | Box-6 | Box-7 | Box-8 | Box-2 | Box-4 | Box-7 | Box-3 | Box-1 | Box-4 | Box-7 | Box-3 |
| 37 | 46 | 50.1 | 62 | 63 | 69 | 74 | 76 | 81 | 83 | 1.1 | 2.2 | 5.5 | 19.1 | 25.1 | 34.2 | 38 | 43 | 52.2 | 64 |
| M | M | M | M | M | M | M | M | M | M | M | M | M | M | M | M | M | M | M | M |
| H2O | H2O | H2O | H2O | H2O | H2O | H2O | H2O | H2O | H2O | Malto | Malto | Malto | Malto | Malto | MD | MD | MD | MD | MD |
| MH | MH | MH | MH | MH | MH | MH | MH | MH | MH | MM | MM | MM | MM | MM | MM | MM | MM | MM | MM |
| 0.118182 | 0.209091 | 0 | 0 | 0 | 0 | 0 | 0 | 0 | 0 | 0.1090909 | 0 | 0.16363636 | 0 | 0.16363636 | 0 | 0.10909091 | 0.24545455 | 0 | 0 |
| 0.1 | 0.163636 | 0 | 0.0866667 | 0 | 0 | 0 | 0 | 0 | 0 | 0.1090909 | 0 | 0.36363636 | 0.01818182 | 0.43636364 | 0.24545455 | 0.24545455 | 0.46363636 | 0 | 0 |
| 0.472727 | 0.245455 | 0 | 0.0633333 | 0.0566667 | 0 | 0 | 0 | 0 | 0.0090909 | 0.1727273 | 0.3 | 0.36363636 | 0.02727273 | 0.30909091 | 0.32727273 | 0.21818182 | 0.26363636 | 0 | 0 |
| 0.272727 | 0.509091 | 0.4 | 0.1033333 | 0.07 | 0.0366667 | 0.0727273 | 0 | 0.2 | 0.0090909 | 0.3 | 0.35454545 | 0.72727273 | 0 | 0.5 | 0.63636364 | 0.24545455 | 0.26363636 | 0.12727273 | 0 |
| 0.6 | 0.445455 | 0.236364 | 0.1133333 | 0.1433333 | 0.06 | 0.1636364 | 0 | 0.1545455 | 0.4636364 | 0.3090909 | 0.51818182 | 0.38181818 | 0.16363636 | 0 | 0.28181818 | 0.10909091 | 0.50909091 | 0.38181818 | 0.063333 |
| 0.372727 | 0.154545 | 0.445455 | 0.12 | 0.0566667 | 0.12 | 0.2454545 | 0 | 0.2727273 | 0.1454545 | 0.1545455 | 0.18181818 | 0.08181818 | 0.15454545 | 0.21818182 | 0.25454545 | 0.50909091 | 0.43636364 | 0.40909091 | 0.083333 |
| 0.2 | 0.245455 | 0.545455 | 0.0466667 | 0.0266667 | 0.1466667 | 0.0818182 | 0 | 0.4272727 | 0.5727273 | 0.2272727 | 0.32727273 | 0.32727273 | 0.21818182 | 0.29090909 | 0 | 0.14545455 | 0.5 | 0.39090909 | 0.056667 |
| 0.372727 | 0.363636 | 0.2 | 0.0566667 | 0.1466667 | 0.04 | 0.2818182 | 0.0272727 | 0.2636364 | 0.4545455 | 0.3 | 0.03636364 | 0 | 0.10909091 | 0 | 0.48181818 | 0.02727273 | 0.29090909 | 0.07272727 | 0.076667 |
| 0.390909 | 0.1 | 0.218182 | 0.06 | 0 | 0 | 0.1181818 | 0.0363636 | 0.5 | 0.0090909 | 0.2181818 | 0.3 | 0.13636364 | 0.30909091 | 0.09090909 | 0.07272727 | 0.17272727 | 0.26363636 | 0 | 0.043333 |
| 0.154545 | 0 | 0.154545 | 0.1933333 | 0.0566667 | 0.13 | 0 | 0.2 | 0.1818182 | 0.2454545 | 0 | 0.16363636 | 0.1 | 0.21818182 | 0.48181818 | 0.00909091 | 0.32727273 | 0.19090909 | 0.20909091 | 0 |
| 0.345455 | 0 | 0.172727 | 0 | 0.1566667 | 0.0566667 | 0 | 0.0363636 | 0.0181818 | 0.0818182 | 0.0727273 | 0.20909091 | 0.21818182 | 0.07272727 | 0 | 0.26363636 | 0.16363636 | 0.30909091 | 0.12727273 | 0.023333 |
| 0.109091 | 0.336364 | 0.172727 | 0.1466667 | 0 | 0.0166667 | 0.0272727 | 0.2 | 0.2363636 | 0.0818182 | 0.2363636 | 0.10909091 | 0.25454545 | 0 | 0 | 0.37272727 | 0.47272727 | 0.25454545 | 0.33636364 | 0.086667 |
| 0.218182 | 0 | 0 | 0 | 0 | 0.0466667 | 0.0090909 | 0.0545455 | 0.1454545 | 0.0363636 | 0.1454545 | 0.25454545 | 0.18181818 | 0.31818182 | 0.36363636 | 0.01818182 | 0.00909091 | 0.14545455 | 0.28181818 | 0.023333 |
| 0.072727 | 0.209091 | 0.2 | 0 | 0.0333333 | 0 | 0.1090909 | 0.0454545 | 0 | 0 | 0.0363636 | 0.11818182 | 0.1 | 0.42727273 | 0.02727273 | 0.27272727 | 0.16363636 | 0 | 0 | 0.003333 |
| 0.290909 | 0.2 | 0.072727 | 0.07 | 0 | 0.0166667 | 0 | 0.0272727 | 0.0090909 | 0 | 0 | 0 | 0.18181818 | 0 | 0.28181818 | 0 | 0.13636364 | 0.13636364 | 0.16363636 | 0.023333 |
| 0.009091 | 0.009091 | 0.2 | 0.0266667 | 0 | 0 | 0.1272727 | 0.0090909 | 0.1181818 | 0 | 0.1 | 0.22727273 | 0 | 0.21818182 | 0 | 0 | 0.15454545 | 0 | 0 | 0 |
| 0.145455 | 0.172727 | 0 | 0.0066667 | 0.0033333 | 0.0233333 | 0 | 0.0090909 | 0 | 0 | 0 | 0.00909091 | 0.15454545 | 0.00909091 | 0 | 0.13636364 | 0 | 0 | 0.21818182 | 0.03 |
| 0.136364 | 0.081818 | 0.227273 | 0.01 | 0 | 0 | 0 | 0 | 0.2181818 | 0 | 0.0090909 | 0.21818182 | 0 | 0.39090909 | 0 | 0.00909091 | 0.16363636 | 0.07272727 | 0.18181818 | 0 |
| 0.154545 | 0.136364 | 0.009091 | 0.0866667 | 0.0833333 | 0 | 0 | 0.0090909 | 0 | 0 | 0.0545455 | 0.01818182 | 0.08181818 | 0.07272727 | 0.57272727 | 0.10909091 | 0 | 0.2 | 0 | 0.013333 |
| 0.172727 | 0.118182 | 0 | 0.0533333 | 0 | 0.0666667 | 0 | 0 | 0.0090909 | 0 | 0 | 0.08181818 | 0.13636364 | 0.30909091 | 0 | 0.23636364 | 0.14545455 | 0 | 0.27272727 | 0.003333 |
| 0.090909 | 0.2 | 0.181818 | 0 | 0 | 0 | 0 | 0.0818182 | 0.1090909 | 0 | 0.0090909 | 0 | 0.00909091 | 0.17272727 | 0 | 0.00909091 | 0.18181818 | 0.22727273 | 0 | 0.05 |
| 0.063636 | 0.172727 | 0.236364 | 0.0833333 | 0.0566667 | 0.0766667 | 0 | 0.0090909 | 0.0727273 | 0 | 0 | 0.15454545 | 0.16363636 | 0.18181818 | 0.31818182 | 0.23636364 | 0.14545455 | 0.2 | 0.35454545 | 0 |
| 0.190909 | 0.063636 | 0 | 0.0566667 | 0.0133333 | 0.06 | 0.0363636 | 0.0272727 | 0 | 0 | 0.2090909 | 0 | 0.26363636 | 0.08181818 | 0.47272727 | 0.20909091 | 0.14545455 | 0.16363636 | 0.00909091 | 0.05 |
| 0.345455 | 0.281818 | 0.218182 | 0.0966667 | 0.0733333 | 0.0833333 | 0.0090909 | 0.6636364 | 0.0454545 | 0.0909091 | 0.0909091 | 0.24545455 | 0.29090909 | 0.37272727 | 0 | 0.34545455 | 0.38181818 | 0.38181818 | 0.36363636 | 0.063333 |
| 0.454545 | 0.190909 | 0.363636 | 0.1433333 | 0.12 | 0.11 | 0.1363636 | 0.2363636 | 0.3090909 | 0.1909091 | 0.2363636 | 0.24545455 | 0.63636364 | 0.2 | 0.18181818 | 0.37272727 | 0.21818182 | 0.43636364 | 0.21818182 | 0.083333 |
| 0.336364 | 0.445455 | 0.227273 | 0.0766667 | 0.0466667 | 0.0966667 | 0.2909091 | 0.2090909 | 0.1454545 | 0.4636364 | 0.6454545 | 0.27272727 | 0.35454545 | 0.33636364 | 0.6 | 0.48181818 | 0.34545455 | 0.19090909 | 0.26363636 | 0.083333 |
| 0.145455 | 0.445455 | 0.354545 | 0.08 | 0.1833333 | 0.1233333 | 0.2090909 | 0 | 0.3181818 | 0.3454545 | 0.1181818 | 0.22727273 | 0.58181818 | 0.30909091 | 0.20909091 | 0.26363636 | 0.39090909 | 0.48181818 | 0.48181818 | 0.056667 |
| 0.527273 | 0.2 | 0.418182 | 0.1166667 | 0 | 0.0833333 | 0.3090909 | 0.4727273 | 0.3545455 | 0.2090909 | 0.1636364 | 0.20909091 | 0 | 0 | 0 | 0.27272727 | 0.22727273 | 0.65454545 | 0.08181818 | 0.186667 |
| 0.190909 | 0.263636 | 0.381818 | 0.1066667 | 0.1233333 | 0.11 | 0.2545455 | 0.2727273 | 0.1181818 | 0.2272727 | 0.3454545 | 0.22727273 | 0.37272727 | 0.21818182 | 0 | 0.19090909 | 0.20909091 | 0.38181818 | 0.29090909 | 0 |
| 0.163636 | 0.163636 | 0.145455 | 0.0833333 | 0.09 | 0.1966667 | 0.3090909 | 0 | 0.0545455 | 0.2727273 | 0.3636364 | 0 | 0.34545455 | 0.31818182 | 0.33636364 | 0.13636364 | 0 | 0.00909091 | 0.16363636 | 0.006667 |
| 0.263636 | 0.172727 | 0 | 0.01 | 0.1433333 | 0 | 0.2 | 0.4090909 | 0 | 0.3818182 | 0 | 0.21818182 | 0 | 0.00909091 | 0 | 0.07272727 | 0.00909091 | 0.27272727 | 0 | 0.09 |
| 0.263636 | 0 | 0.418182 | 0 | 0 | 0 | 0 | 0 | 0 | 0.0909091 | 0 | 0 | 0.32727273 | 0 | 0 | 0.22727273 | 0 | 0 | 0.34545455 | 0.076667 |
| 0.409091 | 0.172727 | 0.2 | 0 | 0 | 0.0533333 | 0 | 0.2545455 | 0.1090909 | 0.2181818 | 0.2272727 | 0.17272727 | 0.26363636 | 0 | 0.32727273 | 0.38181818 | 0.14545455 | 0.33636364 | 0 | 0.023333 |
| 0.081818 | 0.190909 | 0.090909 | 0.0766667 | 0.0833333 | 0.05 | 0.5818182 | 0.3727273 | 0.2272727 | 0.1636364 | 0.1363636 | 0.19090909 | 0.01818182 | 0.26363636 | 0 | 0.08181818 | 0.22727273 | 0 | 0.17272727 | 0.056667 |
| 0.218182 | 0.081818 | 0.063636 | 0 | 0 | 0 | 0 | 0.3636364 | 0.0090909 | 0.2272727 | 0.1090909 | 0 | 0.20909091 | 0.29090909 | 0.29090909 | 0.17272727 | 0 | 0.06363636 | 0.16363636 | 0.003333 |
| 0.181818 | 0.081818 | 0.172727 | 0.07 | 0.1066667 | 0 | 0 | 0.0090909 | 0.0818182 | 0 | 0.2 | 0.12727273 | 0 | 0.26363636 | 0 | 0 | 0.24545455 | 0.18181818 | 0 | 0.116667 |
| 0.163636 | 0.154545 | 0.236364 | 0.04 | 0.0033333 | 0.0166667 | 0.0181818 | 0 | 0 | 0.0363636 | 0 | 0.17272727 | 0.22727273 | 0.10909091 | 0 | 0 | 0.06363636 | 0 | 0.31818182 | 0 |
| 0.036364 | 0 | 0 | 0.0666667 | 0 | 0 | 0 | 0.2727273 | 0.0090909 | 0 | 0.0090909 | 0 | 0 | 0 | 0 | 0.11818182 | 0.24545455 | 0.15454545 | 0 | 0.043333 |
| | | 0.172727 | 0.0066667 | 0.09 | 0.0433333 | 0.2363636 | 0 | 0.0363636 | 0.0545455 | 0 | 0.11818182 | 0.05454545 | 0 | 0.24545455 | 0 | | | 0 | 0 |
| | | 0 | 0 | 0 | 0.0633333 | 0.2181818 | 0 | 0 | 0 | 0.0909091 | 0.08181818 | 0.09090909 | 0.60909091 | 0 | | | | 0 | 0.106667 |
| | | 0.027273 | 0 | 0 | 0 | 0 | 0 | 0 | 0 | 0 | 0 | 0.11818182 | 0 | 0 | | | | 0.13636364 | 0.013333 |
| | | 0 | 0.0433333 | 0.03 | 0.0633333 | 0 | 0 | 0 | 0.0363636 | 0.0454545 | 0.08181818 | 0.00909091 | 0.3 | 0.40909091 | | | | 0.20909091 | 0.06 |
| | | 0.127273 | 0.0666667 | 0 | 0.0933333 | 0.2454545 | 0 | 0.0545455 | 0 | 0.3272727 | 0.15454545 | 0.16363636 | 0.22727273 | 0 | | | | 0.08181818 | 0.003333 |
| | | 0.245455 | 0.02 | 0.09 | 0 | 0 | 0.1636364 | 0 | 0.2818182 | 0.0181818 | 0.00909091 | 0.17272727 | 0.40909091 | 0 | | | | 0.19090909 | 0.08 |
| | | 0.163636 | 0.03 | 0 | 0.08 | 0.2 | 0.2727273 | 0 | 0.0363636 | 0.1545455 | 0.08181818 | 0.28181818 | 0.13636364 | 0.36363636 | | | | 0.16363636 | 0.09 |
| | | 0.4 | 0.19 | 0.08 | 0.0966667 | 0.5545455 | 0.2454545 | 0.5090909 | 0.2272727 | 0.3090909 | 0.45454545 | 0.51818182 | 0.12727273 | 0.43636364 | | | | 0.42727273 | 0.096667 |
| | | 0.245455 | 0.02 | 0.1566667 | 0.13 | 0.0636364 | 0.0636364 | 0.4363636 | 0.4363636 | 0.6181818 | 0.38181818 | 0.33636364 | 0.43636364 | 0 | | | | 0.23636364 | 0.143333 |
| | | 0.209091 | 0.0966667 | 0.0433333 | 0.1166667 | 0.3 | 0.3 | 0.0181818 | 0.1636364 | 0.2727273 | 0.30909091 | 0.39090909 | 0.37272727 | 0.02727273 | | | | 0.43636364 | 0.053333 |
| | | 0.209091 | 0.1866667 | 0.0733333 | 0.0966667 | 0.3454545 | 0.1181818 | 0.5 | 0.1818182 | 0.4181818 | 0.29090909 | 0.18181818 | 0.45454545 | 0.47272727 | | | | 0 | 0.02 |
| | | 0.336364 | 0.08 | 0.06 | 0.1333333 | 0.2090909 | 0.2 | 0.4272727 | 0.2454545 | 0.3818182 | 0.18181818 | 0.17272727 | 0.44545455 | 0 | | | | 0.52727273 | 0.033333 |
| | | 0.463636 | 0.01 | 0.0733333 | 0 | 0.2090909 | 0.0181818 | 0.6 | 0.4636364 | 0.3818182 | 0.02727273 | 0.39090909 | 0 | 0 | | | | 0.04545455 | 0.096667 |
| | | 0.172727 | 0.0966667 | 0.1 | 0.07 | 0.3181818 | 0.0181818 | 0 | 0.0090909 | 0 | 0 | 0.19090909 | 0 | 0.43636364 | | | | 0 | 0 |
| | | 0.045455 | 0 | 0 | 0.07 | 0.1636364 | 0.1545455 | 0.0090909 | 0.0727273 | 0.3363636 | 0.32727273 | 0 | 0.26363636 | 0 | | | | 0.22727273 | 0 |
| | | 0.218182 | 0.1 | 0.0366667 | 0 | 0 | 0 | 0.2272727 | 0.2 | 0 | 0.17272727 | 0.22727273 | 0 | 0.45454545 | | | | 0 | 0 |
| | | 0.190909 | 0.06 | 0.03 | 0 | 0 | 0.3 | 0.0181818 | 0.0909091 | 0 | 0.00909091 | 0 | 0.05454545 | 0 | | | | 0.25454545 | 0.036667 |
| | | 0.290909 | 0.03 | 0.0333333 | 0.0433333 | 0.0181818 | 0.0090909 | 0.0636364 | 0.0818182 | 0 | 0.33636364 | 0 | 0.12727273 | 0.09090909 | | | | 0.04545455 | 0.033333 |
| | | 0.209091 | 0 | 0.07 | 0.11 | 0 | 0.2909091 | 0 | 0.1272727 | 0 | 0.02727273 | 0.2 | 0.13636364 | 0 | | | | 0.35454545 | 0.006667 |
| | | 0 | 0.0333333 | 0 | 0 | 0 | 0 | 0 | 0 | 0 | 0.18181818 | 0 | 0.07272727 | 0 | | | | 0 | 0.08 |
| | | 0.154545 | 0 | 0 | 0 | 0 | 0.0909091 | 0 | 0.0636364 | 0 | 0.16363636 | 0.18181818 | 0 | 0 | | | | 0.18181818 | 0.036667 |
| | | 0.009091 | 0.0366667 | 0.05 | 0.0433333 | 0.2181818 | 0.2 | 0 | 0.0090909 | 0 | 0.00909091 | 0 | 0.23636364 | 0 | | | | 0 | 0 |
| | | 0.118182 | 0 | 0 | 0 | | | | | 0.0727273 | 0.09090909 | 0 | 0.13636364 | 0 | | | | 0.24545455 | 0.04 |
| | | | 0.06 | 0 | 0.1133333 | | | | | 0 | 0 | 0.20909091 | | | | | | | 0 |
| 0.4 | 0.036364 | 0.372727 | 0.3363636 | 0.3090909 | 0.3636364 | 0.1818182 | 0.0727273 | 0.0909091 | 0.3818182 | 0.2545455 | 0.01818182 | 0.05454545 | 0.31818182 | 0.26363636 | 0.13636364 | 0.14545455 | 0.32727273 | 0.16363636 | 0.245455 |
| 0.127273 | 0.154545 | 0.236364 | 0 | 0.5 | 0.0363636 | 0.0272727 | 0.1636364 | 0.0909091 | 0.0181818 | 0.3272727 | 0.54545455 | 0.25454545 | 0.16363636 | 0.32727273 | 0.5 | 0.27272727 | 0.2 | 0 | 0.154545 |
| 0.254545 | 0.4 | 0.027273 | 0.3818182 | 0.0090909 | 0.4454545 | 0.1636364 | 0.3909091 | 0.1545455 | 0.2454545 | 0.6272727 | 0.28181818 | 0.18181818 | 0.29090909 | 0.28181818 | 0.27272727 | 0.44545455 | 0.36363636 | 0.23636364 | 0.327273 |
| 0.527273 | 0.218182 | 0.236364 | 0.4090909 | 0.4454545 | 0.1909091 | 0.4090909 | 0.2454545 | 0.1363636 | 0.2454545 | 0.1636364 | 0.30909091 | 0.04545455 | 0 | 0.46363636 | 0.27272727 | 0.33636364 | 0.38181818 | 0.54545455 | 0.309091 |
| 0.154545 | 0.063636 | 0.045455 | 0.4545455 | 0.0181818 | 0.1363636 | 0 | 0.2454545 | 0.0636364 | 0 | 0.1272727 | 0.24545455 | 0.01818182 | 0.03636364 | 0 | 0.27272727 | 0.36363636 | 0 | 0.5 | 0.3 |
| 0.163636 | 0.2 | 0.036364 | 0 | 0.2636364 | 0.2272727 | 0.3181818 | 0.0181818 | 0.1636364 | 0.3272727 | 0.3727273 | 0.20909091 | 0.01818182 | 0.03636364 | 0 | 0.12727273 | 0 | 0.32727273 | 0 | 0.390909 |
| 0.009091 | 0.190909 | 0.245455 | 0.3545455 | 0 | 0.3090909 | 0.1181818 | 0.1727273 | 0.1636364 | 0.2727273 | 0 | 0 | 0.06363636 | 0.41818182 | 0.16363636 | 0.07272727 | 0.1 | 0.29090909 | 0 | 0 |
| 0.163636 | 0.081818 | 0.445455 | 0.3090909 | 0.4818182 | 0.2545455 | 0.5 | 0.2818182 | 0.0545455 | 0 | 0.1636364 | 0.12727273 | 0.14545455 | 0.01818182 | 0.10909091 | 0.05454545 | 0 | 0.11818182 | 0 | 0.190909 |
| 0.545455 | 0.254545 | 0.027273 | 0 | 0.1 | 0 | 0.0818182 | 0.2545455 | 0.3181818 | 0.3636364 | 0 | 0 | 0.06363636 | 0.35454545 | 0 | 0.33636364 | 0.25454545 | 0.09090909 | 0 | 0.027273 |
| 0 | 0.145455 | 0.327273 | 0.1090909 | 0 | 0.3 | 0.2272727 | 0.0727273 | 0.1181818 | 0 | 0 | 0 | 0 | 0.01818182 | 0.10909091 | 0.22727273 | 0.22727273 | 0.22727273 | 0 | 0.063636 |
| 0.218182 | 0.2 | 0 | 0 | 0 | 0.0909091 | 0 | 0.0909091 | 0.1454545 | . | 0.2636364 | 0.02727273 | 0 | 0 | 0.04545455 | 0.37272727 | 0 | 0.3 | 0.26363636 | 0.063636 |
| 0.145455 | 0.190909 | 0 | 0.1636364 | 0 | 0.0363636 | 0.2727273 | 0.2181818 | 0 | . | 0 | 0.01818182 | 0 | 0.29090909 | 0.16363636 | 0.18181818 | 0 | 0.27272727 | 0 | 0.054545 |
| 0 | 0.090909 | 0.654545 | 0.1818182 | 0.1090909 | 0.0363636 | 0 | 0.2 | 0.2909091 | . | 0.0909091 | 0 | 0 | 0.2 | 0.36363636 | 0.16363636 | 0.00909091 | 0.06363636 | 0.13636364 | 0.063636 |
| 0 | 0.181818 | 0 | 0.0545455 | 0.1363636 | 0.1636364 | 0.1181818 | 0 | 0 | . | 0.2272727 | 0.05454545 | 0 | 0 | 0.27272727 | 0.22727273 | 0.41818182 | 0.35454545 | 0.33636364 | 0.145455 |
| 0 | 0.2 | 0 | 0 | 0.1545455 | 0.2454545 | 0.0909091 | 0.0090909 | 0.1 | . | 0.1727273 | 0 | 0 | 0 | 0.22727273 | 0.25454545 | 0.21818182 | 0.00909091 | 0.42727273 | 0.072727 |
| 0.027273 | 0.081818 | 0.372727 | 0.2636364 | 0 | 0.3363636 | 0.0090909 | 0.0090909 | 0.0454545 | . | 0.2909091 | 0 | 0 | 0 | 0 | 0.25454545 | 0 | 0.3 | 0.2 | 0.009091 |
| 0.009091 | 0.254545 | 0 | 0.0454545 | 0.1090909 | 0.2272727 | 0.1818182 | 0.0090909 | 0 | . | 0 | 0 | 0 | 0 | 0.33636364 | 0.31818182 | 0.01818182 | 0 | 0.42727273 | 0.090909 |
| 0.190909 | 0.018182 | 0 | 0.2636364 | 0 | 0 | 0.1454545 | 0.0454545 | 0.0090909 | . | 0.2181818 | 0 | 0 | 0.21818182 | 0.01818182 | 0.01818182 | 0 | 0.26363636 | 0.46363636 | 0 |
| 0.009091 | 0.163636 | 0.281818 | 0 | 0.0454545 | 0.2727273 | 0 | 0 | 0.3 | . | 0.2181818 | 0.24545455 | 0 | 0.02727273 | 0 | 0.02727273 | 0.03636364 | 0.02727273 | 0 | 0.127273 |
| 0.027273 | 0.3 | 0.263636 | 0.1363636 | 0.1636364 | 0.1636364 | 0.0909091 | 0.0545455 | 0 | . | 0 | 0.05454545 | 0 | 0.14545455 | 0.02727273 | 0.10909091 | 0.23636364 | 0.11818182 | 0 | 0.054545 |
| 0.190909 | 0.018182 | 0.009091 | 0.0818182 | 0.4818182 | 0.0545455 | 0.2454545 | 0.4818182 | 0.3545455 | . | 0.3727273 | 0.56363636 | 0.27272727 | 0.23636364 | 0.14545455 | 0.09090909 | 0.15454545 | 0.30909091 | 0 | 0.163636 |
| 0.6 | 0.272727 | 0.072727 | 0.5363636 | 0.2090909 | 0.1636364 | 0.1272727 | 0 | 0.3181818 | . | 0.2727273 | 0 | 0 | 0.50909091 | 0.33636364 | 0.00909091 | 0.30909091 | 0.31818182 | 0.22727273 | 0.272727 |
| 0.018182 | 0.009091 | 0.418182 | 0 | 0.3545455 | 0.3818182 | 0.4363636 | 0.3818182 | 0.4 | . | 0 | 0 | 0.55454545 | 0.51818182 | 0.14545455 | 0.10909091 | 0.12727273 | 0.18181818 | 0.50909091 | 0 |
| 0.345455 | 0.163636 | 0 | 0.3727273 | 0.1636364 | 0.2909091 | 0.0090909 | 0.2454545 | 0.1909091 | . | 0.2 | 0.52727273 | 0.55454545 | 0 | 0.28181818 | 0.2 | 0.35454545 | 0.39090909 | 0 | 0.163636 |
| 0.263636 | 0.272727 | 0.845455 | 0.0272727 | 0 | 0.0181818 | 0.1636364 | 0.6 | 0.0545455 | . | 0.4181818 | 0 | 0.55454545 | 0.2 | 0.33636364 | 0.22727273 | 0.39090909 | 0.02727273 | 0.73636364 | 0.236364 |
| 0.309091 | 0.363636 | 0.027273 | 0.0818182 | 0.0181818 | 0.2181818 | 0.2727273 | 0.4363636 | 0.2181818 | . | 0.0090909 | 0 | 0.32727273 | 0.52727273 | 0.15454545 | 0.18181818 | 0.26363636 | 0.36363636 | 0.20909091 | 0.518182 |
| 0.281818 | 0.263636 | 0.3 | 0.4090909 | 0.2454545 | 0.0363636 | 0.1909091 | 0.0545455 | 0.1545455 | . | 0.1181818 | 0 | 0 | 0.11818182 | 0 | 0.2 | 0.1 | 0.01818182 | 0.18181818 | 0.2 |
| 0.336364 | 0.145455 | 0 | 0 | 0 | 0.4181818 | 0.4545455 | 0 | 0.1636364 | . | 0.0090909 | 0.33636364 | 0 | 0.58181818 | 0.26363636 | 0.05454545 | 0.34545455 | 0.14545455 | 0 | 0.254545 |
| 0.318182 | 0.154545 | 0.145455 | 0.4727273 | 0.2454545 | 0.0454545 | 0.3 | 0.2818182 | 0.3272727 | . | 0.0272727 | 0.30909091 | 0 | 0 | 0.32727273 | 0.12727273 | 0.39090909 | 0.17272727 | 0.02727273 | 0.027273 |
| 0.236364 | 0.118182 | 0 | 0.2454545 | 0.0454545 | 0.2636364 | 0.1454545 | 0.4727273 | 0.0090909 | . | 0.1454545 | 0 | 0 | 0 | 0.33636364 | 0.15454545 | 0 | 0.19090909 | 0.02727273 | 0.154545 |
| 0.327273 | 0.090909 | 0.327273 | 0.1454545 | 0.0454545 | 0.0272727 | 0.3090909 | 0 | 0.3909091 | . | 0 | 0 | 0.28181818 | 0 | 0 | 0.10909091 | 0 | 0.02727273 | 0.01818182 | 0.481818 |
| 0.263636 | 0.236364 | 0 | 0.0181818 | 0.0909091 | 0.3727273 | 0.4545455 | 0.3545455 | 0.0545455 | . | 0.4090909 | 0.31818182 | 0.01818182 | 0.20909091 | 0 | 0.2 | 0 | 0.10909091 | 0.13636364 | 0.090909 |
| 0.054545 | 0.027273 | 0.536364 | 0.5181818 | 0.1545455 | 0.1545455 | 0 | 0.0909091 | 0.0818182 | . | 0.4272727 | 0 | 0 | 0 | 0.18181818 | 0.16363636 | 0.24545455 | 0.22727273 | 0 | 0.309091 |
| 0.163636 | 0.227273 | 0.281818 | 0.3545455 | 0.3181818 | 0.1454545 | 0 | 0.0454545 | 0.0181818 | . | 0 | 0.32727273 | 0 | 0 | 0 | 0.00909091 | 0.24545455 | 0.07272727 | 0 | 0.009091 |
| 0.009091 | 0 | 0 | 0.0090909 | 0.1272727 | 0.5454545 | 0.1 | 0.2545455 | 0.2363636 | . | 0 | 0 | 0 | 0 | 0.21818182 | 0.25454545 | 0.17272727 | 0.14545455 | 0.13636364 | 0.009091 |
| 0 | 0.4 | 0.345455 | 0.2909091 | 0.3727273 | 0 | 0.4272727 | 0.2 | 0.0363636 | . | 0.3636364 | 0.16363636 | 0 | 0.37272727 | 0.01818182 | 0.18181818 | 0.10909091 | 0.36363636 | 0 | 0.245455 |
| 0.154545 | 0.027273 | 0 | 0 | 0 | 0.0090909 | 0 | 0 | 0.0818182 | . | 0.3909091 | 0.08181818 | 0.11818182 | 0.39090909 | 0 | 0.21818182 | 0.00909091 | 0.39090909 | 0 | 0 |
| 0 | 0.236364 | 0 | 0.0272727 | 0 | 0.0454545 | 0.3363636 | 0 | 0 | . | 0.1636364 | 0.3 | 0.00909091 | 0.00909091 | 0.10909091 | 0.11818182 | 0 | 0.09090909 | 0 | 0.063636 |
| 0 | 0.118182 | 0.572727 | 0.1 | 0 | 0.0636364 | 0.0090909 | 0.0181818 | 0.2181818 | . | 0 | 0.00909091 | 0 | 0.41818182 | 0.05454545 | 0.01818182 | 0 | 0.31818182 | 0.00909091 | 0.009091 |

| | | | | | | | | | | | | | | | | | | | |
|---|---|---|---|---|---|---|---|---|---|---|---|---|---|---|---|---|---|---|---|
| | | 0 | 0 | 0.1181818 | 0 | 0 | 0 | 0.3181818 | . | 0.1818182 | 0 | 0.20909091 | 0 | 0.04545455 | 0.01818182 | | | 0 | 0.336364 |
| | | 0 | 0.1181818 | 0.2727273 | 0.0818182 | 0.2454545 | 0 | 0 | . | 0.0727273 | 0.14545455 | 0.00909091 | 0.55454545 | 0.00909091 | | | | 0.05454545 | 0.009091 |
| | | 0 | 0.0090909 | 0.3454545 | 0.2272727 | 0 | 0.0090909 | 0.1727273 | . | 0.1272727 | 0.01818182 | 0.04545455 | 0.06363636 | 0.29090909 | | | | 0 | 0 |
| | | 0.281818 | 0.1272727 | 0 | 0.1545455 | 0.1909091 | 0 | 0 | . | 0.4818182 | 0.37272727 | 0.26363636 | 0.07272727 | 0.47272727 | | | | 0.64545455 | 0.609091 |
| | | 0 | 0.1 | 0.0363636 | 0.0636364 | 0.0181818 | 0.3818182 | 0.1 | . | 0.0090909 | 0.00909091 | 0 | 0.55454545 | 0.00909091 | | | | 0.29090909 | 0.009091 |
| | | 0.154545 | 0.1272727 | 0.0727273 | 0.4454545 | 0.0454545 | 0.3636364 | 0.1727273 | . | 0.0090909 | 0.35454545 | 0.40909091 | 0.35454545 | 0.37272727 | | | | 0 | 0.036364 |
| | | 0.309091 | 0.0181818 | 0.1727273 | 0.3181818 | 0.2818182 | 0.8 | 0.6454545 | . | 0.5636364 | 0.41818182 | 0.46363636 | 0.2 | 0.38181818 | | | | 0.6 | 0 |
| | | 0.254545 | 0.2727273 | 0.0818182 | 0.0090909 | 0.2363636 | 0.3727273 | 0.5818182 | . | 0.0272727 | 0.01818182 | 0.47272727 | 0.08181818 | 0.4 | | | | 0 | 0.018182 |
| | | 0.263636 | 0.2636364 | 0.3363636 | 0.3909091 | 0.1818182 | 0 | 0 | . | 0.1636364 | 0.28181818 | 0 | 0.30909091 | 0 | | | | 1.23636364 | 0.236364 |
| | | 0.009091 | 0.2363636 | 0 | 0 | 0.1818182 | 0 | 0.0090909 | . | 0.0363636 | 0.05454545 | 0 | 0.20909091 | 0 | | | | 0.24545455 | 0.081818 |
| | | 0.172727 | 0.4363636 | 0.1363636 | 0.0363636 | 0.1272727 | 0.2818182 | 0.3 | . | 0 | 0 | 0.32727273 | 0 | 0.30909091 | | | | 0 | 0.445455 |
| | | 0 | 0 | 0.5181818 | 0.1363636 | 0.3090909 | 0 | 0.0545455 | . | 0.1454545 | 0.31818182 | 0.38181818 | 0.12727273 | 0 | | | | 0 | 0.172727 |
| | | 0.145455 | 0.0090909 | 0.2272727 | 0 | 0.0272727 | 0.1818182 | 0.3727273 | . | 0 | 0 | 0 | 0.14545455 | 0.19090909 | | | | 0.00909091 | 0 |
| | | 0 | 0.6545455 | 0 | 0.1272727 | 0.3545455 | 0.1363636 | 0 | . | 0.2909091 | 0 | 0 | 0.39090909 | 0.10909091 | | | | 0 | 0.472727 |
| | | 0.009091 | 0 | 0.1545455 | 0.1545455 | 0 | 0.1818182 | 0.3090909 | . | 0 | 0.30909091 | 0.00909091 | 0.06363636 | 0.07272727 | | | | 0 | 0 |
| | | 0.290909 | 0.0272727 | 0.8363636 | 0.2 | 0.5 | 0.2 | 0.0363636 | . | 0.0090909 | 0.01818182 | 0.41818182 | 0 | 0.08181818 | | | | 0 | 0.445455 |
| | | 0.490909 | 0 | 0 | 0.1818182 | 0.0909091 | 0 | 0.0636364 | . | 0.0454545 | 0.01818182 | 0 | 0 | 0 | | | | 0 | 0.109091 |
| | | 0 | 0.3454545 | 0 | 0.0090909 | 0.3727273 | 0.4272727 | 0.2 | . | 0.6454545 | 0.36363636 | 0 | 0.48181818 | 0.1 | | | | 0 | 0.127273 |
| | | 0.3 | 0 | 0 | 0.4 | 0.0090909 | 0 | 0 | . | 0.0181818 | 0 | 0 | 0.02727273 | 0.06363636 | | | | 0 | 0.018182 |
| | | 0.009091 | 0 | 0.0545455 | 0 | 0.0181818 | 0.0727273 | 0.1090909 | . | 0 | 0.01818182 | 0.45454545 | 0 | 0.01818182 | | | | 0 | 0.009091 |
| | | 0 | 0 | 0 | 0.2727273 | 0.1909091 | 0.1818182 | 0 | . | 0.2 | 0.18181818 | 0 | 0.09090909 | 0.00909091 | | | | 0 | 0.027273 |
| | | 0 | 0 | 0 | 0 | 0.0272727 | 0.1909091 | 0 | . | 0 | 0.02727273 | 0 | | | | | | 0 | 0.018182 |

| | | | | | | | | | | | | | | | | | | | |
|---|---|---|---|---|---|---|---|---|---|---|---|---|---|---|---|---|---|---|---|
| 0.281818 | 0 | 0.136364 | 0.3 | 0.0909091 | 0.0727273 | 0.0818182 | 0.0636364 | 0.0818182 | 0 | 0.1545455 | 0.09090909 | 0.17272727 | 0.02727273 | 0.42727273 | 0.01818182 | 0 | 0.23636364 | 0.26363636 | 0.1 |
| 0.181818 | 0.209091 | 0.209091 | 0.2545455 | 0.2 | 0.2545455 | 0.1454545 | 0.5181818 | 0.1272727 | 0.1818182 | 0.0272727 | 0.25454545 | 0.1 | 0.25454545 | 0.12727273 | 0.42727273 | 0.08181818 | 0.16363636 | 0.22727273 | 0.281818 |
| 0.236364 | 0.209091 | 0.136364 | 0.2090909 | 0.1181818 | 0.0090909 | 0.4636364 | 0 | 0.1545455 | 0.0727273 | 0.2545455 | 0.36363636 | 0.37272727 | 0.08181818 | 0.20909091 | 0.43636364 | 0.29090909 | 0.27272727 | 0.04545455 | 0.209091 |
| 0.245455 | 0.263636 | 0.327273 | 0.3636364 | 0.0727273 | 0.1727273 | 0.3272727 | 0.3272727 | 0.2727273 | 0.4272727 | 0.2454545 | 0.19090909 | 0.43636364 | 0.04545455 | 0.11818182 | 0 | 0.34545455 | 0.05454545 | 0.18181818 | 0.272727 |
| 0 | 0 | 0.218182 | 0 | 0.2 | 0.2 | 0.0818182 | 0.3272727 | 0.1363636 | 0.2 | 0.0545455 | 0.14545455 | 0.03636364 | 0.23636364 | 0.17272727 | 0.19090909 | 0.16363636 | 0.23636364 | 0.28181818 | 0.254545 |
| 0.236364 | 0.172727 | 0.081818 | 0.3181818 | 0.1181818 | 0.1181818 | 0.0454545 | 0 | 0.0090909 | 0.2 | 0.1909091 | 0 | 0.01818182 | 0.34545455 | 0.34545455 | 0 | 0.29090909 | 0 | 0.13636364 | 0 |
| 0 | 0.009091 | 0.218182 | 0.1818182 | 0.0181818 | 0.2090909 | 0.1636364 | 0.2727273 | 0.3818182 | 0.0090909 | 0.1454545 | 0.21818182 | 0.2 | 0 | 0.14545455 | 0.06363636 | 0 | 0.20909091 | 0.14545455 | 0.481818 |
| 0.118182 | 0 | 0.254545 | 0.0545455 | 0.2 | 0 | 0 | 0.2363636 | 0.1545455 | 0.1363636 | 0.0272727 | 0.17272727 | 0.00909091 | 0 | 0.11818182 | 0.08181818 | 0.02727273 | 0.15454545 | 0.08181818 | 0 |
| 0.009091 | 0.045455 | 0 | 0.1545455 | 0.1909091 | 0.1363636 | 0.0636364 | 0.0363636 | 0 | 0.1818182 | 0.2 | 0 | 0.1 | 0.17272727 | 0.19090909 | 0.04545455 | 0 | 0 | 0 | 0 |
| 0.118182 | 0 | 0.045455 | 0 | 0.2818182 | 0.1 | 0.1272727 | 0.4272727 | 0.1818182 | 0 | 0.3181818 | 0.12727273 | 0.00909091 | 0.01818182 | 0.19090909 | 0.12727273 | 0 | 0.02727273 | 0 | 0 |
| 0.127273 | 0.118182 | 0.081818 | 0.1272727 | 0.0090909 | 0.0818182 | 0.1 | 0.0272727 | 0 | 0 | 0.2 | 0.21818182 | 0.02727273 | 0.2 | 0.20909091 | 0.20909091 | 0 | 0.10909091 | 0.2 | 0.163636 |
| 0 | 0.236364 | 0.1 | 0.1818182 | 0.1090909 | 0.1181818 | 0.0545455 | 0.2363636 | 0.2363636 | 0.3727273 | 0 | 0.12727273 | 0.07272727 | 0 | 0 | 0.02727273 | 0.00909091 | 0.01818182 | 0.15454545 | 0.009091 |
| 0.1 | 0.218182 | 0.181818 | 0.0090909 | 0.1090909 | 0.1454545 | 0.2090909 | 0 | 0.2818182 | 0.1 | 0.1090909 | 0 | 0.2 | 0.30909091 | 0.06363636 | 0 | 0.29090909 | 0.21818182 | 0.27272727 | 0.381818 |
| 0 | 0.036364 | 0.227273 | 0.1909091 | 0.0727273 | 0 | 0.0818182 | 0.0909091 | 0 | 0 | 0 | 0.36363636 | 0.15454545 | 0.17272727 | 0.05454545 | 0.23636364 | 0 | 0.16363636 | 0.10909091 | 0.090909 |
| 0.209091 | 0.054545 | 0.127273 | 0.0454545 | 0.1363636 | 0.1909091 | 0.1909091 | 0 | 0.1272727 | 0.0636364 | 0.3545455 | 0 | 0.11818182 | 0.11818182 | 0.02727273 | 0.17272727 | 0.31818182 | 0.00909091 | 0.08181818 | 0.236364 |
| 0 | 0.1 | 0.136364 | 0.1545455 | 0 | 0.0454545 | 0.1636364 | 0.1454545 | 0.0727273 | 0.0454545 | 0.0818182 | 0 | 0.02727273 | 0.00909091 | 0.01818182 | 0.01818182 | 0 | 0.09090909 | 0.10909091 | 0 |
| 0 | 0 | 0.063636 | 0.1545455 | 0.0363636 | 0.0636364 | 0.0454545 | 0 | 0.1363636 | 0.1363636 | 0 | 0.06363636 | 0.08181818 | 0.22727273 | 0.10909091 | 0.11818182 | 0 | 0.01818182 | 0 | 0.018182 |
| 0.118182 | 0.254545 | 0.063636 | 0.1727273 | 0 | 0.1181818 | 0.1272727 | 0.0363636 | 0.0090909 | 0.1727273 | 0.0545455 | 0.13636364 | 0.14545455 | 0.02727273 | 0.1 | 0.15454545 | 0 | 0.16363636 | 0.03636364 | 0.009091 |
| 0.181818 | 0 | 0.063636 | 0 | 0.1090909 | 0.0909091 | 0.1545455 | 0 | 0.0727273 | 0 | 0.0909091 | 0.00909091 | 0.00909091 | 0 | 0.15454545 | 0.00909091 | 0.21818182 | 0.14545455 | 0.24545455 | 0.218182 |
| 0.018182 | 0.218182 | 0.245455 | 0.1545455 | 0.0727273 | 0.1 | 0.1818182 | 0.1090909 | 0.2090909 | 0.2636364 | 0.0454545 | 0.09090909 | 0.09090909 | 0.51818182 | 0.16363636 | 0.27272727 | 0 | 0.18181818 | 0.1 | 0.209091 |
| 0.309091 | 0.345455 | 0.172727 | 0 | 0.3363636 | 0.1 | 0 | 0.1454545 | 0 | 0.0636364 | 0.1545455 | 0.10909091 | 0.19090909 | 0 | 0.10909091 | 0.15454545 | 0.21818182 | 0.24545455 | 0.10909091 | 0.127273 |
| 0.3 | 0 | 0.136364 | 0.2181818 | 0 | 0.0909091 | 0.2181818 | 0.1636364 | 0.0909091 | 0.2363636 | 0.3636364 | 0.10909091 | 0.19090909 | 0.46363636 | 0.39090909 | 0.01818182 | 0 | 0.03636364 | 0.28181818 | 0.3 |
| 0 | 0.290909 | 0.227273 | 0.2727273 | 0.0909091 | 0.3 | 0.0181818 | 0.1636364 | 0.2090909 | 0.3272727 | 0.1636364 | 0.32727273 | 0.12727273 | 0 | 0.01818182 | 0.11818182 | 0.25454545 | 0 | 0.17272727 | 0.009091 |
| 0.409091 | 0.081818 | 0.172727 | 0.3636364 | 0.3181818 | 0.1090909 | 0.4909091 | 0.7272727 | 0.0636364 | 0.2909091 | 0.3363636 | 0.15454545 | 0.15454545 | 0 | 0.58181818 | 0.3 | 0.08181818 | 0.3 | 0.30909091 | 0.009091 |
| 0.009091 | 0.145455 | 0.154545 | 0.0090909 | 0.1818182 | 0.2 | 0.1 | 0 | 0.3636364 | 0.0181818 | 0 | 0.27272727 | 0.37272727 | 0.02727273 | 0.01818182 | 0.21818182 | 0.38181818 | 0.15454545 | 0.29090909 | 0.254545 |
| 0.245455 | 0.145455 | 0.236364 | 0.3636364 | 0.2363636 | 0.2090909 | 0.4363636 | 0.4909091 | 0.3272727 | 0.1818182 | 0.2 | 0.13636364 | 0.04545455 | 0.16363636 | 0 | 0 | 0 | 0.07272727 | 0.1 | 0 |
| 0 | 0.145455 | 0.045455 | 0 | 0.1 | 0.1454545 | 0 | 0.2363636 | 0.2090909 | 0.1 | 0.0909091 | 0.01818182 | 0.12727273 | 0.42727273 | 0.10909091 | 0 | 0.3 | 0.18181818 | 0.11818182 | 0 |
| 0.190909 | 0.154545 | 0.081818 | 0.1909091 | 0.0818182 | 0.0363636 | 0.1818182 | 0.2090909 | 0 | 0.1363636 | 0 | 0.07272727 | 0.01818182 | 0.02727273 | 0.09090909 | 0.1 | 0.11818182 | 0 | 0.13636364 | 0 |
| 0.136364 | 0.1 | 0.172727 | 0.3272727 | 0.1818182 | 0.1545455 | 0.1272727 | 0.1545455 | 0.2 | 0.1909091 | 0.2090909 | 0.14545455 | 0.19090909 | 0.4 | 0.05454545 | 0 | 0.02727273 | 0.20909091 | 0.04545455 | 0.245455 |
| 0.272727 | 0.118182 | 0.109091 | 0 | 0.2454545 | 0.1181818 | 0 | 0.1454545 | 0.0545455 | 0.1818182 | 0.0272727 | 0.11818182 | 0.1 | 0 | 0.13636364 | 0.08181818 | 0.08181818 | 0 | 0.21818182 | 0.163636 |
| 0 | 0.136364 | 0 | 0.0272727 | 0 | 0.1909091 | 0.2090909 | 0.1636364 | 0.1545455 | 0.0909091 | 0.0727273 | 0 | 0.11818182 | 0 | 0.16363636 | 0.26363636 | 0.17272727 | 0.13636364 | 0.11818182 | 0 |
| 0 | 0.163636 | 0.181818 | 0 | 0.0636364 | 0.1272727 | 0.1181818 | 0.2181818 | 0.0909091 | 0.0636364 | 0.1363636 | 0.10909091 | 0.15454545 | 0.32727273 | 0.22727273 | 0 | 0 | 0.1 | 0.15454545 | 0.227273 |
| 0.3 | 0.054545 | 0.1 | 0.3090909 | 0.2818182 | 0.2727273 | 0.1363636 | 0.2181818 | 0.1545455 | 0.1818182 | 0.1636364 | 0.20909091 | 0.00909091 | 0.03636364 | 0.13636364 | 0.07272727 | 0.15454545 | 0.10909091 | 0.18181818 | 0.3 |
| 0 | 0.045455 | 0.254545 | 0 | 0.0090909 | 0 | 0.1727273 | 0.1636364 | 0.0636364 | 0.2 | 0.1727273 | 0.2 | 0.07272727 | 0.37272727 | 0.29090909 | 0.02727273 | 0.09090909 | 0.28181818 | 0.36363636 | 0.163636 |
| 0.109091 | 0.172727 | 0.109091 | 0.1272727 | 0.0636364 | 0.0181818 | 0.2090909 | 0.2818182 | 0.2090909 | 0 | 0.0454545 | 0.14545455 | 0 | 0 | 0.16363636 | 0.34545455 | 0.24545455 | 0.02727273 | 0.00909091 | 0 |
| 0 | 0.1 | 0.172727 | 0.1454545 | 0.0090909 | 0.1636364 | 0.1 | 0 | 0.0272727 | 0.1272727 | 0.2727273 | 0.09090909 | 0.38181818 | 0 | 0.00909091 | 0.14545455 | 0.10909091 | 0.13636364 | 0.07272727 | 0.281818 |
| 0.145455 | 0.190909 | 0.054545 | 0.2545455 | 0.0181818 | 0.2727273 | 0.0545455 | 0 | 0.0727273 | 0 | 0.0909091 | 0.10909091 | 0 | 0.37272727 | 0.07272727 | 0.12727273 | 0.15454545 | 0.11818182 | 0.00909091 | 0.218182 |
| 0.1 | 0.054545 | 0.118182 | 0.0727273 | 0.1545455 | 0.0818182 | 0.2 | 0.1181818 | 0.1636364 | 0.2 | 0.0363636 | 0.08181818 | 0.28181818 | 0.00909091 | 0.12727273 | 0.05454545 | 0.27272727 | 0 | 0.17272727 | 0.036364 |
| 0 | 0 | 0.190909 | 0 | 0.0818182 | 0.1181818 | 0 | 0 | 0 | 0 | 0 | 0.02727273 | 0.00909091 | 0.27272727 | 0.16363636 | 0 | 0 | 0.08181818 | 0.11818182 | 0.009091 |
| 0 | 0.063636 | 0.136364 | 0.1818182 | 0.0363636 | 0 | 0.1454545 | 0 | 0.1363636 | 0 | 0.1181818 | 0.07272727 | 0 | 0.00909091 | 0.15454545 | 0.1 | 0.08181818 | 0.30909091 | 0 | 0.136364 |
| 0.136364 | 0.254545 | 0.018182 | 0.1363636 | 0.2 | 0.1545455 | 0.1909091 | 0.0363636 | 0.0090909 | 0.1363636 | 0.1454545 | 0.01818182 | 0.03636364 | 0 | 0.07272727 | 0 | 0 | 0 | 0.17272727 | 0.172727 |
| 0 | 0.181818 | 0.145455 | 0 | 0 | 0.1272727 | 0.1090909 | 0.1181818 | 0.3272727 | 0.1545455 | 0.1909091 | 0.24545455 | 0.05454545 | 0.26363636 | 0.07272727 | 0.21818182 | 0.11818182 | 0.20909091 | 0.14545455 | 0.145455 |
| 0.254545 | 0.163636 | 0.072727 | 0.1363636 | 0.1181818 | 0.0727273 | 0 | 0 | 0.1 | 0.1454545 | 0.1818182 | 0.27272727 | 0.30909091 | 0.13636364 | 0.21818182 | 0.21818182 | 0.4 | 0.09090909 | 0.15454545 | 0.036364 |
| 0.227273 | 0.136364 | 0.2 | 0.1818182 | 0.3090909 | 0.2181818 | 0.1909091 | 0.2272727 | 0.2636364 | 0.0090909 | 0 | 0.15454545 | 0.22727273 | 0.22727273 | 0.22727273 | 0 | 0 | 0.37272727 | 0.19090909 | 0.363636 |
| 0 | 0.245455 | 0.263636 | 0.3909091 | 0.2181818 | 0.1818182 | 0.2181818 | 0.2636364 | 0 | 0.3818182 | 0.1727273 | 0.05454545 | 0.03636364 | 0.11818182 | 0.16363636 | 0.26363636 | 0.19090909 | 0 | 0.1 | 0.136364 |
| 0.281818 | 0.109091 | 0.154545 | 0.1 | 0 | 0.1090909 | 0.1363636 | 0.3 | 0.2181818 | 0.2272727 | 0.0272727 | 0.40909091 | 0.06363636 | 0.07272727 | 0.33636364 | 0.12727273 | 0.25454545 | 0.16363636 | 0.2 | 0.090909 |
| 0.236364 | 0.209091 | 0.309091 | 0.3545455 | 0.1363636 | 0.2454545 | 0.0090909 | 0.2090909 | 0.3090909 | 0.0090909 | 0.2545455 | 0.19090909 | 0.11818182 | 0.5 | 0.22727273 | 0.12727273 | 0.18181818 | 0.00909091 | 0.29090909 | 0.2 |
| 0.018182 | 0.027273 | 0.190909 | 0.0090909 | 0.0727273 | 0.3090909 | 0.4 | 0.2818182 | 0.2181818 | 0.3545455 | 0.1272727 | 0.27272727 | 0.32727273 | 0.40909091 | 0.00909091 | 0.13636364 | 0.22727273 | 0.22727273 | 0.12727273 | 0.181818 |
| 0.190909 | 0.036364 | 0 | 0.1181818 | 0.3090909 | 0.1 | 0.1818182 | 0.2545455 | 0 | 0 | 0 | 0.08181818 | 0.03636364 | 0 | 0.10909091 | 0.00909091 | 0 | 0 | 0.3 | 0.190909 |
| 0.009091 | 0.072727 | 0.145455 | 0.1090909 | 0.1 | 0.1909091 | 0 | 0.0545455 | 0.2909091 | 0.3 | 0.3 | 0 | 0 | 0 | 0.01818182 | 0.11818182 | 0.25454545 | 0.19090909 | 0 | 0.081818 |
| 0.3 | 0.181818 | 0.163636 | 0.2272727 | 0.1363636 | 0.1363636 | 0.2636364 | 0.1636364 | 0.1545455 | 0.3818182 | 0 | 0.03636364 | 0.17272727 | 0.1 | 0.4 | 0.09090909 | 0.08181818 | 0.00909091 | 0.15454545 | 0.136364 |
| 0.245455 | 0.118182 | 0.063636 | 0.1090909 | 0.0909091 | 0.1090909 | 0.1181818 | 0.2545455 | 0.0090909 | 0.0909091 | 0.1 | 0.14545455 | 0.1 | 0.14545455 | 0 | 0.06363636 | 0 | 0.15454545 | 0.08181818 | 0.245455 |
| 0 | 0.090909 | 0.136364 | 0.0545455 | 0.0818182 | 0.1090909 | 0.0363636 | 0.2 | 0.1909091 | 0.3727273 | 0.0909091 | 0.07272727 | 0.14545455 | 0.42727273 | 0.06363636 | 0.10909091 | 0.27272727 | 0.01818182 | 0.25454545 | 0.009091 |
| 0 | 0.1 | 0.045455 | 0.2363636 | 0.1454545 | 0.1181818 | 0.3272727 | 0.0909091 | 0.0727273 | 0 | 0.0090909 | 0 | 0.03636364 | 0 | 0.01818182 | 0.00909091 | 0.2 | 0.1 | 0.24545455 | 0.054545 |
| 0.218182 | 0.036364 | 0.145455 | 0.0909091 | 0.1181818 | 0.2272727 | 0.1818182 | 0.3090909 | 0.1272727 | 0.2727273 | 0.1363636 | 0.19090909 | 0.2 | 0 | 0.16363636 | 0.12727273 | 0.10909091 | 0.13636364 | 0.11818182 | 0.172727 |
| 0 | 0.036364 | 0.118182 | 0.2363636 | 0.2272727 | 0.1272727 | 0.1363636 | 0.0090909 | 0.2363636 | 0.1363636 | 0.0636364 | 0.18181818 | 0 | 0.13636364 | 0.02727273 | 0.10909091 | 0.09090909 | 0.12727273 | 0.01818182 | 0.136364 |
| 0.181818 | 0.245455 | 0.163636 | 0.0909091 | 0.1363636 | 0.1090909 | 0.2454545 | 0 | 0.2545455 | 0.0090909 | 0.2181818 | 0.1 | 0.26363636 | 0.35454545 | 0.09090909 | 0.10909091 | 0.11818182 | 0.08181818 | 0.16363636 | 0.109091 |
| 0 | 0.154545 | 0.181818 | 0.0090909 | 0.1272727 | 0.0363636 | 0.1272727 | 0.0454545 | 0 | 0 | 0 | 0.10909091 | 0.01818182 | 0.08181818 | 0 | 0.09090909 | 0.13636364 | 0.09090909 | 0.14545455 | 0.163636 |
| 0 | 0.009091 | 0.172727 | 0.2545455 | 0.2 | 0.1545455 | 0.1 | 0 | 0.1363636 | 0.1363636 | 0.0090909 | 0.1 | 0.17272727 | 0 | 0.13636364 | 0.24545455 | 0 | 0.09090909 | 0.20909091 | 0.254545 |
| 0.163636 | 0.190909 | 0.090909 | 0.0090909 | 0.1545455 | 0.1090909 | 0.1181818 | 0.0181818 | 0.0090909 | 0.0454545 | 0.1636364 | 0.11818182 | 0.08181818 | 0.19090909 | 0.14545455 | 0.20909091 | 0.04545455 | 0.13636364 | 0.18181818 | 0.009091 |
| | | 0.136364 | 0.1454545 | 0 | 0.1636364 | 0 | 0.0727273 | 0.1454545 | 0.0818182 | 0 | 0 | 0.04545455 | 0.21818182 | 0.04545455 | 0.00909091 | | | 0.05454545 | 0.118182 |
| | | 0.145455 | 0.0636364 | 0.0727273 | 0 | 0.0181818 | 0 | 0.0636364 | 0.0363636 | 0.1090909 | 0.14545455 | 0.00909091 | 0.03636364 | 0.08181818 | | | | 0.05454545 | 0.172727 |
| | | 0.045455 | 0 | 0.3 | 0.1363636 | 0.1909091 | 0.0545455 | 0.1818182 | 0.0727273 | 0.0181818 | 0.10909091 | 0.06363636 | 0.00909091 | 0.21818182 | | | | 0.20909091 | 0.163636 |
| | | 0.054545 | 0.1727273 | 0 | 0.1545455 | 0.0727273 | 0.1454545 | 0.1090909 | 0.2272727 | 0.1909091 | 0.03636364 | 0.17272727 | 0.15454545 | 0.24545455 | | | | 0.00909091 | 0 |
| | | 0.118182 | 0.1 | 0.1545455 | 0.1090909 | 0.2181818 | 0 | 0.0545455 | 0 | 0.1545455 | 0.12727273 | 0.00909091 | 0.07272727 | 0.01818182 | | | | 0.26363636 | 0.254545 |
| | | 0.145455 | 0.1545455 | 0.1090909 | 0.1181818 | 0.1454545 | 0.0181818 | 0 | 0.2272727 | 0.0454545 | 0.10909091 | 0.29090909 | 0.49090909 | 0.28181818 | | | | 0.23636364 | 0.218182 |
| | | 0.163636 | 0.1636364 | 0.1818182 | 0.1636364 | 0.1272727 | 0.2818182 | 0.2272727 | 0.1363636 | 0.0454545 | 0.20909091 | 0 | 0.26363636 | 0.48181818 | | | | 0.1 | 0.254545 |
| | | 0.109091 | 0.3454545 | 0.1090909 | 0.1909091 | 0.1818182 | 0.4 | 0.1272727 | 0.2363636 | 0.2545455 | 0.4 | 0.31818182 | 0 | 0.00909091 | | | | 0.27272727 | 0.018182 |
| | | 0.136364 | 0.1454545 | 0.1909091 | 0.3363636 | 0.2272727 | 0.2181818 | 0.2 | 0.2727273 | 0.1272727 | 0.13636364 | 0.09090909 | 0 | 0.09090909 | | | | 0.23636364 | 0.109091 |
| | | 0.054545 | 0.2181818 | 0 | 0.0454545 | 0 | 0.2636364 | 0 | 0.1636364 | 0.0727273 | 0.16363636 | 0.05454545 | 0.28181818 | 0.24545455 | | | | 0.30909091 | 0.109091 |
| | | 0.190909 | 0.1454545 | 0.2636364 | 0.1090909 | 0.2090909 | 0.1545455 | 0.4090909 | 0.1909091 | 0.1090909 | 0.12727273 | 0.24545455 | 0 | 0 | | | | 0.18181818 | 0.181818 |
| | | 0.045455 | 0 | 0.1363636 | 0.2 | 0.2454545 | 0.0818182 | 0.2272727 | 0.1727273 | 0.1818182 | 0.17272727 | 0 | 0.12727273 | 0.23636364 | | | | 0.23636364 | 0.145455 |
| | | 0.145455 | 0.2181818 | 0.0181818 | 0.1818182 | 0 | 0.3 | 0.0090909 | 0.3727273 | 0 | 0.16363636 | 0.06363636 | 0.13636364 | 0.11818182 | | | | 0 | 0.163636 |
| | | 0.154545 | 0.2363636 | 0.2181818 | 0.0272727 | 0.1454545 | 0.0090909 | 0.2363636 | 0 | 0.0363636 | 0.19090909 | 0.00909091 | 0 | 0.06363636 | | | | 0.05454545 | 0.027273 |
| | | 0.118182 | 0.1181818 | 0.1545455 | 0.0363636 | 0.1545455 | 0.3727273 | 0.1090909 | 0.2090909 | 0.0727273 | 0.2 | 0.08181818 | 0.13636364 | 0.15454545 | | | | 0.06363636 | 0.154545 |
| | | 0.1 | 0.1454545 | 0.1363636 | 0.2 | 0.2636364 | 0 | 0.0090909 | 0.1818182 | 0.2 | 0.00909091 | 0.03636364 | 0.1 | 0.01818182 | | | | 0.14545455 | 0.290909 |
| | | 0.118182 | 0.2181818 | 0.1818182 | 0.1545455 | 0.1181818 | 0.1363636 | 0.1090909 | 0.1272727 | 0.1727273 | 0.04545455 | 0.18181818 | 0.32727273 | 0.33636364 | | | | 0.32727273 | 0.072727 |
| | | 0.1 | 0.2181818 | 0.1272727 | 0.1272727 | 0.1 | 0.1545455 | 0.3272727 | 0.1727273 | 0.1272727 | 0.21818182 | 0.03636364 | 0.24545455 | 0 | | | | 0.2 | 0.245455 |
| | | 0.118182 | 0.1 | 0.2909091 | 0.0545455 | 0.2727273 | 0 | 0.1363636 | 0.1090909 | 0.1272727 | 0.07272727 | 0 | 0.06363636 | 0.10909091 | | | | 0.09090909 | 0.190909 |
| | | 0.190909 | 0.1181818 | 0.1090909 | 0.1545455 | 0 | 0.1727273 | 0.1090909 | 0.0909091 | 0.2 | 0.03636364 | 0.24545455 | 0 | 0.15454545 | | | | 0.1 | 0.209091 |
| | | 0.109091 | 0 | 0.1818182 | 0.1272727 | 0.1818182 | 0 | 0.0454545 | 0 | 0 | 0.09090909 | 0.09090909 | 0.22727273 | 0 | | | | 0.12727273 | 0.081818 |
| | | 0.1 | 0.1090909 | 0.1 | 0.1181818 | 0.1090909 | 0 | 0.1818182 | 0.1181818 | 0.2090909 | 0.12727273 | 0.03636364 | 0 | 0.18181818 | | | | 0 | 0.009091 |
| | | 0.018182 | | | | 0.0818182 | 0 | 0 | 0 | 0.0090909 | 0.00909091 | 0.1 | 0 | 0 | | | | 0.08181818 | |

| | | | | | | | | | | | | | | |
|---|---|---|---|---|---|---|---|---|---|---|---|---|---|---|
| 9 | 2 | 2 | 5 | 8 | 6 | 6 | 9 | 9 | 11 | 11 | 12 | 11 | 3 | 10 |
| Box-8 | Box-1 | Box-5 | Box-2 | Box-6 | Box-5 | Box-7 | Box-4 | Box-5 | Box-3 | Box-7 | Box-8 | Box-8 | Box-8 | Box-8 |
| 75 | 13.7 | 20.2 | 32.3 | 44.5 | 45 | 47 | 65 | 68 | 78 | 84 | Z | Z | Z | Z |
| M | M | M | M | M | M | M | M | M | M | M | Z | Z | Z | Z |
| Malto | MCT | MCT | MCT | MCT | MCT | MCT | MCT | MCT | MCT | MCT | Z | Z | Z | Z |
| MM | MT | MT | MT | MT | MT | MT | MT | MT | MT | MT | Z | Z | Z | Z |
| | | | | | | | | | | | | | | |
| 0 | 0 | 0 | 0 | 0 | 0.054545 | 0.054545 | 0 | 0 | 0 | 0 | | | | |
| 0 | 0 | 0 | 0 | 0 | 0.236364 | 0.354545 | 0 | 0 | 0 | 0 | | | | |
| 0 | 0 | 0 | 0.081818 | 0 | 0.354545 | 0.381818 | 0.076667 | 0 | 0 | 0 | | | | |
| 0 | 0.281818 | 0.063636 | 0.218182 | 0.6 | 0.545455 | 0.545455 | 0.006667 | 0 | 0.136364 | 0 | | | | |
| 0 | 0.481818 | 0.218182 | 0.409091 | 0.472727 | 0.327273 | 0.190909 | 0.12 | 0.053333 | 0.509091 | 0.227273 | | | | |
| 0 | 0.527273 | 0.236364 | 0.218182 | 0.690909 | 0 | 0.327273 | 0.12 | 0.043333 | 0.472727 | 0.090909 | | | | |
| 0.163333 | 0 | 0.127273 | 0.436364 | 0.345455 | 0.363636 | 0.2 | 0.196667 | 0.076667 | 0.772727 | 0.181818 | | | | |
| 0.03 | 0.027273 | 0.063636 | 0.672727 | 0.254545 | 0 | 0.163636 | 0.026667 | 0.1 | 0 | 0.045455 | | | | |
| 0.133333 | 0.109091 | 0.309091 | 0 | 0 | 0.1 | 0.290909 | 0.053333 | 0 | 0 | 0.036364 | | | | |
| 0.03 | 0.354545 | 0 | 0.309091 | 0.163636 | 0.318182 | 0.045455 | 0.023333 | 0 | 0.372727 | 0 | | | | |
| 0.056667 | 0 | 0 | 0.3 | 0.236364 | 0 | 0.227273 | 0.04 | 0.043333 | 0 | 0 | | | | |
| 0.06 | 0.172727 | 0 | 0.181818 | 0.018182 | 0.263636 | 0.454545 | 0.163333 | 0.026667 | 0.254545 | 0.036364 | | | | |
| 0.07 | 0.072727 | 0 | 0.181818 | 0.118182 | 0 | 0 | 0.04 | 0.016667 | 0.245455 | 0.009091 | | | | |
| 0.19 | 0.527273 | 0 | 0 | 0 | 0.4 | 0.009091 | 0 | 0 | 0.254545 | 0.109091 | | | | |
| 0 | 0.045455 | 0 | 0.118182 | 0.1 | 0.009091 | 0 | 0.096667 | 0.006667 | 0 | 0 | | | | |
| 0.023333 | 0 | 0 | 0.190909 | 0.136364 | 0.290909 | 0.018182 | 0 | 0.06 | 0.027273 | 0.009091 | | | | |
| 0.016667 | 0.018182 | 0 | 0 | 0.109091 | 0.063636 | 0.145455 | 0.046667 | 0.013333 | 0 | 0.081818 | | | | |
| 0.056667 | 0.263636 | 0 | 0 | 0.018182 | 0 | 0.163636 | 0.046667 | 0 | 0.127273 | 0 | | | | |
| 0 | 0 | 0 | 0.127273 | 0 | 0 | 0 | 0.04 | 0.04 | 0.009091 | 0.081818 | | | | |
| 0.096667 | 0.245455 | 0.163636 | 0 | 0 | 0.063636 | 0.227273 | 0 | 0 | 0.063636 | 0.009091 | | | | |
| 0 | 0 | 0 | 0.181818 | 0.309091 | 0.272727 | 0.354545 | 0.04 | 0.043333 | 0 | 0.109091 | | | | |
| 0.046667 | 0.236364 | 0 | 0 | 0.218182 | 0.245455 | 0.254545 | 0.12 | 0 | 0.1 | 0 | | | | |
| 0.076667 | 0 | 0 | 0.190909 | 0.045455 | 0 | 0.172727 | 0 | 0.023333 | 0 | 0.063636 | | | | |
| 0.093333 | 0.309091 | 0.2 | 0.563636 | 0.236364 | 0.281818 | 0.463636 | 0.156667 | 0.093333 | 0 | 0.072727 | | | | |
| 0.046667 | 0.4 | 0.181818 | 0.254545 | 0.454545 | 0.381818 | 0.572727 | 0.06 | 0.006667 | 0.072727 | 0.190909 | | | | |
| 0.13 | 0.3 | 0.118182 | 0.118182 | 0.536364 | 0.372727 | 0.245455 | 0.036667 | 0.073333 | 0.254545 | 0.154545 | | | | |
| 0.143333 | 0.363636 | 0.654545 | 0.645455 | 0.763636 | 0.363636 | 0.536364 | 0.053333 | 0.083333 | 0.481818 | 0.054545 | | | | |
| 0.18 | 0.163636 | 0.372727 | 0.4 | 0.554545 | 0.381818 | 0 | 0.133333 | 0.036667 | 0.354545 | 0.309091 | | | | |
| 0.11 | 0.554545 | 0.481818 | 0.163636 | 0.136364 | 0.036364 | 0.372727 | 0.076667 | 0.07 | 0.445455 | 0.009091 | | | | |
| 0.03 | 0.245455 | 0.318182 | 0.481818 | 0.272727 | 0 | 0.072727 | 0.01 | 0.056667 | 0 | 0.354545 | | | | |
| 0.043333 | 0 | 0 | 0.263636 | 0.1 | 0.245455 | 0.327273 | 0.046667 | 0.133333 | 0.318182 | 0.345455 | | | | |
| 0.093333 | 0.272727 | 0 | 0 | 0.190909 | 0 | 0 | 0 | 0.033333 | 0.290909 | 0 | | | | |
| 0.093333 | 0 | 0.3 | 0.1 | 0.236364 | 0.254545 | 0.236364 | 0.046667 | 0.053333 | 0 | 0 | | | | |
| 0.053333 | 0.072727 | 0 | 0.245455 | 0 | 0 | 0.154545 | 0.07 | 0 | 0.136364 | 0.354545 | | | | |
| 0.116667 | 0.218182 | 0.318182 | 0.245455 | 0.272727 | 0 | 0 | 0.026667 | 0.103333 | 0.090909 | 0 | | | | |
| 0.016667 | 0.227273 | 0 | 0 | 0.054545 | 0.309091 | 0.190909 | 0 | 0.01 | 0.009091 | 0 | | | | |
| 0.006667 | 0.027273 | 0 | 0.009091 | 0.009091 | 0 | 0.109091 | 0.096667 | 0.04 | 0.072727 | 0.027273 | | | | |
| 0.003333 | 0 | 0 | 0.2 | 0.254545 | 0 | 0 | 0.043333 | 0.02 | 0 | 0 | | | | |
| 0.063333 | 0.018182 | 0.054545 | 0 | 0.245455 | | | 0 | 0 | 0.045455 | 0.281818 | | | | |
| 0.043333 | 0.009091 | 0 | | 0 | | | 0.05 | 0.07 | 0 | 0 | | | | |
| 0.1 | 0.272727 | 0 | | 0.163636 | | | 0.006667 | 0 | 0 | 0 | | | | |
| 0 | 0 | 0 | | 0.1 | | | 0.063333 | 0.093333 | 0.018182 | 0.190909 | | | | |
| 0.103333 | 0.2 | 0.427273 | | 0.427273 | | | 0.1 | 0.016667 | 0.045455 | 0.009091 | | | | |
| 0.083333 | 0.054545 | 0 | | 0 | | | 0.056667 | 0.07 | 0.027273 | 0.463636 | | | | |
| 0.046667 | 0.3 | 0.227273 | | 0.254545 | | | 0 | 0.073333 | 0.009091 | 0 | | | | |
| 0.103333 | 0.318182 | 0.127273 | | 0.609091 | | | 0.11 | 0.09 | 0.109091 | 0.236364 | | | | |
| 0.08 | 0.518182 | 0.409091 | | 0.290909 | | | 0.133333 | 0.146667 | 0.654545 | 0.463636 | | | | |
| 0.15 | 0.190909 | 0.290909 | | 0.618182 | | | 0.056667 | 0.12 | 0.227273 | 0.309091 | | | | |
| 0.12 | 0.345455 | 0.645455 | | 0.227273 | | | 0.113333 | 0 | 0.454545 | 0.063636 | | | | |
| 0.11 | 0.045455 | 0.236364 | | 0.236364 | | | 0.126667 | 0.106667 | 0.245455 | 0.672727 | | | | |
| 0.13 | 0.318182 | 0.072727 | | 0.418182 | | | 0.03 | 0 | 0.472727 | 0 | | | | |
| 0.013333 | 0.272727 | 0.181818 | | 0.427273 | | | 0.033333 | 0.096667 | 0.254545 | 0.309091 | | | | |
| 0 | 0.263636 | 0.190909 | | 0.245455 | | | 0.076667 | 0.006667 | 0 | 0 | | | | |
| 0.126667 | 0.009091 | 0 | | 0.309091 | | | 0.066667 | 0 | 0.290909 | 0.009091 | | | | |
| 0.003333 | 0 | 0.045455 | | 0 | | | 0.056667 | 0.106667 | 0 | 0.427273 | | | | |
| 0.076667 | 0.3 | 0.2 | | 0.336364 | | | 0.003333 | 0 | 0.318182 | 0 | | | | |
| 0.046667 | 0.236364 | 0.181818 | | 0.054545 | | | 0.07 | 0.06 | 0 | 0 | | | | |
| 0 | 0 | 0 | | 0.018182 | | | 0 | 0 | 0.372727 | 0 | | | | |
| 0 | 0.209091 | 0.072727 | | 0 | | | 0.04 | 0.026667 | 0.009091 | 0.2 | | | | |
| 0.006667 | 0.081818 | 0.136364 | | 0 | | | 0.043333 | 0 | 0.563636 | 0 | | | | |
| 0 | 0.154545 | 0.181818 | | 0.063636 | | | 0.006667 | 0.05 | | | | | | |
| 0.006667 | | | | | | | 0.056667 | 0 | | | | | | |
| | | | | | | | | | | | | | | |
| 0.281818 | 0.418182 | 0.181818 | 0 | 0.236364 | 0.218182 | 0.245455 | 0.072727 | 0.481818 | 0.390909 | 0.181818 | | | | |
| 0 | 0.390909 | 0.418182 | 0.363636 | 0.445455 | 0.181818 | 0.281818 | 0.054545 | 0.036364 | 0.009091 | 0.390909 | | | | |
| 0.518182 | 0.254545 | 0.445455 | 0.336364 | 0.336364 | 0.272727 | 0.181818 | 0.218182 | 0.563636 | 0.354545 | 0.209091 | | | | |
| 0 | 0.109091 | 0.290909 | 0.209091 | 0.345455 | 0.145455 | 0.445455 | 0.190909 | 0.090909 | 0 | 0.390909 | | | | |
| 0.327273 | 0.027273 | 0.209091 | 0.281818 | 0.027273 | 0.5 | 0.018182 | 0.290909 | 0.218182 | 0.527273 | 0 | | | | |
| 0.536364 | 0.072727 | 0.254545 | 0.245455 | 0.236364 | 0 | 0.245455 | 0.027273 | 0.372727 | 0 | 0.290909 | | | | |
| 0 | 0.5 | 0 | 0.090909 | 0.036364 | 0 | 0.027273 | 0.636364 | 0.145455 | 0.1 | 0 | | | | |
| 0.118182 | 0.127273 | 0.1 | 0.027273 | 0.072727 | 0.281818 | 0.5 | 0 | 0.145455 | 0.372727 | 0 | | | | |
| 0.154545 | 0 | 0.009091 | 0.136364 | 0.045455 | 0 | 0.236364 | 0.172727 | 0.363636 | 0.154545 | 0.363636 | | | | |
| 0.072727 | 0.154545 | 0.490909 | 0 | 0.109091 | 0.245455 | 0 | 0 | 0.018182 | 0 | 0 | | | | |
| 0.136364 | 0.145455 | 0 | 0.209091 | 0.263636 | 0.036364 | 0.490909 | 0.109091 | 0.181818 | 0 | 0.172727 | | | | |
| 0.209091 | 0.236364 | 0.254545 | 0.263636 | 0.090909 | 0.163636 | 0 | 0.3 | 0.018182 | 0.127273 | 0.018182 | | | | |
| 0.1 | 0.172727 | 0.009091 | 0.336364 | 0.227273 | 0.218182 | 0 | 0.409091 | 0.145455 | 0.3 | 0.327273 | | | | |
| 0.281818 | 0.172727 | 0.109091 | 0 | 0.081818 | 0.354545 | 0.236364 | 0.154545 | 0.245455 | 0.2 | 0 | | | | |
| 0.118182 | 0.090909 | 0 | 0.027273 | 0.081818 | 0.045455 | 0.009091 | 0.027273 | 0 | 0.009091 | 0.045455 | | | | |
| 0.181818 | 0.209091 | 0.290909 | 0 | 0 | 0.209091 | 0.190909 | 0 | 0.118182 | 0.027273 | 0.336364 | | | | |
| 0.009091 | 0.181818 | 0 | 0 | 0 | 0.154545 | 0.336364 | 0 | 0.281818 | 0.027273 | 0.236364 | | | | |
| 0.227273 | 0.109091 | 0.009091 | 0.018182 | 0.009091 | 0.145455 | 0.172727 | 0.018182 | 0.027273 | 0.236364 | 0 | | | | |
| 0.127273 | 0.281818 | 0 | 0.054545 | 0 | 0.218182 | 0.190909 | 0.090909 | 0.018182 | 0.027273 | 0 | | | | |
| 0 | 0.254545 | 0.3 | 0.090909 | 0.009091 | 0 | 0.118182 | 0.136364 | 0.009091 | 0 | 0.518182 | | | | |
| 0.3 | 0.127273 | 0 | 0.1 | 0.272727 | 0.181818 | 0.354545 | 0 | 0.154545 | 0.081818 | 0.018182 | | | | |
| 0.518182 | 0.045455 | 0.036364 | 0.072727 | 0 | 0.554545 | 0.3 | 0 | 0.172727 | 0.218182 | 0.345455 | | | | |
| 0.009091 | 0.263636 | 0.090909 | 0.172727 | 0.145455 | 0 | 0.318182 | 0.227273 | 0.136364 | 0.272727 | 0.009091 | | | | |
| 0.163636 | 0.6 | 0.354545 | 0.945455 | 0.5 | 0.272727 | 0.081818 | 0.209091 | 0.718182 | 0.090909 | 0.027273 | | | | |
| 0.054545 | 0.118182 | 0.390909 | 0.272727 | 0.6 | 0 | 0.172727 | 0 | 0.072727 | 0.209091 | 0.590909 | | | | |
| 0.490909 | 0.527273 | 0.209091 | 0.009091 | 0.572727 | 0.290909 | 0.190909 | 0.4 | 0.227273 | 0.372727 | 0.4 | | | | |
| 0 | 0.018182 | 0.1 | 0.018182 | 0 | 0.481818 | 0.336364 | 0.345455 | 0.327273 | 0.418182 | 0 | | | | |
| 0.490909 | 0 | 0.154545 | 0.136364 | 0.018182 | 0.036364 | 0.563636 | 0 | 0.145455 | 0 | 0.227273 | | | | |
| 0.345455 | 0 | 0.445455 | 0.081818 | 0.3 | 0.309091 | 0 | 0.018182 | 0.263636 | 0.363636 | 0.472727 | | | | |
| 0.372727 | . | 0.063636 | 0.190909 | 0.045455 | 0 | 0 | 0.209091 | 0 | 0.472727 | 0 | | | | |
| 0 | . | 0.036364 | 0.109091 | 0.363636 | 0.136364 | 0 | 0.309091 | 0.281818 | 0 | 0.5 | | | | |
| 0.445455 | . | 0.372727 | 0 | 0.327273 | 0 | 0.209091 | 0.6 | 0.027273 | 0 | 0.036364 | | | | |
| 0 | . | 0 | 0.327273 | 0.009091 | 0.490909 | 0.154545 | 0 | 0.463636 | 0 | 0 | | | | |
| 0 | . | 0.181818 | 0.018182 | 0.436364 | 0.118182 | 0.063636 | 0 | 0.145455 | 0.609091 | 0.118182 | | | | |
| 0 | . | 0.009091 | 0.409091 | 0 | 0.118182 | 0.109091 | 0.009091 | 0 | 0 | 0.145455 | | | | |
| 0 | . | 0.609091 | 0.354545 | 0 | 0.154545 | 0.181818 | #VALUE! | 0.354545 | 0 | 0.009091 | | | | |
| 0 | . | 0 | 0.327273 | 0 | 0.254545 | 0.172727 | #VALUE! | 0.218182 | 0.036364 | 0.009091 | | | | |
| 0.009091 | . | 0 | 0.327273 | 0.018182 | 0 | 0.109091 | #VALUE! | 0.018182 | 0.154545 | 0.018182 | | | | |
| 0.127273 | . | 0 | 0 | 0.054545 | 0.054545 | 0.154545 | #VALUE! | 0.145455 | 0 | 0.018182 | | | | |

| | | | | | | | | | | |
|---|---|---|---|---|---|---|---|---|---|---|
| 0.190909 | . | 0.072727 | 0.218182 | 0.045455 | | | #VALUE! | 0.281818 | 0.409091 | 0.372727 |
| 0 | . | 0 | | 0.072727 | | | #VALUE! | 0.054545 | 0 | 0 |
| 0.045455 | . | 0.2 | | 0.181818 | | | #VALUE! | 0 | 0.027273 | 0.327273 |
| 0.327273 | . | 0 | | 0.036364 | | | #VALUE! | 0.018182 | 0.4 | 0 |
| 0.318182 | . | 0.363636 | | 0.127273 | | | #VALUE! | 0.190909 | 0 | 0.445455 |
| 0 | . | 0.172727 | | 0.027273 | | | #VALUE! | 0.154545 | 0.009091 | 0 |
| 0.409091 | . | 0.545455 | | 0.163636 | | | 0 | 0.218182 | 0.009091 | 0.454545 |
| 0.554545 | . | 0.6 | | 0.163636 | | | 0.009091 | 0.236364 | 0.436364 | 0 |
| 0.009091 | . | 0 | | 0.345455 | | | 0.081818 | 0.309091 | 0 | 0.672727 |
| 0.354545 | . | 0.318182 | | 0.090909 | | | 0.063636 | 0.409091 | 0.145455 | 0 |
| 0 | . | 0 | | 0.109091 | | | 0.172727 | 0 | 0 | 0 |
| 0 | . | 0 | | 0.445455 | | | 0 | 0 | 0.009091 | 0.409091 |
| 0.063636 | . | 0 | | 0.327273 | | | 0.063636 | 0.836364 | 0 | 0 |
| 0.081818 | . | 0.145455 | | 0 | | | 0.190909 | 0 | 0.009091 | 0.118182 |
| 0.145455 | . | 0 | | 0.336364 | | | 0.154545 | 0.445455 | 0.018182 | 0.063636 |
| 0 | . | 0 | | 0 | | | 0.245455 | 0.009091 | 0.209091 | 0.154545 |
| 0.045455 | . | 0 | | 0.172727 | | | 0 | 0.009091 | 0 | 0.181818 |
| 0.272727 | . | 0.1 | | 0 | | | 0.309091 | 0 | 0.027273 | 0.009091 |
| 0.254545 | . | 0 | | 0 | | | 0.027273 | 0.045455 | 0.109091 | 0.309091 |
| 0.090909 | . | 0 | | 0 | | | 0.009091 | 0 | 0.036364 | 0 |
| 0.090909 | . | 0 | | 0.381818 | | | 0.336364 | 0 | 0 | 0.281818 |
| 0 | | | | 0 | | | 0 | 0 | 0.009091 | 0.072727 |

| | | | | | | | | | | |
|---|---|---|---|---|---|---|---|---|---|---|
| 0.354545 | 0.563636 | 0.109091 | 0.109091 | 0.509091 | 0.2 | 0.2 | 0.318182 | 0.254545 | 0.354545 | . |
| 0.1 | 0.163636 | 0.2 | 0.109091 | 0.427273 | 0 | 0.036364 | 0.218182 | 0 | 0.045455 | . |
| 0.245455 | 0.163636 | 0.209091 | 0.263636 | 0.336364 | 0.063636 | 0.309091 | 0.063636 | 0 | 0.345455 | . |
| 0.272727 | 0.145455 | 0.436364 | 0.109091 | 0.009091 | 0.154545 | 0.118182 | 0.154545 | 0 | 0.190909 | . |
| 0.045455 | 0.227273 | 0.254545 | 0.218182 | 0.272727 | 0 | 0.045455 | 0.1 | 0 | 0.172727 | . |
| 0.227273 | 0 | 0.063636 | 0 | 0.181818 | 0.209091 | 0.181818 | 0.163636 | 0 | 0.281818 | . |
| 0.363636 | 0.181818 | 0.163636 | 0.290909 | 0.181818 | 0 | 0.190909 | 0.072727 | 0 | 0.227273 | . |
| 0.009091 | 0.181818 | 0.154545 | 0.063636 | 0 | 0.090909 | 0.009091 | 0.018182 | 0 | 0 | . |
| 0.009091 | 0.009091 | 0.236364 | 0 | 0.036364 | 0.2 | 0.009091 | 0.127273 | 0 | 0.109091 | . |
| 0.190909 | 0.363636 | 0.027273 | 0.172727 | 0 | 0 | 0 | 0.154545 | 0.318182 | 0 | . |
| 0 | 0 | 0.081818 | 0.118182 | 0.136364 | 0 | 0.118182 | 0.063636 | 0 | 0.318182 | . |
| 0.054545 | 0.036364 | 0.045455 | 0.136364 | 0.127273 | 0 | 0.209091 | 0.045455 | 0 | 0 | . |
| 0.081818 | 0 | 0.254545 | 0.227273 | 0.009091 | 0.181818 | 0.218182 | 0.090909 | 0.163636 | 0.109091 | . |
| 0.054545 | 0.018182 | 0.318182 | 0.172727 | 0 | 0 | 0.018182 | 0.181818 | 0.218182 | 0.209091 | . |
| 0.1 | 0.054545 | 0 | 0.090909 | 0.336364 | 0.072727 | 0 | 0 | 0 | 0 | . |
| 0.2 | 0.090909 | 0.154545 | 0.009091 | 0 | 0.209091 | 0.263636 | 0.018182 | 0 | 0.181818 | . |
| 0 | 0.363636 | 0 | 0.081818 | 0.172727 | 0.018182 | 0 | 0.136364 | 0 | 0 | . |
| 0.009091 | 0.190909 | 0 | 0.118182 | 0.072727 | 0.254545 | 0.209091 | 0.054545 | 0 | 0.209091 | . |
| 0.109091 | 0.009091 | 0.127273 | 0.081818 | 0.181818 | 0.154545 | 0.2 | 0.109091 | 0 | 0 | . |
| 0.1 | 0.218182 | 0.2 | 0.345455 | 0.172727 | 0 | 0.109091 | 0.218182 | 0 | 0.218182 | . |
| 0.145455 | 0.245455 | 0.209091 | 0 | 0 | 0.245455 | 0.281818 | 0.072727 | 0 | 0.227273 | . |
| 0.327273 | 0 | 0.190909 | 0.172727 | 0.218182 | 0.181818 | 0.009091 | 0.2 | 0.009091 | 0.372727 | . |
| 0.245455 | 0.354545 | 0.163636 | 0 | 0 | 0 | 0.209091 | 0.281818 | 0.672727 | 0 | . |
| 0 | 0.027273 | 0.1 | 0.072727 | 0.372727 | 0.009091 | 0.181818 | 0.081818 | 0 | 0.390909 | . |
| 0.072727 | 0.145455 | 0.463636 | 0.109091 | 0.245455 | 0.527273 | 0 | 0.218182 | 0.918182 | 0 | . |
| 0.327273 | 0.045455 | 0.227273 | 0.227273 | 0.109091 | 0 | 0.136364 | 0.127273 | 0 | 0.527273 | . |
| 0 | 0.127273 | 0 | 0 | 0.109091 | 0 | 0.1 | 0.1 | 0 | 0 | . |
| 0.236364 | 0 | 0.309091 | 0.118182 | 0.027273 | 0.063636 | 0 | 0.063636 | 0.263636 | 0.009091 | . |
| 0 | 0.136364 | 0.054545 | 0.072727 | 0.009091 | 0 | 0.281818 | 0.1 | 0.418182 | 0.109091 | . |
| 0.154545 | 0.145455 | 0.154545 | 0.009091 | 0.2 | 0.190909 | 0 | 0.145455 | 0 | 0.2 | . |
| 0.127273 | 0.090909 | 0.163636 | 0.309091 | 0.027273 | 0 | 0.027273 | 0.245455 | 0 | 0.118182 | . |
| 0 | 0.009091 | 0.172727 | 0 | 0.081818 | 0 | 0.2 | 0.018182 | 0.190909 | 0 | . |
| 0.190909 | 0.109091 | 0.181818 | 0.127273 | 0 | 0.090909 | 0 | 0.018182 | 0 | 0.209091 | . |
| 0.190909 | 0.145455 | 0.009091 | 0.018182 | 0.090909 | 0.318182 | 0 | 0.2 | 0 | 0.118182 | . |
| 0.172727 | 0 | 0.190909 | 0.345455 | 0 | 0.254545 | 0.4 | 0 | 0.127273 | 0.018182 | . |
| 0.190909 | 0.154545 | 0.045455 | 0.190909 | 0 | 0 | 0.154545 | 0.236364 | 0.009091 | 0.227273 | . |
| 0 | 0.127273 | 0.009091 | 0.154545 | 0.427273 | 0.009091 | 0.127273 | 0.154545 | 0 | 0.209091 | . |
| 0.181818 | 0.081818 | 0 | 0 | 0.181818 | 0.227273 | 0 | 0 | 0.127273 | 0.009091 | . |
| 0.127273 | 0.118182 | 0.1 | 0.218182 | 0 | 0 | 0.218182 | 0.090909 | 0 | 0.036364 | . |
| 0.190909 | 0.063636 | 0.1 | 0.136364 | 0 | 0 | 0.181818 | 0 | 0 | 0.081818 | . |
| 0.009091 | 0.127273 | 0.027273 | 0 | 0.354545 | 0.118182 | 0.190909 | 0.018182 | 0.045455 | 0.109091 | . |
| 0.109091 | 0.172727 | 0.009091 | 0.1 | 0 | 0.345455 | 0.218182 | 0.236364 | 0 | 0.072727 | . |
| 0.172727 | 0.263636 | 0.218182 | 0.154545 | 0.463636 | 0.063636 | 0.218182 | 0.154545 | 0.345455 | 0.445455 | . |
| 0.409091 | 0.063636 | 0.345455 | 0.227273 | 0.3 | 0 | 0.018182 | 0.081818 | 0.009091 | 0 | . |
| 0.1 | 0.2 | 0.072727 | 0.145455 | 0 | 0.327273 | 0.018182 | 0.236364 | 0.481818 | 0.245455 | . |
| 0.009091 | 0.327273 | 0.527273 | 0.127273 | 0.263636 | 0.009091 | 0.254545 | 0.109091 | 0.009091 | 0.263636 | . |
| 0.263636 | 0.172727 | 0.154545 | 0.281818 | 0.363636 | 0 | 0 | 0.2 | 0.672727 | 0.1 | . |
| 0.418182 | 0.081818 | 0.009091 | 0 | 0.009091 | 0.263636 | 0.154545 | 0.009091 | 0 | 0.245455 | . |
| 0.154545 | 0.154545 | 0.345455 | 0.081818 | 0 | 0.027273 | 0.054545 | 0.190909 | 0.218182 | 0 | . |
| 0.009091 | 0.163636 | 0.118182 | 0.1 | 0.009091 | 0 | 0 | 0.127273 | 0.427273 | 0.254545 | . |
| 0.145455 | 0.2 | 0.172727 | 0.145455 | 0.136364 | 0 | 0.2 | 0.172727 | 0.063636 | 0.009091 | . |
| 0.072727 | 0.127273 | 0.163636 | 0.1 | 0.354545 | 0.136364 | 0.109091 | 0.109091 | 0.181818 | 0.136364 | . |
| 0.172727 | 0 | 0.236364 | 0.009091 | 0.009091 | 0.036364 | 0 | 0.118182 | 0.263636 | 0.090909 | . |
| 0.290909 | 0.136364 | 0.163636 | 0.190909 | 0 | 0 | 0.172727 | 0.209091 | 0 | 0.190909 | . |
| 0 | 0.1 | 0 | 0.027273 | 0.281818 | 0.136364 | 0.081818 | 0.154545 | 0 | 0.054545 | . |
| 0.2 | 0.045455 | 0.009091 | 0.327273 | 0.009091 | 0 | 0.190909 | 0.009091 | 0.281818 | 0.036364 | . |
| 0.036364 | 0.181818 | 0.254545 | 0.190909 | 0 | 0.145455 | 0.281818 | 0.009091 | 0.1 | 0.290909 | . |
| 0.136364 | 0.054545 | 0 | 0.054545 | 0.390909 | 0.127273 | 0 | 0.190909 | 0.218182 | 0.236364 | . |
| 0.072727 | 0.136364 | 0.181818 | 0.090909 | 0.045455 | 0.036364 | 0.109091 | 0.190909 | 0.090909 | 0.136364 | . |
| 0.181818 | 0.154545 | 0.009091 | 0.072727 | 0 | 0 | 0.045455 | 0.1 | 0 | 0.027273 | . |
| 0.009091 | 0.072727 | 0.009091 | 0.109091 | 0.145455 | | | 0.063636 | 0 | 0.072727 | . |
| 0.090909 | 0.072727 | 0.209091 | | 0 | | | 0.154545 | 0.090909 | 0.2 | . |
| 0.154545 | 0.136364 | 0.118182 | | 0.254545 | | | 0.018182 | 0 | 0 | . |
| 0.172727 | 0.1 | 0.127273 | | 0 | | | 0.072727 | 0.2 | 0.136364 | . |
| 0.318182 | 0.109091 | 0.136364 | | 0.427273 | | | 0.281818 | 0.209091 | 0.154545 | . |
| 0.2 | 0.254545 | 0.145455 | | 0.018182 | | | 0.109091 | 0.154545 | 0.236364 | . |
| 0 | 0 | 0.190909 | | 0.3 | | | 0.145455 | 0.336364 | 0 | . |
| 0.018182 | 0.227273 | 0.163636 | | 0.145455 | | | 0.263636 | 0.309091 | 0.490909 | . |
| 0.227273 | 0.236364 | 0.145455 | | 0 | | | 0.118182 | 0.381818 | 0 | . |
| 0.2 | 0.154545 | 0.3 | | 0 | | | 0.127273 | 0.254545 | 0.118182 | . |
| 0.172727 | 0 | 0.081818 | | 0.318182 | | | 0.172727 | 0 | 0.254545 | . |
| 0.136364 | 0.218182 | 0.181818 | | 0.009091 | | | 0.272727 | 0.309091 | 0.309091 | . |
| 0.190909 | 0.1 | 0.245455 | | 0 | | | 0.127273 | 0.254545 | 0.1 | . |
| 0 | 0.081818 | 0.163636 | | 0.236364 | | | 0.081818 | 0.272727 | 0.045455 | . |
| 0.190909 | 0.172727 | 0.045455 | | 0 | | | 0.127273 | 0.009091 | 0.090909 | . |
| 0.227273 | 0.1 | 0.272727 | | 0 | | | 0.190909 | 0.3 | 0.018182 | . |
| 0 | 0.172727 | 0.045455 | | 0.318182 | | | 0.081818 | 0.1 | 0.190909 | . |
| 0.136364 | 0.109091 | 0.054545 | | 0.245455 | | | 0.109091 | 0.036364 | 0.081818 | . |
| 0.254545 | 0.081818 | 0.190909 | | 0.118182 | | | 0.109091 | 0.072727 | 0.109091 | . |
| 0.081818 | 0.045455 | 0.109091 | | 0.281818 | | | 0.2 | 0.090909 | 0.263636 | . |
| 0.036364 | 0.018182 | 0.027273 | | 0 | | | 0.136364 | 0 | 0.009091 | . |
| 0 | 0.209091 | 0.009091 | | 0 | | | 0.009091 | 0.154545 | 0.027273 | . |
| | 0 | 0.118182 | | 0.172727 | | | | | 0.181818 | . |
