# Supplementary material for: Does prenatal alcohol exposure cause a metabolic syndrome? (Non-)evidence from a mouse model of fetal alcohol spectrum disorder
Source: PLoS One. 2018 Jun 28;13(6):e0199213. doi: 10.1371/journal.pone.0199213 (PMC6023152; doi:10.1371/journal.pone.0199213)
Supplement: S1 Dataset — (ZIP) [file pone.0199213.s010.zip › New folder/Food Intake.pdf]

Blk	3	3	3	3	4	4	5	7	7	8	3	4	4	7	7	8	10	10	10	12	12	12	12	3
Box	Box-3	Box-4	Box-5	Box-6	Box-2	Box-3	Box-4	Box-3	Box-4	Box-4	Box-7	Box-5	Box-8	Box-1	Box-7	Box-2	Box-6	Box-5	Box-4	Box-1	Box-2	Box-5	Box-6	Box-1
ID	6.4	9.6	10.6	11.1	17.8	18.7	28.7	41	42	53.5	12.4	23.1	27.7	37	46	50.6	62	63	69	74	76	81	83	1.7
Sex	F	F	F	F	F	F	F	F	F	F	F	F	F	F	F	F	F	F	F	F	F	F	F	F
Exposure	EtOH	EtOH	EtOH	EtOH	EtOH	EtOH	EtOH	EtOH	EtOH	EtOH	H2O	H2O	H2O	H2O	H2O	H2O	H2O	H2O	H2O	H2O	H2O	H2O	H2O	Malto
Tx	FE	FE	FE	FE	FE	FE	FE	FE	FE	FE	FH	FH	FH	FH	FH	FH	FH	FH	FH	FH	FH	FH	FH	FM
18:00	0	0	0	0	0	0	0	0	0	0	0	0	0	0	0	0	0	0	0	0	0	0	0	0
	0	0	0	0.01	0	0.03	0.02	0	0.01	0	0	0	0	0	0	0	0	0	0	0	0	0	0	0.03
	0	0	0	0.01	0	0.06	0.23	0.12	0.19	0.03	0	0	0	0	0	0	0	0	0	0	0	0	0	0.05
	0	0	0	0.01	0	0.06	0.23	0.19	0.19	0.21	0	0	0	0	0	0	0	0	0	0	0	0	0	0.05
	0	0	0.14	0.01	0	0.06	0.28	0.36	0.19	0.21	0	0	0	0	0	0	0	0.17	0	0	0	0	0.01	0.05
	0	0	0.15	0.22	0	0.06	0.34	0.36	0.19	0.21	0	0	0	0	0	0	0	0.17	0	0	0	0	0.02	0.12
	0	0	0.19	0.22	0	0.06	0.51	0.43	0.26	0.34	0	0	0	0	0	0	0	0.17	0	0	0	0	0.02	0.24
	0	0	0.23	0.31	0	0.1	0.51	0.59	0.28	0.51	0	0	0	0	0	0	0	0.3	0	0	0	0	0.02	0.32
	0	0	0.32	0.39	0	0.14	0.58	0.7	0.3	0.69	0	0	0	0	0	0	0	0.57	0	0	0	0	0.02	0.44
	0	0	0.42	0.44	0	0.42	0.68	0.73	0.69	0.85	0	0	0.07	0	0.02	0	0	0.86	0	0	0.13	0.04	0.02	0.44
	0	0	0.5	0.64	0	0.43	0.72	0.76	0.69	0.86	0	0.3	0.11	0	0.19	0	0	0.9	0	0	0.13	0.14	0.02	0.44
	0	0	0.61	0.64	0	0.55	0.83	0.81	0.97	0.93	0.03	0.56	0.27	0	0.39	0	0	0.9	0	0	0.18	0.2	0.02	0.5
	0	0	0.78	0.88	0	0.55	0.83	0.88	1.18	0.99	0.25	0.56	0.54	0.04	0.39	0.02	0	0.9	0	0	0.21	0.3	0.02	0.54
	0	0	0.93	0.88	0	0.55	0.88	0.9	1.19	1.1	0.36	0.58	0.8	0.17	0.62	0.11	0	1.16	0	0	0.37	0.61	0.02	0.73
	0.07	0.09	0.93	0.88	0	0.82	1.06	0.93	1.19	1.13	0.51	0.93	1.05	0.34	0.81	0.4	0	1.51	0	0.01	0.44	0.74	0.02	0.73
	0.2	0.42	1.04	0.88	0	0.87	1.2	0.93	1.5	1.2	0.51	1	1.05	0.46	0.81	0.57	0	1.59	0	0.17	0.57	0.87	0.02	0.73
	0.63	0.58	1.28	1.17	0	0.98	1.32	0.95	1.5	1.42	0.52	1.06	1.05	0.63	1.07	0.57	0	1.85	0	0.22	0.68	1.01	0.25	0.73
	0.95	0.72	1.64	1.17	0	1.1	1.73	1.06	1.66	1.64	0.62	1.51	1.15	0.76	1.29	0.57	0	2.08	0	0.38	0.79	1.37	0.43	0.73
	1.2	0.72	1.73	1.17	0	1.13	1.96	1.15	1.72	1.64	0.86	1.62	1.33	1.04	1.52	0.57	0	2.08	0	0.54	0.93	1.73	0.6	0.9
	1.2	0.74	1.73	1.17	0	1.41	1.96	1.15	1.73	1.64	1.04	1.62	1.78	1.21	1.52	0.85	0	2.09	0	0.54	1.02	1.73	0.9	0.99
	1.2	0.83	1.73	1.17	0	1.41	1.96	1.15	1.73	1.64	1.04	1.62	2.02	1.39	1.52	1.1	0	2.09	0	0.72	1.03	2.01	0.98	1.07
	1.41	1.09	1.86	1.17	0	1.41	1.96	1.16	1.73	1.73	1.3	1.62	2.02	1.58	1.52	1.42	0	2.09	0	0.96	1.03	2.23	1.05	1.2
	1.41	1.32	2.01	1.17	0	1.41	1.96	1.16	1.73	1.87	1.33	1.66	2.02	1.77	1.82	1.55	0	2.09	0.06	1.06	1.04	2.48	1.36	1.37
	1.41	1.37	2.01	1.48	0	1.64	1.96	1.17	1.73	1.87	1.33	2.08	2.02	1.87	1.97	1.55	0	2.09	0.13	1.06	1.04	2.48	1.47	1.44
	1.5	1.62	2.17	1.48	0	1.64	1.96	1.34	1.73	1.87	1.73	2.14	2.02	1.98	2.23	1.55	0	2.34	0.22	1.22	1.04	2.48	1.47	1.7
	1.76	1.62	2.35	1.48	0	1.64	1.96	1.34	1.73	1.87	1.73	2.26	2.02	1.98	2.23	1.55	0	2.34	0.29	1.22	1.04	2.48	1.47	1.79
	1.77	1.62	2.38	1.48	0	1.64	2.07	1.34	1.73	1.87	1.74	2.26	2.02	1.98	2.23	1.55	0	2.34	0.38	1.22	1.04	2.48	1.47	1.9
	1.77	1.71	2.38	1.48	0	1.64	2.12	1.34	1.73	1.87	1.9	2.26	2.03	1.98	2.23	1.59	0	2.53	0.42	1.22	1.04	2.48	1.47	1.9
	1.79	1.72	2.5	1.48	0	1.77	2.36	1.34	1.73	1.87	1.9	2.26	2.25	1.99	2.23	1.59	0	2.61	0.42	1.22	1.04	2.51	1.47	1.9
	1.79	1.72	2.56	1.48	0	1.77	2.37	1.34	1.73	1.87	1.9	2.26	2.25	1.99	2.23	1.94	0	2.61	0.42	1.22	1.15	2.91	1.47	1.9
	1.79	1.88	2.74	1.48	0	1.77	2.37	1.34	1.73	1.93	1.9	2.26	2.25	1.99	2.49	2.16	0	2.61	0.42	1.29	1.15	3.36	1.52	1.9
	1.79	2.19	2.74	1.48	0	1.77	2.37	1.45	1.73	1.93	1.9	2.26	2.25	1.99	2.49	2.17	0	2.76	0.42	1.41	1.15	3.36	1.85	1.9
	1.79	2.21	2.77	1.48	0.14	1.77	2.37	1.45	1.73	1.99	1.9	2.38	2.36	1.99	2.49	2.17	0	2.93	0.42	1.64	1.18	3.36	2.29	1.91
	1.79	2.22	3.02	1.48	0.2	2.02	2.37	1.45	1.78	1.99	1.9	2.71	2.45	1.99	2.78	2.22	0	2.93	0.42	1.65	1.18	3.39	2.37	1.92
	1.79	2.22	3.02	1.48	0.36	2.14	2.58	1.45	1.78	2	1.98	3.09	2.45	2.35	3.03	2.23	0	2.93	0.43	1.65	1.18	3.39	2.37	1.92
	1.79	2.24	3.02	1.48	0.55	2.14	2.97	1.46	1.78	2	2.29	3.3	2.45	2.72	3.19	2.42	0	3.04	0.43	1.65	1.18	3.5	2.37	1.92
	1.79	2.24	3.02	1.48	0.72	2.14	2.97	1.52	1.78	2	2.29	3.3	2.57	2.72	3.19	2.59	0	3.04	0.43	1.65	1.18	3.75	2.37	1.92
	1.79	2.58	3.02	1.61	0.91	2.14	2.97	1.81	1.78	2.01	2.29	3.3	2.64	2.72	3.19	2.61	0	3.27	0.43	1.65	1.18	3.78	2.44	1.92
	1.79	2.58	3.22	1.87	1.14	2.48	2.97	1.85	1.78	2.32	2.29	3.3	2.65	2.72	3.19	2.61	0	3.27	0.51	1.65	1.18	3.81	2.84	1.92
	1.79	2.58	3.22	1.87	1.27	2.51	3.05	1.85	1.78	2.47	2.29	3.46	2.7	2.95	3.19	2.61	0	3.27	0.63	1.82	1.4	3.81	2.9	1.92
	1.79	2.58	3.22	1.87	1.27	2.51	3.35	1.85	1.78	2.47	2.29	3.58	2.85	3.2	3.19	2.61	0	3.27	0.68	1.86	1.5	3.81	2.9	1.92
	2.04	2.72	3.22	1.87	1.27	2.51	3.35	1.91	1.78	2.47	2.29	3.58	2.85	3.2	3.42	2.61	0	3.27	0.7	1.88	1.5	3.81	2.9	1.92
	2.14	2.76	3.43	1.87	1.27	2.51	3.35	1.91	1.78	2.47	2.41	3.58	2.85	3.2	3.42	2.61	0	3.27	0.7	1.88	1.56	3.81	2.9	1.92
	2.14	2.76	3.43	2.06	1.27	2.51	3.35	1.91	1.78	2.53	2.41	3.81	2.91	3.2	3.42	2.61	0	3.27	0.7	1.88	1.57	3.81	3.18	1.92
	2.14	2.76	3.43	2.21	1.27	2.51	3.35	1.91	1.78	2.6	2.41	3.84	2.91	3.2	3.66	2.61	0	3.27	0.7	1.88	1.58	3.81	3.18	1.92
	2.21	3.01	3.44	2.21	1.27	2.8	3.35	1.91	1.78	2.83	2.41	3.84	3.06	3.2	3.66	2.61	0	3.27	0.7	1.88	1.72	3.81	3.18	1.92
	2.41	3.01	3.54	2.21	1.3	2.8	3.37	1.94	1.78	2.83	2.41	3.84	3.06	3.47	3.66	2.68	0	3.27	0.7	1.88	1.72	3.81	3.18	1.92
	2.41	3.01	3.54	2.21	1.3	2.8	3.63	1.98	1.88	2.83	2.57	4	3.06	3.47	3.66	2.68	0	3.27	0.7	1.98	1.72	3.91	3.18	1.92
	2.41	3.04	3.54	2.21	1.3	2.8	3.63	1.98	1.95	2.83	2.58	4.12	3.06	3.47	3.71	2.68	0	3.27	0.7	1.98	1.76	3.92	3.18	1.92
	2.41	3.14	3.54	2.21	1.39	2.8	3.7	1.98	1.95	2.83	2.58	4.12	3.18	3.77	3.95	2.68	0	3.27	0.7	1.98	2.07	3.92	3.18	1.92
	2.61	3.14	3.69	2.21	1.67	3	3.7	1.98	1.95	2.83	2.58	4.12	3.26	3.77	3.95	2.68	0	3.27	0.7	1.98	2.07	3.92	3.18	1.92
	2.89	3.14	3.69	2.21	2.02	3	3.7	1.98	1.95	2.83	2.58	4.12	3.26	3.77	3.96	2.84	0	3.27	0.81	1.98	2.07	4.08	3.46	1.92
	3.01	3.14	3.72	2.21	2.27	3	3.7	1.98	1.95	2.96	2.58	4.12	3.33	3.77	3.96	3	0	3.27	0.81	1.99	2.07	4.08	3.46	1.92
	3.01	3.14	3.84	2.53	2.27	3	4.03	1.98	1.97	3.07	2.58	4.12	3.44	3.77	3.97	3	0	3.27	0.81	1.99	2.2	4.08	3.46	1.92
	3.01	3.14	3.84	2.58	2.27	3	4.04	1.98	2.33	3.07	2.69	4.12	3.44	4.03	4.22	3	0	3.27	0.81	1.99	2.2	4.08	3.46	1.92
	3.01	3.14	3.84	2.58	2.27	3	4.04	1.98	2.34	3.07	2.77	4.12	3.44	4.03	4.28	3	0	3.27	0.89	1.99	2.24	4.08	3.46	1.92
	3.01	3.14	3.84	2.58	2.27	3	4.04	1.98	2.34	3.2	2.77	4.24	3.55	4.03	4.28	3	0	3.28	0.93	1.99	2.24	4.18	3.46	1.92
	3.11	3.14	3.99	2.58	2.59	3	4.04	1.98	2.34	3.41	3.05	4.24	3.55	4.03	4.28	3.13	0	3.28	0.93	2.08	2.24	4.21	3.46	1.92
	3.29	3.14	3.99	2.58	2.59	3.31	4.26	1.99	2.34	3.41	3.05	4.24	3.55	4.06	4.33	3.13	0	3.28	0.93	2.19	2.28	4.48	3.48	1.92
	3.63	3.14	3.99	2.58	2.59	3.31	4.26	1.99	2.34	3.41	3.05	4.24	3.64	4.06	4.5	3.13	0	3.28	0.93	2.19	2.39	4.48	3.65	1.92
	3.63	3.14	4.23	2.89	2.75	3.31	4.31	1.99	2.53	3.41	3.05	4.48	3.89	4.23	4.5	3.13	0	3.36	0.94	2.19	2.39	4.48	3.65	2.17
	3.63	3.15	4.27	2.89	2.9	3.31	4.54	2.02	2.53	3.41	3.05	4.56	3.89	4.23	4.5	3.23	0	3.47	0.94	2.19	2.39	4.48	3.65	2.35
	3.63	3.24	4.27	2.89	2.94	3.59	4.54	2.26	2.53	3.69	3.05	4.69	3.89	4.23	4.5	3.54	0	3.47	0.94	2.19	2.39	4.48	3.65	2.63
	3.63	3.26	4.27	2.89	2.94	3.64	4.54	2.45	2.53	3.69	3.05	4.73	3.89	4.23	4.5	3.54	0	3.47	0.96	2.19	2.58	4.53	3.65	2.63
	3.63	3.29	4.38	2.89	2.94	3.64	4.64	2.45	2.8	3.69	3.11	4.84	3.89	4.23	4.51	3.54	0	3.47	1.05	2.43	2.58	4.53	3.65	2.63
	3.74	3.29	4.52	2.89	2.94	3.64	4.82	2.45	2.8	3.69	3.21	4.84	3.89	4.23	4.74	3.54	0	3.71	1.11	2.43	2.58	4.53	3.65	2.63
	3.76	3.29	4.52	3.01	2.98	3.64	4.82	2.53	2.8	3.92	3.26	4.84	3.89	4.32	4.74	3.7	0	3.74	1.12	2.43	2.58	4.53	4.02	2.63
	3.78	3.34	4.52	3.18	3.27	3.64	5.06	2.6	3.1	3.92	3.26	4.84	3.89	4.59	4.93	3.8	0	3.9	1.12	2.43	2.63	4.67	4.25	2.63
	3.78	3.41	4.56	3.18	3.45	3.64	5.06	2.6	3.1	4.1	3.26	4.84	4.19	4.59	4.93	3.8	0	3.9	1.12	2.43	2.63	4.71	4.38	2.71
	3.78	3.48	4.87	3.18	3.46	3.94	5.06	2.84	3.3	4.24	3.26	5.07	4.19	4.87	4.93	4.09	0	4.07	1.12	2.56	2.93	4.75	4.38	2.71
	4.01	3.53	4.87	3.18	3.46	3.94	5.33	2.84	3.3	4.24	3.26	5.08	4.19	4.87	4.93	4.09	0	4.08	1.18	2.56	2.93	4.75	4.38	3.06
	4.01	3.58	5.14	3.5	3.46	3.94	5.59	2.84	3.65	4.56	3.32	5.08	4.19	4.87	4.93	4.09	0	4.32	1.29	2.64	2.93	4.88	4.38	3.06
	4.11	3.72	5.19	3.5	3.59	3.94	5.59	3.16	3.65	4.56	3.34	5.29	4.41	4.87	5.05	4.42	0	4.34	1.3	2.83	3.16	4.92	4.38	3.06
	4.14	4.14	5.35	3.5	3.76	3.94	5.71	3.16	3.91	4.56	3.34	5.49	4.41	5.23	5.25	4.45	0	4.4	1.5	2.83	3.16	5.24	4.6	3.33
	4.14	4.14	5.36	3.5	3.76	3.94	5.88	3.3	3.91	4.76	3.34	5.79	4.41	5.23	5.25	4.45	0	4.64	1.55	2.83	3.27	5.24	4.6	3.33
	4.14	4.14	5.63	3.5	3.96	3.94	5.88	3.55	3.92	4.84	3.63	5.79	4.63	5.24	5.25	4.45	0	4.84	1.79	3.03	3.45	5.54	4.92	3.33
	4.37	4.24	5.63	3.84	4.05	3.94	6.17	3.56	4.21	4.96	3.63	5.86	4.71	5.24	5.42	4.87	0	4.84	1.88	3.03	3.55	5.54	5.37	3.68
	4.57	4.28	5.63	3.84	4.05	4.14	6.17	3.56	4.21	5.22	3.63	5.93	4.88	5.39	5.42	4.87	0	4.84	1.88	3.03	3.88	5.56	5.37	3.68
	4.68	4.28	5.8	3.84	4.28	4.15	6.17	3.94	4.59	5.22	3.63	6.02	4.88	5.4	5.66	4.87	0	5.08	1.98	3.17	3.88	5.99	5.62	3.74
	4.91	4.28	5.8	3.84	4.28	4.18	6.17	3.94	4.59	5.22	3.88	6.03	5.01	5.49	5.66	5.17	0	5.35	2.26	3.17	4.1	5.99	5.62	3.93
	5.06	4.31	5.87	3.84	4.28	4.43	6.35	3.94	4.59	5.42	3.88	6.04	5.43	5.49	5.66	5.17	0	5.35	2.26	3.48	4.1	5.99	5.62	3.93
	5.21	4.4	6.1	3.88	4.28	4.43	6.53	3.94	4.59	5.42	3.88	6.07	5.43	5.78	5.88	5.17	0.24	5.6	2.26	3.48	4.23	6.06	5.62	3.93
	5.45	4.85	6.1	4.02	4.36	4.74	6.53	3.94	4.59	5.42	4.07	6.07	5.43	5.78	5.88	5.58	0.55	5.6	2.39	3.48	4.44	6.62	5.81	4.09
	5.45	4.88	6.1	4.09	4.36	4.75	6.76	3.94	4.59	5.42	4.1	6.07	5.43	5.82	6.04	5.85	0.77	5.6	2.64	3.79	4.63	6.76	5.92	4.33
	5.72	5.11	6.26	4.34	4.58	4.75	6.76	4.42	4.77	5.75	4.37	6.07	5.43	6.08	6.04	5.85	0.96	5.82	3.03	3.8	4.63	6.99	5.92	4.49
	5.84	5.11	6.51	4.34	4.63	4.75	6.76	4.42	5.04	5.75	4.37	6.07	5.69	6.15	6.04	6.11	1.24	5.86	3.05	4.03	4.63	6.99	6.2	4.49
	5.84	5.12	6.51	4.34	4.63	4.75	6.76	4.42	5.04	6	4.37	6.07	5.74	6.35	6.04	6.14	1.29	5.86	3.05	4.18	4.85	7.45	6.47	4.49
	5.93	5.37	6.51	4.34	4.63	4.75	6.87	4.74	5.04	6	4.65	6.07	5.74	6.35	6.06	6.14	1.29	5.86	3.05	4.18	4.85	7.47	6.47	4.49
	5.93	5.61	6.51	4.58	4.63	4.75	6.87	4.74	5.11	6	4.65	6.51	5.74	6.36	6.06	6.15	1.38	5.86	3.05	4.28	5.12	7.57	6.8	4.49
	5.93	5.62	6.65	4.58	4.63	4.75	6.89	4.74	5.22	6	4.65	6.51	5.74	6.36	6.19	6.21	1.49	5.91	3.05	4.3	5.12	7.84	6.82	4.66
	5.93	5.62	6.67	4.58	4.63	4.75	7.17	4.74	5.22	6.16	4.65	6.51	5.9	6.36	6.46	6.22	1.49	6.1	3.05	4.63	5.12	7.84	6.82	4.71
	5.93	5.62	6.67	4.58	4.63	4.75	7.17	4.74	5.22	6.16	4.65	6.51	5.9	6.36	6.46	6.22	1.49	6.1	3.05	4.63	5.34	7.84	6.82	5.07
	6.16	5.62	6.67	4.58	4.63	4.75	7.17	4.74	5.23	6.16	4.9	6.51	5.9	6.36	6.46	6.22	1.62	6.1	3.05	4.63	5.35	7.98	7.13	5.1
	6.16	5.77	6.85	4.58	4.63	4.75	7.17	4.74	5.24	6.16	4.9	6.51	5.91	6.36	6.46	6.22	2	6.1	3.05	4.63	5.35	8.38	7.13	5.1
	6.16	5.94	7.03	4.58	4.72	4.86	7.17	4.97	5.24	6.16	4.9	6.51	6.05	6.54	6.68	6.22	2.08	6.1	3.05	4.63	5.62	8.38	7.13	5.48
	6.2	5.94	7.03	4.58	4.72	4.86	7.17	4.97	5.24	6.16	5.02	6.7	6.11	6.54	6.68	6.46	2.08	6.36	3.05	4.63	5.72	8.38	7.13	5.48
	6.2	5.94	7.12	4.68	4.72	4.86	7.33	4.97	5.25	6.51	5.24	6.88	6.11	6.54	6.68	6.46	2.08	6.6	3.05	4.8	5.72	8.38	7.13	5.48
	6.29	5.94	7.12	4.81	4.72	4.86	7.33	4.97	5.25	6.58	5.29	6.88	6.11	6.54	6.69	6.46	2.08	6.6	3.51	4.8	5.72	8.38	7.13	5.48
	6.29	5.94	7.12	4.81	4.72	4.88	7.33	4.98	5.28	6.82	5.29	6.88	6.11	6.54	6.77	6.49	2.41	6.6	3.65	4.8	5.72	8.66	7.13	5.48
	6.29	6.04	7.39	4.81	4.79	4.88	7.33	4.98	5.28	6.82	5.29	6.88	6.2	6.54	6.8	6.49	2.52	6.6	3.66	4.8	5.88	8.87	7.13	5.48
	6.29	6.04	7.39	4.81	4.9	4.88	7.33	4.98	5.38	6.82	5.29	7.03	6.38	6.78	6.8	6.49	2.52	6.6	3.69	4.92	5.88	8.87	7.13	5.48
	6.29	6.04	7.39	4.81	4.99	5.12	7.52	4.98	5.67	6.82	5.29	7.32	6.38	6.93	6.8	6.49	2.52	6.66	3.69	4.92	5.88	9.02	7.5	5.48
	6.44	6.04	7.39	4.81	5.29	5.46	7.52	4.98	5.96	6.82	5.29	7.49	6.38	6.93	6.8	6.49	2.84	6.68	3.69	4.92	5.88	9.1	7.5	5.48
	6.65	6.25	7.39	4.81	5.47	5.46	7.52	4.98	6.01	7	5.5	7.49	6.38	6.94	7.06	6.74	2.84	6.68	3.69	4.92	5.88	9.1	7.5	5.48
	6.65	6.25	7.65	4.81	5.47	5.46	7.75	4.98	6.01	7	5.5	7.49	6.38	7.09	7.06	6.74	2.84	6.68	3.69	4.92	5.88	9.1	7.5	5.48
	6.65	6.3	7.67	4.81	5.47	5.46	7.76	5.25	6.01	7	5.5	7.49	6.52	7.1	7.31	6.74	2.84	6.68	3.69	5.14	5.88	9.1	7.71	5.48
	6.65	6.32	7.74	4.81	5.47	5.5	7.76	5.25	6.01	7.07	5.54	7.54	6.53	7.1	7.31	6.74	2.84	6.68	3.69	5.16	5.88	9.1	7.79	5.48
	6.65	6.54	7.8	4.81	5.47	5.7	7.76	5.25	6.01	7.07	5.54	7.54	6.53	7.16	7.31	6.91	3.15	6.68	3.69	5.16	5.88	9.1	7.79	5.53
	6.9	6.54	7.8	4.9	5.47	5.7	7.9	5.25	6.18	7.07	5.54	7.64	6.53	7.41	7.31	6.91	3.33	6.68	3.69	5.16	5.92	9.1	7.79	5.53
	6.9	6.54	7.92	5.1	5.47	5.7	7.9	5.25	6.44	7.1	5.62	7.64	6.64	7.41	7.62	6.91	3.33	6.68	3.69	5.16	5.92	9.43	7.79	5.53
	6.9	6.54	7.92	5.1	5.48	5.7	7.9	5.25	6.44	7.1	5.91	7.64	6.64	7.41	7.62	7.16	3.33	6.7	3.69	5.16	5.92	9.43	7.79	5.53
	6.9	6.54	7.92	5.1	5.48	5.7	7.9	5.29	6.44	7.1	5.97	7.64	6.64	7.46	7.62	7.16	3.33	6.7	3.91	5.16	5.92	9.43	7.79	5.63
	7	6.72	7.92	5.1	5.48	5.7	7.9	5.37	6.44	7.1	5.97	7.69	6.64	7.65	7.82	7.18	3.33	6.7	4.08	5.16	5.92	9.43	7.79	5.65
	7.14	6.72	7.92	5.31	5.48	5.7	8.05	5.37	6.44	7.3	5.97	7.98	6.78	7.65	7.82	7.37	3.33	6.7	4.29	5.43	5.92	9.43	7.79	5.65

	7.14	6.72	8.16	5.31	5.48	5.84	8.32	5.37	6.52	7.3	5.97	7.98	6.78	7.82	7.82	7.37	3.33	6.71	4.29	5.43	5.93	9.43	7.79	5.65
	7.14	6.72	8.16	5.31	5.48	5.85	8.32	5.37	6.87	7.3	6.06	7.98	6.92	7.82	8.1	7.37	3.33	6.71	4.29	5.43	5.93	9.43	7.79	5.65
	7.31	6.85	8.16	5.31	5.53	5.85	8.32	5.37	6.87	7.3	6.06	7.98	7.04	7.82	8.1	7.37	3.33	6.71	4.29	5.43	5.93	9.43	7.79	5.86
	7.35	6.85	8.16	5.31	5.7	5.91	.	5.61	6.87	7.3	6.06	8.09	7.04	7.82	8.1	7.53	3.54	6.71	4.29	5.47	6.19	9.43	7.79	5.86
	7.59	6.85	8.16	5.31	5.7	5.97	.	5.61	6.87	7.3	6.06	8.37	7.04	7.82	8.35	7.53	3.54	6.71	4.42	5.47	6.2	9.43	7.79	5.86
	7.59	7.07	8.16	5.59	5.7	5.97	.	5.61	6.87	7.3	6.06	8.37	7.04	7.82	8.35	7.53	3.54	6.71	4.42	5.47	6.35	9.43	7.79	5.86
	7.59	7.07	8.26	5.59	5.7	5.97	.	5.61	6.87	7.38	6.43	8.37	7.32	8.03	8.35	7.53	3.54	6.94	4.42	5.75	6.35	9.59	7.79	5.86
	7.59	7.07	8.26	5.59	5.7	6.19	.	5.61	7.24	7.45	6.43	8.37	7.32	8.16	8.39	7.53	3.54	6.94	4.43	5.78	6.35	9.59	7.89	5.86
	7.71	7.07	8.26	5.59	5.71	6.3	.	5.61	7.25	7.61	6.43	8.37	7.32	8.16	8.39	7.53	3.54	6.94	4.43	5.78	6.35	9.59	7.89	5.86
	7.71	7.11	8.47	5.59	5.79	6.3	.	5.61	7.25	7.61	6.43	8.37	7.32	8.16	8.39	7.75	3.54	6.98	4.43	5.78	6.44	9.87	7.89	6.19
	7.71	7.11	8.62	5.59	5.79	6.3	.	5.81	7.25	7.61	6.62	8.79	7.32	8.16	8.43	7.75	3.54	7.25	4.79	5.78	6.56	9.87	7.89	6.2
	7.84	7.13	8.62	5.59	5.79	6.3	.	5.9	7.25	7.61	6.72	8.86	7.32	8.45	8.91	7.75	3.54	7.25	4.79	5.78	6.75	9.9	7.89	6.2
	7.84	7.13	8.62	5.87	5.79	6.3	.	5.9	7.25	7.85	6.72	8.86	7.67	8.73	8.91	7.75	3.54	7.25	4.79	5.78	6.76	9.9	8.29	6.2
	7.85	7.13	8.62	5.92	5.79	6.49	.	5.9	7.41	7.85	6.72	8.86	7.67	8.73	8.91	7.85	3.54	7.25	4.94	5.78	7.01	9.9	8.29	6.21
	8.13	7.37	8.79	5.92	6.13	6.49	.	5.91	7.87	7.85	6.72	8.99	7.67	8.73	8.91	8.18	3.62	7.25	5.05	5.78	7.01	9.9	8.29	6.47
	8.29	7.42	8.79	5.92	6.13	6.49	.	6.16	7.89	7.88	6.95	9	7.67	8.73	8.91	8.18	3.82	7.42	5.05	5.93	7.01	9.98	8.29	6.47
	8.29	7.42	8.79	5.92	6.13	6.49	.	6.16	7.89	8.03	6.95	9.01	7.67	8.73	8.91	8.18	3.82	7.51	5.05	5.93	7.01	10.27	8.29	6.47
	8.29	7.42	9.01	6.27	6.13	6.5	.	6.16	7.89	8.03	6.95	9.01	7.67	8.73	8.93	8.41	3.87	7.64	5.05	5.93	7.04	10.27	8.41	6.76
	8.29	7.46	9.01	6.27	6.14	6.81	.	6.16	7.89	8.25	6.95	9.01	7.79	8.78	9.33	8.45	4.19	7.65	5.05	5.93	7.04	10.27	8.58	6.76
	8.29	7.66	9.24	6.27	6.28	6.81	.	6.16	7.9	8.39	7.14	9.17	7.79	8.78	9.33	8.46	4.26	7.9	5.34	5.93	7.11	10.27	8.58	6.76
	8.3	7.66	9.45	6.63	6.28	7.07	.	6.48	8.42	8.45	7.28	9.25	7.94	9.15	9.33	8.66	4.26	7.9	5.42	5.96	7.26	10.6	8.58	6.76
	8.58	7.79	9.52	6.63	6.31	7.07	.	6.48	8.5	8.45	7.28	9.26	8.01	9.15	9.61	8.67	4.57	8.11	5.43	6.08	7.35	10.6	9.18	6.94
	8.62	7.79	9.52	6.63	6.56	7.07	.	6.49	8.53	8.64	7.28	9.58	8.01	9.15	9.61	8.68	4.81	8.12	5.66	6.14	7.48	10.71	9.18	7.38
	8.91	7.92	9.8	6.63	6.81	7.07	.	6.8	8.57	8.65	7.61	9.58	8.01	9.44	9.62	9.15	4.81	8.39	5.77	6.33	7.61	10.9	9.63	7.52
	9.04	8.15	9.8	6.63	6.81	7.07	.	6.8	8.89	8.86	7.64	9.77	8.2	9.63	9.96	9.29	4.81	8.39	5.97	6.33	7.79	10.9	9.64	7.52
	9.04	8.19	10.19	6.84	7.08	7.47	.	6.8	8.9	8.91	7.64	10.04	8.21	9.94	9.96	9.4	4.81	8.39	6.05	6.37	7.79	11.35	10.08	7.52
	9.14	8.29	10.2	6.86	7.18	7.47	.	6.8	9.19	8.98	7.64	10.06	8.49	9.98	9.96	9.4	4.81	8.65	6.17	6.56	8.12	11.36	10.08	7.52
	9.45	8.29	10.33	6.86	7.18	7.47	.	6.8	9.19	9.22	8.03	10.48	8.49	9.98	10.23	9.4	5.13	8.65	6.17	6.57	8.12	11.36	10.08	7.81
	9.45	8.5	10.34	6.87	7.57	7.47	.	7.15	9.19	9.33	8.04	10.48	8.55	9.98	10.23	9.62	5.13	8.73	6.17	6.57	8.29	11.36	10.08	8.05
	9.53	8.61	10.48	7.18	7.57	7.47	.	7.15	9.19	9.48	8.06	10.63	8.62	9.98	10.23	9.62	5.13	8.99	6.46	6.81	8.52	11.36	10.09	8.16
	9.62	8.61	10.48	7.18	7.6	7.84	.	7.63	9.19	9.67	8.06	10.64	8.62	10.05	10.35	9.69	5.14	9.14	6.5	6.82	8.52	11.36	10.22	8.16
	9.87	8.72	10.48	7.18	7.65	7.84	.	7.63	9.19	9.67	8.06	10.64	8.88	10.47	10.59	9.69	5.5	9.29	6.62	6.88	8.52	11.52	10.22	8.16
	9.87	8.72	10.66	7.18	7.65	8.12	.	7.63	9.19	9.67	8.21	10.64	8.88	10.5	10.59	9.85	5.5	9.41	6.62	6.92	8.76	11.53	10.22	8.16
	10.07	8.95	10.78	7.56	7.65	8.13	.	7.78	9.34	9.67	8.21	10.64	8.88	10.5	10.88	9.87	5.82	9.41	6.64	7.15	8.76	11.88	10.95	8.16
	10.08	8.95	10.78	7.58	7.65	8.13	.	8.01	9.66	9.67	8.21	10.85	8.94	10.5	10.88	10.21	5.82	9.41	6.64	7.15	8.96	11.9	10.96	8.16
	10.47	9.22	10.94	7.58	7.65	8.13	.	8.01	9.66	9.91	8.27	10.85	9.02	10.5	10.88	10.23	6.16	9.42	6.64	7.19	8.96	12.39	11.19	8.34
	10.47	9.22	10.94	7.58	7.77	8.13	.	8.01	9.66	9.91	8.48	10.92	9.02	10.62	10.88	10.23	6.16	9.45	6.64	7.19	8.96	12.4	11.19	8.49
	10.47	9.25	11.16	7.58	8.04	8.13	.	8.01	9.66	10.18	8.48	10.96	9.26	10.81	10.88	10.4	6.24	9.45	6.7	7.62	9.06	12.79	11.45	8.8
	10.73	9.26	11.16	7.58	8.04	8.14	.	8.43	9.66	10.19	8.48	11.2	9.28	10.81	10.88	10.4	6.39	9.71	7.04	7.62	9.06	12.79	11.67	8.81
	10.73	9.26	11.16	7.58	8.04	8.15	.	8.43	9.66	10.19	8.48	11.37	9.28	10.82	11.02	10.4	6.39	9.71	7.46	7.62	9.29	12.79	11.67	8.95
	10.73	9.26	11.17	7.58	8.04	8.15	.	8.43	9.66	10.21	8.73	11.37	9.28	10.82	11.02	10.4	6.39	9.71	7.5	7.62	9.29	12.8	11.67	9
	10.73	9.4	11.2	7.58	8.04	8.15	.	8.43	9.95	10.21	8.73	11.37	9.28	10.82	11.02	10.4	6.39	9.71	7.5	7.62	9.29	12.86	11.67	9
	10.73	9.4	11.2	7.58	8.23	8.15	.	8.43	9.95	10.21	8.73	11.56	9.52	10.82	11.02	10.4	6.39	9.91	7.51	7.62	9.29	13.19	11.67	9
	10.73	9.66	11.2	7.58	8.28	8.15	.	8.43	9.95	10.21	8.76	11.56	9.52	10.82	11.02	10.51	6.59	9.91	7.64	7.62	9.29	13.19	11.67	9
	10.73	9.66	11.33	7.58	8.34	8.15	.	8.43	9.99	10.21	9.13	11.56	9.52	10.82	11.02	10.75	6.59	9.91	7.64	7.62	9.29	13.19	11.7	9.1
	10.99	9.66	11.33	7.92	8.34	8.15	.	8.43	9.99	10.21	9.13	11.56	9.61	11.12	11.35	10.75	6.59	9.92	7.64	7.62	9.29	13.19	12.14	9.31
	11.01	9.68	11.51	7.92	8.34	8.15	.	8.82	9.99	10.39	9.13	11.56	9.61	11.12	11.35	10.75	6.87	10.16	7.64	7.62	9.29	13.19	12.14	9.31
	11.01	9.78	11.51	7.92	8.34	8.17	.	8.82	9.99	10.39	9.13	11.56	9.86	11.12	11.35	10.75	6.9	10.16	7.64	7.62	9.52	13.19	12.14	9.31
	11.01	9.78	11.57	7.92	8.34	8.51	.	8.82	9.99	10.49	9.13	11.56	9.86	11.12	11.35	10.75	7.19	10.16	7.64	7.62	9.52	13.19	12.14	9.31
	11.01	9.78	11.57	7.92	8.34	8.51	.	8.82	9.99	10.52	9.13	11.56	9.86	11.12	11.56	11.04	7.19	10.16	7.64	7.62	9.52	13.19	12.34	9.31
	11.13	10.16	11.57	7.92	8.56	8.79	.	8.82	10.24	10.71	9.15	11.59	9.86	11.38	11.56	11.04	7.21	10.16	7.65	7.62	9.61	13.21	12.74	9.31
	11.14	10.22	11.57	7.92	8.87	8.79	.	8.82	10.32	10.71	9.47	11.6	9.86	11.42	11.56	11.12	7.21	10.16	7.65	7.62	9.8	13.59	12.74	9.31
	11.14	10.22	11.75	7.92	8.87	8.79	.	8.82	10.32	10.71	9.47	11.6	9.86	11.42	11.56	11.12	7.21	10.3	7.65	7.93	9.94	13.59	12.74	9.31
	11.14	10.22	11.75	7.92	8.87	8.79	.	8.82	10.32	10.71	9.47	11.72	9.99	11.42	11.56	11.12	7.21	10.44	8.04	7.93	9.95	13.59	12.74	9.31
	11.14	10.22	11.75	7.92	8.87	8.79	.	9.13	10.49	10.71	9.47	11.95	9.99	11.42	11.84	11.26	7.55	10.44	8.04	7.93	9.95	13.59	12.74	9.31
	11.14	10.29	11.99	8.27	8.87	8.79	.	9.27	10.49	10.8	9.47	11.95	9.99	11.74	11.84	11.33	7.56	10.44	8.36	7.93	9.95	13.59	12.74	9.31
	11.14	10.33	12	8.27	8.94	9.11	.	9.27	10.49	10.96	9.47	11.95	9.99	11.74	11.84	11.33	7.56	10.44	8.36	7.93	9.95	13.91	12.74	9.51
	11.15	10.35	12	8.27	9.33	9.11	.	9.27	10.49	10.97	9.47	11.95	9.99	11.74	11.84	11.33	7.56	10.44	8.36	8.18	10.15	13.91	12.74	9.51
	11.29	10.5	12.06	8.27	9.36	9.11	.	9.27	10.86	10.97	9.59	11.95	10.02	11.74	11.84	11.33	7.56	10.44	8.36	8.18	10.15	13.91	12.74	9.51
	11.29	10.5	12.13	8.27	9.36	9.11	.	9.27	10.89	10.97	9.59	12.28	10.1	11.74	12.15	11.46	7.59	10.44	8.36	8.18	10.15	13.91	12.74	9.51
	11.29	10.5	12.13	8.27	9.36	9.11	.	9.27	10.89	10.97	9.59	12.28	10.11	12.03	12.15	11.46	7.92	10.44	8.36	8.18	10.15	13.91	12.74	9.51
	11.29	10.5	12.13	8.27	9.36	9.11	.	9.27	10.89	11.11	9.59	12.28	10.11	12.03	12.15	11.46	7.92	10.44	8.37	8.18	10.31	13.91	12.78	9.51
	11.52	10.5	12.13	8.27	9.36	9.11	.	9.27	10.89	11.11	9.81	12.28	10.11	12.03	12.15	11.52	7.92	10.9	8.38	8.37	10.31	13.91	13	9.51
	11.52	10.5	12.29	8.27	9.36	9.11	.	9.27	10.89	11.11	9.81	12.38	10.3	12.03	12.15	11.54	7.92	10.9	8.38	8.38	10.63	14.15	13	9.51
	11.52	10.55	12.29	8.27	9.4	9.32	.	9.27	11.17	11.11	9.81	12.38	10.3	12.03	12.15	11.54	7.92	10.9	8.38	8.38	10.63	14.4	13	9.51
	11.52	10.55	12.29	8.45	9.7	9.32	.	9.35	11.17	11.27	9.81	12.56	10.3	12.03	12.38	11.54	**7.92**	**10.9**	**8.86**	8.38	10.63	14.4	13	9.51
**10kcal%**	0	0	0	0	0	0	0	0	0	0	0	0	0	0	0	0	**0**	**0**	**0**	0	0	0	0	0
14:22	0.15	0	0.07	0.06	0.27	0	0	0	0.22	0	0.15	0.14	0.12	0.46	0	0.38	0	0	0.25	0.17	0	0.04	0	0.05
	0.28	0.43	0.18	0.21	0.44	0.38	0.12	0.21	0.22	0	0.35	0.4	0.21	0.46	0.34	0.38	0	0	0.35	0.17	0.2	0.22	0	0.38
	0.47	0.47	0.27	0.21	0.6	0.38	0.32	0.21	0.44	0	0.43	0.4	0.27	0.46	0.34	0.38	0.51	0.06	0.55	0.17	0.22	0.49	0.3	0.38
	0.47	0.47	0.37	0.43	0.6	0.38	0.32	0.42	0.7	0	0.43	1	0.4	0.46	0.34	0.38	0.51	0.28	0.6	0.17	0.22	0.49	0.3	0.67
	0.62	0.53	0.37	0.43	0.7	0.38	0.32	0.42	0.7	0.36	0.58	1	0.56	0.46	0.39	0.61	0.62	0.47	0.7	0.53	0.42	0.52	0.63	0.67
	0.62	0.86	0.58	0.49	0.76	0.69	0.76	0.42	0.7	0.36	0.73	1	0.56	0.88	0.57	0.61	1.04	0.47	0.7	0.53	0.43	0.82	0.63	0.67
	0.97	0.86	0.58	0.49	0.78	0.69	0.76	0.48	0.7	0.36	0.73	1	0.56	0.88	0.63	0.61	1.04	0.57	0.87	0.54	0.46	0.82	0.63	0.9
	0.97	1.15	0.9	0.49	0.78	0.69	0.76	0.63	0.7	0.36	0.73	1	0.85	0.88	0.79	0.92	1.04	0.72	0.87	0.54	0.72	1.11	0.63	1.08
	0.97	1.15	1.25	0.64	1.14	0.69	0.98	0.63	1.21	0.71	1.08	1.25	0.85	0.88	0.79	0.92	1.04	0.72	1.04	0.74	0.72	1.46	0.94	1.08
	1.26	1.15	1.25	0.65	1.14	0.89	0.98	0.63	1.21	0.71	1.08	1.25	0.85	1.17	0.79	1.01	1.04	0.72	1.04	0.74	0.72	1.46	1.04	1.08
18:00	1.26	1.15	1.25	0.91	1.14	1.21	0.98	0.93	1.21	1.02	1.08	1.25	1.06	1.17	1.06	1.01	1.04	1.06	1.04	0.74	0.83	1.46	1.04	1.15
	1.26	1.15	1.25	1.05	1.15	1.22	1.31	0.93	1.21	1.02	1.09	1.25	1.06	1.17	1.06	1.13	1.04	1.06	1.04	0.74	1.07	1.46	1.04	1.15
	1.48	1.44	1.29	1.05	1.17	1.22	1.31	0.93	1.21	1.02	1.34	1.28	1.08	1.22	1.06	1.22	1.04	1.06	1.39	0.76	1.09	1.46	1.04	1.49
	1.6	1.46	1.3	1.05	1.17	1.22	1.31	1.46	1.66	1.02	1.34	1.52	1.3	1.22	1.06	1.6	1.24	1.06	1.39	0.76	1.09	1.89	1.46	1.49
	1.76	1.46	1.6	1.37	1.25	1.22	1.32	1.46	1.73	1.02	1.6	1.52	1.3	1.22	1.06	1.64	1.24	1.12	1.39	0.76	1.09	1.89	1.48	1.83
	1.96	1.46	1.6	1.38	1.25	1.22	1.34	1.46	1.73	1.03	1.6	1.52	1.3	1.24	1.14	1.64	1.24	1.23	1.39	1	1.09	1.89	1.48	1.84
	1.96	1.46	1.67	1.38	1.25	1.22	1.34	1.46	1.74	1.5	1.6	1.76	1.39	1.25	1.43	1.64	1.54	1.23	1.39	1	1.09	1.89	1.75	2.16
	2.29	1.87	1.78	1.62	1.46	1.22	1.42	1.46	1.74	1.5	1.6	1.76	1.59	1.25	1.43	1.87	1.54	1.57	1.39	1.02	1.09	1.89	1.75	2.16
	2.3	1.87	1.96	1.62	1.47	1.22	1.47	1.46	1.74	1.5	1.94	1.76	1.72	1.57	1.43	1.87	1.55	1.57	1.43	1.02	1.21	1.89	1.87	2.16
	2.3	1.87	2.15	1.62	1.79	1.22	1.47	1.51	1.74	1.68	1.94	1.83	1.72	1.57	1.75	2.03	1.61	1.57	1.43	1.27	1.21	1.94	2.37	2.35
	2.3	1.87	2.15	1.62	1.79	1.22	1.59	1.6	1.74	1.68	1.94	2.14	1.72	1.63	1.75	2.08	1.61	2.04	1.43	1.27	1.52	1.94	2.46	2.35
	2.3	1.97	2.15	1.63	1.79	1.43	1.64	1.6	1.74	1.68	1.94	2.15	1.72	1.63	1.75	2.08	1.61	2.04	1.79	1.27	1.71	1.94	2.62	2.35
	2.3	2.04	2.15	1.77	1.9	1.46	1.77	1.6	1.74	1.72	1.94	2.15	1.98	1.74	2.08	2.08	2.05	2.17	2.03	1.27	1.76	1.94	2.86	2.35
	2.34	2.04	2.42	1.79	1.9	1.46	1.89	1.6	2.14	1.73	2.04	2.15	1.98	1.74	2.08	2.08	2.05	2.17	2.03	1.27	1.76	1.94	2.86	2.35
	2.36	2.41	2.43	1.79	1.96	1.8	1.93	1.67	2.14	1.78	2.45	2.15	1.98	1.77	2.08	2.08	2.05	2.17	2.03	1.6	1.83	2.22	2.88	2.52
	2.51	2.56	2.43	1.79	2.37	1.8	1.93	1.67	2.14	1.84	2.45	2.41	1.98	1.87	2.08	2.21	2.05	2.46	2.03	1.6	2.03	2.22	3.05	2.7
	2.51	2.56	2.43	1.95	2.37	1.8	1.93	1.84	2.14	1.87	2.45	2.42	1.98	1.88	2.08	2.33	2.05	2.46	2.03	1.6	2.04	2.7	3.06	2.7
	2.75	2.56	2.43	1.98	2.37	1.8	2.29	1.84	2.14	1.95	2.45	2.42	2.11	1.88	2.08	2.44	2.55	2.63	2.03	1.6	2.05	2.7	3.16	2.86
	2.75	2.56	2.43	1.98	2.37	1.99	2.29	1.84	2.14	1.95	2.45	2.42	2.2	2.18	2.34	2.49	2.55	2.63	2.03	1.6	2.05	2.83	3.16	2.88
	2.79	2.56	2.43	1.98	2.37	2.13	2.29	2.02	2.41	1.95	2.45	2.52	2.2	2.18	2.34	2.49	2.55	2.69	2.03	1.61	2.05	3.18	3.16	2.88
	2.83	2.56	2.43	1.98	2.48	2.13	2.48	2.02	2.54	1.95	2.48	2.53	2.2	2.18	2.34	2.55	2.55	2.69	2.03	1.76	2.29	3.18	3.37	2.88
	3.24	2.56	2.43	1.98	2.48	2.13	2.48	2.02	2.54	2.06	2.59	2.57	2.2	2.36	2.34	2.55	2.55	2.69	2.09	1.76	2.29	3.18	3.44	2.88
	3.24	2.69	2.43	1.98	2.68	2.13	2.48	2.02	2.54	2.29	2.59	2.57	2.31	2.36	2.34	2.59	2.55	2.69	2.09	2.15	2.3	3.18	3.67	2.99
	3.24	2.72	2.65	2.18	2.68	2.13	2.7	2.02	2.54	2.29	2.59	2.57	2.31	2.36	2.87	2.86	2.55	2.69	2.09	2.15	2.51	3.18	3.73	2.99
	3.24	2.73	2.65	2.4	2.68	2.13	2.7	2.02	2.54	2.29	2.71	2.57	2.31	2.36	2.87	2.88	3.18	2.69	2.09	2.15	2.51	3.28	3.73	2.99
	3.24	2.73	2.65	2.4	2.8	2.13	2.7	2.02	2.54	2.29	2.71	2.57	2.31	2.36	2.87	2.88	3.46	2.69	2.25	2.15	2.51	3.28	3.73	3.15
	3.24	2.77	2.65	2.57	2.8	2.13	2.7	2.14	2.54	2.29	3.04	2.57	2.31	2.36	2.87	2.88	3.46	2.69	2.57	2.15	2.51	3.62	4.16	3.15
	3.27	2.78	2.65	2.57	2.8	2.53	2.7	2.14	2.54	2.29	3.04	2.57	2.63	2.36	2.87	2.88	3.46	2.89	2.58	2.15	2.52	3.62	4.16	3.15
	3.27	2.78	2.65	2.57	2.8	2.53	2.7	2.14	2.54	2.46	3.14	2.57	2.63	2.36	2.87	3.38	3.46	2.89	2.58	2.15	2.52	3.62	4.16	3.15
	3.27	2.78	2.65	2.57	2.81	2.53	2.84	2.21	2.54	2.46	3.14	2.57	2.63	2.36	2.87	3.4	3.46	2.89	2.58	2.15	2.52	3.62	4.16	3.15
	3.27	2.78	2.72	2.57	2.81	2.53	2.99	2.21	2.54	2.52	3.14	2.57	2.63	2.36	2.87	3.4	3.46	2.89	2.58	2.27	2.52	3.62	4.16	3.15
	3.27	2.78	2.72	2.57	2.83	2.53	2.99	2.42	2.54	2.67	3.14	2.57	3.01	2.44	2.87	3.4	3.46	2.89	2.58	2.4	2.52	3.62	4.27	3.15
	3.27	2.78	2.73	2.57	2.84	2.53	2.99	2.42	2.54	2.67	3.14	2.58	3.02	2.45	2.88	3.4	3.46	2.89	2.58	2.4	2.52	3.69	4.47	3.15
	3.38	2.78	2.73	2.57	2.84	2.53	2.99	2.42	3.11	2.67	3.25	2.64	3.02	2.54	3.07	3.4	3.46	2.89	2.58	2.4	2.59	3.71	4.47	3.15
	3.38	2.89	2.73	2.57	3.08	2.53	2.99	2.67	3.11	2.67	3.25	2.74	3.02	2.54	3.07	3.79	3.46	2.89	2.94	2.4	2.59	3.94	4.47	3.18
	3.46	2.89	2.73	2.57	3.15	2.86	2.99	2.67	3.11	2.76	3.25	2.75	3.02	2.54	3.07	3.79	3.59	3	3.05	2.4	2.6	3.94	4.47	3.18
	3.47	2.89	2.73	2.57	3.2	2.86	3.4	2.88	3.11	2.76	3.52	2.75	3.18	2.75	3.07	3.79	3.64	3	3.05	2.4	2.69	3.94	4.57	3.18
	3.57	3.17	2.92	2.77	3.2	2.86	3.41	2.88	3.11	2.76	3.54	2.77	3.18	2.75	3.17	3.79	3.64	3	3.05	2.4	2.74	3.94	4.57	3.24
	3.69	3.17	2.92	2.77	3.2	2.86	3.41	2.88	3.11	2.76	3.54	3.02	3.18	3.02	3.17	3.79	3.64	3.26	3.05	2.76	2.74	4.43	4.57	3.3
	3.69	3.17	2.99	2.77	3.29	2.86	3.41	2.88	3.11	2.85	3.54	3.02	3.53	3.02	3.17	3.87	3.64	3.26	3.05	2.76	2.74	4.43	4.57	3.3
	3.74	3.39	2.99	2.77	3.29	2.86	3.64	2.92	3.43	2.96	3.54	3.16	3.53	3.02	3.17	3.88	3.86	3.26	3.17	2.76	2.74	4.43	4.57	3.38
	3.88	3.41	3.11	2.78	3.52	3.05	3.64	2.92	3.7	2.96	3.59	3.16	3.53	3.17	3.17	3.88	3.86	3.33	3.2	2.76	2.96	4.43	4.57	3.38
	3.88	3.41	3.11	2.95	3.53	3.26	3.64	2.92	3.7	2.96	3.59	3.26	3.53	3.44	3.18	3.88	4.38	3.33	3.2	2.76	2.96	4.43	4.57	3.38

3.88	3.41	3.11	2.95	3.64	3.26	3.64	2.92	3.7	2.96	3.59	3.54	3.53	3.44	3.23	3.88	4.38	3.51	3.2	2.91	2.96	4.43	4.57	3.38
3.88	3.46	3.24	2.95	3.64	3.26	3.64	3.18	3.7	3.01	3.59	3.82	3.64	3.44	3.23	3.9	4.38	3.51	3.5	2.91	2.99	4.51	4.75	3.51
3.88	3.47	3.33	3.13	3.64	3.26	3.91	3.18	3.7	3.01	3.59	3.82	3.64	3.76	3.23	3.9	4.38	3.52	3.53	2.91	3.06	4.51	4.75	3.51
3.91	3.81	3.33	3.13	3.66	3.54	3.91	3.18	3.7	3.01	3.59	3.82	3.94	3.77	3.23	3.9	4.38	3.81	3.53	3.25	3.07	4.51	4.75	3.67
3.91	3.81	3.33	3.13	3.72	3.54	3.91	3.2	3.7	3.01	3.76	4.01	3.94	3.77	3.23	3.97	4.38	3.81	3.53	3.26	3.07	4.8	4.87	3.67
3.91	3.81	3.33	3.16	3.83	3.54	3.91	3.2	4.28	3.05	3.77	4.3	3.94	3.77	3.23	3.97	4.38	3.81	3.53	3.26	3.28	4.8	4.87	3.67
3.97	3.83	3.65	3.16	3.9	3.54	3.96	3.2	4.28	3.05	3.77	4.3	3.94	3.77	3.47	3.97	4.38	3.81	3.53	3.26	3.28	4.8	4.87	3.91
3.97	3.85	3.65	3.16	3.9	3.57	3.96	3.2	4.28	3.05	3.77	4.3	3.94	3.77	3.47	3.97	4.38	3.81	3.53	3.26	3.43	4.81	4.87	3.91
3.97	3.85	3.65	3.16	4.25	3.57	3.96	3.37	4.28	3.12	3.77	4.3	4.24	3.77	3.47	3.97	4.38	3.81	3.53	3.55	3.43	4.81	4.94	3.91
4.04	3.85	3.65	3.19	4.25	3.98	3.96	3.37	4.28	3.13	3.83	4.3	4.24	3.77	3.47	3.97	4.38	4	3.8	3.55	3.43	4.81	4.95	3.91
4.04	3.97	4.1	3.31	4.28	3.98	3.98	3.37	4.28	3.22	3.96	4.3	4.24	3.81	3.54	3.97	4.38	4	3.8	3.56	3.43	4.99	4.95	4.33
4.14	4.02	4.1	3.31	4.28	3.98	4.01	3.37	4.28	3.22	3.96	4.8	4.24	3.81	3.54	4.06	4.7	4	3.8	3.85	3.43	4.99	4.95	4.33
4.29	4.03	4.11	3.31	4.28	3.98	4.01	3.59	4.28	3.22	3.96	4.94	4.24	3.96	3.54	4.06	4.7	4	3.8	3.9	3.43	4.99	4.97	4.41
4.32	4.26	4.11	3.31	4.68	3.98	4.01	3.59	4.28	3.22	3.96	4.94	4.5	3.96	3.83	4.06	4.7	4.41	3.8	3.9	3.82	5.4	4.97	4.41
4.32	4.26	4.11	3.31	4.69	3.98	4.09	3.59	4.28	3.47	4.43	4.94	4.5	3.96	3.83	4.1	4.7	4.41	3.8	3.9	3.82	5.4	5.12	4.41
4.55	4.26	4.36	3.31	4.93	4.53	4.09	3.59	4.28	3.47	4.43	5.1	4.5	3.96	3.83	4.28	4.7	4.41	3.8	3.9	4.12	5.4	5.12	4.41
4.55	4.29	4.36	3.69	4.93	4.53	4.09	4.14	4.28	3.47	4.43	5.16	5.01	3.96	3.83	4.28	4.91	4.41	3.98	3.98	4.12	5.4	5.12	4.65
4.71	4.54	4.36	3.69	5.08	4.53	4.42	4.14	4.51	3.47	4.43	5.26	5.01	3.96	3.83	4.28	5.14	4.64	4.2	3.98	4.29	5.4	5.22	4.65
4.73	4.57	4.36	3.69	5.09	4.53	4.42	4.14	4.55	3.48	4.43	5.26	5.01	4.26	3.83	4.28	5.14	4.64	4.2	3.98	4.29	5.4	5.22	4.65
4.79	4.57	4.56	3.73	5.14	4.53	4.42	4.14	4.55	3.74	5.03	5.26	5.01	4.28	4.42	4.28	5.14	4.64	4.2	3.98	4.29	5.94	5.31	5.08
4.79	4.69	4.64	4	5.17	4.53	4.66	4.14	4.55	3.74	5.03	5.45	5.01	4.28	4.42	4.42	5.14	4.88	4.39	3.98	4.82	5.94	5.46	5.08
5.25	5.06	4.64	4	5.21	4.96	4.66	4.76	4.55	3.94	5.03	5.65	5.01	4.28	4.42	4.42	5.52	4.88	4.54	4.49	4.82	5.94	5.46	5.08
5.25	5.06	4.64	4	5.21	4.96	4.66	4.76	4.55	3.95	5.03	5.66	5.25	4.28	4.42	4.67	5.52	4.88	4.54	4.49	4.82	6.44	5.46	5.1
5.25	5.09	5.01	4	5.22	4.96	4.67	4.76	4.61	4.11	5.03	5.66	5.57	4.53	4.42	4.67	5.52	4.99	4.54	4.49	4.82	6.44	5.48	5.1
5.25	5.09	5.01	4.37	5.22	4.96	4.67	4.76	4.61	4.11	5.03	5.66	5.57	4.53	4.42	4.67	5.52	4.99	4.54	4.63	4.82	6.44	5.59	5.4
5.55	5.09	5.01	4.37	5.22	4.96	4.67	4.76	4.61	4.11	5.03	6.09	5.57	4.56	4.42	4.88	5.52	5.19	4.54	4.63	4.82	6.46	5.96	5.4
5.55	5.09	5.3	4.37	5.22	4.96	4.86	4.76	4.61	4.11	5.03	6.09	5.57	4.56	4.42	4.88	5.52	5.2	4.68	4.77	4.82	6.47	6.03	5.8
5.55	5.57	5.3	4.37	5.23	4.96	4.89	4.76	4.61	4.11	5.19	6.09	5.57	4.56	4.88	4.97	5.52	5.2	4.68	4.77	4.96	6.47	6.41	5.8
5.55	5.57	5.3	4.66	5.23	4.96	5.35	4.76	4.93	4.11	5.2	6.27	5.57	4.56	4.88	5.06	5.9	5.2	4.68	4.77	4.96	6.47	6.41	5.8
5.62	5.57	5.3	4.66	5.27	4.96	5.38	4.9	4.93	4.11	5.2	6.48	5.57	4.56	4.88	5.06	6.18	5.2	4.68	4.99	5.29	6.47	6.66	5.8
5.62	5.57	5.3	4.8	5.32	4.96	5.38	4.9	4.93	4.6	5.33	6.48	5.57	4.56	4.88	5.17	6.19	5.2	4.68	5.03	5.29	6.47	6.66	6.15
5.81	5.75	5.3	5.09	5.35	5.34	5.38	4.9	4.93	4.6	5.38	6.48	5.57	5.18	4.88	5.17	6.19	5.65	4.93	5.05	5.29	6.5	6.66	6.15
5.81	5.75	5.47	5.1	5.56	5.34	5.38	4.9	4.93	4.6	5.38	6.82	5.73	5.24	4.97	5.35	6.54	5.65	5.57	5.05	5.29	6.5	6.66	6.15
5.91	5.97	5.47	5.1	5.56	5.34	5.38	5.03	4.93	4.61	5.53	6.82	6.03	5.24	5.19	5.35	6.54	5.65	5.57	5.05	5.29	6.5	6.99	6.59
5.91	6.01	5.47	5.1	5.56	5.34	5.38	5.03	4.94	4.61	5.73	6.82	6.03	5.24	5.19	5.35	6.54	5.66	5.57	5.27	5.29	6.5	6.99	6.59
5.91	6.01	5.47	5.1	5.56	5.34	5.42	5.03	4.94	4.62	6.04	6.82	6.03	5.24	5.19	5.6	6.54	5.94	5.57	5.27	5.29	7.02	6.99	6.59
5.91	6.31	5.68	5.4	5.69	5.34	5.42	5.03	4.94	4.62	6.22	6.82	6.03	5.24	5.19	5.62	6.56	5.96	5.57	5.64	5.6	7.03	7.32	6.6
6	6.36	5.72	5.4	5.96	5.34	5.42	5.03	4.94	4.65	6.22	6.82	6.03	5.24	5.31	5.62	6.73	5.96	5.57	5.64	5.6	7.03	7.39	6.62
6	6.36	5.72	5.4	5.96	5.34	5.95	5.03	4.94	4.65	6.22	6.91	6.05	5.27	5.31	5.62	6.73	5.96	5.57	5.64	5.6	7.03	7.7	6.62
6	6.69	5.81	5.4	5.96	5.34	6.01	5.19	4.94	4.65	6.51	7.32	6.48	5.76	5.31	5.66	6.73	6.15	5.57	5.64	5.62	7.03	7.7	6.64
6	6.69	5.81	5.4	5.97	5.34	6.01	5.19	5.75	4.72	6.51	7.32	6.48	5.76	5.31	5.66	6.73	6.15	5.58	5.64	5.94	7.55	7.7	6.64
6	6.76	5.81	5.4	5.97	5.34	6.01	5.19	5.75	4.82	6.51	7.32	6.64	.	5.52	5.66	6.73	6.15	5.58	5.64	5.94	7.55	7.7	6.64
6	6.78	5.95	5.4	6.1	5.78	6.01	5.19	5.75	4.82	6.51	7.32	6.64	.	5.52	5.97	6.91	6.17	5.87	5.93	6.04	7.55	7.7	6.64
6.09	6.78	5.95	5.4	6.1	5.78	6.08	5.19	5.75	5.1	6.51	7.32	6.64	.	5.52	5.97	6.91	6.28	5.87	5.93	6.04	7.55	7.93	6.64
6.09	6.78	6.01	5.69	6.15	5.78	6.08	5.65	5.75	5.1	6.51	7.32	6.64	.	5.52	5.97	6.97	6.28	5.87	5.93	6.04	7.55	7.93	6.74
6.09	6.8	6.01	5.69	6.15	5.78	6.08	5.65	5.75	5.1	6.51	7.32	6.64	.	5.52	5.97	6.97	6.28	5.87	5.93	6.04	7.55	7.93	7.01
6.09	6.88	6.01	5.69	6.15	5.78	6.08	5.65	5.75	5.26	6.53	7.32	6.64	.	5.52	5.97	7.32	6.28	5.88	5.93	6.04	7.55	7.93	7.05
6.09	6.9	6.01	5.93	6.21	5.78	6.08	5.65	5.75	5.26	6.54	7.32	6.64	.	5.52	5.97	7.32	6.44	5.88	5.93	6.04	7.55	7.93	7.05
6.09	6.94	6.01	5.94	6.23	5.78	6.08	5.65	5.75	5.26	6.54	7.32	7.08	.	5.52	5.97	7.37	6.45	5.88	6.06	6.04	7.55	8.11	7.05
6.16	6.94	6.01	5.94	6.24	5.78	6.08	5.65	5.75	5.51	6.84	7.32	7.08	.	5.52	5.97	7.37	6.45	5.88	6.06	6.04	7.55	8.11	7.05
6.16	6.94	6.33	5.94	6.33	5.78	6.08	5.65	5.75	5.51	6.84	7.32	7.08	.	5.74	6.05	7.8	6.45	5.88	6.06	6.04	7.55	8.11	7.2
6.16	6.94	6.33	5.94	6.33	5.78	6.08	5.95	5.75	5.51	6.84	7.32	7.08	.	5.74	6.05	7.8	6.54	6.42	6.06	6.04	7.55	8.13	7.2
6.16	7.11	6.34	5.94	6.33	6.01	6.08	5.95	5.75	5.63	6.84	7.32	7.08	.	5.74	6.05	7.8	6.54	6.42	6.29	6.11	7.55	8.13	7.2
6.26	7.11	6.34	6.17	6.36	6.01	6.08	5.95	5.75	5.63	6.84	7.32	7.08	.	5.74	6.05	7.8	6.54	6.42	6.29	6.11	7.55	8.13	7.2
6.26	7.11	6.39	6.45	6.44	6.01	6.08	5.95	5.75	5.63	6.84	7.66	7.08	.	5.74	6.05	7.8	6.56	6.42	6.29	6.11	7.55	8.13	7.2
6.26	7.11	6.46	6.45	6.52	6.09	6.08	5.95	5.75	5.63	7.12	7.66	7.46	.	5.74	6.4	8.33	6.56	6.42	6.29	6.11	7.55	8.16	7.2
6.36	7.21	6.46	6.45	6.61	6.09	6.08	5.95	6.08	5.66	7.15	7.66	7.46	.	5.86	6.41	8.33	6.56	6.42	6.29	6.11	7.7	8.28	7.44
6.36	7.26	6.46	6.46	6.73	6.09	6.08	5.95	6.08	5.66	7.15	7.67	7.46	.	5.86	6.41	8.33	6.57	6.47	6.29	6.11	7.76	8.28	7.44
6.36	7.28	6.46	6.47	6.73	6.09	6.09	5.95	6.08	5.66	7.15	7.67	7.46	.	6.12	6.41	8.33	6.57	6.47	6.29	6.11	7.76	8.28	7.44
6.36	7.56	6.46	6.47	6.73	6.09	6.14	5.95	6.08	5.66	7.15	7.97	7.46	.	6.12	6.41	8.33	6.57	6.47	6.49	6.21	7.76	8.28	7.44
6.43	7.56	6.55	6.47	6.81	6.31	6.14	5.97	6.08	5.82	7.5	7.97	7.46	.	6.12	6.75	8.33	6.57	6.47	6.49	6.21	7.76	8.44	7.44
6.57	7.56	6.55	6.47	6.81	6.31	6.14	6.33	6.08	5.83	7.5	7.98	7.71	.	6.12	6.75	8.33	6.57	6.95	6.49	6.21	8.06	8.44	7.44
6.67	7.56	6.55	6.47	6.81	6.31	6.14	6.33	6.08	5.83	7.5	7.98	7.71	.	6.12	6.75	8.33	6.72	6.95	6.65	6.32	8.07	8.44	7.92
6.67	7.56	6.55	6.75	6.9	6.31	6.15	6.33	6.08	5.83	7.5	8.04	7.71	.	6.12	6.75	8.33	6.72	6.95	6.66	6.33	8.07	8.7	7.95
6.83	7.56	6.55	6.75	6.9	6.31	6.22	6.33	6.08	6.03	7.5	8.04	7.71	.	6.12	6.77	8.33	6.82	6.95	6.86	6.33	8.07	8.7	7.95
6.83	7.93	6.55	6.75	6.9	6.31	6.22	6.33	6.08	6.04	7.5	8.04	7.71	.	6.29	6.77	8.33	6.82	6.95	6.86	6.33	8.31	8.7	7.95
6.93	7.93	6.55	6.75	6.9	6.31	6.22	6.34	6.08	6.04	7.5	8.04	8.28	.	6.3	6.77	8.33	6.85	7.14	6.86	6.33	8.31	8.85	7.95
6.93	7.95	6.55	6.97	6.9	6.31	6.22	6.34	6.08	6.04	7.5	8.24	8.28	.	6.3	7.11	8.59	7.01	7.14	6.87	6.65	8.31	8.85	7.95
7.11	7.95	6.55	6.97	6.99	6.99	6.35	6.34	7.06	6.04	7.5	8.28	8.29	.	6.3	7.23	8.59	7.01	7.14	7.2	6.65	8.33	9.1	7.95
7.11	7.96	6.55	6.97	6.99	7	6.35	6.34	7.06	6.04	7.5	8.28	8.29	.	6.3	7.23	8.59	7.01	7.14	7.27	6.97	8.33	9.1	7.95
7.11	7.97	6.82	6.97	7.05	7	6.35	6.78	7.06	6.39	7.51	8.41	8.29	.	6.3	7.23	8.59	7.1	7.14	7.46	6.97	8.33	9.1	7.95
7.11	7.99	6.82	6.97	7.11	7	6.35	6.78	7.06	6.39	7.51	8.41	8.29	.	6.3	7.23	8.59	7.1	7.14	7.46	7.21	8.33	9.1	7.95
7.11	7.99	6.82	6.97	7.11	7	6.35	6.78	7.06	6.39	7.68	8.41	8.29	.	6.3	7.23	8.59	7.1	7.15	7.69	7.21	8.33	9.57	7.95
7.35	8.03	6.82	6.97	7.11	7	6.35	6.78	7.06	6.39	7.68	8.52	8.29	.	6.3	7.33	8.59	7.15	7.15	7.69	7.21	8.33	9.57	7.95
7.35	8.03	6.82	6.97	7.19	7	6.35	6.78	7.06	6.39	7.68	8.52	8.29	.	6.3	7.33	8.77	7.47	7.15	7.69	7.56	8.33	9.57	7.95
7.35	8.03	7.03	6.97	7.19	7	6.35	6.79	7.06	6.39	7.68	8.53	8.29	.	6.84	7.33	9.02	7.47	7.83	7.69	7.56	8.33	9.61	7.95
7.35	8.03	7.13	6.97	7.19	7.55	.	6.79	7.06	6.82	7.91	8.64	8.45	.	6.84	7.33	9.02	7.47	7.83	7.69	7.56	8.33	9.61	8.1
7.35	8.03	7.13	6.98	7.38	7.64	.	6.87	7.06	6.82	7.91	8.64	8.86	.	6.84	7.33	9.02	7.47	7.83	7.69	7.94	8.33	9.61	8.1
7.5	8.42	7.13	7.07	7.47	7.64	.	6.87	7.06	6.82	7.91	8.64	8.86	.	6.84	7.35	9.07	7.47	7.83	7.69	7.94	8.38	9.8	8.74
7.5	8.43	7.13	7.12	7.47	7.64	.	6.87	7.06	6.82	8.46	8.64	8.86	.	6.84	7.35	9.13	7.47	7.83	8.29	7.94	8.38	9.8	8.74
7.82	8.46	7.13	7.12	7.47	7.64	.	6.87	7.06	6.82	8.46	8.64	8.86	.	6.84	7.35	9.15	7.78	7.83	8.29	7.96	8.98	9.8	8.74
7.91	8.46	7.13	7.17	7.61	7.64	.	6.87	7.06	6.82	8.46	8.87	8.86	.	6.84	7.35	9.15	7.78	7.83	8.55	8.49	8.98	10.15	8.74
7.97	8.73	7.33	7.24	8.03	7.64	.	7.55	7.06	6.82	8.46	8.94	8.86	.	7.36	7.35	9.15	7.78	7.83	8.55	8.49	8.98	10.15	9.12
7.97	8.73	7.51	7.29	8.03	7.99	.	7.55	7.06	6.82	8.46	8.94	8.86	.	7.36	7.37	9.15	7.78	7.84	8.55	8.49	8.98	10.15	9.12
7.97	8.73	7.51	7.29	8.03	7.99	.	7.55	7.06	6.82	8.46	9.16	8.86	.	7.36	7.52	9.15	7.78	7.96	8.55	8.81	8.98	10.15	9.12
8.1	9.04	7.55	7.29	8.03	7.99	.	7.55	7.06	6.9	8.93	9.16	8.88	.	7.36	7.52	9.88	8.17	7.97	8.55	8.81	8.98	10.5	9.12
8.1	9.04	8	7.54	8.06	8.04	.	7.55	7.96	6.9	8.93	9.32	9.03	.	7.36	7.52	10.05	8.17	8.25	8.81	8.81	8.98	10.5	9.12
8.23	9.07	8	7.54	8.06	8.04	.	7.55	7.96	7.13	8.93	9.78	9.03	.	7.61	7.83	10.05	8.17	8.25	8.81	8.81	8.98	10.5	9.37
8.23	9.42	8	7.54	8.06	8.04	.	7.56	7.96	7.15	8.93	9.78	9.18	7.31	7.61	7.83	10.05	8.18	8.25	8.81	8.81	8.98	10.5	9.37
8.23	9.42	8	7.54	8.09	8.04	.	7.69	7.96	7.24	8.94	9.78	9.18	7.43	7.61	7.83	10.05	8.18	8.25	8.81	8.81	8.98	10.53	9.37
8.42	9.42	8	7.79	8.09	8.04	.	7.7	7.96	7.24	8.94	10.29	9.18	7.43	7.61	7.85	10.05	8.18	8.25	8.85	9.13	9.15	10.53	9.37
8.43	9.42	8.1	7.8	8.19	8.34	.	7.75	7.96	7.24	8.97	10.77	9.18	7.43	7.61	7.85	10.05	8.22	8.25	8.85	9.13	9.24	10.53	9.42
8.82	9.46	8.1	7.8	8.34	8.34	.	7.75	7.96	7.24	8.97	10.77	9.36	7.43	7.61	7.85	10.05	8.58	8.25	8.85	9.13	9.28	10.53	9.52
8.82	9.46	8.45	7.8	8.34	8.34	.	7.75	7.96	7.49	9.32	11.01	9.36	7.43	7.84	8.33	10.05	8.61	8.25	8.85	9.22	9.28	10.95	9.52
8.82	9.46	8.54	7.8	8.41	8.34	.	7.77	7.96	7.49	9.32	11.01	9.36	7.43	7.84	8.33	10.06	8.61	8.25	9.15	9.22	9.28	10.95	9.52
8.82	9.52	8.54	8.13	8.59	8.34	.	7.8	7.96	7.5	9.32	11.01	9.36	7.43	7.84	8.33	10.16	8.61	8.25	9.15	9.23	9.28	11.38	9.86
8.82	9.56	8.54	8.13	8.59	8.85	.	8.06	7.96	7.5	9.32	11.01	9.4	7.98	7.84	8.33	10.72	9.03	8.3	9.15	9.23	9.28	11.39	9.87
8.82	9.69	8.54	8.44	8.62	8.85	.	8.06	7.96	7.51	9.32	11.07	9.72	8.16	7.84	8.34	10.72	9.03	8.3	9.42	9.75	9.29	11.39	10.02
8.94	9.69	8.71	8.44	8.66	8.85	.	8.06	7.96	7.51	9.32	11.14	9.72	8.16	7.84	8.78	10.72	9.03	8.3	9.42	9.75	9.69	11.72	10.02
8.94	9.79	8.71	8.44	8.81	8.85	.	8.06	7.96	7.63	9.32	11.14	9.72	8.16	7.84	8.78	10.72	9.03	8.3	9.43	9.75	9.69	11.84	10.02
9.31	9.84	8.71	8.44	8.88	9.03	.	8.06	7.96	7.63	9.32	11.14	9.72	8.16	7.84	8.78	10.72	9.03	8.69	9.6	9.75	9.69	11.84	10.46
9.31	9.87	8.71	8.52	8.88	9.03	.	8.06	7.96	7.63	9.32	11.14	9.72	8.16	7.84	8.78	10.73	9.03	8.69	9.6	9.75	9.69	11.84	10.46
9.65	9.87	9.21	8.87	8.89	9.03	.	8.07	7.96	7.74	9.43	11.14	9.72	9.08	7.84	8.78	10.73	9.49	8.69	9.6	9.75	9.69	11.84	10.47
9.65	9.87	9.21	8.89	8.9	9.03	.	8.07	7.96	7.74	9.43	11.14	9.72	9.08	8.07	8.78	10.73	9.49	8.69	9.72	9.75	9.69	11.84	10.47
9.65	9.9	9.21	8.89	8.9	9.03	.	8.15	7.96	7.74	9.43	11.14	10.24	9.08	8.07	8.78	10.73	9.49	8.69	10.11	9.75	9.69	12.24	10.92
9.65	9.96	9.21	8.89	8.93	9.03	.	8.56	7.96	7.74	9.43	11.21	10.24	9.08	8.07	8.78	10.77	9.5	8.69	10.11	9.75	9.69	12.39	10.92
9.65	9.96	9.21	8.89	8.93	9.03	.	8.56	7.96	7.83	9.43	11.21	10.24	9.08	8.07	8.78	10.77	9.64	8.69	10.11	9.75	10.23	12.39	10.92
10.04	9.96	9.21	8.89	8.93	9.03	.	8.56	7.96	7.83	9.86	11.21	10.24	9.08	8.07	9.03	11.27	9.65	8.69	10.11	9.75	10.5	12.39	10.92
10.04	10.09	9.59	8.89	8.93	9.16	.	8.56	7.96	7.83	9.86	11.21	10.24	9.08	8.07	9.03	11.27	9.66	8.69	10.11	9.75	10.5	12.94	10.92
10.04	10.09	9.59	9.1	8.93	9.16	.	8.56	7.96	8.41	10.2	11.23	10.24	9.08	8.07	9.03	11.3	9.66	8.69	10.11	9.75	10.5	13.13	10.92
10.28	10.09	9.59	9.38	8.95	9.16	.	8.56	7.96	8.42	10.2	11.23	10.24	9.08	8.15	9.5	11.3	9.7	8.69	10.15	9.75	10.5	13.37	10.92
10.28	10.09	9.6	9.38	9.04	9.16	.	8.56	7.96	8.42	10.2	11.27	10.24	9.08	8.15	9.5	11.3	9.7	8.69	10.15	9.75	10.5	13.37	11.11
10.28	10.09	9.6	9.38	9.04	9.16	.	8.56	7.96	8.42	10.2	11.27	10.24	9.08	8.15	9.5	11.49	9.7	8.69	10.15	9.75	10.5	13.37	11.48
10.28	10.12	9.6	9.38	9.04	9.16	.	8.56	7.96	8.46	10.45	11.34	10.24	9.08	8.15	9.5	11.49	9.7	8.69	10.31	9.75	10.5	13.37	11.48
10.28	10.38	9.6	9.82	9.04	9.22	.	8.56	7.96	8.46	10.45	11.34	10.6	9.08	8.19	9.5	11.49	9.7	8.69	10.31	9.75	10.5	13.37	11.55
10.28	10.38	9.6	9.82	9.05	9.22	.	8.77	7.96	8.46	10.45	11.34	10.6	9.08	8.19	9.5	11.49	9.7	8.73	10.45	9.75	10.5	13.37	11.56
10.28	10.38	9.6	9.98	9.05	9.22	.	9.18	7.96	8.46	10.45	11.35	10.6	9.08	8.19	9.5	11.5	9.7	8.95	10.53	9.75	10.5	13.37	11.56
10.47	10.7	9.78	9.98	9.05	9.22	.	9.19	7.96	8.48	10.45	11.35	10.6	9.08	8.19	9.5	11.5	9.7	8.95	10.53	9.75	10.5	13.37	11.56
10.47	10.72	9.78	9.98	9.08	9.22	.	9.19	7.96	8.48	10.45	11.35	10.6	9.08	8.22	9.5	11.67	9.7	8.95	10.53	9.75	10.5	13.37	11.85
10.47	10.72	9.78	9.98	9.11	9.22	.	9.19	7.96	8.63	10.48	11.35	10.6	9.08	8.38	9.5	12.11	9.76	8.95	10.53	9.75	10.5	13.37	11.87
10.47	10.72	9.78	9.98	9.11	9.22	.	9.19	7.97	8.63	10.48	11.35	10.68	9.08	8.38	9.5	12.11	9.76	8.95	10.53	9.75	10.5	13.37	11.87

	10.47	10.72	9.78	9.98	9.11	9.22	.	9.19	7.97	8.64	10.48	11.35	10.68	9.08	8.38	9.57	12.11	9.76	8.95	10.82	9.75	10.5	13.37	11.87
	10.47	10.74	9.78	9.98	9.11	9.22	.	9.19	7.97	8.64	10.48	11.51	10.68	9.08	8.38	9.57	12.11	9.76	8.95	10.82	9.95	10.59	13.37	11.87
	10.47	10.74	9.78	9.98	9.13	9.22	.	9.19	7.97	8.64	10.48	11.52	10.72	9.08	8.38	9.57	12.61	9.76	8.95	10.82	9.95	10.6	13.85	11.97
	10.47	10.74	9.83	9.98	9.13	9.22	.	9.19	7.97	8.64	10.48	11.52	10.72	9.08	8.38	9.65	12.61	9.76	8.95	10.84	10.2	10.6	13.85	11.97
	10.83	10.74	9.85	9.98	9.13	9.68	.	9.19	7.97	8.64	10.48	11.52	10.77	9.08	8.38	10.26	12.61	9.76	8.95	10.84	10.2	10.6	13.85	11.97
	10.83	10.99	9.86	9.98	9.39	9.68	.	9.19	7.97	9.19	10.48	11.52	10.85	9.08	8.39	10.26	12.61	9.76	9.07	11	10.2	10.98	13.86	11.97
	11.05	10.99	10.06	9.98	9.39	9.68	.	9.38	8.12	9.22	10.72	11.8	10.85	9.08	8.39	10.26	12.61	9.76	9.07	11.06	10.2	10.98	13.86	12.18
	11.05	11.32	10.06	10.21	9.39	9.68	.	9.61	9.08	9.22	10.72	11.93	10.85	9.08	8.39	10.26	12.61	9.79	9.07	11.24	10.2	10.98	13.86	12.2
	11.05	11.33	10.06	10.21	9.39	9.68	.	9.61	9.08	9.22	10.72	11.93	10.88	9.08	8.51	10.26	12.61	9.79	9.07	11.24	10.5	10.98	13.86	12.2
	11.05	11.33	10.06	10.21	9.39	9.68	.	9.61	9.08	9.22	10.72	11.93	10.96	9.08	8.51	10.26	13.15	9.79	9.07	11.24	10.5	10.98	13.86	12.75
	11.05	11.33	10.06	10.21	9.39	9.91	.	9.61	9.08	9.67	10.72	12.03	10.96	9.08	8.67	10.26	13.15	9.79	9.07	11.24	10.5	10.98	13.86	12.78
	11.05	11.33	10.07	10.21	9.55	9.91	.	9.61	9.08	9.67	10.72	12.03	10.96	9.08	8.67	10.26	13.15	9.81	9.07	11.24	10.51	10.98	13.86	12.78
	11.14	11.33	10.07	10.21	9.55	9.91	.	9.61	9.08	9.67	10.72	12.03	10.96	9.08	8.67	10.26	13.15	9.81	9.07	11.36	10.51	10.98	13.86	12.78
	11.14	11.36	10.07	10.21	9.72	9.91	.	9.61	9.13	9.67	11.05	12.03	11.21	9.08	8.67	10.26	13.15	9.81	9.07	11.36	10.51	11.18	13.86	12.78
	11.14	11.36	10.07	10.21	9.72	9.91	.	9.62	9.13	9.67	11.05	12.04	11.21	9.08	8.67	10.26	13.15	9.82	9.43	11.55	10.51	11.18	13.86	12.78
60kcal%	0	0	0	0	0	0	0	0	0	0	0	0	0	0	0	0	0.01	0.25	0.2	0	0	0	0	0
2:22	0.04	0	0.17	0.01	0.07	0.28	0.03	0.2	0.07	0	0.02	0.01	0.29	0.18	0.1	0	0.04	0.51	0.35	0.01	0.12	0	0.03	0.02
	0.12	0	0.17	0.18	0.17	0.28	0.03	0.2	0.21	0.15	0.02	0.26	0.29	0.32	0.1	0.3	0.13	0.51	0.45	0.01	0.34	0.19	0.03	0.02
	0.16	0.01	0.17	0.2	0.34	0.3	0.35	0.33	0.21	0.15	0.03	0.26	0.64	0.33	0.17	0.3	0.13	0.51	0.45	0.23	0.34	0.37	0.03	0.02
	0.16	0.01	0.17	0.2	0.35	0.51	0.35	0.52	0.52	0.42	0.07	0.26	0.64	0.33	0.38	0.3	0.13	0.51	0.75	0.24	0.34	0.37	0.3	0.02
	0.24	0.21	0.18	0.4	0.35	0.52	0.35	0.52	0.52	0.48	0.14	0.29	0.64	0.33	0.55	0.3	0.25	0.72	0.75	0.36	0.4	0.49	0.3	0.09
	0.24	0.22	0.23	0.4	0.61	0.53	0.46	0.52	0.52	0.48	0.24	0.37	0.64	0.36	0.55	0.3	0.52	0.81	0.75	0.36	0.8	0.49	0.33	0.33
	0.42	0.43	0.34	0.4	0.61	0.88	0.46	0.8	0.52	0.6	0.25	0.37	0.95	0.47	0.82	0.38	0.52	0.81	0.75	0.57	0.8	0.49	0.47	0.55
	0.42	0.47	0.36	0.55	0.61	0.88	0.79	0.8	0.84	0.65	0.36	0.37	0.95	0.47	0.83	0.48	0.52	0.81	1	0.57	0.8	0.49	0.47	0.55
	0.43	0.47	0.36	0.55	0.64	0.88	1.05	1.09	0.84	0.65	0.37	0.51	0.95	0.47	0.83	0.69	0.52	0.81	1	0.57	0.8	0.58	0.47	0.55
	0.54	0.57	0.59	0.55	0.64	0.88	1.05	1.09	1.1	0.67	0.57	0.7	0.95	0.81	1	0.69	0.7	1.07	1	0.57	1.05	0.67	0.76	0.55
18:00	0.54	0.57	0.59	0.55	0.89	0.89	1.05	1.09	1.1	0.67	0.58	0.7	1.21	0.81	1	0.69	0.85	1.07	1	0.84	1.06	0.67	0.76	0.55
	0.55	0.58	0.59	0.65	0.89	1.18	1.05	1.12	1.11	0.68	0.59	0.74	1.21	0.99	1.15	0.69	0.85	1.07	1.06	0.84	1.06	0.67	0.76	0.71
	0.62	0.65	0.59	0.69	0.89	1.18	1.36	1.12	1.12	0.8	0.79	0.74	1.21	0.99	1.15	0.75	0.92	1.07	1.11	0.84	1.06	0.96	0.8	0.71
	0.72	0.65	0.72	0.69	0.93	1.29	1.37	1.38	1.12	0.81	0.79	0.74	1.21	1.22	1.15	0.81	1.06	1.16	1.11	1.12	1.06	0.96	0.84	0.71
	0.72	0.66	0.72	0.69	0.93	1.29	1.37	1.38	1.12	0.91	0.79	0.89	1.22	1.26	1.34	0.81	1.15	1.16	1.11	1.12	1.28	0.96	0.84	0.71
	0.91	0.68	0.82	0.92	0.93	1.29	1.37	1.38	1.38	0.91	0.79	0.89	1.22	1.39	1.34	0.94	1.31	1.35	1.11	1.12	1.28	0.96	0.84	0.88
	1.2	0.78	0.82	0.93	0.93	1.34	1.65	1.38	1.44	0.92	0.94	0.89	1.3	1.4	1.34	0.99	1.31	1.35	1.11	1.23	1.59	1.31	1.11	0.88
	1.2	0.78	1.1	1.08	0.93	1.34	1.65	1.68	1.58	1.01	0.94	0.95	1.31	1.72	1.34	0.99	1.73	1.35	1.11	1.24	1.59	1.41	1.27	0.88
	1.2	0.78	1.19	1.08	1.03	1.34	1.65	1.68	1.58	1.02	0.94	1.04	1.31	1.73	1.34	0.99	1.73	1.35	1.11	1.24	1.78	1.41	1.31	0.88
	1.22	0.78	1.19	1.08	1.03	1.34	1.65	1.68	1.58	1.03	1.05	1.13	1.36	1.75	1.63	1.15	1.73	1.53	1.11	1.24	1.78	1.41	1.32	0.89
	1.23	0.78	1.19	1.08	1.18	1.34	1.65	1.68	1.58	1.08	1.05	1.23	1.36	1.75	1.63	1.15	1.9	1.81	1.46	1.47	1.78	1.41	1.49	1.21
	1.29	0.9	1.19	1.08	1.18	1.36	1.86	1.83	1.58	1.08	1.05	1.25	1.36	1.96	1.63	1.47	1.9	1.93	1.46	1.47	1.78	1.41	1.49	1.21
	1.44	1.06	1.19	1.08	1.18	1.5	1.86	1.83	1.58	1.22	1.23	1.25	1.36	1.96	1.63	1.47	2.09	1.93	1.77	1.47	1.94	1.41	1.49	1.21
	1.44	1.06	1.19	1.08	1.26	1.58	1.86	1.83	1.82	1.22	1.24	1.25	1.36	1.96	1.63	1.47	2.09	1.93	1.77	1.52	1.95	1.41	1.49	1.21
	1.44	1.09	1.19	1.29	1.27	1.58	1.86	1.97	1.82	1.29	1.24	1.29	1.4	1.96	1.63	1.49	2.09	1.93	1.77	1.74	1.95	1.77	1.79	1.36
	1.44	1.24	1.19	1.29	1.27	1.58	1.86	2.05	1.82	1.37	1.45	1.51	1.5	1.96	1.63	1.79	2.09	1.93	1.77	1.74	1.95	1.77	1.8	1.39
	1.66	1.31	1.33	1.29	1.28	1.71	1.86	2.05	1.83	1.41	1.45	1.51	1.5	1.96	1.64	1.79	2.09	2.08	1.77	1.74	2.22	1.77	1.91	1.39
	1.66	1.31	1.36	1.29	1.38	1.71	1.86	2.05	1.83	1.53	1.45	1.69	1.5	1.96	1.64	1.84	2.1	2.08	1.77	1.76	2.22	1.77	1.99	1.39
	1.73	1.32	1.36	1.32	1.38	1.71	1.86	2.05	1.83	1.53	1.53	1.69	1.5	2.47	1.65	1.84	2.11	2.08	1.77	1.76	2.22	1.77	1.99	1.39
	1.78	1.52	1.36	1.33	1.38	1.71	1.86	2.05	1.84	1.53	1.53	1.69	1.5	2.47	1.65	2.05	2.11	2.08	1.77	1.76	2.22	1.77	1.99	1.49
	1.78	1.52	1.51	1.34	1.38	1.71	1.86	2.05	2.03	1.53	1.53	1.69	1.67	2.47	1.65	2.05	2.11	2.08	1.77	1.8	2.59	1.77	1.99	1.5
	1.78	1.52	1.59	1.41	1.38	1.85	1.86	2.05	2.03	1.53	1.53	1.69	1.67	2.47	1.65	2.05	2.21	2.08	1.77	1.86	2.63	2.03	1.99	1.68
	1.8	1.52	1.59	1.45	1.38	1.85	1.87	2.05	2.03	1.53	1.53	1.69	1.67	2.47	1.65	2.11	2.21	2.08	1.77	1.86	2.63	2.16	1.99	1.76
	1.8	1.52	1.72	1.45	1.5	1.85	1.87	2.05	2.03	1.62	1.54	1.86	1.67	2.47	1.65	2.11	2.21	2.25	1.77	1.86	2.63	2.22	1.99	1.78
	1.8	1.52	1.72	1.45	1.5	1.85	1.87	2.24	2.03	1.83	1.54	1.88	1.67	2.47	1.82	2.11	2.21	2.25	1.77	1.86	2.75	2.22	2.11	1.78
	1.8	1.56	1.72	1.45	1.5	1.85	1.87	2.24	2.03	1.83	1.54	1.88	1.67	2.47	1.82	2.11	2.21	2.25	1.77	1.86	2.75	2.22	2.43	1.78
	1.91	1.64	1.72	1.45	1.68	1.85	1.87	2.24	2.03	1.83	1.68	1.88	1.75	2.47	1.82	2.11	2.21	2.31	1.77	1.86	2.87	2.5	2.43	1.78
	1.91	1.64	1.72	1.45	1.68	1.85	1.89	2.24	2.03	1.83	1.68	1.88	1.76	2.47	1.83	2.11	2.22	2.42	1.77	1.86	2.87	2.63	2.43	1.78
	1.91	1.64	1.72	1.49	1.68	1.85	1.89	2.36	2.03	1.89	1.68	1.88	1.76	2.47	1.83	2.21	2.22	2.42	1.78	1.92	2.87	2.63	2.59	1.92
	1.92	1.64	1.83	1.74	1.68	1.85	1.89	2.48	2.31	1.97	1.68	1.9	1.76	2.47	1.83	2.41	2.22	2.42	1.78	1.99	2.87	2.7	2.6	1.92
	1.97	1.64	1.95	1.74	1.68	1.85	2.09	2.48	2.31	1.98	1.68	1.9	1.76	2.47	1.83	2.41	2.22	2.53	1.78	2.02	2.87	2.7	2.6	1.93
	1.97	1.64	1.95	1.74	1.68	1.92	2.09	2.48	2.31	1.98	1.68	1.9	1.76	2.47	1.83	2.43	2.22	2.59	1.78	2.02	2.87	2.7	2.6	1.94
	1.97	1.64	2.01	1.75	1.68	1.92	2.09	2.51	2.31	1.98	1.68	1.9	1.76	2.47	1.83	2.43	2.22	2.61	1.92	2.02	2.87	2.7	2.6	1.94
	2.08	1.69	2.01	1.83	1.68	1.99	2.09	2.51	2.31	2.01	1.68	1.9	1.79	2.47	1.86	2.44	2.23	2.67	1.95	2.16	2.87	2.7	2.6	1.94
	2.08	1.69	2.02	1.86	1.8	1.99	2.09	2.51	2.31	2.01	2.04	1.99	1.79	2.51	1.86	2.44	2.23	2.67	2.29	2.16	2.88	2.7	2.6	1.94
	2.08	1.69	2.02	1.86	1.8	1.99	2.09	2.51	2.31	2.01	2.04	1.99	1.79	2.51	1.86	2.44	2.23	2.67	2.29	2.21	2.98	2.7	2.6	1.94
	2.08	1.69	2.02	1.86	1.8	1.99	2.49	2.51	2.42	2.01	2.04	1.99	1.79	2.51	1.86	2.44	2.37	2.67	2.29	2.62	2.99	2.7	2.69	1.94
	2.24	1.69	2.02	1.86	1.84	2	2.49	2.51	2.42	2.26	2.21	1.99	1.79	2.57	2.23	2.44	2.37	2.67	2.29	2.63	3.06	2.7	2.82	1.94
	2.24	1.93	2.06	1.86	1.94	2	2.49	2.83	2.42	2.26	2.21	1.99	2.03	2.57	2.33	2.52	2.62	2.67	2.29	2.63	3.06	2.8	2.82	2.01
	2.24	1.95	2.09	1.91	2.15	2.27	2.49	2.83	2.42	2.26	2.21	2.04	2.03	2.57	2.33	2.65	2.62	2.67	2.31	2.63	3.07	2.8	2.82	2.01
	2.24	1.95	2.14	1.91	2.15	2.27	2.49	2.83	2.63	2.26	2.37	2.04	2.03	2.57	2.33	2.65	2.62	2.67	2.48	2.78	3.31	2.8	2.9	2.01
	2.29	1.95	2.14	1.91	2.15	2.46	2.7	2.83	2.9	2.37	2.37	2.04	2.11	2.57	2.33	2.65	2.62	2.67	2.48	2.78	3.31	2.84	2.9	2.01
	2.29	1.98	2.14	1.91	2.16	2.46	2.7	2.83	2.9	2.4	2.38	2.05	2.11	2.58	2.48	2.65	2.62	2.86	2.48	2.78	3.5	2.93	2.9	2.1
	2.29	2.05	2.25	2.01	2.16	2.46	2.7	2.83	2.9	2.4	2.58	2.13	2.11	2.58	2.48	2.65	2.9	2.86	2.48	2.78	3.5	2.93	2.9	2.1
	2.33	2.08	2.25	2.01	2.16	2.67	2.7	2.83	2.9	2.4	2.58	2.13	2.11	2.58	2.76	2.91	2.9	2.86	2.6	2.78	3.5	2.93	2.97	2.21
	2.41	2.08	2.27	2.01	2.16	2.67	2.97	2.83	2.9	2.45	2.58	2.14	2.11	2.65	2.76	2.91	2.9	2.86	2.6	3.04	3.76	3.07	3.15	2.21
	2.41	2.08	2.27	2.01	2.29	2.67	2.97	2.83	2.9	2.55	2.58	2.14	2.11	2.74	2.76	2.91	2.9	2.96	2.6	3.04	3.76	3.07	3.15	2.21
	2.41	2.14	2.27	2.01	2.29	2.68	2.97	2.95	2.9	2.55	2.58	2.21	2.22	2.74	2.76	2.98	2.9	2.96	2.6	3.04	3.76	3.07	3.15	2.21
	2.41	2.14	2.27	2.01	2.29	2.77	2.97	2.95	3.09	2.55	2.58	2.21	2.23	2.74	2.76	2.98	2.9	2.96	2.6	3.12	3.76	3.07	3.15	2.21
	2.42	2.14	2.39	2.01	2.29	2.78	2.97	2.95	3.09	2.71	2.67	2.21	2.23	2.94	2.76	2.98	2.9	2.96	2.6	3.12	3.76	3.07	3.15	2.32
	2.48	2.3	2.39	2.01	2.29	2.78	3	2.95	3.21	2.71	2.72	2.37	2.23	2.94	2.76	3.11	3.05	2.96	2.6	3.12	3.76	3.07	3.35	2.32
	2.48	2.3	2.57	2.01	2.58	2.78	3.03	2.95	3.38	2.71	2.72	2.37	2.5	2.94	2.89	3.11	3.05	2.96	2.81	3.12	3.76	3.07	3.4	2.32
	2.48	2.3	2.57	2.04	2.58	2.78	3.03	3.27	3.38	2.71	2.72	2.54	2.5	2.94	2.89	3.11	3.05	3.1	2.81	3.29	4.18	3.07	3.4	2.33
	2.62	2.3	2.57	2.09	2.58	3.02	3.03	3.27	3.38	2.79	2.92	2.54	2.5	2.94	2.89	3.11	3.19	3.1	2.81	3.29	4.24	3.07	3.4	2.33
	2.82	2.5	2.57	2.09	2.58	3.02	3.29	3.27	3.38	2.79	2.92	2.54	2.76	3.19	2.89	3.11	3.19	3.1	2.81	3.29	4.24	3.46	3.4	2.33
	2.82	2.5	2.57	2.09	2.58	3.02	3.36	3.27	3.38	2.89	2.92	2.54	2.76	3.19	3.05	3.11	3.19	3.1	3.18	3.42	4.28	3.46	3.66	2.33
	2.83	2.5	2.73	2.15	2.78	3.02	3.36	3.27	3.38	2.89	2.92	2.75	2.76	3.19	3.06	3.57	3.38	3.1	3.18	3.45	4.3	3.46	3.66	2.33
	2.83	2.54	2.73	2.15	2.78	3.02	3.36	3.62	3.63	3.14	3.27	2.75	2.76	3.19	3.06	3.62	3.38	3.43	3.32	3.45	4.3	3.46	3.66	2.6
	2.94	2.57	2.89	2.15	2.78	3.39	3.53	3.62	3.63	3.14	3.27	2.75	2.95	3.19	3.34	3.62	3.38	3.43	3.32	3.71	4.48	3.81	4.03	2.6
	2.94	2.57	2.89	2.35	2.97	3.39	3.53	3.62	3.63	3.14	3.27	2.75	2.95	3.4	3.34	3.62	3.67	3.43	3.32	3.71	4.48	3.81	4.11	2.6
	3.07	2.74	3.19	2.35	2.97	3.39	3.53	3.63	3.63	3.41	3.56	2.75	2.95	3.41	3.49	3.62	3.67	3.43	3.32	4.04	4.75	3.81	4.11	2.6
	3.22	2.74	3.19	2.35	3.15	3.39	3.53	3.63	3.63	3.41	3.56	2.96	3.1	3.41	3.49	3.62	3.67	3.43	3.32	4.04	4.76	3.81	4.19	2.6
	3.28	2.74	3.19	2.35	3.15	3.39	3.77	3.64	3.82	3.41	3.56	2.96	3.16	3.77	3.49	3.88	3.9	3.68	3.32	4.04	4.76	3.81	4.19	2.71
	3.28	2.85	3.19	2.35	3.15	3.79	3.77	4.02	3.82	3.41	3.56	3.09	3.16	3.77	3.78	3.88	3.9	3.68	3.61	4.04	4.76	4.17	4.19	2.76
	3.28	2.87	3.4	2.46	3.33	3.8	3.77	4.02	3.82	3.51	3.6	3.09	3.16	3.77	3.78	4.11	3.9	3.68	3.61	4.2	4.76	4.17	4.33	2.93
	3.29	2.88	3.4	2.47	3.33	3.8	3.77	4.02	3.82	3.62	3.97	3.09	3.42	3.77	3.79	4.11	3.9	3.68	3.61	4.32	5.01	4.17	4.52	2.93
	3.29	2.93	3.4	2.47	3.33	3.8	3.77	4.03	3.82	3.63	3.97	3.09	3.42	3.77	3.81	4.11	3.9	3.86	3.61	4.32	5.01	4.17	4.52	2.93
	3.42	2.95	3.58	2.47	3.35	3.8	3.98	4.03	3.83	3.63	3.97	3.09	3.42	3.77	3.81	4.11	4.1	3.86	3.61	4.32	5.02	4.4	4.59	2.94
	3.68	2.99	3.6	2.47	3.35	3.92	4	4.03	3.83	3.63	4.16	3.26	3.42	3.8	4.05	4.11	4.11	3.86	3.61	4.32	5.05	4.4	4.82	3.09
	3.68	2.99	3.6	2.69	3.35	3.93	4	4.03	3.85	3.63	4.17	3.27	3.67	3.8	4.05	4.11	4.12	4.07	3.61	4.62	5.05	4.4	4.85	3.11
	3.68	2.99	3.75	2.69	3.35	3.94	4	4.03	4.27	3.64	4.17	3.5	3.67	3.81	4.05	4.11	4.42	4.07	3.61	4.63	5.05	4.62	4.88	3.11
	3.74	2.99	3.75	2.71	3.35	3.94	4	4.03	4.27	3.67	4.17	3.5	3.67	3.82	4.05	4.36	4.42	4.07	3.61	4.64	5.29	4.69	5.24	3.34
	3.92	2.99	3.75	2.75	3.35	3.94	4.04	4.03	4.27	3.67	4.28	3.5	3.68	3.82	4.08	4.36	4.42	4.08	3.61	4.64	5.3	4.69	5.24	3.34
	3.92	3.1	3.75	2.75	3.35	3.94	4.21	4.03	4.27	3.77	4.28	3.51	3.69	4.11	4.08	4.46	4.43	4.23	3.61	4.64	5.36	4.87	5.24	3.34
	3.92	3.14	3.92	2.87	3.53	4	4.21	4.28	4.27	3.77	4.28	3.51	3.71	4.25	4.37	4.46	4.43	4.23	4.01	4.64	5.45	4.87	5.24	3.34
	3.92	3.14	3.93	2.89	3.53	4	4.21	4.28	4.27	3.89	4.28	3.54	3.72	4.26	4.37	4.47	4.63	4.23	4.01	4.64	5.46	4.87	5.35	3.4
	4.09	3.14	3.93	2.89	3.53	4.12	4.21	4.28	4.27	3.89	4.39	3.61	3.72	4.26	4.37	4.47	4.63	4.23	4.01	4.64	5.72	4.87	5.35	3.42
	4.09	3.24	3.93	2.89	3.53	4.12	4.21	4.28	4.27	3.98	4.39	3.61	3.87	4.49	4.72	4.47	4.91	4.44	4.01	4.64	5.72	4.87	5.35	3.42
	4.09	3.3	4.15	2.89	3.53	4.12	4.64	4.28	4.27	4.16	4.39	3.61	3.87	4.49	4.72	4.47	4.91	4.44	4.01	4.74	5.72	4.87	5.44	3.42
	4.09	3.3	4.15	2.9	3.53	4.38	4.64	4.28	4.27	4.16	4.39	3.61	3.87	4.49	4.72	4.47	4.91	4.44	4.01	4.91	5.72	5.01	5.44	3.42
	4.09	3.39	4.15	2.99	3.79	4.38	4.64	4.28	4.27	4.16	4.39	3.69	3.87	4.52	4.72	4.61	4.91	4.55	4.01	4.91	5.72	5.07	5.44	3.53
	4.09	3.42	4.15	3	3.79	4.38	4.64	4.28	4.28	4.27	4.57	3.7	4	4.52	4.72	4.61	4.91	4.55	4.28	4.91	5.78	5.07	5.44	3.53
	4.13	3.46	4.24	3	3.81	4.38	4.89	4.28	4.28	4.27	4.57	3.78	4.01	4.52	4.73	4.74	4.91	4.55	4.66	5.13	5.86	5.64	5.75	3.54
	4.17	3.46	4.24	3.11	3.83	4.38	4.89	4.28	4.28	4.28	4.57	3.78	4.01	4.52	4.73	4.74	4.91	4.58	4.66	5.13	5.86	5.64	5.75	3.54
	4.27	3.46	4.24	3.13	4.13	4.53	4.89	4.28	4.28	4.29	4.57	3.78	4.01	4.52	4.73	4.74	4.91	4.58	4.66	5.13	6.13	5.64	5.77	3.54
	4.27	3.52	4.24	3.3	4.13	4.54	4.89	4.28	4.28	4.4	4.79	3.78	4.01	4.52	4.73	4.74	5.07	4.58	4.66	5.13	6.19	5.64	5.81	3.54
	4.27	3.52	4.29	3.3	4.13	4.54	4.89	4.28	4.28	4.4	4.79	3.78	4.01	4.52	4.73	4.9	5.07	4.58	4.66	5.29	6.19	5.64	5.83	3.59
	4.27	3.56	4.29	3.3	4.13	4.54	4.89	4.28	4.28	4.4	4.79	3.78	4.08	4.52	4.73	5.1	5.09	4.58	4.66	5.29	6.24	5.64	5.83	3.59
	4.37	3.58	4.29	3.3	4.13	4.54	5.21	4.28	4.28	4.41	4.8	3.78	4.08	4.52	4.73	5.11	5.09	4.58	4.66	5.37	6.26	5.64	5.83	3.59
	4.37	3.58	4.29	3.3	4.13	4.54	5.21	4.49	4.28	4.41	5.06	3.78	4.08	4.52	4.73	5.11	5.09	4.58	4.95	5.37	6.26	5.64	6.01	3.66
	4.44	3.69	4.29	3.3	4.13	4.54	5.21	4.49	4.28	4.58	5.07	3.78	4.08	4.52	4.73	5.14	5.11	4.58	4.95	5.37	6.49	5.64	6.25	3.66
	4.49	3.73	4.29	3.3	4.14	4.54	5.21	4.49	4.28	4.65	5.07	3.78	4.08	4.52	4.74	5.14	5.11	4.73	4.95	5.5	6.49	5.65	6.25	3.66
	4.49	3.77	4.34	3.37	4.15	4.79	5.21	4.49	4.28	4.65	5.07	3.78	4.18	4.52	4.92	5.16	5.11	4.73	4.95	5.5	6.49	5.66	6.41	3.66
	4.49	3.77	4.47	3.39	4.15	4.79	5.21	4.49	4.28	4.66	5.07	3.78	4.18	4.52	4.92	5.16	5.11	4.73	4.95	5.63	6.49	5.66	6.42	3.66

4.6	3.77	4.47	3.4	4.35	4.89	5.21	4.49	4.31	4.8	5.07	3.78	4.18	4.52	5	5.27	5.46	4.73	4.95	5.64	6.79	5.77	6.48	3.66
4.61	3.84	4.47	3.44	4.35	4.89	5.21	4.49	4.63	4.8	5.07	3.78	4.26	4.64	5.01	5.27	5.48	4.73	4.95	5.64	6.79	5.77	6.49	3.71
4.61	3.87	4.47	3.56	4.36	4.89	5.39	4.49	4.63	4.8	5.24	3.78	4.26	4.65	5.01	5.27	5.48	4.73	4.95	5.64	6.81	5.77	6.61	3.72
4.61	3.87	4.47	3.56	4.36	4.89	5.39	4.49	4.63	4.8	5.24	3.78	4.26	4.65	5.01	5.27	5.48	4.73	4.95	5.64	6.85	5.77	6.61	3.72
4.68	3.87	4.53	3.56	4.36	4.89	5.39	4.49	4.63	4.93	5.24	3.79	4.26	4.65	5.01	5.48	5.48	4.73	5.18	5.64	6.87	5.96	6.61	3.72
4.68	3.89	4.61	3.56	4.39	4.89	5.39	4.49	4.81	4.93	5.24	3.79	4.29	4.66	5.01	5.48	5.48	4.73	5.18	5.77	7.04	6.17	6.74	3.72
4.68	3.89	4.63	3.56	4.4	4.89	5.39	4.83	4.81	4.93	5.44	3.87	4.29	4.66	5.01	5.5	5.48	4.73	5.18	5.77	7.04	6.17	6.81	3.72
4.68	3.89	4.63	3.56	4.4	4.89	5.6	4.88	4.81	4.96	5.44	3.87	4.29	4.66	5.21	5.52	5.48	5.02	5.18	5.77	7.04	6.22	6.85	3.73
4.69	3.92	4.63	3.56	4.4	5.13	5.6	4.88	4.81	5.05	5.44	3.87	4.49	4.66	5.21	5.52	5.48	5.02	5.18	5.77	7.04	6.22	6.85	3.73
4.69	3.96	4.78	3.65	4.59	5.13	5.6	4.88	4.81	5.05	5.45	3.87	4.49	4.66	5.37	5.52	5.48	5.02	5.19	5.8	7.04	6.22	6.85	3.8
4.69	4.02	4.8	3.65	4.59	5.13	5.6	5.07	4.81	5.06	5.59	3.94	4.7	4.66	5.37	5.62	5.48	5.02	5.19	5.8	7.04	6.22	6.85	3.8
4.84	4.14	4.81	3.65	4.67	5.13	5.6	5.07	4.81	5.06	5.59	3.98	4.7	4.66	5.37	5.63	5.64	5.19	5.19	5.8	7.05	6.22	6.85	3.8
4.84	4.14	4.81	3.68	4.67	5.13	5.78	5.1	4.81	5.14	5.59	4.03	4.82	4.8	5.37	5.63	5.64	5.3	5.19	5.8	7.22	6.25	6.91	3.8
4.84	4.17	4.82	3.68	4.7	5.24	5.78	5.1	4.81	5.14	5.6	4.03	4.82	4.8	5.37	5.63	5.64	5.3	5.19	6.02	7.22	6.35	6.91	3.81
4.92	4.17	5.03	3.68	4.7	5.24	5.78	5.1	4.81	5.15	5.61	4.05	4.82	4.8	5.38	5.81	5.64	5.45	5.61	6.02	7.22	6.36	6.91	3.81
4.92	4.17	5.03	3.68	4.7	5.38	5.91	5.1	5.13	5.21	5.61	4.16	4.96	4.93	5.38	5.81	5.64	5.45	5.61	6.02	7.22	6.36	7	3.85
4.92	4.17	5.03	3.68	4.7	5.38	5.91	5.34	5.13	5.21	5.7	4.33	4.96	4.93	5.52	5.81	5.64	5.45	5.62	6.15	7.39	6.37	7	3.86
4.92	4.17	5.09	3.74	4.78	5.38	5.91	5.34	5.13	5.21	5.81	4.33	4.96	4.95	5.52	5.81	5.64	5.45	5.62	6.15	7.39	6.56	7	3.86
5.03	4.22	5.11	3.74	4.87	5.38	5.91	5.34	5.13	5.29	5.81	4.34	4.96	4.95	5.52	5.81	5.64	5.46	5.9	6.15	7.39	6.56	7	3.86
5.03	4.22	5.11	3.76	4.88	5.47	5.91	5.34	5.13	5.29	5.81	4.37	4.96	4.95	5.52	5.81	5.64	5.46	5.9	6.15	7.39	6.56	7.13	3.98
5.03	4.22	5.12	3.79	4.88	5.47	6.13	5.34	5.13	5.29	5.81	4.41	4.97	4.95	5.52	5.81	5.84	5.63	5.9	6.26	7.39	6.56	7.13	3.98
5.03	4.22	5.27	3.79	5.05	5.57	6.13	5.34	5.13	5.36	5.81	4.41	4.97	4.98	5.52	5.81	5.84	5.63	5.9	6.26	7.65	6.56	7.13	4.01
5.13	4.22	5.36	3.85	5.05	5.57	6.13	5.51	5.13	5.36	5.81	4.46	4.97	5.12	5.52	5.93	5.84	5.63	5.91	6.26	7.65	6.56	7.13	4.13
5.13	4.22	5.36	3.87	5.06	5.57	6.13	5.51	5.31	5.36	6.02	4.71	4.97	5.12	5.75	5.93	6.06	5.63	5.91	6.33	7.65	6.56	7.3	4.13
5.14	4.28	5.36	3.87	5.06	5.65	6.13	5.51	5.32	5.51	6.02	4.79	5.24	5.12	5.75	5.93	6.07	5.63	6.21	6.33	7.72	6.56	7.3	4.2
5.25	4.45	5.36	4.09	5.2	5.65	6.13	5.51	5.32	5.56	6.02	4.85	5.24	5.52	5.75	5.93	6.07	5.72	6.21	6.33	7.73	6.61	7.3	4.2
5.25	4.45	5.53	4.09	5.2	5.92	6.21	5.51	5.32	5.59	6.03	4.85	5.26	5.52	5.75	6.15	6.07	5.73	6.21	6.38	7.73	6.63	7.3	4.26
5.25	4.45	5.58	4.09	5.42	5.92	6.21	5.73	5.32	5.59	6.1	4.85	5.54	5.52	5.75	6.15	6.35	5.73	6.21	6.38	7.73	6.91	7.46	4.26
5.35	4.54	5.64	4.09	5.42	5.92	6.55	5.73	5.32	5.72	6.25	4.85	5.55	5.52	5.75	6.2	6.35	5.9	6.21	6.38	7.91	6.92	7.46	4.26
5.35	4.56	5.78	4.09	5.42	5.92	6.55	5.73	5.32	5.72	6.35	4.9	5.69	5.52	5.99	6.2	6.35	5.9	6.21	6.64	7.91	6.92	7.46	4.59
5.36	4.8	5.78	4.13	5.43	6.03	6.55	5.82	5.32	5.72	6.35	4.91	5.69	5.52	5.99	6.2	6.35	6.04	6.49	6.64	7.91	6.92	7.65	4.63
5.47	4.85	5.97	4.13	5.64	6.03	6.55	6.03	5.32	5.95	6.35	4.91	5.69	5.9	6	6.29	6.35	6.04	6.61	6.64	7.91	7.04	7.65	4.63
5.47	4.85	5.98	4.13	5.64	6.03	6.55	6.03	5.85	5.95	6.35	5.17	5.83	5.9	6.36	6.29	6.42	6.04	6.63	7.02	8.02	7.06	7.65	4.63
5.47	4.85	6	4.13	5.64	6.2	6.85	6.12	5.85	6.06	6.72	5.17	5.83	5.9	6.36	6.29	6.42	6.04	6.63	7.03	8.13	7.08	7.76	4.63
5.74	4.9	6.14	4.38	5.77	6.2	6.85	6.33	5.85	6.06	6.72	5.22	6.01	5.9	6.36	6.29	6.42	6.04	6.63	7.03	8.29	7.08	7.76	4.63
6.01	4.93	6.14	4.38	5.77	6.2	6.85	6.33	5.85	6.12	6.72	5.26	6.01	5.91	6.36	6.47	6.85	6.23	6.63	7.03	8.32	7.35	7.76	4.67
6.01	5.1	6.48	4.38	5.84	6.5	6.85	6.33	5.85	6.25	6.72	5.49	6.01	6.02	6.37	6.47	6.85	6.23	6.63	7.22	8.47	7.35	7.76	4.67
6.02	5.1	6.48	4.54	5.84	6.5	6.85	6.34	5.85	6.25	6.72	5.5	6.03	6.29	6.55	6.57	6.85	6.23	7.05	7.28	8.48	7.56	7.99	4.67
6.02	5.1	6.52	4.56	5.85	6.51	6.85	6.35	5.86	6.26	6.78	5.53	6.03	6.3	6.55	6.67	6.85	6.23	7.05	7.28	8.48	7.56	8.09	4.67
6.1	5.17	6.68	4.56	5.85	6.73	6.85	6.35	5.86	6.26	7.21	5.56	6.03	6.3	6.56	6.67	6.85	6.23	7.05	7.28	8.62	7.72	8.09	4.68
6.13	5.17	6.68	4.57	5.85	6.73	6.86	6.35	5.86	6.28	7.21	5.59	6.03	6.3	6.56	6.67	6.85	6.3	7.05	7.52	8.69	7.88	8.09	4.82
6.22	5.17	6.86	4.69	5.85	6.73	6.86	6.35	5.86	6.28	7.21	5.59	6.2	6.3	6.56	6.67	6.85	6.3	7.05	7.52	8.97	7.88	8.29	4.84
6.22	5.17	6.87	4.69	6.02	6.83	6.87	6.35	5.86	6.28	7.21	5.59	6.2	6.33	6.56	6.67	6.85	6.3	7.05	7.74	8.98	7.88	8.55	5.05
6.22	5.36	6.87	4.79	6.02	6.83	6.87	6.35	5.86	6.35	7.21	5.72	6.21	6.33	6.56	6.79	6.85	6.45	7.05	7.74	9.08	8.03	8.57	5.07
6.46	5.46	6.87	4.79	6.08	6.83	6.87	6.35	5.86	6.36	7.21	5.72	6.22	6.44	6.83	6.79	7.15	6.45	7.05	7.74	9.23	8.04	8.57	5.07
6.46	5.53	6.93	4.93	6.09	6.88	7.21	6.78	5.86	6.37	7.54	5.85	6.37	6.44	6.83	6.79	7.42	6.48	7.05	7.74	9.23	8.26	8.85	5.07
6.46	5.58	6.93	4.94	6.09	6.88	7.21	6.78	6.34	6.39	7.54	5.85	6.37	6.44	6.83	6.79	7.54	6.48	7.05	7.74	9.44	8.26	8.95	5.17
6.56	5.58	6.93	5.13	6.12	6.88	7.21	6.78	6.34	6.39	7.54	5.98	6.37	6.49	7.02	6.79	7.55	6.48	7.21	7.92	9.44	8.26	8.96	5.19
6.56	5.58	7.07	5.13	6.12	6.88	7.21	6.78	6.34	6.73	7.54	6.02	6.37	6.84	7.02	6.97	7.55	6.54	7.22	8.13	9.44	8.26	8.96	5.28
6.61	5.64	7.19	5.15	6.31	6.88	7.21	6.78	6.34	6.74	7.91	6.02	6.37	6.84	7.02	6.97	7.85	6.65	7.22	8.13	9.55	8.26	9.07	5.31
6.64	5.64	7.21	5.15	6.34	6.99	7.21	7.13	6.34	6.93	7.91	6.24	6.37	6.84	7.02	6.97	7.85	6.65	7.22	8.13	9.55	8.26	9.07	5.31
6.65	5.74	7.38	5.15	6.34	6.99	7.31	7.13	6.34	6.94	7.91	6.25	6.73	6.84	7.36	6.97	7.85	6.76	7.22	8.13	9.65	8.26	9.07	5.31
6.65	5.75	7.39	5.15	6.34	6.99	7.31	7.13	6.34	6.94	8.15	6.26	6.73	6.84	7.37	6.97	7.85	6.76	7.23	8.13	9.66	8.26	9.22	5.31
6.65	5.75	7.39	5.15	6.34	7.25	7.31	7.13	6.35	6.94	8.15	6.27	6.73	7.15	7.37	6.97	8.15	6.76	7.57	8.13	9.85	8.26	9.34	5.5
6.65	5.75	7.42	5.15	6.34	7.25	7.73	7.13	6.35	6.94	8.15	6.29	6.88	7.16	7.37	7.05	8.16	6.76	7.58	8.13	9.85	8.34	9.36	5.5
6.77	5.75	7.42	5.29	6.54	7.25	7.73	7.13	6.35	6.94	8.15	6.29	6.88	7.16	7.37	7.06	8.17	6.92	7.58	8.3	10.04	8.34	9.36	5.5
6.77	5.75	7.42	5.29	6.54	7.25	7.73	7.14	6.35	6.95	8.17	6.29	6.88	7.16	7.37	7.06	8.17	6.92	7.58	8.3	10.04	8.46	9.36	5.5
6.77	5.75	7.42	5.29	6.54	7.3	7.73	7.14	6.35	6.95	8.17	6.29	6.88	7.16	7.37	7.06	8.17	7.05	7.58	8.36	10.04	8.46	9.42	5.69
6.77	5.75	7.51	5.3	6.54	7.3	7.73	7.14	6.35	6.95	8.17	6.5	6.88	7.16	7.37	7.23	8.17	7.05	7.58	8.36	10.19	8.46	9.44	5.76
6.77	5.9	7.51	5.3	6.55	7.3	7.73	7.14	6.35	6.95	8.17	6.51	6.88	7.16	7.37	7.23	8.18	7.08	7.58	8.36	10.19	8.47	9.58	5.76
6.77	5.92	7.6	5.37	6.62	7.36	7.74	7.14	6.35	6.95	8.17	6.52	7.15	7.16	7.37	7.23	8.18	7.09	7.58	8.36	10.19	8.59	9.58	5.77
6.81	5.96	7.61	5.38	6.69	7.36	8.09	7.3	6.35	6.95	8.17	6.52	7.15	7.52	7.67	7.23	8.18	7.09	7.89	8.41	10.19	8.59	9.58	5.77
6.81	5.96	7.61	5.47	6.69	7.36	8.09	7.3	6.36	7.01	8.49	6.54	7.15	7.52	7.67	7.23	8.52	7.09	7.89	8.41	10.22	8.59	9.66	5.77
6.82	5.96	7.61	5.47	6.69	7.36	8.09	7.3	6.36	7.03	8.49	6.59	7.15	7.52	7.67	7.33	8.52	7.28	7.89	8.41	10.3	8.59	9.82	5.77
6.86	5.96	7.71	5.62	6.69	7.36	8.09	7.31	6.36	7.04	8.5	6.59	7.34	7.52	7.95	7.34	8.52	7.28	8	8.41	10.3	8.59	9.84	5.77
6.86	5.96	7.81	5.62	6.69	7.45	8.1	7.48	6.36	7.04	8.55	6.59	7.34	7.52	7.95	7.34	8.52	7.28	8	8.54	10.31	8.59	9.91	5.88
6.86	5.96	7.89	5.62	6.69	7.45	8.1	7.48	6.36	7.05	8.64	6.59	7.34	7.52	7.95	7.34	8.52	7.44	8	8.54	10.34	8.6	9.91	5.88
6.86	5.97	7.89	5.62	6.69	7.45	8.12	7.48	6.36	7.07	8.64	6.59	7.34	7.52	8.1	7.34	8.52	7.44	8.22	8.54	10.34	8.6	9.91	5.88
6.86	5.97	7.9	5.62	6.69	7.62	8.12	7.48	6.36	7.07	8.64	6.59	7.34	7.53	8.1	7.35	8.63	7.44	8.22	8.54	10.34	8.6	9.92	5.88
6.89	5.97	7.99	5.62	6.69	7.62	8.12	7.48	6.36	7.16	8.64	6.6	7.34	7.53	8.1	7.38	8.63	7.44	8.22	8.61	10.34	8.61	9.92	5.88
6.9	6.02	8.07	5.65	6.75	7.71	8.12	7.48	6.36	7.16	8.7	6.8	7.34	7.53	8.1	7.38	8.63	7.53	8.24	8.61	10.53	8.61	9.95	5.88
6.9	6.02	8.08	5.66	6.75	7.71	8.12	7.48	6.36	7.2	8.7	6.8	7.34	7.53	8.1	7.38	8.9	7.53	8.24	8.8	10.73	8.75	9.95	5.88
6.9	6.18	8.08	5.66	6.75	7.71	8.12	7.69	6.37	7.2	8.7	6.8	7.38	7.53	8.1	7.38	8.9	7.53	8.24	8.8	10.74	8.75	10.07	6.07
6.9	6.21	8.08	5.66	6.75	7.71	8.39	7.69	6.68	7.28	8.7	6.8	7.43	8	8.1	7.38	8.9	7.55	8.24	8.8	10.74	8.79	10.07	6.07
7.02	6.23	8.11	5.77	6.87	7.71	8.39	7.69	6.68	7.35	8.7	6.96	7.43	8	8.1	7.39	8.9	7.59	8.45	8.82	10.74	8.79	10.07	6.07
7.02	6.47	8.28	5.77	6.87	7.71	8.39	7.69	6.68	7.49	8.7	6.96	7.43	8	8.43	7.56	8.9	7.6	8.46	8.82	10.74	8.82	10.07	6.07
7.02	6.47	8.28	5.77	6.87	7.89	8.39	7.91	6.68	7.49	8.81	6.96	7.62	8	8.43	7.56	8.9	7.72	8.46	8.82	10.82	8.82	10.07	6.07
7.09	6.6	8.28	5.81	7	7.89	8.39	7.91	6.69	7.49	8.81	6.97	7.72	8	8.44	7.56	9.08	7.79	8.46	9.01	10.82	8.82	10.22	6.2
7.09	6.6	8.35	5.86	7	7.89	8.39	7.91	6.69	7.56	8.81	6.97	7.72	8	8.44	7.61	9.08	7.79	8.46	9.01	10.95	8.82	10.22	6.2
7.14	6.6	8.39	5.86	7	7.9	8.39	7.91	7.13	7.57	8.97	6.97	7.72	8	8.44	7.61	9.08	7.79	8.46	9.01	10.95	8.82	10.22	6.2
7.14	6.6	8.39	5.86	7	7.9	8.58	7.91	7.13	7.57	8.97	6.97	7.72	8	8.44	7.75	9.08	7.9	8.46	9.01	11.01	8.83	10.22	6.2
7.14	6.6	8.39	5.86	7	8.14	8.58	8.02	7.13	7.57	8.98	7	7.91	8	8.44	7.75	9.08	7.9	8.51	9.01	11.01	8.83	10.23	6.2
7.2	6.69	8.39	5.86	7	8.14	8.58	8.03	7.13	7.63	8.98	7	7.91	8	8.44	7.75	9.08	7.9	8.51	9.11	11.01	8.83	10.23	6.2
7.2	6.72	8.39	5.87	7	8.14	8.58	8.03	7.13	7.68	8.98	7	7.91	8	8.44	7.75	9.08	7.9	8.51	9.11	11.14	8.83	10.23	6.2
7.2	6.74	8.54	5.87	7.09	8.14	8.58	8.16	7.13	7.68	9.06	7.11	7.91	8.1	8.46	7.75	9.08	8.04	8.51	9.11	11.14	8.85	10.25	6.2
7.33	6.75	8.54	5.99	7.09	8.14	8.58	8.16	7.13	7.69	9.06	7.11	7.99	8.3	8.46	7.96	9.08	8.04	8.51	9.24	11.15	8.85	10.26	6.2
7.33	6.9	8.72	6.11	7.09	8.14	8.58	8.16	7.13	7.69	9.06	7.11	7.99	8.3	8.46	7.96	9.08	8.04	8.51	9.24	11.19	8.85	10.26	6.38
7.33	6.91	8.72	6.11	7.13	8.16	.	8.16	7.13	7.69	9.06	7.2	7.99	8.3	8.58	7.96	9.08	8.17	8.51	9.24	11.19	8.86	10.26	6.38
7.39	6.91	8.72	6.25	7.14	8.16	.	8.16	7.13	7.69	9.15	7.2	8.05	8.3	8.58	7.96	9.08	8.17	8.51	9.37	11.31	8.86	10.26	6.38
7.39	6.91	8.72	6.25	7.14	8.2	.	8.16	7.13	7.83	9.15	7.2	8.18	8.3	8.61	7.96	9.08	8.17	8.51	9.37	11.31	8.93	10.39	6.5
7.45	6.95	8.72	6.25	7.16	8.31	.	8.39	7.32	7.89	9.15	7.31	8.18	8.3	8.87	7.96	9.36	8.17	8.69	9.37	11.31	8.93	10.4	6.5
7.54	6.95	8.72	6.25	7.16	8.31	.	8.39	7.46	7.89	9.15	7.31	8.24	8.3	8.87	7.96	9.36	8.38	8.69	9.55	11.31	8.93	10.4	6.5
7.54	7	8.78	6.25	7.16	8.36	.	8.39	7.46	7.9	9.28	7.31	8.25	8.3	8.88	7.96	9.36	8.38	8.69	9.56	11.31	8.93	10.4	6.5
7.54	7.01	8.78	6.25	7.26	8.45	.	8.39	7.46	7.92	9.28	7.31	8.41	8.4	8.88	7.96	9.36	8.38	8.69	9.7	11.51	9.01	10.51	6.6
7.54	7.19	8.78	6.31	7.26	8.45	.	8.39	7.46	7.99	9.28	7.44	8.41	8.4	8.88	7.97	9.36	8.6	8.69	9.7	11.51	9.01	10.51	6.6
7.62	7.19	9.02	6.31	7.38	8.45	.	8.74	7.46	8.06	9.53	7.44	8.75	8.4	8.88	8.09	9.82	8.63	8.69	9.7	11.51	9.01	10.51	6.6
7.62	7.19	9.02	6.31	7.39	8.45	.	8.74	7.46	8.23	9.53	7.44	8.75	8.4	8.89	8.09	9.82	8.63	8.93	9.7	11.61	9.02	10.51	6.6
7.62	7.2	9.02	6.38	7.39	8.57	.	8.75	7.46	8.23	9.53	7.44	8.75	8.4	8.9	8.27	9.82	8.63	8.94	9.86	11.61	9.02	10.64	6.86
7.82	7.35	9.19	6.57	7.4	8.57	.	8.75	7.68	8.3	9.53	7.71	8.75	8.68	8.9	8.27	9.82	8.68	8.94	10.17	11.76	9.15	10.64	6.86
7.82	7.35	9.19	6.59	7.44	8.57	.	8.75	7.69	8.32	9.54	7.71	9.05	8.79	8.99	8.27	10.14	8.95	8.94	10.17	11.76	9.15	10.64	6.86
7.83	7.42	9.39	6.68	7.44	8.58	.	8.76	7.69	8.32	9.85	7.97	9.05	8.79	8.99	8.45	10.14	8.95	9.27	10.17	11.78	9.26	10.64	6.86
8.01	7.44	9.46	6.68	7.44	8.77	.	8.76	7.69	8.32	9.85	7.97	9.05	8.79	9.16	8.45	10.14	8.95	9.27	10.27	11.8	9.49	10.89	7.08
8.01	7.66	9.46	6.69	7.44	8.77	.	9.25	7.69	8.39	10.23	7.97	9.22	8.79	9.16	8.46	10.14	9.03	9.27	10.27	12.01	9.49	10.94	7.08
8.04	7.69	9.46	6.69	7.51	8.77	.	9.25	7.69	8.39	10.23	7.97	9.22	8.8	9.25	8.46	10.23	9.03	9.27	10.27	12.01	9.49	10.94	7.08
8.13	7.69	9.56	6.69	7.51	8.77	.	9.25	7.69	8.4	10.49	7.97	9.22	8.81	9.25	8.46	10.23	9.04	9.27	10.34	12.02	9.56	11.22	7.11
8.26	7.69	9.56	6.89	7.51	8.77	.	9.25	7.69	8.4	10.5	8.02	9.22	8.82	9.25	8.59	10.61	9.04	9.27	10.43	12.07	9.71	11.22	7.11
8.34	7.69	9.85	6.93	7.51	8.77	.	9.25	7.69	8.4	10.5	8.02	9.31	8.82	9.25	8.59	10.61	9.04	9.27	10.5	12.07	9.98	11.26	7.11
8.34	7.69	9.85	7.11	7.75	8.94	.	9.25	7.69	8.55	10.57	8.02	9.31	8.82	9.25	8.67	10.62	9.05	9.31	10.61	12.21	10.03	11.4	7.3
8.34	7.79	9.97	7.11	7.77	8.94	.	9.25	7.69	8.57	10.57	8.11	9.32	8.82	9.25	8.8	10.62	9.2	9.33	10.63	12.21	10.03	11.4	7.31
8.44	7.86	9.97	7.11	7.98	8.94	.	9.25	8.19	8.57	10.57	8.14	9.32	8.82	9.25	8.8	10.62	9.2	9.33	10.64	12.27	10.03	11.44	7.32
8.58	7.88	10.09	7.11	7.99	9.03	.	9.25	8.19	8.67	10.57	8.14	9.46	9.06	9.37	8.81	10.62	9.2	9.33	10.79	12.31	10.03	11.47	7.32
8.64	8	10.09	7.11	7.99	9.03	.	9.25	8.19	8.69	10.57	8.14	9.46	9.26	9.37	8.86	10.99	9.2	9.59	10.79	12.39	10.25	11.47	7.41
8.64	8.16	10.09	7.2	7.99	9.03	.	9.25	8.19	8.69	10.64	8.35	9.46	9.26	9.5	8.86	10.99	9.2	9.65	10.79	12.47	10.26	11.47	7.52
8.64	8.18	10.12	7.28	7.99	9.03	.	9.72	8.19	8.72	10.64	8.35	9.46	9.26	9.5	9.06	10.99	9.2	9.68	10.85	12.47	10.26	11.54	7.52
8.64	8.18	10.12	7.28	8.17	9.03	.	9.72	8.19	8.72	10.91	8.35	9.64	9.26	9.5	9.06	11	9.36	9.68	11.07	12.47	10.26	11.74	7.52
8.78	8.18	10.12	7.28	8.17	9.03	.	9.72	8.19	8.9	10.93	8.35	9.64	9.27	9.5	9.07	11	9.36	9.68	11.07	12.47	10.51	11.74	7.52
8.89	8.19	10.3	7.28	8.17	9.03	.	9.72	8.19	8.96	10.93	8.35	9.64	9.27	9.5	9.07	11	9.36	9.68	11.18	12.47	10.51	11.89	7.52
8.94	8.19	10.37	7.57	8.17	9.2	.	9.72	8.19	8.96	10.93	8.35	9.65	9.27	9.51	9.13	11.14	9.36	10.02	11.31	12.48	10.58	11.89	7.52
8.94	8.38	10.37	7.57	8.17	9.2	.	9.73	8.19	9.07	11.15	8.35	9.65	9.27	9.64	9.13	11.14	9.52	10.02	11.31	12.5	10.58	11.93	7.71
8.94	8.44	10.48	7.57	8.17	9.2	.	10.14	8.19	9.07	11.15	8.35	9.81	9.27	9.64	9.31	11.14	9.52	10.02	11.37	12.53	10.58	11.93	7.75
9.02	8.45	10.59	7.58	8.17	9.21	.	10.14	8.19	9.08	11.15	8.35	9.82	9.29	9.64	9.31	11.33	9.7	10.02	11.46	12.53	10.58	11.93	7.75

9.06	8.46	10.6	7.58	8.17	9.21	.	10.14	8.19	9.08	11.15	8.35	9.82	9.39	9.64	9.31	11.33	9.75	10.03	11.46	12.53	10.58	11.93	7.96
9.06	8.46	10.6	7.69	8.17	9.21	.	10.14	8.19	9.08	11.15	8.36	9.82	9.39	9.64	9.51	11.33	9.75	10.03	11.46	12.74	10.72	11.93	7.96
9.15	8.46	10.75	7.7	8.35	9.39	.	10.14	8.19	9.11	11.44	8.36	9.82	9.39	9.75	9.51	11.33	9.75	10.03	11.6	12.75	10.84	12.06	7.96
9.16	8.46	10.75	7.7	8.35	9.39	.	10.14	8.19	9.11	11.44	8.36	10.02	9.39	9.75	9.68	11.33	9.75	10.04	11.73	12.78	10.84	12.07	7.96
9.22	8.46	10.81	7.79	8.35	9.39	.	10.5	8.19	9.11	11.58	8.36	10.02	9.39	9.75	9.68	11.34	9.75	10.04	11.75	12.78	10.84	12.08	7.96
9.27	8.46	10.82	7.8	8.35	9.39	.	10.5	8.19	9.24	11.58	8.36	10.02	9.39	9.75	9.68	11.53	9.75	10.04	11.75	12.83	10.84	12.13	7.96
9.3	8.69	10.93	7.8	8.36	9.39	.	10.5	8.19	9.24	11.58	8.36	10.02	9.4	9.75	9.68	11.53	9.82	10.04	11.75	12.83	10.84	12.19	7.96
9.31	8.69	11.01	7.8	8.36	9.39	.	10.51	8.19	9.25	11.58	8.38	10.02	9.4	9.86	9.68	11.53	9.82	10.04	11.92	12.83	10.84	12.28	8.22
9.34	8.69	11.01	7.89	8.36	9.39	.	10.51	8.19	9.32	11.58	8.38	10.03	9.4	9.86	9.71	11.54	9.82	10.04	11.93	12.86	10.96	12.33	8.22
9.34	8.69	11.01	7.89	8.36	9.39	.	10.51	8.57	9.32	11.62	8.38	10.18	9.4	9.86	9.71	11.54	9.82	10.04	11.93	12.86	10.96	12.35	8.22
9.42	8.69	11.03	7.89	8.36	9.39	.	10.52	8.57	9.32	11.75	8.38	10.18	9.4	9.86	9.79	11.54	9.82	10.04	11.97	12.94	10.96	12.35	8.22
9.42	8.69	11.18	7.89	8.36	9.53	.	10.53	8.57	9.37	11.79	8.38	10.18	9.48	9.88	9.79	11.54	10.02	10.04	11.97	12.94	10.96	12.35	8.22
9.42	8.69	11.18	7.89	8.44	9.57	.	10.54	8.6	9.37	11.79	8.38	10.18	9.48	9.88	9.79	11.54	10.02	10.04	12.06	12.94	10.96	12.49	8.22
9.42	8.69	11.18	7.97	8.44	9.66	.	10.54	8.6	9.37	11.79	8.38	10.19	9.48	9.88	9.79	11.81	10.02	10.04	12.06	12.94	10.96	12.49	8.22
9.47	8.69	11.18	7.97	8.44	9.66	.	10.54	8.6	9.43	11.79	8.38	10.3	9.48	10.04	9.92	11.81	10.02	10.08	12.06	12.94	10.96	12.49	8.22
9.48	8.69	11.27	7.98	8.45	9.68	.	10.92	8.6	9.53	11.8	8.43	10.36	9.48	10.04	9.92	11.81	10.02	10.2	12.18	12.95	11.21	12.51	8.41
9.48	8.75	11.35	7.98	8.45	9.68	.	10.92	8.6	9.54	11.8	8.43	10.36	9.48	10.04	9.92	11.81	10.02	10.2	12.19	13.07	11.21	12.52	8.41
9.48	8.75	11.35	8.11	8.45	9.68	.	10.92	8.6	9.72	11.85	8.44	10.39	9.48	10.04	10.18	11.81	10.16	10.2	12.19	13.07	11.21	12.52	8.41
9.54	8.77	11.4	8.11	8.45	9.68	.	10.92	8.6	9.73	11.85	8.44	10.39	9.48	10.04	10.18	11.97	10.16	10.2	12.19	13.07	11.21	12.64	8.41
9.55	8.77	11.4	8.11	8.5	9.79	.	11.24	8.6	9.75	11.86	8.44	10.52	9.48	10.21	10.23	12.08	10.16	10.21	12.19	13.07	11.21	12.64	8.6
9.59	8.79	11.43	8.12	8.5	9.79	.	11.24	9.15	9.75	11.99	8.44	10.55	9.48	10.21	10.23	12.08	10.16	10.36	12.31	13.07	11.21	12.64	8.6
9.59	8.88	11.46	8.14	8.5	9.79	.	11.24	9.15	9.75	11.99	8.44	10.55	9.48	10.21	10.23	12.08	10.16	10.36	12.31	13.23	11.21	12.67	8.6
9.59	8.99	11.46	8.19	8.62	9.79	.	11.24	9.15	9.87	11.99	8.44	10.55	9.48	10.21	10.23	12.23	10.37	10.36	12.31	13.23	11.21	12.67	8.6
9.66	8.99	11.56	8.26	8.65	9.92	.	11.24	9.15	9.87	12	8.44	10.69	9.48	10.22	10.25	12.23	10.37	10.37	12.31	13.26	11.21	12.9	8.6
9.66	8.99	11.56	8.26	8.65	9.92	.	11.24	9.15	9.91	12.08	8.44	10.69	9.48	10.22	10.25	12.23	10.37	10.42	12.31	13.26	11.31	12.91	8.6
9.71	8.99	11.56	8.26	8.75	9.92	.	11.24	9.15	9.92	12.08	8.44	10.69	9.48	10.22	10.25	12.23	10.37	10.42	12.36	13.27	11.37	12.91	8.6
9.71	8.99	11.63	8.26	8.75	9.92	.	11.24	9.15	9.92	12.08	8.44	10.72	9.48	10.22	10.25	12.23	10.37	10.42	12.36	13.5	11.37	12.91	8.6
9.71	8.99	11.63	8.26	8.75	10.01	.	11.24	9.15	9.92	12.08	8.44	10.72	9.48	10.22	10.25	12.24	10.37	10.42	12.48	13.56	11.37	12.93	8.6
9.75	8.99	11.63	8.26	8.75	10.01	.	11.24	9.15	9.92	12.09	8.44	10.72	9.48	10.22	10.47	**12.24**	**10.37**	**10.42**	12.48	13.56	11.37	12.93	8.6
9.75	8.99	11.74	8.27	8.75	10.01	.	11.25	9.15	10.08	12.09	8.44	10.93	9.48	10.4	10.47	12.24	10.48	10.61	12.48	13.56	11.52	12.93	8.6
																			12.66	13.56	11.55	13.09	
																			12.66	13.57	11.55	13.09	
																			12.66	13.57	11.55	13.09	

3	4	4	5	7	7	8	10	10	4	4	5	8	7	7	10	10	12	12	12	1	1	1	1	2
Box-2	Box-6	Box-7	Box-6	Box-2	Box-5	Box-3	Box-1	Box-3	Box-1	Box-4	Box-5	Box-1	Box-6	Box-8	Box-7	Box-2	Box-3	Box-4	Box-7	Box-1	Box-3	Box-4	Box-5	Box-2
2.3	25.3	26.6	34.6	38	43.6	52.6	64	75	13.3	21.5	32.6	44.6	45	47	65	68	77	78	84	6.1	9.4	10.1	11.6	16.4
F	F	F	F	F	F	F	F	F	F	F	F	F	F	F	F	F	F	F	F	M	M	M	M	M
Malto	Malto	Malto	Malto	Malto	Malto	Malto	Malto	Malto	MCT	MCT	MCT	MCT	MCT	MCT	MCT	MCT	MCT	MCT	MCT	EtOH	EtOH	EtOH	EtOH	EtOH
FM	FM	FM	FM	FM	FM	FM	FM	FM	FT	FT	FT	FT	FT	FT	FT	FT	FT	FT	FT	ME	ME	ME	ME	ME
0	0	0	0	0	.	0	0	0	0	0	0	0	0	0	0	0	0	0	0	0	0	0	0	0
0	0	0	0.1	0	.	0	0	0	0	0	0.08	0	0.01	0	0	0	0	0	0	0.09	0.08	0.01	0.01	0
0	0	0.01	0.1	0	.	0	0	0	0	0	0.16	0	0.01	0	0	0	0	0	0	0.14	0.13	0.12	0.09	0.06
0.01	0	0.03	0.1	0	.	0	0	0	0	0	0.22	0	0.01	0.01	0	0	0	0	0	0.25	0.26	0.18	0.2	0.06
0.13	0	0.03	0.19	0	.	0	0	0	0	0	0.27	0	0.01	0.01	0	0	0	0	0	0.26	0.54	0.31	0.29	0.06
0.13	0	0.03	0.31	0.02	.	0	0	0	0	0	0.4	0.09	0.01	0.01	0	0	0.01	0	0	0.41	0.54	0.31	0.32	0.06
0.15	0	0.03	0.54	0.19	.	0	0	0	0	0	0.42	0.17	0.01	0.01	0	0	0.01	0	0	0.43	0.58	0.4	0.41	0.06
0.64	0	0.13	0.71	0.19	.	0	0	0	0	0	0.53	0.17	0.01	0.01	0	0	0.01	0	0	0.64	0.78	0.45	0.78	0.13
0.84	0	0.15	0.71	0.3	.	0	0	0	0	0	0.59	0.29	0.01	0.01	0	0.11	0.17	0	0	0.64	0.92	0.62	0.93	0.15
1	0	0.15	0.75	0.37	.	0	0	0	0	0	0.73	0.46	0.01	0.01	0	0.14	0.17	0	0	0.85	1.21	0.84	0.93	0.15
1.43	0	0.19	1.09	0.64	.	0	0	0	0	0	0.84	0.67	0.02	0.01	0	0.2	0.19	0	0	0.86	1.26	0.89	1.21	0.15
1.43	0.01	0.32	1.44	0.72	.	0	0.07	0	0	0.05	0.93	0.68	0.02	0.01	0	0.25	0.29	0.05	0	0.96	1.48	1.12	1.35	0.15
1.44	0.01	0.32	1.78	0.79	.	0	0.5	0	0	0.05	1.1	0.92	0.02	0.01	0	0.36	0.37	0.05	0	1.11	1.61	1.19	1.45	0.15
1.52	0.01	0.43	1.8	0.98	.	0	0.76	0	0	0.05	1.3	0.93	0.02	0.01	0	0.52	0.37	0.07	0	1.13	1.61	1.6	1.61	0.15
1.57	0.01	0.45	1.8	1.16	.	0	0.88	0	0	0.11	1.45	1.07	0.02	0.01	0	0.53	0.39	0.16	0	1.13	1.77	1.69	1.61	0.15
1.57	0.01	0.65	1.8	1.26	.	0	0.99	0.19	0	0.32	1.6	1.15	0.02	0.01	0	0.64	0.39	0.43	0.01	1.4	1.77	1.87	1.73	0.15
1.65	0.03	0.7	1.8	1.26	.	0	0.99	0.33	0.09	0.32	1.6	1.17	0.12	0.01	0	0.75	0.39	0.69	0.08	1.4	1.77	1.87	1.8	0.15
1.74	0.35	0.87	1.86	1.26	.	0	0.99	0.43	0.2	0.6	1.61	1.17	0.13	0.01	0	0.75	0.39	0.78	0.08	1.4	2.15	1.96	1.88	0.15
1.99	0.35	1.07	2.04	1.26	.	0	1.22	0.55	0.34	0.76	1.84	1.17	0.13	0.01	0	0.75	0.45	0.97	0.08	1.65	2.15	1.96	1.88	0.15
2.3	0.35	1.09	2.04	1.26	.	0	1.49	0.72	0.68	1.05	1.84	1.17	0.13	0.01	0	0.75	0.45	1.17	0.08	1.65	2.15	1.99	2.04	0.15
2.54	0.35	1.29	2.26	1.41	.	0.24	1.78	1.09	0.78	1.12	1.89	1.17	0.29	0.01	0	0.98	0.6	1.3	0.31	1.81	2.35	2.27	2.26	0.15
2.54	0.55	1.31	2.56	1.57	.	0.24	1.78	1.09	0.93	1.12	1.91	1.17	0.43	0.01	0	1.07	0.6	1.45	0.31	1.81	2.35	2.41	2.26	0.15
2.68	0.59	1.31	2.56	1.57	.	0.24	1.78	1.09	0.93	1.12	1.98	1.17	0.53	0.01	0	1.23	0.6	1.45	0.31	2.07	2.35	2.57	2.26	0.15
2.68	0.82	1.31	2.69	1.65	.	0.24	1.78	1.09	0.93	1.25	2.18	1.17	0.84	0.01	0	1.33	0.6	1.45	0.31	2.19	2.36	2.59	2.33	0.15
2.68	0.82	1.34	2.69	1.69	.	0.24	1.78	1.09	0.93	1.63	2.18	1.49	1.11	0.01	0	1.33	0.6	1.48	0.31	2.19	2.36	2.68	2.33	0.15
2.68	0.82	1.34	2.69	1.94	.	0.45	2.02	1.09	0.93	1.84	2.37	1.55	1.11	0.01	0	1.33	0.6	1.48	0.36	2.19	2.47	2.68	2.41	0.15
2.94	0.82	1.53	2.69	1.94	.	0.46	2.16	1.09	0.93	1.84	2.58	1.55	1.11	0.01	0	1.33	0.6	1.48	0.38	2.19	2.52	2.68	2.41	0.15
2.94	0.82	1.74	2.71	1.94	.	0.6	2.16	1.34	1.06	1.84	2.58	1.55	1.11	0.01	0	1.33	0.61	1.49	0.38	2.34	2.72	2.89	2.41	0.15
2.94	0.98	1.74	2.92	1.94	.	0.88	2.16	1.48	1.18	1.84	2.58	1.55	1.11	0.01	0	1.33	0.68	1.73	0.38	2.34	2.75	2.94	2.64	0.15
3.2	1.11	1.77	2.92	1.94	.	0.98	2.16	1.48	1.37	1.84	2.58	1.55	1.11	0.01	0	1.49	0.68	1.95	0.38	2.34	2.75	2.94	2.65	0.15
3.21	1.11	1.79	2.92	1.94	.	0.98	2.32	1.48	1.37	1.91	2.84	1.55	1.2	0.01	0	1.49	0.68	2.28	0.38	2.34	2.75	2.94	2.87	0.15
3.21	1.11	1.88	2.94	1.94	.	0.98	2.32	1.48	1.37	1.91	2.84	1.55	1.3	0.01	0	1.49	0.68	2.28	0.38	2.51	2.85	2.94	2.87	0.15
3.34	1.11	1.95	3.11	1.94	.	0.98	2.42	1.48	1.37	1.96	2.84	1.55	1.3	0.01	0	1.74	0.68	2.28	0.38	2.7	2.85	3.02	2.87	0.15
3.34	1.33	1.95	3.11	1.97	.	0.99	2.42	1.69	1.54	2.02	2.84	1.55	1.3	0.01	0	1.74	0.68	2.28	0.38	2.7	2.85	3.02	2.87	0.15
3.54	1.59	2.08	3.11	2.08	.	0.99	2.42	1.69	1.86	2.24	2.92	1.56	1.3	0.01	0	1.86	0.69	2.28	0.38	2.72	2.85	3.19	2.89	0.15
3.79	1.59	2.08	3.2	2.31	.	0.99	2.42	1.69	2.1	2.24	2.92	1.76	1.44	0.01	0	1.86	0.83	2.28	0.38	2.72	2.85	3.2	3.06	0.25
3.79	1.59	2.08	3.45	2.48	.	0.99	2.42	1.69	2.1	2.41	2.92	1.99	1.56	0.01	0	1.86	0.83	2.28	0.38	2.72	2.85	3.2	3.06	0.36
3.79	1.59	2.08	3.46	2.48	.	0.99	2.42	1.69	2.1	2.66	3.09	2	1.59	0.01	0	1.86	0.83	2.28	0.38	2.72	2.85	3.21	3.06	0.47
3.79	1.88	2.08	3.46	2.48	.	0.99	2.42	1.69	2.1	2.66	3.23	2	1.59	0.01	0	1.89	0.83	2.28	0.38	2.92	2.85	3.33	3.2	0.74
3.79	1.88	2.08	3.71	2.48	.	0.99	2.42	1.89	2.1	2.66	3.23	2	1.59	0.01	0	2.06	0.83	2.68	0.38	2.92	2.86	3.33	3.2	0.87
3.79	1.88	2.31	3.71	2.48	.	0.99	2.42	1.98	2.31	2.66	3.23	2	1.59	0.01	0	2.07	0.83	2.97	0.38	2.92	3.12	3.5	3.2	1.08
3.79	1.88	2.34	3.71	2.57	.	0.99	2.42	2.08	2.4	2.66	3.23	2.23	1.59	0.01	0	2.07	0.83	2.97	0.38	2.92	3.14	3.5	3.36	1.08
3.79	1.88	2.64	3.71	2.76	.	0.99	2.42	2.08	2.4	2.66	3.23	2.48	1.59	0.01	0	2.07	0.85	2.97	0.38	2.92	3.14	3.5	3.36	1.08
3.81	2.13	2.64	3.71	2.76	.	0.99	2.42	2.08	2.4	2.66	3.23	2.58	1.59	0.01	0	2.07	0.85	2.97	0.38	3.1	3.14	3.5	3.36	1.08
3.81	2.26	2.64	3.71	2.76	.	0.99	2.42	2.08	2.4	2.79	3.41	2.58	1.59	0.01	0	2.07	0.85	2.97	0.38	3.1	3.2	3.5	3.51	1.08
3.81	2.51	2.64	4.01	2.76	.	0.99	2.42	2.08	2.4	3	3.41	2.58	1.75	0.01	0	2.07	0.85	2.97	0.38	3.1	3.2	3.6	3.51	1.11
3.81	2.52	2.64	4.01	2.84	.	0.99	2.42	2.08	2.4	3	3.41	2.58	2.02	0.01	0	2.07	0.85	2.97	0.38	3.1	3.2	3.62	3.51	1.11
3.81	2.52	2.64	4.01	2.91	.	0.99	2.42	2.08	2.4	3.05	3.49	2.58	2.14	0.01	0	2.36	0.89	2.97	0.38	3.1	3.21	3.62	3.59	1.11
3.81	2.52	2.8	4.01	2.91	.	0.99	2.42	2.15	2.4	3.06	3.49	2.58	2.14	0.01	0	2.36	0.89	2.97	0.38	3.1	3.21	3.83	3.59	1.13
3.81	2.75	2.82	4.01	2.97	.	0.99	2.42	2.3	2.4	3.06	3.59	2.58	2.14	0.01	0.01	2.36	0.89	2.97	0.38	3.34	3.32	3.83	3.59	1.13
3.94	2.79	2.82	4.19	3.22	.	0.99	2.42	2.3	2.4	3.07	3.69	2.58	2.14	0.01	0.01	2.36	0.89	3.17	0.38	3.34	3.4	3.83	3.59	1.13
3.94	2.79	2.83	4.19	3.22	.	0.99	2.42	2.3	2.4	3.42	3.69	2.58	2.14	0.01	0.01	2.36	0.89	3.49	0.38	3.34	3.4	3.83	3.64	1.13
3.94	2.79	2.83	4.19	3.22	.	0.99	2.42	2.3	2.4	3.46	3.84	2.75	2.14	0.01	0.01	2.36	0.89	3.58	0.39	3.34	3.4	3.88	3.64	1.13
3.94	2.79	2.96	4.19	3.22	.	0.99	2.42	2.47	2.4	3.48	4.13	2.75	2.26	0.01	0.01	2.54	0.89	3.58	0.89	3.34	3.4	3.95	3.64	1.13
3.94	2.79	2.97	4.19	3.23	.	0.99	2.42	2.69	2.4	3.49	4.14	3.04	2.37	0.01	0.01	2.59	0.89	3.58	0.89	3.34	3.47	3.95	3.64	1.13
3.94	2.79	2.97	4.19	3.44	.	0.99	2.42	2.69	2.47	3.49	4.14	3.04	2.37	0.01	0.02	2.59	0.89	3.58	0.89	3.34	3.55	3.96	3.74	1.13
3.94	3.17	2.97	4.29	3.44	.	0.99	2.42	2.69	2.47	3.49	4.14	3.04	2.37	0.01	0.02	2.59	0.89	3.58	1.03	3.45	3.55	3.99	3.75	1.13
3.94	3.17	3.27	4.37	3.44	.	0.99	2.42	2.69	2.47	3.6	4.14	3.04	2.37	0.01	0.02	2.59	0.89	3.58	1.03	3.45	3.55	3.99	3.75	1.13
3.94	3.17	3.31	4.37	3.44	.	0.99	2.42	2.69	2.62	3.61	4.14	3.04	2.43	0.01	0.02	2.8	0.89	3.58	1.03	3.45	3.55	4.2	3.75	1.13
3.94	3.17	3.32	4.37	3.44	.	0.99	2.42	2.89	2.62	3.61	4.14	3.22	2.61	0.01	0.02	2.82	0.9	3.58	1.03	3.45	3.63	4.2	3.95	1.13
3.94	3.17	3.34	4.4	3.52	.	1.16	2.42	2.89	2.62	3.61	4.14	3.39	2.73	0.01	0.02	2.82	0.9	3.58	1.27	3.45	3.63	4.2	3.95	1.13
3.94	3.41	3.38	4.6	3.68	.	1.38	2.42	2.89	2.68	3.61	4.25	3.39	2.87	0.01	0.02	2.82	0.92	3.58	1.64	3.67	3.63	4.2	3.95	1.13
4.15	3.41	3.38	4.6	3.68	.	1.38	2.42	2.89	2.77	3.61	4.32	3.39	2.87	0.25	0.02	2.95	0.92	3.58	1.73	3.67	3.63	4.41	3.95	1.21
4.15	3.41	3.46	4.6	3.68	.	1.38	2.42	2.92	2.77	3.82	4.42	3.39	2.87	0.55	0.02	2.95	1.16	3.58	1.73	3.67	3.75	4.42	4.07	1.37
4.15	3.41	3.47	4.6	3.99	.	1.38	2.42	3.19	2.77	3.82	4.42	3.39	2.87	0.57	0.02	2.95	1.18	4.01	1.73	3.67	3.75	4.42	4.14	1.64
4.15	3.41	3.48	4.6	3.99	.	1.39	2.42	3.19	2.77	3.82	4.42	3.66	2.93	0.57	0.02	2.97	1.18	4.15	1.73	3.67	3.75	4.54	4.14	1.64
4.41	3.41	3.65	4.67	3.99	.	1.39	2.42	3.19	3.06	3.82	4.45	3.8	2.98	0.68	0.02	3.16	1.18	4.15	1.73	3.67	3.75	4.69	4.14	1.64
4.41	3.74	3.65	4.73	3.99	.	1.71	2.85	3.19	3.09	3.82	4.5	3.8	2.98	0.9	0.02	3.16	1.19	4.15	1.73	3.85	3.87	4.69	4.16	1.64
4.56	3.74	3.65	5	3.99	.	1.71	3.15	3.44	3.09	3.87	4.51	4.15	2.98	1.09	0.02	3.16	1.19	4.3	1.96	3.85	3.98	4.77	4.17	1.64
4.56	3.74	3.82	5	4.3	.	1.87	3.15	3.44	3.27	4.18	4.67	4.15	2.98	1.3	0.02	3.3	1.19	4.51	2.29	3.88	4.17	4.83	4.45	1.64
4.56	4.08	3.82	5.25	4.34	.	1.97	3.15	3.71	3.27	4.18	4.78	4.15	3.09	1.46	0.02	3.32	1.3	4.51	2.29	4.22	4.32	5.09	4.45	1.84
4.56	4.08	4.02	5.25	4.34	.	2.04	3.25	3.76	3.49	4.18	4.85	4.37	3.22	1.69	0.02	3.32	1.3	4.71	2.58	4.22	4.44	5.11	4.66	1.96
4.93	4.36	4.06	5.25	4.34	.	2.36	3.25	3.76	3.5	4.47	5	4.53	3.29	1.69	0.02	3.32	1.34	4.71	2.9	4.42	4.72	5.15	4.69	1.96
4.93	4.48	4.06	5.5	4.64	.	2.61	3.25	3.76	3.68	4.49	5	4.78	3.47	1.9	0.02	3.54	1.56	4.92	2.97	4.43	4.72	5.27	4.92	1.99
5.1	4.48	4.06	5.5	4.67	.	2.83	3.49	4.09	3.75	4.51	5.21	4.79	3.47	2.12	0.02	3.54	1.56	5.2	2.97	4.53	5.08	5.35	4.92	2.23
5.1	4.48	4.37	5.56	4.67	.	3.06	3.84	4.09	3.76	4.51	5.21	5.08	3.47	2.12	0.02	3.54	1.73	5.21	3.17	4.54	5.08	5.78	5.25	2.63
5.39	4.62	4.37	5.59	4.67	.	3.1	3.84	4.11	3.94	4.86	5.34	5.08	3.47	2.12	0.02	3.61	1.73	5.21	3.4	4.77	5.3	5.78	5.31	2.82
5.42	4.69	4.4	5.86	4.89	.	3.28	3.84	4.42	4.08	4.87	5.45	5.08	3.61	2.12	0.02	3.66	1.73	5.21	3.63	4.77	5.3	6.1	5.52	2.82
5.42	4.69	4.66	5.86	4.93	.	3.43	3.84	4.42	4.08	5.21	5.6	5.08	3.9	2.36	0.02	3.86	1.73	5.52	3.67	4.95	5.3	6.23	5.52	3.25
5.42	4.69	4.66	6.13	5.26	.	3.43	4.4	4.42	4.09	5.23	5.61	5.08	3.93	2.65	0.02	3.87	1.85	5.52	3.74	4.95	5.39	6.23	5.58	3.28
5.52	4.83	4.66	6.13	5.36	.	3.7	4.46	4.42	4.31	5.23	5.92	5.08	4.19	2.65	0.02	3.87	2.25	5.52	3.74	4.95	5.61	6.36	5.58	3.28
5.82	5.03	4.66	6.13	5.37	.	3.7	4.78	4.72	4.31	5.42	5.92	5.36	4.34	2.82	0.02	3.87	2.65	5.72	3.74	5.11	5.61	6.36	5.58	3.28
5.82	5.03	4.99	6.13	5.53	.	3.7	4.83	4.72	4.31	5.43	6.17	5.42	4.34	2.97	0.02	3.87	2.81	5.8	3.74	5.11	5.61	6.58	5.8	3.28
5.98	5.03	5.1	6.34	5.53	.	3.7	5.12	4.72	4.64	5.43	6.17	5.42	4.34	3.14	0.02	3.87	2.89	6.09	4.06	5.38	5.61	6.62	5.8	3.62
5.98	5.03	5.12	6.35	5.54	.	3.7	5.26	5.1	4.64	5.43	6.17	5.42	4.34	3.33	0.02	4.09	3.24	6.15	4.06	5.39	5.61	6.62	5.95	3.62
6.19	5.03	5.12	6.61	5.54	.	3.7	5.26	5.11	4.64	5.43	6.17	5.42	4.34	3.33	0.02	4.14	3.24	6.3	4.27	5.67	5.83	6.85	5.95	3.62
6.19	5.03	5.14	6.81	5.54	.	3.7	5.54	5.11	5.03	5.43	6.34	5.74	4.59	3.33	0.02	4.42	3.24	6.41	4.43	5.67	5.83	6.89	5.95	3.86
6.3	5.03	5.57	6.81	5.83	.	3.73	5.55	5.11	5.03	5.43	6.34	5.74	4.79	3.33	0.02	4.43	3.56	6.55	4.44	5.67	6.13	6.89	6.18	3.86
6.41	5.03	5.57	6.81	5.83	.	4.11	5.55	5.11	5.31	5.43	6.34	5.74	4.79	3.33	0.02	4.43	3.64	6.55	4.63	5.67	6.2	7.08	6.18	3.86
6.69	5.03	5.57	7.09	5.83	.	4.11	5.55	5.11	5.31	5.7	6.56	5.74	4.79	3.33	0.02	4.43	3.66	6.55	4.63	5.98	6.2	7.09	6.24	3.86
6.69	5.03	5.57	7.1	5.9	.	4.11	5.55	5.11	5.47	5.92	6.56	5.74	4.9	3.43	0.02	4.43	3.66	6.55	4.63	5.98	6.2	7.09	6.59	4.17
6.69	5.03	5.57	7.1	6.14	.	4.11	5.55	5.11	5.58	5.97	6.56	5.74	5.13	3.43	0.02	4.57	3.66	6.55	4.63	5.98	6.35	7.23	6.59	4.17
6.69	5.06	5.57	7.1	6.14	.	4.11	5.55	5.11	5.58	5.97	6.72	5.74	5.13	3.49	0.02	4.57	3.66	6.7	4.63	5.98	6.39	7.33	6.59	4.17
6.69	5.26	5.57	7.18	6.14	.	4.11	5.55	5.11	5.58	5.97	6.98	5.95	5.38	3.49	0.02	4.57	3.89	6.79	4.63	6.21	6.39	7.33	6.59	4.17
6.77	5.56	5.57	7.19	6.14	.	4.47	5.55	5.11	5.58	6	6.98	6.28	5.49	3.49	0.02	4.57	3.89	6.79	4.78	6.21	6.39	7.41	6.78	4.17
6.97	5.56	5.57	7.19	6.14	.	4.47	5.88	5.45	5.58	6.22	6.98	6.3	5.74	3.7	0.02	4.57	3.89	6.79	4.97	6.54	6.4	7.53	6.78	4.42
6.97	5.56	5.77	7.33	6.21	.	4.47	6.13	5.45	5.58	6.23	7	6.3	5.74	3.7	0.02	4.97	3.89	7	5.09	6.54	6.6	7.53	6.78	4.52
7.3	5.56	5.9	7.33	6.5	.	4.47	6.13	5.45	5.58	6.25	7.25	6.3	5.74	3.82	0.02	4.98	3.92	7	5.22	6.54	6.6	7.6	7.01	4.78
7.3	5.61	5.92	7.33	6.51	.	4.69	6.13	5.45	5.76	6.26	7.25	6.3	5.74	3.96	0.02	4.98	4.13	7	5.22	6.67	6.94	7.6	7.01	4.78
7.3	5.97	6	7.55	6.51	.	4.69	6.13	5.45	5.9	6.26	7.25	6.3	5.74	4.21	0.02	5.03	4.13	7	5.22	6.68	7.01	7.64	7.17	4.78
7.33	5.97	6.26	7.56	6.51	.	4.69	6.13	5.45	5.9	6.62	7.29	6.3	5.74	4.22	0.02	5.18	4.48	7.36	5.22	6.68	7.01	7.65	7.28	4.78
7.33	5.97	6.26	7.56	6.51	.	4.69	6.5	5.7	6.02	6.62	7.49	6.7	5.74	4.22	0.02	5.18	4.49	7.36	5.22	6.68	7.01	7.65	7.28	4.78
7.33	5.97	6.26	7.56	6.51	.	4.69	6.5	5.88	6.33	6.62	7.49	6.7	6.03	4.22	0.02	5.18	4.49	7.36	5.22	6.68	7.01	7.69	7.28	5.34
7.45	5.97	6.26	7.56	6.75	.	4.69	6.5	5.88	6.33	6.62	7.49	6.7	6.23	4.22	0.02	5.18	4.49	7.6	5.22	6.7	7.01	7.71	7.28	5.37
7.45	6.34	6.26	7.6	6.82	.	4.69	6.5	5.88	6.33	6.62	7.49	6.7	6.23	4.22	0.02	5.18	4.49	7.68	5.22	6.7	7.26	7.8	7.41	5.37
7.45	6.35	6.34	7.81	6.82	.	4.69	6.5	5.88	6.33	6.94	7.49	6.7	6.23	4.22	0.02	5.4	4.49	7.68	5.41	6.7	7.26	7.88	7.41	5.37
7.45	6.35	6.4	7.81	6.82	.	4.69	6.5	5.88	6.43	6.96	7.49	6.7	6.23	4.22	0.02	5.4	4.7	7.68	5.41	6.7	7.26	7.88	7.41	5.5
7.45	6.35	6.4	7.81	6.82	.	4.73	6.63	6	6.43	6.96	7.56	6.76	6.44	4.34	0.03	5.4	4.7	7.68	5.41	6.7	7.26	7.88	7.41	5.5
7.45	6.35	6.4	7.88	6.82	.	4.73	6.63	6	6.44	6.96	7.57	6.76	6.44	4.45	0.03	5.55	4.7	7.73	5.41	6.82	7.26	7.88	7.41	5.5
7.45	6.64	6.77	7.9	6.82	.	4.73	6.63	6.01	6.44	6.96	7.57	6.76	6.44	4.45	0.03	5.65	4.7	8.14	5.41	6.82	7.26	7.96	7.45	5.5
7.49	6.64	6.77	7.9	7.41	.	4.73	6.63	6.02	6.69	7.36	7.57	6.8	6.44	4.45	0.05	5.67	4.7	8.14	5.55	6.82	7.26	7.96	7.45	5.5
7.49	6.64	6.79	7.9	7.45	.	4.73	6.63	6.02	6.69	7.36	7.57	7.12	6.62	4.69	0.41	5.67	4.87	8.14	5.55	6.82	7.27	7.96	7.45	5.56
7.49	6.64	6.79	7.96	7.45	.	4.87	6.64	6.02	6.69	7.36	7.57	7.12	6.62	4.69	0.51	5.67	4.87	8.19	5.55	6.83	7.38	7.97	7.45	5.56
7.66	6.64	6.79	7.96	7.45	.	4.87	6.64	6.36	6.69	7.44	7.57	7.12	6.62	4.69	0.72	5.67	4.87	8.53	5.55	6.89	7.38	7.98	7.6	5.56

7.66	6.64	7.07	7.96	7.45	.	4.87	6.64	6.36	6.76	7.8	7.65	7.12	6.62	4.69	0.88	5.67	4.87	8.53	5.55	6.89	7.38	8.02	7.6	5.56
7.66	6.79	7.08	8.13	7.45	.	4.87	6.64	6.36	6.76	7.8	7.65	7.3	6.62	4.69	0.88	6.03	5.12	8.53	5.61	6.89	7.38	8.05	7.6	5.61
7.66	6.79	7.08	8.13	7.45	.	4.87	6.64	6.48	6.76	7.8	7.65	7.3	6.91	4.97	0.89	6.03	5.12	8.53	5.77	6.89	7.38	8.13	7.6	5.85
7.79	6.79	7.08	.	7.45	.	4.87	6.64	6.48	6.87	7.8	.	7.3	6.91	4.97	1.04	6.03	5.12	8.98	5.77	6.89	7.51	8.13	7.75	5.85
7.79	6.79	7.08	.	7.45	.	4.94	6.64	6.48	6.87	7.8	.	7.3	6.91	4.97	1.19	6.03	5.12	8.98	5.77	6.89	7.51	8.13	7.75	5.85
7.79	6.79	7.08	.	7.45	.	4.94	6.64	6.48	6.87	7.8	.	7.3	7.06	4.97	1.3	6.03	5.12	8.98	5.77	6.89	7.51	8.17	7.75	5.94
7.79	6.96	7.28	.	7.45	.	4.94	6.64	6.48	6.87	7.8	.	7.3	7.17	5	1.3	6.03	5.12	8.98	5.77	6.89	7.71	8.17	7.75	6.11
8	7.12	7.28	.	7.45	.	4.94	6.65	6.79	6.87	7.8	.	7.3	7.37	5.28	1.3	6.06	5.12	8.98	5.77	6.89	7.71	8.19	7.75	6.11
8.01	7.36	7.3	.	7.59	.	4.94	6.66	6.79	7.03	7.86	.	7.62	7.37	5.28	1.3	6.42	5.12	9.04	5.87	6.9	7.71	8.19	7.96	6.11
8.01	7.59	7.3	.	7.59	.	4.94	6.66	6.79	7.1	7.86	.	7.62	7.37	5.28	1.33	6.42	5.39	9.04	6.08	6.99	7.72	8.23	7.96	6.11
8.01	7.59	7.3	.	7.59	.	5.07	6.66	6.79	7.1	7.86	.	7.62	7.37	5.39	1.46	6.42	5.39	9.04	6.08	6.99	8.01	8.49	8.1	6.14
8.01	7.59	7.3	.	7.59	.	5.07	6.66	6.79	7.1	7.86	.	7.62	7.61	5.39	1.46	6.42	5.39	9.04	6.08	6.99	8.01	8.51	8.1	6.44
8.31	7.59	7.3	.	7.84	.	5.07	6.66	6.79	7.35	7.88	.	7.62	7.61	5.39	1.46	6.61	5.39	9.04	6.15	6.99	8.01	8.51	8.1	6.44
8.35	7.59	7.3	.	7.85	.	5.07	6.66	6.79	7.35	8.05	.	7.88	7.61	5.39	1.46	6.89	5.39	9.3	6.38	7.07	8.01	8.51	8.22	6.44
8.35	7.59	7.4	.	7.85	.	5.2	6.66	7.12	7.35	8.14	.	7.88	7.71	5.61	1.46	6.89	5.79	9.32	6.38	7.11	8.01	8.51	8.23	6.44
8.35	7.59	7.64	.	7.85	.	5.2	6.75	7.12	7.4	8.17	.	7.88	7.71	5.61	1.46	6.89	5.79	9.58	6.38	7.11	8.16	8.68	8.23	6.44
8.35	7.74	7.65	.	7.86	.	5.2	7.16	7.12	7.4	8.17	.	8.16	7.79	5.72	1.82	6.89	5.79	9.58	6.51	7.11	8.16	8.68	8.23	6.75
8.65	7.74	7.65	.	8.05	.	5.25	7.16	7.12	7.77	8.17	.	8.16	7.94	5.84	2.1	7.18	5.79	9.58	6.71	7.33	8.16	8.68	8.39	6.79
8.65	7.74	7.65	.	8.05	.	5.34	7.16	7.12	7.77	8.34	.	8.21	7.95	6.17	2.1	7.18	5.79	9.58	6.71	7.33	8.19	8.7	8.52	6.79
8.65	7.83	7.65	.	8.05	.	5.42	7.16	7.52	7.77	8.34	.	8.51	7.95	6.17	2.1	7.18	5.79	9.77	6.71	7.33	8.44	8.87	8.64	6.88
8.67	8.11	7.91	.	8.22	.	5.52	7.48	7.52	7.98	8.34	.	8.51	8.07	6.35	2.1	7.18	6.06	9.77	7.04	7.58	8.44	8.98	8.64	6.98
8.67	8.11	7.92	.	8.44	.	5.73	7.48	7.64	7.98	8.34	.	8.57	8.2	6.64	2.35	7.18	6.14	9.89	7.11	7.79	8.64	9.06	8.8	7.21
9.01	8.11	8.14	.	8.45	.	5.91	7.48	7.83	8.31	8.49	.	8.76	8.2	6.64	2.55	7.22	6.46	9.89	7.34	7.79	8.94	9.06	9.1	7.53
9.01	8.45	8.17	.	8.45	.	5.91	7.53	7.83	8.62	8.62	.	8.76	8.31	6.82	2.61	7.22	6.46	10.17	7.48	7.98	8.94	9.33	9.1	7.53
9.22	8.45	8.47	.	8.81	.	6.16	7.78	7.83	8.62	8.64	.	9.02	8.32	7.12	2.61	7.5	6.47	10.36	7.74	7.98	8.95	9.33	9.39	7.78
9.22	8.45	8.47	.	8.85	.	6.29	7.78	8.22	8.62	8.75	.	9.18	8.32	7.12	2.61	7.5	6.84	10.53	8.05	8.23	9.36	9.74	9.39	7.79
9.22	8.46	8.73	.	8.85	.	6.59	7.78	8.22	8.99	8.85	.	9.18	8.56	7.34	2.61	7.66	6.84	10.53	8.26	8.24	9.36	9.74	9.39	7.79
9.23	8.46	8.73	.	8.85	.	6.59	8.11	8.22	8.99	8.88	.	9.18	8.56	7.34	2.61	7.9	7.05	10.53	8.35	8.24	9.75	9.83	9.68	8.16
9.23	8.46	8.73	.	8.85	.	6.59	8.11	8.28	9.36	8.98	.	9.18	8.74	7.34	2.77	7.9	7.05	10.71	8.71	8.56	9.75	9.83	9.68	8.16
9.46	8.46	8.73	.	9	.	6.77	8.11	8.57	9.36	9.03	.	9.18	8.74	7.34	2.9	7.91	7.05	10.71	8.82	8.56	9.75	10.1	9.68	8.56
9.6	8.87	8.97	.	9.02	.	7.02	8.42	8.57	9.36	9.03	.	9.52	8.74	7.5	2.9	8.15	7.5	10.82	8.83	8.83	9.75	10.1	9.86	8.56
9.6	8.88	8.97	.	9.02	.	7.1	8.58	8.57	9.36	9.08	.	9.52	9.01	7.5	3.1	8.3	7.74	10.96	8.83	8.9	9.75	10.17	10.1	8.56
9.85	8.88	8.97	.	9.02	.	7.32	8.58	8.57	9.36	9.08	.	9.52	9.01	7.5	3.1	8.39	7.91	11.14	8.83	8.93	9.86	10.19	10.1	8.56
9.92	8.88	9.11	.	9.02	.	7.32	8.84	8.81	9.51	9.2	.	9.52	9.01	7.65	3.1	8.39	8.26	11.16	9.01	9.24	9.92	10.19	10.3	8.81
9.92	8.88	9.13	.	9.02	.	7.52	9.11	8.98	9.74	9.21	.	9.52	9.01	7.82	3.34	8.39	8.26	11.46	9.01	9.25	9.92	10.41	10.3	8.81
9.93	8.88	9.13	.	9.34	.	7.52	9.11	8.98	9.74	9.45	.	9.52	9.01	8.08	3.34	8.39	8.26	11.46	9.01	9.38	9.92	10.42	10.3	8.81
9.93	9.09	9.13	.	9.34	.	7.52	9.32	8.98	9.74	9.47	.	9.91	9.06	8.08	3.34	8.39	8.26	11.58	9.15	9.38	10.05	10.83	10.34	8.81
10.14	9.15	9.13	.	9.34	.	7.73	9.45	8.98	9.82	9.57	.	9.91	9.16	8.08	3.34	8.39	8.26	11.58	9.51	9.38	10.05	10.84	10.52	9.06
10.15	9.32	9.13	.	9.34	.	7.74	9.66	8.98	9.82	9.57	.	9.91	9.48	8.08	3.34	8.39	8.32	11.59	9.66	9.38	10.24	10.84	10.52	9.06
10.15	9.57	9.13	.	9.34	.	7.74	9.67	8.98	9.82	9.57	.	9.91	9.48	8.08	3.34	8.39	8.66	11.59	9.87	9.65	10.24	10.84	10.52	9.06
10.15	9.57	9.46	.	9.34	.	7.74	9.67	8.98	10.03	9.59	.	9.91	9.48	8.26	3.34	8.39	8.95	11.95	9.92	9.65	10.24	10.84	10.52	9.06
10.27	9.57	9.46	.	9.34	.	7.74	9.67	9.29	10.03	9.59	.	9.91	9.5	8.28	3.34	8.39	9.12	11.95	9.92	9.65	10.24	10.84	10.55	9.06
10.27	9.57	9.46	.	9.34	.	7.74	9.67	9.41	10.11	9.59	.	9.91	9.7	8.62	3.34	8.54	9.12	12.15	9.94	9.67	10.24	11.04	10.55	9.06
10.49	9.57	9.46	.	9.64	.	7.74	9.67	9.41	10.11	9.59	.	9.91	9.7	8.62	3.34	8.58	9.12	12.15	9.94	9.67	10.58	11.11	10.84	9.06
10.49	9.57	9.51	.	9.64	.	8.08	9.67	9.41	10.42	9.82	.	9.91	9.7	8.62	3.34	8.59	9.12	12.15	10.13	9.69	10.58	11.11	10.85	9.06
10.5	9.68	9.54	.	9.64	.	8.08	9.93	9.41	10.42	9.9	.	10.25	9.97	8.62	3.34	8.59	9.24	12.15	10.13	10.01	10.58	11.35	10.85	9.18
10.5	9.81	9.55	.	9.64	.	8.08	9.99	9.41	10.42	9.9	.	10.25	9.97	8.68	3.56	8.59	9.24	12.15	10.13	10.01	10.69	11.38	10.85	9.18
10.65	9.82	9.55	.	9.64	.	8.51	9.99	9.8	10.42	9.9	.	10.25	9.97	8.81	3.56	8.59	9.24	12.54	10.29	10.01	10.69	11.4	11.21	9.18
10.65	9.82	9.55	.	9.64	.	8.53	9.99	9.8	10.42	9.9	.	10.25	9.97	8.81	3.56	8.59	9.24	12.57	10.45	10.01	10.9	11.4	11.21	9.18
10.65	9.82	9.55	.	9.64	.	8.53	9.99	9.8	10.42	10.21	.	10.25	9.97	8.81	3.56	8.82	9.24	12.78	10.45	10.01	11.03	11.53	11.21	9.18
10.89	9.99	9.89	.	9.64	.	8.72	9.99	9.82	10.72	10.26	.	10.25	10.1	8.99	3.56	8.82	9.24	12.96	10.48	10.23	11.03	11.55	11.21	9.37
10.89	9.99	9.93	.	10.02	.	8.72	9.99	9.82	10.72	10.27	.	10.25	10.16	8.99	3.56	8.85	9.43	12.96	10.62	10.23	11.03	11.56	11.21	9.4
10.89	9.99	10.31	.	10.02	.	8.72	10.28	9.82	10.72	10.36	.	10.25	10.35	8.99	3.56	8.85	9.43	12.96	10.62	10.23	11.35	11.56	11.21	9.7
10.89	9.99	10.31	.	10.02	.	8.72	10.44	9.82	10.72	10.38	.	10.25	10.35	9.08	3.8	8.85	9.43	12.96	10.77	10.23	11.35	11.56	11.21	9.7
11.06	9.99	10.31	.	10.02	.	8.72	10.44	9.82	10.73	10.38	.	10.25	10.35	9.08	3.8	8.85	9.43	13.06	10.77	10.23	11.35	11.56	11.3	9.7
11.06	9.99	10.31	.	10.02	.	8.72	10.44	9.82	11.01	10.69	.	10.25	10.35	9.08	3.8	9	9.43	13.06	10.87	10.23	11.36	11.86	11.3	9.77
11.06	10.26	10.31	.	10.02	.	8.72	10.56	9.82	11.01	10.69	.	10.46	10.48	9.08	4	9	9.61	13.08	10.87	10.23	11.36	11.86	11.42	9.77
11.19	10.26	10.4	.	10.12	.	8.72	10.66	10.06	11.01	10.71	.	10.46	10.48	9.37	4.06	9	9.63	13.4	10.87	10.23	11.43	11.91	11.42	9.77
11.19	10.26	10.53	.	10.36	.	8.72	10.66	10.06	11.01	10.71	.	10.46	10.48	9.38	4.1	9	9.63	13.4	10.87	10.24	11.43	11.93	11.42	9.97
11.19	10.26	10.53	.	10.36	.	8.72	10.66	10.06	11.16	10.76	.	10.46	10.6	9.38	4.1	9	9.63	13.4	11.09	10.29	11.43	11.93	11.42	10.02
11.19	10.26	10.53	.	10.36	.	8.72	10.66	10.06	11.16	10.76	.	10.46	10.6	9.38	4.1	9.18	9.63	13.4	11.09	10.29	11.43	11.93	11.42	10.02
11.2	10.26	10.54	.	10.36	.	8.72	10.66	10.06	11.16	10.76	.	10.46	10.6	9.38	4.1	9.18	9.63	13.5	11.09	10.29	11.43	11.93	11.42	10.02
11.2	10.54	10.81	.	10.36	.	8.72	10.71	10.06	11.43	11	.	10.46	10.6	9.57	4.1	9.18	9.63	13.57	11.09	10.29	11.43	12.05	11.42	10.02
11.2	10.54	10.82	.	10.36	.	8.72	10.92	10.28	11.43	11.14	.	10.63	10.76	9.58	4.19	9.18	9.91	13.57	11.09	10.31	11.43	12.06	11.42	10.02
11.44	10.54	10.84	.	10.36	.	8.72	10.93	10.29	11.43	11.14	.	10.63	10.76	9.65	4.22	9.18	10.01	13.57	11.09	10.31	11.44	12.07	11.42	10.02
11.44	10.54	10.84	.	10.36	.	8.72	**10.93**	**10.29**	11.43	11.16	.	10.63	10.89	9.65	**4.22**	**9.18**	10.02	13.57	11.31	10.31	11.44	12.07	11.53	10.02

0	0	0	0	0	.	0	**0**	**0**	0	0	0	0	0	0	**0**	**0**	0	0	0	0	0	0	0	0
0.11	0.07	0.09	0.13	0	.	0	0	0.3	0.07	0.2	0.18	0	0.26	0.02	0.24	0.14	0.1	0.25	0	0.08	0.02	0.12	0.11	0
0.19	0.4	0.33	0.27	0	.	0.21	0	0.3	0.07	0.45	0.18	0	0.26	0.02	0.29	0.3	0.1	0.25	0.41	0.09	0.02	0.37	0.28	0.33
0.39	0.4	0.42	0.27	0	.	0.48	0.01	0.68	0.07	0.45	0.21	0.33	0.47	0.47	0.46	0.64	0.1	0.51	0.41	0.31	0.23	0.38	0.36	0.33
0.39	0.4	0.57	0.5	0	.	0.48	0.03	0.68	0.42	0.45	0.58	0.33	0.67	0.47	0.48	0.64	0.1	0.72	0.66	0.31	0.24	0.47	0.55	0.33
0.68	0.58	0.72	0.5	0.31	.	0.48	0.49	0.68	0.42	0.82	0.59	0.74	0.68	0.47	0.61	0.91	0.1	0.72	0.72	0.59	0.58	0.47	0.61	0.78
0.68	0.58	0.72	0.51	0.31	.	0.48	0.6	1.15	0.42	0.82	0.59	0.74	0.95	0.84	0.61	0.91	0.1	0.72	0.72	0.59	0.58	0.58	0.61	0.78
0.68	0.58	0.72	0.78	0.31	.	0.79	0.66	1.15	0.8	0.83	0.6	0.74	1.05	0.84	0.65	0.91	0.1	0.72	1	0.59	0.58	0.81	0.91	0.91
1.03	0.74	0.89	0.78	0.31	.	0.79	0.66	1.15	0.8	0.83	1.01	0.74	1.05	0.84	0.8	0.91	0.1	0.98	1.03	0.98	0.96	0.82	0.91	0.91
1.03	0.74	0.89	0.95	0.79	.	0.79	0.66	1.15	0.8	1.25	1.01	0.9	1.05	1.1	0.8	1.43	0.1	1.08	1.03	0.98	0.96	1.19	1.03	0.91
1.03	0.94	1.22	0.96	0.79	.	0.79	1.19	1.15	0.8	1.45	1.01	0.9	1.05	1.1	1.04	1.43	0.1	1.08	1.38	0.98	0.96	1.2	1.03	0.91
1.35	0.94	1.22	0.96	0.79	.	0.89	1.23	1.15	0.8	1.46	1.01	0.9	1.39	1.1	1.04	1.44	0.1	1.08	1.38	1.09	0.96	1.2	1.17	0.91
1.35	0.94	1.22	1.07	0.79	.	0.89	1.23	1.67	0.8	1.46	1.01	1.05	1.39	1.1	1.04	1.44	0.1	1.08	1.38	1.09	1.04	1.53	1.18	0.91
1.35	0.94	1.22	1.07	0.79	.	0.89	1.23	1.67	1.16	1.46	1.41	1.07	1.39	1.2	1.04	1.44	0.1	1.08	1.49	1.19	1.34	1.56	1.35	1.04
1.35	0.94	1.61	1.07	0.79	.	0.89	1.65	1.67	1.16	1.46	1.41	1.15	1.39	1.21	1.22	1.67	0.1	1.08	1.49	1.21	1.34	1.58	1.35	1.37
1.35	0.94	1.61	1.07	0.79	.	1.06	1.65	1.67	1.16	1.46	1.43	1.31	1.41	1.21	1.23	1.75	0.1	1.08	1.49	1.33	1.79	1.58	1.6	1.37
1.53	1.43	1.61	1.53	0.98	.	1.34	1.69	1.67	1.62	1.46	1.43	1.37	1.49	1.41	1.23	1.75	0.32	1.6	1.84	1.42	1.79	1.58	1.6	1.37
1.62	1.43	1.61	1.54	1.33	.	1.35	2.02	2.06	1.62	1.46	1.43	1.37	1.49	1.41	1.29	1.75	0.56	1.61	1.84	1.42	1.79	1.85	1.85	1.37
1.62	1.43	1.77	1.54	1.33	.	1.6	2.02	2.06	1.62	1.46	1.43	1.74	1.75	1.55	1.37	2.07	0.57	1.61	2.17	1.69	1.79	1.98	1.91	1.37
1.85	1.52	1.77	1.61	1.33	.	1.6	2.02	2.06	1.62	1.46	1.7	1.75	2	1.55	1.37	2.07	0.93	1.74	2.17	1.8	1.79	1.98	1.97	1.37
1.85	1.83	1.77	1.63	1.54	.	2.01	2.37	2.06	1.62	1.53	1.72	1.75	2	1.55	1.58	2.07	0.93	1.74	2.17	1.8	1.79	2	1.97	1.37
1.85	1.83	1.77	1.67	1.54	.	2.01	2.38	2.06	1.62	1.6	1.72	1.75	2	1.84	1.58	2.07	1.2	1.74	2.17	1.99	2.23	2	2.21	1.65
1.86	1.83	1.77	1.7	1.54	.	2.13	2.38	2.06	1.77	1.65	1.72	1.75	2	1.84	1.77	2.4	1.38	1.74	2.32	2.19	2.23	2.27	2.38	1.65
2.2	1.99	1.77	1.91	1.54	.	2.13	2.72	2.06	1.77	1.65	1.72	1.94	2.23	2.13	1.82	2.42	1.38	1.81	2.32	2.33	2.23	2.29	2.38	2.04
2.2	2.08	1.77	1.91	1.54	.	2.13	2.73	2.06	1.77	1.75	1.79	1.94	2.23	2.13	1.83	2.42	1.88	1.81	2.61	2.5	2.23	2.31	2.38	2.04
2.2	2.23	2.11	2.17	1.54	.	2.39	2.88	2.06	2.21	1.77	1.79	1.95	2.23	2.13	1.83	2.42	1.88	2.02	2.61	2.5	2.23	2.31	2.38	2.04
2.2	2.24	2.11	2.17	1.55	.	2.39	2.88	2.07	2.22	1.78	1.95	1.99	2.35	2.45	1.83	2.42	1.88	2.02	2.61	2.5	2.23	2.31	2.38	2.04
2.2	2.24	2.11	2.27	1.55	.	2.64	2.96	2.07	2.22	2.14	2.16	2.43	2.44	2.45	1.83	2.54	2.33	2.02	3.04	2.63	2.23	2.31	2.44	2.39
2.39	2.24	2.11	2.35	1.78	.	2.65	2.96	2.37	2.22	2.14	2.16	2.43	2.48	2.45	1.99	2.54	2.33	2.02	3.04	2.64	2.61	2.31	2.44	2.39
2.39	2.34	2.11	2.4	1.84	.	2.65	3.06	2.37	2.22	2.2	2.16	2.43	2.5	2.45	1.99	2.54	2.33	2.02	3.04	2.77	2.61	2.37	2.44	2.39
2.39	2.34	2.45	2.4	1.92	.	3.06	3.06	2.37	2.22	2.2	2.32	2.43	2.53	2.83	1.99	2.54	2.68	2.02	3.04	2.77	2.61	2.37	2.44	2.39
2.39	2.34	2.45	2.4	2.23	.	3.06	3.23	2.37	2.46	2.2	2.5	2.44	2.53	2.83	2.07	2.54	2.68	2.14	3.31	2.77	2.61	2.43	2.6	2.77
2.63	2.4	2.45	2.43	2.23	.	3.06	3.23	2.37	2.46	2.2	2.5	2.44	2.58	2.83	2.08	2.73	2.68	2.31	3.31	3.01	2.7	2.44	2.6	2.77
2.65	2.48	2.45	2.67	2.23	.	3.06	3.32	2.47	2.46	2.2	2.5	2.44	2.58	3.07	2.08	2.73	3.05	2.6	3.31	3.15	2.7	2.5	2.72	2.77
2.85	2.75	2.45	2.67	2.23	.	3.06	3.32	2.69	2.46	2.2	2.57	2.44	2.58	3.07	2.33	2.73	3.05	2.64	3.31	3.15	2.7	2.52	2.72	2.77
2.86	2.75	2.45	2.67	2.23	.	3.06	3.32	2.69	2.52	2.2	2.57	2.44	2.73	3.07	2.33	2.73	3.05	2.64	3.57	3.15	2.7	2.61	2.72	2.77
2.86	2.75	2.72	3.07	2.23	.	3.06	3.32	2.69	2.52	2.2	2.73	2.44	2.73	3.07	2.34	2.73	3.05	2.64	3.57	3.36	2.7	2.64	2.85	2.77
2.86	2.75	2.72	3.14	2.66	.	3.13	3.36	3.13	2.52	2.21	2.73	2.44	2.73	3.07	2.34	2.91	3.05	2.64	3.57	3.36	2.83	2.64	2.85	3.09
2.86	2.95	2.72	3.14	2.66	.	3.13	3.36	3.13	2.67	2.47	2.73	2.44	2.87	3.17	2.34	2.91	3.05	2.64	3.57	3.36	2.83	2.64	2.85	3.09
2.86	2.95	2.91	3.14	2.66	.	3.13	3.62	3.13	2.67	2.47	2.73	2.63	2.96	3.17	2.34	2.91	3.05	2.89	3.57	3.47	2.95	2.64	2.93	3.09
3.07	2.95	2.91	3.17	2.66	.	3.29	3.62	3.13	2.67	2.5	2.97	2.64	2.96	3.17	2.34	2.91	3.05	3.02	3.89	3.59	3.05	2.64	3.06	3.09
3.19	2.95	2.91	3.17	3.11	.	3.29	3.62	3.13	2.7	2.5	2.97	2.79	2.96	3.17	2.34	2.91	3.05	3.02	3.89	3.59	3.48	2.64	3.06	3.09
3.46	2.95	2.91	3.17	3.11	.	3.29	3.79	3.13	2.7	2.65	2.97	2.79	2.96	3.17	2.35	3.02	3.05	3.02	3.89	3.59	3.48	3.05	3.22	3.09
3.46	2.95	2.91	3.17	3.11	.	3.29	3.79	3.13	3.21	2.67	2.97	2.79	3	3.23	2.46	3.02	3.05	3.15	3.89	3.75	3.48	3.21	3.22	3.09
3.46	3.03	2.99	3.28	3.11	.	3.38	3.92	3.13	3.21	2.94	3	2.84	3.11	3.23	2.46	3.02	3.21	3.15	3.91	3.75	3.48	3.22	3.27	3.44
3.46	3.11	3.03	3.41	3.11	.	3.38	3.92	3.4	3.23	2.96	3.2	2.98	3.19	3.23	2.46	3.02	3.21	3.46	3.91	3.75	3.58	3.22	3.27	3.44
3.46	3.13	3.33	3.54	3.11	.	3.38	3.92	3.4	3.23	2.96	3.2	3.01	3.19	3.23	2.46	3.26	3.21	3.46	3.91	3.76	3.58	3.38	3.27	3.8
3.46	3.13	3.33	3.54	3.11	.	3.63	4.1	3.46	3.23	2.96	3.2	3.01	3.24	3.23	2.46	3.26	3.21	3.46	3.91	3.76	3.58	3.43	3.46	3.84
3.46	3.13	3.33	3.54	3.12	.	3.63	4.11	3.46	3.23	3.02	3.51	3.01	3.24	3.36	2.66	3.28	3.3	3.46	4.07	3.76	3.76	3.64	3.46	3.84
3.47	3.13	3.33	3.54	3.13	.	3.63	4.11	3.46	3.37	3.32	3.51	3.01	3.24	3.36	2.83	3.28	3.3	3.46	4.28	3.84	3.76	3.67	3.55	3.92
3.48	3.26	3.33	3.77	3.13	.	3.82	4.11	3.46	3.37	3.32	3.51	3.01	3.24	3.36	2.84	3.28	3.79	3.46	4.29	3.91	3.76	3.67	3.55	4.11
3.48	3.29	3.39	3.77	3.13	.	3.82	4.11	3.55	3.71	3.36	4.14	3.03	3.55	3.48	2.84	3.28	3.79	3.46	4.29	3.96	3.86	3.69	3.61	4.11
3.48	3.29	3.39	3.77	3.13	.	4.09	4.14	3.55	3.71	3.36	4.14	3.03	3.57	3.48	2.84	3.38	3.79	3.46	4.47	3.96	3.86	3.69	3.72	4.11
3.48	3.29	3.39	3.97	3.13	.	4.09	4.14	3.99	3.71	3.36	4.14	3.03	3.57	3.7	2.84	3.38	3.79	3.83	4.47	3.96	3.86	3.69	3.72	4.33

3.7	3.29	3.57	3.97	3.13	.	4.09	4.14	3.99	3.71	3.36	4.14	3.03	3.57	3.7	2.84	3.38	3.79	3.83	4.69	3.96	4.11	3.84	3.74	4.33
3.7	3.29	3.57	3.97	3.13	.	4.1	4.14	3.99	3.96	3.4	4.14	3.2	3.57	3.7	2.85	3.38	3.79	3.83	4.69	4.14	4.11	3.87	3.74	4.33
3.7	3.29	3.57	3.97	3.13	.	4.1	4.35	3.99	3.96	3.82	4.14	3.2	3.71	3.7	3.2	3.65	3.79	4.03	4.69	4.14	4.11	3.96	3.88	4.33
3.7	3.29	3.57	3.97	3.13	.	4.11	4.35	3.99	3.96	3.82	4.14	3.2	3.72	3.77	3.2	3.65	3.79	4.03	4.95	4.14	4.11	3.96	3.88	4.33
3.74	3.29	3.68	3.98	3.13	.	4.11	4.4	3.99	3.96	3.82	4.14	3.2	3.86	3.77	3.2	3.65	4.18	4.03	4.95	4.14	4.11	4.05	3.89	4.54
3.74	3.29	3.68	3.98	3.13	.	4.11	4.49	4.34	4.44	3.82	4.14	3.2	3.86	3.77	3.2	3.82	4.18	4.03	4.95	4.14	4.12	4.17	3.97	4.54
3.8	3.54	3.94	4.05	3.14	.	4.12	4.53	4.34	4.44	3.82	4.14	3.2	3.86	4.06	3.2	3.82	4.18	4.03	5.16	4.25	4.12	4.17	3.97	4.54
3.8	3.54	3.94	4.05	3.35	.	4.12	4.53	4.34	4.44	3.85	4.14	3.2	4.17	4.06	3.44	3.82	4.18	4.24	5.16	4.25	4.12	4.17	3.97	4.54
3.94	3.54	3.94	4.05	3.35	.	4.12	4.53	4.34	4.44	3.86	4.14	3.4	4.17	4.06	3.44	3.82	4.52	4.45	5.16	4.25	4.14	4.29	4.01	4.54
3.94	3.54	4.24	4.09	3.35	.	4.16	4.53	4.34	4.44	3.86	4.14	3.4	4.17	4.06	3.44	3.82	4.52	4.45	5.16	4.25	4.14	4.3	4.01	4.64
4.04	3.61	4.24	4.15	3.35	.	4.16	4.62	4.34	4.44	3.86	4.32	3.4	4.24	4.18	3.44	3.82	4.52	4.45	5.16	4.25	4.14	4.3	4.01	4.85
4.04	3.81	4.24	4.15	3.35	.	4.16	4.62	4.8	4.46	3.86	4.32	3.4	4.24	4.18	3.44	3.97	4.52	4.57	5.4	4.28	4.14	4.32	4.02	4.85
4.25	3.81	4.24	4.25	3.35	.	4.17	4.69	4.8	4.46	4.09	4.32	3.68	4.24	4.18	3.67	3.97	4.52	4.57	5.4	4.28	4.15	4.34	4.02	4.85
4.26	3.81	4.24	4.31	3.76	.	4.18	4.87	4.8	4.46	4.32	4.32	3.78	4.73	4.18	3.67	3.97	4.52	4.57	5.4	4.39	4.25	4.47	4.22	4.85
4.26	3.81	4.24	4.31	3.76	.	4.18	4.87	4.8	4.49	4.34	4.32	3.78	4.73	4.76	3.67	3.97	4.86	4.57	5.4	4.39	4.25	4.5	4.22	5.12
4.26	3.82	4.58	4.31	3.76	.	4.22	4.87	4.8	4.49	4.34	4.32	3.78	4.73	4.76	3.67	4.44	4.86	4.72	5.4	4.39	4.25	4.52	4.58	5.12
4.26	4.06	4.66	4.31	3.76	.	4.22	5.23	4.8	4.58	4.34	4.32	4.3	4.73	4.76	3.67	4.44	4.86	4.72	5.4	4.39	4.25	4.63	4.58	5.12
4.26	4.06	4.66	4.31	3.76	.	4.22	5.23	4.8	4.58	4.34	4.33	4.3	4.73	4.76	3.68	4.44	4.86	4.72	5.46	4.39	4.48	4.87	4.58	5.7
4.54	4.06	4.66	4.37	4.07	.	4.26	5.23	4.93	4.8	4.34	4.53	4.3	4.73	4.76	3.68	4.44	4.86	4.72	5.81	4.63	4.49	4.87	4.58	5.7
4.58	4.06	4.84	4.4	4.35	.	4.26	5.53	4.93	4.8	4.43	4.53	4.3	4.87	5.22	3.68	4.44	4.86	5.12	5.81	4.63	4.76	4.87	4.65	5.7
4.58	4.45	4.84	4.44	4.35	.	4.26	5.9	5.15	4.88	4.68	4.53	4.87	4.87	5.22	3.98	4.78	4.86	5.12	6.02	4.63	4.76	4.98	4.93	5.7
4.58	4.45	4.84	4.45	4.35	.	4.27	5.9	5.15	5.1	4.72	4.61	4.87	4.87	5.22	4.01	4.78	4.86	5.12	6.02	4.8	4.76	4.98	4.93	5.7
4.99	4.45	4.84	4.57	4.35	.	4.34	5.9	5.15	5.1	4.72	4.61	4.87	4.87	5.22	4.28	4.78	5.34	5.12	6.16	4.85	4.76	5.14	4.94	5.7
4.99	4.45	4.84	4.57	4.68	.	4.34	5.9	5.17	5.1	4.87	5.02	4.87	5.08	5.22	4.28	5.15	5.36	5.12	6.16	4.85	4.78	5.15	5.13	5.7
4.99	4.45	4.84	4.57	4.68	.	4.43	5.94	5.17	5.1	4.87	5.03	4.87	5.08	5.23	4.28	5.15	5.36	5.17	6.16	5.13	4.89	5.17	5.13	5.7
5.5	4.45	4.84	4.72	4.68	.	4.43	5.94	5.17	5.3	4.87	5.03	5	5.08	5.61	4.28	5.15	5.51	5.17	6.16	5.13	5.03	5.18	5.37	5.71
5.5	4.45	4.84	4.73	4.68	.	4.43	6.14	5.17	5.3	4.89	5.03	5	5.08	5.61	4.28	5.43	5.51	5.17	6.16	5.26	5.03	5.3	5.38	6.18
5.52	4.81	5.21	4.96	4.68	.	4.43	6.26	5.2	5.3	4.89	5.4	5	5.1	5.61	4.68	5.43	5.51	5.48	6.16	5.26	5.07	5.43	5.48	6.18
5.52	4.81	5.21	4.96	5.13	.	4.57	6.26	5.54	5.32	5.13	5.4	5	5.38	5.92	4.86	5.43	5.51	5.59	6.24	5.62	5.08	5.77	5.7	6.59
5.52	4.81	5.21	5.19	5.13	.	4.57	6.26	5.55	5.74	5.17	5.4	5.06	5.46	5.92	4.86	5.43	5.64	5.6	6.53	5.62	5.09	5.77	5.7	6.63
5.52	4.81	5.21	5.19	5.26	.	4.57	6.26	5.55	5.74	5.17	5.4	5.06	5.55	6.11	4.86	5.61	6.04	5.6	6.53	5.78	5.4	6	5.7	6.63
5.52	4.81	5.57	5.19	5.26	.	4.62	6.59	5.55	6.03	5.17	5.49	5.46	5.55	6.42	4.86	5.76	6.04	5.78	6.69	5.79	5.49	6	5.7	6.85
5.91	4.81	5.57	5.19	5.26	.	4.62	6.82	5.94	6.04	5.49	5.55	5.46	5.64	6.42	4.86	5.76	6.04	5.8	6.69	5.79	5.62	6	5.71	7.1
5.91	5.42	5.57	5.21	5.26	.	4.62	6.83	5.94	6.04	5.5	5.55	5.46	5.64	6.79	4.86	5.76	6.49	6.07	6.97	5.99	5.63	6.31	5.71	7.17
5.91	5.42	5.57	5.42	5.26	.	4.64	6.96	5.94	6.04	5.55	5.82	5.46	5.64	6.79	5.27	5.84	6.49	6.07	6.97	5.99	5.83	6.31	5.71	7.17
5.91	5.42	6.11	5.42	5.26	.	4.64	7.1	5.94	6.04	5.56	6.04	5.46	5.7	6.79	5.27	5.93	6.49	6.08	7.32	6.14	5.83	6.31	6.14	7.17
6.49	5.42	6.11	5.42	5.26	.	4.74	7.12	5.94	6.04	5.72	6.04	5.46	5.71	7.11	5.27	5.97	6.49	6.2	7.32	6.31	5.83	6.31	6.17	7.17
6.49	.	6.11	5.84	5.31	.	4.76	7.26	5.94	6.04	5.77	6.04	5.46	5.71	7.11	5.27	5.97	6.9	6.25	7.32	6.31	5.83	6.31	6.17	7.17
6.49	.	6.11	5.84	5.31	.	4.76	7.36	6.24	6.2	5.77	6.04	5.46	5.78	7.11	5.27	5.97	6.9	6.27	7.32	6.31	6.06	6.48	6.17	7.17
6.49	.	6.11	5.84	5.31	.	4.76	7.36	6.24	6.2	5.77	6.04	5.86	5.94	7.11	5.27	5.97	6.9	6.62	7.32	6.31	6.06	6.71	6.17	7.17
6.5	.	6.61	5.84	5.31	.	4.88	7.51	6.24	6.2	5.77	6.08	5.86	5.94	7.11	5.74	6.09	6.9	6.62	7.32	6.39	6.06	6.71	6.17	7.17
6.5	.	6.61	5.84	5.56	.	4.98	7.64	6.52	6.24	6.04	6.08	5.87	5.94	7.11	5.74	6.09	6.9	6.62	7.32	6.6	6.29	6.71	6.25	7.51
6.5	.	6.61	6.15	5.56	.	4.98	7.64	6.52	6.26	6.07	6.08	5.87	5.94	7.51	5.74	6.09	7.09	6.62	7.49	6.61	6.29	6.71	6.32	7.51
6.5	.	6.61	6.18	5.56	.	4.98	7.64	6.52	6.28	6.11	6.08	5.87	6.06	7.51	5.74	6.09	7.32	6.75	7.57	6.69	6.29	6.71	6.32	7.51
6.5	.	6.61	6.18	5.56	.	4.98	7.64	6.52	6.28	6.11	6.08	5.87	6.06	7.51	5.74	6.09	7.32	6.75	7.63	6.69	6.29	6.71	6.32	7.51
6.5	.	6.61	6.18	5.69	.	4.98	7.64	6.7	6.28	6.11	6.59	5.87	6.06	7.51	5.74	6.09	7.32	6.75	7.94	6.73	6.29	6.71	6.32	7.62
6.5	.	6.61	6.18	5.69	.	5.01	7.76	6.73	6.28	6.11	6.59	5.87	6.06	7.51	5.74	6.09	7.32	6.75	8.29	6.73	6.29	6.71	6.32	7.62
6.5	.	6.61	6.18	5.69	.	5.01	7.76	6.74	6.28	6.11	6.59	5.87	6.06	7.51	5.74	6.09	7.32	6.75	8.29	6.74	6.29	6.74	6.32	7.95
6.72	.	6.61	6.18	5.69	.	5.07	7.76	6.74	6.28	6.11	6.59	5.87	6.27	7.84	5.74	6.09	7.32	6.75	8.29	6.78	6.7	7.07	6.36	7.95
6.72	.	6.61	6.71	5.69	.	5.07	7.8	6.74	6.28	6.11	6.59	5.87	6.27	7.84	5.74	6.09	7.32	6.82	8.29	6.8	6.7	7.07	6.36	7.95
6.72	.	6.61	6.71	5.69	.	5.07	7.8	6.93	6.28	6.11	6.59	5.87	6.27	7.84	5.74	6.09	7.32	6.82	8.7	6.8	7.08	7.07	6.4	7.95
6.72	.	6.85	6.71	5.69	.	5.07	7.8	6.93	6.28	6.11	6.59	5.98	6.27	7.84	5.74	6.09	7.32	6.82	8.7	7.06	7.08	7.07	6.62	7.95
6.72	.	6.85	6.71	5.69	.	5.07	7.8	6.93	6.55	6.11	6.97	6.02	6.35	7.84	5.74	6.09	7.32	6.82	8.7	7.06	7.08	7.36	6.62	7.95
6.72	.	6.85	6.71	5.69	.	5.09	7.8	6.93	6.55	6.11	6.97	6.24	6.35	7.84	5.74	6.09	7.32	6.82	8.84	7.07	7.08	7.36	6.63	7.96
6.72	.	6.85	6.71	5.69	.	5.23	7.8	6.93	6.55	6.11	6.97	6.24	6.38	7.84	5.83	6.09	7.32	6.98	8.84	7.11	7.08	7.36	6.82	8.13
6.72	.	6.85	6.81	5.69	.	5.24	7.8	6.93	6.71	6.19	6.97	6.24	6.49	7.84	6.29	6.09	7.32	6.98	9.04	7.11	7.08	7.36	6.82	8.28
7.22	.	6.85	6.82	5.82	.	5.24	7.8	7.06	6.71	6.24	6.98	6.27	6.49	7.84	6.29	6.09	7.32	6.98	9.04	7.12	7.08	7.36	6.82	8.28
7.24	.	6.85	6.82	5.82	.	5.24	7.8	7.06	6.71	6.24	7.06	6.27	6.75	7.84	6.29	6.15	7.32	7.13	9.04	7.12	7.66	7.36	6.82	8.45
7.24	.	7.33	6.83	6.03	.	5.24	7.8	7.06	6.71	6.24	7.06	6.27	6.75	8.27	6.32	6.15	7.61	7.13	9.04	7.12	7.66	7.36	7.07	8.45
7.24	.	7.35	6.83	6.03	.	5.32	7.9	7.06	6.99	6.24	7.06	6.27	6.75	8.27	6.32	6.15	7.61	7.13	9.04	7.39	7.66	7.36	7.07	8.45
7.24	.	7.35	6.83	6.03	.	5.32	7.9	7.06	6.99	6.35	7.06	6.27	6.75	8.27	6.32	6.15	7.61	7.13	9.04	7.39	7.66	7.43	7.07	8.46
7.24	.	7.35	6.83	6.03	.	5.32	7.9	7.06	6.99	6.38	7.2	6.38	6.75	8.27	6.32	6.25	7.61	7.13	9.27	7.39	7.66	7.69	7.21	8.46
7.24	.	7.35	6.83	6.03	.	5.41	7.9	7.06	7.38	6.47	7.2	6.42	6.75	8.27	6.33	6.32	7.87	7.13	9.27	7.49	7.67	7.74	7.22	8.46
7.24	.	7.35	6.83	6.28	.	5.41	8.07	7.06	7.38	6.47	7.2	6.42	6.75	8.46	6.33	6.32	7.88	7.2	9.27	7.49	7.68	7.75	7.31	8.52
7.24	.	7.35	6.83	6.28	.	5.57	8.07	7.19	7.38	6.47	7.2	6.42	7.12	8.46	6.33	6.32	7.88	7.2	9.49	7.56	7.76	7.86	7.31	8.52
7.62	.	7.35	6.83	6.28	.	5.57	8.1	7.19	7.38	6.67	7.21	6.42	7.12	8.46	6.33	6.33	7.88	7.2	9.49	7.56	7.76	7.92	7.31	8.52
7.62	.	7.35	6.83	6.4	.	5.77	8.16	7.23	7.54	6.69	7.21	6.42	7.12	8.46	6.46	6.33	7.95	7.2	9.5	7.74	7.76	7.92	7.53	8.52
7.62	.	7.42	6.88	6.59	.	5.77	8.16	7.23	7.54	6.72	7.21	6.42	7.12	8.67	6.62	6.33	7.95	7.57	9.5	7.74	7.96	7.93	7.53	8.52
7.77	.	7.42	6.88	6.59	.	6.05	8.23	7.51	7.54	7.08	7.21	6.42	7.12	8.67	6.62	6.5	7.95	7.57	9.65	7.74	7.96	8.23	7.62	8.52
7.77	.	7.42	6.88	6.59	.	6.05	8.23	7.51	7.54	7.11	7.33	6.62	7.12	8.67	6.62	6.5	8.13	7.57	9.65	7.79	7.96	8.23	7.66	8.52
7.77	.	7.42	6.88	6.59	.	6.05	8.23	7.51	7.56	7.15	7.34	6.62	7.41	8.67	6.62	6.5	8.13	7.57	9.65	7.8	8.33	8.3	7.74	8.52
7.77	.	7.43	6.88	6.59	.	6.05	8.23	7.68	7.56	7.15	7.34	6.62	7.41	8.67	6.62	6.5	8.13	7.57	9.65	7.8	8.33	8.3	7.74	8.52
8.01	.	7.6	6.88	6.59	.	6.05	8.57	7.68	7.56	7.15	7.43	6.62	7.41	8.67	6.62	6.5	8.13	7.57	9.65	7.8	8.34	8.3	7.74	8.52
8.01	.	7.6	6.88	6.59	.	6.06	8.58	7.84	7.56	7.15	7.57	6.62	7.41	8.99	6.62	6.5	8.13	7.57	9.65	8.04	8.34	8.3	7.9	8.52
8.01	.	7.68	6.88	6.84	.	6.06	8.58	7.84	7.56	7.15	7.57	6.74	7.8	9	6.62	6.75	8.13	7.76	9.65	8.04	8.34	8.3	7.9	8.52
8.01	.	7.68	6.88	6.85	.	6.06	8.58	7.84	7.56	7.15	7.57	6.74	7.8	9	6.63	6.75	8.13	7.76	9.65	8.04	8.35	8.33	7.9	8.52
8.01	.	7.68	.	6.85	.	6.06	8.58	7.84	7.56	7.15	.	6.74	7.8	9	6.63	6.75	8.13	7.76	9.65	8.23	8.35	8.37	7.9	8.52
8.01	.	8.07	.	6.85	.	6.07	8.84	7.84	7.56	7.49	.	6.74	8.15	9	7.05	6.75	8.84	7.76	10.08	8.23	8.35	8.37	8	8.52
8.35	.	8.07	.	6.85	.	6.07	8.84	8.03	7.64	7.49	.	6.74	8.15	9.17	7.06	6.75	8.84	7.76	10.09	8.23	8.35	8.51	8	8.65
8.62	.	8.07	.	6.85	.	6.61	8.84	8.03	7.64	7.51	.	6.74	8.15	9.17	7.06	6.75	8.84	7.76	10.09	8.29	8.35	8.52	8.29	8.65
8.62	.	8.07	.	6.97	.	6.61	8.84	8.04	7.64	7.52	.	7.1	8.15	9.17	7.06	6.76	8.84	7.76	10.09	8.29	8.77	8.55	8.29	9.14
8.62	.	8.49	.	6.97	.	6.61	8.84	8.04	7.82	7.52	.	7.1	8.56	9.71	7.06	7.03	8.84	8.39	10.09	8.29	8.77	8.77	8.29	9.14
8.64	.	8.49	.	6.97	.	6.61	8.84	8.06	7.83	7.56	.	7.1	8.56	9.71	7.06	7.04	8.84	8.39	10.09	8.56	8.77	8.86	8.29	9.58
8.64	.	8.49	.	6.97	.	6.61	9.25	8.43	7.83	7.56	.	7.1	8.56	9.71	7.06	7.05	8.84	8.39	10.09	8.66	8.77	8.87	8.67	9.58
8.64	.	8.49	.	7.75	.	6.61	9.25	8.43	7.83	7.56	.	7.1	8.56	9.71	7.57	7.05	9.53	8.39	10.09	8.66	8.77	9.07	8.67	9.58
9.1	.	8.49	.	7.75	.	6.61	9.25	8.43	8.09	7.56	.	7.72	8.56	9.71	7.58	7.05	9.53	8.39	10.36	8.66	8.77	9.07	8.67	9.58
9.1	.	8.49	.	7.75	.	6.61	9.76	8.45	8.09	7.6	.	7.72	8.56	9.71	7.58	7.05	9.53	8.88	10.36	8.66	8.77	9.07	8.67	9.58
9.1	.	8.49	.	7.75	.	6.61	9.76	8.45	8.09	8.15	.	7.72	8.86	9.72	7.58	7.65	9.53	8.88	10.36	8.66	8.77	9.47	8.71	9.58
9.1	.	8.71	.	7.75	.	6.61	9.76	8.74	8.19	8.15	.	7.72	8.87	9.72	7.58	7.65	9.53	8.88	10.36	8.84	8.81	9.47	8.71	10.1
9.1	.	8.71	.	7.76	.	6.67	9.76	8.74	8.38	8.17	.	7.72	8.87	10.31	7.59	7.65	9.53	8.88	10.83	8.84	8.81	9.47	8.87	10.1
9.1	.	8.72	.	7.76	.	6.67	10.07	8.74	8.53	8.17	.	8.07	8.88	10.31	7.59	7.69	9.53	8.88	10.83	9.18	8.93	9.47	8.92	10.1
9.1	.	8.72	.	7.76	.	6.67	10.25	8.74	8.53	8.19	.	8.08	8.88	10.31	7.62	8.02	10.02	8.88	11.21	9.24	8.93	9.65	8.92	10.58
9.1	.	9.1	.	7.76	.	6.97	10.3	8.74	8.54	8.19	.	8.08	8.88	10.31	8.16	8.02	10.1	8.88	11.21	9.24	8.93	9.66	9.31	10.59
9.1	.	9.1	.	7.76	.	6.97	10.35	8.74	8.54	8.19	.	8.08	8.88	10.31	8.16	8.02	10.1	9.26	11.21	9.24	8.95	10.18	9.31	10.59
9.1	.	9.1	.	7.76	.	7.18	10.36	8.74	8.82	8.19	.	8.08	8.88	10.31	8.16	8.02	10.1	9.26	11.21	9.65	9.25	10.18	9.31	10.59
9.26	.	9.54	.	7.96	.	7.18	10.6	9.22	8.82	8.19	.	8.08	8.88	10.31	8.16	8.63	10.1	9.26	11.65	9.65	9.31	10.19	9.63	10.59
9.29	.	9.54	.	8.27	.	7.18	10.62	9.22	9.11	8.19	.	8.08	8.88	10.31	8.16	8.63	10.1	9.26	11.66	9.65	9.31	10.19	9.63	10.59
9.29	.	9.54	.	8.27	.	7.18	10.63	9.22	9.11	8.23	.	8.08	8.88	10.38	8.34	8.63	10.1	9.26	11.66	9.66	9.31	10.27	9.63	10.59
9.38	.	9.54	.	8.27	.	7.18	10.98	9.63	9.11	8.24	.	8.08	9.24	10.45	8.98	8.63	10.1	9.26	11.99	9.94	9.31	10.3	9.63	11.01
9.51	.	9.57	.	8.27	.	7.18	10.98	9.63	9.11	8.27	.	8.08	9.24	10.59	8.98	8.63	10.7	9.26	12.22	10.01	9.31	10.33	9.63	11.01
9.51	.	9.57	.	8.27	.	7.18	11.48	9.63	9.11	8.27	.	8.59	9.24	10.59	8.98	8.63	10.7	9.26	12.31	10.01	9.53	10.35	9.63	11.01
9.51	.	9.57	.	8.27	.	7.28	11.5	9.68	9.11	8.75	.	8.59	9.24	10.59	8.98	9.19	10.7	9.26	12.31	10.15	9.53	10.37	9.63	11.01
9.51	.	9.57	.	8.27	.	7.28	11.5	10.07	9.11	8.76	.	8.59	9.24	10.59	8.98	9.2	10.7	9.42	12.31	10.15	9.54	10.37	9.84	11.14
9.51	.	9.57	.	8.27	.	7.32	11.83	10.07	9.25	8.81	.	8.59	9.24	10.9	8.98	9.2	10.7	9.83	12.31	10.15	9.54	10.37	9.84	11.14
9.52	.	9.86	.	8.27	.	7.32	11.83	10.07	9.29	8.81	.	8.59	9.44	10.9	8.98	9.2	10.7	9.89	12.31	10.16	9.54	10.43	9.84	11.14
9.79	.	9.96	.	8.27	.	7.33	11.83	10.07	9.29	8.81	.	8.59	9.44	10.9	9.2	9.2	10.7	9.89	12.31	10.29	9.54	10.43	9.84	11.14
9.79	.	9.96	.	8.27	.	7.61	11.83	10.07	9.6	8.81	.	8.59	9.44	10.9	9.2	9.2	10.7	9.89	12.31	10.29	9.57	10.43	9.84	11.14
9.8	.	9.96	.	8.27	.	7.61	11.83	10.07	9.68	8.81	.	8.59	9.69	10.9	9.2	9.2	10.7	9.9	12.31	10.38	9.57	10.45	9.84	11.14
9.8	.	9.96	.	8.27	.	7.61	11.83	10.07	9.68	8.81	.	8.59	9.69	10.9	9.2	9.2	10.7	9.9	12.73	10.39	9.57	10.45	9.98	11.55
9.8	.	9.96	.	8.27	.	7.72	11.83	10.07	9.68	8.81	.	8.77	9.69	10.9	9.2	9.2	10.7	10.14	12.73	10.39	9.86	10.45	9.98	11.55
9.8	.	9.96	.	8.27	.	7.86	11.83	10.07	9.68	9.26	.	8.77	9.69	10.9	9.21	9.2	10.7	10.14	12.73	10.39	9.86	10.61	10.08	11.55
9.8	.	9.96	.	8.27	.	7.86	11.88	10.07	9.68	9.29	.	8.77	9.72	10.9	9.21	9.2	10.7	10.14	12.73	10.39	10	10.61	10.12	11.72
9.83	.	9.96	.	8.27	.	7.86	12.26	10.07	9.68	9.29	.	8.77	9.72	10.9	9.25	9.2	11.17	10.14	12.73	10.46	10	10.61	10.12	11.72
9.83	.	9.96	.	8.27	.	7.86	12.26	10.07	9.68	9.29	.	8.77	9.72	10.9	9.38	9.2	11.17	10.14	12.73	10.47	10	10.68	10.13	11.72
9.83	.	9.96	.	8.5	.	7.87	12.26	10.07	9.68	9.29	.	8.77	9.72	10.9	9.38	9.2	11.18	10.14	12.73	10.47	10	10.68	10.23	11.72
9.83	.	9.96	.	8.51	.	7.87	12.26	10.07	9.68	9.29	.	8.77	9.72	10.9	9.38	9.2	11.18	10.14	12.73	10.47	10	10.68	10.23	11.72
9.83	.	9.96	.	8.51	.	7.87	12.54	10.07	9.68	9.29	.	8.87	9.72	10.9	9.38	9.2	11.18	10.14	12.73	10.55	10	10.68	10.46	11.72
9.83	.	9.96	.	8.51	.	8.15	12.55	10.07	9.68	9.29	.	8.87	9.72	10.9	9.38	9.2	11.18	10.14	12.99	10.55	10.11	10.68	10.47	11.72
9.83	.	9.96	.	8.51	.	8.15	12.55	10.07	9.68	9.29	.	8.87	9.72	10.9	9.38	9.2	11.18	10.14	12.99	10.59	10.11	10.68	10.47	11.72
10.05	.	9.96	.	8.51	.	8.15	12.55	10.07	9.69	9.29	.	8.87	9.72	10.9	9.38	9.2	11.18	10.14	12.99	10.59	10.33	10.8	10.47	12.04
10.06	.	9.96	.	8.62	.	8.16	12.55	10.07	9.69	9.32	.	8.87	9.72	10.9	9.4	9.2	11.18	10.14	12.99	10.73	10.33	10.81	10.5	12.04

10.09	.	10.13	.	8.62	.	8.76	12.55	10.07	9.69	9.52	.	8.87	9.87	10.9	9.45	9.2	11.33	10.14	13	10.73	10.43	10.81	10.63	12.07
10.09	.	10.13	.	8.62	.	8.77	12.55	10.07	9.69	9.52	.	8.87	9.87	10.9	9.57	9.2	11.84	10.14	13.26	10.73	10.44	10.81	10.64	12.07
10.09	.	10.13	.	8.62	.	8.77	12.88	10.11	9.69	9.59	.	8.87	9.87	11.13	9.77	9.5	11.84	10.14	13.26	10.73	10.44	10.82	10.64	12.07
10.09	.	10.13	.	8.75	.	8.77	12.88	10.28	10.04	9.65	.	8.87	9.87	11.13	9.77	9.5	11.84	10.17	13.26	10.73	10.72	10.82	10.75	12.07
10.09	.	10.13	.	9.06	.	8.77	12.88	10.34	10.05	9.66	.	8.99	9.87	11.13	9.77	9.51	11.84	10.17	13.26	10.73	10.72	11.05	10.75	12.07
10.09	.	10.13	.	9.06	.	8.77	12.88	10.34	10.05	9.66	.	9	9.95	11.13	9.77	9.51	11.84	10.17	13.26	10.98	10.72	11.05	10.98	12.25
10.09	.	10.13	.	9.06	.	8.77	12.88	10.34	10.2	9.72	.	9.33	9.95	11.13	9.78	9.51	11.84	10.17	13.63	10.98	10.72	11.05	10.99	12.25
10.09	.	10.13	.	9.06	.	8.78	12.98	10.62	10.2	9.73	.	9.33	9.95	11.13	9.79	9.51	11.85	10.17	13.63	10.98	10.72	11.05	11	12.25
10.09	.	10.13	.	9.08	.	8.78	12.98	10.62	10.2	9.74	.	9.33	9.95	11.13	9.79	9.51	11.85	10.28	13.63	10.98	10.72	11.05	11	12.54
10.3	.	10.51	.	9.08	.	8.78	12.98	10.62	10.2	9.74	.	9.33	10.07	11.13	9.79	9.51	11.85	10.28	13.63	11.15	10.72	11.18	11	12.54
10.3	.	10.53	.	9.08	.	8.9	12.98	10.74	10.2	9.74	.	9.33	10.07	11.13	9.94	9.51	11.85	10.28	13.63	11.15	10.72	11.19	11.15	12.54
10.3	.	10.53	.	9.08	.	8.9	12.98	10.99	10.2	9.75	.	9.34	10.07	11.13	9.94	9.51	11.98	10.28	13.63	11.15	10.72	11.46	11.15	12.54
10.3	.	10.53	.	9.08	.	8.9	13.35	10.99	10.71	9.75	.	9.34	10.07	11.45	9.94	9.51	11.98	10.53	13.63	11.15	10.9	11.46	11.15	12.54
10.3	.	10.53	.	9.5	.	8.9	13.35	11	10.72	10.02	.	9.34	10.07	11.45	9.94	9.51	11.98	10.68	14.09	11.15	10.9	11.46	11.15	12.54
10.41	.	10.53	.	9.5	.	8.9	**13.35**	**11.04**	10.72	10.09	.	9.34	10.25	11.45	**9.94**	**9.51**	11.98	10.68	14.09	11.24	10.9	11.5	11.18	12.64

0	0	0	0	0	.	0	0.02	0.04	0	0	0	0	0	0	0.54	0.14	0	0	0	0	0	0	0	0
0.02	0	0	0.13	0.16	.	0	0.15	0.08	0.3	0	0.14	0.25	0.12	0.18	0.54	0.31	0	0	0.03	0	0.11	0.09	0.03	0
0.02	0	0	0.22	0.31	.	0.06	0.17	0.16	0.32	0	0.14	0.34	0.15	0.18	0.55	0.31	0.01	0.12	0.03	0.12	0.12	0.11	0.08	0
0.02	0.08	0.23	0.22	0.39	.	0.06	0.26	0.17	0.32	0	0.28	0.69	0.15	0.18	0.58	0.35	0.01	0.27	0.15	0.21	0.13	0.11	0.1	0.26
0.27	0.17	0.23	0.58	0.39	.	0.18	0.27	0.19	0.32	0.31	0.28	0.69	0.15	0.44	0.59	0.42	0.02	0.33	0.16	0.38	0.49	0.24	0.14	0.3
0.32	0.17	0.24	0.58	0.59	.	0.25	0.27	0.2	0.32	0.31	0.42	0.69	0.44	0.55	0.59	0.42	0.14	0.33	0.38	0.41	0.52	0.24	0.23	0.3
0.32	0.17	0.47	0.58	0.59	.	0.25	0.57	0.66	0.32	0.31	0.42	0.69	0.44	0.58	0.59	0.42	0.15	0.33	0.38	0.41	0.52	0.57	0.24	0.3
0.32	0.27	0.47	0.79	0.59	.	0.25	0.57	0.69	0.5	0.31	0.54	1.02	0.6	0.8	0.59	0.73	0.16	0.33	0.38	0.41	0.64	0.57	0.24	0.42
0.32	0.28	0.48	0.79	0.67	.	0.46	0.57	0.69	0.5	0.7	0.64	1.02	0.6	0.83	0.59	0.75	0.16	0.66	0.38	0.51	0.64	0.65	0.28	0.42
0.33	0.28	0.7	0.79	0.67	.	0.72	0.62	0.7	0.72	0.7	0.85	1.13	0.84	0.83	0.71	0.75	0.17	0.66	0.66	0.52	0.81	0.75	0.34	0.42
0.48	0.44	0.7	1.01	0.67	.	0.72	0.9	0.71	0.72	0.7	0.85	1.13	0.84	0.86	0.71	0.93	0.17	0.79	0.69	0.67	0.81	0.75	0.34	0.58
0.48	0.44	0.71	1.01	0.88	.	0.73	1.03	0.72	0.81	0.7	0.85	1.27	0.92	0.89	0.74	0.94	0.32	0.89	0.69	0.67	0.95	0.9	0.34	0.67
0.64	0.44	0.73	1.01	1.06	.	0.73	1.03	0.85	0.93	0.72	1.12	1.27	0.92	1	0.74	1.11	0.32	0.89	0.69	0.69	0.95	0.92	0.49	0.67
0.66	0.55	0.77	1.01	1.06	.	0.95	1.03	0.85	0.93	0.74	1.12	1.52	0.92	1	1.03	1.11	0.33	0.89	0.73	0.81	0.96	0.92	0.52	0.67
0.66	0.73	1.04	1.01	1.06	.	0.95	1.1	0.86	0.93	0.74	1.12	1.67	1.07	1	1.03	1.12	0.47	0.89	0.91	0.81	1.1	1.09	0.73	0.9
0.73	0.93	1.06	1.01	1.2	.	0.95	1.1	0.86	0.94	0.74	1.31	1.67	1.07	1.02	1.03	1.44	0.6	0.89	0.92	0.96	1.29	1.33	0.73	0.9
0.73	0.93	1.06	1.01	1.2	.	1.17	1.45	0.86	0.94	0.74	1.41	1.68	1.11	1.13	1.23	1.44	0.6	0.89	0.92	1.09	1.33	1.52	0.8	0.9
1	1.05	1.06	1.33	1.2	.	1.17	1.45	0.86	1.14	0.74	1.42	1.68	1.24	1.18	1.3	1.59	0.87	1.04	1.11	1.1	1.33	1.68	0.8	1.13
1	1.05	1.06	1.33	1.5	.	1.17	1.45	0.86	1.14	0.74	1.42	1.68	1.46	1.19	1.3	1.59	0.87	1.31	1.13	1.4	1.54	1.68	0.85	1.13
1.06	1.05	1.23	1.34	1.51	.	1.49	1.46	1.25	1.14	0.74	1.42	1.68	1.46	1.32	1.46	1.59	0.87	1.4	1.13	1.41	1.54	1.68	0.95	1.4
1.06	1.25	1.23	1.34	1.51	.	1.49	1.53	1.25	1.14	0.74	1.42	1.89	1.46	1.32	1.47	1.69	0.87	1.4	1.13	1.41	1.54	1.68	0.95	1.4
1.26	1.25	1.23	1.34	1.51	.	1.49	1.81	1.27	1.14	0.74	1.42	1.89	1.47	1.33	1.47	1.83	0.87	1.47	1.13	1.41	1.78	1.68	0.95	1.4
1.26	1.25	1.32	1.34	1.51	.	1.49	1.82	1.27	1.33	0.8	1.52	1.89	1.47	1.33	1.68	1.86	0.87	1.47	1.14	1.41	1.78	1.68	0.95	1.4
1.39	1.25	1.49	1.34	1.51	.	1.49	1.82	1.36	1.33	0.87	1.64	1.89	1.47	1.33	1.69	1.86	1.12	1.47	1.34	1.47	1.78	1.85	0.97	1.4
1.39	1.38	1.49	1.61	1.51	.	1.49	2.02	1.38	1.33	0.87	1.64	1.89	1.62	1.33	1.69	1.86	1.41	1.47	1.34	1.47	1.78	1.91	1.11	1.67
1.39	1.38	1.49	1.61	1.51	.	1.63	2.02	1.38	1.33	0.87	1.64	2.01	1.63	1.6	1.98	2.09	1.41	1.47	1.34	1.47	1.91	1.93	1.11	1.67
1.52	1.66	1.49	1.61	1.64	.	1.63	2.02	1.45	1.57	0.96	1.64	2.01	1.63	1.65	1.98	2.09	1.41	1.54	1.34	1.61	1.99	1.96	1.11	1.67
1.53	1.66	1.49	1.61	1.64	.	1.63	2.03	1.45	1.57	0.98	1.64	2.01	1.63	1.65	1.98	2.09	1.41	1.54	1.35	1.61	2.16	1.97	1.11	1.68
1.54	1.67	1.49	1.77	1.68	.	1.63	2.19	1.45	1.57	1	1.87	2.01	1.72	1.73	1.99	2.09	1.41	1.54	1.57	1.61	2.16	1.97	1.4	1.68
1.54	1.67	1.61	1.77	1.68	.	1.64	2.19	1.45	1.57	1.19	1.87	2.01	1.73	1.73	1.99	2.09	1.42	1.54	1.83	1.7	2.16	2.07	1.55	1.8
1.54	1.77	1.84	1.78	1.68	.	1.82	2.19	1.6	1.57	1.22	1.87	2.08	1.73	1.73	2	2.09	1.87	1.57	1.85	1.76	2.16	2.08	1.55	1.91
1.54	1.77	1.87	1.78	1.68	.	1.82	2.19	1.6	1.69	1.25	1.87	2.08	1.73	1.73	2.12	2.34	1.87	1.75	1.85	1.86	2.26	2.08	1.56	1.91
1.54	1.77	1.87	1.78	1.68	.	1.82	2.19	1.61	1.75	1.25	1.87	2.23	1.9	1.73	2.13	2.35	1.87	1.75	1.85	1.86	2.26	2.08	1.59	1.91
1.54	1.91	1.87	1.91	1.68	.	1.82	2.31	1.62	1.75	1.25	2.32	2.23	1.93	1.76	2.13	2.36	1.87	1.75	1.85	1.86	2.26	2.14	1.59	1.91
1.54	1.95	1.87	1.91	1.71	.	1.9	2.32	1.63	2	1.25	2.33	2.23	1.93	1.89	2.13	2.36	1.87	1.75	1.85	1.86	2.26	2.27	1.61	1.91
1.54	1.95	1.87	1.91	1.71	.	1.9	2.32	1.64	2	1.25	2.33	2.23	1.93	1.89	2.13	2.36	1.87	1.75	1.85	1.98	2.26	2.27	1.73	1.91
1.54	1.95	1.87	1.91	1.71	.	1.9	2.32	1.74	2	1.25	2.33	2.23	1.93	1.89	2.13	2.36	1.87	1.75	1.85	2.04	2.26	2.27	1.73	1.91
1.54	1.95	1.87	1.91	1.71	.	1.9	2.32	1.91	2	1.25	2.33	2.23	1.93	2.05	2.13	2.58	1.87	1.92	1.85	2.04	2.26	2.27	1.73	1.97
1.54	1.95	1.87	1.93	1.71	.	1.92	2.32	1.91	2	1.25	2.33	2.23	1.98	2.05	2.26	2.58	1.87	2.09	1.87	2.04	2.39	2.27	1.73	2.22
1.54	1.99	1.87	1.98	1.71	.	2.05	2.32	1.91	2	1.31	2.33	2.23	1.99	2.05	2.26	2.59	1.87	2.09	2.12	2.19	2.39	2.37	1.73	2.22
1.62	1.99	2	2.17	1.71	.	2.11	2.41	1.91	2	1.31	2.33	2.23	1.99	2.05	2.26	2.7	1.87	2.09	2.12	2.19	2.48	2.38	1.88	2.22
1.62	1.99	2	2.17	1.8	.	2.11	2.42	1.91	2	1.31	2.33	2.23	1.99	2.22	2.26	2.7	1.87	2.09	2.12	2.19	2.48	2.59	1.88	2.27
1.62	1.99	2	2.17	1.8	.	2.11	2.42	1.98	2	1.31	2.33	2.23	1.99	2.22	2.26	2.7	1.87	2.09	2.12	2.22	2.48	2.59	1.88	2.36
1.72	1.99	2	2.4	1.8	.	2.27	2.42	1.98	2	1.32	2.33	2.23	2.23	2.22	2.26	2.7	1.87	2.09	2.12	2.31	2.7	2.63	1.97	2.36
1.72	2.14	2	2.4	1.85	.	2.27	2.42	2.26	2	1.48	2.33	2.36	2.25	2.28	2.51	2.7	2.02	2.33	2.13	2.31	2.7	3	1.97	2.36
1.72	2.14	2	2.4	1.85	.	2.27	2.42	2.26	2.13	1.69	2.33	2.36	2.25	2.33	2.52	2.7	2.02	2.44	2.22	2.31	2.7	3.04	1.97	2.37
1.72	2.14	2.03	2.41	1.85	.	2.27	2.42	2.26	2.13	1.72	2.42	2.36	2.25	2.33	2.52	2.7	2.02	2.44	2.22	2.53	2.7	3.04	1.97	2.54
1.72	2.51	2.04	2.41	1.9	.	2.65	2.42	2.26	2.13	1.72	2.45	2.36	2.25	2.33	2.52	2.7	2.02	2.44	2.22	2.55	2.7	3.04	1.97	2.54
1.84	2.52	2.04	2.41	1.9	.	2.65	2.49	2.26	2.2	1.72	2.45	2.45	2.31	2.33	2.52	2.82	2.02	2.44	2.22	2.55	2.95	3.13	1.97	2.54
1.84	2.52	2.26	2.51	2.08	.	2.65	2.49	2.43	2.32	1.72	2.45	2.46	2.32	2.5	2.62	2.82	2.02	2.44	2.26	2.73	2.95	3.26	1.97	2.55
1.86	2.52	2.27	2.51	2.08	.	2.66	2.49	2.43	2.51	1.72	2.45	2.46	2.6	2.51	2.62	2.97	2.11	2.57	2.26	2.73	2.95	3.26	2.03	2.55
1.86	2.52	2.27	2.51	2.08	.	2.66	2.6	2.43	2.52	1.72	2.45	2.54	2.6	2.74	2.69	2.98	2.11	2.57	2.26	2.79	2.95	3.26	2.03	2.67
1.91	2.52	2.27	2.51	2.08	.	2.77	2.61	2.44	2.52	1.9	2.45	2.54	2.6	2.74	2.79	2.98	2.11	2.57	2.26	2.79	3.1	3.26	2.1	2.67
1.91	2.52	2.5	2.64	2.08	.	2.77	2.61	2.44	2.52	1.9	2.48	2.54	2.6	2.83	2.79	2.98	2.11	2.57	2.48	2.79	3.11	3.26	2.25	2.67
1.91	2.69	2.5	2.64	2.37	.	2.77	2.61	2.44	2.52	1.9	2.6	2.54	2.6	2.83	2.79	2.98	2.11	2.57	2.48	2.87	3.11	3.43	2.25	2.67
2.11	2.69	2.5	2.64	2.37	.	2.77	2.61	2.45	2.52	2.09	2.6	2.82	2.6	2.83	2.79	2.98	2.11	2.58	2.48	2.87	3.11	3.43	2.25	2.67
2.11	2.69	2.5	2.64	2.55	.	2.9	2.61	2.78	2.64	2.09	2.6	2.83	2.6	2.83	2.79	3.01	2.31	2.58	2.48	2.87	3.26	3.43	2.25	2.67
2.11	2.69	2.67	2.64	2.55	.	2.9	2.61	2.78	2.64	2.09	2.71	2.83	2.6	2.88	2.79	3.22	2.31	2.58	2.48	2.87	3.27	3.43	2.25	2.67
2.24	2.79	2.68	2.64	2.55	.	2.9	2.76	2.79	2.64	2.09	2.71	2.83	2.6	3	2.79	3.22	2.31	2.61	2.48	2.91	3.27	3.43	2.3	2.78
2.24	2.79	2.68	2.64	2.66	.	2.9	2.85	2.79	2.83	2.09	2.71	2.83	2.61	3	2.79	3.22	2.31	2.61	2.48	2.91	3.28	3.48	2.41	2.79
2.25	2.79	2.68	2.64	2.66	.	2.9	2.85	2.79	2.83	2.12	2.71	2.89	2.61	3.15	2.86	3.22	2.31	2.61	2.48	2.91	3.28	3.48	2.41	2.96
2.42	2.9	2.9	2.74	2.66	.	2.93	2.85	2.79	2.83	2.12	2.71	2.89	2.61	3.23	2.86	3.22	2.31	2.88	2.74	2.91	3.4	3.48	2.41	2.96
2.44	2.9	2.9	2.77	2.66	.	2.93	2.85	2.79	2.89	2.12	2.73	2.89	2.61	3.26	2.86	3.22	2.31	2.94	2.74	3.03	3.5	3.48	2.46	2.96
2.45	2.9	2.9	2.77	2.91	.	2.93	3.01	2.92	2.89	2.12	2.73	2.89	2.74	3.27	2.96	3.35	2.65	2.94	2.74	3.03	3.5	3.48	2.46	2.96
2.45	2.9	2.9	2.77	2.91	.	2.93	3.01	2.93	3.04	2.26	2.73	3.06	2.75	3.27	3.11	3.35	2.65	2.94	2.74	3.03	3.5	3.48	2.46	2.96
2.45	3.1	2.9	2.88	2.91	.	2.93	3.01	2.93	3.04	2.27	2.73	3.07	2.75	3.27	3.11	3.35	2.65	2.94	2.74	3.03	3.66	3.48	2.54	3.29
2.48	3.1	3.09	2.88	2.91	.	3.24	3.01	2.93	3.04	2.28	2.73	3.07	2.92	3.48	3.11	3.46	2.65	3.03	2.74	3.18	3.66	3.48	2.54	3.29
2.54	3.1	3.09	2.94	3.16	.	3.24	3.01	3.08	3.04	2.28	2.96	3.07	2.92	3.48	3.11	3.46	2.65	3.03	2.74	3.18	4	3.48	2.54	3.29
2.54	3.1	3.09	2.94	3.16	.	3.24	3.19	3.08	3.04	2.31	2.96	3.07	2.92	3.94	3.11	3.46	2.65	3.04	2.99	3.29	4	3.56	2.76	3.29
2.54	3.43	3.36	2.95	3.38	.	3.25	3.19	3.09	3.25	2.32	2.96	3.07	3.16	3.94	3.32	3.46	2.66	3.23	2.99	3.29	4	3.56	2.76	3.65
2.72	3.43	3.36	3.07	3.42	.	3.46	3.19	3.09	3.25	2.34	2.96	3.07	3.17	3.98	3.68	3.46	2.66	3.23	2.99	3.29	4	3.56	2.79	3.65
2.72	3.43	3.7	3.08	3.43	.	3.46	3.38	3.29	3.26	2.54	3.13	3.32	3.17	3.98	3.68	3.74	2.67	3.23	2.99	3.29	4.13	3.8	2.81	3.65
3.1	3.43	3.7	3.08	3.43	.	3.46	3.55	3.31	3.62	2.57	3.45	3.32	3.17	4.14	3.7	3.74	3.21	3.23	3.18	3.57	4.13	3.8	2.94	3.65
3.1	3.43	3.7	3.08	3.43	.	3.47	3.55	3.4	3.63	2.57	3.45	3.32	3.26	4.18	3.7	4.06	3.22	3.63	3.19	3.57	4.2	3.88	2.97	3.65
3.11	3.43	3.7	3.21	3.43	.	3.62	3.55	3.4	3.63	2.57	3.45	3.74	3.27	4.18	3.9	4.06	3.22	3.63	3.41	3.57	4.2	3.94	2.98	3.65
3.11	3.59	3.87	3.21	3.82	.	3.62	3.55	3.4	3.63	2.57	3.61	3.74	3.27	4.18	3.9	4.06	3.22	3.63	3.43	3.61	4.27	4.08	3.05	3.94
3.44	3.59	3.89	3.33	3.82	.	3.62	3.99	3.42	3.78	2.91	3.61	3.74	3.5	4.18	3.9	4.3	3.22	3.63	3.43	3.61	4.42	4.08	3.05	3.94
3.52	3.74	3.89	3.33	3.82	.	3.62	3.99	3.44	3.78	2.91	3.61	4.06	3.67	4.18	3.9	4.3	3.22	3.63	3.43	3.67	4.42	4.2	3.05	3.94
3.53	3.74	3.89	3.33	3.87	.	3.76	3.99	3.66	3.78	2.93	3.65	4.26	3.69	4.19	4.13	4.3	3.22	3.63	3.48	3.67	4.42	4.2	3.2	3.94
3.53	3.75	3.89	3.35	3.89	.	3.77	4.21	3.66	3.79	2.93	3.65	4.26	3.69	4.19	4.13	4.3	3.46	3.63	3.74	4.1	4.43	4.2	3.2	3.94
3.53	4.26	3.89	3.35	3.9	.	3.77	4.21	3.9	3.79	2.97	3.89	4.26	3.69	4.19	4.13	4.3	3.72	3.63	3.75	4.1	4.43	4.34	3.21	4.22
3.53	4.27	3.89	3.35	4.1	.	3.77	4.22	3.9	4.04	3	3.89	4.27	3.69	4.26	4.2	4.3	3.72	3.97	3.76	4.19	4.69	4.48	3.43	4.22
3.53	4.27	4.16	3.47	4.1	.	3.98	4.23	3.9	4.04	3	3.89	4.27	3.69	4.26	4.2	4.3	3.72	3.97	3.85	4.19	4.69	4.48	3.43	4.22
3.53	4.27	4.16	3.47	4.1	.	3.98	4.63	3.9	4.04	3	3.96	4.27	3.69	4.44	4.2	4.36	3.83	3.97	3.85	4.19	4.75	4.61	3.43	4.37
3.53	4.27	4.16	3.63	4.1	.	4.04	4.64	3.9	4.04	3	3.98	4.56	3.69	4.48	4.36	4.71	3.98	4.08	3.85	4.4	4.75	4.61	3.52	4.37
3.79	4.27	4.16	3.63	4.24	.	4.05	4.64	4.08	4.04	3	3.98	4.56	3.69	4.48	4.36	4.71	3.98	4.08	3.85	4.41	4.75	4.77	3.52	4.37
3.79	4.42	4.26	3.63	4.35	.	4.07	4.64	4.08	4.04	3.12	3.98	4.56	3.82	4.53	4.36	4.71	3.98	4.18	4.04	4.41	4.75	4.97	3.58	4.37
3.79	4.6	4.44	3.63	4.36	.	4.07	4.83	4.08	4.25	3.14	4.25	4.56	3.83	4.66	4.44	4.71	3.98	4.18	4.04	4.51	4.92	4.97	3.62	4.59
3.79	4.6	4.44	3.63	4.36	.	4.07	4.83	4.09	4.37	3.16	4.25	4.56	3.83	4.66	4.44	4.71	3.98	4.18	4.04	4.59	5.01	4.97	3.78	4.59
4	4.61	4.44	3.63	4.36	.	4.21	4.99	4.09	4.37	3.16	4.25	4.68	4.02	4.66	4.44	4.72	3.98	4.19	4.04	4.65	5.01	4.97	3.78	4.6
4	4.61	4.44	3.63	4.67	.	4.21	4.99	4.27	4.4	3.35	4.25	4.69	4.02	4.66	4.44	4.91	4.2	4.19	4.27	4.7	5.01	4.97	3.78	4.6
4	4.61	4.44	3.63	4.67	.	4.21	5.25	4.4	4.41	3.39	4.48	4.87	4.14	4.98	4.44	4.91	4.2	4.28	4.27	4.73	5.01	5.04	3.78	4.6
4	4.61	4.44	3.63	4.9	.	4.31	5.25	4.4	4.54	3.39	4.62	4.88	4.28	5.02	4.65	4.91	4.5	4.45	4.27	4.73	5.1	5.12	3.85	4.86
4.01	4.74	4.61	3.63	4.91	.	4.44	5.25	4.4	4.55	3.39	4.62	4.88	4.38	5.02	4.65	5.04	4.5	4.65	4.27	4.86	5.1	5.12	3.85	4.86
4.01	4.74	4.61	3.63	4.91	.	4.44	5.25	4.4	4.55	3.41	4.63	4.88	4.38	5.02	4.78	5.04	4.5	4.74	4.42	4.86	5.15	5.24	3.97	4.86
4.01	4.74	4.61	3.63	4.91	.	4.46	5.46	4.4	4.55	3.41	4.63	4.88	4.38	5.02	4.78	5.29	4.5	4.74	4.5	4.86	5.21	5.26	3.97	4.86
4.09	4.92	4.61	3.77	4.91	.	4.46	5.47	4.4	4.79	3.41	4.63	4.88	4.38	5.02	4.78	5.4	4.5	4.74	4.5	4.91	5.21	5.3	3.98	4.86
4.09	4.92	4.61	3.77	4.91	.	4.8	5.47	4.56	4.82	3.41	4.63	4.88	4.54	5.02	4.78	5.4	4.5	4.74	4.5	4.91	5.21	5.35	4.04	4.86
4.09	4.92	4.93	3.77	4.91	.	4.8	5.48	4.57	4.82	3.41	4.63	4.88	4.54	5.41	4.78	5.41	4.5	4.74	4.5	4.92	5.26	5.43	4.24	5.04
4.09	4.99	4.95	3.77	5.04	.	4.8	5.48	4.57	4.82	3.41	5.04	5.11	4.54	5.41	4.92	5.41	4.68	4.74	4.53	4.99	5.27	5.43	4.24	5.04
4.26	5	4.95	3.77	5.05	.	4.8	5.48	4.81	4.82	3.42	5.04	5.11	4.54	5.41	4.92	5.59	4.68	4.74	4.53	4.99	5.27	5.43	4.24	5.05
4.26	5	4.95	3.77	5.05	.	4.96	5.48	4.83	4.82	3.73	5.04	5.11	4.66	5.41	4.92	5.59	4.68	4.77	4.53	4.99	5.29	5.43	4.28	5.07
4.38	5	4.97	3.77	5.21	.	4.96	5.48	4.83	4.82	3.73	5.04	5.11	4.67	5.41	4.93	5.72	4.68	4.93	4.53	4.99	5.33	5.44	4.39	5.07
4.39	5	4.97	3.77	5.21	.	4.96	5.48	4.93	4.82	3.73	5.04	5.11	4.67	5.41	4.93	5.72	4.7	4.93	4.76	4.99	5.46	5.63	4.39	5.07
4.39	5	5.14	3.77	5.21	.	5.11	5.48	4.99	4.82	3.73	5.04	5.11	4.7	5.41	4.93	5.72	4.7	4.93	4.97	5.06	5.46	5.63	4.44	5.07

4.39	5.1	5.25	3.77	5.22	.	5.11	5.59	4.99	4.82	3.73	5.04	5.32	4.7	5.67	4.93	5.78	4.79	5.08	4.97	5.1	5.46	5.64	4.44	5.27
4.39	5.1	5.25	3.94	5.22	.	5.11	5.59	5	4.96	3.73	5.09	5.32	4.71	5.67	5.03	5.79	4.79	5.1	4.97	5.1	5.64	5.71	4.53	5.28
4.4	5.11	5.25	3.94	5.22	.	5.11	5.59	5.08	4.96	3.85	5.09	5.33	4.71	5.67	5.03	5.79	4.79	5.1	4.97	5.22	5.72	5.76	4.65	5.28
4.4	5.13	5.25	3.94	5.22	.	5.13	5.59	5.08	4.96	3.85	5.09	5.33	4.71	5.68	5.03	5.79	4.79	5.1	4.97	5.24	5.72	5.87	4.65	5.36
4.52	5.18	5.25	3.94	5.22	.	5.25	5.73	5.09	4.96	3.88	5.09	5.33	4.75	5.72	5.03	5.79	4.79	5.17	4.97	5.24	5.82	5.88	4.8	5.37
4.59	5.29	5.41	3.94	5.22	.	5.25	5.73	5.14	5.13	3.9	5.09	5.34	4.75	5.72	5.2	5.84	4.79	5.17	5.11	5.41	5.99	5.89	4.81	5.37
4.59	5.31	5.46	3.95	5.22	.	5.25	5.85	5.14	5.15	3.9	5.09	5.34	4.83	5.72	5.22	5.84	4.8	5.23	5.11	5.41	5.99	5.89	4.81	5.37
4.59	5.31	5.53	4.1	5.22	.	5.25	5.91	5.25	5.16	3.9	5.09	5.34	4.9	5.72	5.22	5.84	4.96	5.25	5.11	5.41	6.3	6.11	4.81	5.61
4.83	5.31	5.53	4.1	5.38	.	5.34	5.91	5.25	5.23	3.9	5.09	5.34	4.92	5.72	5.22	5.92	5.17	5.25	5.12	5.63	6.31	6.11	4.92	5.61
4.83	5.4	5.53	4.1	5.38	.	5.34	5.91	5.25	5.23	3.9	5.36	5.34	4.92	5.72	5.22	6	5.17	5.44	5.12	5.63	6.31	6.11	4.92	5.96
4.83	5.4	5.53	4.1	5.49	.	5.35	5.92	5.46	5.47	4	5.36	5.46	4.92	6.05	5.37	6	5.17	5.44	5.29	5.63	6.31	6.17	4.92	5.96
4.83	5.49	5.57	4.1	5.5	.	5.35	5.92	5.46	5.47	4.01	5.36	5.46	4.92	6.05	5.37	6	5.17	5.46	5.29	5.71	6.42	6.18	5.05	5.96
4.83	5.51	5.62	4.18	5.5	.	5.47	5.92	5.47	5.47	4.01	5.36	5.46	4.92	6.05	5.39	6.05	5.17	5.8	5.29	5.71	6.42	6.22	5.05	5.96
4.83	5.51	5.62	4.18	5.52	.	5.47	5.92	5.56	5.47	4.01	5.36	5.58	4.92	6.14	5.44	6.1	5.17	5.8	5.29	5.71	6.42	6.22	5.05	5.96
4.83	5.51	5.64	4.18	5.52	.	5.47	6.22	5.56	5.47	4.06	5.36	5.58	5.09	6.14	5.44	6.1	5.17	5.8	5.29	5.72	6.55	6.22	5.12	5.96
4.89	5.67	5.64	4.18	5.6	.	5.48	6.22	5.59	5.62	4.12	5.36	5.58	5.09	6.14	5.44	6.1	5.32	5.8	5.3	5.72	6.55	6.28	5.13	6.12
4.89	5.67	5.64	4.3	5.6	.	5.65	6.22	5.75	5.62	4.13	5.36	5.58	5.09	6.15	5.44	6.18	5.32	5.99	5.3	5.72	6.55	6.28	5.13	6.12
4.95	5.7	5.64	4.34	5.66	.	5.65	6.22	5.76	5.62	4.13	5.36	5.58	5.09	6.15	5.44	6.21	5.32	5.99	5.38	5.72	6.55	6.37	5.13	6.12
4.95	5.7	5.64	4.34	5.66	.	5.65	6.22	5.76	5.62	4.13	5.56	5.68	5.21	6.17	5.54	6.21	5.32	5.99	5.54	5.78	6.67	6.37	5.13	6.36
4.99	5.71	5.86	4.34	5.66	.	5.65	6.22	5.76	5.74	4.21	5.56	5.68	5.21	6.17	5.54	6.21	5.47	5.99	5.55	5.78	6.67	6.37	5.22	6.36
4.99	5.71	5.86	4.57	5.66	.	5.65	6.22	5.76	5.74	4.24	5.56	5.68	5.21	6.17	5.58	6.21	5.47	5.99	5.55	5.78	6.67	6.89	5.25	6.36
5.03	5.82	5.86	4.57	5.68	.	5.75	6.22	5.86	5.74	4.24	5.56	5.68	5.21	6.24	5.58	6.23	5.47	6.16	5.55	5.78	6.67	7	5.26	6.36
5.03	5.82	5.86	4.57	5.68	.	5.75	6.43	5.86	5.89	4.24	5.56	5.68	5.21	6.24	5.58	6.42	5.48	6.16	5.55	5.79	6.75	7.23	5.32	6.36
5.1	5.82	6.05	4.74	5.68	.	5.75	6.43	6	5.89	4.24	5.56	5.8	5.21	6.24	5.58	6.42	5.48	6.16	5.69	5.79	6.75	7.23	5.32	6.36
5.1	5.93	6.05	4.74	5.68	.	5.75	6.43	6	5.89	4.24	5.7	5.8	5.21	6.24	5.8	6.42	5.72	6.16	5.69	5.91	6.75	7.23	5.43	6.36
5.1	5.93	6.28	4.74	5.68	.	5.85	6.43	6.18	6	4.49	5.71	5.8	5.35	6.35	5.8	6.52	5.76	6.23	5.69	5.91	6.94	7.42	5.43	6.5
5.29	6	6.28	4.89	5.83	.	5.85	6.43	6.18	6	4.5	5.71	5.8	5.35	6.36	5.8	6.52	5.76	6.23	5.69	5.91	6.95	7.42	5.44	6.5
5.29	6	6.28	4.89	5.83	.	5.85	6.47	6.18	6	4.5	5.71	5.8	5.45	6.36	5.8	6.52	5.76	6.25	5.69	5.91	6.95	7.42	5.52	6.5
5.46	6.2	6.28	4.89	5.83	.	5.95	6.47	6.18	6	4.5	5.71	6.15	5.45	6.43	6.09	6.52	5.76	6.29	5.94	5.91	7.3	7.69	5.52	6.5
5.47	6.2	6.28	4.89	6.06	.	5.95	6.81	6.37	6	4.73	5.92	6.15	5.57	6.44	6.09	6.74	6.08	6.29	5.95	6.13	7.31	7.72	5.52	6.74
5.47	6.2	6.44	5.04	6.06	.	6.11	6.81	6.37	6	4.73	5.92	6.15	5.58	6.44	6.09	6.82	6.08	6.43	5.95	6.13	7.31	7.72	5.6	6.74
5.47	6.39	6.44	5.04	6.06	.	6.11	6.81	6.49	6.21	4.73	5.92	6.28	5.58	6.89	6.09	6.82	6.09	6.43	5.97	6.13	7.32	7.77	5.6	6.74
5.56	6.39	6.44	5.04	6.39	.	6.11	7.07	6.49	6.21	4.73	5.92	6.28	5.58	6.89	6.17	6.82	6.09	6.43	5.97	6.25	7.44	7.93	5.77	6.81
5.56	6.39	6.75	5.27	6.39	.	6.12	7.08	6.49	6.21	4.83	6.01	6.28	5.72	6.89	6.36	6.82	6.4	6.44	6.13	6.25	7.44	7.93	5.77	7.04
5.79	6.52	6.75	5.3	6.39	.	6.27	7.08	6.49	6.21	4.83	6.02	6.43	5.73	6.89	6.36	6.82	6.4	6.44	6.13	6.25	7.45	8.03	5.97	7.04
5.79	6.52	6.75	5.3	6.39	.	6.27	7.08	6.68	6.21	4.83	6.13	6.43	6	7.25	6.36	7.05	6.4	6.44	6.13	6.25	7.45	8.03	5.97	7.17
5.79	6.52	6.75	5.3	6.55	.	6.33	7.26	6.68	6.36	4.83	6.13	6.43	6	7.25	6.36	7.06	6.4	6.7	6.13	6.4	7.46	8.03	5.97	7.17
5.79	6.52	6.78	5.3	6.55	.	6.34	7.26	6.87	6.36	4.83	6.13	6.43	6.01	7.26	6.36	7.06	6.55	6.7	6.27	6.4	7.58	8.03	5.97	7.17
5.79	6.52	6.78	5.3	6.55	.	6.34	7.26	6.87	6.5	4.83	6.54	6.43	6.01	7.27	6.59	7.33	6.55	6.7	6.27	6.66	7.62	8.03	5.97	7.41
5.98	6.6	7.05	5.3	6.6	.	6.34	7.37	6.94	6.72	4.83	6.54	6.43	6.01	7.27	6.72	7.33	6.55	6.82	6.6	6.68	7.62	8.27	6.14	7.42
5.98	6.6	7.1	5.3	6.6	.	6.34	7.37	6.94	6.72	4.83	6.54	6.69	6.03	7.27	6.85	7.33	6.55	6.82	6.61	6.68	7.62	8.39	6.31	7.54
5.98	6.76	7.1	5.31	6.6	.	6.34	7.37	6.94	6.72	4.83	6.54	6.69	6.03	7.27	6.85	7.33	6.57	7	6.9	6.84	7.69	8.4	6.38	7.79
6.19	6.76	7.1	5.32	6.6	.	6.35	7.5	6.94	6.73	4.83	6.55	6.69	6.11	7.43	6.97	7.41	6.57	7.01	6.91	6.87	7.71	8.58	6.38	7.79
6.19	6.76	7.1	5.32	6.73	.	6.35	7.5	7.04	6.73	5.08	6.55	6.69	6.27	7.43	6.98	7.61	6.82	7.03	6.95	6.99	7.72	8.6	6.38	7.79
6.19	6.76	7.1	5.38	6.73	.	6.47	7.65	7.04	7.14	5.16	6.55	6.69	6.27	7.43	6.98	7.62	6.82	7.03	6.95	6.99	7.92	8.6	6.46	7.8
6.19	6.96	7.1	5.39	6.87	.	6.47	7.7	7.07	7.14	5.16	6.55	6.69	6.41	7.43	6.98	7.62	6.82	7.03	7.14	7.07	7.92	8.72	6.59	7.8
6.19	6.96	7.29	5.39	6.98	.	6.47	7.7	7.18	7.14	5.16	6.55	7	6.41	7.43	6.98	7.62	6.95	7.03	7.15	7.1	7.97	8.72	6.6	7.83
6.4	6.96	7.29	5.43	6.98	.	6.47	7.7	7.18	7.14	5.17	6.55	7	6.41	7.43	7.02	7.62	6.95	7.23	7.15	7.17	7.97	8.73	6.65	8.11
6.4	7.09	7.29	5.43	6.98	.	6.47	7.81	7.18	7.14	5.17	6.77	7.12	6.41	7.43	7.02	7.76	7.23	7.37	7.15	7.19	7.97	8.87	6.67	8.11
6.41	7.1	7.29	5.43	7.24	.	6.47	7.81	7.21	7.26	5.17	6.77	7.13	6.41	7.69	7.02	7.77	7.38	7.37	7.15	7.28	7.97	8.87	6.67	8.11
6.41	7.11	7.62	5.6	7.24	.	6.58	7.81	7.21	7.48	5.17	6.77	7.19	6.49	7.73	7.02	7.8	7.39	7.37	7.35	7.28	7.98	8.88	6.7	8.23
6.41	7.13	7.62	5.61	7.25	.	6.58	7.81	7.35	7.48	5.22	6.77	7.19	6.49	7.74	7.02	7.81	7.39	7.38	7.35	7.4	8.22	8.88	6.83	8.24
6.66	7.32	7.62	5.61	7.49	.	6.58	7.99	7.35	7.48	5.22	6.77	7.19	6.5	7.74	7.02	7.81	7.48	7.38	7.53	7.5	8.22	9.15	6.87	8.24
6.7	7.36	7.62	5.61	7.5	.	6.58	7.99	7.35	7.64	5.33	6.99	7.2	6.5	7.74	7.02	7.81	7.48	7.52	7.53	7.5	8.25	9.18	6.87	8.24
6.7	7.36	7.62	5.64	7.5	.	6.59	7.99	7.48	7.65	5.4	7	7.2	6.51	7.74	7.02	7.82	7.48	7.75	7.53	7.59	8.25	9.26	6.87	8.24
6.7	7.36	7.77	5.64	7.5	.	6.59	8.06	7.49	7.65	5.48	7.01	7.2	6.6	7.74	7.02	7.85	7.48	7.75	7.61	7.63	8.25	9.29	6.89	8.35
6.95	7.5	7.93	5.84	7.7	.	6.63	8.06	7.55	7.65	5.48	7.01	7.2	6.6	7.74	7.02	7.86	7.48	7.75	7.67	7.63	8.25	9.29	6.93	8.36
6.95	7.5	7.93	5.85	7.7	.	6.63	8.07	7.55	7.8	5.48	7.01	7.2	6.6	7.74	7.02	7.86	7.48	7.85	7.67	7.74	8.25	9.29	6.93	8.57
6.95	7.51	7.93	6	7.7	.	6.63	8.07	7.55	7.8	5.48	7.19	7.61	6.6	7.74	7.02	7.86	7.48	7.85	7.67	7.74	8.4	9.34	7.08	8.57
6.95	7.51	7.93	6	7.7	.	6.63	8.07	7.66	7.8	5.48	7.19	7.62	6.6	8.11	7.02	7.86	7.48	7.85	7.67	7.74	8.4	9.34	7.19	8.57
6.95	7.51	7.93	6.09	8	.	6.63	8.07	7.66	7.8	5.48	7.19	7.62	6.6	8.11	7.02	7.86	7.62	7.85	7.79	7.75	8.4	9.43	7.19	8.57
6.95	7.57	8.01	6.09	8	.	6.86	8.07	7.68	7.8	5.48	7.19	7.62	6.7	8.11	7.02	7.87	7.62	7.91	7.9	7.75	8.48	9.51	7.27	8.77
6.95	7.57	8.01	6.18	8	.	6.87	8.12	7.68	7.94	5.48	7.19	7.62	6.71	8.19	7.02	7.87	7.62	7.91	7.9	7.92	8.49	9.51	7.29	8.77
7.13	7.57	8.01	6.29	8	.	6.89	8.12	7.68	7.95	5.48	7.19	7.62	6.71	8.21	7.02	7.87	7.62	7.91	7.9	7.92	8.5	9.51	7.29	8.77
7.15	7.57	8.06	6.29	8.08	.	6.89	8.12	7.68	7.96	5.48	7.19	7.62	6.93	8.21	7.02	7.87	7.62	7.98	7.9	7.92	8.62	9.51	7.37	8.77
7.15	7.7	8.06	6.3	8.09	.	6.89	8.12	7.68	7.97	5.53	7.23	7.62	6.94	8.22	7.02	7.87	7.62	7.98	8.13	7.93	8.62	9.56	7.42	8.77
7.15	7.7	8.06	6.3	8.27	.	6.89	8.26	7.8	7.97	5.59	7.23	7.63	6.94	8.22	7.02	7.87	7.75	7.99	8.13	7.96	8.69	9.56	7.43	8.84
7.15	7.7	8.18	6.3	8.27	.	6.89	8.26	7.8	7.97	5.6	7.23	7.75	6.94	8.22	7.02	8.22	7.75	8	8.13	7.96	8.74	9.63	7.53	8.84
7.15	7.7	8.18	6.3	8.36	.	6.89	8.26	7.8	7.97	5.6	7.23	7.76	6.94	8.22	7.02	8.22	7.75	8	8.44	8.05	8.75	9.65	7.57	9.07
7.27	7.7	8.18	6.31	8.36	.	6.9	8.26	7.8	7.97	5.65	7.23	7.76	6.94	8.22	7.02	8.22	7.75	8	8.44	8.15	8.76	9.77	7.57	9.09
7.37	7.7	8.19	6.31	8.36	.	6.9	8.26	7.97	8.25	5.74	7.23	7.77	6.94	8.22	7.02	8.23	7.75	8.16	8.44	8.15	8.9	9.95	7.57	9.09
7.37	7.7	8.27	6.36	8.36	.	6.9	8.26	7.97	8.25	5.74	7.23	7.77	6.94	8.22	7.02	8.23	7.93	8.27	8.44	8.15	8.9	9.96	7.57	9.09
7.52	7.79	8.38	6.36	8.36	.	6.95	8.26	7.97	8.25	5.74	7.23	7.77	6.94	8.22	7.02	8.23	7.93	8.36	8.45	8.15	8.91	9.99	7.65	9.35
7.52	7.79	8.38	6.36	8.38	.	6.96	8.26	8.13	8.51	5.74	7.38	7.77	6.94	8.39	7.05	8.23	7.93	8.36	8.45	8.15	8.91	9.99	7.67	9.35
7.52	7.79	8.38	6.52	8.38	.	6.96	8.26	8.17	8.52	5.74	7.39	7.91	6.94	8.39	7.17	8.24	7.93	8.36	8.45	8.35	9.14	10.24	7.67	9.35
7.52	7.79	8.38	6.52	8.38	.	7.09	8.26	8.17	8.52	5.74	7.39	7.91	7.03	8.39	7.17	8.24	7.93	8.36	8.46	8.35	9.14	10.24	7.7	9.45
7.52	7.82	8.38	6.52	8.53	.	7.09	8.26	8.17	8.52	5.74	7.4	7.91	7.03	8.39	7.17	8.24	7.93	8.56	8.46	8.35	9.31	10.24	7.7	9.45
7.8	7.98	8.41	6.55	8.53	.	7.09	8.28	8.26	8.52	5.78	7.51	7.91	7.17	8.39	7.17	8.24	8.15	8.56	8.61	8.35	9.31	10.38	7.71	9.45
7.8	7.98	8.41	6.55	8.53	.	7.11	8.28	8.26	8.52	5.8	7.51	7.91	7.17	8.39	7.17	8.24	8.15	8.56	8.61	8.35	9.32	10.44	7.76	9.67
7.8	7.98	8.63	6.55	8.53	.	7.11	8.44	8.28	8.52	5.82	7.51	7.91	7.17	8.39	7.17	8.24	8.16	8.69	8.61	8.35	9.34	10.44	7.76	9.67
7.8	7.98	8.63	6.55	8.53	.	7.11	8.44	8.35	8.54	5.82	7.51	7.91	7.17	8.39	7.33	8.38	8.16	8.69	8.61	8.35	9.34	10.48	7.77	9.67
7.8	7.98	8.63	6.67	8.53	.	7.11	8.44	8.35	8.54	5.82	7.51	7.91	7.18	8.39	7.33	8.38	8.17	8.69	8.61	8.35	9.47	10.55	7.88	9.67
7.84	7.98	8.63	6.67	8.53	.	7.28	8.44	8.41	8.54	6.01	7.51	8.19	7.18	8.39	7.51	8.38	8.17	8.69	8.72	8.52	9.47	10.55	7.88	9.8
7.84	7.98	8.64	6.67	8.53	.	7.28	8.44	8.41	8.54	6.08	7.74	8.19	7.3	8.39	7.55	8.49	8.18	8.69	8.72	8.52	9.47	10.55	8.02	9.8
7.84	8.06	8.64	6.68	8.66	.	7.29	8.57	8.48	8.78	6.08	7.74	8.19	7.3	8.75	7.55	8.49	8.34	8.69	8.72	8.52	9.47	10.62	8.02	9.8
7.87	8.06	8.64	6.69	8.66	.	7.29	8.57	8.51	8.78	6.19	7.74	8.19	7.3	8.75	7.55	8.49	8.34	8.71	8.77	8.52	9.47	10.63	8.02	9.98
7.88	8.06	8.64	6.89	8.67	.	7.3	8.57	8.51	8.78	6.2	7.74	8.19	7.3	8.77	7.55	8.49	8.34	8.71	8.77	8.52	9.47	10.64	8.03	9.98
7.88	8.06	8.64	.	8.67	.	7.31	8.57	8.53	8.78	6.31	.	8.2	7.38	8.77	7.57	8.49	8.34	8.82	8.78	8.52	9.47	10.71	8.12	9.98
7.88	8.21	8.64	.	8.69	.	7.47	8.68	8.53	8.82	6.32	.	8.2	7.38	8.77	7.57	8.49	8.34	8.82	8.78	8.52	9.62	10.72	8.12	9.98
7.89	8.21	8.64	.	8.69	.	7.47	8.68	8.53	8.82	6.36	.	8.2	7.38	8.77	7.57	8.76	8.34	8.84	8.95	8.6	9.62	10.77	8.19	10.16
8	8.21	8.8	.	8.69	.	7.47	8.68	8.68	8.93	6.45	.	8.2	7.56	8.77	7.57	8.76	8.54	8.84	9.03	8.6	9.62	10.77	8.19	10.16
8	8.21	8.8	.	8.69	.	7.47	8.68	8.82	8.93	6.48	.	8.2	7.56	8.77	7.57	8.76	8.55	8.84	9.04	8.6	9.97	10.96	8.19	10.16
8	8.34	8.8	.	8.91	.	7.5	8.7	8.82	8.94	6.48	.	8.21	7.56	8.84	7.76	8.76	8.55	9.03	9.04	8.6	9.97	10.96	8.2	10.16
8	8.34	8.8	.	8.91	.	7.51	8.96	8.82	8.94	6.48	.	8.49	7.81	8.88	7.77	8.91	8.74	9.03	9.04	8.6	9.97	10.96	8.2	10.2
8	8.34	9.03	.	8.91	.	7.65	8.96	8.82	8.94	6.48	.	8.49	7.81	8.88	7.77	9.05	8.74	9.1	9.04	8.65	9.98	10.96	8.25	10.2
8.26	8.34	9.03	.	8.91	.	7.65	8.96	8.85	8.94	6.62	.	8.49	7.81	8.88	7.77	9.06	8.75	9.14	9.04	8.78	9.98	11.03	8.25	10.2
8.26	8.65	9.03	.	9.25	.	7.65	9	9	9.1	6.62	.	8.5	7.87	8.88	7.99	9.21	8.9	9.14	9.34	8.78	9.98	11.03	8.4	10.44
8.26	8.65	9.03	.	9.25	.	7.89	9	9	9.1	6.69	.	8.5	7.88	9.25	8.01	9.21	8.91	9.14	9.34	8.78	9.98	11.09	8.41	10.44
8.26	8.65	9.03	.	9.25	.	7.89	9.21	9.02	9.1	6.69	.	8.5	7.88	9.25	8.01	9.21	8.91	9.34	9.34	8.82	10.07	11.16	8.41	10.44
8.49	8.65	9.03	.	9.37	.	7.9	9.21	9.02	9.26	6.76	.	8.69	7.92	9.25	8.01	9.29	8.92	9.53	9.34	8.85	10.07	11.18	8.44	10.68
8.49	8.77	9.03	.	9.37	.	8.09	9.21	9.06	9.26	6.79	.	8.69	7.93	9.25	8.01	9.29	9.21	9.71	9.34	8.85	10.07	11.18	8.56	10.68
8.49	8.77	9.27	.	9.37	.	8.09	9.4	9.07	9.26	6.81	.	8.94	8.02	9.25	8.01	9.46	9.21	9.74	9.64	9	10.14	11.29	8.56	10.68
8.63	8.77	9.27	.	9.51	.	8.09	9.41	9.09	9.31	6.87	.	8.94	8.02	9.28	8.01	9.46	9.22	9.74	9.64	9	10.14	11.48	8.73	10.95
8.63	8.77	9.27	.	9.56	.	8.09	9.41	9.17	9.32	6.87	.	8.95	8.16	9.28	8.29	9.46	9.22	9.74	9.64	9.08	10.14	11.48	8.73	10.95
8.63	8.77	9.27	.	9.57	.	8.09	9.44	9.17	9.33	6.92	.	8.95	8.17	9.28	8.29	9.46	9.23	9.74	9.64	9.09	10.23	11.48	8.8	11.13
8.63	8.78	9.27	.	9.57	.	8.09	9.63	9.17	9.33	6.96	.	8.95	8.2	9.31	8.29	9.67	9.32	9.74	9.86	9.23	10.23	11.69	8.99	11.13
8.66	9.01	9.48	.	9.57	.	8.18	9.63	9.17	9.43	6.96	.	8.95	8.2	9.31	8.29	9.67	9.58	9.74	9.86	9.35	10.33	11.69	8.99	11.24
8.67	9.01	9.48	.	9.59	.	8.43	9.75	9.29	9.51	6.96	.	8.96	8.2	9.31	8.65	9.68	9.58	9.82	9.95	9.43	10.33	11.69	8.99	11.24
8.85	9.21	9.48	.	9.59	.	8.43	9.89	9.29	9.56	6.96	.	8.96	8.22	9.57	8.65	9.68	9.65	9.82	10.15	9.43	10.33	11.78	9.05	11.24
8.85	9.24	9.61	.	9.74	.	8.43	9.89	9.29	9.67	6.96	.	8.96	8.34	9.58	8.65	9.87	9.9	9.95	10.15	9.54	10.37	11.94	9.05	11.24
8.85	9.24	9.61	.	9.96	.	8.43	10.04	9.44	9.67	7.15	.	9.28	8.34	9.6	8.66	9.87	10	9.96	10.36	9.54	10.37	11.94	9.14	11.24
8.87	9.5	9.61	.	9.96	.	8.43	10.05	9.44	9.86	7.16	.	9.28	8.42	9.6	8.66	10.2	10.01	9.96	10.36	9.69	10.37	12.06	9.17	11.42
8.87	9.5	9.79	.	9.98	.	8.51	10.05	9.45	9.86	7.17	.	9.31	8.5	9.6	8.9	10.21	10.01	9.96	10.48	9.69	10.46	12.08	9.17	11.42
9.15	9.69	9.79	.	10.17	.	8.51	10.05	9.45	9.86	7.17	.	9.31	8.57	9.6	8.9	10.21	10.01	9.96	10.48	9.82	10.47	12.09	9.19	11.42
9.15	9.69	9.79	.	10.26	.	8.72	10.23	9.57	9.86	7.17	.	9.31	8.63	9.6	8.91	10.21	10.15	10.09	10.58	9.82	10.49	12.28	9.24	11.57
9.15	9.7	9.79	.	10.32	.	8.72	10.25	9.57	9.86	7.17	.	9.31	8.64	9.7	9.17	10.39	10.38	10.22	10.58	9.83	10.52	12.29	9.24	11.71
9.17	9.91	9.87	.	10.32	.	8.72	10.5	9.57	10.03	7.37	.	9.49	8.75	9.7	9.17	10.4	10.52	10.33	10.6	9.99	10.52	12.29	9.24	11.71
9.29	9.91	9.87	.	10.32	.	8.98	10.5	9.65	10.03	7.37	.	9.49	8.81	9.7	9.17	10.4	10.52	10.33	10.6	9.99	10.52	12.29	9.24	11.71
9.29	10.01	9.87	.	10.32	.	9.04	10.5	9.66	10.04	7.37	.	9.49	8.88	9.7	9.17	10.4	10.52	10.33	10.6	10.07	10.52	12.37	9.34	11.71
9.29	10.01	9.87	.	10.32	.	9.04	10.5	9.66	10.12	7.37	.	9.68	8.89	9.7	9.17	10.4	10.52	10.6	10.7	10.07	10.52	12.4	9.38	11.87
9.29	10.01	9.87	.	10.32	.	9.06	10.68	9.79	10.14	7.47	.	9.71	8.89	9.7	9.17	10.4	10.52	10.61	10.7	10.15	10.55	12.4	9.48	11.87

9.52	10.01	9.87	.	10.32	.	9.06	10.68	9.79	10.16	7.47	.	9.78	8.89	9.7	9.17	10.4	10.59	10.65	10.7	10.16	10.55	12.6	9.48	11.91
9.52	10.01	10.05	.	10.32	.	9.06	10.68	9.79	10.16	7.47	.	9.78	8.95	9.7	9.17	10.4	10.78	10.65	10.7	10.16	10.55	12.6	9.48	12
9.52	10.01	10.05	.	10.48	.	9.14	10.95	9.79	10.34	7.47	.	9.78	8.96	9.7	9.17	10.66	10.88	10.65	10.7	10.16	10.91	12.6	9.53	12
9.52	10.01	10.05	.	10.48	.	9.14	10.96	9.87	10.34	7.47	.	9.78	8.96	9.7	9.4	10.66	10.88	10.65	10.7	10.16	10.91	12.61	9.53	12
9.52	10.06	10.05	.	10.49	.	9.14	11.02	9.88	10.34	7.64	.	9.78	8.97	9.7	9.4	10.66	10.88	10.65	10.71	10.16	10.91	12.66	9.53	12.27
9.61	10.08	10.05	.	10.5	.	9.14	11.02	9.88	10.34	7.66	.	9.78	8.97	9.7	9.4	10.66	10.88	10.65	10.87	10.23	10.97	12.66	9.58	12.27
9.61	10.08	10.05	.	10.57	.	9.14	11.02	9.88	10.34	7.71	.	9.78	8.97	9.7	9.4	10.66	10.88	10.66	10.87	10.23	10.97	12.66	9.59	12.27
9.65	10.08	10.05	.	10.64	.	9.2	11.13	9.88	10.34	7.71	.	10	9.15	9.7	9.65	10.66	11.08	10.66	11.02	10.37	10.97	12.67	9.7	12.44
9.78	10.2	10.11	.	10.64	.	9.21	11.14	9.88	10.34	7.71	.	10.01	9.24	9.7	9.65	10.94	11.3	10.66	11.02	10.4	10.97	13.06	9.7	12.44
9.78	10.2	10.11	.	10.64	.	9.21	11.15	9.88	10.34	7.71	.	10.01	9.24	9.7	9.66	10.94	11.3	10.76	11.02	10.42	11.06	13.06	9.7	12.57
9.79	10.2	10.11	.	10.64	.	9.21	11.16	9.88	10.34	7.71	.	10.06	9.3	9.7	9.66	10.94	11.3	10.76	11.02	10.42	11.06	13.06	9.75	12.57
9.79	10.2	10.11	.	10.86	.	9.21	11.16	9.88	10.38	7.71	.	10.06	9.3	9.71	9.66	10.97	11.3	10.76	11.02	10.46	11.06	13.08	9.75	12.57
9.79	10.21	10.11	.	10.87	.	9.24	11.16	9.88	10.38	7.71	.	10.06	9.3	9.71	9.66	11.06	11.3	10.76	11.02	10.46	11.06	13.08	9.82	12.69
9.79	10.21	10.11	.	10.89	.	9.24	11.19	9.88	10.38	7.71	.	10.06	9.3	9.78	9.81	11.14	11.31	10.76	11.12	10.61	11.06	13.08	9.82	12.7
9.82	10.29	10.33	.	10.91	.	9.24	11.19	9.88	10.43	7.75	.	10.06	9.46	9.78	9.95	11.14	11.32	10.91	11.12	10.66	11.14	13.08	9.89	12.99
9.82	10.29	10.33	.	10.91	.	9.35	11.19	9.88	10.44	7.75	.	10.06	9.46	9.78	9.95	11.14	11.4	10.91	11.12	10.66	11.14	13.32	9.99	12.99
9.91	10.42	10.33	.	10.91	.	9.35	11.19	9.97	10.44	7.78	.	10.06	9.55	9.78	9.95	11.14	11.46	10.93	11.12	10.66	11.14	13.33	9.99	12.99
9.91	10.42	10.33	.	10.91	.	9.35	11.19	9.97	10.44	7.78	.	10.25	9.57	9.78	9.98	11.27	11.46	11.11	11.12	10.66	11.14	13.33	10	12.99
9.91	10.42	10.33	.	11.13	.	9.35	11.32	9.97	10.44	7.78	.	10.25	9.57	9.78	9.98	11.27	11.46	11.11	11.12	10.66	11.14	13.39	10	13.12
9.94	10.42	10.33	.	11.14	.	9.36	11.32	9.98	10.44	7.85	.	10.25	9.63	9.78	9.98	11.27	11.46	11.11	11.24	10.7	11.32	13.6	10.04	13.12
9.94	10.53	10.34	.	11.14	.	9.48	11.32	10.03	10.54	7.85	.	10.25	9.63	9.85	9.98	11.27	11.46	11.11	11.24	10.7	11.32	13.6	10.05	13.29
10.06	10.53	10.34	.	11.34	.	9.48	11.32	10.03	10.54	8.14	.	10.25	9.7	9.85	9.98	11.27	11.61	11.11	11.24	10.9	11.32	13.6	10.05	13.29
10.06	10.53	10.34	.	11.36	.	9.61	11.32	10.03	10.54	8.17	.	10.25	9.78	9.85	9.98	11.34	11.61	11.11	11.4	10.91	11.57	13.6	10.12	13.44
10.06	10.83	10.34	.	11.36	.	9.61	11.43	10.03	10.61	8.17	.	10.25	9.78	9.85	10.15	11.49	11.62	11.11	11.4	10.91	11.57	13.61	10.12	13.44
10.06	10.99	10.6	.	11.36	.	9.61	11.44	10.03	10.61	8.17	.	10.28	9.78	9.85	10.16	11.49	11.63	11.11	11.4	10.91	11.57	13.61	10.12	13.44
10.06	10.99	10.6	.	11.36	.	9.61	11.44	10.03	10.61	8.17	.	10.28	9.78	9.85	10.16	11.49	11.63	11.11	11.41	10.91	11.58	13.68	10.23	13.44
10.06	10.99	10.61	.	11.36	.	9.62	11.44	10.2	10.61	8.17	.	10.28	9.93	9.85	10.16	11.49	11.63	11.11	11.41	10.91	11.58	13.77	10.29	13.44
10.2	10.99	10.61	.	11.37	.	9.72	11.63	10.2	10.62	8.17	.	10.28	9.93	9.89	10.17	11.5	11.84	11.25	11.41	10.91	11.58	13.78	10.29	13.44
10.2	10.99	10.61	.	11.37	.	9.72	**11.64**	**10.21**	10.62	8.17	.	10.28	9.93	9.89	**10.17**	**11.59**	11.84	11.25	11.61	10.91	11.59	13.78	10.29	13.44
10.2	11.06	10.76	.	11.37	.	9.72	11.64	10.21	10.62	8.17	.	10.28	9.93	9.9	10.18	11.59	11.85	11.25	11.61	11.03	11.59	13.79	10.29	13.44
																	11.85	11.25	11.61					
																	11.85	11.25	11.73					
																	11.86	11.25	11.73					

2	5	6	6	8	9	11		1	2	2	6	6	8	9	9	9	11	11	11	11	1	1	1
Box-3	Box-1	Box-2	Box-3	Box-8	Box-7	Box-5		Box-6	Box-6	Box-8	Box-8	Box-6	Box-5	Box-1	Box-2	Box-6	Box-1	Box-2	Box-4	Box-6	Box-7	Box-8	Box-2
17.4	28.1	41	42	53.2	71	82		12.1	23.3	27.5	37	46	50.1	62	63	69	74	76	81	83	1.1	2.2	5.5
M	M	M	M	M	M	M		M	M	M	M	M	M	M	M	M	M	M	M	M	M	M	M
EtOH	EtOH	EtOH	EtOH	EtOH	EtoH	EtOH		H2O	H2O	H2O	H2O	H2O	H2O	H2O	H2O	H2O	H2O	H2O	H2O	H2O	Malto	Malto	Malto
ME	ME	ME	ME	ME	ME	ME		MH	MH	MH	MH	MH	MH	MH	MH	MH	MH	MH	MH	MH	MM	MM	MM
0	0	0	0	0	0	0		0	0	0	0	0	0	0	0	0	0	0	0	0	0	0	0
0	0	0.14	0	0	0	0		0.15	0	0	0	0	0	0	0	0	0	0	0	0	0.05	0	0.12
0	0.02	0.26	0.16	0	0	0		0.2	0	0	0	0.14	0	0	0	0	0	0	0	0	0.1	0	0.12
0	0.35	0.26	0.22	0	0	0		0.34	0.03	0	0.11	0.18	0	0.26	0	0	0	0	0	0	0.12	0	0.4
0	0.36	0.52	0.35	0	0	0		0.37	0.37	0.18	0.12	0.32	0	0.28	0	0	0	0	0	0	0.19	0.01	0.55
0	0.36	0.52	0.35	0	0	0		0.37	0.39	0.18	0.38	0.44	0	0.28	0	0	0	0	0	0	0.31	0.19	0.75
0	0.58	0.67	0.59	0	0	0		0.47	0.39	0.18	0.63	0.45	0	0.45	0.17	0	0	0	0	0.01	0.31	0.33	0.8
0.03	0.7	0.77	0.59	0	0	0		0.48	0.64	0.18	0.63	0.72	0	0.45	0.18	0	0	0	0	0.01	0.42	0.43	1.21
0.03	0.88	0.9	0.93	0.19	0	0		0.48	0.64	0.24	0.82	0.73	0	0.59	0.28	0	0.08	0	0	0.01	0.64	0.61	1.44
0.03	0.9	0.9	0.93	0.29	0	0		0.71	0.64	0.24	0.93	1.01	0.44	0.76	0.38	0.11	0.08	0	0.09	0.02	0.64	0.72	1.6
0.03	0.97	1.28	0.94	0.4	0	0		0.84	0.64	0.24	1.01	1.01	0.54	0.85	0.63	0.16	0.08	0	0.22	0.02	0.74	0.94	1.76
0.03	1.12	1.28	1.06	0.52	0	0		0.84	0.95	0.24	1.28	1.25	0.54	0.97	0.7	0.2	0.08	0	0.29	0.12	0.84	1.07	1.89
0.03	1.15	1.37	1.29	0.75	0	0.04		0.84	0.95	0.49	1.59	1.5	0.7	1.1	0.81	0.29	0.17	0	0.35	0.44	0.98	1.29	2.02
0.14	1.15	1.61	1.29	0.76	0.17	0.04		0.88	1.27	0.49	1.65	1.5	0.83	1.31	0.83	0.38	0.26	0	0.39	0.53	0.98	1.29	2.02
0.17	1.32	1.77	1.9	1	0.18	0.04		1.07	1.28	0.49	1.88	1.67	0.92	1.32	0.93	0.52	0.26	0	0.54	0.53	1.02	1.46	2.02
0.17	1.46	1.8	1.9	1.08	0.18	0.24		1.21	1.51	0.49	2	1.67	1.19	1.46	0.98	0.65	0.42	0	0.64	0.58	1.15	1.49	2.11
0.17	1.46	1.8	1.9	1.32	0.25	0.3		1.49	1.69	0.49	2	1.79	1.3	1.54	1.03	0.73	0.53	0	0.69	0.69	1.15	1.65	2.2
0.17	1.6	1.8	1.9	1.32	0.25	0.34		1.49	1.69	0.49	2	1.93	1.51	1.6	1.06	0.89	0.53	0	0.89	0.69	1.2	1.84	2.47
0.17	1.75	1.99	1.93	1.32	0.27	0.37		1.74	1.69	0.6	2.22	1.94	1.79	1.6	1.06	1.09	0.56	0	1.13	1	1.4	1.85	2.47
0.17	1.75	2.17	2.02	1.63	0.44	0.42		1.74	1.87	1.04	2.44	2.03	1.79	1.6	1.14	1.19	0.62	0	1.16	1.32	1.56	1.89	2.47
0.17	1.89	2.17	2.03	1.68	0.48	0.52		1.74	1.87	1.04	2.63	2.09	1.86	1.6	1.21	1.19	0.77	0	1.4	1.51	1.73	1.89	2.47
0.17	2.05	2.17	2.22	1.68	0.82	0.65		1.83	2.17	1.04	2.63	2.34	2.01	1.77	1.5	1.21	0.78	0	1.45	1.82	1.73	1.89	2.47
0.17	2.05	2.17	2.38	1.68	1.01	0.76		1.86	2.17	1.04	2.63	2.34	2.01	1.77	1.5	1.21	0.93	0.03	1.45	1.82	1.73	1.89	2.47
0.17	2.05	2.45	2.38	1.73	1.01	0.77		1.97	2.17	1.04	2.91	2.45	2.17	1.77	1.5	1.21	1.02	0.06	1.59	1.82	1.97	2.22	2.62
0.17	2.05	2.45	2.38	1.85	1.01	0.78		2.26	2.17	1.11	3.06	2.45	2.25	1.95	1.5	1.21	1.06	0.07	1.63	1.83	1.97	2.22	2.62
0.17	2.16	2.45	2.38	1.85	1.01	0.78		2.31	2.17	1.14	3.23	2.45	2.25	2.16	1.5	1.33	1.06	0.07	2	1.83	1.97	2.22	2.62
0.17	2.17	2.45	2.38	1.85	1.01	0.81		2.33	2.3	1.14	3.23	2.45	2.42	2.16	1.65	1.47	1.06	0.07	2.02	1.84	1.97	2.22	2.62
0.17	2.17	2.45	2.55	2.17	1.1	0.81		2.47	2.3	1.14	3.23	2.45	2.42	2.53	1.67	1.6	1.06	0.25	2.02	1.98	1.97	2.4	2.73
0.17	2.17	2.45	2.55	2.17	1.25	0.81		2.47	2.3	1.49	3.41	2.45	2.59	2.53	1.82	1.6	1.06	0.29	2.2	2.1	2.05	2.4	2.97
0.17	2.17	2.45	2.67	2.17	1.25	0.81		2.67	2.32	1.64	3.61	2.45	2.59	2.53	1.82	1.6	1.06	0.29	2.2	2.11	2.05	2.62	2.97
0.35	2.17	2.66	2.67	2.17	1.25	0.81		2.98	2.32	1.64	3.61	2.45	2.61	2.53	2.14	1.77	1.06	0.29	2.21	2.19	2.05	2.63	2.97
0.35	2.4	2.68	2.67	2.21	1.25	0.81		2.98	2.74	1.64	3.61	2.45	2.8	2.53	2.14	1.77	1.06	0.33	2.22	2.19	2.31	2.63	2.97
0.35	2.4	2.68	2.68	2.58	1.25	0.83		3.27	2.74	1.64	3.73	2.71	2.8	2.53	2.14	1.78	1.06	0.34	2.48	2.19	2.31	2.64	3.15
0.35	2.4	2.68	2.99	2.58	1.41	0.88		3.27	2.74	1.92	3.73	2.82	2.8	2.97	2.14	1.82	1.06	0.44	2.48	2.27	2.31	2.75	3.25
0.44	2.6	2.68	2.99	2.58	1.41	1.1		3.27	2.74	1.92	3.73	2.82	2.8	2.97	2.14	1.96	1.09	0.55	2.48	2.28	2.32	3.03	3.42
0.58	2.6	2.68	2.99	2.58	1.41	1.19		3.44	3.04	2.08	3.97	2.82	2.8	2.97	2.14	1.96	1.1	0.59	2.48	2.3	2.33	3.03	3.45
0.58	2.6	2.97	3.06	2.58	1.63	1.19		3.44	3.04	2.08	3.97	2.82	2.8	2.97	2.14	1.96	1.1	0.59	2.55	2.32	2.47	3.03	3.45
0.58	2.69	3.02	3.06	2.58	1.67	1.19		3.44	3.04	2.08	3.97	2.87	2.96	2.97	2.22	1.96	1.1	0.61	2.64	2.32	2.51	3.03	3.45
0.58	2.75	3.02	3.06	2.67	1.67	1.19		3.44	3.04	2.08	4.05	3.05	3.02	2.97	2.24	1.96	1.1	0.61	2.64	2.32	2.51	3.03	3.56
0.62	2.75	3.02	3.06	2.67	1.67	1.25		3.44	3.04	2.08	4.05	3.05	3.02	2.97	2.24	1.96	1.17	0.62	2.64	2.32	2.51	3.16	3.56
0.74	2.75	3.02	3.3	2.67	1.67	1.25		3.67	3.15	2.08	4.15	3.05	3.02	3.18	2.24	2.01	1.22	0.66	2.64	2.32	2.51	3.16	3.56
0.8	2.75	3.24	3.3	2.67	1.67	1.25		3.67	3.15	2.08	4.28	3.05	3.06	3.18	2.24	2.01	1.22	0.68	2.65	2.32	2.51	3.16	3.76
0.8	2.8	3.24	3.3	2.67	1.67	1.25		3.67	3.39	2.08	4.37	3.27	3.1	3.18	2.24	2.01	1.22	0.69	2.65	2.32	2.51	3.16	3.76
0.8	2.8	3.24	3.3	2.67	1.82	1.25		3.67	3.4	2.08	4.38	3.28	3.1	3.18	2.24	2.01	1.22	0.69	2.65	2.32	2.52	3.41	3.76
0.8	2.8	3.24	3.3	2.67	1.82	1.26		3.88	3.4	2.08	4.38	3.28	3.1	3.18	2.24	2.01	1.22	0.7	2.78	2.32	2.62	3.41	3.76
0.91	2.8	3.24	3.43	2.67	1.82	1.27		3.88	3.4	2.09	4.38	3.28	3.32	3.26	2.24	2.01	1.22	0.7	2.78	2.32	2.62	3.41	3.76
0.91	2.96	3.24	3.43	2.67	1.82	1.27		3.88	3.4	2.39	4.38	3.47	3.32	3.28	2.24	2.01	1.36	0.7	2.78	2.32	2.62	3.42	3.76
0.91	2.96	3.24	3.43	2.81	1.82	1.27		3.88	3.4	2.39	4.42	3.47	3.32	3.28	2.25	2.08	1.36	0.7	2.78	2.32	2.62	3.42	3.93
0.91	2.96	3.24	3.43	2.81	1.82	1.27		3.95	3.4	2.39	4.54	3.47	3.32	3.28	2.25	2.08	1.36	0.7	2.78	2.32	2.62	3.42	3.93
0.91	2.96	3.39	3.67	2.81	1.84	1.27		4	3.4	2.39	4.54	3.49	3.32	3.28	2.25	2.08	1.36	0.71	2.78	2.32	2.62	3.66	3.93
0.91	3	3.42	3.67	2.81	1.93	1.27		4	3.79	2.39	4.69	3.56	3.32	3.31	2.25	2.08	1.36	0.71	2.81	2.32	2.63	3.66	3.93
0.91	3	3.42	3.67	3	1.93	1.27		4	3.79	2.72	4.69	3.56	3.57	3.31	2.25	2.08	1.36	0.71	3.02	2.32	2.63	3.66	3.93
0.91	3	3.42	3.67	3.01	1.93	1.27		4	3.79	2.72	4.69	3.71	3.58	3.57	2.5	2.08	1.36	0.71	3.02	2.32	2.64	3.66	3.93
0.91	3	3.42	3.71	3.01	1.93	1.27		4.22	3.79	2.72	4.86	3.71	3.58	3.57	2.5	2.08	1.36	0.71	3.02	2.32	2.68	3.66	3.93
0.91	3.24	3.51	3.71	3.01	2.28	1.27		4.22	3.79	2.72	4.86	3.71	3.58	3.57	2.5	2.08	1.36	0.72	3.02	2.32	2.69	3.68	4.02
0.91	3.24	3.51	3.71	3.01	2.28	1.27		4.22	3.79	2.72	4.86	3.71	3.58	3.57	2.5	2.16	1.36	0.72	3.02	2.32	2.69	3.68	4.02
1.19	3.24	3.51	3.71	3.12	2.28	1.27		4.22	3.79	2.72	5.05	3.78	3.58	3.57	2.5	2.28	1.36	0.72	3.03	2.32	2.69	3.68	4.02
1.19	3.41	3.51	3.71	3.13	2.28	1.27		4.22	3.79	2.72	5.05	3.84	3.58	3.73	2.5	2.28	1.36	0.72	3.03	2.32	2.69	3.77	4.17
1.19	3.41	3.51	3.8	3.13	2.28	1.37		4.24	3.79	2.72	5.05	3.84	3.58	3.73	2.5	2.28	1.36	0.72	3.03	2.32	2.69	3.77	4.17
1.19	3.41	3.59	3.8	3.13	2.63	1.37		4.24	3.94	2.72	5.05	3.95	3.58	3.73	2.5	2.28	1.36	0.72	3.15	2.32	2.69	3.77	4.17
1.19	3.41	3.59	3.8	3.13	2.64	1.37		4.24	4.08	3.18	5.15	4.06	3.78	3.73	2.5	2.28	1.36	0.77	3.15	2.32	2.7	3.77	4.18
1.19	3.41	3.59	3.8	3.13	2.64	1.37		4.39	4.08	3.19	5.22	4.06	3.78	3.9	2.63	2.34	1.36	0.81	3.15	2.32	2.7	3.8	4.36
1.48	3.71	3.59	3.8	3.3	2.65	1.37		4.39	4.08	3.19	5.22	4.06	3.78	3.98	2.67	2.51	1.36	0.82	3.15	2.32	2.7	3.94	4.36
1.48	3.73	3.59	4.1	3.3	2.66	1.37		4.39	4.33	3.19	5.22	4.25	4.04	3.98	2.67	2.51	1.36	0.82	3.17	2.32	2.7	3.94	4.36
1.48	3.73	3.78	4.1	3.3	2.66	1.37		4.39	4.4	3.19	5.43	4.25	4.04	3.98	2.67	2.51	1.36	0.82	3.23	2.32	2.7	3.94	4.36
1.48	3.73	3.81	4.1	3.3	2.8	1.37		4.6	4.4	3.19	5.43	4.25	4.04	3.98	2.71	2.64	1.36	0.82	3.23	2.32	2.7	3.94	4.36
1.48	3.73	3.81	4.62	3.3	2.8	1.37		4.61	4.4	3.19	5.43	4.32	4.04	4.15	2.71	2.69	1.36	0.82	3.23	2.32	2.93	3.94	4.65
1.62	3.73	3.99	4.62	3.31	2.8	1.37		4.61	4.4	3.32	5.6	4.49	4.28	4.15	2.71	2.69	1.4	0.85	3.23	2.32	3	4.08	4.76
1.74	4.02	4	4.62	3.4	3.05	1.4		4.61	4.4	3.63	5.6	4.63	4.28	4.24	2.93	2.69	1.41	1.17	3.27	2.41	3	4.16	4.76
1.74	4.02	4.16	4.62	3.75	3.06	1.4		4.61	4.4	3.63	5.81	4.63	4.28	4.44	2.93	2.94	1.41	1.38	3.27	2.42	3.03	4.21	4.97
2.06	4.08	4.31	4.62	3.75	3.08	1.4		4.88	4.69	3.63	6.04	4.73	4.5	4.47	3.08	3.25	1.41	1.58	3.28	2.42	3.17	4.32	5.08
2.06	4.23	4.31	4.81	3.75	3.08	1.54		4.89	4.88	4.08	6.04	4.82	4.5	4.6	3.08	3.27	1.5	1.58	3.46	2.45	3.17	4.45	5.45
2.06	4.43	4.64	4.81	3.83	3.08	1.6		4.89	4.88	4.1	6.31	4.84	4.68	4.87	3.29	3.27	1.56	1.8	3.46	2.61	3.29	4.48	5.67
2.06	4.45	4.64	5.07	4.09	3.08	1.78		5.18	5.19	4.1	6.31	5.07	4.91	4.87	3.43	3.27	1.56	1.84	3.62	2.63	3.76	4.63	5.74
2.06	4.61	4.93	5.16	4.33	3.18	1.86		5.18	5.23	4.1	6.5	5.28	4.93	4.87	3.43	3.54	1.66	1.85	3.78	2.9	3.79	4.78	6.06
2.35	4.61	5.17	5.34	4.46	3.18	2.04		5.18	5.58	4.34	6.68	5.33	4.93	5.1	3.43	3.56	1.77	2.07	3.78	2.99	4	4.78	6.06
2.37	4.77	5.17	5.34	4.48	3.34	2.04		5.4	5.58	4.34	6.68	5.55	5.18	5.1	3.64	3.56	1.88	2.07	3.78	3.14	4	4.86	6.21
2.37	4.77	5.46	5.56	4.87	3.34	2.13		5.45	5.58	4.34	6.68	5.55	5.18	5.1	3.64	3.64	1.88	2.07	3.78	3.2	4.13	5.03	6.5
2.37	5.01	5.46	5.56	4.87	3.45	2.2		5.5	5.58	4.34	6.84	5.82	5.32	5.34	3.98	3.93	1.89	2.07	4.05	3.28	4.13	5.03	6.7
2.37	5.01	5.46	5.57	4.87	3.46	2.27		5.68	5.58	4.69	7.01	6.02	5.52	5.39	3.98	4.18	2.11	2.07	4.13	3.52	4.31	5.11	6.7
2.56	5.15	5.8	5.59	4.87	3.46	2.27		5.68	5.96	4.69	7.15	6.04	5.64	5.39	3.98	4.18	2.13	2.37	4.24	3.54	4.31	5.26	6.7
2.56	5.2	5.8	5.59	5.11	3.52	2.27		5.68	5.96	4.69	7.42	6.04	5.78	5.69	3.98	4.18	2.13	2.59	4.31	3.75	4.31	5.26	6.7
2.56	5.2	5.8	5.75	5.23	3.52	2.48		5.81	5.96	4.69	7.42	6.33	6.02	5.96	3.98	4.4	2.45	2.59	4.52	3.75	4.48	5.51	6.94
2.8	5.21	5.8	5.75	5.23	3.52	2.69		5.87	5.96	4.69	7.63	6.33	6.2	5.96	4.35	4.51	2.48	2.88	4.52	3.75	4.49	5.51	6.95
3.06	5.4	6.01	6.01	5.23	3.55	2.74		5.95	5.96	5.06	7.63	6.33	6.2	6.01	4.35	4.51	2.73	2.88	4.65	3.77	4.69	5.51	7.11
3.06	5.44	6.01	6.01	5.23	3.87	3.06		5.95	6.19	5.15	7.81	6.51	6.2	6.01	4.35	4.82	2.73	2.89	4.65	4	4.69	5.51	7.11
3.06	5.63	6.01	6.01	5.23	4.03	3.06		5.95	6.34	5.15	7.81	6.51	6.36	6.01	4.62	4.82	2.73	2.89	4.71	4	4.86	5.51	7.48
3.06	5.63	6.28	6.01	5.68	4.03	3.25		6.09	6.61	5.15	7.81	6.51	6.36	6.26	4.62	5.1	2.8	2.89	4.71	4	5.09	5.51	7.49
3.28	5.81	6.28	6.01	5.68	4.03	3.33		6.18	6.61	5.15	7.81	6.51	6.36	6.27	4.62	5.1	3.07	2.89	4.71	4.3	5.09	5.51	7.49
3.28	5.81	6.28	6.23	5.68	4.03	3.65		6.18	6.61	5.15	8.1	6.6	6.36	6.29	4.94	5.1	3.28	2.89	4.71	4.39	5.09	5.75	7.49
3.28	5.81	6.29	6.23	5.68	4.09	3.66		6.18	6.61	5.15	8.1	6.7	6.36	6.29	5.05	5.1	3.29	3.34	4.71	4.39	5.09	5.75	7.49
3.4	5.81	6.55	6.23	5.68	4.19	3.66		6.18	6.61	5.15	8.1	6.7	6.82	6.29	5.05	5.1	3.29	3.34	4.71	4.72	5.09	5.75	7.83
3.4	6.01	6.55	6.51	6.03	4.19	3.66		6.18	6.99	5.15	8.39	6.7	6.82	6.29	5.05	5.1	3.29	3.34	4.71	4.8	5.09	5.75	7.85
3.4	6.01	6.55	6.51	6.03	4.19	3.66		6.33	6.99	5.15	8.39	6.7	6.82	6.29	5.05	5.1	3.29	3.34	4.71	4.8	5.09	5.75	7.85
3.4	6.24	6.56	6.51	6.03	4.46	3.66		6.43	6.99	5.45	8.59	6.73	6.82	6.29	5.05	5.14	3.29	3.34	4.71	4.82	5.09	5.94	7.85
3.4	6.24	6.56	6.51	6.03	4.65	3.66		6.48	6.99	5.48	8.59	6.73	6.82	6.29	5.05	5.26	3.29	3.34	4.72	4.82	5.09	5.94	7.85
3.4	6.36	6.72	6.86	6.03	4.65	3.78		6.48	6.99	5.48	8.84	6.89	7.04	6.29	5.05	5.26	3.29	3.34	4.83	4.82	5.34	5.94	8.14
3.4	6.44	6.72	6.86	6.03	4.65	3.79		6.86	6.99	5.48	8.84	6.89	7.04	6.29	5.05	5.26	3.29	3.62	4.83	5.06	5.49	5.99	8.14
3.53	6.47	6.72	6.93	6.03	4.66	3.79		6.91	7.2	5.67	8.93	6.89	7.04	6.38	5.3	5.26	3.63	3.65	4.92	5.24	5.49	6.07	8.14
3.7	6.47	6.72	6.93	6.03	4.93	3.79		6.91	7.31	5.67	8.93	7.1	7.14	6.52	5.3	5.41	3.93	3.88	5.08	5.24	5.49	6.15	8.16
3.7	6.47	6.76	6.93	6.33	4.93	3.79		6.97	7.31	5.67	9.07	7.1	7.21	6.52	5.3	5.41	3.93	4.03	5.08	5.24	5.49	6.15	8.16
3.72	6.61	6.86	6.93	6.75	4.93	4		7.12	7.31	5.67	9.07	7.1	7.21	6.52	5.3	5.41	3.93	4.03	5.08	5.24	5.49	6.15	8.16
4.09	6.61	6.86	6.93	6.75	4.93	4		7.12	7.63	5.67	9.17	7.19	7.21	6.52	5.3	5.41	3.93	4.03	5.09	5.49	5.61	6.15	8.39
4.09	6.61	6.86	7.01	6.75	5.23	4		7.12	7.63	5.67	9.22	7.28	7.21	6.56	5.3	5.41	3.93	4.43	5.09	5.49	5.83	6.29	8.39
4.09	6.61	6.86	7.1	6.75	5.23	4		7.34	7.63	5.67	9.22	7.28	7.4	6.73	5.3	5.41	3.93	4.44	5.09	5.49	5.83	6.29	8.39
4.09	6.61	6.86	7.1	6.75	5.23	4.29		7.38	7.63	5.67	9.37	7.28	7.4	6.73	5.62	5.41	3.93	4.44	5.18	5.49	5.83	6.29	8.39
4.47	6.61	6.86	7.1	6.75	5.23	4.29		7.38	7.63	5.67	9.37	7.45	7.4	6.73	5.63	5.41	3.93	4.44	5.18	5.49	5.83	6.29	8.43
4.5	6.61	6.86	7.15	6.79	5.23	4.29		7.57	7.63	5.92	9.37	7.45	7.66	6.85	5.63	5.42	3.95	4.44	5.18	5.49	5.83	6.42	8.43
4.5	6.61	7.1	7.15	7.09	5.42	4.29		7.57	7.63	5.92	9.55	7.45	7.66	6.85	5.63	5.46	3.95	4.44	5.18	5.53	5.83	6.48	8.64
4.5	6.61	7.1	7.15	7.09	5.42	4.29		7.57	7.63	5.92	9.55	7.45	7.66	6.85	5.63	5.46	3.95	4.44	5.18	5.53	5.83	6.48	8.64
4.5	6.75	7.1	7.15	7.09	5.42	4.29		7.57	7.64	5.92	9.55	7.45	7.66	6.85	5.63	5.46	3.95	4.44	5.19	5.53	5.84	6.48	8.64
4.5	6.75	7.1	7.15	7.09	5.42	4.29		7.57	7.64	5.97	9.59	7.45	7.66	7.05	5.63	5.46	3.95	4.73	5.19	5.53	5.84	6.48	8.64
4.5	6.75	7.1	7.15	7.09	5.42	4.29		7.57	7.64	5.97	9.59	7.63	7.66	7.07	5.63	5.55	3.95	4.74	5.19	5.53	5.84	6.54	8.7
4.5	6.75	7.1	7.15	7.47	5.47	4.29		7.69	7.86	5.97	9.76	7.67	7.66	7.07	5.9	5.59	3.95	4.74	5.19	5.53	5.84	6.61	8.7

4.79	6.76	.	.	7.47	5.71	4.29	7.69	7.88	5.97	.	.	7.85	7.07	5.9	5.59	3.95	4.74	5.19	5.59	5.84	6.61	8.7
4.79	6.76	.	.	7.47	5.71	4.29	7.69	7.88	5.97	.	.	7.85	7.07	5.9	5.59	4.21	4.74	5.23	5.59	5.84	6.61	8.8
4.79	6.76	.	.	7.47	5.71	4.29	7.8	7.88	5.97	.	.	7.85	7.07	5.9	5.78	4.21	4.74	5.23	5.59	5.86	6.61	8.8
4.79	.	.	.	7.47	5.71	4.4	7.95	8.22	6.34	.	.	7.85	7.07	5.9	5.78	4.36	4.74	5.23	5.59	5.94	6.7	8.8
4.79	.	.	.	7.64	5.99	4.42	7.95	8.22	6.35	.	.	7.87	7.07	5.9	5.78	4.45	4.74	5.23	5.59	5.94	6.7	8.86
4.79	.	.	.	7.64	5.99	4.42	7.95	8.22	6.35	.	.	7.87	7.07	5.9	5.78	4.45	4.74	5.23	5.59	5.94	6.7	8.93
4.79	.	.	.	7.65	5.99	4.42	7.95	8.22	6.35	.	.	7.88	7.07	5.9	5.78	4.45	4.74	5.23	5.59	5.94	6.7	8.93
4.79	.	.	.	7.65	5.99	4.42	7.95	8.24	6.35	.	.	7.88	7.2	5.99	5.97	4.45	4.74	5.23	5.59	5.95	6.7	8.93
4.79	.	.	.	7.65	5.99	4.42	7.95	8.24	6.35	.	.	7.88	7.2	5.99	5.97	4.45	4.74	5.23	5.59	5.95	6.7	8.93
5.17	.	.	.	7.82	6.17	4.58	8.28	8.24	6.35	.	.	7.88	7.2	5.99	5.97	4.45	4.74	5.23	5.59	5.99	6.79	8.94
5.17	.	.	.	7.87	6.17	4.58	8.28	8.24	6.69	.	.	8.02	7.2	5.99	5.97	4.45	4.74	5.23	5.63	6.35	6.79	9.12
5.17	.	.	.	7.87	6.34	4.58	8.28	8.59	6.86	.	.	8.02	7.2	5.99	5.97	4.45	4.74	5.23	5.63	6.35	6.79	9.12
5.17	.	.	.	7.87	6.34	4.58	8.49	8.59	6.86	.	.	8.02	7.4	5.99	6.25	4.72	4.74	5.27	5.63	6.35	6.96	9.12
5.17	.	.	.	7.87	6.34	4.58	8.62	8.59	6.86	.	.	8.29	7.46	6.26	6.25	4.72	4.74	5.29	5.63	6.36	6.97	9.24
5.17	.	.	.	7.87	6.34	4.87	8.62	8.59	6.86	.	.	8.29	7.46	6.26	6.25	4.72	4.74	5.29	5.71	6.37	6.97	9.31
5.17	.	.	.	7.87	6.57	4.87	8.67	8.84	6.86	.	.	8.29	7.46	6.26	6.25	4.72	4.86	5.29	5.94	6.37	6.97	9.31
5.24	.	.	.	7.93	6.57	4.87	8.67	8.94	7.25	.	.	8.29	7.46	6.26	6.25	4.72	4.92	5.29	5.94	6.38	6.97	9.31
5.24	.	.	.	8.29	6.57	4.87	8.67	8.94	7.3	.	.	8.41	7.48	6.26	6.49	4.72	4.92	5.29	5.94	6.54	6.97	9.55
5.36	.	.	.	8.29	6.57	4.87	8.67	8.94	7.3	.	.	8.47	7.55	6.26	6.49	4.94	5	5.29	5.94	6.54	7.06	9.62
5.73	.	.	.	8.29	6.68	5.08	8.79	8.94	7.3	.	.	8.71	7.84	6.29	6.49	4.94	5.22	5.29	5.98	6.68	7.35	9.81
5.73	.	.	.	8.29	7.04	5.08	8.79	8.94	7.73	.	.	8.71	7.84	6.5	6.72	5.24	5.22	5.58	5.98	6.88	7.56	9.81
5.74	.	.	.	8.56	7.05	5.08	9.03	9.26	7.73	.	.	8.91	8.12	6.5	6.78	5.24	5.34	5.67	6.23	6.88	7.56	10.19
6.22	.	.	.	8.68	7.13	5.38	9.05	9.26	7.73	.	.	8.93	8.18	6.88	6.78	5.55	5.49	5.85	6.23	7.3	7.97	10.23
6.24	.	.	.	8.69	7.56	5.4	9.22	9.39	7.75	.	.	9.01	8.18	6.97	6.83	5.55	5.56	6	6.4	7.44	7.98	10.56
6.24	.	.	.	8.95	7.56	5.4	9.23	9.57	7.75	.	.	9.18	8.18	6.97	7.17	5.62	5.56	6.33	6.5	7.56	7.98	10.56
6.24	.	.	.	8.95	7.56	5.72	9.4	9.61	8.34	.	.	9.18	8.47	6.97	7.17	5.62	5.56	6.33	6.71	7.56	7.98	10.77
6.24	.	.	.	9.06	7.56	5.72	9.48	9.71	8.36	.	.	9.18	8.47	7.1	7.34	5.95	5.79	6.33	6.73	7.79	8.13	10.99
6.24	.	.	.	9.28	7.66	6.02	9.5	9.94	8.36	.	.	9.41	8.47	7.1	7.52	5.95	5.89	6.35	6.73	7.86	8.32	10.99
6.48	.	.	.	9.49	7.66	6.02	9.71	9.94	8.36	.	.	9.58	8.74	7.1	7.55	5.95	5.89	6.35	6.89	7.98	8.38	11.17
6.48	.	.	.	9.49	7.66	6.02	9.71	9.94	8.36	.	.	9.59	8.74	7.15	7.55	6.31	6.02	6.74	6.91	8.1	8.38	11.19
6.76	.	.	.	9.49	7.66	6.28	9.83	9.94	8.43	.	.	9.64	9.03	7.32	7.81	6.31	6.02	6.9	6.95	8.32	8.64	11.19
6.78	.	.	.	9.62	7.85	6.42	9.86	10.16	8.74	.	.	10.01	9.07	7.38	7.81	6.33	6.02	6.9	7.09	8.74	8.64	11.2
6.78	.	.	.	9.62	7.85	6.42	9.86	10.21	8.74	.	.	10.01	9.07	7.5	7.86	6.33	6.06	7.37	7.22	8.74	8.64	11.38
6.94	.	.	.	9.75	7.85	6.43	9.86	10.21	8.74	.	.	10.01	9.27	7.5	8.21	6.33	6.15	7.37	7.33	8.74	8.84	11.38
6.97	.	.	.	10.07	7.89	6.58	10.09	10.21	8.74	.	.	10.26	9.3	7.7	8.21	6.56	6.24	7.37	7.36	8.74	8.87	11.47
6.98	.	.	.	10.34	7.89	6.58	10.09	10.32	8.74	.	.	10.28	9.3	7.72	8.21	6.56	6.26	7.51	7.6	8.74	8.87	11.81
6.98	.	.	.	10.34	7.89	6.63	10.09	10.35	8.74	.	.	10.52	9.3	7.72	8.21	6.71	6.26	7.62	7.72	9.16	8.87	11.81
6.98	.	.	.	10.34	7.89	6.63	10.37	10.35	8.74	.	.	10.52	9.59	7.96	8.21	6.79	6.26	8.03	7.87	9.16	8.87	11.81
7.08	.	.	.	10.34	7.91	6.66	10.37	10.35	9.18	.	.	10.52	9.59	8.02	8.38	6.94	6.26	8.03	7.88	9.16	8.87	12.02
7.08	.	.	.	10.56	8.21	6.78	10.37	10.72	9.18	.	.	10.71	9.59	8.02	8.42	7.14	6.28	8.03	7.88	9.16	8.87	12.02
7.08	.	.	.	10.56	8.21	6.85	10.37	10.72	9.18	.	.	10.76	9.59	8.02	8.42	7.14	6.28	8.03	7.88	9.16	9.23	12.02
7.08	.	.	.	10.56	8.21	6.85	10.42	10.72	9.18	.	.	10.76	9.59	8.02	8.42	7.14	6.28	8.03	7.96	9.24	9.23	12.02
7.29	.	.	.	10.56	8.21	6.85	10.42	10.72	9.18	.	.	10.76	9.59	8.02	8.63	7.28	6.4	8.04	7.96	9.53	9.23	12.02
7.29	.	.	.	10.56	8.21	7.14	10.68	10.72	9.18	.	.	10.76	9.59	8.02	8.63	7.32	6.45	8.04	7.96	9.53	9.42	12.02
7.29	.	.	.	10.56	8.21	7.14	10.68	10.72	9.53	.	.	11	9.59	8.02	8.63	7.32	6.45	8.12	8.08	9.53	9.42	12.27
7.29	.	.	.	10.68	8.21	7.14	10.71	10.73	9.54	.	.	11	9.89	8.13	8.63	7.32	6.45	8.18	8.08	9.53	9.42	12.27
7.29	.	.	.	10.95	8.21	7.23	10.85	10.73	9.54	.	.	11	9.89	8.13	8.63	7.32	6.45	8.29	8.18	9.53	9.42	12.27
7.32	.	.	.	10.95	8.21	7.32	10.85	11.08	9.54	.	.	11.21	10.07	8.22	8.63	7.32	6.47	8.31	8.19	9.53	9.42	12.27
7.32	.	.	.	11.01	8.21	7.32	11.05	11.08	9.54	.	.	11.21	10.07	8.22	8.63	7.32	6.78	8.31	8.19	9.53	9.43	12.27
7.32	.	.	.	11.26	8.24	7.32	11.16	11.08	9.54	.	.	11.53	10.09	8.32	8.63	7.32	6.78	8.31	8.28	9.53	9.66	12.27
7.6	.	.	.	11.26	8.38	7.32	11.16	11.08	9.58	.	.	11.53	10.16	8.32	8.76	7.34	6.78	8.31	8.32	9.53	9.8	12.27
7.65	.	.	.	11.26	8.38	7.33	11.19	11.08	9.58	.	.	11.53	10.16	8.32	8.76	7.34	6.78	8.35	8.37	9.53	9.8	12.27
7.81	.	.	.	11.5	8.53	7.33	11.19	11.08	9.58	.	.	11.53	10.16	8.53	9.09	7.34	6.79	8.38	8.37	9.53	9.8	12.39
8.03	.	.	.	11.5	8.71	7.33	11.19	11.08	9.58	.	.	11.53	10.16	8.53	9.09	7.34	6.79	8.38	8.37	9.53	9.8	12.49
8.03	.	.	.	11.57	8.71	7.41	11.19	11.08	9.58	.	.	11.76	10.16	8.53	9.09	7.34	6.99	8.38	8.37	9.53	9.83	12.49
8.04	.	.	.	11.57	8.71	7.58	11.33	11.36	9.88	.	.	11.76	10.16	8.53	9.09	7.34	7.11	8.38	8.51	9.53	9.83	12.49
8.04	.	.	.	11.57	8.71	7.58	11.33	11.36	9.9	.	.	11.76	10.26	8.53	9.09	7.34	7.11	8.38	8.51	9.53	9.83	12.49
8.14	.	.	.	11.8	8.71	7.58	11.33	11.36	9.9	.	.	11.76	10.26	8.53	9.09	7.34	7.11	8.38	8.51	9.53	10.03	12.49
8.14	.	.	.	11.8	9.13	7.58	11.51	11.36	9.9	.	.	11.76	10.26	8.53	9.09	7.34	7.11	8.38	8.51	9.53	10.03	12.49
8.14	.	.	.	11.83	9.13	7.58	11.51	11.36	9.9	.	.	11.76	10.26	8.53	9.09	7.34	7.11	8.38	8.51	9.53	10.03	12.49
8.14	.	.	.	11.83	9.13	7.6	11.51	11.36	9.9	.	.	11.93	10.26	8.53	9.09	7.34	7.21	8.38	8.51	9.53	10.21	12.69
8.37	.	.	.	11.83	9.13	7.67	11.51	11.36	9.9	.	.	11.94	10.26	8.53	9.21	7.34	7.21	8.38	8.58	9.53	10.21	12.69
8.37	.	.	.	11.93	9.15	7.67	11.66	11.36	9.9	.	.	11.94	10.37	8.68	9.22	7.44	7.21	8.38	8.59	9.53	10.21	12.69
8.37	.	.	.	11.93	9.38	7.67	11.66	11.7	10.31	.	.	11.94	10.37	8.68	9.22	7.58	7.42	8.38	8.59	9.53	10.22	12.69
8.37	.	.	.	11.93	9.38	7.67	11.66	11.7	10.31	.	.	12.07	10.37	8.68	9.22	7.58	7.43	8.38	8.59	9.53	10.22	12.69
8.37	.	.	.	11.93	**9.38**	**7.67**	11.86	11.7	10.31	.	.	12.07	**10.37**	**8.68**	**9.22**	**7.58**	**7.43**	**8.38**	**8.59**	9.61	10.32	12.69

0	0	0	0	0	**0**	0.28	0	0	0	0	0	0	**0**	**0**	**0**	0.49	0.46	0.63	0.65	0	0	0
0	0.24	0	0.17	0.26	0.09	0.65	0.14	0.21	0	0.12	0.09	0.14	0.14	0.04	0.09	0.67	0.53	0.64	0.82	0.11	0	0.28
0.33	0.48	0.23	0.35	0.48	0.09	0.65	0.28	0.26	0.21	0.12	0.09	0.26	0.19	0.1	0.33	0.91	0.96	0.84	0.82	0.16	0	0.46
0.41	0.5	0.23	0.35	0.48	0.17	0.65	0.28	0.26	0.21	0.17	0.26	0.29	0.3	0.1	0.34	1.02	0.96	0.86	1.08	0.46	0	0.47
0.41	0.5	0.23	0.35	0.48	0.53	0.65	0.34	0.46	0.21	0.36	0.26	0.39	0.51	0.21	0.37	1.16	0.96	1.01	1.14	0.46	0	0.47
0.57	0.88	0.53	0.58	0.92	0.53	0.92	0.63	0.59	0.57	0.36	0.42	0.58	0.51	0.35	0.46	1.16	0.96	1.3	1.14	0.46	0.05	0.47
0.92	0.88	0.53	0.58	0.92	0.53	1.25	0.63	0.59	0.57	0.36	0.42	0.65	0.52	0.35	0.55	1.54	1.28	1.3	1.14	0.76	0.05	0.78
0.92	0.94	0.53	0.58	0.92	0.85	1.25	0.63	0.59	0.7	0.54	0.72	0.71	0.68	0.64	0.69	1.68	1.28	1.63	1.41	0.76	0.05	0.78
0.92	0.95	0.53	0.87	0.92	0.86	1.25	0.94	0.77	1.06	0.54	0.72	0.71	0.68	0.64	0.69	1.68	1.28	1.63	1.41	0.95	0.31	0.92
0.92	1.33	0.67	0.87	1.33	0.89	1.44	0.94	0.77	1.06	0.98	0.76	0.85	0.85	0.64	0.84	1.72	1.28	1.63	1.71	0.95	0.31	0.92
1	1.33	0.73	0.87	1.33	0.92	1.44	1.06	0.77	1.06	0.98	0.76	0.97	0.85	0.98	0.99	1.88	1.36	1.73	1.83	1.23	0.31	0.92
1.32	1.33	0.73	1.24	1.33	0.92	1.44	1.08	1.06	1.06	0.98	0.76	1.12	1.05	0.98	1.09	1.88	1.36	1.73	1.83	1.23	0.33	0.98
1.32	1.48	1.02	1.24	1.33	1.26	1.67	1.08	1.06	1.5	0.98	0.93	1.38	1.05	0.98	1.09	1.88	1.37	1.73	1.83	1.23	0.33	0.98
1.32	1.49	1.02	1.62	1.59	1.26	1.69	1.34	1.49	1.5	1.12	0.93	1.46	1.05	0.98	1.13	1.91	1.54	1.83	1.85	1.59	0.39	1.15
1.32	1.58	1.14	1.62	1.62	1.26	1.69	1.47	1.49	1.5	1.12	1.09	1.83	1.05	1.53	1.13	1.91	1.81	1.94	2.12	1.59	0.93	1.26
1.79	1.59	1.14	1.62	1.62	1.64	1.8	1.56	1.49	1.5	1.39	1.36	1.83	1.36	1.54	1.24	2.08	1.81	2	2.12	1.79	0.93	1.26
1.79	1.59	1.32	2.03	1.62	1.65	1.8	1.56	1.49	1.5	1.4	1.37	1.83	1.36	1.54	1.25	2.09	1.97	2	2.12	1.99	1.24	1.26
1.79	1.61	1.48	2.03	1.64	1.89	2.12	1.7	1.8	1.5	1.66	1.37	1.83	1.47	1.54	1.62	2.09	1.97	2.06	2.12	2.28	1.24	1.46
1.82	1.64	1.5	2.03	1.65	1.92	2.12	1.76	1.8	1.74	1.67	1.61	2.21	1.75	1.57	1.62	2.52	1.97	2.15	2.39	2.32	1.24	1.46
1.82	1.76	1.5	2.03	1.88	1.92	2.12	1.86	1.8	1.75	1.98	1.61	2.21	1.92	2.03	1.83	2.54	2.24	2.15	2.39	2.32	1.58	1.51
2.17	1.76	1.78	2.03	1.88	1.92	2.5	1.86	1.8	1.75	1.98	1.61	2.21	1.92	2.03	1.83	2.54	2.35	2.17	2.39	2.46	1.58	1.51
2.17	1.92	1.78	2.18	1.93	2.36	2.5	1.95	1.8	1.75	2.15	1.61	2.21	1.92	2.03	1.92	2.54	2.51	2.22	2.39	2.48	1.81	1.51
2.17	1.93	1.78	2.18	1.93	2.36	2.5	1.95	1.8	1.75	2.15	1.68	2.24	2.41	2.03	1.97	2.54	2.51	2.22	2.39	2.6	1.85	1.53
2.17	2.16	1.78	2.18	1.93	2.36	2.61	1.98	1.93	2.27	2.15	1.69	2.25	2.42	2.05	1.98	2.89	2.51	2.23	2.47	2.6	1.85	1.53
2.21	2.16	2.24	2.18	1.93	2.36	2.79	1.98	2.01	2.27	2.15	1.86	2.35	2.42	2.34	2.22	2.89	2.51	2.37	2.75	2.6	1.85	1.53
2.28	2.4	2.24	2.18	1.97	2.39	2.79	2.01	2.01	2.27	2.33	1.9	2.5	2.42	2.34	2.23	2.89	2.53	2.4	2.75	3.01	1.85	1.53
2.28	2.42	2.24	2.64	2.13	2.39	2.86	2.11	2.27	2.27	2.33	1.92	2.5	2.42	2.34	2.23	2.89	2.53	2.45	2.75	3.01	2.08	1.55
2.28	2.66	2.24	2.64	2.24	2.41	2.86	2.11	2.27	2.27	2.33	1.95	2.51	2.48	2.34	2.57	3.02	2.72	2.58	2.75	3.01	2.08	1.62
2.28	2.76	2.24	2.64	2.24	2.57	2.86	2.11	2.27	2.27	2.34	2.11	2.54	2.81	2.34	2.57	3.02	2.72	2.58	3.05	3.01	2.08	1.62
2.37	2.76	2.45	2.64	2.24	2.57	2.97	2.3	2.27	2.27	2.52	2.18	2.54	2.81	2.34	2.57	3.41	2.72	2.64	3.05	3.01	2.08	1.62
2.37	2.76	2.45	2.71	2.73	2.58	2.98	2.57	2.27	2.27	2.52	2.19	2.6	2.81	2.34	2.85	3.41	2.85	2.64	3.05	3.01	2.08	1.78
2.37	2.99	2.45	2.71	2.73	2.73	2.98	2.57	2.27	2.81	2.52	2.2	2.62	3.15	2.87	2.85	3.57	3.03	2.64	3.05	3.01	2.08	1.78
2.37	3.02	2.45	2.71	2.73	2.73	2.98	2.84	2.28	2.81	2.83	2.21	2.67	3.15	2.87	2.85	3.66	3.03	2.87	3.45	3.19	2.22	1.78
2.42	3.02	2.45	2.81	2.73	2.73	2.98	2.84	2.28	2.81	2.83	2.35	2.67	3.15	2.87	2.85	3.66	3.03	2.88	3.45	3.19	2.22	1.78
2.42	3.03	2.45	2.81	2.76	2.73	2.98	2.84	2.28	2.81	3.12	2.48	2.67	3.15	2.87	2.85	3.66	3.31	2.99	3.45	3.19	2.22	1.78
2.42	3.03	2.45	2.81	2.83	3.03	3.11	3.03	2.39	3.21	3.12	2.48	2.67	3.15	2.98	2.85	3.91	3.39	3.06	3.45	3.19	2.22	1.85
2.42	3.03	2.91	2.81	2.89	3.03	3.11	3.03	2.39	3.21	3.12	2.62	2.7	3.27	2.98	3.18	3.91	3.39	3.06	3.45	3.19	2.22	1.85
2.42	3.04	2.91	3.46	3.12	3.03	3.25	3.03	2.42	3.21	3.12	2.64	2.79	3.27	2.98	3.18	3.91	3.39	3.12	3.45	3.19	2.22	1.85
2.68	3.04	2.91	3.46	3.12	3.03	3.3	3.03	2.45	3.21	3.36	2.77	2.97	3.27	2.98	3.18	3.91	3.39	3.22	#VALUE!	3.19	2.22	1.85
2.68	3.04	2.91	3.47	3.12	3.15	3.48	3.03	2.59	3.46	3.36	2.77	2.97	3.27	2.98	3.23	3.91	3.49	3.22	#VALUE!	3.19	2.22	1.85
2.68	3.05	2.91	3.47	3.12	3.27	3.48	3.05	2.73	3.46	3.36	2.86	3.08	3.27	2.98	3.23	3.91	3.49	3.28	#VALUE!	3.19	2.22	1.85
2.68	3.08	2.91	3.47	3.12	3.27	3.57	3.05	2.73	3.46	3.52	2.87	3.17	3.27	2.98	3.28	3.91	3.49	3.28	#VALUE!	3.48	2.25	1.85
2.95	3.12	3.25	3.47	3.12	3.27	3.73	3.26	2.73	3.5	3.52	2.94	3.18	3.34	2.98	3.32	3.96	3.62	3.28	#VALUE!	3.48	2.27	1.85
2.95	3.19	3.25	3.51	3.12	3.27	3.76	3.28	3	3.5	3.52	3.07	3.18	3.34	2.98	3.32	4.21	3.73	3.28	#VALUE!	3.48	2.27	1.85
3.26	3.23	3.25	3.51	3.36	3.27	3.76	3.55	3.09	3.5	3.52	3.07	3.26	3.45	2.98	3.32	4.21	3.84	3.6	#VALUE!	3.48	2.27	1.85
3.28	3.29	3.25	3.51	3.84	3.55	3.87	3.55	3.09	3.5	3.52	3.07	3.43	3.45	2.98	3.32	4.21	3.84	3.6	#VALUE!	3.48	2.27	1.85
3.28	3.29	3.27	3.51	3.84	3.6	4.07	3.55	3.09	3.5	3.52	3.17	3.43	3.47	3.02	3.35	4.21	3.95	3.6	#VALUE!	3.48	2.27	1.85
3.28	3.32	3.27	3.76	3.84	3.6	4.07	3.86	3.2	3.5	3.52	3.17	3.46	3.65	3.1	3.36	4.21	3.95	3.6	#VALUE!	3.58	2.27	1.85
3.28	3.34	3.27	3.76	3.84	3.6	4.21	3.86	3.21	3.5	3.52	3.29	3.46	3.66	3.25	3.36	4.34	3.95	3.6	#VALUE!	3.58	2.27	1.85
3.5	3.34	3.27	3.76	3.84	3.6	4.21	3.86	3.21	3.5	3.52	3.37	3.52	3.66	3.25	3.54	4.34	3.95	3.6	#VALUE!	3.58	2.27	1.85
3.5	3.38	3.27	3.76	3.84	3.6	4.43	3.96	3.35	3.5	3.52	3.42	3.61	3.71	3.25	3.54	4.44	3.95	3.7	#VALUE!	3.83	2.33	1.85
3.5	3.38	3.27	3.8	3.84	3.6	4.43	4.1	3.35	3.5	3.52	3.46	3.61	3.71	3.25	3.54	4.44	3.96	3.71	#VALUE!	3.83	2.33	1.85
3.5	3.38	3.27	3.8	3.84	3.92	4.43	4.1	3.35	3.5	3.52	3.59	3.61	3.71	3.25	3.81	4.44	3.96	3.71	#VALUE!	3.83	2.33	1.85
3.52	3.67	3.27	3.89	4.15	3.92	4.43	4.1	3.35	4.12	3.52	3.63	3.61	3.71	3.42	3.81	4.45	3.96	3.71	#VALUE!	4.02	2.33	1.85

3.59	3.67	3.27	3.89	4.25	3.92	4.48	4.29	3.35	4.12	3.55	3.68	3.61	3.71	3.42	3.9	4.45	3.97	3.76	#VALUE!	4.03	2.33	1.85
3.84	3.67	3.27	4	4.25	4.31	4.48	4.29	3.67	4.12	3.55	3.68	3.61	4	3.42	3.92	4.45	3.97	3.76	#VALUE!	4.03	2.33	1.85
3.84	3.8	3.27	4	4.25	4.31	4.48	4.29	3.67	4.12	3.55	3.73	3.61	4	3.42	4.18	4.65	3.98	3.76	#VALUE!	4.34	2.33	1.85
3.85	3.8	3.58	4.23	4.25	4.31	4.62	4.29	3.7	4.12	3.56	3.73	3.61	4	3.49	4.18	4.65	3.98	3.76	#VALUE!	4.34	2.33	1.85
3.86	3.8	3.58	4.26	4.25	4.31	4.62	4.43	3.7	4.12	3.56	3.96	3.61	4.05	3.49	4.22	4.65	3.98	3.76	#VALUE!	4.34	2.33	1.85
3.88	3.8	3.58	4.26	4.25	4.53	4.62	4.43	3.7	4.12	3.57	3.96	3.62	4.05	3.54	4.43	4.65	3.98	3.76	#VALUE!	4.34	2.33	1.85
3.97	3.92	3.62	4.26	4.25	4.57	4.87	4.43	3.99	4.12	3.57	3.98	3.62	4.05	3.54	4.43	4.65	4.03	3.77	#VALUE!	4.34	2.33	1.85
3.97	3.92	3.62	4.26	4.25	4.57	4.87	4.58	3.99	4.12	3.77	3.98	3.62	4.07	3.54	4.43	4.81	4.03	3.77	#VALUE!	4.34	2.33	1.85
3.97	3.92	3.62	4.28	4.25	4.57	4.87	4.58	3.99	4.12	3.77	4.16	3.62	4.34	3.54	4.43	4.81	4.03	3.87	#VALUE!	4.58	2.33	1.85
4.06	3.92	3.62	4.28	4.56	4.57	4.87	4.58	3.99	4.86	3.77	4.16	3.62	4.34	3.54	4.73	4.81	4.03	3.87	#VALUE!	4.58	2.33	1.85
4.07	4.14	3.62	4.29	4.56	4.57	4.87	4.58	4.07	4.86	3.78	4.16	3.62	4.34	3.59	4.73	4.81	4.03	4.1	#VALUE!	4.58	2.33	1.85
4.07	4.14	3.62	4.64	4.56	4.57	4.92	4.79	4.31	4.86	3.78	4.49	3.62	4.34	3.59	4.73	4.81	4.03	4.1	#VALUE!	4.82	2.6	1.85
4.32	4.14	3.62	4.64	4.85	4.63	4.92	4.79	4.31	4.86	3.78	4.49	3.62	4.48	3.76	4.91	4.82	4.03	4.1	#VALUE!	4.82	2.66	1.85
4.39	4.32	3.62	4.64	4.85	4.63	4.92	4.79	4.31	5.03	3.81	4.49	3.63	4.48	3.77	4.91	4.91	4.09	4.1	#VALUE!	4.82	2.66	1.85
4.39	4.32	3.89	4.65	4.86	4.64	4.98	4.94	4.59	5.03	4.02	4.5	3.63	4.49	3.77	4.91	5.02	4.09	4.49	#VALUE!	4.82	2.66	1.85
4.74	4.33	3.89	4.65	4.86	4.64	4.98	4.94	4.59	5.03	4.02	4.51	3.93	4.56	3.77	4.92	5.02	4.09	4.49	#VALUE!	4.98	2.66	1.85
4.74	4.33	3.89	4.77	4.86	4.73	4.98	5.02	4.59	5.03	4.02	4.51	3.93	4.56	4.04	4.97	5.18	4.62	4.49	#VALUE!	4.98	2.66	1.85
4.75	4.54	3.89	4.78	4.86	4.77	5.13	5.02	4.92	5.03	4.24	4.53	3.93	4.58	4.3	4.97	5.32	4.62	4.49	#VALUE!	5.23	3.28	2.15
4.76	4.54	3.89	4.78	4.86	4.8	5.13	5.02	4.92	5.03	4.33	4.63	3.93	5.17	4.3	5.01	5.32	4.62	4.83	#VALUE!	5.23	3.28	2.15
4.78	4.81	3.89	4.78	4.94	4.8	5.13	5.17	4.92	5.03	4.68	4.81	3.93	5.17	4.53	5.15	5.32	4.62	4.84	#VALUE!	5.23	3.28	2.15
4.9	4.92	3.9	4.79	5.4	4.82	5.13	5.17	4.92	5.58	4.69	4.81	4.09	5.17	4.53	5.15	5.8	4.67	4.84	#VALUE!	5.53	3.28	2.15
5.45	4.92	3.99	5.29	5.4	4.9	5.13	5.19	4.92	5.58	4.69	4.82	4.09	5.17	4.54	5.28	5.8	4.73	5.28	#VALUE!	5.53	3.28	2.15
5.45	4.92	4.19	5.3	5.4	4.95	5.42	5.39	4.92	5.58	4.7	4.82	4.09	5.17	4.55	5.29	5.8	5.04	5.28	#VALUE!	5.53	3.28	2.41
5.45	4.92	4.19	5.3	5.4	4.95	5.43	5.39	5.31	5.86	5.08	4.83	4.09	5.17	4.92	5.57	5.8	5.04	5.28	#VALUE!	5.53	3.28	2.76
5.45	5.17	4.72	5.3	5.4	5.11	5.43	5.39	5.66	6.09	5.08	4.93	4.63	5.17	4.94	5.68	5.8	5.31	5.46	#VALUE!	5.53	3.3	2.8
5.51	5.17	4.72	5.3	5.4	5.11	5.43	5.54	5.66	6.09	5.08	5	4.64	5.17	5.1	5.79	5.81	5.31	5.49	#VALUE!	5.53	3.85	2.8
5.82	5.48	5.08	5.3	5.8	5.12	5.53	5.54	5.66	6.09	5.09	5.12	4.8	5.58	5.1	5.89	5.81	5.64	5.49	#VALUE!	5.75	3.86	3.37
5.83	5.5	5.08	5.77	5.82	5.12	5.6	5.54	5.66	6.09	5.09	5.13	4.83	5.59	5.1	5.91	5.96	5.65	5.55	#VALUE!	5.83	3.86	3.51
5.83	5.5	5.08	5.77	6.33	5.13	5.6	5.83	5.66	6.09	5.37	5.3	5.3	5.59	5.1	5.91	5.99	5.97	5.55	#VALUE!	6.21	3.86	3.98
5.83	6.04	5.08	6.19	6.36	5.24	5.96	5.98	5.66	6.09	5.37	5.52	5.31	5.61	5.1	5.91	5.99	5.97	5.59	#VALUE!	6.21	3.86	3.98
5.83	6.05	5.08	6.19	6.36	5.28	5.97	5.98	5.68	6.56	5.47	5.59	5.31	5.64	5.12	5.92	6.29	6.45	5.78	#VALUE!	6.22	3.86	3.98
5.98	6.05	5.08	6.2	6.36	5.43	6.02	5.98	6.07	6.56	5.71	5.7	5.93	5.64	5.12	5.92	6.29	6.45	5.79	#VALUE!	6.22	3.86	4.34
5.98	6.05	5.28	6.21	6.68	5.43	6.16	6	6.07	6.56	5.71	5.72	5.93	5.7	5.12	6.15	6.3	6.46	5.79	#VALUE!	6.22	3.86	4.34
5.98	6.05	5.32	6.21	6.69	5.43	6.16	6	6.07	6.56	6.02	5.86	5.93	5.73	5.12	6.17	6.5	6.46	5.95	#VALUE!	6.26	3.86	4.34
6.09	6.05	5.32	6.63	6.69	5.68	6.16	6.22	6.37	6.56	6.02	5.99	5.93	6.01	5.39	6.17	6.5	6.51	5.96	#VALUE!	6.34	3.86	4.34
6.1	6.05	5.32	6.63	6.69	5.68	6.35	6.22	6.37	6.56	6.15	5.99	5.93	6.15	5.39	6.19	6.5	6.51	5.96	#VALUE!	6.35	3.86	4.34
6.1	6.06	5.32	6.63	6.69	5.79	6.64	6.29	6.37	6.56	6.39	6.02	5.94	6.15	5.39	6.19	6.65	6.51	5.96	#VALUE!	6.36	4.15	4.34
6.1	6.06	5.6	6.63	6.69	5.84	6.64	6.29	6.37	7.02	6.39	6.15	5.95	6.15	5.39	6.64	7	6.51	6.14	#VALUE!	6.36	4.23	4.34
6.1	6.06	5.6	6.63	6.85	5.84	6.64	6.31	6.37	7.02	6.74	6.19	5.95	6.15	5.39	6.65	7.03	6.57	6.24	#VALUE!	6.36	4.23	4.34
6.1	6.06	5.6	6.63	6.85	5.93	6.64	6.31	6.37	7.02	6.74	6.3	5.96	6.15	5.39	6.67	7.03	6.74	6.35	#VALUE!	6.38	4.23	4.34
6.24	6.13	5.6	6.63	6.85	5.94	6.96	6.36	6.37	7.02	6.74	6.32	6.28	6.67	5.39	6.7	7.33	6.82	6.5	#VALUE!	6.38	4.23	4.34
6.24	6.13	5.6	6.63	6.85	6.16	7.09	6.43	6.37	7.02	6.74	6.32	6.33	6.67	5.66	6.7	7.33	6.82	6.5	#VALUE!	6.39	4.57	4.34
6.24	6.37	5.79	6.82	6.85	6.16	7.09	6.43	6.6	7.02	7	6.32	6.33	6.67	5.66	6.83	7.33	6.82	6.5	#VALUE!	6.55	4.57	4.34
6.3	6.43	5.79	7.07	6.85	6.16	7.09	6.43	7.01	7.02	7	6.45	6.33	6.93	5.71	6.84	7.49	7.34	6.51	#VALUE!	6.55	4.57	4.34
6.43	6.43	5.86	7.07	6.85	6.21	7.09	6.62	7.01	7.02	7.35	6.52	6.33	6.94	5.71	6.99	7.83	7.34	6.94	#VALUE!	6.55	4.57	4.34
6.43	6.77	5.87	7.07	7.21	6.25	7.09	6.62	7.01	7.02	7.36	6.55	6.33	6.94	5.71	7.02	7.83	7.34	6.94	#VALUE!	6.55	4.57	4.65
6.43	6.77	5.87	7.07	7.21	6.39	7.11	6.62	7.01	7.03	7.36	6.55	6.73	7.1	5.74	7.02	7.83	7.34	6.94	#VALUE!	6.55	4.57	4.65
6.6	6.77	6.19	7.07	7.21	6.39	7.11	6.62	7.01	7.77	7.36	6.78	6.73	7.1	5.76	7.02	7.83	7.59	6.94	#VALUE!	6.55	4.57	4.65
6.6	6.77	6.19	7.07	7.21	6.4	7.38	6.63	7.01	7.77	7.65	6.78	6.73	7.1	5.79	7.12	7.83	7.73	6.94	#VALUE!	6.95	4.57	4.65
6.6	7.2	6.19	7.07	7.21	6.54	7.38	6.63	7.01	7.77	7.65	6.81	7.09	7.12	5.79	7.12	8.33	7.73	7	#VALUE!	7	4.92	4.65
6.6	7.2	6.28	7.24	7.8	6.55	7.38	6.63	7.2	7.77	7.65	6.84	7.1	7.12	5.86	7.43	8.33	7.83	7	#VALUE!	7	4.92	4.67
6.72	7.21	6.28	7.25	7.8	6.55	7.44	6.82	7.2	7.77	7.71	6.84	7.1	7.12	5.95	7.43	8.33	7.83	7.09	#VALUE!	7	4.92	4.67
6.72	7.21	6.57	7.25	7.8	6.56	7.44	6.93	7.2	7.77	7.71	6.84	7.1	7.69	6.03	7.6	8.33	7.83	7.09	#VALUE!	7	4.92	4.67
6.76	7.21	6.57	7.25	7.82	6.79	7.44	6.93	7.2	8.03	7.71	7.02	7.1	7.69	6.03	7.6	8.33	7.83	7.09	#VALUE!	7.47	4.92	4.67
6.85	7.21	6.57	7.39	7.82	6.94	7.44	6.97	7.2	8.03	7.71	7.02	7.1	7.69	6.32	7.6	8.33	7.83	7.09	#VALUE!	7.47	4.92	4.67
7.18	7.48	6.57	7.48	8.11	7.35	7.9	6.97	7.44	8.03	7.89	7.09	7.58	7.69	6.32	7.76	8.33	7.88	7.11	#VALUE!	7.47	5.28	4.67
7.18	7.49	6.57	7.48	8.11	7.35	7.9	7.09	7.48	8.03	7.89	7.09	7.58	8.08	6.38	7.76	8.44	8.16	7.13	#VALUE!	7.47	5.28	4.67
7.18	7.49	6.87	7.48	8.11	8.01	7.9	7.11	7.48	8.03	7.9	7.09	7.58	8.08	6.51	8.06	8.44	8.16	7.37	#VALUE!	7.47	5.28	4.67
7.6	7.49	6.87	7.48	8.11	8.01	7.9	7.13	7.48	8.03	7.9	7.09	7.58	8.08	6.52	8.36	8.44	8.16	7.37	#VALUE!	7.47	5.28	4.67
7.6	7.49	6.87	7.48	8.11	8.01	7.9	7.13	7.48	8.03	7.9	7.39	7.58	8.09	6.52	8.36	8.44	8.16	7.37	#VALUE!	7.47	5.28	4.67
7.6	7.49	6.87	7.48	8.11	8.01	8.14	7.21	7.48	8.03	7.9	7.39	7.58	8.09	6.63	8.36	8.91	8.35	7.41	#VALUE!	7.86	5.46	4.67
7.61	7.49	6.88	7.53	8.49	8.01	8.28	7.21	7.48	8.03	7.9	7.53	7.58	8.09	6.93	8.36	8.91	8.38	7.41	#VALUE!	7.86	5.46	4.67
7.62	7.49	6.88	7.53	8.49	8.01	8.28	7.53	7.48	8.03	7.9	7.53	7.58	8.41	6.93	8.36	8.91	8.38	7.41	#VALUE!	7.87	5.46	4.67
7.84	7.49	6.88	7.53	8.49	8.01	8.28	7.53	7.48	8.03	7.9	7.56	7.58	8.41	6.93	8.36	8.91	8.38	7.5	#VALUE!	8.26	5.46	4.67
7.84	7.49	7.35	7.53	8.49	8.01	8.48	7.57	7.52	8.1	8.07	7.56	7.58	8.41	6.93	8.37	8.91	8.38	7.5	#VALUE!	8.3	5.55	4.8
7.84	7.49	7.35	7.69	8.49	8.01	8.56	7.57	7.52	8.1	8.07	7.62	7.58	8.41	6.93	8.37	8.96	8.38	7.5	#VALUE!	8.3	5.55	4.8
7.84	7.51	7.35	7.69	8.49	8.01	8.56	7.86	7.52	8.1	8.07	7.63	7.58	8.41	6.93	8.37	8.96	8.38	7.5	#VALUE!	8.3	5.55	4.8
8.14	7.51	7.35	7.69	8.49	8.01	8.56	7.86	7.52	8.1	8.07	7.82	7.6	8.44	6.93	8.42	9.28	8.38	7.5	#VALUE!	8.46	5.88	4.81
8.14	7.51	7.35	7.7	8.97	8.01	8.56	7.86	7.52	8.1	8.07	7.82	7.6	8.44	6.93	8.42	9.28	8.4	7.5	#VALUE!	8.48	5.88	4.81
8.14	7.51	7.35	7.71	9.12	8.09	8.56	7.96	7.52	8.1	8.07	7.82	7.6	8.44	6.93	8.49	9.28	8.4	7.74	#VALUE!	8.48	5.88	4.81
8.2	7.51	7.36	7.72	9.12	8.09	8.56	7.96	7.52	8.1	8.07	7.95	7.66	8.55	6.93	8.49	9.29	8.4	7.74	#VALUE!	8.48	5.89	4.81
8.2	7.51	.	.	9.12	8.09	8.76	8	7.52	8.1	.	.	7.67	8.55	6.93	8.49	9.29	8.4	7.74	#VALUE!	8.48	5.89	4.81
8.2	7.51	.	.	9.12	8.22	8.76	8	7.52	8.1	.	.	7.67	8.55	7.06	8.49	9.29	8.4	8.07	#VALUE!	8.48	5.89	4.81
8.42	7.51	.	.	9.12	8.23	8.76	8	7.52	8.1	.	.	7.71	8.55	7.06	8.49	9.29	8.4	8.09	#VALUE!	8.49	5.89	4.81
8.42	7.51	.	.	9.12	8.23	8.76	8	7.55	8.1	.	.	7.71	8.55	7.06	8.49	9.29	8.4	8.09	#VALUE!	8.68	5.89	5.04
8.42	7.52	.	.	9.12	8.23	8.76	8.25	7.55	8.1	.	.	7.79	8.68	7.36	8.58	9.29	8.4	8.09	#VALUE!	8.76	6.05	5.05
8.42	.	.	.	9.12	8.27	8.89	8.25	7.55	8.1	.	.	7.79	8.68	7.36	8.58	9.56	8.4	8.09	#VALUE!	8.76	6.05	5.05
8.42	.	.	.	9.12	8.27	8.89	8.25	7.56	8.14	.	.	7.86	8.68	7.36	8.58	9.56	8.41	8.09	#VALUE!	8.76	6.05	5.05
8.42	.	.	.	9.12	8.27	8.89	8.25	7.56	8.34	.	.	7.86	8.68	7.5	8.62	9.56	8.41	8.28	#VALUE!	8.76	6.07	5.1
8.89	.	.	.	9.12	8.27	8.9	8.35	7.56	8.34	.	.	7.99	8.69	7.51	8.62	9.56	8.41	8.28	#VALUE!	8.9	6.07	5.1
9.1	.	.	.	9.21	8.28	8.9	8.36	7.77	8.89	.	.	7.99	8.69	7.74	8.83	9.56	8.41	8.28	#VALUE!	8.9	6.07	5.1
9.1	.	.	.	9.43	8.44	8.9	8.36	8.11	9.02	.	.	8	8.69	7.74	8.83	9.77	8.41	8.28	#VALUE!	8.91	6.15	5.1
9.1	.	.	.	9.43	8.44	8.9	8.66	8.11	9.02	.	.	8.03	8.69	7.74	8.83	9.77	8.41	8.28	#VALUE!	9.25	6.48	5.1
9.1	.	.	.	9.43	8.49	8.9	8.66	8.58	9.02	.	.	8.04	8.83	7.74	9	9.77	8.76	8.28	#VALUE!	9.43	6.48	5.39
9.54	.	.	.	9.43	8.49	9.43	8.66	8.58	9.83	.	.	8.17	8.84	7.74	9	9.77	8.83	8.28	#VALUE!	9.43	6.49	5.39
9.54	.	.	.	9.43	8.82	9.43	8.91	8.58	9.83	.	.	8.17	8.84	7.78	9	9.79	8.83	8.39	#VALUE!	9.43	6.49	5.39
9.54	.	.	.	9.43	8.82	9.43	8.91	8.69	9.83	.	.	8.18	8.94	7.78	9.07	9.79	9.23	8.45	#VALUE!	9.44	6.49	5.39
9.77	.	.	.	9.59	8.82	9.43	8.91	9.11	9.83	.	.	8.19	9.08	7.78	9.07	9.79	9.23	8.45	#VALUE!	9.45	6.49	5.84
9.77	.	.	.	9.6	8.82	9.43	8.91	9.11	9.83	.	.	8.2	9.08	7.86	9.56	9.84	9.23	8.58	#VALUE!	9.45	6.49	5.84
9.77	.	.	.	9.6	8.83	9.43	9	9.11	9.83	.	.	8.35	9.08	7.86	9.56	9.84	9.24	9.28	#VALUE!	9.45	6.88	5.84
9.77	.	.	.	9.6	9.15	9.43	9.44	9.11	9.83	.	.	8.35	9.08	7.94	9.56	9.86	9.31	9.29	#VALUE!	9.54	7.32	5.94
9.77	.	.	.	9.94	9.15	9.43	9.44	9.11	9.89	.	.	8.38	9.09	8.05	9.72	10.15	10.11	9.29	#VALUE!	9.55	7.32	6.25
9.79	.	.	.	9.94	9.23	9.43	9.44	9.11	9.89	.	.	8.49	9.1	8.05	9.91	10.16	10.12	9.29	#VALUE!	10.07	7.34	6.35
9.79	.	.	.	9.97	9.24	9.99	9.76	9.61	9.89	.	.	8.5	9.22	8.05	9.91	10.41	10.24	9.75	#VALUE!	10.07	7.36	6.38
9.98	.	.	.	10.22	9.46	10	9.76	9.65	10.63	.	.	8.56	9.4	8.14	9.91	10.41	10.52	9.93	#VALUE!	10.1	7.36	6.85
10.31	.	.	.	10.35	9.5	10	9.76	9.65	10.63	.	.	8.69	9.4	8.14	9.92	10.58	10.52	9.93	#VALUE!	10.1	7.36	6.87
10.31	.	.	.	10.39	9.69	10	9.76	9.65	10.63	.	.	8.73	9.69	8.51	9.92	10.6	10.52	9.93	#VALUE!	10.15	7.67	6.87
10.31	.	.	.	10.51	9.75	10.36	9.76	9.65	10.63	.	.	8.94	9.69	8.51	10.35	10.61	10.52	9.93	#VALUE!	10.15	7.67	6.87
10.31	.	.	.	10.51	9.76	10.38	9.76	9.65	10.63	.	.	8.94	9.69	8.51	10.35	10.61	10.52	9.93	#VALUE!	10.28	7.67	6.87
10.31	.	.	.	10.51	9.76	10.38	9.76	9.68	10.88	.	.	9.04	9.69	8.51	10.35	10.8	10.52	9.93	#VALUE!	10.3	7.67	6.87
10.31	.	.	.	10.52	9.8	10.38	9.95	9.77	10.88	.	.	9.04	9.81	8.51	10.35	10.81	10.52	9.94	#VALUE!	10.32	7.67	6.87
10.31	.	.	.	10.71	9.81	10.38	9.95	9.77	10.88	.	.	9.04	9.95	8.51	10.35	10.81	10.52	9.94	#VALUE!	10.32	7.73	6.87
10.31	.	.	.	10.71	10	10.38	9.95	9.77	10.88	.	.	9.16	10.43	8.51	10.35	10.81	10.82	10.27	#VALUE!	10.32	7.73	6.87
10.31	.	.	.	10.71	10.31	10.38	9.95	9.77	10.88	.	.	9.16	10.43	8.51	10.39	10.95	10.83	10.27	#VALUE!	10.32	7.73	7.23
10.31	.	.	.	10.71	10.31	10.61	10.02	9.77	10.88	.	.	9.16	10.43	8.66	10.39	11.08	10.83	10.27	#VALUE!	10.32	7.73	7.23
10.31	.	.	.	10.71	10.34	10.63	10.02	9.77	10.88	.	.	9.47	10.43	8.66	10.39	11.19	10.83	10.31	#VALUE!	10.47	7.73	7.23
10.31	.	.	.	10.71	10.34	10.63	10.06	9.77	10.88	.	.	9.65	10.43	9.23	10.39	11.29	10.83	10.33	#VALUE!	10.48	8.08	7.23
10.31	.	.	.	10.71	10.35	10.63	10.16	9.77	10.88	.	.	9.65	10.43	9.23	10.54	11.32	11.03	10.34	#VALUE!	10.48	8.08	7.65
10.73	.	.	.	10.71	10.35	10.63	10.18	10.02	10.88	.	.	9.65	10.43	9.23	10.54	11.32	11.03	10.34	#VALUE!	10.48	8.08	7.65
10.75	.	.	.	10.87	10.54	10.63	10.18	10.02	10.88	.	.	10.01	10.43	9.48	10.54	11.32	11.03	10.74	#VALUE!	10.48	8.08	7.65
10.75	.	.	.	10.87	10.58	10.83	10.41	10.02	10.88	.	.	10.01	10.44	9.48	10.54	11.32	11.03	10.74	#VALUE!	10.48	8.08	7.65
10.75	.	.	.	10.87	10.97	10.84	10.41	10.02	10.88	.	.	10.01	10.44	9.48	10.68	11.49	11.03	10.74	#VALUE!	10.48	8.08	7.65
10.75	.	.	.	10.87	11.05	10.96	10.41	10.02	10.99	.	.	10.01	10.89	9.48	10.68	11.71	11.18	10.74	#VALUE!	10.54	8.08	7.65
10.8	.	.	.	10.87	11.42	10.96	10.41	10.21	10.99	.	.	10.01	11.16	9.48	10.68	11.71	11.38	10.74	#VALUE!	10.8	8.08	7.65
10.8	.	.	.	10.87	11.42	11	10.41	10.21	10.99	.	.	10.38	11.16	9.48	10.85	11.71	11.38	10.75	#VALUE!	10.8	8.1	7.65
10.8	.	.	.	10.88	11.42	11	10.41	10.21	10.99	.	.	10.38	11.16	9.48	10.85	11.71	11.38	11.08	#VALUE!	10.8	8.1	7.65
11.2	.	.	.	10.88	11.43	11	10.46	10.21	11.48	.	.	10.38	11.16	9.65	10.85	11.73	11.38	11.12	#VALUE!	10.8	8.42	7.66
11.2	.	.	.	10.9	11.43	11.01	10.47	10.21	11.49	.	.	10.38	11.16	10.28	10.86	11.73	11.58	11.12	#VALUE!	10.8	8.42	7.72
11.2	.	.	.	11.2	11.43	11.01	10.47	10.21	11.49	.	.	10.38	11.16	10.28	10.86	12.26	11.6	11.12	#VALUE!	10.8	8.42	8.12
11.2	.	.	.	11.21	11.71	11.06	10.47	10.3	11.49	.	.	10.38	11.19	10.57	11.07	12.36	11.6	11.12	#VALUE!	10.81	8.44	8.12
11.21	.	.	.	11.21	11.71	11.06	10.74	10.3	11.49	.	.	10.54	11.19	10.57	11.07	12.36	11.6	11.16	#VALUE!	10.83	8.46	8.12

11.35	.	.	.	11.74	11.72	11.06	10.74	10.3	11.49	.	.	10.57	11.19	10.57	11.27	12.36	11.6	11.19	#VALUE!	10.86	8.46	8.12
11.35	.	.	.	11.74	11.73	11.22	10.74	10.3	11.49	.	.	10.57	11.19	10.57	11.27	12.77	11.6	11.23	#VALUE!	10.86	8.46	8.12
11.35	.	.	.	11.74	11.73	11.32	10.87	10.3	11.49	.	.	10.57	11.57	10.57	11.28	12.77	11.6	11.23	#VALUE!	10.86	8.86	8.12
11.35	.	.	.	11.74	11.73	11.32	10.87	10.3	11.49	.	.	10.57	11.57	10.57	11.28	12.77	12.07	11.41	#VALUE!	11.37	8.86	8.12
11.56	.	.	.	11.74	11.87	11.32	10.87	10.3	11.49	.	.	10.57	11.57	10.57	11.28	12.78	12.07	11.41	#VALUE!	11.57	8.86	8.12
11.56	.	.	.	11.74	11.87	11.51	10.89	10.3	11.57	.	.	10.57	11.57	10.57	11.28	12.78	12.07	11.41	#VALUE!	11.59	8.86	8.12
11.67	.	.	.	12.07	11.87	11.51	10.91	10.3	12.11	.	.	10.57	11.57	10.57	11.28	12.78	12.07	11.41	#VALUE!	11.59	8.86	8.12
11.67	.	.	.	12.07	11.9	11.51	10.91	10.49	12.11	.	.	10.57	11.57	10.57	11.72	12.8	12.15	11.41	#VALUE!	11.59	8.86	8.12
11.67	.	.	.	12.08	11.9	11.51	11	10.49	12.11	.	.	10.57	11.57	10.59	11.72	12.8	12.15	11.53	#VALUE!	11.59	8.88	8.12
11.96	.	.	.	12.08	11.93	11.52	11	10.49	12.11	.	.	10.57	11.57	10.63	11.72	12.8	12.15	11.53	#VALUE!	11.59	8.88	8.57
11.96	.	.	.	12.08	11.99	11.52	11	10.49	12.11	.	.	10.57	11.57	10.63	11.72	12.8	12.15	11.53	#VALUE!	11.59	8.88	8.62
11.96	.	.	.	12.08	11.99	11.6	11.12	10.63	12.11	.	.	10.57	11.57	10.63	11.72	13.01	12.15	11.53	#VALUE!	11.74	9.08	8.62
11.96	.	.	.	12.08	11.99	11.62	11.12	10.63	12.11	.	.	10.99	11.57	10.63	12.02	13.01	12.35	11.53	#VALUE!	11.78	9.08	8.62
11.96	.	.	.	12.08	12.17	11.62	11.12	10.63	12.11	.	.	10.99	11.57	10.63	12.02	13.01	12.35	11.53	#VALUE!	11.81	9.08	8.62
11.96	.	.	.	12.08	12.18	11.62	11.12	10.63	12.11	.	.	10.99	11.57	10.63	12.02	13.01	12.35	11.53	#VALUE!	11.81	9.08	8.62
0	0	0	0	0	0	0	0	0	0	0	0	0	0	0	0	0	0	0	0	0	0	0
0.19	0.12	0.17	0	0.18	0.19	0	0.01	0	0	0.15	0.19	0.05	0.01	0	0	0	0.12	0.09	0.04	0	0.1	0.01
0.19	0.21	0.17	0.24	0.18	0.31	0.08	0.13	0.11	0	0.15	0.37	0.2	0.01	0	0.06	0.1	0.12	0.09	0.04	0.03	0.25	0.01
0.24	0.22	0.19	0.38	0.39	0.52	0.08	0.23	0.13	0.01	0.34	0.44	0.22	0.24	0	0.23	0.26	0.53	0.12	0.04	0.27	0.25	0.01
0.46	0.35	0.19	0.38	0.39	0.52	0.23	0.23	0.25	0.01	0.34	0.44	0.58	0.24	0	0.24	0.47	0.53	0.37	0.17	0.32	0.55	0.17
0.65	0.35	0.19	0.39	0.67	0.62	0.23	0.23	0.25	0.01	0.34	0.44	0.58	0.24	0.18	0.37	0.54	0.53	0.37	0.17	0.5	0.56	0.17
0.65	0.47	0.19	0.39	0.67	0.62	0.32	0.48	0.25	0.01	0.61	0.44	0.58	0.24	0.31	0.37	0.54	0.53	0.6	0.53	0.61	0.56	0.17
0.79	0.48	0.26	0.58	0.67	0.74	0.46	0.52	0.4	0.02	0.61	0.44	0.73	0.44	0.31	0.5	0.57	0.64	0.6	0.53	0.61	0.56	0.41
0.79	0.67	0.35	0.58	0.67	0.74	0.46	0.63	0.5	0.35	0.61	0.77	0.73	0.44	0.62	0.5	0.66	0.71	0.68	0.53	0.7	0.66	0.47
0.8	0.67	0.37	0.72	0.67	0.97	0.46	0.63	0.5	0.35	0.61	0.77	0.73	0.77	0.62	0.58	0.66	0.71	0.68	0.53	0.71	0.66	0.56
0.94	0.67	0.37	0.72	0.67	1.03	0.63	0.91	0.8	0.35	0.61	0.77	0.73	0.77	0.63	0.58	0.66	0.71	0.69	0.53	0.78	0.66	0.6
0.94	0.88	0.48	0.9	0.67	1.03	0.63	0.91	0.82	0.76	0.78	0.77	0.83	0.77	0.72	0.58	0.66	0.76	0.69	0.53	0.78	0.85	0.6
0.95	0.91	0.56	0.91	0.8	1.04	0.63	0.91	0.82	0.76	0.92	0.77	0.96	0.98	0.94	0.73	0.81	0.81	0.69	0.68	0.78	0.86	0.7
1.23	1.14	0.61	1.04	0.98	1.04	0.85	0.91	1.03	0.76	0.92	0.95	0.99	0.99	0.94	0.73	0.82	1.28	0.83	0.73	0.81	0.94	0.71
1.23	1.14	0.61	1.27	0.99	1.41	0.85	0.93	1.04	0.98	0.92	0.95	1.06	1.05	0.94	0.86	1.19	1.28	0.99	0.81	0.87	1.15	0.91
1.23	1.6	0.66	1.27	1.3	1.41	0.85	1.12	1.04	1.36	1.12	1	1.11	1.24	0.94	0.86	1.19	1.28	1	0.81	0.98	1.16	1.03
1.52	1.6	0.66	1.6	1.3	1.41	1.11	1.12	1.04	1.36	1.36	1	1.25	1.24	1.07	0.86	1.33	1.28	1	0.81	1.09	1.34	1.12
1.52	1.6	0.88	1.6	1.31	1.6	1.11	1.44	1.27	1.36	1.36	1	1.46	1.28	1.07	0.87	1.33	1.33	1.22	1.28	1.12	1.54	1.21
1.52	1.6	0.98	1.6	1.31	1.6	1.22	1.44	1.27	1.36	1.38	1.23	1.47	1.47	1.07	1.06	1.69	1.64	1.22	1.28	1.35	1.54	1.24
1.78	1.6	1.2	1.6	1.48	1.6	1.22	1.44	1.27	1.59	1.38	1.23	1.47	1.48	1.07	1.06	1.69	1.64	1.3	1.28	1.36	1.55	1.6
1.78	1.6	1.21	1.6	1.48	1.74	1.41	1.61	1.27	1.84	1.51	1.23	1.62	1.68	1.15	1.06	1.69	1.92	1.45	1.28	1.36	1.66	1.64
1.78	1.6	1.23	1.86	1.48	1.74	1.43	1.62	1.58	1.84	1.65	1.52	1.71	1.68	1.27	1.19	1.77	2	1.45	1.5	1.38	1.71	1.64
1.78	1.6	1.23	2.06	1.64	1.74	1.43	1.62	1.58	1.84	1.65	1.52	1.71	1.68	1.37	1.19	1.78	2	1.45	1.5	1.42	1.71	1.64
1.79	1.82	1.4	2.06	1.64	1.77	1.43	1.62	1.58	1.84	1.65	1.52	1.71	1.68	1.37	1.28	1.78	2	1.45	1.5	1.47	1.71	1.66
1.8	1.82	1.4	2.06	1.65	2.08	1.43	1.78	1.58	1.85	1.65	1.52	1.8	2.02	1.37	1.28	1.83	2	1.46	1.5	1.49	1.71	1.66
1.8	1.82	1.44	2.06	1.65	2.08	1.43	1.78	1.58	1.85	1.65	1.52	1.88	2.03	1.44	1.28	1.83	2	1.46	1.72	1.63	1.71	1.66
1.97	1.82	1.44	2.06	1.65	2.08	1.55	1.78	1.88	1.92	1.91	1.58	2.02	2.03	1.5	1.41	1.93	2.02	1.46	1.73	1.67	1.71	1.87
2.29	2	1.66	2.06	1.94	2.08	1.55	1.99	1.88	2.18	1.91	1.71	2.04	2.04	1.52	1.41	2.01	2.02	1.61	1.73	1.67	1.94	1.88
2.29	2	1.66	2.26	1.94	2.08	1.55	1.99	1.88	2.18	1.91	1.72	2.09	2.04	1.52	1.64	2.01	2.3	1.88	1.73	1.79	1.95	1.88
2.29	2	1.69	2.33	1.94	2.09	1.55	1.99	1.88	2.18	1.91	1.72	2.1	2.23	1.52	1.64	2.01	2.36	1.88	1.76	1.79	1.95	1.88
2.29	2	1.69	2.52	1.95	2.15	1.77	2.06	1.88	2.33	1.91	1.72	2.32	2.23	1.57	1.64	2.01	2.36	2.04	1.88	1.82	2.14	1.88
2.29	2.14	1.9	2.52	1.95	2.27	1.77	2.15	1.88	2.33	1.91	1.72	2.32	2.23	1.74	1.64	2.01	2.56	2.05	1.88	1.82	2.14	1.89
2.3	2.14	1.93	2.52	1.95	2.38	1.77	2.15	1.88	2.33	2.04	1.72	2.32	2.29	1.74	1.64	2.08	2.6	2.05	1.88	2.04	2.14	1.89
2.45	2.34	1.93	2.52	1.95	2.39	1.77	2.15	1.88	2.33	2.04	1.72	2.32	2.29	1.74	1.79	2.08	2.6	2.05	2.08	2.04	2.14	1.89
2.45	2.34	1.94	2.52	1.95	2.43	1.77	2.15	1.94	2.33	2.04	1.72	2.32	2.29	1.74	1.79	2.08	2.6	2.05	2.08	2.04	2.14	2
2.45	2.34	2	2.52	1.95	2.43	1.77	2.15	1.94	2.61	2.04	1.77	2.36	2.46	1.95	1.79	2.08	2.84	2.05	2.08	2.08	2.14	2
2.51	2.34	2	2.67	1.95	2.48	1.98	2.15	1.95	2.61	2.05	1.77	2.37	2.46	1.95	1.9	2.08	2.84	2.05	2.08	2.32	2.28	2.01
2.51	2.34	2.11	2.68	1.95	2.48	1.98	2.45	1.95	2.61	2.05	1.77	2.39	2.46	2.14	1.9	2.22	3.07	2.25	2.08	2.39	2.28	2.01
2.51	2.34	2.11	2.68	1.95	2.56	2.08	2.45	2.21	2.61	2.16	1.77	2.46	2.46	2.26	1.9	2.22	3.07	2.25	2.08	2.48	2.28	2.02
2.62	2.34	2.11	2.68	1.95	2.66	2.12	2.45	2.21	2.61	2.18	1.77	2.46	2.6	2.26	1.9	2.22	3.07	2.25	2.08	2.61	2.28	2.04
2.62	2.34	2.11	2.7	1.95	2.72	2.12	2.45	2.21	2.61	2.2	1.9	2.54	2.6	2.26	1.9	2.33	3.1	2.25	2.08	2.61	2.52	2.04
2.62	2.34	2.11	2.81	1.95	2.72	2.24	2.54	2.21	2.61	2.32	1.9	2.54	2.6	2.27	1.99	2.33	3.19	2.32	2.36	2.61	2.52	2.04
2.72	2.34	2.11	2.81	2.08	2.88	2.24	2.71	2.21	2.61	2.32	1.9	2.57	2.6	2.39	1.99	2.39	3.36	2.37	2.37	2.61	2.52	2.04
2.72	2.38	2.11	2.93	2.08	2.88	2.24	2.71	2.22	2.61	2.32	2.02	2.64	2.77	2.39	2.12	2.39	3.36	2.51	2.49	2.61	2.66	2.12
2.82	2.38	2.11	2.93	2.08	2.88	2.25	3.05	2.22	2.9	2.32	2.02	2.77	2.8	2.39	2.12	2.62	3.36	2.51	2.6	2.64	2.66	2.27
2.87	2.38	2.11	2.93	2.16	3.01	2.33	3.1	2.22	2.91	2.32	2.16	2.77	2.81	2.39	2.17	2.62	3.36	2.51	2.6	2.73	2.66	2.34
2.87	2.5	2.11	2.93	2.17	3.01	2.33	3.1	2.52	2.91	2.43	2.16	2.85	2.81	2.39	2.28	2.62	3.36	2.82	2.6	2.73	2.66	2.34
3.04	2.5	2.2	3.08	2.17	3.15	2.45	3.1	2.53	2.91	2.43	2.16	2.87	2.81	2.51	2.28	2.7	3.46	2.82	2.6	2.73	2.66	2.51
3.04	2.58	2.2	3.08	2.25	3.19	2.46	3.1	2.53	2.91	2.43	2.4	3.02	3	2.59	2.28	2.71	3.46	2.82	2.6	2.73	2.66	2.51
3.05	2.71	2.25	3.08	2.25	3.33	2.46	3.17	2.53	2.91	2.43	2.4	3.07	3.02	2.59	2.28	2.71	3.46	2.82	2.6	2.73	3.06	2.51
3.05	2.71	2.25	3.18	2.25	3.33	2.46	3.29	2.53	2.91	2.43	2.4	3.16	3.02	2.59	2.28	2.92	3.46	2.82	2.6	2.81	3.06	2.52
3.05	2.71	2.41	3.18	2.25	3.44	2.46	3.29	2.53	2.99	2.43	2.44	3.16	3.02	2.62	2.28	2.92	3.46	2.96	2.6	2.87	3.06	2.63
3.05	2.71	2.41	3.18	2.66	3.44	2.46	3.29	2.84	2.99	2.52	2.44	3.25	3.07	2.74	2.49	2.92	3.46	2.96	2.67	3.12	3.06	2.64
3.29	2.71	2.49	3.29	2.66	3.45	2.46	3.29	2.84	2.99	2.52	2.44	3.31	3.07	2.74	2.49	2.92	3.46	2.96	2.67	3.12	3.06	2.64
3.3	2.84	2.52	3.29	2.66	3.45	2.46	3.29	2.84	2.99	2.66	2.5	3.31	3.07	2.74	2.49	2.92	3.46	2.96	2.67	3.2	3.06	2.67
3.3	2.84	2.52	3.3	2.66	3.46	2.56	3.43	2.84	3	2.66	2.5	3.38	3.14	2.74	2.54	3.1	3.62	3.04	2.72	3.21	3.06	2.67
3.3	2.84	2.59	3.3	2.8	3.46	2.56	3.43	2.84	3	2.66	2.61	3.38	3.24	2.74	2.54	3.1	3.62	3.04	2.86	3.21	3.06	2.76
3.31	2.84	2.59	3.34	2.8	3.52	2.56	3.43	2.96	3	2.66	2.61	3.38	3.41	2.74	2.54	3.1	3.62	3.04	2.87	3.21	3.06	2.76
3.31	2.99	2.59	3.34	2.8	3.52	2.56	3.43	3.1	3	2.66	2.61	3.45	3.41	2.74	2.61	3.15	3.62	3.19	2.87	3.21	3.13	2.76
3.31	2.99	2.59	3.43	2.8	3.52	2.64	3.43	3.1	3	2.66	2.61	3.45	3.41	2.78	2.61	3.29	3.62	3.2	3.06	3.21	3.13	2.88
3.47	2.99	2.63	3.44	2.8	3.95	2.64	3.46	3.1	3	2.66	2.61	3.45	3.41	2.78	2.61	3.29	3.66	3.2	3.06	3.22	3.13	2.88
3.47	2.99	2.71	3.44	2.8	3.95	2.77	3.46	3.11	3	2.79	2.61	3.45	3.6	2.78	2.74	3.29	3.66	3.2	3.06	3.27	3.28	2.92
3.47	3.12	2.72	3.44	2.95	3.96	2.77	3.55	3.11	3	2.79	2.89	3.45	3.6	2.78	2.74	3.46	3.66	3.2	3.06	3.3	3.28	2.93
3.53	3.12	2.72	3.59	2.95	3.96	2.77	3.55	3.12	3	2.79	2.89	3.52	3.6	2.78	2.74	3.46	3.66	3.28	3.06	3.34	3.29	2.93
3.6	3.33	2.72	3.59	2.95	3.96	2.86	3.55	3.41	3.33	2.79	2.89	3.64	3.6	2.78	2.84	3.46	3.66	3.28	3.06	3.37	3.29	2.93
3.6	3.33	2.8	3.59	2.97	3.96	2.86	3.55	3.41	3.33	2.99	2.89	3.64	3.6	2.9	2.84	3.66	3.66	3.28	3.35	3.37	3.39	2.93
3.73	3.33	2.8	3.61	2.97	3.96	2.86	3.87	3.41	3.33	2.99	2.89	3.79	3.6	2.9	2.84	3.66	3.78	3.28	3.35	3.37	3.39	3.03
3.73	3.5	2.8	3.61	3.12	3.96	2.86	3.87	3.41	3.33	2.99	3.13	3.98	3.76	2.9	2.84	3.66	3.78	3.51	3.35	3.42	3.39	3.03
3.73	3.5	2.97	3.98	3.12	4.03	2.97	3.87	3.41	3.33	3.01	3.13	3.98	3.77	2.98	2.95	3.66	3.94	3.51	3.35	3.44	3.51	3.24
3.94	3.5	2.97	3.98	3.12	4.03	2.97	4.08	3.41	3.33	3.01	3.13	3.98	3.77	3.11	2.95	3.66	3.94	3.51	3.42	3.48	3.51	3.24
3.94	3.5	2.97	3.98	3.12	4.03	2.97	4.08	3.41	3.33	3.35	3.26	4.05	3.77	3.11	2.95	3.66	3.94	3.51	3.42	3.59	3.51	3.24
3.94	3.7	3.24	3.98	3.46	4.03	3.21	4.09	3.87	3.68	3.35	3.26	4.13	3.77	3.35	3.06	3.9	4.12	3.51	3.68	3.59	3.51	3.24
3.94	3.7	3.34	4.11	3.47	4.1	3.21	4.09	3.87	3.69	3.35	3.51	4.13	4.01	3.35	3.15	3.9	4.12	3.61	3.68	3.99	3.63	3.41
3.94	3.77	3.34	4.11	3.47	4.23	3.21	4.09	3.87	3.69	3.36	3.51	4.18	4.01	3.35	3.16	3.9	4.12	3.61	3.68	3.99	3.63	3.45
4.15	3.77	3.34	4.45	3.47	4.36	3.41	4.09	3.88	3.69	3.68	3.51	4.19	4.01	3.35	3.16	3.92	4.29	3.61	3.78	3.99	3.63	3.58
4.32	4	3.34	4.45	3.47	4.36	3.41	4.12	4.06	3.91	3.68	3.51	4.38	4.29	3.39	3.16	3.92	4.29	3.84	3.78	4.09	3.75	3.59
4.32	4	3.34	4.73	3.68	4.36	3.41	4.12	4.26	3.91	3.68	3.64	4.49	4.31	3.39	3.49	3.92	4.3	3.84	4.04	4.17	3.99	3.59
4.32	4	3.34	4.73	3.69	4.55	3.41	4.12	4.26	3.91	3.68	3.76	4.5	4.31	3.45	3.49	4.2	4.7	3.84	4.04	4.54	3.99	3.6
4.32	4.01	3.34	4.73	3.7	4.68	3.59	4.29	4.26	3.92	3.68	3.83	4.57	4.31	3.8	3.49	4.2	4.75	3.91	4.04	4.54	4.15	3.63
4.62	4.03	3.4	4.9	3.86	4.68	3.61	4.29	4.27	3.94	3.68	3.92	4.66	4.7	3.8	3.49	4.46	5.1	3.91	4.36	4.54	4.16	3.76
4.64	4.03	3.44	4.9	3.86	4.85	3.62	4.55	4.28	4.21	3.85	3.92	4.66	4.71	3.8	3.61	4.46	5.1	4.29	4.36	4.54	4.16	3.93
4.85	4.18	3.44	5.19	3.86	4.85	3.79	4.55	4.7	4.21	4.13	3.92	4.74	4.72	3.86	3.68	4.46	5.1	4.31	4.37	4.54	4.3	3.97
4.85	4.34	3.48	5.19	4.09	5.28	3.79	4.61	4.7	4.22	4.14	4.08	4.8	4.72	3.93	3.83	4.57	5.1	4.31	4.38	4.54	4.46	4.17
4.85	4.34	3.48	5.19	4.09	5.29	3.88	5.09	4.7	4.22	4.14	4.08	4.8	4.72	4	3.83	4.68	5.12	4.59	4.58	4.54	4.46	4.22
4.85	4.42	3.5	5.19	4.1	5.3	3.89	5.09	4.7	4.22	4.14	4.08	5	4.72	4.25	4.06	4.9	5.43	4.67	4.58	4.73	4.61	4.22
4.9	4.42	3.59	5.32	4.1	5.3	4.08	5.09	4.7	4.61	4.14	4.22	5.04	4.92	4.26	4.06	5.05	5.64	4.67	4.58	4.76	4.61	4.22
4.9	4.42	3.61	5.47	4.1	5.3	4.13	5.09	4.7	4.61	4.41	4.22	5.05	5.12	4.26	4.06	5.05	5.64	4.73	4.58	4.76	4.62	4.36
4.9	4.58	3.72	5.47	4.1	5.31	4.18	5.09	4.71	4.61	4.41	4.24	5.05	5.12	4.27	4.13	5.05	5.64	4.9	4.58	4.82	4.62	4.36
4.9	4.58	3.84	5.47	4.1	5.31	4.18	5.09	4.71	4.61	4.41	4.33	5.05	5.12	4.27	4.22	5.05	5.9	4.9	4.69	4.86	4.63	4.36
4.9	4.59	3.87	5.47	4.1	5.31	4.18	5.09	4.71	4.61	4.41	4.4	5.14	5.12	4.37	4.22	5.05	6.11	4.9	4.84	4.86	4.66	4.37
5.1	4.59	3.93	5.54	4.1	5.42	4.54	5.18	4.71	4.61	4.41	4.4	5.14	5.33	4.38	4.22	5.05	6.11	4.9	4.84	4.86	4.66	4.37
5.1	4.59	3.94	5.68	4.24	5.42	4.56	5.27	4.71	4.61	4.41	4.52	5.23	5.33	4.38	4.22	5.25	6.13	4.9	4.84	4.86	4.71	4.38
5.1	4.75	4	5.73	4.24	5.49	4.56	5.27	4.89	4.61	4.62	4.57	5.33	5.33	4.46	4.26	5.25	6.3	4.9	4.84	5.05	4.71	4.39
5.31	4.75	4	5.73	4.24	5.51	4.56	5.27	5.02	4.61	4.62	4.57	5.33	5.44	4.65	4.32	5.38	6.3	4.9	5.05	5.05	4.87	4.56
5.31	4.75	4	5.73	4.54	5.51	4.64	5.44	5.05	4.88	4.76	4.57	5.34	5.69	4.66	4.43	5.39	6.3	5.12	5.05	5.09	4.87	4.59
5.5	4.84	4.05	5.73	4.54	5.59	4.64	5.44	5.06	4.88	4.77	4.68	5.42	5.69	4.66	4.43	5.39	6.3	5.16	5.05	5.09	4.87	4.6
5.5	4.85	4.25	5.73	4.54	5.61	4.64	5.44	5.08	4.88	4.77	4.68	5.45	5.69	4.66	4.48	5.39	6.46	5.18	5.05	5.12	5	4.69
5.5	4.86	4.25	5.75	4.62	5.7	4.78	5.63	5.08	4.88	4.77	4.73	5.45	5.69	4.92	4.56	5.39	6.46	5.18	5.25	5.12	5	4.7
5.5	4.86	4.25	5.75	4.62	5.73	4.78	5.63	5.11	4.88	5.07	4.81	5.45	5.69	4.93	4.56	5.46	6.46	5.32	5.35	5.12	5	4.7
5.5	5.02	4.47	5.89	4.62	5.73	4.85	5.63	5.36	4.88	5.07	4.81	5.45	5.72	4.93	4.56	5.62	6.46	5.32	5.35	5.2	5	4.7
5.64	5.02	4.47	5.89	4.62	5.96	4.85	5.63	5.37	5.03	5.07	4.81	5.52	5.72	4.93	4.77	5.62	6.64	5.35	5.35	5.2	5	4.83
5.64	5.02	4.47	5.89	4.62	6.02	4.99	5.63	5.37	5.03	5.07	4.81	5.52	5.72	4.93	4.77	5.62	6.64	5.35	5.35	5.2	5.01	4.83
5.83	5.02	4.47	5.93	4.62	6.04	5.06	5.63	5.37	5.03	5.07	4.96	5.65	5.72	4.94	4.77	5.62	6.88	5.45	5.35	5.23	5.12	4.83
5.83	5.08	4.47	5.96	4.62	6.04	5.2	5.64	5.37	5.03	5.07	4.96	5.7	5.72	5	4.9	5.75	6.88	5.45	5.42	5.35	5.12	5
5.83	5.08	4.47	5.96	4.89	6.15	5.21	5.78	5.37	5.03	5.07	5.07	5.7	5.72	5	4.91	5.75	7	5.46	5.42	5.42	5.12	5.01

6.03	5.33	4.52	5.96	4.89	6.31	5.42	5.78	5.37	5.03	5.07	5.14	5.76	6.04	5.06	4.98	5.77	7.12	5.46	5.61	5.43	5.17	5.01
6.03	5.33	4.54	6.17	4.89	6.31	5.43	5.78	5.37	5.03	5.07	5.14	5.76	6.04	5.19	4.98	5.9	7.12	5.62	5.62	5.53	5.35	5.01
6.03	5.33	4.54	6.22	4.89	6.42	5.43	5.78	5.37	5.18	5.07	5.14	5.92	6.06	5.31	5.21	5.9	7.3	5.62	5.62	5.61	5.35	5.08
6.03	5.4	4.59	6.22	5.07	6.42	5.43	5.79	5.58	5.23	5.4	5.2	6.04	6.06	5.31	5.21	6.09	7.3	5.62	5.62	5.61	5.51	5.09
6.03	5.4	4.59	6.61	5.08	6.53	5.67	5.87	5.58	5.23	5.4	5.2	6.04	6.06	5.31	5.21	6.09	7.3	5.69	5.84	5.72	5.57	5.09
6.21	5.53	4.62	6.61	5.08	6.53	5.67	5.88	5.59	5.24	5.4	5.25	6.04	6.06	5.32	5.21	6.09	7.3	5.69	5.84	5.76	5.6	5.09
6.21	5.54	4.66	6.61	5.08	6.71	5.86	5.94	5.59	5.24	5.4	5.25	6.16	6.2	5.32	5.23	6.32	7.3	5.69	5.84	5.77	5.6	5.09
6.21	5.54	4.66	6.61	5.16	6.71	5.86	5.94	5.69	5.24	5.4	5.25	6.16	6.2	5.35	5.23	6.32	7.61	5.92	5.84	5.77	5.73	5.09
6.21	5.74	4.8	6.61	5.16	6.79	5.86	5.94	5.69	5.6	5.52	5.44	6.27	6.2	5.39	5.23	6.4	7.61	5.92	5.84	5.81	5.73	5.09
6.21	5.74	4.97	6.61	5.16	6.79	6.12	5.94	5.82	5.81	5.52	5.44	6.35	6.2	5.39	5.41	6.43	7.61	5.95	5.98	6.03	5.83	5.32
6.21	5.9	5	6.61	5.16	6.79	6.12	5.94	5.82	5.81	5.52	5.44	6.41	6.36	5.39	5.41	6.43	7.61	5.95	5.98	6.07	5.83	5.51
6.42	5.9	5.02	6.77	5.32	6.79	6.12	6.13	5.82	5.81	5.52	5.55	6.41	6.36	5.4	5.41	6.43	7.61	5.95	5.98	6.13	5.88	5.51
6.42	5.9	5.02	6.77	5.32	6.93	6.12	6.13	5.82	5.81	5.52	5.55	6.41	6.44	5.4	5.57	6.43	7.61	5.95	5.98	6.16	5.95	5.51
6.42	5.9	5.02	6.77	5.32	6.93	6.16	6.13	6.05	5.81	5.68	5.55	6.41	6.63	5.42	5.57	6.49	7.61	6.03	5.98	6.17	5.95	5.51
6.43	6.11	5.02	6.77	5.32	6.93	6.16	6.23	6.05	5.98	5.68	5.76	6.45	6.64	5.42	5.71	6.49	7.61	6.03	5.98	6.17	6.02	5.51
6.53	6.11	5.07	6.77	5.32	7.07	6.16	6.23	6.05	5.98	5.68	5.76	6.54	6.64	5.49	5.8	6.71	7.61	6.03	6.19	6.18	6.04	5.82
6.53	6.11	5.07	6.77	5.32	7.07	6.28	6.23	6.29	5.98	5.79	5.76	6.65	6.64	5.59	5.8	6.71	7.74	6.21	6.2	6.21	6.04	5.82
6.53	6.11	5.07	6.78	5.32	7.07	6.28	6.32	6.29	5.98	5.79	5.76	6.75	6.72	5.59	5.8	6.71	7.74	6.21	6.2	6.21	6.04	5.82
6.8	6.11	5.07	6.78	5.32	7.07	6.28	6.32	6.41	6.15	5.79	5.82	6.75	6.72	5.59	5.8	6.71	7.74	6.21	6.2	6.21	6.04	5.83
6.8	6.14	5.09	6.89	5.44	7.07	6.28	6.32	6.41	6.15	5.79	5.82	6.81	6.72	5.64	5.81	6.71	7.74	6.21	6.2	6.21	6.07	5.83
6.8	6.27	5.09	6.89	5.45	7.1	6.31	6.46	6.41	6.15	5.79	5.82	6.81	6.72	5.68	5.93	6.87	7.74	6.21	6.2	6.21	6.07	5.83
6.8	6.27	5.09	6.89	5.45	7.1	6.31	6.46	6.5	6.15	5.79	5.82	6.9	6.92	5.68	5.93	6.87	7.74	6.32	6.2	6.23	6.08	5.83
6.8	6.28	5.09	7.07	5.45	7.22	6.31	6.46	6.5	6.15	5.79	5.89	6.9	6.92	5.68	5.93	6.87	7.74	6.36	6.2	6.34	6.15	5.83
6.8	6.39	5.15	7.07	5.69	7.23	6.4	6.46	6.69	6.38	5.79	5.89	6.92	6.92	5.72	5.93	6.87	7.74	6.36	6.2	6.34	6.15	5.83
6.9	6.4	5.15	7.21	5.69	7.25	6.4	6.52	6.69	6.39	5.79	5.89	6.92	6.92	5.72	5.96	7.08	7.78	6.36	6.2	6.34	6.15	5.87
6.9	6.4	5.15	7.21	5.69	7.25	6.4	6.52	6.69	6.39	5.94	5.98	7.08	6.92	5.92	5.96	7.08	7.78	6.37	6.35	6.5	6.17	5.87
6.9	6.4	5.15	7.21	5.69	7.25	6.4	6.52	6.69	6.64	5.94	6.17	7.08	7.07	5.94	6.1	7.08	7.78	6.5	6.35	6.5	6.37	5.87
6.9	6.65	5.25	7.33	5.69	7.31	6.53	6.66	6.69	6.65	5.94	6.17	7.08	7.07	5.94	6.1	7.08	7.78	6.66	6.52	6.5	6.37	5.88
7.06	6.84	5.25	7.33	5.69	7.42	6.53	6.66	6.99	6.65	5.94	6.36	7.08	7.07	5.94	6.1	7.2	7.91	6.73	6.52	6.71	6.44	5.93
7.06	6.84	5.41	7.33	5.99	7.42	6.53	6.66	6.99	6.65	5.94	6.37	7.08	7.07	5.94	6.24	7.2	7.91	6.74	6.52	6.71	6.46	5.93
7.32	6.84	5.41	7.33	5.99	7.42	6.55	6.78	6.99	6.65	5.94	6.37	7.16	7.21	5.99	6.24	7.2	7.91	6.74	6.52	6.91	6.74	6.27
7.33	6.84	5.44	7.53	5.99	7.42	6.71	6.78	6.99	6.77	6.22	6.37	7.16	7.22	6	6.32	7.2	7.91	6.84	6.68	6.91	6.74	6.27
7.48	6.84	5.44	7.62	6.16	7.6	6.71	6.83	6.99	6.77	6.22	6.55	7.16	7.22	6.07	6.32	7.41	8.12	6.84	6.69	6.91	6.74	6.27
7.57	6.84	5.58	7.84	6.17	7.69	6.71	6.84	7.06	7.01	6.22	6.55	7.38	7.32	6.22	6.45	7.41	8.16	6.84	6.69	6.91	6.82	6.27
7.68	7.05	5.71	7.84	6.17	7.69	6.95	7.16	7.06	7.01	6.22	6.61	7.38	7.32	6.41	6.56	7.41	8.16	7.13	6.69	6.91	6.91	6.52
7.68	7.05	5.71	8.03	6.17	7.83	6.95	7.16	7.2	7.01	6.22	6.61	7.6	7.42	6.41	6.56	7.41	8.16	7.13	6.69	6.91	6.91	6.56
7.85	7.26	5.71	8.03	6.17	7.92	6.95	7.16	7.26	7.37	6.47	6.7	7.67	7.42	6.5	6.71	7.65	8.25	7.13	7.11	7.1	6.97	6.56
7.85	7.26	5.94	8.03	6.44	7.92	6.98	7.29	7.27	7.38	6.47	6.85	7.75	7.85	6.5	6.71	7.65	8.45	7.13	7.11	7.1	6.97	6.56
7.85	7.26	5.94	8.03	6.45	8.04	7.28	7.29	7.28	7.38	6.47	6.85	7.82	7.85	6.65	6.76	7.65	8.45	7.14	7.11	7.1	7.35	6.56
8.05	7.27	5.95	8.26	6.45	8.04	7.28	7.29	7.44	7.39	6.47	6.97	7.84	7.85	6.65	6.76	7.65	8.65	7.14	7.11	7.11	7.35	6.56
8.15	7.28	5.95	8.45	6.45	8.05	7.28	7.42	7.44	7.39	6.48	6.97	7.84	7.85	6.65	6.88	7.8	8.78	7.37	7.36	7.13	7.42	6.63
8.16	7.28	6.11	8.7	6.61	8.37	7.38	7.42	7.55	7.67	6.77	7.05	7.94	7.96	6.65	6.88	7.81	8.89	7.38	7.37	7.18	7.63	6.67
8.16	7.29	6.17	8.7	6.61	8.37	7.38	7.42	7.74	7.67	6.78	7.09	8.18	8.05	6.71	7.09	7.81	9.01	7.71	7.37	7.22	7.63	6.76
8.29	7.43	6.17	8.7	6.62	8.47	7.38	7.74	7.74	7.67	7.04	7.11	8.39	8.05	6.8	7.15	7.81	9.01	7.71	7.37	7.41	7.63	6.76
8.29	7.46	6.18	8.75	6.63	8.83	7.38	7.74	7.74	7.67	7.04	7.32	8.39	8.35	6.8	7.15	8.03	9.32	7.72	7.76	7.41	7.64	6.9
8.3	7.72	6.24	8.75	6.63	8.83	7.58	7.78	7.86	8.05	7.04	7.32	8.39	8.36	6.87	7.28	8.25	9.32	7.74	7.76	7.55	7.93	6.98
8.37	7.72	6.26	8.75	6.63	8.83	7.62	7.78	7.89	8.05	7.06	7.32	8.39	8.36	6.88	7.28	8.25	9.32	7.95	7.76	7.55	7.93	7.12
8.37	7.72	6.26	8.87	6.82	9.04	7.62	7.78	7.89	8.05	7.06	7.32	8.39	8.36	6.88	7.49	8.25	9.45	7.95	7.76	7.55	7.93	7.16
8.53	7.72	6.48	9.01	6.82	9.04	7.7	7.78	7.89	8.05	7.06	7.35	8.39	8.36	7.13	7.49	8.25	9.6	7.95	7.76	7.55	8.02	7.16
8.53	7.72	6.51	9.14	6.82	9.05	7.77	8.05	7.95	8.05	7.26	7.39	8.47	8.49	7.13	7.49	8.45	9.6	7.95	7.76	7.55	8.02	7.16
8.58	7.72	6.51	9.14	6.82	9.22	7.78	8.05	8.01	8.05	7.26	7.39	8.48	8.49	7.22	7.6	8.45	9.6	7.95	8.09	7.63	8.02	7.16
8.66	7.82	6.52	9.14	6.82	9.22	7.78	8.05	8.02	8.28	7.27	7.39	8.55	8.49	7.24	7.6	8.45	9.66	8.11	8.09	7.63	8.02	7.16
8.66	7.83	6.56	9.14	7.1	9.22	7.78	8.05	8.02	8.28	7.28	7.39	8.62	8.61	7.24	7.72	8.45	9.66	8.27	8.09	7.88	8.02	7.16
8.66	7.89	6.61	9.14	7.11	9.35	7.89	8.06	8.13	8.48	7.28	7.39	8.62	8.61	7.33	7.81	8.74	9.66	8.27	8.47	7.88	8.03	7.25
8.78	7.89	6.73	9.14	7.11	9.36	7.92	8.06	8.17	8.49	7.28	7.47	8.73	8.77	7.33	7.85	8.74	9.84	8.43	8.47	7.88	8.06	7.25
8.78	7.89	6.73	9.16	7.11	9.36	8	8.06	8.17	8.49	7.28	7.61	8.75	8.77	7.34	7.96	8.74	9.84	8.44	8.51	7.88	8.06	7.35
8.83	7.99	6.73	9.16	7.11	9.43	8	8.12	8.23	8.49	7.61	7.67	8.75	8.86	7.48	7.96	8.74	9.84	8.44	8.61	7.88	8.06	7.37
8.83	7.99	6.77	9.35	7.11	9.43	8.04	8.12	8.23	8.49	7.61	7.67	8.8	8.88	7.49	7.96	8.74	10.12	8.44	8.61	7.88	8.06	7.46
8.92	8.1	6.77	9.35	7.11	9.61	8.04	8.27	8.23	8.49	7.85	7.67	8.85	8.97	7.57	7.96	8.87	10.12	8.45	8.61	7.99	8.22	7.46
8.93	8.1	6.78	9.35	7.17	9.61	8.27	8.27	8.3	8.58	7.85	7.67	8.85	8.98	7.58	8.08	8.87	10.12	8.65	9.02	7.99	8.22	7.46
8.93	8.1	6.82	9.35	7.17	9.61	8.28	8.27	8.36	8.66	7.88	7.8	8.95	9	7.63	8.19	8.87	10.34	8.66	9.02	7.99	8.29	7.59
9.02	8.1	6.87	9.35	7.17	9.73	8.3	8.35	8.45	8.66	7.88	7.8	8.96	9	7.67	8.2	8.91	10.34	8.66	9.02	8.09	8.3	7.62
9.02	8.1	6.91	9.61	7.17	9.73	8.3	8.36	8.49	8.66	7.88	7.87	8.98	9.04	7.67	8.2	9.27	10.34	8.66	9.02	8.09	8.3	7.62
9.02	8.1	6.91	9.61	7.55	9.87	8.4	8.36	8.49	8.66	7.88	7.9	9	9.04	7.72	8.32	9.27	10.34	8.74	9.02	8.1	8.3	7.64
9.02	8.2	6.94	9.61	7.55	9.87	8.41	8.51	8.49	8.66	7.88	7.94	9.06	9.04	7.75	8.33	9.27	10.44	8.74	9.02	8.1	8.3	7.66
9.08	8.2	7.12	9.72	7.55	9.87	8.61	8.51	8.52	8.66	7.88	7.97	9.12	9.3	7.83	8.33	9.47	10.78	8.74	9.02	8.1	8.5	7.71
9.13	8.2	7.12	9.72	7.55	9.87	8.64	8.6	8.57	8.66	7.88	8.01	9.16	9.3	7.83	8.34	9.47	10.78	8.74	9.02	8.11	8.51	7.88
9.13	8.2	7.12	9.88	7.55	10.1	8.64	8.6	8.57	8.77	7.88	8.01	9.21	9.38	7.92	8.58	9.47	10.78	8.88	9.32	8.25	8.51	7.88
9.27	8.2	7.12	9.88	7.55	10.34	8.64	8.63	8.57	8.91	7.88	8.05	9.21	9.4	7.96	8.58	9.49	10.79	8.88	9.32	8.25	8.51	7.88
9.27	8.46	7.17	9.88	7.55	10.34	8.69	8.68	8.58	8.91	8.12	8.05	9.29	9.4	8.18	8.58	9.62	10.79	8.92	9.32	8.25	8.51	7.88
9.39	8.46	7.17	10.06	7.55	10.34	8.69	8.74	8.74	8.91	8.12	8.08	9.31	9.4	8.18	8.72	9.62	10.79	9.14	9.47	8.32	8.71	7.88
9.39	8.63	7.3	10.06	7.55	10.34	8.69	8.74	8.74	8.91	8.12	8.09	9.41	9.66	8.21	8.72	9.77	10.79	9.14	9.48	8.56	8.73	8
9.39	8.63	7.3	10.06	7.81	10.49	8.69	8.74	8.91	9.2	8.12	8.09	9.47	9.68	8.36	8.76	9.89	10.79	9.14	9.48	8.56	8.73	8.15
9.39	8.63	7.34	10.06	7.81	10.49	8.93	8.96	8.91	9.21	8.12	8.3	9.66	9.68	8.36	8.84	9.89	10.79	9.42	9.48	8.56	8.82	8.17
9.39	8.63	7.34	10.13	7.94	10.49	8.93	8.96	9.14	9.31	8.32	8.3	9.66	9.76	8.36	8.84	10.03	10.79	9.42	9.48	8.56	8.82	8.19
9.51	8.63	7.46	10.13	7.95	10.57	8.93	8.96	9.14	9.31	8.32	8.36	9.67	9.77	8.42	8.84	10.03	10.84	9.42	9.48	8.56	8.82	8.19
9.52	8.82	7.46	10.13	7.95	10.68	8.94	8.96	9.14	9.31	8.32	8.36	9.76	9.77	8.5	8.88	10.03	10.84	9.42	9.48	8.56	8.94	8.19
9.65	8.82	7.78	10.13	7.95	10.68	9.2	9.14	9.3	9.31	8.32	8.53	9.81	9.77	8.5	8.88	10.03	10.84	9.42	9.63	8.57	8.95	8.23
9.82	8.82	7.78	10.13	7.95	10.68	9.2	9.14	9.3	9.63	8.32	8.53	9.86	9.96	8.71	8.89	10.14	10.84	9.42	9.63	8.57	8.95	8.38
9.83	8.82	7.78	10.13	7.95	10.69	9.2	9.14	9.3	9.63	8.32	8.53	9.86	9.96	8.72	8.89	10.14	10.84	9.57	9.63	8.57	9.05	8.38
9.83	8.82	7.78	10.35	8.15	10.69	9.2	9.14	9.44	9.63	8.32	8.54	9.93	10.05	8.72	9.05	10.14	10.84	9.57	9.66	8.57	9.05	8.44
10.04	8.82	7.78	10.35	8.15	10.72	9.32	9.14	9.44	9.76	8.32	8.54	9.96	10.06	8.78	9.05	10.14	10.85	9.57	9.66	8.63	9.05	8.45
10.05	8.82	7.82	10.35	8.15	10.72	9.34	9.14	9.44	9.76	8.32	8.75	9.99	10.06	8.89	9.05	10.27	10.86	9.58	9.68	8.75	9.18	8.47
10.05	8.96	7.82	10.5	8.15	10.82	9.34	9.14	9.55	9.76	8.5	8.75	9.99	10.06	8.89	9.17	10.27	10.86	9.58	9.68	8.75	9.18	8.47
10.06	8.96	7.85	10.5	8.15	10.82	9.34	9.14	9.55	9.91	8.5	8.75	10.11	10.22	8.89	9.17	10.27	10.86	9.74	9.77	8.75	9.18	8.52
10.06	8.96	7.86	10.5	8.15	10.83	9.34	9.27	9.71	9.91	8.5	8.75	10.13	10.22	8.89	9.35	10.27	10.94	9.74	9.77	8.75	9.18	8.52
10.07	8.96	7.86	10.5	8.4	10.83	9.39	9.27	9.71	9.91	8.5	8.75	10.24	10.22	8.89	9.35	10.29	10.94	9.74	9.77	8.75	9.18	8.52
10.22	.	.	.	8.4	10.93	9.39	9.27	9.71	9.91	.	.	10.27	10.22	8.97	9.35	10.29	10.94	9.81	9.81	8.75	9.18	8.52
10.26	.	.	.	8.4	10.93	9.39	9.27	9.8	9.91	.	.	10.27	10.22	8.97	9.35	10.29	10.94	9.81	9.81	8.87	9.34	8.53
10.26	.	.	.	8.4	10.93	9.5	9.27	9.8	9.91	.	.	10.27	10.29	8.97	9.35	10.42	10.95	9.81	9.81	8.87	9.34	8.55
10.26	.	.	.	8.41	10.95	9.66	9.39	9.8	9.91	.	.	10.32	10.29	9.07	9.35	10.5	11	9.81	9.89	8.88	9.46	8.6
10.26	.	.	.	8.41	11.01	9.66	9.47	9.95	10.17	.	.	10.33	10.29	9.14	9.5	10.5	11	10.01	9.89	8.89	9.46	8.6
10.26	.	.	.	8.6	11.02	9.66	9.47	9.96	10.17	.	.	10.38	10.29	9.3	9.5	10.58	11	10.01	9.89	8.89	9.46	8.6
10.26	.	.	.	8.61	11.02	9.66	9.47	9.96	10.18	.	.	10.38	10.29	9.3	9.5	10.58	11	10.01	10.14	9.1	9.5	8.78
10.39	.	.	.	8.61	11.1	9.73	9.72	10.07	10.18	.	.	10.44	10.48	9.3	9.62	10.58	11.16	10.13	10.14	9.1	9.5	8.79
10.39	.	.	.	8.61	11.1	9.87	9.72	10.07	10.35	.	.	10.44	10.48	9.3	9.67	10.58	11.16	10.13	10.14	9.1	9.64	8.8
10.63	.	.	.	9.02	11.23	9.87	9.72	10.24	10.35	.	.	10.51	10.48	9.47	9.79	10.67	11.16	10.13	10.14	9.1	9.64	8.8
10.75	.	.	.	9.02	11.23	9.87	9.88	10.24	10.35	.	.	10.53	10.59	9.47	9.79	10.82	11.16	10.19	10.14	9.27	9.64	8.8
10.75	.	.	.	9.02	11.36	9.87	9.88	10.24	10.35	.	.	10.53	10.59	9.47	9.79	10.82	11.16	10.19	10.14	9.28	9.64	8.82
10.75	.	.	.	9.02	11.36	10.03	9.88	10.25	10.41	.	.	10.67	10.59	9.53	9.79	10.94	11.18	10.19	10.38	9.29	9.76	9.12
10.92	.	.	.	9.02	11.4	10.03	10.11	10.51	10.53	.	.	10.67	10.59	9.53	9.81	10.98	11.18	10.19	10.39	9.32	9.76	9.12
10.92	.	.	.	9.18	11.4	10.03	10.11	10.51	10.8	.	.	10.85	10.76	9.59	9.92	10.98	11.39	10.19	10.39	9.35	9.98	9.12
10.92	.	.	.	9.18	11.43	10.03	10.11	10.51	10.8	.	.	10.85	10.93	9.79	9.92	10.98	11.39	10.44	10.54	9.36	9.99	9.12
10.92	.	.	.	9.19	11.59	10.03	10.11	10.51	10.8	.	.	10.85	10.94	9.79	10.1	11.12	11.49	10.44	10.54	9.37	9.99	9.12
11.01	.	.	.	9.21	11.59	10.27	10.11	10.7	11.08	.	.	10.87	10.94	9.79	10.1	11.12	11.63	10.58	10.54	9.59	10.2	9.13
11.22	.	.	.	9.21	11.59	10.51	10.17	10.86	11.08	.	.	10.97	10.94	9.87	10.1	11.32	11.84	10.58	10.54	9.65	10.21	9.47
11.22	.	.	.	9.21	11.84	10.51	10.46	10.86	11.08	.	.	11.09	11.16	9.91	10.31	11.32	11.93	10.58	10.8	9.65	10.43	9.47
11.22	.	.	.	9.36	11.84	10.51	10.47	10.86	11.26	.	.	11.11	11.32	9.91	10.31	11.52	11.93	10.8	11.06	9.65	10.58	9.47
11.22	.	.	.	9.36	12.02	10.67	10.47	10.86	11.26	.	.	11.12	11.33	9.93	10.48	11.57	12.14	10.8	11.1	9.68	10.58	9.47
11.42	.	.	.	9.59	12.02	10.75	10.65	10.96	11.26	.	.	11.16	11.33	10.04	10.49	11.57	12.17	10.8	11.1	9.79	10.58	9.57
11.42	.	.	.	9.6	12.14	10.75	10.65	11.11	11.26	.	.	11.18	11.48	10.12	10.68	11.57	12.17	10.8	11.25	9.87	10.59	9.63
11.42	.	.	.	9.6	12.14	10.75	10.65	11.11	11.45	.	.	11.18	11.52	10.12	10.68	11.57	12.17	10.8	11.25	9.87	10.76	9.63
11.6	.	.	.	9.6	12.31	10.91	10.65	11.11	11.45	.	.	11.28	11.52	10.12	10.73	11.57	12.46	10.8	11.28	9.87	10.76	9.63
11.6	.	.	.	9.6	12.31	10.92	10.71	11.11	11.45	.	.	11.29	11.72	10.12	10.73	11.72	12.46	11	11.28	9.99	10.76	9.83
11.6	.	.	.	9.6	12.32	11.11	10.71	11.11	11.45	.	.	11.39	11.73	10.17	10.73	11.72	12.52	11	11.48	9.99	10.9	9.87
11.6	.	.	.	9.72	12.46	11.18	10.71	11.11	11.87	.	.	11.41	11.73	10.23	10.75	11.8	12.63	11.25	11.49	9.99	10.9	9.9
11.74	.	.	.	9.72	12.46	11.22	11.04	11.13	11.87	.	.	11.42	11.88	10.41	10.85	11.94	12.72	11.25	11.68	9.99	10.9	9.9
11.75	.	.	.	9.72	12.54	11.29	11.04	11.13	11.87	.	.	11.44	11.88	10.41	10.86	11.94	12.72	11.25	11.68	10.12	10.93	9.9
11.75	.	.	.	9.79	12.54	11.29	11.04	11.13	12.11	.	.	11.59	11.88	10.53	11.07	12.07	12.72	11.5	11.68	10.19	11.09	9.9
11.75	.	.	.	9.8	12.69	11.29	11.04	11.31	12.12	.	.	11.6	11.88	10.56	11.07	12.07	12.86	11.51	11.92	10.19	11.09	9.9
11.75	.	.	.	9.8	12.69	11.39	11.04	11.31	12.12	.	.	11.6	12.09	10.56	11.26	12.07	12.86	11.51	12.09	10.19	11.27	9.9

11.75	.	.	.	9.8	12.81	11.39	11.04	11.31	12.12	.	.	11.64	12.12	10.56	11.27	12.07	13.05	11.51	12.09	10.19	11.27	9.97
12.01	.	.	.	9.8	12.95	11.58	11.04	11.39	12.12	.	.	11.65	12.12	10.58	11.27	12.07	13.05	11.51	12.09	10.2	11.48	9.98
12.01	.	.	.	9.8	12.95	11.6	11.41	11.4	12.12	.	.	11.77	12.12	10.62	11.27	12.23	13.06	11.77	12.09	10.2	11.48	9.98
12.01	.	.	.	9.8	12.96	11.6	11.41	11.4	12.26	.	.	11.77	12.38	10.71	11.3	12.23	13.06	11.77	12.09	10.23	11.48	9.98
12.01	.	.	.	9.8	12.96	11.6	11.41	11.52	12.27	.	.	11.9	12.38	10.82	11.3	12.23	13.06	11.77	12.32	10.3	11.61	10
12.12	.	.	.	9.8	13.02	11.76	11.41	11.52	12.27	.	.	11.9	12.38	10.85	11.3	12.23	13.07	11.89	12.32	10.31	11.61	10.02
12.12	.	.	.	9.8	13.02	11.77	11.66	11.52	12.27	.	.	11.97	12.38	10.92	11.33	12.4	13.47	11.89	12.32	10.31	11.7	10.07
12.17	.	.	.	9.98	13.15	11.9	11.66	11.52	12.52	.	.	11.97	12.51	10.99	11.34	12.4	13.47	11.89	12.52	10.53	11.7	10.07
12.17	.	.	.	9.98	13.15	11.91	11.66	11.7	12.52	.	.	12.01	12.51	11.04	11.37	12.69	13.47	11.89	12.52	10.53	11.7	10.11
12.28	.	.	.	9.98	13.25	12.04	11.66	11.7	12.52	.	.	12.05	12.67	11.08	11.37	12.69	13.47	11.9	12.52	10.53	11.71	10.11
12.28	.	.	.	9.98	13.25	12.04	11.66	11.7	12.52	.	.	12.05	12.67	11.14	11.56	12.82	13.58	12.02	12.52	10.53	11.71	10.11
12.36	.	.	.	9.98	13.33	12.04	11.66	12.05	12.52	.	.	12.14	12.78	11.21	11.66	12.82	13.62	12.02	12.66	10.69	11.71	10.31
12.37	.	.	.	9.98	13.63	12.04	11.96	12.05	12.62	.	.	12.14	12.78	11.22	11.66	12.82	13.62	12.02	12.66	10.72	11.76	10.31
12.37	.	.	.	10	13.63	12.14	11.96	12.05	12.62	.	.	12.14	12.91	11.34	11.73	12.93	13.62	12.19	12.66	10.85	11.92	10.31
12.39	.	.	.	10	13.63	12.14	11.96	12.07	12.79	.	.	12.25	12.91	11.35	11.8	12.93	13.79	12.38	12.85	10.85	11.92	10.31
12.39	.	.	.	10	13.71	12.32	12.06	12.08	12.8	.	.	12.32	12.91	11.4	11.8	12.93	13.79	12.38	12.85	10.86	12	10.35
12.71	.	.	.	10	13.93	12.32	12.06	12.11	12.8	.	.	12.38	13.15	11.48	11.87	13.1	13.79	12.38	12.85	10.86	12	10.35
12.71	.	.	.	10.33	13.93	12.32	12.06	12.11	12.8	.	.	12.38	13.16	11.69	11.93	13.23	13.79	12.38	12.85	11	12	10.35
12.71	.	.	.	10.33	13.93	12.55	12.06	12.14	12.98	.	.	12.39	13.26	11.73	11.93	13.23	13.79	12.53	12.97	11	12.08	10.35
12.71	.	.	.	10.33	13.93	12.55	12.06	12.14	12.98	.	.	12.59	13.26	11.8	11.93	13.23	13.98	12.53	13.07	11.01	12.08	10.37
12.71	.	.	.	10.33	14.1	12.55	12.06	12.27	13.09	.	.	12.59	13.26	11.8	11.98	13.23	13.98	12.65	13.07	11.22	12.11	10.61
12.77	.	.	.	10.33	14.1	12.56	12.06	12.27	13.09	.	.	12.64	13.39	11.92	11.99	13.23	13.98	12.65	13.07	11.22	12.12	10.62
12.86	.	.	.	10.33	14.14	12.56	12.22	12.27	13.09	.	.	12.71	13.39	11.92	12.1	13.23	13.98	12.7	13.07	11.22	12.12	10.62
12.86	.	.	.	10.33	14.21	12.67	12.22	12.27	13.09	.	.	12.71	13.39	12.12	12.23	13.43	13.98	12.7	13.07	11.22	12.22	10.71
12.86	.	.	.	10.33	14.21	12.67	12.22	12.3	13.09	.	.	12.75	13.39	12.12	12.24	13.43	13.98	12.7	13.07	11.22	12.22	10.72
12.92	.	.	.	10.47	14.22	12.67	12.22	12.3	13.09	.	.	12.77	13.39	12.12	12.24	13.43	13.98	12.78	13.07	11.22	12.22	10.72
12.92	.	.	.	10.47	14.22	12.75	12.22	12.3	13.37	.	.	12.82	13.39	12.12	12.24	13.43	13.98	12.9	13.2	11.22	12.22	10.72
12.99	.	.	.	10.62	14.28	12.75	12.22	12.3	13.37	.	.	12.84	13.39	12.23	12.28	13.55	13.98	12.9	13.2	11.45	12.36	10.76
12.99	.	.	.	10.62	14.28	12.75	12.38	12.5	13.37	.	.	12.84	13.51	12.23	12.37	13.55	13.98	12.9	13.2	11.45	12.37	10.87
12.99	.	.	.	10.62	**14.29**	12.75	12.38	12.5	13.37	.	.	12.84	**13.51**	**12.23**	**12.37**	13.55	13.98	12.9	13.2	11.45	12.37	10.87
						12.75										13.64	13.98	12.9	13.2			
						12.85										13.64	13.98	12.9	13.2			

2	2	5	6	6	8	9	9	2	2	5	8	6	6	9	9	11	11
Box-4	Box-7	Box-3	Box-1	Box-4	Box-7	Box-3	Box-8	Box-1	Box-5	Box-2	Box-6	Box-5	Box-7	Box-4	Box-5	Box-3	Box-7
19.1	25.1	34.2	38	43	52.2	64	75	13.7	20.2	32.3	44.5	45	47	65	68	78	84
M	M	M	M	M	M	M	M	M	M	M	M	M	M	M	M	M	M
Malto	Malto	MD	MD	MD	MD	MD	Malto	MCT	MCT	MCT	MCT	MCT	MCT	MCT	MCT	MCT	MCT
MM	MM	MM	MM	MM	MM	MM	MM	MT	MT	MT	MT	MT	MT	MT	MT	MT	MT
0	0	0	0	0	0	0	0	0	0	0	0	0	0	0	0	0	0
0.02	0.14	0	0.06	0	0	0	0	0	0	0	0	0.13	0	0	0	0	0
0.02	0.39	0.16	0.06	0.33	0	0	0	0	0	0	0.06	0.31	0	0	0	0	0
0.03	0.4	0.27	0.27	0.51	0	0	0	0	0	0	0.26	0.39	0	0	0	0	0
0.05	0.6	0.41	0.3	0.51	0	0	0	0	0	0	0.59	0.45	0	0	0	0	0
0.05	0.73	0.55	0.3	0.75	0	0	0	0	0	0	0.59	0.56	0	0.08	0	0	0
0.05	0.73	0.63	0.51	0.8	0	0	0	0	0.07	0.09	0.65	0.81	0	0.23	0	0	0
0.05	1.26	0.91	0.52	0.98	0	0	0	0.14	0.07	0.19	0.96	0.95	0.29	0.25	0	0	0
0.05	1.28	1.08	0.69	1.09	0.05	0	0	0.31	0.07	0.23	0.96	1.17	0.4	0.25	0	0	0
0.05	1.28	1.33	0.78	1.09	0.14	0	0	0.59	0.26	0.33	1.25	1.41	0.66	0.25	0	0	0
0.05	1.28	1.46	0.79	1.22	0.32	0.04	0	0.75	0.26	0.34	1.27	1.42	0.77	0.39	0.05	0.15	0
0.23	1.28	1.63	0.9	1.48	0.43	0.15	0	0.84	0.31	0.59	1.61	1.62	0.97	0.46	0.05	0.26	0
0.34	1.52	1.64	0.9	1.65	0.56	0.19	0	1.13	0.33	0.78	1.61	1.62	1.18	0.61	0.16	0.41	0.1
0.4	1.52	1.92	1.06	1.77	0.66	0.23	0	1.19	0.5	0.78	1.61	1.91	1.4	0.75	0.17	0.71	0.25
0.4	1.52	1.92	1.24	1.95	0.88	0.35	0	1.42	0.57	0.83	1.61	1.98	1.7	0.89	0.17	0.84	0.33
0.51	1.76	1.92	1.46	2.13	1.01	0.44	0	1.42	0.71	1.02	1.61	1.98	1.94	0.97	0.29	0.93	0.34
0.62	1.84	1.92	1.46	2.13	1.17	0.53	0.15	1.42	0.71	1.15	1.77	1.98	2.02	1.25	0.44	1.23	0.35
0.64	1.84	1.92	1.62	2.47	1.32	0.54	0.43	1.42	0.71	1.41	1.82	2.19	2.32	1.39	0.52	1.47	0.35
0.68	1.84	1.92	1.62	2.68	1.44	0.61	0.49	1.43	0.71	1.5	2.01	2.2	2.32	1.56	0.52	1.53	0.47
0.76	1.84	2.11	1.65	2.91	1.52	0.61	0.58	1.45	0.72	1.81	2.01	2.33	2.32	1.63	0.52	2.08	0.55
0.76	1.84	2.26	1.65	2.98	1.52	0.61	0.58	1.45	0.78	2.13	2.01	2.33	2.59	1.64	0.63	2.08	0.58
0.91	1.84	2.45	1.65	3	1.52	0.84	0.58	1.45	1.05	2.24	2.01	2.38	2.6	1.64	0.82	2.08	0.58
1.1	1.84	2.53	1.84	3	1.52	0.84	0.58	1.57	1.12	2.24	2.01	2.7	2.6	1.64	0.82	2.08	0.6
1.1	1.94	2.53	1.84	3.27	1.52	0.84	0.58	1.57	1.12	2.24	2.01	2.7	2.6	1.8	0.82	2.08	0.63
1.1	2.14	2.53	1.84	3.29	1.52	0.97	0.98	1.57	1.12	2.24	2.12	2.7	2.6	1.8	0.82	2.08	0.64
1.1	2.47	2.53	2.07	3.29	1.75	0.97	1.07	1.96	1.12	2.32	2.47	2.7	2.6	1.8	0.82	2.08	0.64
1.34	2.47	2.54	2.07	3.29	1.75	0.97	1.07	1.96	1.12	2.43	2.47	2.7	2.78	1.8	0.82	2.08	0.64
1.38	2.47	2.54	2.2	3.5	1.75	0.97	1.07	1.96	1.12	2.58	2.47	2.75	2.78	1.87	0.82	2.08	0.64
1.42	2.47	2.83	2.2	3.59	1.75	0.97	1.07	1.96	1.12	2.79	2.47	3	2.78	1.99	0.82	2.49	0.64
1.42	2.47	2.83	2.2	3.59	1.75	1.04	1.07	1.96	1.12	2.91	2.47	3	2.99	1.99	0.82	2.49	0.64
1.42	2.47	2.83	2.38	3.84	1.89	1.04	1.24	2.15	1.12	2.91	2.47	3	3.04	1.99	0.95	2.49	0.64
1.42	2.47	2.83	2.38	3.98	2.25	1.04	1.24	2.15	1.12	2.91	2.47	3	3.06	1.99	0.97	2.49	0.64
1.42	2.47	3.09	2.51	4.11	2.26	1.08	1.42	2.15	1.12	2.91	2.76	3	3.06	2.06	1.03	2.77	0.68
1.43	2.47	3.24	2.9	4.12	2.26	1.3	1.42	2.15	1.12	3.11	2.76	3.5	3.06	2.48	1.03	2.77	0.68
1.77	2.47	3.25	2.91	4.12	2.26	1.3	1.63	2.15	1.12	3.31	2.76	3.5	3.06	2.6	1.03	2.77	0.68
1.77	2.87	3.25	2.91	4.24	2.26	1.37	1.63	2.23	1.12	3.31	2.76	3.5	3.08	2.6	1.03	3.04	0.69
1.77	2.9	3.26	2.91	4.28	2.57	1.37	1.63	2.29	1.12	3.31	2.76	3.5	3.19	2.6	1.08	3.04	0.69
1.77	2.9	3.26	2.91	4.28	2.57	1.37	1.63	2.55	1.12	3.31	3.2	3.5	3.19	2.6	1.08	3.04	0.69
2.24	2.9	3.56	2.91	4.28	2.57	1.37	1.98	2.81	1.12	3.31	3.2	3.5	3.19	2.6	1.08	3.04	0.69
2.24	2.9	3.56	3.09	4.28	2.57	1.38	2.2	2.86	1.12	3.31	3.2	3.51	3.19	2.6	1.08	3.04	0.8
2.24	2.9	3.56	3.24	4.28	2.57	1.39	2.2	2.86	1.12	3.31	3.2	3.51	3.19	2.77	1.1	3.32	0.81
2.24	3.21	3.56	3.24	4.28	2.73	1.45	2.2	2.86	1.12	3.44	3.2	3.51	3.3	2.89	1.1	3.32	0.81
2.24	3.21	3.56	3.24	4.43	2.75	1.45	2.2	2.86	1.12	3.44	3.21	3.51	3.3	2.89	1.1	3.32	0.81
2.24	3.21	3.56	3.24	4.43	2.75	1.45	2.2	2.86	1.12	3.44	3.21	3.53	3.3	2.89	1.1	3.32	0.81
2.48	3.21	3.56	3.24	4.43	2.75	1.45	2.2	2.86	1.12	3.46	3.21	3.53	3.3	2.89	1.1	3.35	0.81
2.49	3.21	3.56	3.41	4.43	2.75	1.45	2.27	2.88	1.12	3.65	3.53	3.53	3.45	2.89	1.28	3.35	0.82
2.49	3.21	3.56	3.41	4.43	2.75	1.45	2.32	2.88	1.12	3.65	3.6	3.55	3.57	2.89	1.32	3.35	0.82
2.49	3.21	3.71	3.41	4.43	2.99	1.54	2.32	2.88	1.12	3.65	3.6	3.69	3.57	2.89	1.32	3.35	0.9
2.5	3.21	3.71	3.41	4.43	2.99	1.54	2.32	2.88	1.12	3.65	3.6	3.69	3.57	3.03	1.32	3.35	0.91
2.79	3.21	3.72	3.41	4.51	2.99	1.54	2.32	2.89	1.12	3.65	3.6	3.69	3.57	3.17	1.32	3.35	0.91
2.92	3.21	3.72	3.49	4.51	3	1.54	2.49	3.17	1.12	3.65	3.6	3.78	3.59	3.17	1.32	3.46	0.91
2.99	3.8	3.72	3.59	4.51	3.19	1.54	2.49	3.17	1.12	3.65	3.6	3.87	3.59	3.17	1.32	3.49	0.91
2.99	3.84	3.84	3.59	4.51	3.19	1.54	2.49	3.17	1.12	3.65	3.6	3.87	3.59	3.17	1.44	3.49	0.91
3	3.84	3.84	3.59	4.53	3.19	1.54	2.49	3.17	1.12	3.65	3.6	3.87	3.59	3.29	1.44	3.49	1
3	3.84	3.84	3.59	4.73	3.19	1.58	2.49	3.17	1.18	3.79	3.6	3.87	3.59	3.29	1.44	3.5	1
3.02	3.84	3.84	3.73	4.73	3.19	1.58	2.78	3.44	1.3	3.79	3.67	4.08	3.59	3.29	1.44	3.5	1
3.34	3.84	4.09	3.75	4.73	3.48	1.59	2.78	3.44	1.3	3.79	3.67	4.12	3.59	3.29	1.44	3.5	1
3.37	3.84	4.1	3.75	4.73	3.49	1.59	2.78	3.44	1.3	3.79	3.67	4.12	3.59	3.29	1.44	3.5	1.01
3.37	3.84	4.1	3.75	4.85	3.49	1.6	2.78	3.44	1.3	3.79	3.85	4.12	3.82	3.4	1.51	3.57	1.01
3.53	3.84	4.11	3.94	4.85	3.49	1.73	2.78	3.44	1.3	3.85	3.97	4.31	3.93	3.41	1.57	3.57	1.03
3.7	4.19	4.11	3.95	4.98	3.49	1.74	2.78	3.44	1.3	3.99	3.97	4.51	3.93	3.41	1.57	3.57	1.13
3.7	4.19	4.12	3.95	5.05	3.49	1.74	2.92	3.44	1.3	3.99	3.97	4.57	3.93	3.5	1.57	3.57	1.13
3.73	4.19	4.37	3.95	5.05	3.88	1.74	2.92	3.7	1.3	3.99	3.97	4.79	3.93	3.77	1.57	3.57	1.13
3.8	4.19	4.37	4.11	5.2	3.88	1.74	2.92	3.7	1.3	3.99	4.24	4.79	4.17	3.77	1.57	3.67	1.13
3.8	4.19	4.38	4.11	5.25	3.88	1.89	3.15	3.7	1.3	4.2	4.24	4.89	4.22	3.77	1.57	3.68	1.13
3.82	4.71	4.38	4.11	5.25	3.89	1.89	3.15	3.7	1.3	4.2	4.24	4.98	4.22	3.77	1.57	3.68	1.2
3.98	4.71	4.6	4.27	5.38	3.89	1.89	3.15	3.75	1.51	4.2	4.24	4.98	4.22	3.77	1.64	3.68	1.2
4.23	4.71	4.73	4.39	5.53	3.89	1.9	3.15	3.75	1.52	4.48	4.24	5.3	4.48	4.01	1.71	3.68	1.2
4.23	4.71	4.78	4.42	5.76	3.91	2.08	3.15	4.04	1.52	4.48	4.55	5.34	4.48	4.01	1.71	3.68	1.21
4.29	4.71	4.98	4.69	5.8	4.29	2.08	3.43	4.07	1.52	4.82	4.55	5.49	4.48	4.24	1.92	3.68	1.21
4.45	4.74	5.22	4.69	6.05	4.29	2.24	3.43	4.07	1.55	4.98	4.91	5.74	4.71	4.24	1.94	3.68	1.28
4.45	4.91	5.22	4.69	6.05	4.29	2.24	3.43	4.48	1.72	5.08	4.91	5.89	4.71	4.42	1.94	3.72	1.3
4.77	4.91	5.39	4.93	6.28	4.53	2.33	3.57	4.51	1.72	5.1	4.97	6.12	4.98	4.42	1.94	3.72	1.48
4.82	5.57	5.51	5.02	6.28	4.82	2.39	3.79	4.81	1.85	5.23	5.01	6.39	5.05	4.42	1.94	3.76	1.49
4.82	5.57	5.51	5.02	6.49	4.82	2.39	3.88	4.81	1.85	5.23	5.38	6.39	5.36	4.53	2.16	3.86	1.49
5.16	5.57	5.92	5.31	6.49	4.82	2.58	3.96	5.13	1.99	5.23	5.38	6.39	5.57	4.53	2.16	3.99	1.66
5.16	5.8	6.13	5.32	6.78	4.89	2.58	4.19	5.13	1.99	5.4	5.38	6.61	5.8	4.66	2.21	4.04	1.66
5.16	5.8	6.13	5.52	6.78	5.06	2.69	4.19	5.21	2.57	5.6	5.5	6.75	6.01	4.66	2.34	4.17	1.68
5.16	5.8	6.21	5.74	7.02	5.35	2.75	4.39	5.39	2.8	5.94	5.78	6.98	6.41	4.69	2.41	4.39	1.72
5.16	5.8	6.3	5.74	7.23	5.35	3	4.42	5.39	2.8	6.06	5.78	6.98	6.42	4.93	2.52	4.57	1.72
5.16	5.8	6.36	5.99	7.49	5.44	3	4.61	5.39	2.98	6.38	5.78	6.98	6.84	5.08	2.52	4.67	1.72
5.35	5.8	6.51	5.99	7.74	5.44	3.31	4.93	5.68	3.16	6.38	6.2	6.98	7.02	5.09	2.52	4.92	2.06
5.4	5.8	6.51	5.99	7.75	5.75	3.31	4.93	5.69	3.19	6.56	6.23	7.38	7.02	5.18	2.52	4.96	2.06
5.4	5.8	6.51	5.99	7.96	5.76	3.31	5.26	6	3.51	6.56	6.24	7.39	7.02	5.18	2.62	5.08	2.07
5.41	6.17	6.72	6.22	8.16	5.76	3.31	5.26	6.02	3.52	6.56	6.24	7.39	7.17	5.32	2.73	5.26	2.07
5.45	6.17	6.72	6.22	8.16	5.76	3.31	5.26	6.02	3.52	6.79	6.24	7.39	7.22	5.32	2.73	5.45	2.07
5.75	6.17	6.72	6.22	8.16	5.94	3.31	5.26	6.27	3.86	7.02	6.24	7.47	7.47	5.35	2.73	5.45	2.07
5.75	6.17	6.87	6.22	8.17	5.94	3.33	5.35	6.27	3.86	7.09	6.24	7.47	7.47	5.35	2.9	5.45	2.43
5.75	6.17	6.94	6.22	8.17	5.94	3.6	5.48	6.27	3.86	7.09	6.36	7.47	7.47	5.49	2.9	5.45	2.46
5.76	6.17	6.95	6.23	8.22	5.94	3.6	5.48	6.27	3.86	7.26	6.51	7.83	7.5	5.49	2.9	5.45	2.49
5.76	6.17	6.95	6.23	8.47	5.94	3.6	5.48	6.27	3.86	7.38	6.51	7.83	7.58	5.49	3.3	5.52	2.71
5.76	6.17	7.19	6.23	8.47	5.94	3.6	5.76	6.27	3.86	7.38	6.51	7.83	7.58	5.49	3.4	5.8	2.84
5.76	6.17	7.19	6.23	8.47	5.94	3.83	5.76	6.57	3.86	7.38	6.51	7.83	7.58	5.49	3.4	6.06	2.84
5.76	6.17	7.2	6.23	8.47	6.32	3.83	5.76	6.57	3.86	7.38	6.51	7.83	7.79	5.49	3.4	6.12	2.84
5.76	6.17	7.42	6.23	8.47	6.32	3.83	6.02	6.57	4.19	7.49	6.72	7.93	8.05	5.59	3.4	6.12	2.84
5.76	6.53	7.42	6.39	8.53	6.32	3.89	6.04	6.57	4.19	7.49	6.79	8.09	8.05	5.63	3.4	6.12	2.84
6.05	6.53	7.62	6.39	8.84	6.32	3.9	6.04	6.62	4.19	7.49	6.79	8.09	8.05	5.63	3.56	6.12	2.84
6.05	6.53	7.67	6.39	8.84	6.32	3.9	6.04	6.65	4.19	7.76	6.79	8.09	8.05	5.68	3.56	6.12	2.84
6.05	6.53	7.71	6.39	8.84	6.32	3.91	6.2	6.65	4.19	7.76	6.79	8.09	8.05	5.69	3.56	6.12	2.85
6.05	6.53	7.71	6.64	8.84	6.51	4.07	6.2	6.84	4.48	7.76	6.79	8.26	8.05	5.84	3.56	6.12	3.06
6.05	6.85	7.71	6.64	8.91	6.6	4.07	6.2	6.84	4.54	7.76	6.79	8.26	8.35	5.92	3.56	6.27	3.23
6.37	6.85	7.71	6.64	8.91	6.69	4.07	6.2	6.89	4.54	8.03	6.79	8.26	8.35	5.92	3.69	6.37	3.23
6.66	6.85	7.9	6.64	8.91	6.69	4.08	6.55	6.89	4.54	8.03	6.79	8.26	8.35	5.92	3.87	6.37	3.23
6.66	6.85	7.9	6.72	8.91	6.69	4.42	6.55	7.07	4.54	8.03	7.13	8.47	8.35	5.92	3.9	6.37	3.23
6.66	6.85	7.9	6.9	8.91	6.69	4.42	6.55	7.14	4.54	8.03	7.13	8.47	8.35	5.92	3.9	6.38	3.23
6.66	6.85	7.9	6.91	9.11	6.69	4.43	6.6	7.17	4.54	8.03	7.13	8.47	8.41	5.92	3.9	6.38	3.23
6.66	6.85	7.9	6.91	9.11	6.69	4.43	6.62	7.17	4.54	8.04	7.13	8.47	8.42	5.98	3.9	6.38	3.23
6.78	6.85	7.9	6.91	9.11	6.87	4.43	6.62	7.17	4.54	8.04	7.13	8.47	8.42	6.19	3.9	6.38	3.23
6.78	6.85	7.9	6.98	9.11	7.04	4.43	6.62	7.17	4.54	8.04	7.13	8.59	8.42	6.21	4.02	6.46	3.26
6.78	6.85	7.91	7.25	9.24	7.04	4.56	6.62	7.17	4.54	8.04	7.13	8.59	8.51	6.21	4.08	6.46	3.26
6.78	6.85	8.03	7.25	9.28	7.04	4.56	6.62	7.17	4.54	8.23	7.13	8.59	8.7	6.21	4.08	6.46	3.26
6.78	7.12	8.03	7.25	9.28	7.04	4.56	6.63	7.17	4.6	8.26	7.13	8.59	8.7	6.34	4.08	6.46	3.26
6.78	7.12	8.03	7.25	9.28	7.04	4.56	6.82	7.17	4.6	8.26	7.13	8.69	8.7	6.34	4.08	6.46	3.26
6.78	7.12	8.03	7.36	9.28	7.04	4.56	6.82	7.19	4.6	8.26	7.35	8.69	8.7	6.34	4.08	6.47	3.56

7.01	7.12	8.03	.	.	7.04	4.56	6.82	7.2	4.6	8.26	.	.	8.97	6.34	4.08	6.51	3.57
7.45	7.12	8.16	.	.	7.04	4.88	6.95	7.2	4.6	8.41	.	.	8.97	6.34	4.29	6.51	3.57
7.45	7.12	8.16	.	.	7.04	4.88	6.95	7.2	4.6	8.41	.	.	8.97	6.49	4.29	6.51	3.57
7.45	7.12	.	.	.	7.04	4.88	6.95	7.2	4.6	.	.	.	8.97	6.49	4.29	6.51	3.57
7.45	7.12	.	.	.	7.04	4.88	6.95	7.5	4.6	.	.	.	8.97	6.49	4.29	6.51	3.57
7.45	7.12	.	.	.	7.04	4.88	6.95	7.5	4.6	.	.	.	8.97	6.49	4.29	6.51	3.57
7.45	7.12	.	.	.	7.19	4.92	7.25	7.5	4.6	.	.	.	9.15	6.51	4.29	6.51	3.57
7.45	7.26	.	.	.	7.42	5.1	7.25	7.5	4.6	.	.	.	9.26	6.69	4.57	6.51	3.57
7.78	7.57	.	.	.	7.42	5.1	7.25	7.5	4.6	.	.	.	9.26	6.7	4.57	6.53	3.59
8.03	7.57	.	.	.	7.42	5.1	7.25	7.6	4.6	.	.	.	9.26	6.7	4.57	6.53	3.78
8.03	7.57	.	.	.	7.51	5.1	7.25	7.72	4.91	.	.	.	9.26	6.7	4.62	6.53	3.78
8.03	7.57	.	.	.	7.51	5.11	7.56	7.72	5.07	.	.	.	9.72	6.7	4.62	6.53	3.78
8.4	7.57	.	.	.	7.51	5.11	7.56	7.72	5.07	.	.	.	9.73	7	4.62	6.54	3.78
8.48	7.57	.	.	.	7.72	5.2	7.56	7.72	5.07	.	.	.	9.73	7	4.83	6.58	3.79
8.48	7.57	.	.	.	7.72	5.35	7.65	7.78	5.07	.	.	.	9.73	7.17	4.83	6.6	3.82
8.48	7.57	.	.	.	7.72	5.35	7.81	8.11	5.07	.	.	.	9.73	7.17	4.83	6.6	4.3
8.63	7.96	.	.	.	7.9	5.35	7.81	8.11	5.07	.	.	.	9.73	7.17	4.9	6.61	4.3
8.63	7.97	.	.	.	7.9	5.39	7.81	8.11	5.32	.	.	.	10	7.17	5.05	6.61	4.3
8.66	7.97	.	.	.	7.9	5.62	7.95	8.11	5.32	.	.	.	10.01	7.17	5.05	6.62	4.3
8.76	7.97	.	.	.	8.03	5.62	7.95	8.46	5.46	.	.	.	10.37	7.28	5.22	6.62	4.3
8.77	8.45	.	.	.	8.11	5.91	7.95	8.46	5.46	.	.	.	10.37	7.37	5.22	6.64	4.3
8.84	8.45	.	.	.	8.37	5.91	8.26	8.72	5.68	.	.	.	10.68	7.5	5.32	6.64	4.55
8.98	8.45	.	.	.	8.37	6.3	8.27	8.8	5.91	.	.	.	10.82	7.75	5.6	6.74	4.56
9.25	8.45	.	.	.	8.61	6.34	8.27	9.03	5.91	.	.	.	11	7.75	5.66	6.99	4.56
9.29	8.45	.	.	.	8.63	6.34	8.5	9.03	5.92	.	.	.	11	7.9	5.76	7.17	4.86
9.63	8.45	.	.	.	8.63	6.34	8.81	9.03	6.22	.	.	.	11.28	8	5.76	7.46	5.07
9.66	8.48	.	.	.	8.77	6.5	8.95	9.24	6.23	.	.	.	11.28	8	5.76	7.56	5.07
10.12	8.48	.	.	.	9.11	6.5	8.95	9.25	6.64	.	.	.	11.68	8.07	6.12	7.56	5.29
10.13	9	.	.	.	9.11	6.5	8.95	9.25	6.64	.	.	.	11.79	8.25	6.12	7.71	5.41
10.16	9	.	.	.	9.11	6.5	9.31	9.62	6.94	.	.	.	11.79	8.29	6.12	7.71	5.41
10.16	9	.	.	.	9.11	6.56	9.31	9.67	7.16	.	.	.	11.93	8.41	6.12	7.78	5.41
10.18	9	.	.	.	9.45	6.56	9.64	9.67	7.16	.	.	.	11.97	8.53	6.23	8.21	5.48
10.65	9	.	.	.	9.45	6.56	9.64	9.67	7.2	.	.	.	12.08	8.53	6.38	8.37	5.84
10.65	9	.	.	.	9.69	6.66	9.64	9.67	7.25	.	.	.	12.19	8.79	6.44	8.44	6.04
10.65	9	.	.	.	9.74	6.77	9.64	9.67	7.28	.	.	.	12.49	8.79	6.44	8.48	6.22
10.65	9	.	.	.	9.74	6.94	9.76	10.02	7.28	.	.	.	12.65	8.79	6.44	8.87	6.22
10.65	9.47	.	.	.	9.74	6.95	10.03	10.02	7.48	.	.	.	12.65	8.88	6.44	8.94	6.22
10.65	9.48	.	.	.	9.74	6.95	10.07	10.02	7.48	.	.	.	12.65	8.92	6.6	9	6.22
10.65	9.48	.	.	.	9.74	6.95	10.07	10.32	7.48	.	.	.	12.65	8.96	6.73	9.28	6.22
10.94	9.48	.	.	.	9.74	6.95	10.07	10.36	7.48	.	.	.	13.12	8.98	6.73	9.28	6.22
10.94	9.48	.	.	.	9.78	6.95	10.07	10.36	7.48	.	.	.	13.26	8.98	6.73	9.28	6.56
10.94	9.48	.	.	.	9.78	6.95	10.07	10.61	7.69	.	.	.	13.39	8.98	6.73	9.28	6.56
10.94	9.48	.	.	.	9.99	6.95	10.07	10.61	7.69	.	.	.	13.39	9.21	6.75	9.28	6.56
10.94	9.48	.	.	.	9.99	6.95	10.07	10.61	7.69	.	.	.	13.39	9.21	6.75	9.28	6.56
10.94	9.98	.	.	.	9.99	6.95	10.07	10.62	7.69	.	.	.	13.73	9.37	6.75	9.28	6.57
11	9.98	.	.	.	9.99	6.95	10.45	10.62	7.69	.	.	.	13.73	9.41	6.75	9.6	6.57
11	9.98	.	.	.	10.27	6.95	10.46	10.62	7.69	.	.	.	13.73	9.41	7.07	9.6	6.57
11	9.98	.	.	.	10.27	6.95	10.46	10.62	7.74	.	.	.	13.73	9.58	7.07	9.6	6.9
11	9.98	.	.	.	10.27	7.06	10.46	10.76	7.96	.	.	.	13.73	9.58	7.07	9.6	6.99
11	9.98	.	.	.	10.27	7.16	10.46	10.95	7.96	.	.	.	13.73	9.58	7.07	9.6	7.04
11.14	10.08	.	.	.	10.27	7.16	10.69	10.95	7.96	.	.	.	14.08	9.59	7.07	9.6	7.04
11.29	10.08	.	.	.	10.32	7.16	10.69	11.21	7.96	.	.	.	14.1	9.59	7.07	9.95	7.04
11.29	10.08	.	.	.	10.45	7.16	10.83	11.21	7.99	.	.	.	14.1	9.66	7.13	9.95	7.04
11.29	10.08	.	.	.	10.7	7.17	10.83	11.21	8.16	.	.	.	14.1	9.8	7.25	9.95	7.04
11.37	10.08	.	.	.	10.71	7.18	10.83	11.21	8.16	.	.	.	14.16	9.8	7.25	9.95	7.04
11.37	10.08	.	.	.	10.71	7.42	10.83	11.21	8.16	.	.	.	14.16	9.8	7.25	9.95	7.04
11.37	10.08	.	.	.	10.71	7.42	10.83	11.21	8.16	.	.	.	14.18	9.8	7.25	10.36	7.04
11.37	10.08	.	.	.	10.71	7.42	10.83	11.21	8.17	.	.	.	14.18	9.8	7.25	10.36	7.04
11.37	10.08	.	.	.	10.9	7.43	10.83	11.21	8.24	.	.	.	14.18	9.92	7.33	10.36	7.04
11.37	10.08	.	.	.	10.91	7.43	10.83	11.44	8.24	.	.	.	14.18	9.92	7.33	10.36	7.04
11.37	10.08	.	.	.	10.91	7.53	10.83	11.44	8.39	.	.	.	14.18	9.92	7.33	10.36	7.13
11.59	10.08	.	.	.	10.91	7.53	10.83	11.44	8.39	.	.	.	14.18	9.92	7.33	10.37	7.26
11.63	10.08	.	.	.	10.91	7.53	10.83	11.53	8.39	.	.	.	14.18	9.92	7.33	10.37	7.26
11.66	10.08	.	.	.	10.91	7.53	10.85	11.7	8.39	.	.	.	14.18	10.05	7.33	10.57	7.26
11.73	10.08	.	.	.	10.91	7.53	10.85	11.7	8.59	.	.	.	14.18	10.07	7.41	10.99	7.26
11.78	10.08	.	.	.	10.92	**7.65**	**10.85**	11.7	8.59	.	.	.	14.18	**10.07**	**7.48**	**10.99**	**7.29**

0	0	0	0	0	0	**0**	**0**	0	0	0	0	0	0	**0**	**0**	0.69	0.34
0.25	0.01	0.01	0.15	0.14	0	0.11	0.12	0	0.08	0.11	0	0.17	0.44	0.03	0.2	0.7	0.6
0.34	0.36	0.16	0.19	0.14	0.27	0.15	0.33	0.22	0.32	0.26	0.19	0.23	0.44	0.18	0.27	1.05	0.6
0.41	0.36	0.17	0.38	0.14	0.27	0.18	0.44	0.35	0.32	0.44	0.19	0.5	0.44	0.27	0.27	1.07	0.74
0.49	0.66	0.19	0.46	0.14	0.28	0.33	0.44	0.35	0.32	0.44	0.19	0.56	0.7	0.35	0.51	1.07	0.89
0.51	0.66	0.39	0.46	0.59	0.64	0.45	0.74	0.35	0.59	0.73	0.37	0.57	1.1	0.5	0.57	1.07	0.89
0.94	0.82	0.4	0.61	0.59	0.64	0.64	0.74	0.35	0.59	0.74	0.58	0.85	1.1	0.5	0.7	1.4	0.89
1.03	1.19	0.4	0.61	0.59	0.64	0.64	0.74	0.67	0.59	0.74	0.59	0.85	1.1	0.77	0.92	1.41	0.95
1.03	1.19	0.61	0.77	0.95	0.65	0.84	1.11	0.67	0.78	1.21	0.78	1.12	1.1	0.87	1.01	1.41	1.15
1.03	1.19	0.61	0.77	0.95	0.82	0.93	1.11	0.67	0.78	1.21	0.78	1.12	1.11	0.87	1.09	1.49	1.15
1.2	1.48	0.61	0.77	0.95	1.03	0.93	1.11	1.13	0.85	1.21	0.83	1.12	1.28	0.87	1.15	1.84	1.15
1.25	1.48	0.76	0.83	0.97	1.03	1.11	1.42	1.13	0.98	1.21	1.03	1.12	1.28	0.95	1.54	1.84	1.17
1.38	1.48	0.76	0.97	1.17	1.03	1.27	1.42	1.13	0.98	1.4	1.03	1.43	1.28	0.95	1.55	1.84	1.58
1.45	1.48	0.84	1.07	1.17	1.34	1.27	1.42	1.56	1.17	1.43	1.03	1.43	1.28	1.01	1.55	1.85	1.58
1.51	1.84	1.06	1.07	1.48	1.34	1.28	1.42	1.56	1.31	1.76	1.1	1.43	1.38	1.01	1.58	1.85	1.7
1.56	1.84	1.31	1.26	1.48	1.34	1.38	1.69	1.56	1.44	1.8	1.33	1.63	1.38	1.11	1.87	2.24	1.7
1.58	1.84	1.38	1.56	1.57	1.56	1.63	1.69	1.56	1.47	1.8	1.33	1.63	1.54	1.11	1.87	2.24	1.81
1.59	2.15	1.39	1.56	1.57	1.57	1.64	1.99	1.56	1.68	2.17	1.33	1.76	1.57	1.25	2.2	2.24	2.24
1.88	2.15	1.61	1.56	1.57	1.57	1.97	1.99	1.84	1.93	2.17	1.49	1.76	2.13	1.33	2.3	2.24	2.24
1.88	2.15	1.91	1.93	1.99	1.58	1.97	1.99	1.94	1.94	2.17	1.49	2.12	2.14	1.38	2.3	2.24	2.24
1.88	2.15	1.91	1.93	1.99	1.79	1.98	1.99	1.96	1.94	2.17	1.64	2.14	2.14	1.46	2.3	2.24	2.24
1.88	2.66	1.91	1.93	1.99	1.79	1.99	2.35	1.96	2.25	2.4	1.65	2.14	2.69	1.68	2.47	2.24	2.24
1.88	2.66	2.2	2.33	1.99	2.03	2.29	2.35	1.99	2.3	2.71	2.04	2.14	2.69	1.68	2.54	2.82	2.24
1.88	2.66	2.21	2.33	2.35	2.03	2.31	2.35	1.99	2.3	2.71	2.04	2.28	2.69	1.78	2.54	2.82	2.56
1.92	2.66	2.21	2.33	2.35	2.03	2.31	2.45	1.99	2.48	2.71	2.04	2.28	2.69	1.81	2.78	2.82	2.56
1.92	2.66	2.21	2.33	2.35	2.31	2.31	2.6	2.07	2.53	2.98	2.04	2.41	2.69	1.81	2.95	2.82	2.56
1.93	2.66	2.35	2.44	2.67	2.31	2.74	2.94	2.07	2.53	2.98	2.04	2.44	2.69	1.81	2.95	2.82	2.56
1.96	2.66	2.35	2.44	2.67	2.31	2.74	2.94	2.07	2.76	2.98	2.04	2.44	2.69	2.15	2.95	2.84	2.56
2.42	2.84	2.4	2.44	2.67	2.31	2.74	2.94	2.09	2.76	2.98	2.04	2.44	2.69	2.15	2.97	2.93	2.56
2.42	2.84	2.4	2.44	2.72	2.48	2.74	2.94	2.62	2.76	3.08	2.04	2.72	2.69	2.51	3.11	2.93	2.56
2.42	2.84	2.43	2.44	2.72	2.48	2.95	3.07	2.62	2.76	3.08	2.35	2.72	2.69	2.51	3.11	2.93	2.56
2.42	2.96	2.43	2.44	2.8	2.48	2.95	3.07	2.62	2.76	3.08	2.35	2.99	2.69	2.51	3.11	3.34	2.56
2.44	2.96	2.49	2.44	2.89	2.48	2.95	3.07	2.76	2.76	3.11	2.35	3	2.69	2.51	3.27	3.51	2.96
2.44	2.96	2.49	2.72	2.9	2.48	2.95	3.07	2.76	2.87	3.11	2.35	3	2.69	2.51	3.34	3.51	2.96
2.81	2.96	2.54	2.72	2.9	2.66	2.98	3.24	2.76	2.88	3.25	2.35	3.25	2.69	2.51	3.34	3.51	2.96
2.83	2.96	2.54	2.72	2.9	2.66	2.98	3.24	2.76	2.88	3.26	2.6	3.25	2.69	2.7	3.67	3.51	2.96
2.83	2.96	2.86	2.72	3.14	2.66	2.98	3.24	2.76	2.88	3.26	2.62	3.25	2.69	2.7	3.68	3.51	2.96
2.85	2.99	2.86	2.97	3.15	3.04	3.05	3.25	2.76	3.42	3.26	2.62	3.25	2.69	2.7	3.68	3.51	2.96
2.85	2.99	2.86	2.97	3.15	3.04	3.05	3.32	2.76	3.42	3.26	2.62	3.55	2.69	2.7	3.69	3.51	2.96
2.85	3.08	3.11	2.97	3.48	3.04	3.06	3.32	2.93	3.42	3.26	2.65	3.55	2.69	2.7	3.69	3.51	3.14
2.85	3.08	3.11	2.97	3.48	3.05	3.06	3.47	2.95	3.42	3.49	2.66	3.79	2.98	2.82	3.87	3.51	3.15
2.85	3.13	3.27	2.97	3.48	3.05	3.12	3.47	3.09	3.42	3.49	2.84	3.79	2.98	2.82	3.89	3.51	3.15
2.85	3.13	3.3	2.97	3.78	3.05	3.18	3.47	3.09	3.42	3.68	2.84	3.79	2.98	3.14	3.89	3.51	3.15
2.85	3.31	3.3	2.97	3.78	3.58	3.18	3.7	3.35	3.42	3.68	2.84	3.79	2.98	3.14	3.89	3.65	3.17
3.06	3.31	3.5	2.97	3.85	3.58	3.18	3.7	3.35	3.42	3.97	3.08	3.79	3.13	3.15	3.91	3.65	3.53
3.17	3.31	3.5	2.98	3.85	3.58	3.18	3.7	3.35	3.7	3.97	3.08	3.79	3.13	3.15	4.07	3.86	3.53
3.17	3.31	3.67	2.98	3.85	3.58	3.25	3.81	3.54	3.71	3.97	3.08	3.79	3.13	3.15	4.07	3.98	3.53
3.17	3.31	3.67	3.06	3.85	3.58	3.25	3.81	3.54	3.71	3.97	3.18	3.79	3.5	3.6	4.07	3.98	3.53
3.39	3.71	3.68	3.06	4.19	4.04	3.29	3.81	3.54	3.71	4.34	3.44	4.05	3.5	3.6	4.33	3.98	3.53
3.39	3.71	3.68	3.44	4.24	4.04	3.37	4.12	3.73	3.71	4.34	3.47	4.05	3.5	3.6	4.34	4.2	3.53
3.39	3.71	3.92	3.44	4.24	4.04	3.41	4.12	3.73	3.71	4.34	3.47	4.05	3.88	3.77	4.34	4.2	3.58
3.39	4.01	3.93	3.44	4.25	4.04	3.43	4.12	3.73	3.83	4.34	3.52	4.05	3.88	3.77	4.34	4.2	3.58
3.39	4.02	3.93	3.68	4.25	4.04	3.43	4.12	3.8	3.83	4.34	3.52	4.06	3.97	3.8	4.34	4.21	3.58
3.39	4.02	3.94	3.68	4.25	4.34	3.49	4.25	3.83	3.83	4.37	3.75	4.06	4.19	3.8	4.34	4.21	3.64

3.39	4.26	4.21	3.68	4.47	4.34	3.5	4.25	3.83	3.83	4.37	3.75	4.27	4.19	3.8	4.47	4.24	3.95
3.39	4.26	4.22	3.68	4.58	4.34	3.5	4.25	4.06	3.9	4.37	3.75	4.27	4.19	3.8	4.47	4.24	3.95
3.39	4.26	4.22	3.68	4.58	4.34	3.5	4.45	4.06	4.15	4.37	3.91	4.27	4.19	3.8	4.47	4.25	3.95
3.39	4.26	4.23	3.68	4.58	4.34	3.56	4.45	4.06	4.15	4.37	3.91	4.27	4.66	3.8	4.57	4.25	3.95
3.39	4.27	4.57	3.7	4.58	4.35	3.56	4.45	4.13	4.15	4.37	3.92	4.64	4.66	3.8	4.78	4.27	4.21
3.39	4.63	4.58	3.7	4.87	4.35	3.6	4.46	4.26	4.15	4.37	4.08	4.64	4.66	3.8	4.78	4.27	4.21
3.39	4.63	4.58	3.7	4.87	4.36	3.6	4.71	4.26	4.15	4.37	4.08	4.64	4.66	3.81	4.78	4.53	4.21
3.61	4.64	4.59	3.7	4.87	4.36	3.6	4.71	4.26	4.15	4.37	4.08	4.83	5.17	3.82	4.81	4.53	4.21
3.61	4.64	4.59	3.74	4.87	4.36	3.6	4.71	4.26	4.15	4.39	4.08	4.83	5.17	3.82	4.81	4.53	4.21
3.63	4.65	4.6	3.74	4.87	4.36	3.62	4.85	4.38	4.16	4.39	4.08	4.83	5.17	3.82	4.81	4.54	4.21
3.63	4.65	4.6	3.74	4.9	4.36	3.74	4.85	4.38	4.16	4.39	4.32	5.04	5.17	3.91	4.81	4.56	4.21
3.66	4.65	4.61	3.91	5.03	4.51	3.74	4.85	4.38	4.16	4.45	4.32	5.17	5.17	3.92	4.83	4.56	4.21
3.66	4.65	4.63	4	5.03	4.51	3.74	4.85	4.69	4.16	4.45	4.32	5.17	5.17	3.93	4.83	4.56	4.78
3.82	4.68	4.65	4	5.03	4.51	3.8	4.85	4.69	4.49	4.55	4.32	5.17	5.17	4.06	4.83	4.56	4.78
3.82	4.68	4.66	4.07	5.22	4.51	3.8	4.85	4.69	4.49	4.55	4.51	5.18	5.17	4.07	4.84	4.65	4.78
3.82	4.68	4.75	4.17	5.35	4.84	3.8	5.18	4.97	4.49	4.55	4.52	5.56	5.17	4.07	4.85	4.65	4.78
4.08	4.76	4.75	4.17	5.37	4.84	3.81	5.18	5.11	4.49	4.66	4.52	5.56	5.17	4.07	4.96	4.65	4.8
4.08	4.76	4.76	4.24	5.37	4.84	3.98	5.18	5.11	4.49	4.66	5.12	5.57	5.42	4.07	5.01	4.89	5.18
4.08	4.84	5.03	4.24	5.37	4.84	3.98	5.18	5.11	4.49	4.74	5.13	5.89	5.42	4.07	5.2	4.89	5.18
4.64	5.08	5.04	4.51	5.72	4.85	3.98	5.33	5.11	4.49	4.82	5.13	5.89	5.42	4.07	5.2	4.89	5.18
4.64	5.08	5.04	4.52	5.72	5.04	4.28	5.75	5.15	4.49	4.82	5.13	6.24	5.98	4.07	5.2	5.03	5.19
4.64	5.21	5.04	4.65	5.74	5.04	4.28	5.76	5.16	4.53	4.82	5.13	6.24	5.98	4.32	5.2	5.19	5.19
4.91	5.21	5.05	4.65	5.92	5.05	4.28	5.76	5.16	4.58	5.01	5.13	6.24	5.98	4.32	5.35	5.19	5.19
4.93	5.22	5.06	4.65	5.92	5.05	4.28	5.76	5.39	4.63	5.01	5.21	6.33	5.98	4.32	5.35	5.19	5.19
5.21	5.37	5.16	4.65	5.92	5.37	4.46	5.76	5.45	4.63	5.01	5.21	6.33	5.98	4.32	5.72	5.19	5.21
5.21	5.37	5.26	5.04	6.35	5.37	4.46	5.94	5.86	4.88	5.26	5.43	6.33	5.98	4.55	5.72	5.29	5.22
5.21	5.68	5.38	5.04	6.35	5.38	4.46	5.94	5.86	4.88	5.55	5.43	6.52	6.58	4.55	6.14	5.52	5.22
5.21	5.68	5.38	5.47	6.38	5.66	4.47	5.94	6.11	5.02	6.05	5.43	6.52	6.79	4.55	6.14	5.52	5.87
5.21	5.68	5.6	5.47	6.38	5.66	4.59	6	6.22	5.28	6.35	5.43	6.52	6.79	4.55	6.14	5.52	5.87
5.42	6.05	5.63	5.47	6.72	5.66	4.72	6	6.22	5.28	6.35	5.75	6.72	6.79	4.55	6.22	5.76	5.87
5.43	6.05	5.63	5.47	6.78	6.1	4.87	6	6.24	5.45	6.35	5.75	6.73	7.02	4.59	6.23	5.93	6.31
5.46	6.05	5.75	5.76	6.78	6.12	5.12	6.26	6.24	5.45	6.35	5.75	6.73	7.02	4.99	6.3	5.93	6.31
5.55	6.22	5.83	5.87	6.8	6.12	5.29	6.54	6.57	5.68	6.36	6.09	7.1	7.09	4.99	6.47	5.93	6.31
6.01	6.22	5.83	5.87	6.8	6.4	5.29	6.54	6.82	5.68	6.36	6.09	7.1	7.22	4.99	6.47	5.93	6.31
6.04	6.22	6.05	5.87	6.8	6.4	5.51	6.54	6.82	5.79	6.36	6.28	7.1	7.22	4.99	6.59	6.39	6.31
6.08	6.22	6.05	6.25	6.81	6.55	5.51	6.54	6.82	5.79	6.38	6.3	7.12	7.22	5.37	6.83	6.39	6.31
6.14	6.22	6.05	6.25	6.96	6.55	5.79	7.08	6.84	5.79	6.38	6.32	7.52	7.22	5.37	6.85	6.39	6.56
6.78	6.51	6.05	6.25	6.96	6.55	5.79	7.08	6.84	5.79	6.53	6.32	7.72	7.22	5.37	6.85	6.39	6.56
6.78	6.51	6.11	6.25	7.1	6.56	5.79	7.08	6.84	5.79	6.53	6.32	7.72	7.22	5.37	6.99	6.66	6.7
6.78	6.51	6.11	6.64	7.1	6.56	5.79	7.32	6.84	5.96	6.53	6.65	7.72	7.22	5.37	6.99	6.79	6.7
6.78	6.51	6.11	6.68	7.15	6.79	5.79	7.46	6.84	6.15	6.53	6.66	7.72	7.25	5.37	7.28	6.79	7.08
6.78	6.87	6.25	6.68	7.15	6.79	5.82	7.46	6.84	6.17	6.53	6.66	7.72	7.25	5.39	7.28	6.8	7.08
6.78	6.87	6.25	6.68	7.36	6.79	5.82	7.87	.	6.45	6.62	6.66	7.72	7.28	5.49	7.28	7.31	7.08
6.78	6.88	6.31	6.68	7.36	6.79	5.82	7.87	.	6.45	6.83	6.66	7.72	7.28	5.62	7.28	7.31	7.08
6.78	6.88	6.31	6.68	7.36	6.82	5.99	7.87	.	6.45	6.83	6.66	7.72	7.28	5.62	7.28	7.31	7.08
6.78	7.24	6.42	6.68	7.39	6.82	6.51	7.87	.	6.52	6.83	6.66	7.72	7.3	5.79	7.28	7.31	7.63
6.78	7.24	6.42	6.68	7.39	7.09	6.51	7.87	.	6.52	6.95	6.81	7.72	7.3	5.79	7.29	7.31	7.63
6.78	7.24	6.43	6.68	7.51	7.09	6.52	7.87	.	6.52	6.95	6.81	7.9	7.45	5.96	7.59	7.31	7.63
6.78	7.24	6.43	6.68	7.51	7.09	6.52	7.87	.	6.56	6.95	6.81	7.9	7.45	6.29	7.62	7.31	7.63
6.98	7.24	6.65	6.68	7.51	7.09	6.52	8.36	.	6.8	6.95	6.81	7.95	7.45	6.29	7.62	7.31	7.67
7.01	7.24	6.65	6.94	7.73	7.09	6.62	8.36	.	6.97	6.95	6.86	8.02	7.45	6.62	7.62	7.31	7.67
7.01	7.24	6.65	6.95	7.76	7.32	6.96	8.36	.	6.97	6.95	7.35	8.12	7.45	6.66	8.13	7.31	7.67
7.01	7.24	6.83	6.95	7.76	7.32	6.96	8.36	.	6.97	6.95	7.35	8.12	7.45	6.7	8.13	7.31	7.67
7.01	7.44	6.83	6.95	7.84	7.33	6.96	8.36	.	6.97	7.31	7.35	8.12	7.45	6.74	8.13	7.31	7.67
7.01	7.44	6.83	7.22	7.84	7.33	6.96	8.36	.	6.97	7.31	7.44	8.19	7.45	6.78	8.26	7.33	7.67
7.01	7.44	6.84	7.22	7.84	7.33	6.97	8.36	.	6.97	7.32	7.48	8.19	7.45	6.82	8.27	7.98	7.8
7.01	7.44	6.84	7.22	8	7.42	6.97	8.36	.	7.17	7.33	7.49	8.31	7.45	6.86	8.29	7.98	7.8
7.01	7.44	6.84	7.22	8	7.45	6.98	8.36	.	7.17	7.33	7.61	8.31	7.6	6.9	8.29	7.98	7.86
7.01	7.44	6.84	7.41	8	7.45	6.98	8.36	.	7.17	7.33	7.61	8.31	7.6	6.94	8.29	7.98	7.96
7.01	7.68	7.12	7.53	8.39	7.45	6.98	8.36	.	7.18	7.39	7.73	8.32	7.6	6.98	8.29	7.98	7.96
7.01	7.68	7.12	7.53	8.4	7.57	6.99	8.36	.	7.18	7.78	7.78	8.32	7.6	7.02	8.29	7.98	7.96
7.01	7.68	7.12	7.53	8.4	7.57	7.25	8.36	.	7.18	7.78	7.78	8.51	7.6	7.06	8.68	7.98	7.97
7.01	7.7	7.32	7.54	8.4	7.57	7.25	8.36	.	7.18	7.78	7.78	8.51	7.6	7.1	8.68	7.98	7.98
7.42	7.7	7.49	7.54	8.83	7.57	7.25	8.36	.	7.85	7.78	8.05	8.51	7.6	7.14	8.68	7.98	7.98
7.48	7.7	7.49	7.54	8.83	7.57	7.25	8.36	.	7.85	8.13	8.06	8.7	7.6	7.18	8.75	8.02	7.98
7.58	7.7	7.5	7.54	8.93	7.59	7.25	8.36	.	7.85	8.13	8.06	8.7	7.6	7.22	8.92	8.19	8
7.85	7.7	7.73	7.54	8.93	7.59	7.25	8.37	.	7.85	8.14	8.06	8.82	7.6	7.26	8.92	8.19	8
7.86	7.7	7.85	7.54	8.93	7.59	7.32	8.37	.	7.85	8.5	8.06	8.82	7.6	7.3	8.94	8.19	8
7.86	7.81	7.86	7.54	8.95	7.59	7.32	8.37	.	7.85	8.5	8.06	8.82	7.61	7.34	8.94	8.19	8.02
7.86	7.82	7.86	7.54	9.26	7.65	7.32	8.37	.	7.85	8.5	8.06	8.99	7.61	7.38	8.96	8.19	8.02
8.1	7.87	7.87	7.54	9.28	7.65	7.32	8.37	.	7.85	8.5	8.12	8.99	7.61	7.42	8.96	8.19	8.02
8.1	7.87	7.87	.	.	7.65	7.33	8.51	.	7.85	8.5	.	.	7.61	7.46	9.1	8.64	8.02
8.32	7.88	7.88	.	.	7.65	7.33	8.51	.	7.85	8.5	.	.	7.61	7.5	9.41	8.64	8.43
8.32	7.93	7.88	.	.	7.82	7.33	8.72	.	7.86	8.5	.	.	7.61	7.54	9.41	8.64	8.43
8.32	7.93	7.89	.	.	7.82	7.7	8.72	.	7.86	8.5	.	.	7.61	7.58	9.41	8.64	8.43
8.32	7.93	7.9	.	.	7.82	7.7	8.72	.	7.93	8.74	.	.	7.61	7.59	9.41	8.64	8.43
8.32	7.93	.	.	.	7.82	7.71	8.72	.	7.93	.	.	.	7.67	7.6	9.47	8.64	8.43
8.91	7.93	.	.	.	8.13	7.71	8.72	.	7.93	.	.	.	7.67	7.61	9.47	8.67	8.43
8.93	7.94	.	.	.	8.13	7.71	8.72	.	7.93	.	.	.	7.67	7.62	9.47	8.67	8.46
8.93	7.97	.	.	.	8.13	7.71	8.74	.	7.93	.	.	.	7.67	7.63	9.47	8.67	8.79
9	8.03	.	.	.	8.19	7.71	8.77	.	8.04	.	.	.	7.67	7.64	9.47	8.67	8.79
9	8.26	.	.	.	8.19	7.71	9.13	.	8.15	.	.	.	7.77	7.65	9.49	8.74	8.79
9.04	8.26	.	.	.	8.19	8.38	9.13	.	8.15	.	.	.	8.38	7.66	9.49	9.11	8.79
9.04	8.49	.	.	.	8.5	8.38	9.13	.	8.15	.	.	.	8.39	7.67	9.49	9.11	9.09
9.08	8.78	.	.	.	8.5	8.39	9.13	.	8.15	.	.	.	8.39	7.68	9.49	9.11	9.28
9.58	8.78	.	.	.	8.5	8.39	9.13	.	8.15	.	.	.	8.7	7.69	9.7	9.11	9.28
9.58	8.78	.	.	.	8.51	8.39	9.48	.	8.55	.	.	.	8.7	7.7	9.7	9.11	9.28
9.69	8.79	.	.	.	8.51	8.39	9.48	.	8.55	.	.	.	8.7	7.76	9.86	9.12	9.28
10.07	8.79	.	.	.	8.92	8.39	9.48	.	8.55	.	.	.	8.7	7.76	9.87	9.12	9.28
10.08	9.2	.	.	.	8.92	8.43	9.48	.	8.74	.	.	.	8.7	7.76	9.87	9.12	9.28
10.08	9.2	.	.	.	8.92	8.43	9.93	.	8.74	.	.	.	8.72	7.76	10.11	9.13	9.64
10.08	9.25	.	.	.	9.13	8.43	9.93	.	8.91	.	.	.	9.36	7.76	10.11	9.13	9.78
10.12	9.62	.	.	.	9.13	8.43	9.93	.	9.34	.	.	.	9.36	7.76	10.11	9.13	9.78
10.3	9.62	.	.	.	9.13	8.43	9.93	.	9.34	.	.	.	9.36	7.77	10.29	9.52	9.78
10.33	9.62	.	.	.	9.64	8.43	9.93	.	9.34	.	.	.	9.36	7.77	10.37	9.61	9.78
10.38	10.06	.	.	.	9.66	8.45	10.54	.	9.34	.	.	.	10.34	7.77	10.37	9.61	9.78
10.39	10.06	.	.	.	9.66	8.53	10.55	.	10	.	.	.	10.72	7.77	10.37	9.61	10.2
10.39	10.06	.	.	.	9.66	8.53	10.55	.	10	.	.	.	10.72	7.84	10.37	9.61	10.52
10.39	10.06	.	.	.	9.7	8.71	10.55	.	10	.	.	.	10.72	7.86	10.71	9.61	10.52
10.73	10.06	.	.	.	9.71	8.72	10.94	.	10	.	.	.	10.72	7.9	10.71	9.77	10.52
10.73	10.06	.	.	.	9.71	8.8	10.94	.	10	.	.	.	10.99	7.91	10.71	9.77	10.52
10.73	10.06	.	.	.	10.12	8.8	10.94	.	10.35	.	.	.	10.99	7.93	11.16	9.77	10.52
10.96	10.06	.	.	.	10.12	9.29	10.94	.	10.35	.	.	.	10.99	7.93	11.16	9.77	10.52
10.96	10.06	.	.	.	10.12	9.29	10.94	.	10.35	.	.	.	10.99	8.12	11.16	9.77	10.52
10.96	10.06	.	.	.	10.12	9.29	10.94	.	10.35	.	.	.	10.99	8.12	11.16	9.78	10.97
10.96	10.4	.	.	.	10.12	9.48	10.94	.	10.35	.	.	.	10.99	8.12	11.16	9.78	10.97
11.1	10.4	.	.	.	10.12	9.48	10.94	.	10.35	.	.	.	10.99	8.12	11.16	9.78	10.97
11.1	10.4	.	.	.	10.12	9.48	10.94	.	10.35	.	.	.	10.99	8.12	11.16	9.78	10.97
11.1	10.4	.	.	.	10.14	9.48	10.94	.	10.35	.	.	.	11	8.13	11.16	9.78	10.97
11.25	10.4	.	.	.	10.14	9.48	10.94	.	10.35	.	.	.	11	8.19	11.86	9.78	10.97
11.26	10.4	.	.	.	10.14	9.48	11.01	.	10.35	.	.	.	11	8.19	12.08	9.78	10.97
11.26	10.61	.	.	.	10.44	10	11.01	.	10.35	.	.	.	11	8.24	12.08	9.78	11.1
11.26	10.61	.	.	.	10.44	10	11.1	.	10.35	.	.	.	11	8.4	12.08	9.79	11.1
11.26	10.73	.	.	.	10.45	10	11.1	.	10.44	.	.	.	11	8.4	12.08	9.79	11.1
11.69	10.73	.	.	.	10.45	10	11.25	.	10.51	.	.	.	11	8.56	12.08	9.8	11.17
11.76	10.73	.	.	.	10.45	10	11.26	.	10.51	.	.	.	11	8.56	12.08	9.81	11.17
11.76	10.81	.	.	.	10.45	10	11.26	.	10.51	.	.	.	11	8.57	12.57	10.04	11.34
11.76	10.81	.	.	.	10.65	10	11.26	.	10.51	.	.	.	11	8.59	12.58	10.04	11.34
11.76	10.82	.	.	.	10.65	10	11.26	.	10.51	.	.	.	11	8.69	12.58	10.04	11.34
11.76	10.88	.	.	.	10.65	10.49	11.26	.	10.51	.	.	.	11	8.84	12.58	10.04	11.53
11.76	10.9	.	.	.	10.81	10.61	11.31	.	10.51	.	.	.	11	8.84	12.58	10.04	11.54

11.76	10.9	.	.	.	11.04	10.61	11.31	.	10.51	.	.	.	11	8.84	12.58	10.04	11.54
11.76	10.9	.	.	.	11.04	10.61	11.31	.	10.51	.	.	.	11	8.84	12.59	10.07	11.54
11.76	10.9	.	.	.	11.04	10.61	11.61	.	10.51	.	.	.	11	9.17	12.59	10.07	11.54
11.76	11.01	.	.	.	11.04	10.61	11.61	.	10.62	.	.	.	11	9.18	12.59	10.07	11.55
12.22	11.01	.	.	.	11.04	10.75	11.61	.	10.62	.	.	.	11	9.18	12.59	10.07	11.55
12.29	11.01	.	.	.	11.59	10.76	11.89	.	10.62	.	.	.	11	9.21	12.59	10.19	11.89
12.32	11.01	.	.	.	11.6	10.76	11.89	.	10.62	.	.	.	11	9.21	12.64	10.19	11.89
12.32	11.01	.	.	.	11.6	10.77	11.89	.	10.62	.	.	.	11	9.21	12.64	10.2	11.89
12.32	11.08	.	.	.	11.6	10.77	11.89	.	10.62	.	.	.	11	9.22	12.64	10.2	11.89
12.32	11.08	.	.	.	11.61	10.78	11.89	.	10.62	.	.	.	11	9.22	12.64	10.23	11.89
12.32	11.08	.	.	.	11.61	10.78	11.99	.	10.62	.	.	.	11	9.22	12.64	10.23	12.19
12.32	11.1	.	.	.	11.61	10.78	11.99	.	10.62	.	.	.	11	9.31	12.64	10.23	12.19
12.41	11.1	.	.	.	11.74	10.81	11.99	.	10.62	.	.	.	11	9.54	12.64	10.23	12.2
12.42	11.1	.	.	.	11.74	10.81	12.09	.	10.62	.	.	.	11	9.59	12.64	10.23	12.21
12.42	11.11	.	.	.	11.75	**10.82**	**12.09**	.	10.62	.	.	.	11	**9.59**	**12.64**	10.23	12.27

0	0	0	0	0	0	**0**	**0**	0	0	0	0	0	0	**0**	**0**	0	#VALUE!
0.2	0.04	0	0	0.21	0	0	0.17	0.15	0	0	0.05	0.01	0.01	0	0	0	#VALUE!
0.2	0.04	0.01	0	0.24	0.15	0.1	0.28	0.23	0.07	0.09	0.05	0.19	0.07	0.13	0	0.01	#VALUE!
0.2	0.26	0.02	0	0.33	0.33	0.28	0.4	0.24	0.15	0.22	0.28	0.2	0.07	0.13	0	0.04	#VALUE!
0.2	0.26	0.02	0	0.33	0.48	0.28	0.4	0.42	0.26	0.37	0.28	0.21	0.34	0.18	0	0.05	#VALUE!
0.4	0.46	0.02	0	0.34	0.48	0.57	0.53	0.53	0.26	0.37	0.71	0.44	0.58	0.18	0.22	0.21	#VALUE!
0.4	0.46	0.02	0	0.43	0.59	0.57	0.53	0.6	0.51	0.55	0.71	0.44	0.58	0.3	0.22	0.21	#VALUE!
0.4	0.53	0.02	0	0.5	0.59	0.77	0.65	0.6	0.53	0.55	0.71	0.61	0.78	0.3	0.22	0.21	#VALUE!
0.6	0.53	0.03	0	0.62	0.8	0.8	0.65	0.6	0.66	0.79	0.71	0.61	0.99	0.3	0.22	0.5	#VALUE!
0.63	0.78	0.03	0.01	0.62	0.88	0.9	1.04	0.84	0.66	0.8	0.71	0.62	1.14	0.5	0.22	0.5	#VALUE!
0.63	0.78	0.04	0.01	0.8	1	0.91	1.04	1.03	0.66	0.91	0.81	0.83	1.14	0.62	0.5	0.6	#VALUE!
0.63	1	0.05	0.01	0.8	1	0.91	1.04	1.22	0.78	0.91	0.81	0.83	1.29	0.65	0.5	0.6	#VALUE!
0.88	1	0.16	0.01	0.88	1.13	1.21	1.04	1.22	0.78	0.91	0.93	0.84	1.61	0.8	0.5	0.6	#VALUE!
0.91	1	0.27	0.01	0.89	1.13	1.22	1.09	1.22	1	1.02	0.93	0.84	1.62	0.88	0.5	0.65	#VALUE!
0.91	1.14	0.52	0.01	1.06	1.17	1.22	1.15	1.4	1	1.03	0.93	0.84	1.62	0.89	0.5	1.02	#VALUE!
0.92	1.37	0.68	0.1	1.06	1.18	1.22	1.15	1.41	1.23	1.32	0.93	0.88	1.98	0.89	0.5	1.02	#VALUE!
0.92	1.37	0.77	0.42	1.35	1.38	1.45	1.42	1.54	1.23	1.32	0.93	1.22	1.99	0.96	0.5	1.03	#VALUE!
1	1.37	1	0.42	1.36	1.38	1.45	1.42	1.58	1.23	1.32	1	1.22	1.99	0.96	0.5	1.19	#VALUE!
1.05	1.5	1	0.42	1.36	1.38	1.45	1.42	1.58	1.68	1.33	1	1.22	1.99	1.07	0.5	1.24	#VALUE!
1.05	1.5	1	0.51	1.36	1.38	1.45	1.42	1.74	1.71	1.43	1	1.35	1.99	1.07	0.5	1.24	#VALUE!
1.05	1.5	1	0.52	1.41	1.61	1.75	1.72	1.74	1.71	1.44	1.17	1.35	2.28	1.13	0.5	1.43	#VALUE!
1.31	1.69	1	0.8	1.42	1.69	1.75	1.76	1.75	1.99	1.44	1.17	1.35	2.29	1.19	0.5	1.43	#VALUE!
1.31	1.69	1.21	0.8	1.51	1.84	1.75	1.77	1.88	1.99	1.68	1.17	1.4	2.46	1.2	0.5	1.43	#VALUE!
1.31	1.69	1.21	0.97	1.51	1.84	2.03	1.77	1.99	1.99	1.68	1.17	1.4	2.49	1.24	0.5	1.74	#VALUE!
1.31	1.79	1.21	0.98	1.68	1.84	2.03	2.02	1.99	1.99	1.68	1.17	1.4	2.49	1.24	0.5	1.74	#VALUE!
1.67	1.79	1.21	1.3	1.68	1.86	2.03	2.02	1.99	1.99	1.68	1.27	1.6	2.69	1.3	0.5	1.74	#VALUE!
1.69	2.07	1.21	1.3	1.68	1.86	2.03	2.02	1.99	2.06	1.68	1.4	1.6	2.69	1.42	0.5	1.74	#VALUE!
1.69	2.23	1.28	1.3	1.68	2	2.23	2.42	2.15	2.24	1.82	1.4	1.6	2.69	1.43	0.5	1.74	#VALUE!
1.69	2.23	1.28	1.3	1.73	2	2.56	2.42	2.19	2.24	1.82	1.4	1.6	2.69	1.43	0.5	1.99	#VALUE!
1.69	2.23	1.28	1.3	1.87	2	2.56	2.42	2.19	2.24	2	1.4	1.81	2.69	1.5	0.5	1.99	#VALUE!
1.69	2.36	1.37	1.3	1.91	2.09	2.56	2.42	2.19	2.4	2	1.4	1.81	2.69	1.52	0.5	1.99	#VALUE!
1.69	2.36	1.37	1.3	1.91	2.09	2.56	2.42	2.38	2.4	2	1.4	1.81	2.72	1.52	0.5	1.99	#VALUE!
1.69	2.36	1.37	1.3	1.91	2.09	2.56	2.43	2.39	2.41	2.07	1.4	1.82	2.73	1.52	0.5	1.99	#VALUE!
1.88	2.36	1.42	1.33	2.08	2.09	2.56	2.43	2.4	2.41	2.07	1.5	1.82	2.73	1.66	0.5	1.99	#VALUE!
1.88	2.57	1.42	1.33	2.08	2.09	2.56	2.43	2.4	2.41	2.07	1.5	1.82	2.73	1.66	0.5	2.11	#VALUE!
1.88	2.57	1.42	1.33	2.08	2.09	2.56	2.44	2.4	2.67	2.07	1.5	1.82	2.73	1.66	0.5	2.11	#VALUE!
1.88	2.57	1.52	1.33	2.08	2.09	2.56	2.44	2.48	2.67	2.23	1.72	1.83	2.73	1.7	0.85	2.11	#VALUE!
1.88	2.78	1.52	1.33	2.08	2.09	2.56	2.65	2.48	2.67	2.26	1.72	1.83	2.88	1.83	0.85	2.11	#VALUE!
1.9	2.78	1.56	1.33	2.08	2.09	2.56	2.65	2.8	2.7	2.26	1.72	1.83	2.88	1.83	0.85	2.11	#VALUE!
2.12	2.78	1.61	1.33	2.11	2.31	2.56	2.65	2.8	2.7	2.39	1.72	1.83	2.88	1.83	0.85	2.11	#VALUE!
2.12	2.78	1.78	1.33	2.11	2.38	2.69	2.65	2.8	2.79	2.39	1.72	1.83	2.88	1.83	0.85	2.46	#VALUE!
2.12	3.01	1.79	1.33	2.23	2.38	2.74	2.65	2.8	2.79	2.39	1.72	1.96	2.88	1.9	0.85	2.46	#VALUE!
2.12	3.01	1.79	1.33	2.23	2.48	2.74	2.7	2.8	2.83	2.54	1.72	1.96	3.02	1.9	0.85	2.46	#VALUE!
2.12	3.01	1.79	1.33	2.23	2.48	2.75	2.71	2.8	2.84	2.54	1.72	2.19	3.03	1.95	0.85	2.46	#VALUE!
2.12	3.01	1.82	1.34	2.25	2.6	2.75	2.71	2.84	2.84	2.54	1.72	2.19	3.03	1.95	0.85	2.46	#VALUE!
2.43	3.01	1.82	1.34	2.25	2.78	3.01	2.71	2.84	3.11	2.79	1.72	2.19	3.03	1.98	0.85	2.58	#VALUE!
2.45	3.01	1.82	1.34	2.25	2.78	3.17	2.8	2.84	3.11	2.79	1.92	2.19	3.03	2	0.85	2.58	#VALUE!
2.46	3.08	1.82	1.64	2.25	2.78	3.17	2.8	2.84	3.12	2.79	1.92	2.23	3.03	2.05	1.03	2.58	#VALUE!
2.65	3.14	1.82	1.66	2.49	2.9	3.17	2.8	2.84	3.12	2.9	1.92	2.43	3.03	2.05	1.27	2.81	#VALUE!
2.65	3.14	2.08	1.66	2.49	2.9	3.17	2.8	2.86	3.12	2.98	1.92	2.43	3.34	2.25	1.27	2.81	#VALUE!
2.65	3.14	2.08	1.66	2.49	2.9	3.27	2.86	2.86	3.47	2.98	1.92	2.43	3.34	2.25	1.27	2.81	#VALUE!
2.65	3.15	2.08	1.66	2.67	2.99	3.28	2.86	2.86	3.47	2.98	1.92	2.45	3.4	2.25	1.27	2.81	#VALUE!
2.78	3.15	2.27	1.66	2.67	2.99	3.52	2.86	2.92	3.47	3.08	1.92	2.45	3.4	2.25	1.27	2.81	#VALUE!
2.78	3.17	2.27	2.01	2.68	2.99	3.53	2.97	2.92	3.47	3.08	1.92	2.45	3.4	2.25	1.27	2.81	#VALUE!
2.79	3.19	2.28	2.01	2.68	3.11	3.53	2.99	2.92	3.47	3.08	2	2.45	3.4	2.27	1.27	3.01	#VALUE!
2.79	3.19	2.29	2.01	2.68	3.11	3.53	2.99	3.02	3.47	3.09	2	2.45	3.46	2.27	1.27	3.01	#VALUE!
2.79	3.19	2.29	2.01	2.78	3.11	3.53	3.19	3.02	3.64	3.09	2	2.74	3.46	2.27	1.27	3.01	#VALUE!
2.79	3.19	2.29	2.01	2.78	3.11	3.55	3.19	3.19	3.64	3.17	2.23	2.74	3.59	2.42	1.27	3.01	#VALUE!
3.02	3.31	2.42	2.01	2.8	3.15	3.55	3.19	3.19	3.64	3.18	2.23	2.74	3.59	2.42	1.27	3.01	#VALUE!
3.04	3.31	2.42	2.01	2.8	3.15	3.55	3.19	3.42	3.64	3.18	2.25	2.74	3.59	2.42	1.27	3.01	#VALUE!
3.06	3.31	2.43	2.01	2.8	3.15	3.55	3.19	3.42	3.64	3.18	2.25	2.74	3.67	2.46	1.27	3.02	#VALUE!
3.06	3.42	2.43	2.01	2.98	3.26	3.55	3.2	3.42	3.64	3.31	2.25	2.97	3.67	2.46	1.27	3.24	#VALUE!
3.07	3.42	2.59	2.01	2.98	3.28	3.56	3.2	3.63	3.64	3.31	2.53	2.97	3.87	2.48	1.27	3.24	#VALUE!
3.07	3.42	2.59	2.01	2.98	3.42	3.56	3.2	3.63	3.78	3.31	2.53	2.97	3.87	2.48	1.27	3.24	#VALUE!
3.07	3.59	2.59	2.25	3.14	3.53	3.8	3.31	3.63	3.78	3.4	2.53	2.97	3.87	2.55	1.27	3.24	#VALUE!
3.07	3.59	2.6	2.25	3.14	3.53	3.8	3.32	3.64	3.78	3.4	2.53	3.19	3.87	2.6	1.27	3.24	#VALUE!
3.59	3.6	2.6	2.25	3.14	3.53	3.8	3.32	3.88	3.97	3.56	2.7	3.19	4.06	2.6	1.27	3.48	#VALUE!
3.59	3.6	2.9	2.25	3.34	3.53	3.8	3.43	3.88	3.97	3.56	2.7	3.19	4.06	2.73	1.27	3.48	#VALUE!
3.64	3.77	2.9	2.25	3.34	3.65	4.03	3.43	3.88	4	3.78	2.7	3.19	4.06	2.84	1.27	3.73	#VALUE!
3.64	3.77	2.9	2.25	3.34	3.65	4.03	3.43	4.15	4	3.78	2.7	3.31	4.06	2.84	1.27	3.73	#VALUE!
3.64	3.89	2.9	2.25	3.37	3.87	4.03	3.43	4.15	4.14	3.78	2.97	3.45	4.11	2.87	1.27	3.73	#VALUE!
3.64	3.89	3.07	2.49	3.61	3.87	4.17	3.59	4.15	4.23	3.78	2.97	3.45	4.12	2.92	1.27	3.73	#VALUE!
3.64	4.12	3.08	2.49	3.61	3.96	4.17	3.95	4.15	4.23	3.79	2.97	3.62	4.3	2.92	1.27	3.98	#VALUE!
3.64	4.12	3.08	2.49	3.61	4.15	4.25	3.95	4.15	4.44	3.97	3.05	3.63	4.3	3.13	1.27	4.14	#VALUE!
4.15	4.32	3.09	2.49	3.61	4.15	4.5	3.95	4.15	4.44	3.97	3.17	3.63	4.3	3.14	1.28	4.14	#VALUE!
4.15	4.32	3.09	2.49	3.65	4.15	4.51	4.07	4.41	4.44	3.97	3.17	3.63	4.3	3.14	1.28	4.14	#VALUE!
4.15	4.32	3.21	2.49	3.65	4.28	4.51	4.07	4.41	4.62	3.97	3.17	3.86	4.45	3.45	1.28	4.14	#VALUE!
4.15	4.34	3.22	2.77	3.65	4.49	4.51	4.22	4.54	4.62	3.97	3.17	3.86	4.7	3.45	2.02	4.14	#VALUE!
4.15	4.49	3.22	2.77	3.65	4.49	4.52	4.22	4.57	4.62	4.05	3.17	3.86	4.71	3.49	2.02	4.15	#VALUE!
4.15	4.98	3.36	2.77	3.68	4.62	4.52	4.22	4.57	4.73	4.05	3.18	3.86	4.71	3.49	2.02	4.57	#VALUE!
4.15	4.98	3.55	2.77	3.98	4.81	4.52	4.22	4.57	4.73	4.05	3.18	3.87	4.87	3.54	2.02	4.57	#VALUE!
4.15	5	3.55	2.86	3.98	4.81	4.8	4.29	4.57	4.74	4.17	3.18	4.06	4.98	3.54	2.02	4.57	#VALUE!
4.18	5	3.55	2.86	3.98	4.81	4.8	4.29	4.57	5.24	4.17	3.75	4.06	4.98	3.76	2.65	4.57	#VALUE!
4.18	5	3.79	3.06	4.15	4.81	4.8	4.3	4.73	5.24	4.17	3.76	4.06	4.98	3.78	3.03	4.72	#VALUE!
4.36	5	3.79	3.28	4.15	4.92	4.8	4.3	4.78	5.24	4.17	3.76	4.06	5.1	3.9	3.03	4.72	#VALUE!
4.36	5	3.79	3.28	4.15	5.04	4.8	4.45	4.78	5.24	4.18	3.76	4.07	5.1	3.92	3.03	5.15	#VALUE!
4.36	5	3.79	3.28	4.23	5.05	4.8	4.66	4.78	5.49	4.42	3.76	4.09	5.22	3.92	3.03	5.15	#VALUE!
4.69	5.12	3.79	3.28	4.23	5.05	4.8	4.66	4.78	5.49	4.42	3.76	4.21	5.22	3.93	3.03	5.15	#VALUE!
4.69	5.12	3.79	3.28	4.27	5.05	4.8	4.66	4.78	5.49	4.42	3.76	4.21	5.22	3.98	3.03	5.15	#VALUE!
4.83	5.12	3.79	3.28	4.27	5.09	4.8	4.66	4.92	5.49	4.42	3.76	4.21	5.22	4.03	3.03	5.15	#VALUE!
4.84	5.12	3.9	3.61	4.43	5.2	4.8	4.88	4.92	5.59	4.42	3.76	4.32	5.25	4.03	3.32	5.16	#VALUE!
4.86	5.12	3.9	3.61	4.43	5.2	4.8	4.91	4.92	5.68	4.55	3.76	4.32	5.26	4.1	3.32	5.16	#VALUE!
4.86	5.22	3.9	3.61	4.43	5.23	4.8	4.92	4.92	5.83	4.55	3.83	4.32	5.26	4.1	3.32	5.16	#VALUE!
4.92	5.26	3.9	3.74	4.43	5.25	5.07	4.92	4.92	5.83	4.63	3.83	4.32	5.26	4.14	3.32	5.28	#VALUE!
4.97	5.26	3.9	3.77	4.6	5.25	5.07	4.92	4.97	5.84	4.63	3.83	4.51	5.45	4.21	3.78	5.28	#VALUE!
5.3	5.28	3.9	3.77	4.6	5.44	5.07	4.92	5.07	5.89	4.63	3.83	4.51	5.48	4.21	3.78	5.28	#VALUE!
5.3	5.43	3.96	3.77	4.66	5.49	5.07	5.09	5.07	6.06	4.63	3.83	4.63	5.48	4.37	3.78	5.5	#VALUE!
5.3	5.43	3.99	3.77	4.66	5.62	5.08	5.09	5.07	6.06	4.64	3.83	4.63	5.48	4.37	3.78	5.5	#VALUE!
5.3	5.43	3.99	3.86	4.66	5.62	5.25	5.09	5.23	6.06	4.64	4.04	4.63	5.48	4.37	3.78	5.51	#VALUE!
5.3	5.6	4.28	3.86	4.66	5.62	5.25	5.09	5.23	6.06	4.97	4.04	4.63	5.51	4.64	3.78	5.63	#VALUE!
5.3	5.6	4.28	3.86	4.81	5.79	5.25	5.23	5.23	6.06	4.98	4.04	4.63	5.52	4.64	3.78	5.63	#VALUE!
5.3	5.61	4.28	3.86	4.81	5.79	5.25	5.23	5.33	6.24	4.98	4.04	4.63	5.52	4.64	3.78	5.63	#VALUE!
5.3	5.61	4.28	4.05	4.81	5.79	5.5	5.23	5.33	6.43	4.98	4.04	4.66	5.6	4.66	3.78	5.63	#VALUE!
5.33	5.61	4.28	4.05	4.9	5.79	5.5	5.23	5.34	6.43	4.98	4.04	4.66	5.6	4.66	3.99	5.63	#VALUE!
5.66	5.86	4.28	4.05	4.9	5.93	5.5	5.23	5.34	6.43	4.98	4.04	4.66	5.6	4.66	3.99	5.63	#VALUE!

5.69	5.86	4.32	4.05	4.92	5.99	5.5	5.41	5.46	6.43	4.98	4.04	4.88	5.6	4.66	3.99	5.63	#VALUE!
5.7	5.86	4.36	4.05	4.92	6.17	5.83	5.44	5.46	6.43	4.98	4.14	4.88	5.7	4.66	3.99	5.86	#VALUE!
5.7	6.01	4.36	4.06	5.04	6.17	5.83	5.44	5.46	6.63	5.12	4.14	4.88	5.7	4.68	3.99	5.86	#VALUE!
5.72	6.01	4.39	4.22	5.04	6.39	5.84	5.45	5.46	6.64	5.12	4.14	4.88	5.7	4.9	3.99	5.93	#VALUE!
6.11	6.01	4.39	4.22	5.11	6.4	5.84	5.45	5.46	6.64	5.14	4.49	4.88	5.7	4.9	3.99	5.99	#VALUE!
6.11	6.33	4.39	4.22	5.35	6.4	6.01	5.65	5.62	6.64	5.14	4.49	4.88	5.7	4.9	3.99	5.99	#VALUE!
6.11	6.33	4.39	4.32	5.35	6.4	6.01	5.65	5.62	6.85	5.14	4.49	4.88	5.7	4.9	4.13	5.99	#VALUE!
6.11	6.4	4.71	4.48	5.35	6.4	6.01	5.77	5.62	6.85	5.52	4.49	5.11	5.7	4.9	4.13	6.01	#VALUE!
6.11	6.51	4.77	4.48	5.35	6.4	6.01	5.84	5.62	6.85	5.52	4.49	5.11	5.7	4.9	4.13	6.01	#VALUE!
6.11	6.51	4.77	4.59	5.38	6.48	6.01	5.84	5.79	6.85	5.53	4.77	5.32	5.7	4.9	4.13	6.26	#VALUE!
6.11	6.52	4.93	4.71	5.38	6.49	6.31	6.05	5.79	6.89	5.73	4.77	5.32	5.97	5.12	4.14	6.26	#VALUE!
6.11	6.52	4.93	4.71	5.53	6.49	6.32	6.05	5.79	6.9	5.73	4.77	5.49	6.17	5.16	4.14	6.43	#VALUE!
6.11	6.52	4.93	4.71	5.53	6.49	6.32	6.05	5.79	6.9	5.73	4.77	5.49	6.17	5.19	4.14	6.43	#VALUE!
6.51	6.52	4.93	4.88	5.53	6.49	6.41	6.05	5.93	6.9	5.9	4.78	5.49	6.17	5.2	4.14	6.49	#VALUE!
6.52	6.6	5.07	4.88	5.66	6.49	6.56	6.05	5.93	6.91	5.9	4.78	5.63	6.37	5.33	4.14	6.5	#VALUE!
6.52	6.6	5.07	4.88	5.66	6.68	6.56	6.24	5.93	6.91	5.9	4.78	5.63	6.37	5.33	4.14	6.5	#VALUE!
6.53	6.6	5.07	4.88	5.66	6.68	6.56	6.25	5.93	6.91	5.9	4.78	5.63	6.37	5.33	4.14	6.5	#VALUE!
6.53	6.74	5.13	5.18	5.66	6.68	6.6	6.25	6.02	6.91	5.9	5.02	5.63	6.37	5.33	4.28	6.51	#VALUE!
6.82	6.74	5.13	5.18	5.66	6.81	6.6	6.25	6.02	6.91	5.9	5.03	5.63	6.37	5.43	4.28	6.51	#VALUE!
6.82	6.74	5.13	5.18	5.66	6.81	6.61	6.39	6.02	6.91	6.14	5.03	5.63	6.37	5.43	4.28	6.54	#VALUE!
6.83	6.92	5.13	5.18	5.75	6.81	6.61	6.39	6.15	7.02	6.14	5.03	5.87	6.37	5.43	4.28	6.54	#VALUE!
6.83	6.92	5.16	5.18	5.75	6.81	6.74	6.46	6.15	7.03	6.14	5.03	5.87	6.37	5.43	4.28	6.63	#VALUE!
6.84	6.92	5.16	5.18	5.75	6.88	6.74	6.47	6.15	7.03	6.14	5.03	5.87	6.57	5.43	4.28	6.63	#VALUE!
6.84	7.09	5.24	5.27	5.92	6.99	6.76	6.6	6.22	7.13	6.29	5.03	5.87	6.57	5.43	4.28	6.75	#VALUE!
6.84	7.09	5.24	5.27	6.09	7	6.83	6.61	6.32	7.13	6.29	5.03	6.07	6.76	5.45	4.33	6.75	#VALUE!
6.84	7.09	5.24	5.27	6.09	7	6.83	6.61	6.36	7.13	6.29	5.03	6.07	6.76	5.45	4.33	6.75	#VALUE!
6.84	7.17	5.24	5.27	6.09	7.15	6.95	6.61	6.36	7.16	6.29	5.16	6.07	6.76	5.45	4.33	6.82	#VALUE!
6.84	7.17	5.48	5.27	6.09	7.16	6.95	6.61	6.55	7.16	6.4	5.16	6.28	6.76	5.45	4.33	6.82	#VALUE!
6.84	7.17	5.48	5.4	6.15	7.16	7.11	6.73	6.55	7.17	6.4	5.35	6.28	6.9	5.63	4.33	6.83	#VALUE!
7.13	7.25	5.48	5.4	6.15	7.16	7.11	6.73	6.55	7.17	6.4	5.35	6.28	6.9	5.71	4.33	7.22	#VALUE!
7.22	7.25	5.48	5.4	6.32	7.33	7.12	6.92	6.55	7.41	6.41	5.54	6.52	7.27	5.83	4.59	7.32	#VALUE!
7.28	7.25	5.72	5.61	6.32	7.43	7.12	6.92	6.84	7.41	6.57	5.61	6.52	7.27	5.88	4.71	7.32	#VALUE!
7.28	7.49	5.72	5.61	6.32	7.43	7.15	6.92	6.84	7.41	6.57	5.61	6.52	7.27	5.88	4.71	7.32	#VALUE!
7.53	7.49	5.72	5.84	6.42	7.54	7.34	7.13	6.85	7.58	6.57	5.61	6.76	7.6	5.9	4.72	7.32	#VALUE!
7.53	7.74	5.72	5.84	6.42	7.54	7.34	7.13	6.85	7.6	6.82	5.61	6.76	7.6	5.97	4.72	7.32	#VALUE!
7.53	7.74	5.72	5.84	6.63	7.54	7.55	7.37	6.91	7.79	6.82	5.61	6.76	7.6	5.97	4.72	7.59	#VALUE!
7.58	7.75	6.01	5.84	6.83	7.65	7.56	7.37	6.91	7.79	6.82	5.61	6.78	7.6	6.15	4.83	7.59	#VALUE!
7.58	7.75	6.01	5.84	6.83	7.87	7.56	7.37	7.13	7.83	6.98	5.64	6.8	7.8	6.22	5.25	7.59	#VALUE!
7.66	7.92	6.01	6.05	6.83	7.87	7.7	7.48	7.13	7.87	6.98	5.97	6.8	7.88	6.23	5.25	7.61	#VALUE!
7.7	8.14	6.08	6.05	6.83	7.87	7.7	7.48	7.14	7.99	6.98	5.97	6.8	7.89	6.23	5.25	7.68	#VALUE!
7.72	8.14	6.08	6.05	6.92	8.19	7.7	7.48	7.25	8.22	6.98	5.97	6.8	8.29	6.23	5.26	7.88	#VALUE!
7.74	8.29	6.15	6.33	7.01	8.19	7.8	7.49	7.49	8.45	7.12	5.97	6.8	8.29	6.35	5.26	7.88	#VALUE!
7.74	8.29	6.28	6.33	7.01	8.19	7.88	7.49	7.49	8.45	7.12	5.98	7.08	8.29	6.35	5.48	7.88	#VALUE!
8.28	8.29	6.29	6.53	7.01	8.26	7.88	7.78	7.67	8.45	7.12	5.98	7.08	8.29	6.46	5.78	7.99	#VALUE!
8.29	8.54	6.29	6.53	7.01	8.33	8.02	7.78	7.68	8.62	7.43	5.98	7.08	8.3	6.57	6	8.07	#VALUE!
8.29	8.55	6.31	6.53	7.02	8.33	8.11	8.06	7.68	8.63	7.43	5.98	7.08	8.3	6.57	6	8.07	#VALUE!
8.68	8.55	6.31	6.61	7.27	8.5	8.11	8.06	7.77	8.63	7.43	5.98	7.08	8.3	6.57	6	8.26	#VALUE!
8.74	8.55	6.44	6.78	7.27	8.62	8.22	8.24	7.77	8.63	7.43	6.27	7.24	8.3	6.58	6	8.26	#VALUE!
8.74	8.55	6.45	6.78	7.27	8.66	8.22	8.24	7.77	8.63	7.43	6.27	7.25	8.3	6.78	6	8.26	#VALUE!
8.74	8.67	6.45	6.78	7.27	8.66	8.22	8.24	7.93	8.85	7.43	6.29	7.25	8.31	6.78	6	8.26	#VALUE!
8.74	8.67	6.45	6.78	7.27	8.66	8.43	8.41	7.94	9.01	7.52	6.3	7.31	8.31	6.79	6.24	8.38	#VALUE!
8.74	8.67	6.58	6.78	7.27	8.66	8.43	8.42	7.94	9.01	7.52	6.3	7.31	8.31	6.87	6.47	8.46	#VALUE!
8.74	8.67	6.58	7.06	7.33	8.83	8.43	8.42	8.12	9.01	7.52	6.3	7.31	8.46	6.91	6.56	8.54	#VALUE!
8.74	8.69	6.58	7.06	7.35	8.83	8.52	8.42	8.12	9.14	7.63	6.3	7.31	8.46	6.93	6.71	8.54	#VALUE!
8.74	8.69	6.58	7.06	7.48	8.83	8.52	8.44	8.12	9.15	7.63	6.3	7.31	8.46	6.96	6.71	8.55	#VALUE!
8.85	9.13	6.58	7.15	7.48	8.83	8.6	8.44	8.12	9.24	7.63	6.3	7.53	8.85	7.1	6.78	8.55	#VALUE!
8.85	9.13	6.68	7.15	7.48	8.92	8.67	8.58	8.34	9.33	7.79	6.3	7.53	8.85	7.12	6.78	8.7	#VALUE!
8.89	9.13	6.68	7.15	7.49	8.92	8.67	8.58	8.34	9.43	7.79	6.3	7.53	8.85	7.14	6.78	8.7	#VALUE!
8.92	9.13	6.68	7.15	7.5	9.2	8.91	8.66	8.34	9.51	7.79	6.3	7.53	8.85	7.14	6.78	8.7	#VALUE!
9.01	9.13	6.75	7.15	7.66	9.2	8.94	8.66	8.48	9.51	7.9	6.44	7.53	8.85	7.24	6.98	8.8	#VALUE!
9.38	9.2	6.75	7.15	7.66	9.2	8.94	8.66	8.48	9.51	7.9	6.45	7.65	8.86	7.25	7.08	8.8	#VALUE!
9.48	9.2	6.75	7.15	7.66	9.34	8.94	8.85	8.48	9.51	7.9	6.45	7.65	8.86	7.25	7.27	8.8	#VALUE!
9.48	9.2	6.87	7.32	7.66	9.35	8.95	8.85	8.48	9.77	7.91	6.45	7.65	8.86	7.37	7.27	8.8	#VALUE!
9.48	9.21	6.87	7.45	7.68	9.47	8.95	8.85	8.62	9.77	7.91	6.49	7.65	8.86	7.51	7.27	8.8	#VALUE!
9.48	9.22	6.87	7.45	7.78	9.47	8.95	8.85	8.63	9.94	8.12	6.49	7.82	8.86	7.53	7.27	9.01	#VALUE!
9.48	9.22	6.88	7.45	7.78	9.58	9.01	9.17	8.63	9.95	8.12	6.49	7.84	9.17	7.6	7.27	9.04	#VALUE!
9.48	9.39	6.92	7.67	7.79	9.6	9.13	9.17	8.63	9.95	8.12	6.49	7.84	9.17	7.6	7.27	9.07	#VALUE!
9.48	9.4	7.02	7.67	7.82	9.6	9.13	9.17	8.74	9.95	8.12	6.49	7.84	9.17	7.62	7.27	9.07	#VALUE!
9.48	9.4	7.02	7.67	7.83	9.6	9.2	9.17	8.74	9.95	8.15	6.49	7.93	9.17	7.77	7.27	9.07	#VALUE!
9.48	9.4	7.11	7.79	7.94	9.62	9.23	9.17	8.79	9.96	8.15	6.64	7.93	9.18	7.78	7.27	9.11	#VALUE!
9.48	9.43	7.11	7.89	7.95	9.63	9.34	9.17	8.79	9.96	8.15	6.64	7.93	9.18	7.78	7.58	9.11	#VALUE!
9.63	9.43	7.14	7.89	7.95	9.63	9.35	9.39	8.79	9.96	8.51	6.64	8.14	9.18	7.78	7.58	9.43	#VALUE!
9.63	9.43	7.24	7.89	8.08	9.8	9.37	9.4	8.99	10.09	8.51	6.64	8.14	9.18	7.78	7.69	9.43	#VALUE!
9.63	9.43	7.24	7.89	8.12	9.84	9.47	9.43	8.99	10.18	8.51	6.64	8.25	9.59	7.79	7.69	9.43	#VALUE!
10.02	9.53	7.26	8.02	8.12	9.88	9.47	9.43	8.99	10.24	8.72	6.8	8.25	9.61	7.79	7.69	9.69	#VALUE!
10.02	9.53	7.26	8.02	8.17	9.96	9.47	9.43	9.05	10.24	8.72	6.8	8.45	9.61	8	7.69	9.69	#VALUE!
10.07	9.53	7.35	8.16	8.17	10.04	9.65	9.58	9.05	10.24	8.78	6.8	8.45	9.61	8	7.93	9.69	#VALUE!
10.11	9.53	7.36	8.17	8.27	10.19	9.65	9.58	9.05	10.24	8.78	6.8	8.45	9.61	8	7.93	9.84	#VALUE!
10.11	9.66	7.36	8.17	8.27	10.19	9.79	9.58	9.05	10.24	8.78	6.94	8.45	9.66	8.07	7.93	9.84	#VALUE!
10.11	9.68	7.63	8.17	8.37	10.32	9.79	9.66	9.2	10.36	8.88	6.98	8.45	9.66	8.14	7.93	9.84	#VALUE!
10.11	9.68	7.63	8.17	8.37	10.32	9.93	9.66	9.2	10.44	8.88	6.98	8.45	9.66	8.21	8.03	9.85	#VALUE!
10.11	9.68	7.73	8.17	8.37	10.39	9.93	9.86	9.2	10.44	8.88	6.98	8.57	9.66	8.21	8.03	9.86	#VALUE!
10.11	9.84	7.73	8.17	8.37	10.39	9.94	9.86	9.2	10.45	8.88	6.98	8.57	9.82	8.32	8.03	9.87	#VALUE!
10.32	9.84	7.86	8.22	8.37	10.39	9.94	9.86	9.37	10.45	8.96	6.98	8.62	9.82	8.32	8.03	9.87	#VALUE!
10.32	9.84	7.86	8.22	8.52	10.45	10.07	9.86	9.37	10.46	8.96	6.98	8.62	9.82	8.32	8.03	9.87	#VALUE!
10.32	9.89	7.87	8.25	8.52	10.45	10.07	9.86	9.37	10.46	8.96	7	8.8	9.82	8.39	8.03	9.95	#VALUE!
10.56	9.89	7.87	8.25	8.52	10.45	10.07	9.87	9.45	10.46	9.08	7	8.8	9.82	8.39	8.03	9.95	#VALUE!
10.56	9.97	.	.	.	10.51	10.12	9.87	9.45	10.54	.	.	.	9.82	8.43	8.03	9.95	#VALUE!
10.56	9.97	.	.	.	10.51	10.12	9.96	9.53	10.54	.	.	.	9.82	8.43	8.13	10.17	#VALUE!
10.6	9.98	.	.	.	10.51	10.26	9.97	9.53	10.69	.	.	.	9.82	8.56	8.13	10.17	#VALUE!
10.61	10.2	.	.	.	10.74	10.26	10.14	9.53	10.69	.	.	.	10.1	8.56	8.13	10.17	#VALUE!
10.61	10.22	.	.	.	10.74	10.26	10.14	9.68	10.69	.	.	.	10.1	8.56	8.13	10.17	#VALUE!
10.61	10.22	.	.	.	10.74	10.44	10.14	9.68	10.82	.	.	.	10.1	8.58	8.13	10.17	#VALUE!
10.72	10.22	.	.	.	10.75	10.44	10.32	9.68	10.83	.	.	.	10.1	8.58	8.13	10.18	#VALUE!
10.78	10.23	.	.	.	10.84	10.44	10.33	9.68	10.83	.	.	.	10.1	8.66	8.35	10.32	#VALUE!
10.78	10.49	.	.	.	10.84	10.44	10.33	9.79	10.96	.	.	.	10.48	8.66	8.35	10.32	#VALUE!
10.8	10.49	.	.	.	11.04	10.59	10.61	9.79	10.96	.	.	.	10.57	8.74	8.35	10.32	#VALUE!
10.8	10.5	.	.	.	11.15	10.59	10.68	9.91	11.11	.	.	.	10.59	8.74	8.35	10.49	#VALUE!
10.86	10.51	.	.	.	11.15	10.72	10.68	9.91	11.11	.	.	.	10.59	8.97	8.58	10.49	#VALUE!
11.19	10.82	.	.	.	11.3	10.72	10.68	9.91	11.27	.	.	.	10.59	8.97	8.58	10.49	#VALUE!
11.33	10.82	.	.	.	11.41	10.96	10.71	9.99	11.27	.	.	.	10.59	9.09	8.75	10.75	#VALUE!
11.4	10.82	.	.	.	11.41	10.96	10.9	10.19	11.27	.	.	.	10.92	9.09	8.75	10.75	#VALUE!
11.4	11.09	.	.	.	11.41	10.96	10.9	10.19	11.27	.	.	.	10.92	9.09	8.75	10.75	#VALUE!
11.4	11.09	.	.	.	11.52	11.24	10.9	10.19	11.48	.	.	.	10.92	9.22	8.75	10.75	#VALUE!
11.69	11.35	.	.	.	11.56	11.24	10.9	10.19	11.48	.	.	.	10.92	9.25	9.12	10.88	#VALUE!
11.69	11.35	.	.	.	11.71	11.24	10.9	10.31	11.48	.	.	.	11.08	9.31	9.12	11.17	#VALUE!
11.69	11.36	.	.	.	11.71	11.26	10.91	10.44	11.65	.	.	.	11.08	9.33	9.12	11.29	#VALUE!
11.69	11.36	.	.	.	11.97	11.26	10.92	10.44	11.66	.	.	.	11.08	9.54	9.46	11.29	#VALUE!
11.69	11.36	.	.	.	11.97	11.26	10.92	10.5	11.66	.	.	.	11.08	9.61	9.47	11.29	#VALUE!
11.69	11.36	.	.	.	12.05	11.32	11.16	10.7	11.82	.	.	.	11.08	9.67	9.47	11.29	#VALUE!
11.69	11.46	.	.	.	12.22	11.38	11.17	10.7	11.82	.	.	.	11.08	9.67	9.88	11.42	#VALUE!
11.69	11.46	.	.	.	12.31	11.48	11.17	10.7	11.96	.	.	.	11.08	9.79	9.89	11.42	#VALUE!
11.93	11.46	.	.	.	12.31	11.48	11.17	10.7	12.15	.	.	.	11.08	9.81	10.16	11.42	#VALUE!
12	11.73	.	.	.	12.51	11.5	11.39	10.87	12.15	.	.	.	11.43	9.81	10.16	11.7	#VALUE!
12	11.73	.	.	.	12.51	11.62	11.57	10.87	12.24	.	.	.	11.43	9.88	10.16	11.7	#VALUE!
12	11.73	.	.	.	12.51	11.7	11.57	10.87	12.24	.	.	.	11.43	9.92	10.16	11.7	#VALUE!
12	11.73	.	.	.	12.63	11.7	11.58	10.87	12.24	.	.	.	11.43	10	10.16	11.88	#VALUE!
12	11.91	.	.	.	12.77	11.7	11.58	11.03	12.39	.	.	.	11.44	10.06	10.42	12.04	#VALUE!
12.03	11.99	.	.	.	12.77	11.85	11.73	11.03	12.43	.	.	.	11.44	10.1	10.42	12.04	#VALUE!
12.14	11.99	.	.	.	12.77	11.85	11.73	11.11	12.44	.	.	.	11.44	10.3	10.5	12.15	#VALUE!
12.21	11.99	.	.	.	12.77	11.86	11.73	11.11	12.44	.	.	.	11.44	10.3	10.53	12.15	#VALUE!

12.29	11.99	.	.	.	12.77	11.86	11.94	11.11	12.55	.	.	.	11.44	10.33	10.77	12.15	#VALUE!
12.29	12.12	.	.	.	12.82	12.04	11.94	11.22	12.71	.	.	.	11.67	10.44	10.78	12.16	#VALUE!
12.29	12.19	.	.	.	12.83	12.05	11.94	11.22	12.72	.	.	.	11.7	10.44	10.78	12.16	#VALUE!
12.29	12.19	.	.	.	12.88	12.07	11.94	11.22	12.89	.	.	.	11.7	10.44	10.78	12.2	#VALUE!
12.29	12.19	.	.	.	12.88	12.07	11.94	11.31	12.89	.	.	.	11.7	10.53	11.08	12.2	#VALUE!
12.44	12.19	.	.	.	12.9	12.13	11.94	11.31	12.89	.	.	.	11.7	10.53	11.09	12.2	#VALUE!
12.44	12.19	.	.	.	13.04	12.24	12.15	11.5	12.89	.	.	.	11.7	10.67	11.09	12.3	#VALUE!
12.44	12.36	.	.	.	13.06	12.24	12.15	11.5	12.94	.	.	.	11.7	10.67	11.09	12.3	#VALUE!
12.49	12.36	.	.	.	13.06	12.55	12.38	11.61	12.94	.	.	.	11.7	10.8	11.09	12.31	#VALUE!
12.5	12.36	.	.	.	13.37	12.55	12.4	11.61	13.13	.	.	.	11.7	10.83	11.42	12.32	#VALUE!
12.55	12.38	.	.	.	13.39	12.56	12.4	11.61	13.24	.	.	.	11.7	10.88	11.42	12.42	#VALUE!
12.63	12.51	.	.	.	13.42	12.56	12.4	11.7	13.24	.	.	.	12.05	10.92	11.42	12.52	#VALUE!
12.64	12.51	.	.	.	13.48	12.56	12.4	11.8	13.28	.	.	.	12.05	10.92	11.42	12.53	#VALUE!
12.91	12.75	.	.	.	13.56	12.64	12.4	11.8	13.29	.	.	.	12.05	10.97	11.53	12.62	#VALUE!
12.95	12.75	.	.	.	13.64	12.64	12.42	11.84	13.3	.	.	.	12.32	11.06	11.57	12.62	#VALUE!
12.95	12.75	.	.	.	13.64	12.69	12.55	11.85	13.31	.	.	.	12.32	11.08	11.57	12.62	#VALUE!
13.18	12.75	.	.	.	13.65	12.91	12.55	11.92	13.35	.	.	.	12.32	11.09	11.57	12.62	#VALUE!
13.18	12.75	.	.	.	13.74	13.09	12.61	11.92	13.35	.	.	.	12.45	11.21	11.65	12.74	#VALUE!
13.25	12.86	.	.	.	13.74	13.12	12.61	11.92	13.35	.	.	.	12.45	11.21	11.65	12.74	#VALUE!
13.25	12.87	.	.	.	13.85	13.12	12.83	12.01	13.56	.	.	.	12.45	11.21	11.65	12.74	#VALUE!
13.25	12.87	.	.	.	13.85	13.34	12.85	12.01	13.56	.	.	.	12.76	11.34	11.65	13.03	#VALUE!
13.25	12.87	.	.	.	13.98	13.35	12.85	12.05	13.56	.	.	.	12.76	11.34	11.65	13.03	#VALUE!
13.25	13.04	.	.	.	13.98	13.35	12.92	12.06	13.68	.	.	.	12.76	11.43	11.75	13.04	#VALUE!
13.26	13.04	.	.	.	13.99	13.35	12.92	12.06	13.69	.	.	.	12.76	11.43	11.75	13.04	#VALUE!
13.44	13.04	.	.	.	13.99	13.36	12.96	12.08	13.71	.	.	.	12.76	11.43	11.75	13.04	#VALUE!
13.5	13.04	.	.	.	13.99	13.44	12.96	12.08	13.71	.	.	.	12.76	11.58	11.75	13.06	#VALUE!
13.5	13.15	.	.	.	13.99	13.44	12.96	12.3	13.72	.	.	.	12.76	11.58	11.91	13.06	#VALUE!
13.5	13.15	.	.	.	14.07	13.44	12.96	12.3	13.72	.	.	.	12.76	11.58	11.92	13.07	#VALUE!
13.5	13.24	.	.	.	14.07	13.45	12.96	12.31	13.72	.	.	.	12.95	11.59	11.92	13.27	#VALUE!
13.5	13.24	.	.	.	14.08	**13.55**	**13.14**	12.31	13.72	.	.	.	12.95	**11.71**	**11.92**	13.27	#VALUE!
																13.27	#VALUE!
																13.27	#VALUE!
																	.
																	.
																	.
																	.
