# Supplementary material for: Does prenatal alcohol exposure cause a metabolic syndrome? (Non-)evidence from a mouse model of fetal alcohol spectrum disorder
Source: PLoS One. 2018 Jun 28;13(6):e0199213. doi: 10.1371/journal.pone.0199213 (PMC6023152; doi:10.1371/journal.pone.0199213)
Supplement: S1 Dataset — (ZIP) [file pone.0199213.s010.zip › New folder/IPGTT.pdf]

Mouse	Litter	SUBJID	Group	SEX	BW	0	15	30	60	120	AUC
1.3	1	3	MD	M	26.5	160	364	349	373	183	36787.5
1.4	1	4	MD	F	21.8	116	285	207	195	125	22327.5
2.1	2	1	MD	M	25.4	179	270	307	288	256	32940
2.4	2	4	MD	F	21.4	135	285	241	226	179	26250
5.2	5	2	H2O	F	19.2	170	273	253	217	177	26137.5
6.2	6	2	ETOH	M	24.8	212	235	205	198	232	25597.5
9.3	9	3	ETOH	M	24.5	157	282	273	216	222	27930
9.7	9	7	ETOH	F	21.1	146	321	241	233	239	28987.5
10.2	10	2	ETOH	M	25	220	258	229	247	201	27817.5
10.8	10	8	ETOH	F	21.3	183	261	285	210	211	27480
12.3	12	3	H2O	M	25.1	118	272	278	216	202	27000
13.7	13	7	MCT	F	20.1	152	228	266	192	216	25665
14.6	14	6	MD	F	27.2	220	288	325	239	179	29407.5
16.5	16	5	ETOH	M	27.7	176	317	353	256	180	30937.5
17.3	17	3	ETOH	M	26.2	170	343	323	250	167	29947.5
17.7	17	7	ETOH	F	21.4	95	180	304	260	181	27382.5
18.2	18	2	ETOH	M	26.4	193	220	245	252	215	28050
18.6	18	6	ETOH	F	22.5	136	283	294	267	235	30945
19.2	19	2	ETOH	M	29.4	181	347	342	295	150	32032.5
19.4	19	4	MD	F	20.9	143	345	304	260	158	29527.5
20.3	20	3	MCT	M	26.7	140	250	236	174	171	23070
20.5	20	5	MCT	F	21.1	139	334	305	222	163	27795
21.2	21	2	MCT	M	24.5	162	308	210	204	155	24390
21.6	21	6	MCT	F	20.2	146	304	270	159	159	23655
23.2	23	2	MD	M	19.2	194	332	314	171	198	27135
24.3	24	3	MD	F	20.1	101	286	236	215	196	25912.5
25.4	25	4	MD	F	19.2	154	332	327	240	168	29332.5
26.2	26	2	MD	M	28.1	167	319	349	242	228	31620
26.6	26	6	MD	F	22.2	170	419	361	273	210	34267.5
27.3	27	3	H2O	F	20.1	171	316	221	217	215	27210
28.2	28	2	ETOH	F	21.5	119	301	299	253	152	28080
32.2	32	2	MD	M	25.7	175	370	371	269	167	32325
32.5	32	5	MD	F	20.2	95	180	210	211	124	21352.5
34.1	34	1	MCT	M	23.2	141	429	363	352	214	37920
34.5	34	5	MCT	F	18.3	157	319	321	288	126	29925
37.3	37	3	H2O	M	27.1	146	186	211	160	199	21802.5
38.3	38	3	MD	M	27	185	283	333	186	199	27465
38.6	38	6	MD	F	20.7	112	255	279	153	164	22747.5
40.3	40	3	MCT	F	22.4	104	290	331	188	90	23737.5
41.3	41	3	ETOH	M	26.3	175	337	306	304	254	34552.5
42.2	42	2	ETOH	M	26.3	125	285	172	184	141	21592.5
42.3	42	3	ETOH	F	22.2	110	261	375	229	128	27322.5
43.2	43	2	MD	M	26.5	194	313	361	334	261	37132.5
43.5	43	5	MD	F	21.2	88	274	262	150	136	21495
44.1	44	1	MCT	M	28.9	112	244	266	192	116	22605
44.8	44	8	MCT	F	20.9	109	284	240	210	97	22837.5
45.1	45	1	MCT	M	25.4	130	214	210	197	125	21525
45.4	45	4	MCT	F	22.3	121	279	248	273	135	27007.5
46.2	46	2	H2O	M	25.4	188	337	301	29.7	212	20934
46.5	46	5	H2O	F	19.8	115	255	292	190	178	25147.5
47.4	47	4	MCT	M	25	200	356	380	350	258	38880
50.3	50	3	H2O	M	25.3	93	245	257	186	167	23535
50.5	50	5	H2O	F	21.9	65	197	175	152	86	16800
52.1	52	1	MD	M	27.3	114	282	225	208	159	24277.5
52.5	52	5	MD	F	21.4	91	240	234	202	95	21487.5
53.1	53	1	ETOH	M	26.9	132	248	289	236	154	26452.5
53.6	53	6	ETOH	F	21.8	67	232	271	225	105	23355
78.5				M	30.2	200	260	182	133	102	18540
77.2				M	28.5	183	216	147	133	91	16635
79.8				M	32.6	174	231	182	152	93	18495
75.5				M	28.1	197	296	224	181	218	25642.5
74.2				M	27.9	225	267	248	250	186	28102.5
76.1				M	29	178	244	189	127	189	20632.5
77.6				F	21.7	162	334	291	184	113	24442.5
78.7				F	22.1	174	238	205	156	136	20587.5
82.1				F	21.7	187	227	281	235	207	27915
74.5				F	22.8	176	272	182	183	157	22440
76.4				F	22	146	234	172	169	141	20310
79.6				F	22.2	150	334	201	156	118	21217.5
62.4				M	24.5	133	276	236	194	143	23467.5
65.3				M	26.3	171	334	331	298	178	32490
68.1				M	24	188	353	287	287	158	30817.5
71.4				M	26	132	176	242	171	136	20850
59.5				M	27.1	161	306	256	217	186	26902.5
63.1				M	27.4	157	268	271	226	155	26115
64.3				M	28	123	275	206	160	133	20872.5
71.8				F	21.7	182	245	181	153	66	17977.5
63.6				F	20.3	134	339	282	217	79	24570
69.1				F	21.4	171	376	359	302	134	32610
64.6				F	21.4	107	264	266	157	86	20392.5
65.5				F	19.9	143	222	227	170	129	21030
68.3				F	22.5	144	318	326	209	220	29190
