# Supplementary material for: Does prenatal alcohol exposure cause a metabolic syndrome? (Non-)evidence from a mouse model of fetal alcohol spectrum disorder
Source: PLoS One. 2018 Jun 28;13(6):e0199213. doi: 10.1371/journal.pone.0199213 (PMC6023152; doi:10.1371/journal.pone.0199213)
Supplement: S1 Dataset — (ZIP) [file pone.0199213.s010.zip › New folder/OGTT HF 4wks.pdf]

Mouse	Group	Sex	BW	Baseline	15	30	60	120	AUC
6.4	ETOH	F	25.5	249	393	388	292	240	36832.5
9.6	ETOH	F	28.3	220	468	437	372	369	46312.5
10.6	ETOH	F	26.2	262	433	477	372	328	45772.5
11.1	ETOH	F	19.8	200	442	349	370	331	42562.5
17.8	ETOH	F	22.8	.	.	.	.	.	.
18.7	ETOH	F	25.8	172	446	383	248	203	33847.5
28.7	ETOH	F	28.9	178	361	382	241	183	31680
53.5	ETOH	F	23.8	172	188	259	198	201	24877.5
6.1	ETOH	M	33	220	363	229	265	219	30742.5
9.4	ETOH	M	37.3	228	341	252	265	210	30720
10.1	ETOH	M	40.6	254	407	358	283	271	36930
11.6	ETOH	M	33.7	255	345	279	298	246	34155
16.4	ETOH	M	39.7	231	421	284	318	315	38197.5
17.4	ETOH	M	42.6	264	432	328	363	323	41865
28.1	ETOH	M	37.3	176	274	235	239	214	27892.5
53.2	ETOH	M	34	168	376	316	219	181	29295
82.6	ETOH	M	45.7	275	337	282	346	233	36022.5
12.4	H2O	F	32.4	232	486	290	258	254	34785
23.1	H2O	F	28.8	178	306	339	252	224	31612.5
27.4	H2O	F	29.5	172	430	410	347	309	41850
50.6	H2O	F	29.8	212	316	390	361	302	40410
74.3	H2O	F	36	188	390	241	281	266	33307.5
76.3	H2O	F	36.1	203	324	288	287	227	32587.5
83.6	H2O	F	38.4	192	348	275	217	184	28132.5
81.1	H2O	F	35.8	230	348	397	309	201	35812.5
12.1	H2O	M	42.2	317	345	231	277	263	33105
23.3	H2O	M	36.6	217	368	327	338	316	39195
27.5	H2O	M	40.2	269	421	336	297	218	35797.5
76.2	H2O	M	50.4	252	421	360	257	264	35790
83.1	H2O	M	47.7	214	298	220	229	210	27630
81.6	H2O	M	46.6	219	371	282	236	216	30652.5
74.1	H2O	M	38.7	213	371	345	323	191	35190
13.3	MCT	F	30.6	198	467	405	296	239	38092.5
21.5	MCT	F	22.4	141	330	244	242	201	28417.5
32.6	MCT	F	29.8	184	340	251	208	231	28417.5
44.6	MCT	F	25.9	171	473	344	265	238	35182.5
77.4	MCT	F	34	167	275	337	247	223	30765
78.8	MCT	F	37.6	192	351	251	200	176	26632.5
84.4	MCT	F	38.7	182	349	307	251	226	31582.5
13.7	MCT	M	39.6	292	386	358	308	330	39795
20.2	MCT	M	35.1	214	365	406	346	307	40995
32.3	MCT	M	41.2	274	460	361	256	225	35347.5
44.5	MCT	M	401	209	329	343	363	221	37185
84.1	MCT	M	45.9	219	321	235	230	232	29055
78.1	MCT	M	40.3	197	393	260	240	213	30412.5
1.7	MD	F	25.2	199	410	282	232	259	32197.5
2.3	MD	F	26.9	268	384	412	361	310	42585
25.3	MD	F	28.3	157	370	275	269	221	31650
26.6	MD	F	26.1	191	450	347	367	246	39885
34.6	MD	F	30.1	185	330	419	347	236	38460
52.6	MD	F	26	162	337	334	279	231	33270
1.1	MD	M	34.5	229	297	301	247	297	32970
2.2	MD	M	35.8	247	383	323	270	289	35685
5.5	MD	M	34.9	258	415	417	326	251	39742.5
19.1	MD	M	38.3	196	441	401	337	296	41152.5
25.1	MD	M	39.6	211	448	425	410	371	47445
34.2	MD	M	30.7	163	453	371	341	265	39660
52.1	MD	M	36.1	182	338	399	301	295	37807.5
52.2	MD	M	40.9	198	363	363	274	174	32647.5
