# Supplementary material for: Does prenatal alcohol exposure cause a metabolic syndrome? (Non-)evidence from a mouse model of fetal alcohol spectrum disorder
Source: PLoS One. 2018 Jun 28;13(6):e0199213. doi: 10.1371/journal.pone.0199213 (PMC6023152; doi:10.1371/journal.pone.0199213)
Supplement: S1 Dataset — (ZIP) [file pone.0199213.s010.zip › New folder/OGTT.pdf]

Mouse	Litter	SUBJID	Group	Sex	BW	Fasting	15	30	60	120	AUC
1.3	1	3	MD	M	25.6	142	351	302	254	221	31185
1.4	1	4	MD	F	22.5	81	312	183	170	175	22305
2.1	2	1	MD	M	25.7	162	354	321	255	224	31942.5
2.4	2	4	MD	F	21.1	184	333	230	206	185	26370
5.2	5	2	H2O	F	18.8	128	313	228	221	210	27030
6.2	6	2	ETOH	M	24.4	265	383	282	287	253	34582.5
9.3	9	3	ETOH	M	24.1	186	277	230	218	211	26865
9.7	9	7	ETOH	F	21	131	240	247	190	189	24360
10.2	10	2	ETOH	M	24.1	188	387	313	240	190	30757.5
10.8	10	8	ETOH	F	19.9	102	259	205	162	135	20602.5
11.8	11	8	ETOH	M	24.4	191	338	193	221	236	27870
12.3	12	3	H2O	M	25.9	186	225	239	140	230	23347.5
13.7	13	7	MCT	F	20.4	119	316	265	256	192	28875
14.6	14	6	MD	F	19.9	181	398	372	269	208	34042.5
16.5	16	5	ETOH	M	27.4	123	345	365	272	230	33450
17.3	17	3	ETOH	M	25.7	136	287	219	225	197	26287.5
17.7	17	7	ETOH	F	22.1	130	272	256	243	220	28350
18.2	18	2	ETOH	M	28.7	208	408	310	279	221	33840
18.6	18	6	ETOH	F	22.4	106	304	250	213	123	24255
19.2	19	2	ETOH	M	25	185	331	297	260	221	31365
19.4	19	4	MD	F	20.1	121	220	238	224	172	24802.5
20.3	20	3	MCT	M	27.6	166	274	203	199	215	25327.5
20.5	20	5	MCT	F	21.4	196	384	321	183	193	28477.5
21.2	21	2	MCT	M	24.7	174	332	332	223	192	29550
21.6	21	6	MCT	F	20	137	316	217	171	182	23805
23.2	23	2	MD	M	26.9	169	328	270	229	176	27847.5
24.3	24	3	MD	F	19.2	131	217	208	208	157	22987.5
25.4	25	4	MD	F	18.5	106	354	254	162	142	23370
26.2	26	2	MD	M	27.5	188	310	318	271	206	31590
26.6	26	6	MD	F	21.4	118	310	277	235	196	28222.5
27.3	27	3	H2O	F	20.5	120	308	259	189	171	24982.5
28.2	28	2	ETOH	F	20.2	135	262	199	140	130	19620
32.2	32	2	MCT	M	24.8	149	296	259	259	213	29430
32.5	32	5	MCT	F	20.5	147	253	208	164	177	22267.5
34.1	34	1	MD	M	23.3	182	339	305	305	225	33787.5
34.5	34	5	MD	F	18.4	148	392	320	243	158	29865
37.3	37	3	H2O	M	27.7	139	302	298	220	171	27307.5
37.6	37	6	H2O	F	20.9	73	226	308	211	147	24772.5
38.3	38	3	MD	M	26.2	111	267	199	184	174	22815
38.6	38	6	MD	F	19.6	103	270	181	153	97	18690
40.3	40	3	MCT	F	22.4	90	286	300	148	122	22035
41.3	41	3	ETOH	M	26.1	144	236	225	175	187	23167.5
42.2	42	2	ETOH	M	26.8	170	315	282	189	218	27390
42.3	42	3	ETOH	F	20.8	127	300	309	247	176	28800
43.2	43	2	MD	M	24.8	150	313	240	234	248	29190
43.5	43	5	MD	F	20	86	308	356	140	80	21975
44.1	44	1	MCT	M	28.3	118	313	265	224	185	27172.5
44.8	44	8	MCT	F	20.8	102	355	230	172	144	23325
45.1	45	1	MCT	M	26.1	212	320	314	243	251	31920
45.4	45	4	MCT	F	22.3	122	322	251	198	167	25312.5
46.2	46	2	H2O	M	25.7	174	269	321	238	225	30022.5
46.5	46	5	H2O	F	19.8	122	288	308	181	132	24270
47.4	47	4	MCT	M	24.8	207	285	299	267	221	31200

50.3	50	3	H2O	M	24.4	82	234	237	141	151	20332.5
50.5	50	5	H2O	F	21.5	80	220	239	130	105	18277.5
52.1	52	1	MD	M	25.9	118	315	276	203	238	28095
52.5	52	5	MD	F	20.4	94	208	230	105	109	16995
53.1	53	1	ETOH	M	25.4	123	331	307	248	187	29565
53.6	53	6	ETOH	F	20.5	97	314	212	137	121	20002.5
62.4	62	4	H2O	M	24.8	148	261	234	183	235	25575
63.1	63	1	H2O	M	27	178	199	269	162	181	23092.5
63.6	63	6	H2O	F	20.7	110	232	161	169	160	20332.5
64.3	64	3	MD	M	27.8	136	229	260	216	143	24315
64.6	64	6	MD	F	22	136	251	174	196	158	22260
65.3	65	3	MCT	M	26.3	189	247	255	192	175	24750
65.5	65	5	MCT	F	19.7	175	251	177	193	161	22575
68.1	68	1	MCT	M	23.7	194	300	266	233	153	27015
68.3	68	3	MCT	F	23.1	153	344	286	176	150	25162.5
69.1	69	1	H2O	F	21.8	135	279	200	214	158	24067.5
69.5	69	5	H2O	M	27.3	170	320	301	180	199	26917.5
71.4	71	4	ETOH	M	26.2	143	200	184	170	171	20992.5
71.8	71	8	ETOH	F	21.8	117	250	218	222	157	24232.5
74.2	74	2	H2O	M	27	141	232	190	174	176	21922.5
74.5	74	5	H2O	F	20.8	114	222	232	178	181	22845
75.5	75	5	MD	M	27.8	162	367	208	181	233	26535
76.1	76	1	H2O	M	28.6	158	207	167	157	214	21532.5
76.4	76	4	H2O	F	21.9	113	321	233	164	178	23625
77.2	77	2	MCT	M	27.5	168	193	158	159	219	21435
77.6	77	6	MCT	F	22.3	143	316	243	149	132	21945
78.5	78	5	MCT	M	27.7	175	235	187	173	183	22320
78.7	78	7	MCT	F	21.7	133	181	175	152	150	18990
79.1	79	1	H2O	M	29.4	109	216	176	162	124	19027.5
79.6	79	6	H2O	F	20.1	96	167	135	125	130	15787.5
82.1	82	1	ETOH	F	22.6	157	243	225	180	160	22785
