# Supplementary material for: Does prenatal alcohol exposure cause a metabolic syndrome? (Non-)evidence from a mouse model of fetal alcohol spectrum disorder
Source: PLoS One. 2018 Jun 28;13(6):e0199213. doi: 10.1371/journal.pone.0199213 (PMC6023152; doi:10.1371/journal.pone.0199213)
Supplement: S1 Dataset — (ZIP) [file pone.0199213.s010.zip › New folder/RER.pdf]

Blk	3	3	3	3	4	4	5	7	7	8	3	4	4	7	7	8	10
Box	Box-3	Box-4	Box-5	Box-6	Box-2	Box-3	Box-4	Box-3	Box-4	Box-4	Box-7	Box-5	Box-8	Box-1	Box-7	Box-2	Box-6
ID	6.4	9.6	10.6	11.1	17.8	18.7	28.7	41	42	53.5	12.4	23.1	27.7	37	46	50.6	62
Sex	F	F	F	F	F	F	F	F	F	F	F	F	F	F	F	F	F
Exposure	EtOH	EtOH	EtOH	EtOH	EtOH	EtOH	EtOH	EtOH	EtOH	EtOH	H2O	H2O	H2O	H2O	H2O	H2O	H2O
Tx	FE	FE	FE	FE	FE	FE	FE	FE	FE	FE	FH	FH	FH	FH	FH	FH	FH
Chow	0.716	0.713	0.71	0.812	0.725	0.766	0.656	0.714	0.696	0.689	0.733	0.729	0.697	0.667	0.785	0.679	0.715
	0.76	0.751	0.74	0.852	0.765	0.773	0.695	0.686	0.76	0.657	0.745	0.695	0.718	0.75	0.762	0.67	0.738
	0.737	0.728	0.749	0.817	0.74	0.761	0.715	0.766	0.837	0.727	0.74	0.695	0.715	0.753	0.745	0.721	0.71
	0.703	0.748	0.709	0.797	0.74	0.754	0.785	0.812	0.918	0.844	0.738	0.73	0.717	0.75	0.693	0.746	0.746
	0.73	0.723	0.71	0.771	0.705	0.737	0.831	0.87	0.947	0.883	0.714	0.718	0.716	0.689	0.764	0.739	0.755
	0.719	0.726	0.819	0.851	0.731	0.744	0.849	0.876	0.935	0.862	0.719	0.701	0.704	0.707	0.73	0.713	0.734
	0.711	0.698	0.818	0.86	0.725	0.753	0.843	0.873	0.896	0.85	0.738	0.738	0.7	0.713	0.677	0.656	0.707
	0.719	0.739	0.849	0.866	0.724	0.769	0.874	0.931	0.921	0.915	0.739	0.725	0.712	0.72	0.708	0.71	0.733
	0.737	0.729	0.923	0.918	0.695	0.776	0.875	0.902	0.866	0.933	0.736	0.704	0.723	0.718	0.722	0.716	0.725
	0.712	0.725	0.875	0.925	0.735	0.826	0.822	0.934	0.919	0.935	0.753	0.739	0.756	0.717	0.714	0.717	0.727
	0.723	0.719	0.886	0.907	0.702	0.878	0.87	0.926	0.937	0.963	0.711	0.796	0.789	0.707	0.829	0.735	0.731
	0.716	0.721	0.891	0.903	0.685	0.905	0.872	0.895	0.966	0.934	0.749	0.878	0.833	0.697	0.901	0.649	0.713
	0.701	0.724	0.894	0.912	0.713	0.895	0.834	0.865	0.99	0.913	0.785	0.891	0.9	0.684	0.907	0.694	0.714
	0.711	0.781	0.917	0.898	0.718	0.882	0.856	0.867	0.998	0.954	0.881	0.918	0.89	0.74	0.984	0.787	0.706
	0.706	0.872	0.912	0.917	0.73	0.913	0.831	0.822	0.994	0.946	0.94	0.926	0.932	0.841	0.974	0.821	0.705
	0.806	0.894	0.937	0.889	0.711	0.926	0.869	0.819	1.032	0.937	0.928	0.919	0.897	0.901	1	0.869	0.679
	0.857	0.879	0.954	0.916	0.727	0.927	0.866	0.77	1.004	0.914	0.908	0.902	0.943	0.893	0.992	0.887	0.646
	0.888	0.897	0.923	0.906	0.717	0.93	0.879	0.816	0.995	0.937	0.95	0.921	0.94	0.924	0.977	0.901	0.706
	0.891	0.893	0.936	0.959	0.729	0.909	0.893	0.834	0.966	0.935	0.937	0.925	0.918	0.911	0.966	0.876	0.72
	0.855	0.904	0.935	0.895	0.727	0.913	0.892	0.835	1.019	0.954	0.945	0.942	0.942	0.918	0.978	0.894	0.721
	0.867	0.924	0.984	0.929	0.689	0.928	0.866	0.807	1.047	0.952	0.92	0.937	0.909	0.904	0.991	0.937	0.705
	0.916	0.917	0.962	0.88	0.7	0.903	0.873	0.741	1.089	0.907	0.915	0.917	0.93	0.939	1.004	0.932	0.752
	0.923	0.916	0.95	0.877	0.695	0.903	0.942	0.67	1.04	0.933	0.906	0.988	0.928	0.935	0.996	0.931	0.791
	0.916	0.92	0.941	0.923	0.691	0.914	0.87	0.718	1.003	0.898	0.907	0.936	0.966	0.931	0.987	0.906	0.838
	0.947	0.906	0.926	0.921	0.807	0.983	0.909	0.776	1.035	0.795	0.932	0.974	0.976	0.936	0.977	0.886	0.834
	0.937	0.894	0.962	0.939	0.697	0.915	0.932	0.814	0.951	0.779	0.926	0.924	0.953	0.922	0.972	0.882	0.837
	0.919	0.921	0.943	0.89	0.681	0.923	0.929	0.809	0.943	0.826	0.919	0.907	0.949	1.027	1.039	0.97	0.817
	0.938	0.952	0.974	0.876	0.711	0.933	0.947	0.758	0.881	0.741	0.924	0.986	0.984	0.957	1.042	0.967	0.813
	0.932	0.96	0.962	0.847	0.704	0.916	0.91	0.636	0.763	0.697	0.946	0.965	0.965	0.943	0.987	0.936	0.781
	0.921	0.96	0.962	0.818	0.823	0.948	0.886	0.714	0.773	0.753	0.913	0.991	0.948	0.957	0.997	0.968	0.811
	0.918	0.975	0.955	0.81	0.707	0.936	0.896	0.708	0.718	0.812	0.875	0.994	0.893	0.941	0.983	0.959	0.751
	0.934	0.956	0.933	0.799	0.706	0.879	0.883	0.784	0.752	0.827	0.914	0.959	0.943	1.012	1.017	0.899	0.704
	0.928	0.941	0.919	0.81	0.757	0.869	0.857	0.826	0.759	0.815	0.919	0.954	0.959	0.948	0.982	0.916	0.74
	0.902	0.928	0.98	0.808	0.838	0.83	0.969	0.792	0.774	0.771	0.904	0.963	0.95	0.944	1.006	0.925	0.751
	0.896	0.927	0.949	0.742	0.863	0.887	0.897	0.704	0.757	0.671	0.896	0.971	0.921	0.964	0.995	0.945	0.747
	0.88	0.933	0.985	0.75	0.884	0.927	0.918	0.749	0.733	0.689	0.889	0.955	0.94	0.962	0.998	0.991	0.731
	0.887	0.94	0.979	0.753	0.882	0.922	0.912	0.773	0.672	0.706	0.896	0.963	0.937	0.966	0.98	0.98	0.712
	0.872	0.955	0.999	0.862	0.913	0.959	0.893	0.851	0.639	0.746	0.888	1.023	0.939	0.911	0.941	0.944	0.719
	0.906	0.971	0.967	0.93	0.879	0.91	0.885	0.881	0.712	0.833	0.925	1.008	0.92	0.93	0.993	0.937	0.771
	0.885	0.976	1.006	0.912	0.899	0.926	0.872	0.863	0.699	0.87	0.905	0.977	0.875	0.957	1.02	0.975	0.834
	0.851	0.932	0.956	0.859	0.884	0.931	0.923	0.794	0.576	0.851	0.862	0.987	0.912	0.967	1.038	0.932	0.868
	0.864	0.96	0.965	0.887	0.894	0.936	0.916	0.842	0.689	0.88	0.849	1.009	0.954	0.954	0.981	0.957	0.864
	0.901	0.965	0.969	0.907	0.876	0.861	0.972	0.848	0.732	0.858	0.877	0.961	0.909	0.943	0.967	1	0.847
	0.873	0.935	0.954	0.913	0.88	0.904	0.898	0.864	0.661	0.868	0.876	0.984	0.928	0.955	1.007	0.916	0.758
	0.891	0.952	0.993	0.935	0.876	0.873	0.928	0.812	0.657	0.896	0.841	0.969	0.876	0.962	0.981	0.941	0.829
	0.87	0.945	0.952	0.93	0.919	0.905	0.965	0.763	0.663	0.889	0.784	0.981	0.892	0.983	0.948	0.911	0.736
	0.9	0.935	0.927	0.905	0.932	0.914	0.912	0.828	0.669	0.889	0.749	0.984	0.925	0.966	1.028	0.906	0.757
	0.913	0.934	0.944	0.899	0.889	0.945	0.922	0.871	0.769	0.885	0.843	1.018	0.902	0.986	1.046	0.896	0.77
	0.919	0.938	0.951	0.9	0.918	0.926	0.89	0.787	0.827	0.843	0.888	0.994	0.902	0.992	0.999	0.922	0.72
	0.906	0.959	0.943	0.901	0.911	0.917	0.887	0.783	0.889	0.874	0.879	1.005	0.878	1.003	1.015	0.898	0.743
	0.917	0.951	0.931	0.892	0.897	0.925	0.895	0.717	0.933	0.827	0.824	1.027	0.922	0.996	0.99	0.854	0.749
	0.92	0.919	0.94	0.832	0.924	0.986	0.87	0.743	0.835	0.807	0.807	1.032	0.909	0.969	0.978	0.886	0.808
	0.924	0.956	0.972	0.838	0.911	0.953	0.89	0.719	0.81	0.863	0.819	0.962	0.877	1.009	1.039	0.917	0.79
	0.919	0.927	0.936	0.87	0.916	0.935	0.905	0.751	0.785	0.885	0.778	0.993	0.929	0.976	0.978	0.912	0.794
	0.925	0.889	0.939	0.906	0.888	0.909	0.932	0.727	0.853	0.878	0.818	0.983	0.899	1.015	0.978	0.872	0.783
	0.958	0.875	0.91	0.935	0.9	0.889	0.93	0.75	0.936	0.864	0.9	0.951	0.889	0.978	0.996	0.919	0.785
	0.96	0.895	0.93	0.922	0.918	0.901	0.855	0.735	0.962	0.898	0.891	0.97	0.909	0.993	1.017	0.933	0.809
	0.967	0.852	0.902	0.92	0.923	0.854	0.883	0.647	0.915	0.91	0.893	0.93	0.886	1.043	0.978	0.902	0.801
	0.943	0.804	0.927	0.878	0.935	0.899	0.946	0.705	0.911	0.943	0.917	0.937	0.888	1.016	0.973	0.93	0.833
	0.936	0.77	0.947	0.901	0.921	0.938	0.885	0.732	0.956	0.927	0.925	0.945	0.891	1	0.996	0.94	0.754
	0.951	0.8	0.926	0.939	0.949	0.898	0.92	0.684	0.948	0.905	0.893	0.938	0.956	1.003	1.008	0.905	0.695
	0.96	0.825	0.946	0.949	0.949	0.932	0.972	0.746	0.935	0.949	0.87	0.948	0.941	0.996	0.948	0.926	0.748
	0.951	0.863	0.969	0.953	0.939	0.912	0.887	0.75	0.876	0.94	0.902	0.949	0.924	0.958	0.978	0.975	0.698
	0.978	0.816	0.967	0.943	0.937	0.957	0.932	0.875	0.932	0.946	0.909	0.956	0.934	1.019	1.026	0.95	0.738
	0.983	0.903	0.942	0.942	0.931	0.943	0.961	0.848	0.927	0.973	0.886	0.953	0.915	0.987	0.931	0.976	0.798
	0.963	0.931	0.989	0.957	0.948	0.963	0.918	0.923	0.945	0.9	0.93	1.019	0.93	1.003	0.99	0.941	0.83
	0.976	0.876	0.965	0.935	0.948	0.971	0.942	0.94	0.964	0.951	0.892	1.012	0.87	1.029	0.971	0.961	0.832
	0.965	0.878	0.97	0.95	0.946	0.907	0.94	0.941	0.932	0.945	0.873	0.959	0.82	0.978	0.923	0.958	0.786
	0.931	0.907	0.96	0.937	0.923	0.866	0.927	0.919	0.981	0.966	0.827	0.957	0.88	1	0.954	0.95	0.782
	0.924	0.867	0.951	0.915	0.946	0.863	0.96	0.916	0.978	0.938	0.797	0.959	0.931	0.954	0.931	0.954	0.77
	0.96	0.895	0.94	0.926	0.927	0.921	0.952	0.936	0.955	0.915	0.842	0.965	0.881	0.96	0.869	0.95	0.797
	0.974	0.936	0.958	0.914	0.949	0.888	0.938	0.897	0.976	0.955	0.862	0.932	0.851	0.962	0.775	0.997	0.857
	0.932	0.932	0.947	0.906	0.927	0.895	0.939	0.91	0.968	0.988	0.859	0.945	0.916	0.99	0.922	1.007	0.86
	0.929	0.902	0.957	0.929	0.957	0.882	0.971	0.929	0.959	0.968	0.801	0.948	0.903	0.983	0.965	0.96	0.863
	0.938	0.934	0.948	0.883	0.94	0.817	0.962	0.9	0.967	0.942	0.781	0.955	0.885	1.002	0.958	1	0.874
	0.915	0.902	0.939	0.845	0.938	0.816	0.925	0.928	0.961	0.959	0.86	0.946	0.937	0.934	0.931	0.927	0.892
	0.922	0.951	0.951	0.884	0.993	0.81	0.979	0.937	0.987	0.955	0.894	0.971	0.941	0.966	0.988	0.953	0.933
	0.969	0.972	0.957	0.915	0.973	0.847	0.925	0.901	1.001	0.938	0.863	0.954	0.931	0.988	0.95	0.964	0.891
	0.954	0.949	0.977	0.911	0.99	0.916	1.005	0.93	0.991	0.935	0.843	0.974	0.935	0.994	0.956	0.972	0.896
	0.933	0.946	0.961	0.848	0.98	0.898	0.954	0.964	0.99	0.901	0.916	0.947	0.942	1.003	0.959	0.977	0.929
	0.929	0.956	0.969	0.824	0.969	0.903	0.971	0.919	0.984	0.964	0.912	0.95	0.964	1.003	0.952	0.996	0.921
	0.94	0.941	0.977	0.86	0.993	0.936	0.962	0.945	0.98	0.911	0.911	0.931	0.945	0.97	0.973	0.926	0.88
	0.938	0.965	0.966	0.917	0.982	0.93	0.912	0.903	1.005	0.954	0.901	0.895	0.931	0.98	0.898	1.001	0.937
	0.963	0.964	0.977	0.944	0.969	0.972	0.924	0.941	0.987	0.992	0.934	0.894	0.921	0.955	0.911	0.998	0.903
	0.962	0.95	0.978	0.973	0.94	0.939	0.929	0.935	1.009	0.963	0.975	0.845	0.9	0.968	0.962	0.963	0.907
	0.946	0.95	0.964	0.947	0.948	0.923	1.003	0.951	1.014	0.933	0.943	0.842	0.915	0.977	0.895	0.95	0.891
	0.964	0.928	0.98	0.916	0.938	0.897	0.927	0.916	0.985	0.934	0.913	0.765	0.925	0.959	0.865	0.969	0.925
	0.945	0.954	0.956	0.909	0.958	0.934	0.952	0.946	1.029	0.942	0.898	0.815	0.956	0.989	0.933	0.947	0.896
	0.948	0.977	0.943	0.924	0.967	0.871	0.984	0.945	1.017	0.927	0.912	0.833	0.925	0.963	0.939	0.972	0.848
	0.946	0.938	0.93	0.945	0.993	0.849	0.945	0.952	1.017	0.908	0.913	0.912	0.85	0.933	0.925	0.976	0.956
	0.952	0.951	0.926	0.967	0.976	0.882	0.975	0.912	1.004	0.932	0.919	0.979	0.889	1.007	0.948	0.999	0.929
	0.949	0.965	0.915	0.939	0.986	0.817	0.942	0.964	1.002	0.928	0.868	0.971	0.922	1.013	0.943	0.965	0.923
	0.999	0.987	0.879	0.914	1.004	0.824	0.961	0.953	0.976	0.929	0.914	0.946	0.881	0.93	0.958	0.969	0.934
	0.976	0.974	0.83	0.874	0.976	0.792	0.953	0.941	0.957	0.931	0.908	0.939	0.878	0.918	0.872	0.982	0.903
	0.96	0.96	0.926	0.825	0.958	0.83	0.932	0.921	0.898	0.916	0.904	0.934	0.881	0.902	0.923	0.984	0.916
	1.016	0.939	0.953	0.832	0.958	0.907	0.965	0.951	0.917	0.88	0.908	0.954	0.943	0.961	0.929	1.02	0.936
	0.973	0.939	0.966	0.845	0.967	0.879	0.939	0.929	0.92	0.919	0.922	0.954	0.931	0.916	0.85	0.995	0.953
	0.946	0.962	0.957	0.912	0.979	0.814	0.956	0.929	0.844	0.935	0.915	0.956	0.862	0.904	0.855	0.982	0.926

0.951	0.971	0.963	0.915	0.967	0.805	0.946	0.929	0.811	0.951	0.918	0.944	0.84	0.855	0.874	1.005	0.934
1.01	0.893	0.928	0.855	0.937	0.847	0.895	0.857	0.756	0.935	0.876	0.941	0.903	0.857	0.85	0.909	0.908
0.942	0.936	0.938	0.856	0.926	0.814	0.844	0.856	0.825	0.932	0.864	0.927	0.902	0.852	0.815	0.929	0.899
0.929	0.944	0.954	0.814	0.93	0.864	0.891	0.858	0.908	0.89	0.817	0.958	0.949	0.936	0.761	0.863	0.913
0.933	0.939	0.919	0.833	0.937	0.943	0.918	0.829	0.929	0.948	0.834	0.968	0.942	0.97	0.708	0.872	0.876
0.935	0.883	0.878	0.807	0.968	0.972	0.911	0.752	0.967	0.896	0.893	1.019	0.882	0.931	0.848	0.891	0.932
0.924	0.95	0.876	0.783	0.954	0.934	0.862	0.792	0.929	0.908	0.935	0.99	0.841	0.926	0.853	0.907	0.94
0.948	0.879	0.956	0.776	0.967	0.91	0.917	0.86	0.972	0.933	0.923	1.017	0.875	0.984	0.927	0.885	0.919
0.947	0.873	0.957	0.774	0.984	0.967	0.901	0.886	0.967	0.934	0.921	0.983	0.877	0.936	0.898	0.918	0.874
0.921	0.879	0.95	0.745	0.963	0.961	0.876	0.891	0.95	0.922	0.894	1.02	0.834	0.901	0.921	0.9	0.891
0.959	0.944	0.998	0.777	0.931	0.943	0.895	0.876	0.966	0.831	0.924	0.937	0.801	0.898	0.871	0.931	0.875
0.934	0.908	0.933	0.865	0.923	0.946	0.958	0.82	0.985	0.861	0.87	0.922	0.844	0.95	0.884	0.887	0.869
0.94	0.899	1.011	0.94	0.98	0.945	0.919	0.81	1.003	0.869	0.93	1.004	0.894	0.942	0.983	0.899	0.828
0.969	0.875	0.948	0.918	0.898	0.908	0.893	0.83	1.008	0.822	0.921	0.916	0.834	0.985	0.923	0.959	0.86
0.929	0.887	0.913	0.885	0.913	0.882	0.883	0.803	0.998	0.771	0.927	0.884	0.826	0.923	0.933	0.909	0.924
0.951	0.896	0.883	0.909	0.895	0.817	0.838	0.902	0.999	0.889	0.901	0.932	0.878	0.957	0.964	0.944	0.891
0.936	0.918	0.892	0.921	0.889	0.832	0.917	0.839	0.991	0.925	0.891	0.927	0.895	0.943	0.939	0.984	0.91
0.955	0.86	0.938	0.963	0.87	0.888	0.946	0.815	0.997	0.918	0.915	0.959	0.827	0.942	0.972	0.955	0.882
0.954	0.869	0.949	0.918	0.878	0.93	0.911	0.752	0.97	0.838	0.972	0.924	0.898	0.948	0.955	0.911	0.897
0.972	0.896	0.887	0.888	0.89	0.819		0.814	0.998	0.844	0.921	0.923	0.93	0.946	0.927	0.917	0.881
0.954	0.891	0.881	0.872	0.935	0.868		0.867	0.986	0.816	0.847	0.988	0.905	0.911	0.97	0.946	0.917
0.939	0.82	0.871	0.908	0.942	0.92		0.879	1.028	0.8	0.793	0.992	0.866	0.891	1	0.938	0.937
0.972	0.903	0.876	0.942	0.923	0.882		0.861	0.978	0.834	0.908	0.975	0.911	0.891	0.985	0.919	0.899
0.965	0.862	0.915	0.959	0.899	0.883		0.825	0.958	0.89	0.924	0.99	0.921	0.969	0.998	0.881	0.902
0.924	0.852	0.838	0.89	0.877	0.925		0.823	0.999	0.928	0.908	1.015	0.913	0.978	0.95	0.849	0.927
0.981	0.88	0.89	0.918	0.878	0.934		0.803	1.023	0.911	0.883	0.984	0.905	0.996	0.924	0.884	0.92
0.986	0.921	0.959	0.884	0.938	0.972		0.844	1.01	0.879	0.922	0.985	0.901	0.967	0.921	0.905	0.917
0.947	0.886	0.984	0.838	0.855	0.893		0.907	1.022	0.827	0.929	0.973	0.866	0.926	0.998	0.933	0.884
0.979	0.851	0.926	0.865	0.854	0.908		0.952	0.999	0.884	0.914	0.954	0.927	0.984	0.998	0.918	0.905
0.963	0.854	0.896	0.935	0.835	0.918		0.899	1.009	0.962	0.957	0.995	0.933	1.003	0.984	0.936	0.895
0.966	0.908	0.922	0.928	0.838	0.938		0.878	0.968	0.94	0.924	1.021	0.951	0.988	0.985	0.951	0.957
0.955	0.947	0.978	0.912	0.902	0.951		0.922	0.987	0.85	0.949	0.989	0.912	1.038	0.979	0.958	0.972
0.959	0.914	0.908	0.935	0.919	0.938		0.959	1.01	0.938	0.948	0.952	0.92	1.021	0.985	0.972	0.951
0.984	0.889	0.895	0.927	0.918	0.905		0.948	1.039	0.917	0.948	0.975	0.92	1.017	0.949	0.939	0.975
0.954	0.913	0.953	0.915	0.921	0.915		0.929	1.04	0.909	0.921	0.998	0.919	0.973	0.97	0.952	0.974
0.942	0.893	0.958	0.929	0.942	0.953		0.842	1.014	0.911	0.909	0.923	0.871	0.947	0.969	0.96	0.954
0.944	0.908	0.979	0.951	0.927	0.926		0.793	0.988	0.932	0.93	0.96	0.852	0.95	0.922	0.947	0.935
0.929	0.904	0.974	0.958	0.922	0.954		0.89	0.983	0.918	0.962	0.949	0.89	0.965	0.966	0.955	0.997
0.946	0.917	0.954	0.943	0.932	0.932		0.889	0.992	0.873	0.937	0.918	0.868	0.952	0.957	0.968	0.921
0.979	0.923	0.983	0.938	0.958	0.943		0.878	1.003	0.903	0.955	0.956	0.816	0.957	0.945	0.963	0.916
0.958	0.907	0.961	0.963	0.961	0.934		0.921	0.982	0.892	0.933	0.942	0.866	0.978	0.963	0.976	0.926
0.969	0.932	0.963	0.974	0.925	0.901		0.874	0.991	0.887	0.953	0.989	0.906	0.966	0.932	0.975	0.892
0.98	0.917	0.959	0.945	0.967	0.94		0.897	0.995	0.906	0.909	0.975	0.903	0.996	0.978	0.946	0.951
0.993	0.959	0.969	0.932	0.946	0.949		0.818	1.015	0.901	0.959	0.948	0.916	1.012	0.996	1.012	0.894
0.975	0.933	0.972	0.919	0.97	0.947		0.933	0.978	0.899	0.938	0.981	0.907	1.018	0.985	1.028	0.934
0.988	0.959	0.983	0.946	0.971	0.929		0.938	0.959	0.913	0.94	0.984	0.908	1.012	1.009	0.995	0.935
1.008	0.918	0.992	0.951	0.981	0.939		0.968	0.999	0.931	0.944	0.983	0.921	1.017	1.039	1.006	0.94
0.972	0.883	0.994	0.935	0.97	0.954		0.936	0.904	0.906	0.951	0.999	0.933	0.975	1.007	0.996	0.956
0.943	0.911	0.967	0.885	0.964	0.964		0.928	0.931	0.904	0.934	1.029	0.925	0.988	1.016	0.949	0.921
0.969	0.91	0.973	0.904	0.99	0.951		0.965	0.907	0.887	0.922	0.973	0.857	0.987	0.988	0.928	0.972
0.968	0.899	0.955	0.941	1.001	0.991		1.009	0.994	0.85	0.931	0.954	0.902	0.964	0.97	0.928	0.953
0.997	0.929	0.987	0.938	0.951	0.945		0.966	0.986	0.903	0.94	0.971	0.922	0.976	0.982	0.952	0.933
0.973	0.939	0.95	0.936	0.977	0.963		0.964	1.004	0.902	0.951	0.962	0.893	1.04	1.028	0.955	1.018
0.975	0.918	0.921	0.929	0.989	0.961		0.901	0.929	0.889	0.974	0.97	0.942	0.99	0.959	0.931	0.964
0.951	0.911	0.939	0.881	0.969	0.959		0.94	0.959	0.918	0.934	0.938	0.931	0.993	0.948	0.917	0.929
0.955	0.888	0.91	0.87	0.991	0.966		0.972	0.946	0.909	0.944	0.991	0.901	0.969	0.929	0.909	0.933
0.97	0.85	0.897	0.869	0.957	0.928		0.929	0.877	0.894	0.927	0.962	0.865	0.912	0.887	0.884	0.921
0.964	0.879	0.881	0.85	0.972	0.827		0.953	0.89	0.832	0.917	0.954	0.86	0.915	0.91	0.808	0.944
0.953	0.894	0.909	0.818	0.945	0.846		0.952	0.95	0.797	0.909	0.962	0.919	0.876	0.859	0.774	0.926
0.939	0.9	0.813	0.772	0.977	0.837		0.954	1.002	0.827	0.879	0.967	0.899	0.857	0.874	0.765	0.953
0.948	0.907	0.862	0.783	0.924	0.834		0.949	0.941	0.802	0.911	0.94	0.889	0.817	0.823	0.883	0.943
0.9	0.884	0.906	0.803	0.956	0.799		0.946	0.89	0.796	0.9	0.979	0.9	0.807	0.911	0.923	0.979
0.949	0.872	0.884	0.903	0.934	0.76		0.928	0.847	0.854	0.898	0.933	0.908	0.884	0.922	0.901	0.954
0.965	0.918	0.907	0.92	0.928	0.761		0.946	0.817	0.864	0.881	0.895	0.904	0.902	0.903	0.825	0.936
0.955	0.886	0.907	0.931	0.887	0.808		0.934	0.772	0.857	0.904	0.838	0.889	0.846	0.847	0.806	0.974
0.951	0.87	0.912	0.906	0.943	0.922		0.962	0.784	0.877	0.865	0.884	0.88	0.787	0.904	0.835	0.952
0.912	0.828	0.851	0.875	0.894	0.919		0.899	0.812	0.907	0.829	0.883	0.845	0.824	0.909	0.93	0.952
0.904	0.884	0.799	0.883	0.924	0.936		0.914	0.941	0.934	0.859	0.877	0.804	0.918	0.861	0.931	0.91
0.937	0.903	0.796	0.801	0.953	0.97		0.878	0.936	0.831	0.9	0.876	0.794	0.941	0.83	0.911	0.952
0.886	0.88	0.913	0.799	0.922	0.955		0.862	0.953	0.783	0.908	0.825	0.88	0.961	0.838	0.88	0.921
0.865	0.857	0.94	0.79	0.909	0.913		0.804	0.94	0.73	0.897	0.877	0.849	0.907	0.919	0.883	0.942
0.889	0.834	0.876	0.874	0.926	0.949		0.872	0.978	0.824	0.889	0.931	0.84	0.909	0.894	0.905	0.932
0.837	0.853	0.922	0.915	0.911	0.895		0.899	0.967	0.885	0.833	0.941	0.784	0.948	0.886	0.957	0.954
0.858	0.902	0.942	0.957	0.947	0.932		0.894	0.916	0.895	0.853	0.931	0.829	0.984	0.869	0.902	0.979
0.865	0.887	0.876	0.909	0.945	0.953		0.837	0.903	0.888	0.901	0.899	0.821	0.944	0.833	0.912	0.994
0.915	0.88	0.952	0.912	0.964	0.965		0.882	0.994	0.867	0.905	0.926	0.873	0.961	0.956	0.85	0.965
0.875	0.827	0.935	0.908	0.929	0.93		0.839	0.973	0.792	0.851	0.934	0.855	0.935	0.965	0.928	0.969
0.849	0.77	0.891	0.861	0.953	0.908		0.859	0.978	0.839	0.832	0.969	0.781	1.008	0.94	0.891	0.975
0.867	0.799	0.887	0.842	0.957	0.874		0.81	0.974	0.873	0.869	0.932	0.786	0.964	0.843	0.801	0.973
0.871	0.83	0.851	0.818	0.906	0.869		0.766	0.914	0.768	0.9	0.939	0.833	0.971	0.868	0.858	0.926
0.88	0.812	0.931	0.839	0.947	0.827		0.733	0.968	0.764	0.895	0.962	0.905	0.935	0.911	0.852	0.954
0.86	0.79	0.919	0.845	0.941	0.923		0.802	0.968	0.832	0.844	0.952	0.856	0.908	0.938	0.755	0.933

	0.903	0.934	0.888	0.914	0.976	0.982	0.963	0.951	1.015	0.95	0.902	0.998	0.966	1.004	1.017	1.018	0.957
	0.971	0.982	0.84	0.907	0.981	0.975	1.01	1.008	1.006	0.97	0.939	1.031	0.987	1.025	1.043	1.003	0.971
	0.996	0.931	0.869	0.964	1	0.993	0.977	0.983	1.026	0.926	0.949	0.999	0.983	1.007	1.042	0.986	0.987
	0.988	0.967	0.946	1.008	1.001	0.997	0.968	1.004	1.053	0.95	0.963	1.06	0.995	1.045	1.012	1.014	1.002
	1.03	1.001	1.002	0.993	1.03	1.002	0.98	0.994	0.994	0.94	0.976	1.04	0.981	1.016	1.038	1.028	1.017
	1.031	1.012	0.976	1.003	0.989	1.005	0.967	0.973	1.084	0.936	0.969	1.028	0.974	1.066	1.061	1.022	1.012
	1.01	0.982	0.977	1.047	1.01	1.018	0.971	0.987	1.013	0.973	0.984	1.05	1.019	1.011	0.985	1.038	1.025
	1.029	1	1.006	1	1.027	0.986	0.986	0.966	1.045	0.936	1.016	1.031	0.977	0.986	1.027	1.038	0.959
	1.025	0.99	1.04	1.016	1.005	0.985	0.956	0.959	1.005	0.989	1.014	1.04	0.988	1.015	1	1.052	1.022
	1.057	1.006	1.019	0.983	1.008	1.03	0.993	0.951	1.011	0.992	1.009	1.041	1.005	0.999	1.003	1.037	1.007
	1.032	0.988	1.02	0.974	0.999	1.028	0.908	0.952	1.013	1.035	1.028	1.054	0.982	1.003	1.043	1.052	1.028
10kcal%	1.003	0.999	1.017	0.971	1	1.012	0.946	0.971	1.015	1.032	0.987	1.037	1	1.049	0.997	1.074	0.996
	1.018	1.039	1.024	1.004	1.012	0.991	0.988	0.972	1	0.983	0.994	1.081	0.971	0.993	0.971	1.017	1.007
	1.03	1.001	1.03	0.979	0.999	0.981	0.974	0.984	1.022	0.986	1.022	1.026	0.979	1.029	1.006	1.02	1.026
	1.004	0.98	1.007	0.988	0.979	0.983	0.976	0.945	1.018	0.961	0.993	1.021	0.967	0.998	0.994	1.003	1.023
	1.009	0.996	1.021	1.008	0.998	1.035	1.026	0.951	1.002	0.968	1.004	1.025	1	0.994	0.99	1.031	0.977
	0.989	1.015	1.015	0.984	1.013	1.018	1.018	0.976	1.026	0.962	0.974	1.025	1.021	1.044	1.026	1.017	0.998
	1.03	1.027	1.014	0.986	0.986	1.012	0.961	0.998	1.029	0.986	1.047	1.043	0.977	1.01	1.034	0.989	1.014
	1.022	0.993	1.001	1.004	0.999	1.009	1.016	0.999	1.02	0.97	1.016	1.032	0.989	1.038	1.015	1.022	1
	1.045	1.005	1.022	1.018	1.013	1.043	0.995	1.008	1.043	0.969	1.032	1.044	0.973	1.044	1.064	1.025	0.998
	1.037	1.013	1.034	1.006	1.002	1.029	0.971	0.996	1.093	0.94	0.997	1.067	0.995	1.026	1.025	1.024	0.998
	1.008	0.993	1.002	1.002	1.011	0.999	0.98	0.971	1.081	1.015	0.977	1.042	0.976	1.004	1.056	1.071	0.98

1.029	1.042	1.032	1.012	1.029	1.033	0.977	0.973	1.042	0.985	1.02	1.058	1.011	1.019	1.001	1.048	0.989
1.067	1.027	1.027	0.99	1.04	1.04	0.968	0.961	1.028	0.986	1.012	1.068	1.006	1.025	1.021	1.044	1.006
1.096	1.055	1.072	1.022	1.027	1.017	0.996	0.942	1.016	0.951	1.04	1.093	1.02	1.033	0.996	1.03	1.016
1.07	1.031	1.017	1.032	1.051	1.012	0.982	0.913	1.022	0.943	1.022	1.079	1.035	1.036	1.032	1.02	1.034
1.055	1.013	1.046	1.011	1.041	1	0.958	0.887	1.035	0.935	1.021	1.076	1.044	1.007	1.04	1.019	0.983
1.037	1.012	1.023	1.025	1.014	1.016	0.997	0.845	1.032	0.958	1.014	1.08	1.014	1.016	0.981	1.018	1.057
1.058	1.014	1.024	1.022	1.063	1.037	0.99	0.918	1.008	0.975	1.028	1.07	1.044	0.996	1.02	1.039	1.015
1.036	1.042	1.036	1.031	1.09	1.058	0.997	0.915	1.021	0.94	1.047	1.089	1.056	0.949	1.028	1.048	1.042
1.036	1.003	0.991	1.015	1.032	1.004	0.967	0.9	0.97	0.86	1.027	1.03	0.994	0.98	1.032	1.051	1.031
1.057	1.004	0.965	1.029	1.033	1.03	0.96	0.908	1.011	0.809	1.054	1.035	1.054	0.986	1.075	1.022	0.993
1.077	0.996	0.935	0.986	1.02	1.06	0.973	0.879	1.048	0.814	1.045	1.041	1.003	0.977	1.102	0.992	1.019
1.053	1.004	0.892	1.017	1.049	1.034	0.961	0.833	1.052	0.889	1.043	1.05	0.999	1.017	1.065	1.031	1.043
1.07	0.992	0.858	1.02	1.025	1.037	0.994	0.776	1.041	0.937	1.067	1.016	0.993	0.972	1.058	0.985	1.058
1.066	1.003	0.945	1.05	1.036	1.027	0.987	0.777	1.039	0.905	1.024	1.018	1.062	0.974	1.047	1.011	0.967
1.033	0.94	0.954	1.053	1.085	1.022	1	0.743	1.008	0.879	1.025	0.995	1.008	0.945	1.03	1.008	0.969
1.047	0.872	0.913	1.048	1.045	1.017	0.982	0.802	1.038	0.847	1.029	0.997	1.006	0.909	1.079	1.012	0.983
1.023	0.95	0.896	1.044	1.048	0.951	0.997	0.84	1	0.76	1.05	0.935	1.015	0.854	1.033	0.971	1.016
1.014	0.902	0.904	1.052	1.084	0.993	0.975	0.825	1.015	0.861	1.007	0.944	0.999	0.807	1.097	0.935	1.042
0.994	0.874	0.849	1.074	1.068	1.028	0.931	0.837	0.953	0.956	1.049	0.907	1.005	0.762	1.015	1.022	0.991
0.954	0.865	0.847	1.081	1.024	1.061	0.987	0.807	0.879	0.925	1.037	0.878	1.038	0.708	1.051	1.032	1.002
0.87	0.808	0.894	1.054	1.033	1.022	1.001	0.79	0.841	0.892	1.044	0.889	0.994	0.734	1.026	1.01	0.996
0.847	0.811	0.848	1.038	1.007	1.039	0.993	0.934	0.883	0.943	1.043	0.89	1.019	0.875	1.008	0.998	1.015
0.854	0.797	0.809	0.998	1.009	1.009	0.965	0.918	0.857	0.951	0.999	0.906	1.067	0.815	1.03	0.921	0.929
0.961	0.881	0.822	0.971	1.013	1.016	0.941	0.803	0.976	0.853	1.024	0.901	1.075	0.823	1.079	0.903	0.972
0.882	0.869	0.796	0.967	0.988	0.975	0.954	0.918	1.06	0.918	1.033	0.957	1.065	0.826	1.017	1.019	0.979
0.966	0.842	0.789	0.938	1.01	1.02	0.919	0.929	1.096	0.899	1.034	0.895	1.008	0.836	0.873	1.004	1
0.952	0.793	0.823	0.992	1.015	1.033	0.98	1.017	1.018	0.762	1.038	0.881	1.007	0.927	0.985	1.027	1.051
0.977	0.968	0.962	1.017	1.029	1.045	0.993	1.022	1.044	0.689	1.015	0.9	1.043	0.94	0.978	0.963	1.066
1.057	0.998	0.931	1.078	0.98	1.029	1.022	0.983	1.057	0.784	1.032	1.02	1.025	1.004	0.899	0.951	0.975
1.022	0.94	0.994	1.03	0.974	0.993	0.977	0.94	0.988	0.946	0.962	0.991	0.995	0.991	0.753	0.983	1.043
0.978	1.031	0.948	0.996	0.995	0.98	1.066	0.986	1.028	0.926	0.911	1.049	1.01	0.991	0.791	1.027	1.05
1.026	1.038	1.003	0.983	0.992	0.965	1.026	0.974	1.046	0.834	1.028	1.017	1.015	1.01	0.863	0.96	1.027
0.896	1.051	0.971	1.059	1.02	1.011	1.036	0.905	1.099	0.788	1.026	1.023	1.035	1.053	0.896	0.924	0.976
0.903	0.974	0.901	1.023	1.017	1.097	1.001	0.929	1.065	0.847	0.927	1.085	1.023	1.087	0.791	0.931	0.981
0.905	0.924	0.957	0.931	1.037	1.067	1.002	0.981	1.079	0.899	0.882	1.15	1.059	1.114	0.783	0.941	1.014
0.806	0.955	1.002	1.005	0.995	1.001	1.03	0.934	1.043	0.841	0.831	1.13	1.045	1.086	0.767	0.913	1.031
0.954	1.039	0.95	1.024	0.994	1.037	1.052	0.882	1.063	0.815	0.944	1.076	1.048	1.072	0.741	0.836	1.007
0.94	1	0.908	0.988	1.053	1.074	0.979	0.913	1.061	0.857	0.994	1.137	1.07	1.124	0.736	0.907	0.994
0.913	0.977	0.94	1.056	1.011	1.02	0.97	0.854	1.069	0.917	0.965	1.065	1.078	1.037	0.914	0.903	1.019
0.944	0.976	1.027	0.965	1.014	1.068	0.978	0.813	1.104	0.805	0.909	1.081	1.044	1.069	0.976	0.817	0.957
0.87	0.982	1.028	0.89	1.01	1.076	1.002	0.831	1.128	0.793	0.847	1.114	1.015	1.019	0.905	0.82	0.938
0.832	0.944	0.982	0.899	1.048	1.041	0.9	0.926	1.086	0.921	0.821	1.131	1.02	1.037	0.871	0.765	0.991
0.907	0.986	0.929	0.991	1.054	1.056	0.867	0.931	1.105	0.949	0.958	1.098	1.09	1.036	0.97	0.752	0.973
0.915	1.01	1.017	0.994	1.046	1.062	0.902	0.878	1.089	0.87	0.982	1.042	1.035	0.987	0.949	0.838	0.979
0.976	0.996	1.092	0.927	0.99	1.039	0.891	0.925	1.064	0.761	0.904	1.081	1.008	0.991	0.871	0.893	0.988
0.956	0.988	1.067	0.852	1.01	1.042	0.832	0.989	1.025	0.776	0.879	1.13	1.04	1.008	0.932	0.79	0.952
1.004	1.048	1.017	0.83	1.014	0.995	0.85	1.059	1.047	0.864	0.97	1.119	1.066	1.004	1.01	0.756	0.951
1.034	1.067	1.048	0.875	1.04	0.997	0.876	0.944	1.053	1.011	1.071	1.107	1.066	0.911	1.012	0.878	0.915
1.038	1.034	1.058	0.962	1.067	1.01	0.816	0.956	1.035	0.998	1.056	1.064	1.04	0.83	0.982	0.96	0.933
1.049	1.07	1.019	1.018	1.07	1.049	0.846	0.972	0.857	0.863	1.096	1.078	1.047	0.769	0.837	0.935	1.001
1.091	1.048	1.054	1.088	1.083	1.058	0.927	1.003	0.987	0.832	1.075	1.072	1.072	0.798	0.762	0.839	1.011
1.089	1.036	1.035	1.083	1.088	1.052	0.941	1.033	1.017	0.941	1.057	1.064	1.094	0.92	0.927	0.784	1.044
1.068	1.078	1.041	1.086	1.07	1.045	0.916	1.031	1.01	1	1.083	1.08	1.109	0.991	1.037	0.757	1
1.024	1.048	1.102	1.078	1.035	1.042	1.037	1.028	0.966	1	1.092	1.092	1.052	0.998	1.086	0.923	1.024
1.031	1.051	1.078	1.08	1.033	1.023	1.012	0.996	0.943	1.031	1.108	1.081	1.036	0.937	1.058	0.908	1.031
1.064	1.074	1.047	1.065	1.005	1.039	1.001	1.032	0.919	1.061	1.066	1.09	1.014	0.99	1.036	0.983	1.069
1.087	1.047	1.045	1.054	1.004	1.041	0.959	1.033	0.837	0.998	1.045	1.043	1.036	1.021	0.981	0.992	0.998
1.042	1.036	1.061	1.039	0.952	1.02	0.958	1.02	0.746	0.975	1.02	1.055	1.017	1	0.975	0.979	1.032
1.03	1.012	1.034	1.013	0.958	1.011	0.946	0.988	0.719	0.933	1.018	1.052	1.047	0.947	0.882	1.01	1.042
1.033	1.005	1.03	1.055	0.947	1.004	0.974	0.998	0.707	0.897	0.997	1.056	1.034	0.923	0.978	0.979	1.001
1.017	1.027	1.018	1.07	0.937	0.993	0.973	1.078	0.86	0.809	0.98	1.099	0.999	0.825	0.975	0.993	0.967
1.013	1.042	1.057	1.067	0.874	0.937	1.001	1.01	0.934	0.763	0.966	1.054	1.012	0.783	0.952	1.006	0.859
1.016	1.03	1.026	1.062	0.868	0.919	1.025	1.011	0.958	0.726	0.947	1.052	1.004	0.666	0.912	0.841	0.843
1.011	1.015	1.035	1.059	0.868	0.88	1.03	0.956	0.926	0.877	0.983	1.047	0.929	0.694	0.878	0.944	0.905
1.01	1.078	0.964	1.033	0.852	0.959	1.001	0.881	0.786	0.918	0.958	1.069	1.002	0.949	0.904	0.926	0.963
0.965	1.045	1.022	1.048	0.92	0.987	1.021	0.881	0.768	0.921	0.896	1.059	1.001	1.01	0.982	0.999	0.983
0.99	1.055	1.015	1.063	0.87	1.006	1.002	0.914	0.84	0.885	0.983	1.047	1.033	1.031	1.005	0.945	1.057
0.946	1.054	0.929	1.018	0.84	0.967	0.961	0.86	0.792	0.836	1.027	1.075	1.042	1.024	0.998	0.906	1.053
0.897	1.019	0.935	1.053	0.868	0.916	0.907	0.8	0.739	0.837	1.046	1.027	0.981	1.016	0.93	0.934	0.987
0.877	1.091	1.003	1.046	0.954	0.937	0.779	0.743	0.703	0.872	1.106	1.042	0.945	1.03	0.907	0.865	1.007
0.861	1.039	0.937	1.047	0.995	0.883	0.669	0.758	0.649	0.79	1.054	1.055	0.932	0.959	1.01	0.779	0.991
0.843	1.051	0.943	1.019	0.939	0.831	0.939	0.738	0.667	0.733	1.055	1.034	0.989	0.973	0.929	0.757	0.986
0.828	1.058	0.974	1.053	0.918	0.87	0.987	0.85	0.774	0.836	1.119	1.043	1.021	1.037	0.874	0.915	0.957
0.794	1.059	0.886	1.016	0.939	0.826	1.022	0.857	0.953	0.862	1.099	1.074	1.023	.	0.977	0.886	0.944
0.789	1.047	0.867	0.98	0.961	0.81	0.979	0.798	1.034	0.878	1.087	1.055	1.016	.	0.976	0.794	0.957
0.781	1	0.98	0.932	0.934	0.947	0.974	0.758	1.078	0.9	1.085	1.053	0.995	.	0.943	0.969	1.002
0.842	1.009	0.89	1.019	0.872	1.029	0.972	0.749	1.033	0.972	1.041	1.023	1.018	.	0.832	0.931	0.959
0.847	1.025	0.961	1.07	0.902	0.951	0.981	0.968	1.025	0.926	1.048	1.036	0.968	.	0.789	0.831	0.927
0.781	0.994	0.877	1.037	**0.867**	**0.922**	0.942	0.989	0.976	0.859	1.005	**0.992**	**0.933**	.	0.696	0.851	0.952
0.814	0.99	0.84	0.997	**0.821**	**0.917**	0.88	1.036	0.978	0.956	0.976	**0.987**	**0.955**	.	0.644	0.8	0.862
0.772	0.934	0.818	1.047	**0.874**	**0.846**	0.81	1.026	0.955	0.985	0.949	**0.966**	**0.914**	.	0.676	0.761	0.797
0.768	0.889	0.839	1.031	**0.911**	**0.868**	0.801	0.986	0.917	0.943	0.893	**1.033**	**1.03**	.	0.706	0.746	0.764
0.874	0.878	0.856	1.047	**0.808**	**0.815**	0.788	0.984	0.895	0.962	0.944	**0.913**	**1.037**	.	0.783	0.788	0.819
0.845	0.881	0.96	0.995	**0.836**	**0.817**	0.808	0.933	0.843	0.979	0.925	**0.889**	**1.045**	.	0.89	0.89	0.985
0.789	0.863	0.967	0.967	**0.821**	**0.831**	0.786	1.023	0.796	0.916	0.918	**0.899**	**1.003**	.	0.942	0.805	1.06
0.785	0.96	0.941	0.901	**0.75**	**0.95**	0.742	1.021	0.81	0.926	0.904	**0.854**	**0.935**	.	0.794	0.735	1.053
0.847	0.963	0.891	1	0.8	0.965	0.713	1.038	0.748	0.91	0.911	0.852	0.935	.	0.833	0.694	1.05
0.821	0.92	0.949	1.057	0.887	0.92	0.706	0.941	0.748	0.923	0.981	0.972	1.037	.	0.791	0.712	0.997
0.808	0.871	0.888	1.037	0.894	0.947	0.684	0.842	0.813	0.859	0.946	0.973	1.052	.	0.815	0.826	1.005
0.859	1.001	0.884	1.065	0.97	0.938	0.674	0.855	0.993	0.785	0.965	1.006	1.021	.	0.944	0.958	1.009
0.877	1.018	0.844	1.063	0.98	0.909	0.757	0.832	1.058	0.704	0.931	0.919	0.952	.	1.051	0.878	1.023
0.812	0.942	0.846	1.047	0.948	0.871	0.793	0.759	1.063	0.715	0.886	0.93	0.979	.	1.09	0.866	0.947
0.8	1.004	0.82	0.991	0.837	0.825	0.868	0.723	0.945	0.765	0.885	1.008	0.928	.	0.945	0.849	0.937
0.907	0.998	0.942	0.957	0.927	0.98	0.807	0.881	0.967	0.918	1.02	1.063	1.009	.	0.925	1.022	1.02
0.998	0.926	0.854	0.908	0.917	0.993	0.722	1.044	0.931	0.889	1.008	1.069	1.056	.	0.965	1.045	1.033
1.026	0.955	0.815	0.918	0.835	0.934	0.753	1.1	0.917	0.858	0.959	1.006	1.061	.	0.921	1.007	1.086
0.945	0.964	0.804	1.01	0.901	0.865	0.855	1.002	0.846	0.822	0.992	1.059	0.994	.	0.808	0.955	1.04
1.059	0.893	0.795	0.967	0.883	0.842	0.86	0.92	0.754	0.94	0.911	0.977	0.894	.	0.855	0.988	1.048
0.983	0.976	0.802	0.939	0.809	0.845	0.776	0.84	0.761	0.973	0.887	0.952	1.011	.	0.932	0.953	1.076
1.016	1.077	0.816	0.936	0.737	0.823	0.747	0.913	0.777	0.889	0.972	0.943	1.022	.	0.923	0.855	1.02
0.932	1.011	0.754	1	0.772	0.798	0.798	0.82	0.814	0.877	0.932	0.991	1.062	.	0.839	0.916	1.021
1.056	1.052	0.789	1.046	0.888	0.946	0.885	0.792	0.969	0.86	0.899	1.022	1.086	.	0.751	0.983	1.007
0.989	0.938	0.864	0.975	0.856	1.04	0.829	0.831	1.03	0.943	0.888	0.988	1.01	.	0.718	1	0.928
0.964	0.982	0.994	0.957	0.912	1.069	0.82	0.972	1.138	0.981	0.868	1.031	1.033	.	0.815	0.956	0.952
0.926	0.901	0.993	0.97	0.951	1.075	0.787	1.048	1.088	1.046	0.9	1.025	1.021	.	0.719	0.985	0.901

	0.933	0.94	0.929	0.985	0.852	1.025	0.725	1.054	1.153	0.975	0.958	0.947	1.018	.	0.747	0.943	0.831
	0.986	0.904	0.913	0.919	0.812	0.997	0.704	1.013	1.096	0.967	0.884	1.064	1.035	.	0.705	0.996	0.8
	1.025	0.889	0.861	0.905	0.885	0.97	0.695	0.983	1.053	0.909	0.914	0.995	0.993	.	0.891	1.018	0.931
	0.971	0.824	0.999	0.926	0.812	0.977		0.892	1.013	0.908	0.872	0.901	0.957	.	0.979	0.933	1.015
	0.953	0.808	1.049	0.886	0.767	1.009		0.889	1.048	0.987	1.016	1.001	1.022	.	1.027	0.843	0.974
	0.93	0.796	1.047	0.909	0.868	1.108		0.976	1.057	1.036	0.947	1.012	1.04	.	1.022	0.89	0.97
	1.001	0.989	0.998	1.03	0.936	1.1		0.965	1.025	1.074	0.988	0.897	1.064	.	1.086	0.94	1.012
	1.053	1.064	0.925	0.933	0.94	1.066		0.867	1.001	0.993	1.063	0.817	1.071	.	1.038	0.835	0.963
	1.004	1.067	0.867	0.864	0.93	1.056		0.796	0.935	0.969	1.095	0.766	1.078	.	1.002	0.798	1.003
	1.032	1.013	0.91	0.96	0.996	1.037		0.805	0.903	0.971	1.105	0.935	1.006	.	0.964	0.768	1.018
	1.078	1.063	1.022	0.987	1.013	1.05		0.899	0.825	0.94	1.06	1.023	1.011	.	0.963	0.718	1.006
	1.057	1.097	1.044	0.929	1.003	1.046		1.034	0.814	0.916	1.06	0.979	0.991	.	1.05	0.836	1.007
	1.043	1.053	1.056	0.865	1.001	1.111		1.051	0.761	0.922	1.053	1.083	0.962	.	1.019	0.902	0.995
	1.072	1.066	1.038	0.907	0.989	1.013		1.004	0.817	0.942	1.086	1.048	0.97	.	1.046	0.837	1.04
	1.054	1.12	1.072	1.013	0.991	1.046		1.038	0.987	0.87	1.07	1.035	0.969	.	1.015	0.826	1.014
	1.05	1.084	1.095	1.044	0.919	1.042		0.989	1.04	0.972	1.098	1.116	1.021	1.008	1.017	0.957	1.009
	1.014	1.087	1.045	1.048	0.912	0.97		0.958	1.081	1.017	1.055	1.107	0.997	0.997	1.017	1	1.048
	0.973	1.069	1.064	1.03	0.961	0.96		0.972	1.052	0.964	1.015	1.093	0.924	1.01	0.972	0.987	0.998
	0.976	1.029	1.038	1.04	0.919	0.91		0.928	1.004	0.931	1.005	1.098	0.859	1.009	0.939	0.93	0.928
	1.022	1.005	1.028	1.022	0.893	0.97		0.925	0.987	0.887	1.006	1.058	0.945	1.008	0.867	0.895	0.857
	1.042	1.014	0.979	0.963	0.939	0.984		0.873	0.981	0.812	0.953	1.073	0.969	1.018	0.964	0.814	0.779
	1.059	0.98	1.018	0.891	0.914	0.975		0.839	1.009	0.888	1.009	1.077	0.878	0.979	0.952	0.947	0.742
	1.049	0.899	1.025	0.969	0.944	0.95		0.809	1.009	0.921	0.998	1.095	0.846	0.957	0.891	1.022	0.792
	1.012	0.932	1.02	1.022	0.956	0.876		0.766	0.982	0.894	0.97	1.069	0.893	0.944	0.782	1.01	0.814
	1.011	0.961	1.028	1.056	0.955	0.987		0.912	0.929	0.878	0.922	1.086	0.924	0.972	0.785	0.968	0.774
	0.956	0.89	0.962	1.02	0.919	1.023		0.964	0.951	0.859	0.885	1.062	0.956	1.019	0.763	0.938	0.739
	0.96	0.924	1.033	1.014	0.85	1.05		0.939	0.858	0.853	0.869	1.097	0.929	1.096	0.795	1.011	0.796
	0.916	0.94	1.019	0.997	0.948	1.004		0.928	0.826	0.839	0.837	1.089	0.87	1.054	0.788	1.01	0.917
	0.998	1.012	0.978	0.953	0.958	1.043		0.816	0.781	0.751	0.808	1.034	0.874	1.057	0.722	0.997	0.962
	1.024	0.917	0.897	1.011	0.879	1.055		0.787	0.727	0.803	0.792	1.078	0.831	1.072	0.715	0.999	0.909
	1.057	0.859	1.016	1.049	0.862	1.018		0.799	0.742	0.939	0.863	1.057	0.805	1.058	0.908	0.957	0.936
	1.054	0.812	1.049	1.062	0.816	0.973		0.762	0.729	0.826	0.837	1.061	0.901	1.027	0.923	0.928	0.863
	1.061	0.914	1.045	1.024	0.781	0.888		0.729	0.693	0.745	0.811	1.032	1.006	1.09	0.902	0.888	0.778
	1.022	0.851	1.026	1.002	0.773	0.868		0.904	0.651	0.855	0.768	1.008	1.041	1.051	0.755	0.826	0.781
	1.006	0.816	0.99	0.996	0.74	0.833		1.002	0.658	0.825	0.85	1.013	1.039	1.108	0.696	0.819	0.763
	1.017	0.891	0.981	0.963	0.724	0.8		1.004	0.655	0.811	0.985	0.99	0.968	1.115	0.677	0.98	0.715
	1.025	0.924	1.036	0.968	0.718	0.898		1.002	0.693	0.851	0.984	0.942	0.928	1.069	0.707	0.891	0.727
	1.069	0.87	1.003	1.043	0.71	0.902		0.913	0.639	0.936	1.028	0.93	0.948	1.053	0.81	0.889	0.712
	1.06	0.824	1.008	1.046	0.754	0.86		0.891	0.713	0.969	0.993	0.934	0.936	1.024	0.789	0.981	0.745
	1.023	0.754	0.979	1.037	0.816	0.794		0.803	0.704	0.989	0.993	0.864	0.863	1.028	0.776	1.066	0.742
	1.032	0.764	0.982	1.027	0.782	0.768		0.874	0.702	1.014	1.014	0.851	0.836	1.019	0.722	1.105	0.683
	1.017	0.845	0.914	1.061	0.752	0.755		0.878	0.668	1.017	1.03	0.977	0.939	1.003	0.778	1.052	0.71
	0.964	0.903	0.901	1.023	0.748	0.876		0.82	0.641	1.013	1.007	0.899	1	1.073	0.744	1.059	0.818
	0.986	0.886	0.919	1.058	0.739	0.863		0.941	0.659	1.021	0.974	0.856	0.939	1.027	0.669	0.982	0.93
	0.913	0.83	0.888	1.076	0.709	0.804		1.046	0.685	0.938	0.951	0.828	0.917	1.006	0.682	0.974	0.946
	1.018	1.013	0.984	1.065	0.728	0.795		1.07	0.698	0.933	0.922	0.828	0.9	0.934	0.736	0.956	0.878
	1.013	1.053	0.936	1.039	0.752	0.782		1.061	0.71	0.969	0.861	0.801	0.84	0.906	0.845	0.901	0.843
	0.952	0.987	0.899	0.944	0.783	0.8		1.039	0.704	0.95	0.894	0.779	0.957	0.89	0.965	0.848	0.826
	0.959	0.918	0.856	0.954	0.742	0.751		1.007	0.737	0.905	0.885	0.793	1.009	0.838	0.905	0.786	0.779
	0.902	0.957	0.845	1.007	0.748	0.736		0.929	0.68	0.887	0.884	0.751	0.899	0.845	0.78	0.855	0.783
	0.865	0.884	0.822	0.949	0.743	0.762		0.989	0.692	0.81	0.89	0.915	0.879	0.862	0.699	0.787	0.724
	0.814	0.767	0.742	0.868	0.733	0.728		0.976	0.685	0.719	0.831	0.91	0.875	0.876	0.676	0.723	0.703
	0.895	0.821	0.888	0.903	0.69	0.776		0.957	0.653	0.698	0.821	0.859	0.935	0.809	0.719	0.818	0.783
	1.005	0.909	0.918	0.848	0.728	0.949		0.88	0.662	0.855	0.783	0.817	0.984	0.71	0.75	0.996	0.832
	0.992	1	0.815	0.842	0.882	1.009		0.84	0.726	0.966	0.883	0.798	0.92	0.746	0.723	1.046	0.822
	1.092	0.969	0.984	0.953	0.863	0.932		0.875	0.868	1.029	0.998	0.945	0.781	0.78	0.711	1.013	0.782
	1.066	1.088	0.963	1.054	0.859	0.975		1.034	0.979	0.988	1.014	1.105	0.751	0.792	0.758	0.998	0.705
	1.026	1.093	0.91	0.994	0.876	0.941		1.071	1.151	0.961	0.894	1.128	0.938	0.738	0.927	1.008	0.734
	0.95	1.049	0.848	0.93	0.837	0.957		0.992	1.089	0.976	0.946	1.051	0.877	0.683	0.888	1	0.775
	0.987	0.945	0.807	0.894	0.749	0.998		0.956	1.138	0.997	0.886	1.062	0.83	0.689	0.966	0.954	0.765
	0.93	0.978	0.82	0.853	0.863	1.027		0.912	1.081	1.013	0.825	1.003	0.812	0.708	0.886	0.932	0.715
	1.008	0.918	0.887	0.873	0.926	1.018		0.922	1.08	1.039	0.961	0.936	0.868	0.689	0.858	0.941	0.804
	1.059	0.986	0.865	0.868	0.959	0.939		0.887	1.087	1.034	1.012	0.899	0.992	0.703	0.787	0.957	0.943
	0.772	0.789	0.852	0.847	0.839	0.862	.	0.989	0.977	0.93	0.853	0.797	0.805	0.761	1.013	0.713	0.944
	0.796	0.815	0.896	0.852	0.809	0.807	.	0.996	0.978	0.921	0.873	0.795	0.858	0.904	0.939	0.784	0.871
	0.816	0.769	0.838	0.826	0.816	0.867	.	0.927	0.955	0.922	0.828	0.79	0.865	0.922	0.854	0.817	0.871
	0.843	0.774	0.809	0.821	0.831	0.816	.	0.916	0.958	0.928	0.739	0.829	0.858	0.876	0.932	0.873	0.847
	0.791	0.778	0.758	0.773	0.863	0.867	.	0.917	0.917	0.881	0.764	0.802	0.865	0.839	0.865	0.797	0.812
	0.75	0.851	0.787	0.841	0.818	0.828	.	0.95	0.942	0.86	0.772	0.828	0.845	0.838	0.905	0.761	0.863
	0.805	0.803	0.737	0.847	0.802	0.805	.	0.894	0.942	0.858	0.812	0.812	0.798	0.814	0.856	0.748	0.839
	0.824	0.886	0.786	0.831	0.832	0.84	.	0.934	0.923	0.875	0.807	0.776	0.851	0.848	0.861	0.772	0.844
	0.812	0.84	0.832	0.825	0.809	0.868	.	0.902	0.863	0.822	0.796	0.779	0.806	0.831	0.839	0.793	0.854
	0.792	0.811	0.771	0.823	0.795	0.828	.	0.895	0.869	0.846	0.791	0.794	0.794	0.836	0.817	0.793	0.824
	0.794	0.829	0.831	0.808	0.815	0.794	.	0.92	0.835	0.823	0.844	0.78	0.818	0.819	0.842	0.822	0.84
60kcal%	0.818	0.835	0.825	0.814	0.755	0.783	.	0.854	0.847	0.837	0.823	0.804	0.861	0.831	0.811	0.833	0.81
	0.805	0.794	0.84	0.838	0.821	0.801	.	0.835	0.866	0.784	0.831	0.794	0.823	0.829	0.807	0.811	0.774
	0.839	0.773	0.776	0.818	0.801	0.8	.	0.866	0.842	0.766	0.808	0.772	0.783	0.827	0.82	0.77	0.82
	0.77	0.759	0.78	0.788	0.784	0.799	.	0.824	0.815	0.773	0.752	0.777	0.795	0.785	0.776	0.778	0.805
	0.776	0.755	0.807	0.779	0.76	0.8	.	0.841	0.818	0.77	0.786	0.761	0.77	0.817	0.814	0.769	0.78
	0.764	0.756	0.78	0.797	0.761	0.757	.	0.847	0.8	0.779	0.791	0.77	0.764	0.817	0.818	0.749	0.79
	0.778	0.748	0.789	0.841	0.743	0.768	.	0.833	0.831	0.794	0.783	0.756	0.738	0.779	0.779	0.783	0.768
	0.822	0.752	0.831	0.815	0.718	0.793	.	0.84	0.794	0.792	0.785	0.757	0.742	0.794	0.777	0.795	0.771
	0.778	0.76	0.791	0.814	0.752	0.781	.	0.827	0.808	0.783	0.77	0.775	0.741	0.795	0.779	0.754	0.781
	0.802	0.762	0.801	0.79	0.768	0.756	.	0.832	0.841	0.754	0.818	0.761	0.752	0.838	0.801	0.754	0.74
	0.83	0.738	0.806	0.82	0.756	0.745	.	0.825	0.8	0.752	0.812	0.782	0.74	0.827	0.785	0.775	0.775
	0.823	0.784	0.763	0.776	0.794	0.743	.	0.847	0.861	0.754	0.813	0.786	0.729	0.8	0.774	0.798	0.783
	0.82	0.775	0.75	0.814	0.772	0.786	.	0.86	0.805	0.667	0.818	0.745	0.718	0.806	0.783	0.791	0.786
	0.827	0.808	0.769	0.804	0.777	0.791	.	0.855	0.836	0.774	0.788	0.761	0.73	0.814	0.779	0.783	0.816
	0.804	0.78	0.787	0.787	0.774	0.827	.	0.816	0.815	0.771	0.789	0.754	0.736	0.844	0.756	0.775	0.757
	0.779	0.774	0.766	0.803	0.757	0.77	.	0.804	0.825	0.802	0.779	0.721	0.753	0.784	0.766	0.836	0.769
	0.756	0.812	0.827	0.808	0.763	0.747	.	0.834	0.825	0.818	0.786	0.788	0.749	0.776	0.74	0.817	0.777
	0.765	0.774	0.784	0.771	0.774	0.794	.	0.805	0.829	0.784	0.766	0.798	0.763	0.763	0.747	0.803	0.775
	0.795	0.766	0.779	0.774	0.757	0.749	.	0.813	0.783	0.777	0.764	0.79	0.758	0.811	0.742	0.762	0.773
	0.792	0.748	0.738	0.773	0.715	0.745	.	0.809	0.826	0.738	0.765	0.754	0.741	0.822	0.754	0.761	0.777
	0.794	0.798	0.772	0.81	0.741	0.768	.	0.767	0.789	0.726	0.763	0.791	0.786	0.781	0.738	0.793	0.797
	0.773	0.797	0.789	0.759	0.758	0.757	.	0.784	0.817	0.746	0.734	0.814	0.768	0.775	0.728	0.781	0.776
	0.769	0.782	0.798	0.766	0.742	0.767	.	0.777	0.835	0.711	0.751	0.75	0.757	0.779	0.719	0.785	0.785
	0.762	0.734	0.807	0.751	0.764	0.782	.	0.783	0.813	0.737	0.768	0.775	0.75	0.794	0.729	0.826	0.773
	0.743	0.747	0.801	0.746	0.797	0.785	.	0.839	0.866	0.768	0.76	0.812	0.75	0.835	0.813	0.751	0.77
	0.745	0.765	0.781	0.735	0.748	0.753	.	0.841	0.847	0.834	0.767	0.789	0.727	0.813	0.771	0.774	0.817
	0.774	0.787	0.73	0.751	0.757	0.741	.	0.826	0.861	0.822	0.774	0.766	0.748	0.812	0.786	0.79	0.786
	0.77	0.788	0.785	0.737	0.774	0.713	.	0.814	0.857	0.784	0.785	0.761	0.744	0.805	0.748	0.762	0.804
	0.767	0.79	0.762	0.761	0.784	0.735	.	0.806	0.862	0.779	0.772	0.774	0.777	0.801	0.767	0.798	0.783
	0.752	0.783	0.779	0.778	0.767	0.735	.	0.844	0.846	0.86	0.777	0.742	0.726	0.824	0.732	0.853	0.768

0.762	0.781	0.805	0.797	0.782	0.741 .	0.844	0.816	0.832	0.767	0.771	0.713	0.781	0.728	0.841	0.764
0.781	0.779	0.789	0.787	0.779	0.729 .	0.833	0.873	0.771	0.77	0.778	0.733	0.792	0.72	0.8	0.753
0.751	0.75	0.756	0.78	0.746	0.775 .	0.809	0.861	0.764	0.732	0.749	0.758	0.778	0.781	0.785	0.755
0.759	0.754	0.816	0.775	0.751	0.781 .	0.764	0.788	0.768	0.734	0.769	0.757	0.756	0.782	0.759	0.78
0.772	0.766	0.778	0.771	0.747	0.785 .	0.835	0.886	0.747	0.81	0.799	0.781	0.766	0.77	0.771	0.81
0.74	0.77	0.75	0.757	0.786	0.745 .	0.743	0.82	0.764	0.825	0.795	0.752	0.781	0.721	0.749	0.805
0.75	0.772	0.783	0.796	0.782	0.728 .	0.789	0.846	0.715	0.798	0.806	0.73	0.778	0.695	0.725	0.823
0.815	0.734	0.769	0.776	0.769	0.715 .	0.783	0.866	0.783	0.81	0.739	0.724	0.804	0.818	0.745	0.81
0.822	0.774	0.778	0.742	0.74	0.72 .	0.822	0.858	0.848	0.827	0.736	0.782	0.802	0.799	0.757	0.788
0.81	0.825	0.823	0.833	0.8	0.718 .	0.877	0.841	0.814	0.785	0.781	0.813	0.782	0.861	0.771	0.774
0.784	0.809	0.817	0.778	0.817	0.801 .	0.863	0.848	0.802	0.823	0.804	0.776	0.792	0.805	0.861	0.853
0.829	0.796	0.831	0.765	0.85	0.811 .	0.835	0.847	0.793	0.857	0.759	0.797	0.754	0.774	0.8	0.809
0.815	0.817	0.788	0.766	0.796	0.895 .	0.797	0.838	0.774	0.829	0.782	0.779	0.752	0.839	0.722	0.83
0.796	0.845	0.778	0.786	0.798	0.878 .	0.819	0.841	0.767	0.845	0.784	0.806	0.782	0.804	0.734	0.825
0.811	0.803	0.853	0.794	0.741	0.818 .	0.805	0.835	0.728	0.849	0.782	0.765	0.768	0.832	0.755	0.849
0.834	0.8	0.832	0.761	0.769	0.907 .	0.79	0.872	0.745	0.836	0.814	0.765	0.737	0.862	0.834	0.831
0.809	0.769	0.798	0.751	0.818	0.855 .	0.781	0.853	0.776	0.78	0.763	0.767	0.767	0.821	0.773	0.797
0.778	0.823	0.781	0.785	0.813	0.843 .	0.758	0.853	0.813	0.823	0.784	0.778	0.769	0.792	0.771	0.825
0.819	0.852	0.751	0.758	0.805	0.821 .	0.851	0.878	0.795	0.801	0.812	0.801	0.788	0.838	0.801	0.817
0.778	0.815	0.771	0.746	0.788	0.856 .	0.787	0.825	0.773	0.848	0.771	0.761	0.762	0.784	0.759	0.796
0.756	0.786	0.818	0.758	0.724	0.832 .	0.797	0.844	0.811	0.852	0.742	0.775	0.799	0.809	0.768	0.767
0.8	0.807	0.793	0.74	0.758	0.797 .	0.787	0.848	0.786	0.777	0.796	0.779	0.796	0.837	0.823	0.835
0.795	0.786	0.838	0.785	0.797	0.783 .	0.72	0.844	0.78	0.801	0.761	0.811	0.829	0.815	0.805	0.836
0.837	0.814	0.83	0.82	0.809	0.808 .	0.815	0.827	0.817	0.853	0.84	0.824	0.781	0.786	0.741	0.794
0.817	0.846	0.811	0.783	0.783	0.868 .	0.814	0.819	0.784	0.838	0.814	0.807	0.753	0.793	0.733	0.851
0.858	0.838	0.807	0.772	0.771	0.822 .	0.838	0.812	0.726	0.823	0.807	0.839	0.788	0.833	0.746	0.847
0.81	0.78	0.839	0.794	0.794	0.798 .	0.766	0.793	0.803	0.814	0.779	0.813	0.803	0.806	0.796	0.843
0.808	0.805	0.83	0.736	0.839	0.758 .	0.816	0.824	0.827	0.861	0.809	0.848	0.8	0.831	0.85	0.838
0.806	0.838	0.888	0.752	0.771	0.734 .	0.874	0.861	0.889	0.857	0.786	0.802	0.835	0.792	0.858	0.852
0.866	0.842	0.845	0.791	0.797	0.833 .	0.818	0.817	0.789	0.839	0.787	0.811	0.824	0.774	0.767	0.817
0.823	0.835	0.87	0.843	0.81	0.824 .	0.824	0.783	0.774	0.882	0.794	0.81	0.809	0.792	0.743	0.837
0.855	0.823	0.838	0.75	0.821	0.812 .	0.832	0.833	0.807	0.863	0.81	0.822	0.811	0.833	0.77	0.831
0.859	0.802	0.833	0.79	0.84	0.811 .	0.821	0.824	0.807	0.842	0.788	0.799	0.815	0.801	0.792	0.846
0.842	0.79	0.826	0.776	0.811	0.771 .	0.86	0.814	0.775	0.848	0.816	0.777	0.803	0.866	0.841	0.811
0.8	0.841	0.85	0.845	0.778	0.843 .	0.863	0.815	0.752	0.845	0.805	0.793	0.803	0.819	0.805	0.837
0.816	0.843	0.834	0.822	0.832	0.873 .	0.887	0.821	0.803	0.854	0.8	0.794	0.853	0.84	0.835	0.785
0.79	0.813	0.824	0.824	0.802	0.842 .	0.825	0.813	0.778	0.829	0.795	0.822	0.837	0.8	0.814	0.818
0.822	0.787	0.85	0.793	0.825	0.787 .	0.818	0.808	0.773	0.818	0.802	0.825	0.808	0.81	0.79	0.796
0.831	0.776	0.85	0.788	0.776	0.797 .	0.8	0.793	0.732	0.844	0.77	0.791	0.786	0.804	0.758	0.779
0.828	0.78	0.823	0.804	0.777	0.798 .	0.788	0.764	0.742	0.821	0.796	0.78	0.789	0.802	0.769	0.772
0.808	0.754	0.812	0.797	0.744	0.773 .	0.794	0.771	0.741	0.813	0.759	0.754	0.78	0.805	0.76	0.758
0.802	0.774	0.799	0.784	0.761	0.766 .	0.775	0.76	0.731	0.81	0.816	0.776	0.765	0.78	0.74	0.755
0.818	0.76	0.828	0.762	0.734	0.755 .	0.78	0.814	0.727	0.826	0.774	0.751	0.767	0.808	0.751	0.77
0.817	0.749	0.803	0.748	0.756	0.761 .	0.786	0.809	0.759	0.814	0.794	0.761	0.744	0.816	0.757	0.736
0.813	0.785	0.821	0.772	0.756	0.749 .	0.777	0.765	0.733	0.792	0.779	0.761	0.753	0.801	0.816	0.751
0.785	0.78	0.826	0.801	0.769	0.752 .	0.809	0.759	0.738	0.793	0.767	0.757	0.81	0.848	0.771	0.776
0.78	0.767	0.818	0.79	0.778	0.745 .	0.794	0.773	0.718	0.795	0.806	0.744	0.824	0.843	0.741	0.789
0.768	0.74	0.78	0.724	0.776	0.793 .	0.774	0.782	0.731	0.8	0.798	0.797	0.778	0.878	0.749	0.799
0.783	0.763	0.816	0.731	0.751	0.785 .	0.746	0.752	0.704	0.795	0.772	0.772	0.807	0.824	0.711	0.792
0.798	0.761	0.838	0.789	0.736	0.8 .	0.723	0.778	0.773	0.788	0.747	0.78	0.805	0.818	0.697	0.75
0.778	0.782	0.833	0.795	0.754	0.812 .	0.747	0.793	0.788	0.802	0.758	0.753	0.817	0.876	0.721	0.784
0.784	0.793	0.796	0.768	0.786	0.749 .	0.75	0.781	0.772	0.84	0.778	0.747	0.792	0.873	0.717	0.817
0.785	0.788	0.766	0.777	0.812	0.796 .	0.713	0.749	0.764	0.835	0.76	0.766	0.778	0.82	0.743	0.807
0.786	0.766	0.789	0.756	0.81	0.747 .	0.729	0.742	0.754	0.841	0.775	0.769	0.757	0.848	0.761	0.839
0.773	0.752	0.748	0.742	0.793	0.786 .	0.728	0.726	0.742	0.808	0.752	0.775	0.755	0.814	0.721	0.813
0.788	0.77	0.767	0.711	0.812	0.802 .	0.743	0.727	0.726	0.834	0.742	0.727	0.769	0.835	0.696	0.818
0.765	0.772	0.797	0.775	0.804	0.804 .	0.708	0.748	0.756	0.833	0.726	0.713	0.752	0.817	0.733	0.782
0.771	0.789	0.768	0.77	0.825	0.809 .	0.719	0.745	0.73	0.842	0.731	0.788	0.729	0.786	0.77	0.798
0.781	0.727	0.763	0.767	0.784	0.77 .	0.712	0.759	0.725	0.827	0.709	0.763	0.725	0.767	0.816	0.812
0.791	0.737	0.749	0.768	0.786	0.78 .	0.728	0.755	0.721	0.793	0.735	0.762	0.751	0.827	0.798	0.815
0.795	0.761	0.742	0.752	0.804	0.751 .	0.751	0.721	0.663	0.825	0.725	0.772	0.741	0.756	0.743	0.85
0.784	0.801	0.762	0.73	0.807	0.74 .	0.77	0.704	0.737	0.801	0.74	0.733	0.734	0.775	0.722	0.813
0.814	0.789	0.783	0.766	0.776	0.791 .	0.758	0.706	0.811	0.797	0.724	0.763	0.728	0.843	0.688	0.827
0.795	0.773	0.777	0.768	0.773	0.81 .	0.749	0.73	0.757	0.815	0.715	0.764	0.738	0.81	0.709	0.802
0.764	0.763	0.791	0.758	0.77	0.804 .	0.752	0.73	0.785	0.805	0.734	0.744	0.7	0.855	0.727	0.799
0.802	0.763	0.792	0.771	0.787	0.838 .	0.743	0.769	0.731	0.823	0.721	0.757	0.74	0.807	0.736	0.795
0.771	0.762	0.763	0.727	0.816	0.809 .	0.731	0.823	0.735	0.786	0.73	0.791	0.728	0.817	0.723	0.789
0.764	0.789	0.733	0.763	0.782	0.785 .	0.738	0.836	0.715	0.815	0.705	0.718	0.782	0.815	0.676	0.787
0.763	0.761	0.751	0.741	0.756	0.791 .	0.724	0.761	0.713	0.846	0.697	0.756	0.745	0.745	0.679	0.786
0.787	0.763	0.794	0.725	0.762	0.778 .	0.694	0.793	0.719	0.814	0.711	0.757	0.75	0.767	0.757	0.856
0.761	0.776	0.837	0.72	0.772	0.758 .	0.689	0.842	0.655	0.829	0.72	0.732	0.709	0.777	0.743	0.803
0.755	0.735	0.81	0.716	0.756	0.76 .	0.789	0.83	0.682	0.843	0.77	0.722	0.696	0.783	0.773	0.802
0.755	0.781	0.742	0.757	0.74	0.75 .	0.854	0.81	0.756	0.829	0.716	0.803	0.698	0.814	0.738	0.79
0.794	0.776	0.725	0.759	0.769	0.874 .	0.792	0.767	0.826	0.834	0.723	0.798	0.742	0.864	0.694	0.856
0.744	0.81	0.835	0.803	0.818	0.845 .	0.756	0.781	0.763	0.812	0.76	0.844	0.714	0.842	0.706	0.813
0.789	0.755	0.786	0.743	0.806	0.838 .	0.849	0.724	0.785	0.85	0.798	0.838	0.684	0.809	0.806	0.793
0.82	0.835	0.788	0.792	0.813	0.781 .	0.81	0.771	0.732	0.873	0.795	0.797	0.72	0.782	0.733	0.774
0.794	0.801	0.815	0.788	0.824	0.79 .	0.806	0.78	0.77	0.847	0.779	0.874	0.724	0.779	0.745	0.779
0.784	0.749	0.799	0.758	0.812	0.873 .	0.825	0.768	0.753	0.819	0.761	0.823	0.743	0.823	0.758	0.761
0.838	0.766	0.865	0.773	0.817	0.846 .	0.755	0.733	0.743	0.832	0.799	0.88	0.743	0.798	0.798	0.841
0.78	0.751	0.821	0.765	0.777	0.831 .	0.769	0.809	0.762	0.827	0.772	0.862	0.776	0.831	0.759	0.865
0.789	0.758	0.842	0.781	0.744	0.758 .	0.874	0.767	0.731	0.866	0.836	0.84	0.763	0.852	0.702	0.849
0.819	0.78	0.894	0.823	0.786	0.752 .	0.854	0.799	0.726	0.855	0.782	0.814	0.796	0.828	0.712	0.807
0.81	0.832	0.819	0.823	0.836	0.776 .	0.831	0.807	0.767	0.806	0.81	0.802	0.751	0.794	0.707	0.842
0.791	0.819	0.799	0.83	0.804	0.836 .	0.794	0.802	0.753	0.81	0.77	0.812	0.695	0.758	0.739	0.84
0.756	0.737	0.886	0.788	0.747	0.766 .	0.789	0.775	0.706	0.82	0.758	0.787	0.701	0.748	0.683	0.848
0.787	0.775	0.879	0.776	0.815	0.814 .	0.815	0.758	0.747	0.785	0.796	0.755	0.781	0.76	0.676	0.813
0.811	0.791	0.88	0.872	0.794	0.776 .	0.814	0.801	0.723	0.775	0.779	0.759	0.825	0.82	0.731	0.82
0.781	0.758	0.86	0.819	0.79	0.807 .	0.829	0.83	0.725	0.871	0.844	0.844	0.762	0.809	0.771	0.845
0.802	0.776	0.789	0.831	0.818	0.805 .	0.797	0.789	0.801	0.862	0.83	0.819	0.742	0.793	0.746	0.887
0.8	0.805	0.762	0.856	0.871	0.763 .	0.848	0.768	0.791	0.829	0.787	0.824	0.817	0.792	0.729	0.876
0.787	0.785	0.883	0.812	0.801	0.843 .	0.747	0.772	0.767	0.798	0.792	0.85	0.835	0.817	0.774	0.874
0.811	0.75	0.894	0.801	0.876	0.831 .	0.842	0.75	0.782	0.841	0.862	0.87	0.823	0.751	0.785	0.839
0.827	0.84	0.899	0.816	0.821	0.818 .	0.804	0.794	0.807	0.857	0.86	0.843	0.818	0.841	0.818	0.847
0.789	0.803	0.83	0.817	0.81	0.825 .	0.789	0.747	0.753	0.827	0.834	0.868	0.851	0.799	0.744	0.847
0.817	0.855	0.87	0.828	0.835	0.846 .	0.846	0.755	0.752	0.871	0.801	0.845	0.847	0.793	0.725	0.877
0.84	0.8	0.89	0.821	0.875	0.831 .	0.858	0.796	0.777	0.854	0.807	0.854	0.852	0.846	0.763	0.875
0.777	0.818	0.892	0.801	0.865	0.784 .	0.818	0.8	0.742	0.872	0.842	0.86	0.836	0.825	0.735	0.828
0.784	0.83	0.869	0.847	0.856	0.872 .	0.876	0.788	0.818	0.836	0.87	0.861	0.905	0.857	0.737	0.811
0.832	0.807	0.845	0.804	0.828	0.822 .	0.861	0.754	0.796	0.802	0.824	0.864	0.825	0.796	0.756	0.829
0.85	0.821	0.87	0.833	0.802	0.81 .	0.842	0.786	0.792	0.83	0.838	0.854	0.857	0.794	0.737	0.815
0.828	0.841	0.879	0.837	0.81	0.862 .	0.86	0.769	0.783	0.817	0.818	0.857	0.853	0.795	0.729	0.841
0.817	0.808	0.864	0.863	0.799	0.845 .	0.824	0.838	0.75	0.841	0.838	0.809	0.836	0.875	0.785	0.855
0.83	0.802	0.839	0.819	0.782	0.798 .	0.83	0.826	0.716	0.844	0.831	0.803	0.819	0.804	0.748	0.833
0.826	0.768	0.845	0.795	0.78	0.811 .	0.802	0.765	0.701	0.823	0.792	0.795	0.813	0.793	0.731	0.802

0.823	0.77	0.839	0.816	0.76	0.825 .	0.807	0.755	0.711	0.848	0.81	0.792	0.847	0.797	0.721	0.79
0.826	0.763	0.834	0.787	0.768	0.792 .	0.759	0.743	0.7	0.822	0.798	0.798	0.779	0.766	0.703	0.794
0.77	0.761	0.811	0.794	0.781	0.81 .	0.756	0.764	0.71	0.803	0.792	0.797	0.78	0.779	0.667	0.79
0.818	0.791	0.816	0.832	0.753	0.79 .	0.752	0.732	0.7	0.797	0.808	0.781	0.773	0.779	0.729	0.766
0.824	0.846	0.796	0.809	0.824	0.789 .	0.749	0.692	0.715	0.83	0.798	0.783	0.782	0.781	0.723	0.762
0.803	0.802	0.818	0.783	0.765	0.8 .	0.819	0.73	0.707	0.833	0.808	0.802	0.796	0.795	0.698	0.771
0.817	0.77	0.81	0.788	0.763	0.787 .	0.841	0.804	0.694	0.82	0.839	0.759	0.756	0.771	0.709	0.794
0.828	0.762	0.819	0.822	0.755	0.761 .	0.823	0.751	0.707	0.806	0.814	0.768	0.8	0.774	0.655	0.804
0.791	0.766	0.838	0.809	0.728	0.751 .	0.831	0.782	0.732	0.864	0.786	0.726	0.843	0.812	0.744	0.799
0.826	0.758	0.853	0.808	0.797	0.786 .	0.819	0.803	0.727	0.817	0.808	0.736	0.817	0.8	0.727	0.756
0.785	0.786	0.836	0.796	0.779	0.775 .	0.84	0.806	0.767	0.82	0.829	0.808	0.81	0.843	0.706	0.736
0.785	0.804	0.882	0.765	0.768	0.781 .	0.863	0.796	0.765	0.821	0.823	0.825	0.809	0.837	0.7	0.739
0.791	0.809	0.844	0.759	0.732	0.75 .	0.84	0.774	0.716	0.823	0.798	0.788	0.791	0.821	0.676	0.722
0.783	0.753	0.846	0.752	0.736	0.824 .	0.818	0.798	0.694	0.815	0.79	0.83	0.852	0.829	0.685	0.823
0.746	0.75	0.82	0.769	0.726	0.804 .	0.805	0.788	0.675	0.802	0.778	0.783	0.839	0.794	0.683	0.826
0.784	0.737	0.782	0.801	0.804	0.794 .	0.811	0.77	0.736	0.809	0.786	0.791	0.799	0.787	0.699	0.793
0.783	0.764	0.795	0.773	0.774	0.748 .	0.831	0.756	0.705	0.826	0.766	0.773	0.799	0.811	0.672	0.773
0.754	0.743	0.776	0.776	0.772	0.772 .	0.826	0.768	0.709	0.812	0.8	0.798	0.785	0.8	0.663	0.76
0.754	0.728	0.81	0.784	0.783	0.788 .	0.775	0.749	0.699	0.809	0.826	0.793	0.771	0.744	0.698	0.754
0.725	0.754	0.804	0.761	0.772	0.77 .	0.753	0.753	0.708	0.775	0.798	0.826	0.815	0.762	0.675	0.78
0.769	0.789	0.812	0.793	0.779	0.8 .	0.783	0.719	0.672	0.812	0.779	0.83	0.832	0.802	0.686	0.805
0.758	0.761	0.782	0.801	0.801	0.76 .	0.846	0.729	0.71	0.842	0.767	0.83	0.873	0.79	0.694	0.827
0.733	0.752	0.752	0.781	0.766	0.732 .	0.87	0.706	0.716	0.849	0.816	0.792	0.862	0.839	0.688	0.799
0.727	0.756	0.788	0.799	0.741	0.743 .	0.809	0.724	0.714	0.824	0.771	0.854	0.796	0.834	0.706	0.796
0.763	0.792	0.859	0.803	0.742	0.767 .	0.84	0.71	0.649	0.818	0.781	0.866	0.831	0.81	0.693	0.778
0.733	0.811	0.844	0.782	0.74	0.785 .	0.815	0.694	0.683	0.823	0.772	0.795	0.822	0.767	0.691	0.786
0.725	0.762	0.853	0.782	0.742	0.793 .	0.855	0.694	0.691	0.817	0.786	0.807	0.835	0.816	0.675	0.786
0.741	0.739	0.823	0.792	0.745	0.781 .	0.804	0.706	0.668	0.813	0.757	0.78	0.803	0.835	0.686	0.81
0.724	0.72	0.799	0.763	0.756	0.825 .	0.795	0.706	0.676	0.787	0.791	0.788	0.781	0.828	0.7	0.788
0.75	0.741	0.799	0.766	0.7	0.788 .	0.75	0.719	0.693	0.813	0.743	0.765	0.802	0.802	0.689	0.817
0.709	0.787	0.801	0.737	0.756	0.817 .	0.702	0.681	0.696	0.79	0.798	0.805	0.766	0.843	0.645	0.811
0.706	0.734	0.763	0.729	0.765	0.786 .	0.797	0.66	0.677	0.808	0.791	0.806	0.754	0.806	0.654	0.816
0.712	0.714	0.748	0.708	0.717	0.747 .	0.838	0.724	0.684	0.777	0.749	0.847	0.727	0.806	0.662	0.796
0.733	0.792	0.808	0.788	0.702	0.768 .	0.855	0.786	0.75	0.784	0.75	0.784	0.785	0.767	0.658	0.761
0.754	0.781	0.814	0.8	0.743	0.726 .	0.804	0.733	0.728	0.781	0.78	0.772	0.828	0.815	0.622	0.84
0.761	0.839	0.857	0.795	0.765	0.741 .	0.754	0.716	0.702	0.849	0.827	0.839	0.815	0.838	0.7	0.829
0.759	0.812	0.859	0.805	0.762	0.835 .	0.852	0.788	0.722	0.856	0.795	0.825	0.79	0.85	0.689	0.815
0.805	0.832	0.842	0.825	0.829	0.803 .	0.806	0.74	0.71	0.781	0.782	0.869	0.838	0.824	0.71	0.813
0.772	0.847	0.854	0.784	0.76	0.78 .	0.822	0.761	0.76	0.85	0.814	0.824	0.867	0.848	0.708	0.844
0.808	0.761	0.838	0.752	0.782	0.787 .	0.814	0.856	0.732	0.789	0.779	0.851	0.802	0.829	0.676	0.795
0.761	0.81	0.821	0.787	0.788	0.788 .	0.811	0.807	0.711	0.798	0.831	0.851	0.831	0.824	0.738	0.809
0.725	0.803	0.776	0.777	0.74	0.844 .	0.87	0.832	0.692	0.833	0.788	0.841	0.828	0.784	0.703	0.838
0.766	0.822	0.731	0.78	0.693	0.813 .	0.803	0.865	0.751	0.833	0.76	0.815	0.824	0.782	0.668	0.792
0.785	0.807	0.784	0.807	0.774	0.786 .	0.79	0.805	0.816	0.797	0.782	0.818	0.794	0.811	0.687	0.787
0.74	0.81	0.803	0.78	0.786	0.758 .	0.815	0.856	0.801	0.767	0.808	0.853	0.786	0.758	0.703	0.813
0.823	0.772	0.883	0.868	0.777	0.807 .	0.83	0.854	0.785	0.772	0.796	0.887	0.833	0.751	0.763	0.815
0.774	0.832	0.823	0.856	0.753	0.814	0.837	0.842	0.728	0.731	0.792	0.819	0.839	0.809	0.749	0.777
0.786	0.842	0.773	0.845	0.847	0.807	0.809	0.795	0.703	0.787	0.826	0.863	0.821	0.833	0.761	0.754
0.829	0.772	0.774	0.813	0.828	0.765	0.716	0.774	0.678	0.808	0.781	0.86	0.781	0.747	0.746	0.711
0.799	0.788	0.84	0.82	0.753	0.81	0.76	0.845	0.725	0.766	0.767	0.816	0.821	0.801	0.752	0.728
0.819	0.826	0.772	0.827	0.741	0.803	0.877	0.839	0.769	0.751	0.812	0.808	0.813	0.832	0.749	0.782
0.843	0.848	0.839	0.803	0.743	0.787	0.847	0.801	0.787	0.796	0.82	0.855	0.812	0.818	0.738	0.784
0.801	0.814	0.796	0.804	0.773	0.811	0.789	0.8	0.737	0.813	0.794	0.839	0.827	0.817	0.658	0.763
0.744	0.781	0.793	0.77	0.802	0.812	0.796	0.807	0.735	0.796	0.802	0.861	0.849	0.845	0.659	0.767
0.761	0.851	0.806	0.775	0.764	0.804	0.818	0.817	0.734	0.815	0.831	0.855	0.811	0.848	0.667	0.734
0.807	0.817	0.826	0.775	0.766	0.784	0.849	0.777	0.783	0.849	0.792	0.876	0.733	0.83	0.755	0.787
0.773	0.784	0.808	0.813	0.817	0.778	0.833	0.822	0.789	0.822	0.778	0.858	0.812	0.76	0.718	0.798
0.757	0.786	0.847	0.803	0.783	0.827	0.827	0.819	0.807	0.823	0.788	0.8	0.798	0.769	0.787	0.777
0.779	0.809	0.826	0.808	0.8	0.795	0.766	0.786	0.832	0.806	0.826	0.865	0.749	0.771	0.753	0.726
0.821	0.797	0.811	0.795	0.778	0.779	0.786	0.748	0.789	0.864	0.8	0.849	0.812	0.801	0.763	0.76
0.832	0.804	0.883	0.833	0.748	0.772	0.835	0.779	0.731	0.857	0.875	0.845	0.83	0.846	0.797	0.816
0.802	0.833	0.855	0.83	0.706	0.83	0.833	0.824	0.754	0.856	0.832	0.872	0.87	0.846	0.75	0.802
0.833	0.835	0.837	0.837	0.762	0.802	0.866	0.819	0.776	0.841	0.822	0.869	0.83	0.8	0.76	0.785
0.828	0.787	0.808	0.774	0.749	0.782	0.818	0.803	0.728	0.805	0.831	0.816	0.825	0.803	0.715	0.793
0.808	0.78	0.824	0.794	0.769	0.767	0.806	0.758	0.709	0.807	0.809	0.813	0.792	0.797	0.716	0.834
0.842	0.777	0.815	0.775	0.755	0.737	0.8	0.764	0.711	0.835	0.796	0.792	0.771	0.789	0.729	0.809
0.807	0.802	0.824	0.833	0.739	0.734	0.82	0.77	0.702	0.799	0.78	0.822	0.749	0.762	0.723	0.779
0.792	0.751	0.816	0.832	0.766	0.78	0.822	0.718	0.708	0.807	0.776	0.803	0.726	0.755	0.749	0.788
0.755	0.806	0.836	0.824	0.788	0.794	0.816	0.737	0.712	0.81	0.778	0.791	0.733	0.774	0.769	0.767
0.827	0.821	0.828	0.789	0.805	0.769	0.786	0.768	0.721	0.798	0.786	0.809	0.701	0.766	0.749	0.768
0.816	0.794	0.822	0.754	0.781	0.788	0.766	0.733	0.717	0.798	0.777	0.806	0.722	0.746	0.741	0.738
0.823	0.79	0.805	0.767	0.808	0.756	0.77	0.773	0.75	0.798	0.77	0.79	0.805	0.801	0.747	0.778
0.761	0.805	0.809	0.833	0.771	0.746	0.759	0.791	0.693	0.786	0.8	0.761	0.797	0.814	0.734	0.758
0.752	0.808	0.785	0.795	0.753	0.715	0.822	0.819	0.712	0.765	0.785	0.81	0.766	0.762	0.723	0.79
0.743	0.815	0.746	0.761	0.801	0.704	0.805	0.828	0.662	0.834	0.779	0.87	0.813	0.741	0.747	0.768
0.812	0.799	0.753	0.764	0.796	0.698	0.851	0.809	0.733	0.806	0.766	0.827	0.785	0.714	0.756	0.753
0.815	0.798	0.817	0.785	0.798	0.749	0.83	0.779	0.757	0.809	0.774	0.813	0.76	0.731	0.699	0.774
0.81	0.77	0.845	0.826	0.783	0.794	0.78	0.785	0.76	0.819	0.768	0.805	0.792	0.807	0.714	0.783
0.797	0.809	0.854	0.843	0.761	0.753	0.843	0.741	0.769	0.835	0.782	0.817	0.774	0.798	0.665	0.788
0.782	0.808	0.823	0.797	0.746	0.724	0.853	0.722	0.722	0.795	0.768	0.8	0.743	0.751	0.75	0.77
0.812	0.757	0.84	0.779	0.73	0.746	0.857	0.745	0.7	0.776	0.75	0.806	0.769	0.768	0.711	0.753
0.811	0.769	0.843	0.8	0.724	0.723	0.843	0.753	0.684	0.78	0.748	0.758	0.79	0.703	0.694	0.766
0.792	0.769	0.817	0.798	0.731	0.739	0.84	0.781	0.708	0.788	0.742	0.735	0.759	0.763	0.778	0.735
0.831	0.762	0.83	0.777	0.761	0.804	0.826	0.768	0.71	0.801	0.737	0.833	0.778	0.754	0.779	0.755
0.794	0.782	0.805	0.775	0.747	0.749	0.848	0.772	0.714	0.805	0.692	0.805	0.706	0.76	0.763	0.784
0.806	0.778	0.826	0.807	0.754	0.727	0.836	0.749	0.674	0.806	0.721	0.797	0.683	0.725	0.734	0.76
0.782	0.786	0.808	0.762	0.748	0.758	0.842	0.753	0.729	0.789	0.72	0.777	0.715	0.758	0.732	0.739
0.776	0.803	0.816	0.745	0.737	0.749	0.862	0.748	0.715	0.789	0.72	0.752	0.728	0.781	0.71	0.741
0.796	0.776	0.828	0.807	0.725	0.733	0.801	0.693	0.743	0.787	0.735	0.747	0.634	0.757	0.737	0.732
0.8	0.75	0.794	0.807	0.725	0.717	0.819	0.704	0.75	0.814	0.712	0.831	0.651	0.755	0.755	0.722
0.757	0.754	0.789	0.75	0.733	0.71	0.828	0.742	0.712	0.809	0.72	0.807	0.689	0.728	0.722	0.71
0.761	0.772	0.793	0.797	0.727	0.697	0.802	0.767	0.717	0.838	0.728	0.809	0.652	0.717	0.733	0.696
0.795	0.764	0.824	0.825	0.733	0.805	0.813	0.783	0.729	0.828	0.711	0.794	0.713	0.743	0.715	0.723
0.788	0.776	0.824	0.789	0.717	0.797	0.814	0.776	0.658	0.806	0.717	0.769	0.709	0.712	0.668	0.688
0.771	0.777	0.797	0.783	0.765	0.786	0.817	0.78	0.649	0.792	0.695	0.789	0.714	0.773	0.699	0.717
0.777	0.737	0.759	0.789	0.764	0.777	0.818	0.773	0.707	0.771	0.726	0.806	0.694	0.799	0.774	0.71
0.798	0.755	0.799	0.756	0.767	0.78	0.867	0.8	0.727	0.768	0.727	0.8	0.709	0.78	0.733	0.744
0.739	0.718	0.762	0.709	0.734	0.756	0.826	0.723	0.716	0.734	0.724	0.826	0.651	0.7	0.791	0.701
0.722	0.728	0.817	0.795	0.714	0.733	0.852	0.783	0.791	0.799	0.736	0.759	0.658	0.71	0.757	0.671
0.763	0.746	0.827	0.79	0.701	0.728	0.819	0.747	0.764	0.777	0.69	0.79	0.658	0.741	0.772	0.698
0.752	0.724	0.797	0.805	0.73	0.81	0.862	0.759	0.751	0.787	0.731	0.854	0.679	0.798	0.748	0.726
0.799	0.774	0.789	0.803	0.708	0.796	0.82	0.807	0.654	0.81	0.724	0.808	0.685	0.744	0.692	0.727
0.775	0.778	0.785	0.755	0.724	0.766	0.824	0.822	0.73	0.767	0.733	0.776	0.661	0.758	0.714	0.731
0.779	0.775	0.814	0.817	0.804	0.743	0.823	0.781	0.735	0.743	0.71	0.798	0.652	0.757	0.704	0.733
0.79	0.765	0.793	0.78	0.754	0.815	0.855	0.844	0.777	0.821	0.712	0.846	0.65	0.747	0.771	0.743

0.765	0.752	0.78	0.771	0.786	0.778	0.797	0.809	0.796	0.784	0.752	0.866	0.679	0.689	0.703	0.781
0.831	0.761	0.835	0.746	0.811	0.76	0.844	0.822	0.769	0.757	0.733	0.827	0.683	0.729	0.729	0.738
0.733	0.794	0.769	0.755	0.78	0.748	0.841	0.88	0.749	0.744	0.719	0.818	0.658	0.723	0.729	0.69
0.733	0.763	0.762	0.794	0.733	0.847	0.826	0.866	0.668	0.757	0.726	0.776	0.729	0.697	0.694	0.689
0.765	0.769	0.829	0.764	0.776	0.816	0.788	0.824	0.723	0.793	0.718	0.834	0.686	0.772	0.797	0.713
															0.774
															0.829
															0.763

10	10	12	12	12	12	3	3	4	4	5	7	7	8	10	10	4	4	5
Box-5	Box-4	Box-1	Box-2	Box-5	Box-6	Box-1	Box-2	Box-6	Box-7	Box-6	Box-2	Box-5	Box-3	Box-1	Box-3	Box-1	Box-4	Box-5
63	69	74	76	81	83	1.7	2.3	25.3	26.6	34.6	38	43.6	52.6	64	75	13.3	21.5	32.6
F	F	F	F	F	F	F	F	F	F	F	F	F	F	F	F	F	F	F
H2O	H2O	H2O	H2O	H2O	H2O	Malto	Malto	Malto	Malto	Malto	Malto	Malto	Malto	Malto	Malto	MCT	MCT	MCT
FH	FH	FH	FH	FH	FH	FM	FM	FM	FM	FM	FM	FM	FM	FM	FM	FT	FT	FT
0.744	0.725	0.711	0.753	0.761	0.757	0.71	0.721	0.744	0.735	0.697	0.679	.	0.658	0.726	0.719	0.74	0.702	0.742
0.754	0.738	0.752	0.787	0.74	0.778	0.75	0.747	0.758	0.71	0.741	0.722	.	0.749	0.718	0.73	0.717	0.725	0.748
0.725	0.741	0.738	0.741	0.755	0.74	0.76	0.732	0.744	0.727	0.814	0.749	.	0.752	0.736	0.719	0.736	0.743	0.773
0.708	0.702	0.704	0.729	0.737	0.743	0.756	0.8	0.716	0.726	0.763	0.714	.	0.67	0.701	0.728	0.75	0.73	0.727
0.741	0.723	0.742	0.765	0.745	0.774	0.746	0.828	0.736	0.759	0.796	0.709	.	0.693	0.753	0.725	0.704	0.735	0.745
0.821	0.737	0.746	0.747	0.744	0.784	0.769	0.846	0.746	0.722	0.856	0.689	.	0.712	0.729	0.732	0.713	0.727	0.726
0.834	0.724	0.723	0.718	0.754	0.781	0.789	0.823	0.717	0.712	0.893	0.746	.	0.702	0.716	0.709	0.711	0.709	0.763
0.85	0.732	0.759	0.75	0.729	0.739	0.827	0.835	0.732	0.757	0.894	0.873	.	0.711	0.733	0.721	0.707	0.736	0.74
0.883	0.713	0.729	0.793	0.717	0.735	0.852	0.893	0.723	0.802	0.905	0.897	.	0.723	0.714	0.696	0.672	0.738	0.779
0.906	0.721	0.728	0.803	0.719	0.73	0.855	0.901	0.727	0.771	0.88	0.912	.	0.682	0.721	0.715	0.634	0.736	0.754
0.913	0.714	0.742	0.848	0.838	0.77	0.877	0.917	0.683	0.765	0.916	0.918	.	0.727	0.705	0.71	0.706	0.7	0.736
0.922	0.685	0.718	0.839	0.869	0.732	0.842	0.925	0.707	0.846	0.914	0.949	.	0.661	0.704	0.723	0.702	0.721	0.783
0.918	0.693	0.728	0.872	0.849	0.727	0.868	0.905	0.758	0.825	0.889	0.928	.	0.665	0.746	0.681	0.689	0.698	0.839
0.956	0.72	0.741	0.848	0.915	0.703	0.881	0.936	0.736	0.808	0.868	0.92	.	0.685	0.833	0.696	0.681	0.711	0.809
0.941	0.713	0.755	0.881	0.9	0.739	0.902	0.942	0.774	0.886	0.897	0.92	.	0.672	0.876	0.696	0.67	0.733	0.789
0.946	0.714	0.81	0.884	0.918	0.742	0.899	0.938	0.745	0.871	0.904	0.956	.	0.7	0.886	0.781	0.666	0.713	0.861
0.951	0.699	0.855	0.904	0.922	0.771	0.891	0.946	0.743	0.886	0.875	0.925	.	0.579	0.866	0.854	0.632	0.717	0.866
0.938	0.704	0.868	0.904	0.924	0.852	0.869	0.956	0.818	0.914	0.92	0.926	.	0.643	0.915	0.887	0.55	0.749	0.911
0.934	0.711	0.861	0.906	0.909	0.858	0.871	0.942	0.847	0.91	0.927	0.927	.	0.656	0.924	0.87	0.691	0.816	0.901
0.939	0.718	0.889	0.934	0.929	0.888	0.88	0.934	0.88	0.901	0.905	0.933	.	0.653	0.951	0.901	0.667	0.868	0.914
0.978	0.671	0.906	0.901	0.937	0.884	0.903	0.958	0.873	0.888	0.968	0.916	.	0.783	0.941	0.899	0.617	0.876	0.899
0.959	0.665	0.891	0.895	0.952	0.929	0.875	0.944	0.838	0.91	0.946	0.948	.	0.808	0.923	0.915	0.526	0.887	0.917
0.953	0.704	0.9	0.905	0.955	0.911	0.874	0.931	0.867	0.883	0.943	0.946	.	0.783	0.886	0.881	-	0.877	0.887
1.001	0.695	0.91	0.887	0.96	0.923	0.899	0.955	0.897	0.904	0.935	0.925	.	0.795	0.921	0.907	-	0.88	0.935
0.958	0.658	0.888	0.833	0.959	0.893	0.907	0.97	0.918	0.938	0.923	0.882	.	0.803	0.91	0.907	0.543	0.949	0.957
0.931	0.623	0.898	0.853	0.997	0.95	0.897	1.004	0.885	0.891	0.909	0.924	.	0.81	0.926	0.896	0.675	0.92	0.927
1.036	0.651	0.897	0.792	0.99	0.922	0.904	0.976	0.85	0.879	0.989	0.957	.	0.844	0.952	0.905	0.673	0.888	0.904
0.988	0.698	0.863	0.803	0.976	0.927	0.925	0.952	0.903	0.916	0.961	0.978	.	0.872	0.935	0.917	0.55	0.902	0.934
0.978	0.706	0.938	0.809	1.034	0.947	0.909	0.999	0.907	0.941	0.961	0.858	.	0.879	0.944	0.949	0.482	0.917	0.913
0.982	0.7	0.937	0.81	1.011	0.949	0.92	0.98	0.909	0.889	0.945	0.901	.	0.899	0.954	0.935	0.439	0.924	0.952
0.966	0.673	0.892	0.858	0.995	0.949	0.894	0.98	0.892	0.88	0.943	0.866	.	0.879	0.967	0.985	-	0.882	0.961
0.997	0.668	0.934	0.828	0.967	0.96	0.917	0.974	0.908	0.916	0.957	0.83	.	0.87	0.935	0.924	-	0.942	0.931
0.994	0.713	0.948	0.825	0.982	0.961	0.924	0.977	0.918	0.916	0.941	0.827	.	0.881	0.968	0.904	0.565	0.903	0.958
0.984	0.705	0.948	0.856	0.981	0.94	0.89	0.966	0.909	0.927	0.951	0.787	.	0.868	0.95	0.934	0.667	0.928	0.949
0.967	0.707	0.918	0.783	1.008	0.916	0.847	0.957	0.912	0.941	0.919	0.859	.	0.809	0.948	0.907	0.642	0.934	0.963
0.994	0.708	0.954	0.821	1.015	0.943	0.842	0.962	0.893	0.909	0.928	0.889	.	0.842	0.913	0.923	0.588	0.949	0.944
0.979	0.695	0.913	0.764	1.002	0.947	0.845	0.954	0.952	0.969	0.956	0.929	.	0.836	0.953	0.905	-	0.928	0.967
0.981	0.638	0.896	0.747	0.987	0.957	0.805	0.952	0.939	0.908	0.949	0.914	.	0.917	0.923	0.891	-	0.961	0.963
0.983	0.696	0.87	0.738	0.978	0.955	0.78	0.963	0.931	0.909	0.917	0.93	.	0.81	0.911	0.892	-	0.948	0.961
0.967	0.708	0.92	0.802	0.99	0.959	0.756	0.97	0.937	0.851	0.95	0.929	.	0.787	0.918	0.893	-	0.934	0.894
0.979	0.726	0.906	0.871	0.982	0.943	0.75	0.967	0.94	0.895	0.945	0.831	.	0.756	0.866	0.922	0.559	0.944	0.925
0.971	0.687	0.942	0.893	0.963	0.936	0.74	0.982	0.977	0.917	0.914	0.877	.	0.735	0.85	0.926	0.656	0.971	0.998
0.969	0.734	0.914	0.875	0.984	0.929	0.794	0.99	0.919	0.908	0.917	0.923	.	0.689	0.905	0.963	0.669	0.901	0.917
0.907	0.679	0.89	0.878	0.953	0.986	0.811	0.968	0.93	0.88	0.895	0.927	.	0.71	0.782	0.929	0.637	0.939	0.986
0.903	0.694	0.909	0.9	0.952	0.965	0.792	0.959	0.924	0.835	0.869	0.929	.	0.709	0.811	0.913	0.597	0.946	0.925
0.913	0.7	0.884	0.916	0.941	0.992	0.803	0.936	0.948	0.866	0.924	0.907	.	0.643	0.779	0.923	0.483	0.95	0.946
0.884	0.707	0.86	0.858	0.917	0.95	0.727	0.9	0.916	0.822	0.926	0.856	.	0.667	0.77	0.93	-	0.918	0.937
0.897	0.707	0.881	0.852	0.869	0.947	0.734	0.923	0.927	0.837	0.863	0.938	.	0.693	0.806	0.911	-	0.943	0.957
0.861	0.714	0.893	0.859	0.95	0.984	0.762	0.891	0.927	0.923	0.835	0.948	.	0.658	0.773	0.884	0.599	0.952	0.967
0.867	0.677	0.869	0.903	0.959	0.969	0.737	0.856	0.965	0.937	0.856	0.882	.	0.674	0.784	0.932	0.641	0.897	0.965
0.833	0.661	0.883	0.944	0.917	0.954	0.739	0.878	0.928	0.921	0.927	0.881	.	0.67	0.744	0.903	0.659	0.894	0.959
0.793	0.677	0.837	0.93	0.894	0.972	0.72	0.903	0.947	0.908	0.882	0.921	.	0.705	0.782	0.884	0.639	0.9	0.973
0.766	0.65	0.82	0.939	0.933	0.981	0.711	0.901	0.935	0.886	0.875	0.955	.	0.684	0.758	0.863	0.658	0.864	0.941
0.752	0.694	0.849	0.921	0.943	0.939	0.747	0.86	0.972	0.972	0.841	0.953	.	0.679	0.724	0.899	0.559	0.892	0.945
0.825	0.721	0.828	0.938	0.929	0.97	0.747	0.85	0.936	0.929	0.882	0.929	.	0.591	0.721	0.934	0.497	0.804	0.949
0.816	0.661	0.805	0.964	0.864	0.949	0.737	0.831	0.914	0.905	0.867	0.907	.	0.627	0.721	0.944	-	0.848	0.937
0.807	0.697	0.794	0.906	0.851	0.945	0.757	0.845	0.958	0.893	0.801	0.978	.	0.678	0.717	0.942	-	0.877	0.959
0.775	0.697	0.8	0.933	0.938	0.92	0.741	0.824	0.925	0.91	0.889	0.97	.	0.666	0.721	0.934	0.638	0.846	0.928
0.784	0.692	0.852	0.901	0.961	0.918	0.689	0.814	0.95	0.927	0.878	1.008	.	0.685	0.78	0.926	-	0.832	0.926
0.744	0.663	0.887	0.91	0.969	0.962	0.71	0.847	0.951	0.946	0.823	0.98	.	0.714	0.724	0.927	0.532	0.888	0.933
0.78	0.698	0.902	0.926	0.947	0.925	0.766	0.797	0.923	0.923	0.805	0.956	.	0.698	0.712	0.95	0.584	0.895	0.923
0.881	0.694	0.91	0.917	0.921	0.952	0.834	0.794	0.946	0.943	0.942	0.923	.	0.831	0.764	0.959	0.689	0.914	0.936
0.885	0.701	0.893	0.887	0.901	0.955	0.902	0.853	0.93	0.924	0.881	0.95	.	0.894	0.757	0.932	0.691	0.909	0.959
0.805	0.698	0.875	0.895	0.964	0.944	0.909	0.921	0.961	0.943	0.872	0.97	.	0.889	0.718	0.923	0.801	0.91	0.943
0.821	0.677	0.905	0.939	0.913	0.915	0.922	0.906	0.932	0.956	0.89	0.886	.	0.94	0.716	0.936	0.861	0.874	0.944
0.829	0.668	0.901	0.916	0.89	0.933	0.892	0.88	0.956	0.899	0.883	0.957	.	0.894	0.697	0.913	0.793	0.876	0.963
0.915	0.686	0.907	0.917	0.879	0.93	0.928	0.882	0.952	0.957	0.84	0.96	.	0.915	0.719	0.968	0.831	0.863	0.967
0.9	0.692	0.912	0.909	0.872	0.931	0.938	0.92	0.925	0.916	0.892	0.9	.	0.93	0.756	0.919	0.887	0.913	0.964
0.956	0.691	0.898	0.893	0.959	0.938	0.95	0.957	0.926	0.929	0.869	0.903	.	0.891	0.868	0.958	0.88	0.889	0.931
0.966	0.683	0.937	0.883	0.927	0.993	0.938	0.941	0.95	0.938	0.944	0.913	.	0.927	0.901	0.941	0.879	0.935	0.951
0.942	0.674	0.933	0.942	0.859	0.993	0.908	0.945	0.948	0.919	0.973	0.922	.	0.933	0.922	0.94	0.869	0.925	0.926
0.945	0.693	0.911	0.932	0.927	0.986	0.923	0.945	0.933	0.913	0.94	0.953	.	0.943	0.944	0.973	0.883	0.94	0.939
0.966	0.7	0.943	0.917	0.918	0.981	0.932	0.92	0.917	0.926	0.941	0.919	.	0.946	0.955	0.954	0.864	0.946	0.938
0.936	0.691	0.939	0.939	0.958	0.997	0.922	0.939	0.949	0.923	0.961	0.936	.	0.928	0.935	0.951	0.869	0.925	0.936
0.947	0.706	0.923	0.921	0.949	0.968	0.946	0.915	0.93	0.877	0.952	0.954	.	0.932	0.954	0.974	0.932	0.965	0.961
0.961	0.686	0.925	0.943	0.968	0.975	0.925	0.942	0.936	0.907	0.91	0.936	.	0.934	0.962	0.974	0.889	0.951	0.958
0.947	0.67	0.927	0.923	0.956	0.971	0.938	0.932	0.954	0.933	0.939	0.933	.	0.925	0.95	0.951	0.847	0.948	0.971
1.014	0.623	0.941	0.964	0.951	0.975	0.976	0.96	0.959	0.947	0.928	0.906	.	0.953	0.946	0.953	0.907	0.968	0.97
0.966	0.68	0.934	0.943	0.979	0.973	0.936	0.95	0.953	0.95	0.937	0.978	.	0.923	0.959	0.944	0.914	0.961	0.963
0.956	0.694	0.953	0.938	1.002	0.986	0.919	0.948	0.964	0.958	0.905	0.942	.	0.925	0.97	0.98	0.933	0.995	0.957
0.971	0.677	0.944	0.959	0.994	1.004	0.929	0.911	0.933	0.987	0.946	0.955	.	0.957	0.943	0.997	0.922	0.998	0.977
0.948	0.734	0.941	0.955	0.946	0.988	0.932	0.937	0.943	0.98	0.907	0.966	.	0.937	0.952	0.964	0.888	0.974	0.957
0.974	0.819	0.934	0.96	0.992	1.001	0.958	0.96	0.94	0.943	0.861	0.961	.	0.945	0.939	0.956	0.927	0.932	0.961
0.922	0.83	0.955	0.972	0.973	0.992	0.961	0.966	0.954	0.998	0.861	0.95	.	0.946	0.926	0.924	0.922	0.991	0.993
0.944	0.832	0.95	0.939	0.987	0.971	0.974	0.978	0.929	0.965	0.875	0.933	.	0.942	0.917	0.935	0.903	0.971	0.988
0.932	0.833	0.939	0.944	0.989	0.992	0.956	0.998	0.934	0.953	0.938	0.99	.	0.98	0.927	0.916	0.927	0.968	0.973
0.938	0.838	0.943	0.958	0.979	0.967	0.957	0.971	0.922	0.937	0.903	0.947	.	1.001	0.921	0.929	0.892	0.972	0.935
0.974	0.833	0.941	0.979	1.005	0.972	0.937	0.952	0.913	0.975	0.919	0.94	.	0.962	0.945	0.92	0.919	0.979	0.965
0.947	0.866	0.927	0.974	1.011	0.976	1.022	0.958	0.911	0.976	0.889	0.964	.	0.989	0.933	0.934	0.97	0.951	0.937
0.936	0.887	0.942	0.969	1.008	0.978	0.967	0.953	0.907	0.935	0.882	0.976	.	0.981	0.961	0.909	0.948	0.957	0.934
0.94	0.853	0.928	0.986	1.01	0.982	0.968	0.945	0.877	0.974	0.965	0.93	.	0.971	0.959	0.914	0.993	0.953	0.947
0.975	0.874	0.953	1.006	1.012	0.985	0.94	0.932	0.854	0.984	0.945	0.909	.	0.993	0.958	0.861	0.9	0.943	0.959
0.935	0.9	0.938	0.971	1.048	0.984	0.944	0.953	0.866	0.961	0.909	0.949	.	0.996	0.969	0.91	0.93	0.96	0.93
0.942	0.89	0.945	0.978	1.034	0.962	0.942	0.924	0.902	0.967	0.861	0.933	.	0.942	0.979	0.868	0.94	0.962	0.971
0.957	0.906	0.961	0.97	1.036	1.017	0.933	0.915	0.899	0.948	0.892	0.882	.	0.995	1.006	0.9	0.957	1.009	0.948
0.952	0.868	0.942	0.979	1.029	1.017	0.967	0.944	0.919	0.953	0.839	0.892	.	0.981	0.987	0.872	0.985	0.985	0.969
0.971	0.919	0.951	0.974	1.008	1.009	0.931	0.977	0.945	0.9	0.834	0.838	.	0.988	0.96	0.938	0.967	0.985	0.961
0.951	0.915	0.944	0.951	1.042	0.984	0.926	0.984	0.971	0.944	0.839	0.881	.	0.968	0.943	0.925	0.908	1.008	1.013

0.95	0.916	0.975	0.981	1.022	1.01	0.972	0.972	0.962	0.957	0.811	0.925 .	0.974	0.946	0.925	0.948	1.006	1
0.957	0.888	0.953	0.983	0.996	0.978	0.917	0.993	0.944	0.914	0.829	0.911 .	0.971	0.93	0.905	0.926	0.975	0.963
0.908	0.846	0.951	0.99	1.04	0.984	0.929	0.978	0.948	0.942	0.871	0.877 .	0.963	0.938	0.898	0.946	0.994	0.963
0.881	0.914	0.961	0.955	1.011	0.953	0.953	0.943	0.944	0.957	0.836	0.893 .	0.962	0.942	0.925	0.93	0.975	0.97
0.924	0.932	0.943	0.984	1.011	0.99	0.967	0.933	0.926	0.969	0.819	0.88 .	0.978	0.951	0.939	0.96	0.981	0.96
0.894	0.889	0.944	0.997	0.989	0.972	0.969	0.928	0.981	0.992	0.825	0.809 .	0.95	0.977	0.926	0.969	0.987	0.955
0.842	0.927	0.962	0.95	0.974	0.947	0.915	0.969	0.958	0.906	0.733	0.91 .	0.916	1.006	0.947	0.929	0.947	0.906
0.784	0.899	0.94	0.951	0.963	0.942	0.887	0.954	0.939	0.913	0.844	0.932 .	0.916	1.011	0.951	0.917	0.981	0.96
0.755	0.882	0.942	0.921	0.988	0.977	0.93	0.952	0.952	0.966	0.891	0.937 .	0.857	0.979	0.927	0.96	0.994	0.988
0.767	0.907	0.927	0.942	0.965	0.937	0.899	0.876	0.986	0.967	0.812	0.842 .	0.883	0.95	0.901	0.933	0.976	0.973
0.755	0.902	0.908	0.875	0.974	0.968	0.938	0.889	0.962	0.896	0.815	0.876 .	0.912	0.948	0.929	0.954	0.96	0.937
0.798	0.883	0.885	0.915	0.975	0.929	0.915	0.823	0.988	0.926	0.876	0.798 .	0.836	0.939	0.942	0.919	0.933	0.948
0.781	0.887	0.909	0.891	0.96	0.929	0.947	0.863	0.98	0.967	0.854	0.877 .	0.819	0.884	0.848	0.931	0.951	1
0.769	0.918	0.91	0.903	0.993	0.982	0.922	0.877	0.987	0.935	0.787	0.975 .	0.904	0.894	0.889	0.953	0.94	0.97
0.749	0.928	0.875	0.825	0.939	0.919	0.908	0.844	0.956	0.951	0.804	0.92 .	0.826	0.889	0.86	0.932	0.992	0.949
0.715	0.878	0.857	0.841	0.951	0.929	0.929	0.814	0.954	0.926	0.853	0.949 .	0.878	0.888	0.824	0.931	0.954	0.979
0.776	0.913	0.936	0.874	0.915	0.898	0.898	0.876	0.976	0.935	0.822	0.945 .	0.934	0.923	0.925	1.018	0.974	0.979
0.704	0.907	0.94	0.876	0.899	0.865	0.874	0.872	0.947	0.952	0.862	0.932 .	0.849	0.856	0.893	0.974	0.92	0.968
0.725	0.89	0.924	0.805	0.916	0.843	0.856	0.84	0.928	0.993	0.887	0.906 .	0.819	0.859	0.887	0.962	0.923	1.023
0.715	0.885	0.94	0.821	0.863	0.798	0.934	0.86	0.897	0.953		0.868 .	0.847	0.833	0.909	0.953	0.912	0.965
0.705	0.872	0.92	0.905	0.881	0.828	0.93	0.901	0.906	0.974		0.882 .	0.844	0.844	0.915	0.966	0.941	0.995
0.714	0.921	0.878	0.931	0.863	0.809	0.909	0.865	0.879	0.944		0.835 .	0.886	0.822	0.892	0.947	0.943	0.969
0.795	0.885	0.915	0.919	0.932	0.795	0.871	0.864	0.893	0.959		0.812 .	0.804	0.812	0.927	0.953	0.895	0.91
0.834	0.853	0.925	0.914	0.909	0.839	0.908	0.835	0.959	0.975		0.791 .	0.82	0.773	0.885	0.957	0.925	0.976
0.853	0.869	0.952	0.937	0.876	0.877	0.83	0.907	0.916	0.954		0.911 .	0.763	0.799	0.909	0.958	0.909	0.956
0.854	0.899	0.896	0.89	0.865	0.826	0.842	0.93	0.967	0.932		0.93 .	0.745	0.82	0.973	0.952	0.941	0.943
0.876	0.888	0.959	0.965	0.958	0.837	0.913	0.896	0.956	0.876		0.883 .	0.857	0.777	0.955	0.954	0.947	0.938
0.91	0.904	0.907	0.971	0.947	0.877	0.949	0.87	0.971	0.852		0.81 .	0.899	0.729	0.934	0.951	0.967	0.909
0.911	0.878	0.928	0.977	0.898	0.941	0.978	0.835	0.937	0.847		0.831 .	0.854	0.785	0.921	0.984	0.932	0.925
0.952	0.906	0.864	0.96	0.873	0.915	0.967	0.901	0.98	0.863		0.933 .	0.858	0.757	0.866	0.995	0.952	0.94
0.903	0.901	0.872	0.976	0.865	0.945	0.947	0.943	0.998	0.904		0.92 .	0.843	0.749	0.906	0.959	0.983	0.965
0.927	0.888	0.892	1.007	0.905	0.925	0.933	0.919	0.967	0.923		0.864 .	0.904	0.763	0.934	0.954	0.963	0.962
0.938	0.923	0.941	1.01	0.986	0.954	0.941	0.922	0.969	0.952		0.84 .	0.907	0.835	0.933	0.996	0.954	0.916
0.962	0.912	0.968	0.995	0.994	0.972	0.917	0.893	0.965	0.939		0.899 .	0.857	0.897	0.952	0.988	0.943	0.9
0.947	0.907	0.938	0.993	0.948	0.985	0.925	0.953	0.924	0.956		0.951 .	0.874	0.928	0.937	0.938	0.955	0.908
0.948	0.926	0.899	0.973	0.973	0.984	0.955	0.953	0.911	0.938		0.878 .	0.927	0.958	0.911	0.963	0.959	0.934
0.933	0.91	0.927	0.978	1.001	0.971	0.99	0.961	0.924	0.972		0.837 .	0.949	0.93	0.929	0.953	0.968	0.91
0.97	0.968	0.891	0.975	0.984	0.979	0.971	0.89	0.912	0.918		0.872 .	0.924	0.948	0.941	0.963	0.982	0.857
0.961	0.926	0.899	0.966	0.977	0.982	0.972	0.88	0.932	0.903		0.914 .	0.945	0.931	0.93	0.94	0.987	0.846
0.934	0.901	0.899	0.959	0.973	0.966	0.964	0.921	0.942	0.952		0.897 .	0.954	0.942	0.915	0.937	0.967	0.905
0.961	0.925	0.903	0.976	0.972	0.975	0.974	0.92	0.936	0.959		0.833 .	0.944	0.95	0.931	0.963	0.965	0.875
0.944	0.914	0.928	0.972	0.975	0.976	0.968	0.96	0.935	0.941		0.939 .	0.945	0.915	0.936	0.953	0.988	0.85
0.944	0.9	0.938	0.974	0.981	0.944	0.965	0.961	0.918	0.974		0.955 .	0.951	0.932	0.928	0.952	0.987	0.908
0.963	0.935	0.948	0.99	0.974	0.98	0.994	0.973	0.907	0.971		0.927 .	0.989	0.962	0.925	0.952	0.995	0.91
0.94	0.918	0.939	0.976	0.993	0.957	0.989	0.931	0.882	0.988		0.861 .	0.932	0.922	0.903	0.955	0.975	0.928
0.995	0.912	0.891	1.002	0.954	0.965	0.972	0.891	0.831	0.974		0.864 .	0.944	0.946	0.97	0.97	0.982	0.923
0.944	0.93	0.942	0.988	0.942	0.982	0.965	0.95	0.918	0.977		0.953 .	0.945	0.981	0.929	0.935	0.978	0.884
0.986	0.967	0.943	0.978	0.989	0.982	0.979	0.939	0.939	0.964		0.902 .	0.952	0.985	0.957	0.967	0.973	0.861
0.958	0.917	0.935	0.974	0.981	0.969	0.962	0.903	0.933	0.946		0.899 .	0.952	0.954	0.907	0.991	0.976	0.828
0.944	0.962	0.929	0.983	0.98	0.992	0.973	0.941	0.89	0.919		0.822 .	0.974	0.981	0.921	1.004	0.957	0.791
0.923	0.934	0.939	0.963	0.979	0.977	0.986	0.935	0.881	0.937		0.779 .	0.937	0.966	0.932	0.99	1.006	0.901
0.926	0.93	0.939	0.967	1	0.993	1.027	0.919	0.833	0.898		0.819 .	0.951	0.953	0.936	0.996	0.967	0.912
0.914	0.918	0.935	0.981	0.995	0.977	0.979	0.874	0.847	0.874		0.916 .	0.95	0.978	0.988	0.954	1.002	0.894
0.912	0.932	0.91	0.981	0.97	0.979	0.986	0.867	0.909	0.823		0.887 .	0.939	0.946	0.929	1.001	0.982	0.931
0.86	0.889	0.944	1.016	1.001	0.996	0.946	0.899	0.925	0.836		0.893 .	0.95	0.941	0.895	0.968	1.018	0.954
0.937	0.958	0.957	0.984	0.988	0.988	0.939	0.871	0.927	0.844		0.809 .	0.894	0.954	0.955	0.952	0.97	0.921
0.891	0.943	0.917	0.983	0.988	0.978	0.951	0.87	0.93	0.922		0.839 .	0.936	0.936	0.872	0.978	1.03	0.886
0.893	0.95	0.902	0.962	0.955	1.013	0.972	0.87	0.937	0.898		0.767 .	0.928	0.932	0.896	0.968	0.992	0.821
0.875	0.939	0.879	0.929	1	0.997	0.94	0.893	0.956	0.878		0.801 .	0.906	0.953	0.926	0.993	1.007	0.845
0.932	0.968	0.894	0.93	1.026	1.028	0.961	0.883	0.947	0.864		0.799 .	0.911	0.956	0.912	0.961	0.964	0.824
0.973	0.981	0.941	0.923	1.017	0.982	0.98	0.891	0.936	0.854		0.902 .	0.903	0.963	0.925	0.98	0.982	0.821
0.908	0.949	0.883	0.896	0.95	1.013	0.935	0.879	0.963	0.889		0.881 .	0.983	0.933	0.939	0.937	0.989	0.912
0.928	0.97	0.882	0.901	0.935	0.995	0.966	0.868	0.937	0.907		0.834 .	0.931	0.958	0.927	0.962	0.955	0.925
0.934	0.928	0.87	0.873	0.923	1.02	0.933	0.813	0.939	0.858		0.801 .	0.911	0.929	0.855	0.913	0.945	0.899
0.903	0.955	0.831	0.894	0.864	0.98	1.004	0.859	0.921	0.839		0.808 .	0.972	0.989	0.927	0.971	0.897	0.87
0.886	0.945	0.853	0.939	0.884	1.002	0.925	0.865	0.914	0.866		0.743 .	0.94	0.955	0.952	0.972	0.937	0.926
0.784	0.935	0.819	0.929	0.863	1.012	0.89	0.809	0.922	0.897		0.762 .	0.939	0.92	0.895	1.007	0.897	0.935
0.828	0.92	0.761	0.884	0.898	0.994	0.866	0.895	0.947	0.943		0.786 .	0.927	0.918	0.883	0.953	0.962	0.931
0.841	0.97	0.792	0.942	0.969	1.015	0.864	0.899	0.926	0.951		0.904 .	0.925	0.871	0.923	1	0.953	0.927
0.914	0.916	0.88	0.941	0.999	0.987	0.884	0.873	0.902	0.953		0.881 .	0.969	0.881	0.836	0.96	0.93	0.925
0.908	0.961	0.885	0.957	0.977	1.008	0.846	0.811	0.84	0.942		0.834 .	0.918	0.915	0.787	0.948	0.883	0.889
0.849	0.922	0.883	0.934	0.929	0.966	0.832	0.932	0.832	0.965		0.809 .	0.926	0.936	0.824	0.963	0.9	0.933
0.854	0.958	0.881	0.912	0.941	0.967	0.826	0.899	0.874	0.94		0.799 .	0.913	0.912	0.79	0.909	0.942	0.956
0.854	0.989	0.869	0.9	0.998	0.974	0.937	0.877	0.92	0.94		0.864 .	0.925	0.925	0.878	0.935	0.958	0.977
0.847	0.963	0.927	0.952	1.014	0.89	0.91	0.865	0.923	0.943		0.844 .	0.921	0.944	0.916	0.997	0.918	0.935
0.807	0.952	0.919	0.975	0.98	0.907	0.95	0.893	0.9	0.958		0.918 .	0.869	0.929	0.91	0.977	0.867	0.936
0.802	0.983	0.9	0.923	0.909	0.892	0.948	0.85	0.845	0.921		0.896 .	0.858	0.954	0.899	1	0.938	0.94
0.767	0.949	0.891	0.873	0.917	0.881	0.878	0.841	0.838	0.929		0.903 .	0.81	0.925	0.878	0.953	0.933	0.883
0.791	0.949	0.913	0.944	0.894	0.94	0.839	0.82	0.885	0.953		0.857 .	0.821	0.881	0.84	0.889	0.883	0.876
0.898	0.925	0.912	0.935	0.87	0.956	0.821	0.785	0.922	0.99		0.864 .	0.789	0.907	0.846	0.883	0.94	0.921
0.902	0.947	0.938	0.966	0.95	0.916	0.843	0.82	0.94	0.939		0.792 .	0.731	0.936	0.915	0.934	0.938	0.94
0.897	0.914	0.934	0.983	1.03	0.911	0.798	0.884	0.893	0.954		0.884 .	0.713	0.907	0.901	0.989	0.971	0.95
0.909	0.932	0.996	1.008	1.085	0.957	0.964	0.86	0.978	0.989	0.932	1.036 .	0.995	0.834	0.957	1.03	0.945	0.98
0.9	0.901	0.973	1.037	1.067	1.04	0.999	0.929	0.97	1	0.917	0.994 .	0.997	0.84	1.002	1.012	0.972	1.007
0.899	0.943	1.009	1.052	1.085	1.072	1.019	0.957	0.972	1.009	0.929	1.001 .	0.993	0.819	1.028	0.988	0.996	0.997
0.931	0.972	1.026	1.03	1.059	1.023	0.999	0.991	0.985	1	0.983	0.992 .	1.001	0.794	1.019	1.028	1.057	1.011
0.943	0.963	1.034	1.044	1.096	1.053	1.035	0.989	1.04	1.015	0.958	0.992 .	1.019	0.818	1.022	0.995	1.043	1.112
0.996	0.988	1.003	1.035	1.109	1.064	0.995	1.03	0.981	1.004	0.952	0.998 .	1.052	0.952	1.026	1.013	1.001	1.009
1.021	1.029	1.016	1.034	1.035	1.031	1.025	1.017	1.003	1.057	0.953	0.958 .	1.046	0.981	0.999	1.041	1.002	1.022
0.991	0.974	1.021	1.057	1.101	1.067	1.072	1.021	0.997	1.024	0.946	0.944 .	1.013	0.984	0.998	1.021	1.022	1.017
1	0.98	1.012	1.061	1.106	1.054	1.021	1.026	0.986	1.02	0.941	0.976 .	1.007	1.011	0.997	1.025	0.997	1.009
1.021	1.03	0.992	1.036	1.045	1.056	1.052	1.055	1.006	1.043	0.95	0.976 .	1.021	1.038	1.04	1.022	1.035	1.048
0.997	1.009	0.999	1.045	1.104	1.035	1.039	1.024	0.995	1.035	0.932	0.951 .	1.058	1.007	1.016	1.041	1.039	0.985
0.995	0.988	1.009	1.078	1.079	1.051	1.029	1.014	0.977	1.006	0.924	0.978 .	1.021	0.984	1.001	1.048	1.023	0.992
1.016	0.99	1.006	1.031	1.073	1.053	1.068	1	1.013	1.04	0.986	0.975 .	1.021	1.077	1.009	1.028	1.042	1.047
1.014	0.981	1.023	1.035	1.089	1.052	1.028	1.02	0.966	1.012	0.959	0.974 .	1.057	1.024	0.977	1.017	1.018	1.008
1.024	1.01	1.001	1.042	1.094	1.043	1.008	1.004	0.966	1.016	0.978	0.947 .	1.042	1.009	0.987	1.015	1.009	0.996
0.998	1.015	0.976	1.062	1.055	1.044	1.022	1.014	0.934	1.027	1.01	0.973 .	1.002	1.016	0.992	0.998	1.019	1.014
1.003	0.986	1.012	1.02	1.064	1.053	1.013	1.035	0.979	1.035	1.005	1.014 .	0.988	1.006	1.028	0.988	1.03	1.018
0.996	0.979	0.987	1.012	1.09	1.047	1.034	1.04	0.969	1.034	0.976	1.019 .	0.996	1.007	0.996	1.007	1.016	0.988
0.985	0.987	1.014	1.028	1.074	1.065	1.03	0.968	0.975	1.043	0.971	0.969 .	0.986	0.973	0.992	1.009	1.013	1.012
1.006	0.982	1.026	1.066	1.087	1.048	1.049	1.039	0.984	1.015	0.978	1.054 .	0.978	1.015	0.991	1	1.017	1.028
1.011	1.021	1.002	1.04	1.076	1.062	1.054	1.012	0.999	1.033	0.988	1.043 .	0.989	1.022	1.017	1.001	1.011	1.008
0.984	0.995	1.007	1.023	1.044	1.027	0.99	0.999	0.98	1.022	0.961	0.997 .	1.016	1.004	1.026	1.016	1.031	1.004

1.031	1.006	1.03	1.067	1.092	1.051	1.052	1.02	1.007	1.033	0.969	0.995 .	1.037	1.044	1.038	0.987	1.044	1.01
1.024	1.011	1.008	1.038	1.066	1.038	1.046	1.026	1	1.055	0.965	1.029 .	1.036	1.009	1.048	1.058	1.046	1.018
1.038	1.005	1.016	1.03	1.073	1.018	1.071	1.026	1.024	1.033	0.978	1.009 .	1.004	1.017	1.072	1.024	1.049	1.015
1.053	1.035	1.032	1.061	1.063	1.052	1.061	1.065	1.019	1.032	0.931	0.996 .	1.035	1.033	1.008	1.022	1.061	1.016
1.002	1.011	1.023	1.041	1.077	1.054	1.071	1.057	1.011	1.023	0.961	1.011 .	0.991	1.028	1.013	1.006	1.031	1.026
1.018	1.015	1.015	1.064	1.066	1.086	1.05	1.083	0.982	1.06	1.014	1.005 .	1.029	1.016	1.047	1.02	1.013	0.98
1.032	1	1.009	1.042	1.045	1.053	1.078	1.06	1.057	1.049	0.965	0.988 .	1.022	1.033	0.997	1.011	1.029	0.996
1.004	0.96	1.029	1.057	1.066	1.055	1.068	1.043	1.043	1.042	0.984	0.977 .	1.013	1.027	1.002	1.019	1.047	1.064
1.031	1.019	1.042	1.064	1.063	1.039	1.067	1.048	1.002	1.007	0.965	0.99 .	1.021	1.049	0.988	1.024	1.047	0.997
1.023	0.989	1.017	1.085	1.074	1.069	1.081	1.091	1.011	1.018	0.959	0.995 .	1.012	1.053	1.029	1.05	1.062	0.981
1.009	1.007	1.012	1.058	1.102	1.061	1.078	1.066	0.953	1.021	0.946	1.015 .	1.038	1.039	0.984	0.991	1.069	0.999
1.013	1.016	0.998	1.063	1.09	1.061	1.085	1.053	0.973	1.046	0.97	1.018 .	1.014	1.01	1.007	1.015	1.096	0.97
1.039	1.022	1.024	1.054	1.079	1.067	1.06	1.048	1.015	1.076	0.984	1.045 .	1.028	1.035	1.041	1.034	1.062	0.916
0.969	0.998	1.041	1.072	1.083	1.067	1.12	1.034	0.998	1.06	0.946	1.054 .	0.98	1.06	1.016	1.007	1.011	0.885
0.956	1.017	1.028	1.067	1.089	1.078	1.047	1.04	0.969	1.003	0.933	0.97 .	0.994	1.055	0.997	1.051	1.052	0.876
0.933	1.016	1.033	1.077	1.054	1.093	1.053	1.037	0.993	1.042	0.997	1 .	0.978	1.016	1.02	1.038	1.056	0.847
1	1.043	1.014	1.055	1.075	1.057	1.081	1.021	0.979	1.096	1.021	1.021 .	1.036	1.034	1.014	1.036	0.967	0.917
0.959	1.008	1.01	1.042	1.064	1.076	1.055	1.015	0.997	1.022	1.048	1.036 .	1.001	1.038	1.025	1.025	1.001	0.984
0.938	1.002	1.018	0.99	1.08	1.07	1.053	0.995	0.992	1.069	1.032	1.044 .	0.922	1.031	1.019	1.04	0.996	0.973
0.923	1.017	1.008	1.004	1.039	1.074	0.991	1.058	0.961	1.014	0.992	1.034 .	1	1.04	1.015	1.028	0.956	0.991
0.852	1.007	1.029	0.965	1.102	1.087	0.965	1.037	0.94	0.962	0.96	1.053 .	0.951	1.029	1.02	0.98	0.89	0.946
0.855	1.04	1.004	0.926	1.081	1.098	0.928	1.043	0.903	0.965	0.952	1.074 .	0.918	1.029	1.008	0.967	0.844	0.986
0.804	1.043	1.049	0.906	1.075	1.111	0.9	1.076	0.927	1.007	0.911	1.025 .	0.908	1.052	0.975	0.979	0.998	0.943
0.78	1.033	1.026	0.98	1.075	1.112	0.878	1.075	0.965	1.036	0.979	1.053 .	0.95	1.063	0.988	0.931	1.01	0.987
0.829	0.99	1.031	1.001	1.098	1.1	0.951	1.036	0.979	1.011	1.017	1.086 .	0.844	1.008	1.005	0.882	1.009	0.954
0.82	1.004	1.023	0.973	1.066	1.092	0.935	1.038	0.918	1.017	1.031	1.036 .	0.884	1.012	1.021	0.846	1.023	0.92
0.793	0.991	0.983	0.995	1.008	1.092	0.878	1.067	0.864	1.061	0.994	1.041 .	0.951	1.036	1.051	0.823	0.987	0.901
0.978	0.993	0.943	1.036	0.984	1.075	0.948	1.02	0.832	1.031	0.934	1.056 .	0.894	1.049	1.017	0.952	0.969	0.948
0.913	0.945	0.98	0.954	1.03	1.074	0.988	1.022	0.936	1.037	0.941	1.007 .	0.864	1.041	0.974	0.928	1.034	1.003
0.878	0.993	1.004	0.87	1.112	1.093	0.927	1.031	0.956	0.994	1.013	0.964 .	0.992	0.977	1.036	0.927	1.053	1.047
0.949	0.968	1.018	0.864	1.111	1.102	0.954	0.974	0.912	1.041	0.957	0.982 .	1.004	0.971	0.958	0.973	1.021	0.981
0.903	1	1.035	0.969	1.097	1.035	0.948	0.932	0.877	1.046	0.9	0.953 .	1.02	0.933	0.976	0.975	1.054	0.965
0.985	1.033	1.048	1.029	1.083	0.998	0.892	0.932	0.852	0.979	0.979	0.918 .	0.991	0.908	1.013	0.938	1.044	0.97
0.971	1.021	1.045	0.977	1.058	0.991	0.819	0.99	0.894	1.028	0.919	0.948 .	0.929	0.894	0.989	0.988	1.045	0.972
1.012	1.053	1.034	0.995	1.118	1.091	0.921	1.009	0.867	0.993	0.91	0.897 .	0.968	0.997	0.999	0.954	1.067	1.02
1.007	1.017	1.032	1.021	1.054	1.004	0.951	0.973	0.808	0.968	0.896	0.873 .	0.968	0.987	1.001	0.97	1.046	1.007
0.978	1.03	0.978	1.004	1.051	0.968	1.015	0.929	0.756	0.979	0.848	0.792 .	0.941	0.994	1.03	1.005	1.039	1.013
0.987	0.986	1.047	0.903	1.082	1.049	0.985	0.979	0.802	1.078	0.907	0.803 .	0.957	0.972	1.027	0.944	1.051	1.019
0.983	0.992	1.074	1	1.083	1.001	0.961	0.929	0.851	1.044	0.929	0.81 .	0.928	1.003	1.072	0.9	1.038	1.003
0.914	0.997	1.033	0.986	1.064	0.976	1.022	0.954	0.954	1.081	0.938	0.824 .	0.881	0.972	1.029	0.952	1.079	0.961
0.951	0.981	1.078	1.046	1.07	1.032	1.093	0.975	0.942	1.099	0.892	0.901 .	0.857	0.922	0.988	1.067	1.075	1
1.005	0.975	1.068	1.031	1.027	1.04	1.047	1.014	0.919	1.026	0.851	0.933 .	0.813	0.913	1.042	1.012	1.071	1.06
1.009	0.944	1.092	1.03	1.068	1.022	1.014	1.008	0.898	1.097	0.849	0.886 .	0.85	0.931	1.027	0.986	1.065	0.978
0.987	0.998	1.102	1.012	1.085	0.946	1.051	1.054	0.98	1.111	0.918	0.833 .	0.824	0.97	1.052	1.049	1.034	0.965
0.952	1.016	1.064	1.035	1.076	0.906	1.109	1.019	1.006	1.064	0.846	0.889 .	0.796	0.943	1.068	1.029	1.027	0.909
0.991	1.009	1.051	0.959	1.051	0.948	1.062	1.001	1.006	1.035	0.937	0.869 .	0.794	1.026	1.011	0.94	1.029	0.96
1.022	0.972	1.049	1.056	1.067	0.904	1.091	1.037	0.927	1.027	0.884	1 .	0.783	0.977	1.032	0.887	1.044	1.022
1.047	0.973	1.087	1.079	1.129	1.025	1.114	1.033	0.903	0.991	0.824	1.003 .	0.849	0.964	1.017	1.001	1.013	1.015
0.989	1.005	1.088	1.099	1.088	0.95	1.058	0.978	0.999	1.008	0.81	1.008 .	0.854	1.005	1.007	1.054	0.995	1.003
1.04	1.054	1.045	1.095	1.037	0.921	1.065	0.931	1.022	1.028	0.8	0.967 .	0.789	1.086	1.026	1.07	0.977	0.967
1.054	1.071	1.056	1.097	1.04	1.013	1.056	0.9	0.992	1.033	0.869	0.974 .	0.81	1.043	1.013	1.082	0.959	0.941
1.051	1.062	1.046	1.131	1.037	0.965	1.028	0.947	0.938	1.016	0.864	1.022 .	0.844	1.043	1.003	1.065	1.013	0.906
1.015	0.985	0.987	1.094	1.104	1.042	1.081	1.022	0.974	1.05	0.913	1.018 .	0.746	1.061	0.991	1.038	1.001	0.989
1.014	1.029	0.944	1.098	1.107	1.086	1.112	1.026	1.021	1.048	0.829	1.041 .	0.676	1.077	1.006	1.081	0.995	1.008
1.009	1.093	0.995	1.108	1.069	1.028	1.08	1.038	1.008	1.025	0.899	1.008 .	0.748	1.088	0.963	1.123	0.993	0.984
1.06	1.056	1.034	1.107	1.093	1.035	1.091	1.048	1	0.962	0.959	1.061 .	0.853	1.072	1.022	1.094	1.022	0.99
0.98	1.003	1.067	1.106	1.102	1.014	1.059	1.062	0.974	0.952	0.872	1.047 .	0.73	1.016	0.915	1.017	1.004	1.002
1.011	1.024	1.044	1.072	1.078	1.021	1.061	1.089	0.925	0.844	0.83	1.02 .	0.753	1.055	0.874	0.981	0.985	0.963
1.05	1.002	1.065	1.063	1.097	1.067	1.074	1.054	0.849	0.841	0.931	1.031 .	0.805	1.016	0.817	0.991	1.016	0.962
0.997	1.022	1.061	1.053	1.105	1.091	1.055	1.068	0.87	0.942	0.918	1.042 .	0.782	1.045	0.809	0.996	1.031	0.912
1.001	1.026	1.061	1.046	1.065	1.117	1.076	1.044	0.959	0.977	1.005	1.056 .	0.736	1.07	0.933	0.988	0.966	0.868
0.959	1.058	1.032	1.016	1.05	1.102	1.097	1.057	0.982	0.988	1.029	1.066 .	0.886	1.069	0.999	1.033	0.912	0.938
0.978	1.092	1.047	1.039	0.996	1.09	1.113	1.05	0.964	0.972	1.011	1.052 .	0.842	1.064	1.015	1.022	0.942	0.923
1.003	1.058	1.051	1.05	1.014	1.103	1.114	1.049	0.883	0.999	0.995	1.049 .	0.756	1.052	0.976	1.056	0.997	0.869
1.018	1.037	1.042	1	0.994	1.069	1.077	0.996	0.817	1.043	0.922	1.058 .	0.764	1.074	1.022	1.055	1.02	0.924
0.98	1.045	1.011	1.006	0.924	1.085	1.118	1.006	0.919	1.066	0.847	1.016 .	0.731	1.057	1.002	1.069	1.087	0.985
1.058	1.071	0.992	0.977	0.843	1.078	1.095	1.046	0.973	1.015	0.916	1.059 .	0.718	1.05	0.995	1.067	1.083	0.953
1.045	1.061	1.014	0.922	0.839	1.075	1.095	1.058	0.986	1.005	0.892	0.926 .	0.718	1.059	1.012	1.096	1.062	0.955
1.024	1.008	1.017	0.891	0.988	1.098	1.055	1.023	0.974	1.027	0.87	0.964 .	0.671	1.047	0.979	1.058	0.984	0.863
0.988	1.012	1.052	1.003	1.063	1.081	1.066	1.04 .		1.054	0.939	0.951 .	0.838	1.054	0.923	1.059	1.006	0.991
0.979	1.015	1.047	0.982	1.027	1.083	1.047	1.043 .		1.088	0.988	0.974 .	0.815	1.026	0.983	1.04	0.965	0.99
1.011	1.011	1.06	0.981	0.974	1.096	1.037	1.112 .		1.022	1.029	0.937 .	0.729	1.053	0.97	1.035	0.962	0.958
0.973	1.002	1.042	1.007	1.049	1.127	1.039	1.13 .		1.022	0.981	0.868 .	0.728	1.042	0.946	1.03	0.954	0.921
0.935	0.994	0.986	1.028	1.061	1.103	1.009	1.048 .		1.024	0.873	0.886 .	0.882	1.07	1.059	1.024	0.885	0.925
0.941	0.961	0.942	0.992	1.096	1.06	0.994	1.041 .		1.036	1.002	0.942 .	0.859	1.022	1.017	0.996	0.859	0.969
0.927	1.013	1.012	1.025	1.042	1.082	0.948	0.999 .		1.073	1.018	0.929 .	0.822	1.04	1.016	1.035	0.92	0.927
0.867	0.978	1.034	1.003	0.96	1.09	0.911	0.975 .		1.065	0.999	0.877 .	0.741	0.993	0.971	1.082	0.845	0.881
0.889	0.956	1.016	0.954	0.985	1.095	1.018	1.025 .		1.054	0.949	0.883 .	0.745	0.953	0.993	1.017	0.817	0.868
0.914	1.01	1.002	0.92	0.961	1.087	1.053	0.981 .		**0.967**	0.879	0.935 .	0.761	0.931	1.011	1.028	**0.727**	**0.851**
0.99	1.026	0.959	0.886	0.964	1.027	1.043	0.95 .		**0.956**	0.831	0.94 .	0.756	0.976	1.002	0.987	**0.742**	**0.899**
0.947	1.051	0.871	0.842	0.934	1.039	1.075	0.919 .		**0.949**	0.78	0.879 .	0.747	0.902	0.975	0.939	**0.75**	**0.861**
0.915	1.04	0.996	0.823	0.895	1.018	1.042	0.998 .		**0.913**	0.797	0.843 .	0.761	0.909	0.946	1.036	**0.758**	**0.79**
0.879	1.057	0.945	0.801	0.855	0.979	1.001	0.987 .		**0.844**	0.989	0.832 .	0.749	0.846	1.002	1.035	**0.731**	**0.768**
0.942	1.071	0.874	0.79	0.831	0.942	1.019	0.93 .		**0.933**	0.986	0.854 .	0.699	0.806	0.955	0.987	**0.731**	**0.747**
0.918	1.114	0.836	0.782	0.822	0.976	1.015	0.873 .		**0.961**	1.014	0.827 .	0.692	0.773	0.951	0.963	**0.756**	**0.739**
0.862	1.05	0.92	0.85	0.82	0.916	0.971	0.874 .		**0.907**	0.993	0.773 .	0.698	0.767	0.822	0.936	**0.799**	**0.742**
0.809	1.038	0.978	0.824	0.808	0.858	0.895	0.843 .		0.863	0.977	0.764 .	0.744	0.746	0.839	0.904	0.961	0.76
0.775	1.078	0.976	0.842	0.825	0.919	0.873	0.827 .		0.841	0.928	0.738 .	0.833	0.747	0.807	0.861	0.961	0.753
0.777	1.104	0.911	0.806	0.866	0.884	0.854	0.785 .		0.81	0.973	0.759 .	0.834	0.757	0.899	0.795	1.014	0.899
0.82	1.082	0.916	0.808	0.96	1.015	0.957	0.935 .		0.838	1.009	0.862 .	0.76	0.747	0.905	0.779	0.988	0.793
0.785	1.07	0.895	0.785	0.929	0.882	1.057	1.026 .		0.846	0.902	0.903 .	0.675	0.739	0.822	0.744	0.94	0.737
0.739	1.059	0.82	0.822	0.866	0.83	1.036	1.045 .		0.998	0.821	1.041 .	0.724	0.849	0.814	0.945	0.882	0.751
0.726	1.025	0.916	0.887	0.889	0.994	0.946	1.022 .		1.059	0.716	1.002 .	0.842	0.839	0.794	0.987	1.026	0.833
0.747	1.04	0.997	0.879	0.899	0.988	0.955	1.068 .		1.04	0.735	0.852 .	0.767	0.756	0.815	0.989	1.016	0.841
0.953	1.006	0.93	0.82	0.989	0.922	0.884	0.93 .		0.984	0.77	0.859 .	0.707	0.776	0.756	0.991	0.976	0.962
0.866	0.933	0.972	0.906	1.023	0.906	0.983	0.947 .		0.972	0.753	0.892 .	0.841	0.968	0.839	0.975	1.021	0.876
1.049	0.929	1.081	0.936	0.981	1.078	1.057	0.926 .		0.974	0.673	1.028 .	0.782	0.897	1.031	0.962	1.085	0.826
0.899	0.871	1.12	0.891	0.939	1.034	1.064	0.901 .		0.976	0.699	1.039 .	0.898	0.9	0.88	1.037	1.074	0.794
0.992	0.875	1.082	0.821	1.049	1.059	1.048	0.957 .		0.901	0.678	0.97 .	0.891	0.943	0.894	0.964	1.027	0.973
0.998	0.972	1.071	0.833	1.058	1.09	1.015	1.093 .		0.895	0.739	0.932 .	0.984	0.862	0.835	0.918	1.044	0.937
0.963	0.918	1.016	0.998	1.002	1.066	1.009	1.064 .		0.977	0.724	1.074 .	0.94	0.962	0.975	1.017	1.055	0.933
0.981	0.902	1.128	0.998	1.009	1.14	1.023	1.075 .		0.909	0.676	1.029 .	0.993	0.848	0.944	0.948	0.971	0.992
1.006	0.883	1.082	1.102	0.93	1.069	0.965	0.975 .		0.856	0.673	0.996 .	1.032	0.825	0.916	0.873	0.923	1.007
0.854	0.867	1.113	1.079	0.947	1.022	0.913	0.97 .		0.855	0.706	0.94 .	0.994	0.77	1.015	0.876	0.943	1.019
0.819	0.928	1.117	1.089	0.942	0.971	0.938	0.967 .		0.861	0.804	0.928 .	0.949	0.876	1.018	0.912	0.939	1.012

0.974	0.868	1.073	1.141	0.926	1.076	0.858	0.973 .		0.986	0.702	0.909 .	0.928	0.988	1.063	0.903	0.904	0.991
1.044	0.946	1.125	1.092	0.89	1.115	0.849	1.036 .		0.976	0.646	0.906 .	0.973	1.013	1.041	0.949	0.861	0.969
0.989	1.054	1.11	1.154	0.864	1.161	0.84	1.057 .		0.967	0.709	0.97 .	0.873	0.962	0.944	0.974	0.788	0.877
1.027	1.029	1.096	1.174	0.859	1.109	0.821	1.071 .		0.891		0.982 .	0.825	0.972	0.97	0.953	0.768	0.898
0.952	1.019	1.068	1.159	0.856	1.062	0.988	1.025 .		0.897		1.002 .	0.848	1.023	0.931	0.86	0.752	0.928
0.907	1.059	1.064	1.101	0.835	1.09	1.027	0.949 .		0.988		0.933 .	0.877	1.02	0.987	0.836	0.804	0.994
0.902	1.015	1.025	1.179	0.911	1.123	1.029	0.979 .		1.018		0.918 .	0.847	1.015	0.969	0.98	0.844	1.004
1.004	1.018	1.089	1.092	0.944	1.042	1.175	1.035 .		1.012		0.894 .	0.944	0.989	0.901	0.952	0.812	1.001
1.089	1.014	1.135	1.162	1.075	1.065	1.143	1.072 .		0.984		0.946 .	1.022	0.939	0.955	0.892	0.75	0.972
1.07	0.984	1.109	1.105	1.111	1.112	1.153	1.047 .		1.036		0.951 .	1.067	0.868	0.91	1.01	0.888	0.985
1.039	0.971	1.15	1.15	1.046	1.1	1.079	1.03 .		1.066		0.814 .	1.039	1.005	1.012	1.03	0.962	1.003
1.008	0.908	1.142	1.092	1.012	1.076	1.118	1.091 .		1.046		0.84 .	1.04	1.028	1.029	1.076	0.912	0.905
1.063	1.016	1.151	1.115	0.995	1.083	1.128	1.028 .		1.086		0.98 .	0.976	1.043	1.05	0.999	0.899	0.898
1.096	1.081	1.141	1.111	0.975	1.108	1.142	1.06 .		1.004		1.062 .	0.942	1.067	1.007	0.947	0.998	0.865
1.124	1.053	1.146	1.142	1.011	1.123	1.108	1.128 .		0.928		1.047 .	0.917	1.107	0.905	0.944	0.958	0.907
1.074	1.044	1.135	1.115	1.006	1.096	1.113	1.134 .		0.956		1.029 .	0.934	1.115	0.963	0.953	0.961	1.024
1.032	1.076	1.135	1.115	0.954	1.056	1.167	1.078 .		0.97		0.982 .	0.898	1.117	1.079	0.931	0.948	1.034
0.983	1.01	1.09	1.087	0.877	1.043	1.067	1.037 .		0.93		0.969 .	0.807	1.099	1.021	1	1.024	1.015
0.929	1.003	1.091	1.085	0.95	1.043	1.04	1.025 .		0.889		0.949 .	0.735	1.099	0.964	1.012	1.051	0.981
0.966	0.992	1.038	1.089	1.02	0.905	1.02	0.984 .		0.903		0.895 .	0.873	1.045	0.959	1.019	1.01	0.962
0.963	0.983	0.976	1.084	0.971	0.932	1.05	0.971 .		0.977		0.883 .	0.971	1.062	0.834	1.016	0.991	0.942
1.004	0.938	0.971	1.063	0.908	1.045	1.031	0.944 .		1.019		0.846 .	0.96	1.072	0.83	0.978	0.923	0.952
1.007	0.983	1.023	1.065	0.894	1.067	0.966	0.873 .		1.021		0.802 .	1.03	1.076	0.966	0.939	1.012	0.896
1.028	1.014	0.996	1.052	0.885	1.059	1.003	0.92 .		1.044		0.843 .	1.026	1.087	0.989	0.841	1.047	0.835
1.006	1.015	1.035	1.098	0.838	1.118	1.019	0.914 .		1.085		0.959 .	0.971	1.085	1.014	0.819	1.03	0.847
1.036	0.996	1.061	1.09	0.924	1.084	1.046	0.881 .		1.062		1.019 .	0.971	1.079	1.013	0.856	1.043	0.822
1.003	0.979	1.075	1.102	1.062	1.131	1.028	0.922 .		0.995		0.995 .	0.941	1.068	1.019	0.896	0.988	0.786
0.989	0.98	1.045	1.086	1.029	1.143	0.962	0.985 .		0.962		0.941 .	0.854	1.086	1.049	0.934	0.97	0.739
0.964	0.969	1.06	1.064	0.971	1.123	1.046	0.926 .		0.923		0.894 .	0.784	1.055	1.013	0.856	0.932	0.72
1.034	0.975	1.033	1.036	0.936	1.137	1.038	0.915 .		0.866		0.895 .	0.805	1.073	1.025	0.859	0.893	0.911
1.041	0.938	1.029	1.038	0.927	1.111	1.1	0.886 .		0.805		0.891 .	0.844	1.07	1.059	0.795	0.81	0.959
1.035	0.914	1.055	0.983	0.865	1.061	1.066	0.821 .		0.777		0.833 .	0.895	1.066	1.032	0.9	0.807	0.954
1.023	0.918	1.074	0.946	0.842	1.07	1.102	0.83 .		0.96		0.822 .	0.823	1.061	1.032	0.922	0.907	0.957
1.008	0.93	1.059	0.919	0.843	1.095	1.142	0.978	0.971	1.035		0.811 .	0.8	1.017	0.996	0.873	0.861	0.947
0.946	0.998	1.026	0.868	0.969	1.078	1.116	0.977	0.955	1.009		0.73 .	0.91	1.016	1.004	0.832	0.889	0.899
0.95	0.994	1.031	0.841	1.025	1.065	1.105	0.953	0.904	0.93		0.728 .	0.93	0.994	0.982	0.811	0.987	0.883
0.909	0.98	1.022	0.847	1.059	1.135	1.049	0.922	0.863	0.876		0.733 .	0.881	0.975	0.948	0.834	1.037	0.87
0.848	0.964	1.007	0.837	1.021	1.111	1.015	0.894	0.95	0.835		0.721 .	0.937	0.941	0.831	0.772	0.985	0.934
0.849	0.892	1.048	0.817	0.993	1.131	0.983	0.834	0.912	0.808		0.716 .	0.973	0.99	0.79	0.753	0.965	0.993
0.849	0.998	1.014	0.851	1.006	1.114	0.959	0.831	0.974	0.798		0.704 .	0.985	1.046	0.814	0.783	0.93	1.014
0.818	1.005	0.955	0.841	1.021	1.137	1.035	0.838	0.987	0.775		0.692 .	0.957	1.078	0.792	0.741	0.881	0.971
0.757	0.988	1.041	0.789	0.983	1.099	1.038	0.81	0.99	0.771		0.739 .	0.936	1.094	0.775	0.77	0.835	0.962
0.777	0.92	0.989	0.832	0.949	1.072	1.036	0.763	0.968	0.769		0.817 .	0.918	1.083	0.737	0.725	0.835	0.98
0.845	0.889	1.045	0.808	0.935	1.069	1.054	0.772	0.935	0.758		0.877 .	0.892	1.064	0.723	0.722	0.796	0.932
0.777	0.841	1.049	0.804	0.924	1.088	1.039	0.752	0.973	0.762		0.88 .	0.854	1.103	0.717	0.732	0.78	0.943
0.808	0.952	1.087	0.836	0.943	1.07	1.048	0.762	1.017	0.765		0.883 .	0.962	1.053	0.722	0.701	0.769	0.938
0.826	1.038	1.065	0.806	0.914	1.038	1.046	0.765	1.001	0.737		0.817 .	0.995	1.015	0.726	0.722	0.77	0.863
0.772	1.029	0.993	0.776	0.921	1.055	1.077	0.878	0.932	0.742		0.843 .	0.964	0.96	0.686	0.725	0.777	0.805
0.751	1.004	0.936	0.817	0.836	1.02	1.132	0.942	0.87	0.878		0.945 .	0.924	0.955	0.711	0.723	0.771	0.821
0.721	0.997	1.04	0.788	0.83	1.017	1.064	0.914	0.848	0.916		0.791 .	1.026	0.892	0.694	0.701	0.745	0.884
0.7	1.038	1.034	0.899	0.842	1.007	0.997	0.874	0.813	0.887		0.788 .	1.065	0.997	0.761	0.7	0.756	0.901
0.706	1.072	1.035	0.953	0.866	1.086	1.038	0.79	0.789	0.785		0.736 .	1.04	1.064	0.937	0.73	0.717	0.925
0.719	1.051	1.025	1.013	0.839	1.088	1.111	0.821	0.785	0.767		0.859 .	1.057	0.977	0.933	0.89	0.817	0.867
0.719	1.024	0.965	1	0.874	1.018	1.055	0.785	0.782	0.779		0.997 .	1.12	0.911	0.842	0.93	0.959	0.899
0.774	1.042	1.072	1.014	1.008	1.043	0.941	0.773	0.803	0.786		1.058 .	1.032	0.913	0.792	0.848	0.975	0.867
0.861	0.994	1.024	1.01	1.056	1.027	0.981	0.792	0.886	0.752		0.995 .	1.048	1.011	0.978	0.816	1.012	0.854
0.722	0.994	1.094	0.935	1.026	0.978	1.064	0.787	0.985	0.784		0.981 .	0.992	0.983	0.99	0.812	1.005	0.864
0.723	1.061	1.099	1.02	0.959	0.949	1.126	0.808	1.003	0.749		0.95 .	0.969	0.949	1.031	0.769	0.958	0.883
0.774	1.066	1.04	1.102	0.904	0.909	1.057	0.958	1.024	0.953		0.917 .	0.962	0.892	1.017	0.784	1.004	0.82
0.755	1.094	1.018	1.076	0.895	0.979	1.084	0.924	0.985	1.016		0.804 .	0.944	0.895	1.069	0.757	0.896	0.762
0.725	1.028	0.987	1.05	0.876	0.941	1.096	0.876	0.965	1.014		0.773 .	0.898	1.018	1.07	0.723	0.863	0.721
0.753	0.998	1.054	1.004	0.838	0.934	1.142	0.886	1.004	0.963		0.835 .	0.809	1.028	1.049	0.842	0.946	0.732
0.758	1.039	1.022	0.973	0.929	0.929	1.119	0.858	0.923	0.889		0.92 .	0.811	0.985	1	1.001	1.067	0.844

0.886	0.933	0.898	0.853	0.866	0.887	0.915	0.846	0.806	0.82 .	0.88 .	0.757	0.867	0.932	0.748	0.926	0.796
0.857	0.901	0.906	0.87	0.791	0.843	0.927	0.812	0.771	0.793 .	0.952 .	0.81	0.853	0.84	0.846	0.85	0.851
0.874	0.911	0.869	0.887	0.843	0.808	0.841	0.8	0.812	0.738 .	0.895 .	0.766	0.873	0.826	0.814	0.923	0.793
0.821	0.871	0.849	0.831	0.874	0.803	0.818	0.765	0.821	0.776 .	0.862 .	0.798	0.816	0.853	0.811	0.88	0.768
0.79	0.793	0.88	0.845	0.854	0.876	0.788	0.759	0.83	0.837 .	0.873 .	0.776	0.869	0.751	0.814	0.839	0.817
0.852	0.861	0.854	0.821	0.782	0.832	0.773	0.845	0.785	0.787 .	0.813 .	0.77	0.802	0.723	0.859	0.835	0.833
0.803	0.897	0.86	0.829	0.803	0.833	0.788	0.822	0.782	0.847 .	0.892 .	0.819	0.765	0.742	0.842	0.821	0.78
0.817	0.865	0.832	0.877	0.806	0.854	0.914	0.797	0.762	0.791 .	0.875 .	0.777	0.88	0.873	0.826	0.829	0.78
0.802	0.841	0.867	0.831	0.788	0.825	0.921	0.802	0.818	0.769 .	0.807 .	0.785	0.85	0.86	0.821	0.874	0.792
0.779	0.862	0.848	0.819	0.809	0.83	0.892	0.78	0.771	0.831 .	0.828 .	0.838	0.832	0.804	0.85	0.773	0.86
0.868	0.819	0.864	0.824	0.853	0.839	0.817	0.789	0.829	0.799 .	0.822 .	0.805	0.859	0.8	0.816	0.839	0.819
0.786	0.882	0.858	0.888	0.799	0.855	0.849	0.814	0.783	0.777 .	0.831 .	0.776	0.85	0.779	0.871	0.814	0.792
0.758	0.808	0.873	0.847	0.792	0.813	0.856	0.772	0.804	0.788 .	0.846 .	0.774	0.796	0.812	0.798	0.808	0.795
0.816	0.841	0.843	0.817	0.812	0.825	0.843	0.82	0.784	0.764 .	0.85 .	0.778	0.843	0.836	0.825	0.839	0.748
0.784	0.847	0.835	0.841	0.827	0.81	0.803	0.772	0.766	0.755 .	0.798 .	0.8	0.833	0.783	0.759	0.826	0.751
0.783	0.816	0.843	0.798	0.794	0.784	0.8	0.774	0.815	0.777 .	0.814 .	0.818	0.828	0.726	0.774	0.814	0.758
0.809	0.839	0.856	0.813	0.805	0.778	0.786	0.77	0.771	0.762 .	0.797 .	0.814	0.781	0.745	0.76	0.779	0.752
0.8	0.808	0.839	0.805	0.794	0.839	0.818	0.764	0.799	0.739 .	0.783 .	0.852	0.825	0.739	0.749	0.774	0.731
0.752	0.843	0.837	0.852	0.831	0.847	0.786	0.816	0.764	0.714 .	0.779 .	0.811	0.795	0.753	0.783	0.777	0.725
0.775	0.808	0.848	0.811	0.833	0.856	0.765	0.82	0.758	0.799 .	0.791 .	0.766	0.801	0.797	0.8	0.791	0.733
0.809	0.824	0.81	0.824	0.797	0.841	0.781	0.836	0.802	0.766 .	0.787 .	0.795	0.795	0.798	0.789	0.773	0.753
0.792	0.829	0.816	0.821	0.785	0.8	0.77	0.802	0.771	0.787 .	0.779 .	0.782	0.815	0.783	0.781	0.744	0.743
0.787	0.817	0.804	0.804	0.765	0.819	0.781	0.82	0.756	0.801 .	0.786 .	0.751	0.797	0.755	0.762	0.769	0.691
0.778	0.825	0.836	0.836	0.78	0.8	0.777	0.791	0.772	0.786 .	0.754 .	0.763	0.796	0.728	0.76	0.777	0.733
0.792	0.809	0.836	0.852	0.793	0.775	0.74	0.82	0.776	0.794 .	0.803 .	0.742	0.79	0.758	0.759	0.795	0.737
0.746	0.808	0.82	0.839	0.841	0.812	0.772	0.797	0.787	0.776 .	0.801 .	0.79	0.81	0.768	0.747	0.827	0.732
0.764	0.806	0.827	0.833	0.815	0.845	0.831	0.773	0.759	0.757 .	0.773 .	0.813	0.806	0.754	0.839	0.758	0.745
0.792	0.821	0.805	0.837	0.779	0.808	0.813	0.799	0.763	0.745 .	0.779 .	0.74	0.777	0.757	0.8	0.781	0.75
0.776	0.808	0.801	0.844	0.798	0.84	0.758	0.766	0.773	0.787 .	0.764 .	0.79	0.781	0.75	0.797	0.8	0.786
0.763	0.785	0.809	0.839	0.779	0.808	0.769	0.763	0.755	0.786 .	0.782 .	0.774	0.78	0.727	0.767	0.784	0.752
0.748	0.766	0.796	0.848	0.774	0.799	0.748	0.755	0.772	0.798 .	0.795 .	0.764	0.748	0.753	0.755	0.794	0.764
0.774	0.787	0.791	0.813	0.774	0.794	0.801	0.761	0.774	0.788 .	0.751 .	0.771	0.747	0.773	0.78	0.819	0.764
0.756	0.78	0.781	0.827	0.768	0.78	0.782	0.749	0.783	0.803 .	0.739 .	0.781	0.726	0.756	0.734	0.786	0.766
0.765	0.778	0.795	0.828	0.831	0.781	0.788	0.732	0.771	0.72 .	0.756 .	0.747	0.757	0.762	0.734	0.758	0.758
0.815	0.753	0.78	0.826	0.816	0.796	0.772	0.747	0.773	0.733 .	0.777 .	0.78	0.811	0.754	0.778	0.766	0.731
0.765	0.735	0.78	0.815	0.819	0.781	0.776	0.759	0.76	0.764 .	0.803 .	0.763	0.788	0.743	0.789	0.793	0.735
0.797	0.771	0.791	0.823	0.792	0.858	0.757	0.735	0.756	0.78 .	0.72 .	0.754	0.77	0.818	0.834	0.794	0.735
0.8	0.759	0.794	0.823	0.788	0.845	0.752	0.734	0.744	0.765 .	0.753 .	0.774	0.789	0.787	0.815	0.771	0.74
0.798	0.765	0.773	0.821	0.833	0.807	0.771	0.754	0.764	0.765 .	0.767 .	0.743	0.784	0.785	0.829	0.766	0.744
0.787	0.758	0.78	0.809	0.856	0.806	0.794	0.729	0.818	0.801 .	0.738 .	0.791	0.784	0.744	0.796	0.786	0.79
0.807	0.759	0.836	0.84	0.844	0.818	0.799	0.733	0.792	0.807 .	0.748 .	0.811	0.761	0.772	0.812	0.75	0.768

0.779	0.753	0.809	0.84	0.793	0.781	0.788	0.747	0.767	0.796 .	0.76 .	0.804	0.776	0.736	0.793	0.776	0.757
0.779	0.733	0.845	0.83	0.825	0.786	0.792	0.727	0.778	0.772 .	0.77 .	0.777	0.769	0.715	0.825	0.792	0.767
0.79	0.706	0.802	0.839	0.818	0.785	0.773	0.726	0.781	0.748 .	0.764 .	0.78	0.741	0.762	0.813	0.796	0.808
0.789	0.711	0.793	0.836	0.82	0.774	0.76	0.747	0.78	0.758 .	0.739 .	0.818	0.736	0.744	0.816	0.757	0.801
0.784	0.727	0.82	0.855	0.828	0.779	0.761	0.759	0.824	0.762 .	0.782 .	0.803	0.728	0.827	0.802	0.791	0.819
0.776	0.71	0.848	0.857	0.812	0.76	0.762	0.745	0.807	0.787 .	0.732 .	0.786	0.719	0.807	0.814	0.864	0.786
0.77	0.728	0.868	0.879	0.786	0.794	0.766	0.728	0.827	0.762 .	0.758 .	0.759	0.729	0.756	0.805	0.829	0.784
0.789	0.756	0.861	0.846	0.817	0.826	0.761	0.76	0.865	0.754 .	0.798 .	0.826	0.709	0.752	0.761	0.801	0.783
0.805	0.809	0.849	0.874	0.806	0.856	0.758	0.772	0.823	0.829 .	0.776 .	0.817	0.782	0.786	0.772	0.824	0.783
0.792	0.79	0.829	0.841	0.829	0.823	0.787	0.765	0.799	0.804 .	0.828 .	0.812	0.746	0.822	0.842	0.812	0.788
0.814	0.814	0.845	0.84	0.801	0.811	0.789	0.77	0.82	0.81 .	0.797 .	0.801	0.765	0.833	0.852	0.819	0.781
0.757	0.739	0.868	0.848	0.798	0.843	0.769	0.768	0.787	0.782 .	0.771 .	0.837	0.777	0.782	0.849	0.797	0.772
0.822	0.754	0.882	0.851	0.83	0.812	0.768	0.823	0.815	0.83 .	0.738 .	0.802	0.803	0.778	0.844	0.813	0.856
0.852	0.794	0.87	0.894	0.802	0.816	0.826	0.768	0.833	0.841 .	0.742 .	0.768	0.779	0.794	0.838	0.803	0.811
0.804	0.836	0.84	0.862	0.792	0.788	0.834	0.753	0.819	0.805 .	0.847 .	0.764	0.752	0.79	0.858	0.772	0.809
0.792	0.804	0.831	0.856	0.822	0.852	0.792	0.82	0.796	0.782 .	0.866 .	0.806	0.746	0.84	0.809	0.827	0.835
0.811	0.833	0.823	0.839	0.843	0.864	0.818	0.781	0.806	0.784 .	0.874 .	0.808	0.756	0.841	0.805	0.831	0.802
0.833	0.791	0.852	0.846	0.818	0.823	0.808	0.823	0.851	0.85 .	0.845 .	0.791	0.769	0.83	0.795	0.851	0.792
0.79	0.811	0.846	0.845	0.794	0.794	0.807	0.819	0.805	0.822 .	0.846 .	0.75	0.821	0.792	0.857	0.805	0.778
0.82	0.793	0.846	0.874	0.777	0.798	0.743	0.801	0.835	0.787 .	0.827 .	0.732	0.838	0.822	0.815	0.847	0.783
0.762	0.793	0.829	0.845	0.776	0.799	0.834	0.82	0.819	0.802 .	0.856 .	0.751	0.791	0.748	0.831	0.824	0.81
0.822	0.845	0.81	0.846	0.779	0.848	0.793	0.821	0.796	0.853 .	0.814 .	0.802	0.79	0.757	0.864	0.83	0.798
0.787	0.807	0.798	0.823	0.774	0.864	0.772	0.839	0.788	0.852 .	0.827 .	0.804	0.745	0.759	0.867	0.831	0.759
0.831	0.823	0.841	0.856	0.767	0.813	0.804	0.858	0.825	0.818 .	0.845 .	0.752	0.808	0.828	0.813	0.805	0.763
0.79	0.9	0.835	0.836	0.841	0.783	0.787	0.797	0.812	0.8 .	0.815 .	0.756	0.889	0.853	0.783	0.818	0.818
0.808	0.814	0.804	0.843	0.858	0.829	0.751	0.816	0.839	0.81 .	0.818 .	0.742	0.846	0.787	0.79	0.816	0.786
0.815	0.836	0.849	0.864	0.826	0.859	0.75	0.821	0.826	0.844 .	0.817 .	0.834	0.813	0.76	0.799	0.791	0.773
0.849	0.834	0.842	0.858	0.788	0.823	0.757	0.804	0.807	0.811 .	0.857 .	0.841	0.817	0.8	0.835	0.792	0.796
0.862	0.799	0.797	0.844	0.809	0.851	0.854	0.77	0.746	0.844 .	0.839 .	0.836	0.826	0.805	0.863	0.752	0.744
0.803	0.834	0.846	0.849	0.839	0.854	0.836	0.773	0.818	0.839 .	0.86 .	0.828	0.789	0.808	0.852	0.819	0.8
0.812	0.803	0.853	0.823	0.843	0.88	0.779	0.815	0.802	0.824 .	0.881 .	0.785	0.788	0.833	0.816	0.793	0.82
0.788	0.804	0.838	0.87	0.825	0.857	0.774	0.813	0.811	0.858 .	0.843 .	0.772	0.815	0.821	0.839	0.79	0.833
0.827	0.828	0.834	0.832	0.831	0.828	0.764	0.889	0.812	0.816 .	0.797 .	0.8	0.868	0.825	0.844	0.818	0.768
0.848	0.849	0.822	0.863	0.874	0.854	0.841	0.841	0.813	0.814 .	0.835 .	0.827	0.843	0.785	0.832	0.843	0.783
0.816	0.832	0.809	0.81	0.844	0.827	0.836	0.862	0.839	0.856 .	0.841 .	0.809	0.814	0.795	0.8	0.801	0.813
0.787	0.819	0.82	0.839	0.846	0.866	0.818	0.837	0.807	0.834 .	0.875 .	0.804	0.822	0.809	0.788	0.818	0.782
0.833	0.811	0.85	0.852	0.839	0.84	0.784	0.86	0.833	0.8 .	0.822 .	0.772	0.845	0.785	0.805	0.848	0.836
0.803	0.801	0.841	0.861	0.821	0.837	0.8	0.828	0.813	0.809 .	0.84 .	0.772	0.836	0.794	0.782	0.849	0.831
0.798	0.827	0.84	0.846	0.852	0.853	0.787	0.797	0.799	0.77 .	0.817 .	0.802	0.84	0.811	0.763	0.826	0.799
0.781	0.802	0.812	0.81	0.848	0.844	0.819	0.791	0.804	0.75 .	0.817 .	0.78	0.804	0.781	0.758	0.802	0.778
0.814	0.779	0.815	0.774	0.812	0.811	0.796	0.782	0.807	0.755 .	0.804 .	0.784	0.799	0.792	0.753	0.785	0.765
0.804	0.826	0.796	0.783	0.818	0.804	0.78	0.778	0.806	0.796 .	0.804 .	0.787	0.781	0.778	0.794	0.786	0.758
0.805	0.806	0.804	0.818	0.823	0.83	0.81	0.792	0.782	0.786 .	0.818 .	0.787	0.765	0.771	0.783	0.792	0.769
0.769	0.762	0.802	0.816	0.821	0.817	0.779	0.775	0.778	0.779 .	0.816 .	0.776	0.81	0.799	0.786	0.768	0.737
0.8	0.769	0.771	0.788	0.837	0.806	0.788	0.739	0.774	0.774 .	0.793 .	0.794	0.798	0.805	0.774	0.772	0.757
0.771	0.803	0.783	0.808	0.817	0.818	0.793	0.808	0.789	0.772 .	0.866 .	0.756	0.78	0.817	0.734	0.774	0.757
0.779	0.803	0.768	0.813	0.804	0.814	0.786	0.827	0.802	0.817 .	0.883 .	0.748	0.773	0.819	0.716	0.776	0.776
0.791	0.795	0.752	0.831	0.801	0.804	0.771	0.789	0.81	0.814 .	0.867 .	0.751	0.804	0.779	0.776	0.804	0.75
0.813	0.8	0.78	0.819	0.792	0.783	0.782	0.838	0.789	0.81 .	0.835 .	0.735	0.799	0.787	0.794	0.852	0.735
0.792	0.817	0.808	0.8	0.799	0.813	0.733	0.809	0.777	0.789 .	0.816 .	0.749	0.795	0.771	0.764	0.831	0.793
0.801	0.794	0.819	0.793	0.818	0.798	0.771	0.812	0.783	0.792 .	0.917 .	0.713	0.78	0.798	0.768	0.834	0.792
0.816	0.806	0.799	0.767	0.802	0.754	0.786	0.815	0.758	0.773 .	0.857 .	0.755	0.786	0.807	0.797	0.802	0.771
0.761	0.783	0.795	0.802	0.846	0.78	0.78	0.815	0.776	0.822 .	0.872 .	0.782	0.827	0.79	0.806	0.832	0.797
0.766	0.789	0.809	0.782	0.82	0.847	0.776	0.784	0.783	0.81 .	0.85 .	0.794	0.781	0.78	0.789	0.812	0.761
0.751	0.792	0.805	0.772	0.816	0.85	0.763	0.777	0.793	0.805 .	0.831 .	0.764	0.802	0.723	0.789	0.82	0.797
0.75	0.773	0.798	0.801	0.805	0.792	0.722	0.774	0.787	0.775 .	0.824 .	0.763	0.793	0.761	0.734	0.804	0.752
0.759	0.769	0.771	0.828	0.813	0.836	0.726	0.778	0.793	0.783 .	0.816 .	0.717	0.852	0.764	0.774	0.807	0.749
0.715	0.78	0.817	0.832	0.788	0.787	0.758	0.79	0.795	0.846 .	0.828 .	0.771	0.82	0.815	0.719	0.833	0.75
0.737	0.763	0.834	0.87	0.815	0.773	0.731	0.763	0.799	0.828 .	0.822 .	0.78	0.762	0.775	0.788	0.813	0.737
0.72	0.744	0.849	0.828	0.808	0.836	0.715	0.751	0.795	0.821 .	0.874 .	0.75	0.766	0.793	0.836	0.817	0.731
0.732	0.733	0.822	0.811	0.782	0.836	0.763	0.804	0.768	0.788 .	0.879 .	0.697	0.753	0.832	0.823	0.808	0.728
0.727	0.731	0.795	0.852	0.826	0.819	0.747	0.787	0.768	0.758 .	0.848 .	0.798	0.745	0.789	0.825	0.804	0.789
0.765	0.737	0.845	0.841	0.793	0.837	0.734	0.85	0.759	0.769 .	0.903 .	0.774	0.801	0.785	0.786	0.794	0.798
0.742	0.719	0.813	0.854	0.787	0.82	0.735	0.81	0.751	0.794 .	0.832 .	0.755	0.798	0.785	0.754	0.817	0.771
0.729	0.727	0.805	0.832	0.788	0.812	0.722	0.788	0.786	0.833 .	0.88 .	0.772	0.776	0.804	0.761	0.784	0.749
0.726	0.792	0.8	0.826	0.816	0.812	0.74	0.8	0.801	0.814 .	0.878 .	0.739	0.819	0.804	0.867	0.792	0.776
0.707	0.792	0.796	0.847	0.81	0.803	0.727	0.759	0.782	0.806 .	0.839 .	0.737	0.829	0.79	0.833	0.854	0.775
0.713	0.787	0.791	0.836	0.765	0.831	0.76	0.751	0.749	0.798 .	0.845 .	0.733	0.78	0.776	0.848	0.826	0.776
0.719	0.765	0.771	0.814	0.799	0.8	0.739	0.753	0.757	0.76 .	0.834 .	0.732	0.759	0.793	0.809	0.801	0.773
0.712	0.771	0.783	0.821	0.823	0.815	0.726	0.82	0.775	0.762 .	0.817 .	0.771	0.806	0.775	0.822	0.769	0.808
0.692	0.78	0.816	0.84	0.855	0.819	0.715	0.821	0.789	0.843 .	0.845 .	0.737	0.821	0.807	0.787	0.813	0.751
0.757	0.746	0.828	0.833	0.82	0.844	0.729	0.773	0.745	0.81 .	0.801 .	0.719	0.848	0.787	0.786	0.857	0.753
0.764	0.73	0.814	0.819	0.817	0.814	0.735	0.746	0.727	0.797 .	0.753 .	0.735	0.791	0.824	0.749	0.828	0.788
0.768	0.736	0.817	0.838	0.788	0.81	0.769	0.829	0.795	0.792 .	0.879 .	0.752	0.786	0.846	0.755	0.811	0.782
0.785	0.733	0.815	0.791	0.807	0.82	0.765	0.84	0.817	0.767 .	0.823 .	0.746	0.782	0.818	0.776	0.775	0.76
0.799	0.813	0.779	0.814	0.77	0.778	0.732	0.788	0.805	0.78 .	0.84 .	0.756	0.804	0.828	0.856	0.817	0.828
0.815	0.795	0.753	0.816	0.774	0.851	0.74	0.822	0.826	0.807 .	0.884 .	0.73	0.821	0.846	0.819	0.836	0.782
0.847	0.81	0.737	0.866	0.79	0.83	0.729	0.778	0.81	0.818 .	0.818 .	0.796	0.802	0.837	0.852	0.832	0.798
0.816	0.819	0.796	0.821	0.816	0.792	0.712	0.802	0.76	0.795 .	0.819 .	0.735	0.816	0.842	0.864	0.821	0.753
0.839	0.801	0.82	0.812	0.791	0.772	0.743	0.804	0.768	0.795 .	0.802 .	0.72	0.864	0.837	0.867	0.823	0.807
0.846	0.758	0.789	0.774	0.779	0.823	0.775	0.825	0.848	0.801 .	0.85 .	0.706	0.854	0.834	0.805	0.856	0.813
0.807	0.788	0.826	0.836	0.789	0.8	0.795	0.772	0.809	0.788 .	0.78 .	0.805	0.841	0.876	0.817	0.848	0.771
0.798	0.738	0.825	0.798	0.822	0.76	0.755	0.845	0.784	0.785 .	0.828 .	0.763	0.787	0.849	0.773	0.826	0.743
0.795	0.725	0.796	0.797	0.819	0.779	0.755	0.812	0.784	0.848 .	0.86 .	0.757	0.815	0.79	0.774	0.832	0.747
0.815	0.76	0.772	0.782	0.807	0.849	0.782	0.841	0.771	0.869 .	0.773 .	0.719	0.819	0.806	0.735	0.841	0.782
0.871	0.823	0.781	0.766	0.775	0.782	0.805	0.811	0.778	0.777 .	0.811 .	0.739	0.834	0.854	0.742	0.808	0.758
0.856	0.792	0.801	0.832	0.756	0.761	0.818	0.837	0.816	0.801 .	0.778 .	0.76	0.76	0.824	0.744	0.772	0.751
0.85	0.79	0.792	0.833	0.776	0.781	0.819	0.761	0.769	0.773 .	0.759 .	0.726	0.84	0.81	0.775	0.833	0.749
0.774	0.865	0.819	0.788	0.729	0.825	0.831	0.827	0.818	0.856 .	0.728 .	0.712	0.831	0.845	0.772	0.827	0.731
0.854	0.855	0.767	0.836	0.807	0.79	0.81	0.784	0.787	0.809 .	0.708 .	0.736	0.826	0.843	0.798	0.826	0.759
0.851	0.844	0.744	0.814	0.797	0.786	0.772	0.786	0.778	0.866 .	0.707 .	0.8	0.793	0.871	0.798	0.833	0.808
0.79	0.844	0.768	0.78	0.775	0.811	0.799	0.806	0.821	0.846 .	0.805 .	0.792	0.801	0.822	0.743	0.834	0.778
0.79	0.862	0.778	0.771	0.872	0.845	0.833	0.809	0.812	0.825 .	0.763 .	0.792	0.851	0.828	0.759	0.848	0.828
0.848	0.831	0.749	0.848	0.797	0.792	0.766	0.855	0.83	0.801 .	0.775 .	0.82	0.807	0.806	0.766	0.772	0.801
0.843	0.832	0.821	0.825	0.807	0.785	0.831	0.844	0.831	0.82 .	0.817 .	0.754	0.842	0.854	0.789	0.824	0.845
0.869	0.868	0.799	0.804	0.839	0.842	0.83	0.842	0.812	0.861 .	0.81 .	0.789	0.884	0.826	0.742	0.805	0.835
0.851	0.86	0.787	0.799	0.839	0.805	0.817	0.799	0.853	0.839 .	0.836 .	0.77	0.865	0.886	0.82	0.85	0.824
0.787	0.847	0.826	0.823	0.806	0.837	0.814	0.831	0.817	0.859 .	0.827 .	0.761	0.847	0.852	0.808	0.833	0.808
0.784	0.8	0.797	0.789	0.803	0.822	0.771	0.802	0.82	0.865 .	0.83 .	0.783	0.849	0.85	0.792	0.826	0.826
0.848	0.834	0.781	0.812	0.748	0.772	0.809	0.863	0.803	0.845 .	0.776 .	0.797	0.846	0.864	0.783	0.772	0.774
0.879	0.852	0.767	0.76	0.806	0.739	0.841	0.848	0.775	0.809 .	0.839 .	0.756	0.811	0.862	0.837	0.795	0.755
0.885	0.85	0.809	0.812	0.861	0.805	0.791	0.846	0.794	0.823 .	0.819 .	0.778	0.808	0.847	0.813	0.815	0.762
0.839	0.818	0.857	0.817	0.833	0.847	0.798	0.807	0.791	0.79 .	0.821 .	0.766	0.8	0.828	0.785	0.81	0.778
0.793	0.803	0.858	0.813	0.871	0.815	0.782	0.79	0.787	0.806 .	0.799 .	0.716	0.784	0.841	0.766	0.819	0.748
0.794	0.798	0.816	0.81	0.856	0.8	0.773	0.797	0.785	0.806 .	0.799 .	0.687	0.854	0.824	0.766	0.83	0.755

0.797	0.789	0.807	0.815	0.829	0.818	0.805	0.815	0.746	0.814 .	0.811 .	0.695	0.825	0.807	0.762	0.828	0.737
0.765	0.753	0.81	0.842	0.837	0.805	0.785	0.779	0.793	0.794 .	0.801 .	0.68	0.773	0.783	0.761	0.812	0.721
0.767	0.734	0.808	0.811	0.823	0.833	0.8	0.796	0.775	0.775 .	0.793 .	0.682	0.819	0.787	0.798	0.802	0.713
0.779	0.738	0.793	0.801	0.85	0.802	0.776	0.782	0.738	0.762 .	0.816 .	0.668	0.78	0.802	0.798	0.762	0.773
0.773	0.773	0.787	0.809	0.822	0.811	0.739	0.783	0.781	0.754 .	0.787 .	0.725	0.804	0.795	0.813	0.841	0.781
0.744	0.815	0.781	0.801	0.824	0.826	0.747	0.787	0.768	0.825 .	0.818 .	0.696	0.804	0.789	0.771	0.82	0.722
0.747	0.817	0.808	0.832	0.824	0.817	0.822	0.754	0.744	0.816 .	0.858 .	0.686	0.78	0.815	0.759	0.805	0.791
0.742	0.775	0.807	0.811	0.795	0.789	0.798	0.816	0.792	0.799 .	0.806 .	0.657	0.749	0.792	0.708	0.816	0.793
0.769	0.813	0.815	0.814	0.795	0.796	0.799	0.814	0.769	0.753 .	0.833 .	0.708	0.806	0.776	0.76	0.81	0.766
0.742	0.821	0.807	0.822	0.779	0.801	0.768	0.807	0.753	0.775 .	0.828 .	0.68	0.78	0.81	0.795	0.831	0.733
0.8	0.823	0.8	0.815	0.77	0.784	0.77	0.769	0.743	0.8 .	0.814 .	0.74	0.74	0.783	0.833	0.838	0.735
0.744	0.828	0.785	0.838	0.767	0.788	0.736	0.767	0.753	0.818 .	0.8 .	0.695	0.729	0.825	0.786	0.878	0.755
0.751	0.834	0.766	0.808	0.771	0.826	0.748	0.787	0.776	0.784 .	0.839 .	0.67	0.747	0.782	0.747	0.807	0.744
0.734	0.835	0.781	0.816	0.784	0.852	0.802	0.781	0.786	0.768 .	0.862 .	0.667	0.778	0.787	0.778	0.863	0.779
0.819	0.817	0.768	0.826	0.764	0.807	0.802	0.751	0.743	0.769 .	0.797 .	0.683	0.787	0.811	0.747	0.826	0.767
0.804	0.788	0.81	0.828	0.789	0.781	0.782	0.778	0.796	0.819 .	0.772 .	0.678	0.765	0.771	0.762	0.872	0.775
0.787	0.795	0.786	0.827	0.779	0.755	0.806	0.809	0.763	0.816 .	0.854 .	0.666	0.765	0.798	0.743	0.85	0.728
0.801	0.801	0.791	0.799	0.727	0.811	0.803	0.804	0.76	0.789 .	0.835 .	0.679	0.742	0.726	0.713	0.878	0.743
0.771	0.797	0.782	0.819	0.752	0.769	0.783	0.773	0.73	0.792 .	0.815 .	0.655	0.724	0.779	0.689	0.818	0.738
0.802	0.82	0.754	0.83	0.736	0.812	0.792	0.791	0.728	0.756 .	0.814 .	0.658	0.745	0.81	0.775	0.795	0.727
0.794	0.826	0.754	0.805	0.772	0.776	0.776	0.787	0.739	0.78 .	0.834 .	0.677	0.729	0.787	0.802	0.805	0.734
0.739	0.807	0.772	0.788	0.773	0.799	0.78	0.828	0.739	0.808 .	0.836 .	0.699	0.723	0.784	0.816	0.794	0.734
0.803	0.859	0.774	0.781	0.734	0.791	0.74	0.807	0.722	0.764 .	0.859 .	0.701	0.747	0.762	0.831	0.823	0.754
0.798	0.846	0.762	0.769	0.755	0.823	0.749	0.807	0.745	0.805 .	0.764 .	0.701	0.764	0.743	0.811	0.814	0.772
0.789	0.828	0.763	0.795	0.75	0.824	0.769	0.797	0.741	0.82 .	0.857 .	0.669	0.73	0.748	0.793	0.815	0.711
0.819	0.826	0.803	0.817	0.721	0.777	0.795	0.792	0.746	0.788 .	0.82 .	0.674	0.725	0.742	0.758	0.797	0.721
0.825	0.815	0.777	0.801	0.747	0.768	0.795	0.776	0.752	0.798 .	0.853 .	0.685	0.772	0.731	0.768	0.794	0.752
0.807	0.809	0.769	0.784	0.728	0.778	0.78	0.772	0.744	0.768 .	0.864 .	0.677	0.771	0.713	0.776	0.776	0.733
0.775	0.828	0.722	0.778	0.72	0.764	0.73	0.771	0.748	0.759 .	0.852 .	0.695	0.744	0.744	0.763	0.757	0.729
0.77	0.783	0.75	0.753	0.726	0.764	0.717	0.805	0.728	0.762 .	0.821 .	0.696	0.73	0.769	0.735	0.747	0.716
0.794	0.852	0.775	0.82	0.738	0.788	0.701	0.781	0.738	0.826 .	0.848 .	0.672	0.704	0.791	0.762	0.829	0.794
0.794	0.877	0.817	0.827	0.766	0.798	0.765	0.764	0.733	0.816 .	0.844 .	0.648	0.739	0.78	0.747	0.848	0.765
0.802	0.815	0.794	0.84	0.747	0.805	0.786	0.804	0.758	0.761 .	0.82 .	0.704	0.701	0.781	0.772	0.818	0.744
0.838	0.798	0.794	0.814	0.76	0.754	0.778	0.816	0.706	0.74 .	0.817 .	0.683	0.738	0.821	0.798	0.825	0.696
0.834	0.801	0.789	0.786	0.735	0.75	0.766	0.772	0.741	0.733 .	0.811 .	0.669	0.723	0.839	0.809	0.818	0.724
0.788	0.816	0.756	0.79	0.773	0.768	0.761	0.792	0.745	0.717 .	0.842 .	0.687	0.715	0.77	0.718	0.845	0.742
0.83	0.829	0.761	0.824	0.735	0.744	0.794	0.808	0.76	0.805 .	0.827 .	0.682	0.724	0.783	0.721	0.83	0.773
0.777	0.851	0.835	0.814	0.774	0.83	0.849	0.849	0.846	0.801 .	0.847 .	0.696	0.748	0.801	0.764	0.813	0.8
0.755	0.828	0.778	0.809	0.731	0.774	0.817	0.83	0.764	0.785 .	0.83 .	0.708	0.712	0.792	0.718	0.822	0.768
0.865	0.828	0.809	0.831	0.742	0.765	0.794	0.807	0.789	0.849 .	0.82 .	0.665	0.768	0.822	0.698	0.846	0.761
0.844	0.817	0.788	0.833	0.724	0.794	0.797	0.825	0.796	0.82 .	0.824 .	0.654	0.817	0.826	0.766	0.833	0.765
0.84	0.852	0.788	0.818	0.749	0.767	0.797	0.829	0.766	0.783 .	0.777 .	0.684	0.787	0.786	0.743	0.791	0.769
0.772	0.822	0.828	0.791	0.738	0.746	0.747	0.791	0.741	0.813 .	0.789 .	0.75	0.751	0.817	0.677	0.758	0.805
0.795	0.802	0.843	0.841	0.774	0.793	0.722	0.781	0.812	0.808 .	0.78 .	0.772	0.784	0.834	0.701	0.791	0.849
0.832	0.839	0.797	0.803	0.786	0.817	0.756	0.762	0.841	0.768 .	0.865 .	0.727	0.849	0.811	0.732	0.862	0.863
0.853	0.822	0.86	0.822	0.745	0.739	0.748	0.817	0.766	0.761 .	0.804 .	0.699	0.81	0.864	0.784	0.853	0.834
0.811	0.799	0.829	0.807	0.73	0.752	0.842	0.778	0.749	0.741	0.865 .	0.728	0.781	0.854	0.794	0.818	0.809
0.838	0.809	0.807	0.784	0.739	0.759	0.794	0.785	0.799	0.806	0.792 .	0.741	0.794	0.853	0.814	0.811	0.856
0.832	0.736	0.822	0.78	0.756	0.787	0.77	0.808	0.806	0.745	0.799 .	0.781	0.838	0.767	0.781	0.852	0.839
0.808	0.766	0.834	0.802	0.78	0.768	0.826	0.761	0.784	0.792	0.753 .	0.717	0.801	0.755	0.756	0.801	0.844
0.803	0.85	0.814	0.82	0.754	0.786	0.826	0.803	0.773	0.811	0.741 .	0.711	0.751	0.798	0.761	0.859	0.842
0.852	0.816	0.854	0.789	0.713	0.771	0.784	0.824	0.803	0.818	0.761 .	0.745	0.736	0.867	0.769	0.824	0.781
0.833	0.831	0.861	0.819	0.74	0.828	0.779	0.794	0.812	0.734	0.808 .	0.697	0.777	0.816	0.75	0.825	0.76
0.816	0.797	0.866	0.828	0.823	0.789	0.838	0.755	0.772	0.766	0.782 .	0.699	0.865	0.801	0.772	0.824	0.777
0.853	0.838	0.847	0.794	0.787	0.758	0.829	0.731	0.784	0.849	0.832 .	0.772	0.829	0.787	0.706	0.795	0.809
0.791	0.851	0.848	0.8	0.761	0.756	0.774	0.826	0.82	0.827	0.821 .	0.803	0.806	0.81	0.74	0.731	0.866
0.782	0.835	0.82	0.85	0.753	0.796	0.772	0.795	0.863	0.799	0.851 .	0.78	0.825	0.831	0.749	0.785	0.818
0.783	0.829	0.863	0.788	0.803	0.784	0.804	0.786	0.82	0.75	0.826 .	0.8	0.785	0.784	0.812	0.785	0.775
0.769	0.854	0.854	0.839	0.795	0.778	0.839	0.806	0.842	0.768	0.796 .	0.769	0.819	0.791	0.787	0.804	0.803
0.841	0.822	0.883	0.805	0.77	0.764	0.781	0.833	0.859	0.762	0.839 .	0.764	0.829	0.761	0.806	0.837	0.83
0.817	0.833	0.856	0.821	0.862	0.83	0.818	0.806	0.807	0.827	0.852 .	0.811	0.786	0.768	0.778	0.803	0.833
0.849	0.858	0.855	0.809	0.835	0.883	0.851	0.794	0.824	0.793	0.88 .	0.808	0.84	0.784	0.794	0.817	0.828
0.822	0.861	0.854	0.844	0.838	0.828	0.819	0.833	0.842	0.778	0.875 .	0.796	0.823	0.759	0.753	0.829	0.841
0.831	0.837	0.835	0.834	0.815	0.804	0.809	0.806	0.794	0.769	0.799 .	0.748	0.789	0.779	0.731	0.819	0.82
0.812	0.825	0.857	0.834	0.84	0.826	0.798	0.781	0.782	0.777	0.786 .	0.733	0.795	0.786	0.711	0.772	0.801
0.804	0.82	0.848	0.807	0.854	0.809	0.781	0.779	0.793	0.808	0.791 .	0.74	0.809	0.763	0.705	0.78	0.782
0.793	0.813	0.855	0.788	0.857	0.803	0.769	0.777	0.783	0.798	0.808 .	0.762	0.809	0.727	0.692	0.787	0.756
0.808	0.833	0.825	0.821	0.856	0.818	0.772	0.76	0.808	0.786	0.78 .	0.75	0.776	0.756	0.694	0.803	0.752
0.809	0.794	0.835	0.795	0.804	0.794	0.775	0.785	0.805	0.8	0.79 .	0.772	0.83	0.738	0.743	0.801	0.746
0.796	0.82	0.802	0.745	0.815	0.817	0.781	0.779	0.784	0.779	0.803 .	0.696	0.814	0.727	0.735	0.815	0.755
0.788	0.816	0.82	0.785	0.817	0.796	0.761	0.756	0.82	0.771	0.813 .	0.699	0.795	0.811	0.734	0.773	0.812
0.797	0.847	0.827	0.794	0.843	0.767	0.771	0.752	0.81	0.783	0.798 .	0.771	0.799	0.793	0.723	0.83	0.789
0.769	0.828	0.812	0.771	0.815	0.787	0.792	0.738	0.796	0.828	0.79 .	0.768	0.764	0.756	0.712	0.804	0.757
0.8	0.815	0.832	0.775	0.807	0.827	0.78	0.774	0.812	0.787	0.833 .	0.745	0.767	0.732	0.673	0.799	0.754
0.802	0.796	0.841	0.761	0.806	0.82	0.749	0.76	0.808	0.789	0.862 .	0.758	0.822	0.804	0.709	0.801	0.759
0.763	0.782	0.819	0.738	0.832	0.788	0.731	0.78	0.788	0.763	0.811 .	0.768	0.804	0.767	0.753	0.791	0.768
0.773	0.809	0.845	0.719	0.823	0.758	0.759	0.78	0.81	0.796	0.801 .	0.784	0.827	0.76	0.73	0.802	0.812
0.793	0.789	0.82	0.807	0.805	0.789	0.751	0.793	0.807	0.755	0.804 .	0.8	0.792	0.759	0.705	0.805	0.787
0.776	0.801	0.804	0.787	0.786	0.768	0.81	0.782	0.794	0.719	0.808 .	0.773	0.808	0.795	0.697	0.792	0.782
0.798	0.772	0.828	0.765	0.78	0.747	0.787	0.76	0.794	0.75	0.802 .	0.77	0.771	0.735	0.714	0.782	0.787
0.798	0.818	0.836	0.743	0.763	0.765	0.777	0.739	0.803	0.74	0.774 .	0.761	0.797	0.74	0.687	0.799	0.796
0.804	0.828	0.817	0.723	0.813	0.731	0.804	0.798	0.799	0.768	0.784 .	0.736	0.841	0.783	0.678	0.778	0.746
0.764	0.787	0.814	0.781	0.82	0.738	0.775	0.775	0.792	0.773	0.793 .	0.75	0.833	0.746	0.686	0.773	0.73
0.779	0.776	0.839	0.783	0.809	0.793	0.778	0.771	0.78	0.735	0.84 .	0.79	0.838	0.717	0.708	0.79	0.734
0.766	0.768	0.841	0.775	0.808	0.739	0.75	0.762	0.756	0.749	0.8 .	0.768	0.812	0.756	0.677	0.773	0.799
0.785	0.804	0.824	0.73	0.748	0.755	0.772	0.752	0.83	0.716	0.777 .	0.758	0.807	0.749	0.673	0.765	0.815
0.767	0.8	0.813	0.75	0.754	0.775	0.756	0.817	0.775	0.728	0.776 .	0.694	0.82	0.755	0.694	0.74	0.819
0.787	0.793	0.802	0.745	0.756	0.804	0.774	0.786	0.764	0.734	0.809 .	0.684	0.795	0.731	0.672	0.754	0.793
0.759	0.803	0.811	0.727	0.767	0.784	0.799	0.79	0.779	0.743	0.793 .	0.813	0.791	0.741	0.691	0.786	0.747
0.72	0.768	0.825	0.736	0.781	0.785	0.809	0.781	0.8	0.762	0.784 .	0.773	0.826	0.737	0.668	0.737	0.724
0.733	0.755	0.807	0.75	0.778	0.768	0.8	0.779	0.751	0.737	0.733 .	0.762	0.808	0.713	0.68	0.726	0.716
0.77	0.735	0.807	0.764	0.746	0.749	0.772	0.769	0.743	0.723	0.728 .	0.695	0.774	0.702	0.687	0.741	0.766
0.796	0.754	0.805	0.746	0.771	0.779	0.786	0.761	0.768	0.713	0.843 .	0.679	0.764	0.712	0.682	0.744	0.804
0.757	0.721	0.812	0.758	0.747	0.777	0.761	0.79	0.75	0.71	0.814 .	0.743	0.761	0.683	0.671	0.748	0.772
0.76	0.785	0.786	0.739	0.735	0.745	0.738	0.734	0.76	0.742	0.823 .	0.724	0.802	0.696	0.687	0.741	0.743
0.736	0.799	0.789	0.733	0.756	0.755	0.772	0.736	0.799	0.783	0.797 .	0.715	0.789	0.703	0.655	0.74	0.747
0.706	0.785	0.809	0.762	0.779	0.77	0.82	0.74	0.812	0.738	0.75 .	0.752	0.769	0.671	0.665	0.805	0.766
0.761	0.746	0.821	0.767	0.793	0.743	0.788	0.74	0.773	0.739	0.761 .	0.736	0.74	0.724	0.71	0.761	0.73
0.747	0.739	0.819	0.74	0.758	0.755	0.792	0.731	0.755	0.738	0.734 .	0.684	0.679	0.708	0.698	0.7	0.747
0.768	0.794	0.818	0.738	0.75	0.79	0.756	0.747	0.76	0.77	0.807 .	0.717	0.748	0.741	0.664	0.719	0.731
0.751	0.766	0.821	0.749	0.75	0.777	0.812	0.775	0.791	0.757	0.85 .	0.717	0.766	0.703	0.673	0.698	0.738
0.758	0.757	0.8	0.755	0.759	0.784	0.821	0.745	0.815	0.76	0.766 .	0.697	0.765	0.714	0.671	0.787	0.727
0.795	0.825	0.849	0.826	0.774	0.743	0.821	0.759	0.779	0.724	0.82 .	0.741	0.739	0.702	0.713	0.753	0.794
0.831	0.821	0.821	0.78	0.773	0.749	0.792	0.754	0.822	0.722	0.81 .	0.756	0.743	0.703	0.7	0.741	0.793
0.746	0.786	0.802	0.803	0.783	0.782	0.81	0.699	0.809	0.803	0.759 .	0.75	0.799	0.696	0.681	0.744	0.789

0.715	0.776	0.796	0.772	0.826	0.821	0.766	0.74	0.845	0.832		0.769	.	0.736	0.795	0.679				0.682	0.748	0.804
0.771	0.823	0.84	0.806	0.805	0.813	0.775	0.742	0.806	0.813		0.768	.	0.724	0.763	0.732				0.664	0.708	0.807
0.769	0.754	0.786	0.804	0.774	0.812	0.783	0.774	0.817	0.778		0.797	.	0.722	0.751	0.724				0.675	0.74	0.798
0.736	0.768	0.797	0.825	0.759	0.79	0.777	0.755	0.806	0.742		0.81	.	0.782	0.769	0.709				0.757	0.767	0.743
0.811	0.732	0.836	0.818	0.77	0.752	0.748	0.748	0.842	0.795		0.807	.	0.721	0.835	0.729				0.736	0.763	0.762
0.801	0.727	0.83	0.804	0.815	0.813									0.778	0.724						
0.8	0.782	0.846	0.808	0.8	0.857									0.856	0.845						
0.762	0.821	0.858	0.816	0.797	0.817									0.805	0.736						

8	7	7	10	10	12	12	12	1	1	1	1	2	2	5	6	6	8	9	11		1
Box-1	Box-6	Box-8	Box-7	Box-2	Box-3	Box-4	Box-7	Box-1	Box-3	Box-4	Box-5	Box-2	Box-3	Box-1	Box-2	Box-3	Box-8	Box-7	Box-5		Box-6
44.6	45	47	65	68	77	78	84	6.1	9.4	10.1	11.6	16.4	17.4	28.1	41	42	53.2	71	82		12.1
F	F	F	F	F	F	F	F	M	M	M	M	M	M	M	M	M	M	M	M		M
MCT	MCT	MCT	MCT	MCT	MCT	MCT	MCT	EtOH	EtOH	EtOH	EtOH	EtOH	EtOH	EtOH	EtOH	EtOH	EtOH	EtoH	EtOH		H2O
FT	FT	FT	FT	FT	FT	FT	FT	ME	ME	ME	ME	ME	ME	ME	ME	ME	ME	ME	ME		MH
0.756	0.712	0.748	0.718	0.741	0.77	0.72	0.729	0.833	0.906	0.787	0.833	0.888	0.73	0.897	0.884	0.923	0.728	0.732	0.8		0.797
0.821	0.679	0.768	0.742	0.745	0.796	0.732	0.755	0.776	0.86	0.75	0.793	0.873	0.752	0.869	0.874	0.874	0.676	0.694	0.787		0.782
0.869	0.678	0.75	0.713	0.69	0.755	0.725	0.734	0.763	0.855	0.751	0.766	0.854	0.756	0.905	0.891	0.924	0.736	0.706	0.799		0.824
0.862	0.696	0.708	0.733	0.717	0.718	0.739	0.767	0.802	0.86	0.75	0.839	0.851	0.743	0.884	0.907	0.93	0.726	0.714	0.78		0.844
0.834	0.72	0.715	0.737	0.74	0.754	0.751	0.746	0.839	0.893	0.839	0.899	0.846	0.775	0.886	0.909	0.896	0.734	0.753	0.746		0.906
0.882	0.708	0.731	0.743	0.734	0.862	0.753	0.746	0.856	0.91	0.881	0.893	0.814	0.775	0.887	0.897	0.887	0.713	0.748	0.735		0.884
0.874	0.842	0.735	0.737	0.729	0.87	0.737	0.75	0.843	0.908	0.899	0.883	0.802	0.786	0.882	0.867	0.927	0.699	0.705	0.759		0.824
0.878	0.887	0.741	0.744	0.72	0.849	0.747	0.732	0.851	0.894	0.877	0.891	0.802	0.762	0.91	0.875	0.927	0.718	0.718	0.72		0.857
0.888	0.848	0.692	0.724	0.717	0.825	0.775	0.743	0.9	0.906	0.883	0.905	0.803	0.8	0.914	0.896	0.901	0.769	0.721	0.755		0.864
0.869	0.954	0.706	0.842	0.739	0.891	0.752	0.768	0.863	0.918	0.872	0.899	0.811	0.767	0.898	0.88	0.944	0.876	0.716	0.733		0.801
0.896	0.901	0.696	0.88	0.727	0.869	0.797	0.793	0.888	0.918	0.9	0.916	0.785	0.776	0.913	0.913	0.941	0.889	0.712	0.75		0.889
0.879	0.912	0.686	0.863	0.717	0.884	0.849	0.77	0.883	0.934	0.903	0.945	0.77	0.78	0.901	0.93	0.929	0.895	0.737	0.757		0.914
0.91	0.92	0.683	0.852	0.684	0.884	0.831	0.792	0.889	0.928	0.909	0.949	0.759	0.777	0.891	0.903	0.894	0.927	0.711	0.753		0.875
0.906	0.931	0.696	0.865	0.71	0.887	0.774	0.773	0.903	0.914	0.912	0.956	0.762	0.768	0.913	0.871	0.894	0.92	0.699	0.8		0.881
0.895	0.942	0.693	0.882	0.692	0.838	0.8	0.769	0.918	0.932	0.92	0.95	0.763	0.794	0.904	0.911	0.944	0.927	0.79	0.762		0.923
0.905	0.933	0.715	0.901	0.705	0.858	0.858	0.776	0.843	0.934	0.902	0.966	0.754	0.811	0.89	0.921	0.922	0.916	0.806	0.78		0.902
0.871	0.95	0.731	0.893	0.699	0.783	0.897	0.836	0.925	0.971	0.958	0.951	0.734	0.816	0.892	0.905	0.931	0.936	0.837	0.834		0.931
0.877	0.917	0.78	0.893	0.657	0.766	0.912	0.837	0.885	0.944	0.941	0.925	0.729	0.801	0.93	0.879	0.936	0.898	0.878	0.858		0.932
0.906	0.905	0.737	0.867	0.691	0.752	0.902	0.846	0.909	0.935	0.937	0.943	0.732	0.727	0.914	0.945	0.945	0.915	0.829	0.832		0.923
0.905	0.966	0.729	0.855	0.664	0.825	0.915	0.828	0.925	0.926	0.952	0.957	0.744	0.789	0.908	0.925	0.941	0.93	0.86	0.819		0.91
0.932	0.837	0.734	0.856	0.713	0.803	0.932	0.871	0.912	0.96	0.95	0.966	0.736	0.778	0.936	0.878	0.926	0.95	0.847	0.828		0.927
0.93	0.812	0.858	0.911	0.683	0.865	0.961	0.863	0.906	0.903	0.933	0.936	0.732	0.758	0.926	0.91	0.939	0.948	0.897	0.84		0.912
0.94	0.844	0.905	0.881	0.651	0.851	0.94	0.884	0.92	0.959	0.963	0.966	0.731	0.745	0.908	0.876	0.917	0.952	0.88	0.861		0.963
0.908	0.779	0.906	0.884	0.632	0.797	0.943	0.848	0.906	0.972	0.954	0.962	0.706	0.735	0.897	0.91	0.904	0.931	0.88	0.867		0.952
0.888	0.857	0.926	0.857	0.683	0.783	0.98	0.838	0.905	0.926	0.93	0.944	0.737	0.763	0.835	0.936	0.95	0.939	0.867	0.835		0.937
0.925	0.904	0.92	0.847	0.671	0.791	0.964	0.882	0.901	0.894	0.945	0.941	0.741	0.749	0.893	0.894	0.943	0.953	0.912	0.873		0.939
0.94	0.889	0.962	0.851	0.644	0.791	0.987	0.843	0.891	0.941	0.956	0.953	0.729	0.755	0.897	0.873	0.898	0.938	0.874	0.817		0.926
0.937	0.898	0.945	0.857	0.67	0.751	0.953	0.837	0.857	0.916	0.955	0.913	0.725	0.75	0.905	0.923	0.912	0.942	0.892	0.816		0.908
0.931	0.828	0.912	0.85	0.677	0.842	0.988	0.835	0.891	0.909	0.932	0.898	0.754	0.74	0.925	0.878	0.953	0.909	0.931	0.815		0.907
0.87	0.869	0.953	0.875	0.662	0.817	0.96	0.797	0.882	0.923	0.953	0.93	0.725	0.736	0.819	0.896	0.919	0.927	0.904	0.784		0.877
0.899	0.79	0.909	0.892	0.697	0.789	0.969	0.742	0.876	0.917	0.939	0.946	0.734	0.76	0.845	0.836	0.914	0.937	0.903	0.838		0.945
0.921	0.797	0.94	0.877	0.741	0.734	0.932	0.754	0.868	0.909	0.919	0.921	0.739	0.819	0.843	0.85	0.883	0.92	0.916	0.79		0.939
0.93	0.74	0.893	0.889	0.711	0.761	0.954	0.752	0.868	0.896	0.914	0.903	0.702	0.791	0.868	0.858	0.937	0.947	0.918	0.786		0.918
0.954	0.807	0.936	0.914	0.715	0.77	0.998	0.769	0.879	0.871	0.904	0.913	0.723	0.814	0.885	0.829	0.907	0.914	0.914	0.775		0.901
0.899	0.823	0.913	0.903	0.697	0.765	0.968	0.757	0.886	0.834	0.873	0.91	0.731	0.8	0.84	0.81	0.934	0.891	0.92	0.823		0.9
0.95	0.855	0.916	0.912	0.686	0.775	1.003	0.789	0.895	0.84	0.926	0.916	0.734	0.836	0.899	0.753	0.876	0.884	0.853	0.852		0.926
0.978	0.913	0.916	0.886	0.689	0.841	0.963	0.768	0.883	0.857	0.909	0.92	0.767	0.848	0.876	0.778	0.865	0.925	0.881	0.861		0.93
0.93	0.862	0.879	0.865	0.711	0.816	0.966	0.774	0.839	0.84	0.884	0.88	0.815	0.837	0.87	0.845	0.852	0.939	0.886	0.892		0.898
0.925	0.869	0.87	0.838	0.715	0.758	0.969	0.739	0.808	0.804	0.874	0.866	0.841	0.853	0.869	0.835	0.857	0.923	0.938	0.806		0.903
0.892	0.897	0.828	0.889	0.709	0.787	0.962	0.797	0.867	0.813	0.906	0.913	0.852	0.756	0.873	0.831	0.833	0.902	0.829	0.856		0.889
0.926	0.871	0.787	0.899	0.686	0.762	0.992	0.775	0.85	0.829	0.867	0.884	0.874	0.876	0.82	0.832	0.872	0.858	0.839	0.815		0.907
0.876	0.921	0.753	0.892	0.665	0.765	0.979	0.746	0.848	0.874	0.922	0.904	0.848	0.864	0.8	0.844	0.837	0.79	0.845	0.797		0.913
0.895	0.943	0.741	0.898	0.721	0.775	0.978	0.758	0.835	0.862	0.862	0.911	0.878	0.873	0.789	0.868	0.903	0.759	0.836	0.818		0.924
0.911	0.949	0.77	0.796	0.655	0.79	0.99	0.741	0.789	0.87	0.876	0.88	0.878	0.856	0.804	0.844	0.866	0.773	0.869	0.787		0.918
0.894	0.911	0.768	0.819	0.701	0.78	0.982	0.752	0.845	0.844	0.868	0.911	0.856	0.841	0.793	0.835	0.815	0.732	0.893	0.836		0.889
0.904	0.946	0.841	0.788	0.685	0.763	0.998	0.743	0.86	0.899	0.839	0.89	0.865	0.805	0.756	0.818	0.835	0.8	0.92	0.816		0.916
0.849	0.99	0.883	0.823	0.677	0.729	0.952	0.7	0.867	0.887	0.853	0.887	0.857	0.851	0.746	0.917	0.884	0.802	0.87	0.85		0.899
0.849	1.003	0.915	0.814	0.701	0.763	0.964	0.705	0.837	0.826	0.863	0.889	0.847	0.827	0.788	0.825	0.843	0.862	0.862	0.81		0.869
0.862	0.927	0.912	0.871	0.68	0.795	0.948	0.77	0.81	0.812	0.84	0.882	0.897	0.811	0.822	0.78	0.795	0.808	0.854	0.757		0.84
0.867	0.919	0.905	0.873	0.712	0.77	0.939	0.767	0.852	0.855	0.877	0.876	0.823	0.807	0.807	0.788	0.83	0.814	0.846	0.749		0.875
0.93	0.924	0.901	0.896	0.702	0.769	0.961	0.773	0.873	0.846	0.889	0.85	0.809	0.77	0.772	0.837	0.875	0.746	0.834	0.761		0.896
0.882	0.837	0.917	0.836	0.672	0.779	0.971	0.7	0.855	0.89	0.914	0.868	0.79	0.844	0.824	0.862	0.911	0.85	0.806	0.81		0.882
0.863	0.926	0.934	0.867	0.642	0.783	0.968	0.77	0.846	0.824	0.889	0.894	0.783	0.826	0.791	0.821	0.914	0.9	0.839	0.781		0.9
0.899	0.925	0.907	0.834	0.711	0.767	0.952	0.826	0.842	0.83	0.88	0.832	0.782	0.763	0.827	0.809	0.887	0.867	0.86	0.759		0.887
0.915	0.947	0.928	0.901	0.701	0.741	1.003	0.854	0.85	0.838	0.918	0.795	0.767	0.724	0.786	0.852	0.912	0.8	0.897	0.766		0.914
0.942	0.952	0.91	0.887	0.699	0.732	1.012	0.874	0.819	0.882	0.876	0.844	0.773	0.77	0.849	0.838	0.843	0.82	0.86	0.76		0.892
0.903	0.986	0.959	0.882	0.714	0.728	0.982	0.879	0.844	0.866	0.879	0.85	0.787	0.796	0.852	0.84	0.818	0.857	0.867	0.73		0.851
0.91	0.963	0.877	0.898	0.681	0.767	0.99	0.907	0.846	0.817	0.846	0.852	0.765	0.826	0.809	0.813	0.813	0.859	0.848	0.738		0.886
0.939	0.96	0.902	0.904	0.67	0.713	0.968	0.91	0.828	0.839	0.879	0.863	0.767	0.862	0.85	0.777	0.819	0.89	0.877	0.785		0.876
0.92	0.971	0.926	0.899	0.652	0.747	0.937	0.95	0.835	0.844	0.896	0.855	0.76	0.868	0.864	0.828	0.876	0.786	0.895	0.773		0.834
0.876	0.945	0.923	0.862	0.651	0.739	0.964	0.953	0.832	0.905	0.95	0.923	0.746	0.86	0.835	0.83	0.864	0.783	0.886	0.759		0.854
0.927	0.92	0.937	0.906	0.718	0.775	0.994	0.947	0.848	0.861	0.905	0.94	0.754	0.83	0.817	0.826	0.795	0.797	0.89	0.73		0.872
0.893	0.94	0.958	0.903	0.674	0.791	0.971	0.944	0.875	0.874	0.912	0.903	0.777	0.843	0.758	0.753	0.788	0.88	0.921	0.77		0.902
0.919	0.98	0.995	0.918	0.707	0.764	0.903	0.938	0.879	0.851	0.914	0.886	0.798	0.898	0.832	0.78	0.808	0.87	0.944	0.752		0.836
0.909	0.915	0.893	0.901	0.684	0.848	0.947	0.962	0.887	0.866	0.936	0.91	0.866	0.884	0.842	0.801	0.849	0.843	0.936	0.801		0.828
0.877	0.952	0.945	0.893	0.677	0.878	0.97	0.915	0.869	0.87	0.949	0.933	0.864	0.865	0.877	0.841	0.912	0.803	0.917	0.77		0.877
0.894	0.973	0.94	0.919	0.665	0.858	0.966	0.954	0.874	0.853	0.941	0.895	0.856	0.918	0.884	0.852	0.896	0.835	0.945	0.757		0.899
0.887	0.93	0.889	0.908	0.649	0.882	0.972	0.925	0.814	0.878	0.953	0.902	0.899	0.893	0.857	0.872	0.903	0.808	0.921	0.787		0.927
0.835	0.976	0.851	0.878	0.677	0.85	1.009	0.994	0.877	0.873	0.968	0.852	0.91	0.918	0.836	0.883	0.935	0.86	0.921	0.792		0.909
0.848	0.969	0.809	0.901	0.696	0.816	0.979	0.939	0.932	0.922	0.936	0.865	0.883	0.908	0.857	0.846	0.909	0.909	0.907	0.77		0.92
0.827	0.932	0.917	0.901	0.679	0.809	0.982	0.924	0.868	0.906	0.935	0.915	0.865	0.871	0.848	0.873	0.918	0.888	0.907	0.773		0.87
0.88	0.978	0.915	0.887	0.674	0.872	0.967	0.944	0.89	0.921	0.938	0.924	0.903	0.88	0.854	0.875	0.927	0.918	0.897	0.845		0.91
0.929	0.952	0.935	0.84	0.707	0.824	0.982	0.962	0.906	0.943	0.929	0.93	0.925	0.904	0.882	0.886	0.915	0.946	0.853	0.872		0.899
0.906	1.002	0.947	0.837	0.661	0.839	0.973	0.934	0.918	0.93	0.914	0.935	0.929	0.918	0.903	0.877	0.917	0.906	0.862	0.896		0.912
0.866	0.98	0.922	0.879	0.675	0.875	0.954	0.919	0.906	0.932	0.916	0.932	0.903	0.856	0.879	0.899	0.928	0.927	0.85	0.895		0.903
0.92	0.965	0.857	0.877	0.677	0.875	0.977	0.975	0.915	0.937	0.949	0.939	0.915	0.919	0.887	0.87	0.905	0.899	0.85	0.934		0.92
0.909	0.97	0.868	0.883	0.717	0.894	0.959	0.952	0.922	0.928	0.939	0.955	0.921	0.913	0.907	0.893	0.915	0.923	0.836	0.896		0.933
0.899	0.954	0.96	0.874	0.69	0.927	1.032	0.962	0.896	0.938	0.926	0.907	0.903	0.926	0.918	0.891	0.919	0.94	0.83	0.907		0.916
0.915	0.976	0.937	0.874	0.669	0.872	0.996	0.96	0.901	0.941	0.937	0.95	0.925	0.892	0.927	0.876	0.921	0.917	0.86	0.919		0.909
0.912	0.989	0.947	0.875	0.683	0.873	0.993	0.977	0.926	0.925	0.942	0.949	0.927	0.975	0.892	0.878	0.904	0.879	0.886	0.907		0.92
0.899	1.018	0.96	0.858	0.684	0.92	0.993	0.937	0.946	0.959	0.956	0.949	0.926	0.94	0.884	0.921	0.904	0.922	0.882	0.926		0.892
0.932	0.969	0.951	0.836	0.68	0.918	1.002	0.967	0.928	0.955	0.936	0.954	0.929	0.924	0.919	0.895	0.894	0.924	0.859	0.91		0.908
0.902	0.973	0.936	0.778	0.661	0.916	1	0.975	0.928	0.947	0.976	0.944	0.936	0.917	0.888	0.845	0.849	0.92	0.847	0.911		0.911
0.912	0.965	0.954	0.729	0.574	0.922	1.002	0.968	0.912	0.987	0.963	0.94	0.967	0.948	0.857	0.904	0.88	0.942	0.784	0.921		0.909
0.883	0.993	0.948	0.693	0.637	0.924	0.987	0.952	0.901	0.941	0.943	0.923	0.934	0.909	0.834	0.905	0.856	0.87	0.818	0.941		0.894
0.879	0.964	0.952	0.838	0.659	0.949	0.969	0.971	0.927	0.986	0.961	0.939	0.939	0.92	0.861	0.881	0.862	0.981	0.83	0.937		0.903
0.917	0.975	0.955	0.872	0.648	0.925	0.979	0.958	0.893	0.93	0.926	0.91	0.972	0.917	0.886	0.89	0.846	0.95	0.904	0.937		0.844
0.898	0.962	0.954	0.884	0.625	0.94	0.983	1.001	0.898	0.983	0.948	0.932	0.962	0.928	0.883	0.917	0.84	0.938	0.873	0.931		0.834
0.883	0.962	0.978	0.859	0.595	0.945	0.965	0.984	0.916	0.975	0.967	0.953	0.923	0.911	0.893	0.916	0.863	0.935	0.879	0.943		0.879
0.914	0.961	1.005	0.901	0.544	0.959	0.966	0.981	0.913	0.959	0.991	0.95	0.92	0.94	0.913	0.933	0.869	0.908	0.858	0.947		0.906
0.97	0.945	0.965	0.876	0.622	0.938	0.986	0.966	0.922	0.928	0.945	0.939	0.956	0.905	0.892	0.932	0.904	0.911	0.89	0.945		0.865
0.945	0.946	0.964	0.893	0.677	0.949	0.994	1.007	0.916	0.98	0.958	0.954	0.941	0.933	0.893	0.924	0.877	0.932	0.867	0.933		0.867
0.901	0.932	0.986	0.901	0.656	0.932	0.994	0.991	0.913	0.98	0.965	0.97	0.937	0.911	0.882	0.904	0.885	0.955	0.885	0.944		0.83
0.935	0.937	0.989	0.862	0.619	0.96	1.004	0.999	0.889	0.955	0.926	0.951	0.941	0.932	0.891	0.882	0.914	0.9	0.875	0.913		0.871
0.921	0.92	0.962	0.915	0.686	0.97	0.991	0.993	0.926	0.953	0.944	0.947	0.954	0.953	0.899	0.905	0.908	0.95	0.897	0.971		0.869
0.913	0.938	0.956	0.896	0.633	0.963	0.989	0.972	0.903	0.941	0.922	0.952	0.967	0.932	0.922	0.915	0.882	0.953	0.896	0.976		0.883
0.898	0.927	0.944	0.858	0.699	0.96	0.989	0.957	0.942	0.988	0.943	0.947	0.963	0.931	0.881	0.872	0.86	0.973	0.913	0.951		0.886
0.921	0.933	0.952	0.906 -		0.97	0.99	0.97	0.907	0.933	0.955	0.975	0.956	0.927	0.885	0.865	0.888	0.955	0.887	0.948		0.904

0.931	0.908	0.994	0.883	0.68	0.988	0.982	0.97	0.924	0.97	0.979	0.984	0.939	0.869	0.904	0.893	0.882	0.931	0.904	0.968	0.933
0.902	0.87	0.991	0.861	0.666	0.958	1.002	0.945	0.91	0.943	0.909	0.951	0.928	0.892	0.886	0.816	0.884	0.936	0.939	0.943	0.913
0.912	0.907	0.976	0.881	0.648	0.955	0.942	0.964	0.931	0.951	0.944	0.94	0.973	0.921	0.874	0.828	0.9	0.923	0.915	0.936	0.906
0.907	0.97	1.016	0.864	0.588	0.949	0.984	0.979	0.902	0.947	0.928	0.97	0.975	0.915	0.821	0.863	0.839	0.936	0.872	0.953	0.938
0.909	0.922	0.984	0.874	0.621	0.944	0.959	0.99	0.877	0.925	0.891	0.936	0.957	0.909	0.858	0.855	0.845	0.916	0.911	0.927	0.96
0.924	0.939	0.956	0.917	0.623	0.936	0.996	0.977	0.881	0.909	0.827	0.948	0.963	0.939	0.863	0.812	0.779	0.882	0.928	0.934	0.924
0.859	0.904	0.951	0.851	0.634	0.935	0.983	0.974	0.877	0.935	0.828	0.947	0.951	0.956	0.832	0.82	0.859	0.853	0.879	0.937	0.879
0.852	0.912	0.947	0.892	0.794	0.962	0.958	0.954	0.821	0.967	0.874	0.965	0.945	0.959	0.843	0.771	0.86	0.918	0.899	0.924	0.951
0.823	0.885	0.972	0.878	0.69	0.939	0.936	0.956	0.836	0.993	0.903	0.957	0.954	0.945	0.801	0.779	0.851	0.911	0.884	0.93	0.954
0.803	0.933	0.958	0.879	0.698	0.942	0.963	0.947	0.839	0.964	0.874	0.929	0.966	0.93	0.78	0.764	0.86	0.93	0.898	0.899	0.932
0.842	0.931	0.988	0.913	0.689	0.949	0.972	0.974	0.822	0.97	0.868	0.905	0.956	0.928	0.774	0.804	0.849	0.943	0.908	0.951	0.93
0.854	0.861	0.982	0.885	0.689	0.929	0.982	0.97	0.82	0.956	0.859	0.852	0.983	0.965	0.787	0.833	0.795	0.907	0.863	0.882	0.885
0.811	0.851	0.98	0.876	0.682	0.956	0.981	0.968	0.837	0.928	0.862	0.879	0.96	0.957	0.762	0.826	0.74	0.884	0.894	0.887	0.874
0.773	0.929	0.972	0.886	0.742	0.957	0.995	0.986	0.799	0.918	0.847	0.888	0.97	0.959	0.822	0.839	0.736	0.954	0.825	0.919	0.88
0.788	0.916	0.954	0.904	0.804	0.963	1.005	0.935	0.834	0.914	0.857	0.874	0.96	0.974	0.8	0.812	0.72	0.912	0.804	0.872	0.852
0.783	0.942	0.974	0.841	0.804	0.962	1.006	0.945	0.841	0.919	0.845	0.869	1.005	0.969	0.823	0.783	0.689	0.933	0.867	0.904	0.891
0.819	0.893	1	0.889	0.82	0.946	1.021	0.957	0.85	0.902	0.861	0.925	0.97	0.946	0.795			0.949	0.876	0.845	0.928
0.823	0.87	0.942	0.86	0.799	0.947	1.018	0.973	0.821	0.857	0.841	0.898	0.936	0.935	0.784			0.945	0.884	0.815	0.947
0.822	0.935	0.952	0.902	0.806	0.952	1.003	0.964	0.826	0.862	0.842	0.827	0.949	0.914	0.73			0.917	0.872	0.791	0.872
	0.897	0.976	0.897	0.817	0.96	0.974	0.974	0.811	0.883	0.835	0.862	0.951	0.958				0.931	0.883	0.86	0.922
	0.841	0.969	0.92	0.851	0.942	0.998	0.969	0.811	0.936	0.844	0.912	0.941	0.919				0.961	0.921	0.855	0.929
	0.913	0.996	0.924	0.862	0.887	0.997	0.933	0.834	0.887	0.852	0.928	0.944	0.947				0.932	0.902	0.884	0.914
	0.863	0.991	0.931	0.83	0.887	1.001	0.954	0.798	0.899	0.863	0.885	0.941	0.935				0.935	0.936	0.857	0.905
	0.93	0.992	0.881	0.827	0.853	1.021	0.955	0.775	0.906	0.823	0.882	0.965	0.944				0.918	0.957	0.89	0.873
	0.973	1.008	0.907	0.831	0.885	1	0.966	0.785	0.932	0.849	0.916	0.951	0.931				0.932	0.914	0.906	0.874
	0.984	0.998	0.898	0.843	0.885	0.979	0.935	0.824	0.904	0.814	0.948	0.975	0.931				0.907	0.934	0.894	0.93
	0.965	1.022	0.881	0.894	0.978	1.026	0.964	0.849	0.894	0.831	0.932	0.947	0.918				0.905	0.923	0.93	0.928
	0.894	0.999	0.885	0.84	0.933	0.986	0.943	0.845	0.964	0.896	0.912	0.938	0.884				0.918	0.925	0.923	0.912
	0.941	1.024	0.916	0.858	0.95	0.999	0.97	0.804	0.949	0.907	0.924	0.951	0.932				0.859	0.946	0.835	0.908
	0.951	1.011	0.927	0.896	0.9	1.009	0.962	0.801	0.971	0.901	0.934	0.928	0.905				0.922	0.919	0.841	0.93
	0.959	0.994	0.922	0.885	0.926	1.021	0.963	0.826	0.914	0.844	0.93	0.942	0.943				0.817	0.957	0.928	0.945
	0.916	0.985	0.926	0.927	0.969	1.006	0.972	0.822	0.909	0.859	0.895	0.939	0.925				0.847	0.91	0.944	0.949
	0.937	0.98	0.968	0.895	0.974	0.996	0.977	0.805	0.942	0.892	0.923	0.941	0.906				0.866	0.931	0.941	0.952
	0.95	0.971	0.967	0.91	0.971	0.998	0.965	0.808	0.932	0.899	0.908	0.941	0.912				0.908	0.934	0.957	0.972
	0.968	0.968	0.933	0.894	0.983	1.011	0.959	0.886	0.884	0.907	0.916	0.961	0.916				0.9	0.899	0.924	0.95
	0.972	0.976	0.931	0.863	0.958	1.005	0.987	0.876	0.895	0.886	0.936	0.97	0.906				0.921	0.899	0.943	0.935
	0.966	0.937	0.915	0.911	0.956	0.988	0.966	0.871	0.932	0.912	0.953	0.941	0.93				0.936	0.913	0.93	0.944
	0.936	0.95	0.965	0.932	0.987	0.965	0.96	0.93	0.936	0.933	0.943	0.945	0.906				0.913	0.887	0.937	0.938
	0.957	0.951	0.958	0.913	0.978	0.992	0.979	0.922	0.945	0.935	0.949	0.929	0.922				0.893	0.865	0.947	0.95
	0.969	0.951	0.924	0.876	0.971	0.979	0.958	0.897	0.957	0.901	0.923	0.947	0.925				0.905	0.898	0.94	0.919
	0.979	0.959	0.884	0.899	0.961	0.957	0.955	0.939	0.967	0.937	0.963	0.944	0.928				0.931	0.908	0.95	0.924
	0.98	0.941	0.902	0.878	0.98	0.972	0.975	0.929	0.954	0.946	0.962	0.944	0.926				0.926	0.876	0.946	0.926
	0.993	0.976	0.883	0.854	0.968	0.96	0.955	0.943	0.941	0.943	0.939	0.954	0.968				0.89	0.926	0.951	0.936
	0.961	0.966	0.913	0.869	1.004	0.989	0.971	0.933	1.003	0.971	0.988	0.959	0.94				0.933	0.93	0.946	0.927
	0.905	0.943	0.906	0.888	0.987	0.992	0.968	0.931	0.985	0.97	0.969	0.969	0.941				0.918	0.924	0.931	0.938
	0.899	0.962	0.91	0.907	0.971	0.996	0.994	0.93	0.955	0.937	0.949	0.965	0.959				0.956	0.905	0.966	0.899
	0.966	0.964	0.919	0.854	0.975	0.97	0.975	0.933	0.93	0.987	0.989	0.953	0.939				0.924	0.925	0.936	0.942
	0.929	0.898	0.922	0.948	0.973	0.975	0.963	0.986	0.983	0.961	0.983	0.953	0.928				0.924	0.92	0.932	0.966
	0.923	0.967	0.913	0.891	0.978	0.967	0.984	0.957	0.977	0.965	0.97	0.989	0.946				0.958	0.91	0.966	0.969
	0.904	0.97	0.904	0.904	0.976	0.968	0.989	0.928	0.959	0.97	0.977	1.009	0.932				0.965	0.889	0.936	0.907
	0.863	0.939	0.871	0.919	0.976	0.99	1.015	0.942	0.947	0.969	0.948	0.961	0.951				0.939	0.897	0.958	0.921
	0.832	0.9	0.897	0.894	0.995	0.999	1.021	0.931	0.941	0.963	0.954	0.975	0.965				0.947	0.876	0.951	0.957
	0.967	0.939	0.94	0.915	0.986	0.978	0.982	0.929	0.947	0.959	0.994	0.993	0.949				0.908	0.859	0.939	0.897
	0.944	0.925	0.875	0.92	0.989	0.963	0.98	0.926	0.954	0.937	0.995	0.962	0.95				0.911	0.816	0.946	0.921
	0.939	0.958	0.891	0.874	0.984	0.995	0.995	0.934	0.921	0.964	0.964	0.964	0.951				0.931	0.817	0.946	0.909
	0.852	0.948	0.892	0.916	1.028	0.953	0.975	0.92	0.938	0.96	0.962	0.966	0.92				0.926	0.872	0.939	0.914
	0.857	0.89	0.805	0.909	0.985	0.991	0.957	0.953	0.902	0.938	0.92	0.969	0.909				0.922 -		0.929	0.872
	0.82	0.927	0.846	0.919	0.985	0.983	0.967	0.939	0.889	0.897	0.924	0.985	0.919				0.937 -		0.953	0.909
	0.78	0.953	0.858	0.918	0.976	1.003	0.987	0.953	0.825	0.915	0.922	0.997	0.936				0.936	0.827	0.914	0.897
	0.802	0.974	0.888	0.885	0.984	0.995	1.02	0.924	0.874	0.904	0.922	0.968	0.96				0.883	0.835	0.914	0.902
	0.783	0.962	0.888	0.94	0.99	1.026	0.99	0.871	0.894	0.938	0.929	0.954	0.927				0.952	0.861	0.91	0.909
	0.818	0.939	0.807	0.939	1.009	0.982	1.011	0.897	0.9	0.902	0.913	0.966	0.946				0.904	0.834	0.93	0.912
	0.903	0.93	0.819	0.925	1.015	1.024	1.015	0.925	0.93	0.947	0.911	0.923	0.909				0.922	0.844	0.946	0.938
	0.923	0.974	0.768	0.885	0.997	0.976	1.008	0.915	0.928	0.932	0.932	0.949	0.926				0.9	0.839	0.928	0.941
	0.852	0.925	0.746	0.9	0.991	0.973	0.974	0.88	0.926	0.913	0.955	0.929	0.905				0.918	0.787	0.935	0.902
	0.833	0.94	0.716	0.915	0.986	0.983	0.981	0.866	0.907	0.899	0.924	0.902	0.877				0.95	0.836	0.923	0.917
	0.848	0.917	0.83	0.89	0.979	1.013	0.993	0.826	0.925	0.911	0.931	0.882	0.891				0.918	0.848	0.905	0.912
	0.821	0.954	0.862	0.909	0.953	0.984	0.981	0.876	0.925	0.907	0.893	0.927	0.91				0.922	0.884	0.918	0.907
	0.828	0.938	0.842	0.937	0.952	1.017	1.004	0.89	0.878	0.888	0.84	0.915	0.854				0.946	0.84	0.872	0.889
	0.781	0.943	0.854	0.943	1.003	0.984	0.989	0.885	0.954	0.86	0.795	0.925	0.912				0.939	0.863	0.871	0.837
	0.734	0.894	0.781	0.929	0.998	1.001	1	0.835	0.959	0.832	0.855	0.945	0.939				0.903	0.846	0.879	0.839
	0.72	0.797	0.813	0.923	0.968	0.988	1.005	0.809	0.914	0.869	0.847	0.964	0.944				0.909	0.866	0.881	0.863
	0.849	0.839	0.848	0.921	0.92	1	0.975	0.798	0.944	0.929	0.862	0.944	0.943				0.88	0.871	0.876	0.92
	0.904	0.904	0.854	0.954	0.972	1	0.991	0.802	0.913	0.876	0.916	0.949	0.911				0.888	0.837	0.863	0.86
	0.839	0.874	0.798	0.943	0.963	1.031	0.981	0.791	0.941	0.904	0.87	0.94	0.927				0.938	0.862	0.892	0.908
	0.818	0.875	0.796	0.97	0.946	1.016	0.983	0.801	0.877	0.913	0.868	0.926	0.926				0.923	0.896	0.894	0.903
	0.766	0.915	0.808	0.971	0.895	0.997	1.001	0.814	0.889	0.846	0.924	0.941	0.942				0.905	0.885	0.841	0.882
	0.845	0.872	0.877	0.944	0.863	0.984	1.008	0.792	0.856	0.847	0.851	0.969	0.914				0.913	0.9	0.877	0.879
	0.849	0.852	0.843	0.925	0.839	0.958	0.936	0.828	0.852	0.904	0.855	0.934	0.906				0.913	0.887	0.869	0.89
	0.857	0.891	0.858	0.956	0.86	1.003	0.931	0.818	0.837	0.862	0.847	0.939	0.934				0.884	0.917	0.865	0.892
	0.932	0.904	0.849	0.935	0.944	0.978	0.919	0.819	0.838	0.91	0.815	0.89	0.905				0.884	0.887	0.861	0.895
	0.862	0.941	0.82	0.944	0.96	0.931	0.959	0.775	0.808	0.88	0.839	0.883	0.873				0.822	0.92	0.809	0.914
0.911	1.014	0.994	0.907	0.944	1.017	1.051	1.016	0.81	0.845	0.871	0.916	0.975	0.946	0.787	0.875	0.732	0.906	0.876	0.976	0.909
0.956	1.008	0.982	0.966	0.977	1.032	1.066	1.037	0.874	0.905	0.93	0.941	0.991	0.962	0.768	0.905	0.822	0.945	0.911	0.963	0.959
0.931	1.004	1.016	0.989	0.978	1.031	1.057	1.033	0.944	0.949	0.975	0.978	1.007	0.977	0.845	0.916	0.889	0.949	0.9	0.945	0.967
0.986	1.017	1.008	0.97	0.967	1.071	1.115	1.079	0.948	0.917	1.002	1.001	1.002	0.957	0.909	0.92	0.903	0.976	0.909	1.012	0.984
0.961	1.001	1	0.975	0.963	1.04	1.063	1.045	0.934	0.968	1.009	0.99	1.001	1.012	0.895	0.898	0.939	0.98	0.93	0.974	0.971
0.941	1.028	1.037	0.968	0.967	1.014	1.072	1.008	0.945	0.991	1.001	1.01	1.035	0.991	0.913	0.941	0.935	0.971	0.931	0.987	0.972
0.952	0.992	0.995	0.968	0.95	0.995	1.08	1.065	0.964	0.997	1.015	0.992	0.99	0.979	0.929	0.94	0.932	0.986	0.939	0.994	0.973
0.989	0.99	0.995	0.937	0.939	0.982	1.065	1.072	0.973	0.996	0.988	1.01	0.988	0.975	0.941	0.969	0.965	1.032	0.934	0.972	0.955
0.959	1.005	0.989	0.982	0.923	0.968	1.088	1.048	0.957	1.027	0.995	0.993	0.986	0.943	0.911	0.971	0.97	1.032	0.924	1.009	0.974
0.975	0.999	0.978	0.973	0.951	0.928	1.031	1.068	0.98	1.001	0.996	1.013	1.031	0.953	0.925	0.911	0.915	0.999	0.919	0.978	1.005
0.91	0.997	1.033	0.962	0.964	0.919	1.059	1.042	0.943	1.006	0.997	0.968	1.024	0.966	0.908	0.957	0.949	0.974	0.934	0.99	0.97
0.936	1.031	0.998	0.952	0.931	0.91	1.138	1.057	0.979	0.999	0.982	0.982	1.017	0.938	0.91	0.965	0.943	1.001	0.939	0.99	0.966
0.968	1.009	0.981	0.983	0.957	0.911	1.112	1.081	0.973	1.026	1.011	1.035	0.995	0.953	0.922	0.949	0.962	0.978	0.923	0.973	1.019
0.964	1.022	1.013	0.992	0.959	0.832	1.108	1.046	0.947	0.997	0.978	1.005	1.018	0.97	0.925	0.938	0.964	0.978	0.92	0.982	0.983
0.955	1.012	0.981	0.988	0.986	0.826	1.056	1.058	0.955	0.984	0.981	0.994	0.995	0.953	0.919	0.924	0.963	0.958	0.882	0.987	0.963
0.989	1.016	1.019	0.983	0.957	0.84	1.104	1.032	0.964	0.98	0.988	0.988	0.999	0.971	0.954	0.939	0.951	0.952	0.904	0.996	0.977
0.983	1.032	1.034	0.984	0.951	0.911	1.071	1.02	0.956	1.006	0.979	0.981	1.001	0.976	0.942	0.933	0.937	0.969	0.904	0.977	0.978
0.98	1.06	1.022	0.98	0.961	0.997	1.065	1.035	0.953	0.989	0.986	1.007	1	0.974	0.917	0.926	0.921	0.973	0.936	0.986	0.982
0.991	1.028	1.027	0.975	0.919	1.007	1.049	1.029	0.961	0.988	0.985	1.004	0.984	0.956	0.931	0.945	0.956	0.974	0.918	0.954	0.985
0.974	1.026	1.038	0.958	0.971	1.036	1.069	1.021	0.976	1.003	1.003	1.019	1.009	0.971	0.935	0.924	0.952	0.991	0.907	0.967	1.006
0.96	1.03	1.039	0.97	0.956	1.049	1.046	1.049	0.979	1.002	1.002	1.01	0.997	1.001	0.965	0.937	0.902	0.976	0.914	0.995	0.99
0.95	1.011	1.024	1.012	0.935	1.041	1.06	1.081	0.969	0.991	1.003	1.009	1.032	0.998	0.949	0.944	0.974	1.004	0.932	0.967	0.987

0.951	1.037	1.027	1.012	0.973	1.023	1.093	1.069	0.982	1	1.025	1.039	1.021	1.014	0.932	0.921	0.957	0.992	0.965	0.964	1.011
0.95	1.045	1.023	1	0.984	1.024	1.055	1.075	0.979	1.031	1.008	1.019	0.997	0.983	0.929	0.946	0.958	1.011	0.937	0.987	1.006
0.965	1.008	1.03	0.999	1.01	1.04	1.075	1.058	0.993	1.022	1.014	1.024	1.033	1.019	0.95	0.971	0.968	0.983	0.929	0.979	1.028
0.936	1.052	1.014	1.005	0.999	1.036	1.082	1.055	1.024	1.036	1.023	1.069	1.018	1.004	0.911	0.953	0.944	0.993	0.942	0.985	1.01
0.897	1.065	1.021	1.018	0.985	1.05	1.084	1.086	0.994	1.032	1.011	1.026	0.994	0.994	0.955	0.937	0.932	0.98	0.942	0.984	0.999
0.954	1.056	1.032	1.018	0.969	1.063	1.069	1.066	1.002	1.041	1.016	1.046	1.024	0.982	0.946	0.944	0.977	0.997	0.931	1.001	1.018
0.943	1.051	1.015	1.004	0.961	1.026	1.033	1.034	1.02	1.035	1.024	1.035	1.02	0.992	0.979	0.952	0.949	0.994	0.955	0.981	0.991
0.923	1.021	0.997	0.987	0.977	1.035	1.026	1.043	1.002	1.015	1.02	1.015	1.003	0.995	0.939	0.935	0.958	1.018	0.927	0.992	0.992
0.947	1.043	0.993	0.974	0.946	1.029	1.015	1.045	1.004	1.006	1.029	1.035	1.023	0.999	0.936	0.964	1.002	0.998	0.95	0.994	0.979
0.951	1.007	0.945	0.98	1.003	1.033	0.987	1.056	0.999	1.007	1.021	1.054	1	0.96	0.932	0.952	0.958	1.02	0.942	0.992	1.017
0.944	1.028	0.963	0.985	1.018	1.057	1.008	1.068	0.994	0.994	1.012	1.037	1.024	0.994	0.963	0.966	0.947	1.001	0.929	0.966	0.993
0.961	0.972	0.916	0.972	0.945	1.051	1.054	1.064	1.02	1.023	1.006	1.036	1.02	0.973	0.963	0.965	0.936	1	0.951	0.968	1.008
0.957	0.956	0.865	1.003	0.988	1.059	1.079	1.05	0.998	1.018	1.009	1.058	1.031	0.954	0.931	0.931	0.947	1.005	0.924	0.988	1.01
0.932	0.984	1.007	0.953	0.994	1.069	1.063	1.103	0.973	1.023	1.011	1.023	1.031	0.967	0.937	0.909	0.938	0.985	0.972	0.983	1.021
0.965	1.004	0.972	0.969	0.964	1.063	1.091	1.063	0.992	1.017	1.002	1.033	1.019	0.968	0.935	0.922	0.904	0.994	0.936	1.004	0.994
0.956	1.028	0.902	0.956	0.966	1.046	1.064	1.093	1.012	0.99	0.997	1.028	1.012	0.959	0.973	0.948	0.904	0.988	0.921	0.988	1.027
0.935	0.947	0.842	0.967	0.995	1.061	1.057	1.069	1.029	1.02	0.946	1.04	1.024	0.947	0.966	0.945	0.927	1.003	0.946	0.996	1.026
0.915	0.968	1.001	0.951	0.999	1.057	1.01	1.062	0.979	1.014	0.924	1.064	1.038	0.973	0.959	0.917	0.937	0.991	0.97	0.99	1.019
0.867	1.011	0.986	0.951	0.976	1.072	1.064	1.101	0.999	1.014	0.926	1.01	1.033	1	0.964	0.93	0.97	1.038	0.988	0.974	1.015
0.947	1.012	0.973	0.912	0.963	1.089	1.093	1.068	0.995	1.024	0.971	1.058	1.035	0.98	0.984	0.916	0.973	0.974	0.943	0.995	1.03
0.923	1.049	0.924	0.969	0.965	1.086	1.102	1.093	1.014	1.034	0.99	1.034	0.989	0.954	0.952	0.915	0.96	0.979	0.994	0.983	1.007
0.916	1.022	0.885	0.978	0.94	1.097	1.081	1.059	1.005	1.03	1.004	1.017	0.995	1.011	0.986	0.954	0.962	0.936	0.938	0.981	1.021
0.932	0.972	0.967	0.939	0.966	1.109	1.109	1.093	1.028	1.045	1.025	1.042	0.977	1.04	0.961	0.954	0.956	0.963	0.973	0.999	0.996
0.995	1.011	0.992	0.914	0.988	1.05	1.072	1.077	1.006	1.06	1.027	1.045	1.008	1.02	0.927	0.978	0.96	0.995	0.943	1.02	1.023
1.013	1.054	0.891	0.944	0.921	1.1	1.088	1.1	1.022	1.055	1.023	1.013	1.023	0.996	0.942	0.989	0.915	1.057	0.976	1.027	0.984
0.934	1.069	0.857	0.97	0.92	1.086	1.119	1.104	1.009	1.046	1.033	1.002	1.042	1.003	0.938	0.953	0.916	1.039	0.938	1.015	1.044
0.961	0.948	0.874	0.955	0.938	1.062	1.085	1.068	1.009	1.028	1.039	1.033	1.053	0.994	0.843	1.047	1.011	1.021	0.941	1.005	0.995
0.996	0.912	0.847	0.933	1.012	1.069	1.068	1.059	1.019	1.011	1.055	1.042	1.065	1.009	0.881	0.948	0.958	1.039	0.944	1.016	1.033
1.005	0.909	0.814	0.873	0.961	1.048	1.076	1.105	1.041	1.054	1.069	1.061	1.058	0.979	0.882	0.913	0.905	1.015	0.943	1.025	1.024
0.96	0.826	0.856	0.88	1.034	1.057	1.041	1.076	1.044	1.042	1.069	1.072	1.082	1.037	0.819	0.925	0.902	1.03	0.936	1.059	1.058
1.023	0.89	0.969	0.929	0.972	1.051	0.983	1.066	1.037	1.035	1.008	1.031	1.113	1.057	0.883	0.875	0.925	1.051	0.944	1.046	1.037
0.984	0.865	0.997	0.93	0.959	1.054	0.986	1.086	1.007	1.031	1.018	1.052	1.042	0.996	0.837	0.817	0.895	1.025	0.984	1.063	1.032
1.006	0.8	0.933	0.918	0.961	1.028	1.079	1.087	1.011	1.023	1.029	1.036	1.109	1.025	0.916	0.786	0.919	1.019	0.997	1.04	1.048
1.023	0.89	0.92	0.856	0.983	1.048	1.083	1.09	0.99	1.027	1.069	1.031	1.098	1.027	0.934	0.771	0.859	1.056	1.008	1.032	1.048
0.958	0.936	0.935	0.982	0.997	1.058	1.077	1.096	0.984	1.084	1.042	1.052	1.074	1.024	0.934	0.764	0.932	1.03	1.01	1.086	1.058
0.981	0.933	1.017	0.929	0.995	1.042	1.079	1.103	0.992	1.043	1.036	1.042	1.104	1.059	0.97	0.839	0.913	1.057	1.031	1.035	1.029
0.98	0.863	1.001	0.923	1.03	1.029	1.094	1.106	1.008	1.027	1.028	1.018	1.074	1.039	0.97	0.921	0.909	0.987	0.971	1.06	1.038
0.941	0.817	1.032	0.964	0.989	1.034	1.095	1.077	0.993	1.002	1.022	1.02	1.022	1.03	0.943	0.936	0.955	0.989	0.986	1.032	1.026
0.972	0.781	0.914	0.958	0.99	1.089	1.083	1.061	0.988	0.953	1.041	1.013	1.094	1.04	0.88	0.926	0.953	1.026	1.008	1.049	1.019
0.907	0.795	0.914	0.926	0.99	1.078	1	1.051	0.986	0.926	1.005	0.981	1.112	1.018	0.922	0.907	0.94	1.001	1.019	1.089	1.035
0.876	0.867	1.009	0.953	1.003	1.057	1.043	1.096	0.983	0.934	1	0.975	1.074	1.011	0.967	0.934	0.922	0.964	0.993	1.073	1.058
0.868	1.005	1.03	0.918	1.021	1.054	1.099	1.107	0.977	0.956	1.009	1.034	1.036	1	0.927	0.932	0.929	0.9	1.005	1.035	1.057
0.96	1.002	1.037	0.873	1.01	1.094	1.126	1.069	0.944	0.965	1.021	0.922	1.046	1.022	0.903	0.905	0.863	0.996	0.971	1.082	1.05
0.953	0.893	1.034	0.948	0.989	1.099	1.095	1.083	0.971	0.921	1.06	0.953	1.081	1.028	0.925	0.917	0.85	1.079	0.964	1.056	1.02
0.909	0.879	0.966	0.97	0.969	1.099	1.152	1.069	0.986	0.883	0.994	0.881	1.071	1.025	0.957	0.886	0.95	1.023	0.964	1.06	1.019
0.95	1.019	1.025	0.971	1.027	1.086	1.138	1.068	0.967	0.864	0.998	0.965	1.093	1.006	0.957	0.759	0.974	1.058	0.952	1.046	1.052
0.931	1.089	0.996	0.931	1.013	1.064	1.083	1.066	0.999	0.923	1.018	1.05	1.046	1.039	0.982	0.824	0.985	1.039	0.916	0.995	1.061
0.907	1.05	1.019	1.003	1.013	1.094	1.088	1.067	1.026	0.949	0.999	1.022	1.027	1.044	0.987	0.876	0.946	1.111	0.958	1.007	1.047
0.908	1.047	1.006	1.001	0.95	1.155	1.101	1.071	0.971	0.905	1.025	1.044	1.093	1.069	0.972	1.013	0.956	1.112	0.919	1.042	1.035
0.928	1.019	1.009	1.051	1.03	1.101	1.094	1.012	0.997	0.897	1.059	1.077	1.085	1.092	0.986	0.97	0.938	1.05	0.943	1.016	1.051
0.906	1.059	1.056	1.009	0.974	1.081	1.062	1.035	0.945	1.027	1.076	1.096	1.064	1.066	0.959	0.989	0.99	1.038	0.937	1.033	1.051
0.895	1.078	1.019	1.007	0.967	1.066	1.013	1.072	0.978	1.038	1.097	1.079	1.106	1.017	0.99	0.899	0.906	0.971	0.945	1.028	1.031
0.877	1.087	0.999	1.006	0.981	1.037	1.056	1.054	1.052	1.089	1.044	1.084	1.117	1.032	0.983	0.936	0.936	1.025	0.928	0.994	1.076
0.868	1.081	0.938	0.988	0.969	1.001	1.102	1.084	1.01	1.117	1.032	1.081	1.118	1.054	1	0.819	0.869	1.058	0.985	1.056	1.013
0.941	1.087	0.888	0.986	1.004	0.922	1.039	1.071	0.978	1.109	1.052	1.108	1.104	1.061	1.019	0.861	0.958	1.087	0.938	0.964	1.005
0.985	1.105	0.978	1.018	1.059	1.004	1.057	1.104	1.026	1.039	1.073	1.065	1.107	1.077	1.016	0.95	0.983	1.056	0.944	1	1.028
0.994	1.054	0.971	0.977	1.033	1.093	1.052	1.132	1.045	1.031	1.041	1.072	1.078	1.045	0.968	0.915	1.001	1.007	0.955	1.009	1.051
0.973	1.04	0.926	1.026	1.033	1.083	1.026	1.08	1.002	1.036	1.03	1.084	1.05	1.042	0.989	0.995	1.009	0.984	0.973	1.027	0.984
0.983	1.004	0.866	1.006	1.003	1.116	1.017	1.047	0.997	1.031	1.003	1.057	1.036	1.039	1.018	1.003	1.001	0.973	0.966	1.009	0.998
0.983	1.042	0.834	1.026	1.016	1.068	1.038	1.028	1.005	1.053	1.021	1.068	1.058	1.056	1.001	0.996	1.008	1.013	0.861	1.001	1.022
1.016	1.024	0.786	0.99	1.036	1.116	1.036	0.97	1.009	1.021	1.047	1.072	1.06	1.053	1.007	1.039	0.988	1.027	0.829	1.023	1.02
1.004	1.036	0.91	1.008	1.019	1.085	1.037	0.993	1.037	1.005	1.051	1.109	1.049	1.026	0.986	0.991	1.003	1.059	0.894	1.012	1.048
1.017	1.092	0.963	1.005	1.003	1.05	1.005	0.993	1.042	1.003	1.053	1.076	1.055	1.043	1.033	1.013	1	1.092	0.878	1.047	1.067
0.969	1.019	1.01	1.015	1.061	1.086	1.028	0.982	1.034	1.029	1.063	1.109	1.073	1.013	1.022	1.013	0.986	1.075	0.94	1.047	1.031
0.987	1.036	0.979	0.988	1.07	1.082	1.045	0.987	1.049	1.049	1.055	1.055	1.095	1.054	1.04	1.013	0.977	1.066	0.925	1.008	1.017
0.977	1.04	0.991	1.02	1.03	1.062	1.041	0.982	1.005	1.053	1.04	1.028	1.08	1.028	1.021	0.983	0.983	1.063	0.921	1.016	0.948
0.911	1.039	0.921	0.98	1.047	1.076	1.028	1.025	1.031	1.081	1.043	1.031	1.07	1.006	0.991	0.989	0.999	1.098	0.93	1.056	1.024
0.998	1.021	0.864	1.01	1.068	1.072	1.057	1.016	1.027	1.066	1.046	1.014	1.085	1.005	0.979	0.958	1	1.044	0.948	1.042	1.018
1.053	0.991	0.925	0.979	1.053	1.063	1.043	1.093	1	1.068	1.051	1.077	1.029	1.009	0.947	0.946	1.011	1.035	0.934	1.054	1.02
1.026	0.926	0.815	0.963	1.049	1.071	1.023	1.068	1.02	1.059	1.053	1.074	1.02	0.927	0.951	0.886	1.001	1.028	0.95	1.014	1.011
1.028	0.943	0.803	0.914	1.035	1.099	1.09	1.059	1.045	1.008	1.043	1.037	1.005	0.93	0.921	0.888	0.953	1.01	0.975	1.021	1.002
1.027	0.991	0.947	0.939	1.035	1.083	1.104	1.027	1.024	1.023	1.045	1.048	1.002	0.885	0.945	0.931	0.958	1.028	0.925	1.032	0.96
1.021	1.032	0.888	0.994	1.016	1.089	1.08	1.014	1.026	1.034	1.063	1.033	0.977	0.839	0.905	0.933	0.952	1.01	0.931	1.025	0.952
0.997	0.982	0.862	0.986	1.073	1.039	1.069	1.011	1.004	0.984	1.06	0.998	0.989	0.935	0.893	0.913	0.933	0.997	0.962	1.032	0.931
0.961	0.945	0.821	0.882	1.018	1.05	1.062	1.026	1.022	1.003	1.045	1.04	1.028	0.9	0.85	0.961	0.93	0.961	0.932	1.021	0.944
0.87	0.934	0.841	0.874	1.037	1.08	1.031	1.054	1.02	1.001	1.018	1.044	1.042	0.855	0.888	0.965	0.952	0.943	0.909	1.023	0.884
0.781	0.882	0.853	0.821	0.997	1.099	1.121	1.046	0.994	0.949	0.954	0.94	1.041	0.921	0.968	0.949	0.974	0.919	0.961	1.024	0.859
0.802	0.816	0.831	0.804	1.059	1.088	1.028	1.092	0.984	0.928	0.95	0.904	0.989	0.951	0.929	0.939	0.973	0.921	0.958	1.024	0.926
0.95	0.819	0.808	0.789	1.018	1.06	0.977	1.08	0.989	0.885	0.881	0.847	0.971	0.921	0.973	0.899	0.984	0.982	0.945	1.009	0.953
0.981	0.797	0.785	0.798	1.067	1.016	0.961	1.105	1.004	0.848	0.86	0.8	0.974	0.861	0.97	0.9	0.949	1.021	0.916	1.036	0.912
0.998	0.821	0.818	0.756	1.023	1.013	0.881	1.092	0.928	0.884	0.823	0.904	1.035	0.896	1.006	0.935	0.899	0.987	0.873	0.994	0.841
0.97	0.839	0.978	0.758	1.005	0.998	0.881	1.068	0.966	0.993	0.99	0.864	1.027	0.862	0.967	1.013	0.814	0.975	0.812	1.029	0.873
0.982	0.893	0.946	0.775	0.957	0.929	0.963	1.058	0.933	1.048	1.011	0.87	1	0.806	1.017	0.97	0.85	0.968	0.89	0.989	0.794
0.916	0.803	0.859	0.747	0.959	0.974	0.939	1.047	0.903	1.039	0.956	0.899	0.972	0.791	1.01	0.98	0.928	1.042	0.945	0.99	0.787
0.922	0.941	0.925	0.75	0.964	0.939	0.898	1.092	0.999	1.085	0.934	1.021	0.949	0.924	0.988	0.952	0.857	1.065	0.831	0.976	0.925
1.001	0.938	0.846	0.73	0.915	0.914	0.89	1.087	0.99	1.056	1.025	0.979	0.893	0.972	0.992	0.98	0.738	1.026	0.882	0.94	0.996
0.977	0.998	0.844	0.722	1.003	0.889	0.932	1.108	0.947	1.023	1.002	0.938	0.817	1.005	0.995	1.032	0.771	1.096	0.908	0.869	1.027
1.041	0.948	0.97	0.71	1.028	0.859	1.004	1.098	0.954	0.995	0.982	0.985	0.907	1.001	0.955	0.974	0.953	1.021	0.96	0.868	1.005
1.019	0.956	0.928	0.741	1.049	0.85	0.908	1.102	0.923	0.957	0.916	0.975	0.999	1.006	0.961	0.988	0.947	1.092	0.997	0.943	0.98
1.001	0.921	0.9	0.794	1.056	0.855	0.948	1.125	0.848	0.953	0.92	0.957	0.987	1.011	0.983	0.891	0.897	1.103	1.023	0.954	0.974
0.975	0.853	1.068	0.744	1.073	0.801	1.012	1.06	0.79	1.033	0.857	0.912	1.025	1.021	0.942	0.933	0.845	1.005	0.983	0.963	0.965
0.784	0.832	1.005	0.751	1.059	0.969	0.932	1.063	0.759	1.042	0.847	1.019	1.063	1.056	0.891	1.002	0.783	1.075	1.039	0.976	0.883
0.725	0.774	0.937	0.735	1.047	1.02	0.924	1.032	0.88	1.107	0.847	1.035	1.007	1.073	0.838	1.008	0.792	0.997	0.984	0.919	0.825
0.816	0.804	0.97	0.838	1.017	0.992	0.881	1.035	0.956	1.029	0.9	1.018	0.998	1.076	0.905	0.981	0.852	0.995	1.018	0.969	0.912
0.924	0.961	0.901	0.93	1.02	0.956	0.826	1.098	0.912	1.027	0.963	0.994	0.887	1.05	0.874	0.975	0.886	1.069	0.959	1.045	0.881
0.939	0.943	0.854	0.864	1.038	1.075	0.899	1.065	0.967	1.037	1.012	1.012	0.867	1.046	0.889	0.916	0.761	1.096	0.98	1.028	0.988
0.838	0.811	0.985	0.833	1.062	1.096	0.983	1.075	0.902	0.958	0.956	1.006	0.926	1.032	0.838	0.922	0.715	1.071	0.985	1.044	0.972
0.835	0.765	1.038	0.794	0.956	1.037	0.92	1.161	0.961	1.006	1.003	0.957	0.894	1.053	0.795	0.986	0.856	1.009	0.997	1.038	1.009
0.862	0.724	1.029	0.732	1.009	0.987	0.875	1.09	0.884	0.939	1.033	0.958	0.832	1.047	0.776	1.012	0.907	1.026	0.993	1.052	1.008
0.766	0.848	1.032	0.8	1.074	1.053	0.924	1.072	0.952	0.88	1.021	1.052	0.796	1.006	0.82	1.068	0.785	1.035	0.969	1.042	1.049
0.766	0.815	0.919	0.934	1.04	0.991	1.081	1.052	0.897	0.926	0.986	1.03	0.789	1.012	0.777	1.047	0.758	0.959	0.934	1.029	1.042
0.859	0.795	0.871	0.844	1.002	0.998	1.109	1.064	0.91	0.998	1.032	1.059	0.806	1.062	0.747	1.019	0.795	0.992	0.936	1.061	1.038
0.855	0.926	0.964	0.859	1.002	1.072	1	1.069	0.924	0.955	1.046	1.063	0.798	1.013	0.763	1.023	0.78	1.06	0.954	1.033	1.034
0.818	0.94	1.067	0.81	0.923	0.971	1.013	0.963	0.933	1.026	1.075	1.065	0.824	0.992	0.722			1.079	0.909	0.991	1.009
0.783	0.88	1.039	0.821	0.929	0.949	0.962	0.983	0.887	1.074	1.064	1.031	0.79	0.997	0.772			1.109	0.915	1.016	1.035

0.92	0.869	0.987	0.825	0.889	0.905	0.956	0.962	0.872	1.047	1.011	0.961	0.774	0.938	0.72	1.078	0.945	1.013	0.972
0.955	0.877	1.049	0.976	0.857	0.865	0.988	0.954	0.935	0.993	0.89	1.039	0.8	0.986	0.702	1.078	0.99	0.965	0.921
0.888	1.011	1.088	0.933	0.861	0.871	1.086	0.922	0.945	1.019	0.949	1.066	0.792	0.986	0.759	1	0.915	0.983	0.857
	0.907	1.028	0.911	0.951	0.864	1.039	0.965	0.979	0.963	1.02	1.037	0.767	0.957		1.014	0.925	0.956	0.923
	0.809	1.038	0.905	1	0.863	0.961	0.941	1.033	0.927	0.952	0.991	0.795	0.946		0.995	0.959	1.007	0.983
	0.789	1.104	0.849	1.017	1.011	0.965	1.056	1.009	0.889	0.882	1.021	0.803	0.919		1.025	0.872	0.909	0.991
	0.783	1.141	0.795	1.05	1.117	0.912	1.064	0.985	0.885	0.971	0.952	0.883	0.912		0.928	0.855	0.99	0.979
	0.864	1.098	0.814	0.973	1.124	0.864	0.996	0.979	0.911	0.964	0.999	0.859	0.957		0.877	0.819	0.942	1.023
	1	1.043	0.945	0.989	1.127	0.937	1.03	0.972	0.983	0.96	1.051	0.88	1.011		0.955	0.904	0.945	0.989
	1.041	1.08	0.992	0.976	1.044	1.082	0.986	0.931	1.058	0.982	1.055	1.051	1.041		1.013	0.94	0.938	0.948
	0.984	1.072	0.994	1.035	1.125	1.142	1.011	0.954	1.049	1.003	1.039	1.026	1.054		0.986	0.89	0.913	1.07
	0.935	1.052	0.939	1.061	1.067	1.089	0.95	1.019	1.012	1.001	1.077	1.072	1.053		0.986	0.89	0.906	1.087
	0.925	1.015	0.868	1.06	1.1	1.116	1.011	1.003	1.001	1.067	1.112	1.087	1.077		0.963	0.911	0.976	1.031
	0.995	1.032	0.921	1.141	1.159	1.027	1.043	0.992	0.963	1.055	1.071	1.096	1.067		0.905	0.971	1.04	1.056
	1.054	1.007	1.012	1.08	1.145	1.087	1.07	1.015	0.919	1.034	1.052	1.091	1.086		0.929	0.932	1.063	1.043
	1.045	1.001	1.047	1.03	1.132	1.122	1.025	0.999	0.927	1.067	1.08	1.065	1.074		1.008	0.958	1.062	1.042
	0.96	1.011	1.082	1.012	1.111	1.109	1.031	1.005	0.922	1.06	1.04	1.083	1.051		0.934	0.967	1.048	0.989
	1.016	0.985	1.026	0.997	1.078	1.1	1.035	0.991	0.891	1.033	1.019	1.078	1.04		0.876	0.947	0.996	1.009
	1.003	0.941	1.025	0.995	1.088	1.038	1.021	0.984	0.948	1.015	1.024	1.07	1.037		0.896	0.955	0.97	1.019
	0.978	0.918	0.999	1.035	1.089	1.014	1.045	0.994	0.913	1.037	0.975	1.044	1.016		0.96	0.96	0.992	1.05
	0.969	0.891	0.989	1.004	1.104	1.014	1.057	0.995	0.908	1.049	1.03	1.067	1.005		0.944	0.925	0.938	1.03
	0.964	0.815	0.974	1.03	1.081	1.065	1.071	1.06	0.977	1.088	1.1	1.083	1.012		1	0.946	1.003	1.09
	0.903	0.792	1.015	1.012	1.117	1.111	1.072	1.051	1.022	1.073	1.069	1.085	1.025		1.022	0.992	1.033	1.05
	0.897	0.766	1.009	1.018	1.08	1.051	1.066	1.041	1.047	1.044	1.055	1.109	1.089		1.039	0.955	1.024	1.043
	0.806	0.726	1.028	1.045	1.058	1.028	1.057	1.053	1.01	1.056	1.063	1.065	1.066		1.108	0.981	1.024	1.02
	0.835	0.883	1.006	1.044	1.026	1.015	1.052	1.017	0.997	1.047	1.037	1.057	1.044		1.064	0.963	1.027	0.994
	0.833	0.946	1.025	1.036	1.022	0.987	1.135	1.026	0.969	1.01	0.994	1.068	1.016		1.042	0.969	1.021	0.978
	0.865	0.971	1.032	1.051	1.037	0.941	1.113	1.046	0.963	1.018	1.039	1.074	1.014		0.938	0.938	1.032	1.023
	0.955	0.941	1.017	1.01	1.084	0.917	1.075	0.997	1.001	1.012	0.999	1.075	0.976		0.991	0.931	1.024	0.953
	0.953	0.88	1.019	1.049	1.04	0.866	1.036	1.028	0.936	0.997	0.986	1.042	0.936		1.032	0.939	1.004	0.914
	0.958	0.822	1.025	1.077	1.043	0.975	1.056	1.015	0.95	0.991	1.093	1.008	0.869		0.974	0.954	1.004	0.893
	0.915	1.002	1.006	1.037	1	1.059	1.069	0.992	0.874	0.973	1.032	0.995	0.844		0.92	0.999	0.994	0.923
	0.874	1.047	0.971	1.066	1.005	1.066	1.058	0.955	0.853	0.944	0.948	1.02	0.814		0.905	1.001	0.978	0.964
	0.828	0.929	0.961	1.064	0.948	1.058	1.047	0.973	0.906	0.899	0.899	0.971	0.824		0.845	0.969	0.957	0.919
	0.883	1.091	0.954	1.047	0.993	1.024	1.019	0.952	0.876	0.873	0.851	0.912	0.802		0.788	0.98	0.951	0.914
	0.901	1.034	0.976	1.048	0.993	0.991	1.005	0.952	0.769	0.885	0.836	0.875	0.757		0.774	0.941	0.91	0.934
	0.925	0.957	0.964	1.081	0.95	0.952	1.036	0.969	0.853	0.861	0.938	0.975	0.916		0.828	0.919	0.895	0.902
	0.995	0.953	0.906	1.012	0.944	1.048	1.07	0.924	1.008	0.829	1.004	0.986	1.028		0.931	0.924	0.866	0.936
	0.975	0.903	0.91	1.049	0.875	1.06	1.007	0.892	0.961	0.89	0.96	0.956	1.012		0.88	0.953	0.948	0.937
	0.943	0.909	0.888	1.035	0.857	1.014	0.967	0.886	1.004	0.865	0.994	1.032	0.971		0.86	0.961	0.969	0.914
	0.89	0.844	0.882	1.079	0.966	1.013	0.974	0.918	0.999	0.823	0.925	1.006	0.942		0.808	0.966	1.01	0.857
	0.785	0.812	0.821	1.04	1.019	0.958	0.963	0.873	0.914	0.892	0.833	0.971	0.886		0.819	0.976	0.975	0.876
	0.817	0.851	0.843	1.048	1.04	0.902	0.934	0.837	0.932	0.882	0.916	0.936	0.856		0.793	0.965	0.952	0.873
	0.811	0.791	0.849	1.017	1.004	0.882	0.939	0.801	0.84	0.846	0.954	0.877	0.794		0.76	0.977	0.92	0.858
	0.896	0.745	0.85	0.983	0.974	0.851	0.902	0.867	0.876	0.796	1.051	0.823	0.984		0.772	0.97	0.865	0.899
	0.876	0.768	0.793	0.87	0.955	0.866	1.011	0.83	0.995	0.773	1.023	0.813	1.052		0.862	0.965	0.822	0.862
	0.844	0.735	0.8	0.964	0.906	0.84	0.991	0.872	0.899	0.762	0.959	0.807	1.018		1.029	1.014	0.847	0.76
	0.751	0.785	0.752	0.939	0.908	0.806	0.963	0.831	0.977	0.903	1.01	0.875	1.023		1.022	0.984	0.839	0.881
	0.715	0.857	0.73	0.964	0.864	0.824	0.949	0.893	0.985	0.926	0.867	0.969	0.982		1.033	0.982	0.789	0.95
	0.696	0.931	0.741	0.973	0.992	0.805	1.001	0.874	0.964	0.896	0.992	1.001	0.976		1.097	0.945	0.79	0.942
	0.718	0.918	0.932	1.014	1.004	0.775	1	0.83	0.955	0.808	0.99	1.034	0.946		1.171	0.942	0.934	0.946
	0.716	0.802	0.99	1.078	1.127	0.81	1.011	0.778	0.912	0.784	0.938	0.937	0.969		1.1	0.97	1.025	1.01
	0.744	0.817	0.966	0.959	1.039	0.873	0.983	0.762	0.987	0.775	1.013	0.908	0.916		1.057	0.907	0.979	0.963
	0.912	0.91	0.941	0.937	1.014	0.836	0.957	0.763	1.028	0.86	0.999	0.854	0.966		1.017	0.983	0.901	0.932
	0.916	0.93	0.854	0.933	1.011	0.852	0.96	0.891	0.998	0.997	1.092	0.956	1.062		1.01	0.934	0.987	0.958
	1.023	0.904	0.812	0.906	0.971	0.836	1.073	0.969	0.937	0.94	1.068	1.059	1.049		1.131	0.913	0.984	0.875
	1.077	0.857	0.766	0.803	0.951	0.83	1.12	0.971	0.886	0.912	1.055	1.004	1.075		1.07	0.938	0.93	0.947
	0.976	0.987	0.803	0.918	0.967	0.897	1.046	0.888	0.908	0.843	1.042	1.111	1.001		1.035	0.893	0.871	0.918
	0.913	0.934	0.778	0.999	0.936	0.885	1.059	0.913	0.905	0.952	0.952	1.092	1.031		0.976	0.903	0.885	0.859
	0.893	0.866	0.763	0.974	0.919	0.86	1.092	0.966	0.845	0.992	1.016	1.099	1.062		0.998	0.907	0.861	0.817
	0.94	0.856	0.737	0.906	1.022	0.865	0.986	0.942	0.802	1.007	1.036	0.99	0.999		0.971	0.833	0.956	0.932
	0.859	0.823	0.748	0.799	0.983	0.984	0.985	0.934	0.919	1.037	0.981	1.037	1		0.933	0.89	0.916	0.883
	0.809	0.799	0.782	0.796	0.9	1.133	1.036	0.851	0.982	1.016	0.907	1.006	0.98		0.921	0.928	0.832	0.884

.	0.984	1.015	0.856	0.908	0.86	0.914	0.92	0.817	0.871	0.815	0.882	0.866	0.873 .	0.885	0.869	0.819	0.88	0.93	0.856
.	0.971	0.908	0.86	0.878	0.859	0.888	0.893	0.788	0.833	0.869	0.842	0.896	0.861 .	0.874	0.89	0.846	0.912	0.912	0.836
.	0.93	0.905	0.872	0.865	0.834	0.789	0.843	0.766	0.87	0.877	0.799	0.834	0.861 .	0.818	0.852	0.821	0.902	0.929	0.815
.	0.926	0.866	0.848	0.851	0.848	0.867	0.827	0.736	0.812	0.815	0.806	0.827	0.788 .	0.824	0.847	0.84	0.868	0.891	0.813
.	0.898	0.848	0.815	0.817	0.814	0.906	0.877	0.856	0.809	0.752	0.8	0.889	0.839 .	0.833	0.819	0.832	0.857	0.9	0.808
.	0.864	0.886	0.816	0.819	0.799	0.836	0.885	0.849	0.896	0.826	0.832	0.85	0.852 .	0.769	0.798	0.843	0.859	0.858	0.794
.	0.843	0.889	0.773	0.774	0.82	0.782	0.895	0.853	0.896	0.863	0.825	0.839	0.867 .	0.816	0.792	0.839	0.869	0.877	0.757
.	0.912	0.888	0.809	0.806	0.803	0.795	0.82	0.791	0.853	0.861	0.764	0.84	0.866 .	0.794	0.853	0.846	0.879	0.893	0.859
.	0.869	0.84	0.865	0.806	0.785	0.812	0.795	0.818	0.862	0.895	0.811	0.848	0.846 .	0.807	0.805	0.828	0.85	0.851	0.858
.	0.851	0.883	0.86	0.824	0.803	0.884	0.867	0.823	0.843	0.891	0.79	0.826	0.834 .	0.854	0.811	0.827	0.826	0.875	0.827
.	0.857	0.872	0.844	0.847	0.788	0.838	0.85	0.81	0.844	0.814	0.78	0.843	0.847 .	0.844	0.82	0.805	0.858	0.856	0.871
.	0.822	0.871	0.848	0.802	0.814	0.875	0.814	0.851	0.821	0.803	0.798	0.849	0.84 .	0.821	0.817	0.803	0.835	0.859	0.805
.	0.821	0.838	0.809	0.779	0.805	0.85	0.826	0.85	0.86	0.861	0.774	0.869	0.861 .	0.799	0.791	0.791	0.8	0.869	0.866
.	0.835	0.789	0.879	0.857	0.814	0.816	0.825	0.822	0.853	0.828	0.825	0.821	0.824 .	0.792	0.81	0.779	0.795	0.839	0.83
.	0.836	0.803	0.809	0.83	0.8	0.803	0.809	0.814	0.822	0.846	0.815	0.808	0.799 .	0.818	0.833	0.777	0.787	0.856	0.824
.	0.835	0.822	0.808	0.782	0.823	0.821	0.786	0.776	0.814	0.878	0.818	0.822	0.821 .	0.787	0.85	0.793	0.772	0.822	0.813
.	0.818	0.803	0.844	0.783	0.822	0.801	0.788	0.804	0.834	0.885	0.844	0.794	0.78 .	0.783	0.833	0.815	0.779	0.842	0.804
.	0.799	0.782	0.836	0.816	0.797	0.79	0.809	0.823	0.826	0.916	0.812	0.809	0.793 .	0.779	0.821	0.833	0.805	0.829	0.83
.	0.798	0.847	0.838	0.788	0.822	0.832	0.802	0.803	0.834	0.921	0.789	0.787	0.795 .	0.808	0.837	0.793	0.806	0.828	0.785
.	0.81	0.792	0.827	0.826	0.794	0.826	0.801	0.835	0.849	0.909	0.822	0.808	0.817 .	0.813	0.807	0.794	0.773	0.853	0.82
.	0.807	0.796	0.807	0.817	0.788	0.801	0.787	0.816	0.832	0.851	0.819	0.827	0.826 .	0.806	0.789	0.82	0.786	0.839	0.809
.	0.843	0.791	0.829	0.793	0.812	0.807	0.765	0.813	0.844	0.855	0.787	0.812	0.814 .	0.789	0.802	0.798	0.773	0.824	0.837
.	0.788	0.791	0.811	0.781	0.789	0.802	0.801	0.812	0.843	0.846	0.786	0.809	0.831 .	0.767	0.812	0.804	0.778	0.801	0.799
.	0.81	0.787	0.791	0.789	0.795	0.799	0.849	0.781	0.821	0.842	0.788	0.785	0.795 .	0.79	0.853	0.804	0.768	0.803	0.789
.	0.816	0.797	0.765	0.789	0.814	0.787	0.812	0.805	0.832	0.873	0.816	0.806	0.8 .	0.816	0.845	0.793	0.768	0.809	0.812
.	0.769	0.79	0.779	0.819	0.823	0.773	0.85	0.809	0.836	0.856	0.81	0.792	0.8 .	0.779	0.793	0.79	0.787	0.811	0.818
.	0.786	0.801	0.837	0.799	0.81	0.794	0.799	0.788	0.828	0.84	0.785	0.819	0.805 .	0.75	0.785	0.796	0.776	0.819	0.825
.	0.738	0.776	0.787	0.795	0.767	0.792	0.762	0.795	0.824	0.812	0.792	0.808	0.819 .	0.804	0.801	0.809	0.788	0.806	0.791
.	0.775	0.802	0.76	0.795	0.783	0.778	0.81	0.778	0.808	0.785	0.799	0.785	0.819 .	0.813	0.775	0.823	0.803	0.82	0.786
.	0.799	0.819	0.752	0.8	0.769	0.763	0.807	0.785	0.823	0.828	0.826	0.808	0.832 .	0.81	0.819	0.806	0.792	0.813	0.807
.	0.807	0.807	0.729	0.782	0.805	0.769	0.793	0.797	0.816	0.802	0.845	0.804	0.81 .	0.77	0.788	0.76	0.792	0.826	0.804
.	0.781	0.793	0.777	0.836	0.821	0.817	0.799	0.791	0.806	0.799	0.83	0.807	0.792 .	0.722	0.776	0.772	0.812	0.843	0.818
.	0.796	0.809	0.802	0.812	0.795	0.804	0.793	0.79	0.824	0.806	0.829	0.808	0.783 .	0.778	0.789	0.786	0.805	0.83	0.797
.	0.764	0.784	0.773	0.8	0.796	0.777	0.773	0.803	0.785	0.813	0.807	0.79	0.791 .	0.803	0.795	0.765	0.814	0.816	0.793
.	0.767	0.799	0.753	0.779	0.795	0.787	0.787	0.756	0.773	0.778	0.783	0.762	0.805 .	0.785	0.801	0.783	0.796	0.795	0.806
.	0.8	0.83	0.742	0.758	0.772	0.759	0.77	0.777	0.79	0.831	0.804	0.779	0.794 .	0.801	0.792	0.778	0.828	0.834	0.795
.	0.779	0.803	0.764	0.83	0.788	0.758	0.767	0.801	0.815	0.805	0.804	0.767	0.818 .	0.76	0.734	0.802	0.796	0.833	0.812
.	0.796	0.819	0.747	0.812	0.764	0.81	0.766	0.793	0.781	0.804	0.811	0.783	0.81 .	0.766	0.793	0.798	0.804	0.811	0.823
.	0.781	0.8	0.828	0.832	0.766	0.84	0.778	0.793	0.791	0.769	0.804	0.789	0.803 .	0.786	0.773	0.768	0.783	0.806	0.846
.	0.782	0.818	0.836	0.821	0.767	0.84	0.801	0.736	0.786	0.769	0.771	0.806	0.81 .	0.764	0.77	0.798	0.821	0.827	0.819
.	0.792	0.827	0.839	0.793	0.824	0.844	0.846	0.817	0.85	0.824	0.794	0.797	0.808 .	0.764	0.79	0.792	0.819	0.825	0.853

.	0.793	0.816	0.811	0.767	0.762	0.796	0.777	0.803	0.815	0.806	0.832	0.791	0.774	.	0.707	0.749	0.79	0.813	0.805	0.83
.	0.791	0.757	0.785	0.777	0.784	0.783	0.775	0.792	0.8	0.816	0.81	0.792	0.819	.	0.743	0.779	0.761	0.794	0.829	0.841
.	0.798	0.788	0.771	0.796	0.783	0.833	0.801	0.799	0.759	0.768	0.776	0.796	0.81	.	0.714	0.777	0.81	0.774	0.811	0.811
.	0.788	0.849	0.79	0.803	0.783	0.808	0.769	0.788	0.786	0.803	0.782	0.792	0.835	.	0.707	0.812	0.822	0.836	0.799	0.812
.	0.812	0.873	0.786	0.855	0.846	0.882	0.819	0.789	0.788	0.792	0.791	0.78	0.808	.	0.694	0.773	0.779	0.796	0.812	0.841
.	0.767	0.857	0.779	0.845	0.836	0.852	0.802	0.77	0.79	0.79	0.779	0.784	0.807	.	0.751	0.749	0.844	0.838	0.805	0.845
.	0.794	0.779	0.759	0.815	0.786	0.819	0.77	0.808	0.757	0.815	0.749	0.797	0.798	.	0.746	0.76	0.795	0.831	0.778	0.855
.	0.837	0.796	0.791	0.772	0.805	0.79	0.792	0.831	0.782	0.836	0.773	0.779	0.841	.	0.789	0.815	0.835	0.835	0.829	0.823
.	0.798	0.818	0.849	0.819	0.787	0.797	0.786	0.858	0.832	0.814	0.753	0.775	0.807	.	0.812	0.816	0.846	0.823	0.844	0.842
.	0.78	0.823	0.821	0.792	0.807	0.827	0.815	0.837	0.842	0.803	0.807	0.8	0.867	.	0.752	0.812	0.789	0.83	0.83	0.873
.	0.846	0.864	0.869	0.81	0.811	0.861	0.801	0.851	0.838	0.807	0.796	0.788	0.83	.	0.767	0.821	0.788	0.822	0.84	0.874
.	0.802	0.829	0.8	0.831	0.785	0.842	0.818	0.832	0.827	0.776	0.792	0.824	0.819	.	0.78	0.829	0.819	0.834	0.776	0.836
.	0.817	0.838	0.814	0.808	0.785	0.817	0.815	0.863	0.84	0.803	0.839	0.847	0.811	.	0.779	0.824	0.833	0.805	0.826	0.827
.	0.75	0.773	0.84	0.799	0.796	0.831	0.832	0.848	0.84	0.817	0.809	0.793	0.844	.	0.801	0.894	0.817	0.835	0.826	0.838
.	0.756	0.823	0.81	0.791	0.768	0.833	0.786	0.839	0.833	0.828	0.805	0.789	0.865	.	0.807	0.857	0.816	0.812	0.809	0.855
.	0.866	0.833	0.793	0.826	0.802	0.822	0.819	0.841	0.808	0.798	0.832	0.758	0.836	.	0.79	0.835	0.804	0.821	0.839	0.853
.	0.823	0.816	0.827	0.819	0.84	0.81	0.825	0.851	0.851	0.858	0.808	0.76	0.824	.	0.801	0.81	0.85	0.805	0.816	0.841
.	0.842	0.828	0.85	0.813	0.824	0.788	0.814	0.834	0.83	0.837	0.82	0.803	0.817	.	0.767	0.796	0.822	0.823	0.853	0.877
.	0.821	0.812	0.862	0.803	0.801	0.842	0.776	0.845	0.863	0.805	0.804	0.789	0.812	.	0.735	0.806	0.803	0.826	0.85	0.853
.	0.79	0.759	0.841	0.835	0.799	0.802	0.784	0.819	0.853	0.876	0.843	0.799	0.804	.	0.752	0.799	0.774	0.841	0.847	0.83
.	0.853	0.755	0.811	0.8	0.779	0.793	0.787	0.838	0.845	0.854	0.832	0.812	0.833	.	0.799	0.785	0.769	0.813	0.879	0.843
.	0.784	0.764	0.83	0.825	0.751	0.86	0.827	0.807	0.852	0.833	0.824	0.828	0.83	.	0.769	0.78	0.76	0.855	0.829	0.837
.	0.803	0.747	0.766	0.819	0.784	0.842	0.841	0.847	0.853	0.801	0.816	0.787	0.781	.	0.741	0.776	0.823	0.845	0.842	0.861
.	0.795	0.801	0.823	0.837	0.86	0.812	0.808	0.824	0.88	0.799	0.794	0.816	0.831	.	0.742	0.795	0.829	0.841	0.859	0.837
.	0.813	0.745	0.866	0.84	0.811	0.793	0.755	0.801	0.846	0.806	0.812	0.783	0.819	.	0.795	0.809	0.836	0.826	0.852	0.837
.	0.796	0.789	0.806	0.808	0.8	0.823	0.794	0.802	0.882	0.846	0.865	0.84	0.824	.	0.788	0.833	0.814	0.834	0.865	0.839
.	0.764	0.784	0.828	0.807	0.802	0.84	0.776	0.826	0.873	0.863	0.806	0.837	0.809	.	0.76	0.812	0.81	0.85	0.834	0.843
.	0.751	0.814	0.794	0.828	0.766	0.801	0.826	0.842	0.832	0.851	0.815	0.855	0.817	.	0.774	0.781	0.804	0.832	0.869	0.852
.	0.745	0.83	0.784	0.831	0.796	0.842	0.831	0.855	0.824	0.858	0.833	0.821	0.822	.	0.739	0.804	0.805	0.835	0.854	0.861
.	0.734	0.802	0.792	0.841	0.778	0.813	0.83	0.801	0.82	0.833	0.837	0.82	0.817	.	0.775	0.852	0.775	0.826	0.858	0.854
.	0.803	0.765	0.801	0.875	0.804	0.823	0.815	0.807	0.851	0.799	0.828	0.826	0.824	.	0.772	0.853	0.765	0.828	0.836	0.83
.	0.809	0.816	0.848	0.816	0.81	0.814	0.832	0.806	0.852	0.848	0.79	0.827	0.783	.	0.801	0.855	0.805	0.841	0.853	0.806
.	0.817	0.808	0.866	0.848	0.86	0.784	0.799	0.82	0.86	0.887	0.823	0.801	0.775	.	0.784	0.847	0.841	0.856	0.853	0.819
.	0.852	0.809	0.864	0.863	0.86	0.886	0.805	0.816	0.858	0.892	0.812	0.783	0.792	.	0.778	0.855	0.811	0.868	0.875	0.817
.	0.792	0.764	0.877	0.849	0.804	0.842	0.806	0.81	0.852	0.859	0.825	0.812	0.816	.	0.79	0.85	0.777	0.827	0.836	0.808
.	0.856	0.849	0.852	0.819	0.82	0.828	0.812	0.828	0.853	0.848	0.825	0.806	0.832	.	0.774	0.844	0.78	0.848	0.84	0.811
.	0.833	0.814	0.862	0.844	0.829	0.833	0.805	0.778	0.818	0.808	0.788	0.798	0.806	.	0.767	0.856	0.784	0.827	0.865	0.816
.	0.807	0.812	0.817	0.797	0.798	0.815	0.804	0.809	0.836	0.863	0.831	0.801	0.826	.	0.766	0.83	0.782	0.834	0.845	0.828
.	0.835	0.793	0.787	0.809	0.81	0.794	0.813	0.804	0.816	0.823	0.832	0.798	0.782	.	0.747	0.85	0.792	0.824	0.83	0.807
.	0.859	0.806	0.764	0.784	0.817	0.783	0.799	0.802	0.796	0.816	0.806	0.78	0.792	.	0.743	0.813	0.803	0.787	0.827	0.824
.	0.82	0.788	0.759	0.771	0.814	0.783	0.791	0.82	0.811	0.829	0.779	0.776	0.789	.	0.696	0.786	0.766	0.783	0.82	0.804
.	0.816	0.78	0.753	0.772	0.823	0.778	0.788	0.815	0.837	0.835	0.792	0.774	0.8	.	0.718	0.802	0.791	0.798	0.855	0.841
.	0.821	0.777	0.768	0.808	0.812	0.789	0.775	0.814	0.838	0.835	0.802	0.775	0.784	.	0.736	0.823	0.804	0.841	0.836	0.826
.	0.853	0.783	0.772	0.816	0.815	0.798	0.784	0.782	0.829	0.818	0.799	0.784	0.772	.	0.706	0.808	0.785	0.799	0.839	0.845
.	0.864	0.759	0.817	0.822	0.791	0.769	0.758	0.805	0.823	0.838	0.818	0.778	0.792	.	0.75	0.784	0.789	0.787	0.834	0.834
.	0.831	0.763	0.797	0.761	0.784	0.785	0.826	0.798	0.817	0.829	0.801	0.751	0.795	.	0.761	0.794	0.776	0.805	0.823	0.836
.	0.84	0.768	0.767	0.792	0.78	0.79	0.805	0.787	0.788	0.842	0.823	0.743	0.812	.	0.767	0.793	0.747	0.769	0.817	0.812
.	0.856	0.791	0.771	0.78	0.783	0.776	0.789	0.827	0.83	0.838	0.803	0.789	0.765	.	0.729	0.746	0.78	0.783	0.811	0.812
.	0.832	0.762	0.741	0.787	0.767	0.792	0.794	0.838	0.828	0.829	0.828	0.8	0.797	.	0.761	0.777	0.752	0.805	0.825	0.799
.	0.84	0.773	0.724	0.773	0.812	0.806	0.808	0.828	0.82	0.787	0.816	0.78	0.803	.	0.736	0.785	0.726	0.819	0.835	0.84
.	0.821	0.827	0.806	0.802	0.808	0.796	0.792	0.805	0.807	0.805	0.797	0.772	0.811	.	0.755	0.822	0.798	0.798	0.817	0.848
.	0.822	0.811	0.773	0.806	0.78	0.798	0.761	0.794	0.838	0.839	0.823	0.771	0.77	.	0.731	0.799	0.738	0.79	0.827	0.868
.	0.833	0.831	0.779	0.818	0.813	0.83	0.773	0.792	0.812	0.822	0.788	0.76	0.806	.	0.741	0.783	0.73	0.782	0.815	0.839
.	0.815	0.799	0.811	0.816	0.803	0.813	0.777	0.846	0.809	0.813	0.813	0.785	0.815	.	0.749	0.827	0.78	0.812	0.825	0.818
.	0.84	0.825	0.797	0.784	0.79	0.807	0.78	0.842	0.812	0.849	0.857	0.784	0.795	.	0.755	0.812	0.797	0.796	0.822	0.842
.	0.786	0.835	0.819	0.781	0.79	0.794	0.786	0.808	0.829	0.823	0.803	0.779	0.796	.	0.737	0.785	0.794	0.823	0.821	0.814
.	0.824	0.774	0.808	0.755	0.787	0.794	0.778	0.817	0.811	0.831	0.813	0.768	0.807	.	0.787	0.785	0.766	0.836	0.828	0.823
.	0.797	0.822	0.795	0.787	0.782	0.782	0.773	0.791	0.803	0.823	0.828	0.76	0.778	.	0.754	0.75	0.792	0.83	0.835	0.835
.	0.819	0.795	0.771	0.77	0.788	0.79	0.796	0.815	0.78	0.837	0.847	0.763	0.783	.	0.746	0.747	0.787	0.826	0.823	0.835
.	0.855	0.8	0.767	0.822	0.82	0.819	0.833	0.809	0.784	0.814	0.817	0.801	0.797	.	0.77	0.793	0.778	0.824	0.84	0.839
.	0.833	0.819	0.772	0.797	0.825	0.836	0.815	0.79	0.762	0.762	0.783	0.785	0.835	.	0.747	0.791	0.762	0.804	0.806	0.8
.	0.796	0.828	0.806	0.777	0.775	0.843	0.823	0.799	0.787	0.799	0.812	0.776	0.821	.	0.758	0.746	0.771	0.822	0.83	0.807
.	0.806	0.813	0.823	0.783	0.797	0.866	0.817	0.778	0.81	0.764	0.821	0.765	0.851	.	0.769	0.808	0.769	0.805	0.811	0.827
.	0.813	0.793	0.854	0.779	0.795	0.831	0.804	0.779	0.806	0.832	0.827	0.775	0.837	.	0.74	0.775	0.751	0.802	0.813	0.839
.	0.843	0.776	0.801	0.758	0.796	0.851	0.81	0.78	0.787	0.815	0.831	0.771	0.819	.	0.744	0.709	0.822	0.812	0.823	0.858
.	0.848	0.768	0.849	0.816	0.832	0.828	0.832	0.812	0.817	0.788	0.822	0.828	0.817	.	0.744	0.728	0.807	0.8	0.831	0.8
.	0.827	0.776	0.813	0.786	0.793	0.81	0.797	0.829	0.849	0.838	0.845	0.788	0.792	.	0.737	0.802	0.781	0.824	0.811	0.8
.	0.863	0.787	0.781	0.749	0.769	0.821	0.819	0.809	0.828	0.839	0.853	0.769	0.783	.	0.753	0.792	0.757	0.858	0.827	0.8
.	0.787	0.778	0.76	0.731	0.773	0.795	0.772	0.822	0.823	0.816	0.81	0.795	0.792	.	0.769	0.743	0.839	0.802	0.835	0.79
.	0.798	0.746	0.792	0.811	0.774	0.833	0.84	0.769	0.808	0.819	0.836	0.787	0.838	.	0.765	0.81	0.827	0.845	0.855	0.811
.	0.836	0.805	0.84	0.798	0.753	0.826	0.834	0.805	0.852	0.798	0.795	0.773	0.863	.	0.728	0.802	0.775	0.828	0.84	0.808
.	0.774	0.823	0.816	0.776	0.755	0.824	0.782	0.817	0.851	0.827	0.805	0.773	0.865	.	0.713	0.802	0.8	0.814	0.861	0.814
.	0.73	0.8	0.756	0.789	0.848	0.789	0.819	0.788	0.861	0.827	0.836	0.809	0.812	.	0.71	0.831	0.798	0.804	0.851	0.804
.	0.759	0.783	0.789	0.763	0.872	0.798	0.82	0.8	0.872	0.831	0.843	0.834	0.842	.	0.736	0.778	0.796	0.806	0.849	0.777
.	0.77	0.767	0.832	0.807	0.818	0.838	0.834	0.809	0.9	0.872	0.853	0.837	0.831	.	0.807	0.789	0.766	0.796	0.856	0.803
.	0.805	0.694	0.823	0.847	0.825	0.819	0.841	0.832	0.912	0.86	0.857	0.851	0.763	.	0.813	0.839	0.771	0.783	0.862	0.822
.	0.788	0.722	0.813	0.839	0.767	0.834	0.807	0.817	0.85	0.82	0.839	0.833	0.818	.	0.813	0.856	0.805	0.825	0.854	0.838
.	0.787	0.725	0.817	0.816	0.771	0.853	0.768	0.833	0.856	0.824	0.834	0.818	0.857	.	0.81	0.837	0.825	0.887	0.836	0.834
.	0.848	0.769	0.81	0.788	0.782	0.857	0.781	0.812	0.865	0.828	0.818	0.8	0.823	.	0.789	0.796	0.796	0.835	0.853	0.843
.	0.765	0.819	0.794	0.769	0.813	0.814	0.809	0.789	0.853	0.833	0.847	0.791	0.854	.	0.779	0.796	0.778	0.851	0.866	0.838
.	0.765	0.768	0.812	0.767	0.869	0.85	0.801	0.791	0.866	0.803	0.831	0.853	0.845	.	0.762	0.791	0.755	0.833	0.881	0.816
.	0.751	0.744	0.847	0.744	0.795	0.846	0.808	0.818	0.842	0.833	0.821	0.852	0.809	.	0.77	0.775	0.749	0.853	0.903	0.85
.	0.83	0.776	0.813	0.782	0.769	0.845	0.823	0.785	0.817	0.821	0.762	0.842	0.81	.	0.786	0.76	0.731	0.828	0.894	0.841
.	0.821	0.807	0.811	0.861	0.8	0.804	0.829	0.812	0.849	0.831	0.852	0.848	0.847	.	0.783	0.786	0.761	0.8	0.863	0.8
.	0.79	0.805	0.747	0.836	0.838	0.82	0.819	0.8	0.846	0.809	0.855	0.833	0.842	.	0.777	0.833	0.782	0.814	0.864	0.839
.	0.766	0.787	0.778	0.801	0.765	0.811	0.792	0.787	0.828	0.831	0.833	0.817	0.829	.	0.741	0.823	0.774	0.823	0.859	0.843
.	0.775	0.799	0.773	0.729	0.748	0.848	0.781	0.78	0.816	0.794	0.81	0.837	0.844	.	0.689	0.807	0.729	0.805	0.876	0.812
.	0.741	0.732	0.862	0.741	0.764	0.812	0.787	0.815	0.893	0.791	0.844	0.788	0.821	.	0.728	0.796	0.764	0.814	0.86	0.809
.	0.861	0.741	0.796	0.78	0.786	0.829	0.831	0.748	0.845	0.759	0.769	0.8	0.801	.	0.767	0.805	0.779	0.829	0.868	0.807
.	0.752	0.776	0.819	0.822	0.844	0.798	0.839	0.809	0.846	0.802	0.826	0.833	0.849	.	0.71	0.814	0.792	0.83	0.823	0.839
.	0.768	0.803	0.868	0.794	0.81	0.862	0.781	0.783	0.854	0.828	0.836	0.836	0.801	.	0.667	0.82	0.786	0.816	0.812	0.818
.	0.761	0.802	0.821	0.767	0.801	0.824	0.795	0.811	0.841	0.841	0.802	0.821	0.777	.	0.679	0.851	0.776	0.83	0.853	0.812
.	0.792	0.784	0.752	0.837	0.763	0.873	0.819	0.768	0.823	0.813	0.826	0.818	0.792	.	0.737	0.814	0.757	0.834	0.857	0.833
.	0.797	0.76	0.799	0.854	0.78	0.834	0.835	0.743	0.848	0.874	0.814	0.795	0.835	.	0.722	0.796	0.757	0.826	0.853	0.816
.	0.805	0.793	0.856	0.86	0.843	0.82	0.826	0.82	0.846	0.873	0.841	0.817	0.786	.	0.708	0.781	0.832	0.838	0.863	0.804
.	0.845	0.783	0.908	0.861	0.816	0.851	0.823	0.819	0.858	0.857	0.811	0.851	0.83	.	0.745	0.802	0.782	0.827	0.832	0.845
.	0.867	0.756	0.854	0.843	0.787	0.824	0.803	0.777	0.819	0.856	0.793	0.826	0.839	.	0.755	0.816	0.775	0.813	0.835	0.79
.	0.835	0.77	0.777	0.873	0.789	0.79	0.802	0.803	0.855	0.838	0.819	0.845	0.847	.	0.72	0.831	0.788	0.822	0.876	0.833
.	0.823	0.823	0.742	0.831	0.835	0.765	0.83	0.836	0.856	0.87	0.851	0.832	0.812	.	0.728	0.828	0.783	0.816	0.839	0.84
.	0.837	0.83	0.755	0.825	0.833	0.746	0.775	0.788	0.863	0.87	0.818	0.832	0.844	.	0.787	0.798	0.759	0.827	0.85	0.821
.	0.821	0.827	0.815	0.828	0.81	0.772	0.767	0.771	0.833	0.874	0.846	0.836	0.844	.	0.783	0.856	0.767	0.848	0.818	0.86
.	0.828	0.82	0.814	0.821	0.81	0.814	0.821	0.815	0.811	0.838	0.853	0.828	0.826	.	0.804	0.842	0.789	0.838	0.842	0.838
.	0.824	0.808	0.796	0.829	0.827	0.845	0.799	0.806	0.843	0.837	0.842	0.825	0.826	.	0.813	0.789	0.787	0.831	0.831	0.849
.	0.79	0.77	0.842	0.843	0.799	0.821	0.817	0.828	0.837	0.817	0.819	0.845	0.832	.	0.821	0.84	0.766	0.83	0.855	0.841
.	0.813	0.768	0.813	0.849	0.791	0.843	0.808	0.826	0.831	0.829	0.823	0.841	0.818	.	0.792	0.813	0.747	0.799	0.812	0.825

.	0.821	0.767	0.783	0.847	0.803	0.824	0.825	0.844	0.831	0.85	0.845	0.828	0.808	.	0.758	0.825	0.751	0.799	0.831	0.826
.	0.793	0.754	0.769	0.851	0.79	0.806	0.807	0.811	0.826	0.839	0.828	0.828	0.802	.	0.789	0.826	0.769	0.793	0.809	0.832
.	0.757	0.765	0.792	0.827	0.797	0.805	0.8	0.819	0.815	0.815	0.783	0.842	0.804	.	0.79	0.835	0.792	0.796	0.829	0.839
.	0.818	0.814	0.804	0.806	0.816	0.8	0.8	0.805	0.78	0.833	0.783	0.804	0.817	.	0.754	0.812	0.769	0.829	0.809	0.83
.	0.726	0.811	0.799	0.826	0.766	0.772	0.814	0.801	0.812	0.808	0.818	0.828	0.814	.	0.787	0.803	0.794	0.802	0.819	0.822
.	0.779	0.799	0.779	0.783	0.805	0.746	0.825	0.813	0.785	0.829	0.823	0.81	0.818	.	0.76	0.815	0.732	0.804	0.824	0.8
.	0.83	0.786	0.78	0.791	0.811	0.784	0.803	0.828	0.832	0.821	0.819	0.799	0.809	.	0.776	0.81	0.801	0.823	0.816	0.826
.	0.817	0.766	0.784	0.798	0.785	0.831	0.812	0.821	0.817	0.813	0.82	0.825	0.811	.	0.748	0.779	0.789	0.822	0.804	0.797
.	0.823	0.805	0.772	0.799	0.825	0.834	0.826	0.789	0.766	0.814	0.799	0.843	0.815	.	0.772	0.78	0.795	0.807	0.82	0.793
.	0.826	0.776	0.798	0.772	0.835	0.805	0.8	0.807	0.768	0.803	0.805	0.83	0.832	.	0.821	0.8	0.78	0.81	0.829	0.84
.	0.837	0.794	0.785	0.786	0.818	0.781	0.812	0.799	0.773	0.808	0.813	0.82	0.797	.	0.769	0.786	0.801	0.838	0.827	0.804
.	0.829	0.753	0.777	0.78	0.829	0.766	0.807	0.806	0.829	0.803	0.799	0.821	0.82	.	0.756	0.774	0.763	0.826	0.811	0.809
.	0.812	0.78	0.742	0.743	0.803	0.783	0.81	0.78	0.828	0.817	0.792	0.81	0.795	.	0.765	0.757	0.778	0.842	0.801	0.789
.	0.805	0.763	0.738	0.744	0.825	0.863	0.799	0.793	0.806	0.822	0.772	0.802	0.808	.	0.777	0.803	0.782	0.829	0.801	0.792
.	0.77	0.766	0.748	0.762	0.795	0.844	0.792	0.802	0.797	0.833	0.798	0.791	0.802	.	0.763	0.762	0.806	0.819	0.814	0.805
.	0.756	0.767	0.758	0.738	0.806	0.85	0.817	0.783	0.749	0.797	0.809	0.811	0.828	.	0.746	0.793	0.775	0.857	0.831	0.793
.	0.729	0.734	0.745	0.722	0.773	0.806	0.8	0.805	0.772	0.794	0.785	0.819	0.828	.	0.746	0.798	0.733	0.855	0.802	0.772
.	0.754	0.726	0.738	0.72	0.766	0.805	0.79	0.807	0.777	0.808	0.801	0.809	0.803	.	0.766	0.808	0.747	0.823	0.786	0.744
.	0.803	0.713	0.73	0.705	0.777	0.786	0.814	0.798	0.788	0.814	0.835	0.776	0.819	.	0.733	0.794	0.758	0.84	0.805	0.84
.	0.809	0.722	0.716	0.749	0.758	0.809	0.828	0.781	0.792	0.784	0.797	0.786	0.812	.	0.806	0.753	0.768	0.813	0.804	0.797
.	0.821	0.708	0.715	0.725	0.828	0.808	0.821	0.782	0.799	0.821	0.821	0.766	0.788	.	0.74	0.744	0.759	0.843	0.798	0.786
.	0.781	0.738	0.724	0.719	0.805	0.821	0.829	0.797	0.801	0.824	0.806	0.841	0.789	.	0.691	0.748	0.748	0.853	0.777	0.812
.	0.819	0.742	0.709	0.707	0.763	0.764	0.807	0.814	0.796	0.804	0.814	0.845	0.804	.	0.767	0.809	0.768	0.835	0.794	0.779
.	0.795	0.742	0.704	0.716	0.777	0.79	0.794	0.804	0.826	0.805	0.812	0.824	0.793	.	0.75	0.813	0.784	0.829	0.806	0.782
.	0.822	0.749	0.69	0.722	0.757	0.801	0.83	0.742	0.802	0.765	0.812	0.793	0.754	.	0.741	0.805	0.772	0.822	0.81	0.823
.	0.78	0.752	0.71	0.707	0.783	0.808	0.828	0.762	0.783	0.805	0.828	0.792	0.783	.	0.79	0.841	0.778	0.791	0.839	0.821
.	0.803	0.716	0.699	0.689	0.816	0.746	0.825	0.751	0.806	0.792	0.817	0.81	0.801	.	0.796	0.797	0.754	0.818	0.791	0.813
.	0.806	0.749	0.754	0.71	0.766	0.75	0.832	0.757	0.807	0.827	0.804	0.783	0.792	.	0.765	0.786	0.762	0.855	0.797	0.783
.	0.809	0.764	0.766	0.695	0.778	0.758	0.807	0.796	0.793	0.813	0.804	0.818	0.79	.	0.707	0.808	0.771	0.826	0.779	0.787
.	0.798	0.77	0.757	0.698	0.772	0.749	0.85	0.799	0.735	0.825	0.781	0.836	0.817	.	0.752	0.806	0.772	0.825	0.809	0.789
.	0.767	0.736	0.749	0.711	0.767	0.844	0.84	0.777	0.785	0.818	0.747	0.815	0.818	.	0.737	0.801	0.748	0.826	0.794	0.769
.	0.744	0.712	0.747	0.71	0.794	0.862	0.834	0.784	0.77	0.812	0.785	0.802	0.834	.	0.767	0.802	0.743	0.851	0.792	0.757
.	0.734	0.725	0.718	0.722	0.763	0.844	0.807	0.715	0.802	0.79	0.816	0.805	0.783	.	0.809	0.777	0.785	0.88	0.785	0.782
.	0.757	0.707	0.709	0.717	0.775	0.813	0.831	0.748	0.788	0.826	0.796	0.854	0.787	.	0.784	0.758	0.818	0.836	0.841	0.856
.	0.759	0.714	0.727	0.718	0.734	0.788	0.846	0.775	0.852	0.87	0.829	0.854	0.81	.	0.754	0.801	0.824	0.852	0.805	0.843
.	0.779	0.723	0.737	0.726	0.763	0.79	0.828	0.778	0.835	0.823	0.789	0.869	0.817	.	0.736	0.83	0.831	0.843	0.815	0.822
.	0.771	0.788	0.742	0.741	0.732	0.824	0.827	0.814	0.848	0.833	0.776	0.852	0.81	.	0.745	0.821	0.788	0.832	0.81	0.849
.	0.806	0.796	0.764	0.734	0.828	0.829	0.876	0.792	0.834	0.845	0.837	0.831	0.843	.	0.771	0.792	0.784	0.873	0.826	0.878
.	0.79	0.799	0.73	0.731	0.798	0.803	0.802	0.805	0.83	0.845	0.829	0.846	0.871	.	0.775	0.763	0.772	0.831	0.837	0.827
.	0.741	0.763	0.736	0.799	0.776	0.851	0.813	0.77	0.821	0.821	0.817	0.848	0.831	.	0.783	0.8	0.766	0.851	0.844	0.81
.	0.734	0.745	0.786	0.82	0.8	0.833	0.802	0.789	0.827	0.891	0.771	0.811	0.82	.	0.749	0.792	0.839	0.852	0.841	0.822
.	0.796	0.718	0.816	0.866	0.804	0.808	0.83	0.771	0.855	0.843	0.829	0.835	0.831	.	0.802	0.813	0.837	0.832	0.805	0.779
.	0.766	0.808	0.766	0.841	0.776	0.816	0.833	0.796	0.803	0.854	0.88	0.85	0.83	.	0.781	0.816	0.817	0.808	0.824	0.784
.	0.755	0.768	0.79	0.796	0.765	0.834	0.803	0.811	0.82	0.816	0.843	0.843	0.845	.	0.751	0.84	0.778	0.863	0.811	0.773
.	0.778	0.728	0.836	0.813	0.852	0.829	0.852	0.799	0.832	0.823	0.842	0.817	0.837	.	0.794	0.837	0.783	0.817	0.803	0.816
.	0.784	0.728	0.811	0.82	0.825	0.784	0.818	0.806	0.797	0.841	0.82	0.848	0.82	.	0.783	0.805	0.776	0.768	0.861	0.832
	0.792	0.819	0.783	0.838	0.805	0.786	0.79	0.768	0.838	0.854	0.811	0.854	0.807				0.796	0.803	0.845	0.821
	0.813	0.741	0.735	0.821	0.754	0.832	0.793	0.759	0.816	0.861	0.847	0.822	0.84				0.834	0.826	0.88	0.827
	0.734	0.741	0.75	0.801	0.74	0.822	0.808	0.763	0.802	0.886	0.828	0.828	0.852				0.794	0.796	0.848	0.851
	0.822	0.781	0.855	0.794	0.804	0.8	0.838	0.81	0.807	0.878	0.797	0.858	0.82				0.772	0.82	0.837	0.787
	0.81	0.822	0.861	0.797	0.844	0.814	0.856	0.807	0.829	0.841	0.795	0.858	0.808				0.804	0.844	0.875	0.818
	0.764	0.838	0.849	0.844	0.804	0.795	0.828	0.76	0.858	0.864	0.793	0.834	0.82				0.789	0.833	0.843	0.818
	0.777	0.833	0.855	0.842	0.843	0.829	0.777	0.788	0.829	0.835	0.782	0.841	0.838				0.772	0.809	0.859	0.825
	0.811	0.818	0.859	0.79	0.877	0.814	0.803	0.784	0.825	0.835	0.799	0.829	0.777				0.784	0.824	0.807	0.86
	0.797	0.799	0.858	0.727	0.833	0.847	0.778	0.81	0.85	0.822	0.819	0.808	0.831				0.766	0.789	0.863	0.864
	0.777	0.819	0.811	0.737	0.846	0.814	0.836	0.821	0.826	0.818	0.799	0.811	0.829				0.772	0.791	0.838	0.823
	0.791	0.835	0.86	0.841	0.86	0.819	0.821	0.807	0.814	0.872	0.832	0.839	0.842				0.81	0.829	0.822	0.849
	0.778	0.8	0.829	0.818	0.843	0.783	0.803	0.807	0.839	0.854	0.793	0.812	0.848				0.796	0.846	0.839	0.847
	0.778	0.771	0.796	0.827	0.79	0.845	0.802	0.801	0.853	0.807	0.785	0.795	0.862				0.793	0.839	0.84	0.835
	0.787	0.821	0.865	0.78	0.776	0.876	0.801	0.781	0.819	0.809	0.874	0.829	0.85				0.802	0.819	0.83	0.837
	0.83	0.85	0.828	0.793	0.865	0.87	0.842	0.794	0.837	0.803	0.806	0.858	0.84				0.801	0.774	0.847	0.834
	0.831	0.813	0.862	0.788	0.877	0.864	0.826	0.801	0.837	0.85	0.808	0.875	0.819				0.809	0.818	0.802	0.851
	0.816	0.815	0.82	0.781	0.848	0.839	0.836	0.784	0.802	0.845	0.876	0.837	0.828				0.782	0.804	0.836	0.847
	0.806	0.834	0.797	0.824	0.819	0.86	0.824	0.816	0.784	0.863	0.871	0.845	0.806				0.762	0.807	0.833	0.816
	0.799	0.805	0.814	0.813	0.836	0.832	0.809	0.799	0.816	0.824	0.847	0.85	0.808				0.754	0.84	0.845	0.822
	0.807	0.788	0.821	0.794	0.856	0.841	0.825	0.804	0.813	0.816	0.835	0.833	0.835				0.765	0.811	0.806	0.814
	0.796	0.795	0.805	0.769	0.836	0.807	0.812	0.795	0.81	0.805	0.811	0.803	0.786				0.761	0.812	0.817	0.801
	0.773	0.789	0.782	0.807	0.821	0.814	0.845	0.789	0.786	0.817	0.795	0.805	0.798				0.765	0.827	0.839	0.816
	0.744	0.825	0.754	0.837	0.831	0.795	0.823	0.778	0.761	0.827	0.801	0.794	0.817				0.77	0.792	0.8	0.811
	0.794	0.815	0.8	0.833	0.828	0.82	0.826	0.788	0.767	0.812	0.785	0.783	0.796				0.759	0.821	0.817	0.808
	0.809	0.817	0.812	0.818	0.816	0.816	0.829	0.786	0.746	0.806	0.803	0.79	0.79				0.783	0.836	0.811	0.791
	0.814	0.786	0.833	0.832	0.814	0.777	0.809	0.793	0.772	0.821	0.796	0.805	0.773				0.774	0.817	0.819	0.797
	0.811	0.817	0.812	0.855	0.8	0.754	0.814	0.803	0.786	0.818	0.783	0.814	0.805				0.764	0.816	0.831	0.826
	0.75	0.82	0.822	0.817	0.793	0.788	0.806	0.785	0.807	0.828	0.792	0.782	0.804				0.772	0.822	0.829	0.808
	0.769	0.822	0.819	0.835	0.842	0.809	0.825	0.801	0.761	0.81	0.776	0.822	0.797				0.79	0.816	0.827	0.764
	0.803	0.827	0.84	0.828	0.843	0.818	0.832	0.811	0.779	0.83	0.772	0.837	0.806				0.776	0.826	0.811	0.816
	0.805	0.803	0.795	0.824	0.834	0.808	0.805	0.8	0.726	0.761	0.753	0.803	0.836				0.784	0.793	0.791	0.821
	0.794	0.839	0.777	0.829	0.825	0.817	0.806	0.787	0.715	0.796	0.731	0.809	0.793				0.748	0.823	0.806	0.804
	0.797	0.848	0.784	0.819	0.827	0.788	0.794	0.8	0.738	0.816	0.787	0.785	0.788				0.774	0.812	0.795	0.805
	0.816	0.807	0.754	0.8	0.794	0.807	0.8	0.791	0.755	0.793	0.801	0.821	0.798				0.749	0.833	0.829	0.791
	0.851	0.808	0.752	0.792	0.833	0.817	0.816	0.829	0.767	0.819	0.775	0.828	0.754				0.741	0.809	0.825	0.787
	0.807	0.806	0.739	0.805	0.825	0.797	0.761	0.805	0.747	0.82	0.757	0.825	0.786				0.725	0.811	0.827	0.757
	0.774	0.805	0.722	0.778	0.807	0.77	0.779	0.795	0.754	0.794	0.775	0.824	0.815				0.717	0.826	0.842	0.816
	0.801	0.791	0.813	0.837	0.846	0.774	0.786	0.776	0.778	0.783	0.778	0.792	0.8				0.718	0.8	0.827	0.82
	0.793	0.761	0.848	0.841	0.845	0.79	0.781	0.736	0.786	0.802	0.772	0.787	0.756				0.72	0.791	0.82	0.842
	0.775	0.735	0.8	0.817	0.802	0.785	0.8	0.773	0.783	0.802	0.742	0.814	0.755				0.708	0.79	0.826	0.813
	0.759	0.71	0.813	0.804	0.795	0.769	0.797	0.773	0.817	0.775	0.756	0.835	0.803				0.701	0.798	0.828	0.793
	0.799	0.778	0.763	0.807	0.797	0.743	0.761	0.782	0.788	0.769	0.727	0.803	0.803				0.702	0.825	0.827	0.796
	0.816	0.772	0.776	0.818	0.839	0.748	0.79	0.802	0.758	0.823	0.772	0.818	0.825				0.76	0.822	0.842	0.829
	0.816	0.803	0.823	0.83	0.856	0.761	0.812	0.813	0.787	0.814	0.793	0.81	0.796				0.758	0.816	0.827	0.824
	0.775	0.779	0.83	0.799	0.845	0.784	0.765	0.751	0.817	0.796	0.758	0.816	0.829				0.741	0.817	0.818	0.791
	0.783	0.78	0.808	0.809	0.817	0.799	0.766	0.736	0.782	0.81	0.781	0.839	0.809				0.741	0.81	0.828	0.81
	0.785	0.794	0.785	0.801	0.85	0.776	0.786	0.768	0.753	0.796	0.741	0.81	0.804				0.712	0.814	0.818	0.801
	0.753	0.715	0.767	0.826	0.821	0.756	0.792	0.757	0.778	0.815	0.799	0.819	0.828				0.724	0.824	0.824	0.83
	0.774	0.812	0.781	0.841	0.821	0.736	0.812	0.807	0.798	0.804	0.786	0.83	0.796				0.733	0.813	0.831	0.845
	0.715	0.822	0.763	0.835	0.804	0.791	0.771	0.814	0.798	0.809	0.79	0.828	0.786				0.743	0.817	0.831	0.835
	0.736	0.772	0.736	0.836	0.822	0.822	0.773	0.783	0.8	0.839	0.797	0.819	0.777				0.699	0.809	0.83	0.847
	0.754	0.779	0.758	0.822	0.849	0.829	0.762	0.732	0.762	0.86	0.803	0.815	0.842				0.774	0.812	0.825	0.812
	0.806	0.786	0.799	0.787	0.802	0.767	0.761	0.732	0.74	0.771	0.77	0.796	0.799				0.797	0.8	0.832	0.784
	0.786	0.806	0.816	0.812	0.771	0.747	0.83	0.776	0.768	0.848	0.756	0.835	0.782				0.814	0.798	0.861	0.804
	0.788	0.775	0.735	0.771	0.767	0.77	0.788	0.756	0.773	0.827	0.83	0.86	0.815				0.782	0.815	0.876	0.778
	0.728	0.784	0.728	0.735	0.803	0.761	0.784	0.761	0.804	0.851	0.774	0.871	0.788				0.764	0.816	0.864	0.808
	0.708	0.815	0.736	0.763	0.842	0.794	0.812	0.789	0.807	0.858	0.842	0.874	0.837				0.769	0.839	0.866	0.827
	0.733	0.841	0.803	0.8	0.845	0.749	0.821	0.808	0.85	0.864	0.788	0.889	0.868				0.709	0.825	0.837	0.807
	0.733	0.782	0.807	0.79	0.79	0.803	0.796	0.812	0.849	0.829	0.775	0.867	0.862				0.752	0.865	0.849	0.838

0.77	0.755	0.821	0.851	0.756	0.79	0.781	0.777	0.822	0.814	0.757	0.865	0.837				0.767	0.83	0.851		0.803
0.741	0.827	0.811	0.82	0.789	0.767	0.774	0.765	0.821	0.858	0.828	0.832	0.825				0.776	0.847	0.816		0.813
0.723	0.853	0.795	0.792	0.803	0.756	0.776	0.765	0.817	0.865	0.846	0.813	0.814				0.796	0.84	0.819		0.827
0.719	0.801	0.772	0.796	0.827	0.824	0.855	0.76	0.806	0.844	0.8	0.822	0.84				0.812	0.836	0.825		0.851
0.734	0.766	0.771	0.791	0.81	0.838	0.84	0.819	0.821	0.834	0.846	0.811	0.816				0.788	0.832	0.824		0.868
		0.83	0.737	0.82	0.769	0.846												0.815		
		0.821	0.749	0.818	0.768	0.871												0.811		
		0.787	0.815	0.794	0.765	0.844														

2	2	6	6	8	9	9	9	11	11	11	11	1	1	1	2	2	5	6	6	8	9
Box-6	Box-8	Box-8	Box-6	Box-5	Box-1	Box-2	Box-6	Box-1	Box-2	Box-4	Box-6	Box-7	Box-8	Box-2	Box-4	Box-7	Box-3	Box-1	Box-4	Box-7	Box-3
23.3	27.5	37	46	50.1	62	63	69	74	76	81	83	1.1	2.2	5.5	19.1	25.1	34.2	38	43	52.2	64
M	M	M	M	M	M	M	M	M	M	M	M	M	M	M	M	M	M	M	M	M	M
H2O	H2O	H2O	H2O	H2O	H2O	H2O	H2O	H2O	H2O	H2O	H2O	Malto	Malto	Malto	Malto	Malto	MD	MD	MD	MD	MD
MH	MH	MH	MH	MH	MH	MH	MH	MH	MH	MH	MH	MM	MM	MM	MM	MM	MM	MM	MM	MM	MM
0.896	0.817	0.895	0.904	0.692	0.734	0.764	0.713	0.816	0.821	0.816	0.878	0.811	0.837	0.847	0.78	0.917	0.778	0.829	0.88	0.679	0.775
0.856	0.818	0.836	0.809	0.716	0.704	0.72	0.752	0.814	0.785	0.81	0.86	0.83	0.785	0.776	0.745	0.909	0.77	0.869	0.865	0.766	0.731
0.869	0.768	0.925	0.797	0.743	0.73	0.751	0.726	0.807	0.772	0.782	0.837	0.86	0.78	0.769	0.773	0.91	0.796	0.887	0.929	0.747	0.726
0.868	0.813	0.923	0.871	0.735	0.718	0.718	0.708	0.769	0.772	0.749	0.829	0.84	0.782	0.766	0.738	0.912	0.839	0.894	0.92	0.75	0.731
0.89	0.861	0.92	0.853	0.723	0.751	0.732	0.729	0.783	0.763	0.747	0.81	0.883	0.816	0.774	0.751	0.912	0.853	0.902	0.947	0.731	0.738
0.897	0.851	0.882	0.838	0.72	0.828	0.731	0.731	0.762	0.744	0.73	0.756	0.906	0.831	0.825	0.758	0.946	0.857	0.867	0.948	0.699	0.725
0.905	0.804	0.898	0.895	0.702	0.845	0.717	0.696	0.749	0.742	0.74	0.77	0.913	0.836	0.853	0.758	0.905	0.869	0.877	0.945	0.724	0.732
0.913	0.846	0.938	0.882	0.692	0.865	0.785	0.755	0.756	0.724	0.731	0.78	0.908	0.857	0.874	0.768	0.95	0.896	0.927	0.936	0.724	0.728
0.918	0.846	0.911	0.904	0.723	0.866	0.853	0.718	0.751	0.728	0.738	0.748	0.92	0.892	0.891	0.753	0.946	0.893	0.922	0.951	0.716	0.722
0.924	0.83	0.923	0.931	0.755	0.878	0.867	0.714	0.766	0.735	0.728	0.736	0.921	0.866	0.894	0.782	0.959	0.875	0.918	0.985	0.819	0.728
0.932	0.783	0.915	0.909	0.848	0.892	0.859	0.774	0.765	0.718	0.796	0.748	0.939	0.876	0.909	0.794	0.947	0.886	0.921	0.967	0.881	0.783
0.935	0.752	0.939	0.918	0.86	0.887	0.886	0.804	0.756	0.71	0.848	0.728	0.946	0.901	0.901	0.854	0.98	0.886	0.906	0.963	0.893	0.83
0.912	0.821	0.93	0.915	0.899	0.895	0.889	0.804	0.763	0.769	0.853	0.794	0.933	0.885	0.912	0.826	0.953	0.88	0.892	0.947	0.889	0.842
0.933	0.856	0.884	0.914	0.913	0.888	0.858	0.795	0.797	0.736	0.853	0.84	0.932	0.874	0.92	0.854	0.971	0.891	0.885	0.953	0.92	0.805
0.941	0.857	0.929	0.958	0.92	0.914	0.869	0.819	0.824	0.736	0.846	0.881	0.946	0.858	0.901	0.86	1.009	0.897	0.877	0.992	0.908	0.824
0.918	0.838	0.922	0.933	0.941	0.91	0.861	0.855	0.816	0.728	0.847	0.889	0.945	0.905	0.9	0.847	0.953	0.879	0.899	0.95	0.933	0.839
0.935	0.828	0.906	0.951	0.951	0.907	0.864	0.852	0.845	0.7	0.865	0.893	0.966	0.901	0.892	0.843	0.959	0.869	0.91	0.978	0.907	0.822
0.918	0.787	0.926	0.951	0.914	0.917	0.871	0.854	0.869	0.743	0.867	0.909	0.954	0.829	0.896	0.89	0.977	0.857	0.893	0.964	0.9	0.858
0.903	0.802	0.92	0.992	0.905	0.871	0.863	0.854	0.862	0.712	0.871	0.899	0.942	0.906	0.899	0.881	0.919	0.913	0.907	0.953	0.916	0.835
0.914	0.867	0.936	0.952	0.928	0.885	0.817	0.844	0.839	0.733	0.878	0.891	0.935	0.917	0.9	0.864	0.951	0.909	0.911	0.977	0.905	0.83
0.936	0.894	0.894	0.93	0.957	0.885	0.85	0.881	0.83	0.735	0.882	0.916	0.959	0.903	0.901	0.848	0.981	0.931	0.879	0.945	0.908	0.795
0.956	0.911	0.924	0.953	0.947	0.942	0.892	0.894	0.831	0.733	0.888	0.902	0.941	0.871	0.879	0.887	0.981	0.88	0.812	0.964	0.891	0.81
0.919	0.905	0.892	0.924	0.933	0.916	0.883	0.896	0.83	0.77	0.882	0.915	0.956	0.899	0.9	0.896	0.944	0.903	0.825	0.94	0.921	0.86
0.91	0.896	0.933	0.937	0.922	0.915	0.859	0.888	0.843	0.769	0.898	0.919	0.97	0.897	0.935	0.877	0.965	0.872	0.882	0.939	0.868	0.808
0.961	0.899	0.919	0.933	0.908	0.916	0.882	0.855	0.856	0.746	0.897	0.904	0.951	0.883	0.935	0.914	0.96	0.855	0.906	0.977	0.892	0.806
0.945	0.902	0.883	0.971	0.941	0.927	0.914	0.903	0.852	0.767	0.893	0.924	0.943	0.916	0.946	0.922	0.956	0.887	0.874	0.95	0.923	0.85
0.977	0.911	0.877	0.969	0.964	0.931	0.883	0.903	0.84	0.744	0.917	0.905	0.941	0.875	0.895	0.913	0.941	0.951	0.901	0.95	0.943	0.862
0.96	0.865	0.904	0.986	0.931	0.915	0.86	0.881	0.8	0.758	0.905	0.902	0.926	0.838	0.915	0.915	0.967	0.923	0.914	1.007	0.935	0.84
0.954	0.861	0.91	0.975	0.926	0.933	0.882	0.851	0.788	0.817	0.9	0.911	0.944	0.908	0.912	0.918	0.944	0.929	0.909	0.98	0.887	0.791
0.979	0.898	0.864	0.969	0.943	0.932	0.916	0.887	0.787	0.814	0.943	0.905	0.934	0.885	0.944	0.92	0.969	0.895	0.859	0.987	0.881	0.841
0.953	0.909	0.856	0.933	0.938	0.91	0.869	0.873	0.754	0.79	0.972	0.893	0.923	0.88	0.928	0.883	0.955	0.889	0.875	0.965	0.907	0.826
0.939	0.901	0.783	0.93	0.933	0.937	0.896	0.892	0.771	0.823	0.949	0.874	0.896	0.917	0.887	0.907	0.981	0.908	0.878	0.972	0.916	0.756
0.948	0.913	0.868	0.976	0.943	0.928	0.892	0.891	0.768	0.787	0.962	0.868	0.893	0.906	0.892	0.85	0.951	0.91	0.881	0.962	0.907	0.807
0.906	0.905	0.833	0.925	0.901	0.914	0.89	0.878	0.753	0.767	0.934	0.83	0.909	0.879	0.918	0.781	0.956	0.921	0.918	0.938	0.881	0.792
0.954	0.916	0.844	0.951	0.92	0.919	0.828	0.919	0.783	0.787	0.965	0.856	0.918	0.883	0.92	0.797	0.953	0.897	0.919	0.982	0.885	0.84
0.972	0.956	0.824	0.964	0.917	0.887	0.815	0.894	0.796	0.813	0.91	0.813	0.936	0.92	0.926	0.89	0.95	0.904	0.901	0.996	0.904	0.835
0.948	0.928	0.813	0.931	0.903	0.914	0.809	0.843	0.823	0.822	0.955	0.808	0.916	0.898	0.92	0.87	0.964	0.911	0.877	0.969	0.937	0.854
0.949	0.884	0.818	0.953	0.914	0.922	0.842	0.821	0.794	0.799	0.977	0.811	0.914	0.87	0.885	0.887	0.906	0.886	0.876	0.934	0.931	0.837
0.94	0.891	0.878	0.946	0.906	0.932	0.883	0.856	0.778	0.775	0.932	0.758	0.903	0.862	0.893	0.916	0.946	0.907	0.846	0.953	0.947	0.783
0.976	0.895	0.866	0.925	0.933	0.882	0.872	0.812	0.783	0.815	0.912	0.773	0.936	0.831	0.917	0.913	0.937	0.903	0.842	0.923	0.934	0.753
0.964	0.889	0.834	0.96	0.863	0.912	0.809	0.844	0.757	0.761	0.879	0.769	0.895	0.811	0.865	0.898	0.964	0.891	0.895	0.949	0.913	0.758
0.967	0.865	0.793	0.919	0.88	0.922	0.911	0.839	0.773	0.771	0.852	0.76	0.886	0.785	0.891	0.886	0.969	0.915	0.86	0.836	0.909	0.785
0.957	0.86	0.865	0.991	0.902	0.916	0.821	0.843	0.807	0.766	0.839	0.771	0.903	0.781	0.864	0.889	0.968	0.911	0.883	0.917	0.937	0.802
0.952	0.827	0.898	0.938	0.897	0.933	0.842	0.834	0.763	0.762	0.843	0.764	0.92	0.791	0.906	0.93	0.969	0.876	0.884	0.873	0.919	0.802
0.951	0.817	0.881	0.947	0.866	0.934	0.839	0.821	0.765	0.786	0.922	0.763	0.899	0.813	0.884	0.908	0.967	0.875	0.791	0.832	0.923	0.78
0.95	0.84	0.802	0.948	0.855	0.888	0.822	0.813	0.76	0.799	0.873	0.779	0.909	0.797	0.894	0.932	0.965	0.92	0.808	0.861	0.846	0.768
0.955	0.867	0.867	0.917	0.882	0.907	0.785	0.773	0.774	0.776	0.901	0.777	0.864	0.83	0.89	0.904	0.963	0.827	0.88	0.851	0.877	0.746
0.93	0.903	0.882	0.936	0.869	0.878	0.786	0.786	0.777	0.78	0.869	0.792	0.877	0.806	0.846	0.902	0.93	0.871	0.835	0.769	0.877	0.764
0.972	0.899	0.85	0.932	0.849	0.898	0.811	0.8	0.796	0.71	0.79	0.764	0.908	0.774	0.839	0.945	0.906	0.855	0.817	0.736	0.909	0.801
0.94	0.864	0.883	0.962	0.877	0.878	0.805	0.825	0.775	0.728	0.814	0.774	0.903	0.775	0.887	0.925	0.913	0.838	0.773	0.833	0.877	0.778
0.953	0.901	0.882	0.948	0.852	0.934	0.767	0.817	0.75	0.743	0.849	0.746	0.889	0.762	0.885	0.918	0.892	0.859	0.783	0.868	0.895	0.767
0.931	0.919	0.872	1.002	0.843	0.871	0.764	0.783	0.811	0.767	0.84	0.75	0.882	0.777	0.9	0.936	0.904	0.858	0.85	0.783	0.899	0.734
0.957	0.917	0.856	0.948	0.901	0.899	0.772	0.797	0.767	0.786	0.881	0.759	0.861	0.778	0.918	0.926	0.932	0.835	0.902	0.783	0.903	0.762
0.933	0.894	0.908	0.934	0.911	0.906	0.841	0.781	0.784	0.759	0.883	0.771	0.843	0.775	0.887	0.902	0.919	0.882	0.871	0.764	0.873	0.772
0.94	0.9	0.899	0.957	0.875	0.904	0.846	0.754	0.783	0.729	0.865	0.746	0.847	0.811	0.906	0.924	0.965	0.884	0.863	0.851	0.839	0.774
0.945	0.942	0.847	0.942	0.833	0.864	0.848	0.789	0.75	0.729	0.843	0.733	0.869	0.777	0.844	0.933	0.985	0.859	0.839	0.866	0.854	0.782
0.955	0.902	0.813	0.919	0.885	0.859	0.844	0.827	0.761	0.745	0.821	0.736	0.853	0.747	0.861	0.929	0.967	0.87	0.873	0.821	0.903	0.743
0.953	0.878	0.896	0.925	0.885	0.885	0.846	0.825	0.74	0.76	0.891	0.721	0.827	0.768	0.867	0.933	0.955	0.85	0.834	0.792	0.906	0.762
0.983	0.871	0.879	0.961	0.823	0.914	0.844	0.82	0.737	0.756	0.847	0.752	0.871	0.772	0.877	0.955	1.022	0.915	0.833	0.843	0.933	0.756
0.934	0.888	0.847	0.897	0.802	0.957	0.764	0.854	0.785	0.742	0.844	0.735	0.875	0.769	0.84	0.958	0.93	0.889	0.796	0.887	0.935	0.757
0.951	0.9	0.874	0.931	0.872	0.893	0.797	0.82	0.77	0.761	0.85	0.744	0.849	0.803	0.837	0.973	0.963	0.837	0.878	0.865	0.927	0.815
0.933	0.917	0.91	0.921	0.879	0.91	0.841	0.833	0.73	0.751	0.847	0.763	0.863	0.809	0.869	0.947	0.947	0.891	0.926	0.896	0.909	0.816
0.971	0.957	0.931	0.97	0.882	0.941	0.855	0.869	0.736	0.792	0.831	0.742	0.902	0.8	0.897	0.966	0.996	0.896	0.907	0.963	0.94	0.752
0.945	0.923	0.894	0.888	0.94	0.916	0.868	0.874	0.732	0.776	0.808	0.74	0.91	0.768	0.838	0.949	0.965	0.873	0.933	0.907	0.956	0.733
0.928	0.905	0.847	0.899	0.968	0.918	0.836	0.848	0.735	0.762	0.863	0.742	0.903	0.764	0.837	0.971	0.991	0.866	0.89	0.885	0.95	0.782
0.915	0.894	0.913	0.95	0.936	0.946	0.87	0.897	0.774	0.79	0.88	0.793	0.871	0.791	0.867	0.923	0.938	0.876	0.938	0.928	0.937	0.839
0.975	0.92	0.888	0.9	0.938	0.937	0.879	0.91	0.746	0.822	0.82	0.718	0.868	0.814	0.847	0.938	0.973	0.882	0.919	0.942	0.929	0.856
0.928	0.874	0.908	0.935	0.912	0.932	0.882	0.893	0.757	0.767	0.813	0.766	0.914	0.868	0.915	0.946	0.951	0.907	0.901	0.924	0.937	0.822
0.94	0.896	0.893	0.913	0.94	0.924	0.846	0.915	0.79	0.799	0.846	0.817	0.927	0.874	0.919	0.93	0.984	0.884	0.898	0.895	0.932	0.8
0.923	0.901	0.908	0.907	0.927	0.915	0.869	0.904	0.797	0.867	0.834	0.866	0.912	0.901	0.898	0.927	0.931	0.905	0.883	0.931	0.915	0.837
0.947	0.91	0.898	0.933	0.928	0.915	0.888	0.897	0.798	0.893	0.833	0.865	0.938	0.93	0.904	0.938	0.937	0.892	0.893	0.955	0.919	0.811
0.927	0.953	0.887	0.931	0.944	0.918	0.895	0.898	0.783	0.878	0.833	0.896	0.937	0.895	0.893	0.93	0.944	0.918	0.904	0.934	0.946	0.848
0.97	0.943	0.904	0.929	0.945	0.903	0.916	0.894	0.806	0.902	0.873	0.899	0.943	0.883	0.894	0.948	0.951	0.939	0.925	0.944	0.94	0.84
0.95	0.938	0.913	0.957	0.94	0.945	0.92	0.94	0.835	0.899	0.877	0.899	0.946	0.905	0.903	0.95	0.972	0.911	0.926	0.959	0.956	0.846
0.923	0.921	0.902	0.925	0.961	0.909	0.89	0.913	0.806	0.882	0.924	0.911	0.947	0.91	0.918	0.94	0.946	0.925	0.91	0.961	0.966	0.828
0.948	0.955	0.896	0.934	0.946	0.936	0.902	0.925	0.836	0.9	0.931	0.896	0.945	0.903	0.918	0.931	0.94	0.909	0.913	0.949	0.964	0.823
0.955	0.94	0.885	0.957	0.95	0.941	0.927	0.911	0.856	0.908	0.919	0.887	0.949	0.932	0.944	0.957	0.965	0.92	0.926	0.947	0.926	0.846
0.944	0.918	0.907	0.938	0.979	0.937	0.911	0.934	0.892	0.884	0.926	0.896	0.947	0.923	0.909	0.926	0.966	0.899	0.927	0.968	0.952	0.877
0.972	0.948	0.913	0.912	0.956	0.927	0.891	0.918	0.895	0.921	0.936	0.907	0.957	0.897	0.916	0.972	0.983	0.936	0.918	0.954	0.933	0.855
1.031	0.949	0.913	0.908	0.954	0.93	0.925	0.925	0.901	0.93	0.932	0.924	0.953	0.96	0.937	0.991	0.958	0.899	0.929	0.939	0.926	0.909
0.98	0.949	0.886	0.93	0.933	0.942	0.912	0.951	0.927	0.928	0.928	0.916	0.943	0.932	0.952	0.967	0.989	0.925	0.874	0.949	0.959	0.889
0.965	0.983	0.9	0.926	0.94	0.932	0.926	0.965	0.91	0.938	0.924	0.93	0.956	0.947	0.954	0.963	0.992	0.919	0.915	0.925	0.926	0.902
0.943	0.956	0.909	0.938	0.956	0.933	0.937	0.941	0.87	0.932	0.943	0.929	0.978	0.942	0.96	0.952	0.979	0.908	0.915	0.922	0.937	0.889
0.964	0.946	0.917	0.927	0.943	0.93	0.951	0.935	0.888	0.943	0.943	0.958	0.956	0.94	0.935	0.955	0.94	0.889	0.932	0.944	0.938	0.911
0.966	0.95	0.915	0.953	0.95	0.941	0.913	0.906	0.904	0.952	0.94	0.944	0.971	0.949	0.919	0.94	0.945	0.882	0.904	0.944	0.915	0.86
0.988	0.958	0.922	0.953	0.964	0.927	0.908	0.946	0.917	0.912	0.917	0.943	0.968	0.952	0.901	0.943	0.959	0.926	0.895	0.966	0.907	0.851
0.953	0.922	0.916	0.937	0.988	0.927	0.929	0.936	0.903	0.929	0.924	0.959	0.95	0.902	0.832	0.939	0.949	0.899	0.891	0.927	0.925	0.869
0.983	0.954	0.906	0.908	0.938	0.905	0.918	0.939	0.918	0.923	0.918	0.932	0.978	0.948	0.809	0.946	0.913	0.937	0.846	0.948	0.921	0.839
0.955	0.933	0.908	0.915	0.922	0.928	0.892	0.893	0.933	0.949	0.909	0.943	0.976	0.927	0.86	0.942	0.905	0.962	0.857	0.948	0.971	0.876
0.932	0.932	0.863	0.93	0.933	0.903	0.921	0.914	0.932	0.935	0.858	0.956	0.982	0.925	0.899	0.921	0.898	0.909	0.84	0.965	0.953	0.87
0.962	0.911	0.859	0.968	0.955	0.892	0.934	0.893	0.941	0.919	0.862	0.927	0.958	0.899	0.871	0.926	0.839	0.965	0.847	0.936	0.953	0.884
0.968	0.89	0.899	0.952	0.942	0.883	0.891	0.947	0.92	0.954	0.822	0.961	0.966	0.955	0.876	0.938	0.796	0.897	0.781	0.938	0.908	0.864
0.979	0.872	0.897	0.949	0.936	0.892	0.889	0.961	0.937	0.945	0.839	0.96	0.981	0.94	0.858	0.894	0.797	0.937	0.764	0.938	0.902	0.919
0.976	0.826	0.837	0.996	0.928	0.878	0.886	0.93	0.964	0.942	0.857	0.941	0.963	0.91	0.885	0.863	0.783	0.904	0.759	0.95	0.903	0.861
0.987	0.904	0.876	0.963	0.936	0.838	0.91	0.941	0.963	0.944	0.813	0.937	0.954	0.933	0.877	0.88	0.83	0.918	0.749	0.918	0.917	0.892
0.949	0.915	0.809	0.965	0.908	0.873	0.918	0.941	0.96	0.95	0.841	0.948	0.937	0.909	0.858	0.83	0.895	0.951	0.731	0.852	0.91	0.89
0.991	0.876	0.866	0.965	0.926	0.835	0.944	0.9	0.946	0.949	0.818	0.973	0.977	0.918	0.888	0.893	0.898	0.899	0.773	0.88	0.912	0.872
0.991	0.881	0.867	0.959	0.918	0.762	0.89	0.869	0.949	0.936	0.857	0.929	0.967	0.932	0.882	0.909	0.915	0.923	0.84	0.902	0.882	0.847

0.976	0.921	0.911	0.964	0.95	0.817	0.87	0.885	0.928	0.945	0.859	0.963	0.989	0.954	0.891	0.909	0.926	0.946	0.833	0.871	0.905	0.85
0.958	0.899	0.881	0.951	0.932	0.858	0.915	0.914	0.949	0.913	0.869	0.928	0.958	0.9	0.889	0.937	0.927	0.897	0.819	0.86	0.909	0.864
0.99	0.878	0.879	0.941	0.967	0.868	0.873	0.888	0.957	0.947	0.901	0.946	0.986	0.909	0.905	0.93	0.927	0.911	0.886	0.882	0.924	0.83
1	0.875	0.891	0.915	0.949	0.834	0.847	0.881	0.937	0.952	0.894	0.946	0.98	0.956	0.874	0.927	0.955	0.857	0.852	0.894	0.913	0.808
0.975	0.869	0.839	0.974	0.898	0.803	0.839	0.889	0.914	0.965	0.876	0.973	0.925	0.904	0.837	0.913	0.915	0.878	0.832	0.834	0.917	0.819
0.983	0.838	0.863	0.953	0.876	0.782	0.888	0.908	0.925	0.962	0.879	0.979	0.94	0.936	0.869	0.933	0.985	0.878	0.775	0.847	0.88	0.843
1.01	0.819	0.884	0.948	0.833	0.833	0.89	0.851	0.965	0.942	0.846	0.975	0.957	0.916	0.863	0.915	0.956	0.875	0.82	0.746	0.839	0.857
1.028	0.806	0.874	0.957	0.896	0.861	0.889	0.815	0.944	0.967	0.847	0.949	0.96	0.951	0.89	0.948	0.957	0.845	0.888	0.824	0.811	0.865
0.977	0.826	0.871	0.964	0.936	0.895	0.881	0.778	0.911	0.999	0.873	0.959	0.943	0.902	0.846	0.933	0.913	0.867	0.878	0.844	0.784	0.899
0.973	0.875	0.875	0.968	0.916	0.864	0.864	0.84	0.902	0.952	0.859	0.941	0.928	0.915	0.868	0.91	0.902	0.864	0.838	0.906	0.89	0.874
0.949	0.897	0.867	0.953	0.916	0.857	0.861	0.841	0.906	0.967	0.851	0.905	0.932	0.903	0.906	0.926	0.927	0.808	0.794	0.85	0.906	0.822
0.977	0.89	0.828	0.946	0.875	0.859	0.875	0.818	0.888	0.944	0.802	0.915	0.926	0.902	0.848	0.919	0.823	0.799	0.866	0.838	0.842	0.848
0.955	0.862	0.794	0.964	0.878	0.805	0.903	0.749	0.852	0.928	0.812	0.889	0.952	0.87	0.819	0.971	0.817	0.857	0.892	0.836	0.878	0.866
0.974	0.893	0.829	0.92	0.885	0.782	0.87	0.76	0.871	0.939	0.82	0.864	0.938	0.834	0.789	0.956	0.899	0.845	0.88	0.85	0.89	0.842
0.93	0.912	0.793	0.915	0.875	0.837	0.839	0.823	0.83	0.924	0.755	0.847	0.949	0.812	0.856	0.944	0.967	0.841	0.871	0.826	0.862	0.834
0.943	0.904	0.869	0.908	0.822	0.855	0.881	0.812	0.813	0.95	0.78	0.864	0.93	0.796	0.85	0.884	0.936	0.813	0.854	0.842	0.85	0.769
0.939	0.897			0.9	0.815	0.893	0.834	0.819	0.924	0.794	0.845	0.945	0.788	0.876	0.914	0.911	0.82			0.885	0.769
0.932	0.826			0.895	0.831	0.888	0.847	0.838	0.909	0.781	0.811	0.893	0.761	0.866	0.91	0.92	0.789			0.804	0.824
0.928	0.831			0.83	0.806	0.855	0.857	0.857	0.889	0.807	0.788	0.898	0.808	0.849	0.949	0.867	0.836			0.72	0.863
0.925	0.885			0.836	0.85	0.855	0.839	0.881	0.858	0.759	0.798	0.908	0.836	0.907	0.944	0.868				0.765	0.895
0.939	0.903			0.857	0.81	0.813	0.852	0.913	0.887	0.775	0.816	0.898	0.831	0.895	0.958	0.865				0.747	0.866
0.992	0.918			0.846	0.807	0.807	0.857	0.926	0.876	0.745	0.831	0.92	0.804	0.885	0.954	0.839				0.749	0.822
0.965	0.924			0.824	0.773	0.806	0.851	0.898	0.901	0.79	0.833	0.954	0.811	0.865	0.98	0.79				0.801	0.848
0.977	0.902			0.796	0.857	0.889	0.874	0.935	0.885	0.77	0.815	0.983	0.819	0.853	0.957	0.843				0.881	0.886
1.003	0.911			0.795	0.854	0.896	0.892	0.91	0.882	0.817	0.8	0.946	0.791	0.882	0.953	0.925				0.891	0.891
0.937	0.892			0.773	0.878	0.903	0.935	0.91	0.863	0.767	0.809	0.957	0.845	0.88	0.934	0.915				0.884	0.937
0.899	0.882			0.815	0.823	0.838	0.845	0.903	0.861	0.801	0.837	0.898	0.872	0.807	0.987	0.895				0.899	0.867
0.913	0.913			0.827	0.801	0.814	0.819	0.901	0.869	0.795	0.915	0.932	0.864	0.793	0.938	0.94				0.952	0.903
0.938	0.923			0.877	0.852	0.85	0.893	0.888	0.862	0.791	0.816	0.973	0.974	0.879	0.968	0.965				0.945	0.911
0.934	0.934			0.875	0.878	0.897	0.895	0.911	0.814	0.786	0.835	0.942	0.924	0.902	0.951	0.911				0.927	0.843
0.969	0.956			0.899	0.892	0.884	0.913	0.893	0.792	0.78	0.847	0.954	0.92	0.89	0.956	0.873				0.906	0.877
0.928	0.917			0.936	0.849	0.906	0.872	0.931	0.829	0.76	0.896	0.959	0.939	0.882	0.944	0.868				0.894	0.879
0.938	0.916			0.944	0.813	0.933	0.899	0.913	0.867	0.794	0.917	0.956	0.931	0.882	0.961	0.873				0.93	0.876
0.952	0.929			0.935	0.872	0.882	0.883	0.923	0.895	0.828	0.886	0.938	0.908	0.896	0.943	0.917				0.914	0.878
0.957	0.928			0.904	0.86	0.879	0.877	0.924	0.906	0.822	0.888	0.948	0.896	0.859	0.948	0.923				0.884	0.865
0.954	0.98			0.918	0.867	0.861	0.913	0.946	0.901	0.81	0.93	0.953	0.928	0.895	0.961	0.913				0.922	0.88
0.944	0.952			0.951	0.876	0.879	0.915	0.956	0.901	0.817	0.94	0.978	0.924	0.946	0.938	0.937				0.905	0.894
0.942	0.935			0.947	0.876	0.867	0.912	0.954	0.899	0.88	0.933	0.979	0.92	0.921	0.941	0.933				0.939	0.869
0.938	0.944			0.934	0.902	0.892	0.89	0.934	0.935	0.893	0.923	0.981	0.939	0.948	0.914	0.929				0.921	0.859
0.93	0.941			0.906	0.882	0.872	0.892	0.934	0.924	0.921	0.935	0.983	0.922	0.92	0.945	0.952				0.928	0.874
0.949	0.924			0.942	0.904	0.876	0.902	0.98	0.938	0.93	0.962	0.989	0.92	0.928	0.926	0.907				0.923	0.886
0.938	0.938			0.934	0.891	0.859	0.895	0.945	0.965	0.919	0.93	0.995	0.944	0.941	0.953	0.894				0.93	0.832
0.951	0.947			0.934	0.906	0.917	0.918	0.967	0.934	0.932	0.934	0.995	0.957	0.946	0.934	0.887				0.916	0.895
0.962	0.939			0.953	0.888	0.883	0.928	0.981	0.924	0.955	0.965	1.007	0.965	0.947	0.973	0.879				0.954	0.909
0.966	0.923			0.939	0.9	0.895	0.892	0.934	0.925	0.926	0.953	1.016	0.949	0.955	0.971	0.898				0.943	0.882
0.967	0.961			0.936	0.888	0.885	0.893	0.948	0.925	0.914	0.927	0.995	0.953	0.941	0.949	0.934				0.949	0.865
0.955	0.945			0.911	0.901	0.883	0.918	0.943	0.912	0.917	0.92	1.005	0.962	0.957	0.942	0.888				0.932	0.863
0.972	0.956			0.913	0.914	0.891	0.938	0.947	0.915	0.933	0.926	1.022	0.968	0.963	0.993	0.902				0.94	0.876
1.001	0.97			0.963	0.899	0.91	0.906	0.982	0.909	0.937	0.947	1	0.977	0.972	0.992	0.917				0.952	0.855
0.975	0.963			0.909	0.902	0.891	0.914	0.936	0.882	0.919	0.911	0.998	0.946	0.967	0.971	0.909				0.924	0.869
0.955	0.974			0.955	0.904	0.917	0.926	0.947	0.875	0.929	0.93	1.027	0.96	0.955	0.965	0.834				0.918	0.878
0.96	0.954			0.961	0.894	0.895	0.931	0.959	0.875	0.956	0.925	0.996	0.93	0.962	0.952	0.834				0.95	0.878
0.945	0.94			0.94	0.887	0.876	0.924	0.954	0.87	0.979	0.92	1.001	0.984	0.931	0.958	0.888				0.953	0.872
0.928	0.925			0.942	0.895	0.869	0.946	0.96	0.858	0.969	0.917	1.021	0.993	0.944	0.937	0.916				0.956	0.846
0.932	0.919			0.9	0.917	0.899	0.933	0.93	0.815	0.938	0.931	1.017	0.956	0.924	0.929	0.901				0.898	0.815
0.917	0.949			0.882	0.894	0.865	0.873	0.954	0.798	0.944	0.934	0.991	0.948	0.92	0.932	0.897				0.862	0.869
0.918	0.949			0.927 -	-	-		0.947	0.768	0.979	0.914	0.997	0.971	0.917	0.945	0.875				0.878 -	
0.971	0.932			0.881 -	-	-		0.95	0.757	0.973	0.932	0.998	0.982	0.922	0.94	0.872				0.839 -	
0.946	0.93			0.908	0.879	0.861	0.874	0.951	0.755	0.981	0.912	1.016	0.978	0.93	0.971	0.856				0.891	0.772
0.935	0.939			0.893	0.86	0.849	0.899	0.968	0.818	0.974	0.92	0.964	0.979	0.938	0.963	0.826				0.928	0.79
0.938	0.921			0.905	0.877	0.842	0.878	0.952	0.805	0.988	0.882	0.976	0.972	0.941	0.961	0.86				0.851	0.789
0.94	0.927			0.931	0.868	0.862	0.905	0.926	0.795	0.964	0.865	0.957	0.938	0.933	0.942	0.907				0.849	0.746
0.896	0.901			0.925	0.9	0.86	0.885	0.949	0.816	0.963	0.873	0.993	0.941	0.917	0.922	0.909				0.898	0.714
0.891	0.881			0.921	0.877	0.847	0.925	0.936	0.836	0.952	0.866	0.953	0.971	0.903	0.905	0.909				0.875	0.781
0.927	0.843			0.946	0.878	0.865	0.85	0.928	0.845	0.949	0.887	0.935	0.971	0.886	0.879	0.916				0.827	0.794
0.94	0.901			0.93	0.893	0.847	0.896	0.895	0.858	0.94	0.835	0.942	0.932	0.893	0.882	0.888				0.81	0.826
0.914	0.878			0.937	0.849	0.849	0.878	0.901	0.861	0.934	0.859	0.91	0.932	0.9	0.875	0.893				0.85	0.803
0.866	0.837			0.917	0.86	0.831	0.864	0.898	0.847	0.971	0.859	0.933	0.907	0.909	0.92	0.859				0.859	0.801
0.847	0.807			0.885	0.776	0.764	0.89	0.854	0.861	0.925	0.837	0.887	0.878	0.887	0.897	0.81				0.871	0.76
0.872	0.82			0.867	0.83	0.819	0.862	0.837	0.848	0.921	0.813	0.883	0.871	0.86	0.901	0.809				0.861	0.723
0.866	0.817			0.909	0.769	0.831	0.848	0.866	0.824	0.936	0.831	0.909	0.846	0.897	0.918	0.83				0.899	0.749
0.861	0.822			0.916	0.751	0.808	0.862	0.804	0.852	0.886	0.848	0.956	0.861	0.909	0.905	0.785				0.907	0.794
0.925	0.909			0.889	0.772	0.743	0.852	0.765	0.865	0.881	0.851	0.9	0.831	0.89	0.883	0.819				0.934	0.792
0.948	0.92			0.893	0.822	0.718	0.877	0.777	0.845	0.88	0.862	0.901	0.818	0.904	0.867	0.798				0.878	0.823
0.897	0.887			0.907	0.808	0.753	0.847	0.828	0.883	0.925	0.837	0.869	0.818	0.915	0.901	0.783				0.923	0.809
0.89	0.899			0.894	0.791	0.747	0.837	0.787	0.857	0.882	0.791	0.857	0.817	0.922	0.835	0.724				0.914	0.745
0.907	0.923			0.922	0.777	0.74	0.808	0.764	0.888	0.848	0.778	0.856	0.773	0.923	0.842	0.78				0.958	0.801
0.857	0.861			0.906	0.765	0.711	0.826	0.753	0.876	0.859	0.813	0.898	0.775	0.901	0.876	0.772				0.938	0.823
0.863	0.84			0.875	0.751	0.747	0.818	0.797	0.843	0.885	0.84	0.937	0.774	0.928	0.901	0.764				0.911	0.774
0.88	0.928			0.865	0.795	0.799	0.867	0.821	0.821	0.843	0.823	0.956	0.772	0.903	0.946	0.777				0.851	0.777
0.903	0.922			0.835	0.831	0.807	0.81	0.854	0.85	0.857	0.787	0.92	0.809	0.876	0.876	0.733				0.819	0.759
0.936	0.955			0.881	0.77	0.8	0.827	0.854	0.878	0.814	0.791	0.897	0.797	0.882	0.854	0.75				0.806	0.767
0.967	0.948	0.942	0.829	0.816	0.828	0.813	0.859	0.951	0.924	0.992	0.969 .		0.882	0.912	0.976	0.941	0.925	0.92	0.95	0.903	0.818
0.971	0.962	0.969	0.914	0.78	0.865	0.884	0.904	1.011	0.97	1.032	0.964 .		0.93	0.905	0.99	0.962	0.949	0.954	1.045	0.952	0.863
0.989	0.953	0.941	0.938	0.865	0.893	0.903	0.93	0.975	0.987	1.022	0.978 .		0.968	0.841	0.99	0.984	0.925	0.93	0.996	0.954	0.895
0.983	0.994	0.951	0.96	0.924	0.923	0.926	0.947	0.976	0.957	0.977	0.998 .		0.993	0.853	1.008	0.982	0.942	0.966	1.022	0.971	0.885
0.981	0.997	0.963	0.936	0.944	0.916	0.906	0.966	0.985	1	0.993	0.984 .		0.987	0.846	0.998	0.977	0.975	0.938	1.015	0.992	0.893
0.992	0.964	0.948	1.012	0.939	0.954	0.924	0.942	0.974	1.011	0.965	0.984 .		1.006	0.84	0.988	0.97	0.927	0.94	0.968	0.991	0.91
0.972	0.984	0.951	1.042	0.962	0.917	0.934	0.969	1.014	0.99	0.999	1.012 .		1.001	0.889	0.989	0.976	0.944	0.957	1.024	1.033	0.897
0.971	0.996	0.968	0.974	0.997	0.944	0.933	0.933	0.985	0.951	0.995	0.967 .		0.967	0.882	0.992	0.967	0.936	0.969	1.007	0.978	0.903
0.959	0.973	0.928	0.992	0.998	0.915	0.915	0.936	1.002	0.987	1.003	0.984 .		1.005	0.947	0.975	0.957	0.901	0.989	0.995	1.008	0.931
0.969	0.992	0.932	0.96	0.97	0.934	0.941	0.952	0.966	0.98	0.998	0.984 .		0.995	0.974	1.002	0.987	0.93	0.948	1.005	0.996	0.877
0.983	0.971	0.969	0.988	0.985	0.942	0.934	0.942	0.99	0.968	0.987	0.955 .		0.997	0.963	0.998	0.963	0.892	0.976	1.008	0.988	0.927
0.965	0.991	0.972	0.992	0.999	0.937	0.93	0.942	0.979	0.948	0.984	0.951 .		0.979	0.961	0.979	0.955	0.901	0.949	1.013	0.966	0.918
0.973	0.957	0.947	0.997	0.995	0.93	0.885	0.921	1.006	0.992	1.003	0.974 .		0.993	0.982	0.989	0.982	0.92	0.947	0.997	0.979	0.897
0.983	0.988	0.973	0.977	0.96	0.95	0.909	0.933	1.007	0.96	0.984	1 .		0.984	0.928	0.977	0.966	0.904	0.974	0.997	0.964	0.913
0.96	0.96	0.964	0.976	0.964	0.904	0.911	0.949	0.993	0.96	0.966	0.985 .		0.981	0.951	0.962	0.942	0.943	0.954	0.974	0.913	0.896
0.981	0.976	0.969	0.988	0.968	0.931	0.9	0.939	1.005	0.969	0.982	0.964 .		0.977	0.943	0.982	0.978	0.946	0.972	0.978	0.948	0.886
0.984	1.018	0.958	0.997	0.954	0.914	0.906	0.921	0.973	0.981	0.977	0.984 .		1.003	0.981	0.997	0.988	0.957	0.94	0.996	0.98	0.891
0.995	0.991	0.909	0.958	0.929	0.9	0.905	0.938	0.989	0.967	0.97	0.962 .		1.008	0.98	1.006	0.986	0.922	0.939	0.992	0.944	0.898
0.978	0.97	0.958	1.027	0.977	0.929	0.903	0.91	0.988	0.967	0.988	0.968 .		0.979	0.961	0.982	0.96	0.954	0.943	0.979	0.947	0.9
0.99	0.993	0.913	0.983	0.972	0.92	0.912	0.941	0.983	0.972	0.988	0.988 .		1.001	0.956	0.989	0.96	0.941	0.946	0.967	0.951	0.907
1.031	0.987	0.945	0.97	0.948	0.933	0.914	0.921	1.001	1.007	1.003	0.991 .		1.014	0.984	1.029	0.976	0.949	0.944	0.964	0.944	0.887
0.986	1.011	0.967	1.009	0.999	0.909	0.917	0.94	0.973	0.969	0.987	0.98 .		0.996	0.986	1.011	1.003	0.95	0.94	0.978	0.99	0.921

1.009	0.997	0.937	1	0.993	0.921	0.908	0.916	1.004	0.977	0.981	0.971 .	1.005	1	1.031	0.987	0.932	0.928	0.984	0.985	0.924
1.012	1.009	0.985	0.997	0.967	0.957	0.92	0.952	0.989	0.968	0.973	0.994 .	1.012	0.963	1.016	0.996	0.939	0.967	0.988	1.003	0.916
0.989	0.994	0.968	1.036	0.979	0.924	0.903	0.948	1.001	1	0.992	0.993 .	1.018	0.992	1.01	0.992	0.967	0.965	0.993	0.986	0.914
0.992	1.011	0.929	1.001	1.008	0.926	0.926	0.945	0.989	0.98	0.997	0.981 .	1.042	1.011	1.004	1.009	0.945	0.925	0.973	0.981	0.914
0.993	1.023	0.955	1.009	0.969	0.923	0.914	0.976	1.01	0.977	0.997	0.994 .	1.04	1.001	1	0.98	0.924	0.951	0.956	0.958	0.924
1.002	1.008	0.914	1.002	1.002	0.95	0.924	0.959	0.99	0.986	0.988	0.993 .	1.053	1.011	1.012	1.006	0.966	0.974	0.99	0.995	0.913
0.991	0.99	0.948	0.985	0.974	0.924	0.918	0.927	0.976	0.967	1.006	0.983 .	1.042	1	0.98	0.987	0.958	0.951	0.973	0.999	0.917
1.002	0.999	0.944	0.993	0.984	0.946	0.957	0.962	0.979	0.951	0.972	0.984 .	1.017	0.992	0.992	0.993	0.959	0.957	0.965	0.987	0.923
0.995	0.987	0.954	0.971	0.992	0.939	0.921	0.964	0.995	0.981	1	0.996 .	1.015	0.978	0.985	0.98	0.924	0.971	1.02	0.969	0.937
0.971	0.984	0.943	1.018	0.975	0.918	0.938	0.962	0.985	0.979	1.004	1 .	1.057	1.012	0.984	0.958	0.922	0.935	1.008	1.004	0.913
1.006	1.007	0.925	1.011	0.972	0.947	0.929	0.957	1.019	0.991	1.007	0.985 .	1.027	1.01	1.008	0.987	0.93	0.975	1.031	0.958	0.939
0.966	1.021	0.931	1.008	0.929	0.928	0.941	0.943	0.991	0.953	0.993	0.974 .	1.015	1.016	0.969	0.992	0.93	0.926	1.013	0.955	0.948
0.922	1.02	0.939	0.987	0.847	0.92	0.94	0.983	0.983	0.997	1.045	0.998 .	1.048	1.01	0.982	0.998	0.919	0.96	0.99	0.988	0.898
0.908	1.049	0.937	1.016	0.834	0.946	0.977	0.98	0.982	0.971	1.001	0.986 .	1.028	0.997	1.016	0.984	0.949	0.961	0.958	0.943	0.947
0.896	1.014	0.902	0.992	0.792	0.971	0.913	0.978	0.988	0.985	1.013	0.996 .	1.016	1.003	1.018	1.006	0.931	0.914	0.917	0.963	0.932
0.881	1.031	0.942	1.023	0.857	0.937	0.924	0.97	1.008	0.971	1.015	1.011 .	1.053	1.036	1.024	0.97	0.95	0.951	0.942	0.97	0.899
0.95	0.999	0.949	1.013	0.907	0.946	0.923	0.97	1.018	0.993	1.023 .	.	1.036	1.006	1.019	0.98	0.982	0.952	0.934	0.986	0.925
0.897	1.031	0.917	0.972	0.948	0.939	0.937	0.937	1.015	0.983	1.01 .	.	1.007	0.989	1.014	0.959	0.951	0.942	0.894	1.016	0.92
0.95	1.063	0.949	1.024	0.944	0.948	0.941	0.961	1.016	0.993	1.014 .	.	1.019	0.973	1.005	1.008	0.969	0.925	0.973	1.012	0.929
0.993	1.052	0.968	1.026	0.958	0.949	0.951	0.953	1.007	0.976	1.029 .	.	1.044	0.965	1.02	0.979	0.976	0.931	1.004	0.995	0.917
0.945	1.025	0.976	0.989	0.927	0.97	0.958	0.95	1	0.978	1.051 .	.	1.018	0.957	0.985	0.947	0.985	0.927	0.997	1	0.918
0.975	1.035	0.966	1.02	0.907	0.955	0.925	0.973	0.998	0.977	1.013 .	.	1.016	0.943	1.012	0.965	0.985	0.932	1.009	1.013	0.921
0.971	1.05	1.008	1.055	0.789	0.925	0.966	0.998	1.003	0.989	1.006 .	.	1.014	0.94	0.972	0.965	0.98	0.901	1.005	0.981	0.893
1.017	1.038	0.956	1.031	0.905	0.906	0.887	0.923	0.999	0.997	0.983 .	.	0.999	0.895	0.98	0.989	0.983	0.846	0.997	1.016	0.841
1.004	1.026	0.937	0.971	0.912	0.939	0.9	0.911	1.028	1.004	1.053 .	.	0.98	0.877	1.04	0.906	0.959	0.883	1.044	0.983	0.843
0.985	1.018	0.943	0.93	0.906	0.914	0.865	0.903	1.041	1.007	1.085 .	.	0.956	0.906	1.018	0.895	1.007	0.87	1.004	0.978	0.858
1.017	1.015	0.965	0.93	0.931	0.937	0.917	0.884	1.014	0.999	1.032 .	.	0.992	0.823	0.98	0.883	0.968	0.912	0.982	0.993	0.882
1.014	0.968	1.008	0.887	0.916	0.932	0.923	0.818	1.031	1.007	1.04 .	.	0.997	0.854	1.033	0.977	1	0.938	1.036	0.978	0.867
0.997	0.971	0.991	0.845	0.893	0.939	0.937	0.903	1.017	1.005	0.997 .	.	1.006	0.848	1.018	0.996	0.99	0.929	1.043	1.017	0.874
1.052	0.938	1.051	0.784	0.972	0.943	0.92	0.919	1.042	1.013	1.035 .	.	1.039	0.821	0.994	1.032	0.971	0.97	1.038	1.008	0.846
1.031	0.901	0.992	0.754	0.871	0.985	0.922	0.9	1.046	0.991	1.053 .	.	1.019	0.782	1.008	1.035	1.016	0.957	1.008	0.989	0.907
0.992	0.967	0.993	0.785	0.776	0.944	0.92	0.973	1.034	1.003	1.009 .	.	1.029	0.825	1.005	1.02	0.973	1.023	1.097	1.025	0.881
0.944	1.047	1.001	0.791	0.75	0.942	0.964	0.996	1.026	0.99	1.014 .	.	1.036	0.756	1.023	1.026	1.01	1.009	1.007	1.02	0.913
0.933	1.068	1.002	0.86	0.797	0.914	0.993	1.001	1.062	1.018	1.036 .	.	1.055	0.765	0.956	1.043	0.964	0.982	1.014	1.024	0.917
1.023	1.055	0.983	0.788	0.744	0.938	0.929	0.99	1.027	0.999	1.028 .	.	1.045	0.817	0.885	0.998	0.965	0.972	1.042	0.984	0.876
1.014	1.06	1.011	0.773	0.754	0.934	0.914	1.01	1.021	0.968	1.001 .	.	1.076	0.771	0.826	1.038	0.962	0.948	1.033	1.015	0.847
1.035	1.055	0.995	0.781	0.727	0.936	0.916	0.967	1.053	0.972	0.974 .	.	1.075	0.774	0.824	1.057	0.991	0.941	1.037	1.018	0.873
1.034	1.042	1.02	0.784	0.753	0.949	0.891	0.963	1.047	0.95	0.979 .	.	1.073	0.827	0.896	0.996	0.972	0.931	0.969	1.011	0.818
0.973	1.054	0.998	0.805	0.743	0.952	0.882	0.987	1.037	0.94	0.967 .	.	1.035	0.817	0.918	1.051	0.954	0.943	1.012	1.007	0.862
0.999	1.038	0.98	0.791	0.717	0.97	0.936	0.986	1.038	0.971	0.947 .	.	0.994	0.817	0.869	1.074	0.958	0.876	1.039	1.013	0.851
1.003	1	1.038	0.951	0.738	0.932	0.882	0.948	1.056	0.987	0.926 .	.	1.005	0.807	0.96	1.049	1.004	0.866	1.017	1.012	0.836
1.022	1.011	1.001	0.942	0.748	0.943	0.907	0.963	1.027	0.922	0.96 .	.	1.078	0.794	0.95	0.999	0.988	0.872	0.992	0.939	0.831
1.05	1.081	1.002	0.978	0.763	0.951	0.858	0.981	1.042	0.92	0.959 .	.	1.071	0.8	0.907	1.011	0.946	0.774	0.966	0.966	0.863
1.052	1.096	1.009	0.963	0.733	0.967	0.938	1.01	1.006	0.87	1.013 .	.	1.052	0.846	0.891	1.006	0.916	0.786	0.938	0.968	0.895
1.066	1.05	1.014	0.943	0.733	0.973	0.903	1.01	1.034	0.895	1.057 .	.	1.039	0.933	0.921	1	0.933	0.832	1.017	0.924	0.916
1.066	1.073	0.991	0.842	0.724	0.971	0.943	0.998	1.01	0.91	1.052 .	.	1.082	1.007	0.857	1.005	0.944	0.931	0.996	0.867	0.843
1.027	1.063	0.995	0.939	0.723	0.955	0.916	1.008	1.007	0.95	1.026 .	.	1.081	1.043	0.913	0.99	0.941	0.942	0.975	0.95	0.88
1.042	1.093	0.99	0.994	0.776	0.965	0.938	1.012	1.001	0.905	1.073 .	.	1.041	1.034	0.988	0.96	0.89	0.981	1.01	0.984	0.901
1.075	1.078	1.018	1.017	0.933	0.993	0.922	1.025	1.037	0.889	1.097 .	.	1.025	0.994	0.928	1.004	0.925	1.031	1.069	1.032	0.885
1.034	1.057	0.973	0.992	1.002	0.965	0.909	1.017	0.994	0.96	1.109 .	.	1.043	0.992	0.955	1.022	0.851	0.99	1.031	0.996	0.876
1.046	1.09	1.01	1.027	1.001	0.975	0.962	0.993	1.038	1.053	1.069	1.025 .	1.116	1.031	1.042	0.995	0.852	0.999	1	1.052	0.908
1.076	1.068	0.972	1.033	0.984	0.969	0.999	1.02	1.076	1.045	1.073	1.048 .	1.067	1.08	0.99	1.019	0.93	0.962	0.991	1.011	0.911
1.097	1.035	1.024	1.088	0.944	0.983	1.011	0.982	1.056	1.048	1.1	1.068 .	1.073	1.111	0.991	1.054	0.95	1.016	1.069	1.015	0.915
1.08	1.065	0.994	1.092	0.963	1.022	1.025	1.04	1.05	1.055	1.062	1.048 .	1.086	1.046	1.067	1.034	0.973	1.024	1.063	1.032	0.983
1.046	1.093	0.967	1.053	0.933	0.989	1.027	0.997	1.083	1.046	1.055	1.016 .	1.114	1.041	1.059	1.028	0.945	1.003	1.051	1.042	0.973
1.042	1.065	0.933	1.053	0.936	1.013	1.008	1.012	1.067	1.011	1.073	1.005 .	1.065	1.055	1.022	1.062	0.908	1.043	1.036	1.019	0.987
1.025	1.039	0.901	1.013	0.935	0.995	1.021	1.037	1.077	1.039	1.088	1.001 .	1.065	1.021	1.023	1.017	0.875	0.961	0.988	0.996	0.951
1.061	1.055	0.945	1.029	0.979	0.975	1	1.014	1.054	1.029	1.048	1.034 .	1.007	1.01	1.009	1.006	0.918	0.95	0.994	0.998	0.958
1.065	1.054	0.998	1.039	0.984	0.967	1.01	1.022	1.047	1.061	1.07	1.059 .	1.005	0.995	1.026	1.044	0.963	0.968	1.053	0.966	0.966
1.053	1.033	0.953	1.003	0.993	0.953	0.953	0.975	1.025	1.023	1.061	1.048 .	1.037	1.018	1.008	1.03	0.971	0.986	1.029	1.013	0.921
1.059	1.089	0.96	0.99	1.026	0.983	0.965	0.99	1.034	1.029	1.045	1.054 .	1.06	1.018	1.024	1.054	0.986	0.988	1.018	1.05	0.875
1.062	1.059	0.974	0.99	1.027	0.98	0.981	1.006	1.043	1.036	1.055	1.007 .	1.067	1.069	0.991	1.033	0.988	0.99	0.962	1.01	0.889
1.063	1.051	1.035	0.992	1.012	0.976	0.983	0.968	1.046	1.049	1.063	1.027 .	1.054	1.028	1.02	1.026	1.006	0.993	1.003	1.016	0.902
1.064	1.089	0.986	1.015	1.041	0.973	0.987	0.98	1.038	1.052	1.074	1.053 .	1.055	1.038	1.038	1.054	1.014	0.994	1.029	1.041	0.937
1.076	1.066	0.994	0.988	0.99	0.97	0.967	1.005	1.062	1.055	1.1	1.028 .	1.074	1.016	1.014	1.054	0.981	1.008	1.019	1.013	0.957
1.05	1.032	1.01	1.002	1.028	0.988	0.985	1.008	1.04	1.071	1.066	1.015 .	1.03	0.951	1.02	1.038	0.99	1.027	0.965	1.019	1.002
1.049	1.012	1.006	1.034	1	0.981	0.989	0.947	1.041	1.062	1.085	1.044 .	1.056	0.95	1.015	1.037	0.898	1.007	0.96	1.053	0.983
1.07	1.015	0.989	1.014	0.99	0.978	0.992	0.949	1.061	1.048	1.035	1.039 .	1.006	0.908	1.043	0.995	0.964	0.988	0.92	1.065	0.993
1.022	0.971	1.012	1.036	1.011	0.97	0.988	0.947	1.05	1.069	1.09	1.064 .	1.003	0.952	1.047	0.93	0.95	1.032	0.905	1.079	0.993
1.007	0.946	0.992	1.027	1.001	0.98	0.97	0.92	1.039	1.051	1.054	1.042 .	0.989	1.014	0.988	0.964	0.927	1	0.948	1.013	0.997
1.014	1.037	0.978	1.043	0.985	0.962	0.893	0.981	1.042	1.042	1.034	1.053 .	0.904	1.034	1.028	1	0.895	0.98	0.921	0.995	0.986
1.018	1.047	0.978	1.064	0.99	0.955	0.893	0.951	1.035	1.052	1.021	1.044 .	0.951	1.049	1.066	0.97	0.879	1.006	0.942	0.979	0.985
1.004	1.001	0.98	1.06	0.999	0.949	0.902	1.001	1.047	1.032	1.074	1.051 .	0.881	0.977	1.032	0.931	0.869	0.983	0.899	0.985	0.976
0.894	1.012	0.898	1.095	0.984	0.951	0.894	0.962	1.02	1.033	1.028	1.035 .	0.828	0.989	1.015	1.006	0.746	0.969	0.883	1.02	0.929
0.84	0.999	0.84	1.062	0.997	1.013	0.947	0.96	1.075	1.055	1.062	1.054 .	0.964	0.99	0.992	1.019	0.84	1.045	0.878	1.032	0.928
0.929	0.93	0.899	1.004	0.989	0.992	0.957	0.965	1.034	1.038	1.052	1.05 .	0.974	1	0.986	0.986	0.835	1.002	0.958	0.976	0.853
0.997	0.902	0.948	1.055	1	0.993	0.943	0.928	1.07	1.056	1.031	1.05 .	0.896	0.999	0.993	1.013	0.856	1.014	0.924	0.921	0.923
1.012	0.842	0.957	1.047	0.961	1.027	0.905	0.974	1.054	1.08	1.08	1.09 .	0.936	0.954	0.992	1.006	0.862	1.009	0.846	0.892	0.973
1.031	0.808	0.9	1.088	0.987	1.017	0.876	1.007	1.057	1.068	1.051	1.083 .	0.826	0.924	0.988	1.009	0.888	0.952	0.817	0.857	0.969
1.026	0.95	0.926	1.066	1.017	1.009	0.855	0.928	1.074	1.062	1.055	1.044 .	0.871	0.905	0.992	1.004	0.843	0.953	0.847	0.968	0.993
1.03	1.03	0.961	1.038	0.977	1.015	0.876	0.938	1.066	1.053	1.056	1.044 .	0.982	0.854	0.949	0.99	0.815	0.887	0.882	0.964	1.006
0.993	1.046	0.953	1.138	0.99	0.971	0.849	0.94	1.065	1.029	1.042	1.038 .	1.053	0.934	0.879	0.945	0.777	0.796	0.876	0.921	0.984
0.967	1.048	0.988	1.062	1.028	0.96	0.776	0.924	1.047	1.045	1.027	1.008 .	1.053	1.004	0.916	0.919	0.883	0.735	0.788	0.923	0.92
0.988	1.044	0.957	1.046	1.019	0.949	0.865	0.986	1.041	1.027	1.007	1.003 .	0.994	1.022	0.97	0.893	0.913	0.859	0.927	0.961	0.966
0.999	1.007	0.833	1.045	1.024	0.928	0.841	0.97	1.036	1.014	1.036	1.025 .	0.958	0.988	0.957	0.809	0.903	0.91	0.917	0.938	0.974
0.926	0.976	0.798	1.075	1.001	0.999	0.87	0.978	1.034	1.022	1.033	1.038 .	0.981	0.984	0.967	0.909	0.932	0.911	0.925	0.9	0.992
0.866	1.085	0.915	1.056	1.03	1.001	0.806	0.92	1.034	1.033	0.97	1.018 .	1.022	0.928	0.932	0.927	0.887	0.914	1.039	0.916	0.986
0.908	1.099	0.966	0.997	1.008	1.033	0.884	0.929	1.05	1.027	0.93	1.016 .	1.005	0.963	0.888	0.924	0.923	0.96	0.956	0.865	0.995
0.967	1.058	0.948	1.04	1.009	1.003	0.932	0.998	1.052	1.051	0.988	1.038 .	1.024	0.992	0.84	0.794	0.863	0.997	0.875	0.858	0.958
1.008	1.017	0.839	1.127	1.041	1.019	1.037	0.992	1.037	1.031	0.969	0.991 .	1.011	1.016	0.808	0.789	0.778	1.007	0.98	0.931	0.922
0.975	1.01	0.776	1.11	1.023	1.001	0.947	0.985	1.033	1.027	1.024	1.007 .	1.005	1.022	0.826	0.796	0.733	0.942	0.951	0.864	0.912
0.9	1.01	0.893	1.041	1.051	1.021	0.997	1.012	1.027	1.011	1.009	1.033 .	0.961	0.979	0.87	0.862	0.75	0.897	0.845	0.796	0.89
0.903	0.953	1.05	1.093	0.982	1.015	0.963	1.026	1.006	1.018	0.971	1.02 .	1.028	0.965	0.866	0.991	0.84	1.013	0.988	0.821	0.835
0.906	0.889	1.014	1.075	1.015	0.981	0.968	1.027	1.037	1.023	0.966	1.045 .	1.052	1.038	0.828	0.955	0.926	1.024	1.086	0.897	0.904
0.824	0.887	1.039	1.012	0.96	0.964	1.012	1.023	1.037	1.066	0.937	1.039 .	1.032	0.98	0.826	0.956	0.856	1.015	1.041	0.848	0.916
0.815	0.844	1.018	0.922	0.963	0.979	0.958	0.982	1.047	1.043	0.901	1.007 .	1.042	0.982	0.773	0.904	0.944	0.967	1.038	0.778	0.924
0.798	0.809	1.018	0.992	0.945	0.993	0.974	0.956	1.01	1.006	0.944	1.059 .	1.021	0.966	0.903	0.841	0.925	0.932	1.091	0.806	0.923
0.819	0.827	0.953	1.05	0.883	0.993	0.946	0.974	1.001	1.014	0.897	1.022 .	1.09	1.001	1.02	0.825	0.969	0.868	1.099	0.785	0.896
0.808	0.834	0.981	1.021	0.824	1.006	0.882	0.979	1.014	1.038	0.872	1.027 .	1.085	0.933	1.006	0.83	0.952	0.92	1.083	0.766	0.891
0.777	0.78	0.978	0.945	0.804	0.996	0.907	0.909	1.009	0.974	0.861	1.004 .	1.051	0.992	1.054	0.789	0.981	0.856	1.048	0.71	0.878
0.796	0.824	0.971	0.844	0.812	0.97	0.882	0.868	1.003	1.002	0.857	0.999 .	1.075	1.001	1.103	0.912	0.993	0.811	1.036	0.684	0.912
0.789	0.78	0.9	0.87	0.772	0.949	0.877	0.823	1.089	0.992	0.866	1.004 .	1.107	1.044	1.068	0.911	1	0.836	1.013	0.758	0.934
0.773	0.82	0.922	0.815	0.761	0.961	0.867	0.849	1.082	0.983	1.006	0.987 .	1.072	0.992	0.996	0.829	1.008	0.805	1.066	0.795	0.897
0.79	0.845			0.893	0.936	0.801	0.871	1.019	0.922	1.035	0.964 .	1.089	1.029	1.06	0.924	1.002			0.725	0.879
0.787	0.801			0.84	0.98	0.805	0.866	1.036	0.892	0.986	1.05 .	1.091	0.994	1.019	0.842	0.949			0.716	0.792

0.82	0.813	0.864	0.938	0.873	0.855	1.045	0.889	1.03	1.079 .	1.028	0.969	1.112	0.849	0.914	0.829	0.791
0.802	0.781	0.862	0.916	0.925	0.86	1.005	0.888	1.11	1.009 .	0.964	0.9	1.088	0.889	0.899	0.892	0.851
0.795	0.832	0.846	0.893	0.847	0.82	0.929	0.866	1.096	1.059 .	1.037	0.989	1.061	0.811	0.958	0.82	0.9
0.765	0.781	0.881	0.954	0.915	0.898	0.952	0.865	1.047	1.025 .	1.078	1.009	1.099	0.77		0.799	0.954
0.793	0.786	0.883	0.956	0.968	0.857	0.981	0.859	1.043	1.014 .	1.021	1.011	1.023	0.822		0.828	0.966
0.875	0.955	0.939	0.902	0.909	0.799	0.977	0.875	1.004	0.938 .	1.01	0.98	1.032	0.785		0.938	0.926
0.79	1.009	0.879	0.924	0.928	0.864	1.027	0.832	1.059	0.961 .	0.99	0.973	1.088	0.835		0.978	0.953
0.823	0.988	0.89	0.934	0.974	0.848	0.991	0.833	1.01	0.984 .	1.032	0.932	1.083	0.935		0.975	0.915
0.941	1.05	0.906	0.908	0.989	0.942	0.975	0.826	0.942	1.033 .	0.972	0.926	1.049	0.978		0.999	0.919
1.061	1.133	0.888	0.85	1.014	0.933	0.999	0.807	0.945	1.004 .	0.943	1.009	1.103	1.046		0.986	0.885
1.031	1.06	0.914	0.82	1.028	0.916	1.046	0.824	0.956	0.986 .	1.03	1.058	1.028	1.034		0.958	0.961
1.072	1.048	0.859	0.889	0.966	0.956	1.065	0.88	0.943	0.976 .	1.094	1.042	1.047	1.05		1.062	1
1.111	1.06	0.924	0.914	0.968	0.932	1.011	0.983	0.856	0.991 .	1.077	1.059	1.022	1.049		1.031	1.021
1.088	1.086	0.912	0.871	0.919	0.911	0.987	1.036	0.954	1.054 .	1.048	0.995	1.072	1.074		1.016	1.011
1.051	1.116	0.871	0.873	0.91	0.932	0.97	1.036	1.006	1.056 .	1.051	1.004	1.086	1.065		1.018	0.981
1.06	1.092	0.788	0.948	0.934	0.926	0.909	1.067	0.981	1.052 .	0.999	1.002	1.076	1.051		0.998	0.973
1.077	1.072	0.81	0.968	0.892	0.98	0.95	1.045	1.021	1.049 .	1.003	0.944	1.085	1.04		0.97	1.004
1.065	1.04	0.877	0.932	0.875	1.028	0.876	1.024	1.063	1.016 .	0.96	0.998	1.035	1.044		0.933	0.958
1.066	1.02	0.858	0.915	0.897	0.964	0.834	1.02	1.053	0.974 .	0.945	1.034	1.049	1.058		0.973	0.926
1.062	1.052	0.807	0.843	0.886	0.964	0.874	1.028	1.047	1.022 .	0.943	1.035	1.058	1.027		1.006	0.894
1.053	1.053	0.87	0.873	0.839	0.975	0.958	1.072	1.072	1.052 .	1.001	1.028	1.047	1.047		1.015	0.869
1.045	1.044	0.893	0.862	0.848	0.994	1.006	1.061	1.071	1.071 .	1.054	1.076	1.04	1.054		0.992	0.787
1.077	1.106	0.896	0.908	0.832	0.972	1.048	1.076	1.066	1.041 .	1.056	1.069	1.058	1.038		1.026	0.761
1.095	1.074	0.91	0.934	0.888	0.97	1.04	1.069	1.088	1.028 .	1.016	1.033	1.066	1.054		1.007	0.741
1.097	1.092	0.908	0.961	0.939	0.968	1.07	1.125	1.094	0.986 .	1.005	1.03	1.064	1.055		1.015	0.818
1.037	1.078	0.951	0.955	0.937	0.995	1.078	1.081	1.086	0.975 .	0.99	1.041	1.067	1.036		0.999	0.873
1.018	1.09	0.949	0.976	0.956	0.982	1.016	1.087	1.081	0.869 .	1.01	1.068	1.042	1.031		0.999	0.926
1.029	1.113	0.975	0.921	0.886	0.951	1.04	1.073	1.051	0.899 .	1.013	1.092	1.077	1.009		0.964	0.932
1.048	1.114	0.885	0.902	0.863	0.951	1.023	1.04	1.019	1.017 .	0.945	1.035	1.109	1.02		0.947	0.952
1.03	1.129	0.93	0.927	0.839	0.937	1	1.056	1.021	1.061 .	0.9	1.01	1.033	0.994		0.995	0.931
1.05	1.031	0.895	0.95	0.836	0.89	0.941	1.058	1.097	1.077 .	0.89	0.989	1.09	0.981		0.992	0.925
0.986	1.029	0.82	0.96	0.784	0.856	0.971	1.089	1.063	1.031 .	0.838	0.969	1.11	0.897		1.001	0.918
0.962	1.002	0.9	0.991	0.89	0.816	0.983	1.054	1.069	1.029 .	0.967	0.937	1.085	0.91		0.947	0.939
0.904	0.983	0.935	1.004	0.907	0.747	0.99	1.024	1.051	1.046 .	0.975	1.003	1.03	0.979		0.918	0.929
0.859	0.973	0.987	0.984	0.978	0.775	1.014	0.973	1.056	1.047 .	0.917	1.023	1.086	1.019		0.895	0.947
0.908	0.903	0.928	0.958	0.958	0.807	0.974	1.023	1.024	0.987 .	0.876	0.99	1.117	0.983		0.861	0.952
0.958	0.862	0.975	0.898	0.954	0.778	0.953	1.05	1.009	0.899 .	0.846	0.993	1.061	0.901		0.868	0.91
0.923	0.814	1.031	0.857	0.961	0.756	0.828	1.026	1.07	0.954 .	0.832	0.927	1.047	0.862		0.844	0.877
0.911	0.79	1.04	0.841	0.96	0.795	0.884	1	1.056	0.976 .	0.772	0.926	1.022	0.954		0.868	0.845
0.857	0.915	1.02	0.83	0.979	0.865	0.958	0.983	1.06	0.994 .	0.893	0.894	1.007	0.965		0.978	0.893
0.867	0.94	0.992	0.84	0.975	0.768	1.026	1.012	1.034	0.939 .	0.931	0.792	0.931	0.977		0.948	0.91
0.935	0.865	0.949	0.921	0.915	0.821	1.002	1.027	1.041	0.922 .	1.019	0.822	0.911	0.958		0.943	0.931
0.936	0.847	0.997	0.968	0.953	0.834	0.964	1.05	0.98	0.852 .	1.01	0.852	0.999	0.909		0.871	0.905
0.901	0.898	1.044	0.932	0.922	0.83	0.93	0.982	1.013	0.929 .	0.928	0.905	1.058	0.865		0.838	0.875
0.865	1.019	1.022	0.965	0.919	0.811	0.939	0.986	1.023	0.992 .	0.892	1.024	1.064	0.94		0.962	0.873
0.825	1.072	0.963	0.968	0.955	0.757	0.839	0.997	1.017	1.033 .	0.94	1.043	1.051	0.847		0.95	0.851
0.827	1.021	0.934	0.933	0.962	0.782	0.936	1.007	1.032	0.998 .	0.864	0.989	1.018	0.808		0.926	0.812
0.901	0.98	0.835	0.925	1.017	0.886	1.046	0.943	0.972	1.001 .	0.949	1.004	0.99	0.884		0.836	0.891
0.855	0.986	0.878	0.933	0.999	0.92	1.082	0.898	1.004	0.988 .	0.878	0.994	0.957	0.796		0.972	0.948
0.815	0.962	0.898	0.896	0.991	0.965	1.036	0.863	0.976	0.984 .	0.85	0.919	0.962	0.757		1	0.959
0.767	0.919	0.888	0.868	0.936	0.905	1.053	0.828	0.952	0.9 .	0.843	0.955	0.941	0.781		0.97	0.935
0.806	0.835	0.791	0.895	0.969	0.872	1.071	0.879	0.903	0.922 .	0.815	1.038	0.919	0.809		0.919	0.923
0.807	0.829	0.789	0.932	0.948	0.83	1.07	1.009	0.987	1.033 .	0.982	1.076	0.865	0.867		0.844	0.885
0.784	0.829	0.78	0.98	0.954	0.745	1.07	1.062	1.014	1.097 .	1.084	1.069	0.943	0.828		0.955	0.918
0.751	0.999	0.774	0.868	0.942	0.704	1.074	1.034	0.928	1.07 .	1.102	1.058	1.024	0.862		1.036	0.924
0.834	1.106	0.739	0.883	0.927	0.872	1.042	0.999	0.886	1.067 .	1.113	0.984	1.129	0.803		1.06	0.928
0.949	1.081	0.736	0.833	0.921	0.908	1.034	1.054	0.943	1.086 .	1.079	1.003	1.103	0.898		1.061	0.89
1.005	1.06	0.699	0.853	0.89	0.955	0.998	0.997	0.981	1.055 .	1.026	0.937	1.071	0.927		0.99	0.887
0.93	1.058	0.687	0.833	0.88	0.93	1.037	0.972	0.948	1.044 .	1.045	0.878	1.068	0.881		0.978	0.871
0.881	1.039	0.709	0.82	0.884	0.924	0.959	0.937	0.95	1.029 .	0.987	0.902	1.051	0.876		0.968	0.867
0.984	0.999	0.81	0.789	0.898	0.916	1.034	0.931	0.907	1.018 .	1.031	0.98	1.037	0.861		0.957	0.816
0.965	0.987	0.94	0.74	0.881	0.937	1.003	1	0.855	0.996 .	1.078	0.968	0.994	0.775		1.005	0.869
0.952	0.999	0.985	0.804	0.876	0.987	1.013	1.005	0.824	0.942 .	1.073	0.977	1.025	0.796		0.921	0.847

0.785	1.002	0.847	0.909	0.852	0.789	0.869	0.902	0.928	0.948	0.911	0.929	0.898	0.855	0.851	0.844	0.843 .	0.784	0.882	0.865	0.824
0.773	1.019	0.86	0.915	0.85	0.863	0.84	0.954	0.932	0.908	0.895	0.926	0.903	0.799	0.818	0.859	0.853 .	0.797	0.85	0.838	0.844
0.744	1.001	0.879	0.866	0.864	0.794	0.74	0.937	0.901	0.934	0.922	0.897	0.895	0.819	0.854	0.855	0.79 .	0.744	0.895	0.836	0.852
0.785	0.938	0.889	0.882	0.881	0.777	0.72	0.912	0.891	0.92	0.89	0.879	0.848	0.767	0.846	0.817	0.812 .	0.727	0.89	0.863	0.836
0.841	0.906	0.835	0.863	0.866	0.808	0.705	0.934	0.881	0.935	0.887	0.845	0.861	0.773	0.88	0.796	0.873 .	0.705	0.903	0.853	0.836
0.833	0.805	0.828	0.823	0.821	0.856	0.761	0.964	0.896	0.888	0.903	0.847	0.898	0.833	0.868	0.831	0.85 .	0.722	0.876	0.862	0.859
0.814	0.777	0.798	0.865	0.837	0.865	0.84	0.936	0.856	0.872	0.887	0.862	0.886	0.863	0.876	0.834	0.848 .	0.691	0.841	0.823	0.868
0.846	0.79	0.812	0.815	0.818	0.814	0.875	0.927	0.889	0.874	0.896	0.882	0.865	0.844	0.825	0.827	0.861 .	0.67	0.842	0.834	0.866
0.831	0.85	0.832	0.828	0.829	0.8	0.854	0.898	0.887	0.894	0.875	0.875	0.901	0.884	0.848	0.836	0.825 .	0.672	0.833	0.832	0.852
0.794	0.882	0.825	0.812	0.811	0.801	0.854	0.858	0.879	0.89	0.891	0.855	0.867	0.817	0.775	0.832	0.861 .	0.689	0.817	0.837	0.863
0.816	0.87	0.803	0.832	0.798	0.831	0.829	0.849	0.882	0.89	0.848	0.806	0.866	0.838	0.815	0.815	0.868 .	0.708	0.798	0.838	0.855
0.847	0.871	0.787	0.816	0.822	0.844	0.834	0.836	0.864	0.896	0.875	0.833	0.842	0.836	0.838	0.835	0.87 .	0.666	0.822	0.843	0.83
0.829	0.848	0.782	0.805	0.794	0.844	0.797	0.821	0.857	0.87	0.838	0.852	0.868	0.863	0.848	0.815	0.842 .	0.678	0.803	0.799	0.808
0.824	0.845	0.8	0.81	0.786	0.81	0.803	0.793	0.851	0.873	0.871	0.865	0.846	0.823	0.828	0.823	0.786 .	0.7	0.827	0.801	0.802
0.794	0.814	0.816	0.822	0.783	0.8	0.792	0.781	0.828	0.865	0.836	0.84	0.846	0.823	0.851	0.785	0.808 .	0.687	0.815	0.796	0.829
0.786	0.88	0.816	0.83	0.78	0.788	0.774	0.761	0.826	0.823	0.834	0.82	0.835	0.789	0.828	0.767	0.835 .	0.692	0.843	0.842	0.763
0.801	0.824	0.791	0.855	0.794	0.808	0.793	0.76	0.809	0.807	0.828	0.811	0.843	0.811	0.818	0.78	0.791 .	0.792	0.818	0.823	0.76
0.8	0.838	0.767	0.835	0.794	0.787	0.781	0.744	0.829	0.809	0.824	0.825	0.847	0.797	0.812	0.828	0.799 .	0.851	0.828	0.844	0.783
0.788	0.826	0.791	0.808	0.799	0.81	0.787	0.795	0.833	0.825	0.832	0.835	0.855	0.825	0.836	0.794	0.791 .	0.787	0.801	0.815	0.792
0.779	0.802	0.806	0.801	0.779	0.825	0.768	0.77	0.845	0.811	0.81	0.833	0.86	0.81	0.842	0.772	0.769 .	0.793	0.811	0.772	0.781
0.778	0.814	0.768	0.838	0.811	0.792	0.774	0.772	0.864	0.826	0.851	0.848	0.859	0.816	0.82	0.753	0.78 .	0.822	0.78	0.777	0.78
0.801	0.854	0.785	0.84	0.784	0.81	0.76	0.755	0.832	0.845	0.814	0.848	0.853	0.837	0.816	0.796	0.811 .	0.786	0.793	0.781	0.761
0.811	0.837	0.768	0.819	0.791	0.81	0.796	0.765	0.833	0.813	0.826	0.845	0.834	0.821	0.813	0.788	0.819 .	0.862	0.795	0.824	0.775
0.811	0.841	0.739	0.835	0.772	0.802	0.809	0.768	0.813	0.822	0.824	0.842	0.834	0.8	0.812	0.799	0.788 .	0.809	0.809	0.789	0.791
0.788	0.808	0.76	0.858	0.815	0.806	0.768	0.762	0.823	0.807	0.807	0.809	0.819	0.808	0.817	0.772	0.782 .	0.776	0.763	0.781	0.806
0.789	0.787	0.727	0.808	0.791	0.815	0.787	0.746	0.809	0.804	0.787	0.801	0.819	0.819	0.835	0.764	0.776 .	0.77	0.776	0.772	0.797
0.796	0.792	0.778	0.796	0.793	0.793	0.768	0.758	0.814	0.79	0.782	0.818	0.81	0.813	0.835	0.793	0.826 .	0.792	0.752	0.785	0.788
0.793	0.81	0.798	0.817	0.803	0.797	0.767	0.763	0.835	0.826	0.802	0.821	0.829	0.799	0.828	0.78	0.828 .	0.805	0.775	0.78	0.766
0.772	0.827	0.769	0.824	0.777	0.796	0.747	0.763	0.84	0.817	0.809	0.822	0.831	0.787	0.83	0.786	0.821 .	0.789	0.783	0.793	0.793
0.784	0.82	0.75	0.826	0.77	0.77	0.725	0.781	0.818	0.832	0.806	0.807	0.834	0.791	0.835	0.789	0.786 .	0.77	0.769	0.775	0.783
0.762	0.798	0.717	0.811	0.775	0.766	0.715	0.779	0.806	0.831	0.81	0.821	0.829	0.781	0.822	0.776	0.786 .	0.735	0.754	0.758	0.801
0.746	0.791	0.732	0.787	0.783	0.806	0.769	0.767	0.81	0.866	0.837	0.809	0.833	0.796	0.805	0.788	0.766 .	0.732	0.769	0.752	0.789
0.743	0.792	0.754	0.789	0.777	0.775	0.783	0.731	0.803	0.826	0.818	0.796	0.822	0.828	0.788	0.781	0.764 .	0.785	0.79	0.761	0.772
0.744	0.76	0.72	0.773	0.755	0.825	0.794	0.746	0.809	0.872	0.836	0.823	0.801	0.834	0.809	0.809	0.773 .	0.746	0.763	0.783	0.781
0.771	0.774	0.722	0.801	0.775	0.812	0.759	0.785	0.832	0.867	0.794	0.825	0.813	0.808	0.776	0.795	0.783 .	0.773	0.792	0.772	0.794
0.761	0.817	0.775	0.81	0.798	0.805	0.777	0.784	0.831	0.852	0.796	0.828	0.81	0.837	0.818	0.803	0.81 .	0.79	0.804	0.723	0.81
0.768	0.779	0.733	0.785	0.795	0.77	0.766	0.742	0.812	0.857	0.821	0.825	0.817	0.865	0.821	0.81	0.785 .	0.752	0.767	0.728	0.75
0.741	0.804	0.742	0.821	0.808	0.778	0.77	0.783	0.802	0.842	0.8	0.797	0.788	0.83	0.784	0.783	0.817 .	0.771	0.764	0.758	0.744
0.769	0.775	0.729	0.762	0.779	0.796	0.785	0.766	0.813	0.879	0.798	0.802	0.784	0.838	0.809	0.778	0.789 .	0.748	0.769	0.726	0.742
0.785	0.795	0.718	0.8	0.792	0.773	0.777	0.775	0.798	0.844	0.807	0.823	0.791	0.795	0.796	0.802	0.829 .	0.713	0.775	0.806	0.778
0.761	0.781	0.755	0.806	0.813	0.766	0.766	0.761	0.804	0.828	0.806	0.793	0.795	0.841	0.833	0.781	0.812 .	0.729	0.779	0.852	0.78

0.793	0.8	0.733	0.812	0.811	0.772	0.745	0.773	0.808	0.819	0.814	0.829	0.777	0.819	0.83	0.811	0.805 .		0.692	0.749	0.813	0.78
0.77	0.797	0.72	0.843	0.813	0.765	0.767	0.772	0.804	0.827	0.809	0.833	0.744	0.816	0.797	0.795	0.795 .		0.687	0.754	0.812	0.767
0.765	0.8	0.748	0.795	0.78	0.78	0.773	0.769	0.802	0.816	0.804	0.823	0.732	0.815	0.803	0.789	0.807 .		0.734	0.753	0.815	0.774
0.733	0.826	0.794	0.807	0.791	0.795	0.787	0.772	0.822	0.843	0.831	0.819	0.772	0.799	0.818	0.834	0.836 .		0.702	0.77	0.804	0.768
0.765	0.816	0.777	0.797	0.796	0.784	0.783	0.764	0.808	0.814	0.799	0.793	0.788	0.785	0.792	0.785	0.811 .		0.706	0.774	0.801	0.741
0.786	0.794	0.776	0.787	0.805	0.778	0.775	0.762	0.823	0.851	0.844	0.816	0.821	0.832	0.796	0.827	0.801 .		0.697	0.786	0.844	0.793
0.798	0.767	0.754	0.829	0.81	0.752	0.78	0.811	0.851	0.856	0.838	0.787	0.789	0.816	0.809	0.84	0.821 .		0.715	0.733	0.805	0.788
0.801	0.793	0.821	0.834	0.825	0.765	0.794	0.733	0.835	0.86	0.864	0.827	0.791	0.813	0.814	0.852	0.81 .		0.785	0.764	0.836	0.824
0.771	0.793	0.843	0.786	0.808	0.797	0.81	0.764	0.845	0.842	0.871	0.82	0.798	0.827	0.847	0.845	0.811 .		0.787	0.756	0.827	0.812
0.782	0.782	0.829	0.795	0.811	0.806	0.819	0.803	0.843	0.851	0.846	0.846	0.822	0.859	0.864	0.805	0.825 .		0.78	0.831	0.79	0.847
0.788	0.82	0.813	0.78	0.824	0.811	0.768	0.814	0.861	0.839	0.863	0.831	0.842	0.848	0.855	0.822	0.811 .		0.737	0.829	0.828	0.802
0.83	0.837	0.794	0.785	0.833	0.825	0.82	0.816	0.827	0.826	0.805	0.839	0.835	0.83	0.815	0.832	0.818 .		0.734	0.844	0.812	0.773
0.876	0.835	0.794	0.809	0.807	0.783	0.775	0.801	0.878	0.826	0.846	0.853	0.833	0.834	0.83	0.848	0.855 .		0.733	0.836	0.788	0.779
0.835	0.847	0.821	0.83	0.822	0.858	0.777	0.844	0.843	0.849	0.847	0.844	0.835	0.835	0.824	0.845	0.826 .		0.813	0.856	0.82	0.839
0.811	0.784	0.818	0.882	0.82	0.826	0.738	0.805	0.826	0.836	0.856	0.831	0.853	0.829	0.871	0.812	0.76 .		0.84	0.83	0.813	0.788
0.821	0.776	0.793	0.908	0.825	0.796	0.76	0.799	0.861	0.84	0.885	0.85	0.803	0.852	0.816	0.806	0.755 .		0.775	0.786	0.774	0.798
0.824	0.771	0.849	0.871	0.806	0.829	0.76	0.773	0.864	0.86	0.874	0.852	0.849	0.872	0.837	0.795	0.772 .		0.793	0.82	0.776	0.795
0.819	0.777	0.803	0.784	0.795	0.835	0.748	0.803	0.868	0.867	0.855	0.835	0.82	0.9	0.881	0.794	0.808 .		0.758	0.828	0.767	0.806
0.817	0.793	0.791	0.79	0.805	0.836	0.79	0.797	0.855	0.877	0.905	0.875	0.836	0.863	0.867	0.821	0.819 .		0.774	0.838	0.814	0.797
0.839	0.781	0.774	0.747	0.807	0.839	0.818	0.828	0.86	0.887	0.889	0.836	0.838	0.848	0.823	0.811	0.814 .		0.759	0.827	0.791	0.813
0.827	0.786	0.772	0.831	0.826	0.82	0.763	0.807	0.866	0.895	0.863	0.854	0.847	0.852	0.869	0.794	0.832 .		0.78	0.814	0.82	0.778
0.822	0.8	0.829	0.845	0.814	0.832	0.753	0.805	0.872	0.858	0.861	0.849	0.852	0.863	0.856	0.823	0.824 .		0.716	0.775	0.778	0.792
0.837	0.766	0.804	0.791	0.801	0.844	0.762	0.83	0.879	0.859	0.842	0.844	0.853	0.889	0.845	0.786	0.83 .		0.749	0.785	0.81	0.805
0.848	0.793	0.841	0.822	0.818	0.863	0.781	0.803	0.889	0.848	0.873	0.869	0.811	0.873	0.849	0.797	0.801 .		0.722	0.77	0.814	0.836
0.826	0.809	0.776	0.798	0.827	0.824	0.743	0.773	0.844	0.82	0.865	0.852	0.805	0.894	0.874	0.788	0.845 .		0.815	0.796	0.847	0.796
0.837	0.828	0.769	0.839	0.849	0.849	0.824	0.816	0.869	0.836	0.848	0.87	0.795	0.839	0.859	0.824	0.845 .		0.799	0.785	0.84	0.832
0.846	0.839	0.827	0.86	0.834	0.84	0.794	0.82	0.854	0.848	0.839	0.861	0.813	0.873	0.838	0.846	0.853 .		0.791	0.783	0.811	0.82
0.806	0.811	0.836	0.822	0.824	0.829	0.773	0.792	0.888	0.89	0.879	0.854	0.778	0.876	0.854	0.862	0.839 .		0.78	0.814	0.78	0.84
0.819	0.806	0.847	0.803	0.811	0.836	0.804	0.829	0.855	0.863	0.862	0.882	0.831	0.86	0.855	0.849	0.844 .		0.786	0.823	0.81	0.845
0.793	0.791	0.824	0.808	0.834	0.839	0.825	0.81	0.842	0.888	0.889	0.879	0.801	0.816	0.819	0.818	0.83 .		0.752	0.876	0.834	0.823
0.82	0.83	0.805	0.832	0.788	0.826	0.812	0.795	0.847	0.857	0.879	0.865	0.805	0.799	0.815	0.825	0.847 .		0.763	0.838	0.824	0.833
0.827	0.808	0.827	0.876	0.79	0.821	0.829	0.808	0.878	0.854	0.886	0.896	0.818	0.858	0.819	0.798	0.845 .		0.82	0.787	0.801	0.82
0.811	0.814	0.836	0.831	0.794	0.842	0.812	0.799	0.88	0.842	0.833	0.875	0.827	0.854	0.841	0.801	0.848 .		0.814	0.8	0.853	0.843
0.815	0.809	0.821	0.882	0.81	0.806	0.801	0.808	0.866	0.875	0.819	0.867	0.834	0.834	0.829	0.806	0.854 .		0.782	0.797	0.84	0.847
0.818	0.852	0.817	0.877	0.816	0.835	0.797	0.754	0.842	0.872	0.857	0.824	0.84	0.847	0.831	0.83	0.851 .		0.766	0.816	0.832	0.857
0.84	0.807	0.804	0.862	0.809	0.833	0.812	0.809	0.811	0.878	0.875	0.879	0.833	0.816	0.845	0.835	0.829 .		0.762	0.785	0.801	0.844
0.831	0.821	0.81	0.83	0.813	0.807	0.824	0.78	0.817	0.858	0.86	0.834	0.811	0.87	0.83	0.84	0.825 .		0.791	0.786	0.792	0.804
0.829	0.797	0.813	0.808	0.775	0.818	0.814	0.807	0.85	0.856	0.826	0.856	0.819	0.884	0.829	0.799	0.815 .		0.797	0.783	0.754	0.84
0.807	0.763	0.806	0.77	0.772	0.801	0.825	0.784	0.852	0.864	0.834	0.862	0.823	0.834	0.816	0.773	0.822 .		0.792	0.76	0.744	0.797
0.773	0.791	0.786	0.778	0.79	0.793	0.813	0.776	0.83	0.822	0.827	0.837	0.802	0.81	0.775	0.787	0.82 .		0.786	0.764	0.801	0.791
0.776	0.786	0.745	0.793	0.789	0.793	0.815	0.766	0.842	0.82	0.832	0.811	0.825	0.81	0.789	0.777	0.817 .		0.741	0.777	0.813	0.755
0.79	0.771	0.748	0.798	0.785	0.763	0.773	0.758	0.824	0.811	0.846	0.821	0.817	0.807	0.796	0.758	0.787 .		0.792	0.749	0.79	0.759
0.81	0.781	0.743	0.823	0.777	0.783	0.785	0.77	0.824	0.819	0.843	0.837	0.818	0.805	0.791	0.757	0.775 .		0.78	0.783	0.784	0.756
0.802	0.79	0.767	0.828	0.787	0.769	0.789	0.771	0.821	0.841	0.808	0.834	0.819	0.798	0.819	0.771	0.793 .		0.776	0.754	0.767	0.753
0.803	0.816	0.77	0.816	0.786	0.784	0.768	0.75	0.834	0.819	0.806	0.818	0.817	0.832	0.832	0.789	0.791 .		0.76	0.749	0.769	0.757
0.788	0.807	0.767	0.814	0.792	0.784	0.812	0.767	0.846	0.819	0.826	0.829	0.816	0.835	0.808	0.764	0.781 .		0.744	0.77	0.801	0.732
0.804	0.79	0.797	0.808	0.758	0.784	0.795	0.779	0.833	0.845	0.819	0.828	0.825	0.799	0.813	0.766	0.778 .		0.77	0.802	0.759	0.757
0.788	0.791	0.758	0.791	0.768	0.788	0.77	0.758	0.85	0.852	0.851	0.834	0.8	0.804	0.813	0.79	0.796 .		0.714	0.782	0.778	0.734
0.776	0.787	0.779	0.809	0.77	0.787	0.789	0.762	0.835	0.851	0.821	0.808	0.803	0.797	0.808	0.81	0.814 .		0.754	0.788	0.755	0.747
0.785	0.781	0.752	0.787	0.777	0.789	0.798	0.755	0.83	0.839	0.838	0.825	0.803	0.83	0.807	0.81	0.781 .		0.74	0.75	0.754	0.727
0.789	0.761	0.751	0.776	0.778	0.79	0.813	0.781	0.815	0.846	0.824	0.825	0.808	0.841	0.804	0.791	0.767 .		0.767	0.776	0.793	0.755
0.769	0.751	0.792	0.807	0.749	0.784	0.772	0.77	0.836	0.85	0.829	0.822	0.801	0.839	0.799	0.793	0.776 .		0.76	0.779	0.75	0.737
0.812	0.754	0.782	0.833	0.797	0.782	0.766	0.778	0.835	0.844	0.835	0.832	0.788	0.83	0.832	0.777	0.802 .		0.767	0.773	0.761	0.736
0.822	0.783	0.798	0.832	0.791	0.824	0.793	0.82	0.808	0.792	0.817	0.789	0.824	0.844	0.814	0.823	0.798 .		0.785	0.783	0.757	0.788
0.795	0.804	0.785	0.801	0.773	0.811	0.778	0.794	0.822	0.854	0.826	0.813	0.82	0.846	0.807	0.824	0.759 .		0.768	0.772	0.769	0.767
0.798	0.799	0.779	0.832	0.785	0.804	0.776	0.792	0.796	0.839	0.815	0.819	0.808	0.823	0.829	0.816	0.805 .		0.775	0.776	0.794	0.739
0.775	0.779	0.779	0.814	0.78	0.792	0.764	0.783	0.817	0.865	0.808	0.821	0.804	0.783	0.827	0.792	0.79 .		0.79	0.788	0.801	0.755
0.777	0.771	0.771	0.854	0.753	0.825	0.798	0.777	0.799	0.877	0.828	0.817	0.805	0.775	0.78	0.827	0.778 .		0.769	0.757	0.811	0.764
0.75	0.746	0.759	0.822	0.749	0.833	0.813	0.782	0.825	0.853	0.828	0.812	0.779	0.806	0.808	0.793	0.787 .		0.775	0.758	0.803	0.764
0.803	0.758	0.761	0.845	0.763	0.81	0.794	0.793	0.835	0.852	0.834	0.827	0.783	0.844	0.804	0.811	0.814 .		0.78	0.759	0.836	0.786
0.823	0.805	0.774	0.838	0.732	0.768	0.789	0.817	0.813	0.838	0.817	0.805	0.801	0.799	0.776	0.818	0.83 .		0.78	0.764	0.789	0.76
0.809	0.789	0.719	0.804	0.743	0.792	0.781	0.802	0.794	0.829	0.814	0.807	0.828	0.793	0.802	0.812	0.782 .		0.793	0.775	0.768	0.732
0.798	0.763	0.801	0.793	0.776	0.795	0.792	0.798	0.81	0.836	0.826	0.81	0.799	0.829	0.804	0.782	0.773 .		0.777	0.764	0.755	0.785
0.781	0.782	0.783	0.799	0.772	0.809	0.83	0.836	0.808	0.844	0.812	0.797	0.814	0.855	0.797	0.784	0.783 .		0.798	0.771	0.773	0.798
0.786	0.782	0.803	0.775	0.757	0.805	0.822	0.821	0.843	0.845	0.805	0.832	0.816	0.841	0.803	0.793	0.811 .		0.79	0.779	0.787	0.786
0.773	0.767	0.766	0.767	0.738	0.821	0.781	0.788	0.822	0.851	0.817	0.831	0.802	0.842	0.801	0.822	0.805 .		0.737	0.731	0.797	0.767
0.782	0.795	0.774	0.746	0.771	0.817	0.802	0.791	0.856	0.849	0.817	0.818	0.799	0.835	0.854	0.799	0.783 .		0.773	0.727	0.822	0.8
0.774	0.807	0.77	0.825	0.78	0.812	0.818	0.82	0.865	0.845	0.816	0.814	0.818	0.824	0.857	0.812	0.779 .		0.75	0.781	0.793	0.808
0.822	0.766	0.783	0.827	0.792	0.781	0.791	0.788	0.86	0.85	0.795	0.814	0.775	0.878	0.865	0.791	0.791 .		0.773	0.814	0.811	0.78
0.825	0.735	0.764	0.811	0.793	0.761	0.78	0.77	0.857	0.865	0.804	0.819	0.77	0.837	0.846	0.841	0.826 .		0.734	0.788	0.8	0.778
0.801	0.731	0.741	0.793	0.734	0.789	0.767	0.764	0.843	0.885	0.776	0.82	0.783	0.874	0.806	0.826	0.837 .		0.729	0.82	0.758	0.786
0.825	0.746	0.709	0.789	0.782	0.832	0.765	0.791	0.855	0.869	0.775	0.82	0.799	0.82	0.813	0.814	0.851 .		0.795	0.817	0.742	0.788
0.813	0.775	0.727	0.834	0.816	0.817	0.767	0.76	0.845	0.858	0.821	0.781	0.751	0.798	0.813	0.836	0.848 .		0.841	0.827	0.781	0.745
0.84	0.838	0.814	0.901	0.781	0.804	0.73	0.764	0.877	0.851	0.836	0.822	0.753	0.842	0.892	0.826	0.86 .		0.807	0.812	0.761	0.748
0.857	0.856	0.839	0.866	0.823	0.817	0.701	0.787	0.824	0.86	0.798	0.843	0.794	0.906	0.877	0.824	0.861 .		0.841	0.818	0.807	0.756
0.816	0.83	0.838	0.874	0.845	0.817	0.731	0.815	0.816	0.891	0.825	0.797	0.861	0.899	0.883	0.806	0.815 .		0.834	0.81	0.84	0.823
0.806	0.847	0.866	0.853	0.805	0.818	0.754	0.828	0.801	0.856	0.813	0.856	0.842	0.828	0.853	0.814	0.816 .		0.815	0.837	0.813	0.81
0.848	0.864	0.824	0.842	0.823	0.838	0.764	0.8	0.819	0.88	0.817	0.822	0.866	0.846	0.874	0.83	0.787 .		0.814	0.807	0.785	0.839
0.831	0.861	0.828	0.869	0.769	0.82	0.714	0.819	0.837	0.88	0.856	0.805	0.845	0.86	0.826	0.878	0.822 .		0.835	0.803	0.804	0.845
0.829	0.863	0.847	0.864	0.783	0.841	0.703	0.819	0.846	0.871	0.837	0.793	0.823	0.881	0.846	0.863	0.829 .		0.872	0.845	0.809	0.806
0.844	0.851	0.779	0.839	0.834	0.832	0.726	0.825	0.881	0.855	0.842	0.858	0.842	0.849	0.824	0.858	0.809 .		0.796	0.826	0.823	0.834
0.824	0.828	0.787	0.884	0.831	0.829	0.789	0.812	0.894	0.918	0.868	0.863	0.838	0.875	0.84	0.814	0.842 .		0.798	0.838	0.81	0.82
0.849	0.873	0.836	0.863	0.822	0.837	0.757	0.797	0.892	0.905	0.852	0.824	0.826	0.843	0.813	0.827	0.835 .		0.837	0.77	0.772	0.799
0.867	0.828	0.83	0.834	0.814	0.814	0.766	0.799	0.834	0.871	0.825	0.83	0.825	0.794	0.793	0.859	0.845 .		0.856	0.752	0.826	0.792
0.844	0.826	0.75	0.794	0.819	0.836	0.784	0.812	0.848	0.865	0.839	0.808	0.819	0.834	0.825	0.833	0.812 .		0.858	0.76	0.838	0.805
0.81	0.839	0.729	0.744	0.823	0.838	0.765	0.811	0.895	0.899	0.827	0.798	0.828	0.82	0.802	0.853	0.86 .		0.815	0.779	0.787	0.832
0.837	0.818	0.753	0.733	0.795	0.815	0.705	0.827	0.87	0.865	0.856	0.834	0.824	0.801	0.824	0.837	0.826 .		0.8	0.779	0.726	0.81
0.845	0.81	0.787	0.794	0.802	0.831	0.759	0.839	0.844	0.816	0.835	0.815	0.836	0.854	0.814	0.85	0.844 .		0.764	0.779	0.813	0.817
0.863	0.838	0.795	0.756	0.808	0.846	0.852	0.795	0.845	0.876	0.826	0.775	0.789	0.798	0.771	0.821	0.835 .		0.832	0.826	0.828	0.837
0.863	0.849	0.786	0.752	0.821	0.828	0.809	0.823	0.849	0.862	0.828	0.83	0.819	0.849	0.786	0.814	0.848 .		0.863	0.832	0.791	0.836
0.812	0.844	0.78	0.77	0.819	0.807	0.822	0.774	0.882	0.837	0.826	0.808	0.826	0.819	0.814	0.786	0.851 .		0.804	0.832	0.825	0.839
0.836	0.861	0.822	0.816	0.776	0.846	0.87	0.83	0.88	0.831	0.845	0.795	0.81	0.85	0.832	0.839	0.833 .		0.807	0.838	0.809	0.837
0.841	0.844	0.809	0.791	0.811	0.845	0.85	0.825	0.856	0.849	0.852	0.876	0.795	0.821	0.848	0.767	0.83 .		0.816	0.819	0.816	0.827
0.857	0.844	0.814	0.764	0.786	0.823	0.802	0.834	0.852	0.855	0.866	0.864	0.801	0.86	0.844	0.787	0.84 .		0.825	0.785	0.786	0.798
0.834	0.845	0.831	0.723	0.799	0.87	0.795	0.798	0.854	0.836	0.831	0.825	0.781	0.828	0.835	0.848	0.842 .		0.808	0.783	0.808	0.846
0.82	0.82	0.749	0.74	0.778	0.822	0.874	0.831	0.86	0.855	0.871	0.834	0.819	0.844	0.852	0.841	0.804 .		0.746	0.79	0.775	0.845
0.806	0.813	0.797	0.776	0.748	0.825	0.821	0.812	0.834	0.808	0.855	0.849	0.828	0.83	0.837	0.834	0.805 .		0.805	0.813	0.785	0.807
0.812	0.824	0.79	0.768	0.764	0.831	0.822	0.816	0.852	0.824	0.813	0.802	0.846	0.821	0.842	0.863	0.835 .		0.809	0.814	0.793	0.83
0.793	0.842	0.798	0.783	0.8	0.819	0.817	0.808	0.859	0.853	0.842	0.794	0.816	0.825	0.848	0.837	0.823 .		0.822	0.801	0.807	0.814
0.793	0.839	0.798	0.808	0.772	0.793	0.852	0.806	0.819	0.86	0.868	0.83	0.852	0.799	0.844	0.828	0.846 .		0.808	0.802	0.815	0.828
0.84	0.823	0.796	0.785	0.794	0.825	0.881	0.815	0.83	0.845	0.856	0.849	0.846	0.852	0.802	0.82	0.837 .		0.833	0.843	0.791	0.862
0.861	0.822	0.796	0.845	0.798	0.824	0.781	0.803	0.852	0.844	0.858	0.851	0.837	0.839	0.828	0.832	0.818 .		0.812	0.833	0.772	0.811
0.821	0.839	0.81	0.82	0.802	0.83	0.791	0.796	0.839	0.844	0.806	0.81	0.841	0.828	0.835	0.823	0.834 .		0.8	0.824	0.775	0.806
0.813	0.806	0.789	0.772	0.779	0.819	0.855	0.822	0.846	0.846	0.839	0.826	0.83	0.816	0.841	0.82	0.834 .		0.786	0.803	0.754	0.847
0.826	0.786	0.78	0.785	0.761	0.805	0.837	0.794	0.81	0.812	0.807	0.826	0.832	0.8	0.821	0.815	0.823 .		0.802	0.788	0.762	0.807

0.803	0.812	0.757	0.78	0.776	0.811	0.811	0.791	0.815	0.827	0.833	0.842	0.827	0.81	0.855	0.802	0.805 .	0.769	0.796	0.776	0.793
0.797	0.812	0.814	0.801	0.779	0.78	0.773	0.777	0.801	0.819	0.816	0.797	0.834	0.803	0.844	0.784	0.814 .	0.779	0.815	0.776	0.788
0.81	0.793	0.785	0.852	0.788	0.778	0.771	0.784	0.8	0.806	0.802	0.794	0.807	0.778	0.825	0.808	0.815 .	0.767	0.795	0.798	0.787
0.787	0.789	0.763	0.821	0.763	0.813	0.801	0.791	0.8	0.802	0.814	0.823	0.792	0.814	0.819	0.82	0.794 .	0.794	0.771	0.78	0.812
0.794	0.805	0.792	0.802	0.79	0.813	0.807	0.775	0.824	0.843	0.801	0.829	0.819	0.772	0.843	0.834	0.836 .	0.795	0.783	0.785	0.803
0.79	0.808	0.798	0.833	0.785	0.805	0.816	0.799	0.809	0.806	0.806	0.795	0.825	0.814	0.822	0.821	0.818 .	0.803	0.804	0.771	0.803
0.824	0.823	0.795	0.801	0.776	0.811	0.778	0.81	0.803	0.824	0.817	0.798	0.819	0.824	0.817	0.832	0.8 .	0.815	0.821	0.775	0.81
0.787	0.808	0.731	0.782	0.77	0.803	0.784	0.821	0.808	0.832	0.831	0.807	0.824	0.801	0.831	0.83	0.847 .	0.799	0.793	0.786	0.799
0.788	0.82	0.743	0.81	0.8	0.795	0.768	0.813	0.803	0.849	0.815	0.811	0.793	0.79	0.814	0.824	0.838 .	0.778	0.775	0.81	0.765
0.811	0.791	0.761	0.836	0.771	0.808	0.793	0.811	0.823	0.823	0.805	0.811	0.781	0.787	0.805	0.819	0.815 .	0.78	0.814	0.77	0.793
0.814	0.844	0.731	0.79	0.797	0.799	0.81	0.819	0.835	0.841	0.82	0.836	0.813	0.791	0.802	0.818	0.812 .	0.788	0.759	0.779	0.794
0.768	0.829	0.735	0.781	0.796	0.812	0.794	0.818	0.835	0.864	0.85	0.826	0.781	0.786	0.788	0.822	0.806 .	0.766	0.721	0.799	0.821
0.807	0.826	0.707	0.786	0.774	0.817	0.778	0.821	0.834	0.85	0.846	0.828	0.806	0.789	0.791	0.822	0.802 .	0.804	0.752	0.77	0.8
0.815	0.831	0.741	0.82	0.767	0.819	0.809	0.824	0.819	0.839	0.834	0.819	0.784	0.758	0.814	0.795	0.779 .	0.853	0.784	0.759	0.797
0.783	0.808	0.834	0.771	0.806	0.802	0.783	0.799	0.838	0.832	0.839	0.834	0.76	0.747	0.796	0.817	0.792 .	0.819	0.784	0.762	0.786
0.744	0.789	0.826	0.764	0.801	0.817	0.802	0.842	0.806	0.831	0.822	0.827	0.783	0.736	0.76	0.811	0.825 .	0.81	0.78	0.785	0.799
0.783	0.809	0.783	0.842	0.766	0.796	0.796	0.801	0.799	0.817	0.808	0.804	0.816	0.763	0.792	0.803	0.844 .	0.816	0.762	0.743	0.789
0.768	0.798	0.727	0.867	0.782	0.793	0.79	0.825	0.818	0.813	0.807	0.826	0.798	0.844	0.776	0.785	0.786 .	0.801	0.742	0.807	0.78
0.783	0.792	0.733	0.833	0.784	0.804	0.817	0.815	0.815	0.832	0.8	0.816	0.795	0.842	0.83	0.731	0.81 .	0.806	0.771	0.798	0.81
0.798	0.838	0.786	0.827	0.787	0.804	0.783	0.797	0.8	0.822	0.817	0.833	0.786	0.784	0.818	0.805	0.815 .	0.816	0.813	0.782	0.806
0.79	0.847	0.768	0.821	0.729	0.817	0.779	0.8	0.787	0.797	0.808	0.817	0.779	0.799	0.791	0.83	0.817 .	0.831	0.756	0.803	0.787
0.811	0.814	0.755	0.809	0.771	0.816	0.782	0.833	0.814	0.822	0.78	0.838	0.8	0.819	0.798	0.835	0.793 .	0.768	0.771	0.826	0.793
0.804	0.791	0.804	0.844	0.769	0.8	0.759	0.774	0.842	0.831	0.8	0.831	0.806	0.797	0.791	0.843	0.808 .	0.824	0.77	0.759	0.775
0.8	0.805	0.799	0.815	0.757	0.788	0.773	0.808	0.846	0.835	0.809	0.811	0.812	0.786	0.757	0.825	0.795 .	0.822	0.754	0.791	0.782
0.749	0.757	0.77	0.852	0.753	0.786	0.769	0.814	0.834	0.816	0.811	0.819	0.77	0.767	0.785	0.797	0.767 .	0.809	0.771	0.774	0.791
0.768	0.787	0.796	0.825	0.777	0.812	0.765	0.805	0.836	0.837	0.808	0.836	0.774	0.759	0.793	0.833	0.776 .	0.793	0.787	0.808	0.754
0.791	0.819	0.766	0.859	0.772	0.825	0.767	0.812	0.831	0.836	0.797	0.838	0.808	0.771	0.817	0.808	0.832 .	0.832	0.765	0.784	0.777
0.782	0.836	0.719	0.798	0.797	0.825	0.802	0.839	0.821	0.83	0.811	0.844	0.811	0.787	0.767	0.811	0.805 .	0.827	0.756	0.752	0.775
0.742	0.799	0.727	0.81	0.748	0.808	0.784	0.797	0.798	0.803	0.812	0.822	0.801	0.793	0.78	0.781	0.775 .	0.815	0.761	0.755	0.784
0.805	0.785	0.772	0.858	0.769	0.807	0.803	0.839	0.849	0.825	0.855	0.828	0.793	0.764	0.827	0.761	0.776 .	0.834	0.777	0.768	0.801
0.786	0.765	0.75	0.834	0.752	0.791	0.777	0.818	0.84	0.804	0.848	0.819	0.774	0.772	0.83	0.783	0.824 .	0.834	0.781	0.739	0.776
0.774	0.838	0.727	0.866	0.756	0.792	0.811	0.805	0.84	0.815	0.823	0.822	0.807	0.833	0.792	0.817	0.761 .	0.821	0.74	0.752	0.796
0.856	0.857	0.705	0.835	0.795	0.832	0.804	0.814	0.838	0.845	0.812	0.817	0.813	0.835	0.777	0.787	0.782 .	0.858	0.783	0.804	0.787
0.83	0.868	0.79	0.796	0.827	0.828	0.822	0.81	0.841	0.836	0.841	0.839	0.823	0.811	0.81	0.799	0.825 .	0.796	0.824	0.778	0.8
0.844	0.84	0.769	0.847	0.791	0.835	0.803	0.803	0.865	0.812	0.844	0.809	0.854	0.82	0.826	0.848	0.817 .	0.842	0.849	0.806	0.73
0.86	0.831	0.814	0.835	0.789	0.825	0.777	0.788	0.831	0.834	0.84	0.825	0.817	0.802	0.816	0.872	0.775 .	0.846	0.806	0.813	0.756
0.842	0.804	0.817	0.848	0.792	0.85	0.803	0.782	0.834	0.85	0.83	0.858	0.791	0.794	0.829	0.846	0.735 .	0.864	0.774	0.813	0.781
0.849	0.843	0.829	0.88	0.792	0.831	0.825	0.825	0.849	0.783	0.836	0.856	0.834	0.767	0.819	0.855	0.811 .	0.846	0.781	0.772	0.815
0.828	0.859	0.787	0.847	0.787	0.821	0.824	0.773	0.832	0.807	0.829	0.862	0.835	0.825	0.835	0.808	0.831 .	0.807	0.805	0.792	0.821
0.845	0.82	0.825	0.827	0.752	0.844	0.879	0.814	0.863	0.845	0.841	0.839	0.826	0.811	0.812	0.822	0.794 .	0.831	0.841	0.782	0.828
0.876	0.838	0.762	0.819	0.802	0.853	0.815	0.823	0.827	0.832	0.841	0.823	0.853	0.767	0.816	0.832	0.773 .	0.765	0.797	0.825	0.815
0.84	0.813	0.791	0.749	0.835	0.867	0.824	0.814	0.812	0.806	0.831	0.806	0.839	0.829	0.826	0.815	0.801 .	0.76	0.751	0.775	0.792
0.849	0.833	0.83	0.803	0.774	0.826	0.863	0.841	0.858	0.822	0.848	0.8	0.802	0.813	0.855	0.822	0.833 .	0.758	0.752	0.759	0.828
0.838	0.857	0.811	0.874	0.77	0.83	0.801	0.813	0.856	0.791	0.836	0.824	0.83	0.824	0.812	0.852	0.818 .	0.813	0.729	0.805	0.822
0.829	0.875	0.804	0.814	0.781	0.868	0.815	0.82	0.819	0.789	0.827	0.837	0.826	0.822	0.82	0.857	0.859 .	0.819	0.781	0.814	0.806
0.836	0.845	0.796	0.857	0.787	0.829	0.794	0.817	0.853	0.843	0.86	0.839	0.798	0.799	0.827	0.84	0.835 .	0.817	0.825	0.752	0.81
0.839	0.854			0.8	0.809	0.805	0.8	0.862	0.827	0.822	0.846	0.794	0.803	0.77	0.866	0.833			0.758	0.808
0.872	0.835			0.793	0.801	0.826	0.8	0.82	0.811	0.858	0.85	0.777	0.785	0.839	0.846	0.819			0.825	0.801
0.834	0.815			0.79	0.844	0.836	0.818	0.849	0.814	0.858	0.838	0.793	0.86	0.836	0.844	0.789			0.773	0.827
0.846	0.797			0.772	0.837	0.796	0.827	0.842	0.835	0.834	0.838	0.775	0.834	0.832	0.844	0.789			0.804	0.862
0.832	0.785			0.809	0.812	0.825	0.818	0.85	0.804	0.851	0.861	0.789	0.837	0.852	0.841	0.867			0.855	0.845
0.856	0.898			0.825	0.819	0.847	0.821	0.869	0.802	0.863	0.823	0.789	0.781	0.813	0.829	0.851			0.811	0.85
0.829	0.874			0.794	0.83	0.824	0.82	0.861	0.814	0.848	0.851	0.786	0.805	0.851	0.828	0.829			0.794	0.838
0.826	0.811			0.743	0.786	0.854	0.842	0.839	0.822	0.849	0.849	0.825	0.829	0.832	0.801	0.791			0.81	0.832
0.817	0.83			0.746	0.811	0.821	0.837	0.85	0.834	0.857	0.82	0.843	0.848	0.847	0.821	0.863			0.768	0.821
0.851	0.848			0.799	0.84	0.81	0.829	0.818	0.803	0.862	0.785	0.811	0.802	0.843	0.808	0.881			0.818	0.782
0.856	0.83			0.793	0.804	0.86	0.807	0.86	0.793	0.812	0.824	0.825	0.828	0.807	0.804	0.834			0.844	0.831
0.824	0.801			0.757	0.823	0.861	0.793	0.853	0.842	0.832	0.844	0.786	0.84	0.816	0.771	0.804			0.822	0.861
0.841	0.838			0.721	0.82	0.855	0.832	0.815	0.781	0.827	0.787	0.804	0.843	0.811	0.82	0.847			0.804	0.891
0.801	0.832			0.745	0.804	0.839	0.788	0.838	0.789	0.829	0.832	0.845	0.873	0.807	0.845	0.824			0.795	0.847
0.832	0.846			0.77	0.834	0.892	0.842	0.834	0.775	0.84	0.836	0.836	0.839	0.831	0.824	0.84			0.79	0.894
0.844	0.849			0.796	0.851	0.844	0.86	0.827	0.791	0.816	0.814	0.832	0.851	0.831	0.83	0.865			0.769	0.905
0.822	0.842			0.776	0.842	0.828	0.803	0.828	0.835	0.822	0.845	0.85	0.829	0.843	0.837	0.861			0.814	0.824
0.821	0.833			0.767	0.812	0.824	0.786	0.819	0.832	0.799	0.834	0.823	0.792	0.818	0.815	0.829			0.811	0.819
0.817	0.839			0.751	0.846	0.816	0.814	0.84	0.84	0.829	0.851	0.794	0.8	0.813	0.824	0.822			0.774	0.831
0.809	0.82			0.759	0.79	0.825	0.792	0.82	0.8	0.791	0.827	0.814	0.804	0.796	0.827	0.829			0.773	0.809
0.809	0.808			0.774	0.813	0.833	0.797	0.83	0.804	0.815	0.844	0.812	0.775	0.82	0.787	0.804			0.787	0.802
0.799	0.816			0.732	0.802	0.805	0.791	0.818	0.825	0.814	0.824	0.785	0.787	0.853	0.797	0.787			0.766	0.791
0.813	0.819			0.754	0.792	0.789	0.798	0.825	0.811	0.793	0.829	0.796	0.792	0.814	0.796	0.798			0.806	0.792
0.775	0.795			0.737	0.813	0.812	0.793	0.822	0.837	0.779	0.837	0.808	0.817	0.809	0.752	0.787			0.792	0.801
0.813	0.808			0.76	0.808	0.801	0.777	0.822	0.814	0.808	0.842	0.804	0.776	0.776	0.756	0.803			0.826	0.814
0.821	0.824			0.758	0.803	0.779	0.804	0.809	0.828	0.795	0.844	0.789	0.807	0.835	0.798	0.814			0.795	0.793
0.8	0.819			0.705	0.791	0.786	0.802	0.812	0.837	0.806	0.817	0.784	0.78	0.789	0.799	0.806			0.791	0.822
0.805	0.795			0.754	0.8	0.765	0.781	0.831	0.826	0.837	0.844	0.828	0.773	0.807	0.81	0.811			0.774	0.802
0.784	0.832			0.758	0.814	0.791	0.783	0.834	0.852	0.829	0.818	0.821	0.788	0.821	0.797	0.799			0.784	0.795
0.797	0.86			0.73	0.823	0.797	0.8	0.817	0.819	0.839	0.83	0.795	0.774	0.824	0.817	0.792			0.8	0.795
0.792	0.83			0.737	0.799	0.796	0.782	0.825	0.833	0.783	0.807	0.799	0.747	0.806	0.769	0.818			0.812	0.792
0.735	0.825			0.737	0.811	0.807	0.828	0.804	0.791	0.781	0.821	0.772	0.766	0.798	0.811	0.827			0.807	0.804
0.765	0.84			0.773	0.767	0.8	0.77	0.818	0.819	0.812	0.829	0.772	0.811	0.794	0.843	0.811			0.762	0.786
0.822	0.857			0.748	0.808	0.813	0.818	0.824	0.835	0.817	0.831	0.796	0.811	0.833	0.835	0.832			0.762	0.799
0.808	0.846			0.712	0.811	0.783	0.799	0.79	0.795	0.796	0.82	0.794	0.775	0.826	0.814	0.81			0.77	0.81
0.808	0.845			0.722	0.814	0.77	0.806	0.802	0.833	0.799	0.837	0.809	0.754	0.829	0.802	0.815			0.802	0.783
0.81	0.843			0.718	0.828	0.816	0.792	0.808	0.845	0.811	0.835	0.769	0.738	0.826	0.797	0.81			0.811	0.78
0.806	0.811			0.771	0.792	0.797	0.78	0.791	0.807	0.822	0.829	0.756	0.715	0.822	0.744	0.793			0.749	0.757
0.767	0.827			0.719	0.805	0.796	0.773	0.825	0.822	0.822	0.837	0.761	0.804	0.816	0.742	0.797			0.814	0.754
0.808	0.813			0.75	0.809	0.811	0.802	0.786	0.817	0.82	0.827	0.744	0.766	0.826	0.745	0.772			0.756	0.784
0.786	0.795			0.724	0.775	0.808	0.803	0.767	0.823	0.829	0.838	0.76	0.775	0.82	0.798	0.775			0.732	0.758
0.773	0.832			0.75	0.813	0.823	0.811	0.798	0.83	0.82	0.845	0.77	0.794	0.809	0.832	0.784			0.787	0.802
0.795	0.83			0.763	0.799	0.776	0.809	0.83	0.831	0.823	0.834	0.76	0.794	0.8	0.84	0.83			0.789	0.817
0.818	0.832			0.765	0.806	0.784	0.805	0.814	0.835	0.821	0.825	0.763	0.788	0.82	0.801	0.813			0.797	0.826
0.809	0.843			0.741	0.824	0.794	0.797	0.842	0.808	0.814	0.827	0.755	0.768	0.78	0.784	0.792			0.788	0.8
0.813	0.806			0.738	0.794	0.832	0.798	0.815	0.819	0.823	0.85	0.746	0.765	0.774	0.792	0.813			0.792	0.775
0.839	0.796			0.763	0.807	0.795	0.789	0.822	0.837	0.789	0.842	0.814	0.795	0.8	0.766	0.801			0.782	0.764
0.815	0.818			0.744	0.825	0.807	0.805	0.823	0.847	0.802	0.832	0.834	0.817	0.85	0.753	0.797			0.765	0.75
0.806	0.814			0.711	0.806	0.785	0.785	0.837	0.825	0.84	0.871	0.798	0.79	0.828	0.836	0.817			0.783	0.765
0.81	0.854			0.757	0.797	0.802	0.824	0.835	0.841	0.853	0.851	0.778	0.797	0.805	0.802	0.798			0.794	0.748
0.832	0.846			0.774	0.792	0.796	0.79	0.829	0.83	0.852	0.839	0.822	0.77	0.783	0.827	0.773			0.766	0.787
0.788	0.793			0.815	0.81	0.806	0.816	0.848	0.855	0.846	0.858	0.802	0.823	0.776	0.837	0.78			0.775	0.816
0.834	0.826			0.779	0.785	0.829	0.816	0.878	0.799	0.835	0.857	0.752	0.827	0.801	0.812	0.809			0.803	0.829
0.853	0.842			0.803	0.822	0.824	0.745	0.842	0.802	0.834	0.876	0.746	0.837	0.842	0.815	0.829			0.765	0.825
0.827	0.85			0.823	0.824	0.828	0.775	0.844	0.844	0.841	0.868	0.746	0.838	0.804	0.825	0.832			0.832	0.824
0.832	0.837			0.793	0.808	0.817	0.775	0.819	0.868	0.835	0.831	0.839	0.872	0.819	0.793	0.788			0.769	0.806
0.795	0.826			0.796	0.828	0.833	0.828	0.83	0.87	0.839	0.867	0.826	0.845	0.823	0.777	0.792			0.812	0.824
0.821	0.851			0.789	0.822	0.833	0.802	0.81	0.848	0.871	0.846	0.834	0.829	0.798	0.787	0.864			0.792	0.833
0.822	0.81			0.788	0.863	0.864	0.846	0.875	0.807	0.866	0.846	0.839	0.83	0.787	0.772	0.8			0.804	0.84

0.821	0.819	0.8	0.85	0.831	0.816	0.872	0.832	0.85	0.822	0.834	0.827	0.82	0.851	0.829	0.809	0.803
0.759	0.811	0.791	0.821	0.841	0.804	0.864	0.854	0.857	0.843	0.8	0.839	0.821	0.838	0.825	0.75	0.849
0.749	0.846	0.817	0.82	0.829	0.861	0.854	0.828	0.876	0.849	0.792	0.804	0.834	0.841	0.845	0.766	0.835
0.779	0.835	0.745	0.779	0.843	0.802	0.849	0.836	0.859	0.817	0.821	0.862	0.874	0.831	0.852	0.829	0.81
0.83	0.834	0.716	0.831	0.848	0.818	0.849	0.816	0.844	0.847	0.828	0.851	0.823	0.824	0.863	0.787	0.825
						0.853	0.843	0.86	0.802							
						0.849	0.831	0.86	0.796							

9	2	2	5	8	6	6	9	9	11	11	12	11	3	10
Box-8	Box-1	Box-5	Box-2	Box-6	Box-5	Box-7	Box-4	Box-5	Box-3	Box-7	Box-8	Box-8	Box-8	Box-8
75	13.7	20.2	32.3	44.5	45	47	65	68	78	84	Z	Z	Z	Z
M	M	M	M	M	M	M	M	M	M	M	Z	Z	Z	Z
Malto	MCT	MCT	MCT	MCT	MCT	MCT	MCT	MCT	MCT	MCT	Z	Z	Z	Z
MM	MT	MT	MT	MT	MT	MT	MT	MT	MT	MT	Z	Z	Z	Z
0.756	0.736	0.741	0.709	0.662	0.936	0.948	0.786	0.704	0.815	0.8	0.735	-	.	.
0.72	0.745	0.774	0.756	0.683	0.911	0.928	0.677	0.721	0.814	0.79	0.795	-	.	.
0.748	0.766	0.744	0.735	0.709	0.918	0.96	0.695	0.732	0.789	0.788	0.738	-	.	.
0.726	0.706	0.721	0.738	0.681	0.929	0.958	0.73	0.711	0.773	0.765	0.802	-	.	.
0.742	0.746	0.753	0.738	0.661	0.92	0.932	0.728	0.74	0.744	0.751	0.869	-	.	.
0.714	0.716	0.755	0.724	0.683	0.911	0.917	0.738	0.702	0.73	0.754	0.863	-	.	.
0.71	0.731	0.733	0.751	0.688	0.882	0.94	0.772	0.732	0.757	0.75	0.772	-	.	.
0.739	0.788	0.749	0.753	0.724	0.943	0.938	0.845	0.713	0.745	0.75	0.795	-	.	.
0.715	0.873	0.818	0.792	0.758	0.943	0.936	0.842	0.711	0.744	0.736	0.824	-	.	.
0.706	0.896	0.852	0.793	0.809	0.978	0.946	0.857	0.72	0.729	0.711	0.769	-	.	.
0.727	0.91	0.891	0.828	0.778	0.972	0.948	0.838	0.726	0.721	0.719	0.87	-	.	.
0.709	0.889	0.887	0.849	0.768	0.946	0.946	0.858	0.726	0.828	0.739	0.894	-	.	.
0.686	0.905	0.881	0.813	0.823	0.928	0.927	0.886	0.731	0.865	0.749	0.709	-	.	.
0.694	0.915	0.915	0.831	0.835	0.939	0.926	0.879	0.734	0.88	0.812	0.907	-	.	.
0.691	0.904	0.921	0.861	0.848	0.993	0.969	0.878	0.729	0.903	0.849	0.911	-	.	.
0.712	0.945	0.916	0.882	0.86	0.952	0.98	0.876	0.721	0.915	0.863	0.905	-	.	.
0.712	0.913	0.89	0.892	0.851	0.975	0.943	0.877	0.805	0.905	0.838	0.919	-	.	.
0.824	0.936	0.898	0.883	0.865	0.978	0.986	0.884	0.793	0.915	0.832	0.972	-	.	.
0.827	0.927	0.929	0.842	0.87	0.956	0.946	0.891	0.787	0.9	0.824	0.943	-	.	.
0.812	0.927	0.924	0.847	0.877	0.951	0.95	0.895	0.754	0.922	0.841	0.996	-	.	.
0.834	0.935	0.927	0.85	0.901	0.922	0.925	0.918	0.795	0.915	0.832	1.067	-	.	.
0.88	0.931	0.985	0.87	0.891	0.937	0.937	0.924	0.84	0.896	0.821	1.029	-	.	.
0.859	0.902	0.931	0.878	0.875	0.933	0.922	0.921	0.833	0.926	0.801	1.04	-	.	.
0.869	0.944	0.935	0.888	0.878	0.961	0.937	0.942	0.803	0.965	0.811	1.05	-	.	.
0.901	0.916	0.93	0.867	0.859	0.928	0.952	0.904	0.783	0.939	0.774	1.08	-	.	.
0.929	0.947	0.925	0.848	0.921	0.962	0.986	0.926	0.781	0.952	0.777	0.995	-	.	.
0.878	0.984	0.934	0.846	0.917	0.929	0.976	0.95	0.761	0.949	0.774	1.063	-	.	.
0.854	0.935	0.898	0.848	0.904	0.904	0.972	0.963	0.736	0.944	0.755	1.096	-	.	.
0.916	0.96	0.921	0.832	0.929	0.911	0.932	0.955	0.795	0.923	0.76	1.169	-	.	.
0.903	0.962	0.914	0.798	0.911	0.939	0.959	0.924	0.766	0.93	0.744	1.039	-	.	.
0.913	0.969	0.902	0.799	0.885	0.921	0.925	0.908	0.79	0.936	0.75	0.965	-	.	.
0.885	0.915	0.932	0.813	0.903	0.865	0.926	0.944	0.785	0.928	0.774	0.981	-	.	.
0.931	0.92	0.944	0.779	0.916	0.907	0.996	0.945	0.776	0.929	0.77	1.006	-	.	.
0.93	0.889	0.909	0.801	0.909	0.908	0.912	0.942	0.779	0.924	0.759	0.917	-	.	.
0.919	0.87	0.913	0.8	0.9	0.939	0.955	0.925	0.746	0.942	0.774	0.941	-	.	.
0.892	0.901	0.936	0.786	0.911	0.905	0.909	0.899	0.717	0.919	0.736	0.981	-	.	.
0.906	0.9	0.954	0.769	0.897	0.933	0.942	0.91	0.779	0.947	0.734	0.838	-	.	.
0.918	0.886	0.93	0.78	0.888	0.891	0.953	0.891	0.768	0.939	0.748	0.865	-	.	.
0.942	0.873	0.921	0.764	0.914	0.941	0.921	0.975	0.794	0.937	0.762	0.983	-	.	.
0.926	0.804	0.929	0.772	0.859	0.898	0.904	0.941	0.761	0.979	0.776	1.066	-	.	.
0.885	0.814	0.918	0.782	0.851	0.96	0.887	0.955	0.797	0.969	0.773	0.913	-	.	.
0.891	0.869	0.905	0.744	0.871	0.885	0.849	0.931	0.767	0.934	0.758	1.017	-	.	.
0.884	0.899	0.952	0.767	0.886	0.958	0.877	0.899	0.749	0.939	0.754	1.098	-	.	.
0.942	0.787	0.931	0.719	0.862	0.906	0.89	0.927	0.774	0.957	0.737	0.993	-	.	.
0.95	0.794	0.893	0.719	0.818	0.852	0.842	0.951	0.756	0.983	0.755	1.053	-	.	.
0.952	0.823	0.937	0.755	0.87	0.889	0.787	0.93	0.794	0.962	0.786	1.089	-	.	.
0.93	0.877	0.903	0.735	0.873	0.932	0.833	0.92	0.828	0.991	0.79	0.984	-	.	.
0.885	0.843	0.887	0.704	0.816	0.921	0.862	0.883	0.777	0.944	0.804	0.862	-	.	.
0.918	0.811	0.904	0.747	0.801	0.953	0.865	0.92	0.767	0.929	0.783	1.003	-	.	.
0.906	0.858	0.85	0.7	0.801	0.906	0.845	0.949	0.78	0.893	0.775	1.026	-	.	.
0.906	0.844	0.89	0.739	0.796	0.906	0.83	0.923	0.78	0.903	0.763	0.955	-	.	.
0.911	0.788	0.906	0.733	0.825	0.93	0.866	0.894	0.767	0.937	0.768	0.975	-	.	.
0.901	0.767	0.909	0.743	0.78	0.913	0.888	0.932	0.801	0.936	0.745	0.936	-	.	.
0.877	0.778	0.877	0.692	0.751	0.888	0.809	0.924	0.846	0.913	0.795	0.965	-	.	.
0.903	0.762	0.842	0.721	0.794	0.876	0.838	0.908	0.821	0.903	0.775	1.005	-	.	.
0.921	0.721	0.907	0.783	0.843	0.879	0.857	0.888	0.799	0.921	0.814	0.975	-	.	.
0.918	0.755	0.911	0.806	0.85	0.872	0.913	0.942	0.784	0.899	0.765	1.005	-	.	.
0.887	0.777	0.929	0.834	0.771	0.832	0.902	0.903	0.757	0.873	0.78	0.975	-	.	.
0.913	0.83	0.923	0.831	0.781	0.777	0.877	0.95	0.784	0.897	0.758	0.928	-	.	.
0.905	0.89	0.92	0.827	0.754	0.869	0.88	0.934	0.793	0.91	0.799	0.975	-	.	.
0.892	0.89	0.924	0.818	0.849	0.894	0.937	0.905	0.746	0.855	0.797	1.003	-	.	.
0.927	0.929	0.902	0.81	0.867	0.942	0.929	0.889	0.738	0.832	0.802	0.857	-	.	.
0.944	0.941	0.918	0.829	0.852	0.938	0.94	0.93	0.736	0.85	0.795	0.92	-	.	.
0.925	0.93	0.928	0.802	0.801	0.906	0.946	0.907	0.764	0.873	0.749	1.105	-	.	.
0.931	0.926	0.95	0.78	0.863	0.887	0.955	0.94	0.73	0.885	0.824	1.014	-	.	.
0.916	0.988	0.923	0.758	0.858	0.929	0.949	0.93	0.745	0.884	0.823	1.035	-	.	.
0.939	0.926	0.948	0.775	0.879	0.941	0.944	0.923	0.758	0.853	0.819	1.061	-	.	.
0.939	0.996	0.929	0.824	0.877	0.92	0.942	0.931	0.831	0.827	0.817	1.034	-	.	.
0.918	0.957	0.91	0.82	0.88	0.933	0.931	0.927	0.794	0.875	0.862	1.073	-	.	.
0.916	0.924	0.922	0.806	0.889	0.93	0.918	0.934	0.81	0.882	0.817	0.838	-	.	.
0.897	0.968	0.919	0.808	0.9	0.933	0.951	0.933	0.841	0.85	0.82	0.934	-	.	.
0.915	0.938	0.909	0.86	0.914	0.92	0.941	0.918	0.825	0.833	0.819	0.965	-	.	.
0.93	0.948	0.961	0.875	0.917	0.936	0.949	0.936	0.769	0.809	0.834	0.965	-	.	.
0.942	0.974	0.938	0.88	0.921	0.94	0.951	0.915	0.737	0.801	0.862	-	-	.	.
0.92	0.95	0.944	0.876	0.927	0.946	0.958	0.941	0.794	0.873	0.851	1.009	-	.	.
0.954	0.949	0.937	0.909	0.907	0.906	0.939	0.943	0.843	0.892	0.86	1.03	-	.	.
0.933	0.964	0.962	0.913	0.924	0.946	0.961	0.977	0.83	0.898	0.878	1.089	-	.	.
0.937	0.967	0.95	0.901	0.919	0.948	0.952	0.957	0.819	0.88	0.831	1.114	-	.	.
0.946	0.939	0.956	0.916	0.947	0.954	0.957	0.953	0.805	0.922	0.838	0.896	-	.	.
0.948	0.949	0.971	0.909	0.914	0.925	0.946	0.944	0.83	0.923	0.818	0.905	-	.	.
0.95	0.94	0.982	0.9	0.919	0.978	0.958	0.965	0.844	0.95	0.82	0.905	-	.	.
0.942	0.937	0.973	0.928	0.901	0.924	0.931	0.918	0.797	0.938	0.865	0.927	-	.	.
0.929	0.94	0.977	0.93	0.928	0.95	0.946	0.911	0.801	0.95	0.884	0.935	-	.	.
0.979	0.961	0.966	0.933	0.913	0.917	0.93	0.931	0.837	0.935	0.865	1.053	-	.	.
0.944	0.971	0.963	0.917	0.891	0.894	0.922	0.895	0.827	0.95	0.898	1.06	-	.	.
0.931	0.962	0.965	0.922	0.942	0.901	0.905	0.895	0.829	0.946	0.854	0.98	-	.	.
0.952	0.979	0.988	0.967	0.931	0.95	0.925	0.942	0.831	0.938	0.876	0.958	-	.	.
0.958	0.951	0.969	0.91	0.936	0.902	0.939	0.889	0.851	0.912	0.889	1.017	-	.	.
0.938	0.976	0.948	0.946	0.93	0.948	0.958	0.915	0.822	0.982	0.916	1.047	-	.	.
0.95	1	0.956	0.933	0.896	0.949	0.954	0.9	0.816	0.947	0.894	0.987	-	.	.
0.95	0.967	0.954	0.941	0.925	0.962	0.932	0.864	0.831	0.946	0.902	1.05	-	.	.
0.932	0.987	0.942	0.918	0.93	0.921	0.911	0.88	0.852	0.956	0.918	1.043	-	.	.
0.939	0.982	0.962	0.979	0.943	0.924	0.91	0.892	0.861	0.95	0.921	0.98	-	.	.
0.916	0.979	0.961	0.971	0.937	0.917	0.941	0.78	0.822	0.964	0.956	0.988	-	.	.
0.953	0.988	0.98	0.945	0.91	0.895	0.924	0.846	0.852	0.939	0.97	0.95	-	.	.
0.951	0.956	0.945	0.94	0.908	0.934	0.929	0.887	0.87	0.948	0.932	1.036	-	.	.
0.952	0.974	0.947	0.938	0.898	0.937	0.949	0.855	0.868	1.011	0.938	0.996	-	.	.
0.898	0.982	0.958	0.928	0.916	0.92	0.951	0.871	0.845	0.966	0.876	0.965	-	.	.

0.95	1.02	0.93	0.936	0.899	0.872	0.915	0.837	0.868	0.982	0.868	0.997	-	.	.
0.939	0.977	0.929	0.957	0.929	0.861	0.867	0.864	0.846	0.971	0.898	0.905	-	.	.
0.908	1.004	0.96	0.968	0.901	0.813	0.927	0.863	0.832	0.968	0.929	0.911	-	.	.
0.888	0.92	0.92	0.953	0.899	0.838	0.929	0.837	0.844	0.962	0.931	1	-	.	.
0.921	0.951	0.88	0.932	0.898	0.775	0.879	0.786	0.846	0.935	0.959	1.083	-	.	.
0.921	0.964	0.907	0.977	0.886	0.849	0.88	0.859	0.851	0.982	0.918	0.965	-	.	.
0.916	0.894	0.906	0.979	0.865	0.882	0.873	0.741	0.864	0.945	0.931	0.965	-	.	.
0.914	0.885	0.93	0.969	0.886	0.922	0.909	0.783	0.809	0.94	0.921	1.161	-	.	.
0.926	0.935	0.919	0.937	0.835	0.921	0.894	0.799	0.818	0.879	0.895	-	-	.	.
0.911	0.882	0.903	0.926	0.85	0.897	0.807	0.839	0.804	0.827	0.871	0.917	-	.	.
0.934	0.875	0.948	0.912	0.815	0.867	0.849	0.862	0.829	0.878	0.887	1.132	-	.	.
0.888	0.868	0.922	0.896	0.8	0.818	0.854	0.813	0.835	0.877	0.84	0.92	-	.	.
0.92	0.913	0.891	0.885	0.824	0.861	0.826	0.885	0.836	0.874	0.818	1.245	-	.	.
0.891	0.954	0.874	0.899	0.85	0.821	0.801	0.868	0.823	0.849	0.804	1.302	-	.	.
0.903	0.883	0.854	0.902	0.852	0.738	0.833	0.889	0.781	0.825	0.801	1.017	-	.	.
0.891	0.881	0.815	0.945	0.864	0.812	0.864	0.841	0.836	0.817	0.831	0.891	-	.	.
0.867	0.92	0.87	0.884	0.82			0.808	0.763	0.841	0.868	1.096	-	.	.
0.905	0.933	0.867	0.871	0.827			0.856	0.817	0.81	0.858	1.275	-	.	.
0.93	0.964	0.856	0.855	0.86			0.886	0.834	0.761	0.867	1.039	-	.	.
0.93	0.924	0.808	0.819				0.913	0.828	0.755	0.853	1.039	-	.	.
0.884	0.869	0.853	0.826				0.874	0.825	0.764	0.86	1.105	-	.	.
0.913	0.907	0.865	0.829				0.896	0.812	0.786	0.89	0.946	-	.	.
0.931	0.883	0.927	0.829				0.839	0.784	0.805	0.857	1.058	-	.	.
0.974	0.951	0.897	0.798				0.894	0.841	0.797	0.826	1.105	-	.	.
0.961	0.955	0.894	0.806				0.919	0.865	0.817	0.861	0.975	-	.	.
0.94	0.946	0.904	0.804				0.891	0.852	0.824	0.895	1.048	-	.	.
0.938	0.933	0.89	0.86				0.867	0.88	0.787	0.916	1.146	-	.	.
0.922	0.949	0.928	0.873				0.884	0.887	0.817	0.928	1.044	-	.	.
0.929	0.936	0.931	0.901				0.891	0.893	0.765	0.859	0.975	-	.	.
0.974	1.038	0.916	0.897				0.924	0.873	0.841	0.926	1.156	-	.	.
0.965	0.968	0.924	0.912				0.923	0.849	0.808	0.816	1.078	-	.	.
0.939	0.939	0.926	0.919				0.88	0.835	0.85	0.938	0.992	-	.	.
0.938	0.985	0.929	0.925				0.905	0.84	0.828	0.942	0.993	-	.	.
0.97	0.951	0.916	0.951				0.899	0.859	0.869	0.924	0.956	-	.	.
0.917	0.925	0.949	0.938				0.9	0.863	0.8	0.944	0.965	-	.	.
0.963	0.955	0.949	0.948				0.929	0.874	0.818	0.967	0.869	-	.	.
0.942	0.971	0.945	0.928				0.924	0.88	0.856	0.972	0.981	-	.	.
0.914	0.978	0.955	0.929				0.918	0.874	0.798	0.963	0.98	-	.	.
0.924	0.953	0.931	0.933				0.89	0.858	0.803	0.952	0.922	-	.	.
0.897	0.95	0.942	0.92				0.901	0.888	0.865	0.937	0.95	-	.	.
0.93	0.958	0.919	0.936				0.916	0.875	0.908	0.955	0.995	-	.	.
0.938	0.959	0.957	0.937				0.912	0.878	0.918	0.947	1.05	-	.	.
0.932	0.969	0.957	0.947				0.905	0.874	0.917	0.93	0.915	-	.	.
0.932	0.97	0.948	0.935				0.908	0.876	0.929	0.969	1.026	-	.	.
0.954	0.974	0.946	0.944				0.945	0.889	0.924	0.954	1.029	-	.	.
0.942	0.985	0.958	0.966				0.914	0.846	0.957	0.961	1.056	-	.	.
0.93	0.966	0.94	0.947				0.935	0.865	0.923	0.909	1.024	-	.	.
0.955	0.972	0.938	0.965				0.923	0.873	0.945	0.954	0.972	-	.	.
0.92	0.989	0.966	0.978				0.911	0.865	0.944	0.972	0.951	-	.	.
0.952	0.952	0.979	0.956				0.908	0.897	0.941	0.943	1.01	-	.	.
0.942	0.961	0.964	0.949				0.901	0.848	0.935	0.96	0.986	-	.	.
0.95	0.948	0.935	0.983				0.905	0.845	0.943	0.952	1.127	-	.	.
0.938	0.951	0.948	0.961				0.928	0.822	0.943	0.937	1.153	-	.	.
0.927	0.997	0.95	0.938				0.878	0.754	0.938	0.961	1.17	-	.	.
0.951	0.965	0.964	0.998				0.93	0.866	0.957	0.954	1	-	.	.
0.874	0.941	0.958	0.986				0.871	0.873	0.961	0.945	1.026	-	.	.
-	0.963	0.941	0.967				-	-	0.947	0.971	1.01	-	.	.
-	0.951	0.94	0.987				-	-	0.96	0.959	1.056	-	.	.
0.905	0.983	0.982	0.965				0.911	0.824	0.968	0.947	1.128	-	.	.
0.903	1.005	0.983	0.998				0.906	0.791	1.002	0.962	1.05	-	.	.
0.951	0.978	0.95	0.984				0.896	0.812	0.993	0.977	1.06	-	.	.
0.92	1.013	0.952	0.982				0.902	0.8	0.983	0.946	1.029	-	.	.
0.922	1.011	0.903	0.961				0.913	0.839	0.985	0.941	1.039	-	.	.
0.943	0.951	0.911	0.97				0.92	0.841	0.984	0.925	0.981	-	.	.
0.959	1.001	0.877	0.94				0.887	0.853	0.995	0.948	0.998	-	.	.
0.93	0.968	0.904	0.924				0.928	0.852	0.972	0.945	0.981	-	.	.
0.922	0.953	0.931	0.965				0.917	0.822	0.973	0.92	0.829	-	.	.
0.929	0.98	0.953	0.956				0.905	0.851	0.986	0.973	0.935	-	.	.
0.944	0.946	0.945	0.884				0.866	0.813	0.962	0.915	0.919	-	.	.
0.919	0.988	0.962	0.945				0.886	0.828	0.913	0.904	1.176	-	.	.
0.91	0.952	0.965	0.921				0.914	0.808	0.967	0.894	0.897	-	.	.
0.867	0.961	0.986	0.904				0.865	0.773	0.951	0.935	0.928	-	.	.
0.86	0.94	0.976	0.935				0.861	0.729	0.945	0.881	0.913	-	.	.
0.817	0.917	0.888	0.857				0.847	0.751	0.928	0.877	1.035	-	.	.
0.826	0.877	0.921	0.88				0.861	0.775	0.933	0.862	-	-	.	.
0.816	0.86	0.884	0.896				0.87	0.858	0.963	0.831	-	-	.	.
0.783	0.865	0.9	0.88				0.889	0.774	0.974	0.855	-	-	.	.
0.757	0.876	0.92	0.886				0.825	0.799	0.935	0.916	0.917	-	.	.
0.781	0.851	0.935	0.899				0.808	0.781	0.93	0.903	0.942	-	.	.
0.823	0.789	0.951	0.901				0.823	0.778	0.919	0.892	0.874	-	.	.
0.818	0.787	0.953	0.874				0.859	0.749	0.936	0.865	1.212	-	.	.
0.746	0.779	0.968	0.891				0.852	0.807	0.959	0.925	0.965	-	.	.
0.863	1.071	0.958	0.806	0.907	0.997	0.962	0.867	0.781	0.975	0.97	-	.	.	.
0.918	1.01	0.981	0.819	0.921	0.993	0.961	0.902	0.817	0.982	0.977	0.785	.	.	.
0.931	0.992	1.014	0.891	0.947	0.978	0.961	0.909	0.866	1.006	0.99	1.043	.	.	.
0.991	1.043	0.984	0.922	0.943	0.961	0.977	0.935	0.881	1.013	0.97	1.014	.	.	.
0.961	1.029	0.992	0.993	0.958	0.963	0.961	0.939	0.845	1.025	0.976	1.07	.	.	.
0.966	1.017	1.001	0.934	0.948	0.996	0.983	0.962	0.888	1.001	0.981	1.078	.	.	.
0.997	1.062	1.014	0.962	0.933	0.966	1.003	0.957	0.903	1.007	1.018	1.073	.	.	.
0.998	1.041	1.021	1.017	0.964	1.002	1.038	0.971	0.896	1	0.99	1.087	.	.	.
0.939	1.072	0.957	0.959	0.909	0.975	0.998	0.952	0.917	1.031	0.969	1.029	.	.	.
0.938	1.024	1.007	0.949	0.923	0.96	1.001	0.963	0.883	1.002	0.987	0.996	.	.	.
0.987	1.002	0.999	0.961	0.901	0.997	0.989	0.949	0.915	0.981	0.965	1.05	.	.	.
0.971	1.08	0.962	0.943	0.907	0.962	0.994	0.958	0.928	0.988	0.969	0.972	.	.	.
0.956	1.032	1.022	0.986	0.926	0.981	0.987	0.924	0.903	0.994	0.989	1.003	.	.	.
0.963	1.028	1.004	0.97	0.949	0.988	0.964	0.953	0.904	1.015	0.964	1.031	.	.	.
0.928	1.003	0.972	0.954	0.946	0.995	0.975	0.901	0.868	0.999	0.958	0.946	.	.	.
0.945	1.028	0.978	0.96	0.97	0.976	0.984	0.918	0.895	1.003	0.954	1.104	.	.	.
0.931	1.006	0.99	0.978	0.928	0.971	0.989	0.936	0.878	1.016	0.993	1.041	.	.	.
0.93	0.98	0.996	0.972	0.952	0.965	0.978	0.927	0.891	0.991	0.972	1.059	.	.	.
0.953	1.016	0.982	0.935	0.977	0.983	0.986	0.926	0.898	0.995	0.963	1.018	.	.	.
0.985	1.006	0.993	0.957	0.963	0.938	0.986	0.942	0.899	1.025	0.982	1.121	.	.	.
0.973	1.033	0.989	1.023	0.964	0.986	0.972	0.926	0.873	1.026	0.984	1.127	.	.	.
0.963	1.073	1.008	0.999	0.957	0.978	0.987	0.931	0.907	0.998	0.982	0.923	.	.	.

0.969	1.042	1.023	0.999	0.956	0.967	1.003	0.942	0.926	0.99	0.989	1.099	.	.	.
0.973	1.05	1.006	0.983	0.95	0.989	0.986	0.956	0.904	0.996	0.993	1.133	.	.	.
0.948	1.032	1.038	0.974	0.948	0.989	0.995	0.959	0.916	0.999	0.972	1.338	.	.	.
0.95	1.07	1.032	0.988	0.937	0.945	0.94	0.963	0.912	1.01	0.961	1.178	.	.	.
0.962	1.036	1.019	0.991	0.953	0.977	0.974	0.95	0.897	0.995	1	1.178	.	.	.
0.965	1.045	1.028	1	0.935	0.957	0.948	0.956	0.919	1.019	0.981	1.225	.	.	.
0.956	1.02	1.013	0.977	0.977	0.966	0.879	0.934	0.916	0.989	0.977	1.24	.	.	.
0.983	0.995	1.001	0.976	0.949	0.956	0.945	0.942	0.928	1.015	0.983	1.201	.	.	.
0.985	0.981	1.018	1.002	0.963	0.964	0.981	0.936	0.932	1.03	0.988	1.15	.	.	.
0.989	0.959	1.025	1	0.94	0.972	0.967	0.942	0.914	1.023	0.996	1.21	.	.	.
0.976	0.924	1.018	1.006	0.955	0.971	0.99	0.949	0.941	1.003	0.997	1.205	.	.	.
0.983	0.898	1.037	1.025	0.958	0.975	0.972	0.978	0.93	0.999	0.982	1.2	.	.	.
0.967	0.868	1.022	1.02	0.959	0.948	0.989	0.939	0.905	1.007	0.991	1.338	.	.	.
0.979	0.829	1.029	1.022	0.976	0.969	1.019	0.948	0.925	1.01	0.962	1.266	.	.	.
0.978	0.799	1.025	0.985	0.965	0.953	0.989	0.942	0.918	1.002	1.023	1.083	.	.	.
0.952	0.756	1.006	1.023	0.938	0.976	0.998	0.926	0.937	1.025	1	1.254	.	.	.
0.967	0.734	0.987	1.024	0.955	0.985	0.99	0.953	0.935	1.035	1.019	1.186	.	.	.
0.941	0.746	1.016	1.012	0.985	0.976	0.966	0.927	0.926	1.019	1.014	1.23	.	.	.
0.952	0.803	1.019	1.042	0.975	0.96	0.995	0.954	0.932	1.022	0.986	1.139	.	.	.
0.963	0.921	1.021	1.017	0.979	0.948	1.001	0.961	0.946	1.018	0.99	1.274	.	.	.
0.945	0.991	1.005	0.993	0.986	0.901	1.02	0.94	0.959	1.053	0.991	1.195	.	.	.
0.962	0.939	1.035	1.03	0.978	0.906	1.041	0.963	0.902	1.01	0.979	1.05	.	.	.
1.013	0.874	1.025	1.013	0.975	0.917	0.996	0.953	0.968	1.01	0.997	1.161	.	.	.
0.966	0.967	1.096	0.978	1.004	1.001	0.998	0.952	0.917	1.051	1.025	1.108	.	.	.
0.984	0.929	1.034	1.001	0.992	0.989	0.901	0.98	0.94	1.023	1.021	1.064	.	.	.
0.955	0.912	1.04	1.015	1.021	0.983	0.859	0.969	0.897	1.061	1.034	1.179	.	.	.
0.969	0.992	1.088	1.004	1.021	1.003	0.834	0.917	0.911	1.022	1.027	1.075	.	.	.
0.952	1.03	1.064	0.997	1.002	1.068	0.971	0.972	0.946	1.007	0.988	1.031	.	.	.
0.978	1.037	1.038	0.978	1.008	1.018	0.983	0.997	0.935	1.041	1.006	1.087	.	.	.
1.034	1.06	1.052	1.004	1.01	1.043	0.943	0.956	0.93	1.038	1.019	1.185	.	.	.
0.985	1.093	1.085	1.051	1.031	1.055	0.907	1.01	0.946	1.036	1.031	1.11	.	.	.
0.963	1.068	1.016	1.002	0.984	1.05	0.87	0.982	0.933	1.042	1.012	1.281	.	.	.
1.034	1.063	1.078	1.012	0.969	1.045	0.957	1.034	0.94	1.035	1.024	1.37	.	.	.
1.061	1.062	1.065	1.009	0.944	1.042	0.978	1.01	0.956	1.037	1.07	1.184	.	.	.
1.006	1.073	1.012	0.957	0.975	1.053	0.961	0.979	0.992	1.055	1.054	1.066	.	.	.
0.995	1.09	1.085	0.986	0.943	1.059	0.914	0.959	0.907	1.04	1.064	0.939	.	.	.
0.996	1.087	1.08	1.047	1.001	1.085	0.957	0.953	0.926	1.002	1.041	1.085	.	.	.
0.979	1.127	1.061	1.046	0.922	1.013	1.006	0.975	0.935	0.981	1.034	1.07	.	.	.
0.982	1.118	1.047	0.998	0.899	1.029	1.031	1	0.958	0.953	1.06	1.034	.	.	.
1.007	1.062	1.084	1.012	0.89	0.977	0.995	0.95	0.921	1.017	1.074	0.973	.	.	.
1.01	1.16	1.029	0.982	0.861	1.051	1.037	0.968	0.939	1.056	1.053	1.01	.	.	.
1.006	1.107	0.997	0.97	0.844	1.019	1.039	0.955	0.95	1.013	1.03	0.949	.	.	.
1.018	1.155	1.047	0.96	0.826	0.964	1.01	0.904	0.942	1.005	1.022	0.905	.	.	.
0.99	1.115	1.027	0.979	0.811	0.998	1.024	0.896	0.948	0.988	1.029	1.107	.	.	.
0.988	1.081	0.999	0.892	0.852	1.014	1.041	0.942	0.947	0.973	0.982	1.042	.	.	.
0.984	1.041	1.038	0.914	0.877	0.996	1.058	0.902	0.969	0.975	1.021	1.031	.	.	.
0.95	1.03	1.076	0.954	0.922	0.971	1.034	0.942	0.95	0.951	1.069	1.064	.	.	.
0.957	1.091	1.066	0.98	0.865	0.971	0.985	0.957	0.921	0.995	1.064	1.185	.	.	.
1.044	1.081	1.046	1.021	0.874	0.972	1.03	0.955	0.88	0.991	1.092	0.989	.	.	.
0.976	1.019	1.09	1.045	0.861	0.953	0.994	0.92	0.895	0.966	1.065	1.043	.	.	.
1.007	1.078	1.092	1.01	0.886	1.028	1.03	0.859	0.919	0.995	1.087	1.101	.	.	.
0.99	1.077	1.073	0.926	0.894	0.991	0.99	0.835	0.963	1.077	1.092	1.01	.	.	.
1.009	1.071	1.06	0.855	0.965	1.06	1.046	0.829	0.944	0.983	1.05	1.084	.	.	.
1.045	1.073	1.055	0.864	0.965	1.04	1.032	0.921	0.952	1.021	1.088	1.109	.	.	.
1.017	1.136	1.055	0.932	0.947	1.037	1.067	0.945	0.919	1.049	1.046	1.134	.	.	.
1.057	1.074	1.031	0.95	0.987	1.008	1.033	0.967	0.98	1.039	1.062	1.059	.	.	.
1.03	1.054	1.013	0.954	0.971	0.97	1.014	0.93	0.974	1.039	1.06	1.056	.	.	.
1.005	1.053	1.005	0.887	0.929	0.992	1.046	0.952	0.989	1.007	1.044	1.173	.	.	.
1.003	1.009	1.032	0.964	0.975	1.027	1.026	0.956	0.994	1.017	1.032	1.022	.	.	.
0.987	1.033	1.046	1.016	0.973	1.025	1.03	0.978	0.965	1.066	1.005	1.229	.	.	.
0.966	1.071	1.072	1.038	1.031	1.02	0.989	0.941	0.984	1.031	1.065	1.061	.	.	.
0.957	1.126	1.08	1.03	1.044	1	0.937	0.927	1.011	1.015	1.044	1.156	.	.	.
0.933	1.073	1.069	1.031	1.05	0.997	0.951	0.899	0.969	1.032	1.027	1.069	.	.	.
0.982	1.126	1.06	1.05	1.049	0.991	0.909	0.912	0.986	1.035	1.089	1.092	.	.	.
0.979	1.05	1.043	1.064	1.022	1.001	0.919	0.941	0.975	1.08	1.094	1.045	.	.	.
1.001	1.084	1.027	1.043	1.015	0.981	0.96	0.994	0.988	1.043	1.064	1.121	.	.	.
1.045	1.116	1.055	1.042	0.98	1.044	1.002	0.983	0.989	1.022	1.092	1.045	.	.	.
1.021	1.092	1.096	1.054	0.971	0.983	0.964	0.956	0.995	1	1.041	1.126	.	.	.
1.027	1.064	1.03	1.013	0.992	0.985	0.975	0.985	0.965	1.041	1.056	0.971	.	.	.
1.027	1.016	1.041	0.979	0.962	0.973	0.99	0.999	0.979	1.072	1.048	1.085	.	.	.
1.027	1.015	1.01	0.939	0.972	1.008	1.03	0.969	0.96	1.027	1.063	1.089	.	.	.
1.033	1	1.001	0.924	0.995	0.898	1.01	0.98	0.985	1.039	1.068	1.05	.	.	.
1.051	0.915	1.002	0.957	0.984	0.941	0.981	1.003	0.974	1.038	1.062	1.121	.	.	.
1.079	0.902	0.952	0.98	0.955	0.996	0.975	0.957	1.009	1.049	1.048	1.215	.	.	.
1.034	0.882	.	0.971	0.863	0.99	0.964	0.944	0.963	1.041	1.079	1.153	.	.	.
1.019	0.84	.	0.959	0.929	0.924	0.964	0.924	0.92	1.051	1.037	1.199	.	.	.
1.074	0.75	.	1.006	0.947	0.863	0.913	0.951	0.946	1.069	1.036	1.103	.	.	.
1.012	0.745	.	0.97	0.941	0.826	0.818	0.962	0.977	1.071	1.061	1.288	.	.	.
1.032	0.833	.	0.987	0.873	0.855	0.708	0.961	0.911	1.061	1.071	1.134	.	.	.
0.999	0.798	.	0.985	0.894	0.916	0.853	0.944	0.926	1.042	1.076	1.033	.	.	.
1.039	0.953	.	0.941	0.913	0.872	0.906	0.988	0.95	1.023	1.078	0.972	.	.	.
1.009	0.928	.	0.98	0.897	0.792	0.98	0.972	0.943	1.026	1.064	1.214	.	.	.
0.999	0.878	.	0.998	0.792	0.705	0.904	1.015	0.939	1.002	1.048	1.076	.	.	.
1.062	0.841	.	1.04	0.807	0.914	0.932	1.002	0.924	1.003	1.018	0.928	.	.	.
1.068	0.78	.	0.976	0.746	0.989	0.931	0.992	0.974	0.942	1.02	0.979	.	.	.
1.013	0.777	.	0.958	0.782	1	0.894	1.002	0.976	0.883	1.013	1.031	.	.	.
1.013	0.776	.	0.906	0.928	1.058	0.908	0.978	0.931	0.878	0.952	0.965	.	.	.
1.023	0.791	.	0.877	0.969	1.06	0.878	0.988	0.968	0.874	0.971	1.115	.	.	.
1.003	0.735	.	0.959	0.921	1.056	0.862	1.012	0.934	0.978	1.028	1.073	.	.	.
0.998	0.711	.	0.958	0.912	1.049	0.898	1.014	0.974	0.992	0.977	1.043	.	.	.
0.963	0.891	.	1.002	0.794	1.058	0.845	0.97	0.928	1.028	1.007	1.084	.	.	.
0.953	1.012	.	0.919	0.753	1.032	0.744	0.956	0.913	1.01	1.025	1.064	.	.	.
0.911	0.874	.	0.877	0.826	1.03	0.741	0.949	0.905	1.011	0.974	0.988	.	.	.
0.897	0.84	.	0.877	0.958	1.023	0.801	0.954	0.891	1.004	1.009	1.012	.	.	.
0.847	0.813	.	0.798	0.949	0.995	0.958	.	0.97	0.97	0.96	1.016	.	.	.
0.783	0.796	.	0.858	0.997	0.988	0.951	.	0.955	0.931	0.934	0.865	.	.	.
0.792	0.803	.	0.985	0.928	1.045	0.99	.	0.948	0.917	0.887	0.997	.	.	.
0.776	0.739	.	1.014	0.965	1.088	1.071	.	0.986	0.934	0.832	0.975	.	.	.
0.794	0.789	.	1.028	0.991	1.021	0.962	.	0.973	1.001	0.903	1.029	.	.	.
0.834	0.774	.	1.05	1.051	0.931	1.006	.	0.978	0.969	0.829	1.161	.	.	.
0.767	0.754	.	1.026	1.02	0.936	0.995	.	0.964	0.893	0.872	0.957	.	.	.
0.743	0.742	.	1.016	1.042	0.869	0.961	.	0.954	0.869	0.817	1.099	.	.	.
0.744	0.7	.	1.037	1.044	0.871	0.999	.	0.937	0.866	0.812	-	.	.	.
0.88	0.72	.	1.037	1.005			.	0.939	0.935	0.808	-	.	.	.
0.908	0.73	.	0.979	1.015			.	0.936	1.018	0.918	-	.	.	.

0.921	0.734 .	0.975	0.97	.		1.006	1.023	0.997	1.121 .	.	.
1.04	0.719 .	0.933	0.935	.		0.979	0.974	1.034	1.22 .	.	.
0.961	0.729 .	0.919	0.942	.		0.984	1.008	1.032	1.237 .	.	.
0.951	0.685 .	0.891		.		0.967	0.969	1.011	1.139 .	.	.
0.885	0.781 .	0.862		.		0.991	1.003	0.981	1.176 .	.	.
0.81	0.798 .	0.836		.		0.938	0.957	0.933	1.026 .	.	.
0.868	0.782 .	0.808		.		0.921	0.958	1	1.037 .	.	.
0.819	0.722 .	0.83		.		0.904	0.903	1.067	1.182 .	.	.
0.964	0.704 .	0.893		.		0.875	0.885	1.05	1.008 .	.	.
1.041	0.841 .	0.981		.		0.905	0.947	1.02 -	.	.	.
1.045	0.971 .	0.917		.		0.88	1.035	0.991 -	.	.	.
0.981	1.058 .	0.932		.		0.806	1.038	1.067	1.111 .	.	.
0.925	1.015 .	0.919		.		0.877	1.027	1.078	1.411 .	.	.
0.937	1.087 .	0.922		.		0.902	1.037	1.057	1.308 .	.	.
0.977	1.061 .	0.975			0.938	0.879	0.996	1.051	1.282 .	.	.
0.967	0.985 .	1.05			0.976	0.92	1	1.02	1.272 .	.	.
0.958	1.009 .	1.069			1.026	0.92	0.96	1.005	1.208 .	.	.
0.943	0.963 .	1.031			1.002	0.921	0.902	0.966	1.209 .	.	.
0.953	0.954 .	1.031			0.959	0.92	0.865	0.999	1.297 .	.	.
0.971	0.998 .	1.04			0.968	0.951	0.838	1.051	1.168 .	.	.
0.967	0.996 .	1.046			0.941	0.924	0.808	1.025	1.121 .	.	.
0.929	0.992 .	1.037			0.934	0.918	0.943	1.039	1.085 .	.	.
0.956	1.03 .	1.038			0.919	0.951	1.013	0.993	1.022 .	.	.
1.007	1.008 .	1.034			0.89	0.958	1.019	0.974	1.085 .	.	.
1.015	1.017 .	1.036			0.874	0.912	0.978	1.022	1.143 .	.	.
0.981	1.056 .	1.046			0.902	0.957	0.977	1.022	1.022 .	.	.
0.996	1.05 .	1.037			0.878	0.954	0.924	1.047	1.024 .	.	.
0.986	1.017 .	1.068			0.8	0.946	0.976	1.05	1.05 .	.	.
1.014	1.07 .	1.053			0.781	0.936	0.937	1.026	0.965 .	.	.
0.961	1.084 .	1.033			0.722	0.957	0.914	1.019	0.941 .	.	.
0.972	1.039 .	1.072			0.765	0.969	0.825	0.984	1.02 .	.	.
0.967	1.036 .	1.067			0.851	0.937	0.807	1.008	1.05 .	.	.
0.955	1.112 .	1.065			0.874	0.946	0.804	1.019	0.993 .	.	.
0.917	1.026 .	1.037			0.837	0.925	0.792	1.065	1.031 .	.	.
0.889	1.012 .	1.04			0.789	0.938	0.776	1.027	1.033 .	.	.
0.886	1.006 .	1.045			0.763	0.874	0.76	0.976	0.958 .	.	.
0.825	1.035 .	1.038			0.763	0.832	0.76	0.975	0.945 .	.	.
0.789	0.997 .	0.99			0.735	0.901	0.776	0.918	1.02 .	.	.
0.85	0.915 .	0.91			0.764	0.939	0.758	0.968	0.953 .	.	.
0.854	0.979 .	0.851			0.821	0.995	0.747	0.953	0.917 .	.	.
0.816	0.981 .	0.883			0.892	0.959	0.777	0.888	0.992 .	.	.
0.814	0.923 .	0.903			0.909	0.983	0.74	0.89	0.952 .	.	.
0.882	0.936 .	0.948			0.913	0.949	0.746	0.86	0.833 .	.	.
0.865	0.911 .	0.871			0.897	0.94	0.791	0.951	0.972 .	.	.
0.822	0.828 .	0.83			0.857	0.941	0.895	0.99	1.015 .	.	.
0.785	0.773 .	0.779			0.894	0.974	0.9	0.941	0.999 .	.	.
0.771	0.845 .	0.762			0.917	0.969	0.826	0.876	0.972 .	.	.
0.79	0.785 .	0.786			0.937	0.966	0.777	0.995	0.844 .	.	.
0.814	0.787 .	0.773			0.883	0.955	0.753	0.986	0.938 .	.	.
0.703	0.858 .	0.755			0.843	0.954	0.774	0.954	0.958 .	.	.
0.836	0.754 .	0.739			0.795	0.946	0.8	0.902	0.987 .	.	.
0.944	0.742 .	0.803			0.863	0.921	0.781	0.898	0.843 .	.	.
0.937	0.754 .	0.927			0.92	0.923	0.768	0.826	1.039 .	.	.
0.934	0.751 .	0.914			0.932	0.886	0.773	0.87	0.997 .	.	.
0.982	0.779 .	0.898			0.917	0.854	0.855	0.995	0.869 .	.	.
1.021	0.762 .	0.792			0.9	0.88	0.824	1.03	0.989 .	.	.
0.953	0.738 .	0.799			0.857	0.823	0.776	0.983	1.129 .	.	.
0.893	0.714 .	0.779			0.868	0.75	0.763	0.97	0.973 .	.	.
0.858	0.739 .	0.775			0.791	0.715	0.811	1.001	0.998 .	.	.
0.915	0.746 .	0.792			0.763	0.691	0.805	1.095	1.135 .	.	.
0.898	0.761 .	0.743			0.866	0.763	0.767	1.058	0.99 .	.	.
0.903	0.809 .	0.795			0.921	0.729	0.75	0.997	1.136 .	.	.
0.928	0.728 .	0.73			0.959	0.705	0.749	0.983	1.398 .	.	.

0.889	0.846	0.891	0.806 .	0.754	0.898	0.882	0.821	0.857	0.94	0.762 .	.	.
0.893	0.827	0.91	0.807 .	0.816	0.899	0.915	0.832	0.891	0.902	0.991 .	.	.
0.865	0.823	0.904	0.839 .	0.76	0.867	0.844	0.764	0.878	0.906	0.869 .	.	.
0.866	0.807	0.873	0.817 .	0.762	0.839	0.843	0.669	0.887	0.902	0.807 .	.	.
0.805	0.837	0.858	0.878 .	0.816	0.85	0.84	0.688	0.863	0.923	0.874 .	.	.
0.869	0.839	0.859	0.833 .	0.847	0.872	0.855	0.784	0.857	0.863	0.891 .	.	.
0.846	0.787	0.853	0.823 .	0.867	0.818	0.83	0.811	0.847	0.881	0.758 .	.	.
0.851	0.828	0.889	0.89 .	0.836	0.83	0.839	0.791	0.846	0.888	0.888 .	.	.
0.815	0.802	0.842	0.889 .	0.841	0.818	0.848	0.746	0.836	0.86	0.843 .	.	.
0.833	0.844	0.837	0.883 .	0.836	0.815	0.802	0.761	0.868	0.835	0.843 .	.	.
0.872	0.821	0.886	0.869 .	0.863	0.825	0.822	0.792	0.839	0.813	0.801 .	.	.
0.821	0.837	0.871	0.872 .	0.8	0.793	0.789	0.803	0.832	0.828	0.902 .	.	.
0.806	0.848	0.869	0.869 .	0.808	0.776	0.787	0.773	0.849	0.851	0.843 .	.	.
0.789	0.847	0.848	0.835 .	0.812	0.796	0.778	0.758	0.81	0.822	0.772 .	.	.
0.78	0.844	0.825	0.818 .	0.814	0.781	0.78	0.772	0.807	0.84	0.756 .	.	.
0.79	0.828	0.836	0.852 .	0.788	0.79	0.75	0.786	0.82	0.849	0.806 .	.	.
0.796	0.833	0.808	0.821 .	0.767	0.836	0.751	0.748	0.818	0.818	0.814 .	.	.
0.768	0.841	0.831	0.809 .	0.747	0.783	0.754	0.723	0.786	0.81	0.832 .	.	.
0.781	0.848	0.82	0.83 .	0.747	0.798	0.762	0.711	0.829	0.847	0.871 .	.	.
0.781	0.822	0.794	0.826 .	0.755	0.798	0.748	0.701	0.809	0.807	0.809 .	.	.
0.775	0.822	0.811	0.822 .	0.753	0.777	0.751	0.699	0.819	0.832	0.835 .	.	.
0.78	0.823	0.84	0.826 .	0.76	0.808	0.745	0.714	0.817	0.806	0.922 .	.	.
0.769	0.788	0.812	0.855 .	0.755	0.769	0.759	0.741	0.82	0.81	0.782 .	.	.
0.776	0.786	0.828	0.815 .	0.772	0.765	0.724	0.712	0.795	0.824	0.851 .	.	.
0.782	0.811	0.807	0.815 .	0.748	0.755	0.747	0.683	0.814	0.808	0.774 .	.	.
0.803	0.824	0.781	0.8 .	0.748	0.781	0.773	0.703	0.818	0.803	0.867 .	.	.
0.748	0.847	0.783	0.805 .	0.749	0.766	0.759	0.696	0.818	0.794	0.79 .	.	.
0.813	0.825	0.785	0.81 .	0.746	0.771	0.768	0.671	0.793	0.829	0.836 .	.	.
0.803	0.841	0.786	0.817 .	0.726	0.759	0.769	0.708	0.824	0.843	0.761 .	.	.
0.798	0.807	0.823	0.794 .	0.775	0.801	0.753	0.675	0.796	0.822	0.778 .	.	.
0.782	0.788	0.799	0.788 .	0.727	0.772	0.766	0.669	0.818	0.83	0.741 .	.	.
0.768	0.797	0.799	0.796 .	0.733	0.794	0.751	0.681	0.824	0.829	0.867 .	.	.
0.745	0.794	0.785	0.793 .	0.725	0.791	0.746	0.656	0.814	0.809	0.756 .	.	.
0.78	0.811	0.799	0.802 .	0.758	0.744	0.751	0.698	0.832	0.858	0.793 .	.	.
0.767	0.793	0.784	0.791 .	0.778	0.785	0.772	0.691	0.82	0.841	0.801 .	.	.
0.791	0.789	0.783	0.801 .	0.747	0.743	0.767	0.704	0.835	0.827	0.835 .	.	.
0.767	0.786	0.77	0.798 .	0.733	0.756	0.753	0.686	0.824	0.831	0.717 .	.	.
0.803	0.821	0.795	0.807 .	0.741	0.763	0.759	0.712	0.813	0.817	0.737 .	.	.
0.795	0.801	0.784	0.812 .	0.727	0.757	0.764	0.717	0.825	0.824	0.818 .	.	.
0.796	0.836	0.804	0.805 .	0.764	0.727	0.749	0.701	0.814	0.835	0.781 .	.	.
0.81	0.818	0.831	0.811 .	0.766	0.714	0.779	0.735	0.811	0.818	0.746 .	.	.

0.796	0.803	0.801	0.809	.	0.719	0.763	0.801	0.713	0.843	0.795	0.774	.	.	.
0.787	0.801	0.801	0.808	.	0.734	0.794	0.778	0.719	0.815	0.788	0.774	.	.	.
0.782	0.807	0.793	0.804	.	0.712	0.768	0.756	0.738	0.807	0.785	0.807	.	.	.
0.762	0.791	0.81	0.822	.	0.724	0.821	0.775	0.692	0.831	0.778	0.755	.	.	.
0.791	0.777	0.801	0.834	.	0.684	0.827	0.753	0.715	0.822	0.822	0.736	.	.	.
0.809	0.828	0.777	0.821	.	0.699	0.817	0.78	0.703	0.849	0.854	0.798	.	.	.
0.792	0.809	0.756	0.824	.	0.799	0.803	0.78	0.727	0.846	0.84	0.885	.	.	.
0.797	0.85	0.754	0.844	.	0.778	0.86	0.748	0.753	0.841	0.832	0.948	.	.	.
0.82	0.804	0.753	0.84	.	0.749	0.857	0.757	0.749	0.85	0.823	0.915	.	.	.
0.829	0.811	0.814	0.846	.	0.74	0.865	0.799	0.81	0.868	0.806	0.993	.	.	.
0.796	0.842	0.762	0.857	.	0.751	0.835	0.797	0.754	0.874	0.836	0.913	.	.	.
0.799	0.842	0.796	0.877	.	0.734	0.802	0.839	0.784	0.86	0.835	1.003	.	.	.
0.763	0.824	0.826	0.844	.	0.754	0.832	0.757	0.727	0.874	0.842	0.891	.	.	.
0.847	0.825	0.809	0.845	.	0.781	0.8	0.829	0.762	0.869	0.842	0.862	.	.	.
0.8	0.792	0.78	0.833	.	0.768	0.794	0.762	0.747	0.839	0.82	0.974	.	.	.
0.834	0.816	0.836	0.845	.	0.767	0.825	0.794	0.726	0.879	0.825	0.947	.	.	.
0.798	0.811	0.795	0.856	.	0.801	0.853	0.799	0.705	0.895	0.87	0.767	.	.	.
0.822	0.838	0.806	0.888	.	0.787	0.847	0.799	0.756	0.9	0.86	0.872	.	.	.
0.797	0.826	0.884	0.881	.	0.812	0.85	0.816	0.741	0.908	0.879	1.003	.	.	.
0.827	0.8	0.892	0.824	.	0.765	0.813	0.849	0.741	0.909	0.837	0.922	.	.	.
0.772	0.819	0.849	0.783	.	0.778	0.807	0.785	0.719	0.848	0.853	1.041	.	.	.
0.794	0.804	0.828	0.795	.	0.744	0.829	0.797	0.73	0.874	0.852	0.965	.	.	.
0.83	0.849	0.861	0.837	.	0.792	0.827	0.82	0.739	0.875	0.835	0.993	.	.	.
0.789	0.8	0.836	0.83	.	0.812	0.868	0.825	0.737	0.87	0.882	0.918	.	.	.
0.851	0.809	0.82	0.87	.	0.793	0.834	0.794	0.688	0.859	0.856	1.014	.	.	.
0.813	0.82	0.826	0.882	.	0.798	0.812	0.801	0.72	0.877	0.874 -		.	.	.
0.813	0.815	0.832	0.898	.	0.813	0.841	0.81	0.727	0.869	0.885	0.914	.	.	.
0.855	0.83	0.832	0.872	.	0.775	0.834	0.843	0.674	0.88	0.884	0.932	.	.	.
0.8	0.863	0.817	0.855	.	0.794	0.823	0.831	0.72	0.873	0.827	0.922	.	.	.
0.782	0.809	0.828	0.829	.	0.819	0.869	0.817	0.749	0.898	0.849	0.831	.	.	.
0.801	0.764	0.835	0.84	.	0.814	0.821	0.817	0.724	0.84	0.825	1.005	.	.	.
0.824	0.791	0.83	0.864	.	0.861	0.854	0.83	0.731	0.856	0.861	0.824	.	.	.
0.827	0.815	0.802	0.832	.	0.817	0.797	0.822	0.691	0.868	0.885	0.956	.	.	.
0.862	0.813	0.808	0.826	.	0.842	0.816	0.839	0.713	0.882	0.849	0.993	.	.	.
0.82	0.82	0.779	0.817	.	0.838	0.82	0.83	0.708	0.854	0.842	0.915	.	.	.
0.825	0.77	0.833	0.844	.	0.789	0.807	0.84	0.751	0.879	0.855	1.026	.	.	.
0.802	0.81	0.836	0.823	.	0.804	0.801	0.847	0.725	0.845	0.833	0.864	.	.	.
0.856	0.78	0.843	0.798	.	0.773	0.801	0.835	0.809	0.862	0.878	0.803	.	.	.
0.795	0.773	0.805	0.783	.	0.761	0.783	0.836	0.798	0.842	0.833	0.82	.	.	.
0.764	0.748	0.796	0.791	.	0.737	0.747	0.801	0.792	0.838	0.81	0.814	.	.	.
0.794	0.783	0.763	0.811	.	0.718	0.749	0.751	0.785	0.856	0.839	0.823	.	.	.
0.789	0.773	0.751	0.801	.	0.719	0.783	0.758	0.776	0.854	0.853	0.826	.	.	.
0.765	0.774	0.756	0.814	.	0.76	0.788	0.772	0.784	0.823	0.857	0.847	.	.	.
0.744	0.768	0.781	0.816	.	0.785	0.77	0.765	0.781	0.815	0.84	0.84	.	.	.
0.746	0.795	0.788	0.805	.	0.782	0.779	0.77	0.792	0.817	0.817	0.783	.	.	.
0.787	0.805	0.763	0.781	.	0.771	0.748	0.775	0.778	0.84	0.818	0.874	.	.	.
0.778	0.787	0.754	0.8	.	0.789	0.764	0.788	0.793	0.858	0.828	0.821	.	.	.
0.764	0.781	0.745	0.82	.	0.743	0.781	0.771	0.782	0.834	0.85	0.689	.	.	.
0.745	0.769	0.724	0.801	.	0.767	0.741	0.804	0.762	0.829	0.823	0.823	.	.	.
0.781	0.747	0.787	0.828	.	0.752	0.751	0.806	0.778	0.805	0.815	0.796	.	.	.
0.793	0.78	0.775	0.81	.	0.75	0.765	0.81	0.769	0.819	0.835	0.753	.	.	.
0.766	0.746	0.744	0.819	.	0.74	0.777	0.808	0.778	0.81	0.824	0.806	.	.	.
0.758	0.709	0.744	0.817	.	0.751	0.754	0.809	0.773	0.834	0.833	0.84	.	.	.
0.779	0.747	0.737	0.822	.	0.759	0.724	0.792	0.788	0.832	0.798	0.812	.	.	.
0.754	0.774	0.723	0.816	.	0.754	0.783	0.774	0.767	0.833	0.803	0.79	.	.	.
0.762	0.791	0.777	0.814	.	0.723	0.797	0.778	0.799	0.811	0.818	0.715	.	.	.
0.766	0.777	0.729	0.804	.	0.74	0.798	0.785	0.777	0.813	0.804	0.722	.	.	.
0.77	0.757	0.749	0.81	.	0.76	0.791	0.777	0.778	0.827	0.797	0.875	.	.	.
0.761	0.764	0.775	0.826	.	0.752	0.769	0.741	0.778	0.805	0.787	0.839	.	.	.
0.821	0.758	0.792	0.807	.	0.781	0.769	0.815	0.802	0.805	0.774	0.835	.	.	.
0.784	0.704	0.755	0.826	.	0.76	0.744	0.785	0.811	0.814	0.805	0.915	.	.	.
0.777	0.744	0.759	0.866	.	0.758	0.733	0.823	0.831	0.807	0.815	0.804	.	.	.
0.756	0.781	0.807	0.846	.	0.743	0.735	0.809	0.797	0.811	0.81	0.899	.	.	.
0.781	0.737	0.76	0.842	.	0.696	0.709	0.822	0.792	0.8	0.796	0.831	.	.	.
0.79	0.746	0.744	0.82	.	0.716	0.722	0.831	0.83	0.805	0.819	0.806	.	.	.
0.792	0.726	0.8	0.829	.	0.723	0.751	0.8	0.817	0.802	0.824	0.812	.	.	.
0.792	0.761	0.756	0.838	.	0.74	0.782	0.763	0.828	0.828	0.805	0.641	.	.	.
0.766	0.757	0.752	0.832	.	0.721	0.822	0.773	0.8	0.838	0.824	0.856	.	.	.
0.754	0.732	0.746	0.833	.	0.732	0.788	0.791	0.78	0.846	0.8	0.835	.	.	.
0.797	0.752	0.758	0.839	.	0.765	0.75	0.801	0.796	0.811	0.828	0.804	.	.	.
0.809	0.685	0.808	0.849	.	0.778	0.714	0.785	0.792	0.835	0.832	0.822	.	.	.
0.798	0.697	0.811	0.829	.	0.752	0.786	0.796	0.789	0.777	0.82	0.847	.	.	.
0.85	0.741	0.751	0.834	.	0.817	0.88	0.776	0.786	0.804	0.79	0.982	.	.	.
0.809	0.685	0.723	0.809	.	0.812	0.817	0.736	0.795	0.775	0.829	0.974	.	.	.
0.827	0.733	0.807	0.852	.	0.799	0.839	0.751	0.81	0.826	0.847	0.965	.	.	.
0.805	0.865	0.814	0.839	.	0.865	0.886	0.79	0.761	0.87	0.821	0.776	.	.	.
0.781	0.822	0.792	0.838	.	0.839	0.87	0.817	0.785	0.851	0.853	1.023	.	.	.
0.818	0.856	0.744	0.834	.	0.842	0.875	0.852	0.811	0.861	0.86 -		.	.	.
0.809	0.825	0.771	0.829	.	0.844	0.9	0.827	0.794	0.855	0.85	0.965	.	.	.
0.822	0.834	0.81	0.828	.	0.782	0.87	0.81	0.814	0.862	0.842	1.018	.	.	.
0.817	0.821	0.803	0.805	.	0.821	0.886	0.805	0.83	0.821	0.823	0.891	.	.	.
0.825	0.796	0.748	0.798	.	0.794	0.894	0.781	0.816	0.831	0.862	0.825	.	.	.
0.794	0.803	0.783	0.833	.	0.814	0.877	0.779	0.805	0.853	0.869	0.869	.	.	.
0.836	0.775	0.802	0.81	.	0.848	0.833	0.818	0.808	0.813	0.83	0.975	.	.	.
0.824	0.759	0.771	0.805	.	0.791	0.786	0.805	0.8	0.829	0.842	0.955	.	.	.
0.828	0.778	0.814	0.865	.	0.79	0.785	0.813	0.813	0.827	0.835	1.035	.	.	.
0.83	0.76	0.812	0.819	.	0.76	0.775	0.797	0.789	0.862	0.872	0.994	.	.	.
0.83	0.818	0.792	0.798	.	0.772	0.819	0.809	0.819	0.831	0.834	0.904	.	.	.
0.856	0.802	0.804	0.826	.	0.777	0.832	0.807	0.801	0.875	0.842	0.838	.	.	.
0.826	0.821	0.804	0.866	.	0.805	0.883	0.811	0.82	0.825	0.841	1.035	.	.	.
0.801	0.807	0.838	0.836	.	0.78	0.808	0.747	0.761	0.839	0.852	0.911	.	.	.
0.826	0.842	0.781	0.844	.	0.817	0.842	0.765	0.817	0.831	0.846	1.012	.	.	.
0.85	0.793	0.797	0.791	.	0.764	0.848	0.8	0.794	0.876	0.864	1.053	.	.	.
0.823	0.809	0.794	0.782	.	0.793	0.853	0.796	0.793	0.835	0.845	0.781	.	.	.
0.825	0.821	0.79	0.803	.	0.829	0.847	0.822	0.805	0.822	0.846	0.731	.	.	.
0.844	0.818	0.754	0.84	.	0.809	0.794	0.85	0.835	0.854	0.863	0.887	.	.	.
0.797	0.822	0.814	0.857	.	0.784	0.828	0.808	0.824	0.835	0.827	1.005	.	.	.
0.876	0.787	0.811	0.812	.	0.757	0.808	0.812	0.848	0.831	0.872	1.035	.	.	.
0.824	0.815	0.775	0.836	.	0.795	0.802	0.786	0.807	0.826	0.833	0.884	.	.	.
0.815	0.821	0.783	0.832	.	0.768	0.821	0.804	0.819	0.82	0.855	0.887	.	.	.
0.857	0.796	0.812	0.837	.	0.785	0.802	0.825	0.843	0.857	0.868	1.041	.	.	.
0.818	0.791	0.808	0.849	.	0.805	0.788	0.815	0.821	0.868	0.846	1.018	.	.	.
0.821	0.777	0.844	0.839	.	0.776	0.763	0.837	0.808	0.82	0.812	0.911	.	.	.
0.836	0.763	0.809	0.817	.	0.773	0.8	0.854	0.848	0.801	0.821	0.916	.	.	.
0.811	0.769	0.802	0.82	.	0.755	0.757	0.803	0.788	0.797	0.835	0.803	.	.	.

0.803	0.751	0.805	0.834	.	0.775	0.738	0.8	0.791	0.82	0.857	0.877	.	.	.
0.775	0.771	0.802	0.812	.	0.794	0.746	0.79	0.794	0.802	0.82	0.826	.	.	.
0.794	0.784	0.8	0.821	.	0.767	0.786	0.8	0.796	0.806	0.811	0.798	.	.	.
0.813	0.768	0.797	0.814	.	0.732	0.78	0.827	0.824	0.8	0.789	0.793	.	.	.
0.815	0.811	0.817	0.805	.	0.716	0.806	0.819	0.81	0.827	0.807	0.836	.	.	.
0.797	0.772	0.771	0.807	.	0.734	0.782	0.797	0.817	0.816	0.803	0.764	.	.	.
0.81	0.765	0.809	0.83	.	0.751	0.771	0.779	0.802	0.831	0.797	0.793	.	.	.
0.813	0.76	0.785	0.796	.	0.789	0.776	0.773	0.803	0.812	0.825	0.722	.	.	.
0.772	0.761	0.77	0.833	.	0.803	0.767	0.81	0.791	0.831	0.828	0.851	.	.	.
0.835	0.777	0.783	0.82	.	0.797	0.777	0.794	0.824	0.818	0.8	0.764	.	.	.
0.816	0.772	0.813	0.84	.	0.758	0.777	0.773	0.81	0.82	0.82	0.793	.	.	.
0.796	0.777	0.792	0.822	.	0.735	0.762	0.781	0.798	0.815	0.814	0.859	.	.	.
0.802	0.781	0.792	0.823	.	0.721	0.775	0.792	0.797	0.838	0.806	0.801	.	.	.
0.798	0.732	0.816	0.835	.	0.768	0.745	0.786	0.833	0.833	0.781	0.788	.	.	.
0.786	0.785	0.794	0.844	.	0.749	0.736	0.786	0.806	0.825	0.81	0.788	.	.	.
0.836	0.839	0.753	0.829	.	0.743	0.79	0.81	0.825	0.815	0.827	0.77	.	.	.
0.799	0.795	0.79	0.864	.	0.72	0.792	0.82	0.825	0.828	0.796	0.851	.	.	.
0.786	0.823	0.821	0.817	.	0.713	0.775	0.812	0.819	0.8	0.811	0.768	.	.	.
0.832	0.768	0.783	0.835	.	0.721	0.754	0.81	0.815	0.801	0.806	0.77	.	.	.
0.798	0.774	0.784	0.817	.	0.809	0.769	0.79	0.811	0.821	0.805	0.743	.	.	.
0.822	0.783	0.802	0.806	.	0.795	0.805	0.784	0.846	0.805	0.804	0.78	.	.	.
0.826	0.777	0.779	0.826	.	0.727	0.765	0.773	0.855	0.799	0.797	0.859	.	.	.
0.809	0.772	0.772	0.853	.	0.721	0.774	0.819	0.819	0.794	0.795	0.735	.	.	.
0.799	0.711	0.791	0.856	.	0.738	0.768	0.82	0.821	0.769	0.822	0.795	.	.	.
0.839	0.803	0.8	0.857	.	0.768	0.802	0.845	0.825	0.803	0.813	0.901	.	.	.
0.807	0.811	0.798	0.849	.	0.769	0.812	0.798	0.8	0.81	0.839	0.855	.	.	.
0.796	0.826	0.769	0.842	.	0.726	0.812	0.793	0.845	0.792	0.832	0.915	.	.	.
0.792	0.797	0.828	0.816	.	0.7	0.774	0.788	0.84	0.761	0.817	0.922	.	.	.
0.813	0.806	0.792	0.817	.	0.732	0.797	0.785	0.818	0.8	0.791	0.807	.	.	.
0.824	0.801	0.798	0.788	.	0.762	0.823	0.784	0.833	0.806	0.83	0.843	.	.	.
0.782	0.798	0.768	0.771	.	0.786	0.771	0.801	0.811	0.801	0.83	0.79	.	.	.
0.835	0.741	0.765	0.781	.	0.766	0.895	0.795	0.83	0.819	0.783	0.933	.	.	.
0.859	0.755	0.781	0.874	.	0.735	0.872	0.804	0.824	0.848	0.797	0.798	.	.	.
0.824	0.857	0.821	0.86	.	0.777	0.836	0.781	0.817	0.827	0.844	0.911	.	.	.
0.796	0.801	0.811	0.865	.	0.796	0.838	0.757	0.831	0.853	0.833	0.938	.	.	.
0.834	0.808	0.842	0.857	.	0.796	0.872	0.799	0.819	0.81	0.858	1.044	.	.	.
0.828	0.789	0.812	0.789	.	0.716	0.81	0.809	0.82	0.834	0.859	1.121	.	.	.
0.791	0.827	0.798	0.811	.	0.711	0.809	0.783	0.876	0.853	0.853	0.92	.	.	.
0.86	0.812	0.768	0.864	.	0.764	0.804	0.838	0.831	0.825	0.809	0.902	.	.	.
0.845	0.804	0.819	0.868	.	0.795	0.775	0.87	0.857	0.861	0.839	0.747	.	.	.
0.859	0.784	0.839	0.886	.	0.772	0.806	0.81	0.856	0.861	0.835	0.916	.	.	.
0.835	0.798	0.8	0.865	.	0.763	0.87	0.849	0.836	0.819	0.826	0.901	.	.	.
0.847	0.805	0.792	0.796	.	0.79	0.846	0.864	0.875	0.838	0.829	0.831	.	.	.
0.829	0.813	0.849	0.817	.	0.751	0.857	0.826	0.843	0.831	0.832	0.822	.	.	.
0.798	0.822	0.817	0.806	.	0.756	0.839	0.835	0.852	0.806	0.865	0.868	.	.	.
0.835	0.792	0.807	0.773	.	0.766	0.886	0.824	0.787	0.834	0.864	0.878	.	.	.
0.795	0.761	0.805	0.802				0.79	0.831	0.807	0.855	0.897	.	.	.
0.808	0.783	0.838	0.85				0.83	0.838	0.844	0.836	0.782	.	.	.
0.804	0.771	0.801	0.852				0.848	0.846	0.831	0.828	0.887	.	.	.
0.877	0.74	0.824	0.881				0.851	0.841	0.868	0.883	0.808	.	.	.
0.863	0.819	0.802	0.815				0.8	0.787	0.846	0.814	0.866	.	.	.
0.819	0.799	0.819	0.874				0.818	0.822	0.863	0.834	-	.	.	.
0.845	0.757	0.806	0.889				0.825	0.826	0.835	0.859	-	.	.	.
0.844	0.777	0.772	0.807				0.837	0.861	0.822	0.849	0.847	.	.	.
0.822	0.806	0.782	0.851				0.836	0.861	0.838	0.824	0.79	.	.	.
0.825	0.826	0.82	0.833				0.842	0.816	0.806	0.867	0.787	.	.	.
0.849	0.821	0.815	0.832				0.856	0.811	0.853	0.84	0.869	.	.	.
0.853	0.819	0.836	0.84				0.845	0.857	0.817	0.831	0.945	.	.	.
0.852	0.806	0.809	0.83				0.866	0.834	0.784	0.807	0.808	.	.	.
0.798	0.778	0.768	0.855				0.855	0.838	0.854	0.831	0.901	.	.	.
0.811	0.799	0.798	0.867				0.856	0.854	0.829	0.865	0.93	.	.	.
0.82	0.808	0.833	0.82				0.859	0.872	0.797	0.838	0.865	.	.	.
0.807	0.792	0.85	0.822				0.848	0.826	0.824	0.851	0.834	.	.	.
0.8	0.756	0.83	0.839				0.778	0.816	0.831	0.82	0.798	.	.	.
0.817	0.745	0.789	0.835				0.844	0.86	0.859	0.845	0.783	.	.	.
0.809	0.756	0.787	0.803				0.806	0.856	0.795	0.79	0.832	.	.	.
0.809	0.762	0.801	0.8				0.78	0.829	0.8	0.804	0.816	.	.	.
0.783	0.745	0.774	0.793				0.791	0.806	0.823	0.815	0.851	.	.	.
0.784	0.75	0.778	0.814				0.803	0.817	0.807	0.826	0.858	.	.	.
0.831	0.733	0.805	0.802				0.812	0.832	0.816	0.804	0.893	.	.	.
0.795	0.749	0.789	0.797				0.815	0.843	0.823	0.811	0.796	.	.	.
0.765	0.751	0.763	0.814				0.805	0.828	0.826	0.824	0.893	.	.	.
0.815	0.729	0.774	0.819				0.827	0.851	0.807	0.806	0.836	.	.	.
0.822	0.777	0.785	0.804				0.825	0.837	0.815	0.822	0.817	.	.	.
0.826	0.805	0.798	0.825				0.823	0.84	0.841	0.827	0.772	.	.	.
0.802	0.747	0.776	0.834				0.841	0.838	0.834	0.811	0.755	.	.	.
0.804	0.736	0.75	0.809				0.81	0.856	0.817	0.8	0.812	.	.	.
0.788	0.736	0.784	0.833				0.817	0.847	0.816	0.793	0.935	.	.	.
0.779	0.71	0.806	0.829				0.833	0.845	0.813	0.816	0.988	.	.	.
0.797	0.723	0.824	0.83				0.84	0.834	0.834	0.825	0.798	.	.	.
0.808	0.707	0.787	0.826				0.836	0.815	0.814	0.801	0.823	.	.	.
0.794	0.745	0.772	0.822				0.819	0.807	0.82	0.814	0.843	.	.	.
0.802	0.811	0.784	0.828				0.849	0.813	0.823	0.802	0.793	.	.	.
0.783	0.782	0.79	0.811				0.83	0.822	0.801	0.801	0.784	.	.	.
0.817	0.759	0.762	0.811				0.803	0.846	0.839	0.832	0.753	.	.	.
0.796	0.733	0.764	0.818				0.824	0.831	0.805	0.8	0.935	.	.	.
0.807	0.737	0.778	0.82				0.787	0.801	0.788	0.805	0.867	.	.	.
0.81	0.705	0.808	0.816				0.828	0.835	0.779	0.788	0.823	.	.	.
0.796	0.75	0.799	0.825				0.816	0.83	0.83	0.813	0.877	.	.	.
0.82	0.727	0.8	0.817				0.839	0.868	0.797	0.816	0.885	.	.	.
0.821	0.717	0.796	0.851				0.815	0.845	0.796	0.839	0.855	.	.	.
0.803	0.716	0.764	0.831				0.829	0.844	0.812	0.818	0.899	.	.	.
0.773	0.809	0.841	0.814				0.819	0.839	0.836	0.807	1.039	.	.	.
0.797	0.811	0.823	0.835				0.838	0.853	0.822	0.792	1.014	.	.	.
0.763	0.811	0.774	0.819				0.829	0.824	0.818	0.834	0.843	.	.	.
0.819	0.809	0.814	0.821				0.816	0.828	0.826	0.798	0.856	.	.	.
0.839	0.781	0.794	0.819				0.807	0.832	0.809	0.843	0.79	.	.	.
0.848	0.79	0.78	0.784				0.804	0.832	0.783	0.866	0.746	.	.	.
0.774	0.816	0.785	0.78				0.75	0.829	0.808	0.822	0.729	.	.	.
0.778	0.825	0.729	0.794				0.769	0.821	0.834	0.784	0.838	.	.	.
0.837	0.84	0.825	0.834				0.796	0.813	0.799	0.812	0.74	.	.	.
0.797	0.799	0.799	0.83				0.842	0.821	0.812	0.801	0.783	.	.	.
0.833	0.817	0.803	0.839				0.828	0.85	0.867	0.821	-	.	.	.
0.819	0.786	0.863	0.865				0.826	0.841	0.865	0.774	0.84	.	.	.
0.851	0.835	0.787	0.861				0.856	0.855	0.807	0.758	0.922	.	.	.

0.798	0.781	0.808	0.843	0.83	0.821	0.802	0.77	0.814	.	.	.
0.811	0.813	0.757	0.833	0.852	0.854	0.846	0.805	0.898	.	.	.
0.788	0.794	0.8	0.83	0.85	0.814	0.792	0.802	0.831	.	.	.
0.779	0.807	0.828	0.808	0.862	0.824	0.821	0.79	0.936	.	.	.
0.834	0.823	0.825	0.813	0.812	0.833	0.828	0.801	0.908	.	.	.
						0.814	0.836		.	.	.
						0.84	0.847		.	.	.
									.	.	.
