# Supplementary material for: Does prenatal alcohol exposure cause a metabolic syndrome? (Non-)evidence from a mouse model of fetal alcohol spectrum disorder
Source: PLoS One. 2018 Jun 28;13(6):e0199213. doi: 10.1371/journal.pone.0199213 (PMC6023152; doi:10.1371/journal.pone.0199213)
Supplement: S1 Dataset — (ZIP) [file pone.0199213.s010.zip › New folder/Tissue wts.pdf]

SUBJID	SEX	TREAT	Weight	Mammary	Liver	Kidneys	Pancreas	Adrenals	Spleen	Heart	Thymus	Repro Fat	Muscle
1.2	1	MD	26	0.1222	1.4433	0.3373	0.5731	0.0061	0.0625	0.1404	0.0367	0.4055	0.0894
2.5	2	MD	20.8	0.0711	0.9059	0.2579	0.3492	0.0044	0.0745	0.162	0.0422	0.1155	0.0448
6.6	2	ETOH	19.9	0.0543	1.0289	0.2778	0.3356	0.0061	0.0661	0.1164	0.0321	0.1136	0.0455
9.2	1	ETOH	27.8	0.116	1.321	0.3393	0.57	0.006	0.0711	0.1391	0.0497	0.4314	0.1036
9.8	2	ETOH	20.1	0.0545	0.958	0.2517	0.3296	0.0053	0.0615	0.1309	0.037	0.0961	0.0387
10.4	1	ETOH	24.1	0.1184	1.1735	0.3086	0.367	0.0049	0.0657	0.107	0.0267	0.3409	0.1188
10.7	2	ETOH	18.9	0.0477	0.9544	0.2321	0.2729	0.0075	0.0642	0.1339	0.0448	0.1236	0.0351
11.5	1	ETOH	26.6	0.1149	1.2673	0.3455	0.3591	0.0079	0.0756	0.1491	0.0385	0.3861	0.1552
12.2	1	H2O	24.6	0.1576	1.162	0.3559	0.4062	0.0101	0.0605	0.1307	0.0474	0.3825	0.1164
12.5	2	H2O	22	0.0888	0.9406	0.2842	0.3706	0.0082	0.0809	0.1436	0.0441	0.1416	0.0365
13.5	2	MCT	22	0.0654	0.9982	0.2762	0.294	0.008	0.0789	0.1322	0.0516	0.1599	0.0449
14.2	2	MD	21.8	0.0678	1.1002	0.2905	0.4816	0.0071	0.1003	0.1245	0.0472	0.1739	0.0914
16.2	1	ETOH	25.1	0.0953	1.1899	0.3166	0.4191	0.005	0.0642	0.1617	0.0275	0.3149	0.0599
17.2	1	ETOH	27.9	0.0927	1.3771	0.3512	0.3533	0.0046	0.0709	0.1703	0.0313	0.4394	0.0522
18.3	1	ETOH	30.8	0.1105	1.592	0.3762	0.3079	0.0044	0.0801	0.1591	0.0345	0.6152	0.0615
19.3	1	MD	27.1	0.113	1.3464	0.3653	0.3441	0.0044	0.0734	0.1523	0.0445	0.3952	0.0392
20.1	1	MCT	28.9	0.1259	1.0865	0.373	0.4157	0.0071	0.094	0.1883	0.0386	0.4747	0.0762
20.4	2	MCT	20.9	0.074	0.9906	0.266	0.3274	0.0078	0.0829	0.1467	0.057	0.184	0.0301
21.1	1	MCT	26.5	0.1064	1.3261	0.3105	0.3516	0.005	0.0826	0.1514	0.00436	0.3797	0.0494
21.8	2	MCT	20	0.0589	0.8847	0.2528	0.2699	0.0089	0.0715	0.1253	0.0357	0.0881	0.0388
24.2	2	MD	21.8	0.0562	1.0251	0.2778	0.3255	0.0091	0.08	0.1315	0.0422	0.099	0.0437
25.6	2	MD	21.3	0.0646	0.7643	0.3273	0.3898	0.0054	0.0985	0.1784	0.0505	0.1595	0.0425
26.1	1	MD	25.2	0.0584	1.3164	0.3564	0.4086	0.0048	0.0555	0.1324	0.263	0.2541	0.046
26.5	2	MD	22.4	0.0888	1.239	0.2779	0.3943	0.0096	0.103	0.118	0.0481	0.1725	0.0436
28.3	2	ETOH	21.5	0.1037	0.9765	0.2821	0.4062	0.0069	0.0743	0.1287	0.0381	0.1492	0.1091
32.4	1	MCT	23.8	0.1214	1.0706	0.2917	0.341	0.0056	0.0621	0.1292	0.0368	0.3805	0.1305
32.7	2	MCT	21.4	0.0658	1.0205	0.2737	0.2955	0.0063	0.0832	0.1363	0.0433	0.1142	0.0746
34.7	2	MD	18.6	0.0729	0.8616	0.2291	0.3173	0.0077	0.0689	0.1007	0.0525	0.1287	0.0491
37.4	1	H2O	28.6	0.1188	1.2932	0.3622	0.332	0.0069	0.0745	0.18	0.0357	0.5984	0.0444
37.7	2	H2O	21.9	0.0619	0.9623	0.2752	0.2464	0.0072	0.0839	0.1313	0.057	0.1396	0.0396
38.4	1	MD	27.2	0.1424	1.2109	0.3529	0.3185	0.0075	0.0756	0.1477	0.0355	0.5286	0.1562
38.7	2	**MD**	**21**	**0.0616**	**1.0019**	**0.2718**	0.3071	0.0065	0.0947	0.1311	0.0344	0.1051	0.0352
40.1	2	MCT	20.5	0.0692	0.9572	0.2596	0.2646	0.0077	0.0773	0.1531	0.0403	0.1024	0.0353
41.6	1	ETOH	26.8	0.1792	1.1233	0.3299	0.4775	0.0041	0.0719	0.144	0.0363	0.4522	0.1049
42.6	2	ETOH	21.6	0.0726	1.0193	0.293	0.2596	0.008	0.0729	0.1257	0.0479	0.1254	0.0626
43.1	1	MD	25.6	0.1263	1.0501	0.3165	0.3069	0.0022	0.0694	0.1577	0.0357	0.3829	0.1447
43.7	2	MD	19.3	0.0569	0.8415	0.2529	0.2149	0.0061	0.0657	0.136	0.0311	0.1231	0.0405
44.2	1	MCT	28.8	0.1011	1.4152	0.351	0.3329	0.0063	0.0802	0.1635	0.0357	0.3919	0.167
45.6	2	MCT	20	0.0439	0.9154	0.2799	0.2517	0.0072	0.0747	0.1479	0.044	0.0749	0.0301
46.1	1	H2O	24.9	0.1234	0.9629	0.3275	0.2878	0.0055	0.0678	0.1195	0.0444	0.4489	0.1563
46.7	2	H2O	19.3	0.0517	0.8806	0.2548	0.2503	0.008	0.0643	0.1434	0.0369	0.0851	0.0365
47.3	1	MCT	.	0.0802	1.2206	0.3566	0.2911	0.004	0.0636	0.1508	0.0328	0.4152	0.0499
50.2	1	H2O	26.9	0.0952	0.954	0.3814	0.4336	0.0068	0.0831	0.1834	0.0369	0.3129	0.1784
50.4	2	H2O	20.1	0.086	0.797	0.2595	0.2647	0.0088	0.0695	0.143	0.0447	0.1164	0.0408
52.3	1	MD	26	0.1318	1.0529	0.3144	0.336	.	0.0641	0.1278	0.0388	0.1503	0.1445
53.4	2	ETOH	21.6	0.0714	0.9917	0.2732	0.3074	0.0068	0.0802	0.156	0.031	0.1202	0.044
62.5	1	H2O	28.2	0.2219	1.1206	0.3292	0.5457	0.0081	0.0701	0.1295	0.0417	0.7104	0.1376
63.1	1	H2O	24.3	0.1867	0.953	0.2701	0.4175	0.0028	0.0539	0.1232	0.0271	0.5356	0.1937
63.7	2	H2O	19.8	0.0614	0.8676	0.2268	0.279	0.008	0.0611	0.1104	0.0472	0.2126	0.0399
64.1	1	MD	26.7	0.0615	1.1138	0.35	0.4177	0.0054	0.0647	0.1455	0.0381	0.3825	0.1486
65.2	1	MCT	24	0.065	1.0598	0.2809	0.3835	0.0043	0.066	0.1171	0.0415	0.2983	0.1342
65.7	2	MCT	20.5	0.0716	0.8494	0.2449	0.309	0.0071	0.0704	0.1513	0.0291	0.1361	0.0295
68.5	2	MCT	21.5	0.1007	0.6854	0.285	0.4103	0.0103	0.087	0.132	0.0506	0.2127	0.0381
69.3	2	H2O	19.7	0.094	0.735	0.223	0.3135	0.0071	0.0597	0.1078	0.0367	0.2404	0.0573
69.7	1	H2O	24.2	0.1668	0.9889	0.2719	0.3868	0.0037	0.055	0.1213	0.0473	0.4434	0.137
71.2	1	ETOH	25.30	0.16	1.06	0.28	0.4699	0.0045	0.0634	0.1183	0.0417	0.394	0.1275
71.6	2	ETOH	21.2	0.1124	0.9588	0.2553	0.3802	0.0071	0.0912	0.1246	0.0396	0.2166	0.0345
74.4	1	H2O	27.8	0.088	1.2772	0.3366	0.4915	0.0026	0.0635	0.1543	0.0494	0.5335	0.0339
74.6	2	H2O	21.3	0.1129	0.9045	0.2791	0.3702	.	0.0814	0.1157	0.0439	0.129	0.1247
75.2	1	MD	28.4	0.1036	1.2165	0.3331	0.5075	0.0058	0.0648	0.1476	0.043	0.5	0.0385
76.6	2	H2O	21.1	0.0985	0.8646	0.2446	0.4175	0.0079	0.0706	0.107	0.0482	0.1353	0.1051
77.1	1	MCT	28.1	0.1021	0.8999	0.3652	0.5199	0.008	0.0892	0.2342	0.0315	0.288	0.0344
77.5	2	MCT	20.3	0.0752	0.64	0.3	0.4627	0.0094	0.0727	0.14	0.033	0.1131	0.028
78.2	1	MCT	27.1	0.1033	0.936	0.3729	0.5973	0.0063	0.0768	0.1843	0.0366	0.3836	0.0325
79.5	2	H2O	20.8	0.0931	0.9574	0.2438	0.4079	0.0067	0.0724	0.1149	0.0444	0.2167	0.1145
82.2	1	ETOH	21.3	0.1112	1.0067	0.268	0.3716	0.0087	0.0708	0.1073	0.0559	0.1613	0.1254

Tibia	Brown Fat	Brain	Intestine	Colon	% Mammary	% Liver	% Kidneys	% Pancreas	% Adrenals	% Spleen	% Heart	% Thymus
18.32	0.0841	0.4704	37.5	7	0.4700	5.5512	1.2973	2.2042	0.0235	0.2404	0.5400	0.1412
18.24	0.0544	0.4561	38	6.4	0.3418	4.3553	1.2399	1.6788	0.0212	0.3582	0.7788	0.2029
17.94	0.0489	0.415	36	7.7	0.2729	5.1704	1.3960	1.6864	0.0307	0.3322	0.5849	0.1613
12.6	0.0866	0.4269	37	8.5	0.4173	4.7518	1.2205	2.0504	0.0216	0.2558	0.5004	0.1788
18.9	0.0489	0.4443	40	7.2	0.2711	4.7662	1.2522	1.6398	0.0264	0.3060	0.6512	0.1841
17.18	0.057	0.4033	34	7	0.4913	4.8693	1.2805	1.5228	0.0203	0.2726	0.4440	0.1108
18.95	0.0278	0.4463	45.6	7.5	0.2524	5.0497	1.2280	1.4439	0.0397	0.3397	0.7085	0.2370
17.62	0.0505	0.4119	35	6	0.4320	4.7643	1.2989	1.3500	0.0297	0.2842	0.5605	0.1447
17.79	0.0557	0.4539	32.5	6.5	0.6407	4.7236	1.4467	1.6512	0.0411	0.2459	0.5313	0.1927
17.08	0.0612	0.4761	38.4	5.7	0.4036	4.2755	1.2918	1.6845	0.0373	0.3677	0.6527	0.2005
16.41	0.0267	0.4501	39.6	7.1	0.2973	4.5373	1.2555	1.3364	0.0364	0.3586	0.6009	0.2345
17.69	0.0692	0.4732	41.5	7	0.3110	5.0468	1.3326	2.2092	0.0326	0.4601	0.5711	0.2165
17.65	0.0337	0.4452	42	6.8	0.3797	4.7406	1.2614	1.6697	0.0199	0.2558	0.6442	0.1096
16.64	0.0324	0.4644	40	7.8	0.3323	4.9358	1.2588	1.2663	0.0165	0.2541	0.6104	0.1122
18.65	0.0917	0.4514	40.2	8.9	0.3588	5.1688	1.2214	0.9997	0.0143	0.2601	0.5166	0.1120
18.3	0.0826	0.4347	41	7.6	0.4170	4.9683	1.3480	1.2697	0.0162	0.2708	0.5620	0.1642
18.15	0.0619	0.4818	42.3	7.5	0.4356	3.7595	1.2907	1.4384	0.0246	0.3253	0.6516	0.1336
17.57	0.0564	0.4479	37.3	7.8	0.3541	4.7397	1.2727	1.5665	0.0373	0.3967	0.7019	0.2727
17.68	0.0484	0.4528	40	7.1	0.4015	5.0042	1.1717	1.3268	0.0189	0.3117	0.5713	0.0165
17.6	0.0455	0.437	40.1	7.2	0.2945	4.4235	1.2640	1.3495	0.0445	0.3575	0.6265	0.1785
18.14	0.0242	0.4645	40.5	8	0.2578	4.7023	1.2743	1.4931	0.0417	0.3670	0.6032	0.1936
17.6	0.0353	0.4762	52.4	7.4	0.3033	3.5883	1.5366	1.8300	0.0254	0.4624	0.8376	0.2371
16.61	0.0357	0.4489	35	9	0.2317	5.2238	1.4143	1.6214	0.0190	0.2202	0.5254	1.0437
18.76	0.326	0.4586	36.5	.	0.3964	5.5313	1.2406	1.7603	0.0429	0.4598	0.5268	0.2147
.	0.0348	0.4509	38	6.5	0.4823	4.5419	1.3121	1.8893	0.0321	0.3456	0.5986	0.1772
.	0.0527	0.4632	33.5	6.3	0.5101	4.4983	1.2256	1.4328	0.0235	0.2609	0.5429	0.1546
.	0.0305	0.4646	34	.	0.3075	4.7687	1.2790	1.3808	0.0294	0.3888	0.6369	0.2023
.	0.0341	0.4083	34.5	6.5	0.3919	4.6323	1.2317	1.7059	0.0414	0.3704	0.5414	0.2823
18.44	0.0446	0.45	38.1	7.1	0.4154	4.5217	1.2664	1.1608	0.0241	0.2605	0.6294	0.1248
17.87	0.0326	0.4499	41.1	8	0.2826	4.3941	1.2566	1.1251	0.0329	0.3831	0.5995	0.2603
17.71	0.0513	0.4291	38	10	0.5235	4.4518	1.2974	1.1710	0.0276	0.2779	0.5430	0.1305
17.09	0.0227	0.4543	38	4.5	0.2933	4.7710	1.2943	1.4624	0.0310	0.4510	0.6243	0.1638
15.85	0.0267	0.4627	38.7	7.8	0.3376	4.6693	1.2663	1.2907	0.0376	0.3771	0.7468	0.1966
18.5	0.0534	0.4339	49	8	0.6687	4.1914	1.2310	1.7817	0.0153	0.2683	0.5373	0.1354
20.67	0.0285	0.4443	39	8.2	0.3361	4.7190	1.3565	1.2019	0.0370	0.3375	0.5819	0.2218
16.4	0.0537	0.4033	38	9	0.4934	4.1020	1.2363	1.1988	0.0086	0.2711	0.6160	0.1395
17.38	0.032	0.444	38	7.6	0.2948	4.3601	1.3104	1.1135	0.0316	0.3404	0.7047	0.1611
17.04	0.0389	0.4643	40.5	9.5	0.3510	4.9139	1.2188	1.1559	0.0219	0.2785	0.5677	0.1240
17.14	0.0265	0.4699	38	8	0.2195	4.5770	1.3995	1.2585	0.0360	0.3735	0.7395	0.2200
16.78	0.0602	0.4593	39.5	9	0.4956	3.8671	1.3153	1.1558	0.0221	0.2723	0.4799	0.1783
17.97	0.0187	0.4518	40.5	7.1	0.2679	4.5627	1.3202	1.2969	0.0415	0.3332	0.7430	0.1912
17.27	0.0468	0.4586	40.5	8.7	.	.	.	.	.	.	.	.
18.07	0.0537	0.4864	41	10	0.3539	3.5465	1.4178	1.6119	0.0253	0.3089	0.6818	0.1372
17.51	0.0262	0.4335	38.7	8	0.4279	3.9652	1.2910	1.3169	0.0438	0.3458	0.7114	0.2224
14.73	0.0396	0.4621	40	9	0.5069	4.0496	1.2092	1.2923	.	0.2465	0.4915	0.1492
17.86	0.0249	0.4695	45	8.8	0.3306	4.5912	1.2648	1.4231	0.0315	0.3713	0.7222	0.1435
17.07	0.058	0.4755	36.5	7.5	0.7869	3.9738	1.1674	1.9351	0.0287	0.2486	0.4592	0.1479
17.15	0.0525	0.4377	34	.	0.7683	3.9218	1.1115	1.7181	0.0115	0.2218	0.5070	0.1115
17.6	0.0357	0.4385	35	7.4	0.3101	4.3818	1.1455	1.4091	0.0404	0.3086	0.5576	0.2384
17.64	0.0414	0.446	36.5	9	0.2303	4.1715	1.3109	1.5644	0.0202	0.2423	0.5449	0.1427
17.75	0.042	0.4643	39	8	0.2708	4.4158	1.1704	1.5979	0.0179	0.2750	0.4879	0.1729
19.53	0.0321	0.4556	39.4	7.1	0.3493	4.1434	1.1946	1.5073	0.0346	0.3434	0.7380	0.1420
17.75	0.0331	0.4541	37.4	8.4	0.4684	3.1879	1.3256	1.9084	0.0479	0.4047	0.6140	0.2353
17.74	0.0335	0.4194	37.1	7.3	0.4772	3.7310	1.1320	1.5914	0.0360	0.3030	0.5472	0.1863
18.35	0.057	0.4161	38.5	8	0.6893	4.0864	1.1236	1.5983	0.0153	0.2273	0.5012	0.1955
18.39	0.0533	0.4685	.	8	0.6332	4.2043	1.1253	1.8573	0.0178	0.2506	0.4676	0.1648
17.95	0.0335	0.466	36.2	7.2	0.5302	4.5226	1.2042	1.7934	0.0335	0.4302	0.5877	0.1868
15.73	0.0508	0.4463	37	10	0.4131	5.9962	1.5803	2.3075	0.0122	0.2981	0.7244	0.2319
17.49	0.033	0.4523	38.2	7.5	0.4018	3.2189	0.9932	1.3174	.	0.2897	0.4117	0.1562
17.74	0.049	0.455	40	8.5	0.4864	5.7113	1.5638	2.3826	0.0272	0.3042	0.6930	0.2019
19.06	0.0406	0.4773	39	9.1	0.3468	3.0444	0.8613	1.4701	0.0278	0.2486	0.3768	0.1697
17.21	0.0565	0.4332	36.5	10	0.4839	4.2649	1.7308	2.4640	0.0379	0.4227	1.1100	0.1493
17.25	0.039	0.4681	37	8	0.2705	2.3022	1.0791	1.6644	0.0338	0.2615	0.5036	0.1187
17.57	0.0643	0.4854	41.3	8.5	0.5089	4.6108	1.8369	2.9424	0.0310	0.3783	0.9079	0.1803
18.31	0.0393	0.4543	32.3	5.8	0.4476	4.6029	1.1721	1.9611	0.0322	0.3481	0.5524	0.2135
18.68	0.0415	0.4416	35	7.5	0.4103	3.7148	0.9889	1.3712	0.0321	0.2613	0.3959	0.2063

% Repro Fat	% Muscle	% Tibia	% Brown Fat	% Brain
1.5596	0.3438	70.4615	0.3235	1.8092
0.5553	0.2154	87.6923	0.2615	2.1928
0.5709	0.2286	90.1508	0.2457	2.0854
1.5518	0.3727	45.3237	0.3115	1.5356
0.4781	0.1925	94.0299	0.2433	2.2104
1.4145	0.4929	71.2863	0.2365	1.6734
0.6540	0.1857	100.2646	0.1471	2.3614
1.4515	0.5835	66.2406	0.1898	1.5485
1.5549	0.4732	72.3171	0.2264	1.8451
0.6436	0.1659	77.6364	0.2782	2.1641
0.7268	0.2041	74.5909	0.1214	2.0459
0.7977	0.4193	81.1468	0.3174	2.1706
1.2546	0.2386	70.3187	0.1343	1.7737
1.5749	0.1871	59.6416	0.1161	1.6645
1.9974	0.1997	60.5519	0.2977	1.4656
1.4583	0.1446	67.5277	0.3048	1.6041
1.6426	0.2637	62.8028	0.2142	1.6671
0.8804	0.1440	84.0670	0.2699	2.1431
1.4328	0.1864	66.7170	0.1826	1.7087
0.4405	0.1940	88.0000	0.2275	2.1850
0.4541	0.2005	83.2110	0.1110	2.1307
0.7488	0.1995	82.6291	0.1657	2.2357
1.0083	0.1825	65.9127	0.1417	1.7813
0.7701	0.1946	83.7500	1.4554	2.0473
0.6940	0.5074	.	0.1619	2.0972
1.5987	0.5483	.	0.2214	1.9462
0.5336	0.3486	.	0.1425	2.1710
0.6919	0.2640	.	0.1833	2.1952
2.0923	0.1552	64.4755	0.1559	1.5734
0.6374	0.1808	81.5982	0.1489	2.0543
1.9434	0.5743	65.1103	0.1886	1.5776
0.5005	0.1676	81.3810	0.1081	2.1633
0.4995	0.1722	77.3171	0.1302	2.2571
1.6873	0.3914	69.0299	0.1993	1.6190
0.5806	0.2898	95.6944	0.1319	2.0569
1.4957	0.5652	64.0625	0.2098	1.5754
0.6378	0.2098	90.0518	0.1658	2.3005
1.3608	0.5799	59.1667	0.1351	1.6122
0.3745	0.1505	85.7000	0.1325	2.3495
1.8028	0.6277	67.3896	0.2418	1.8446
0.4409	0.1891	93.1088	0.0969	2.3409
.	.	.	.	.
1.1632	0.6632	67.1747	0.1996	1.8082
0.5791	0.2030	87.1144	0.1303	2.1567
0.5781	0.5558	56.6538	0.1523	1.7773
0.5565	0.2037	82.6852	0.1153	2.1736
2.5191	0.4879	60.5319	0.2057	1.6862
2.2041	0.7971	70.5761	0.2160	1.8012
1.0737	0.2015	88.8889	0.1803	2.2146
1.4326	0.5566	66.0674	0.1551	1.6704
1.2429	0.5592	73.9583	0.1750	1.9346
0.6639	0.1439	95.2683	0.1566	2.2224
0.9893	0.1772	82.5581	0.1540	2.1121
1.2203	0.2909	90.0508	0.1701	2.1289
1.8322	0.5661	75.8264	0.2355	1.7194
1.5573	0.5040	72.6877	0.2107	1.8518
1.0217	0.1627	84.6698	0.1580	2.1981
2.5047	0.1592	73.8498	0.2385	2.0953
0.4591	0.4438	62.2420	0.1174	1.6096
2.3474	0.1808	83.2864	0.2300	2.1362
0.4764	0.3701	67.1127	0.1430	1.6806
1.3649	0.1630	81.5640	0.2678	2.0531
0.4068	0.1007	62.0504	0.1403	1.6838
1.8897	0.1601	86.5517	0.3167	2.3911
1.0418	0.5505	88.0288	0.1889	2.1841
0.5952	0.4627	68.9299	0.1531	1.6295
