# Supplementary material for: Does prenatal alcohol exposure cause a metabolic syndrome? (Non-)evidence from a mouse model of fetal alcohol spectrum disorder
Source: PLoS One. 2018 Jun 28;13(6):e0199213. doi: 10.1371/journal.pone.0199213 (PMC6023152; doi:10.1371/journal.pone.0199213)
Supplement: S1 Dataset — (ZIP) [file pone.0199213.s010.zip › New folder/VO2 one.pdf]

Blk	3	3	3	3	4	4	5	7	7	8	3	4	4	7	7	8	10	10	10	12	12	12
Box	Box-3	Box-4	Box-5	Box-6	Box-2	Box-3	Box-4	Box-3	Box-4	Box-4	Box-7	Box-5	Box-8	Box-1	Box-7	Box-2	Box-6	Box-5	Box-4	Box-1	Box-2	Box-5
ID	6.4	9.6	10.6	11.1	17.8	18.7	28.7	41	42	53.5	12.4	23.1	27.7	37	46	50.6	62	63	69	74	76	81
Sex	F	F	F	F	F	F	F	F	F	F	F	F	F	F	F	F	F	F	F	F	F	F
Exposure	EtOH	EtOH	EtOH	EtOH	EtOH	EtOH	EtOH	EtOH	EtOH	EtOH	H2O	H2O	H2O	H2O	H2O	H2O	H2O	H2O	H2O	H2O	H2O	H2O
Tx	FE	FE	FE	FE	FE	FE	FE	FE	FE	FE	FH	FH	FH	FH	FH	FH	FH	FH	FH	FH	FH	FH
Chow	2350	2380	2481	4467	2673	2824	1947	1844	1742	1972	2543	2606	2720	1829	2407	1855	2130	2163	2526	2757	2433	3097
	2468	3229	4191	4041	3897	3948	3775	1818	2437	1882	3468	2227	2447	2638	3223	1995	2232	3550	4007	3468	2810	2643
	3588	3576	3544	2589	3648	3928	4173	2856	3424	3981	3445	2196	3712	3136	1824	3229	2246	3754	3685	2764	2479	2655
	2518	2641	2085	2723	2435	3598	2370	3564	2118	3769	3295	4180	4119	1758	1974	1952	2539	3238	2699	2841	2561	4379
	2338	2538	4178	3811	2665	2456	2744	3616	1737	3371	2363	3978	3680	1797	2708	3276	3006	4335	2792	3897	3524	3748
	2506	3372	3661	4363	3409	2608	3773	4050	2422	2257	2505	3308	2246	3087	2023	2952	2513	3090	3774	3986	3591	2404
	2502	2201	2383	4371	4306	2688	3457	3937	2829	3379	3499	3063	4279	2566	1698	1580	2242	2323	3744	3307	2547	3961
	3830	2801	3121	3958	4047	3839	2829	3995	2705	3774	3760	3800	3964	2636	1770	2891	2451	3918	3111	2802	2969	3804
	3400	3814	3658	4013	2353	4041	2915	3935	2594	3882	2419	4055	3711	2817	3077	2712	3123	4530	3033	2608	3716	3256
	3084	3587	3573	4020	2631	4038	3535	3695	3320	3976	2189	3558	4669	3604	3607	2847	3477	4958	3618	4123	3685	4312
	2865	2004	4120	4310	4260	4026	3663	4049	2815	3868	2405	4325	3105	3386	3541	1722	3223	3530	3608	4048	3099	4166
	3553	3168	3272	3931	3052	4048	3755	3878	2939	3861	3977	4671	4070	3406	3399	1138	3414	2650	2312	4040	2427	3802
	3754	3361	3512	4245	2227	2562	2414	3934	2961	4442	4148	3302	4186	3528	2030	3021	3235	2374	2226	4421	3776	4135
	3356	4040	4683	3647	2479	2481	2704	3563	2956	3856	4051	3651	5052	3982	3756	3738	3404	3999	3664	4104	4389	4846
	4341	3667	2981	2390	3975	4114	3648	3390	1992	3921	3781	4728	4711	4167	2843	2969	3284	4396	3397	4114	3783	4853
	4514	4455	4019	4021	3953	4291	3426	3139	3066	3656	2577	4791	3081	4342	1951	3693	2588	3295	3628	4772	4088	4617
	4390	4109	4062	4484	3537	4170	3538	3281	3163	3849	2326	3078	2491	4336	4086	2373	1391	4089	3571	4647	3995	5036
	4423	4266	4494	3323	3949	4178	4044	3320	3438	3869	2777	4793	4067	4408	3691	1383	2795	4475	3826	4902	3386	5107
	4811	3101	3524	2458	3528	4704	3812	3515	2917	3297	3693	4133	4816	4437	3973	1360	3033	3158	2883	4859	3839	5043
	3501	3697	2456	2465	3687	4340	3242	3156	2072	1832	3975	2828	4907	4293	2397	2160	3122	3623	1772	4735	4138	4561
	2624	4406	2446	2479	2451	3708	1849	1926	1566	1480	4499	2407	4486	4311	1869	3473	2837	3429	1929	4583	3930	4479
	3109	4691	4003	2622	4104	2385	1636	1554	1393	3408	4591	2773	4133	4432	2202	3833	4366	2840	2039	4814	2375	4437
	3654	4608	4093	3598	3697	2160	1677	1716	1898	3803	3955	2292	2827	4486	4289	3827	4607	2309	3658	4954	3081	4120
	2777	4468	2743	4235	1962	2445	1841	1602	1775	3226	4214	4278	3218	4294	4106	3240	4399	2151	3323	4430	2181	3554
	3574	4818	2165	4367	1144	3541	2784	2858	1816	2526	4198	4240	3079	3860	3799	1912	4169	3735	2601	4281	2217	2905
	4127	4154	4016	3654	1213	2832	2044	2995	3023	1966	3917	4056	2729	2977	3129	1722	3737	2983	1578	3227	2290	2692
	4367	2653	3348	3330	995	2370	3253	1396	2349	1646	3700	2838	2917	1650	1912	1762	3747	2090	1399	2397	2163	2973
	4129	3489	2551	2744	876	2631	3003	1441	1268	1710	3911	2332	3978	1579	1733	2200	3517	2160	3515	2679	2510	3015
	4096	2827	3427	2470	837	3093	4171	1630	1397	1752	2989	2284	4362	2769	2058	2083	1733	3741	2950	2444	3549	3101
	3892	2805	4044	2515	687	3128	3540	1626	1753	2236	2302	2080	3444	2279	2764	3352	1161	3501	2943	3576	3138	4257
	2573	2323	3798	2783	3508	2425	1813	2409	1569	3765	2248	2709	2429	1925	3668	3459	1015	2848	1570	2828	3219	4530
	1905	4406	3092	2353	3870	2352	1959	3311	2038	3049	2594	2330	2663	1833	2113	3165	1592	2138	1316	3868	1966	4120
	3011	4133	2208	3231	4466	2417	1705	2182	2405	3296	2239	3308	3765	2099	2092	2011	1387	3567	1362	4284	1881	3103
	2071	3649	3204	2529	4358	2326	1403	1426	2699	1992	2879	4758	4189	2109	3270	2597	1604	3367	3669	4068	3293	3519
	2623	3738	3248	2502	4492	3983	3678	1363	1869	1454	3737	4485	2697	2800	3478	2135	2317	2354	3014	2830	2336	3079
	1851	2533	2307	2543	4347	3396	4034	1534	869	1504	4298	3704	2419	4157	3299	1765	1609	2183	2163	2176	1713	3780
	2030	2464	1978	2548	4300	2174	2729	2543	1104	1659	3684	2720	4551	3778	2166	3509	1607	2664	1370	2331	1922	4537
	2128	3878	2209	4587	4065	2229	1873	3470	1528	2861	3181	2375	4496	2419	2086	3377	1798	3675	1249	2336	2001	4317
	1896	2847	4032	4151	4326	2920	1748	3417	2484	3657	2033	2268	3227	1979	2219	2386	3899	3379	1621	2411	2259	3918
	1927	2145	2477	2949	4116	3910	1969	2477	1281	3578	2138	2909	3777	3135	2050	1685	4212	3094	2325	4454	4087	3320
	3030	2311	2075	2504	3321	3647	4265	1638	1068	2260	2302	3358	2944	3982	2636	1928	3559	2159	2854	4374	3539	2161
	4293	3644	2194	2671	2152	2964	3441	2200	1647	1794	2341	2534	2420	3742	3442	2715	3288	2082	1856	4090	3240	2496
	3745	3398	3415	2525	2043	2202	2064	2406	3232	1729	4042	2428	2440	2620	2618	1861	2042	2842	1804	3624	2012	2511
	2704	2390	2517	4040	2200	2410	1885	1760	1328	2219	3628	2799	4063	2724	2261	1925	1533	2538	2348	2418	2374	2767
	2119	2113	2186	3977	2447	2234	2627	1714	1194	3651	2082	3576	3804	2137	3084	3174	1555	2344	2119	2400	1905	2532
	2456	3853	3605	3094	2208	3580	2045	1886	1478	3721	2400	3353	3061	2035	2740	1647	1681	2538	2132	2396	3532	2970
	3882	2705	3588	2613	3519	3352	2014	2960	1557	2332	2477	2328	2301	3773	2021	1795	1855	2375	2037	2223	2460	2453
	2704	2343	3332	2760	2550	2195	3711	2748	3425	1697	4145	2356	2540	3927	1890	3331	1867	2353	1994	3709	2020	3744
	2331	3449	2288	2811	2180	2396	2447	1814	3359	1879	3297	2910	2313	2676	3356	2067	1453	3432	2623	3494	2105	3907
	2349	3526	2124	2834	2393	2166	2180	1694	2282	2074	2263	2305	3414	1880	3213	1617	1509	3375	1972	2426	3389	2619
	2363	2179	3035	2571	4314	3258	2711	1421	1513	1904	2251	2151	3753	2963	2329	1781	1905	2506	1877	2435	3531	2720
	4004	2231	2559	2572	4194	2936	1945	1728	1385	2056	2733	2117	2463	2229	3133	3109	3833	2173	1990	2409	2211	3954
	3813	2989	2561	2616	4188	2268	1929	1568	1525	2402	2445	3321	3360	2916	1896	4051	3033	2531	2023	2393	1951	3416
	2749	2463	2746	3826	3485	2237	2258	1912	1649	3592	2436	2338	3823	2177	2966	3171	1599	2132	2147	3251	3182	2546
	2494	2302	2519	3997	2337	2816	3263	2967	3234	2356	4031	2168	2783	3293	3769	1956	1704	2947	1953	2432	2438	2311
	2500	2282	2290	3264	2212	2425	2053	2259	3119	1894	3434	2606	2828	3787	2693	1868	3459	3247	1971	2492	2199	2521
	2551	3321	2373	2677	2482	2435	1836	1441	2238	2660	3411	3785	3594	2587	2085	1971	3722	2250	2686	2521	2701	3516
	2781	3284	2816	2666	3216	2413	1891	1706	1498	3604	3460	3765	2555	1897	1926	2419	2204	2222	3013	2473	2203	3753
	4059	2056	2561	2678	3533	3510	3870	1633	1286	2924	2506	2407	2435	3588	3221	3102	1436	2101	2037	3740	2353	4015
	4188	2270	2272	3426	2558	3760	2356	1516	1656	1913	2382	2178	3535	2161	3198	1819	1609	2185	2221	3916	3713	3756
	3505	2066	3889	3806	2564	3018	2013	1245	3282	1860	2435	3823	4155	1859	2186	1777	1717	4057	2014	3041	3346	3152
	2551	3360	3682	3202	3445	2288	3742	2212	3519	2036	2722	4119	4221	3082	2415	2411	1653	3804	2129	2419	2207	2640
	2544	2247	2343	2610	2862	2455	2683	3147	1320	3310	2387	3902	2795	2395	2672	3659	1786	2462	3327	2406	2211	2535
	2434	2145	2085	2627	3341	3712	2519	3156	1358	3210	2369	3906	2396	2099	1978	3097	1732	2286	3127	2281	3428	3688
	2479	3691	2766	2947	2897	3685	2224	2208	3355	1968	2240	3651	2570	2025	3247	3042	3479	2076	1773	3434	2819	2556
	2519	3009	3339	2602	2498	2725	3394	1805	3095	1877	3656	2359	2987	1924	3703	2360	3539	3879	2152	3886	2171	2586
	3639	2070	2517	3514	2922	2938	2724	1719	2297	3501	3781	2167	4524	2130	3589	2400	3409	3926	3253	2651	2191	2640
	4046	3847	2630	4096	4126	3994	3811	3209	3413	2933	3373	2333	4280	3485	3634	3341	2004	3924	2830	2482	3670	3922
	3002	4215	3871	3175	4102	4452	3724	2421	2992	2783	2455	3219	4171	4008	3780	2319	1540	2622	3090	2630	3602	3844
	2765	3804	4076	3229	3912	3986	2891	3183	3536	4282	2614	3793	3879	3738	3499	3464	1723	3305	3004	3718	2964	3721
	3559	4100	3877	4460	3417	4490	3588	3910	2521	3359	2936	4298	4546	3716	2228	3539	2850	4185	1935	3857	4254	2754
	3981	3841	3960	4247	2883	4777	3937	3681	3272	2915	4276	3833	4101	2461	2233	2177	4189	4353	3020	2630	3509	4590
	3941	3270	4096	4895	2818	3651	3653	3746	3604	4035	4226	3677	4144	2659	2688	2187	3505	3990	3137	3723	3041	3987
	4254	3909	3966	4053	3871	2377	2379	3606	3981	3828	4126	3879	2735	3014	3326	3434	4150	4010	3394	4223	3964	4357
	4129	4005	4107	4035	3290	2506	3582	3480	3385	4008	3976	4201	2526	4448	2226	2676	3629	4189	3144	3839	3759	3697
	3340	2926	4136	3852	2770	2563	2548	3794	2953	3615	4033	4555	4348	3478	2480	2077	4040	4626	2823	3537	3804	4511
	3684	3703	3915	4043	3524	2451	3439	3464	3827	3648	3193	4325	4572	2718	3781	3114	3490	3126	1791	4046	4258	4570
	3730	2736	2896	4195	3474	3229	2572	3241	3104	3160	2396	4546	4637	1888	3439	2997	2125	2148	1815	2820	3791	4048
	4115	2518	3548	3550	2461	4243	2177	2480	3556	4048	3103	4361	4273	3454	3340	2371	1738	2855	2287	2892	3948	4727
	3975	2531	3305	2716	3530	4031	2227	3317	3389	2171	3403	4102	4312	2288	3559	3485	4087	4639	3164	4109	2832	3650
	4249	3924	2547	2659	2687	3870	3277	2150	2920	2801	2576	4110	4257	2650	3023	2779	3001	4353	3332	4149	3114	2854
	4419	4194	4480	3874	2642	4011	3681	1720	1719	1891	2375	3668	4441	3742	3238	2311	1960	4181	4169	3810	3640	3450
	4563	4442	3531	4032	3261	3489	3845	1839	1324	1612	3519	2649	4014	3894	2704	2959	2181	3768	4434	3128	4433	4674
	4418	3952	2700	3154	3905	3912	3799	2437	1509	2393	3022	1994	2824	3082	3006	4115	3749	3771	4612	2687	4182	4795
	4512	4063	3508	4097	4000	4026	3358	3547	2173	3489	3808	2245	2394	3252	1801	4391	4526	3593	4272	4311	3912	4813
	4576	4417	4312	3972	4024	3713	2037	4023	3359	3698	4218	2201	4160	4268	1981	3842	3531	3732	4541	4487	2581	3455
	4201	4161	4054	3737	3438	2920	2191	3514	2408	3526	3892	2149	4473	4056	1970	3495	3235	3148	3635	4469	3252	4520
	4135	3965	3777	2507	2462	2273	2952	3113	1594	3458	4480	2468	3950	3806	2235	3628	2503	1877	2300	4499	3088	4353
	4153	3917	2299	3896	2799	2389	2048	3257	2000	2978	4002	3774	2730	3466	1741	3331	1821	1945	3132	4339	3601	3150
	3568	4086	3687	3084	2497	2397	2690	2534	3146	2105	3337	3101	2793	2447	2799	3234	1517	1849	3421	4279	3066	4128
	2835	2877	3948	2769	2454	2361	3848	1940	2654	2084	2062	2287	4055	1986	4343	3351	1607	2981	2277	4340	2385	3207
	2579	2469	3865	2612	2635	2271	2752	1699	1684	2374	2203	2064	3405	1765	2363	3279	1795	2748	2226	4061	2217	2714
	3173	2664	2752	3435	2514	2421	2479	1872	2101	2028	3442	2072	2396	1870	2633	2117	2571	2318	3037	3386	3743	3353
	3751	3577	2197	3074	2480	2223	2005	1799	2203	2015	2780	2201	3843	2851	2194	2156	1651	2202	4057	2375	3719	4032
	2905	4037	3581	2894	3803	3440	1911	3013	1536	2057	2472	2316	3998	3214	3499	2275	1743	2182	3550	2298	3747	3113
	2456	3789	2698	2773	3423	3219	1971	3025	1631	1924	4061	2374	3707	3366	2099	2181	2065	3649	2521	2357	3763	2660
	3503	2613	3259	3892	2565	2136	3525	2024	2272	2489	3798	3768	2431	2244	2372	2473	2051	2462	2016	2636	3494	2846
	3639	3141	2992	4027	2346	2319	2588	2523	1355	3547	3502	3556	2573	1685	2758	2066	3871	3559	2896	3345	2396	2865
	3131	2196	2205	3517	2357	2129	1749	3086	1588	3321	3269	2326	2384	1783	3820	2863	3554	2554	4238	2431	2175	4068
	2397	2293	3484	4170	2763	2701	2233	2666	1723	3302	3570	2062	4053	2201	3126	3165	3258	1920	3672	2376	2393	4304
	2527	3697	3736	3433	4355	1987	1755	1665	3333	2440	2348	3955	3693	2148	1887	1980	3078	2189	2522	3335	3239	2913
	2380	2311	3268	2526	4055	3683	3967	1578	3167	1650	2005	4308	2650	3911	1696	1839	2557	3022	1902	3593	2601	3878
	3398	2035	2142	2450	3913	3703	2628	2429	3322	1894	1975	4064	2403	2940	2194	1883	1535	3608	3704	2523	2305	4262
	4176	4063	2133	2731	3485	3408	1809	1677	2890	2260	3518	2764	2662	2031	3537	3029	1146	2601	3499	2687	2598	3861
	2874	3481	2518	2656	3422	3126	2702	1994	2269	3099	2599	2119	3954	3093	2267	3281	1842	1970	2537	2179	3713	3300
	2352	2355	3008	3466	2311	2124	4290	3261	1612	2008	1950	1781	3807	3705	3210	2726	1416	2047	2008	2653	3291	2490
	2266	1902	2764	2446	2470	2208	2314	3149	1727	1843	3048	3019	3128	2500	2710	1995	1646	2030	2115	4315	2097	2911
	2456	3416	2872	2643	2390	3644	1730	1810	1625	2335	2307	2079	2581	1617	1922	3271	1625	2515	3472	3773	2167	2556
	3655	2353	2278	3672	2463	2914	3853	1876	3012	1950	2069	2868	2740	3470	2133	2266	2922	2377	2948	2642	2200	2712
	3982	2033	3952	4015	2410	2118	2169	1864	3525	3971	3136	2902	4063	3206	3405	2235	1753	3008	2387	2285	3293	4281
	3303	2030	2442	2693	2757	2007	2088	2211	2185	2897	3899	1839	2825	2104	2666	3593	3287	2542	2848	2431	3290	3359
	2408	2088	2480	2754	2984	2974	2145	2905	1772	1990	3529	2073	2526	1925	2092	3427	3813	2230	2275	2361	2171	2494
	3333	3705	2488	2800	2317	2227	1990	2824	1582	2098	2492	2952	2936	3239	2456	2487	3269	2213	2208	2517	2264	2732
	2652	3231	3243	3608	2320	2218	2179	2620	1948	3317	2228	3653	3112	3905	2045	3142	3416	2291	2721	2334	2250	2745
	3397	1897	3385	2964	2884	2330	3601	1764	2132	2411	2045	2914	2565	3437	2082	2292	2079	3027	2244	3638	3082	2749
	2359	1870	2703	2702	2388	3262	3018	1948	3268	1880	2984	2042	3584	3449	3192	2184	2005	2216	2228	2750	2616	2441
	2403	3419	2256	2540	2391	2304	1767	1677	2943	1905	2479	1848	3511	2052	2971	2074	1780	2194	2177	2383	2177	3165
	3385	2141	2160	2652	3208	2237		2432	1786	1968	2047	2221	3376	1784	2248	3362	1683	2414	3857	3390	2343	2436
	3768	2008	2265	2802	2829	2894		3294	1732	2099	2332	3341	2595	2568	2955	3190	3607	2144	2684	2589	3671	2577
	3424	2718	2416	4036	2431	2272		2074	1696	1852	2377	2744	2648	1927	1973	2437	2326	2134	2047	2565	3345	3057
	2641	2674	3529	3146	2516	2222		1836	2763	2781	3247	2225	4325	2293	1979	2205	1963	3534	2104	2534	2445	3943
	2417	2127	2432	2790	2757	2463		1843	3161	3893	3035	2165	3252	3763	2821	2582	1912	2707	1942	3830	2163	2701

	2430	2101	2341	2701	2598	3882		1627	2549	3768	2326	2415	2537	2347	1864	2567	1766	1958	2441	3692	2214	2617
	2584	3940	3476	2997	3357	3473		1737	1782	3100	2210	2372	2514	1813	1947	3270	1813	2772	2381	3359	2239	3991
	2559	3184	3280	2774	2615	2535		1876	1520	2060	3422	3412	2878	1787	3119	3501	3574	3465	3230	2391	3464	4047
	3179	2394	2446	2899	2623	2438		3359	1615	2059	3767	4151	2791	3289	3812	2158	3219	2219	1957	2313	3347	3721
	2626	2122	2363	4069	2639	2417		2356	1713	3262	3046	2850	4074	3700	2647	2162	1938	2068	1909	2813	3495	2687
	2522	2224	2408	3982	2638	3413		1791	2622	2389	2128	2185	4130	3425	2224	2103	1652	3260	2259	2558	3484	2766
	3367	3919	3538	2843	3370	3186		1892	3195	1882	2318	2127	2988	2287	2060	3670	3210	2024	3698	2420	2580	2651
	3897	3155	2581	2827	3020	2502		3210	2992	2144	3552	3463	2914	1981	2964	3329	2064	2043	3560	3790	2239	3418
	3200	2386	2431	2799	2509	2523		2652	1958	3443	2813	3432	2921	2004	2060	2298	1636	3309	2133	2820	2126	4020
	2559	2182	3332	3815	2568	3009		1913	1787	3749	2289	2252	3548	1920	2007	3309	2027	3191	3707	2404	3024	2850
	2890	3867	2968	3538	2667	3995		3453	1956	3834	2348	2700	4783	3163	3710	4157	1783	3697	3152	3104	2294	2888
	2747	4208	3816	3042	3589	4225		3750	2602	4156	3962	3716	4380	3095	3843	3889	3550	3798	3132	2726	2395	3419
	3839	3919	4615	4206	2950	3971		3773	3547	4344	4016	4431	4508	3557	2465	3632	3062	3041	2456	3412	3704	4606
	4121	3510	4141	4342	2795	4071		4014	3658	4361	2866	4191	4301	3992	3525	3590	2097	3420	3366	4033	3915	3973
	3310	3530	3245	3301	3079	3794		3689	3474	4006	2562	4154	3150	3501	4295	2867	3463	3871	4076	3348	3961	2844
	3766	4195	3720	3283	3826	3381		3800	2363	3779	4000	4126	2813	2010	2905	2538	2888	4337	2719	4338	3585	4591
	4079	4450	4309	2886	4167	2494		3700	3595	3994	4018	2724	4176	3253	3674	3418	3679	3513	3376	3328	3983	4089
	3372	3882	4097	3632	3509	2780		2481	3445	4238	2874	4127	4343	3750	3656	3596	2408	2185	2468	2649	3898	4442
	2930	3662	4273	4440	3844	4242		1633	3526	3813	2548	4253	4537	3700	2303	2759	3269	3883	2464	3939	3967	4348
	3753	2903	3692	4510	3254	4000		1597	3415	3754	3709	4398	4543	2664	2710	2294	2437	2932	3414	3847	3243	3385
	4407	2635	3544	3422	3530	2660		3296	3192	3797	4214	4362	4288	2339	2672	2500	2106	2426	3481	3909	3249	2711
	3215	4537	3692	4350	3762	3103		3755	3070	3676	3934	4111	4323	2248	2047	3514	3428	3919	2567	2684	3342	2736
	4093	4367	4034	4529	3751	2958		3754	2859	3477	3721	4016	3252	2438	2527	3568	3926	3683	2627	4094	3973	2750
	4605	4435	3018	3982	3783	4088		3943	2172	2098	2527	2590	4018	3216	3641	3538	3645	3650	3485	4106	4101	3379
	4439	4251	4165	3167	3304	3862		2819	1169	1613	3987	2278	3288	4364	2522	3903	2545	3895	3192	3557	3991	2717
	3305	4360	3963	3912	2441	4113		1836	2454	1833	2671	2158	2751	3962	3232	4042	2517	3777	3335	3639	3989	4537
	4428	4164	3209	4283	2475	3162		3042	3385	1712	2273	3071	4121	2761	3240	3708	2007	3936	3685	4571	3434	4270
	4196	3869	4194	4574	2399	2650		3734	3392	3867	3133	3594	4093	2084	2110	3916	1693	3792	4138	4328	3206	4528
	4477	4132	3972	3419	3777	2461		3395	3141	4164	3351	3417	3326	2832	2510	3795	1643	3556	3786	4100	2581	4530
	4211	3866	3663	2748	3741	2299		2455	2034	2583	2507	4308	4335	4022	2023	3743	3036	3367	3613	4221	3256	5024
	4647	3894	3630	3086	3449	3216		2102	1238	3340	2246	4100	4060	4182	2599	3782	3972	3553	3139	4270	3036	4490
	4316	2777	3286	3291	2514	3408		3404	1427	3498	2258	4123	2916	3647	2554	3494	4336	3416	3251	3838	3234	4283
	4145	2569	3213	4061	2413	2450		2802	1439	2904	4353	4043	2803	3680	2067	3106	4200	3474	3605	3505	3728	4057
	4106	3759	3005	3803	2815	2424		1918	3262	1887	4154	3697	3314	3992	2342	2362	2802	2060	3221	2303	3207	4240
	3789	4121	2503	3470	3563	2217		2560	2784	1808	3825	3373	4152	3276	2117	1929	3293	2180	2279	2329	2387	4605
	4185	4412	2776	2828	3605	2272		1989	1732	1958	3514	2594	3202	3118	2611	2907	3650	2761	3327	2427	2302	3353
	2882	3753	4152	2876	3304	2934		2593	2714	1906	4238	2097	2770	1896	1995	3398	2390	2089	2828	3041	2553	2418
	2639	2647	2935	3909	3705	2239		2096	2952	2379	4013	2172	3309	2846	3839	3611	1749	2874	2554	2862	2585	2658
	3724	3640	2581	3887	2533	2297		2838	2065	3885	3605	2206	3499	2934	3738	2330	1643	3123	3776	3043	3247	2585
	2928	4158	3938	3034	2356	2761		3546	1492	3002	3520	2333	4342	2246	3230	2112	1918	2224	3787	2649	3327	2720
	2643	3693	3281	3024	2543	3808		2562	1782	2864	2114	2191	4055	2137	3176	2064	1511	2416	3346	2146	2888	2556
	2579	2637	2553	3096	2573	3934		1774	1819	1878	1805	2833	3084	2345	3233	3437	1897	2318	3936	2003	2308	2182
	2640	2713	2277	3972	3867	3954		1961	3348	3211	3350	3709	3068	3798	3242	3649	2416	2115	3362	1769	2100	2625
	4090	3999	2549	3439	4091	3646		2518	2802	2156	4288	2472	2310	3840	1861	3447	1606	2194	3183	2110	3543	4624
	3024	3466	3874	2797	3804	2484		1980	1722	1601	3765	1987	2445	2607	1888	2223	1725	3747	2389	4424	3909	4152
	2511	2216	3113	2599	3232	2169		2002	1648	1619	2780	1953	4119	1928	2143	1980	3675	3334	2416	4415	3505	2561
	2341	2189	2226	2557	2324	2506		1908	2892	1645	1826	3835	2883	2283	3223	2142	3894	2297	3462	3576	3560	2184
	2654	2353	3805	3793	2266	2755		3452	2788	3012	1981	2473	2597	3675	2945	3085	3706	2128	3373	2674	2639	3147
	2396	2892	3566	3538	3244	3530		3101	2023	2996	2078	2040	2438	3499	1902	2210	2321	2470	2453	2036	2034	4333
	2515	2544	2440	2496	3886	3952		2636	1463	3242	2499	2074	2619	2303	2005	2177	1751	3040	2504	2136	3127	3486
	3751	3562	3255	2772	3993	3432		1849	2843	2258	3762	2024	4259	2106	2559	2104	1768	2105	2604	3586	2869	2616
	2545	3465	2563	2566	3634	2198		1929	3325	2153	2277	3746	4141	1974	3068	2235	2508	2253	2459	2622	2158	2788
	2453	2428	2604	3552	2845	2332		1685	2975	1934	2068	2693	2631	3594	2973	3222	1748	2222	3152	2333	2208	2681
	2763	2462	2749	2650	3054	2667		2591	1863	3403	2237	2387	2587	3140	1897	2351	1730	2133	2449	2400	3063	2775
	3801	3587	3377	3506	2303	2357		1935	1318	2192	3413	2307	2571	2369	2036	2397	2807	3684	2619	2712	3277	2761
	2698	2580	3474	2618	2354	2047		2066	2210	2168	3239	3412	3915	2099	2020	3444	2253	3242	2405	3731	2206	3436
	2428	2509	3384	2601	2629	2497		1816	2852	1935	2094	2691	2887	2235	2022	3162	1687	2125	2375	2469	3270	4325
	2728	2555	2448	3759	3520	3608		3055	2197	3089	2122	2114	2517	2263	3032	2362	3351	2268	2675	2335	2487	2809
	4088	3791	2957	3997	4254	4271	3930	3818	2169	3117	3223	3181	4306	3869	2717	3548	4120	2403	2245	4188	3941	3297
	4038	3236	3230	4356	4010	4190	2954	2508	3449	2927	3772	3701	4347	3356	2404	3801	3999	2335	2530	3506	3641	4098
	4058	3173	2796	4129	4026	3105	2638	3738	2228	2315	4034	3579	4344	3780	2870	3827	3763	3650	4468	4055	3359	3227
	3966	4066	3006	2828	3613	3892	4095	2946	1716	2121	3770	2822	3041	2882	2171	3083	3838	3654	2981	2973	3372	4224
	3784	3002	2869	4069	3480	2949	3086	2632	3022	2209	3138	3640	3727	2750	2381	2691	3382	3616	3801	3283	2768	3106
	2781	2694	2914	3170	3282	2824	2527	3081	1907	3496	3720	2991	3968	2722	2992	3114	2425	3150	3952	4166	2766	2915
	3845	3720	3276	2864	3654	3630	3815	2704	2149	3125	3673	2719	2981	3645	2513	2656	3029	2565	2448	3551	3933	4160
	3213	3121	2910	3121	3016	3397	2457	2782	2171	2090	2599	2689	3026	3706	2169	2460	2614	3542	2379	3197	2761	3481
	3636	4175	3139	3111	3104	3240	2543	3558	2110	2095	3503	3032	4307	2561	3316	3425	2368	3296	2576	3754	2818	3039
	3028	3412	3135	4464	3046	3010	3615	2640	3325	3307	3321	3262	3255	2756	2556	2868	3246	2610	2458	3399	3862	4104
	3748	3151	3382	4038	4189	3271	2980	2577	2247	2346	2785	2771	2977	2500	2261	2490	2275	3944	2606	4202	3017	3236
10kcal%	3124	3040	3362	4695	3152	3989	2392	3209	2174	2544	2771	2853	4145	3216	3521	2765	2087	3006	2607	3535	2652	3215
	2996	3567	3619	4438	3162	4210	2376	3733	3149	3551	2816	2895	3251	3042	2570	3793	3440	2712	3103	3478	3814	3649
	3952	4437	4137	4168	4240	4254	3120	3630	3337	3865	3843	3095	4334	3499	3226	3899	2831	3112	4181	3813	3866	3315
	3523	4501	3996	4600	4714	3523	3316	3604	3860	3571	3081	3663	4524	4118	3813	3340	2580	3481	3130	4050	3274	3253
	4204	3451	4130	4638	4562	3236	2840	3737	3265	3673	3763	4034	3345	3052	4087	3669	2341	4297	3582	3349	2969	4484
	3988	3240	4164	4518	4667	4001	3559	2694	3476	3596	4486	3987	2987	3148	3631	3572	3291	3754	4420	3710	4065	3756
	4071	3597	3444	3298	4709	4036	3385	2744	3248	3900	2995	3674	4230	3678	4328	3199	3300	4311	3901	3734	4519	3711
	3800	3672	3598	4280	4643	3176	3648	2949	2945	3924	2800	4077	4740	3282	2823	3434	3411	3800	3671	4496	3401	3418
	4213	4400	4086	4280	4542	3198	3733	3135	1964	3525	3672	3125	3934	3477	2131	2816	4117	2910	3509	3992	3377	3308
	4277	4195	3404	3233	4402	3649	3914	3983	1819	3704	4320	2852	4562	3492	2947	3180	3473	4002	2847	4381	4052	4051
	3778	3428	3569	3132	4603	4166	3768	3902	1814	3354	5001	3811	4160	3481	3172	3832	3801	3258	2549	4303	3885	4084
	3843	2922	3446	4385	4203	3094	4207	4231	1943	3399	3954	4088	4190	2930	3630	3949	4014	3400	3757	3990	3817	3311
	3418	3969	4186	4537	3333	4116	3079	3703	3129	3370	3170	3177	4981	3450	3769	3330	3619	2992	3735	3557	4200	3232
	2945	3245	3989	4139	3530	4233	4003	3869	3312	4327	4012	2857	3259	3762	3862	2650	3219	3137	3266	3452	4261	3465
	3959	4339	4501	3139	2798	4326	3605	3653	3183	3472	4310	2764	2851	3513	3336	2864	3221	3314	2539	3303	3393	4482
	3891	3846	4550	3320	4140	4820	3257	2767	2186	2708	3548	2897	2868	3212	3038	3861	2471	3661	2784	3794	4010	3889
	4228	4474	4266	4273	4213	4159	2473	3205	1809	2305	3568	4624	3101	3939	2583	3337	1823	3457	3774	3679	3821	4491
	4176	4105	4273	4422	3115	3793	3897	3666	1750	1866	2925	3333	3637	3442	2078	3724	2483	3086	4028	3438	3130	4561
	4281	4023	3731	4140	2802	3045	3310	3477	1966	2325	2966	2985	3578	2544	2576	3596	2045	3793	4011	3656	3226	3320
	3698	4096	3270	3647	2975	4125	2429	3507	3013	1658	2776	3791	3197	3855	2631	2838	1888	3627	3795	3448	3821	4463
	4305	3777	3818	2867	4122	3159	3766	3174	3448	1703	2900	3769	2911	4015	2141	3122	3130	3118	4149	4422	3351	4528
	3865	3216	3801	2960	3280	2719	2889	2911	2216	3054	3364	3594	3607	3633	2083	2917	2489	3167	3017	4694	4202	3640
	4150	4008	3576	2642	2873	2752	2429	3010	1682	3467	3837	3012	4588	3483	2288	2405	2022	2943	2469	4670	4071	3418
	3315	4954	2939	4552	4366	3546	3334	2979	1545	3284	3872	3037	4173	3257	3313	3421	1744	2765	3473	4915	3745	3402
	3113	4036	4056	3950	3258	2768	2720	3032	1823	3129	3768	3079	2817	3223	3067	4220	1751	3168	4201	4109	4020	4032
	3628	3651	3553	3934	2801	2626	2370	3086	2177	3300	3866	3260	2920	3165	2406	3540	3469	2970	3089	4216	3411	3607
	3359	3705	3588	4134	3256	2361	2138	3495	1707	2919	4478	3155	2842	3797	2134	2594	3585	3710	2609	3371	2977	4332
	3672	3265	4048	3173	2936	2849	2861	3220	3037	2321	3381	3583	4164	3102	2271	2690	2299	2977	2555	3374	2987	3705
	3848	3193	3570	3096	2772	4291	2312	3640	2770	2634	4077	3309	3576	2931	2061	3543	1895	3679	2768	3658	3274	3889
	3511	3409	3310	3277	3306	3615	2439	3349	2357	2739	3842	3369	2865	2967	2206	4081	1968	3496	2743	3393	3189	3694
	3019	3154	3667	3848	2977	2796	3609	3227	1711	2531	4059	3175	2661	2708	3333	3630	2476	2878	3799	3436	2920	4305
	2754	2886	3259	3349	3123	2700	2646	3102	1775	3072	3309	2961	4232	3129	2217	2713	2100	2789	2855	4366	2874	3367
	3019	3064	3077	3024	4048	2624	2584	3409	2280	2247	2470	2850	4138	2772	2752	2564	1813	2959	3182	3896	2763	3812
	3469	3903	4150	2890	4590	3009	2237	2739	3085	2091	3685	3526	3086	3531	3496	2581	2525	3401	2552	3028	2917	4237
	2936	3896	3465	2888	4110	2770	2130	2345	2463	1952	2602	3672	2772	3385	2367	3325	3434	3325	3293	2922	3805	4286
	2986	3091	2891	2687	4101	3705	2074	2683	1670	3320	2454	2956	2620	2741	2025	3994	3262	3336	3236	2897	2767	3267
	3113	2587	2887	3432	3857	4077	3688	2391	1692	2248	3961	2735	3979	2864	2233	3494	3007	2827	3541	2957	3463	2975
	3682	2945	3135	4225	3438	3294	3353	2673	2168	2064	3860	3340	2829	3084	3218	2826	2023	3757	3564	2699	3635	3009
	2944	3331	2871	3521	2922	2795	2610	2358	2446	1875	3509	3136	2655	2600	2778	3078	2021	2693	2589	2722	3799	3431
	3237	2827	3260	2613	2803	2778	2066	2767	2166	2916	3133	2894	4456	3276	2755	2603	2977	2794	4177	4043	3143	3789
	2734	3286	2779	2821	3880	2730	3413	2649	2198	3098	2622	2608	4406	3439	2640	2447	2769	3024	2816	3373	2790	3161
	3691	3815	2647	3024	3058	2694	2425	2747	3757	2055	2704	2601	3077	3035	2421	2833	1823	2515	4060	3527	2865	3160
	2690	3283	3076	4091	4070	4048	2168	2465	3115	2252	2779	2429	2862	3746	2954	2520	1873	2633	3036	3054	4063	2992
	2840	2880	2910	4005	3921	3697	2876	2447	2255	2350	2305	3526	2851	2942	2307	2845	3330	2748	2782	3603	2883	2902
	3321	3341	2901	3093	3527	2589	2259	3651	2205	3204	3045	3022	3700	2613	2273	3232	3450	2581	2822	4129	2848	3540
	2921	2606	3835	3970	3024	2855	2729	3381	2528	2263	2432	2611	3042	2488	2327	2756	2724	3228	2650	3309	3711	3170
	2855	3901	3354	3311	3081	3062	3327	2579	2181	2016	2568	2538	4148	3207	2395	2613	2003	2647	3236	2969	3131	3390
	3503	2716	2937	3041	3664	3863	2611	2603	2132	2220	3258	2918	3953	2596	2269	2471	2220	2671	2773	3597	2724	3530
	2781	2789	2896	3228	2893	2833	2821	2618	2786	3031	3686	2517	3017	2232	1914	3304	2780	2713	3648	3188	2766	3459
	3460	2625	3579	3059	3542	2809	3051	2487	2799	2559	3408	3644	2893	2388	3618	2946	2105	3017	3292	3123	3018	2861
	3327	3392	3656	3148	3054	3418	2465	2316	2211	2187	2612	3023	2864	2232	2929	2639	2015	2589	2908	3636	2865	2968
	2657	2641	3057	3079	3517	2824	2938	2724	2114	2567	2578	2566	4360	2435	2264	2684	3429	3303	2632	3092	3065	3150
	3357	3150	2940	3632	3878	3820	2003	3003	2214	3162	3148	2323	4174	2654	2274	2661	3387	2974	2451	3538	2757	2868
	2912	3488	2958	4049	2811	3837	2297	2293	1948	2702	3707	2524	2845	3658	2212	2488	2386	2609	3726	3407	2503	3073
	3417	2666	3503	3124	2908	3009	3110	2080	1819	2088	2590	3488	3084	2376	1928	3137	1910	2569	2727	3030	3355	2888
	2849	2732	2863	2859	3039	2901	2037	3640	2407	2093	2576	3122	3727	3013	2068	2499	2025	3198	2684	3966	2566	3134
	3281	3542	2870	2975	3034	3321	2306	3067	3003	1963	2589	2621	3133	3412	2838	2452	2186	2986	2559	4658	2961	4067
	3002	3137	2882	2766	4145	2891	3606	2211	2610	2843	3945	2647	3569	2797	3395	2619	1978	2790	3133	4349	2961	3994
	3353	2772	3253	2899	3137	2807	2647	1979	1882	3070	2868	2486	2915	2177	2974	3552	2855	2496	3846	3528	3623	3067

3001	2671	3391	4095	3376	4187	2397	3208	1863	2280
3371	4113	2866	3294	3026	4134	3882	3335	2192	2068
2792	3874	2828	2856	3157	3045	3297	2488	2843	1838
3319	3152	2868	3203	3066	3118	2440	2296	1946	2915
2851	2712	3816	4161	3853	3108	2483	2312	1915	2273
3003	3845	2861	3299	3377	3251	2787	2924	2161	2108
3975	3124	2713	3243	4322	4210	2879	3625	3044	2992
3297	3331	3677	4501	4898	3312	2762	3302	3463	3228
3234	4575	4358	4941	5123	4226	3555	3251	3202	3753
3924	4570	3534	4979	5306	4674	3738	4284	3390	4319
4546	4445	4119	4525	5217	4176	3350	3987	3163	4115
4343	4446	4558	3208	4605	4335	3895	2813	2069	4376
4221	4477	4752	4485	4366	3878	3863	2311	3639	3841
4142	3750	3998	4457	4416	4120	3930	3084	3123	3753
4436	2820	4319	4729	4477	3612	3449	3660	2734	3603
3673	3764	3382	4547	4416	2828	3284	3403	2043	3831
4172	3028	3591	4993	3890	4498	3436	3191	2027	4792
3405	4256	3696	4287	4446	4371	2437	3306	2289	3921
4348	4118	3018	3305	3408	3088	3172	3320	3067	3395
4336	3370	2580	3246	2856	2664	3042	3387	3039	3235
4109	3483	4075	4411	3474	2882	2437	3544	2759	2140
4741	2987	4037	4946	4634	2549	2012	3224	2364	3228
4424	2986	4028	4166	3576	3582	2154	3257	2031	1881
4215	4063	4055	4095	3663	4258	3356	3000	2180	1962
4183	5000	3847	2988	2866	4197	2513	3082	3628	3265
3723	4541	2809	2806	4300	3784	2395	3094	3587	3448
4012	4206	3462	2766	4342	4213	2286	2449	2281	2950
4056	4055	3040	2982	4279	4571	3467	2239	2177	3474
3670	3894	3304	4405	2978	3103	3360	2761	2346	2235
3491	3507	3468	3250	3507	3794	3205	4465	2921	1970
3549	3876	2579	2934	**3291**	**3583**	3137	3055	3012	2161
3275	3537	2599	4092	**3003**	**2821**	2035	2178	2319	3384
3527	2731	2873	4425	**4461**	**2902**	2174	2064	2881	2783
2997	2673	3207	4075	**4760**	**2843**	1968	2300	2457	1996
3618	3262	4123	3862	**4036**	**2669**	1915	3043	2081	3965
3282	3831	4272	3020	**4246**	**2781**	2812	2488	2050	2978
3518	4167	4123	2973	**3417**	**3244**	2076	3820	1982	2443
3469	4243	3515	4247	**3042**	**4096**	2201	3368	2047	2553
3346	3550	3789	4657	3436	3760	1970	2478	1779	2179
3197	2777	3510	4467	3094	2702	2053	2042	1586	2934
3418	3665	3574	3947	4084	3865	2264	1907	3150	2993
3969	3713	2806	3831	3999	3533	1986	2679	2936	1980
3119	2727	2461	3849	3038	2817	3742	2142	1875	1780
3034	2864	2395	3214	2878	2458	3613	1884	1658	1987
3248	3804	3521	3213	2658	3376	2852	1968	2272	2635
2891	2687	2543	3292	3821	3251	2145	3471	2043	3057
2973	2837	2640	3058	2908	2772	2138	3099	1586	2071
3642	3649	2572	4213	2619	2739	3392	2100	2150	1883
3291	3018	2449	3509	4223	3370	3147	2137	1641	2099
3103	2743	3276	3181	3600	2812	2800	2102	1794	2934
2923	3214	3428	3008	2902	3126	2167	2837	1782	2753
2850	2604	2550	4123	2957	2657	2101	2281	1611	2002
3150	2529	2640	3917	2816	2889	3534	2188	2880	1970
3018	2726	2482	3035	3486	4290	2475	2048	3566	1885
3029	3625	3385	3041	3034	3333	2271	2038	3255	3158
3314	2833	2745	3240	3598	3096	3187	3191	2234	3009
2840	2750	2505	2962	3616	2946	2260	2444	1952	2151
3314	3096	2580	3670	2764	2419	2141	2185	1814	1909
2900	3845	3079	2981	3559	2527	2213	2756	2082	2034
2659	3345	2512	2998	3126	2587	1289	3352	2376	1981
2752	2608	3757	2981	2915	2594		2707	2098	2852
2447	2623	3670	3924	2960	4123		3039	1921	3319
3323	3930	2828	4410	3693	2959		2637	1861	2163
2496	4067	2569	3623	3111	2767		2266	1960	2003
2542	3119	2460	2933	2916	2800		2208	2839	2058
3701	2816	2547	4085	3414	2883		2080	2554	2552
4261	4064	3639	4387	4758	3039		2882	1685	2739
3229	3528	3417	3630	4179	3733		3665	1847	2012
2673	2882	2638	3211	3143	3823		2560	1718	1675
2715	3627	2588	3047	3021	2706		2273	1962	2757
3095	3265	3770	4416	4317	3849		2436	3424	1873
2957	2865	3047	3632	4160	3188		3248	3616	2052
3422	3971	2775	3327	3335	3065		3423	2458	3337
3273	3322	4016	3806	3343	4316		4547	2454	2481
3815	4772	4010	4706	4404	4110		3954	3473	4331
4346	4614	4241	4485	4640	4275		4312	3522	3784
3267	4783	3708	4448	4765	4559		3843	3240	3844
3479	4296	3084	4876	4357	4306		3193	2897	3474
3569	4250	4802	4325	4117	3937		4192	1703	3797
3384	3719	4134	4739	3649	3592		3241	1840	3394
4110	4412	3729	4758	4854	4130		3116	3193	3147
4151	3584	2817	4584	4214	4960		3580	3070	2965
3696	2972	3944	4407	4389	4324		2405	2976	2537
3906	3572	3989	3331	3884	3311		2023	2947	3148
2964	3347	2779	2920	3254	3008		2150	2755	1991
3266	4013	2473	3043	4121	3557		2494	2766	1587
2842	4129	3849	5016	3646	3267		3898	2919	2353
4051	3629	4477	4474	3877	3025		3324	2820	2885
4053	3849	3391	3204	4748	3354		3000	2327	1916
3181	3065	2995	3190	4226	3862		3182	1590	1638
3225	2905	2782	2995	4285	3106		3598	1605	3773
2835	2855	3250	3218	3805	3127		2945	1561	3213
4049	4365	4157	3140	4271	4504		2845	1602	2848
4056	4180	4199	4700	4256	4136		2522	1647	3518
4104	3812	3920	4870	3687	4024		1891	1594	3332
3847	3018	3344	4834	3832	3538		1942	2665	2547
3607	2936	2795	4733	3976	3106		2134	2878	2069
2827	2519	2577	4294	3427	3159		2612	1908	1645
3200	4071	2774	4471	4321	3643		1922	1424	2462
3211	3898	3068	4284	3428	3350		1864	1521	1855
2853	2895	2522	4057	4187	3095		4086	1639	1619
3859	3730	3685	4059	3664	3274		3563	1513	1759
3899	4441	3859	3503	3420	3074		2739	1458	3302
3681	3687	3686	3202	3676	3177		2604	2998	3120
3592	3815	3528	3292	3621	3106		2001	2904	3349
3578	4064	3785	4151	4275	2738		1944	2331	2872
3365	3275	3592	3877	4226	3114		2001	1333	1677
3123	2676	2795	3202	3904	2845		2424	2007	1401
2762	2630	2887	3355	3660	2970		2690	1561	1515
3508	2614	2753	3317	3373	3957		2682	1522	1584
3420	3661	2481	3275	3650	3912		2033	1595	3330
3572	2786	3345	3115	2998	2767		2794	3025	3017
2840	3756	2543	4196	3095	2925		3802	3863	2502
2890	2761	2730	3469	3025	2719		2821	3147	1777
2732	2700	2843	2931	3804	2810		2314	1877	1614
2893	2608	2662	3739	2908	2804		2329	1854	2402
2945	3071	2879	3301	2901	3429		2381	1716	2448
2934	2342	3368	3183	3079	2945		3136	2146	1966
3220	2688	2976	3007	3625	2735		1999	2445	1869
2661	3347	2525	3125	3705	2781		2040	1829	1881

3928	3646	4378	4134	3129	3730 .		4030	3379	3191
3793	3545	4081	3784	3309	3187 .		3624	3342	2525
3875	3241	4052	4175	3004	3448 .		3013	3713	3201
3628	3011	3945	3957	3686	3215 .		2833	2520	2470
3600	2937	3656	3620	3893	3165 .		3598	2289	2930
3193	3550	3755	3902	3296	3030 .		2804	2901	2415
3173	3136	3320	3345	3236	3249 .		2780	2122	2442
3320	3385	3019	3386	3499	3516 .		2536	2468	2895

2766	3829	3925	2135	2265	3591	4042	3753	3640	3290	3749	3082
2595	3482	3914	2160	2065	2778	3571	2894	2575	3588	2946	2972
2734	3792	2922	2742	2044	2596	2509	2417	2623	3030	3133	3008
3525	3054	2894	3487	3004	2682	2575	3441	2635	3540	2787	4064
3429	3091	2856	2436	2728	2706	4268	3374	3894	3176	3128	3330
2732	3311	3823	2292	2551	3445	3050	2725	3060	3977	3452	3253
2785	2996	4101	2465	2359	2642	2167	2824	2600	4513	3141	4064
3433	3299	4897	3308	3808	3722	2033	2889	2737	3566	3063	4366
4374	4129	4826	2845	4113	4114	2168	3998	4090	3510	3423	4042
4642	4372	5201	3649	3307	4130	3327	3233	3843	4713	4244	4683
4709	4719	4401	3803	3067	4004	2593	3207	3854	4364	4221	4394
4299	4145	4198	3299	3553	3958	3822	3680	4415	5181	3730	3642
4518	3043	4138	3136	3278	4436	3901	3298	4259	4561	3812	3609
4335	3985	4472	2973	3056	3632	3464	2554	3755	4379	3569	3471
4091	3924	3808	2210	1941	3201	3591	3548	3191	3980	4293	3506
4403	3846	4245	3001	1839	3511	3605	3264	3387	4318	3694	3904
4710	3756	4009	3767	3579	3704	4056	3309	3876	3994	3796	3632
4641	3442	4889	3956	3506	3753	3671	2841	3927	4212	4044	3180
4377	2755	3609	3479	3109	3266	2334	3139	3077	4510	3827	3260
4567	2644	2933	2265	2098	3581	2395	3739	3400	4845	3659	4330
4045	2831	2879	2123	1920	3343	2131	3588	3748	4760	3839	4707
2913	3065	2828	2243	2944	3181	2113	3556	3702	4909	4033	3696
2851	3056	4362	2192	2286	2973	2432	3454	3930	5107	3988	3855
3048	2629	4969	3508	1875	2984	3645	3469	3298	4090	4168	3593
2841	3494	4435	3922	2352	3618	3466	3381	3228	4838	4050	4692
3289	2689	4588 .		3632	2964	3388	3337	4127	4654	3731	4393
2890	2655	4297 .		3187	3774	3707	2755	4017	4319	3714	3354
2653	2619	3964 .		2767	3989	3333	2248	3752	4688	3168	3524
2604	2542	2794 .		3159	2932	3276	2391	3507	4192	3307	3723
2370	2594	3070 .		2600	3297	3141	2862	3750	4055	3158	3417
2915	**2600**	**2885** .		1936	3240	2755	3454	4118	3830	3300	4331
4360	**3935**	**2838** .		1991	2820	1901	3209	3717	3880	3316	3417
4447	**3543**	**4220** .		1908	3010	1841	3167	2790	4325	3729	3569
4293	**2534**	**4316** .		1956	3216	1907	2506	4045	3759	3586	3305
4802	**2517**	**3271** .		3489	3397	3359	2913	3253	4082	3654	3342
4373	**3653**	**3078** .		3138	3202	2985	2908	2959	3584	3431	3426
3999	**3573**	**3048** .		2549	3265	2175	2836	2670	3606	3320	3457
3771	**3515**	**4364** .		2094	3097	2123	3672	2887	3671	3112	3676
3310	3285	4296 .		1836	2676	2191	3443	4087	4097	3444	3443
3847	3803	4340 .		1581	2626	2141	3480	3742	3301	3872	3181
3989	3496	3162 .		3417	3463	3394	3226	3640	4461	3809	4044
3187	2895	2859 .		2945	2953	2862	3019	2596	4477	3245	4306
2614	2577	3103 .		3157	2483	2000	2934	2673	3843	3152	3739
2711	3330	2611 .		1995	2345	1987	2560	2622	3431	3400	3391
3469	3726	3081 .		2098	3080	3615	2458	2719	3652	3116	3107
2581	3044	3538 .		1920	2614	3469	3439	3010	3425	3041	3617
2513	2253	2898 .		2499	2663	2647	2640	2543	3296	2711	3317
3305	3053	2738 .		1916	2558	1967	2506	2805	3950	3536	3092
2390	2500	2720 .		1898	2613	2103	2300	2979	3151	3627	3313
2446	2477	3133 .		2963	3174	2856	2454	2624	3396	2861	3578
4225	2793	4357 .		2964	2609	2284	3160	4091	2986	2894	3200
3201	2436	4135 .		2450	2389	3200	2425	3432	3028	2549	2812
2699	3734	3654 .		1931	3321	2966	2503	2649	3962	3304	2845
2542	3105	3052 .		1864	3537	2488	3126	2812	3196	3414	2712
2541	2406	3407 .		2014	2651	2582	2788	2451	3727	2875	2859
3290	2712	3040 .		2506	2532	2966	2607	2490	3170	2785	3097
3418	2435	3157 .		1952	2566	2158	2534	2749	3246	3464	2858
2467	2820	3042 .		1989	2612	2080	3320	2725	3445	2788	2793
2475	2575	2828 .		1979	2863	2995	2531	2877	3155	3166	2715
2337	2674	3478 .		3508	2530	3542	2598	2753	3739	2798	2968
3508	3163	4120 .		3512	2479	2341	2986	2610	3945	3145	2749
2614	3029	4581 .		2763	2288	2645	2621	2501	3492	2750	2625
2648	2232	4062 .		2333	3393	2335	2480	2497	2852	3006	2867
3997	2457	3442 .		2278	3308	3107	3603	3980	3870	2809	3421
3584	2469	2837 .		2259	3031	2142	3522	3211	3476	2982	3664
2675	2425	2624 .		2079	2616	2138	2490	2718	3381	2763	2980
2477	3650	2940 .		3603	2793	2888	2514	2865	3737	3691	2688
2708	2539	3704 .		3556	2511	3672	2540	2566	3267	2755	2570
3077	3095	2896 .		2242	3455	3518	3304	4048	3389	3414	3045
3725	3450	2849 .		2176	2555	2454	3070	3467	2990	3390	2653
2908	3747	4148 .		2173	2381	3533	2632	2752	3240	3012	2657
2949	3979	3153 .		3241	3479	3304	2479	3201	3759	2885	2698
3101	2971	3960	2733	3990	2924	2820	2505	3129	3514	3009	3357
4107	2745	3745	4384	4034	2509	2476	3652	4454	3188	2896	3156
4808	3873	4784	4695	3663	3620	2617	3570	3898	4284	3940	3247
4564	3914	4381	3995	3266	3764	4168	2766	4331	4610	4322	4245
4971	4512	4675	2652	2392	3481	4397	3785	3588	4835	3932	3835
4159	3670	4440	3346	3213	3340	3683	3533	3713	4364	3422	3669
4144	4541	4477	3050	3171	3624	2658	2782	3716	3664	4347	3128
3261	3896	3924	1971	2112	3241	3478	3472	4418	4576	4010	3180
3403	4039	4040	1599	2022	2597	3638	3965	4116	4531	4356	2734
4845	2902	4298	3585	2371	2280	3012	3439	3858	3927	3063	2988
4813	3243	4114	3842	2186	2781	2475	2698	3542	4265	3899	4156
4423	3027	3172	2335	3210	3701	3701	2490	3403	3504	3129	3223
4250	2508	3306	2200	3439	3630	3819	2393	3792	4018	2998	2855
4509	3145	2891	2291	3180	3075	3055	3638	3777	4576	3259	3064
4192	3209	2890	3259	3812	3104	3004	3647	3283	4319	4025	3211
4241	2515	2918	4489	3066	3679	2736	3411	3694	3562	3273	3422
4395	2682	4571	3920	3064	2938	2372	3685	4145	3731	3931	3902
3023	2576	4376	2414	2585	2974	2169	3409	4144	4943	3662	3978
3055	4209	3264	2168	2265	2944	2646	3138	4158	4470	3732	4546
4079	3777	2822	1949	2048	2987	3115	3367	4068	4208	3337	4285
3426	3514	2895	1947	2030	2277	2473	2967	3786	4078	2709	4088
4346	3759	2954	2864	2137	2342	2851	3408	3507	3376	2411	3374
4703	3499	3970	3605	3041	1919	2886	3185	3048	2820	2476	2860
4258	3246	4057	3396	2133	2849	3349	3250	2994	3046	3328	2747
3697	2834	3921	3063	2450	2411	2919	3050	3022	2732	2677	2694
4019	2821	3560	1990	3573	2361	2094	2694	3334	3271	2394	2616
3477	2878	4118	1967	2884	2316	2668	2444	3184	3253	2839	2824
2896	2679	3031	1942	1877	3054	3896	3296	3772	3870	2507	3058
3379	2626	2978	2823	1893	3036	3320	2482	4027	3367	2471	2865
2749	2685	3191	3052	1855	3518	2300	2923	3386	3875	2566	2768
2659	3922	2677	2244	2362	3113	2398	3827	4047	2907	2210	2704
3922	3110	2869	1941	3368	2878	2257	2969	3105	2654	2455	3475
3739	2576	3527	1805	2884	2776	2913	2939	2810	3162	3577	3345
3940	2367	2760	1840	2695	3286	3748	2842	2649	3748	3324	3363
3480	2607	2775	3274	2394	2619	3017	2298	3565	4459	3329	3163
3428	3487	3086	2810	1894	2068	2546	2230	3766	3120	3587	3112
2565	3586	3326	2625	1906	2142	2323	2177	2661	2684	3227	2573
2461	3078	4114	2136	2194	3333	3141	2110	2812	2919	3495	2621
2725	2523	4051	1806	2872	3094	2545	2110	3678	3725	3395	4278
2983	2314	3505	1925	1692	2487	2880	3446	2945	2956	3141	3841
2628	3942	2913	3305	2091	2459	2160	2828	3050	3752	2463	3073
2386	2760	3056	2914	3166	2500	2542	2496	3551	2939	3004	2760
3607	2502	2902	2110	2053	2301	1989	2200	3118	2936	3180	2572
2877	3110	2920	2115	2685	2648	2178	3213	3171	2768	2444	3022
2620	2730	3173	2112	2263	2503	2168	2179	2871	2952	2710	2600
2564	2458	3126	2680	2073	2194	2007	2391	3685	3435	2458	2436
3186	2774	3918	1987	2037	3104	3457	2759	2680	2804	2563	3115
2848	2338	3263	1856	2222	2837	2778	2269	2808	2667	2364	2983

3955	2968	3025	4111	3236	3245	4073	4004	3756	4321	3016	3440
3615	3396	3400	4051	3435	3140	4147	3993	3717	4056	3298	3246
3995	2721	3070	3654	2902	3661	3701	3238	3486	4268	3781	4501
3808	2715	3171	3579	2769	3345	3538	2973	3397	4121	3061	4449
3543	2641	2974	2777	3406	3252	3120	2750	3204	4314	2959	3993
3220	2934	2961	2614	2914	2979	3265	3503	3339	4049	3527	3307
3446	2975	3160	2635	2675	2964	3057	2926	3344	3881	2973	3155
3067	2775	4449	2992	3102	3372	2620	3153	3192	3383	3127	3275

	3146	3364	3477	4198	3313	3455 .	2810	2593	2518	3221	2737	3406	3117	2905	2885	3036	2438	3177	3295	3013	3540
	3189	3218	3291	3371	3108	3244 .	2468	3078	2311	3361	3234	3320	2601	2873	2751	2781	2719	3108	3131	3084	3705
	2976	3396	3654	3673	3879	3343 .	2756	2403	2624	3347	2786	3310	2496	3333	3030	2835	2951	3372	3380	2999	3968
60kcal%	3320	3689	3462	3815	3372	3295 .	3196	2668	3741	3313	3011	4203	2838	3079	3382	3110	2729	3560	3692	3549	3270
	3340	4791	3922	4966	3773	4436 .	4347	2421	4110	4295	3999	3259	2922	3346	5235	2929	3017	3444	3400	3789	3163
	4130	4682	5077	4258	4269	4367 .	3538	3501	4007	4030	3408	3486	3631	3896	4497	3859	2846	3851	3166	3326	4595
	4449	4784	5098	4345	4888	4302 .	3694	3322	3664	3877	4233	5056	2760	2998	4088	4432	3631	3872	3390	4192	4496
	4892	4692	4664	3683	4599	3710 .	3377	3120	3588	3568	4133	4096	3701	3545	3515	4219	3041	3529	4098	4811	4143
	4464	4322	4587	4430	4578	3565 .	3201	3284	3886	3372	4315	4222	3568	3079	3711	3873	3422	3575	3294	4637	3566
	3404	4609	4553	3655	4529	3355 .	2986	2894	3628	4319	4254	4310	3530	3515	3685	3873	3259	3538	3254	4072	3622
	3342	4108	4414	4409	3668	4217 .	3104	3366	3685	4002	3549	4391	4213	3267	3245	3881	3014	3683	3812	4220	4770
	3342	3750	4408	3719	3901	4013 .	2672	2859	3632	3542	3702	4276	3760	3294	2917	3380	2764	3617	3815	4238	4045
	3105	3679	4275	3577	3528	3443 .	2591	2618	3646	3078	2991	3895	3810	3049	3499	2554	3722	3112	4129	4226	4014
	3134	2959	3985	3701	3431	3542 .	3055	1996	3624	3270	3464	4187	3502	2772	3162	3609	3831	3587	4339	4025	3139
	2958	3487	3369	3575	4252	3202 .	3593	2051	3127	3341	3428	4194	2917	2765	3519	2859	4070	3508	3726	3190	3040
	4309	3218	3493	3394	4435	3732 .	3521	1984	2279	4546	2772	3421	3158	3597	3069	3410	3546	3565	3290	3477	3136
	4577	3302	3614	3891	4166	3883 .	2625	3051	2611	4009	2784	3105	2538	3653	3184	3582	3775	3857	3452	3307	3193
	4132	3122	3376	4787	4016	3954 .	2610	2443	2487	4395	3614	3226	2725	2944	2880	2711	3746	4651	4266	2859	4012
	4050	3543	3485	4108	3905	3904 .	4249	1985	2330	4171	2702	3604	2922	3238	3893	2624	3711	3944	4390	3045	3489
	3192	3934	4778	4298	4094	4191 .	3301	2314	3476	4301	2778	3228	2668	3159	3645	2541	3946	3881	4601	3365	3428
	4434	3987	4426	4225	4061	3891 .	3722	3528	3585	4216	2650	3135	2590	2579	3999	3520	3475	4029	4583	3690	3707
	3931	3713	3391	4721	3797	3297 .	3213	3140	3513	4477	3513	3494	3636	3161	3457	3664	3154	4120	4352	3052	3273
	3714	3084	3741	4121	3350	3456 .	2883	2566	3356	4106	2956	3196	3592	3532	3348	3648	3101	3907	4795	3503	3273
	3910	3939	4192	3663	3014	3640 .	2633	3615	3267	3503	2919	4429	3326	3181	3454	2779	2944	3717	3878	3517	3051
	3989	4518	4542	4338	2932	4136 .	2448	2964	3110	3556	3133	3866	3227	2921	2970	2435	2952	3968	4824	4421	3788
	3582	3477	4143	4256	2843	3970 .	2399	2065	2356	3415	3485	3972	2493	2515	3108	2588	2878	3456	4071	4229	4491
	3328	2950	4317	3569	3567	3666 .	2474	2177	2774	3496	3644	3624	3031	2687	3170	2516	3185	3361	3814	4204	4472
	3202	2938	4216	3496	3448	3816 .	2683	2099	2561	3276	3925	2864	2714	2835	2923	2322	3073	3692	3280	4366	4421
	2997	2759	3925	3453	2954	3446 .	3049	1928	2568	3434	3596	3160	2410	3415	2669	2519	2917	3832	3112	3838	3922
	3590	3087	3368	3556	3715	3819 .	2355	2352	2126	4167	3574	4287	2687	2477	2708	2554	3314	3366	3568	4095	4366
	3164	2905	3792	3563	2992	3430 .	2316	2039	2126	3648	3658	3874	2569	3271	2662	3318	3747	3823	3218	3979	4256
	3118	2740	3734	4052	2978	3888 .	3295	2090	2171	3941	3186	3633	2667	2994	2933	3711	3284	3567	3478	3252	3960
	3195	4009	4516	4502	3226	3888 .	3334	3376	3427	3791	2915	3013	2991	2611	3797	4063	3187	4001	3973	2857	3956
	4229	4040	4174	3584	4443	3168 .	2989	3151	3370	3396	3433	2864	3322	2562	3456	3568	2654	3578	3801	3207	3670
	3701	3711	3948	3215	4381	3357 .	2447	2858	3386	3408	3067	3171	3595	2549	2923	2287	3727	3407	3124	2956	3217
	3340	4045	3586	4709	4048	3434 .	2673	2638	3523	3082	2600	3739	3364	2517	2922	2865	3679	3336	3099	3872	3541
	3575	3760	4042	3745	3917	3891 .	3243	2176	3251	2975	2407	3702	4030	3232	3464	3850	3878	3885	2862	2991	3443
	3475	3882	3899	3405	4174	3884 .	2486	1696	2913	4272	3632	3375	3512	3021	3008	3524	3266	3462	3804	2569	4100
	3233	3286	3484	3449	3671	3200 .	2284	2739	2353	3258	3760	2950	2954	2529	2626	3540	3408	3323	3987	3346	3703
	2842	3056	3126	3424	3423	3143 .	2288	2206	1951	3105	3100	2900	2613	2509	2516	3218	2715	3166	3176	3035	3233
	3398	2962	3288	3155	3275	2921 .	2286	2001	2723	2873	2677	2702	2934	2368	2552	3057	2716	3358	4030	2946	3263
	2937	2821	3206	3392	3589	3018 .	3178	1984	2379	3179	2594	2991	2465	2761	2783	2557	2639	3111	3791	2814	2967
	3053	2992	3157	4026	2980	2835 .	2555	1996	1983	3103	3149	3042	2578	2962	3317	2683	2853	3025	3261	3008	3249
	2906	2714	3698	3429	4233	3048 .	2356	2498	2213	3644	2650	3119	2315	2469	2800	2808	2741	2910	2898	3182	3170
	3193	2637	3370	3158	3501	3184 .	2406	3270	3234	3010	2638	3442	2326	2478	2978	2817	2672	3159	3012	3221	3249
	2948	2994	3165	3098	4078	3449 .	2833	2824	3229	3185	2935	3139	2514	2890	2800	2339	3117	3077	2939	3236	4421
	3004	3269	3764	3754	3345	3205 .	3121	2626	2687	3509	2661	3506	3024	2503	2776	2457	2695	3572	3114	3230	3422
	3405	2988	3166	3083	3291	2902 .	3099	2373	2216	3744	2551	3006	2990	3280	3439	3630	2845	2995	3346	2857	3260
	3750	2808	3703	3310	3271	3119 .	2596	2220	2539	3221	2703	3164	2611	2614	3083	3302	2710	2917	3154	3745	4095
	3083	2827	2947	3253	3714	2855 .	2407	2123	2419	2974	2656	2993	3313	2488	2644	2929	3038	2892	3654	3761	4161
	3005	3734	3174	3593	3252	2987 .	2564	2004	2709	2990	3567	3666	2673	2303	2711	2544	2730	2836	3827	3455	3496
	3207	3414	3734	3222	3574	2914 .	3082	2739	2519	3128	3225	2979	3135	2631	3141	2646	2985	2899	3665	3210	3344
	2957	3489	3748	3419	3591	3854 .	3022	2865	2515	3652	2910	2985	3021	2644	2886	2551	3068	2888	3517	3032	3277
	2965	3296	3416	3240	3129	3489 .	2747	2795	2497	3751	2809	3067	2656	2677	2790	2534	3023	2839	3291	3135	3208
	2965	3129	3276	3287	2900	3100 .	3304	2659	2500	2865	2727	3331	2665	3115	3443	3375	3121	3277	3232	2789	3305
	2950	3014	3923	3517	3667	3082 .	2836	2644	2185	2878	2850	3304	3635	2736	3029	3062	3534	3151	2945	3072	3139
	3382	3014	3671	4230	3313	3510 .	2782	2733	2958	3171	2919	3131	2911	2630	2873	2962	3151	3064	3370	3899	3173
	2943	3706	3460	3405	3089	3529 .	2922	2988	2673	3079	2748	3869	2519	2451	3124	2705	3036	2930	3189	3306	3816
	3799	3917	3328	3484	3010	3387 .	3013	2355	2706	2886	3037	3028	2762	2836	3080	3235	2858	2914	3195	3190	3636
	2930	2995	3893	3735	2893	3031 .	2906	2217	2398	2870	2983	3120	2802	3576	3404	2904	3052	3639	3816	3732	3286
	3204	3031	3608	3774	3836	2894 .	3247	2868	2990	3240	3409	3716	2758	3008	3508	2810	4065	3017	3194	3019	3298
	3161	2955	3332	3205	3144	3047 .	2999	3189	2184	3299	3103	3247	2540	2911	3263	3015	3013	3178	3365	2875	3710
	3009	3260	3401	4029	3617	3379 .	2967	2486	2618	3277	3008	3230	4062	2584	3230	2837	2988	3417	3398	3874	3505
	3077	3231	4091	3909	3281	2965 .	2839	2109	3433	2962	2896	3298	3566	3069	3390	2751	2682	3306	3228	3459	3141
	3715	3425	3602	3497	3118	3206 .	2674	2031	3909	3596	3463	3955	3038	2938	3197	2451	2971	3178	3989	3574	3335
	3702	3738	3502	3302	3925	3200 .	2621	2773	3424	3116	3249	3968	2448	2631	3726	3692	4003	3327	4239	2955	3804
	3698	3301	3390	3439	3371	3087 .	3736	2553	2674	3203	3336	3193	3357	2890	3117	3464	2999	3088	4191	2876	4156
	3464	3268	4180	3615	3513	3977 .	2913	2298	2665	3328	3230	3250	2770	2840	3351	3403	3126	3180	3554	3027	4172
	3143	3498	3894	3388	3196	3239 .	2821	2450	3723	4279	3024	4064	2703	3402	3662	2853	3189	3580	3742	3534	3413
	3132	4517	3918	4858	3557	3283 .	3886	3069	4752	3680	3677	3323	3427	4262	4666	2815	3600	3968	3222	3177	3471
	3946	4830	5604	4509	4481	4804 .	4202	3811	4382	3395	3741	3861	3661	3975	4254	3947	3601	4113	3433	3091	3348
	4431	4499	5073	4587	5016	4602 .	4357	3543	4609	3614	4155	5068	4305	3359	4546	4293	3908	3921	3909	3979	4355
	4202	4296	4514	4352	4456	4280 .	4509	3397	4479	4558	3708	4293	3789	3830	4890	4239	3865	3901	5261	4503	4268
	3813	4475	4870	4057	4454	4312 .	3817	3475	3829	4371	3512	4292	3350	3510	3966	4112	3696	4382	4882	4516	4406
	3998	4106	4852	4035	3875	4290 .	3825	3151	3980	3515	3223	3480	3823	3309	3962	3578	3272	3960	4902	5078	4995
	3943	4141	4264	4029	4369	4082 .	3904	2940	3793	4104	3642	4357	3304	3245	3461	3795	3935	4024	4760	4431	4200
	3953	4219	3885	4007	4269	4107 .	3650	3125	3640	4295	4257	4543	3240	2527	3073	3555	4210	3993	5109	4112	4556
	4226	4135	4051	4437	4214	3914 .	3591	3376	3864	4132	3725	4278	3615	2949	3933	3241	3797	3451	4690	4198	4245
	4122	3644	4160	4434	3941	3990 .	3838	2552	3609	4513	3531	3987	3281	2645	4180	3626	4206	3360	5314	3974	3833
	3625	3239	4051	3750	4183	3551 .	3726	2115	3418	3909	3881	3190	3480	2681	3851	3306	4185	3203	5161	3458	3765
	3227	3767	4252	3526	3849	3273 .	2943	2050	3311	3888	3958	3247	2970	2700	4624	3355	3762	3955	4400	4005	3968
	3159	3685	4549	3567	4144	3214 .	3264	2133	2558	3304	3138	3325	3311	2719	3626	2691	3368	3195	4101	4323	3829
	3775	3337	4592	4288	3255	4266 .	3288	3438	2754	3073	3744	3969	2898	2498	3503	2719	3439	2902	5149	3540	4278
	4670	3730	4747	4510	3685	3725 .	3415	3148	2516	3299	3777	3418	3316	2438	3481	2896	3970	2856	5239	4656	4993
	4223	4343	4678	4551	3193	3201 .	3584	3129	3307	3368	3952	4099	3924	2445	3491	3538	3755	3556	4334	4294	4333
	4269	4086	4791	4330	3050	2651 .	3291	3066	3736	3784	3459	4198	3876	2706	3471	3520	3713	3115	4142	4080	4507
	4112	3213	4390	4242	3578	3206 .	3806	2882	4133	2896	3546	3856	2820	3887	3164	3623	3398	2894	5366	4298	4740
	4411	3440	3707	3726	3544	3822 .	3220	2078	3679	3041	3623	3881	2897	3733	2662	2780	3514	4067	4136	4088	4217
	3737	3736	4183	3722	4256	4337 .	3027	1510	3796	4076	3480	3190	3649	3528	3002	2811	3815	4115	3722	4226	3781
	3080	4016	3979	4227	3149	3930 .	2838	1619	3534	3324	3941	3235	3387	3016	3380	2765	4047	3850	3524	4008	3581
	2984	3672	3869	4260	2905	3536 .	3099	3413	3207	3030	3849	4546	3340	3385	2966	2714	3751	3988	3831	3271	3467
	3299	3049	3963	4362	3253	3562 .	2932	2749	3098	4176	3669	3914	3343	3262	3982	2977	3440	3891	3456	3015	3475
	3372	2904	4064	4058	3004	3433 .	3225	2615	2914	4528	3466	3166	3849	2906	3865	2512	3027	4048	4079	3523	3319
	3951	3497	4308	4004	3200	3177 .	3514	1822	2795	4114	3377	3573	3327	2393	3523	4365	3866	3407	3601	3014	3692
	4284	3193	4332	3783	4597	2924 .	3309	2119	2750	4320	3686	3513	2919	2096	3761	4545	3721	3724	3597	4114	4260
	3464	4258	3996	4337	4345	3561 .	3172	1937	3416	4191	3454	3903	3384	2247	3717	3966	3881	3085	3750	3226	3660
	3049	4238	3515	3739	4245	2880 .	3503	3133	2917	3914	3323	3259	3269	2367	3854	3904	3192	2734	5047	3071	3991
	3110	3623	3963	4613	3909	3137 .	3120	3098	3227	3937	3312	3229	3571	3409	3432	3585	2989	3383	4724	3834	4099
	3849	3417	4002	4401	3895	3084 .	3194	3248	3832	3836	3668	3571	2854	3273	3497	3607	3063	3833	4298	4434	4154
	3592	3952	3869	4043	3705	3818 .	2639	2866	3232	4192	3343	3345	3095	2910	2863	3302	3439	3477	4270	4128	3989
	3679	3610	3698	3547	3891	3558 .	2777	2608	3073	3503	3537	3147	2861	1940	2519	3046	3217	2885	3627	3223	3365
	3865	3541	4316	3444	3154	3599 .	2850	3351	3158	3817	3136	3669	3568	2249	3237	2504	3535	3570	3236	3305	4121
	3324	3343	4594	3761	4617	3272 .	2582	2912	2715	3739	3637	3396	3780	2811	2774	2967	2977	3187	2992	4242	4660
	2964	3017	4092	3177	3529	2909 .	2258	2654	2495	3684	3763	2856	3363	2087	3117	2782	2924	2879	3358	4243	4180
	3072	3048	4060	3118	2996	2916 .	3060	1973	2684	3376	3195	2969	2919	2475	3793	2760	3314	3090	3014	3867	4436
	2983	2798	3721	2920	2705	2617 .	2605	1972	2498	2907	3272	2921	2901	2379	2785	2511	3142	2613	3186	3954	3931
	2965	2816	3474	3456	3637	3401 .	2774	1942	2749	2680	3005	3475	3098	2792	2881	3281	2904	2648	3844	3219	3656
	3012	2924	3228	3292	2901	3064 .	2322	2265	3034	3135	2897	2973	2710	3542	2741	3281	2976	2686	3948	2762	3572
	3279	2963	3081	3798	3237	2881 .	2343	2012	2766	2844	2879	3055	2776	2869	2668	2773	2766	2721	2929	2979	3431
	2756	3758	3427	3504	2975	2781 .	2567	1830	2607	2725	2899	3700	3230	2472	2536	3054	2901	2795	3173	3510	2911
	3009	3337	3549	3320	2695	3123 .	2659	2619	2519	2758	2811	2799	2642	2498	2686	2600	3195	2663	3160	3272	3858
	2778	3144	3692	3244	3827	3302 .	3145	2243	2333	2800	2724	2811	2929	2406	3025	2580	2980	2689	3806	3047	3491
	2970	2848	3188	3404	3269	3658 .	2831	2989	2399	2731	2858	3068	3162	2353	3736	3493	2965	2936	3191	3100	3497
	3033	2869	3105	3226	3533	3553 .	3074	2655	2795	4055	2963	2928	2636	3091	3087	3449	2718	2729	3233	3304	3069
	2760	2830	3432	3190	3076	3114 .	3275	2675	2349	3406	3216	3451	2858	3042	2990	2945	2982	3186	3194	3652	3516
	3775	3196	3341	2887	3966	2875 .	2732	2627	2168	2940	2949	2918	2887	2792	2814	3134	3490	2916	3311	3069	3500
	3707	3626	3500	2887	3364	2936 .	2693	2323	2991	2776	3376	3819	2469	3206	2876	2733	2910	2677	3290	3327	3649
	2949	3439	3338	3173	3716	3411 .	2544	2232	2882	2687	2883	3610	2443	2681	3290	2767	2768	2710	3345	3112	3914
	2935	3245	3479	3062	3295	2980 .	2396	2134	2619	3034	2876	3295	2203	2469	2908	2636	2801	2736	3274	3144	3784
	3394	3281	3641	3133	3637	3175 .	3362	2093	2860	2645	2821	2978	3218	2519	3036	3048	3032	2917	3501	3552	3364
	3152	3048	4236	3752	3132	3011 .	2574	3080	2349	3186	2734	3229	3160	2728	2947	2578	2989	3064	3713	3281	3279
	2988	3058	3403	3218	3115	3436 .	2376	2896	2406	2881	3758	2951	2710	2655	3263	2571	2936	2952	3413	3280	3178
	3105	2935	3324	3714	3711	2947 .	2394	2330	2580	2957	3119	3149	2621	2414	3092	3448	3108	2798	3328	3759	3246
	3169	3142	3318	3133	3555	2948 .	2906	2319	2703	2889	3117	3098	3135	2560	2787	3066	3223	2773	3258	3315	3240
	3156	3282	3315	3332	3335	3364 .	2369	2125	2618	2913	2936	3165	2529	2898	3729	3037	3143	3323	3384	3147	3463
	3006	3008	3930	3344	3192	3095 .	3180	2158	2981	2868	2658	3089	2724	2561	3373	2832	2854	3195	3293	2987	3710
	3150	3688	3646	3140	3040	3405 .	2451	2732	3553	3282	3184	3226	2551	3099	3373	2692	2999	2939	3603	3258	3677
	2994	3543	3387	3363	3200	3325 .	2675	2060	3071	2894	3411	3294	2690	2707	3073	2880	2923	2688	4115	3152	3509
	3912	3218	3614	3106	3003	3302 .	2907	2299	3026	2846	2954	2961	3790	2494	3300	3775	3503	2644	3666	3065	3741

3308	3208	3344	3120	3251	3092 .	2786	2894	2495
3225	3381	3351	3184	2976	3067 .	2532	2739	2647
3312	3629	4155	3810	3466	3128 .	3050	2520	2353
3538	3899	3701	3435	4335	3346 .	2787	2565	2737
3185	3784	3731	3103	3769	3230 .	3013	2516	3195
3382	4511	3774	4011	3281	3320 .	3626	2478	4821
3426	4497	5022	4566	3493	4229 .	4410	3542	5079
4610	4335	5086	4467	5104	4407 .	3843	3914	5116
4883	4477	5985	4310	4365	4688 .	3866	3478	5334
4332	4174	4588	4049	4549	4128 .	3775	3355	5189
3934	4014	4465	4244	4480	4090 .	3598	3549	4777
3901	3560	4769	4001	3530	4189 .	3277	3165	4945
4452	3430	4289	3753	3489	3852 .	3163	2421	4801
4158	3808	4202	3693	4045	3714 .	3209	2384	4821
3481	3454	3872	3739	4347	3657 .	3276	3169	3925
4326	3286	3368	3434	4247	3806 .	2781	3187	3694
3905	3287	3500	4641	3479	3162 .	2216	2375	3422
3419	3131	3932	4522	3023	3212 .	2428	2343	3048
4232	3301	4143	4171	4473	3357 .	2719	3265	2906
4193	3815	3428	3754	4216	3892 .	3693	3432	3714
3622	4447	3886	3384	4202	2989 .	2889	3385	4567
4157	3952	3689	3000	3258	3338 .	2472	3141	4654
4175	3380	4195	3111	2930	4286 .	2396	3431	4167
3944	3443	5073	4270	3271	4147 .	2373	2895	3107
4197	3276	4771	3978	3497	3992 .	3662	2987	3804
3595	3588	4242	4462	2981	3809 .	3251	3058	3934
3733	3413	4478	4229	3029	3895 .	3024	2503	4123
4177	3997	4342	3959	3159	3359 .	2989	3217	4523
3822	3409	4421	3706	4615	3430 .	2265	2791	3668
4063	3331	3912	3782	4462	2867 .	2562	2739	2790
3654	3478	3292	4000	4603	3048 .	2072	3303	3654
3743	3317	3624	3186	4784	2910 .	2056	2654	3932
3975	3788	3936	3810	4061	3202 .	2439	3060	3658
3713	4001	3648	3233	3884	3166 .	2755	2796	3146
3193	3699	4794	3172	4043	3403 .	3479	2728	3887
3244	3456	4028	4257	3655	3167 .	2866	2809	3953
3262	3217	3881	4248	3808	4018 .	3008	2313	3132
3997	2802	4580	4283	2751	4100 .	2888	2247	3273
3649	2822	4858	3749	3033	4040 .	2576	2372	3103
3192	3310	4022	3323	4003	3691 .	2024	2225	2640
3027	2977	3364	3029	2971	3024 .	2861	1946	2390
3049	3084	3490	3069	3034	2897 .	2117	2552	2373
3101	3021	3415	3430	2696	2914 .	2376	2600	2732
2866	3036	3441	2982	3001	2910 .	2216	2320	2547
3226	2851	3584	3100	2971	2897 .	3143	2354	2458
3447	2931	3233	3080	3789	2988 .	2876	2881	2563
2947	3473	3120	3277	3466	3103 .	2388	1875	2308
3160	3077	3107	2894	3179	2819 .	2488	2350	3524
3011	2717	3932	3205	3820	3103 .	2179	3114	3288
2828	2732	3913	2825	2995	3344 .	2860	2285	2558
2962	2992	3194	2887	2805	3349 .	2669	2404	2218
3670	2673	3409	3436	3053	2957 .	2567	2273	2438
2982	2760	3358	3206	3323	3058 .	1928	1954	2305
3122	3242	3398	3067	3436	2901 .	2252	2334	2806
3418	2748	3592	3389	2817	3206 .	2338	2852	3283
3011	3704	3400	3385	3109	3503	2944	2336	2970
2971	3323	3683	3087	2942	2904	2854	2079	2741
2965	3001	3420	3112	3233	2802	2648	2227	2656
3035	3024	3734	3158	2851	3325	2773	2707	2547
4150	2984	3256	3712	2989	3043	3079	2886	2424
3700	4166	4290	4500	2779	3167	2480	2264	2351
2954	3169	3366	3654	3347	3470	2428	2060	3042
3311	2932	3195	3971	3472	2935	2686	1998	2507
3292	3425	3644	3686	3247	3137	2131	2330	2334
3175	3090	3382	3231	3084	2877	3359	2082	2606
2975	3011	3420	3572	3269	3067	3834	3286	2690
2986	2895	3624	3630	3089	3489	3078	3315	2582
3121	3183	3611	3673	4338	3142	3238	3177	3035
2952	3710	3396	3708	3855	3241	3058	2570	3164
3387	2957	3898	3389	3069	3280	2959	2413	2875
3164	3924	4500	3487	3301	3700	3191	2350	3017
3317	4568	4406	4542	3577	4209	4424	3635	4486
4624	4458	5109	4528	4909	4778	4234	3710	4953
4658	4188	4935	4339	4394	5441	4059	3319	4943
4132	3917	5398	3691	3557	4417	3749	3014	4609
4042	3770	4798	4068	3313	4237	3408	2917	3861
3510	3471	4507	4002	4097	4781	2979	2873	4022
3207	4142	3654	3984	4914	4176	3226	3206	3459
4397	3946	3760	4007	4288	4644	2935	3065	3510
4356	3542	4264	3413	4028	4598	3022	2356	3498
3875	3182	4473	3552	4005	4098	2921	2374	4221
3126	2946	4035	3967	3282	3728	3199	2261	3758
2838	3010	3657	3531	3283	3441	2939	2077	3486
3537	2995	3390	3382	4268	3414	2576	2956	2894
4374	3664	3618	3338	4895	3152	2211	3469	2365
4233	4270	5928	4254	4731	3731	2617	3107	2285
4034	4149	4379	4360	4324	4113	2750	2931	2766
3149	4082	3793	3850	4343	3854	2696	2089	4047
2889	3635	3555	4193	4065	4192	3725	2118	3784
3248	3189	4226	3633	3534	3597	3473	2075	3394
3089	2559	3802	3857	4036	2959	2977	1993	3300
2970	2708	3963	3926	4340	3724	2971	1597	3289
4154	2549	3612	4194	4251	3231	2421	3377	2978
4129	4231	3896	4192	3968	3632	2917	3138	2727
4322	3897	3770	3568	3610	3346	2405	2631	2356
3803	4058	3634	3330	4126	3522	2518	2688	2306
3768	3432	4957	3377	3755	4448	2375	1810	2808
4446	3065	4150	3809	4497	3438	2445	1768	3598
3739	2622	4399	2927	3789	2736	2661	2529	3292
2923	3005	4360	3288	4569	2928	3418	3010	3527
2983	4160	4375	3939	3612	3010	3577	2898	2836
2793	3803	4276	3525	3833	4201	3509	3013	3030
2790	3575	4047	3459	3515	4385	3218	2889	2543
3841	3421	3866	3875	4031	3853	2433	3180	1957
3561	3140	3863	3472	5082	3731	2516	2997	2319
3375	3423	4086	3330	3754	3331	3021	2433	2219
3086	3089	4010	3426	4202	2895	2908	1845	2305
2905	2598	3108	3277	3251	3177	2233	1732	3380
2857	2722	3598	3302	3209	3032	3060	1740	2971
2930	2656	3647	4029	3300	3215	2813	1877	2418
3403	3465	3324	3351	2812	2866	2555	3079	2212
2868	2632	3386	3353	3280	3010	2348	2777	2342
3157	3174	3642	3311	3497	2896	2707	2115	2631
2682	2748	3646	3148	3163	3061	2709	1982	2382
2929	2640	3702	3383	3252	3336	2626	2089	3595
3025	2735	3634	3107	3507	2892	2458	1882	3002
2960	3239	3664	3455	3236	3129	2881	2514	2589
2654	3387	3599	3134	2956	3665	2435	2083	2299
2899	3293	3748	3171	3026	2985	2609	2069	3000

3117	3139	3555	3318	2638	3001	3619	3485	3318	3847	3934	4468
4071	3067	3030	2565	2558	2823	3331	2880	3170	4147	4003	3913
3629	2911	3731	2319	3369	3011	3369	2902	2863	4480	4212	3574
3145	2926	3388	2538	2993	3608	2850	2685	3122	3649	4059	3561
3205	3546	3200	2403	2873	3283	2696	2730	2803	3555	3387	3747
3122	3293	3845	3631	2783	3403	3433	2766	3448	3506	3313	3475
4495	3581	3309	4381	3993	4321	3679	3805	4227	3381	3316	3977
4640	4351	4884	3046	3622	4527	3816	3787	4006	3663	3710	4429
4304	4036	5507	2723	3446	4832	4599	4209	3982	4378	4706	4260
4314	3864	4251	3462	3305	4892	4054	3507	4188	4816	4533	4028
4147	3660	4214	4097	3490	4243	3495	3375	3642	3911	4046	4379
3414	3900	3925	3987	2707	3781	4165	3648	3937	4369	4606	5083
2996	3683	3923	3522	3530	4253	3614	3247	3685	4605	4262	4501
4264	3221	4165	3548	3317	4457	3842	3429	3370	4107	4306	4364
3561	3151	4018	3220	2479	4362	3616	3295	4105	4108	3803	4097
3386	2868	4141	2378	2799	3865	4666	3429	3634	4156	4186	4224
3250	3130	3595	3031	2471	3403	3884	3788	3312	4688	4238	4002
4049	2894	2887	3736	2180	4619	3397	3571	3348	4604	4065	4378
4510	3110	2656	3418	2226	4160	2975	3068	3103	4416	4201	4179
3647	3555	3886	3324	2861	3957	2613	3536	2979	4226	4366	4778
3850	3678	2989	2430	3887	4150	2890	2999	3028	5065	3932	4252
4673	3666	3321	2480	3348	4391	2767	2795	4201	4619	4148	4144
4251	3767	3887	3408	3254	4403	4730	2712	4167	4844	4838	3877
4195	3602	4149	4005	3636	4280	3982	3517	4097	4728	3784	4105
4086	3414	3513	3534	3066	4633	3445	3723	3924	4903	4589	4590
3803	3022	2804	3254	2923	4165	2839	3887	3856	4641	4131	4044
3170	2983	2830	2347	2609	3867	2596	3239	3372	5058	3825	3855
2888	2890	2855	2089	2188	3765	2729	3511	4046	3928	3659	4032
3071	2943	4501	2038	2110	3951	2585	3249	3477	3870	4240	3814
2914	2990	3045	2913	2779	3651	4241	3050	2678	3611	4118	4324
3749	2920	2926	3338	2772	3800	3806	2690	3066	3878	3849	3971
3319	3101	2940	2215	2301	3896	3589	2672	3368	4125	4086	3735
3579	2851	3562	2345	3385	3804	4361	2358	3150	4356	4391	3552
4299	2904	4324	2285	2244	3684	3853	2954	2909	4130	3744	3828
4137	4092	4111	2337	2552	3675	3569	3764	3191	4452	4022	4067
4354	3915	3901	3401	3454	3739	3183	3509	3573	4223	3730	4048
3566	3616	4152	3641	3446	3651	2789	3475	3365	3876	3786	3563
4133	3395	3662	3608	3073	3799	2669	2637	2596	3749	3342	3472
3838	3867	3602	3699	3110	3333	4102	2978	2888	3510	3259	3763
3725	3221	3331	3336	2737	3069	3237	2883	3334	3450	4296	4085
3327	3076	3753	3057	2793	3099	3282	2645	3094	3926	3729	3806
2700	2861	3408	2262	2349	3370	2923	2855	2815	3923	3096	3831
2792	2712	3147	2689	2460	2668	2732	3378	2860	3639	3297	3429
2650	2817	3122	2522	2904	2980	3154	3102	2752	3535	3114	3357
3704	2626	2728	2414	2427	3147	2999	3944	3036	3848	3199	3294
3025	2805	2777	2348	2465	3047	2783	3223	3203	3833	3378	3371
2873	2540	2824	2447	2594	3188	3561	2990	3122	3843	3071	3865
2876	2835	2836	3078	2911	3431	3331	2737	2874	3505	3436	3454
2821	2637	2566	2631	3167	3467	3131	3249	2912	3118	3067	3385
2755	2873	3072	2227	2931	3479	2854	3491	2905	3283	3558	3436
3175	3151	2815	2334	2718	3264	2570	2968	2680	3436	3331	3171
3374	2443	2653	3071	2423	3317	2520	2865	2666	3824	3313	3546
3786	2778	2882	2338	2686	2778	2925	2688	2773	3295	3069	3420
3624	2515	3207	2708	2672	2763	3598	2671	3109	3379	3124	3801
3182	2581	3372	2516	2431	2892	3265	2793	2751	3434	3758	3396
2937	2939	2993	2689	3094	2948	3326	2751	3373	3000	3554	3340
3163	2872	2814	2694	2987	2885	2732	2609	2706	3320	3135	3122
3231	2576	2678	2666	2642	2734	2924	2707	2724	3468	3326	3176
3117	2590	3144	2691	2737	3434	3273	2513	4041	3021	3271	3317
2863	2662	3729	2775	3007	3255	3008	2617	3273	2952	3369	3097
4120	2602	3051	2531	2574	3211	2930	2813	2867	3354	3354	3336
2970	2492	2880	3159	2447	3096	2948	2782	2742	3282	3627	3167
3114	3507	3080	3164	2769	3019	3215	3134	2805	3737	3374	3128
3589	2890	3088	2642	3764	3214	2936	3335	3278	3201	3362	3952
3369	2486	3848	2393	2929	2760	3829	3131	2870	3480	3159	3373
3117	2608	2927	3974	3018	2921	3277	2644	2852	3399	3663	3324
3146	2683	3345	3100	3273	2935	2783	2628	2824	4095	3340	3574
3467	2634	3078	3322	3002	3272	2752	2800	3536	4097	3580	3354
3831	2833	3237	2948	3342	3166	2767	2877	2955	3381	3272	3305
3164	2620	3565	2760	3593	3610	3895	2999	3113	3375	3500	3979
3653	2910	3132	2947	3862	3006	3206	2662	3037	3364	3496	3631
3637	2806	3411	2906	3311	4052	3032	3169	3786	3497	3492	3666
4959	4362	4812	3999	4297	4274	3250	3155	4873	3480	3850	3910
5092	4113	4762	3920	3748	4210	3978	4402	4113	4326	4511	4636
4318	3976	4895	3882	3693	3953	4547	3813	4353	4809	4584	4352
4593	3871	4460	3405	3895	3471	3987	3969	3870	4357	4496	4320
4600	4086	4937	3253	3177	3164	4227	3561	3821	4832	4754	4487
3961	3646	3722	3345	3023	3276	3698	3268	3598	4604	4244	3970
4587	3320	4086	2741	2778	3365	3921	3258	3403	3991	3645	3656
3857	2665	3742	3413	2734	3887	3924	3489	3409	4390	4401	3980
3606	4033	3620	3784	2981	4134	3897	3546	3300	3809	4375	4196
3473	3569	3080	2640	3437	3636	3587	2800	3822	3846	4340	3802
3474	3203	3140	2155	2510	3697	3275	3541	4105	4539	4278	3777
3532	2637	2802	2872	2615	3130	3047	2910	4323	4408	4108	4279
3536	2770	2947	2422	2465	3782	3499	3082	4059	3646	3876	4735
3348	2877	4805	2233	2543	3446	4162	2709	3901	3435	3727	4562
4174	2702	4277	2945	3793	2895	3393	3189	3817	3894	4281	4273
4645	4339	4303	3967	3308	3196	3787	2553	3354	3679	4090	3539
3559	3900	3612	3305	3278	3191	4243	3197	3002	4029	4334	3656
3390	3814	3397	3562	3289	2716	3740	3897	4228	3610	4353	4041
3035	3408	2889	3085	2505	2725	3882	3679	3766	3184	4314	4837
4704	2989	2851	2648	2271	3574	3414	3947	3519	3596	3665	4496
4514	2459	4058	2987	2252	3856	3742	3535	2981	4310	4211	4232
4559	2465	3773	1949	2244	3531	3768	3537	2872	4610	3821	4285
4173	2295	3700	1998	2191	3454	3671	3306	2910	4350	3639	4000
4225	3908	2629	3281	3059	3346	3902	3740	3046	4160	3708	3566
3090	3807	2768	2192	3303	3386	3694	3284	2835	4625	3723	4308
2934	3181	2798	2164	3530	3702	3735	2929	4076	4402	3846	4310
4321	3647	2836	2233	3114	2952	3400	2689	3623	4337	3880	3676
4141	3042	2828	3110	3270	3364	3532	2533	3648	4128	4281	4038
3953	2436	3997	2314	3120	2970	2910	3902	3409	4123	4023	3556
3950	2423	4300	3356	2313	2997	3504	3539	2920	3836	4037	4003
3672	2555	3545	3072	2280	2717	3403	3293	3428	3323	3608	3652
4012	2353	4395	2895	3309	2632	3058	2721	4071	3017	3627	3231
3726	3134	3401	2961	3159	2749	3386	2417	3404	3706	3904	4200
2823	3364	3575	2645	2673	2651	3208	2457	3000	3991	4022	4233
3635	3146	3089	2080	2479	2980	3104	2864	3165	3586	3829	3645
3126	2686	2972	2088	2218	3461	3175	2694	2994	2969	3342	3476
3288	2427	3443	1916	3180	3043	2693	2477	3057	3712	3082	3441
3593	2184	3529	1956	2971	3091	3164	2710	3123	2991	3402	3316
2912	2272	2809	3052	2405	2664	2718	2464	3193	3547	3157	3033
2917	2872	2848	2287	2474	2837	2873	3516	2928	3232	3539	3333
3024	2925	3145	2094	3085	3025	3086	3194	3001	3314	3070	3245
3411	2228	2827	2155	2854	3494	3629	2730	2864	3164	3533	3731
3187	2162	3062	2472	2460	2888	3634	2740	3041	3612	3526	4040
3162	2178	3040	1997	2447	2795	2734	2950	3060	3551	3595	3527
3142	2561	2647	2121	2726	2743	2783	2731	3607	3317	3888	3333
3249	2926	2912	2478	2442	2996	2541	2732	3160	3470	2893	3313
3887	2583	3737	2226	3087	3194	2896	3378	3339	3161	2898	3460
						3514	2982	3433	3233	2844	3320
						3210	2737	3257	4000	3389	3241
						2664	2728	3347	3294	3080	3404

12	3	3	4	4	5	7	7	8	10	10	4	4	5	8	7	7	10	10	12	12	12	1	1
Box-6	Box-1	Box-2	Box-6	Box-7	Box-6	Box-2	Box-5	Box-3	Box-1	Box-3	Box-1	Box-4	Box-5	Box-1	Box-6	Box-8	Box-7	Box-2	Box-3	Box-4	Box-7	Box-1	Box-3
83	1.7	2.3	25.3	26.6	34.6	38	43.6	52.6	64	75	13.3	21.5	32.6	44.6	45	47	65	68	77	78	84	6.1	9.4
F	F	F	F	F	F	F	F	F	F	F	F	F	F	F	F	F	F	F	F	F	F	M	M
H2O	Malto	Malto	Malto	Malto	Malto	Malto	Malto	Malto	Malto	Malto	MCT	MCT	MCT	MCT	MCT	MCT	MCT	MCT	MCT	MCT	MCT	EtOH	EtOH
FH	FM	FM	FM	FM	FM	FM	FM	FM	FM	FM	FT	FT	FT	FT	FT	FT	FT	FT	FT	FT	FT	ME	ME
2471	4299	2513	3702	2608	2994	2037	.	1631	1676	2892	2357	2688	2419	3614	2956	3750	2080	2990	4377	1998	2548	3595	3494
2258	4355	3140	2611	2525	4342	2026	.	3111	3540	2293	2148	2584	3957	3573	1897	3266	3352	3201	3906	2160	2728	3112	3329
2470	4070	4261	2619	2570	3384	2877	.	2434	2181	1829	3547	3625	2681	3393	1792	3122	2518	2121	2762	2247	2489	3595	3330
4235	2531	3795	2858	3816	2001	1874	.	1670	2144	2882	3283	4316	2807	2650	1867	2232	2287	2528	2917	3946	3515	3736	3360
3639	2402	2631	3807	3568	3706	1980	.	1760	3569	2804	2170	3421	2410	2715	3255	2104	3674	2295	4110	3447	2740	3895	3268
3443	3994	4066	3661	2706	3577	3611	.	1698	3612	2831	3681	2655	4268	3720	3949	2125	3155	2264	4210	2521	2519	3659	3787
2936	4560	4036	2430	3320	4071	3818	.	3116	3039	2609	3094	2903	3887	3557	3525	2696	2023	3518	2911	3283	3462	3754	3584
2608	4578	4378	3470	4001	3983	2443	.	2647	2601	3251	3310	3496	2312	3250	1908	2973	2465	3181	4123	3889	2644	3904	3571
3894	4624	4254	3703	3184	3400	3111	.	2885	3329	3337	1891	2585	3580	3575	3343	2260	4504	2741	4858	3695	3476	4644	3606
3949	3926	4385	2754	2476	3168	3549	.	1877	3845	3199	1549	3152	2475	3870	3702	2150	4078	2405	4407	4183	4094	3632	4189
3364	2371	4415	2612	3629	3770	3797	.	2556	3734	3130	1548	4350	2361	3655	4240	3479	4201	3528	4368	3801	2419	3929	3801
3558	2352	4505	4390	3896	4362	3853	.	1385	3684	3089	3548	3356	4183	3281	4192	1941	4207	2933	4360	3928	3661	3009	3489
2303	3868	3460	3349	2760	4060	4136	.	2335	4435	2928	3214	4128	3167	3658	4249	1962	4357	2026	4100	3046	3765	3663	3262
2439	4662	3748	2527	3866	3628	4135	.	1505	4478	2757	2534	3161	2277	3667	4003	1876	4278	1852	4036	3622	2401	3822	2839
3670	3320	3841	2238	4411	2421	4125	.	2908	4670	3309	2771	2572	3774	3836	3810	2980	4021	3231	4165	3922	2928	2563	3233
3924	2316	2926	2369	4381	2224	4069	.	2475	4624	4027	2501	2400	4332	3663	3641	3290	3988	3145	3932	4328	4130	2530	2845
4662	2500	2861	3984	3868	2226	3711	.	1160	2860	4421	1486	4241	3023	2494	3505	3694	4004	2444	2916	4517	3562	3553	2396
4835	2410	4392	4438	4052	3016	2079	.	2767	2148	4401	711	4684	4359	2114	3096	2673	3018	1365	2763	4139	2307	2673	3249
5083	4121	4322	2980	4362	3320	1638	.	2734	3205	4268	3159	4329	4181	2816	1414	1497	1864	1477	4313	4318	2396	3155	3701
5212	4445	4596	2263	4100	2440	1674	.	2494	4214	4323	2696	4524	4491	3167	1149	1780	1992	1555	4155	4466	3701	4171	2594
4697	4580	4577	2243	3993	3275	3234	.	3330	4464	4456	1375	4542	3277	3349	1971	3710	3212	1732	4027	4603	3807	4170	2794
3988	4418	4408	4483	2414	4258	3943	.	2371	4339	3951	764	4258	2502	2508	1559	3404	4019	3139	3539	4121	2517	4079	2647
4838	4519	4407	3881	2312	3818	3650	.	1446	3594	2807	499	3662	2236	2874	2530	3911	4010	1840	2465	2678	2131	3907	2359
4443	4685	3621	4294	3595	3865	2735	.	2307	2579	1848	328	2453	4006	3020	3245	4085	4188	1530	2509	2041	2131	3701	2740
2835	4640	2413	2590	2774	3594	3214	.	1796	2346	1656	1173	2251	4251	2295	3838	3871	2986	3109	2422	2783	2360	3573	2412
2264	4790	2181	1958	2369	3308	3478	.	3177	2638	1881	3606	2949	4469	3223	3459	2335	2010	2184	3541	2459	4067	2765	2606
2315	4666	3157	2140	4077	2227	3122	.	3306	4205	2036	2864	2965	3986	3414	2817	1580	1961	1263	3932	1819	4524	2291	3875
2978	3585	3787	3193	4188	2235	1662	.	3290	3034	3855	1420	3769	2800	3422	1465	1672	2032	955	3923	1935	3381	2446	4137
2844	2409	2628	2634	3824	3501	1717	.	3678	2500	4195	859	4853	2296	3115	1193	1983	2053	1393	4067	3870	1820	3246	3335
2555	2360	2596	4106	3073	2631	1828	.	3858	2480	2629	789	4334	2448	2017	1292	2071	3734	948	2552	4397	2660	2818	3728
4193	2215	3776	2639	2275	2383	2417	.	1976	3769	1801	438	2972	3634	2005	1653	3432	2241	667	2274	4410	1845	2860	3381
4495	2310	2722	1978	3530	2409	1829	.	1303	3055	1805	307	2164	2936	2391	1664	2932	1987	1180	2393	3072	1590	2491	3478
4655	4233	2416	2004	3251	3637	1917	.	1259	3303	1816	1324	2403	2557	2134	1956	1898	2900	2775	2320	2094	1334	3784	3110
4712	4477	3388	4492	3921	2517	1886	.	2780	3715	3813	3599	2701	3940	1981	2256	1911	3528	3611	2377	1908	4105	3216	2063
4198	3943	4068	4329	3814	2561	2988	.	2282	3351	3415	2665	4236	4422	3267	3808	3165	3697	2035	4153	1871	3480	3087	2305
2690	2799	3804	2807	2585	3047	3724	.	1616	2356	1865	1164	4266	3940	2324	3927	3808	3746	1526	3510	2305	2958	3726	2233
2508	1674	3186	2060	2065	4147	3905	.	953	2020	1585	626	3403	2270	2059	3700	3964	2071	1672	4061	2239	1928	2566	2708
3645	2003	2598	2240	2304	3094	3578	.	895	2077	1601	367	2558	3952	3244	2570	3440	1786	3235	2276	2042	1417	2210	2101
4440	1984	2412	4020	2688	2529	2137	.	997	3809	1831	282	2428	3478	3502	1515	2989	2043	2679	2154	2779	1179	2413	2219
3006	2180	2392	2773	2908	3805	1810	.	1491	3195	3707	149	2489	2468	2655	1689	1455	3485	2342	2399	3697	927	3240	2284
2475	2080	2506	2152	4043	3472	1813	.	2960	3079	3550	753	4717	2206	1932	1891	1269	3715	1474	2400	3940	1178	3704	3640
2552	2330	2305	2654	3475	2650	2915	.	1793	1832	3654	3721	4444	2517	2238	3233	1743	3066	1152	2694	3038	3910	2347	3262
2771	2310	2333	2319	3713	2843	3725	.	844	1674	2301	2951	3037	2499	2079	3925	1936	1908	2951	4387	2122	2986	2373	2454
3951	3322	3037	4118	2898	2625	3214	.	905	2616	1993	2135	2266	2003	2126	3060	1824	2256	2178	3198	2075	1766	2563	2291
3621	3639	3414	4041	2135	2626	2006	.	1963	2227	2076	1283	2444	3305	3185	1976	3460	2314	1689	1957	2280	1426	3445	2301
2590	2359	2339	3916	2283	3983	1995	.	1508	2181	2494	830	2938	3872	2363	1936	3747	2317	1697	2258	3009	1579	3014	2386
2731	2283	2399	3207	2194	3665	2448	.	1421	2775	2059	550	2436	2657	2204	1872	3843	3390	1703	2562	2129	1943	2367	2385
2622	2336	2850	2473	2947	2763	3180	.	1600	2091	2008	448	3766	2351	2550	2671	3278	3657	1706	3837	1921	1997	2430	2326
3258	2515	2581	2387	3382	2765	1901	.	1401	2207	2182	1309	3817	2364	2671	1589	1910	2643	1911	2209	1764	1710	2535	2193
2303	2677	2322	3193	2353	2711	1905	.	1223	2185	3845	1898	3493	2378	2187	1675	1839	2150	2758	2377	2049	3152	3241	2815
3085	2021	3152	3768	2383	3595	3161	.	1296	2028	2848	2371	2374	2372	2950	1834	1858	2118	2186	2499	3050	1953	3412	3096
3754	2348	3469	2430	2240	2995	3349	.	2773	3520	1770	2975	2249	3842	2187	1973	1726	2148	1350	2493	3610	1707	2398	2162
2793	2126	3324	2696	2453	2799	2034	.	2502	3173	2221	2104	2664	3795	2259	3384	1701	2212	1573	2422	3142	2594	2480	2307
2620	2160	2627	2571	3377	2782	1867	.	1218	1893	3459	1056	2250	2572	2760	3145	3365	2176	1389	3068	2523	4423	2379	2336
2324	3087	2647	2435	2970	3143	1922	.	1491	1989	3826	779	2548	2276	3345	3242	2741	3390	1704	2438	2029	2610	2369	2357
2443	2208	2428	2538	2435	2478	3430	.	1442	1851	3512	580	3982	2461	2829	2033	1655	2620	3076	2500	1862	2227	2365	2955
3042	2236	3081	3992	2397	2559	2596	.	2302	1921	2342	462	2784	3620	2305	1746	1557	2136	2232	2452	2254	3280	3047	2325
2571	2369	2415	3838	4033	3563	1875	.	1364	1880	1983	885	2483	3774	2277	1867	1904	1985	1671	2722	2945	2305	3067	2261
3797	2474	3102	2945	3357	2613	1858	.	1417	2917	2176	380	2504	2989	2188	1881	3282	2960	1910	2478	2074	2628	2371	2419
3015	2468	2656	2493	2357	2503	1924	.	2366	1983	2795	631	3676	2288	2034	3276	3534	4073	1926	2474	2087	2342	2421	2932
2819	4053	2363	2482	2403	2374	2125	.	3025	2203	3513	937	2402	2607	2181	3729	3516	2612	1874	2651	2479	3571	2442	2129
2748	4466	2425	4254	2372	3342	3437	.	3272	2156	2837	2056	3466	2503	3642	2440	2584	2026	1972	3870	2187	4262	2652	2252
2614	4510	3390	3183	2668	2713	3486	.	1693	3015	1848	3837	4014	2309	3631	1639	1711	2990	2046	3656	2001	2945	2813	2102
2776	3930	3592	2395	4179	2723	1829	.	1537	2862	1993	4068	3115	3831	3509	1891	1739	2613	2039	3712	1944	2398	2296	3301
2542	2693	3233	2705	2910	2593	2609	.	1554	1782	3264	3525	2356	3303	2499	2114	1933	2072	3014	3443	3522	2854	2182	2762
3095	2234	2545	2621	2394	3330	3537	.	1413	2046	3031	2166	2562	2909	2226	2950	3209	2401	2817	2433	3316	2629	2293	2188
4163	2740	2491	4047	3650	3456	2380	.	1812	2092	1987	1946	2774	2407	2968	3788	3525	3413	1731	2501	2506	2434	2996	2058
4296	2576	4064	4783	2688	3872	2155	.	3303	3482	2263	3748	4184	3811	3917	3358	2695	3963	2233	2701	2148	2873	2834	2817
3255	2754	3759	4419	2471	4041	2125	.	2822	4164	3695	4119	3010	4002	3717	2916	1790	2434	1872	3920	2576	3760	2767	2468
2841	4239	3286	2975	3789	3502	2588	.	1882	3801	2982	3967	2875	4081	3890	3883	2380	3461	3267	3704	4198	4745	2972	3415
3047	4453	2690	4133	4424	3476	3877	.	3167	2585	2358	3916	3333	4146	3294	2656	3706	4251	3331	3683	4000	3270	3875	3116
2814	4552	3729	4398	4121	3521	3594	.	1952	2458	3656	4010	2873	3304	3396	2577	3669	3973	2618	3968	4080	3846	3749	3245
2786	3132	4262	5024	4409	3075	2719	.	3471	3021	3465	3028	3999	3713	2907	4402	3545	3698	1826	3911	3989	4495	3678	3383
4021	2821	4252	4917	4671	3675	3277	.	3859	2867	2233	2673	2987	4319	2170	4080	3692	4048	1756	4034	4013	4062	3809	3907
3927	4247	4226	4672	3141	4193	3848	.	3225	2355	3345	3472	4038	3040	2157	3736	3174	3016	3239	4205	3952	3058	3555	3716
4277	3354	4307	4561	3928	3893	3684	.	3311	4265	3626	2518	4009	2583	3575	4040	2031	1950	2167	3633	3903	4018	3506	4388
4274	2796	3995	4299	3551	3700	2171	.	3158	3954	3311	1927	2886	3966	3465	3660	2344	3464	1200	3381	2827	4282	3164	2695
4354	3683	4039	4265	2382	3982	2920	.	3494	3083	3371	2178	4226	4514	3765	3184	3565	3791	2047	2391	2557	4197	3026	3416
3966	4369	4622	4019	3293	3645	3625	.	3602	2513	2754	3015	3161	4130	3652	2954	3716	4041	1755	2614	3995	4021	2559	3584
3165	4908	3872	4218	3880	3809	3779	.	3059	3569	2387	3545	2431	4468	3613	1707	3712	3756	3172	3607	3231	3752	2786	2867
2821	3968	2361	4164	2547	3670	3960	.	2859	4258	2081	3680	3417	3953	3698	1635	3471	2464	2993	4618	2422	2977	2761	3393
2980	2710	3510	4417	2097	3521	3688	.	2909	4095	2967	3179	3992	4451	3827	2809	3271	1723	2149	4713	3188	2449	3624	2686
3661	2673	3525	4326	3178	2860	3703	.	2986	4157	4042	3660	3298	4312	3718	3636	3010	1506	1724	4006	4070	2809	4288	2429
3743	4236	3220	3909	4188	3045	3458	.	2794	4487	3908	3866	2842	3909	3125	2166	1740	1633	1457	4274	4207	3750	4175	2239
2974	4831	3166	2606	4132	3352	2189	.	1833	4292	3945	3617	3692	3474	2507	1902	1728	1837	1369	4791	4447	2923	3830	2473
4022	3498	2559	2435	3349	3908	1943	.	1724	4152	3795	3417	2814	2241	2000	2095	1819	4059	2644	4270	4169	3964	3802	3341
4912	2781	4426	3928	2179	3829	2447	.	1714	3997	3876	3388	3676	2324	2678	3411	3350	4257	2828	4658	4090	3457	3647	2651
4382	2994	4158	2618	3440	3606	3328	.	2868	4100	3390	2084	4423	2212	2161	3320	2786	4327	1581	4444	4160	3248	3641	2243
4391	2440	4221	2564	4086	2827	3638	.	3444	3489	3411	1847	4291	2484	2093	2085	1761	2621	1251	4127	3714	3253	2586	3218
4403	4028	4101	3194	3062	3106	2179	.	3213	2397	3355	2029	4515	4227	2596	1978	1546	1714	770	4029	2327	2544	3021	2629
4159	4664	4372	2714	2368	3068	1962	.	2494	2305	2207	3075	4078	4049	2491	2132	3321	1779	879	3806	2086	2558	3643	2470
3919	4740	3927	2560	2112	3194	3265	.	1792	3034	1820	2645	4052	3995	1943	1915	2965	2090	3092	2800	2255	2616	3049	2251
4228	4790	2730	2847	2261	3104	2878	.	1688	2555	2214	2119	3904	2891	2440	3586	2075	3803	2332	2824	3340	2604	2917	2873
3435	4581	2287	4128	2602	3539	1971	.	1862	2372	2058	2641	2697	2192	3575	3327	3123	2273	1183	3321	3457	2651	2840	2465
2833	4700	2391	4175	2257	3189	2388	.	3566	2254	2711	1958	2410	2631	3266	3851	3488	1772	746	3534	2354	3779	3811	2343
2769	4374	3467	3307	2126	3088	1945	.	3122	3273	3406	1894	2511	3628	2197	3415	3981	1780	1247	3180	1948	4314	2841	2377
2777	4044	2779	2512	4078	3444	3104	.	2525	4008	2965	2994	3351	3455	2286	3070	3152	2956	615	2808	3509	4362	3587	2646
2746	3691	2396	2383	4240	2779	3218	.	2308	3533	2110	2422	2361	2384	2765	2013	1992	4474	412	2822	3445	3556	2882	3991
3430	2660	3414	3287	4229	3806	3369	.	3157	2530	2023	3410	2545	2098	2180	1819	1563	3740	2122	3750	2425	4014	2676	2926
2946	3212	2450	4141	4047	3786	2913	.	3326	2395	1864	3447	4142	2254	2144	2016	1660	3311	3262	4283	1902	3688	3562	3455
3032	2190	2374	3380	4167	3170	1855	.	2357	2336	2301	3124	3184	4074	2727	3133	1939	3499	2400	4508	3653	2726	3631	3653
4433	2659	3618	2315	2901	2645	1810	.	1624	3552	1869	2280	2334	3767	4046	3468	1647	2677	1225	4151	2839	2376	3175	3210
3052	2129	2629	2301	2131	2495	2625	.	1579	3647	3350	2049	4164	3399	3311	2600	3338	1675	861	3574	2229	3009	2693	2135
2536	3292	3231	2240	2294	2146	2153	.	1886	2627	3340	2092	4147	2367	2223	1964	3496	1323	634	2576	2808	2323	2360	2111
2549	2215	2449	4064	2287	2520	3245	.	1707	2231	2174	2175	3276	2166	1922	1979	2344	1759	806	2535	3619	3590	2668	2685
3958	2195	2355	3826	3559	3660	2358	.	2203	2252	1739	2241	2334	3691	2578	1932	1733	3896	468	2856	3397	3486	2542	2627
3743	2503	2987	2758	3653	3141	1809	.	1721	2274	1830	2096	3251	3641	2230	1930	2138	3090	522	3631	2299	2993	2318	2205
3236	3899	3945	2139	2308	2612	1815	.	3177	3247	3115	2118	3211	2404	2000	2931	3190	2052	1652	3774	2021	2313	2747	2326
2580	2882	2515	3176	2285	3367	1931	.	2002	3740	3150	3757	2939	2151	3189	2223	2061	1970	3512	2583	2040	2394	2315	2085
2566	2410	2353	3656	3417	3655	1869	.	2206	3230	2117	3549	2702	2746	2157	2047	1885	3689	2852	2728	3742	3028	3026	2636
3081	2613	3192	2623	4079	2663	3155	.	1961	2887	2909	2522	2851	3518	2058	2416	3205	2853	2573	2789	2748	3247	2562	2288
2304	2496	2736	2464	2827	2785	3917	.	1853	2354	2918	2168	3466	2718	2309	3897	3302	2891	3255	3753	2302	2294	2593	2327
2365	3666	2485	2465	2207	3413	3396	.	2451	3416	1893	3382	2515	2233	2108	2957	2572	2720	3780	2879	2058	2333	2268	3080
3060	3510	2734	3360	2278	2869	2871	.	2571	2924	3339	2630	2584	2052	2322	1948	1969	2232	3875	2775	3527	2307	3248	3136
3402	2353	3307	2428	3711	2582	1877	.	1803	2149	3595	2269	3614	3731	3100	1950	1854	1979	3524	2833	2736	2457	3285	2171
2418	2342	2457	4066	3149	3674	1928	.	1784	2426	2274	2274	3245	3301	2780	2571	1982	3231	2241	4300	2374	3085	2445	2244
2450	3554	2473	2692	2350	3123	1947	.	1773	2574	2209	2025	2256	2241	2291	3080	2952	3493	2223	4029	2287	3906	2278	2229
2635	2516	3465	2461	2255		1872	.	2725	2140	3106	3320	3498	1987		2104	1870	2225	3273	2843	3190	2391	2172	2743
2885	2394	2495	2875	2371		2664	.	2818	2848	1948	2712	3454	2123		2134	1954	1964	3857	2678	3146	2502	2211	2558
2383	2584	2624	2463	2332		1859	.	1677	2098	2039	2196	2473	3103		2102	2743	1975	3105	3192	2352	2230	2548	2289
2426	2522	2508	3796	3646		1973	.	2492	2393	2094	1874	2522	2267		2098	3538	2259	2016	2844	2299	2387	2431	2411
3800	2537	2476	3986	4162		1874	.	1901	2618	3315	3476	2417	2068		2151	3242	2151	2094	2822	2763	2351	2342	3164

2422	2282	3586	3996	3734		3484 .	2015	3603	3068
2446	2563	3456	4004	2297		3109 .	1990	3113	2025
2596	3950	2462	3038	2228		2008 .	2999	2811	2066
3065	3156	2550	2428	2426		1966 .	1904	2136	2756
3288	2189	2476	2445	2317		1903 .	1777	2932	2012
3306	2426	3515	2667	2629		3433 .	1955	2121	2014
2505	3780	3490	2480	4173		2420 .	1909	2105	3454
2739	4057	2502	2638	4206		1845 .	2999	2310	3479
2511	2671	2380	3734	3647		2099 .	1890	3943	2241
3898	2976	3156	4214	2550		3419 .	2283	3705	1924
3503	3622	3914	4517	2461		3496 .	3367	2404	2805
3039	2821	2853	5067	2625		2020 .	2163	2451	3331
2981	2563	3250	4898	4346		2317 .	3534	3859	4139
4445	3887	3946	4021	4440		3431 .	3714	3812	2691
3932	4615	4042	2575	4388		3800 .	3870	2636	3850
4043	4614	4049	4411	4143		3289 .	3313	2859	4013
4277	3808	2697	4672	4160		1875 .	3042	3674	2586
4335	3036	3151	4239	2769		3636 .	3507	3159	3187
3704	2999	2985	3958	4096		3444 .	3790	2481	4189
4168	2843	3208	3973	3178		3145 .	3676	3297	3739
4051	4475	3082	2927	2331		1797 .	3196	4013	2340
3395	4742	3662	2524	2409		2182 .	3367	2811	3385
3762	3871	4739	4257	4454		3193 .	3751	3053	4042
3453	3021	4090	4553	4301		3591 .	3461	4364	3437
3048	2828	2926	4049	4295		3507 .	3845	3203	3245
4921	2967	4306	3761	4321		2183 .	3255	2473	3378
4666	2714	4039	2403	3804		1689 .	3418	3842	4026
4824	3466	3761	2495	2709		3057 .	3166	4107	2587
4680	4772	3857	3281	1927		3745 .	3096	3096	1905
4666	5023	3238	4232	2393		2626 .	2865	4219	2687
4709	4849	4073	3948	2362		1483 .	2912	4557	2193
4437	4510	2918	4104	2886		1767 .	1967	4570	1974
3332	4417	3175	3183	4258		2055 .	1612	4186	2514
2795	4263	3246	2451	3824		1683 .	2159	3643	2030
3003	3185	3043	2596	2365		2828 .	1743	2593	3750
2708	2386	3512	2430	2139		3173 .	1736	2257	2969
4113	3331	4190	2804	2218		3529 .	3351	2338	1979
4189	4437	3752	2443	3166		2811 .	3470	2345	1772
3158	3975	3476	3963	2215		1686 .	2829	3737	1961
2736	2885	2483	3478	2214		1820 .	2727	3306	3072
2790	2149	3218	2373	2235		2111 .	3080	2352	3796
3854	3599	2750	2312	2657		1830 .	2250	2067	3561
4655	3838	2704	4438	4140		1619 .	2846	2300	3613
3533	3838	4017	3987	3939		3590 .	2981	2825	3065
3019	3446	3202	3467	3962		3831 .	2519	3242	1867
2631	2227	2520	3066	3762		2788 .	1490	4031	1821
2818	2224	2988	2153	2878		1684 .	1720	2816	2099
2397	2345	2972	2438	2159		1932 .	1715	2315	1887
3046	3786	2549	4044	1961		1853 .	2184	2095	2621
2804	2506	2347	3077	2821		3125 .	2419	3411	3274
2805	2541	2914	2150	3875		3349 .	1720	3564	2663
2585	2514	2950	3120	2382		3220 .	2514	2312	2126
2664	2501	2468	2512	2302		2170 .	2147	2207	1830
3457	2358	3457	2737	2450		1569 .	1809	1964	2790
2994	2517	2574	3916	3653		2312 .	2371	2448	2182
2991	3418	2395	3548	2453		1730 .	2301	3779	2941
2824	3394	3355	2302	2381		1915 .	1931	3564	3413
2845	2566	3571	2321	2301		2694 .	1835	2397	3069

3293	2336	3671		3197	2093	3716	2081	2689	3452	3293	2239	2249
2485	3597	3563		2157	1843	3175	2965	4024	2372	3603	3176	2321
3374	2551	2177		1899	1995	2098	3658	3743	2053	2549	3718	2838
2705	2449	1882		2069	2799	1908	2519	2585	2231	2551	2458	2552
2246	2733	2975		2447	2079	2175	2306	2610	1955	3274	2584	2440
2194	3453	3735		3212	2038	3265	2935	2467	3473	3438	2287	2313
3617	2273	3722		2192	3451	3690	2137	4266	3693	2446	3474	2205
3122	2723	2776		2134	3014	2308	2847	3766	3780	2391	2577	2427
2209	2845	2266		3021	3189	2103	4110	2570	2866	3345	2550	3288
3306	2302	3133		3601	3449	2777	3901	2703	2475	3728	2698	2955
4051	3984	4029		2400	3281	3891	2305	3580	2394	2749	2752	2235
4182	3621	4260		3467	1873	2957	2273	3717	3525	2771	2499	3248
4235	2656	4104		3045	3040	2252	2307	4473	4061	4401	3655	3354
4501	3755	4150		2607	2533	2248	3849	3577	4510	4647	3813	3276
3906	2919	4414		3570	2204	2981	4027	4258	3704	4036	3762	3066
4075	4169	4619		3311	3450	4529	2677	3275	4275	4788	2870	2942
3982	3975	4125		3040	2972	4034	2005	3013	4221	4785	3805	3429
3835	3031	4389		3778	2486	3279	3168	4453	4140	4717	2825	3601
4221	3705	4830		3475	3677	2453	2206	4593	3184	4417	3638	3104
3971	4222	4318		3864	3311	3624	1993	3263	2449	4634	3261	2611
3424	4237	4061		2271	3658	4341	3489	4584	3142	4675	3315	3797
2251	4238	4047		2150	2384	4092	2763	3784	4259	4451	3974	3930
2057	4586	3233		3502	1565	3409	2751	3896	4154	4270	2829	3388
2544	4089	2163		3973	2732	3796	3126	4972	4292	3987	3284	2347
2113	3903	2252		2489	3188	3624	3254	4752	4142	2618	3028	2466
2777	2956	2526		2602	2255	3842	2980	4704	4106	3421	3757	3370
3002	3580	4333		2135	1736	2548	3015	4105	4176	3464	3991	3022
3249	3966	4377		2281	1692	1745	2149	3531	4137	2526	4052	2896
3274	2958	4288		3732	3250	1811	2618	2963	3951	3694	3556	2811
2007	2621	4098		4076	3466	1912	1857	2648	2779	4726	3531	2801
2779	3495	3243		2286	3282	2675	2117	2713	2720	4683	2327	2739
2321	2643	2218		2360	2103	2185	2003	3953	2447	4655	2615	3729
2051	2938	1932		3813	1978	2100	2690	4348	3682	4388	3815	3500
3457	3941	2307		2587	2482	1875	2151	4571	2923	3703	2750	2605
3845	2739	2410		2333	3196	3240	2103	4663	3219	3454	2726	2878
3858	3204	2256		3313	2057	4303	2145	4039	2463	2476	3585	3067
3796	3496	3576		2536	1822	3621	2052	2908	2427	3900	2719	2842
3062	4250	3925		3546	3026	3793	2824	2634	2280	3467	3686	2925
2076	4514	2763		2950	2294	2962	3398	3770	2265	2475	3201	2977
3354	3768	2084		2557	2929	1877	2339	2779	3928	3546	2913	3084
3346	2496	2027		3053	1983	1765	2122	2609	4398	3776	3161	2675
2252	2473	3531		2352	1887	1724	2387	2758	3681	2805	2937	3486
2034	2467	4034		2922	3639	3748	1894	2719	3763	2881	2868	2740
3104	4402	3634		2109	3585	4036	2031	4279	2880	2747	3300	2132
2054	3701	2297		1860	3281	2918	2392	3458	2335	2645	3586	3201
2006	2369	2052		2010	3069	1761	3482	2568	2142	3627	2338	2616
3114	2441	2305		2066	2166	1754	2764	2529	3328	2486	2570	2445
2113	3862	3880		2170	1917	3461	1891	2831	2501	3433	2322	2288
2144	3810	3096		3479	2631	3317	3563	2757	2772	2771	2353	2053
2152	2548	2232		2720	2107	2137	2818	3857	3411	2477	2380	2633
3399	2433	1994		2167	1956	1820	3571	3788	2995	2887	2208	2325
3409	3006	2898		1910	3032	2097	3124	2592	2288	3581	3010	2128
2284	3040	2209		2159	1936	3506	2018	3094	2325	2554	2442	2297
2179	2421	2189		1949	1714	3471	2202	2685	2554	2283	2343	2527
2325	2669	2522		2736	1933	2016	2262	2787	3454	2798	2418	2513
3615	3837	4079		3230	3199	2054	3019	3342	2264	2318	2711	2145
3507	3414	3332		2158	3038	2346	2782	4049	2044	3502	3389	2973
2507	2275	2191		2227	2634	2014	1894	3792	2222	3502	2414	2530

3997	4446	3096	4023	5025	3563	3001 .	3392	3159	4499
3123	3983	3114	3971	5005	3751	3267 .	2908	2179	3614
2640	4099	3500	4544	4374	3800	2401 .	2415	2106	2453
4226	3764	3702	3288	4236	3230	2252 .	3290	2366	3247
3225	3079	2968	3353	4287	3807	2039 .	2952	3911	2478
4383	3579	3431	4126	3675	3227	3432 .	2364	4348	3188
3277	2935	3207	3292	2779	3511	3265 .	2860	4143	3870
3016	3070	2614	3189	2795	3567	2075 .	3433	3189	2603
3032	4272	3413	4134	3331	3231	2122 .	3217	2384	2432
4199	3144	2928	3157	2879	3173	2586 .	2530	3062	2530
4152	3106	2558	3986	3755	3138	3389 .	2267	4489	2649
3255	3511	3278	3298	3473	3397	2390 .	2933	3338	3431
3270	3123	2858	4118	2997	3460	3626 .	2429	2382	3290
3742	3428	2973	4927	3341	4120	3858 .	2336	3826	2747
4370	4586	3868	4279	4293	4057	3654 .	3565	3356	2906
4676	4483	3702	4421	3519	3793	2894 .	3905	3446	3255
3639	3890	2862	4856	3301	3865	2369 .	3688	3343	3758
4521	3703	3413	4597	3149	3943	3679 .	4194	4277	3292
3703	4048	3664	4325	4236	3412	3841 .	3952	4222	3664
3926	3579	3274	4244	4472	3229	2342 .	3730	3173	3618
4391	4285	3806	4541	3507	3549	2153 .	3697	3713	2917
4366	4118	2986	3958	3957	3624	2735 .	3725	3549	2934
4401	3248	2608	3285	3171	3641	2404 .	3197	3711	2524
4517	3136	3225	4247	2742	4260	2304 .	2430	3986	2442
4387	3164	3891	4094	2737	3780	3307 .	2804	4033	2755
4482	4235	2688	4011	3670	3603	3536 .	3485	3449	3542
4355	4021	2628	4609	3191	3296	3866 .	3870	3936	3572
4074	4353	2402	4542	2700	3311	3457 .	3781	3178	2939
4118	3436	2545	3316	2667	3128	3564 .	3550	3672	4113
3798	3929	3741	4172	2593	3838	3945 .	3265	3521	3658
3851	4686	2874	4120	4400	3621	3507 .	3975	2987	3645
3616	3980	2586	3820	4154	2982	3385 .	3553	3315	3614
4094	3670	3304	3400	3863	3061	2789 .	3385	3930	3824
3902	4256	3580	3880	3121	3545	2339 .	2743	4118	3328
3847	4151	3439	4350	2685	3017	2183 .	2636	3327	3566
3896	3178	3764	3632	2514	2686	2088 .	3681	2788	2361
4321	3962	3770	3207	3816	3168	2294 .	3380	3045	3654
4026	4428	3589	3493	3585	4211	2084 .	3557	3023	3585
4313	4030	3515	3529	2656	3212	3621 .	3436	2776	2930
3621	4347	3263	3971	3440	2660	3374 .	3486	3742	2640
3473	3577	3683	3276	2930	3513	2130 .	3479	2923	2519
3491	3176	3044	3523	2935	3718	2616 .	2924	3103	2453
3862	3296	3657	3208	3082	2988	3641 .	2713	3714	2643
3567	3296	3621	2833	2603	2856	2737 .	3258	3648	2566
3238	3249	2962	4327	3895	3187	3614 .	3303	2770	3171
3170	3079	2514	4167	4148	3701	2440 .	2444	2590	3550
3513	3510	2470	4051	3850	2685	2167 .	2560	3036	2797
4030	3239	2413	3260	3899	3634	2351 .	2759	3655	3133
3276	3876	2166	2861	2770	2967	2162 .	2516	2749	2373
3429	3154	2433	3906	2611	2898	2867 .	2488	2529	2364
3269	3070	3163	3513	2399	3572	3254 .	3073	2655	3054
3877	3574	2544	3350	3187	2999	2727 .	2874	2647	2489
3168	3332	2415	2921	2633	2920	2492 .	2973	3434	2471
3156	3067	2510	3112	2292	2859	2284 .	2875	2464	3962
3214	2819	3446	2914	3399	2996	2665 .	2742	2487	3478
3668	2870	3352	3401	2601	2684	2375 .	2874	4099	2704
3249	4121	2768	3753	2642	3708	2844 .	2712	2642	2866
3223	4064	2474	3109	2530	2970	2459 .	2705	2479	2565
3133	3060	2469	2913	3647	2976	2454 .	2790	3114	2757
3488	2807	2730	2815	2872	3031	2393 .	2378	3500	3486
3290	2927	2463	4170	3132	4247	2913 .	2698	3262	2403
3010	3109	3166	3384	2688	3507	3193 .	3016	2560	2460
3791	2781	2522	3070	2397	3128	3627 .	2454	2805	3228
3430	2847	2720	3074	3890	2925	2525 .	2342	2938	2635
2999	3338	2453	3619	2928	3872	2355 .	3107	3010	3264
3135	3511	2637	4290	2620	3227	2300 .	2602	2658	3681
2957	2846	2697	3392	2761	3179	2326 .	2758	3882	2491
3237	3775	3609	3414	3449	3547	2946 .	2607	2745	2724
3568	2828	3378	3303	2669	2982	3560 .	2296	2670	3065
3261	2808	3060	3152	3705	3000	2979 .	2867	3803	3475

2966	3861	2941	3080	3703	2652	4491	4564	4077	3583	2791	3831	3314
3743	3536	3731	2709	2903	3116	4018	3981	3889	4005	2549	3839	3196
3159	3250	4231	2963	2617	3299	2979	3803	3363	3062	3124	3355	2393
3029	2663	3014	2646	3346	2787	3251	3829	3167	2619	2388	2497	3463
3332	2972	2338	3163	2779	3433	2716	3700	3522	3818	3590	3637	2985
3027	4106	3929	2777	3623	2557	3680	3794	3321	2839	3981	2894	3169
3240	3290	3961	2512	2970	3446	2937	3912	3211	2602	2614	3239	3316
4202	3884	2780	2946	2590	3680	2673	4018	3428	2675	3506	2825	2282
3199	3804	2649	3280	2601	2654	2521	3970	3170	2774	2887	2875	2978
2729	3029	2696	2910	3585	2541	4094	4254	3044	3783	2562	3045	2578
2948	2927	3908	2784	2896	2501	3910	3649	3395	3047	3256	2669	2293
2916	3824	2952	2490	3325	3510	3424	2669	3265	2619	2539	2743	2480
2957	3007	3165	3399	4913	3065	2730	2799	3490	2720	2814	3052	2391
4274	3930	3498	3192	4198	3193	3453	3469	3320	2944	3683	3568	3320
4267	4037	3140	4262	3550	3880	3751	2837	3365	3716	3408	3308	3573
4346	4029	3816	3885	3682	3806	3279	2776	2865	2901	4288	3726	3212
4403	3814	4132	3970	4160	3566	3598	3100	4304	3713	4612	4001	2985
4409	4359	4084	3628	2884	3529	3719	3966	4391	4205	4124	3472	3203
4268	4319	3422	3513	3549	3563	4396	2773	3871	4022	4086	3710	3405
4007	4267	3826	2357	3857	3506	4419	3954	3847	3792	3700	3942	4227
3754	4535	4089	3890	3687	3567	3160	3429	3677	3844	3211	3607	3499
3737	3941	4271	3613	3124	3141	2663	4219	3697	4086	2842	3306	3390
3310	3413	3270	3645	2599	2411	3874	3829	4506	3734	3240	3873	3336
3518	3936	2523	3100	3425	3254	3788	3363	3904	3986	3105	4525	3355
4330	3938	4258	3870	3062	2683	3210	3450	4424	3806	3452	3871	3215
4439	2907	3833	3640	2770	2321	2890	2547	4062	3735	3164	3437	3190
4568	4104	2698	2720	2565	3921	2545	2368	3354	3627	3213	3218	2876
3937	4198	3823	3797	3397	3704	3549	3190	3831	2927	4650	3339	3095
3881	3385	4035	3538	3703	3640	4120	2587	4559	2927	4407	3695	3489
3183	3068	2793	2855	3659	3548	4067	2333	4343	2814	4170	4118	3922
3021	2990	2769	2657	2939	3077	3866	2972	4492	2804	4123	3758	3627
3052	2688	3066	2679	2682	2450	4531	2455	4504	2794	4083	3236	3349
3104	3050	3542	2857	2320	2850	3956	2456	4305	3816	3945	3451	2956
3547	2929	2611	2854	2576	2395	3153	3603	4541	3871	3598	4048	2952
3812	3066	2623	3620	2344	3037	2772	2505	4260	3895	2962	3338	3140
2817	3793	2517	3624	2297	2638	2569	4032	4057	3490	2924	3456	2872
2572	3313	2750	3151	3208	2192	2629	3598	4160	2789	3588	3398	3454
3355	2638	3029	2899	2589	2014	4559	2449	3522	2718	2923	4103	3325
2859	2684	4103	2968	2551	3680	3838	2313	3219	2640	3312	3115	3074
3909	3661	4175	2657	3813	3439	2988	2325	3301	3565	2993	3095	3286
3652	3201	3994	3158	3767	3109	2777	2457	3673	3738	2978	3306	3304
2863	3950	4081	3326	3583	2554	2771	2547	3332	3004	2954	3396	3025
2539	3664	3945	2682	2607	2198	2676	3810	3048	2770	3373	3282	3027
2383	3873	3453	2505	2527	3303	2773	2957	3081	3066	3629	3054	3015
3614	4435	3892	3085	3362	3576	2518	2349	3317	2708	3807	3286	2793
3700	4131	3486	2812	3970	2131	2411	2130	4150	3854	3426	3186	2660
3049	3923	2787	2359	3440	2132	4063	2392	3221	3882	2699	3013	2585
2625	3655	2576	2216	2559	3304	3785	4060	3048	2998	2489	3271	2728
3545	2608	2641	2894	2337	2187	3395	3617	3687	2580	2913	2760	3187
2883	3565	3332	2603	3036	2520	3131	2434	3016	2780	3409	3340	2789
2536	2729	3503	2343	2624	2580	2479	2453	3225	2617	2935	2872	2583
3083	2725	2773	2744	3648	4067	3207	2322	4310	2509	2993	2838	2425
2881	3434	2774	3830	2587	3394	2921	2471	4002	2540	3187	2698	2829
2685	2926	2798	2944	2391	2182	2673	2525	3352	3958	2813	2684	2446
3489	2662	2792	2447	2543	2161	2445	2901	3131	3367	3650	2322	2825
3056	3733	2574	2410	3694	2376	3260	3651	3630	2684	2931	2525	2816
3289	3644	3753	2530	3345	3315	3604	3544	3072	2839	2777	3703	2458
3533	3005	3748	2618	2693	2568	2629	2316	2913	3599	2830	2664	2640
3290	2878	2713	3098	2527	3113	2772	2391	3074	3009	2891	2736	2648
2746	4040	2738	3504	2466	2550	2846	2592	4071	2832	2845	2896	3023
2515	3864	2604	2871	2063	2502	2628	3363	3346	2604	3543	2560	2513
3287	2978	3038	2449	2471	3535	2517	2541	3076	2524	2999	3292	2429
2929	2818	3566	2595	3433	2698	2733	2455	3428	3727	2818	2507	2578
2898	2884	2777	2373	3237	2740	2753	2471	3907	2987	2758	2453	2429
3723	2701	2821	3123	2288	3375	2662	2704	3216	2670	3044	2656	2394
3482	3428	2640	2467	2250	2647	3877	4329	3357	2578	3479	3253	2156
2682	3707	2623	2325	3347	2465	3606	3110	3024	2764	3809	2639	2399
2586	3193	3745	2801	3284	3219	2701	2406	3048	2600	3548	2680	2405
2313	3020	2853	2353	2186	2945	2614	2492	3821	2356	2975	2465	2975
3921	3625	2846	2393	2252	2696	3905	2576	4176	3680	3734	2846	2365

3271	2844	2493	4286	4412	2821	2992 .	2328	3610	2683
3306	3905	2493	3285	3924	3036	2533 .	2554	2669	3134
3051	4043	2387	3115	2740	4104	2508 .	2998	2824	2883
3184	4295	3427	3009	3555	3062	3543 .	3102	4411	2550
3852	3225	3074	4218	2896	3190	4314 .	2479	3570	3657
3153	2827	2457	3502	2883	2813	2903 .	2459	3035	2654
3293	2987	2714	3354	2518	3263	2516 .	3392	3047	2575
3498	3768	3857	4063	4451	2974	3463 .	3156	3155	2700
3953	4479	4068	4836	4056	4042	4333 .	2852	3825	2824
4140	4630	3432	4842	4294	4295	4161 .	4086	3505	3448
4489	3793	4013	4466	3855	4008	3704 .	3600	4356	3781
4113	5191	4284	4642	4491	4124	3888 .	3591	4123	3840
4070	4254	4053	4688	3942	3697	3608 .	3818	3644	3872
4033	3593	4164	4204	3544	4123	3858 .	3502	3582	2932
4291	3162	3783	4082	2564	3731	3021 .	3442	4451	2864
3855	4142	3488	4262	3282	3614	4010 .	3678	4247	3258
4170	3903	3741	3523	3246	3527	4011 .	3584	3892	4084
4074	3367	3770	4601	2715	2875	3548 .	3495	4690	3790
4068	3659	3548	5118	2779	3495	2365 .	3292	4494	3827
4096	3116	2750	4541	4381	2672	2181 .	2740	4487	3780
3607	3363	2582	3596	4014	2556	2089 .	2032	4400	3094
4552	4116	3560 .		3326	3629	2628 .	3362	4004	2708
4647	4750	3784 .		2562	3956	3955 .	3293	4122	3533
4197	4148	2794 .		2899	3285	3688 .	2282	3916	2828
3844	4232	2533 .		3631	2870	3339 .	2954	4090	3231
3736	3429	2331 .		4301	3049	3775 .	3168	3981	3000
4222	2687	2301 .		4219	4019	3171 .	3256	3450	2895
4171	2571	2966 .		3155	3820	2110 .	3006	3189	3232
4088	3682	2549 .		2817	3485	2934 .	2630	3283	2971
3685	4468	2318 .		2862	3519	2125 .	3018	3087	3370
3942	4400	2653 .		**2757**	3841	2994 .	3217	3412	3705
4160	4031	2535 .		**2867**	3348	2246 .	2507	3192	4106
4175	3169	3612 .		**2713**	2974	2132 .	3464	3652	2829
3693	3197	3983 .		**3103**	3344	2162 .	2666	3578	2950
3640	4007	3828 .		**2656**	3794	2741 .	2964	3430	3397
3595	4668	3373 .		**4355**	2733	3263 .	2647	3147	3894
3226	3984	3536 .		**3617**	2897	2122 .	2363	3063	3124
3162	2863	2953 .		**2915**	2910	2064 .	2990	3505	2741
3466	2499	2185 .		2715	3132	1956 .	3018	2861	2893
3410	2429	2214 .		2588	3077	2051 .	2424	3066	2677
3646	3139	2206 .		2742	3982	3880 .	3012	2903	3539
3431	3802	3850 .		2524	3182	3532 .	2265	3387	2770
3539	2591	3518 .		3781	3234	3518 .	2150	2907	2615
3310	2477	2999 .		4175	2483	3149 .	2899	3617	2369
2880	3254	2347 .		3113	2488	2187 .	2321	3347	2512
2819	3080	2118 .		2538	2846	2124 .	2364	2865	2539
2982	2716	2653 .		2693	3640	2065 .	2090	3772	2695
3556	4463	2363 .		2456	2501	2777 .	2617	2652	2942
3270	3876	2256 .		3067	2506	3441 .	2122	2587	2539
2988	2901	2219 .		2420	2870	2367 .	2934	3364	2494
3042	2971	3274 .		2522	2537	2285 .	3371	2772	2624
2995	2841	2417 .		4311	3474	3260 .	2292	3236	3047
3524	3327	2278 .		3176	3090	2875 .	2262	2685	3167
2987	3120	2511 .		2707	2835	2228 .	3183	2929	2873
2846	3034	3491 .		2785	2573	2286 .	2226	2522	2663
2850	3100	2587 .		2702	3653	2223 .	2209	2614	2879
3818	2583	2243 .		3588	2898	2162 .	2154	3598	2778
3532	2668	3330 .		3127	2856	2196 .	1973	3838	3019
3311	2614	2589 .		3320	2311	2412 .	2710	2555	2556
2821	2604	2486 .		2696	1435	3961 .	2207	2608	2984
2993	3451	2401 .		2764		3761 .	2039	3681	2560
3034	3211	2294 .		3989		3372 .	2716	3283	3096
3289	2758	2414 .		3847		2636 .	2226	2590	2767
3041	3903	3381 .		2929		2498 .	3341	2435	2711
3118	2909	3473 .		2741		3266 .	3300	3200	2728
3687	2970	2847 .		3472		3712 .	2289	2504	3139
3014	3440	3691 .		4383		2434 .	2040	3725	3318
3017	4145	3499 .		3926		2442 .	2055	4346	3806
2920	3690	2657 .		3068		3478 .	2623	3346	3568
3790	3170	2535 .		2864		3570 .	2379	4020	2778
3107	2960	2813 .		2803		2631 .	2112	3928	2781
3142	3193	2848 .		2779		2703 .	2182	3208	2867
3130	3691	2741 .		4198		4120 .	2241	2847	3200
3927	2918	3741 .		4442		4622 .	3570	4202	2783
3642	4251	3936 .		4438		4087 .	3780	3718	2719
3343	4649	3769 .		4025		3874 .	3296	4088	3808
3689	4884	4049 .		4445		3861 .	3432	4397	4550
4348	4398	3535 .		3656		3801 .	2544	4302	3255
3570	4036	3315 .		3149		3213 .	2764	3829	3743
3813	4115	3345 .		3634		2481 .	2984	4212	3438
4305	4002	3643 .		3463		3561 .	2057	3980	3139
3977	3988	3459 .		3067		4183 .	1980	3969	3453
3528	3484	2622 .		2804		3736 .	3272	3486	4099
3281	2786	3809 .		4335		3487 .	3524	3388	3389
3025	3279	3764 .		4123		2653 .	2868	4333	2924
2896	4599	3534 .		3952		2237 .	2981	4458	3601
3024	4437	2861 .		3637		2255 .	3089	4377	4758
3242	3067	2383 .		3157		4230 .	3058	4763	4037
3744	3650	2388 .		3606		3696 .	2888	4371	3856
4309	3430	3643 .		4116		3194 .	2649	4510	3701
4117	2908	2851	3787	2986		2455 .	2970	3977	3149
3024	2824	2703	3406	2849		2086 .	2195	3992	3188
3663	2760	2384	3300	3676		2252 .	1971	3223	3634
3488	4030	2518	3805	2916		2131 .	1730	2555	3203
3956	3974	3476	3628	3694		2678 .	3107	2370	3042
3681	3982	3844	3805	3691		2260 .	2826	3910	3187
3454	4414	3805	4729	3069		2250 .	2367	3385	3725
3315	4367	3327	4530	3443		2380 .	2131	2927	3193
3643	3788	2590	3932	2901		3587 .	2250	2810	3524
3876	3919	2635	3245	3051		3736 .	2814	2586	3859
3250	3279	2193	3018	3164		3735 .	3115	3345	2990
3025	2927	2221	4767	2996		3471 .	2951	2928	4522
3666	2653	3480	4430	3768		3012 .	2877	3259	3631
3855	3462	3921	4214	3725		3170 .	1893	3653	3302
3458	3025	3608	3704	2912		3231 .	1580	3566	2850
3376	2615	3112	4033	2944		2437 .	2423	3333	2936
3305	2798	2879	3923	2927		2155 .	3489	4005	2923
3738	4102	2654	2911	2698		2009 .	2733	3916	3042
2973	4121	2380	3092	2802		2137 .	2563	2685	2759
2708	2986	2181	2979	2989		3558 .	1986	2464	2728
3555	2585	2418	2788	2425		2712 .	1871	2291	2491
3328	2742	2435	2879	2392		2332 .	1927	2434	2487
2706	3908	2224	4302	2732		2450 .	2292	2849	2864
2624	3161	2327	3149	2370		2629 .	1972	2561	2637
2721	2872	2974	2932	4018		3328 .	2013	2639	2840
2863	3543	2973	3029	3794		2386 .	2554	2752	2611
2857	3897	2465	3131	3136		2354 .	2402	3652	2662
3281	3119	2498	3710	2573		2456 .	2030	3837	2526
2795	2908	2238	3126	2489		2371 .	1906	2688	2566
2638	3308	2263	2884	2344		4390 .	2071	2513	3479

3693	4024	4045	4049	3718 .		4554 .	3189	4756	4102
3954	3955	3483	3346	3890 .		4641 .	3372	4306	4062
3455	3828	3535	3529	3178 .		4430 .	3068	3905	3548
3330	3713	3555	4152	3430 .		3656 .	2621	4525	3285
3413	3007	3883	4031	3125 .		3472 .	3723	3549	2674
3086	2989	3778	3435	2814 .		3063 .	2843	3306	2344
3105	3730	3569	3285	3582 .		3042 .	3084	4074	2609
3403	3909	3024	4030	3272 .		2795 .	3055	3492	3408

3680	3030	2938	2361	3557	2433	2921	2845	3091	3448	3215	2644	2489
2664	2987	3086	3586	3813	2596	2806	2478	3078	2756	2778	2465	2952
2574	3506	2613	3514	2855	2570	2744	2396	2906	2505	2933	2998	2576
3196	3267	3163	3263	2410	3490	4144	4465	3344	3441	2778	3676	2469
4143	3004	4179	2367	3824	3796	3984	3988	3306	3034	2924	2459	2278
3035	3656	3217	2275	2850	2717	2846	4248	3048	2603	3375	2449	2506
2917	3779	2887	3423	2720	2540	2808	3058	3222	2656	3741	3464	2330
3683	3037	2854	3909	3581	3765	3879	2810	4362	2617	3162	2791	3030
4848	4006	4648	3493	4434	4077	3196	2577	3398	3645	3423	3245	3118
4266	4439	4640	3904	4133	3640	3382	3378	3842	3989	4674	3938	3738
4849	4250	3951	3512	3930	3557	4035	4362	4257	3957	4687	4240	3001
4487	4203	4482	3386	3755	3631	4509	4479	4499	3948	4450	3972	3033
4376	4218	3816	3741	3213	3850	4235	3875	3616	3598	3937	3405	3696
4238	3745	4285	3311	2872	2779	3487	3523	4039	4152	4748	3332	3744
3880	3762	3313	2275	2278	3156	4198	3548	4520	3888	4071	3106	3859
3710	3150	2845	2138	3738	2849	4455	2309	4846	3916	4091	3560	3674
3516	3592	3833	3537	3847	3325	3838	2438	4141	3674	4336	3326	3284
3559	3254	3519	3232	3469	2646	3318	3906	4258	4290	4106	3296	3391
3787	2915	3035	2180	2567	3657	4247	3544	4161	4139	3931	4205	3022
2735	2960	3234	3191	2374	3561	4275	2486	4436	3188	3812	4276	2866
2906	4726	3803	2634	2195	3205	3968	2455	4243	2583	3329	4070	2772
4472	3914	4326	2323	2355	2526	4025	2498	4311	3649	3367	3096	3081
4233	3952	4364	2442	3310	3354	3839	2497	4248	3484	3521	3287	3723
4129	4516	4263	2634	3911	3915	2839	4101	3883	3969	4445	3001	3070
3869	4315	3645	3533	3788	3297	3920	4197	4467	3946	4438	3010	2863
3115	4022	4285	3818	3617	2935	3989	2661	4366	3316	4115	4365	3636
3028	3921	3608	3297	3407	2637	3445	3591	4294	2945	3815	4032	3697
3990	3825	3067	3274	3190	2948	3114	3695	4330	3683	3552	3476	3183
3089	4158	3278	3180	2376	2854	3408	3514	3797	2884	2707	3196	2905
3229	3024	3128	3221	2123	2751	3248	2960	3659	3219	3027	2797	2902
3842	**3057**	**4239**	3371	2130	3145	3470	2982	3676	3605	3694	3023	2747
3067	**3021**	**3782**	3208	2187	2665	3403	2517	3287	2660	3421	3017	2936
3421	**2914**	**3106**	2899	2139	3162	3813	2336	3734	2961	3693	3003	3333
3388	**3797**	**3059**	2575	3360	3160	3501	2327	3354	3675	3239	3815	3596
2977	**3081**	**3902**	1974	2581	3605	3711	2339	3773	3409	3535	2571	3052
3264	**3546**	**3300**	1971	3179	2912	3421	3295	3710	2712	3875	3572	3052
4289	**2789**	**2922**	2862	3642	2779	3629	2390	3242	2833	3031	3228	2818
3652	**3331**	**4328**	3476	3960	2454	3705	3480	3262	2774	3072	2999	3389
3609	2879	3720	2386	3253	3633	3548	4061	3798	3589	3037	3468	3602
2999	3069	3674	2028	3865	2709	3132	3993	3515	2679	3292	3706	2703
2853	3905	3837	3879	3229	2097	3640	3547	3853	2673	3191	3069	2131
2734	3683	3095	3504	2605	3122	3688	3234	3244	3757	2889	3032	3450
3000	2747	2885	2008	2244	2103	2850	2570	3285	3252	3438	2601	2615
3135	3912	2681	2217	2168	2159	2681	2565	3468	2500	2783	2897	2449
2902	3052	2741	2231	2240	3071	3179	2280	3197	2638	2756	3027	2263
3493	2632	2782	3362	3303	2169	3425	2313	3098	2480	3218	2762	2435
3069	2707	3465	3274	3111	2005	2862	2354	3838	2506	3015	3409	2268
2744	3997	2750	2352	2434	2131	3822	3229	3320	3203	3010	2596	2482
3331	3196	2605	2211	2317	4020	2870	2187	2884	2441	3426	3985	3756
3117	2646	3607	2469	2253	3312	3210	4238	2932	2353	2573	3076	2666
2800	3653	3277	2580	2674	2351	2908	4124	2767	2497	2676	2807	2300
3394	2978	2856	2108	2228	2363	3288	3434	2936	3690	2689	2909	2496
3564	2607	3390	2251	2291	2247	2754	2458	2990	2876	3884	2867	2701
3059	2897	3926	2930	3168	2025	2424	2506	3575	2442	3446	2580	2499
2873	2589	2782	2180	2690	3339	2664	2284	2991	2580	2479	3453	2895
2959	2741	2793	2345	2480	2611	2307	2368	2949	2513	2866	2769	2448
3143	3326	2735	2226	2275	2225	2265	2913	3204	2541	3029	2551	2223
2756	3556	2762	3323	2262	3123	3024	2480	3054	2451	2719	2703	2423
4052	3028	2564	2823	3792	2910	3971	2748	2922	3494	2503	2847	2462
3834	2736	2498	1180	3098	2563	2496	2231	3354	2605	2755	2612	2644
3483	2943	2587		2646	3457	2367	4366	2829	2464	2487	3062	2453
3265	2743	3622		2695	3667	2264	3909	3903	2491	3331	2642	2741
3252	3222	3682		2238	2598	2328	3180	3940	2496	2919	2686	2689
3422	2737	2728		2370	2288	2306	2153	3114	2316	2608	3214	2558
3302	2811	2623		3758	2321	2518	2187	2772	2530	2797	2905	3075
3518	2850	3208		2939	4056	4521	2428	2584	3607	3196	2575	3362
3971	3773	3635		2300	2604	3222	3453	2921	2836	2696	2666	2420
3875	2881	2858		2934	2698	3441	4366	2755	2535	2550	3375	2374
2938	2767	2588		2590	2539	3280	3985	3410	2455	2907	3188	2416
2868	2726	2530		3515	2465	2619	2847	4187	2349	3770	2735	2557
2970	4147	3255		3565	2456	4024	2392	3040	3353	2988	2626	2957
2987	3251	3645		3111	3895	2968	2881	2874	3599	2662	2874	2338
3582	3022	2793		4547	4277	2445	3415	2941	2824	2645	3439	2640
3894	3378	2781		4544	3944	2441	2792	3157	2872	4009	3143	2624
5025	3709	4449		4113	3883	3813	3905	3993	3183	4061	3597	3332
4830	3599	4039		3941	3467	4762	4394	3902	3690	4770	4096	3612
4434	4152	3292		3597	3751	4391	4101	4136	3875	4064	4033	3655
3132	3838	4101		3303	3626	3575	3875	3583	3855	3633	3844	3652
3730	3747	3850		2679	3357	3915	3754	4077	3839	3392	2888	3438
3708	4383	3480		2364	2976	4670	2512	3441	2880	3752	3417	3539
4045	3454	3302		2177	2465	3872	3721	3327	3155	4404	3251	3234
3093	3863	3672		2191	2275	2711	4296	3704	2949	4127	2932	2853
3351	3353	3338		3011	3621	2339	3864	3227	2940	3761	3554	3338
3552	4649	3660		3427	2910	2384	3702	3189	3346	3232	4215	3158
3257	4097	2724		3742	3277	2045	2681	4502	3683	3445	4142	3112
3140	4537	3184		3701	2728	4418	2626	4085	3569	4364	3355	3090
4133	4086	3988		2968	2176	4629	2498	3886	3626	4086	3207	3173
3977	3851	3739		2126	2391	4222	2343	3555	3855	3441	3557	2727
4155	3754	3668		1920	2755	4068	4725	2961	3448	3052	3977	3555
3677	3443	3196		2017	2281	4431	3023	2824	3078	3315	3051	3647
3076	3197	2528		2142	2454	3599	2441	2987	2776	3426	3663	3390
2860	3753	2508		2106	2251	3567	2399	2753	3390	3405	2837	2863
3575	4101	2520		3282	2245	3392	2318	2790	3496	3605	3489	2635
2617	3134	3293		3599	3459	2802	2394	2710	3784	3940	2967	3179
3401	3649	4029		3799	3336	2606	2408	2822	2927	3254	3367	2732
3426	3671	3864		3011	3619	2398	2348	2950	2677	3491	2911	3335
3144	3303	2741		2118	3161	2188	3867	2958	2886	3940	3566	2709
3177	3096	2980		2023	3394	2371	3247	4026	2873	2802	3426	2733
2585	3145	2715		2269	2947	2138	2666	3611	3090	2831	2945	3031
2619	3071	2425		3282	2148	2471	2488	2903	2757	3340	2990	2927
2910	3299	3550		3884	1907	2397	2481	2905	3313	2836	2984	2430
2647	3205	4014		3081	1912	3815	2358	3214	3236	3355	3120	3183
3808	3146	3993		2641	1876	4467	2464	2976	2491	2985	2914	2775
3106	3790	3851		1910	3141	3142	3040	2938	2984	2998	2860	3663
2255	3889	3695		1909	3070	2294	3942	3721	2749	3489	3077	3130
2250	3744	4143		2007	3355	2305	4213	4478	2511	3743	4347	2354
2191	3457	3351		2064	2872	2977	4071	3849	2521	3705	4091	3029
2306	2850	3226		2073	1949	4671	3556	4041	2671	2787	2866	2332
3751	2722	2785		2215	1980	3736	2071	2616	3209	2547	2586	2042
2899	3821	2677		3343	1970	3363	2324	2897	2458	2887	2516	2755
2225	2933	2774		3097	2160	2397	2250	2684	2338	2476	2355	2288
2503	3219	2373		3245	2416	2073	2311	2676	2215	3344	3700	2323
2278	3054	2578		2910	2412	2010	2299	2610	2501	2515	2602	2469
2312	3234	3581		2297	2186	2348	2450	2920	2270	2343	2750	2129
2976	3071	3486		2197	2333	3406	4011	2887	2431	2583	2363	3099
2890	3088	2440		2167	2269	2243	3740	2885	2424	2826	2822	2397
2473	2768	2556		3287	2804	2286	2741	3770	2491	2298	2522	2253
2250	2692	2660		2616	1991	2269	2394	2915	3442	2422	2616	2252
3286	3862	2554		2514	2141	2328	2358	2712	3078	3698	2460	2390
2537	2738	3403		2297	3510	3113	2310	2938	2373	3686	2722	2150

3219	3736	2989 .		3997	3196	4184	4487	3982	3601	3525	3679	3622
3679	3042	3871 .		3614	3333	3722	4248	4129	3453	3284	3677	3485
3685	3982	2964 .		3291	3293	3646	3905	3445	2932	2591	3073	3196
3179	3168	3158 .		3495	2881	3315	3331	3613	3667	2817	3094	2892
3018	2904	3876 .		3413	2763	3367	3863	3173	3563	2717	2951	2598
3170	2943	2939 .		3101	3491	3084	3473	3112	3587	3592	3657	3397
2962	2952	2866 .		3151	2672	2700	3079	4026	2786	3040	2846	2935
2782	3136	3038 .		3169	3020	2827	2785	3180	2778	2633	2897	2766

3042	3200	3091	3484	3160 .	2458 .	3663	2633	2706	3444	3132	3739 .	2982	2800	3704	2634	3069	3260	2817	2613	2596
3291	3227	3128	3420	3328 .	3018 .	3401	2687	2559	2980	2942	3274 .	2823	2981	2636	3792	3050	2981	3431	2883	2865
3421	2943	3582	4083	3387 .	2801 .	3014	4141	2686	2719	3755	3096 .	3749	2690	2821	3387	3285	3095	2752	2885	2970
3358	2995	3775	3797	3218 .	3043 .	3415	3548	2539	3894	3611	2992 .	3336	3246	2855	3242	3962	3620	2977	2931	2646
3518	3800	3356	4122	4711 .	3737 .	3646	3338	3123	4186	3151	4362 .	4020	3688	2754	2643	3329	2942	2826	2863	2740
3995	4263	4200	4628	4382 .	3435 .	3783	2835	4048	4910	3448	4386 .	3685	3897	3736	3695	3173	3052	3954	3574	2996
4139	3325	4374	4510	4780 .	2730 .	3328	3478	4267	4308	3594	4214 .	3725	3683	3979	3861	3443	2948	4344	3910	3528
3580	3034	4321	4083	4187 .	3193 .	2917	3642	2820	4331	4042	3848 .	3439	3272	3226	3094	4844	4012	4366	3078	3168
3254	3218	4277	3745	4051 .	3588 .	3192	3041	2633	3937	4270	3871 .	3409	3398	3464	3384	4546	4226	4332	3157	3108
3938	4192	3315	4567	3957 .	3423 .	3675	3922	2606	3976	3964	4150 .	2973	3363	2771	3955	4262	4094	3531	3946	3004
4571	4268	3497	4366	3450 .	2729 .	3464	3048	2525	3821	4089	3849 .	3302	2917	2781	3841	3836	3949	3945	3256	3431
3987	3926	3072	3879	3792 .	3496 .	3076	3066	3568	3541	3509	3613 .	2707	3213	2908	3419	3323	3906	3486	3496	4142
3591	3934	3218	4131	2909 .	3630 .	3314	3515	3471	3649	3891	3589 .	3010	3542	2556	3681	3363	4112	2635	3367	3342
4004	4522	3333	3745	3203 .	2881 .	2702	4046	3784	3361	3158	3338 .	3262	3560	3739	3792	3275	3769	2794	3396	3118
3918	4133	3575	3580	4088 .	2717 .	2644	3995	3477	3291	3226	3016 .	2905	4098	3731	4159	3334	3707	2589	3451	3365
3654	3095	3149	4272	4401 .	2585 .	2651	4171	3270	3040	3139	3155 .	4045	2928	3008	3875	3084	3639	3212	3261	3231
3429	3173	3453	4769	3117 .	2547 .	2708	3778	3509	2675	2858	2897 .	4603	3402	2799	3139	4344	3678	2992	3456	2982
3967	3003	3523	4375	3007 .	2920 .	3462	3856	3553	3008	3153	2931 .	3757	3202	2728	3667	4096	2953	3059	4226	3759
3988	4242	3096	4673	2960 .	2675 .	2892	3481	3690	3966	2974	3023 .	3599	3171	3734	2992	3284	3215	2795	3707	3788
3997	3459	4477	4959	3023 .	3361 .	2706	3758	3458	4148	3092	4335 .	3207	2974	3729	3044	3350	3153	2805	3888	3345
3961	3059	4166	4209	2966 .	3935 .	2714	3487	2876	4476	3205	3660 .	3110	2928	3724	2689	3077	2633	4292	2736	2957
3660	3326	4109	4305	4349 .	3525 .	3476	3220	2741	3891	2876	3545 .	3266	2281	2729	2709	3108	2793	4200	3250	2910
3317	3044	4340	4527	4453 .	3168 .	3492	2900	3067	3309	3113	3996 .	4009	3015	2548	2824	4654	2749	3828	3687	3223
3382	3959	4250	4454	4320 .	2901 .	3273	2412	2699	3551	3558	4086 .	3484	2678	3251	3498	4742	3809	3881	3631	2872
3292	4265	3860	4009	3820 .	2698 .	3491	2475	2961	3185	4256	3163 .	3481	3479	3968	3714	4520	3890	3676	3244	3726
3927	3958	3414	4048	3401 .	3374 .	3188	2514	2480	3020	4223	3338 .	3339	3378	4012	2928	4500	2940	3690	3248	3555
3257	4029	3247	3991	3140 .	3366 .	2960	2999	2743	3129	3896	2740 .	3778	3009	3754	2858	4466	2511	3317	3259	3415
3506	3268	4140	3901	3000 .	3344 .	3018	2648	2906	3162	3829	2661 .	3187	2350	3460	2768	4193	2641	2742	3207	2848
4044	2922	3667	3342	2643 .	3118 .	2705	2627	2987	3017	3747	2890 .	3218	2201	3413	2971	3728	2472	2550	3896	3382
4125	3066	3539	3165	2872 .	3212 .	2219	2492	3739	2548	3822	2457 .	3257	2236	2515	2967	3231	3832	2428	3573	2839
4096	3372	3904	3329	3122 .	3226 .	3178	2923	3333	3478	3547	2671 .	3071	3288	3036	3637	3082	3873	2805	3328	2948
3972	4060	3757	3945	3533 .	2887 .	3564	2622	3679	2877	3520	3782 .	3459	3401	2504	3768	3276	3503	3184	3265	3244
3785	4256	3809	3381	3817 .	3060 .	2874	2566	3621	2585	2812	2827 .	2977	2965	3168	3439	3287	3348	3129	3760	3168
4329	3774	3439	3369	3804 .	2740 .	2426	3528	3262	2831	2803	2634 .	3161	2679	2849	2703	3240	2925	2506	3312	3809
3713	3806	3194	3222	3467 .	2960 .	2654	3626	3498	3721	2679	3432 .	2757	2268	2573	2919	3063	2630	2558	2924	3244
3345	3602	3390	4071	3901 .	2734 .	2922	3284	2835	3738	3461	4197 .	3177	2888	2703	2733	3018	2676	3691	3344	2707
3623	3675	3888	4374	4382 .	3757 .	3160	3321	3389	3507	2770	3918 .	3184	2548	3830	3386	3028	2595	3859	2959	3618
3577	3584	3358	3692	3685 .	2789 .	2718	3097	3325	3414	3518	3679 .	2498	2280	3367	3651	4109	3575	3373	2758	3424
3266	3087	3264	3690	3596 .	2527 .	2661	2410	2877	3509	3019	3365 .	2862	2596	3439	3494	3259	3512	2534	2757	2519
3519	3029	3080	3958	2829 .	2823 .	2670	2434	2368	3288	2822	2668 .	3007	2280	2419	2854	3199	2934	2452	3315	2860
3663	2793	3784	3853	2905 .	2787 .	3191	2645	2573	3049	2880	2454 .	3054	3056	2269	2789	3074	2674	2344	2891	2651
3032	3048	2947	3745	3620 .	3119 .	3201	2454	2591	3348	3390	2649 .	2636	2323	2712	2772	3908	2671	3009	3097	2814
3082	2955	3041	3440	3055 .	3199 .	2775	2611	2575	3132	3772	2413 .	2812	2857	3312	3071	4103	2490	2437	3125	2634
3307	3073	2637	3615	2818 .	2572 .	2893	2636	2697	3101	3915	2708 .	2632	2568	2457	2741	3700	3536	2544	3323	2421
3323	2848	3036	3484	2873 .	2441 .	2787	3343	2630	3042	3652	3248 .	2662	2815	2604	3181	3073	3178	2592	2866	2694
3093	2943	3381	3231	3225 .	2628 .	2893	3153	2493	2937	3430	2660 .	3168	3142	2508	3052	2893	2891	3240	2773	3212
3305	3041	2957	4189	3086 .	2783 .	2852	3035	2528	3178	2834	2853 .	2671	2839	2974	2723	3378	2627	3175	2757	2629
3805	3075	3838	3501	3331 .	3178 .	2831	2463	2297	2974	2802	3340 .	2727	2507	3040	2545	2844	3633	2559	3476	2484
4103	3088	3193	3344	3110 .	2611 .	3219	2588	3035	3077	3113	3465 .	3224	3272	3340	2677	3528	2768	2566	2489	2418
3372	2831	2966	3461	3061 .	3107 .	3270	2595	2713	3158	2825	3441 .	2547	2805	2530	3111	3991	2526	2569	2693	2886
3287	3294	3166	3718	3782 .	2683 .	3359	2474	2552	3327	2671	2996 .	2393	3495	3175	2536	3253	3222	2767	2732	2989
3278	3300	3137	3390	3668 .	2835 .	2864	3491	2764	3598	3135	3048 .	2428	3173	2740	2586	3171	2936	2610	3289	2614
3277	3140	2686	3777	2962 .	2793 .	2526	2751	2948	3140	2844	2718 .	2857	3163	2467	2524	3022	2845	2541	2683	2413
3872	3075	2700	4268	3400 .	2848 .	2505	2543	2788	2901	2888	3801 .	2710	2617	2657	2713	3080	3235	3169	2682	2777
3743	2973	3465	3496	2822 .	2617 .	3636	2479	2422	3186	2888	2950 .	2848	2717	2570	2393	2872	3483	3709	2934	2593
3336	2839	2964	3334	3334 .	2609 .	2799	2390	2510	3481	3432	3060 .	2649	2940	2781	3974	3564	2929	3118	3104	2727
3129	3066	3343	3741	3093 .	3130 .	2679	3364	3595	2946	2786	2818 .	3751	2572	3465	3731	3967	2860	2595	2874	2563
3181	3081	2824	3491	2906 .	2605 .	2603	2544	3045	3096	3879	3543 .	3484	2718	2527	3034	3451	2882	2431	2644	2551
3926	3113	3956	3385	4183 .	2684 .	2546	2472	2676	2898	3598	2690 .	3128	2656	2712	3217	3046	3157	2609	2790	2535
3181	2881	3965	3979	3168 .	2665 .	3018	2515	2595	3484	2861	3054 .	2814	2548	3613	2921	3079	2696	2411	2866	2768
3383	2852	3582	3617	3168 .	3331 .	2800	2838	2695	2917	3044	2836 .	2427	2315	2738	2869	3086	2983	3251	2853	3300
4062	4134	3156	3859	3914 .	2625 .	2453	3068	2544	3015	2904	2800 .	2587	3243	2610	3621	3280	3528	2658	3660	2668
3930	3111	3372	4488	3226 .	3166 .	3479	2693	2538	2849	3646	3060 .	2641	3020	2680	3547	3060	3427	2446	2876	2436
3373	3083	3100	3415	3152 .	2977 .	3353	2516	2773	2969	2910	3567 .	3032	2431	3168	2907	3035	2839	2600	2697	2848
3680	3128	3183	3518	3432 .	3383 .	3012	3324	3590	3172	2721	3353 .	2408	2386	2797	3121	3792	2763	3681	3146	2485
3332	3023	3547	3309	3131 .	3637 .	3191	3589	2964	3663	3745	2730 .	2810	3295	3029	2853	3913	2589	3860	3019	3161
3072	3818	3115	3605	3192 .	3183 .	3452	2703	2736	4171	3467	2772 .	3420	2644	3330	3539	3463	3641	3917	2931	2777
3193	3790	3104	4091	3960 .	3473 .	3219	2621	2501	3478	2928	3105 .	2809	2511	3055	3042	3025	2936	3636	3881	3252
4133	3300	3230	3782	4412 .	2700 .	3018	3738	2556	3090	3170	3049 .	2697	2803	3426	3090	3339	2687	3305	3050	2757
3728	3118	4461	4339	3527 .	3191 .	3638	3755	2691	4181	3230	4139 .	3810	3974	3036	3866	3521	3177	2874	2921	2799
3538	3816	4718	4852	4275 .	4020 .	4261	3043	3385	4590	2992	3597 .	4118	4032	3492	4410	3481	3253	3444	2979	2963
3957	4533	4326	5089	4495 .	3786 .	3990	3697	3857	4362	4430	4355 .	3855	3894	4690	4468	4248	4224	4132	3536	4034
4418	4149	4106	4684	4585 .	3434 .	3665	4105	4214	3961	4383	4972 .	5195	3903	4499	4127	4488	4169	4109	4156	3350
4495	4267	4387	4607	3846 .	3712 .	3528	3860	3465	3963	3967	4486 .	3552	3727	4293	4168	4516	4031	3898	3124	3622
4216	4045	4041	4547	4523 .	3692 .	3694	4254	3102	3543	4148	4580 .	3441	3487	4303	4134	3316	4023	3904	3569	3198
4218	3990	4312	4578	4045 .	3436 .	3620	4531	2925	3215	3818	4792 .	3228	3590	3704	3308	3917	3955	3873	3663	3051
4113	3276	3865	3798	3833 .	3391 .	4163	4015	3146	3324	3887	4329 .	2864	3449	4253	3914	4704	4085	3635	3291	2750
4286	3374	3415	3502	3860 .	2622 .	3875	3842	3337	3177	3854	4128 .	2688	3910	4182	3780	4451	4007	3056	3593	3102
4305	3723	3095	4931	3997 .	2439 .	3651	3133	2842	3275	2911	3814 .	2402	2907	3912	3775	4168	3519	3033	3055	3575
4517	4473	2983	5039	3976 .	3082 .	3796	3138	2884	2990	2851	4362 .	2654	3176	3210	3959	3756	3708	3021	3209	3420
4438	4008	3043	4817	4165 .	2563 .	3349	3983	3894	2959	3599	3279 .	2507	2504	2758	3299	3807	2982	3162	3239	3319
4385	3336	3431	4802	3586 .	2815 .	3512	3634	3679	3200	2895	2994 .	4127	2952	2661	2958	3505	2732	3267	3186	3089
4388	3525	3228	4440	3246 .	2656 .	3447	3821	3703	2668	4003	3547 .	3941	2632	2708	2830	4822	2798	4297	3773	3122
3980	5658	3074	4116	2911 .	2550 .	2490	3706	3696	2854	4088	3398 .	3685	2901	4334	2774	4657	3360	3907	3955	3106
3803	3806	3320	4074	2830 .	2607 .	3506	3647	3523	4118	3934	3772 .	3780	3387	4254	3301	4556	4443	3659	3466	3320
3903	3942	4507	4068	4261 .	3974 .	3770	3140	4000	4146	3848	4011 .	3270	3488	4022	4215	4911	4082	4049	3306	3384
3916	4076	3971	3238	3918 .	3355 .	3574	2982	3278	3853	4350	4232 .	2711	3358	3286	3815	4154	3835	3421	2802	3127
3419	3809	4209	3132	2836 .	3132 .	3372	3256	2732	3313	3777	3738 .	2384	3056	3023	3533	4266	3687	3592	3278	2958
3998	3763	3879	4007	2900 .	3339 .	3171	3174	2568	3146	2852	4326 .	2419	2452	3405	3012	3544	3787	2886	3884	2984
4366	3520	3802	3964	2913 .	3391 .	3438	3074	2277	2864	3541	3612 .	2421	2868	4429	3033	3089	3083	2618	2747	3530
3989	4825	3642	3832	4093 .	2977 .	3450	3686	2450	2899	4103	3514 .	2668	2509	4180	2817	3523	2975	2437	3206	3366
4079	4006	2909	4759	4125 .	2665 .	3115	4395	2693	3443	3813	3001 .	3178	2484	3727	3564	3483	2842	2993	2966	3440
4302	3326	2917	4601	3833 .	2402 .	3042	3723	3122	3905	3599	2745 .	3166	2421	4095	3087	4232	2938	3000	2983	3239
4310	3610	3382	4470	3561 .	2478 .	3172	2789	3691	3359	2680	3182 .	3231	3219	4001	3047	3896	2743	2521	3231	2975
4123	3531	2877	4103	3067 .	3158 .	3607	3036	3444	2725	2801	3882 .	2519	3416	3515	3509	3471	3602	2577	2836	3132
4470	4409	3535	4271	2967 .	2556 .	3274	3164	3380	2773	3268	2732 .	2681	3735	2944	2749	4457	2962	3814	3062	2953
4610	3494	4311	3469	3507 .	2720 .	3928	2604	3847	2723	3292	2802 .	3592	3775	3249	3134	4270	2827	3782	3011	3276
4096	3773	3696	4123	4046 .	2364 .	3326	3424	3268	3041	2533	2599 .	3890	3837	2965	4255	3729	3747	3411	3002	2908
4114	3381	3879	4424	4176 .	2510 .	2952	3608	3762	2890	2789	3686 .	4146	3253	4147	3955	3017	3831	3412	2890	2769
3943	3681	3991	4173	3548 .	3184 .	2703	3433	3412	2864	3767	4191 .	3656	2862	3826	3162	3000	3632	2483	3630	3128
3347	3564	3852	3945	3063 .	3562 .	3346	3261	3229	2445	3750	3914 .	3237	2229	3008	3275	3278	3372	2535	3782	2669
3750	3650	4302	3315	2827 .	2919 .	3426	2389	3673	3677	2725	3396 .	4060	2513	2984	2759	3926	3594	2382	3574	2910
4484	3569	3633	4125	3825 .	2976 .	2986	3599	3433	3650	2807	3169 .	3197	2302	2738	3649	2988	3748	3364	2779	3311
4046	3350	3380	3817	4660 .	2714 .	2945	3687	2734	3585	3755	2709 .	2899	2383	3527	2959	3041	3656	2437	3649	2554
3676	3110	3039	3211	3773 .	2366 .	3227	2802	2971	3172	3743	2596 .	2692	3087	2743	2598	2976	3439	2558	2585	2878
3719	3170	2870	3139	3670 .	2807 .	3079	2689	2487	3224	3492	2413 .	2380	3636	2756	2787	3982	2898	2521	2655	2647
3657	3192	3123	3245	3337 .	2542 .	2943	2614	2817	2752	3108	2784 .	2431	3066	3446	2594	3813	3228	2853	2845	2698
3244	3121	2904	3130	2812 .	2301 .	3009	3208	2651	2780	2877	2867 .	2908	2190	2993	2867	3215	2742	2596	2824	2318
3603	3213	2940	3802	3308 .	3543 .	2653	2533	2433	3134	3335	3363 .	2655	2405	2856	3035	3077	2709	2478	2956	2631
3534	2880	2940	3898	2754 .	2876 .	3431	2569	2449	2868	3044	2614 .	2455	2115	3449	3089	3378	3201	2732	2794	2516
3419	3187	3004	3543	3105 .	2494 .	2915	2663	2822	2888	2776	2625 .	3476	2318	3752	3377	3040	3158	3010	2967	2471
3349	2912	3326	3254	2983 .	2678 .	3082	3606	2655	3136	2842	2867 .	3345	3280	3040	2749	3139	2761	2533	3046	2374
3956	2924	3952	3976	2835 .	3603 .	2863	3449	2555	2711	3064	3826 .	2843	2583	2896	3153	4182	2951	2376	2617	3295
3748	3409	3002	3839	3385 .	2791 .	3422	2869	2652	2751	3782	3466 .	2675	2601	3131	2556	3692	3260	2352	2772	3075
3391	3365	2954	4308	3173 .	2751 .	2902	2794	3442	3482	3153	2994 .	2642	2543	3795	2630	3308	3350	2758	2781	2638
3618	2846	2886	3398	3164 .	2777 .	3214	2681	3015	4022	2663	2746 .	3015	2451	3863	3219	3253	2754	3859	2632	2564
3658	2871	2918	4569	4638 .	2445 .	2890	2597	2500	3418	3485	3014 .	2689	2293	3006	3070	3800	2868	3446	3274	2756
3835	2878	3065	3193	3190 .	2608 .	2575	2823	2514	3046	3865	3364 .	2658	3233	3094	3539	3265	2777	3090	2782	2351
3352	2540	3064	3995	3615 .	2918 .	3205	2657	2823	2837	2905	3157 .	2624	3391	2925	2620	3280	3178	2600	2611	2456
3335	2793	3114	3560	3186 .	3036 .	3504	2469	2834	3039	2917	3039 .	2518	2735	3313	2878	3115	2940	2575	2656	2998
3645	3195	3152	3291	3633 .	2683 .	2929	3208	3037	2744	3391	2753 .	2391	2732	2774	2654	3154	2624	2591	2810	2767
3530	3855	3333	3575	3777 .	2511 .	3102	2823	2759	2818	2988	2745 .	2922	2604	2799	3370	4180	2999	3604	2934	2567
3516	3062	2896	3426	4130 .	2430 .	2700	2649	2504	2963	2915	3546 .	2623	2585	2807	3121	4241	3019	2587	2742	2548
3591	3032	3048	3425	3128 .	2490 .	2757	2652	3175	3305	3620	3070 .	2476	2482	3290	2828	3333	2765	2690	2699	2994
3812	3059	3054	3637	3130 .	3161 .	2762	2644	3021	2984	3751	2909 .	2456	2950	3030	3079	3332	2981	2538	3028	2737
3479	3047	2881	4128	3041 .	2956 .	3376	2734	2557	2588	2574	2699 .	3240	2751	2811	2794	3326	3165	2732	2723	2567
3540	3021	3503	3535	2887 .	2409 .	3142	2483	2522	2735	2865	3254 .	2742	3408	3162	2867	3995	2982	2812	2678	2715
3987	3463	3405	3515	4270 .	2576 .	2926	3241	3160	2707	2836	2647 .	2655	3369	2978	3100	3624	3491	2992	3283	2635
3638	3043	3188	3438	3028 .	2284 .	3170	2847	2788	4106	2634	2885 .	2928	2720	3186	2837	3224	3463	3192	3050	2784

3585	2728	2915	3607	3278 .	2669 .	2660	3587	2738	3540	3768	3169 .	3092	2843	3712	3386	3265	3128	2655	2771	2599
4580	2702	3009	3457	3481 .	3536 .	2830	3227	2678	3272	3698	4192 .	2596	2799	3366	3339	3334	2962	4236	2890	2800
3847	2667	3091	4199	3332 .	2783 .	3283	2981	2538	2849	3220	3518 .	2544	2530	3056	3872	4219	3710	3425	2854	2734
3739	3291	3469	3923	3339 .	2812 .	3149	3276	3125	2968	2865	3173 .	3582	3005	3100	3716	3543	3064	3025	3015	2545
3828	3429	3086	4263	3317 .	3015 .	3040	3516	2984	3674	3123	3484 .	2872	2737	3236	2865	3382	3497	3015	2869	2705
4240	3064	3899	4055	4480 .	3063 .	4241	3791	3268	4332	3069	3182 .	3067	4162	3251	2920	3528	3190	3701	3276	2925
3898	3077	4598	4389	4441 .	4049 .	4262	3459	2988	4883	3289	4513 .	4012	4511	3297	4158	3618	2772	3208	3085	3801
4083	4630	4878	5317	4719 .	4042 .	4463	3448	3535	4612	4299	4799 .	3756	4153	3989	4047	3658	3007	3787	3781	3542
4459	4189	4371	4820	4823 .	3919 .	4656	3941	4323	4385	4272	4212 .	3636	3987	4879	3952	3726	3895	4174	3589	3102
4300	4303	4006	4525	4093 .	3599 .	4254	4209	3807	3631	4032	4260 .	3465	3851	4614	3624	4066	3953	4188	3687	4000
4454	4166	4102	4689	4072 .	3515 .	4268	4633	3949	3759	4469	3941 .	3764	3617	4004	3920	4041	3887	3781	4002	3891
4422	3797	4160	4423	4124 .	3548 .	4061	4054	4049	3447	3866	3134 .	3170	3830	3784	3624	4379	3719	4057	3676	3378
4237	3197	3835	4122	3295 .	3502 .	4958	4198	3771	3357	3279	3860 .	3198	3184	3980	4000	4375	3996	3668	3589	2636
4852	3043	3262	4327	2976 .	3441 .	4807	4422	3514	3202	4281	3195 .	2888	3451	4317	3813	3951	3067	3754	3118	2974
4740	2869	3152	4236	4143 .	3306 .	4813	3820	3700	3376	3824	3166 .	2699	3343	4275	4149	4553	3064	3219	4241	3115
4386	4132	3034	3794	3950 .	3163 .	4039	3788	3404	2957	3972	3717 .	2415	3610	4203	3698	3896	3490	2988	3253	4030
4463	4210	4192	4746	3868 .	2639 .	3508	4266	3465	2825	3194	3826 .	2714	3671	4124	3133	4312	3790	2666	3469	2757
4595	3733	4391	4768	3287 .	3118 .	4112	3245	4256	3338	3139	4136 .	2689	3423	4068	3510	4388	3682	3448	3257	3179
4242	3735	4319	5017	3881 .	2647 .	4029	3751	3077	4134	3187	3946 .	3761	3200	3732	3904	4332	3953	4176	3344	3076
4104	3513	4044	4724	4320 .	2633 .	4012	3575	3047	3053	3744	4042 .	3531	3112	4063	3730	4078	3726	4137	4479	3609
4340	2791	3804	4679	3535 .	2391 .	4516	3535	2888	2737	3260	3703 .	3413	2788	4036	3748	4469	3251	3861	4080	3428
4403	2797	3954	4467	3480 .	3726 .	4616	3628	2800	2681	3313	3758 .	3359	3865	3538	3179	4316	3537	3737	3230	2942
3961	3037	3575	4635	3336 .	3100 .	4501	3656	4251	3572	3573	4082 .	3031	3645	3646	3635	3764	2717	3676	3310	3330
4291	2845	3072	4517	3603 .	2920 .	4271	3893	3997	3445	3216	3949 .	2991	3563	4059	3725	3992	4016	3930	3660	2729
3958	2717	3330	5161	4748 .	2393 .	4458	3534	3728	3056	3010	3664 .	3052	3593	4062	3670	3770	4121	3877	3472	2950
4281	3020	3286	4493	4422 .	2883 .	4013	3560	3129	2792	3058	3329 .	2448	2818	3800	3152	4039	3963	2865	3188	2933
4468	4313	2944	4500	4057 .	2301 .	4168	3325	3048	2759	3757	3953 .	2819	3270	3365	3457	3591	3814	3238	3349	3122
4175	4208	2860	4105	3274 .	2662 .	3834	3599	3175	2818	3972	3918 .	2931	2972	3835	3185	3818	3967	3566	3193	3078
3991	3990	2920	4693	3162 .	2974 .	3494	3340	3224	2820	3784	3140 .	2405	3290	3585	3678	4284	3679	4006	3351	3177
4103	3614	3102	4761	3602 .	3945 .	3542	3330	4451	2782	2841	3201 .	2279	3203	3477	3268	4031	3493	3437	3072	3106
3867	3860	3117	4313	3332 .	3561 .	3882	3553	3722	2744	3767	3803 .	2428	3374	3826	3537	3784	3449	3406	4525	3098
3934	4066	3969	4398	3570 .	3177 .	3956	3907	3943	3210	3504	3406 .	2220	3469	3621	3602	3637	3032	4129	4035	3537
3976	3537	4598	4423	4314 .	2563 .	3660	3695	3539	3404	4000	3443 .	2590	3352	3800	3265	4109	3451	2929	3713	3292
4132	3570	4067	4288	4067 .	2493 .	3462	3089	3569	3182	4575	3517 .	2357	3440	3825	4087	4075	3954	2962	3413	3311
3878	3497	4097	4244	3751 .	2567 .	3326	3201	3413	3582	4054	3699 .	4250	3462	3662	3335	3890	3292	2759	3463	3317
3961	3545	3940	3858	3122 .	2816 .	3315	3769	2799	2874	3747	3504 .	3738	3131	3812	3311	4174	2742	2815	2992	2914
3879	3515	3724	3989	3064 .	2798 .	3077	3268	2501	3112	2851	3232 .	3242	3054	3540	3334	3664	2937	3654	3234	3510
3576	3116	3897	3649	3021 .	3514 .	3635	3053	2515	2506	2949	2855 .	2951	3543	3476	3327	3565	2863	2982	3932	3489
4606	2809	3447	3987	4103 .	3481 .	2944	3389	3071	2765	2758	3210 .	3177	3356	4081	3275	4106	3491	2581	2952	2582
3832	2585	3095	3478	4181 .	2990 .	2472	3087	2688	2532	3529	3511 .	2772	3081	3618	3159	4097	3660	3044	4055	2442
3834	2794	3599	3579	3637 .	2867 .	3368	3556	2502	3273	2719	3580 .	2381	2889	3526	3093	3801	3437	2596	3275	2730
3322	3271	3413	3810	3624 .	2402 .	3067	3102	3012	3317	3049	3210 .	2342	2783	3388	3442	3822	2871	2835	2812	2979
3853	2835	2869	3429	3409 .	2363 .	2810	3282	3300	3294	3527	2915 .	2884	2638	3361	3413	3543	2979	2786	2624	2305
4259	2758	2847	3453	2904 .	2376 .	3367	3241	3014	2880	2748	2745 .	2604	2353	3122	3285	3371	2799	3233	3055	2476
3831	2646	2888	3736	2857 .	2444 .	2877	3019	2930	2843	3254	2576 .	2261	2314	3534	3621	3152	2743	2714	2984	2659
3805	2620	2945	3481	3287 .	2786 .	2736	3135	2663	2661	3001	3347 .	2480	2322	3041	3382	3468	3118	3507	2615	2541
3497	3405	3357	3428	3079 .	2521 .	3003	3504	3260	2683	2975	2911 .	3461	2931	3733	2728	3668	2802	3297	2524	2543
3609	2610	2871	3374	3564 .	2434 .	3358	3137	2938	2895	3773	2893 .	2979	2519	2839	3154	3335	3032	3027	2826	2446
3446	2836	2889	3428	3040 .	3185 .	2814	2855	3021	2696	3567	2650 .	2664	2777	3087	2836	3365	3395	2979	2964	2264
3587	3204	2888	3443	3236 .	2627 .	2664	2757	2748	3492	3024	2628 .	2474	2325	2713	2370	3140	2588	3010	2926	2841
3816	3200	3399	3258	3273 .	2650 .	3289	2840	2603	2778	3078	3419 .	2990	2268	2514	2872	3021	3006	2917	2693	2681
3514	2851	2985	3458	4139 .	2444 .	2584	2914	2493	2503	2844	3079 .	2714	3348	2639	2600	3273	2762	2768	2817	2433
3786	2884	2920	4840	3720 .	2409 .	2956	3114	2798	2470	3347	2688 .	2672	3122	2717	2736	3309	3686	3046	2639	2632
3748	2589	3047	3614	3209 .	2630 .	2673	2725	2508	3167	3442	3181 .	2422	2440	2610	2297	3387	2999	2853	2811	2316
3334	3231	3316	3262	3125 .	2575 .	2538	2630	2508	2682	3066	2866 .	2470	2713	3374	2318	3406	3049	2924	3094	2542
3469	3044	3126	3300	3154	2880 .	2435	2643	2721	2708	2836	3195	3064	3127	2962	2740	3808	2979	3016	2563	2601
3360	2979	2917	3872	3552	3308 .	2994	3740	3045	2651	3017	3099	2698	2597	2597	2353	3118	2898	2774	2561	3014
3560	3023	3145	3397	3218	3227 .	2978	3316	2877	2762	3008	2791	2609	2500	2491	2634	3313	3262	3714	2559	2527
3723	3782	3159	3340	3393	2908 .	2693	3074	2932	2908	3189	3201	3153	2564	2618	2842	3828	2922	3905	3290	2431
3532	3114	3751	3402	3327	2720 .	2570	2797	2838	2899	3055	3441	3340	2509	2621	2658	3280	2756	3481	2638	2573
3675	2831	3651	4245	3546	3210 .	2612	2614	3068	3420	2961	3183	2865	2430	2361	3783	3256	3216	2807	2611	2852
3947	2974	3575	3540	3173	2604 .	2585	2836	2641	3249	3869	3005	3207	3296	2916	3485	3584	2968	2577	2746	2714
3765	3293	3163	3284	3368	2650 .	2538	2748	2954	2800	3521	2683	2675	2828	3616	3047	3237	3204	2611	2979	2505
3797	2832	3020	3161	3797	2628 .	3036	2878	3052	2793	2858	3382	2896	2582	3395	2990	3205	3523	2934	2552	2322
3417	2827	3418	3910	4067	3023 .	3099	2983	3173	2693	3107	3469	2695	2880	2837	2718	3321	2991	3437	3003	2577
3529	2839	3292	3370	3191	4132 .	2701	3522	3432	3195	3303	3158	3603	3261	2884	3178	4076	3203	2989	2815	2472
3906	3258	3113	3379	3606	3292 .	2544	2893	3553	2892	3318	2881	3226	3265	2698	2926	3295	3063	2690	2582	3077
3761	2818	3658	3318	3075	2962 .	2593	3094	3326	2798	2896	2782	3905	3047	2647	2759	3119	3670	3076	3440	2682
3982	2760	3884	4043	3594	3386 .	3336	2987	3496	2946	3263	2781	3154	3337	2896	2684	2913	3644	3016	3098	2506
4178	3258	3324	3385	3999	2877 .	3239	2902	3183	3069	3288	2886	4423	3277	3073	3676	3857	3215	3428	2792	3118
3777	3297	3625	3476	3584	3980 .	2595	3797	2973	4368	3112	2775	3407	3220	4268	2941	3267	3125	3154	3203	3068
4038	3264	4493	4046	3889	3906 .	3959	3985	2859	4742	3711	3529	4214	3367	4050	3560	3125	3323	2980	3108	2515
4189	3706	4833	5437	3636	4145 .	3871	3401	4054	4849	4108	4804	4485	3961	2924	3787	3662	3907	3820	3367	2782
4421	4669	4287	4716	4919	4059 .	3745	4062	4038	4597	3540	4196	4227	3825	3624	3936	4323	3939	4098	4029	3257
5043	4055	4574	4492	4606	3961 .	3503	4218	3584	4540	4168	4272	3550	3531	4348	3721	4330	4152	3735	4126	4246
4628	3972	4433	4217	4691	3409 .	3517	3805	3354	3838	4329	3857	3316	3397	3805	3762	3880	4207	3904	3962	3671
4222	4213	4086	4248	4076	3341 .	3550	3560	3492	3888	4069	3699	3485	2821	3792	3280	4016	4107	3259	3493	3169
4669	3958	4311	4637	3734	3712 .	3246	3367	3256	4332	4083	3483	3008	3718	3514	3686	4187	3665	3689	3482	3089
4594	3997	5055	3536	3915	3000 .	2745	3773	3234	3953	3923	3625	3758	3666	3574	3163	4734	3711	4180	3857	3109
4512	3579	4519	4204	3366	2871 .	2515	4391	3825	4042	3557	3771	3435	3295	3275	2591	4272	3751	3469	3946	3172
4159	4130	3373	4186	4037	3119 .	3630	3892	3684	3742	3655	2914	3279	2740	3670	3488	4227	3309	4318	4148	3040
4578	3776	4024	3587	4838	2622 .	3283	3385	3109	2947	3528	2706	3223	2528	2955	3422	3986	3433	3477	4088	2878
5024	3296	3576	4777	4464	3582 .	2734	3359	2835	2858	3704	2920	2687	2701	2798	3672	3726	3997	3400	3396	3643
4973	2754	3120	4398	4497	3651 .	2836	3865	3511	2730	3690	2659	2463	2486	2759	3515	4811	4180	3596	3194	3978
4407	2942	3509	4701	4647	3637 .	2582	3629	3269	4110	4621	3328	2919	3048	4082	3685	4762	4206	4139	3991	3240
4685	2784	4341	4314	4275	4076 .	3014	3732	3348	3870	4440	4114	3004	3044	4299	3087	4297	3921	3984	3848	2840
4249	2838	4567	3975	4025	3485 .	3666	3544	3730	3862	4298	4063	4420	3147	5057	2807	3773	3761	3963	3380	2991
4097	4406	4159	3884	3839	3197 .	4301	3061	3514	3700	4323	3743	4329	3191	4857	2475	3711	3845	4109	3241	2920
4248	3894	4062	4044	4129	2746 .	3387	3025	3020	3307	4386	3731	3462	3345	3848	2805	3576	4131	3731	3206	3392
4212	3634	3109	4169	4346	2669 .	3339	3114	3333	2985	3998	2618	3141	3616	2978	2709	3573	3532	3447	3591	3113
4474	3727	3044	3990	4031	2441 .	3494	2865	3737	3250	3987	2505	3116	3461	2524	2802	4711	3675	3273	3369	3093
4271	3642	3110	3713	3890	2121 .	3084	3205	3228	3624	3804	2683	2914	3387	2544	2544	4245	3756	3250	3303	3234
4362	3708	3321	3175	3728	2720 .	2997	3750	3335	3133	3219	2840	2748	3704	2840	3591	4006	3369	3708	3961	2978
3998	2909	3334	3334	4128	2956 .	3099	3594	3589	2968	3829	3771	2458	2984	2965	3519	3266	3875	3944	3132	3252
3907	2891	3061	3418	3588	2494 .	2627	3124	3368	2974	3797	4203	2354	2575	2796	2704	3303	3785	3838	3411	3059
4242	2758	4132	3348	3738	3483 .	2615	2874	3337	3509	3258	3492	2633	2608	3229	2819	3478	3968	3701	3059	3467
4475	3680	4675	3390	3779	3554 .	2374	2773	3358	3146	4296	3462	3828	2138	2681	3383	3353	3261	3429	3553	3228
4540	3732	3871	5086	4118	3346 .	3751	3273	3395	3264	3387	2659	3913	2566	3123	3887	4468	3504	3270	3682	3430
4179	3442	4252	3803	3877	3322 .	3379	3818	3596	3246	3802	2874	3774	3254	2679	3412	4379	3812	3216	3581	3564
3763	3660	4111	3106	4113	2656 .	2837	3872	3514	3181	2950	2860	3875	3558	2917	3675	3256	3533	2990	3295	3425
3719	4164	4197	2915	3833	3316 .	2037	3635	3304	3250	3093	4215	3491	3258	3874	3646	3434	3258	3197	3090	3089
3911	3557	3636	4947	4130	4001 .	2352	3232	3057	3075	3747	3989	3060	2691	4032	3061	2978	3120	2791	3377	2862
3801	2554	3214	4364	3318	4070 .	2906	3571	3055	2840	3334	3727	2761	2282	4836	2814	3545	3127	3551	3051	2571
4004	2510	3868	3954	3969	3539 .	3412	3092	2805	2725	3100	3322	3024	2932	4741	2991	4002	3274	3363	3927	3455
4014	3252	4062	3386	3562	2568 .	2878	3970	2808	2759	3754	3420	2836	2352	4009	2968	4844	3576	3458	3586	3296
3553	2716	3271	3598	2995	2641 .	2794	3208	2866	3687	3657	3301	2802	3033	3798	2808	3860	3439	2847	3115	2773
4226	2930	3163	3460	3151	2601 .	2435	2840	2967	2892	3481	2664	2720	2307	3276	3603	3555	3205	2734	2862	2567
3920	2848	3031	3377	3364	2457 .	2223	2713	3060	2628	3102	2457	2527	2127	3748	3373	3248	3361	2837	3114	2472
3693	2743	2841	3185	3614	3121 .	2216	2658	3295	2558	3303	2874	3178	2814	3169	3157	3096	3018	2792	3164	2652
4037	3419	3160	3653	3837	2642 .	2698	2661	3851	2474	3126	2617	3464	2465	2712	3035	3286	2779	2728	2742	2430
3671	2788	3116	3778	3308	2760 .	2261	2522	3063	2848	2800	2720	2843	2542	2496	2824	3646	3224	2683	2598	2453
3768	2774	3452	3137	3094	3239 .	2884	2481	3102	2903	2951	2738	2797	3069	2764	2765	3938	3549	3440	3351	2434
3552	2795	3128	3150	3114	2762 .	2769	2574	3172	3323	2921	2624	2670	2779	2791	2805	3641	2899	3024	2678	2780
3457	2802	3140	3243	3033	2874 .	2435	3213	2572	2823	3756	2844	4142	2452	2785	2828	3291	3417	2778	2946	2463
3728	2678	3170	3222	2816	2497 .	2766	2896	2362	2511	3085	2748	3319	2392	2633	3315	3308	3199	2836	3035	2405
3750	3512	3139	3165	3226	2434 .	2482	2754	2954	2579	3112	3639	2661	3377	2609	3073	3081	3053	2798	2781	2658
3836	3755	3214	3275	3069	3336 .	2619	2699	2538	2493	3370	3283	2740	2877	3057	2897	3620	2961	2623	2778	2498
3412	2867	3088	3466	3070	2969 .	2742	2916	2270	3294	3291	2916	2862	2617	2743	3126	3383	3646	2797	2539	2516
3532	2797	2872	4164	3513	2893 .	2360	2772	2553	3238	3089	2559	2773	2629	2770	3142	3269	3228	2752	2994	2456
3577							2602	2810						2576	3158	3154	3290	2647		
3708							3123	2828						2934	2351	3268	2997	3038		
3739							2796	2557						2978	3567	3098	2872	2749		

1	1	2	2	5	6	6	8	9	11	1	2	2	6	6	8	9	9	9	11	11	11	11	1	1	1
Box-4	Box-5	Box-2	Box-3	Box-1	Box-2	Box-3	Box-8	Box-7	Box-5	Box-6	Box-6	Box-8	Box-8	Box-6	Box-5	Box-1	Box-2	Box-6	Box-1	Box-2	Box-4	Box-6	Box-7	Box-8	Box-2
10.1	11.6	16.4	17.4	28.1	41	42	53.2	71	82	12.1	23.3	27.5	37	46	50.1	62	63	69	74	76	81	83	1.1	2.2	5.5
M	M	M	M	M	M	M	M	M	M	M	M	M	M	M	M	M	M	M	M	M	M	M	M	M	M
EtOH	EtOH	EtOH	EtOH	EtOH	EtOH	EtOH	EtOH	EtOH	EtOH	H2O	H2O	H2O	H2O	H2O	H2O	H2O	H2O	H2O	H2O	H2O	H2O	H2O	Malto	Malto	Malto
ME	ME	ME	ME	ME	ME	ME	ME	ME	ME	MH	MH	MH	MH	MH	MH	MH	MH	MH	MH	MH	MH	MH	MM	MM	MM
3403	3519	4208	2050	3006	1866	1499	2859	1879	3775	3361	2169	2805	3495	2745	1830	1788	2603	1760	3984	3111	3360	3637	.	3411	3138
3533	3552	3796	3041	2379	2382	1787	1823	2004	3626	4154	2014	2614	2501	1894	3056	2100	2325	3061	3691	2430	2829	3386	.	3533	3411
2353	2979	2778	3650	2493	3521	2203	1691	1930	3142	3882	2197	2471	3026	2086	1894	2326	2044	2387	3765	2063	1970	2329	.	3465	3686
3766	3537	3245	3364	3869	3382	2986	1759	1897	2026	3316	2545	3471	2215	3359	2645	3344	2314	2957	3912	3107	1987	3230	.	3588	2580
3365	2594	2327	2896	3766	1981	2329	2712	2122	3430	3670	3806	3356	3124	3406	2430	3540	3219	3556	3345	3136	3619	2244	.	3317	2409
2517	3664	2063	2571	3502	3465	2215	2521	2816	2228	3284	3415	2409	3078	3374	1932	2373	2404	2796	2427	2091	3608	2208	.	3367	4049
3215	3853	2077	1967	3115	3710	2258	1483	2016	2006	3754	2107	2373	3110	3155	3073	3200	3879	2064	3728	3045	3182	2068	.	3483	3817
3851	3908	3001	2439	3403	3435	3074	3419	3244	2002	3044	2859	2611	2959	2697	1859	2564	3379	2888	3721	2490	3097	3025	.	3472	3386
3651	3883	3991	2158	3434	3642	2925	3582	2709	2772	2829	3129	3350	3545	3025	2157	2107	3277	3557	3926	1961	2278	3680	.	3481	4097
3769	3515	3543	2052	3463	2870	2976	3314	2319	3406	3363	2274	3180	3534	3352	3367	3841	3696	4201	3932	3180	4027	3469	.	3703	3728
3654	3846	3785	1976	3249	3142	2994	3405	1620	3205	3650	2078	2422	2626	3558	2772	3946	3808	3713	2354	2797	3564	2874	.	3533	3922
3781	3328	3657	1941	3109	3490	3110	3477	3023	1825	2475	3898	2143	2830	2632	2699	3960	3680	3697	2966	1754	3462	3459	.	3491	4283
3660	2807	3516	2889	2463	3769	2540	3258	3112	2699	2303	4097	3480	3145	2914	2430	4037	3581	3819	4119	2806	3668	4279	.	3436	4018
3588	3033	3659	3671	2679	2368	1920	3563	3037	3228	3800	3792	2581	2339	2088	2110	3884	3690	3821	4195	1803	3303	3393	.	2495	4329
3553	2644	3477	3613	1968	2628	2238	3625	2186	3196	3381	3993	2137	3257	2349	2560	3879	3610	3903	3654	2772	3436	2518	.	2628	3898
3364	3152	3645	3284	2784	2996	2977	2896	1761	3606	3251	4206	2396	3528	2600	2722	3530	3609	3655	2578	3194	3660	2673	.	3295	4189
2748	2813	3614	3036	2323	2097	2113	3130	2368	3568	3861	3756	2367	3568	1974	2910	3877	3576	3657	4485	2407	3591	2873	.	2261	4093
2740	2907	3553	1942	2137	2004	1996	2433	1609	3638	3609	2451	2420	3571	1702	2677	3912	3616	3625	4457	1643	3669	2804	.	2774	3851
2595	2692	2583	1736	3148	2173	2507	1456	1937	3504	3590	1998	3740	3081	3354	2715	2871	3105	3477	4267	1630	3721	4248	.	3626	3767
2350	3815	1721	1919	2673	3483	2565	2607	2909	3798	2522	2884	3710	3105	3539	2194	1961	3007	2809	4198	2930	3843	4363	.	3704	3509
3036	3842	1704	2971	2813	2243	2707	2479	2716	3613	2341	2803	2960	2844	3146	1822	2095	3327	1633	4308	2845	3782	4032	.	3773	2481
4003	3683	1817	2153	3585	1967	3111	1665	3189	3683	2765	3242	2124	3130	1888	2891	3187	3338	1581	4205	3697	3839	4089	.	3514	2277
3788	2888	3356	1906	3292	2221	2992	1411	2942	3816	3221	3582	2152	1906	1584	2141	2527	2788	2031	3957	3349	3313	3463	.	3267	2891
3522	2860	3154	1871	3262	3112	2629	2439	2880	3179	2673	2442	2199	2415	2421	2693	1960	1708	1808	4092	2972	3520	3977	.	3356	3306
3128	2719	1748	1958	2190	2862	1900	2046	1721	2445	3803	1859	3001	2147	2132	2612	2491	1613	1866	3996	3218	3351	3927	.	2632	2649
2441	3473	1478	1935	1970	2203	2564	1711	1554	1620	3600	2136	2358	2089	2758	2115	3587	1503	2993	3877	1909	3389	3745	.	1918	2857
2459	3620	1731	1900	2808	1562	1840	2540	1975	2408	3613	2640	2591	1930	2058	2639	3644	2014	3575	3913	1587	3327	4069	.	1814	2700
2406	2566	1765	2119	2188	1835	2605	2776	3153	2215	3541	2145	2025	1827	1686	2133	3215	2964	3419	3514	3190	2967	4021	.	1901	3129
3185	3299	2298	1962	1965	1691	1796	2345	2053	1999	2469	2085	3151	2341	3529	2896	3479	2013	1888	2932	3165	2742	4029	.	2681	2678
2646	3368	1763	2101	2207	1626	2826	1871	1886	1981	2831	2416	3078	2004	3335	2318	3415	3010	1349	2137	2019	1934	4033	.	2061	3081
2666	3348	1677	3405	2214	2671	2012	2232	1742	2489	3569	2551	2346	2038	2059	2057	3176	2515	2791	2198	1670	1928	3856	.	1883	3252
2774	2895	2130	3372	3123	3665	2274	2590	2007	2071	3718	3139	2151	1841	1691	2738	1948	3077	2053	3148	2668	2138	3191	.	3078	2176
2897	3049	1889	2107	3274	2910	2129	3136	2661	2612	4166	3541	1973	3334	2222	2839	1837	2803	2332	2246	2054	2182	3159	.	2259	2549
2232	2525	1748	2587	2044	2363	2900	3230	2646	3473	3437	3115	3232	3999	1720	2870	3865	1583	1951	2233	3403	2968	3855	.	2105	2469
2803	3769	3728	3398	2058	1419	3078	1751	1623	3489	4061	2052	2824	2998	2407	2695	3186	1431	3312	3139	3903	2317	3159	.	1929	3364
3287	3419	3767	3461	3268	1559	1918	1563	1430	3227	3926	2642	3299	1759	2782	2433	2165	1557	2928	2472	3540	1992	3290	.	3242	3175
2266	2535	3884	2987	2379	2053	1197	1721	3415	2062	3240	2423	3127	1537	2004	1643	2009	1678	1618	2035	2810	1984	2918	.	3506	2201
1837	2519	3862	2013	2007	2800	1750	2484	2946	1417	3322	2185	2544	2121	1568	1989	2993	3291	1529	2128	1616	3071	2046	.	3078	2299
3187	2915	4042	1501	2869	2187	1663	3179	2186	1603	2414	2136	1744	2867	2284	2286	2129	3152	1643	2267	2084	3151	2299	.	1799	2629
2101	2409	4009	2094	2305	1515	1866	2709	1472	2846	2357	2040	2075	1848	1720	1811	2167	2401	1625	3684	1790	2862	2235	.	2363	2879
2292	2568	3882	3157	2086	1664	2455	1434	1674	2201	3123	3281	3135	1847	2704	1846	3495	1635	2584	3761	2844	1800	2483	.	1848	2529
2869	3097	2965	2983	2219	2914	1794	1400	1888	2021	2206	2203	3128	1892	2550	2314	3111	1779	2208	3255	2906	2832	3707	.	1878	2096
2218	2523	1896	1872	2105	2656	1851	1513	1935	1720	2424	2829	1970	3138	2178	2462	2356	1704	1885	2023	1817	1827	3102	.	1996	2290
2117	2522	1696	2399	3341	1926	1907	1812	3134	1817	2469	3616	2028	2384	1858	1847	1922	1674	1744	1855	1796	1849	2092	.	1864	3118
2231	3328	1692	1962	2351	1785	1972	1587	2332	3164	3460	2935	2066	1917	1826	1976	2171	2226	1802	2075	1743	2324	2184	.	3006	2509
2328	2516	2757	3025	1966	1539	1692	2652	1623	2731	3137	2071	3059	2059	2532	2849	2088	1555	1967	2152	1819	2445	2064	.	2109	2335
2253	2525	2065	2971	2174	2395	2456	3299	1741	1521	2237	1864	2796	2807	1949	2461	3066	1760	1842	3157	2074	1775	2009	.	1942	2109
2087	3265	1924	2048	3016	1662	1743	2816	2358	1625	2218	2092	1977	2375	2194	1813	2095	2911	3563	3384	2503	2007	2591	.	1905	2510
2477	2782	2053	1705	2324	1700	1480	1624	1881	1685	2257	2258	2189	1923	1806	2037	2065	1875	2578	2782	1998	2001	2097	.	1818	2429
2969	2775	2503	1801	2206	1609	2762	1642	2700	1820	2407	2283	2185	2395	1747	1884	2038	1495	1585	1991	1824	1971	2276	.	2027	2974
2178	2552	1695	1976	2310	2651	1593	1860	2272	1802	2454	3307	2322	2592	2325	1908	2834	1501	1637	2030	1872	1982	2257	.	1959	2296
2012	3000	1662	1805	2173	1588	1683	2976	1514	2582	2488	2495	3278	1911	1724	2585	2185	1663	1670	1941	1863	2996	2169	.	1799	2174
2027	2528	1827	2549	2253	1714	1694	2849	1871	1714	2938	2259	2407	2775	1773	2615	2698	1899	1991	2140	2107	1891	2106	.	1849	1951
2879	2311	2371	1900	2445	1569	2571	1549	2408	1763	3385	2149	2373	2661	2520	1923	2930	2795	1830	2169	1748	1938	3260	.	3107	2301
2271	2330	1940	1863	3011	2163	1970	1614	2852	1802	2492	2152	2180	2144	1641	1652	2146	1634	1789	2510	1938	1999	2245	.	2640	2195
2191	3458	1925	1984	3099	2486	1451	2076	1896	2347	2443	2693	2810	1922	1758	1698	2164	1532	2962	2102	1889	1975	2129	.	1811	2313
2153	2763	1985	3464	2156	1818	1737	2949	1793	1983	2504	2257	2137	1972	2760	2460	2104	1505	2931	1965	1812	1782	2209	.	1882	2597
2048	2329	1991	3143	2180	1565	1683	2144	1909	1974	2246	2175	2296	2724	2173	2624	3302	1634	1753	1954	1927	2136	1953	.	1818	3012
3255	2290	2053	1953	3193	1514	2334	1374	2322	2599	3301	2119	2088	1914	2038	2025	3047	1505	1775	1966	1829	2150	1841	.	1700	2210
2454	3402	1991	1781	2885	3181	1728	1576	3262	2810	2431	3361	2581	2291	1807	1837	1846	1676	1654	2401	1739	2246	2009	.	1707	2333
2051	3217	1963	1948	1984	2654	1496	1724	2338	1865	2411	3677	3486	2442	2755	2595	2088	1629	1642	2993	2444	3082	2228	.	2960	2268
2113	2364	2008	1763	2173	1493	1777	1764	1713	1983	3302	2280	3078	1991	2949	1991	2063	2542	2605	2218	2948	2350	2820	.	2427	3618
2192	2414	3390	2120	2182	1461	1479	2998	1577	1978	2656	1887	2174	1696	1731	1766	3060	3022	3112	2146	2288	1969	2250	.	1804	2867
3036	2790	3988	3211	3391	1640	3228	2230	1759	1989	2421	3630	2345	2959	1764	2725	2576	2699	2067	2099	2018	1949	2019	.	1933	2151
2226	3062	3736	2487	2771	2217	2203	1359	2291	2297	2317	3532	2427	2710	2763	1738	2017	1587	1749	1971	1865	2792	1918	.	1950	2203
2036	2521	3768	2009	2123	2907	1617	1506	2252	1837	3793	2314	2331	2071	1860	1826	2130	1638	2844	2550	2091	2147	2481	.	1811	2311
2461	2340	2275	2653	2832	1801	1768	2460	1821	1989	2555	2771	2730	2431	1899	2656	2986	2615	2749	2179	2124	1917	2023	.	3088	3193
2176	3355	1786	2194	3858	3434	2698	2110	2434	2656	2242	2500	2972	3093	2123	2718	2914	1793	2085	2326	2078	2141	2297	.	3122	2621
2829	4144	1952	3792	3733	3722	2059	3147	3267	3058	2365	2506	3550	2628	3380	2084	2155	2669	2653	3072	3243	2442	3350	.	2205	3166
2659	3968	2259	3776	3957	2881	2650	3322	3379	1835	2682	3330	2554	2852	3356	2186	3394	3280	3439	2217	3440	3335	2996	.	2818	3963
3642	4106	3314	3796	3470	3691	2151	2358	3154	2914	3849	3841	2337	2328	2699	2602	3632	3172	3533	2925	3561	2874	2350	.	3339	3780
3784	4147	3596	3763	3074	3496	2654	2115	3023	3141	3746	3602	2987	2680	2318	2173	3661	2230	2785	3586	3370	3153	3167	.	3331	3808
3677	3964	2510	2839	3209	3564	3156	3089	2959	3492	2660	3290	3830	2635	2733	2425	2589	2980	1804	3618	3643	3262	3453	.	3726	3228
3855	4170	2027	1920	3064	3291	3283	3411	2838	3497	3678	3886	3672	2923	2011	2773	3220	3843	1823	2530	3252	3228	2334	.	3920	3363
3608	3754	4041	1881	2343	2935	2998	3715	2879	3060	2891	3940	3084	3296	2370	2220	2381	3376	2698	2171	3569	2326	3560	.	3675	3520
3520	3339	4218	3122	2603	2859	3043	2989	3093	3170	2134	4202	3433	2954	2530	2164	2912	2629	2903	3917	3363	2457	3767	.	3728	2308
3601	3903	4154	3295	2382	3101	2600	3371	2928	3508	3500	3861	3289	3757	3224	3135	2632	2955	1879	3367	3087	2686	3935	.	3552	2956
3318	3963	2868	2732	3377	3657	2783	3632	2790	3500	3746	2684	2520	3087	4041	2280	2458	2959	2596	3111	2368	2134	3921	.	3339	3384
2454	2789	3475	1965	3356	2531	2310	2872	2799	3273	3693	2179	2358	3114	3326	2360	2686	2764	3548	2088	1871	3071	3546	.	2235	2545
2776	2407	3786	1896	2572	2455	2614	1604	2608	3306	3779	2152	3416	3498	2919	3065	3702	3075	3415	3161	1962	3349	3762	.	3128	2506
2576	2733	2990	3061	2592	3306	3221	1307	2642	2025	2652	3316	2522	3721	2131	2868	3129	2608	2213	3808	2950	3297	3480	.	2181	3347
2848	2551	2205	2622	3460	3726	3155	2702	2506	1774	2598	3397	2497	2220	2373	2597	3176	1899	1832	3645	3315	2329	3603	.	2083	2327
3466	3614	2013	2816	3608	2695	2990	2618	1591	3127	3625	2403	2587	3193	1951	2447	3262	1775	2244	3766	3177	3562	2513	.	3467	3533
3722	3863	2966	3304	3035	1796	2852	1627	1648	3670	3650	2819	2339	2406	2549	2447	2869	2477	2612	4042	2356	3323	2007	.	2846	3657
4032	3654	2886	3680	1965	3195	2876	1460	2598	2947	3463	2715	3551	2347	1949	2334	3480	2952	2009	3309	2946	3292	2340	.	3475	3391
3742	2390	2406	3445	3047	2535	2555	1786	2573	2908	2583	2545	3477	3396	3217	2263	3650	2167	2838	3629	2753	3360	3377	.	3261	2250
2453	2506	2982	3214	2884	1786	2023	1611	2173	3230	2027	3665	2686	3565	3241	3215	3803	2348	3367	2435	2748	2903	2372	.	3508	1888
2451	3193	3587	2046	2715	2583	1929	2831	1745	2594	3452	3701	2186	2882	2918	3024	3414	2898	3248	1941	2079	3262	2557	.	3504	2155
2779	2710	2546	2266	2235	2670	1924	3008	1597	3598	2602	3857	2202	2643	1690	2000	3395	2337	2448	3515	2053	2888	3507	.	2841	2352
2213	2513	2180	2870	2886	2619	3074	2113	1785	3572	2406	3190	2675	2982	1980	1660	3601	2391	1852	3620	2152	2094	3532	.	1863	3325
2140	3892	2973	2233	2317	1978	2190	1435	2563	2567	2354	2410	2509	2805	1759	1893	3415	3482	1722	3882	3036	1807	2462	.	1848	2334
2727	2899	3521	2725	2436	3071	2118	1270	2270	3100	2243	2511	2378	2235	2349	2883	1989	3178	2316	2759	3067	1962	3557	.	1931	2291
3037	2488	2535	2266	2618	2642	2626	2633	1806	2990	2424	3281	2293	2490	2778	2917	2212	2082	2032	2064	2222	1963	3678	.	2032	2741
2408	2485	2159	1972	2381	1968	2557	2574	2083	2082	2944	3065	2238	2008	1976	2765	2646	1704	1877	2173	2132	2044	3435	.	1904	3169
2688	3765	2130	1856	3067	1608	2564	1748	3057	1800	2656	2621	3215	2799	2760	1742	2294	1796	2719	1882	2040	1862	2429	.	2498	3356
3463	3485	2392	2280	3214	1929	1949	1661	2435	1669	2825	2406	2756	1938	1923	2167	2451	2345	3228	1936	2032	2465	1951	.	1957	2303
2394	2419	3493	2125	2266	3073	2706	1800	1683	2682	2798	2357	2248	2327	2401	2951	2103	1600	2901	2217	2500	3269	1937	.	3394	2413
2361	3092	3363	2671	2661	2947	2174	1527	1717	2544	4064	2344	2129	1907	1827	2305	2291	1649	1729	2208	2552	2189	2987	.	3224	3355
3124	2629	3489	2286	2904	1928	3017	1491	1587	1915	3740	3763	3725	1524	2540	2137	3406	2804	1580	3825	3473	2307	3335	.	2040	2596
2616	4084	2534	3838	3278	1397	2368	2508	2760	1851	3635	4451	3146	2726	1719	2126	3382	3187	2771	4266	2924	3024	2965	.	1809	2694
3415	3562	2178	2831	2218	1231	1637	3035	1908	1844	3405	2578	3244	2509	2795	2670	3135	2461	2293	3846	2896	2191	1946	.	1545	1966
2739	2751	2161	2027	2080	3647	1748	3532	1657	2883	2561	1970	2886	2237	2095	1750	1963	1591	1726	3436	2174	1670	1948	.	1669	2147
1822	2404	3908	3099	2930	2960	2220	2880	1730	2665	2004	3159	2037	2094	2602	1514	1861	1423	1590	2959	1886	1430	2906	.	3014	2354
1949	2381	4107	2850	2278	1623	1691	2234	3134	1730	2045	3062	1854	2669	1948	1772	2089	1401	2563	1897	2485	1639	2187	.	3289	2938
3118	3038	3428	2075	2332	1435	2394	1594	2168	1694	3315	2145	1833	1837	1725	2072	3444	1841	1797	1617	3050	1688	1976	.	2303	2014
2959	2322	2409	1856	2507	1477	1833	1561	1607	2974	2572	1953	1909	1904	2357	1837	2254	2016	1777	2500	2403	2898	2104	.	1772	2044
2047	2471	1910	2474	2101	1415	1910	1534	1741	3122	2238	2176	2801	3166	1761	1848	1874	2999	1798	1950	2184	2749	2733	.	1711	2065
1814	2738	2693	3053	2660	1461	2540	2137	2457	2385	3225	2321	2928	2782	1745	2796	3332	2608	3251	3146	1950	1696	2045	.	1706	3382
2026	2280	2328	2402	2152	3366	2733	3129	2644	1644	2283	2406	1991	1904	2528	2648	3124	1635	2799	2504	2118	1776	2769	.	1843	2850
2662	2426	1993	1839	2332	2915	1683	2304	1757	1621	2226	2176	1988	2057	1758	2219	1960	1646	1738	1968	2178	1738	2288	.	2170	2024
2261	2279	1994	1869	2991	1729	1569	1755	1594	2011	2155	2336	1972	1997	1803	2038	1882	2022	1843	2020	1928	2120	2091	.	2662	2106
2195	2336	3007	1836	2361	1548	2015	2523	1889	1793	2456	2222	2587	2415	2581	1728	2087	1589	1816	2012	2343	1675	2073	.	1921	1858
1925	2353	2994	2903	2261	1976	1539	1752	1758	1877	2187	2214	2169	2809	1607	2207	3187	1768	2810	2066	2865	1966	2089	.	1732	3393
3300	3220	2006	1918	2044	1586	1748	2708	3231	2539	2847	3372	2397	2412	2393	2013	2303	2861	2103	2159	2013	1906	3086	.	1756	2190
2741	3015	1975	2461	2459			2303	2625	1986	2329	3055	1835			2523	1813	2673	1655	2131	1974	1731	2922	.	1727	1916
2038	2314	2144	3363	2195			1654	1652	1915	2071	2204	2143			2097	2061	1948	1786	3791	2077	2866	1926	.	1755	2113
1894	2360	3474	2520	2342			1784	1700	1748	2451	2064	2025			1712	1941	1574	2792	3540	2591	2503	2137	.	3040	1969
1868	3031	3026	1938				1774	1890	2277	2936	3265	3112			1738	2882	1635	1961	2831	2103	1692	2108	.	2725	2976
2475	2266	2042	1938				2677	2546	2757	3019	2629	3098			2087	2070	1656	1814	2986	2077	1713	2095	.	1620	1931
2033	2326	2001	2154				2154	2702	1892	2221	2289	1994			2030	1931	1819	1495	2204	2074	1684	3219	.	1718	1910
1884	2233	3253	2530				2578	1737	1843	2277	2309	2480			2684	1852	1630	1631	2167	2733	1696	2891	.	1809	1988
1871	2134	2431	2072				1845	1778	1756	2271	2212	2046			1942	3155	2648	3023	2153	2574	1777	2025	.	1798	1990

1879	3038	2046	2089				2308	1798	2022	2243	2663	2195			1959	2202	2572	2261	2156	2077	2113	2222 .	1811	2501
2214	2256	2460	2564				3195	2305	2936	2871	2476	2082			2088	2090	1530	1692	2142	2127	1787	2092 .	3044	2934
3377	2807	2144	3373				2414	1678	2342	3261	2316	3032			3111	1818	1590	1631	1889	1808	1798	2991 .	2980	2150
2488	2303	3238	2572				1706	2144	1573	2249	3181	3362			1772	2150	1719	1829	2118	2043	1708	2225 .	2155	2118
1873	2424	2953	2094				1721	1715	1852	2459	2956	2347			1784	3121	1657	2991	2504	2151	2437	2054 .	1790	3016
2581	2697	2450	2590				1956	1923	1909	2427	2138	2187			2670	2985	2758	2804	3253	2125	2613	2366 .	1993	2356
2230	2774	2419	1983				1698	1824	3150	2341	2151	2239			2049	2049	2272	1575	2156	1987	1846	3303 .	1882	2140
3299	2342	2273	2715				1690	2652	1997	2854	3424	2653			1939	1781	1751	1785	2033	2396	1822	3121 .	1924	2147
3276	2383	2800	3119				3049	1923	1904	2786	3017	3110			1876	1990	1646	2185	2049	3102	1828	2230 .	2075	1913
2204	3336	3719	2347				3441	1751	2303	2457	2439	3215			2851	2702	2227	2712	2211	2046	1974	2129 .	3049	2124
2085	3557	2673	3191				2362	2266	2130	2749	2494	2537			2699	3183	2581	2583	3383	2349	2220	2295 .	2284	3394
2916	3574	2284	3651				1745	3572	2881	2746	2875	2436			2773	2396	2812	1965	3406	3221	2623	2630 .	3115	3278
3017	2713	3469	3205				2833	3670	2861	2453	2779	3762			2007	3546	2880	3160	2279	2679	3482	2442 .	3584	3551
3208	3736	3925	3629				3312	3522	2650	3621	3699	2798			2389	2801	3229	3355	3243	3417	3238	3194 .	2554	2831
3852	4072	4066	3654				3310	3041	3257	3589	3231	3120			2825	3632	2493	2248	3377	3549	2890	2734 .	3178	3245
3819	3575	3969	3408				2171	3048	3414	3804	3405	3507			2071	2773	3159	2023	2999	3394	2935	3300 .	3434	2925
3639	3363	3103	3009				3556	2489	2444	3777	3994	2636			2527	2863	3056	2864	3071	2836	3249	3600 .	3242	3230
3746	3883	3164	2292				3329	2052	3298	3944	3506	3763			2291	3248	2402	2441	2837	2026	2621	3704 .	2461	2457
3025	3152	3210	2557				3243	1796	2709	3424	2491	3419			2298	3643	2552	2677	2535	2885	2230	3136 .	3297	3191
2780	3140	2760	2327				3241	2646	3256	3370	3728	3350			2975	2612	2881	3400	3548	3741	2367	2539 .	3364	2844
2804	2731	3992	2410				2695	2189	2862	3399	3765	2345			2980	3366	2603	2845	2824	2457	3382	3311 .	2947	2821
3412	2790	3399	3126				1778	1977	2637	2383	2470	2402			2161	3005	3434	2038	3395	3039	3444	3304 .	3061	3810
3139	2623	3970	2984				1990	2226	3291	2352	2409	2690			3028	2838	3297	2847	3769	2530	3385	3550 .	3500	3490
4076	3679	3421	3096				2714	3321	3239	2406	3335	3366			3147	3182	2542	2451	2994	2419	3239	2930 .	3412	2847
3622	3998	2479	3281				1781	3410	2816	2548	3022	2503			2793	3518	3403	2462	3766	2503	3076	3539 .	3623	2463
2640	3571	2056	2636				2311	2810	2426	2393	2829	2131			2061	3398	2457	3570	3032	3305	2703	3795 .	3390	3493
3198	3024	3184	2530				3415	2744	3255	3858	3047	2669			2956	3431	2516	2280	3549	3240	2298	3176 .	2237	2663
3625	2821	2725	2603				3369	2619	3032	2601	4138	2778			2397	2432	3125	2112	3529	3365	2160	3650 .	2085	2499
3556	2486	2768	2230				2639	1512	3157	2864	3744	2300			3062	2573	2396	1879	3456	3054	2447	3868 .	2794	2394
2550	3622	2591	2197				1729	1669	2631	3419	3186	2191			2924	3099	2276	2351	2850	2728	3356	3435 .	2453	2527
2467	2620	3594	2738				1791	2578	2966	2555	2223	3312			2009	3436	2711	2985	3752	2760	3027	3311 .	2746	2324
2823	2779	3148	3074				2532	3119	3248	2598	3112	2779			2206	3424	3333	3214	3611	2730	2330	3039 .	2439	3360
3053	2729	2408	2246				1802	-19	2931	2849	3161	2379			2764	-8	-15	-19	3875	1880	2680	2527 .	2064	3492
2411	3345	2070	2020				1743	36	2114	2413	2408	2055			2234	8	5	37	2746	2200	2099	3265 .	2079	3195
2840	3205	2185	1850				1705	2440	2737	2405	2436	2765			2129	3392	2782	3474	2621	2273	1921	2344 .	3222	2074
2868	3378	2158	2846				1604	1808	2972	3021	2599	2334			2270	2344	1931	2691	3957	2814	1937	2718 .	2678	3010
2426	2897	1979	2366				1634	1850	2286	2999	3107	3130			2772	2410	2128	1845	3634	2071	1907	2947 .	2753	2541
3026	2920	2620	2270				2878	1775	2159	3491	2660	3472			2738	3100	2776	1589	2401	1923	3556	2469 .	3029	2530
2739	2683	3013	2174				3259	1869	2850	3399	2553	3109			2054	3049	2192	1721	2036	1777	3465	3576 .	2817	2675
3080	3611	2627	2190				1957	1732	2902	2601	3741	2004			2256	2413	2535	1796	2327	2453	3093	2796 .	1908	2427
2617	2739	2539	2669				2306	1932	1970	4001	2915	2273			1907	3075	2486	2122	2056	3407	2629	2495 .	1586	2374
3219	3438	2006	2068				2630	2153	1648	3751	2027	2847			2783	3102	2769	1871	1992	2454	1770	2987 .	2433	3289
3618	3107	3018	2574				2160	2305	1517	3413	1974	2136			3085	3424	2191	3191	3064	1911	1508	3463 .	3419	3916
3093	3091	3720	3557				1492	1696	2599	3006	2221	2011			1767	3054	2023	2039	3362	1806	1799	2707 .	2962	2328
1981	2503	3828	2716				2183	3309	2342	2142	2117	2039			1621	2063	1661	2708	3277	2623	3052	2816 .	1903	2020
1581	2701	3002	3095				2738	3110	1701	2100	2439	2197			1727	2745	3086	2828	2165	1908	2381	2473 .	1722	2579
1736	2632	2102	2936				2018	2476	2228	2022	1990	2973			2443	2058	2532	2028	1639	1865	1635	2400 .	1904	2265
2877	2478	2333	2092				1297	2030	3400	2892	3583	3306			1950	2085	1413	1563	1941	3100	1709	3637 .	2330	2039
2094	2549	2291	1985				1511	1522	2828	2396	3284	3094			1773	2962	1447	1589	2000	2901	1727	2212 .	1848	2265
2221	2520	1932	1849				2703	1753	1684	2235	2218	2349			2038	3132	1462	1859	1944	1801	1784	1981 .	1779	2742
2344	2530	2208	2668				2904	3183	1568	3245	1981	2297			1990	2339	2299	1841	2950	1734	2365	2501 .	2675	2120
2234	2450	3462	2481				2384	2125	1573	3226	2100	2163			1977	1889	1643	1737	1940	1852	1922	2211 .	2035	2107
2164	2509	2632	1896				1587	1665	1738	2236	1992	2424			2436	2107	1523	1834	1955	2608	1844	2209 .	2003	2882
2195	2344	1831	2541				1440	1533	2919	2077	2063	2176			2198	1906	1620	2993	2184	1984	1869	3003 .	1947	2307
2432	3015	1676	2942				2522	1811	1944	3075	3065	2280			2107	2144	2653	2224	2870	2054	1943	2928 .	1983	2300
3001	2378	2005	1977				1633	2687	1560	2228	3775	2997			2114	2961	2829	1802	3890	2266	3041	1979 .	1952	2218
2002	2473	2047	2095				2176	2012	1820	2188	2714	3135			2488	2123	1822	1767	3101	2418	1951	1951 .	2374	2092
2039	2832	2435	3286				1767	1736	1887	2489	2272	2304			2609	1934	1679	1828	1903	1709	1762	2014 .	2604	3092

3516	3928	2884	3628	2720	2571	1557	1835	3342	2314	3685	2530	3486	3198	3061	2056	2924	3109	3686	3431	3156	3172	2663 .	3469	3139
3244	4024	2811	2559	2906	2268	3172	2984	3112	3631	3362	3523	2344	3225	2829	2898	3549	2517	3261	2393	2753	2378	3470 .	3404	2982
2884	3813	3642	2518	2573	3380	3533	3163	2185	2454	3337	2727	3164	2380	1716	2709	3356	2888	3753	4195	3019	2161	2818 .	3170	2299
2991	3576	3017	3130	3367	2403	2236	2154	2932	2473	2644	2458	2506	2940	2924	2332	2406	2235	2840	3913	3039	2952	3355 .	3469	2210
2524	2905	2375	2332	2483	1888	2165	1744	3281	2392	2797	3520	2361	2294	2622	2642	3684	2935	2600	4081	2430	2302	2990 .	2608	2341
2584	3179	2252	2354	2239	2558	2959	2767	2316	3200	3662	2846	3721	2806	1492	2114	2708	2937	2962	3759	2280	3144	2560 .	2169	2983
2502	2860	3371	3416	3216	2807	2868	2032	3095	3143	3238	2533	2747	2178	1589	2569	2449	2069	2862	2565	2610	2354	2876 .	2636	2298
2853	3338	2853	3590	2425	1864	1644	1717	2472	2606	2678	2580	3562	3031	2770	2062	3306	2795	3227	3991	2975	2322	3477 .	2542	3384
2669	3150	3206	2751	2956	1812	2906	1823	2323	2609	3229	3426	2924	2399	1699	2118	2365	2588	2413	2882	2156	3170	2542 .	3204	3351
3140	2798	2452	2682	3095	1755	2504	2815	2791	2983	2685	2596	2751	2333	2672	2073	3156	1800	3093	2772	2271	2546	3435 .	2498	2319
2743	2891	2444	2904	2632	2446	1870	2386	2321	2466	3055	2798	2951	2372	2052	2194	2351	3338	3446	3680	3213	2400	2987 .	3331	2598
2588	3391	2433	3185	2500	2477	3274	2135	3266	3053	3022	3198	2759	2836	1953	2642	3235	2375	3219	2875	2454	3082	2870 .	2630	2935
2934	3024	3159	2955	3101	3254	2504	3297	3314	2878	2738	2810	3711	2935	2657	2555	2686	2080	2435	3021	2511	2427	2630 .	2729	3015
3183	3628	2816	3356	3204	3853	2959	2923	2440	2843	3553	3581	3285	2563	2813	2478	2285	2513	2858	3206	3529	3357	3405 .	3730	3761
3127	3186	3519	3840	3209	2693	2449	3681	2517	3090	3708	3441	3545	2756	2096	2952	2543	2656	2412	3201	3720	3324	3689 .	3177	3918
3581	3752	3816	3814	2972	3319	2489	3630	3099	3029	3722	3420	3175	3229	3074	2597	3275	3557	3152	3432	3138	3468	3239 .	3323	3443
3161	3224	3779	3149	3187	3487	3232	2574	2902	3402	3180	2749	2963	3345	2935	2523	3251	2708	2906	3633	3084	2996	3741 .	3419	3253
3397	3755	3562	3448	2768	3113	3609	2888	3370	3869	3846	3562	3559	2703	2392	2572	3623	2522	3866	3802	3133	3283	3447 .	3531	2725
3557	3782	3446	3762	3066	3158	2489	2723	3514	3466	3675	4715	3759	2942	2622	2825	3385	2545	3167	3665	3007	3571	3760 .	3810	2924
3077	3040	3446	3324	3169	2538	2684	3016	2792	3665	3694	3301	3334	2758	3205	3082	3535	3364	3375	4006	3296	3041	3077 .	3105	3270
3144	3478	3331	2844	3586	3142	2409	2308	2805	3912	3041	2728	3063	3008	3200	2350	3201	4213	3187	2945	4070	3327	3470 .	3936	3166
3399	3860	3767	3498	3178	3012	3169	2760	3142	3198	3189	3340	2681	2960	2653	2207	3141	3066	3788	3350	3723	3355	3250 .	3918	3760
3520	3266	4028	3116	3355	2562	2473	2725	2864	3014	3216	3195	2894	2867	2187	2613	3028	2595	3782	2783	3017	2960	3248 .	3617	3673
4137	3231	4135	3029	3111	2377	2398	2098	2569	3879	3011	3847	3379	2983	2282	2626	3365	2887	2775	3351	2809	2933	3860 .	3525	2822
3217	3338	3754	3334	3004	3220	3042	2461	2847	3603	2996	3746	3387	3283	1867	2762	3658	3509	3294	3592	2984	3126	3886 .	3081	2783
3039	3003	3267	3899	3405	3735	2486	3335	3301	3142	3788	3271	2985	3042	2246	2683	3343	3705	3217	3225	3463	3655	3468 .	3611	3154
3082	3164	3185	3567	3390	3298	2535	3079	3471	3899	3473	3773	2957	3008	2630	3559	3363	3297	3368	3313	2959	3335	3678 .	3473	3053
3492	3781	3744	3809	2951	2456	3165	2815	2577	3454	2990	3052	3374	2700	3275	3223	2728	2955	3553	3810	3317	3351	3259 .	3009	3569
3721	4128	4190	3529	3322	2909	2537	2421	3089	3529	3183	3096	3843	3183	2502	3186	3413	2664	3817	4175	2946	3341	4070 .	3137	3448
3132	2959	3371	3149	3154	2499	2374	2582	2608	3257	3676	3014	2691	3428	2648	3122	3979	2689	2920	4038	2829	3448	3839 .	3634	2771
3100	3288	3522	3025	3117	3134	2717	3203	2650	3809	3818	2960	3093	2988	1951	3090	3123	2591	3145	4020	2673	2850	3657 .	3466	2770
3223	3412	3672	2972	3173	2429	3274	2446	3335	3621	3208	3371	3588	3180	1790	3002	2883	2835	3586	3179	3643	2848	3232 .	2717	2581
2983	2854	3969	2854	3428	2573	2597	2480	3182	3214	2843	3534	3152	2922	2548	3236	3654	3432	3050	3775	2971	2905	3814 .	3270	3149
3107	2781	3356	2873	3446	2439	2818	2574	2398	3224	3622	3076	2909	3183	1792	2943	3076	2629	2932	3383	2454	3139	3858 .	3039	2607
3103	2851	3204	3101	2504	2935	2634	2584	2797	3188	2818	2893	2865	3100	2515	2588	3210	3016	2880	3159	2790	2748	3244 .	2885	3223
2906	3193	3319	3087	2749	2350	2387	2427	2473	3539	3084	2957	3275	2708	2173	2498	2865	3721	3142	3108	3627	3299	3299 .	2829	3051
3801	3117	3329	3295	2958	3084	2329	2868	2609	3243	3016	2878	2799	2737	2186	2427	2776	3750	3195	3937	2522	2744	3581 .	3101	2444
2917	3220	3223	2905	2935	2638	3196	2669	2800	3849	2855	3382	2653	2766	1910	2830	3583	2642	2921	3597	2814	2737	3050 .	2895	2859
2802	3067	3646	2840	2945	2371	2505	2263	2532	3695	3052	3133	3027	2738	3081	2806	2959	2652	2755	3152	2826	3026 .	.	3395	2618
2915	3472	3338	2916	2953	2411	2594	2473	2639	3593	3009	3181	2839	3015	3026	2366	2774	3013	2902	2790	2940	2915 .	.	3460	2615
3449	3354	3136	2960	3030	2288	2425	2495	2391	3215	3071	3557	2619	2921	1929	2720	2760	2487	2740	3105	3479	2481 .	.	2738	2844
3143	3086	3030	3221	3544	2425	2486	2341	2788	3857	3092	2737	3212	2632	2777	3258	2777	2719	3030	2960	2821	2522 .	.	3175	3293
2806	2898	3379	3029	2487	2877	2421	2557	2860	3734	2860	2698	3352	2379	2419	2934	3208	3489	3108	2926	2542	2337 .	.	3110	2639
3340	3911	3138	2812	2325	3249	2297	2554	2385	2962	3248	3076	2627	3043	2013	2176	3628	3222	2774	3664	2565	2210 .	.	3023	2490
2718	2791	4048	2637	2498	2163	3038	2364	2197	2715	3441	3993	2255	3015	1782	1967	3374	2274	2574	3996	3380	2771 .	.	3478	2423
2460	2796	4109	3025	3522	1788	2212	2670	2496	2557	3414	2863	2208	2487	1760	2770	3250	2309	3020	3560	3155	3318 .	.	2687	2282
2313	2479	2873	3719	2789	2206	2082	2132	3488	3302	2679	2961	2654	2446	2483	2165	2284	2506	3186	2717	2357	2271 .	.	2080	2124
2564	3043	3336	2699	1963	2243	2219	1967	2916	2386	2844	3634	2236	2120	1577	2203	2562	2744	2942	2618	2488	2287 .	.	3207	2475
2234	2568	3139	2434	3020	1600	1650	2172	2049	3109	3155	2961	2302	3134	1579	2069	3043	2023	2219	3653	2377	2307 .	.	2644	2356
2360	3174	2861	2259	2129	1715	1454	2152	2307	3063	2684	2343	2733	2346	1583	2261	2606	2335	3066	2675	2391	2283 .	.	2520	3362
2351	2652	3205	3085	1982	2309	1962	3006	2299	3064	2455	3104	2337	3338	2052	2911	2353	2350	2391	2616	2247	3137 .	.	2894	2751
2647	2473	2688	2626	2705	1877	2300	2024	2480	2458	2515	2492	2427	2168	1632	2414	2573	1873	2226	2806	2109	2535 .	.	2630	2146
2781	2619	2422	2278	2314	2630	1748	1812	2890	2429	2696	2908	2818	2579	1777	2004	2238	1982	3025	2635	2703	2158 .	.	2494	2577
2372	2719	3123	2499	3373	1830	1956	2874	2104	2607	2795	2299	3533	2397	1669	2292	2639	3204	2424	3333	2230	2035 .	.	2703	2112
2805	3049	2557	2484	3035	1843	1996	2663	2076	2640	3287	2231	2741	2846	2524	2052	2679	2290	2412	3249	2272	2952 .	.	2550	2290
2413	2542	2679	2822	2518	1768	1982	2306	2316	2205	2804	3232	2484	2386	1919	2461	2241	1885	2554	2369	2365	2393 .	.	2543	2107
2578	2861	2613	2893	2261	1549	1766	2079	2363	2421	2681	2558	2490	2405	1721	2181	3258	2468	3031	3121	2769	2184 .	.	2948	2792
2762	2457	2356	2676	2438	3518	1967	2231	2381	3002	2415	2867	2289	2352	1688	1999	2389	2554	2526	2939	2082	2170 .	.	2526	2162
2606	2636	2152	2471	2484	2828	2802	2171	2314	2494	2880	2525	3075	2443	1909	2327	2345	1981	2638	2988	2275	2438 .	.	2524	2349
2603	3205	3159	2356	2668	1690	1612	2325	2915	2357	2687	2132	2439	2451	2576	2016	2372	2122	2943	2808	2136	2189 .	.	2342	2821
2540	2672	2763	2393	2556	1829	1937	1851	2522	2820	2908	3181	2270	2077	1750	1722	2341	2779	2420	2524	2383	2621 .	.	2621	2185
2423	2529	2350	3375	2525	1936	1937	1820	2303	2017	2918	3460	2497	2269	2767	1852	2807	1895	2397	2553	2137	2111 .	.	2510	2182
2369	3095	2095	2757	2076	1956	2119	2067	2105	2225	2332	2726	2626	2890	1915	1914	2649	1879	2443	2758	1941	2562 .	.	2896	2660
3003	2409	2624	2688	2463	2080	1649	2824	2484	2291	2648	2330	3120	2470	1897	1800	3015	1925	3140	2748	1888	2200 .	.	2708	2029
2505	2580	2524	3058	3148	2427	2003	2412	2297	2180	2665	2266	2599	2557	1765	2393	2290	2365	2417	2490	2222	2276 .	.	2468	2087
2302	2292	2540	2372	2517	1488	2906	1785	2152	2229	3153	2552	2481	2934	2090	1909	2270	1898	2391	3472	1995	2370 .	.	2647	3349
2402	2357	2367	2323	2287	1380	2650	2767	2272	3013	2783	2309	2663	2407	1775	2368	2178	2975	3107	2471	2167	2145 .	.	2455	3187
2121	3316	2163	2677	2379	1596	1819	2178	2366	2350	2621	2440	3088	2267	2096	2132	2848	3183	2482	3501	3333	2111 .	.	2453	2207
2925	2816	2553	2433	2902	2274	2058	1999	2237	3690	2434	3013	2557	2212	2293	1772	2375	2068	2515	3824	2490	2799 .	.	2202	2222
2504	2932	3573	2901	2199	2554	1633	1815	2181	3165	2535	2863	2535	2310	1713	3210	2481	1938	2437	3784	2069	2555 .	.	2866	2198

2524	2727	2699	2742	2174	1703	1821	1799	2777	2215	3005	2385	2749	2294	1865	2641	2475	2174	2650	2453	3248	2170 .	.	2939	3238
2874	2748	3192	2530	3170	2427	3211	2442	2974	2858	2656	3698	3142	3190	2966	2060	2409	3133	2355	3198	3129	2128 .		3089	3217
2651	2721	3498	2973	3153	2542	2478	2758	3094	2412	2602	2903	2517	2710	2629	2108	2276	3172	2984	2815	2121	2754	3247 .	2624	2353
2293	3396	2564	3539	2437	2613	2154	2396	2355	2172	3101	2524	2520	3511	3046	2030	3112	2256	3041	2482	2246	2736	3006 .	2438	2223
3051	3203	2477	2525	3493	1888	1767	2405	2272	3462	2623	2507	3762	2619	2095	2658	3478	2280	2645	2463	2240	2445	2443 .	3146	2132
2523	2690	2575	3140	2649	2099	3082	1810	2095	2206	3209	2498	3644	2518	2085	2429	2395	2125	2850	3768	2234	2744	2695 .	2660	2514
2389	2520	2798	2901	2474	2812	3455	1826	2686	3707	3230	2678	2510	2768	2809	2074	2537	2085	2533	2486	3330	3369	2455 .	2429	2235
2502	3709	2527	2870	2539	3369	2259	2912	2479	3187	2836	4089	3635	3760	2875	2303	2456	3048	2978	2663	3492	2515	3574 .	3031	2729
3652	3835	3401	3945	3714	3031	2419	3528	2325	2462	3184	3964	3942	3439	4243	3019	2650	2355	2576	2650	3340	2900	3647 .	4048	4010
4166	3395	3764	4012	3771	3308	2759	3000	2651	2810	4205	3145	3580	2622	3047	2517	2994	2851	3377	3877	3585	3879	3653 .	4001	3938
3529	4050	4320	3937	2743	2777	2056	3013	3421	3860	3775	3077	3752	3287	3105	3239	3416	2896	2676	3713	2723	2937	3833 .	3455	3569
3661	3450	3992	3687	3599	2857	2941	3593	3620	3560	3201	3140	3830	2498	2491	3395	3864	3707	3587	3516	3670	3028	3456 .	3479	3159
3773	3659	4247	3385	3328	2379	2521	2936	3655	2942	3816	3246	3484	3015	3420	3180	3387	2810	2767	3769	3849	3275	3437 .	3454	3362
3686	2757	4176	3458	3162	3086	2764	2733	3492	3439	3169	3127	2862	3252	3015	3140	2777	3051	3021	3326	2943	3169	4027 .	2815	2305
3267	3298	3692	2772	3487	2609	2332	2422	3485	3225	2957	3361	3663	3213	2591	2322	3751	3465	4010	3730	3479	3352	3823 .	3566	3150
2988	2939	3844	3541	2766	2472	3054	2662	3738	3831	3755	4356	3219	3367	3034	2734	3682	3742	3227	3340	2898	3657	3207 .	3402	3606
2876	3617	3503	3164	2530	2832	3074	2575	2993	4086	3403	3117	3039	2752	2864	2747	3801	3393	2717	2723	3215	2897	3953 .	2502	2792
3061	4053	3867	3070	2997	3180	2003	2734	2138	3245	2717	2922	3133	3452	2796	2736	3508	2443	3020	3174	2687	3188	3733 .	3351	2352
3428	3452	3583	3389	3210	3642	2834	2365	3276	3052	2804	3492	3267	3348	2451	2414	3251	3261	3597	4281	3130	2810	4043 .	3518	2134
2807	3168	3505	3923	3017	3332	3127	2665	2120	3841	2487	3058	2943	2688	2460	2423	3893	3807	3274	3267	3152	2883	3882 .	3640	2943
2846	3254	4217	3391	3993	3193	2883	2850	3020	4065	2809	3573	3366	2505	3328	2706	3869	2619	2685	3538	2853	2888	3353 .	3445	3855
3552	3278	3754	2779	3714	2993	2231	2164	2493	3988	2896	3114	3678	3607	2821	2831	2890	3007	3178	4408	2710	3513	3429 .	2634	3397
4126	3384	3429	3028	3262	3106	2634	3089	2153	3731	3605	3345	2889	3011	2478	2365	3063	2613	3313	4199	2719	3807	3292 .	3427	2306
3474	3238	3651	2906	2373	2624	2443	3145	3282	2997	3728	2950	2899	2489	2005	2763	3757	3233	3640	4096	3815	3468	3205 .	3286	2512
2794	3647	4066	3092	3370	2442	2304	2392	3413	3586	2998	2846	2927	3184	2228	2972	3892	3236	2817	3231	3649	3132	3138 .	2918	2522
3195	3286	3932	3669	2797	2433	2386	2041	3020	3135	2923	3071	3032	2538	2029	3122	3728	3106	2992	3583	2767	3720	3694 .	3461	3711
2777	3287	3632	3161	2861	3267	2391	2743	1982	3217	3032	2743	3003	2437	3281	2840	2495	2978	3415	3178	2740	3208	4028 .	2810	3583
3233	2919	3478	2982	3604	2410	3074	3301	2103	3000	2563	3778	2998	2510	3008	2337	2865	2977	2904	2949	3382	2552	3507 .	2583	2381
2706	3178	3588	2895	2728	2440	3041	2266	2171	3550	3290	3149	2882	3410	2621	2424	3611	2523	3454	3951	3627	3518	3408 .	3551	2341
2879	2886	3748	3158	3597	2627	2396	2673	2902	3193	3862	3417	2888	2987	2656	2331	2664	2843	3595	4132	2612	2608	3173 .	2618	2775
2770	3127	3698	2743	2960	2597	2565	2602	2933	3593	3118	2908	3258	2506	1935	2985	2629	2537	2879	3153	2432	2852	3408 .	2680	2870
2798	3432	3539	2821	2990	2479	2332	2315	2220	2808	2661	3104	3512	3006	1964	2828	2804	3470	2408	2876	2429	2697	3588 .	3638	2406
3841	3076	3437	3391	2731	2577	2336	2260	2296	3826	2746	3023	3145	2687	2808	2460	2756	3513	2648	2761	2852	2570	3318 .	3454	2360
3743	2777	3507	3058	3117	2147	2326	2262	2506	3044	2718	2995	2898	3202	1912	2534	3631	2612	3161	3438	2421	2790	3058 .	2763	3174
3082	3027	3227	2830	3540	2609	3075	2693	2782	3499	2253	3611	2908	2379	2048	2778	3309	2859	2955	3845	2650	2916	3312 .	2794	2647
3103	3517	3258	2416	2751	2373	2974	2746	2398	3340	2757	3535	2582	2334	2895	2172	2387	2430	3065	3245	2869	3238	3960 .	3483	2954
3158	2760	2713	3006	3023	2639	2293	2677	2252	3093	3610	3004	2904	2112	2636	2116	3126	3596	3082	4210	2657	2529	3754 .	2645	2464
3278	2975	2875	2557	2770	3168	1701	2798	2877	2737	2568	2614	3094	3537	1703	2298	3276	2534	2679	4091	2950	2048	3098 .	3309	2354
2476	3472	2832	2731	2572	2189	2933	1758	2587	2896	2814	2333	2503	3157	1667	1915	2679	2264	3028	3500	2319	2262	3042 .	3646	2376
2652	3414	3983	3764	3265	2225	2784	2535	3117	3887	2612	3446	2309	3197	2821	2498	2690	3701	2686	2751	2432	3105	2920 .	3108	3232
3332	3506	3206	3681	3662	1601	2004	2056	2364	3731	3819	3469	2342	2247	2063	2646	2686	2399	2704	2973	3216	2821	2467 .	2386	2465
2824	2750	3027	2712	2333	1680	1829	1918	2747	2753	3354	2590	2505	1993	1689	2006	3604	2147	2420	2539	3408	2812	2838 .	2272	2419
2095	3421	3519	2304	2297	2633	1646	3205	3359	2202	3177	3021	2372	1744	1741	2098	3577	3052	3792	2683	2315	2663	3709 .	2305	2081
2580	2877	2419	2725	2522	1813	1937	1760	2147	2258	2083	2795	2226	2859	2497	2107	2461	2232	2693	2452	2099	2461	3087 .	2659	2127
2995	2716	3211	2424	2535	1588	1758	1580	2073	2258	2662	2954	2230	2296	1729	2159	2150	2190	2251	2318	2266	2739	3077 .	2829	3088
2839	2476	2255	2175	2247	1430	2789	3022	2435	2863	2525	2052	2427	2531	1563	2114	2487	2830	2159	4066	3098	2353	2527 .	2332	2341
2511	2649	2480	2910	2174	2491	1713	2221	2015	2818	3121	2461	2356	2296	1593	2257	2547	2408	2419	3709	2201	2115	2947 .	2429	2412
2438	3015	3750	2645	2599	1859	1873	1808	2114	2327	2607	2239	2395	2141	2898	2170	2747	2395	2251	2812	1989	2893	2560 .	3262	2982
3569	3654	3119	3004	2206	3156	1844	1789	2358	2745	3890	3287	3123	2182	2753	2462	2323	2005	2099	2614	2216	2295	2345 .	3796	3412
2593	2746	2346	2819	2390	3258	2573	2776	2098	2588	2561	3110	3031	2786	2466	2223	2344	2231	2243	2856	2511	2067	3120 .	2360	2118
2278	2844	2006	2218	2444	2320	1718	1950	2213	2408	2761	2416	2290	2298	1672	2040	2252	2437	2551	3166	2042	2157	3764 .	2215	3083
2236	2953	2246	3016	3029	1747	1862	1616	2755	2451	2759	2632	2282	3068	1773	1920	2349	2832	3029	2658	2159	2300	2614 .	2606	2446
2802	3098	2618	2562	2123	1832	2039	2586	2060	3047	2751	2618	2124	2445	1703	2157	2317	2259	2035	2915	2294	1891	2540 .	2524	2547
2416	2495	1988	2377	2219	1922	1529	3251	2643	2140	2694	2347	2361	2239	2146	2014	2354	2029	2803	2804	2841	2950	2083 .	2332	2317
2709	2950	2404	3055	2683	2797	2475	2325	2069	2017	2539	2549	2114	3471	2430	2194	2300	1899	2431	2529	2127	2590	2374 .	2305	3107
2430	2709	2148	2594	2146			2010	2098	3412	3038	2324	2583			2615	3208	2264	2234	3521	2105	2034	2957 .	2285	1934
2436	2556	2037	2600	3157			1730	3228	2617	2366	2354	2122			2150	2279	2251	2483	2862	2173	2189	2455 .	2172	2146
2866	3314	2175	2575	2120			1751	2593	2067	2785	2837	2106			2026	2176	2928	2019	2426	2104	2783	2507 .	2250	2362
2556	2591	2084	3421	2009			1847	2024	2295	2614	3166	2112			2016	2313	2253	2528	2393	2431	2175	3095 .	3158	2198
2512	2564	2178	2390	1283			2832	2152	2547	2640	2235	2314			2164	2325	2146	2672	2426	2193	2041	2701 .	2608	2436
2321	2741	2257	2236				2083	2279	2434	2616	2280	2100			2083	2618	2519	2345	2490	2125	2549	2491 .	2343	2302
2320	2891	2693	2429				1925	2086	2381	2571	3784	3562			2725	2288	2203	2378	3613	2204	2157	2485 .	2429	2179
3168	2763	3648	2311				1624	2546	2371	2213	2378	3330			2165	2450	2292	2191	2886	2579	2821	2537 .	2288	2304
2615	3438	2964	2824				1651	2109	2484	3059	2321	2928			2394	2252	2829	2210	2509	2159	2427	3340 .	3322	2298
2629	3382	2120	3563				2498	2340	2319	2608	3797	3821			2030	3237	2855	3051	2627	2341	2101	3760 .	2426	2155
2470	2720	3751	3005				2217	2648	2216	2554	3728	2973			2036	2320	2340	2470	2619	2019	2027	2425 .	2442	3157
2709	2731	2453	2541				1976	2158	2508	3188	2655	2500			2894	2263	2151	2441	3424	2234	2525	2251 .	3172	2780
2536	3266	3078	2594				1876	2917	2108	2560	3436	2584			1984	3403	1950	2906	2789	2041	2023	2671 .	2436	2112
3098	3487	2995	3214				2734	2175	3736	2224	2622	3554			2709	3296	2311	2275	2538	3220	2000	3496 .	2378	2195
2499	2931	2795	2843				2116	3132	3389	3094	2551	3260			2197	2290	2673	2316	2989	2541	2468	3497 .	2430	2157
2289	2899	2914	2504				1909	2121	2041	2732	3132	2874			2050	2244	3126	3425	3403	2281	3286	2959 .	2407	2603
2638	3099	2581	2500				2917	2119	2453	2969	3126	2709			2386	3303	2416	2475	2464	2873	2251	2723 .	2589	2213
2674	2684	3833	3114				2148	2315	2447	2768	3560	3129			2489	2826	2056	2862	3124	2635	2432	2851 .	2684	2805
3108	3677	3114	3743				3655	2160	2297	3601	3333	3079			2897	2386	2307	2729	2928	3251	3252	2938 .	3545	4342
3261	4401	3781	3349				3496	3322	2903	3748	3372	3229			2900	2603	2506	2341	4307	3547	2967	4063 .	3679	3850
4047	3322	3641	3677				3458	2667	3663	4007	3186	4018			3317	3076	2898	2841	4142	4003	3280	3872 .	3278	2760
3573	3698	4708	3963				3378	3660	2725	3214	3445	3289			3252	3791	3320	3829	4141	3870	3151	3702 .	3373	3525
3491	3870	3688	3900				3400	3379	3829	3536	3764	2843			2458	3510	3319	3564	3583	2801	2957	3467 .	3654	3377
3043	3345	3602	3435				2952	3291	4060	2772	3762	3804			3009	4008	3565	2706	4288	3261	3665	3119 .	3007	2247
3461	3426	3733	3586				2982	2517	3655	3122	3230	3230			2892	3846	3265	3741	3466	3832	3517	3186 .	2969	2344
3055	3617	3325	2937				3090	2938	2947	3527	3107	3698			2889	3227	2947	2992	3707	3410	3088	3388 .	3020	3320
3671	3397	4493	3095				2208	2315	3501	3569	3300	3417			3165	4001	3231	3126	3361	2471	3439	4020 .	3349	3702
3255	3545	4152	3333				2451	2560	4093	3185	3751	2788			2755	3103	2492	3093	2935	3055	3293	3442 .	3844	3019
4075	4330	3323	2912				2684	2361	3535	2805	3663	3324			2971	2359	2134	2846	3049	2582	2963	2911 .	3453	2755
3646	3632	3269	3061				3136	2954	3319	2712	3949	2628			2239	2549	2838	3332	3954	3296	3672	3702 .	3152	2627
3490	3232	2967	4122				2834	3464	2811	3007	3068	2437			2020	3291	3422	3123	3401	2760	3709	3296 .	2682	3535
2725	3428	3163	3319				2261	2998	2736	2973	3287	2265			2968	3513	2374	3061	3189	3293	3889	3627 .	2928	3334
3074	3112	4299	2702				2644	3271	3878	2908	2831	2901			2801	3947	2635	3943	3351	3302	2927	3249 .	2695	2330
2899	2929	3998	2773				2440	2481	4057	3234	2960	2648			1926	3686	3404	3535	4424	2556	3081	3673 .	2752	2185
2761	3267	3621	3191				2110	2294	3492	3275	2731	2467			3188	2580	3509	2319	4612	2685	2656	3227 .	3653	3449
2985	2800	3148	3553				2296	2497	2827	3842	3931	2831			3130	2671	3713	2865	4553	2487	3292	3153 .	3252	3643
3154	2919	3344	2761				2136	2344	3262	3599	3327	3287			2839	2629	2719	3297	4056	2536	2855	3468 .	2653	2363
2792	3414	3632	2706				2103	2300	2904	3339	3311	2731			2389	2849	2340	3336	4025	2827	2734	2662 .	2373	2707
2769	3388	3553	3279				2416	3087	3088	2432	3009	2360			2663	3727	2276	2499	2838	2599	3140	2938 .	3106	2233
3684	2783	3026	2533				2366	3688	3712	3032	3003	2464			2343	3872	3468	2548	2903	2602	2951	3870 .	2765	3461
3641	3422	3674	2933				2526	2930	3053	2896	2779	2292			2014	3312	3381	3445	3973	2473	2663	3603 .	2594	3533
3394	3189	3382	2994				2493	2604	3038	2892	3067	3203			2420	1993	2571	2513	4009	3312	2734	3085 .	3330	2387
3196	2849	3360	3012				2045	2573	2668	2689	3444	2579			2173	3399	2420	2808	3006	3831	2911	3096 .	3509	2449
3135	2640	3222	2767				1940	3046	3445	2431	3029	2589			2770	3099	2569	2525	2806	3278	2902	2876 .	2822	2801
2818	3228	3503	2707				1895	2966	2852	2617	2933	2833			2910	2891	2731	2901	3444	2611	3475	2937 .	2513	2383
2753	3290	3003	2634				1714	2737	3395	3293	2817	3584			2109	3508	3454	2734	3815	2613	3896	3601 .	2491	3091
3215	2940	3450	2698				2874	2155	3317	3186	2794	2899			2577	2588	3695	2715	2442	3168	3478	3667 .	2877	2556
2618	2733	3304	2668				2806	2792	2482	3033	2843	2460			2931	2129	3196	2592	2608	2782	2704	2917 .	2076	2375
3692	3101	2445	2881				1997	2519	2922	2346	3187	2870			2253	3169	2947	3449	3840	2795	2378	3226 .	2876	2600
2727	2766	3048	2966				1790	2501	2650	2684	2782	3551			2209	3289	2582	2699	2962	3001	2706	3406 .	3428	2613
3153	3350	3260	3709				2805	2612	2211	2958	2588	3406			2894	2434	3088	2718	2507	3028	3271	3233 .	3325	2115
2516	3104	3814	3102				1957	3320	2949	2505	2247	2294			2599	2056	2194	2556	3501	2124	3524	2044 .	2758	2283
2096	2774	2812	2289				1625	2986	3326	2943	2784	2204			1948	2175	2573	3359	3085	2113	2334	2705 .	1995	3215
2195	2686	2401	1926				1680	2207	2238	2355	2647	2406			2238	3605	2288	3104	2701	3774	2630	3578 .	3394	2549
2419	2595	2289	2316				1745	2813	2038	2492	2218	2304			1956	2504	2619	1939	2693	2654	2950	2869 .	2712	2125
3134	2782	2468	2665				2010	2017	3583	3590	1956	3181			2049	2127	2002	2365	2479	2168	2250	2730 .	2398	2424
2320	2884	2773	2045				3023	2058	2276	2989	2586	3462			2030	2410	2181	2198	2574	2044	2198	2710 .	2328	2112
2308	3062	2244	2513				1912	3028	2162	2253	3510	2465			1966	2214	2193	2867	3077	2833	2241	2955 .	2228	2315
2389	2941	3240	2129				1601	1890	2683	2470	2463	2477			2373	2213	2206	2376	3584	2256	2784	2574 .	2174	2860
2786	2517	2525	3304				1677	2838	2058	2294	2038	2414			1919	2304	2552	2412	3576	2385	2328	2447 .	2338	2355
2323	2658	2609	2666				1819	2304	2132	2577	2108	2306			1898	2370	1924	2311	2191	2419	1986	2563 .	2400	2142
2328	2630	2183	2063				1941	2147	3474	2721	2676	2160			1934	2121	2167	2431	3549	2305	1993	2216 .	3080	2858
2515	3029	2441	2168				1877	2137	3340	2428	2382	2385			2646	2262	2544	3207	2517	3214	2625	2501 .	2637	2183
2083	2549	2397	2479				1832	2996	2199	2457	2248	2296			1834	2475	3267	2391	2311	2872	2112	2890 .	2267	2201
2316	3095	2321	2240				2827	1994	1968	2192	2614	2505			1797	2941	2487	2159	2289	2102	2220	2687 .	2252	2085
4021	3764	3710	3477 .		2797	1895	2094	2928	2431	3741	3644	3082	2326	2132	3141	3319	2963	3415	2500	2405	2376	2684 .	3417	3241
3817	3696	3379	3292 .		2737	1896	2971	2780	2162	3720	3396	2906	2640	2127	2357	3058	2601	2564	2559	2162	3009	2716 .	3287	3245
3374	3514	2736	3151 .		2170	2237	2125	2470	2688	3521	2764	2602	2028	2033	2484	2379	2062	2571	2963	2319	2260	2735 .	3049	3252
3438	2833	3152	2766 .		1985	2125	2053	3005	2223	3172	2708	3106	2485	2732	2041	2439	2292	2976	2525	2865	2405	2724 .	2977	2870
3564	2698	3105	3109 .		2789	1913	2274	2385	2998	3312	2710	2856	2408	2100	2675	2828	1920	2295	2916	2687	3180	3362 .	2431	3303
2613	3030	2619	2450 .		1890	2047	2204	2370	2133	2640	2708	2276	2644	2081	2614	2294	2191	2395	3277	2880	2319	2785 .	2858	2592
3323	2591	2560	2510 .		1982	1826	2147	2267	2472	2455	2816	2312	2107	2456	2285	2272	3096	2451	2685	2052	2552	3152 .	2649	2656
2892	2572	2698	2462 .		1798	2670	2092	2667	2767	2700	3392	2303	2860	2857	2320	2422	2689	2210	2684	2765	2668	2660 .	2302	2657

2531	2388	3306	2332 .	2789	2182	2417	2436	2937	3263	2569	2989	2957	2138	2603	2471	2554	2235	2875	2444	3005	2926 .	2328	3302
2754	2598	2760	2580 .	2309	2050	2465	2683	2382	2783	2819	2449	2630	2128	2426	2256	2169	2596	2878	2382	2471	3156 .	2478	2747
2834	2381	2846	2721 .	2113	2434	2036	2363	2874	3250	3121	2430	2436	2858	2431	2865	1869	2517	2733	2485	2665	2752 .	2860	2631
2571	2897	3052	2602 .	2550	2058	2608	2464	2464	2906	3981	2896	2517	3027	2506	2288	2665	2414	2704	2346	2468	2538 .	2584	3260
2965	3480	2898	2536 .	3138	2247	3637	2494	3361	3090	3008	2538	2939	3430	2778	2258	2201	2366	3700	3175	2715	2914 .	3007	3368
2697	4138	3293	3684 .	2549	2476	3468	2667	3070	3108	3602	3077	3422	3351	3060	2576	2241	3011	2991	3059	3381	3554 .	3496	2963
3504	3948	3742	3722 .	2837	2711	3247	3487	3557	3802	4102	3174	2494	2970	2900	2624	2725	3446	4112	3079	3482	3443 .	3261	3756
3622	3932	4083	3371 .	3089	2717	3001	2647	3043	3651	3619	3291	2494	2493	2474	3624	2724	2959	4009	2735	3879	3147 .	3293	3109
3571	3504	3821	3162 .	2639	2519	2580	2730	3524	2764	3555	2911	2782	2319	3463	2810	3310	2966	3496	2962	2908	3546 .	3632	3742
3728	2882	3683	3101 .	2919	3053	2298	2939	3165	3563	3950	2955	2811	2896	2687	2760	2814	2990	3308	3036	3189	4070 .	2707	3479
3121	3330	2967	2920 .	3078	2516	2377	2789	3141	3519	3642	3361	3398	3050	2741	3177	2900	3715	2936	3796	2934	3066 .	3064	2774
3294	3464	2919	2700 .	2821	2633	3130	2885	3183	2976	3605	3349	3427	2544	2521	2819	2688	2644	3326	3521	3083	3411 .	2971	3369
3307	2749	3652	2893 .	2661	2675	3193	2686	2910	2708	3128	2976	2622	2723	2353	2737	3481	3031	3156	2705	3091	2899 .	2830	3991
3391	2788	3705	3037 .	2629	3305	2377	3054	3731	2939	3296	2768	2743	2745	2575	3005	3678	3131	3647	3262	3247	3147 .	3002	3066
3186	3355	2824	2646 .	2638	3316	2460	2954	3748	3295	3127	2739	3109	2338	2568	2752	2878	2876	3583	2863	3012	3174 .	3599	3299
3853	3683	2673	2532 .	2599	2438	2951	3499	3101	2922	3443	3348	2219	2057	2492	2500	2460	2936	4157	2893	2858	3531 .	3457	3078
3773	3455	2749	3085 .	3210	2509	3201	2609	2728	2901	3423	3194	2680	2469	2914	2847	2402	2698	3272	2825	3250	3529 .	2861	2855
3353	2652	2922	2753 .	3456	2457	3162	2734	3033	3293	3384	2577	2686	2486	3054	3585	2854	2906	3343	2753	3034	3511 .	2867	3028
3114	3160	2699	2711 .	2671	2873	2160	2914	3172	3035	4037	2990	3135	3369	2326	3158	3697	3860	3311	2945	3247	3170 .	2925	3501
3445	3027	2988	3626 .	2537	2640	2233	2794	2893	4039	3431	2752	3111	3063	2708	2565	3476	2724	3452	2723	3660	3358 .	3983	3701
3076	3650	2897	3105 .	3420	2443	2477	2819	3335	3108	3434	2609	2661	1895	3116	3696	3195	2748	3282	3111	3939	3294 .	3630	2781
3252	3758	2898	2575 .	3071	3029	2990	3396	2977	3455	3858	2987	2942	1838	2718	3536	2379	3278	4468	3614	3559	3245 .	3178	2851
3114	2950	3803	2535 .	2659	3266	3396	3400	3790	3793	4390	3187	1998	2125	2510	2749	2353	2503	3929	2732	3212	4017 .	2612	3794
3193	3418	2842	2563 .	2635	2889	2454	2824	2842	3528	2744	3524	2372	1891	2596	2540	3027	2615	3001	2541	3074	3161 .	2229	2640
3577	3055	3192	2521 .	2972	2555	2849	2748	3032	2947	3388	2704	2505	2049	2511	2657	2584	3015	3651	3261	2690	3077 .	2973	3311
3466	3030	3613	2894 .	2585	2567	2258	2741	3186	3295	3246	3175	2346	2322	2511	3126	2590	2714	3131	2456	2750	3343 .	3008	3045
3584	3694	2721	2918 .	2528	2692	2843	2925	2975	3373	3643	3180	2546	2722	2693	2459	2545	2538	2780	2765	3477	2988 .	2535	3133
2674	3402	2746	2730 .	2603	2695	2952	2620	2773	2954	3544	3386	2856	3116	2720	3094	2777	2788	3001	2372	3098	3148 .	2775	3152
2943	2563	3126	2975 .	2447	2514	2484	2561	3586	2630	3431	3284	2320	2854	2879	3523	2753	3037	3157	2778	2742	3219 .	2654	2916
3007	2615	2776	3604 .	2577	3055	2529	2954	3438	3260	3223	2473	2211	1773	2691	2663	2892	2857	2975	2720	2990	2804 .	2659	3097
3030	3444	3648	2583 .	2615	2694	2385	3180	3119	2968	3227	2945	2363	2646	3077	3378	2858	2571	3742	3070	2773	3213 .	2614	2984
3193	2974	3605	2685 .	2711	2711	2061	2745	2733	2799	3167	2783	2488	1913	2092	3930	3678	2414	3959	2485	2675	3214 .	2516	2801
2991	3447	2680	2557 .	2867	2447	2752	2907	2846	3232	2960	2995	3152	2737	2729	3478	3139	2583	2901	2654	2734	2821 .	2963	3209
3235	2827	2953	2556 .	2443	2324	2464	2668	3009	3285	3387	2785	2290	2930	2857	2222	2704	3461	3769	2554	3685	3226 .	2814	2964
3401	3653	3386	2784 .	2401	2681	2475	3282	2726	3858	3171	2585	2510	2307	2698	2113	3114	3436	3572	3479	3483	2785 .	3200	2736
2879	3001	3481	2864 .	2830	2672	2857	2909	3454	3310	3042	2606	2192	1796	2144	2672	2538	3025	3040	3342	2651	3113 .	3329	3566
3237	2546	2365	3503 .	2090	2952	1738	2126	3447	2529	2746	3299	2290	1809	2950	3910	2527	2471	3191	2287	2344	3989 .	3669	2607
3021	3381	3562	3460 .	2348	2882	1921	2059	2495	3609	2335	3366	2284	1703	2559	3345	2251	2306	2771	2278	2154	3015 .	2536	3133
2469	2683	3317	2347 .	1986	1898	1873	2221	2450	3198	3422	3104	2248	3183	2312	3383	2298	3274	2408	2473	2175	2850 .	2409	2575
2492	2868	2881	2553 .	2071	1947	1926	2140	2237	2950	3373	2373	2734	2838	2249	2485	2024	2017	2960	2420	2717	2653 .	2490	2287
2799	2467	2443	2559 .	2155	1862	2387	2492	2449	2800	2499	2440	3339	1691	2310	2164	2901	2212	2581	2814	2358	3014 .	2518	2568
2505	2692	2354	2491 .	2442	1979	1819	2315	2234	2587	2512	2804	2246	1786	2646	2988	1991	2162	2570	2417	2340	2811 .	2519	3216
2825	2848	2484	2379 .	2068	1748	2678	2089	2638	3049	2543	2602	2072	1730	2339	3006	2142	2671	2483	2775	2238	3204 .	3017	2742
2459	2667	2327	2622 .	2222	1744	1929	2150	2789	2805	2456	2812	2689	1957	2339	2086	2136	1893	3348	2251	2756	2723 .	3413	2760
2697	3099	3283	2441 .	2014	1860	2511	2454	2477	2666	3337	2574	2337	2367	2573	2282	2363	2827	3022	2445	2365	3179 .	2454	2974
2275	3121	2325	2937 .	1969	2714	1980	2069	2208	2925	2557	2421	2510	1799	2580	2399	2882	2435	2374	2420	2261	2556 .	2538	2543
2802	2679	2313	2654 .	1978	1836	2096	2143	2456	2709	2577	2580	2958	2674	2277	2299	1937	2244	2509	2625	2375	2486 .	2555	2626
2415	2668	3452	2743 .	1967	1870	2054	2369	2987	2986	2581	3055	2168	1901	2738	2839	2202	2316	3162	2125	2529	2821 .	2344	2781
2345	2486	2489	2573 .	2612	2031	2547	2217	2686	2560	2759	2486	2304	1943	2393	2218	2216	2204	3405	3076	2416	3104 .	2312	2344
2954	2825	2370	2462 .	2023	1711	2069	2828	2224	2719	2776	2274	2603	2587	2348	2987	2149	2287	2559	2308	2350	2576 .	2500	2799
2423	2936	3201	3218 .	2270	1974	2384	2096	2197	2722	3549	2421	2208	1869	2429	2378	2025	2812	2582	2200	2398	2721 .	2503	2550
3142	2827	2487	2393 .	2242	2114	2939	2148	2323	2882	2945	2366	2134	2081	2383	3266	3048	2437	3679	2206	2342	2833 .	3016	2605
2430	2683	2662	2354 .	2053	2696	1856	2514	2347	2755	2733	2447	2300	1993	2331	2543	2752	2482	2505	2393	2686	2792 .	2721	2540
2770	2200	3456	3326 .	2594	2232	1986	2736	2571	2765	2862	2944	2281	2745	2200	2529	2362	2546	2846	2153	2419	2816 .	2503	2937
2951	2825	2631	2737 .	3214	2008	2182	2325	2189	2781	2525	2606	2663	2125	2808	3102	2484	2582	3202	2364	2509	2724 .	2649	2762
2460	2596	2427	2409 .	2187	1916	2063	2512	2301	2663	2603	2458	2233	1923	2477	2377	1931	2530	2619	2470	2480	2747 .	2463	2674
2641	2609	2786	2627 .	1933	2119	1783	2551	2594	3119	3349	3010	2018	1705	2477	2447	2145	2637	2941	2593	3160	2996 .	2624	2627
2390	2822	2536	2441 .	2242	1992	2569	2311	2178	2598	2523	2556	2372	2302	2450	2461	2699	2196	2556	2158	2121	3366 .	2640	3009
2408	2546	2808	2355 .	2120	2425	1821	2096	2186	3413	2706	2664	2305	1819	2597	2224	2107	2348	2690	2245	2668	3161 .	2472	2685
2467	2625	2645	2525 .	1891	1777	2639	2280	2187	2835	3113	2623	2059	2231	2425	2385	2091	2825	2905	2264	2612	2605 .	2299	2729
3141	3414	2732	2546 .	2334	2382	2891	2221	2388	2587	2508	2445	2005	2770	2552	2987	2137	2460	2891	2574	2478	2569 .	2476	3243
2732	2553	2667	2598 .	2152	2119	1805	2937	2183	2699	2638	2439	2403	1886	2345	2441	1956	2243	3038	2148	2442	3086 .	2294	2719
2679	2489	3486	3316 .	2064	1825	1901	2328	2545	3171	2723	2444	2564	2666	2534	2780	2681	2696	2648	2388	2295	2675 .	3390	2746
2833	3078	2788	2729 .	2531	2064	3256	2733	2941	2714	3409	2966	2014	2466	3315	2431	2322	2274	3183	3068	2165	3452 .	2520	2627
2822	3041	2708	2521 .	2786	2096	2304	2312	2382	3041	3586	2976	2264	1891	2564	3501	3141	3037	2739	2231	2897	2737 .	2851	2746
2415	3492	2670	2526 .	2263	1937	2060	2201	2591	2791	2849	2435	2235	2157	2483	2310	2250	3060	2733	2294	2670	2930 .	2624	2572
3674	2777	2536	2563 .	2231	2281	2231	2860	2733	3117	2841	2872	2149	2391	2643	2426	1935	2227	3711	2226	2682	3041 .	2281	3132
3810	3735	2881	2702 .	2023	2109	2321	2136	2520	3353	2742	3383	2560	2054	2828	2660	2199	2434	3775	2729	2860	2679 .	3364	3318
3131	3282	3879	3305 .	2715	2166	3586	2306	2495	3101	3405	2719	2582	2625	2999	3648	2064	2669	2649	2714	2474	3330 .	2842	3548
2657	3261	2765	2549 .	2421	2781	3655	2830	2728	3088	3289	3062	3368	2548	3345	2527	2676	2803	3337	3079	2922	3326 .	2723	3313
3280	3044	3204	3175 .	3354	2306	3347	2476	3725	3390	3380	3618	3070	3589	3216	2898	2212	2581	3289	2901	3033	3140 .	2709	3896
3655	3313	3487	4323 .	3369	2978	3208	3506	3714	3549	4258	3583	2770	3739	2893	2722	2421	3059	3665	3288	3594	3719 .	3683	3221
3452	3814	4138	4267 .	3170	3190	2788	3849	3432	3802	4254	3535	2712	3033	3140	3200	2591	3382	3858	3438	3862	3470 .	3822	3369
4022	3941	4464	3354 .	2399	3321	2588	3283	3310	3106	3018	3242	3599	2546	3581	3711	3144	2864	3228	3173	3580	4123 .	3886	3851
3218	3404	3946	3239 .	2660	2483	2581	3034	3213	3706	3928	3282	3214	3225	2781	3715	3098	2774	3208	2880	3325	3573 .	3497	3816
3464	3232	3007	3682 .	2500	2445	2970	2832	3731	3233	2988	2631	2875	2941	2988	3243	2495	3083	4314	3049	2856	3962 .	3581	3255
2959	3481	3601	2883 .	2615	2742	3380	2506	2980	3149	3410	2601	3012	2115	3022	2768	4010	3579	3447	3164	4046	3545 .	3588	3337
3601	2825	3777	2711 .	3094	3081	2784	2536	3058	2888	3002	3156	3393	2124	3376	3855	3739	2888	3379	4026	3818	3360 .	3056	3115
3731	3046	3475	2920 .	3583	3449	2604	3377	3869	3296	3125	3511	2727	3105	2707	3843	2513	2653	3645	3671	3376	3578 .	3438	3322
2904	4022	2961	2511 .	3257	3087	2807	3464	3654	3209	3855	2838	3774	3280	2751	3855	2791	3589	3479	2612	3052	3424 .	2802	2858
2841	3383	3256	2485 .	2421	2505	2673	3053	3410	3209	4024	2638	3575	3457	2783	3226	2576	3578	3366	3545	3130	3793 .	3177	3616
3324	2583	3472	2795 .	2833	2313	2181	2565	3447	3271	3953	2960	2783	2890	2861	2986	2882	3221	3400	2806	3148	4316 .	2956	3731
3160	3235	3510	3249 .	3357	2720	2952	2947	3657	2829	2708	3169	2850	2073	3527	3243	3369	2948	3738	2751	3146	3397 .	3632	2824
3246	2928	3461	3340 .	3084	3105	2546	3174	3637	2929	3275	3238	2871	2101	3325	4071	3083	2848	4186	2902	2965	3430 .	3028	3540
3716	3006	3432	3088 .	2545	3283	3347	3573	3481	3430	2917	3123	3125	2131	3029	3835	3945	2850	3504	3368	3007	3238 .	3002	3261
2801	3043	3285	3414 .	2500	2442	2213	3381	3234	3866	4016	3183	2637	2236	3016	3037	3576	2748	3911	3666	2975	3831 .	3499	2767
2749	3033	3294	3473 .	2525	2419	2006	2372	3337	3801	3133	3159	3136	2935	2780	3274	3173	2560	3623	2639	3734	3853 .	2973	3445
3000	2887	3065	3330 .	2687	2475	2750	2616	3486	3435	3336	3152	2967	3219	2755	3616	2608	2823	4783	2723	3811	3665 .	2610	3225
3236	3000	3697	2856 .	3038	2435	2253	2758	3084	2747	3207	3161	3102	2122	2897	3392	2576	3511	4243	3135	3602	2888 .	2998	4174
3321	3270	4148	2450 .	2530	2557	3029	2424	3943	3075	3834	3559	2991	1889	2551	2935	2417	3677	3276	2694	3133	4056 .	3394	3046
3519	3824	3572	3076 .	2148	2447	2782	2588	2884	2950	3210	3082	2875	2366	2592	2789	3266	2539	3856	2799	3362	3903 .	3165	3301
2738	4007	3044	2525 .	2631	2711	2764	2612	3267	3238	3145	2791	2957	2770	2460	2992	2419	3016	3141	3048	3168	3203 .	2813	3097
3209	2967	3616	3064 .	2465	2549	2975	3182	2858	2932	3538	2839	2888	2139	3500	3572	2606	3052	3826	2740	3227	3459 .	3244	3522
3139	3621	3442	2866 .	2321	2419	2044	2588	3082	2932	3297	3126	3165	2328	2509	2812	3056	2707	3782	3082	3193	3171 .	3190	3133
2772	3475	3064	2846 .	2351	2517	2056	2846	3707	2834	3288	2957	2847	3246	2663	2431	3575	2645	3195	3058	3215	3168 .	2978	2839
3148	3238	3189	2851 .	2624	3041	2057	2401	3892	3139	3262	2772	2490	3025	2649	3129	2597	2780	3100	2830	2915	3444 .	3151	2854
3334	2941	3710	2900 .	2451	2624	2213	2779	3273	3092	3038	2616	2599	2030	2674	2721	2540	2722	3187	3580	3084	3464 .	2804	3276
2531	3501	3101	3284 .	2358	2042	3075	3334	3747	2437	3732	2666	2953	2522	2846	3128	2780	3102	3586	3291	2836	3053 .	2919	3135
2956	3135	3434	3633 .	2430	2781	2192	2746	3898	3259	3068	2968	2740	2368	2477	2648	3017	2736	3596	2845	2792	3000 .	2661	3261
2791	3808	3104	3315 .	2382	2899	2019	2938	3482	3316	2912	3317	2951	2132	2420	2861	3628	2649	3095	3368	3806	3353 .	3385	3176
3156	3441	3415	2283 .	2282	1845	2549	2463	2745	3422	3107	3006	2597	2751	2380	4016	2607	3420	3699	3635	3488	3018 .	3366	3609
3127	2645	3689	2438 .	3307	2087	1841	2570	3271	4032	3539	2732	3491	2671	2984	2974	2351	3748	3251	2671	3549	3641 .	3028	3449
2739	3595	3839	2704 .	3344	2030	1738	2178	2682	3837	3426	2371	3283	2051	2215	2507	2323	3190	3197	2633	3213	2658 .	3556	3274
2534	2506	2914	2693 .	3090	2067	1991	3305	3192	3206	2953	2600	2495	1772	2070	2183	2204	3255	3486	2469	2680	2767 .	2385	2652
2816	2784	3373	2595 .	2029	2032	3093	3316	2469	3251	3068	2791	2220	1730	2245	3326	2940	3113	2957	2618	3176	2848 .	2410	3272
2318	2788	2867	3243 .	2063	2653	2626	2545	2728	2705	3075	2984	2981	1997	2316	2486	3086	2253	3073	3143	2806	3100 .	2196	2740
2283	3089	2275	2541 .	1838	1864	1802	2900	2631	2403	3136	2865	2135	1662	2258	2197	2237	2936	3667	2432	2888	2866 .	2624	2870
2522	2559	2632	2732 .	2227	2118	1865	1980	2572	2844	2916	2440	2051	1722	2462	3034	2065	2348	4006	2295	2545	2604 .	2591	2850
2658	2978	2882	2777 .	1944	2182	2646	2105	2658	3013	2587	2482	2367	2084	2260	2239	2685	2749	3436	2302	2576	3057 .	2381	2975
3226	2993	2687	2433 .	2471	1972	2683	2723	3119	2536	3013	2467	1830	1660	2248	2393	2459	2756	2445	2434	2454	3075 .	2408	2755
2417	2963	2885	2541 .	1865	2617	1755	2048	2636	2441	2963	2691	2437	1831	2524	3013	2239	2697	2558	2375	2570	2723 .	2760	2729
2378	2713	2878	2574 .	1776	2078	1896	2200	2419	3234	3267	2549	2926	1773	2478	2333	1898	2370	2614	2893	2356	2658 .	2392	3041
2923	3148	2529	2717 .	2162	2222	2182	2694	2691	2750	2926	2563	2109	1900	2337	2500	1877	2948	2575	2238	2439	2829 .	2404	3283
2394	2604	2682	3248 .	2278	1873	1826	2265	2413	2474	3199	2566	2361	2162	2234	2472	2425	2973	3349	2408	2333	3406 .	2478	2467
2946	2758	2482	2571 .	2053	2109	2316	2317	2558	2723	2941	2574	2279	1802	2643	2935	2135	2421	2499	2204	2878	2427 .	2655	2819
2589	2793	2635	2726 .	2051	1930	1719	2658	2620	3093	3096	3172	2921	1914	2285	2327	2046	2493	2780	2510	2330	2534 .	2684	2524
2407	2884	3606	2527 .	2236	2553	2989	2270	2735	2829	2939	2949	2726	2735	2115	2607	2511	2468	2568	2540	2299	2848 .	2487	3099
2580	3185	2659	2518 .	1897	1934	2596	2645	2568	3454	2717	2417	2200	2035	2310	3020	3023	2634	2925	2363	2202	2745 .	2454	2793
2492	2852	2520	2476 .	2176	1983	2049	2217	2522	2588	2862	2623	2262	2123	2555	2407	2159	2318	2332	2254	2118	2965 .	2256	3349
3594	3534	2657	2693 .	2018	2348	1921	2799	2693	3315	2634	2578	2441	1908	2385	2657	2281	2211	3471	2689	3030	2758 .	3678	3389
2578	2705	2327	2553 .	2233	2209	1903	2445	3012	2560	3325	3148	1999	2094	2511	2616	2678	2401	2397	2478	2678	2594 .	2458	3013
2538	2835	2524	3244 .	2473	2032	2051	3149	2967	3074	3198	2497	2373	1932	2446	2758	2275	3169	2533	2592	2360	2375 .	2602	2768
2917	2732	3459	2714 .	2037	2186	2028	2241	2446	2808	2691	2600	2060	1906	2558	2462	2260	2640	3155	2748	2457	3157 .	2441	2638
2561	2860	3331	2260 .	1714	2043	2762	2274	2388	2629	2935	2959	2262	1993	2313	2599	2574	2853	2474	2284	2495	2837 .	2486	2629
2396	3333	2527	2324 .	1644	2671	1791	2427	2721	2914	2582	2552	2226	2133	2249	2788	2266	2603	2683	2230	2504	2878 .	3179	2872
2597	2715	2654	2748 .	2705	2450	1920	2445	2354	2941	2660	2488	2099	2613	2118	2219	2074	2489	2567	2682	2303	2341 .	3135	2845
2822	2765	2506	2375 .	2281	1907	2614	2311	2473	2728	3506	2564	2366	1766	2291	2450	2071	2758	2722	2529	2387	2704 .	2611	3126
2744	2579	2912	2770 .	1974	2155	1992	2879	2882	2873	3772	2614	2347	1773	2462	3028	2279	2619	2307	2400	2299	2736 .	2576	3320
2669	2787	2550	2528 .	2208	2572	2011	2362	2729	2564	2806	3182	2414	2615	2364	2403	2276	2794	2771	2367	2634	3517 .	2741	2781

2969	3573	2498	2856 .	2219	1951	2556	2385	2659	3147	2755	2482	2214	1891	2482	2642	2175	2631	2533	2314	2958	2961 .	2578	2808
2586	2812	2896	2725 .	2085	2471	2830	2626	3144	2768	4047	3757	2205	1832	2618	3816	3544	2802	3214	2613	2666	2682 .	2473	2688
2751	3532	3676	3931 .	2070	2153	1941	2827	3346	3446	3571	3540	2672	1996	2867	3744	2210	3037	2729	2535	2318	2649 .	2660	2849
2559	2763	2864	3096 .	2733	2070	2093	2908	3337	2927	2760	2564	3072	2311	2637	2796	2402	2719	2536	2705	2489	2772 .	2584	3543
2881	2835	3076	2924 .	2231	2191	2165	2519	2770	2691	3183	2818	2753	2839	2807	2786	2740	2810	2598	2707	2618	3132 .	3278	3305
3014	2939	3075	2697 .	2672	2833	3627	2890	2557	3248	3424	3094	3145	2289	3016	2857	2540	2964	3455	3307	3079	3508 .	2431	2915
2947	3021	3271	3064 .	3091	2339	3596	2901	2975	3328	3556	2795	3593	2711	3206	3270	2528	2914	2915	2739	3575	2996 .	2815	4143
4134	4256	3479	3378 .	3007	3285	3405	3342	3557	3500	4223	3600	3402	3796	2882	2929	2959	2909	4003	3241	3666	3730 .	3782	3762
3240	4086	3472	4174 .	3864	3394	3127	3862	3577	3807	3643	2852	2695	3269	3371	3666	3357	3152	4487	3621	3846	3373 .	3871	3643
3744	3468	4084	3606 .	3585	3427	2396	3357	3788	3824	3328	3722	2621	3204	3110	3755	3309	3569	3847	2681	3658	3538 .	3233	3270
3487	3142	3443	3111 .	3171	2670	2089	2844	3165	2748	3986	3122	3204	2501	3398	3790	3429	4124	3243	3500	3891	4037 .	3382	3237
3058	3185	3622	3088 .	2893	2699	2952	2731	3438	3725	3464	3324	3212	2449	2717	3199	3274	3506	3230	2852	3651	3200 .	3460	3796
3699	4226	3298	3116 .	2645	2544	3043	2845	3380	3974	3448	3752	2954	2322	2908	3789	3012	2649	3957	2801	3495	4044 .	3405	3056
3636	3666	3104	2727 .	3085	2456	2445	2794	4179	2812	3519	3628	2933	2334	2958	3587	2832	2803	4285	3776	3839	3923 .	3023	3538
2727	3171	3900	2710 .	3479	2516	1993	2732	3625	3427	3170	2906	2705	2875	2490	2931	3664	2721	4122	3067	4043	3382 .	3207	3864
3282	3078	3840	3042 .	3092	3165	1969	2791	3587	2917	3425	3176	3180	3231	2848	3359	3628	3182	3943	2803	3520	3025 .	3229	3023
3357	2712	3239	3251 .	2747	3557	2111	2781	3385	2674	3737	2751	3441	2710	2689	2984	3300	2867	3311	3071	3371	3131 .	3003	3084
3400	3336	3101	2703 .	2924	2926	2711	3905	3164	2691	3278	3307	3183	2409	3334	4150	2835	3382	3316	2901	3550	2943 .	3635	3546
2513	3285	3480	3305 .	2601	2929	2513	3337	3539	3113	3724	3117	2244	2235	3669	3146	2832	3488	3372	3033	3946	4019 .	3356	3655
3303	3859	3901	3855 .	2644	2565	1859	3059	3562	3056	3353	2938	2646	3104	3050	3132	3304	2543	3464	2882	3815	2962 .	2713	3279
3654	3403	3224	3122 .	2973	2930	2417	2916	3349	3051	3069	3449	3040	3420	2316	3473	3915	2935	3557	2711	3634	3147 .	2998	3407
3765	3036	2916	2990 .	3549	2875	2854	3350	3290	3048	3757	3854	2524	2993	2456	4012	2956	3392	4082	2820	3230	3476 .	3004	4120
3214	3007	2838	3062 .	3469	2504	2399	3313	3678	3135	3696	3313	3123	1906	2706	3726	2712	2840	4357	3629	2903	3296 .	2952	3013
2992	3435	2873	2883 .	2614	2582	2231	2537	3930	3053	3332	2899	3393	1932	3209	3050	2826	2990	3466	3765	3230	3395 .	3030	3486
2976	2990	4115	3343 .	2566	2546	2581	2774	3366	3720	3663	2814	3043	2451	2995	3772	3021	2585	3505	3003	3579	3918 .	2894	3312
2883	2915	3755	2984 .	2530	2951	2033	2184	3445	3056	3437	2961	2837	2086	2508	3748	2979	3263	3134	3545	3066	3695 .	3227	3059
3341	3646	3345	3253 .	2525	3193	2242	2566	3470	2992	3444	2947	2642	2006	2809	2952	3615	2687	3506	3109	3364	2943 .	3840	2897
2717	3370	2984	3525 .	2485	2298	2342	2915	3938	2994	3389	2831	2676	2861	2646	3206	2811	3028	3918	2866	4183	3484 .	3332	3446
3110	2924	3065	3711 .	3161	2537	2159	2764	4089	3053	3246	3041	2819	3045	2636	2926	2728	3449	3214	3258	3875	3674 .	2834	2940
3366	2951	2995	2536 .	2768	2623	2562	2373	3762	3251	3398	3148	2752	2225	2758	2933	2961	3365	3131	3446	3427	3060 .	3404	3050
2557	3139	3298	2848 .	2488	2346	2300	2219	3319	3581	3182	3329	2639	1830	2556	3488	2929	2284	3883	2896	3077	3060 .	3186	3123
3208	3222	3552	2832 .	2894	2690	3113	2972	3771	2819	3736	2769	2675	1746	2653	3595	2898	2623	3207	3067	3301	2942 .	3270	3114
3095	3077	3420	3179 .	2195	2969	2172	2969	3163	3273	3883	2788	2587	2190	2500	2571	3386	2537	3129	3051	3003	3303 .	2712	3031
2928	3640	2990	2792 .	2406	2377	2216	2945	3591	3239	3180	2851	2610	2265	3195	3137	2858	2621	3154	3607	3288	3163 .	2881	3243
2702	3788	3335	3042 .	2780	2766	2073	2768	3758	2996	3318	2824	3169	3060	3056	3018	2649	4011	3092	2845	3062	3368 .	2509	3507
2932	3377	3213	2940 .	2473	2286	2278	2692	4130	3225	3403	3415	2673	2227	3204	2773	2826	3559	3044	2544	2911	3605 .	2970	2848
2780	3283	2708	3368 .	2966	2372	3416	2969	3106	3847	2943	3137	2215	2001	2326	2684	3316	2865	3996	3366	3056	3776 .	2991	2668
3387	3207	3915	3200 .	2459	2447	2620	2421	2785	3541	3197	2579	2500	2661	3246	4228	3688	2631	3914	3719	2722	3411 .	2765	2881
2492	2752	4009	2831 .	2042	3173	2152	2472	3579	3179	3742	2634	3029	2392	2943	3554	3120	3271	3473	3256	3403	3674 .	2692	4169
3288	2876	3776	3463 .	2020	2928	1670	2591	2987	3302	2658	2600	2676	1687	2299	2326	2551	3328	2820	2452	3061	3656 .	2560	3304
2332	2842	2497	2485 .	2077	1889	2136	3192	2724	2557	2889	3240	2062	1718	2647	3195	2483	3558	3078	2579	2637	3184 .	2400	2804
2312	2715	2816	2890 .	2784	2269	2076	2215	3461	3265	2853	2695	2791	1812	2350	3335	2274	3218	2848	2888	2961	2408 .	2393	3278
2984	2951	2642	2557 .	3384	2461	2369	2019	2439	2512	2520	2658	2268	2911	2326	2358	2983	2778	3609	2315	2495	2540 .	2513	2888
2454	3006	2677	2905 .	2171	2250	1805	2254	2534	2521	2845	2742	2725	2078	2276	2453	2975	2367	3105	2736	2357	2699 .	3162	2865
2694	2770	2478	2624 .	2163	2051	1632	2825	3007	2574	2965	2614	2190	2110	2589	2482	2636	2286	2655	2995	2649	2916 .	2661	2891
2737	2705	2640	2397 .	2126	1883	2870	2122	2683	2445	2794	2614	2627	2087	2365	2456	2099	2330	2669	2368	2838	2900 .	2429	2655
3329	2480	2694	2796 .	2370	1931	2708	2085	2899	2447	2751	3345	2281	1807	2605	3465	2318	2944	2898	2554	2719	2774 .	2431	2756
2645	2819	3111	2614 .	2265	1872	1984	2199	2775	2556	2911	2801	2916	2568	2595	2240	2238	2290	3543	2566	2683	2825 .	2926	3246
2589	2566	2707	2725 .	2131	2478	2134	2271	2565	2953	3170	3022	2300	2584	2914	2380	2199	2933	3099	2510	2792	3313 .	2417	2877
2485	2761	3013	2492 .	1901	1969	1749	2307	3001	2530	2958	3103	2228	1766	2826	2644	2378	2240	2622	2222	2626	2914 .	2399	2621
2895	2497	3116	2498 .	2190	2135	2002	2125	3588	2628	2906	2587	2515	1931	2627	2634	2507	2244	4410	3333	3494	3288 .	2515	3818
2498	2908	2887	2525 .	2226	2067	2480	2507	2760	2313	3065	2628	2802	1856	2551	2588	2276	2829	3842	2517	2478	2918 .	2373	2907
2662	2719	2655	2330 .	2097	1847	2145	2648	2400	2300	3112	2401	2291	1729	2441	2500	2282	2499	2753	2428	3008	3056 .	2628	2620
2776	2732	2875	2377 .	2195	2021	2031	2411	2364	3432	3349	2812	2129	1925	2551	2805	2275	2381	2725	2344	2986	2942 .	2452	2828
2547	2982	3278	2536 .	2056	2236	2387	2352	2552	2973	3244	2528	2233	1943	2267	2450	2166	2781	3521	2770	3183	3025 .	2511	2669
2927	2632	2690	2352			2478	2240	2383	2513	3090	2244			2598	2446	2450	2348	2598	2526	2730	3187 .	2404	2798
2630	2709	2609	2945			2363	2672	2348	2654	3324	2636			2388	2414	2915	2497	2513	2557	2447	2726 .	2729	2478
2499	2885	2660	3429			2057	2181	2769	2822	3131	2813			2222	2881	3038	2411	3155	2203	2737	2832 .	2467	2866
2879	3216	3305	2524			1863	2500	3093	2498	2896	2469			2442	2520	2355	2311	3276	2555	2765	3129 .	2417	3062
2605	2984	2659	2392			1881	2642	2385	2986	3272	2789			2568	2429	2188	2820	2757	2364	2523	2580 .	2612	2846
2872	2927	2647	2331			2570	2676	2476	2732	3558	2912			2394	2625	2699	2536	2954	2287	2704	2733 .	2424	2867
2563	2794	3035	2736			2739	2356	2709	2658	2916	3165			2629	3113	2344	2557	2860	2246	2634	3422 .	2787	2629
2675	2976	3476	2534			1965	2690	2359	2790	2884	2876			2581	2557	2388	2846	2647	2763	2684	3275 .	2610	2742
2603	2858	2662	2513			1785	2224	3149	2853	2799	2969			2442	2525	2248	3270	3075	2406	2465	2758 .	2427	3039
2502	2976	3081	2442			2189	2348	2499	2600	3008	2694			2745	2468	1989	2429	3161	2276	3058	3179 .	2439	2918
2862	2974	3434	2420			2042	2250	2389	2892	2735	2622			2767	2580	2485	2473	3279	2402	2750	2972 .	2417	2922
3224	2940	2829	2595			2818	2701	2516	3249	2804	2580			2383	3062	2115	2638	2835	2720	3020	2942 .	2416	3274
2762	2613	2722	2364			2216	3372	3123	2871	2964	2849			2444	2571	2331	2753	2720	2323	2875	2943 .	2401	2754
2422	3163	2773	2731			2192	3067	2557	3157	3256	2627			2493	2475	2236	2510	3081	2693	2552	2656 .	2603	3049
3221	2885	2688	3466			2272	2326	2417	2892	3166	3058			2963	3277	2476	2646	2866	2644	2974	3112 .	2789	3270
3209	2961	3231	2853			2699	2994	2550	2736	3337	2545			2539	2397	2297	2393	3455	2670	3378	2776 .	2442	2999
3337	2914	2859	2747			3782	2628	2912	2985	3371	3462			2914	3185	2208	2980	3179	3238	3212	3052 .	2953	3525
3234	3868	3378	3918			3723	2538	3607	3129	4040	3210			3489	2728	2449	2719	3681	2877	3268	3610 .	4099	4223
3959	3947	3316	3789			3534	3878	3465	4068	4138	2677			3465	2870	2909	2598	3963	3051	3802	3121 .	3614	3994
3401	3613	4466	3167			3071	3636	3042	3721	3376	3777			2872	4211	3053	3910	4107	4174	3896	4004 .	3214	3724
3403	3626	3213	3118			2879	2923	3231	3027	3626	3881			2743	3634	3264	2799	3665	3581	3712	3710 .	3021	3510
3660	3861	3230	2768			2413	3075	3746	3637	3939	3366			2906	3218	3652	3528	3097	2764	3463	3671 .	3432	3238
3775	3701	3272	3344			2224	2654	3964	3658	3788	3096			2908	2805	3661	3730	3105	3375	3335	3444 .	3547	3828
3322	3246	3312	4270			2233	2421	3283	3595	3279	2887			2929	3377	3246	2974	3288	3056	3049	3702 .	3362	3908
3365	3157	4249	3800			3194	2829	3353	3033	3167	3221			3095	4031	2884	2943	3504	3068	3596	2985 .	3218	3856
3298	3434	4046	3194			3021	2783	3195	3007	3022	3469			3012	3706	2825	3314	3343	3484	3227	4157 .	3127	3719
3314	3780	3646	3080			2687	2802	3187	3040	3326	3142			2849	2956	3417	3338	3289	3282	3816	3413 .	3088	3482
3501	3563	3716	2779			1975	3672	3279	2973	3500	3003			2718	3141	3054	2876	3490	3384	3573	3743 .	3124	3251
3107	3381	3975	2954			2485	3480	3351	2836	3346	3177			2990	3180	3854	3941	4657	3005	3394	4076 .	3915	3176
3338	3528	3416	3235			2478	2783	3932	3288	3841	3132			2883	3320	3581	3353	4337	3158	3282	3485 .	3172	3248
3109	3125	3309	3262			2224	3003	3983	2991	3137	3426			2920	4003	3641	2975	4107	3411	3427	3507 .	3165	4043
3915	3147	3084	3049			3266	2454	3419	3327	3106	3449			3025	3438	2802	3119	3348	3417	3085	3265 .	2960	3769
2936	4137	3202	2965			2911	3063	3084	3081	3673	3054			3521	3221	3041	2720	3357	3766	2989	3721 .	2633	3051
3113	3405	3478	2971			2296	3071	3133	2752	3036	3009			3171	2772	3272	2680	3617	3253	2934	3928 .	3201	3121
3060	3117	3668	2804			2675	3045	3244	2990	3278	3030			2625	3922	2997	2797	3594	2864	3055	3422 .	3169	3187
2879	3147	3340	3295			2568	3616	3733	2789	3414	3209			2597	4039	3269	2866	3275	3054	3442	3320 .	3190	3440
2867	3766	3444	3940			2692	3123	3536	3391	3363	3204			2826	2766	2952	3249	4289	3227	3134	3509 .	2843	3044
2973	3278	3109	2915			2179	3203	3095	2979	3036	3580			2415	3031	3240	3303	4156	3203	2825	3254 .	2780	3251
3654	3315	3191	2834			2403	2963	3395	3090	3051	3639			2743	3300	2595	2656	3662	2787	3240	3712 .	3072	3120
2924	3370	3155	3002			2778	3005	3155	3356	3032	2614			3531	3458	2787	2892	3126	2911	2994	3285 .	2776	3470
2894	3341	3132	2737			2244	3030	3291	3933	3128	2639			3091	2800	2999	2923	3277	3147	2854	3259 .	2588	3916
3055	3338	3602	2933			2466	2819	3327	3428	2914	2955			3084	2752	3300	2716	3117	2908	3175	3739 .	3573	3152
3460	2803	3307	2582			3058	3029	3181	2662	3831	2876			3125	2726	2861	2901	4263	3056	2878	3804 .	2999	3225
2994	2837	3129	2760			2752	3344	3981	2953	3743	2865			3494	2922	2556	3031	3506	3302	3109	3170 .	2763	3303
3010	3610	2920	2735			2779	2957	3835	2972	2901	3028			2436	2772	2672	3441	3921	2756	3502	3025 .	2574	3643
2942	2948	3223	2871			1873	2783	3177	2742	2977	2831			2479	2940	3117	3458	4226	2754	3216	3637 .	2903	3154
2743	2896	3282	2794			2294	2602	2810	3803	3329	3586			2854	2742	3875	2494	3107	2813	3102	2782 .	3763	4276
2586	3490	4114	2636			2629	2526	3313	3300	3177	2686			2274	3545	2832	3027	3268	2663	2733	3020 .	3467	3764
2830	3060	3133	3614			2343	2586	2841	2564	3167	2828			2289	3771	2858	2699	3120	2560	3262	3370 .	3226	3118
3414	3521	3503	3756			1718	2630	3373	3764	3072	3207			2521	3379	2427	2385	3127	3783	3259	3056 .	2506	3571
3376	2778	3235	3362			1896	3412	2579	3327	2830	2489			2366	2161	2050	3395	2835	3252	2481	2204 .	2386	2755
2585	2986	2387	2395			1912	3159	2561	2795	2989	2366			2248	2511	2596	3462	3235	2249	2703	2415 .	2292	2733
2709	3141	2506	2463			2725	2375	2540	2833	3007	2663			2276	2771	2595	2348	3719	2397	3081	2791 .	2342	2801
2616	3176	2502	2592			1898	2294	2444	2705	2638	2577			2282	2835	2404	2595	3336	2297	2563	2481 .	2317	3378
2461	2536	2389	2948			3252	2215	2167	2762	3286	2966			2164	2366	2238	2390	2429	2739	2654	2944 .	2986	2726
2636	2882	2980	2234			2262	2404	2453	2378	2497	2653			2305	2571	2496	2779	2370	2096	2516	2727 .	2317	3029
2481	2887	2639	2618			1917	2434	2656	3016	2793	2487			2432	2564	2296	2739	2535	2224	2667	3171 .	2480	3216
2448	2897	2811	3148			2438	2531	2652	2739	3423	2739			2390	2632	2623	2528	3304	2097	2488	2573 .	2483	3193
2681	2772	2705	2396			2908	2326	2507	2586	3913	2686			2429	2388	2293	2554	2533	2625	2307	2577 .	2410	2999
3025	2703	2908	2463			2271	2298	2500	2546	2837	2507			2207	2264	2291	2626	2684	2250	2809	2745 .	2693	2872
2843	2534	2521	2248			1922	2427	2995	2608	2951	3145			2353	3235	2428	2492	2955	2440	2579	3498 .	2556	2748
2768	2881	2600	2605			2117	2074	2489	2805	2654	2673			2891	3142	2386	2398	2539	1974	3041	2721 .	2546	3227
3050	2625	2612	2427			2104	2534	2546	3012	2875	2432			2409	2405	2038	3099	3187	2078	3028	2885 .	2506	2774
2573	2831	2632	2463			1990	2942	3353	2719	2838	2402			2623	3055	2187	2341	2689	2194	2446	3265 .	2489	2696
								2410										2441	2625	2477	2918		
								2774										2877	2113	2354	2746		

2	2	5	6	6	8	9	9	2	2	5	8	6	6	9	9	11	11	12	11	3	10
Box-4	Box-7	Box-3	Box-1	Box-4	Box-7	Box-3	Box-8	Box-1	Box-5	Box-2	Box-6	Box-5	Box-7	Box-4	Box-5	Box-3	Box-7	Box-8	Box-8	Box-8	Box-8
19.1	25.1	34.2	38	43	52.2	64	75	13.7	20.2	32.3	44.5	45	47	65	68	78	84	Z	Z	Z	Z
M	M	M	M	M	M	M	M	M	M	M	M	M	M	M	M	M	M	Z	Z	Z	Z
Malto	Malto	MD	MD	MD	MD	MD	Malto	MCT	MCT	MCT	MCT	MCT	MCT	MCT	MCT	MCT	MCT	Z	Z	Z	Z
MM	MM	MM	MM	MM	MM	MM	MM	MT	MT	MT	MT	MT	MT	MT	MT	MT	MT	Z	Z	Z	Z
2026	3569	1548	2071	1835	1513	2571	1844	3226	2152	2233	2141	2684	2458	2351	1723	3310	3378	10779	3519	.	.
2209	3616	1677	3705	1946	2253	1987	2035	2998	2631	2995	2121	2140	3172	1512	1951	2816	3108	11525	1924	.	.
3196	3991	3686	3212	3094	3108	1977	2982	1495	3803	2242	3361	1930	3219	1511	2635	2487	2471	12731	2353	.	.
2230	3024	3594	3047	3136	2747	3131	1910	1379	2641	3299	2328	3528	3074	2454	2991	1884	3563	12038	3365	.	.
2236	3323	3590	3221	2282	1453	3214	1916	3003	3064	2026	1987	3050	2552	1701	2107	2120	3032	10817	2226	.	.
3556	3705	3648	3285	2699	1505	3146	2222	3186	1889	2078	2527	3478	3010	3405	1853	3372	2418	11194	2246	.	.
3423	2664	3668	2507	3325	3196	3143	1891	2975	3475	3596	3539	2182	3684	3313	2518	2272	2807	12814	2371	.	.
2023	3344	3761	3310	3416	3060	3072	3258	3555	3697	2567	3626	3340	4229	2807	2932	1883	1867	12749	1209	.	.
1963	3071	3675	3505	2337	3889	2480	2458	3328	4543	2657	2082	2488	3624	2302	3011	3283	2860	13197	3108	.	.
3543	2417	3832	3347	2074	3399	2523	2321	3355	4015	3478	3183	2658	3782	1628	2907	3295	2044	14797	3786	.	.
3706	2185	3671	3825	2655	3297	3520	2400	3571	4108	2614	2169	3486	3265	3276	2976	4094	3384	13083	3455	.	.
3823	2236	3888	3596	2644	3609	3308	3076	3742	4170	2602	2972	2968	3336	3479	3156	4053	3022	13050	2762	.	.
2435	3231	3759	3567	2261	3321	3162	1760	3901	4374	3510	3719	2470	2440	3371	2704	3653	3752	15375	2655	.	.
2642	2406	3719	3841	2994	3449	3341	2453	3464	4451	3445	2511	2102	2729	3583	2819	3685	3489	14193	3626	.	.
1757	2217	3767	3672	2706	3285	2090	3039	3701	4308	3484	2764	1858	2727	3612	1801	3929	3655	13333	4842	.	.
3648	3497	3461	3702	3507	3101	2907	3083	3538	3974	3695	3440	1996	2052	3491	1680	2693	3441	13680	4340	.	.
4170	3653	3373	3686	2508	3367	3528	3533	3439	2474	2148	4069	2379	1916	3696	2979	3921	3611	13466	5636	.	.
4017	2480	2214	3553	3728	3063	3099	3228	3450	2015	2590	3998	2011	2848	3320	2775	3955	3365	14015	3177	.	.
3592	1948	1783	3746	3706	2683	2425	3419	3192	1964	1828	4056	3376	3245	3550	2144	3843	3592	13931	4344	.	.
2660	1985	3042	3420	3516	2707	1456	3229	2183	3223	3167	3941	3043	3246	2973	1488	4195	3531	13420	2806	.	.
1823	2022	3366	2325	3538	2530	1538	2442	2049	2249	3490	4105	1763	2065	2903	2658	3178	3912	13225	3242	.	.
3201	2764	3418	1941	3304	1607	2638	1916	2084	1822	3982	3774	1736	1734	1739	2621	2443	3460	12991	5197	.	.
2771	3230	3218	1922	3175	1334	2889	2492	1604	3285	2914	3390	1851	3001	1423	1987	2702	3394	13803	4475	.	.
2009	3648	2203	3372	3371	1537	1973	2271	1996	3318	1836	2041	1433	2194	2727	2403	2449	2983	13456	4102	.	.
1628	3462	1862	2064	3103	2859	1480	3706	1903	2126	1720	1776	1733	1981	1944	1594	2412	2838	13523	4671	.	.
1631	3477	1861	1900	2495	2088	2229	2680	2050	3697	2175	2406	2639	1954	1730	1656	2268	2625	13915	3015	.	.
3118	2507	1843	3095	2120	1670	1516	1960	2587	3774	1850	2845	2969	2748	1714	1878	3510	2530	13754	1102	.	.
2231	2138	2029	3200	2024	1832	2293	1786	1972	2808	2048	2385	1805	2919	2283	1648	3682	1711	12663	2590	.	.
1846	1865	2827	2162	3070	1686	1757	1781	1705	1963	1883	3589	1590	3411	2836	2469	3895	1912	11626	1164	.	.
1705	2060	2355	1899	2738	1660	2739	3399	3136	1678	1987	3244	1395	2675	1959	1813	4146	1887	12643	2158	.	.
1824	2226	1901	2568	2887	3014	1994	3278	3190	2662	1557	2610	2002	1989	1648	2431	3939	1700	13336	429	.	.
2268	2507	1950	2933	2393	3193	1399	1791	3037	2517	1872	1848	2065	1879	1976	2730	3575	3283	12608	1488	.	.
1857	2041	2254	2037	2104	2672	2540	3253	2223	3263	1989	1665	2733	2588	1832	2544	3646	3450	12559	1444	.	.
2105	2074	3418	3118	2564	1547	3085	3128	1283	2112	2892	3889	3173	3543	3514	2258	3638	2432	12969	2981	.	.
3638	2346	2557	3596	1931	1412	2765	2853	2890	1842	1589	3725	1902	2916	3393	1583	3308	1694	12872	2308	.	.
3667	3344	1883	3076	2291	1314	2387	1961	2940	2050	1549	3306	1401	1934	2616	1554	2922	1806	12624	1444	.	.
2285	3670	2429	1836	3050	3061	1610	1585	2716	4029	1791	2040	1643	1826	1638	2512	2779	1913	12983	1291	.	.
2690	3051	2045	1543	2728	2299	1524	2201	1401	4266	2915	1843	2554	2193	1630	1720	2030	1909	13447	1488	.	.
3973	1852	3137	1732	1779	1485	1553	3540	1418	4194	1838	2114	2960	2731	1680	1579	2010	2008	11568	1291	.	.
3509	1893	3288	2103	1733	1397	1632	3355	1492	4013	1607	1856	2338	2783	1690	1919	2128	3250	10200	2590	.	.
2746	2700	2403	3791	1800	1701	1972	2522	2963	3604	2509	2364	1500	1897	2585	2127	2379	2687	11927	665	.	.
2833	3278	1869	3164	1949	2894	2712	1874	2297	2782	2082	3140	1550	2015	3449	2526	3404	1702	9966	1944	.	.
2010	2256	1897	1778	2652	2472	2493	1908	1451	1927	1528	2598	1945	2398	3185	1842	2331	1716	10135	-5917	.	.
2550	2075	1815	1807	2462	1576	1603	1903	1553	1871	1767	1937	1744	2646	2943	1851	2132	1583	11233	86	.	.
2965	1892	2038	1843	1627	1547	1465	1941	1766	1871	1807	2010	1742	1711	1768	1696	2599	1709	10576	2661	.	.
2101	2082	1983	2243	1892	1798	1713	3346	3056	2550	2420	3119	2394	1845	1739	2779	2110	1728	9998	3585	.	.
1897	2113	1934	3169	2256	2990	1491	2383	2636	3004	1939	2431	2789	3128	1948	2436	2321	1701	11084	4384	.	.
1761	2131	2938	2281	1934	1952	2648	1544	1602	1969	1795	1927	1914	2882	1561	1804	3081	2922	12057	3308	.	.
1916	2493	3373	1822	2090	1652	2394	1768	1901	1985	2232	1997	1551	1884	2624	1697	2363	2643	10855	2867	.	.
3491	1919	1946	1884	2618	1731	1635	3019	2024	2136	1862	1796	1733	1746	2900	1636	2086	1685	10141	2806	.	.
3422	1773	2019	1985	1739	2605	1684	3105	1590	3188	1903	2042	1738	3560	2748	1820	2064	1625	10635	1488	.	.
2262	3353	1904	2754	1861	2627	1721	1684	1583	3347	2008	2116	2170	3074	1759	1793	3274	1830	9882	3740	.	.
1868	3345	2722	2103	1934	2378	1359	1458	1903	2377	1940	1712	2164	2017	1967	2116	2356	1679	10850	4293	.	.
1870	2316	2960	1685	1924	1696	1644	1661	1810	1991	1911	1914	1637	1960	2316	2400	2062	3019	10758	3156	.	.
2026	2219	2042	1891	2412	1575	2619	1765	1767	1964	3240	2481	1969	1888	2611	1715	2208	1857	10330	2373	.	.
1869	2109	1887	1851	1883	1985	1853	3174	1824	2381	2987	3036	2377	3304	1935	1645	2618	1861	10408	4113	.	.
3356	2473	2910	2900	1472	2780	1605	2516	1727	3315	1811	1637	1776	2616	1976	1806	2354	1778	10355	3087	.	.
2482	2125	2380	2345	1741	2681	1675	1680	2178	2558	1887	1614	1815	1829	1565	1773	2267	1850	10934	3520	.	.
2085	2093	1947	1777	2327	1655	1462	1739	2964	2117	2208	1855	1535	2030	1753	2565	2884	1840	11200	3304	.	.
1897	1829	1906	1902	1806	1578	1885	1821	2889	1910	2052	1977	2772	3105	2694	2395	2261	2775	9907	3740	.	.
3135	3001	1801	2764	1522	1979	2766	2229	1993	1986	1955	2920	1796	2840	1789	1644	2255	2892	10869	3650	.	.
2466	3529	2005	1844	2325	1897	1826	2721	1723	2109	1925	1965	1612	2282	1871	1936	2306	2096	12421	2009	.	.
2530	2107	3015	1815	1499	2658	1476	1638	1733	3132	2703	1760	1350	2932	2990	1803	2236	1710	11578	4529	.	.
3328	2136	2496	2328	2102	2281	1669	2141	2606	3151	1911	1879	3309	2178	2342	2220	3412	1709	9611	3156	.	.
2312	2318	1946	2600	2340	1829	2700	2774	2419	2129	2503	2882	2385	3051	1591	1706	3236	1841	10492	2806	.	.
2236	3297	2303	2342	1620	1763	1838	2060	1687	2269	1983	2318	1952	3258	1739	1836	2109	2636	10265	4541	.	.
2816	2754	2673	2101	2138	1780	1711	1840	1706	2296	3586	1895	2507	2146	1701	2365	2095	1843	10725	2376	.	.
3942	2305	3429	3418	2558	1947	2137	1662	2470	3437	3098	3240	1982	3016	2163	2820	2091	2035	11951	3169	.	.
4054	2872	3521	3060	2858	2825	3464	3273	1949	2392	2379	3588	3224	2700	3021	2272	2043	2220	11551	1444	.	.
2970	3376	3241	2484	2872	3045	3344	3231	2429	3967	2687	3755	2812	3836	3259	2504	2495	2425	13909	1813	.	.
3884	3508	3518	3520	2368	2614	3445	2124	2945	3776	2112	3664	3059	3683	3192	2822	2634	3338	13235	3169	.	.
4041	3987	3677	2396	2529	1942	3166	1902	2888	3217	3320	3268	3827	3052	2076	2711	2509	3159	13849	2810	.	.
4172	3421	2952	2283	3160	2683	3054	3353	3397	3989	2150	3021	2876	3514	3135	1836	3688	3354	13866	2400	.	.
4006	3560	3682	2997	2765	3200	2988	3289	3373	4090	2645	2320	2148	3377	2280	1885	3698	3315	8467	3038	.	.
3649	3847	3489	2970	3081	2798	2831	3652	2973	3932	2105	3149	3568	3044	2364	2779	3764	3266	9294	2830	.	.
3920	3471	3655	3035	2275	1904	1525	2188	3038	3632	3229	2085	2850	2408	2405	2725	3885	3475	9627	2335	.	.
3454	3079	3209	3448	2967	2241	2473	3190	3408	3300	2811	2868	2645	2537	2768	2667	4107	2941	9986	2613	.	.
2424	2550	3413	2507	2665	2763	2534	2195	3365	2653	4094	4054	2345	2576	2131	2320	4086	3154	10446	2892	.	.
2012	2137	3332	3144	3054	2980	1974	3122	3951	3615	3831	3835	3612	3423	2573	2330	3973	2056	13531	1511	.	.
1909	2466	3360	3632	3210	2435	2765	3311	3390	2930	3468	3892	2991	3834	3277	2470	4100	1653	13697	3907	.	.
2343	2140	3386	2706	3417	2838	2940	3263	3183	2503	3223	3988	2005	3071	3343	1524	3845	2243	13697	3344	.	.
3805	2259	3385	3243	3084	3027	2707	3379	2990	2287	3343	2876	2661	2093	3545	1471	4000	3067	13615	2607	.	.
3589	2801	3422	2308	2900	3212	2933	2291	2371	3550	3280	2230	3937	3221	3535	2111	4110	3090	13781	4555	.	.
2404	3107	3034	3248	3454	2846	2736	3009	2049	3061	3825	3046	3378	3191	3315	2451	4111	1993	12911	4319	.	.
1971	3368	2240	3850	2876	2074	2546	2804	2764	4075	3473	2248	2817	2164	3228	2767	3890	1495	13076	4279	.	.
2836	2500	3434	3472	2474	1497	2559	2512	3292	3046	2359	3111	1637	1913	3295	2199	4150	1733	13668	4836	.	.
3820	2259	2368	3532	2403	2347	1460	1964	3377	2488	3629	3284	1558	2367	2705	1816	3679	2191	14743	5721	.	.
2813	2054	1990	2066	2891	2098	1369	2716	2255	3598	4055	3476	1701	2309	1641	2307	2312	3352	13868	5729	.	.
2111	1965	2462	1860	2223	1956	2569	2571	1788	3050	2794	3445	1818	1969	2285	1806	2070	3074	13935	4561	.	.
1984	2629	2447	2971	2089	2225	2006	2067	2262	2389	2074	2712	2941	3088	2736	2028	1985	3343	14296	2724	.	.
2066	2468	2170	2661	3269	2107	1917	2053	2242	3039	2130	3053	1980	3082	1892	2345	3478	2958	13422	2655	.	.
2047	2156	2383	1977	3089	2009	1590	2912	1904	2450	2153	2405	1731	2712	1989	2512	4070	3153	13274	1728	.	.
2198	2392	3021	2121	1976	2426	2260	2158	1704	3345	2630	2087	1593	1778	1668	1703	4218	1986	13651	2305	.	.
2404	2325	2154	2020	2003	3052	1889	1975	2857	3663	2978	2032	1595	2143	1905	1844	3979	1611	13271	3425	.	.
2227	3156	3182	1601	1963	2187	1590	2735	3494	2807	3402	1882	2646	2891	2185	1891	2745	1616	14063	1011	.	.
2246	3343	2440	1743	2132	1597	2103	3125	2570	2700	3009	2850	2643	2935	2929	1775	2023	1816	13141	1228	.	.
3652	2822	2527	2556	3004	1529	1846	2059	1738	2772	2893	2269	1641	2038	1955	2322	1640	2179	13436	2118	.	.
3489	2242	3200	2133	2782	1744	1435	2229	1718	2592	2132	3279	1408	1578	2439	1730	1829	1769	13352	1336	.	.
2415	2123	2488	1692	1943	2262	1821	3124	1679	3008	2050	2480	1361	2851	1765	1582	2103	1780	12907	1250	.	.
2012	2733	3225	1972	1280	2996	3030	2213	2775	2388	3608	2097	2149	2678	1897	1718	1968	3319	13680	2614	.	.
2094	3457	1880	3052	2046	2811	2122	1933	3444	3848	3533	1891	1556	1778	3164	1745	3462	3117	13300	3308	.	.
3105	3454	1777	2715	1796	2489	1423	1867	2345	3825	2115	3014	1687	1575	2406	2163	3739	2177	11896	1663	.	.
3945	2868	2153	1741	1579	1556	1469	3507	1391	2440	1931	3178	1651	2267	1540	2882	2670	1517	10511	1229	.	.
3355	1814	2532	1902	1304	1473	2856	3046	1417	1778	1907	2735	3080	3197	1966	2549	1744	1625	11812	299	.	.
2855	2021	2038	3170	1530	1584	1906	1889	1853	3105	1875	1819	3066	2351	1768	1467	1679	1693	11284	151	.	.
1808	1879	2057	3024	3197	1914	1440	2673	2433	3217	2092	2155	1796	1553	1593	1459	2066	2306	10017	645	.	.
1620	1937	1869	1651	2201	1762	1621	2503	2376	2852	2101	1991	1361	1479	2234	1614	2161	1815	6890	2069	.	.
3430	3070	2230	1608	1435	2751	2033	1991	1679	2076	2965	2512	1400	1996	3249	1611	2116	1876	8625	-1403	.	.
3556	1684	1929	1835	1357	2577	1777	1882	1765	1866	2752	2209	1729	3246	2801	2270	3410	2456	7434	840	.	.
2291	1922	1879	3072	1729	1925	2670	2532	3328	2463	1839	1939	1735	1993	1792	2110	2467	1839	9137	-1360	.	.
1799	2329	2654	3185	2684	2055	2727	1706	3057	1919	1828	2201	1807	1669	1359	1579	1919	1718	7751	-674	.	.
1862	3134	3186	1897	1598	1786	1937	3281	1846	2236	3333	3054	1365	2719	2420	1588	1862	1688	7995	1229	.	.
2000	2280	2063	1771	1539	1853	1358	2853	1915	1917	2570	2490	1559	2691	2644	1513	1912	1786	9991	1941	.	.
2032	1882	2044	1818	1448	2044	1490	1893	1833	2064	1841	2111	2659	1803	1722	1995	1952	3223	11116	5557	.	.
1917	2247	1990			2329	1792	1991	2842	3128	1939	2088			1536	1558	3184	2820	9485	4279	.	.
3639	2001	2340			1547	2714	2694	2825	2477	2464	2327			1574	2274	2601	2240	8359	4692	.	.
2655	2120	2833			1626	2651	1871	1694	1836	1967	2879			2623	1977	2063	1740	9753	3972	.	.
2032	2138				2274	1612	1519	1623	2088	1980				1931	1587	1999	1920	9765	4692	.	.
1882	2442				1887	1456	1806	1882	3355	1886				1495	1618	1957	1697	9623	4837	.	.
1907	2215				1816	1660	2754	1722	3089	1675				1587	1776	1731	2231	10972	4038	.	.
2124	2171				3018	1641	2883	3225	1946	2242				1540	1763	1795	1784	10063	4324	.	.
2085	2119				2897	2236	1974	3239	2144	2016				2707	2347	1936	1904	9635	3866	.	.

3256	3513				1922	1850	1973	2052	2070	2020				1909	2236	3123	2690	10408	4843 .	.
2736	2770				2318	1679	1951	1892	2550	2273				1806	1613	2371	2426	9927	3761 .	.
2072	2147				2088	1686	1714	2341	2935	3615				1802	1955	2079	1621	9731	3907 .	.
2696	2109				1854	2302	3298	2927	2386	3570				1493	1797	1833	1669	10427	3304 .	.
2812	2631				2346	1635	2307	2265	2090	2445				2842	2169	1864	1683	10920	3108 .	.
3488	2046				2775	1872	1747	1675	2350	2686				3023	2509	2804	1899	9183	2175 .	.
2680	2135				1987	2832	2544	1757	2171	1845				2855	1738	3190	1892	11038	1812 .	.
2200	2592				1612	2044	3057	1906	2815	1949				2659	1743	2099	3064	11746	2095 .	.
3111	3202				2359	1658	2050	1979	3547	2436				1787	1855	1915	2853	11233	2308 .	.
2685	3728				2139	2296	2501	2981	2529	2928				1716	2172	2287	1995	11662	796 .	.
2625	2397				1934	3100	2467	2434	2192	2252				2023	1936	2079	1783	12340	1381 .	.
3270	2267				2916	2711	2125	3136	2940	3288				2699	2705	1909	2110	13960	2175 .	.
4069	3557				3039	2621	2316	2529	3554	2898				3297	1934	2731	2067	12608	1965 .	.
3911	3179				3282	3331	3265	3257	3163	3316				3288	2879	3236	3392	13634	535 .	.
2512	2977				2234	2171	3413	2991	3926	3963				3420	1927	3482	3018	14474	3927 .	.
3501	3187				2953	3265	2378	2952	3934	2834				2631	2582	3839	2000	14063	4420 .	.
2654	4001				2886	3004	2852	2586	3338	3560				2299	2141	4081	3113	13950	3993 .	.
3403	3083				1848	2467	3267	3306	3183	3478				3177	2027	4221	3276	13455	4410 .	.
3248	3114				2347	2812	3008	2681	3536	3037				2414	1852	3887	2379	14342	4692 .	.
3118	2471				2889	2801	2524	3280	4142	4043				1721	2309	3541	2684	13488	4561 .	.
3996	3556				2143	2448	1933	3271	3664	2948				2919	2448	3449	2906	13023	6076 .	.
3383	3692				1807	1606	3252	3030	3196	3601				2313	1837	3784	2523	13605	3696 .	.
2599	2752				1793	2431	2933	3702	3585	4414				2570	2007	4105	1818	14017	4863 .	.
2402	2492				2636	2238	3196	3549	3786	2762				3276	2088	4150	2453	15024	1164 .	.
2847	2442				2899	1846	2435	3342	3026	3438				3817	2979	4413	3022	15107	3368 .	.
4013	2764				3314	2480	2400	3731	2742	3677				3477	3324	4207	3036	13702	3238 .	.
3602	2973				2876	2547	2550	3751	2545	3681				2504	2639	3803	3409	14809	4339 .	.
2837	2644				1891	2842	2926	3106	3608	2841				2051	1514	4248	3107	13377	4199 .	.
2362	3473				1572	2988	3680	2029	3975	3407				1824	1867	4033	2491	12667	1426 .	.
3122	3753				1940	2680	3795	1604	2821	2376				2324	2043	2830	1777	13098	4384 .	.
2355	2927				1666	1705	3029	1778	2683	1908				2637	2986	3855	2014	14613	4248 .	.
3376	2332				1585	2937	3401	3282	3683	1947				3587	2581	3143	1830	13520	2522 .	.
3432	3132				2266	-20	-21	3756	2856	2529				-22	-16	2660	2877	13735	4118 .	.
2510	2302				1793	11	37	2627	2459	3118				19	18	2107	2393	13588	2676 .	.
2188	2908				2600	1213	1637	2070	3026	2951				2969	2385	2004	1983	12731	4274 .	.
2001	2679				2288	1567	1904	2077	2914	2123				3232	1690	2089	1936	13455	2965 .	.
1949	3380				1758	2068	1760	2985	2874	1928				2454	1888	2076	2562	13059	3173 .	.
2074	2921				1648	1600	2611	2308	2778	1997				2468	1716	3489	1846	12975	4188 .	.
2002	2662				2609	1658	2969	2042	3079	1954				2417	2237	3222	1948	12643	3177 .	.
2438	2635				2081	1663	1851	2382	2490	3039				3245	1888	2262	3254	13138	4745 .	.
2231	2490				1539	2169	1839	1916	2402	3277				2544	1853	1992	2992	12409	4475 .	.
2227	2339				1400	2885	2029	1652	2882	2362				2190	2211	2087	2917	13154	3817 .	.
3605	2930				2898	1977	2842	3482	2797	1744				1923	1741	2276	2036	13743	4324 .	.
3543	3206				3059	1536	2220	3011	2104	2361				2331	2105	3815	1757	13781	4751 .	.
2967	2980				2936	1232	2951	1902	2721	1990				1933	2651	3683	1930	13482	3847 .	.
2033	1854				2496	1535	3128	1355	2395	3027				2887	2757	2413	1820	10316	4501 .	.
2882	1799				2074	1860	2098	2048	2246	2878				1972	1890	1905	1980	12126	4340 .	.
3062	2186				1519	3297	1477	1766	2078	1892				1645	1477	2180	2347	11199	4258 .	.
2435	1940				1441	1966	1562	2153	1903	1741				1835	1572	3477	2010	11942	-1250 .	.
1821	2151				1644	1275	2407	1672	3304	2392				1755	1747	3597	2020	10290	494 .	.
1853	1971				2460	1552	3140	1875	2193	2380				3192	2280	2426	1813	4421	1209 .	.
2041	1968				2540	1621	1900	1914	2870	1784				2247	1457	2067	1689	5233	1792 .	.
2631	2398				1962	2473	1610	1784	2897	2465				1591	1578	1795	2549	7320	1423 .	.
3427	2059				1944	1462	1549	2249	2205	2992				1549	1647	2467	2865	8635	2741 .	.
2238	2137				1979	1653	3041	2414	2138	1879				1909	1930	2025	2083	8945	4987 .	.
1798	2115				1796	1608	2631	1750	2738	1983				1980	1663	2239	1819	9053	4837 .	.
1843	1941				1933	1669	1508	1609	2568	2876				2878	1951	3234	1840	7976	4384 .	.
3044	2541				1972	2953	1812	1695	2236	2582				2268	2288	3006	2246	9757	4541 .	.

3166	3458	3571	3366	3025	3060	3330	3464	2247	3182	2032	3716	2685	3041	2888	3147	3358	2782	5921 .	.	.
2646	2613	2921	2661	1957	2135	3090	3393	3204	3392	2141	3501	2248	3149	2683	3090	3290	3388	9784 .	.	.
2988	3478	3592	3018	2572	2854	3214	3402	3054	2667	3729	3532	2835	2909	2777	2775	2310	2413	9263 .	.	.
2479	2912	2488	2384	1934	2048	2484	2804	2116	3661	3036	2583	2157	3255	2165	2308	3315	3421	10492 .	.	.
2496	3649	2864	3619	2949	2178	2969	2120	2861	3632	2175	2661	1950	2652	2803	2571	2410	2829	10888 .	.	.
3398	2867	3716	2268	3159	2570	2902	3348	3331	2619	3493	2468	2789	3131	2802	2967	2289	2417	11024 .	.	.
3940	3156	2636	2092	2067	1928	3038	2291	2174	2561	2599	3382	2762	2650	2045	2365	3444	2292	11523 .	.	.
2946	3540	2269	2545	2191	2154	2742	2204	2272	2586	2251	2382	2151	2164	2662	2343	3140	3058	11836 .	.	.
2850	2702	2565	2448	2784	2754	2106	3061	2158	3051	3995	2221	2465	3052	2771	2903	2343	3436	12991 .	.	.
3204	2669	2884	2579	1975	2501	2591	2288	2249	2737	2862	3144	2493	2295	1916	2037	2937	2293	13420 .	.	.
3847	3260	2287	2248	1996	2468	2180	2264	2452	2557	3019	2691	2681	2287	1804	2541	3679	2301	13439 .	.	.
3004	2858	2296	2456	2627	2571	2910	2547	2048	3041	3012	2591	3120	2388	2164	2877	2577	2683	14529 .	.	.
2997	2645	3501	2905	3231	2793	2585	2437	2878	2651	3001	3222	2734	3954	2113	3020	2565	3899	13553 .	.	.
4167	4010	3433	3024	3380	3109	2470	2727	2530	3163	3873	3738	3203	4133	2584	2715	2483	3012	14166 .	.	.
4215	3503	3595	2917	2689	2320	2490	3248	3042	3729	3517	2748	3303	2975	2273	2543	3297	3450	15946 .	.	.
3459	3427	3069	3299	2729	2511	2994	3619	2690	3786	3646	3452	3299	3791	2951	2942	3629	3044	14138 .	.	.
2893	3555	3759	3535	3180	3232	3186	3101	3375	3551	3496	3029	3807	3155	2815	2715	3304	3258	14978 .	.	.
3368	4041	2903	3620	2781	2993	3121	2880	3660	3748	3580	3434	2840	2886	3130	2705	3009	3434	14268 .	.	.
4111	3991	3468	2693	2734	2323	3188	2870	3966	3149	3697	3056	2661	3140	3508	3021	3047	3013	15556 .	.	.
4334	3189	3058	3435	3111	2938	3539	3058	3413	3763	3169	3029	2595	3419	3133	2834	3158	2754	13889 .	.	.
3156	3086	3567	3485	2886	2898	2576	2684	2611	4008	2990	3139	3071	3746	2966	2545	3289	2813	14055 .	.	.
3228	3901	2991	3017	2729	2277	2589	3262	3211	3354	3825	3216	2918	2474	3210	2849	3511	3148	17420 .	.	.
2760	3247	2817	2518	3119	2822	2761	3447	2645	3501	4252	3695	2985	2698	2553	3086	4019	2631	14634 .	.	.
2735	3788	3952	3383	2901	2347	3483	3464	2624	3550	3752	3756	2371	3113	2582	2814	3900	3394	14204 .	.	.
3132	3224	3560	2830	2755	2624	3251	3636	3044	3177	3955	3511	2547	2376	2549	2759	3726	3134	12211 .	.	.
3557	3570	3071	3015	3122	3094	2483	2874	2550	3481	3274	3424	2659	3272	2966	3166	3588	2958	14075 .	.	.
2946	3322	3251	2865	3339	2558	3360	3503	2545	3358	3019	3172	3083	3283	2727	2443	3303	2783	14092 .	.	.
2972	3452	3255	3625	3490	2213	2545	3439	2472	3720	3709	3383	3579	2845	3077	2432	3314	3374	13166 .	.	.
3505	4028	3325	3619	3268	3253	2762	3270	3253	3507	3822	3042	3065	2456	3312	3120	3680	3829	12770 .	.	.
3965	3192	3298	2940	3073	3173	2909	2820	3363	4147	2957	2976	2574	2523	3050	3098	3527	2676	14010 .	.	.
3505	3401	3076	2400	2479	2685	2793	2582	2891	4275	3053	3183	2885	2620	3079	2683	3088	2808	14008 .	.	.
2601	3547	2886	2482	2390	2142	2689	2884	2359	4174	3239	2959	2486	3055	2761	2902	3196	3363	13481 .	.	.
2372	3024	3329	2335	2473	2405	2931	2732	2025	3651	2773	3232	2564	3264	2546	2666	4179	3874	13365 .	.	.
2586	2881	3080	2506	2568	2522	2379	3075	2415	3330	3629	3190	2313	2433	2671	3125	3700	3573	13199 .	.	.
4043	3767	3101	3012	2722	2802	2722	3074	2313	3316	3380	2836	2377	2643	2841	2800	3456	2859	12227 .	.	.
2791	3533	3291	2536	2719	2592	3140	2620	2532	3452	2558	3474	3272	2294	3081	2535	3347	2656	12721 .	.	.
2511	2903	3462	2571	2752	2598	2843	2574	2705	3366	2766	3022	3531	2638	3271	2384	3367	2637	14830 .	.	.
2710	3136	3247	3031	3318	2646	3076	2643	2113	3449	3461	2643	2546	2398	3050	3011	3124	3109	13234 .	.	.
3220	3137	2705	3535	2748	2530	2601	3506	2301	3641	2689	2970	2458	3149	2600	2229	3109	2451	13562 .	.	.
2933	3151	2969	2380	2285	2411	3023	2814	2315	3500	2695	2708	2506	2589	2792	2495	2928	2884	12472 .	.	.
2875	2932	3359	2215	2437	2194	2425	3180	3458	3125	2593	3165	2655	3045	2826	2949	3286	3340	13709 .	.	.
2889	3030	2802	2273	2360	2260	2984	2724	2869	2832	2727	3190	2940	2894	2778	2824	3043	3269	12043 .	.	.
2675	2899	3542	2155	2555	2472	2514	2418	2153	3073	2530	3454	3038	2484	3007	2494	2702	2431	13065 .	.	.
2296	3289	2948	2060	2802	2984	2855	3223	2454	2937	2774	2997	2163	2038	2789	2501	3261	2741	13423 .	.	.
2947	2997	3380	1822	2997	2588	2159	2497	3403	3287	2261	2477	2284	1884	2449	2154	3373	2892	12168 .	.	.
4067	2158	3176	1966	2387	1917	1890	2775	2270	2507	3147	3113	2220	2102	2591	2380	3079	2369	12283 .	.	.
2512	2282	2759	2022	1777	1987	1871	3518	1918	2699	2529	3020	2013	1696	2332	2172	3360	2439	12578 .	.	.
2557	2400	2715	3161	1890	1896	2081	2506	3292	2724	2792	2113	2309	3109	2826	2157	2838	2351	11127 .	.	.
2770	2808	2875	2562	2612	2292	1930	2712	2421	2432	2114	2047	2816	2852	2661	2321	2511	2059	12214 .	.	.
2408	2709	2643	1949	2167	2165	2327	3139	2052	2305	2143	2693	2540	2271	2075	2872	2885	2392	12478 .	.	.
2429	2399	2171	2338	2020	2101	1868	2380	3009	3494	2315	2474	2307	2002	2314	1823	2437	3060	11850 .	.	.
2515	2756	2735	2062	2077	1943	2061	1999	2434	2700	2620	2306	2127	2152	2231	1937	2480	2360	11291 .	.	.
3139	2279	2306	2986	2289	1880	1856	2025	2251	2185	2530	1951	1964	2898	2153	2030	2880	2335	7144 .	.	.
3241	2484	2236	2261	2048	2827	2634	2671	2796	2791	2338	2797	2556	1960	2609	2424	2695	3058	7547 .	.	.
2350	3555	2206	2975	2448	2108	2310	2350	2504	2513	2256	2664	1923	2184	2071	2040	2910	3015	7416 .	.	.
2575	2636	2726	2722	2254	2054	1975	2309	2449	2560	2229	2348	2261	2172	2367	2562	2472	2254	8981 .	.	.
2177	2581	2674	2098	2503	2231	1916	3347	2175	3532	3449	2169	2317	2213	2289	1839	2426	2400	10212 .	.	.
2456	2354	2613	2129	2208	2096	1990	2488	3079	2354	2978	2467	2190	1952	2056	1992	2341	2442	11877 .	.	.
2357	2318	2741	2102	1906	1961	1920	2451	2489	2374	2071	2293	2151	3555	2578	2709	2802	3339	11187 .	.	.
2478	2781	3159	2093	1966	2471	1967	2579	2322	3321	2231	2433	2108	2431	2216	1987	2892	3250	10874 .	.	.
2228	2553	2604	2110	2313	2363	2644	3335	2285	2737	2130	2292	2556	1998	2123	2154	2945	2311	11951 .	.	.
3010	2282	2347	1951	2199	1919	1888	2274	2785	2349	1984	2299	2820	2617	2419	2031	2652	2236	13485 .	.	.
2958	2354	2365	2620	2552	1916	1949	2505	2134	2256	2044	2043	2152	2543	2215	2180	2126	2338	11518 .	.	.
2090	2309	2382	2204	2098	1719	2291	2963	2479	2984	2269	3103	2238	2104	1892	2143	2207	2914	12539 .	.	.
2082	3227	2823	2017	2409	2017	2862	2274	2117	2424	2181	2343	2437	3018	1830	2121	2344	2249	13664 .	.	.
2815	2723	2218	1994	1980	2464	2479	2426	2230	2483	2188	2795	2060	3082	2211	1845	2987	2132	11635 .	.	.
2853	2330	2307	3086	2209	1967	1866	2464	2599	3157	2103	2254	1924	2210	2165	2499	2488	3329	12115 .	.	.
2943	2767	2947	2395	1754	2077	1783	2291	2440	3556	3206	3068	2034	2029	2819	1887	2324	2812	12493 .	.	.
3065	2377	2275	1960	2648	2180	1928	2892	2233	2562	3083	2430	2871	2085	2609	2304	3491	2212	12563 .	.	.
2296	2401	3323	2780	2683	2809	1782	3346	2240	2398	2190	2041	3363	3147	1935	1931	2340	2257	11291 .	.	.

3370	3193	2360	2128	1943	2284	1714	2095	2004	2891	1966	2530	2914	2325	1969	2911	2157	2067	12742 .	.	.
3544	2556	2379	3628	2310	1907	2721	2242	2956	2628	1981	2219	2892	3347	2564	2721	3173	2758	13290 .	.	.
2482	2378	3134	2792	2485	2264	2731	2353	2274	2635	2176	2047	2652	3710	1929	2897	3299	2492	12149 .	.	.
3630	2909	3720	3508	3336	2158	1868	3043	2192	2530	2099	2876	2590	3783	2285	2385	1941	2220	13735 .	.	.
3553	2376	2724	2942	2260	2692	2850	3556	3080	2702	2010	2459	2386	3269	1853	2194	3162	2607	12994 .	.	.
2663	3200	2558	2204	2243	2473	2226	2632	2416	3051	3416	1993	2618	2694	3036	2017	3604	2666	12945 .	.	.
2736	2784	2305	2985	2646	2463	1731	2529	2019	2715	2881	3249	2436	2624	3216	2417	2556	3195	12912 .	.	.
3753	2813	2779	2440	2851	2683	1893	2247	2634	3625	2425	2448	3407	3903	2420	2191	2299	2619	14268 .	.	.
3946	4066	3468	3431	3201	3213	2851	2690	2639	3621	3098	3225	2869	3113	2318	2837	2240	3054	14764 .	.	.
4475	4062	3563	3603	3093	2989	2210	2921	2878	4380	3730	3374	3318	2834	2764	2115	3456	3625	13942 .	.	.
4023	4005	3585	3931	2812	2517	2944	3500	3660	4299	4049	3455	2706	3572	3299	2976	3816	2559	16238 .	.	.
3986	3160	3292	3209	2796	2925	3677	2923	3593	4149	3857	3489	2466	3430	2949	3092	3310	3971	14094 .	.	.
3720	2687	3298	3483	3575	2185	4007	3579	2913	3550	3624	3193	3465	3326	2739	2577	3292	3002	17263 .	.	.
3856	3972	3930	3475	2989	2646	3785	3457	2274	3253	3840	3019	3347	3597	3086	2664	3054	3445	16256 .	.	.
3918	3807	3260	2808	2552	2727	3181	3292	3228	3942	3822	3350	3267	3224	3194	2443	4030	3692	16879 .	.	.
2905	3156	3327	2440	2792	3004	3507	3055	2448	3608	3334	3004	2553	2760	3400	2848	3239	2615	16322 .	.	.
3151	3134	3307	3641	3060	2718	3059	3435	3051	3507	3592	3610	2696	2819	3086	3521	2685	2758	16836 .	.	.
3840	3672	2831	2807	2765	2493	2898	3274	2658	3627	3467	3744	2453	2691	2759	2984	2703	2822	15893 .	.	.
3400	3254	3363	2724	2599	2291	2950	2517	2316	3608	2624	3203	2480	3017	2521	3145	3880	2551	16815 .	.	.
3516	3277	3521	2295	3472	2417	2368	2469	2262	3398	2587	2895	2868	3045	2325	3085	4022	2821	15399 .	.	.
2618	3146	3091	3470	3271	2235	2741	3550	2641	3946	2836	3207	3313	3078	2977	3133	3441	4066	16562 .	.	.
3767	3912	2747	3596	3342	2266	2857	3541	2447	3126	2909	2641	3047	2721	2563	2298	2703	3947	15529 .	.	.
3391	3768	3531	3196	3194	2989	2725	2840	2438	3991	3737	3270	2426	2510	2391	3052	3186	3383	14998 .	.	.
2988	2811	3256	2187	2678	2704	2645	2786	2174	3813	4076	2662	2490	2695	2235	2810	4221	3150	15770 .	.	.
3417	2933	2967	2979	2646	2455	3192	2505	3322	2754	3978	2836	2537	2755	2620	2915	3647	3642	14587 .	.	.
3578	3470	3431	2529	2233	2202	3468	2475	3331	2900	3923	3085	2243	2653	3267	2212	2978	3807	13630 .	.	.
3627	3250	2777	2263	2863	2335	3169	3368	2566 .		3738	2701	2533	3074	3591	2469	3493	2973	12635 .	.	.
3051	3883	3057	2803	2751	2502	1876	2882	1966 .		3124	3551	2463	3351	3511	2772	3227	3052	12402 .	.	.
2982	3317	2923	2392	2477	2796	2681	3115	2158 .		2656	3400	2226	2974	3323	2408	2853	3314	13458 .	.	.
3099	3615	2898	2878	2432	2677	2860	3371	2894 .		2964	2740	2297	2682	3082	2229	2844	3499	11544 .	.	.
3537	2820	2674	2598	2541	2310	2482	2466	1894 .		2697	2699	3244	1990	2382	2391	2990	2762	12881 .	.	.
2746	2975	3244	3241	2872	2396	2591	2523	3155 .		2630	3017	2913	2830	2394	3038	3347	3156	13637 .	.	.
2882	3027	2645	2374	2875	2300	2307	2249	2243 .		2829	2471	2286	2145	3008	3097	3145	2737	15005 .	.	.
2969	3336	3002	2227	2205	1932	3289	3287	2153 .		3511	2539	1903	2246	2326	2417	3163	2833	12024 .	.	.
4040	2907	2688	2064	2499	2367	2346	3403	2516 .		3384	2459	2287	3113	2156	2327	2978	2741	14054 .	.	.
3071	2476	2640	3266	2913	2175	2489	2381	1830 .		2814	2853	2515	3038	2533	2874	2597	3037	16584 .	.	.
3081	3152	3220	2522	2822	2135	3099	2118	2248 .		2818	2315	3027	2117	2757	2433	3032	2609	15173 .	.	.
2988	3772	3173	2460	2445	2057	2255	2229	3090 .		2673	3401	2334	1751	2455	2729	2813	2497	14166 .	.	.
2456	3694	2652	2274	1700	2221	1750	2760	2686 .		2662	2825	2417	3129	2755	2911	3422	2510	15388 .	.	.
2526	2895	2965	3244	1639	1867	1703	2044	1816 .		2672	2478	2071	3103	2637	2758	4014	3030	13095 .	.	.
2434	2265	2197	2416	3048	1888	1911	2478	1813 .		3288	3505	1855	2794	1938	2928	3578	2413	13358 .	.	.
2546	2680	2188	1631	1798	2603	2132	3281	3091 .		2197	2729	2337	1941	2148	2251	2814	2913	13273 .	.	.
3260	2239	2386	1941	1459	1727	2058	3619	2808 .		1815	2199	1985	1716	1836	1905	3329	2789	13011 .	.	.
2515	3068	2779	2839	2690	1714	1679	2371	1628 .		1889	2184	2184	1757	2174	1881	3184	2155	12578 .	.	.
2028	2187	3092	2007	2728	1752	1821	2064	2047 .		2479	3145	3045	1839	1856	2503	3210	2101	13254 .	.	.
2159	2182	2192	1940	1999	1800	3087	2264	2350 .		1969	2475	2687	3114	2785	2664	3215	2190	13189 .	.	.
1992	2771	1947	1977	2166	1828	3074	2409	2068 .		1891	2402	1899	2118 .		2282	2550	3229	12165 .	.	.
3546	2121	2785	2539	2623	1778	1980	2067	2194 .		3577	2275	2154	2084 .		1934	3191	3008	10304 .	.	.
3099	2457	2354	1883	2035	2451	1835	2044	1616 .		2859	1848	2099	2857 .		2079	2445	2258	9692 .	.	.
2474	2072	2328	2030	3117	2551	1843	2066	1962 .		2448	3136	1847	1937 .		2631	3061	2805	10420 .	.	.
2954	2671	2188	2439	3009	1920	1712	3211	1840 .		1926	2221	2038	2921 .		1929	3007	2404	11303 .	.	.
2648	2270	3175	2054	2217	1811	2087	2492	1837 .		2085	2054	2072	2519 .		2219	2341	2269	10030 .	.	.
2214	3039	2502	2033	1999	1720	2834	2029	1872 .		2025	2745	1884	1954 .		1978	2141	2398	12175 .	.	.
2264	2501	2149	2251	2986	2577	1736	2062	1838 .		1990	2201	1768	3154 .		2143	2205	2176	10810 .	.	.
3530	2976	2099	2054	2726	2210	1710	2178	1805 .		1892	2191	2815	3521 .		2287	2098	2180	3480 .	.	.
2518	2553	2347			1988	2073	3171	2367 .		1994	2403		.		2083	3421	2145	6090 .	.	.
3386	2305	2277			1574	1885	2262	1780 .		1849	2225		.		2659	2600	3354	5915 .	.	.
2452	3339	3177			2686	1849	2689	1839 .		2193	2797		.		2500	2188	2490	7284 .	.	.
2363	2776	2320			1917	2940	2353	1796 .		2203	2271		.		2117	2173	2198	7304 .	.	.
2641	2536	1232			1745	2420	2231	1599 .		2883	1241		.		2024	2107	2428	7602 .	.	.
2093	2166				1947	1918	2675	2383 .		2071			.		1825	2290	2263	8919 .	.	.
3815	2114				2819	1935	2043	2064 .		1963			.		2277	2810	2057	8854 .	.	.
3215	2314				2353	2099	2591	1821 .		2041			.		2093	2616	2406	10141 .	.	.
2657	3470				1844	2029	2887	1631 .		1958			.		2256	2124	3039	11466 .	.	.
2858	3930				2257	2410	3127	1645 .		3729			.		2204	2275	2318	10453 .	.	.
3437	3515				1907	2313	2974	1929 .		3376			.		2371	2462	2297	12046 .	.	.
2672	2762				1708	3068	2358	3263 .		2079			.		2742	3514	2650	2836 .	.	.
3217	2933				2760	2373	2563	2401 .		2405			.		2049	2372	3406	6554 .	.	.
2599	3600				2086	2056	2877	2054 .		2810			.		2015	2345	2999	7807 .	.	.
3649	2980				1743	1943	3130	3055 .		2674			.		2938	2234	2472	7184 .	.	.
2887	2565				2212	2021	3128	2048 .		3641			.		2320	2269	2428	8134 .	.	.
2630	2612				1854	2786	2531	2105 .		3479				2308	2326	2712	2595	9470 .	.	.
3240	3644				3201	2128	2290	2885 .		2400				2281	2746	2255	2784	10134 .	.	.
2807	4123				3098	2013	2551	2518 .		3439				2030	2111	3191	3846	11062 .	.	.
3942	3211				2920	3026	3465	3593 .		3813				2026	3031	3504	3829	11889 .	.	.
3547	3306				2852	2192	3454	3608 .		3478				2392	2646	3468	3618	11829 .	.	.
2991	3817				2182	3703	3062	4082 .		4033				2541	2515	2822	2714	13794 .	.	.
3993	3177				2540	3389	3506	3457 .		3058				3688	3129	3550	2996	14569 .	.	.
4118	3853				3037	3104	3231	2901 .		3180				3461	3199	3961	2862	15529 .	.	.
3777	3901				2865	3308	3596	3217 .		3548				2978	3068	3349	3001	16198 .	.	.
3894	3396				2371	3079	2928	3790 .		3810				2345	2395	2844	3506	15511 .	.	.
3251	3524				2101	2569	3128	3560 .		4029				2516	2057	3655	3925	14522 .	.	.
2747	3562				2128	3387	2948	2384 .		3758				2644	3232	3556	3555	16238 .	.	.
3730	3615				2565	2829	3218	2416 .		3076				2866	3044	3532	3104	15705 .	.	.
3078	3057				2132	3495	3911	2886 .		2953				2248	2060	3397	3118	15790 .	.	.
2607	3615				3000	2291	2662	2341 .		3470				2367	2922	3016	2791	15920 .	.	.
3671	3791				3016	3075	2565	2397 .		3866				2352	2804	2796	3304	16821 .	.	.
2926	3786				2621	3618	2947	3487 .		2778				3077	2562	2810	3922	15043 .	.	.
2506	3112				2088	2757	2546	2649 .		2693				2937	2272	2979	4106	14632 .	.	.
2519	3650				2299	2924	2676	1763 .		2851				2832	2642	3733	3441	14959 .	.	.
3865	3798				2124	2682	2991	2232 .		3553				2690	2535	3700	2593	14184 .	.	.
2906	3340				2138	2991	3587	2811 .		3531				3308	2323	2830	3534	13671 .	.	.
2124	3440				2253	2656	3004	3015 .		3417				2787	2223	2473	3770	14743 .	.	.
2458	3165				3088	2424	2080	1995 .		2626				2462	2814	2921	2727	15472 .	.	.
2635	3443				2424	2842	3552	2318 .		2609				2564	3651	3200	2660	15024 .	.	.
3301	3264				2532	3441	2274	2341 .		2609				2012	2976	2670	2804	17161 .	.	.
2551	3082				2123	3302	2958	2175 .		2763				3079	2354	2755	2602	17292 .	.	.
2624	3289				1943	2893	2441	2479 .		3246				2308	2735	3283	3087	15491 .	.	.
3632	3160				2344	2391	3019	2874 .		3457				3090	2233	2787	3060	16134 .	.	.
3675	3219				2015	2584	2755	2602 .		2579				3122	2406	2738	2587	17232 .	.	.
2626	3531				2009	2345	2478	1797 .		2707				2251	2302	3658	2679	15005 .	.	.
2497	3033				2855	2740	2428	2179 .		2412				1983	3217	3567	2930	14399 .	.	.
2438	3442				2052	2374	2088	2425 .		2425				2799	3072	3087	2271	15127 .	.	.
2441	2674				2318	1986	2674	1811 .		2466				2527	2233	2956	3428	15005 .	.	.
2232	3091				2340	3338	3064	2024 .		2208				2719	2314	2023	2279	16996 .	.	.
3661	3051				3090	2674	2222	2877 .		2913				2775	2056	2398	2501	15322 .	.	.
3160	2698				2742	1822	2025	1468 .		1982				1819	2069	3125	1935	15257 .	.	.
2564	2296				2135	1752	3418	1652 .		1996				2523	1985	2241	2116	14314 .	.	.
2130	2820				1508	1647	2174	1926 .		3159				2698	2116	1821	2166	15265 .	.	.
2843	2462				1529	2211	2010	1731 .		3552				1815	1825	1930	2177	12628 .	.	.
3753	2416				2714	1873	3136	1886 .		2934				1794	3111	3198	3747	12923 .	.	.
3254	2600				1996	1999	2145	2006 .		1968				2439	3008	3007	3222	13976 .	.	.
2308	2187				1998	1833	1855	2001 .		1859				2047	2419	2008	2224	12773 .	.	.
2236	2826				1886	2254	1875	1804 .		2852				2101	1639	1875	2061	10731 .	.	.
2321	2344				2225	3097	2115	1996 .		2052				2263	2122	2088	2332	12459 .	.	.
2491	1944				2043	2065	3187	1962 .		1898				1816	2108	2622	2369	12394 .	.	.
2342	2633				2061	1877	2246	2304 .		1862				2286	1796	1830	2275	11542 .	.	.
2228	3000				2288	2026	1822	3028 .		1949				3091	2346	1831	2217	12259 .	.	.
2749	2395				2224	1737	3084	1829 .		2507				2947	2085	1670	2220	10908 .	.	.
2318	2239				1913	2246	2022	1889 .		2059				1867	1822	1978	2974	9551 .	.	.
3623	3602 .		2422	2130	2028	2882	3267	2343	3990	3294 .		1853	2607	3567	2714	2964	2257	14285 .	.	.
3451	3257 .		2426	2402	1895	2787	2928	2266	3740	2678 .		2509	2356	2642	2235	2693	2529	11732 .	.	.
3505	2753 .		2461	2387	2049	2630	3070	2337	3581	3003 .		1841	3119	2811	2211	2575	2403	13959 .	.	.
3337	3128 .		2428	2759	2870	2672	2845	2137	3559	2589 .		2642	2365	2435	1985	3095	2535	15031 .	.	.
2877	2830 .		2384	2166	2420	2357	2416	2916	2767	2729 .		2146	2500	2314	2165	2750	2572	13596 .	.	.
3529	2898 .		2417	2167	2433	2526	2395	2879	3637	2561 .		2748	2983	2365	3110	2832	2360	13877 .	.	.
2763	2872 .		2433	2120	2059	2198	2431	2227	3090	2595 .		2260	2383	2222	1862	3386	2497	15030 .	.	.
2646	3039 .		2254	2499	2149	2264	3118	2606	3076	3052 .		2410	3278	2405	2599	2590	3030	13382 .	.	.

2567	2487	.	2225	2277	2266	2448	2463	2597	2704	2701	.	2015	2465	2432	1738	2713	3427	14406	.	.	.
3214	2902	.	2812	2320	2211	2084	3185	2470	2667	2612	.	2128	2452	2215	1902	2746	3178	14406	.	.	.
2719	2671	.	2866	2143	2181	2080	2500	2778	3009	2579	.	2775	2681	2636	2868	2936	2639	14536	.	.	.
2733	3040	.	2091	2722	2635	2262	2466	2912	3060	3021	.	2495	2660	2420	3181	3128	2581	13151	.	.	.
2819	3525	.	2401	3099	2533	2201	2548	3332	3067	3198	.	2822	3714	2458	2213	3256	2988	15246	.	.	.
3917	3456	.	2402	2864	2398	2591	3118	2928	3047	3321	.	3270	3497	2417	1896	3177	2744	16057	.	.	.
4136	3684	.	2055	3504	2484	2853	3601	3270	3489	3234	.	2927	3495	3099	2189	4267	3017	17322	.	.	.
3329	3657	.	2045	3164	2343	3247	2550	3360	3953	3479	.	3310	3025	2863	2390	3382	2863	16271	.	.	.
2957	3247	.	3471	2705	2591	2853	3409	3209	3517	3728	.	2903	2724	3302	2045	3546	3419	16441	.	.	.
3588	3520	.	2635	2673	2534	2870	2846	2571	3993	3417	.	2727	2689	2969	2353	3987	2615	16375	.	.	.
3898	3238	.	2444	3027	2343	2817	2919	2812	3825	3753	.	2874	2364	3006	2109	3679	3383	15349	.	.	.
3621	3292	.	2540	2781	2830	3021	3213	2835	3813	3709	.	2074	2510	3057	2033	3518	2980	15907	.	.	.
2817	3261	.	3047	3436	2471	3098	3352	3569	3116	3033	.	2458	2737	3108	2521	3941	3118	15115	.	.	.
3862	3468	.	2589	3592	2098	3199	3687	3544	3223	3420	.	2089	2948	3511	2508	3558	2918	14491	.	.	.
3114	3402	.	3250	3618	2607	2713	2752	3450	2913	3208	.	2488	3239	3103	2259	3668	3151	15804	.	.	.
3153	3717	.	3446	2671	2726	2726	2505	3184	3903	3394	.	2104	3858	2935	1808	3371	3620	14502	.	.	.
3124	3653	.	3148	2132	2463	2793	2794	2402	4081	3300	.	3217	2508	2722	1940	3512	3003	15674	.	.	.
3323	3089	.	3625	2601	2398	2960	2615	3199	3451	3681	.	3289	2943	2773	2323	4015	3890	14836	.	.	.
3611	3330	.	3056	2809	2428	3208	2852	2828	3297	3369	.	2794	3011	3493	1905	3520	4103	15972	.	.	.
3735	3528	.	2987	2911	3037	2517	2680	2820	3378	3936	.	1974	2210	2999	1893	3335	3197	16011	.	.	.
3466	3484	.	3227	3090	2885	3462	2540	2613	3939	3219	.	2185	2303	2910	2052	4289	3159	16252	.	.	.
3308	3685	.	2578	3163	3015	2831	2886	2729	3448	2994	.	3070	2921	2942	1904	4278	3073	16571	.	.	.
2740	3927	.	1968	3358	2608	2876	2797	3004	3332	3407	.	2413	2294	2823	1962	3724	2903	17044	.	.	.
2640	3451	.	2633	3044	2098	2568	3666	3630	3559	3925	.	2208	2360	2720	1811	3437	3725	14836	.	.	.
2690	3069	.	2400	2360	2289	2634	3478	2369	3894	3487	.	2281	3113	2951	2094	3196	3366	17365	.	.	.
3542	2883	.	2158	2400	1954	2845	2450	2651	3587	3056	.	3398	2539	3646	2064	3642	3012	15609	.	.	.
2721	3560	.	2099	2470	1811	2492	3152	2670	3616	3445	.	2965	2585	3324	1942	3326	3022	15739	.	.	.
2594	3105	.	2228	2670	1920	3199	2984	2422	2924	3457	.	2219	2483	3153	2520	3845	3018	15097	.	.	.
3031	3112	.	2226	2766	2306	3614	2482	2545	3577	2969	.	2681	3239	3087	2743	3611	3039	15909	.	.	.
2899	3203	.	2691	2570	2103	3076	2775	3073	3027	2803	.	2065	3231	2719	2432	3395	3685	15460	.	.	.
2686	2906	.	2636	2782	1781	2541	2709	2440	3040	3528	.	2041	2438	2984	1704	3215	2919	14799	.	.	.
3553	2500	.	2298	3159	2599	2487	2333	2475	3271	2887	.	2047	2328	2645	1873	3205	2945	14601	.	.	.
3712	3405	.	2566	2922	2659	3059	2518	2277	3048	3113	.	2470	2461	2612	1719	3494	3105	15591	.	.	.
3305	3720	.	2352	2805	1993	2822	2944	2751	2807	2763	.	2102	2745	2502	2005	4061	3435	15675	.	.	.
2736	3411	.	2021	2357	2274	3058	3165	2758	3063	2866	.	2173	2130	3175	2205	3587	3492	15675	.	.	.
3271	2448	.	2651	2639	2424	2677	2350	3035	2661	2824	.	1988	2429	3191	2026	3018	2447	15013	.	.	.
2331	3023	.	2098	3246	2830	2251	2148	2858	3008	2538	.	2531	2002	2726	1874	3259	2242	15720	.	.	.
3272	2419	.	2182	2920	2657	2502	2392	1980	3926	3248	.	1759	2101	2883	1710	3013	2891	15805	.	.	.
2717	2446	.	2042	2083	1763	2942	3522	2207	3286	3493	.	2581	1897	3522	1826	3047	2312	14882	.	.	.
2443	2387	.	2887	1681	1796	1967	2122	2323	2530	2547	.	2131	2075	2948	2796	3285	2584	12885	.	.	.
3051	2662	.	3256	2311	2279	1744	2076	2093	2486	2360	.	2100	2080	2251	2739	3235	2842	12026	.	.	.
2508	2646	.	3408	1994	1801	2024	2116	3270	3405	2630	.	1947	2151	2188	2165	2821	2333	12440	.	.	.
2512	3013	.	2769	2462	1970	2125	3196	2043	3026	3030	.	1909	1999	2493	1570	2811	2685	11219	.	.	.
3547	2531	.	2013	1961	2056	1894	2089	2047	2470	2576	.	2290	2938	2719	1732	2449	2445	11942	.	.	.
3303	2773	.	2041	1949	2138	2001	2354	2242	2514	2397	.	2452	2815	2107	1905	2690	2363	10842	.	.	.
3074	2871	.	2423	2093	2049	2190	2878	2238	2806	2551	.	3405	1975	1936	1991	3400	1967	11116	.	.	.
2539	3269	.	2759	1991	2530	1995	2746	2545	2660	2757	.	2463	1705	2629	1746	2470	2871	12057	.	.	.
3031	2906	.	2018	2357	2840	1967	2273	2799	3744	2497	.	1719	2343	2124	2065	3416	2682	11447	.	.	.
2557	2962	.	1996	1918	2469	2150	2871	2221	2919	2612	.	2081	3087	2552	1813	2560	2509	11484	.	.	.
2409	2584	.	2260	2075	1893	1970	2503	2405	2737	2507	.	1887	2029	2402	1851	2566	2309	13558	.	.	.
2748	3143	.	2721	1955	1953	2157	2060	2190	2651	2374	.	2006	2276	2490	2296	2682	2429	12191	.	.	.
3567	3111	.	2212	2188	2187	2851	2788	2276	2716	3001	.	2494	2258	3103	2495	2507	2794	10843	.	.	.
2684	2768	.	2214	2155	1857	2054	2577	2782	2855	3382	.	2198	2280	2258	1762	2950	2260	12062	.	.	.
2755	3102	.	2708	2565	2425	2257	2299	2560	2697	2592	.	2173	2658	2361	2110	2333	2329	10940	.	.	.
2334	2756	.	2188	2279	2187	2069	2372	2640	2638	2553	.	2018	2270	2667	1806	3355	2223	11284	.	.	.
2523	2856	.	2571	2178	1990	2108	2190	2174	3248	2371	.	2345	2121	2229	2004	2555	2270	11233	.	.	.
2517	3334	.	2117	2958	1996	2262	2932	2547	2694	2550	.	2215	2060	2512	1860	2506	2266	11051	.	.	.
2332	2865	.	2899	2154	2377	2483	2737	2560	2617	2680	.	1988	3156	2584	1876	3144	2139	10466	.	.	.
3816	2895	.	2050	2123	1963	2129	2309	2581	2652	3023	.	2108	2958	2190	1767	2945	2419	7246	.	.	.
2869	2664	.	2105	2071	2042	1884	2485	2412	3249	2572	.	2115	2043	2455	2009	2764	2397	8112	.	.	.
2715	2682	.	2125	2568	2292	2097	2726	2185	2749	2796	.	1992	2206	2435	1629	3803	2601	9309	.	.	.
2474	2817	.	2547	2027	1912	2440	2551	2255	2848	2460	.	1973	2335	2447	1850	3105	2983	9673	.	.	.
2677	3053	.	2050	2035	2617	2039	2363	3103	3756	2738	.	1890	2986	2237	2512	3110	2268	11595	.	.	.
2634	2767	.	1946	2090	2412	2568	2830	2796	3616	2993	.	2617	2448	2782	2554	3478	2443	10342	.	.	.
3265	3479	.	2864	2087	2297	2329	2962	2338	2752	2563	.	1914	3239	2190	1798	2719	2609	13213	.	.	.
2672	2905	.	3001	2057	2798	2146	2798	2569	2704	2899	.	2170	3404	2245	1749	3114	2419	11633	.	.	.
3504	3120	.	2070	2269	2422	2611	2579	2637	3166	2832	.	2634	3084	3085	1908	2567	2991	11233	.	.	.
2722	3057	.	1935	2800	2838	2786	2728	2889	2815	2877	.	2073	2073	2577	1907	2869	2457	12440	.	.	.
2827	3473	.	2346	2969	3053	2275	2673	2588	3482	2822	.	2117	2628	2287	2175	3201	2785	11831	.	.	.
2764	2883	.	2721	2082	3620	2298	3083	3432	2923	3494	.	3121	3245	2934	3107	3119	2998	14324	.	.	.
4002	3834	.	3072	2871	3058	2493	2689	3721	3830	3464	.	3496	2566	2668	2553	3825	2750	16635	.	.	.
4279	3690	.	2769	3548	3129	2975	3782	3444	4042	4076	.	3330	3312	2684	1961	4327	4073	15693	.	.	.
4060	3449	.	2721	3319	2456	3500	3353	2606	3453	3445	.	3315	3358	3263	2340	3938	3081	16421	.	.	.
3777	4038	.	2572	3238	2727	3153	3026	3904	4389	3256	.	2635	2757	3841	2568	3597	3525	17425	.	.	.
4317	4102	.	2663	3054	2377	3019	3166	2858	4067	3951	.	2933	2570	3551	2996	4021	3009	17083	.	.	.
3657	3928	.	3052	2160	2630	2435	3681	2388	4241	4092	.	2779	3092	3547	2900	4153	2887	16675	.	.	.
3720	3682	.	3709	2583	3091	3241	3460	2630	3094	3015	.	2544	2550	3162	2316	4043	3223	16525	.	.	.
3672	3887	.	3316	2575	2220	3320	3177	2497	3658	3531	.	2461	3279	2866	2163	3571	4054	17042	.	.	.
3127	3296	.	2561	2550	2563	2504	3130	2743	3399	3851	.	2469	3550	2454	2297	3800	3817	15842	.	.	.
3708	4281	.	2572	2395	2447	2847	3124	2742	3113	3892	.	2390	3228	2594	2148	3477	3445	16570	.	.	.
3019	4070	.	2633	3294	2858	2981	2608	2540	2922	3605	.	2635	2544	2545	2175	3583	3058	17625	.	.	.
2991	3649	.	3219	3355	2316	2674	2898	3491	3161	3214	.	2596	2672	2429	2556	3723	3081	15330	.	.	.
3196	3406	.	3445	3043	2481	2382	3347	3423	3621	3380	.	2869	2576	2717	2619	4116	3671	16506	.	.	.
4136	3134	.	3712	2597	3148	3215	3312	2627	3411	4084	.	3261	2718	2838	2087	4125	3116	16103	.	.	.
3374	3483	.	3031	2330	3275	3277	3483	1832	3229	3866	.	3250	3083	2804	2838	3706	3090	16271	.	.	.
3155	3843	.	2354	2578	2841	2697	3108	2008	4010	2950	.	2449	2459	3148	2431	4083	3029	16485	.	.	.
3811	3439	.	2901	2564	2035	2395	3038	1969	3740	2925	.	2175	2664	3835	2892	3425	3144	15544	.	.	.
2679	3134	.	2638	3133	2194	2574	2846	2400	3220	3427	.	2650	3137	3349	2897	3961	3868	16012	.	.	.
3264	3490	.	2244	3327	2299	2597	3321	2039	3547	3646	.	2359	2874	3479	2044	3558	3954	16940	.	.	.
3092	3399	.	2743	2712	2431	3005	2841	2447	3086	2878	.	2305	2414	3189	2409	3499	3159	17495	.	.	.
2696	3485	.	2672	2537	2544	3057	2727	3310	3619	3196	.	2639	2337	2774	2434	3717	3290	14967	.	.	.
2856	3677	.	2373	2513	2482	2559	2733	2842	2869	3102	.	3296	2899	3007	2228	3693	2776	14734	.	.	.
2830	3232	.	2296	2966	2198	2352	3335	2826	3403	2886	.	2654	2712	3202	2246	3892	3552	15134	.	.	.
3100	3190	.	2528	3071	2498	2981	3115	1967	3639	3246	.	2493	2429	3202	2014	3729	3954	14342	.	.	.
2955	3118	.	2832	2566	2616	2320	2583	2692	2879	3397	.	2449	3327	2801	1981	3920	2752	15414	.	.	.
3215	3421	.	2661	2631	1977	3039	2451	2280	3461	2747	.	2272	2472	3017	2553	3772	3228	14044	.	.	.
3284	3093	.	2409	2570	2787	3129	2437	2703	3089	3176	.	2237	2414	2640	2347	3460	3216	15517	.	.	.
3796	3328	.	2853	2348	2898	2669	3227	2547	3465	2533	.	2485	2392	2945	1918	3455	2886	16271	.	.	.
3834	3465	.	2220	2606	2230	2530	3259	3234	2663	2738	.	3344	2520	3421	2087	3751	2699	15563	.	.	.
3398	3483	.	3520	2964	2385	2520	2689	2983	3047	3245	.	1919	2076	3143	1948	3718	3248	18526	.	.	.
2676	2806	.	3416	2200	2433	2516	3013	1840	3676	3727	.	2252	2297	2763	1859	3319	2388	14191	.	.	.
2717	2673	.	3065	2438	3328	2307	2502	3139	2711	2901	.	2526	3095	2951	2096	3774	2563	15116	.	.	.
3796	2965	.	1910	3069	2644	2392	2438	1806	3475	2592	.	3262	2873	3221	2670	3693	2925	15414	.	.	.
3287	2938	.	1873	2164	2257	2898	2179	2146	3644	3394	.	2608	1908	2759	2682	2904	2645	14435	.	.	.
2444	3200	.	2923	2223	2260	2931	2924	1935	3408	3639	.	2486	1906	3172	1792	3126	2271	14041	.	.	.
2322	2902	.	1968	2405	2009	1738	2222	2225	2882	3420	.	1848	1640	2366	1731	2818	2211	12110	.	.	.
2372	2796	.	1950	3158	2089	2041	2370	2085	3310	2677	.	1875	2472	2162	1768	3070	2576	11961	.	.	.
2354	3059	.	2854	2024	2027	2207	3169	2289	2738	2782	.	1823	2136	2168	2356	3041	2712	11270	.	.	.
2710	2832	.	2065	2603	2065	2161	2623	1869	2839	2574	.	1761	1807	2407	1870	3152	2439	13707	.	.	.
2513	2879	.	1866	2004	2295	1992	2236	2126	3073	2530	.	2229	2214	2516	1782	2978	2637	10641	.	.	.
2888	2887	.	2014	1852	1957	2090	2294	2018	2718	2624	.	2916	2282	2347	1730	2962	2599	7022	.	.	.
2688	3267	.	1986	1959	1905	2775	2107	2663	2700	2531	.	1879	2102	2624	1752	3326	2374	7694	.	.	.
2337	2657	.	2481	2354	2624	2202	3109	2056	3267	2974	.	1824	2105	2357	1846	3427	2343	7778	.	.	.
3304	3084	.	2160	2015	2086	2299	3032	2143	3092	2461	.	2199	2199	2575	2133	2783	2328	9738	.	.	.
2485	3288	.	2255	2433	1920	2176	2295	2137	2696	2577	.	2616	1991	2395	2400	3111	2956	11110	.	.	.
3073	2859	.	2992	1922	2214	2214	2338	2655	3242	2357	.	2096	2156	2455	1911	2967	2597	10817	.	.	.
2882	3068	.	2272	2096	1978	2016	2332	2260	2855	2583	.	2489	2756	2191	1844	3612	2280	10934	.	.	.
2708	3273	.	2058	2374	2437	2105	2307	1923	2703	3005	.	1940	2461	2325	1931	2853	2285	10622	.	.	.
2501	3012	.	2375	2133	2268	2117	2742	2114	3050	3210	.	1964	2422	2586	2040	2898	2914	10290	.	.	.
2609	2816	.	2696	1911	2128	2952	2615	2864	2813	2565	.	2273	2258	2303	1940	2722	2259	10693	.	.	.
2748	3157	.	2422	2464	2251	1960	2608	2070	2993	2487	.	1766	2272	2215	1891	2731	2080	11792	.	.	.
3361	2787	.	2818	1959	1831	2417	3025	2896	2841	2259	.	1611	2402	3102	2962	2891	2249	12999	.	.	.
2818	2695	.	2145	2496	2010	2097	2796	2290	3216	2372	.	1789	2070	2945	2524	2898	2327	10265	.	.	.
2687	3505	.	2240	2141	2470	2366	2375	2096	3590	3227	.	2456	2284	2367	1915	2578	2943	11428	.	.	.
3073	2601	.	2214	1944	2095	2085	2525	1681	3066	2978	.	2188	2847	2234	2019	2892	2259	11019	.	.	.
2748	2754	.	2268	2158	2266	2103	2628	2857	3266	2436	.	2193	2253	2389	1859	3392	2250	10589	.	.	.
3206	2832	.	2196	2143	2056	2072	2532	2189	3014	2412	.	2034	2347	2453	1973	2756	2604	10123	.	.	.
3179	2821	.	2323	2341	2522	2263	2812	2672	2666	2715	.	2096	2849	2455	2802	3774	2823	11506	.	.	.
2927	2511	.	2282	1940	2484	1874	2873	2153	2865	2522	.	2711	2347	3085	2462	3544	2578	10603	.	.	.

2846	3102 .	2290	2038	2168	2252	2441	2124	3614	2668 .	2134	2559	2486	1830	3012	2406	10330 .	.	.
4639	4157 .	2234	2314	2322	3452	2516	2119	3020	4198 .	2193	3097	3508	2329	2845	2643	10277 .	.	.
4101	3238 .	3009	2271	2234	2401	2827	2361	3795	3671 .	2255	2532	2784	2109	2978	2221	11479 .	.	.
3171	2699 .	2406	2488	2648	2513	3052	2287	2889	3120 .	2047	2530	2686	2295	2913	2555	12007 .	.	.
2710	2951 .	2178	2385	2454	2399	3153	2273	2857	2839 .	2361	3301	2583	2205	2995	2788	10953 .	.	.
3299	2961 .	2418	2154	3153	2114	2560	3399	3210	2854 .	2460	3941	2427	2406	3610	2675	11653 .	.	.
3192	3493 .	3418	2753	3166	2656	2885	3760	3028	3429 .	3448	3314	2335	2266	4441	3566	13333 .	.	.
4138	3849 .	3199	2911	3000	2589	2972	3878	3794	3413 .	2545	3055	2979	2378	3690	3672	14855 .	.	.
4487	3828 .	3652	3023	2854	3060	2879	3739	4676	3866 .	2424	3306	2917	2240	3873	2863	16635 .	.	.
3683	4256 .	4024	3327	2258	3345	4114	3582	3702	3656 .	2555	2834	2938	2571	3373	3128	16375 .	.	.
2943	3672 .	3435	2990	2804	2806	3507	2961	4312	3139 .	3473	3018	2645	3360	3747	3226	17062 .	.	.
4242	3497 .	2667	2427	2540	2743	3511	2410	3268	3015 .	3041	2969	2593	2845	4093	3634	17384 .	.	.
3502	3278 .	2617	2600	3304	2663	2575	3008	3266	3957 .	2113	2470	3486	2789	4213	3685	16829 .	.	.
2968	4065 .	3248	3051	3318	2638	3039	2277	3256	3689 .	2103	2190	2551	2508	3983	3539	16012 .	.	.
2765	3090 .	2534	3247	3018	2756	3041	2446	3436	3213 .	2492	2720	2794	2467	3457	3104	17514 .	.	.
4122	2844 .	3189	3204	2815	2963	3288	2375	3681	3893 .	2360	2701	3821	2445	3238	3032	16829 .	.	.
3901	3425 .	3363	2967	2810	3602	3094	2318	3108	2900 .	2057	3488	3594	2356	3797	2935	17494 .	.	.
3184	3089 .	3172	2489	2640	3167	2866	2269	3364	3877 .	2823	3321	3474	2264	3955	3208	15433 .	.	.
3282	3408 .	2541	2590	2592	3059	2800	3158	3948	3492 .	3585	3055	2770	2952	3445	3203	17471 .	.	.
3330	3649 .	2614	2736	2281	2700	2838	3646	4223	3257 .	2712	2561	2825	3348	3369	3821	16829 .	.	.
3559	3909 .	3079	2604	2855	3009	3309	3100	3825	3408 .	1881	2542	3522	3098	3765	3589	15582 .	.	.
3438	4033 .	3447	3685	3001	2559	2905	2209	3319	3675 .	2252	2725	3047	2739	3350	3094	15758 .	.	.
3311	3029 .	2735	3100	2300	2447	3545	2105	3363	3190 .	2248	2495	2879	2273	3364	3241	16316 .	.	.
3568	3464 .	3150	2424	2366	3309	2969	2597	3249	3097 .	2205	3084	2732	3376	3363	3066	16336 .	.	.
4148	3043 .	3330	2291	2418	3559	2526	2183	3365	3032 .	2178	2904	2763	2037	4199	3568	16422 .	.	.
3567	3040 .	2486	2546	2297	3303	2978	2633	3827	3655 .	2336	2315	3234	2381	3988	3951	15433 .	.	.
2899	3243 .	2545	2531	2627	2789	2878	1960	3255	4032 .	2343	2292	3028	2292	3776	3183	16806 .	.	.
3123	3511 .	2514	2833	2630	3026	2356	2589	3404	3022 .	2596	2458	3117	2534	3590	2998	16401 .	.	.
3608	3283 .	2272	2757	2641	2662	2211	2173	3258	2783 .	2827	2131	3146	3141	3222	2963	16317 .	.	.
3447	3677 .	2707	2291	2604	2950	2481	2649	3545	3118 .	2581	3127	2590	2262	3394	3557	16187 .	.	.
3033	3431 .	2457	2530	2566	2446	3167	2894	3367	3925 .	2305	2406	2226	2482	3468	3551	14705 .	.	.
3505	3739 .	3500	2870	2493	2472	2874	2287	3080	2543 .	2239	2195	2363	2551	3750	3300	16148 .	.	.
3270	3543 .	3333	2503	2543	2920	2691	2042	3390	2957 .	2293	2283	2825	2396	3651	3243	15246 .	.	.
3095	3028 .	2451	2483	2265	2999	2812	2489	3671	2601 .	2126	3246	2910	2376	3202	3616	14557 .	.	.
3312	3144 .	2431	2874	2445	3020	3203	2069	2834	3044 .	2237	2647	3119	2274	3583	3013	15069 .	.	.
2867	3376 .	2881	2263	2930	2238	2250	2639	3115	3471 .	1958	2527	3481	2160	3386	3037	14360 .	.	.
3665	2568 .	2441	2297	2961	2710	2376	2976	3343	3873 .	2283	2249	2538	2196	3396	3182	14492 .	.	.
3766	2447 .	2554	2531	2935	2353	3699	2973	3820	3458 .	2389	2529	2465	2373	3430	3999	15050 .	.	.
3317	2676 .	2505	2782	2639	2352	2243	2478	3116	2795 .	1806	2040	2192	2790	2883	3368	14388 .	.	.
3556	2717 .	2241	2347	2085	2390	3045	2039	3727	2528 .	2038	2019	2502	2191	2771	2442	14733 .	.	.
2678	2418 .	2480	2305	1800	3045	3112	2117	2543	2748 .	2162	1795	3482	2699	2778	2504	12739 .	.	.
2793	2630 .	2121	2045	1891	2907	2397	1950	3161	2484 .	2001	2169	3109	2082	2805	2327	13642 .	.	.
3427	3252 .	2397	2115	2089	2236	2034	1996	3098	2540 .	2983	2061	2262	1921	3364	2413	11428 .	.	.
2606	2794 .	2405	2376	1890	2049	2257	2205	2884	2635 .	2773	2350	2605	1825	2577	2598	11363 .	.	.
2483	2542 .	2527	1916	2041	1844	3222	2086	2520	3154 .	2322	2128	2357	1950	2938	2985	10439 .	.	.
2635	2653 .	2198	2003	2512	1979	2484	2182	2846	2772 .	2026	2820	2424	1739	2967	2241	7302 .	.	.
2843	3256 .	3475	2132	2081	2417	2262	2012	2682	2589 .	1883	3097	2417	1880	2743	2682	9148 .	.	.
2690	2656 .	2993	2217	2298	2275	2271	2547	3114	3031 .	1883	2118	2515	2113	2784	2285	9888 .	.	.
2715	2650 .	2863	2607	2284	2053	2199	2625	2641	2567 .	1672	2513	2420	2120	2523	2430	10942 .	.	.
3040	2436 .	2601	2026	2466	2084	2363	2305	3037	2548 .	2219	2087	2640	1995	2585	2813	10538 .	.	.
2722	2828 .	2474	1822	2256	2265	2471	2892	2552	2863 .	1848	2057	2631	2125	4232	2766	11265 .	.	.
2937	2685 .	2177	2096	2258	2461	2148	2266	3486	2604 .	2398	2094	2385	2170	3450	2294	11624 .	.	.
2909	2410 .	2201	2211	3030	2498	2833	1955	2722	3309 .	1853	2132	2216	1740	2728	2513	12352 .	.	.
2709	2688 .	1972	2018	2408	2085	2295	2264	2641	2541 .	1947	2512	2225	2450	2767	2353	11708 .	.	.
2704	2843 .	2351	2006	2229	2097	2912	2773	2826	2414 .	1816	2324	2205	1865	3343	2555	11843 .	.	.
2738	3086			2025	2123	2365	2345	2709	2607			2225	2062	2979	2328	12156 .	.	.
2772	2714			2043	2101	2368	2050	2584	2650			2222	2550	2711	2607	12980 .	.	.
2807	2592			1975	2076	2757	2052	3449	3214			2953	2460	2697	2278	11963 .	.	.
2747	2485			2395	2055	2324	2680	2780	2721			2996	1887	2930	2792	12536 .	.	.
2709	2852			2124	1982	2374	2013	3306	2798			2467	1830	2765	2628	12570 .	.	.
2714	2672			1958	2347	2491	2302	3002	2314			2454	2014	2740	2898	5986 .	.	.
3252	2693			2242	2842	2826	2256	3288	2949			2473	1942	2695	2522	7244 .	.	.
3460	2679			2193	2087	2760	2256	2673	2806			2431	2318	2946	2657	8776 .	.	.
2758	3018			2230	1965	2555	2890	2758	3037			2335	2182	2662	2462	10648 .	.	.
2783	2730			2206	2082	3312	2205	2830	3030			2290	2308	2836	3052	11013 .	.	.
2769	2808			2256	2104	2732	2311	3438	2966			2643	2159	3670	3079	10831 .	.	.
2891	2590			2197	2267	2535	2324	3440	2617			2608	2651	2612	2236	10460 .	.	.
3307	3275			2356	2304	3368	2804	2924	3657			2423	1973	2518	2617	12536 .	.	.
3076	2839			2924	2180	3397	2327	2896	2641			2631	2190	3112	2590	11279 .	.	.
2905	2862			2469	2443	2734	2485	2590	3375			2939	2194	2813	2326	11727 .	.	.
2985	2765			2213	2218	2420	2867	2957	2801			2436	2263	2879	3203	13447 .	.	.
3404	3662			2386	2080	2526	3490	3132	3278			2828	2340	2958	2966	14239 .	.	.
4269	3921			2827	3079	2564	3863	3352	3038			2479	2698	3955	3803	16142 .	.	.
4182	3635			2948	3094	3106	3217	3829	3209			2524	2153	3975	3526	17085 .	.	.
3482	3674			2875	3848	3788	3121	4070	4157			3952	2508	4253	3031	16376 .	.	.
3282	4009			2640	3221	3008	2047	3863	3456			3922	3437	4212	3163	17319 .	.	.
4113	3295			2321	3000	3145	2584	3288	3056			3872	2900	3345	2988	16270 .	.	.
3867	3460			2503	3144	3085	2601	3461	3023			3474	2100	3439	3327	16460 .	.	.
3128	3380			2255	3092	3262	2716	3821	3101			3197	2898	3705	3267	15797 .	.	.
2955	3655			2556	2962	3127	2660	3289	3948			2592	2803	4179	2951	16486 .	.	.
2899	3599			3070	2758	2418	2908	3285	3889			2740	2417	3464	3558	15816 .	.	.
4354	3994			2877	3218	2460	3476	3280	3573			3009	2267	3495	3716	16011 .	.	.
4001	3764			3101	3515	3209	3027	3712	3252			3521	2142	3548	3131	16077 .	.	.
3820	3520			3089	3139	3189	2607	2985	3152			2850	2534	3465	2952	16037 .	.	.
3047	3269			2588	2634	3035	2387	3142	3384			2880	2251	3608	3252	15739 .	.	.
3258	3520			2505	2749	3601	2547	3024	2965			2773	2699	4173	3322	15563 .	.	.
3712	3553			2970	3093	3594	2411	3739	3157			3359	2506	3931	3976	13764 .	.	.
3618	4002			2425	2922	3133	2355	2997	3326			2569	3124	3542	4172	13800 .	.	.
3338	3311			2300	2762	3079	2321	2844	3037			2552	3155	3748	2986	16142 .	.	.
3307	3553			2306	3329	2868	2505	3157	4030			2990	2908	3903	3199	15348 .	.	.
3827	3882			2487	3596	3018	3360	3326	3972			2959	2948	4048	2975	14405 .	.	.
3730	3335			2627	3067	3001	2632	3405	3299			2940	2195	3824	3069	15609 .	.	.
2976	3282			2210	2948	2626	2607	2956	3076			3548	2191	3764	2895	15479 .	.	.
3225	3174			2554	2543	3288	2635	2943	3008			3478	2544	3690	2974	16122 .	.	.
3171	3129			2408	2559	3365	2727	3162	3242			3378	2876	3828	3361	13764 .	.	.
3341	3021			2514	2537	4128	2273	3326	3226			2568	2269	3314	3298	14817 .	.	.
3311	3340			2574	2555	2830	2555	4018	3331			2468	2065	3580	2931	15348 .	.	.
3163	3353			2252	2838	3058	2530	3587	3034			2565	2118	4054	2963	14126 .	.	.
3560	3453			2505	2697	3163	1934	3329	3837			2856	2751	3138	2614	14275 .	.	.
3332	3519			2950	2562	3056	2289	2912	3700			2748	2112	3393	3285	15013 .	.	.
3309	3892			2926	3313	2692	2753	3091	2648			2839	2341	3177	2522	14061 .	.	.
3372	2619			2804	2793	3225	2470	3002	2491			2519	2131	3187	2852	12628 .	.	.
3520	3344			2899	2505	2392	2416	3023	2646			3383	2812	3357	2675	12708 .	.	.
2567	2794			2962	2789	2426	2511	3125	3058			3506	2872	3819	2545	14370 .	.	.
2586	2648			2100	2057	3071	2054	4120	3719			3107	1944	3616	2249	14191 .	.	.
2993	3142			2111	2500	3285	2429	3687	3086			3408	2870	2700	2452	14732 .	.	.
2701	2761			2264	2133	2905	2380	3367	2459			2341	3068	2860	2063	15570 .	.	.
2439	2487			1997	2041	2572	2044	2603	2438			2412	2622	2958	2817	14928 .	.	.
2530	2470			2408	2004	2912	2277	2600	3550			2424	1849	2563	2294	13014 .	.	.
3059	2315			2114	2052	2313	2692	2564	2439			2332	1974	2962	2468	14714 .	.	.
2530	2483			2346	2093	3132	2521	2535	2690			2638	2005	2556	2322	14237 .	.	.
2603	2779			2249	2177	2502	2249	2940	2669			2361	1978	3223	2934	8587 .	.	.
2539	2838			2746	2253	2896	2368	2644	2816			2404	2152	2579	2291	9129 .	.	.
3152	2516			2104	2238	2502	2890	2764	2555			2376	1855	2888	2215	9673 .	.	.
2530	2401			2314	2106	2630	2406	3058	2434			2302	2007	2795	2310	10947 .	.	.
2589	2982			2218	2054	3454	2544	2700	3017			2468	2596	3002	3179	10752 .	.	.
2431	2591			2227	2194	2704	2234	2671	2879			2408	2162	2814	2263	11638 .	.	.
2986	2659			2279	2033	2532	2494	2958	2334			2627	1996	2523	2258	10863 .	.	.
2667	2790			2052	2151	2425	2226	2625	2543			2409	2031	3382	2756	10901 .	.	.
														2809	2351	.	.	.
														2636	2426	.	.	.
																.		.
