# Supplementary material for: Does prenatal alcohol exposure cause a metabolic syndrome? (Non-)evidence from a mouse model of fetal alcohol spectrum disorder
Source: PLoS One. 2018 Jun 28;13(6):e0199213. doi: 10.1371/journal.pone.0199213 (PMC6023152; doi:10.1371/journal.pone.0199213)
Supplement: S1 Dataset — (ZIP) [file pone.0199213.s010.zip › New folder/VO2 two.pdf]

Blk	3	3	3	3	4	4	5	7	7	8	3	4	4	7	7	8	10
Box	Box-3	Box-4	Box-5	Box-6	Box-2	Box-3	Box-4	Box-3	Box-4	Box-4	Box-7	Box-5	Box-8	Box-1	Box-7	Box-2	Box-6
ID	6.4	9.6	10.6	11.1	17.8	18.7	28.7	41	42	53.5	12.4	23.1	27.7	37	46	50.6	62
Sex	F	F	F	F	F	F	F	F	F	F	F	F	F	F	F	F	F
Exposure	EtOH	EtOH	EtOH	EtOH	EtOH	EtOH	EtOH	EtOH	EtOH	EtOH	H2O	H2O	H2O	H2O	H2O	H2O	H2O
Tx	FE	FE	FE	FE	FE	FE	FE	FE	FE	FE	FH	FH	FH	FH	FH	FH	FH
Chow	54	55	55	89	59	65	42	41	40	42	61	61	61	41	53	43	50
	57	75	93	80	87	91	81	41	56	40	83	52	55	59	71	46	53
	82	83	79	52	81	91	89	64	79	84	83	51	84	70	40	74	53
	58	61	46	54	54	83	51	80	49	80	79	98	93	39	43	45	60
	54	59	93	76	59	57	59	81	40	71	57	93	83	40	59	75	71
	57	78	81	87	76	60	81	91	56	48	60	77	51	69	44	68	59
	57	51	53	87	96	62	74	88	65	71	84	71	96	57	37	36	53
	88	65	69	79	90	89	60	90	62	80	90	89	89	59	39	66	58
	78	88	81	80	52	93	62	88	60	82	58	95	84	63	68	62	74
	71	83	79	80	58	93	75	83	77	84	53	83	105	80	79	65	82
	66	46	92	86	95	93	78	91	65	82	58	101	70	75	78	40	76
	81	73	73	78	68	93	80	87	68	82	96	109	92	76	75	26	80
	86	78	78	84	50	59	52	88	68	94	100	77	94	79	45	69	76
	77	93	104	73	55	57	58	80	68	82	97	85	114	89	82	86	80
	99	85	66	48	88	95	78	76	46	83	91	110	106	93	62	68	77
	103	103	89	80	88	99	73	70	71	77	62	112	69	97	43	85	61
	101	95	90	89	79	96	76	74	73	81	56	72	56	97	90	54	33
	101	99	100	66	88	96	86	74	79	82	67	112	92	98	81	32	66
	110	72	78	49	78	109	81	79	67	70	89	96	109	99	87	31	71
	80	85	55	49	82	100	69	71	48	39	96	66	111	96	53	50	74
	60	102	54	49	54	86	39	43	36	31	108	56	101	96	41	80	67
	71	108	89	52	91	55	35	35	32	72	110	65	93	99	48	88	103
	84	107	91	72	82	50	36	38	44	80	95	53	64	100	94	88	109
	64	103	61	84	44	56	39	36	41	68	101	100	73	96	90	74	104
	82	111	48	87	25	82	59	64	42	53	101	99	69	86	83	44	98
	95	96	89	73	27	65	44	67	70	42	94	95	62	66	69	40	88
	100	61	74	66	22	55	69	31	54	35	89	66	66	37	42	40	88
	95	81	57	55	19	61	64	32	29	36	94	54	90	35	38	50	83
	94	65	76	49	19	71	89	37	32	37	72	53	98	62	45	48	41
	89	65	90	50	15	72	76	36	40	47	55	49	78	51	61	77	27
	59	54	84	55	78	56	39	54	36	80	54	63	55	43	81	79	24
	44	102	69	47	86	54	42	74	47	65	62	54	60	41	46	73	37
	69	96	49	64	99	56	36	49	55	70	54	77	85	47	46	46	33
	47	84	71	50	97	54	30	32	62	42	69	111	94	47	72	60	38
	60	86	72	50	100	92	78	31	43	31	90	105	61	62	76	49	55
	42	59	51	51	97	78	86	34	20	32	103	86	55	93	72	41	38
	47	57	44	51	96	50	58	57	25	35	89	63	103	84	48	81	38
	49	90	49	91	90	51	40	78	35	61	76	55	101	54	46	78	42
	43	66	90	83	96	67	37	77	57	77	49	53	73	44	49	55	92
	44	50	55	59	91	90	42	56	30	76	51	68	85	70	45	39	99
	69	53	46	50	74	84	91	37	25	48	55	78	66	89	58	44	84
	98	84	49	53	48	68	73	49	38	38	56	59	55	83	76	62	77
	86	79	76	50	45	51	44	54	74	37	97	57	55	58	57	43	48
	62	55	56	80	49	56	40	39	31	47	87	65	92	61	50	44	36
	49	49	49	79	54	52	56	38	28	77	50	83	86	48	68	73	37
	56	89	80	62	49	83	44	42	34	79	58	78	69	45	60	38	40
	89	63	80	52	78	77	43	66	36	49	60	54	52	84	44	41	44
	62	54	74	55	57	51	79	62	79	36	100	55	57	88	41	76	44
	53	80	51	56	48	55	52	41	77	40	79	68	52	60	74	47	34
	54	82	47	56	53	50	47	38	53	44	54	54	77	42	71	37	36
	54	50	67	51	96	75	58	32	35	40	54	50	85	66	51	41	45
	92	52	57	51	93	68	42	39	32	43	66	49	56	50	69	71	90
	87	69	57	52	93	52	41	35	35	51	59	77	76	65	42	93	71
	63	57	61	76	77	52	48	43	38	76	59	55	86	49	65	73	38
	57	53	56	80	52	65	70	67	75	50	97	51	63	73	83	45	40
	57	53	51	65	49	56	44	51	72	40	83	61	64	84	59	43	81
	58	77	53	53	55	56	39	32	52	56	82	88	81	58	46	45	88
	64	76	63	53	71	56	40	38	35	76	83	88	58	42	42	56	52
	93	48	57	53	79	81	83	37	30	62	60	56	55	80	71	71	34
	96	52	50	68	57	87	50	34	38	40	57	51	80	48	70	42	38
	80	48	86	76	57	70	43	28	76	39	59	89	94	41	48	41	40
	58	78	82	64	77	53	80	50	53	43	65	96	95	69	53	55	39
	58	52	52	52	64	57	57	71	30	70	57	91	63	53	59	84	42
	56	50	46	52	74	86	54	71	31	68	57	91	54	47	43	71	41
	57	85	61	59	64	85	47	50	77	42	54	85	58	45	71	70	82
	58	70	74	52	56	63	72	40	71	40	88	55	67	43	81	54	83
	83	48	56	70	65	68	58	39	53	74	91	51	102	47	79	55	80
	93	89	58	82	92	92	81	72	79	62	81	54	97	78	80	77	47
	69	97	86	63	91	103	79	54	69	59	59	75	94	89	83	53	36
	63	88	91	64	87	92	62	71	81	91	63	89	87	83	77	80	41
	82	95	86	89	76	104	77	88	58	71	71	100	103	83	49	81	67
	91	89	88	85	64	110	84	83	75	62	103	89	92	55	49	50	99
	90	76	91	97	63	84	78	84	83	85	102	86	93	59	59	50	83
	98	90	88	81	86	55	51	81	92	81	99	90	62	67	73	79	98
	95	93	91	80	73	58	76	78	78	85	96	98	57	99	49	61	85
	77	68	92	77	62	59	54	85	68	76	97	106	98	77	54	48	95
	84	86	87	80	78	57	73	78	88	77	77	101	103	61	83	71	82
	85	63	64	83	77	75	55	73	72	67	58	106	105	42	75	69	50
	94	58	79	71	55	98	46	56	82	86	75	102	96	77	73	54	41
	91	59	73	54	78	93	48	74	78	46	82	96	97	51	78	80	96
	97	91	57	53	60	89	70	48	67	59	62	96	96	59	66	64	71
	101	97	100	77	59	93	79	39	40	40	57	86	100	83	71	53	46
	105	103	78	80	72	80	82	41	31	34	85	62	91	87	59	68	51
	101	91	60	63	87	90	81	55	35	51	73	47	64	69	66	94	88
	103	94	78	82	89	93	72	80	50	74	92	52	54	72	40	101	107
	105	102	96	79	89	86	43	90	77	78	101	51	94	95	43	88	83
	96	96	90	74	76	67	47	79	56	75	94	50	101	90	43	80	76
	95	92	84	50	55	52	63	70	37	73	108	58	89	85	49	83	59
	95	91	51	78	62	55	44	73	46	63	96	88	62	77	38	76	43
	82	94	82	61	56	55	57	57	73	45	80	72	63	55	61	74	36
	65	67	88	55	55	54	82	44	61	44	50	53	91	44	95	77	38
	59	57	86	52	59	52	59	38	39	50	53	48	77	39	52	75	42
	73	62	61	68	56	56	53	42	48	43	83	48	54	42	58	49	61
	86	83	49	61	55	51	43	40	51	43	67	51	87	64	48	49	39
	67	93	80	58	85	79	41	68	35	44	59	54	90	72	77	52	41
	56	88	60	55	76	74	42	68	38	41	98	55	84	75	46	50	49
	80	60	72	77	57	49	75	45	52	53	91	88	55	50	52	57	48
	83	73	66	80	52	53	55	57	31	75	84	83	58	38	61	47	91

72	51	49	70	52	49	37	69	37	70	79	54	54	40	84	66	84
55	53	77	83	61	62	48	60	40	70	86	48	91	49	69	73	77
58	85	83	68	97	46	37	37	77	52	56	92	83	48	41	45	72
55	53	73	50	90	85	85	35	73	35	48	101	60	87	37	42	60
78	47	48	49	87	85	56	54	77	40	47	95	54	66	48	43	36
96	94	47	54	77	79	39	38	67	48	85	64	60	45	78	70	27
66	80	56	53	76	72	58	45	52	66	62	49	89	69	50	75	43
54	54	67	69	51	49	92	73	37	42	47	42	86	83	70	63	33
52	44	61	49	55	51	49	71	40	39	73	70	71	56	59	46	39
56	79	64	53	53	84	37	41	37	49	55	49	58	36	42	75	38
84	54	51	73	55	67	82	42	69	41	50	67	62	77	47	52	69
91	47	88	80	54	49	46	42	81	84	75	68	92	71	75	51	41
76	47	54	54	61	46	45	50	50	61	94	43	64	47	59	82	77
55	48	55	55	66	69	46	65	41	42	85	48	57	43	46	79	90
76	86	55	56	52	51	42	63	36	44	60	69	66	72	54	57	77
61	75	72	72	52	51	46	59	45	70	54	85	70	87	45	72	80
78	44	75	59	64	54	77	40	49	51	49	68	58	77	46	53	49
54	43	60	54	53	75	64	44	75	40	72	48	81	77	70	50	47
55	79	50	51	53	53	38	38	68	40	60	43	79	46	65	48	42
78	50	48	53	71	52		55	41	42	49	52	76	40	49	77	40
86	46	50	56	63	67		74	40	44	56	78	59	57	65	73	85
78	63	54	80	54	52		47	39	39	57	64	60	43	43	56	55
61	62	78	63	56	51		41	64	59	78	52	98	51	43	51	46
55	49	54	56	61	57		41	73	82	73	51	73	84	62	59	45
56	49	52	54	58	90		37	59	80	56	56	57	52	41	59	42
59	91	77	60	75	80		39	41	66	53	55	57	40	43	75	43
59	74	73	55	58	58		42	35	44	82	80	65	40	68	80	84
73	55	54	58	58	56		75	37	44	91	97	63	73	84	50	76
60	49	52	81	59	56		53	39	69	73	66	92	82	58	50	46
58	51	53	79	59	79		40	60	51	51	51	93	76	49	48	39
77	91	79	57	75	73		42	74	40	56	50	67	51	45	84	76
89	73	57	56	67	58		72	69	45	85	81	66	44	65	76	49
73	55	54	56	56	58		59	45	73	68	80	66	45	45	53	39
59	50	74	76	57	69		43	41	79	55	53	80	43	44	76	48
66	89	66	70	59	92		77	45	81	56	63	108	70	81	95	42
63	97	85	61	80	97		84	60	88	95	87	99	69	84	89	84
88	91	102	84	66	92		85	82	92	97	103	102	79	54	83	72
94	81	92	86	62	94		90	84	92	69	98	97	89	77	82	49
76	82	72	66	68	88		83	80	85	62	97	71	78	94	66	82
86	97	83	65	85	78		85	54	80	96	96	63	45	64	58	68
93	103	96	57	93	58		83	83	85	97	64	94	72	81	78	87
77	90	91	72	78	64		56	79	90	69	96	98	84	80	83	57
67	85	95	88	85	98		37	81	81	61	99	102	82	51	63	77
86	67	82	90	72	92		36	79	79	89	103	102	59	59	53	57
101	61	79	68	78	61		74	74	80	101	102	97	52	59	57	50
74	105	82	87	84	72		84	71	78	95	96	97	50	45	81	81
94	101	90	90	83	68		84	66	74	89	94	73	54	55	82	92
106	103	67	79	84	94		88	50	44	61	60	91	72	80	81	86
102	98	93	63	73	89		63	27	34	96	53	74	97	55	90	60
76	101	88	78	54	95		41	57	39	64	50	62	88	71	93	59
101	96	71	85	55	73		68	78	36	55	72	93	62	71	85	47
96	89	93	91	53	61		84	78	82	75	84	92	46	46	90	40
103	96	88	68	84	57		76	72	88	81	80	75	63	55	87	39
97	89	81	55	83	53		55	47	55	60	101	98	90	44	86	72
107	90	81	61	77	74		47	29	71	54	96	92	93	57	87	94
99	64	73	65	56	79		76	33	74	54	96	66	81	56	80	102
95	59	71	81	54	57		63	33	61	105	94	63	82	45	71	99
94	87	67	76	63	56		43	75	40	100	86	75	89	51	54	66
87	95	56	69	79	51		57	64	38	92	79	94	73	46	44	78
96	102	62	56	80	52		45	40	41	84	61	72	69	57	67	86
66	87	92	57	73	68		58	63	40	102	49	62	42	44	78	56
60	61	65	78	82	52		47	68	50	96	51	75	63	84	83	41
85	84	57	77	56	53		64	48	82	87	51	79	65	82	53	39
67	96	87	60	52	64		80	34	64	85	54	98	50	71	48	45
61	85	73	60	57	88		57	41	61	51	51	91	48	70	47	36
59	61	57	62	57	91		40	42	40	43	66	70	52	71	79	45
61	63	51	79	86	91		44	77	68	81	87	69	85	71	84	57
94	92	57	68	91	84		56	65	46	103	58	52	86	41	79	38
69	80	86	56	85	57		44	40	34	90	46	55	58	41	51	41
58	51	69	52	72	50		45	38	34	67	46	93	43	47	45	87
54	51	49	51	52	58		43	67	35	44	89	65	51	71	49	92
61	54	85	75	50	64		77	64	64	48	58	59	82	65	71	87
55	67	79	70	72	81		70	47	63	50	48	55	78	42	51	55
58	59	54	50	86	91		59	34	69	60	48	59	51	44	50	41
86	82	72	55	89	79		41	66	48	90	47	96	47	56	48	42
58	80	57	51	81	51		43	77	46	55	87	93	44	67	51	59
56	56	58	71	63	54		38	69	41	50	63	59	80	65	74	41
63	57	61	53	68	62		58	43	72	54	56	58	70	42	54	41
87	83	75	70	51	54		43	30	46	82	54	58	53	45	55	66
62	60	77	52	52	47		46	51	46	78	80	88	47	44	79	53
56	58	75	52	58	58		41	66	41	50	63	65	50	44	73	40
63	59	54	75	78	83		69	51	65	51	49	57	50	67	54	79

	98	88	69	79	97	97	88	86	51	67	78	78	97	91	62	82	101
	97	75	75	86	91	95	66	56	81	63	91	91	98	79	55	87	98
	98	73	65	82	92	71	59	84	53	50	97	88	97	89	66	88	93
	95	94	70	56	82	89	92	66	41	46	91	70	68	68	50	71	94
	91	69	67	80	79	67	69	59	71	48	76	90	84	65	55	62	83
	67	62	68	63	75	64	57	69	45	75	90	74	89	64	69	72	60
	93	86	76	57	83	83	86	61	51	67	89	67	67	86	58	61	75
	77	72	68	62	69	77	55	62	51	45	63	66	68	87	50	57	64
	88	96	73	61	71	74	57	80	50	45	84	75	97	60	76	79	58
	73	79	73	88	69	68	81	59	78	71	80	80	73	65	59	66	80
	90	73	79	80	95	74	67	58	53	51	67	68	67	59	52	57	56
10kcal%	75	70	78	93	72	91	54	72	51	55	67	70	93	75	81	64	51
	72	82	84	88	72	96	53	84	74	77	68	71	73	71	59	87	85
	95	102	96	82	96	97	70	81	79	83	93	76	97	82	74	90	70
	85	104	93	91	107	80	74	81	91	77	74	90	101	97	88	77	63
	101	80	96	92	104	74	64	84	77	79	91	99	75	72	94	84	58
	96	75	97	89	106	91	80	60	82	78	108	98	67	74	83	82	81
	98	83	80	65	107	92	76	61	77	84	72	91	95	86	100	74	81
	91	85	84	84	106	72	82	66	70	85	68	100	106	77	65	79	84
	101	102	95	84	103	73	84	70	46	76	89	77	88	82	49	65	101
	103	97	79	64	100	83	88	89	43	80	104	70	102	82	68	73	85
	91	79	83	62	105	95	84	87	43	72	121	94	93	82	73	88	94

93	67	80	87	96	70	94	95	46	73	95	101	94	69	83	91	99
82	92	97	90	76	94	69	83	74	73	76	78	112	81	87	77	89
71	75	93	82	80	96	90	87	78	93	97	70	73	88	89	61	79
95	100	105	62	64	98	81	82	75	75	104	68	64	82	77	66	79
94	89	106	66	94	110	73	62	52	58	86	71	64	75	70	89	61
102	103	99	84	96	95	55	72	43	50	86	114	70	92	59	77	45
101	95	99	87	71	86	87	82	41	40	71	82	82	81	48	86	61
103	93	87	82	64	69	74	78	46	50	72	74	80	60	59	83	50
89	95	76	72	68	94	54	79	71	36	67	93	72	90	60	65	46
104	87	89	57	94	72	84	71	81	37	70	93	65	94	49	72	77
93	74	88	58	75	62	65	65	52	66	81	89	81	85	48	67	61
100	93	83	52	65	63	54	67	40	75	93	74	103	82	53	55	50
80	114	68	90	99	81	75	67	36	71	93	75	94	76	76	79	43
75	93	94	78	74	63	61	68	43	68	91	76	63	76	71	97	43
87	84	83	78	64	60	53	69	51	71	93	80	65	74	55	81	85
81	86	83	82	74	54	48	78	40	63	108	78	64	89	49	60	88
88	75	94	63	67	65	64	72	72	50	82	88	93	73	52	62	57
93	74	83	61	63	98	52	82	65	57	98	82	80	69	47	81	47
85	79	77	65	75	82	55	75	56	59	93	83	64	70	51	94	48
73	73	85	76	68	64	81	72	40	55	98	78	60	64	77	83	61
66	67	76	66	71	61	59	69	42	66	80	73	95	73	51	62	52
73	71	72	60	92	60	58	76	54	48	60	70	93	65	63	59	45
84	90	96	57	104	68	50	61	73	45	89	87	69	83	80	59	62
71	90	81	57	93	63	48	53	58	42	63	91	62	79	54	76	84
72	71	67	53	93	84	46	60	39	72	59	73	59	64	47	92	80
75	60	67	68	88	93	83	54	40	49	95	67	89	67	51	80	74
89	68	73	83	78	75	75	60	51	45	93	82	63	72	74	65	50
71	77	67	70	66	64	59	53	58	40	85	77	60	61	64	71	50
78	65	76	52	64	63	46	62	51	63	76	71	100	77	63	60	73
66	76	65	56	88	62	77	59	52	67	63	64	99	81	61	56	68
89	88	62	60	70	61	54	62	89	44	65	64	69	71	56	65	45
65	76	71	81	93	92	49	55	74	49	67	60	64	88	68	58	46
68	66	68	79	89	84	64	55	53	51	56	87	64	69	53	65	82
80	77	67	61	80	59	51	82	52	69	73	74	83	61	52	74	85
70	60	89	78	69	65	61	76	60	49	59	64	68	58	53	63	67
69	90	78	65	70	70	75	58	51	44	62	63	93	75	55	60	49
84	63	68	60	83	88	59	58	50	48	79	72	89	61	52	57	55
67	64	67	64	66	64	63	59	66	65	89	62	68	52	44	76	68
83	61	83	60	81	64	68	56	66	55	82	90	65	56	83	68	52
80	78	85	62	69	78	55	52	52	47	63	75	64	52	67	61	50
64	61	71	61	80	64	66	61	50	55	62	63	98	57	52	62	84
81	73	68	72	88	87	45	67	52	68	76	57	94	62	52	61	83
70	81	69	80	64	87	52	51	46	58	89	62	64	86	51	57	59
82	62	81	62	66	68	70	47	43	45	62	86	69	56	44	72	47
69	63	67	56	69	66	46	82	57	45	62	77	84	71	48	57	50
79	82	67	59	69	76	52	69	71	42	62	65	70	80	65	56	54
72	72	67	55	94	66	81	50	62	61	95	65	80	66	78	60	49
81	64	76	57	71	64	59	44	44	66	69	61	65	51	68	82	70
72	62	79	81	77	95	54	72	44	49	67	94	88	50	52	83	99
81	95	67	65	69	94	87	75	52	45	63	86	88	51	47	64	88
67	89	66	56	72	69	74	56	67	40	66	93	66	64	47	60	62
80	73	67	63	70	71	55	51	46	63	85	75	65	82	69	62	63
69	63	89	82	88	71	56	52	45	49	83	76	64	57	63	62	105
72	89	66	65	77	74	62	66	51	45	66	82	86	54	59	79	75
96	72	63	64	98	96	65	81	72	65	67	74	92	58	54	61	53
79	77	85	89	111	75	62	74	82	70	83	81	110	78	88	86	50
78	106	101	98	117	96	80	73	76	81	105	102	108	67	95	95	53
94	106	82	98	121	106	84	96	80	93	112	108	117	86	76	95	82
109	103	96	89	119	95	75	89	75	89	114	116	99	89	71	92	64
105	103	106	63	105	99	87	63	49	94	104	102	94	77	82	91	94
102	103	110	89	99	88	87	52	86	83	109	75	93	74	75	102	96
100	87	93	88	100	94	88	69	74	81	105	98	100	70	70	84	85
107	65	100	93	102	82	77	82	65	78	99	97	85	52	45	74	88
88	87	79	90	100	64	74	76	48	83	106	95	95	70	42	81	89
100	70	83	99	89	102	77	71	48	103	114	93	90	88	82	85	100
82	98	86	85	101	99	55	74	54	85	112	85	110	93	81	86	90
105	95	70	65	78	70	71	74	72	73	106	68	81	82	71	75	57
104	78	60	64	65	61	68	76	72	70	110	65	66	53	48	82	59
99	80	95	87	79	66	55	79	65	46	98	70	65	50	44	77	52
114	69	94	98	105	58	45	72	56	70	70	76	63	53	68	73	52
106	69	94	82	81	81	48	73	48	41	69	75	98	51	53	68	60
101	94	94	81	83	97	75	67	52	42	73	65	111	82	43	69	90
101	115	89	59	65	95	56	69	86	70	68	86	99	92	54	83	85
90	105	65	55	98	86	54	69	85	74	79	66	103 .		84	68	83
97	97	80	55	99	96	51	55	54	64	70	65	96 .		73	87	91
98	94	71	59	97	104	78	50	51	75	64	65	89 .		64	92	82
88	90	77	87	68	71	75	62	55	48	63	63	63 .		73	67	81
84	81	81	64	80	86	72	100	69	43	57	64	69 .		60	76	77
85	89	60	58	**75**	**82**	70	68	71	47	70	**64**	**65** .		45	75	68
79	82	60	81	**68**	**64**	46	49	55	73	105	**97**	**64** .		46	65	47
85	63	67	87	**101**	**66**	49	46	68	60	107	**87**	**95** .		44	69	45
72	62	75	80	**108**	**65**	44	52	58	43	104	**62**	**97** .		45	74	47
87	75	96	76	**92**	**61**	43	68	49	86	116	**62**	**73** .		80	78	83
79	88	99	60	**97**	**63**	63	56	48	64	105	**90**	**69** .		72	74	73
85	96	96	59	**78**	**74**	47	86	47	53	96	**88**	**68** .		59	75	53
83	98	82	84	**69**	**93**	49	75	48	55	91	**87**	**98** .		48	71	52
81	82	88	92	78	86	44	56	42	47	80	81	96 .		42	62	54
77	64	82	88	70	61	46	46	37	63	93	94	97 .		36	60	53
82	85	83	78	93	88	51	43	74	65	96	86	71 .		79	80	83
96	86	65	76	91	80	45	60	69	43	77	71	64 .		68	68	70
75	63	57	76	69	64	84	48	44	38	63	64	70 .		73	57	49
73	66	56	63	65	56	81	42	39	43	65	82	59 .		46	54	49
78	88	82	63	60	77	64	44	54	57	84	92	69 .		48	71	89
70	62	59	65	87	74	48	78	48	66	62	75	79 .		44	60	85
72	66	61	60	66	63	48	69	37	45	61	56	65 .		57	61	65
88	84	60	83	60	62	76	47	51	41	80	75	61 .		44	59	48
79	70	57	69	96	77	71	48	39	45	58	62	61 .		44	60	52
75	63	76	63	82	64	63	47	42	63	59	61	70 .		68	73	70
70	74	80	59	66	71	49	64	42	59	102	69	98 .		68	60	56
69	60	59	81	67	60	47	51	38	43	77	60	93 .		56	55	79
76	58	61	77	64	66	79	49	68	43	65	92	82 .		44	76	73
73	63	58	60	79	98	55	46	84	41	61	77	68 .		43	81	61
73	84	79	60	69	76	51	46	77	68	61	59	76 .		46	61	64
80	65	64	64	82	70	71	71	53	65	79	67	68 .		58	58	73
68	63	58	58	82	67	51	55	46	46	82	60	71 .		45	59	53

	80	71	60	72	63	55	48	49	43	41	59	70	68 .		46	60	51
	70	89	72	59	81	57	50	62	49	44	60	63	63 .		45	66	74
	64	77	58	59	71	59	29	75	56	43	56	66	78 .		81	58	87
	66	60	87	59	66	59		61	50	62	85	78	92 .		81	57	58
	59	61	85	77	67	94		68	45	72	63	75	103 .		64	53	65
	80	91	66	87	84	67		59	44	47	64	55	91 .		54	78	57
	60	94	60	72	71	63		51	46	43	96	61	77 .		52	76	76
	61	72	57	58	66	64		49	67	44	86	61	64 .		52	70	53
	89	65	59	81	78	66		47	60	55	64	60	59 .		48	60	53
	103	94	85	87	108	69		65	40	59	60	90	66 .		83	64	71
	78	81	79	72	95	85		82	44	43	65	63	83 .		82	58	90
	64	67	61	63	71	87		57	41	36	74	76	65 .		52	79	87
	65	84	60	60	69	62		51	46	59	90	85	64 .		50	59	60
	74	75	88	87	98	88		55	81	40	70	92	93 .		50	55	87
	71	66	71	72	95	73		73	85	44	71	98	71 .		75	80	81
	82	92	64	66	76	70		77	58	72	75	73	89	64	92	67	69
	79	77	93	75	76	98		102	58	54	99	68	84	103	93	58	61
	92	110	93	93	100	94		89	82	93	116	95	107	110	84	83	64
	105	107	99	89	106	97		97	83	82	110	96	98	94	75	87	103
	79	110	86	88	108	104		86	76	83	120	111	105	62	55	80	108
	84	99	72	96	99	98		72	68	75	100	90	100	79	74	77	91
	86	98	112	85	94	90		94	40	82	100	112	100	72	73	83	65
	81	86	96	94	83	82		73	43	73	79	96	88	46	49	75	86
	99	102	87	94	110	94		70	75	68	82	100	91	38	46	60	89
	100	83	65	90	96	113		80	72	64	117	72	96	84	55	52	74
	89	69	92	87	100	98		54	70	55	116	80	92	90	50	64	61
	94	82	93	66	88	75		45	70	68	107	75	71	55	74	85	91
	71	77	65	58	74	68		48	65	43	102	62	74	52	79	83	94
	79	93	57	60	94	81		56	65	34	109	78	65	54	73	71	75
	68	95	89	99	83	74		87	69	51	101	79	65	76	88	71	74
	97	84	104	88	88	69		74	67	62	102	62	65	105	70	85	67
	98	89	79	63	108	76		67	55	41	106	66	103	92	70	68	58
	77	71	70	63	96	88		71	38	35	73	63	98	57	59	68	53
	78	67	65	59	97	71		81	38	81	74	104	73	51	52	68	65
	68	66	76	64	87	71		66	37	69	98	93	63	46	47	69	77
	97	101	97	62	97	102		64	38	61	83	87	65	46	47	52	61
	98	97	98	93	97	94		56	39	76	105	93	66	67	49	54	70
	99	88	91	96	84	92		42	38	72	113	86	89	85	70	44	71
	93	70	78	95	87	80		43	63	55	103	80	91	80	49	66	82
	87	68	65	93	90	71		48	68	45	89	70	88	72	56	55	72
	68	58	60	85	78	72		59	45	35	97	70	80	47	82	54	52
	77	94	64	88	98	83		43	34	53	84	71	92	46	66	53	66
	77	90	71	85	78	76		42	36	40	70	66	68	46	43	70	96
	69	67	59	80	95	70		92	39	35	81	65	67	66	44	70	82
	93	86	86	80	83	74		80	36	38	66	66	72	72	43	81	57
	94	103	90	69	78	70		61	34	71	64	97	60	53	54	72	59
	89	85	86	63	84	72		58	71	67	95	77	64	46	77	66	56
	86	88	82	65	82	71		45	69	72	90	64	79	42	66	64	72
	86	94	88	82	97	62		44	55	62	95	58	62	43	62	76	92
	81	76	83	77	96	71		45	31	36	84	64	62	77	55	60	74
	75	62	65	63	89	65		54	47	30	83	86	69	66	44	48	63
	66	61	67	66	83	68		60	37	33	62	88	75	62	44	49	57
	84	60	64	65	77	90		60	36	34	59	76	92	50	50	77	77
	82	85	58	65	83	89		46	38	72	66	62	91	42	66	71	63
	86	64	78	61	68	63		63	71	65	72	57	79	45	39	57	71
	68	87	59	83	70	67		85	91	54	63	97	65	78	48	57	53
	70	64	63	68	69	62		63	74	38	58	68	69	68	73	57	63
	66	62	66	58	87	64		52	44	35	87	62	65	50	47	53	49
	70	60	62	74	66	64		52	44	52	69	77	65	50	62	61	54
	71	71	67	65	66	78		53	40	53	63	67	71	50	52	58	53
	71	54	78	63	70	67		70	51	42	62	61	70	63	48	50	49
	78	62	69	59	82	62		45	58	40	77	68	88	47	47	71	85
	64	77	59	62	84	63		46	43	41	69	58	73	44	51	65	68
	95	87	92	87	71	87 .		91	77	71	98	75	72	93	72	75	97
	92	85	86	79	75	75 .		81	77	56	90	86	80	92	76	72	98
	94	78	85	88	68	81 .		68	85	71	99	69	73	83	64	84	88
	88	72	83	83	83	75 .		64	58	55	94	69	75	81	61	77	84
	87	70	77	76	88	74 .		81	52	65	88	67	70	63	75	75	74
	78	85	79	82	74	71 .		63	66	54	80	75	70	59	65	69	77
	77	75	70	70	73	76 .		62	49	54	85	76	75	60	59	68	72
	81	81	64	71	79	82 .		57	57	65	76	71	105	68	69	78	62
	76	81	73	88	75	81 .		63	59	56	80	70	81	71	64	66	72
	77	77	69	71	70	76 .		55	71	52	83	82	79	59	64	63	66
	72	81	77	77	88	78 .		62	55	59	83	71	78	57	74	70	67
60kcal%	81	88	73	80	76	77 .		72	61	83	82	77	99	64	68	78	74
	81	115	83	104	85	104 .		98	55	92	106	102	77	66	74	121	69
	100	112	107	89	96	102 .		79	80	89	100	87	82	82	86	104	91
	108	115	107	91	110	101 .		83	76	82	96	108	120	63	66	94	105
	119	112	98	77	104	87 .		76	71	80	88	105	97	84	79	81	100
	108	104	97	93	103	84 .		72	75	87	83	110	100	81	68	85	92
	83	110	96	77	102	79 .		67	66	81	107	108	102	80	78	85	92
	81	98	93	93	83	99 .		70	77	82	99	90	104	96	72	75	92
	81	90	93	78	88	94 .		60	65	81	88	94	101	85	73	67	80
	75	88	90	75	80	81 .		58	60	81	76	76	92	86	68	81	61
	76	71	84	78	78	83 .		69	46	81	81	88	99	79	61	73	86
	72	84	71	75	96	75 .		81	47	70	83	87	99	66	61	81	68
	105	77	73	71	100	87 .		79	45	51	113	70	81	72	80	71	81
	111	79	76	82	94	91 .		59	70	58	99	71	73	58	81	73	85
	100	75	71	100	91	93 .		59	56	55	109	92	76	62	65	66	64
	98	85	73	86	88	92 .		95	45	52	103	69	85	66	72	90	62
	78	94	101	90	92	98 .		74	53	78	107	71	76	60	70	84	60
	108	96	93	89	92	91 .		84	81	80	104	67	74	59	57	92	83
	95	89	71	99	86	77 .		72	72	78	111	89	83	82	70	80	87
	90	74	79	86	76	81 .		65	59	75	102	75	76	81	78	77	86
	95	94	88	77	68	85 .		59	83	73	87	74	105	75	70	80	66
	97	108	96	91	66	97 .		55	68	69	88	80	91	73	65	68	58
	87	83	87	89	64	93 .		54	47	53	85	89	94	57	56	72	61
	81	71	91	75	81	86 .		56	50	62	87	93	86	69	60	73	60
	78	70	89	73	78	89 .		60	48	57	81	100	68	62	63	67	55
	73	66	83	72	67	81 .		69	44	57	85	91	75	55	76	61	60
	87	74	71	75	84	90 .		53	54	47	103	91	101	61	55	62	61
	77	70	80	75	68	80 .		52	47	47	90	93	92	58	72	61	79
	76	66	79	85	67	91 .		74	48	48	98	81	86	60	66	68	88
	78	96	95	94	73	91 .		75	77	76	94	74	71	68	58	87	96

103	97	88	75	100	74 .	67	72	75	84	87	68	75	57	80	85
90	89	83	67	99	79 .	55	65	76	84	78	75	81	56	67	54
81	97	75	99	91	80 .	60	60	79	76	66	88	76	56	67	68
87	90	85	79	88	91 .	73	50	72	74	61	88	91	72	80	91
84	93	82	71	94	91 .	56	39	65	106	92	80	80	67	69	84
78	79	73	72	83	75 .	51	43	52	81	96	70	67	56	60	84
69	73	66	72	77	74 .	51	51	44	77	79	69	59	56	58	76
83	71	69	66	74	68 .	51	46	61	71	68	64	67	52	59	72
71	68	67	71	81	71 .	71	45	53	79	66	71	56	61	64	61
74	72	66	85	67	66 .	57	46	44	77	80	72	58	66	76	64
71	65	78	72	96	71 .	53	57	49	90	67	74	52	55	64	67
78	63	71	66	79	75 .	54	75	72	75	67	81	53	55	69	67
72	72	67	65	92	81 .	64	65	72	79	75	74	57	64	64	55
73	78	79	79	76	75 .	70	60	60	87	68	83	69	55	64	58
83	72	67	65	74	68 .	70	54	49	93	65	71	68	73	79	86
91	67	78	69	74	73 .	58	51	57	80	69	75	59	58	71	78
75	68	62	68	84	67 .	54	49	54	74	67	71	75	55	61	69
73	89	67	75	73	70 .	58	46	60	74	91	87	61	51	62	60
78	82	79	68	81	68 .	69	63	56	77	82	70	71	58	72	63
72	84	79	72	81	90 .	68	66	56	90	74	71	68	59	66	60
72	79	72	68	71	82 .	62	64	56	93	71	73	60	59	64	60
72	75	69	69	66	73 .	74	61	56	71	69	79	60	69	79	80
72	72	83	74	83	72 .	64	61	49	71	72	78	82	61	70	73
82	72	77	89	75	82 .	63	63	66	79	74	74	66	58	66	70
71	89	73	71	70	83 .	66	68	60	76	70	92	57	54	72	64
92	94	70	73	68	79 .	68	54	60	71	77	72	63	63	71	77
71	72	82	78	65	71 .	65	51	53	71	76	74	64	79	78	69
78	73	76	79	87	68 .	73	66	67	80	87	88	63	67	81	67
77	71	70	67	71	71 .	67	73	49	82	79	77	58	65	75	71
73	78	72	85	82	79 .	67	57	58	81	76	76	92	57	74	67
75	77	86	82	74	70 .	64	48	77	73	74	78	81	68	78	65
90	82	76	73	70	75 .	60	47	87	89	88	94	69	65	74	58
90	90	74	69	89	75 .	59	64	76	77	83	94	55	58	86	88
90	79	71	72	76	72 .	84	58	60	79	85	76	76	64	72	82
84	78	88	76	79	93 .	65	53	59	82	82	77	63	63	77	81
76	84	82	71	72	76 .	63	56	83	106	77	96	61	75	84	68
76	108	82	102	80	77 .	87	70	106	91	93	79	78	94	107	67
96	116	118	95	101	113 .	94	87	98	84	95	91	83	88	98	94
108	108	107	96	113	108 .	98	81	103	89	106	120	98	74	105	102
102	103	95	91	101	100 .	101	78	100	113	94	102	86	85	113	100
93	107	102	85	101	101 .	86	80	85	108	89	102	76	78	91	97
97	98	102	85	88	101 .	86	72	89	87	82	82	87	73	91	85
96	99	90	85	99	96 .	88	67	85	102	93	103	75	72	80	90
96	101	82	84	96	96 .	82	72	81	106	108	107	73	56	71	84
103	99	85	93	95	92 .	81	77	86	102	95	101	82	65	91	77
100	87	88	93	89	94 .	86	58	80	112	90	94	74	59	96	86
88	78	85	79	94	83 .	84	48	76	97	99	75	79	59	89	78
78	90	89	74	87	77 .	66	47	74	96	101	77	67	60	106	80
77	88	96	75	94	75 .	73	49	57	82	80	79	75	60	83	64
92	80	97	90	74	100 .	74	79	61	76	95	94	66	55	81	64
113	89	100	95	83	87 .	77	72	56	82	96	81	75	54	80	69
103	104	98	96	72	75 .	81	72	74	83	100	97	89	54	80	84
104	98	101	91	69	62 .	74	70	83	94	88	99	88	60	80	83
100	77	92	89	81	75 .	86	66	92	72	90	91	64	86	73	86
107	82	78	78	80	90 .	72	48	82	75	92	92	66	83	61	66
91	90	88	78	96	102 .	68	35	85	101	88	75	83	78	69	67
75	96	84	89	71	92 .	64	37	79	82	100	77	77	67	78	66
72	88	81	89	66	83 .	70	78	72	75	98	108	76	75	68	64
80	73	83	92	73	84 .	66	63	69	103	93	93	76	72	92	71
82	70	86	85	68	80 .	72	60	65	112	88	75	87	64	89	60
96	84	91	84	72	74 .	79	42	62	102	86	85	75	53	81	103
104	77	91	79	104	69 .	74	49	61	107	94	83	66	46	87	108
84	102	84	91	98	83 .	71	44	76	104	88	92	77	50	86	94
74	102	74	78	96	67 .	79	72	65	97	84	77	74	52	89	93
76	87	83	97	88	74 .	70	71	72	97	84	78	81	76	79	85
93	82	84	92	88	72 .	72	74	85	95	93	84	65	73	81	85
87	95	81	85	84	89 .	59	66	72	104	85	79	70	64	66	78
89	86	78	74	88	83 .	62	60	69	87	90	74	65	43	58	72
94	85	91	72	71	84 .	64	77	70	95	80	87	81	50	75	59
81	80	97	79	104	77 .	58	67	61	93	92	80	86	62	64	70
72	72	86	67	80	68 .	51	61	56	91	96	68	76	46	72	66
75	73	85	65	68	68 .	69	45	60	84	81	70	66	55	87	65
72	67	78	61	61	61 .	59	45	56	72	83	69	66	53	64	60
72	67	73	73	82	80 .	62	44	61	66	76	82	70	62	66	78
73	70	68	69	66	72 .	52	52	68	78	74	70	61	78	63	78
80	71	65	80	73	68 .	53	46	62	70	73	72	63	64	61	66
67	90	72	74	67	65 .	58	42	58	67	74	88	73	55	58	72
73	80	75	70	61	73 .	60	60	56	68	71	66	60	55	62	62
67	75	78	68	86	77 .	71	51	52	69	69	67	66	53	70	61
72	68	67	71	74	86 .	64	68	53	68	73	73	72	52	86	83
74	69	65	68	80	83 .	69	61	62	100	75	69	60	68	71	82
67	68	72	67	69	73 .	74	61	52	84	82	82	65	67	69	70
92	77	70	61	90	67 .	61	60	48	73	75	69	65	62	65	74
90	87	74	61	76	69 .	61	53	67	69	86	90	56	71	66	65
72	82	70	67	84	80 .	57	51	64	67	73	85	55	59	76	66
71	78	73	64	74	70 .	54	49	58	75	73	78	50	55	67	62
82	79	77	66	82	74 .	76	48	64	65	72	70	73	56	70	72
77	73	89	79	71	71 .	58	71	52	79	69	76	72	60	68	61
73	73	72	68	70	81 .	53	66	54	71	95	70	61	59	75	61
75	70	70	78	84	69 .	54	53	58	73	79	75	59	54	71	82
77	75	70	66	80	69 .	65	53	60	72	79	73	71	57	64	73
77	79	70	70	75	79 .	53	49	58	72	75	75	57	64	86	72
73	72	83	70	72	73 .	71	49	66	71	68	73	62	57	78	67
76	88	77	66	69	80 .	55	63	79	81	81	76	58	69	78	64
73	85	71	71	72	78 .	60	47	68	72	87	78	61	60	71	68
95	77	76	65	68	77 .	65	53	67	70	75	70	86	55	76	89
80	77	70	65	73	72 .	63	66	56	77	80	84	75	58	69	86
78	81	70	67	67	72 .	57	63	59	101	78	72	58	57	65	79
80	87	87	80	78	73 .	69	58	52	90	74	88	53	75	69	80
86	93	78	72	98	78 .	63	59	61	78	74	80	58	66	83	68
77	91	78	65	85	76 .	68	58	71	79	90	76	54	64	76	64
82	108	79	84	74	78 .	81	57	108	77	84	91	82	62	78	81
83	108	106	96	79	99 .	99	81	113	111	91	78	99	88	99	87
112	104	107	94	115	103 .	86	90	114	115	111	116	69	80	104	90
119	107	126	90	99	110 .	87	80	119	107	103	130	62	76	111	109

105	100	97	85	103	97 .	85	77	116	107	98	101	78	73	113	96
96	96	94	89	101	96 .	81	81	107	103	93	100	93	77	98	83
95	85	100	84	80	98 .	74	72	110	85	99	93	90	60	87	99
108	82	90	79	79	90 .	71	55	107	74	94	93	80	78	98	86
101	91	88	78	91	87 .	72	55	108	106	82	99	80	74	103	91
85	83	81	78	98	86 .	74	73	88	88	80	95	73	55	100	86
105	79	71	72	96	89 .	62	73	82	84	73	98	54	62	89	111
95	79	74	97	79	74 .	50	54	76	80	80	85	69	55	78	92
83	75	83	95	68	75 .	55	54	68	100	74	68	85	48	106	81
103	79	87	88	101	79 .	61	75	65	112	79	63	77	49	96	71
102	91	72	79	95	91 .	83	79	83	90	90	92	75	63	91	62
88	107	82	71	95	70 .	65	78	102	95	93	71	55	86	96	68
101	95	78	63	74	78 .	56	72	104	116	93	79	56	74	101	66
101	81	88	65	66	100 .	54	79	93	105	96	92	77	72	101	112
96	82	107	90	74	97 .	53	66	69	104	92	98	91	81	99	94
102	78	100	83	79	94 .	82	68	85	101	87	83	80	68	107	82
87	86	89	94	67	89 .	73	70	88	94	77	66	74	65	96	67
91	82	94	89	68	91 .	68	57	92	79	76	67	53	58	89	62
101	96	91	83	71	79 .	67	74	101	71	73	68	47	48	87	65
93	82	93	78	104	80 .	51	64	82	76	75	106	46	47	91	61
99	80	82	79	101	67 .	58	63	62	72	76	72	66	62	84	101
89	83	69	84	104	71 .	47	76	81	93	74	69	76	61	87	90
91	79	76	67	108	68 .	46	61	88	82	79	70	50	51	90	85
97	91	83	80	92	75 .	55	70	82	89	72	84	53	75	88	103
90	96	77	68	88	74 .	62	64	70	106	74	102	52	50	85	91
78	89	101	67	91	80 .	78	62	87	102	104	97	53	57	85	85
79	83	85	89	83	74 .	64	64	88	108	99	92	77	77	86	75
79	77	82	89	86	94 .	68	53	70	88	92	98	83	76	84	66
97	67	96	90	62	96 .	65	51	73	102	86	87	82	68	87	63
89	68	102	79	69	95 .	58	54	69	95	98	85	84	69	77	97
78	79	85	70	90	87 .	45	51	59	92	82	79	76	61	71	77
73	71	71	64	67	71 .	64	45	53	82	78	89	69	62	71	78
74	74	73	64	69	68 .	48	58	53	67	73	81	51	52	78	69
75	72	72	72	61	68 .	53	60	61	69	69	74	61	55	61	65
70	73	72	63	68	68 .	50	53	57	66	72	74	57	64	69	75
78	68	75	65	67	68 .	71	54	55	92	67	65	55	54	72	71
84	70	68	65	86	70 .	65	66	57	75	71	66	53	55	70	66
72	83	66	69	78	73 .	54	43	51	71	65	67	55	57	73	84
77	74	65	61	72	66 .	56	54	79	71	72	67	70	65	79	79
73	65	83	67	86	73 .	49	71	73	70	67	61	60	70	80	74
69	65	82	59	68	78 .	64	52	57	68	73	73	50	65	80	68
72	72	67	61	63	78 .	60	55	49	79	80	67	53	60	75	61
89	64	72	72	69	69 .	58	52	54	84	62	63	70	54	76	60
72	66	71	67	75	72 .	43	45	51	94	71	68	53	60	64	69
76	78	72	64	78	68 .	51	53	63	90	64	76	61	59	64	85
83	66	76	71	64	75 .	53	65	73	79	66	80	57	54	67	77
73	89	72	71	70	82	66	54	66	73	75	71	61	69	68	79
72	80	77	65	66	68	64	48	61	78	73	67	61	66	66	65
72	72	72	65	73	66	60	51	59	80	65	63	60	59	63	69
74	72	79	66	64	78	62	62	57	77	66	74	61	61	79	78
101	72	69	78	68	71	69	66	54	71	68	88	63	67	75	71
90	100	90	94	63	74	56	52	52	102	66	72	57	57	74	69
72	76	71	77	76	81	55	47	68	74	63	68	72	54	71	70
80	70	67	83	78	69	60	46	56	77	89	73	72	61	69	76
80	82	77	77	73	74	48	53	52	89	73	73	60	83	74	70
77	74	71	68	70	67	75	48	58	83	63	91	54	65	64	91
72	72	72	75	74	72	86	75	60	77	66	69	90	67	67	78
72	69	76	76	70	82	69	76	58	78	68	79	70	73	68	66
76	76	76	77	98	74	73	73	68	86	67	73	75	67	75	65
72	89	71	78	87	76	69	59	71	95	72	77	67	74	73	66
82	71	82	71	69	77	66	55	64	78	67	84	63	80	83	92
77	94	95	73	75	87	72	54	67	90	74	74	67	86	69	76
81	109	93	95	81	99	99	83	100	90	71	81	66	73	93	72
112	107	107	95	111	112	95	85	110	123	111	114	91	95	98	77
113	100	104	91	99	128	91	76	110	126	105	113	89	83	97	94
100	94	114	77	80	104	84	69	103	107	101	116	88	82	91	108
98	90	101	85	75	99	77	67	86	114	98	106	77	86	80	94
85	83	95	84	93	112	67	66	90	114	104	117	74	70	73	100
78	99	77	84	111	98	72	73	77	98	93	88	76	67	75	88
107	95	79	84	97	109	66	70	78	114	84	97	62	62	77	93
106	85	90	72	91	108	68	54	78	95	68	89	77	61	89	93
94	76	94	75	90	96	66	54	94	89	102	86	86	66	95	92
76	71	85	83	74	87	72	52	84	86	91	73	60	76	84	85
69	72	77	74	74	81	66	48	78	86	81	74	49	56	85	78
86	72	71	71	96	80	58	68	65	87	67	66	65	58	72	72
106	88	76	70	111	74	50	79	53	88	70	70	55	55	87	83
103	102	125	89	107	87	59	71	51	83	73	114	51	56	79	99
98	99	92	92	98	96	62	67	62	103	69	101	67	84	67	80
76	98	80	81	98	90	61	48	90	115	110	102	90	73	74	90
70	87	75	88	92	98	84	49	84	88	99	85	75	73	73	101
79	76	89	76	80	84	78	48	76	84	97	80	81	73	63	89
75	61	80	81	91	69	67	46	74	75	87	68	70	56	63	92
72	65	83	82	98	87	67	37	73	116	76	67	60	50	82	81
101	61	76	88	96	76	54	77	66	112	62	96	68	50	89	89
100	101	82	88	90	85	66	72	61	113	63	89	44	50	81	89
105	93	79	75	82	78	54	60	53	103	58	88	45	49	80	87
92	97	76	70	93	83	57	62	51	105	99	62	74	68	77	92
91	82	104	71	85	104	53	41	63	77	97	65	50	73	78	88
108	73	87	80	102	81	55	41	80	73	81	66	49	78	85	89
91	63	93	61	86	64	60	58	73	107	93	67	51	69	68	81
71	72	92	69	103	69	77	69	79	103	77	67	71	72	77	84
72	100	92	83	82	71	80	66	63	98	62	95	52	69	68	69
68	91	90	74	87	98	79	69	68	98	62	102	76	51	69	83
68	86	85	73	79	103	72	66	57	91	65	84	70	51	63	81
93	82	81	81	91	90	55	73	44	99	60	104	66	73	61	72
86	75	81	73	115	87	57	69	52	92	80	80	67	70	63	80
82	82	86	70	85	78	68	56	49	70	85	85	60	59	61	76
75	74	84	72	95	68	65	42	51	90	80	73	47	55	69	74
71	62	65	69	73	74	50	40	75	77	68	70	47	49	80	75
69	65	76	69	72	71	69	40	66	81	62	81	43	70	70	64
71	64	77	85	75	75	63	43	54	89	55	84	44	66	71	75
83	83	70	70	64	67	57	71	49	72	58	66	69	53	61	64
70	63	71	70	74	71	53	64	52	72	73	67	52	55	65	68
77	76	77	70	79	68	61	48	59	75	74	74	47	68	70	73
65	66	77	66	71	72	61	45	53	84	57	67	49	63	80	86

71	63	78	71	73	78	59	48	80	79	55	72	56	55	66	86
73	66	76	65	79	68	55	43	67	78	55	72	45	54	64	65
72	78	77	73	73	73	65	58	58	78	65	63	48	60	63	66
64	81	76	66	67	86	55	48	51	80	74	69	56	54	69	60
70	79	79	67	68	70	59	47	67	96	66	88	50	68	74	69
															83
															76
															63

10	10	12	12	12	12	3	3	4	4	5	7	7	8	10	10	4	4	5
Box-5	Box-4	Box-1	Box-2	Box-5	Box-6	Box-1	Box-2	Box-6	Box-7	Box-6	Box-2	Box-5	Box-3	Box-1	Box-3	Box-1	Box-4	Box-5
63	69	74	76	81	83	1.7	2.3	25.3	26.6	34.6	38	43.6	52.6	64	75	13.3	21.5	32.6
F	F	F	F	F	F	F	F	F	F	F	F	F	F	F	F	F	F	F
H2O	H2O	H2O	H2O	H2O	H2O	Malto	Malto	Malto	Malto	Malto	Malto	Malto	Malto	Malto	Malto	MCT	MCT	MCT
FH	FH	FH	FH	FH	FH	FM	FM	FM	FM	FM	FM	FM	FM	FM	FM	FT	FT	FT
48	61	67	65	79	62	96	61	85	61	64	46 .		37	42	66	53	61	54
78	96	84	75	68	57	97	77	60	59	92	46 .		71	88	52	49	59	89
83	89	67	66	68	62	90	104	60	60	72	65 .		55	54	41	80	82	60
71	65	69	68	112	106	56	93	66	90	43	42 .		38	53	65	74	98	63
95	67	95	94	96	91	53	64	88	84	79	45 .		40	89	64	49	78	54
68	91	97	96	62	86	89	99	84	63	76	82 .		39	90	64	83	60	96
51	90	80	68	102	73	101	98	56	78	87	87 .		71	76	59	70	66	87
86	75	68	79	98	65	102	107	80	94	85	55 .		60	65	74	75	79	52
100	73	63	99	84	97	103	104	85	75	72	71 .		66	83	76	43	59	81
109	87	100	98	111	99	87	107	63	58	67	80 .		43	96	73	35	72	56
78	87	98	83	107	84	53	108	60	85	79	86 .		58	93	71	35	99	53
58	56	98	65	98	89	52	110	101	91	93	87 .		32	92	70	80	76	94
52	54	108	101	106	58	86	84	77	65	86	94 .		53	110	66	73	94	71
88	88	100	117	124	61	104	91	58	91	77	94 .		34	112	63	57	72	51
97	82	100	101	125	92	74	94	52	103	51	94 .		66	116	75	63	58	85
73	87	116	109	118	98	51	71	55	103	47	92 .		56	115	91	57	55	97
90	86	113	106	129	117	56	70	92	91	47	84 .		26	71	100	34	96	68
99	92	119	90	131	121	54	107	102	95	64	47 .		63	54	100	16	106	98
70	69	118	102	129	127	92	105	69	102	71	37 .		62	80	97	72	98	94
80	43	115	110	117	130	99	112	52	96	52	38 .		57	105	98	61	103	101
75	46	111	105	115	118	102	112	52	94	70	73 .		76	111	101	31	103	74
63	49	117	63	114	100	98	108	103	57	91	89 .		54	108	90	17	97	56
51	88	120	82	106	121	100	107	89	54	81	83 .		33	90	64	11	83	50
47	80	108	58	91	111	104	88	99	84	82	62 .		53	64	42	7	56	90
82	63	104	59	75	71	103	59	60	65	76	73 .		41	58	38	27	51	96
66	38	78	61	69	57	106	53	45	56	70	79 .		72	66	43	82	67	101
46	34	58	58	76	58	104	77	49	96	47	71 .		75	105	46	65	67	90
48	84	65	67	77	75	80	92	74	98	48	38 .		75	76	87	32	86	63
82	71	59	94	80	71	54	64	61	90	74	39 .		84	62	95	19	110	52
77	71	87	84	109	64	52	63	95	72	56	41 .		88	62	60	18	98	55
63	38	69	86	116	105	49	92	61	53	51	55 .		45	94	41	10	68	82
47	32	94	52	106	113	51	66	46	83	51	41 .		30	76	41	7	49	66
79	33	104	50	80	117	94	59	46	76	77	43 .		29	82	41	30	55	58
74	88	99	88	90	118	100	83	103	92	54	43 .		63	93	86	82	61	89
52	72	69	62	79	105	88	99	100	89	54	68 .		52	83	77	60	96	99
48	52	53	46	97	67	62	93	65	61	65	84 .		37	59	42	26	97	79
59	33	57	51	116	63	37	78	47	48	88	89 .		22	50	36	14	77	51
81	30	57	53	111	91	45	63	52	54	66	81 .		20	52	36	8	58	89
74	39	59	60	101	111	44	59	93	63	54	48 .		23	95	42	6	55	78
68	56	108	109	85	75	48	58	64	68	81	41 .		34	80	84	3	57	55
48	69	106	94	55	62	46	61	50	95	74	41 .		67	77	80	17	107	50
46	45	99	86	64	64	52	56	61	82	56	66 .		41	46	83	84	101	57
63	43	88	54	64	69	51	57	53	87	60	84 .		19	42	52	67	69	56
56	56	59	63	71	99	74	74	95	68	56	73 .		21	65	45	48	51	45
52	51	58	51	65	91	81	83	93	50	56	46 .		45	55	47	29	56	74
56	51	58	94	76	65	52	57	90	54	85	45 .		34	54	57	19	67	87
52	49	54	65	63	68	51	59	74	51	78	56 .		32	69	47	12	55	60
52	48	90	54	96	66	52	70	57	69	59	72 .		36	52	46	10	86	53
76	63	85	56	100	82	56	63	55	79	59	43 .		32	55	49	30	87	53
74	47	59	90	67	58	60	57	74	55	58	43 .		28	54	87	43	79	53
55	45	59	94	70	77	45	77	87	56	76	72 .		30	51	65	54	54	53
48	48	59	59	101	94	52	85	56	53	64	76 .		63	88	40	67	51	86
56	49	58	52	88	70	47	81	62	58	59	46 .		57	79	50	48	61	85
47	52	79	85	65	66	48	64	59	79	59	42 .		28	47	78	24	51	58
65	47	59	65	59	58	69	65	56	70	67	44 .		34	50	87	18	58	51
71	47	61	59	65	61	49	59	58	57	53	78 .		33	46	80	13	90	55
50	65	61	72	90	76	50	75	92	56	54	59 .		52	48	53	10	63	81
49	72	60	59	96	64	53	59	88	95	76	43 .		31	47	45	20	56	85
46	49	91	63	103	95	55	76	68	79	56	42 .		32	73	49	9	57	67
48	53	95	99	96	75	55	65	57	55	53	44 .		54	49	63	14	83	51
89	48	74	89	81	71	90	58	57	56	50	48 .		69	55	80	21	55	59
84	51	59	59	68	69	99	59	98	56	71	78 .		75	54	64	47	79	56
54	80	59	59	65	65	100	83	73	63	58	79 .		39	75	42	87	91	52
50	75	55	91	95	69	87	88	55	98	58	41 .		35	71	45	92	71	86
46	43	84	75	66	64	60	79	62	68	55	59 .		35	44	74	80	54	74
85	52	94	58	66	77	50	62	60	56	71	80 .		32	51	69	49	58	51
86	78	64	58	68	104	61	61	93	86	73	54 .		41	52	45	44	63	54
86	68	60	98	101	108	57	99	110	63	82	49 .		75	87	51	85	95	86
58	74	64	96	99	81	61	92	102	58	86	48 .		64	104	84	93	68	90
73	72	90	79	95	71	94	80	69	89	74	59 .		43	95	68	90	65	92
92	47	94	113	71	76	99	66	95	104	74	88 .		72	64	53	89	76	93
96	73	64	93	118	70	101	91	101	97	75	82 .		44	61	83	91	65	68
88	75	91	81	102	70	70	104	116	103	65	62 .		79	75	79	69	91	83
88	82	103	106	112	101	63	104	113	110	78	74 .		88	71	51	61	68	97
92	76	93	100	95	98	94	103	108	74	89	87 .		73	59	76	79	92	68
102	68	86	101	116	107	75	105	105	92	83	84 .		75	106	82	57	91	58
69	43	98	113	117	107	62	97	99	83	79	49 .		72	98	75	44	66	89
47	44	69	101	104	109	82	99	98	56	85	66 .		80	77	76	49	96	102
63	55	70	105	121	99	97	113	93	77	77	82 .		82	63	62	68	72	93
102	76	100	75	94	79	109	94	97	91	81	86 .		70	89	54	80	55	100
96	80	101	83	73	71	88	58	96	60	78	90 .		65	106	47	83	78	89
92	100	93	97	89	75	60	86	102	49	75	84 .		66	102	67	72	91	100
83	107	76	118	120	92	59	86	100	75	61	84 .		68	104	92	83	75	97
83	111	65	111	123	94	94	79	90	98	65	78 .		64	112	89	88	65	88
79	103	105	104	124	74	107	77	60	97	71	50 .		42	107	89	82	84	78
82	109	109	69	89	101	78	62	56	79	83	44 .		39	103	86	77	64	50
69	87	109	87	116	123	62	108	90	51	81	56 .		39	100	88	77	83	52
41	55	109	82	112	110	67	101	60	81	77	75 .		65	102	77	47	100	50
43	75	106	96	81	110	54	103	59	96	60	83 .		78	87	77	42	97	56
41	82	104	82	106	110	90	100	74	72	66	49 .		73	60	76	46	103	95
66	55	106	63	82	104	104	107	63	56	65	45 .		57	57	50	70	93	91
60	54	99	59	70	98	105	96	59	50	68	74 .		41	76	41	60	92	90
51	73	82	100	86	106	106	67	66	53	66	65 .		38	64	50	48	89	65
48	98	58	99	103	86	102	56	95	61	75	45 .		42	59	47	60	61	49
48	85	56	100	80	71	104	58	96	53	68	54 .		81	56	61	44	55	59
80	61	57	100	68	69	97	85	76	50	66	44 .		71	82	77	43	57	82
54	48	64	93	73	70	90	68	58	96	73	70 .		58	100	67	68	76	78
78	70	81	64	74	69	82	58	55	99	59	73 .		53	88	48	55	54	54

56	102	59	58	104	86	59	83	76	99	81	76 .	72	63	46	77	58	47
42	88	58	64	110	74	71	60	95	95	80	66 .	76	60	42	78	94	51
48	61	81	86	75	76	49	58	78	98	67	42 .	54	58	52	71	72	92
67	46	87	69	100	111	59	88	53	68	56	41 .	37	88	42	52	53	85
79	89	61	61	109	76	47	64	53	50	53	60 .	36	91	76	46	95	76
57	84	65	69	99	63	73	79	52	54	46	49 .	43	65	76	47	94	53
43	61	53	99	85	64	49	60	94	54	54	74 .	39	56	49	49	74	49
45	48	65	88	64	99	49	57	88	83	78	53 .	50	56	39	51	53	83
45	51	105	56	75	94	56	73	64	86	67	41 .	39	57	41	48	74	82
55	83	92	58	66	81	87	96	49	54	56	41 .	72	81	71	48	73	54
52	71	64	59	70	65	64	61	73	54	72	44 .	46	93	71	85	67	48
66	57	56	88	110	64	54	57	84	80	78	42 .	50	80	48	80	61	62
56	68	59	88	86	77	58	78	60	96	57	72 .	45	72	66	57	65	79
49	55	57	58	64	58	55	67	57	66	59	89 .	42	59	66	49	79	61
49	53	61	60	70	59	82	61	57	52	73	77 .	56	85	43	77	57	50
50	65	57	60	70	77	78	67	77	53	61	65 .	59	73	76	60	59	46
67	54	88	82	71	85	52	81	56	87	55	43 .	41	54	81	51	82	84
49	54	67	70	63	61	52	60	94	74	78	44 .	41	60	52	52	74	74
48	52	58	58	81	61	79	60	62	55	66	44 .	40	64	50	46	51	50
53	93	82	62	63	66	56	85	57	53		42 .	62	53	70	75	79	45
47	65	63	98	66	72	53	61	66	56		60 .	64	71	44	62	78	48
47	49	62	89	78	60	57	64	57	55		42 .	38	52	46	50	56	70
78	51	62	65	101	61	56	61	87	86		45 .	57	60	47	43	57	51
60	47	93	58	69	95	56	60	92	98		43 .	43	65	75	79	55	47
43	59	90	59	67	61	51	87	92	88		79 .	46	90	70	75	53	83
61	57	82	60	102	61	57	84	92	54		71 .	45	78	46	56	82	80
76	78	58	92	104	65	88	60	70	52		46 .	68	70	47	77	58	49
49	47	56	89	95	77	70	62	56	57		45 .	43	53	62	61	56	42
46	46	68	93	69	82	49	60	56	54		43 .	40	73	46	51	62	67
72	54	62	93	71	83	54	86	61	62		78 .	45	53	46	50	78	84
45	89	59	69	68	63	84	85	57	98		55 .	43	52	78	82	52	84
45	86	92	60	88	69	90	61	61	99		42 .	68	58	79	71	62	62
73	51	69	57	103	63	59	58	86	86		48 .	43	98	51	50	65	51
70	89	58	80	73	98	66	77	97	60		78 .	52	92	44	75	52	70
81	76	75	61	74	88	81	95	104	58		79 .	77	60	64	92	90	91
84	75	66	64	88	76	63	70	117	62		46 .	49	61	76	95	82	96
67	59	83	99	118	75	57	79	113	102		53 .	81	96	94	96	60	92
75	81	98	104	102	111	86	96	93	104		78 .	85	95	61	102	85	93
85	98	81	105	73	98	103	99	59	103		86 .	88	66	87	89	66	99
95	65	105	95	118	101	103	99	102	97		75 .	75	71	91	92	95	104
77	81	81	106	105	107	85	66	108	98		43 .	69	92	59	90	90	93
48	59	64	104	114	108	67	77	98	65		82 .	80	79	72	87	69	99
85	59	96	106	112	93	67	73	91	96		78 .	86	62	95	96	84	109
65	82	94	86	87	104	63	78	91	75		71 .	84	82	85	90	96	97
53	84	95	86	70	101	99	75	67	55		41 .	73	100	53	78	96	91
86	62	65	89	70	85	105	89	58	57		49 .	77	70	77	51	96	91
81	63	100	106	71	94	86	116	98	105		72 .	85	76	92	47	104	73
80	84	100	109	87	86	67	100	105	101		81 .	79	109	78	58	93	49
86	77	87	106	70	76	63	71	93	101		80 .	88	80	74	48	89	51
83	80	88	106	116	123	66	105	87	101		50 .	74	62	77	63	67	57
87	89	111	91	110	117	60	99	55	89		38 .	78	96	91	68	81	97
83	99	105	85	116	121	77	92	57	64		69 .	72	102	59	74	90	98
78	91	100	69	116	117	106	94	76	45		85 .	71	77	43	74	67	96
74	87	103	87	129	117	112	79	97	56		60 .	65	105	61	46	60	92
78	75	104	81	115	118	108	99	91	55		34 .	66	114	50	63	79	73
75	78	93	86	110	111	100	71	95	68		40 .	45	114	45	53	60	50
76	87	85	99	104	83	98	77	73	100		47 .	37	104	57	47	67	43
45	77	56	85	109	70	95	79	56	90		38 .	49	91	46	78	89	52
48	55	57	64	118	75	71	74	60	55		64 .	40	65	85	87	62	54
61	80	59	61	86	68	53	86	56	50		72 .	40	56	67	88	73	51
46	68	74	68	62	103	74	102	65	52		80 .	76	58	45	86	79	80
63	61	70	69	68	105	99	92	56	74		64 .	79	58	40	69	97	88
69	91	74	86	66	79	88	85	91	52		38 .	64	93	44	47	103	62
49	91	64	89	70	68	64	61	80	52		41 .	62	82	70	76	86	47
53	80	52	77	66	70	48	78	55	52		48 .	70	59	86	76	57	46
51	95	49	61	56	96	80	67	53	62		41 .	51	51	81	51	56	79
47	81	43	56	67	117	85	66	102	97		37 .	65	57	82	46	56	91
48	77	51	94	119	88	85	98	92	92		81 .	68	70	69	70	100	82
82	57	108	104	107	76	77	78	80	93		87 .	57	81	42	47	84	52
73	58	107	93	66	66	50	61	71	88		63 .	34	100	41	46	54	46
51	83	87	95	56	71	49	73	50	68		38 .	39	70	48	71	55	52
47	81	65	70	81	60	52	72	56	51		44 .	39	58	43	48	88	87
54	59	50	54	111	76	84	62	93	46		42 .	50	52	59	49	87	70
67	60	52	83	89	70	56	57	71	66		71 .	55	85	74	49	58	50
46	63	87	76	67	70	56	71	50	91		76 .	39	89	60	77	55	45
50	59	64	57	72	65	56	72	72	56		73 .	57	58	48	77	68	65
49	76	57	59	69	67	56	60	58	54		49 .	49	55	41	52	69	50
47	59	58	82	71	87	52	84	63	57		36 .	41	49	63	49	55	49
81	63	66	87	71	75	56	63	90	86		52 .	54	61	49	53	61	57
71	58	91	59	88	75	76	58	82	58		39 .	52	94	67	82	87	92
47	57	60	87	111	71	75	82	53	56		43 .	44	89	77	80	78	75
50	64	57	66	72	71	57	87	53	54		61 .	42	60	70	57	52	49
55	54	98	107	88	106	104	76	93	120	77	68 .	76	82	105	69	92	68
54	60	82	98	109	83	94	77	92	119	81	74 .	65	56	85	87	84	86
84	106	95	91	86	70	96	86	105	104	82	54 .	54	55	57	74	77	98
84	71	70	91	112	112	88	91	76	101	70	51 .	73	61	76	70	63	70
84	91	77	75	83	86	72	73	78	102	83	46 .	66	101	58	78	71	54
73	94	98	75	78	116	84	85	96	88	70	77 .	53	113	75	70	98	91
59	58	83	106	111	87	69	79	76	66	76	74 .	64	107	91	75	78	92
82	57	75	75	93	80	72	65	74	67	77	47 .	77	83	61	98	92	64
76	61	88	76	81	81	100	84	96	79	70	48 .	72	62	57	74	90	61
60	59	80	104	109	112	74	72	73	69	69	58 .	57	79	59	64	72	62
91	62	99	82	86	110	73	63	92	90	68	76 .	51	116	62	69	70	91
69	62	83	72	86	86	82	81	76	83	74	54 .	66	87	80	68	91	68
63	74	82	103	97	87	73	71	95	71	75	82 .	54	62	77	69	71	73
72	100	89	105	88	99	81	73	114	80	89	87 .	52	99	64	99	93	81
80	75	95	89	87	116	108	96	99	102	88	82 .	80	87	68	99	96	73
99	85	78	80	119	124	105	91	102	84	82	65 .	87	89	76	101	96	88
87	105	87	110	100	97	91	71	113	79	84	53 .	82	87	88	102	91	96
100	93	88	122	99	120	87	84	107	75	86	83 .	94	111	77	103	104	95
88	87	105	92	91	98	95	90	100	101	74	87 .	88	109	86	99	103	79
67	84	94	91	88	104	84	81	98	107	70	53 .	83	82	85	93	101	89
92	68	103	110	108	117	101	94	105	84	77	49 .	83	96	68	87	108	95
75	61	101	105	109	116	97	74	92	94	79	62 .	83	92	69	87	94	99

78	90	94	103	88	117	76	64	76	76	79	54 .		71	96	59	77	81	76
69	89	83	114	86	120	74	80	98	65	92	52 .		54	103	57	82	93	58
72	78	81	115	92	117	74	96	95	65	82	75 .		63	105	65	101	94	99
77	61	77	92	119	119	100	66	93	87	78	80 .		78	89	83	103	69	89
85	66	89	108	103	116	94	65	107	76	72	87 .		86	102	84	106	97	63
80	90	86	103	119	108	102	59	105	64	72	78 .		84	82	69	92	100	89
71	96	81	85	121	109	81	63	77	64	68	80 .		79	95	96	90	80	94
88	96	86	87	88	101	92	92	97	62	83	89 .		73	91	86	74	73	65
84	90	81	103	119	102	110	71	95	105	79	79 .		89	77	85	70	71	64
72	99	104	91	120	96	94	64	89	99	65	76 .		79	86	85	71	64	71
73	72	110	114	97	109	86	82	79	92	66	63 .		76	102	90	72	72	82
68	59	109	110	91	104	100	88	90	74	77	53 .		61	107	78	83	70	61
64	83	115	101	90	102	98	85	101	64	65	49 .		59	86	84	89	73	61
73	100	96	109	107	103	75	93	84	60	58	47 .		82	72	55	66	90	58
69	74	99	92	96	115	93	93	74	91	69	52 .		76	79	86	60	79	64
86	62	79	80	115	107	104	89	81	85	91	47 .		79	78	84	78	63	70
69	61	79	81	99	115	95	87	82	63	70	82 .		77	72	69	67	64	95
85	66	86	89	103	96	102	81	92	82	58	76 .		78	97	62	91	87	97
81	65	80	86	98	92	84	91	76	70	76	48 .		78	76	59	85	76	93
66	91	81	79	115	93	75	75	82	70	81	59 .		65	80	57	67	94	95
64	68	102	78	90	103	77	90	74	73	65	82 .		61	96	62	59	87	91
68	76	91	75	101	95	77	89	66	62	62	62 .		73	95	60	55	92	80
79	61	71	79	113	86	76	73	100	93	69	81 .		74	72	74	84	105	90
77	78	68	103	114	84	72	62	97	99	80	55 .		55	67	83	86	98	81
77	77	68	75	87	93	82	61	94	92	58	49 .		57	79	65	71	93	65
65	84	69	94	79	107	76	60	76	93	79	53 .		62	95	73	61	87	60
87	85	63	98	80	87	91	53	66	66	64	49 .		56	71	56	82	62	61
62	62	64	103	91	91	74	60	91	62	63	65 .		56	66	55	67	85	77
65	100	95	85	101	87	72	78	81	57	78	73 .		69	69	72	59	65	81
70	67	79	75	84	103	84	63	78	76	65	61 .		64	69	58	72	65	64
58	97	83	77	84	84	78	60	68	63	63	56 .		66	89	58	67	82	64
61	72	72	110	80	84	72	62	72	55	62	51 .		64	64	93	62	69	65
63	66	84	78	77	85	66	85	68	81	65	60 .		61	64	81	81	63	65
60	67	97	77	94	97	67	83	79	62	58	54 .		64	106	63	71	89	60
75	63	78	100	84	86	97	68	87	63	80	64 .		61	69	67	77	87	87
61	77	70	85	90	86	96	61	72	60	64	55 .		60	64	60	82	71	87
62	66	84	74	94	83	72	61	67	87	65	55 .		62	81	65	77	68	63
63	87	75	75	92	93	66	67	65	68	66	54 .		53	91	82	64	96	63
70	78	73	82	76	87	69	61	97	75	92	66 .		60	85	56	59	92	60
60	69	85	77	79	80	73	78	78	64	76	72 .		67	66	58	76	71	70
76	63	72	83	84	101	65	62	71	57	68	82 .		55	73	76	68	67	83
69	58	83	75	76	91	67	67	71	93	63	57 .		52	76	62	67	68	64
60	89	80	68	82	80	78	61	84	70	84	53 .		69	78	76	87	64	65
59	65	71	91	77	83	83	65	99	62	70	52 .		58	69	86	81	81	61
74	64	93	69	83	79	67	67	79	66	69	52 .		62	101	58	62	88	61
69	61	109	80	108	86	89	89	79	82	77	66 .		58	71	64	60	76	87
64	75	102	80	106	95	66	83	77	64	65	80 .		51	69	72	54	72	66
58	92	83	98	82	87	66	76	73	88	65	67 .		64	99	81	91	86	66
87	87	77	101	82	87	67	62	99	105	61	67 .		52	94	63	86	72	68
67	61	84	80	79	88	92	62	76	94	66	57 .		57	69	73	62	71	72
56	63	71	85	80	81	95	59	72	65	89	57 .		67	73	68	60	83	61
79	63	83	75	108	85	101	85	70	85	66	80 .		69	114	60	74	78	73
78	93	74	85	89	102	76	76	98	69	69	97 .		55	93	86	96	71	97
63	73	93	93	87	84	66	61	81	69	61	65 .		55	79	62	71	87	75
65	62	106	85	108	87	70	67	78	60	71	57 .		76	79	60	68	90	67
67	65	84	83	116	93	89	95	94	106	65	78 .		71	82	63	86	72	66
92	97	82	93	108	105	105	100	112	97	88	98 .		64	99	66	113	95	108
75	92	110	115	125	110	109	85	112	102	93	94 .		91	91	81	99	105	108
74	92	102	114	117	119	89	99	103	92	87	83 .		80	113	89	113	101	92
85	105	121	101	97	109	122	106	108	107	89	88 .		80	107	90	104	100	104
76	101	107	103	96	108	100	100	109	94	80	81 .		85	94	91	102	100	88
59	89	103	97	92	107	84	103	97	84	89	87 .		78	93	69	99	89	99
82	76	93	116	93	114	74	93	95	61	81	68 .		77	115	67	90	89	77
75	81	101	100	104	102	97	86	99	78	78	90 .		82	110	76	86	75	66
76	92	94	103	97	111	92	92	82	77	77	90 .		80	101	96	82	85	89
66	94	99	109	85	108	79	93	107	65	62	80 .		78	122	89	83	77	82
72	73	106	103	87	108	86	88	119	66	76	53 .		74	117	90	88	69	70
86	81	114	99	115	109	73	68	105	104	58	49 .		61	116	89	64	70	75
83	89	112	104	125	96	79	64	83	96	55	47 .		45	114	72	68	112	88
82	88	115	109	98	121	97	88 .		79	79	59 .		75	104	63	104	93	100
80	94	120	108	103	123	112	93 .		61	86	89 .		74	107	83	99	94	101
80	79	96	113	96	111	97	69 .		69	71	83 .		51	102	66	96	107	99
78	77	113	110	125	102	99	63 .		87	59	75 .		66	106	76	90	102	84
77	98	109	101	117	99	81	58 .		103	66	85 .		71	103	70	72	96	99
64	96	101	100	89	112	63	57 .		101	87	71 .		73	89	68	70	93	84
52	89	110	86	94	111	60	73 .		75	83	48 .		67	83	76	93	91	71
55	84	98	89	99	109	87	63 .		67	76	66 .		59	85	70	72	99	76
66	89	95	85	91	98	105	57 .		68	76	48 .		67	80	79	75	72	73
80	98	90	89	115	105	103	66 .		**66**	83	67 .		72	88	87	89	**73**	**98**
74	89	91	90	91	110	95	63 .		**68**	73	51 .		56	83	96	71	**72**	**88**
73	66	101	101	95	111	74	89 .		**65**	65	48 .		77	95	66	80	**69**	**72**
58	96	88	97	88	98	75	98 .		**74**	73	49 .		60	93	69	79	**90**	**71**
67	78	96	99	89	97	94	95 .		**63**	82	62 .		66	89	80	69	**73**	**90**
67	71	84	93	91	95	110	83 .		**104**	59	74 .		59	82	91	76	**84**	**76**
65	64	85	90	92	86	94	87 .		**86**	63	48 .		53	79	73	100	**66**	**68**
85	69	86	84	98	84	67	73 .		**69**	63	47 .		67	91	64	85	**79**	**100**
79	97	96	93	92	92	59	54 .		65	68	44 .		67	74	68	84	68	86
80	89	77	105	85	91	57	55 .		62	67	46 .		54	80	63	70	73	85
74	87	105	103	108	97	74	54 .		65	86	87 .		67	75	83	66	93	89
70	62	105	88	115	91	89	95 .		60	69	80 .		51	88	65	64	87	72
68	64	90	85	99	94	61	87 .		90	70	79 .		48	75	61	70	65	67
59	62	80	92	90	88	58	74 .		100	54	71 .		65	94	55	73	93	62
57	65	86	84	83	77	76	58 .		74	54	49 .		52	87	59	68	72	64
79	72	80	82	96	75	72	52 .		60	62	48 .		53	74	59	81	63	64
61	61	77	73	88	79	64	66 .		64	79	47 .		47	98	63	71	64	80
58	67	93	96	82	94	105	58 .		59	54	63 .		58	69	69	64	95	64
53	71	74	98	88	87	91	56 .		73	54	78 .		47	67	59	78	76	60
57	63	80	77	95	79	68	55 .		58	62	53 .		66	87	58	73	63	84
73	97	70	78	85	81	70	81 .		60	55	51 .		75	72	61	65	87	76
56	82	71	69	75	80	67	60 .		103	75	73 .		51	84	71	79	71	66
58	63	93	89	76	94	78	56 .		76	67	65 .		51	70	74	83	62	79
72	67	75	92	72	79	73	62 .		65	62	50 .		71	76	67	71	69	91
64	58	87	78	76	76	71	86 .		66	56	52 .		50	65	62	67	61	64
60	59	74	75	82	76	73	64 .		64	79	50 .		49	68	67	69	65	65
59	66	76	94	76	101	61	55 .		86	63	49 .		48	93	65	73	79	63

77	65	81	75	74	94	63	82 .		75	62	49 .	44	100	71	64	84	64
58	69	74	86	72	88	61	64 .		79	50	54 .	61	66	60	94	72	59
60	66	88	76	79	75	61	61 .		64	31	89 .	49	68	70	89	65	58
69	62	92	85	73	79	81	59 .		66		85 .	46	95	60	81	70	60
61	60	82	74	70	81	75	57 .		95		76 .	61	85	73	76	65	84
57	60	67	81	76	87	65	60 .		92		59 .	50	67	65	76	77	85
83	95	91	76	91	81	92	84 .		70		56 .	75	63	64	80	65	63
81	77	81	81	97	83	68	86 .		65		74 .	74	83	64	77	67	61
57	65	79	75	79	98	70	70 .		83		84 .	51	65	74	82	68	74
58	68	88	100	71	80	81	91 .		104		55 .	46	97	78	92	90	84
59	61	77	74	68	80	97	86 .		94		55 .	46	113	89	90	68	66
76	96	79	92	81	78	87	66 .		73		78 .	59	87	84	68	66	60
71	83	70	92	71	101	74	63 .		68		80 .	53	104	65	67	65	59
61	66	76	81	71	83	70	69 .		67		59 .	47	102	65	69	98	75
57	76	88	78	72	83	75	70 .		66		61 .	49	83	67	69	77	84
58	75	82	81	89	83	87	68 .		100		93 .	50	74	75	83	72	65
84	106	75	78	84	104	69	92 .		106		104 .	80	109	65	91	80	64
82	93	100	107	86	97	100	97 .		106		92 .	84	96	64	117	88	103
64	103	108	117	113	89	109	93 .		96		87 .	74	106	89	112	85	94
87	86	113	106	102	98	115	100 .		106		87 .	77	114	107	103	99	76
82	88	102	93	98	115	103	87 .		87		86 .	57	112	76	73	91	95
64	89	86	118	83	95	95	82 .		75		72 .	62	99	88	87	89	89
80	105	107	108	85	101	97	83 .		87		56 .	67	109	81	86	104	81
92	98	106	118	73	114	94	90 .		83		80 .	46	103	74	94	82	77
79	92	92	83	79	106	94	85 .		73		94 .	44	103	81	72	92	85
62	84	100	105	111	94	82	65 .		67		84 .	73	90	96	78	80	77
57	81	82	85	86	87	65	94 .		103		79 .	79	88	79	83	110	85
55	90	94	81	76	80	77	93 .		98		60 .	64	112	68	76	97	63
84	90	107	88	82	77	108	87 .		94		50 .	67	116	84	73	108	74
84	78	101	109	85	80	104	71 .		87		51 .	69	114	111	96	97	92
79	88	83	88	91	86	72	59 .		75		95 .	68	124	95	93	91	87
85	99	87	106	104	99	86	59 .		86		83 .	65	113	90	97	89	85
79	99	116	99	106	114	81	90 .		98		72 .	59	117	87	86	82	74
72	99	105	101	121	109	68	70	88	71		55 .	66	103	74	72	76	59
78	97	99	90	114	80	66	67	79	68		47 .	49	104	75	67	89	58
69	90	96	73	109	97	65	59	76	88		51 .	44	84	85	83	97	58
79	84	79	65	90	93	95	62	88	70		48 .	39	66	75	61	74	76
74	73	66	67	76	105	93	86	84	88		60 .	69	61	71	79	87	93
75	71	71	90	73	98	94	95	88	88		51 .	63	101	75	80	87	90
70	72	64	72	72	92	104	94	110	73		51 .	53	88	87	73	78	64
62	79	77	65	70	88	103	82	105	82		54 .	48	76	75	74	74	69
56	76	76	77	75	97	89	64	91	69		81 .	50	73	83	60	75	63
76	90	91	68	81	103	92	65	75	73		84 .	63	67	90	61	73	56
57	96	79	67	76	86	77	54	70	75		84 .	70	87	70	68	78	82
68	81	91	69	74	80	69	55	110	71		78 .	66	76	106	62	76	93
88	96	68	60	72	97	62	86	103	90		68 .	64	85	85	89	75	93
69	74	62	66	92	102	81	97	98	89		71 .	42	95	77	72	90	89
68	67	74	97	89	92	71	89	86	69		73 .	35	92	67	52	92	86
66	63	88	90	89	90	61	77	93	70		55 .	54	86	69	52	89	96
53	85	105	90	84	88	66	71	91	70		49 .	78	104	68	51	82	78
51	90	73	97	83	99	96	66	67	64		45 .	61	102	71	54	68	75
50	63	63	87	68	79	97	59	72	67		48 .	57	70	65	87	65	65
49	67	68	95	70	72	70	54	69	71		80 .	44	64	64	67	91	62
49	88	87	92	114	94	61	60	65	58		61 .	42	59	58	52	70	64
80	70	69	85	102	88	64	60	67	57		53 .	43	63	58	58	76	55
65	73	88	67	82	72	92	55	100	65		55 .	51	74	67	53	73	60
58	85	69	81	73	70	74	57	73	57		59 .	44	66	62	54	77	83
51	74	69	86	68	72	67	73	68	96		75 .	45	68	67	69	73	81
74	76	65	66	80	76	83	73	70	90		54 .	57	71	61	67	73	57
50	68	69	73	69	76	92	61	73	75		53 .	54	95	62	58	66	59
55	88	81	66	65	87	73	62	86	61		55 .	45	99	59	52	64	62
64	64	66	69	83	74	68	55	72	59		53 .	43	70	60	76	92	59
52	67	63	64	79	70	78	56	67	56		99 .	46	65	81	59	65	79
90	95	113	82	88	101	102	100	94	89 .		106 .	69	132	100	76	90	69
90	94	106	89	83	108	101	86	78	93 .		108 .	73	119	99	87	73	90
73	88	112	102	115	95	97	88	82	76 .		103 .	67	108	86	88	96	69
67	86	108	83	114	91	94	88	97	82 .		85 .	57	125	80	76	76	73
62	81	113	80	102	94	76	96	94	75 .		81 .	81	98	65	72	70	90
79	84	106	95	84	85	76	94	80	67 .		71 .	62	92	57	75	71	68
66	85	102	80	81	85	95	89	76	86 .		71 .	67	113	63	70	71	67
71	81	89	85	84	93	99	75	94	78 .		65 .	67	97	83	66	75	71
55	80	86	82	90	83	81	77	81	76 .		57 .	80	73	66	82	75	87
61	79	82	83	95	90	82	78	80	80 .		70 .	74	74	62	71	71	76
67	85	89	81	101	94	75	89	95	81 .		65 .	66	115	65	65	90	72
62	90	97	96	83	92	76	94	88	77 .		71 .	74	98	62	92	87	69
68	87	89	103	81	97	97	83	96	113 .		87 .	79	92	76	99	76	101
64	97	83	90	117	110	108	104	108	105 .		80 .	82	78	98	117	83	102
82	98	89	113	115	114	85	109	105	114 .		63 .	73	96	104	102	86	98
69	89	107	130	106	98	77	107	95	100 .		74 .	64	101	68	103	97	89
77	90	86	125	91	89	82	106	87	97 .		83 .	70	84	64	94	103	90
74	90	85	110	92	108	107	82	106	95 .		79 .	80	109	63	94	95	96
68	93	100	114	122	125	109	87	102	83 .		63 .	75	84	61	91	98	89
62	92	100	115	103	109	100	76	90	91 .		81 .	67	85	87	84	84	84
84	79	108	114	102	99	100	80	96	70 .		84 .	72	97	84	87	94	83
86	91	114	109	80	110	115	83	87	77 .		67 .	59	112	92	80	76	77
92	89	98	86	78	108	105	89	83	98 .		63 .	58	111	84	78	78	70
80	90	86	94	80	100	79	78	99	105 .		60 .	58	115	79	72	75	73
85	98	91	89	82	94	81	86	111	75 .		59 .	59	105	85	64	69	67
85	118	112	77	102	109	76	87	102	72 .		68 .	75	107	86	71	76	68
84	100	115	82	89	109	108	77	109	71 .		62 .	63	96	90	94	71	70
89	98	121	91	88	110	88	111	115	72 .		78 .	59	104	84	99	74	101
78	102	120	100	95	109	78	103	98	71 .		91 .	59	97	70	106	77	85
71	104	114	83	84	100	85	102	100	104 .		82 .	76	89	67	92	69	82
70	99	126	95	84	91	77	108	105	107 .		74 .	76	80	74	79	75	93
66	94	102	95	78	93	101	105	104	103 .		67 .	71	67	66	84	86	95
67	100	127	120	97	90	108	96	93	91 .		63 .	76	69	72	76	102	73
65	87	107	114	115	108	101	85	94	81 .		78 .	69	70	60	72	102	77
72	85	100	114	114	89	102	81	93	75 .		78 .	65	83	67	74	94	64
69	93	86	118	113	96	83	103	91	72 .		78 .	66	73	71	75	92	62
66	97	82	104	100	111	74	91	78	63 .		72 .	59	73	73	72	90	67
75	85	94	111	111	113	78	88	74	69 .		75 .	48	69	91	61	92	57
85	97	84	108	109	112	86	97	77	75 .		75 .	69	81	81	83	85	62
74	90	91	88	101	109	103	93	92	85 .		67 .	78	73	89	68	85	88
72	101	104	77	101	104	108	95	79	91 .		71 .	63	71	88	61	68	66

60	91	100	87	94	119	96	85	78	91 .	64 .	53	98	79	67	67	61
84	86	82	80	82	102	97	79	75	83 .	69 .	58	100	85	88	64	80
83	84	81	105	90	92	92	84	95	93 .	63 .	64	91	69	89	83	97
88	98	75	81	88	99	93	97	102	105 .	87 .	69	92	82	83	67	91
74	88	100	70	105	98	91	83	86	88 .	65 .	59	86	81	81	85	85
77	84	105	91	95	90	78	81	86	86 .	59 .	58	67	70	83	73	78
61	80	83	82	83	97	77	76	92	68 .	66 .	58	67	57	78	68	62
61	85	106	80	83	101	71	94	90	70 .	65 .	70	73	62	72	69	57
60	79	99	76	76	83	77	73	87	87 .	72 .	70	68	63	80	81	61
64	77	86	81	83	85	75	75	80	73 .	74 .	60	72	62	74	91	56
62	74	76	86	81	91	78	65	84	67 .	60 .	63	73	65	74	94	63
60	80	79	87	83	91	72	75	81	69 .	57 .	61	93	64	72	88	75
70	78	77	88	113	85	75	84	75	77 .	61 .	63	87	61	70	82	62
61	90	82	87	87	91	77	73	97	74 .	65 .	62	84	61	75	68	66
64	76	88	77	83	104	78	95	81	80 .	74 .	62	68	56	71	67	78
61	74	83	101	105	113	79	79	78	74 .	61 .	70	72	74	73	75	80
69	73	96	102	106	93	72	74	80	73 .	72 .	71	72	66	75	68	80
62	72	100	93	89	90	84	79	86	91 .	62 .	73	69	62	79	64	70
67	73	96	87	85	90	84	78	79	88 .	66 .	62	97	67	85	75	71
69	73	92	82	84	90	80	67	88	71 .	65 .	55	76	72	75	68	63
68	72	86	85	82	106	78	67	99	81 .	66 .	55	70	68	69	69	88
70	83	85	75	84	103	76	86	81	68 .	61 .	79	69	59	76	69	68
80	80	77	83	80	92	72	74	78	80 .	61 .	61	66	61	83	83	71
71	78	88	105	81	86	78	83	87	74 .	73 .	58	93	87	70	67	65
69	74	84	89	97	87	78	70	81	70 .	60 .	57	70	74	74	93	82
65	74	84	86	93	108	79	98	79	100 .	62 .	55	68	65	69	86	62
69	92	100	101	84	87	73	98	93	76 .	62 .	66	70	63	83	69	71
92	76	84	82	84	93	72	89	84	76 .	77 .	61	79	65	69	73	66
68	80	88	78	95	111	105	78	90	94 .	61 .	53	85	62	72	70	65
67	86	89	105	89	108	79	84	104	77 .	74 .	76	75	62	68	88	71
61	84	85	94	80	93	78	77	79	76 .	69 .	73	70	67	71	70	83
67	80	105	97	85	101	80	79	82	82 .	79 .	66	92	87	75	65	78
90	84	111	80	97	91	77	88	77	75 .	84 .	70	99	72	87	90	63
68	78	110	78	106	84	97	77	84	76 .	74 .	75	75	66	99	83	64
71	80	93	82	107	88	96	77	95	95 .	81 .	70	73	61	83	70	72
72	91	98	96	87	113	84	80	88	106 .	63 .	66	104	62	73	76	71
81	100	85	86	89	102	79	111	101	84 .	74 .	79	104	65	99	78	96
81	104	90	84	85	97	97	117	113	102 .	93 .	93	84	82	109	72	83
88	99	103	108	111	109	115	107	118	108 .	88 .	87	102	94	104	106	101
87	99	138	122	109	121	105	102	109	110 .	80 .	80	114	102	94	105	115
83	111	128	122	112	123	108	109	107	92 .	86 .	77	107	84	94	95	104
74	100	129	137	128	116	103	100	106	108 .	86 .	80	118	75	84	100	106
89	102	125	120	107	116	101	107	106	97 .	80 .	79	125	71	76	92	111
95	101	134	111	116	113	83	96	88	92 .	79 .	91	111	76	79	93	100
86	87	123	114	108	118	86	85	81	92 .	61 .	84	106	81	75	93	96
95	85	139	108	98	118	95	77	115	96 .	57 .	80	87	69	78	70	89
94	81	135	94	96	124	114	74	117	95 .	72 .	83	87	70	71	69	101
85	100	115	108	101	122	102	76	112	100 .	60 .	73	110	95	70	87	76
76	81	108	117	98	120	85	85	112	86 .	65 .	77	101	89	76	70	70
78	73	135	96	109	120	90	80	103	78 .	62 .	75	106	90	63	96	82
90	72	137	126	127	109	144	76	96	70 .	59 .	54	103	90	68	98	79
85	90	114	116	111	104	97	82	95	68 .	61 .	76	101	86	98	95	88
84	79	109	110	115	107	100	112	95	102 .	92 .	82	87	97	98	93	93
77	73	141	116	121	107	104	99	75	94 .	78 .	78	83	80	92	105	98
79	103	108	111	108	94	97	104	73	68 .	73 .	73	90	66	79	91	87
86	104	98	114	97	110	96	96	93	69 .	78 .	69	88	62	75	69	100
91	97	92	108	91	120	89	94	92	70 .	79 .	75	85	55	68	85	84
85	101	100	89	89	109	123	90	89	98 .	69 .	75	102	59	69	99	82
78	98	91	82	89	112	102	72	111	99 .	62 .	68	122	65	82	92	70
68	102	107	95	85	118	85	72	107	92 .	56 .	66	103	76	93	87	64
87	86	94	82	94	118	92	84	104	85 .	58 .	69	77	90	80	64	74
84	94	94	111	109	113	90	71	95	73 .	73 .	79	84	84	65	67	90
88	78	98	87	93	123	112	88	99	71 .	59 .	71	88	82	66	79	63
72	69	132	83	102	127	89	107	81	84 .	63 .	86	72	93	65	79	65
67	86	124	104	105	112	96	92	96	97 .	55 .	72	95	79	72	61	60
69	97	113	120	106	113	86	96	103	100 .	58 .	64	100	91	69	67	86
78	88	112	112	102	108	94	99	97	85 .	74 .	59	95	83	68	91	97
73	73	95	87	86	92	91	96	92	73 .	83 .	73	90	78	58	90	91
80	90	85	89	105	103	93	107	77	68 .	68 .	75	66	89	87	66	79
67	81	78	115	119	123	91	90	96	92 .	69 .	65	100	83	87	67	74
66	73	88	115	107	111	85	84	89	112 .	63 .	64	102	66	85	90	63
75	78	79	105	113	101	79	75	75	90 .	55 .	70	78	72	75	90	60
71	66	84	107	100	102	81	71	73	88 .	65 .	67	74	60	77	84	56
66	67	101	87	93	100	81	78	75	80 .	59 .	64	72	68	65	75	65
67	68	104	75	91	89	79	72	73	67 .	53 .	66	89	64	66	69	67
62	69	77	81	88	99	82	73	88	79 .	82 .	58	70	59	74	80	78
66	71	83	95	74	97	73	73	91	66 .	67 .	75	71	59	68	73	61
72	67	83	89	98	94	81	75	82	74 .	58 .	64	74	68	69	67	61
67	68	100	82	89	92	74	83	76	71 .	62 .	67	100	64	74	68	67
67	74	84	84	89	109	74	98	92	68 .	84 .	62	95	62	64	74	89
61	69	85	89	78	103	87	74	89	81 .	65 .	75	79	64	65	91	80
67	81	84	99	90	93	86	73	100	76 .	64 .	63	77	84	83	76	69
79	74	87	83	89	99	72	72	79	76 .	64 .	70	74	73	96	64	64
66	68	86	90	93	100	73	72	106	111 .	57 .	63	72	61	81	84	70
62	69	88	84	100	105	73	76	74	76 .	61 .	56	78	61	72	93	78
63	69	86	85	97	92	65	76	93	87 .	68 .	70	74	69	67	70	73
68	74	92	96	86	92	71	77	83	76 .	70 .	76	68	69	72	70	71
67	78	97	89	84	100	81	78	77	87 .	62 .	64	89	74	65	82	64
66	75	90	89	81	97	98	83	83	90 .	58 .	68	78	67	67	72	64
70	71	87	102	83	96	78	72	80	99 .	56 .	59	73	61	70	70	82
73	70	85	90	83	99	77	76	80	75 .	60 .	60	73	77	78	87	71
71	84	89	85	88	105	78	76	85	75 .	73 .	60	73	73	71	90	68
64	81	86	81	95	95	77	71	96	73 .	69 .	74	76	62	61	62	63
68	74	95	88	94	97	77	87	82	69 .	56 .	68	69	61	65	69	76
66	68	108	85	90	109	88	85	82	102 .	60 .	64	90	77	64	68	61
79	67	96	83	96	100	77	79	80	73 .	53 .	69	79	68	98	63	67
79	84	101	106	114	98	69	72	84	79 .	62 .	58	99	66	84	91	74
65	80	109	108	100	126	69	75	80	83 .	82 .	62	89	65	78	89	97
66	72	117	114	91	106	68	77	98	80 .	65 .	72	83	62	68	77	82
61	79	96	110	91	103	84	86	91	80 .	65 .	69	91	76	70	69	74
62	71	93	92	96	105	87	77	99	79 .	70 .	66	97	72	87	75	81
62	87	92	90	89	116	78	97	94	107 .	71 .	92	105	79	103	74	74
86	107	89	90	102	107	78	114	102	106 .	94 .	93	96	73	116	79	105
86	101	96	100	113	112	118	121	124	113 .	94 .	97	95	86	110	103	111
95	101	115	127	109	122	106	108	112	115 .	91 .	101	109	105	104	103	98

79	106	126	123	103	118	109	99	105	98 .	84 .	93	117	92	86	97	99
76	92	103	109	112	122	106	102	109	98 .	82 .	93	128	96	89	107	91
82	100	115	125	130	121	97	103	103	99 .	82 .	88	112	98	82	93	73
73	93	121	115	115	116	81	95	96	79 .	81 .	108	116	92	80	79	90
77	85	108	117	111	133	77	81	101	71 .	80 .	105	122	85	76	103	74
74	104	108	103	105	130	73	78	98	99 .	77 .	105	106	90	80	92	73
77	92	109	113	108	120	105	75	88	95 .	73 .	88	105	83	70	95	86
86	84	123	115	102	122	107	104	110	93 .	61 .	76	118	84	67	77	89
81	85	121	110	112	126	95	109	111	79 .	72 .	90	90	103	79	75	96
69	79	116	114	107	116	95	107	117	93 .	61 .	88	104	75	98	77	92
80	75	111	118	122	113	89	100	110	103 .	61 .	87	99	74	73	90	94
68	77	133	106	109	119	71	94	109	85 .	56 .	98	98	70	65	78	86
63	106	121	112	106	121	71	98	104	83 .	87 .	101	100	68	64	80	87
61	105	127	131	99	109	77	89	108	80 .	72 .	98	101	103	85	86	95
79	104	124	102	105	118	72	76	105	86 .	68 .	93	108	97	82	77	92
84	99	129	124	117	109	69	83	120	114 .	56 .	97	98	90	73	72	85
88	98	122	112	103	117	77	82	104	106 .	67 .	87	99	76	66	74	77
73	85	133	103	98	123	110	73	105	97 .	53 .	91	92	74	66	90	92
79	102	103	99	103	115	107	71	95	78 .	62 .	84	100	77	67	95	91
73	88	102	115	97	110	101	72	109	76 .	69 .	76	92	78	67	91	73
69	68	95	111	110	113	92	77	111	86 .	92 .	77	92	108	66	68	74
61	78	102	104	101	106	98	77	100	80 .	83 .	85	98	90	65	91	88
60	85	108	111	95	108	103	99	102	85 .	74 .	86	108	96	76	84	79
53	80	114	119	91	109	90	114	103	103 .	60 .	80	102	86	81	96	80
67	74	108	101	98	113	91	101	100	97 .	58 .	75	86	87	76	110	82
85	81	117	109	104	106	89	102	99	90 .	60 .	72	89	83	85	97	86
79	90	111	101	103	109	90	98	90	75 .	65 .	72	104	68	68	90	81
78	85	102	102	91	106	89	92	93	73 .	65 .	67	90	61	74	69	75
60	66	98	90	89	98	79	97	85	72 .	82 .	79	85	61	60	71	66
67	73	92	88	96	126	71	86	93	98 .	81 .	64	94	75	66	66	74
65	84	91	116	104	105	66	77	81	100 .	69 .	54	85	65	60	85	81
60	78	103	101	97	105	71	89	83	87 .	67 .	73	98	61	78	65	83
64	71	103	84	98	91	83	85	89	87 .	56 .	67	86	73	79	73	74
76	72	95	89	88	106	72	71	80	82 .	55 .	61	91	80	78	85	68
70	70	93	84	86	117	70	71	80	70 .	55 .	73	90	73	68	66	64
89	77	101	87	84	105	67	72	87	68 .	57 .	63	84	71	68	78	60
73	81	101	91	86	104	67	73	81	79 .	65 .	60	87	65	63	72	78
68	79	101	83	99	96	87	83	80	74 .	59 .	65	97	79	64	72	68
62	73	92	93	88	99	66	71	78	85 .	57 .	73	87	71	69	91	67
73	74	82	83	86	95	72	72	80	73 .	74 .	61	79	73	64	86	62
79	74	86	96	88	98	81	72	80	77 .	61 .	58	76	67	83	73	61
67	68	90	90	81	105	81	84	76	78 .	62 .	72	79	63	66	74	79
65	67	100	90	91	96	72	74	80	99 .	57 .	56	81	61	59	68	71
61	70	86	83	87	104	73	72	113	89 .	56 .	64	86	68	59	80	62
60	79	89	85	97	103	66	76	84	77 .	61 .	58	75	61	75	83	74
63	70	90	102	87	91	82	82	76	75 .	60 .	55	73	61	64	74	67
62	85	79	96	85	95	77	78	77	76	67 .	53	73	66	64	68	74
59	68	87	85	80	92	76	72	90	85	77 .	65	104	74	63	73	72
61	69	91	90	81	98	77	78	79	77	75 .	65	92	70	66	72	65
57	102	79	89	85	102	96	78	78	81	68 .	59	85	71	69	77	74
59	83	77	91	79	97	79	93	79	80	63 .	56	77	69	69	73	80
64	73	88	91	85	101	72	91	99	85	75 .	57	72	74	81	71	74
63	69	86	98	81	108	76	89	82	76	60 .	56	79	64	77	93	70
71	71	98	91	80	103	84	79	76	81	62 .	55	76	72	66	85	62
75	83	84	91	101	104	72	75	73	91	61 .	66	80	74	66	69	78
71	73	91	85	86	94	72	85	91	97	70 .	68	83	77	64	75	81
60	72	89	99	85	97	72	82	78	76	96 .	59	98	83	76	79	73
59	71	107	90	91	107	83	77	79	86	76 .	55	80	86	69	80	67
63	89	107	97	86	103	72	91	77	74	69 .	57	86	81	66	70	65
65	75	89	89	84	109	70	96	94	86	79 .	73	83	85	70	78	65
68	79	89	95	102	115	83	82	79	96	67 .	71	80	77	73	79	67
60	77	88	95	93	104	84	90	81	86	92 .	57	105	72	104	75	64
72	96	92	94	94	111	83	112	94	93	91 .	86	110	69	113	89	82
71	123	91	104	100	115	94	120	126	87	96 .	84	94	98	115	99	112
99	104	113	122	118	121	119	106	110	118	94 .	82	112	98	109	85	97
86	110	126	124	111	138	103	114	104	110	92 .	76	117	87	108	100	99
90	98	114	122	110	127	101	110	98	112	79 .	77	105	81	91	104	90
80	97	127	129	115	116	107	101	99	98	78 .	77	99	85	92	98	86
74	91	121	115	101	128	101	107	108	89	86 .	71	93	79	103	98	81
74	86	105	99	93	126	102	125	82	94	70 .	60	104	78	94	94	84
79	86	115	119	102	124	91	112	98	81	67 .	55	122	93	96	86	88
80	83	100	118	107	114	105	84	97	97	72 .	79	108	89	89	88	68
63	97	101	117	97	126	96	100	83	116	61 .	72	94	75	70	85	63
80	104	119	116	96	138	84	89	111	107	83 .	60	93	69	68	89	68
66	109	116	111	109	136	70	77	102	108	85 .	62	107	85	65	89	62
70	103	96	105	121	121	75	87	109	111	84 .	56	100	79	98	111	77
61	99	90	101	116	129	71	108	100	102	95 .	66	103	81	92	107	95
72	97	102	116	109	117	72	113	92	96	81 .	80	98	91	92	103	94
58	85	96	111	90	112	112	103	90	92	74 .	94	85	85	88	104	87
72	76	106	117	93	117	99	101	94	99	64 .	74	84	73	79	105	87
88	107	95	118	103	116	92	77	97	104	62 .	73	86	81	71	96	61
83	95	84	117	123	123	95	76	93	97	57 .	76	79	91	77	96	58
89	89	94	99	115	117	93	77	86	93	49 .	67	89	78	86	91	62
80	75	113	114	108	120	94	82	74	89	63 .	65	104	81	74	77	66
80	73	121	103	109	110	74	83	78	99	69 .	68	100	87	70	92	88
75	74	114	98	102	107	73	76	79	86	58 .	57	86	82	71	91	98
84	77	109	100	91	116	70	103	78	90	81 .	57	80	81	83	78	81
74	72	121	101	110	123	94	116	79	91	83 .	52	77	82	75	103	80
66	103	115	104	110	125	95	96	118	99	78 .	82	91	82	78	81	62
61	92	114	105	94	115	87	106	88	93	77 .	74	106	87	77	91	67
57	92	108	116	103	103	93	102	72	99	62 .	62	107	85	76	71	66
88	86	108	109	91	102	106	104	68	92	77 .	44	101	80	77	74	98
80	74	101	109	102	107	90	90	115	99	93 .	51	89	74	73	90	93
74	87	87	98	93	104	65	80	101	79	95 .	63	99	74	67	80	86
61	103	79	98	82	110	64	96	92	95	82 .	74	86	68	65	75	77
55	86	97	106	107	110	83	101	79	85	60 .	63	110	68	66	90	79
55	76	105	109	108	97	69	81	84	72	61 .	61	89	70	88	88	77
65	80	94	104	93	116	74	78	80	75	60 .	53	79	72	69	84	62
61	76	78	90	89	108	72	75	79	81	57 .	48	75	74	62	75	57
56	77	97	83	88	101	70	71	74	87	72 .	48	74	80	61	79	67
61	79	78	92	85	111	87	78	85	92	61 .	59	74	93	59	75	61
56	81	93	85	77	101	71	77	88	79	64 .	49	70	74	68	67	63
79	74	85	96	85	103	71	86	73	74	75 .	63	69	75	69	71	64
72	76	87	83	83	97	71	78	73	75	64 .	60	71	77	79	70	61
62	72	83	96	95	95	71	78	75	73	67 .	53	89	62	67	90	66

62	77	95	95	103	102	68	79	75	67	58 .	60	80	57	60	74	64
67	77	93	97	90	103	89	78	74	77	57 .	54	76	72	61	75	84
62	91	87	105	85	105	95	80	76	73	77 .	57	75	62	59	81	76
62	80	91	78	85	94	73	77	81	74	69 .	60	81	55	78	79	68
76	84	83	78	88	97	71	71	97	84	67 .	51	77	62	77	74	59
67	87	85	77	85	98							72	68			
62	82	105	92	83	102							86	69			
62	85	86	83	87	103							77	62			

8	7	7	10	10	12	12	12	1	1	1	1	2	2	5	6	6	8	9	11		1
Box-1	Box-6	Box-8	Box-7	Box-2	Box-3	Box-4	Box-7	Box-1	Box-3	Box-4	Box-5	Box-2	Box-3	Box-1	Box-2	Box-3	Box-8	Box-7	Box-5		Box-6
44.6	45	47	65	68	77	78	84	6.1	9.4	10.1	11.6	16.4	17.4	28.1	41	42	53.2	71	82		12.1
F	F	F	F	F	F	F	F	M	M	M	M	M	M	M	M	M	M	M	M		M
MCT	MCT	MCT	MCT	MCT	MCT	MCT	MCT	EtOH	EtOH	EtOH	EtOH	EtOH	EtOH	EtOH	EtOH	EtOH	EtOH	EtOH	EtOH		H2O
FT	FT	FT	FT	FT	FT	FT	FT	ME	ME	ME	ME	ME	ME	ME	ME	ME	ME	ME	ME		MH
82	67	88	50	64	108	50	64	97	96	96	88	124	59	83	51	42	85	58	116		95
81	43	77	80	68	96	54	68	84	92	100	89	112	88	66	65	50	54	62	112		118
77	40	74	60	45	68	56	62	97	92	67	74	82	105	69	95	62	50	59	97		110
60	42	53	55	54	72	99	88	101	92	107	88	96	97	107	92	84	52	58	62		94
62	73	50	88	49	102	86	69	105	90	95	65	69	83	104	54	65	80	65	106		104
84	89	50	76	48	104	63	63	99	104	71	92	61	74	97	94	62	75	87	69		93
81	80	64	48	75	72	82	87	101	99	91	96	61	57	86	101	63	44	62	62		106
74	43	70	59	68	102	98	66	105	98	109	98	89	70	94	93	86	101	100	62		86
81	75	53	108	59	120	93	87	125	99	103	97	118	62	95	99	82	106	83	85		80
88	84	51	98	51	109	105	102	98	115	107	88	105	59	96	78	83	98	71	105		95
83	96	82	101	75	108	95	61	106	105	103	96	112	57	90	85	84	101	50	99		103
74	95	46	101	63	108	99	92	81	96	107	83	108	56	86	95	87	103	93	56		70
83	96	46	104	43	101	76	94	99	90	104	70	104	83	68	102	71	97	96	83		65
83	90	44	102	40	100	91	60	103	78	101	76	108	106	74	64	54	106	93	100		108
87	86	70	96	69	103	98	73	69	89	100	66	103	104	54	71	63	107	67	99		96
83	82	78	95	67	97	109	103	68	78	95	79	108	94	77	81	83	86	54	111		92
57	79	87	96	52	72	113	89	96	66	78	70	107	87	64	57	59	93	73	110		109
48	70	63	72	29	68	104	58	72	89	77	73	105	56	59	54	56	72	50	112		102
64	32	35	45	32	107	108	60	85	102	73	67	76	50	87	59	70	43	60	110		102
72	26	42	48	33	103	112	93	112	71	66	95	51	55	74	94	72	77	90	117		71
76	44	88	77	37	99	115	95	112	77	86	96	50	85	78	61	76	73	84	111		66
57	35	80	96	67	87	103	63	110	73	113	92	54	62	99	53	87	49	98	114		78
65	57	92	96	39	61	67	53	105	65	107	72	99	55	91	60	84	42	91	118		91
69	73	96	100	33	62	51	53	100	75	100	71	93	54	90	84	74	72	89	98		76
52	87	91	71	66	60	70	59	96	66	88	68	52	56	60	78	53	61	53	75		108
73	78	55	48	47	87	62	102	74	72	69	87	44	56	54	60	72	51	48	50		102
77	64	37	47	27	97	46	113	62	107	70	90	51	55	78	42	52	75	61	74		102
78	33	39	49	20	97	49	85	66	114	68	64	52	61	60	50	73	82	97	68		100
71	27	47	49	30	100	97	46	87	92	90	82	68	56	54	46	50	70	63	62		70
46	29	49	89	20	63	110	67	76	103	75	84	52	60	61	44	79	55	58	61		80
45	37	81	54	14	56	111	46	77	93	75	84	50	98	61	72	56	66	54	77		101
54	38	69	48	25	59	77	40	67	96	78	72	63	97	86	99	64	77	62	64		105
48	44	45	69	59	57	53	33	102	86	82	76	56	61	90	79	60	93	82	81		118
45	51	45	84	77	59	48	103	87	57	63	63	52	74	56	64	81	96	81	107		97
74	86	75	88	43	103	47	87	83	63	79	94	110	98	57	38	86	52	50	108		115
53	89	90	90	33	87	58	74	100	61	93	85	111	100	90	42	54	46	44	100		111
47	84	94	50	36	100	56	48	69	75	64	63	115	86	66	56	34	51	105	64		92
74	58	81	43	69	56	51	35	59	58	52	63	114	58	55	76	49	74	91	44		94
79	34	71	49	57	53	70	29	65	61	90	73	119	43	79	59	47	94	67	49		68
60	38	34	83	50	59	93	23	87	63	59	60	118	60	64	41	52	80	45	88		67
44	43	30	89	31	59	99	29	100	100	65	64	115	91	58	45	69	43	52	68		88
51	73	41	73	25	67	76	98	63	90	81	77	88	86	61	79	50	42	58	62		62
47	89	46	46	63	108	53	75	64	68	63	63	56	54	58	72	52	45	60	53		69
48	69	43	54	47	79	52	44	69	63	60	63	50	69	92	52	53	54	96	56		70
72	45	82	55	36	48	57	36	93	63	63	83	50	56	65	48	55	47	72	98		98
54	44	88	55	36	56	75	39	81	66	66	63	81	87	54	42	47	79	50	84		89
50	42	91	81	36	63	53	49	64	66	64	63	61	85	60	65	69	98	54	47		63
58	60	77	88	36	95	48	50	65	64	59	82	57	59	83	45	49	83	73	50		63
61	36	45	63	41	55	44	43	68	60	70	70	61	49	64	46	41	48	58	52		64
50	38	43	51	59	59	51	79	87	77	84	69	74	52	61	44	77	49	83	56		68
67	41	44	51	47	62	77	49	92	85	62	64	50	57	64	72	45	55	70	56		69
50	45	41	51	29	62	91	43	65	60	57	75	49	52	60	43	47	88	47	80		70
51	76	40	53	34	60	79	65	67	64	57	63	54	73	62	46	47	84	58	53		83
63	71	79	52	30	76	63	111	64	64	81	58	70	55	68	43	72	46	74	54		96
76	73	65	81	36	60	51	65	64	65	64	58	57	54	83	59	55	48	88	56		71
64	46	39	63	66	62	47	56	64	81	62	86	57	57	86	67	41	62	58	72		69
52	39	37	51	48	61	57	58	82	64	61	69	59	100	60	49	49	87	55	61		71
52	42	45	48	36	67	74	58	83	62	58	58	59	90	60	42	47	64	59	61		64
50	42	77	71	41	61	52	66	64	67	92	57	61	56	88	41	65	41	72	80		93
46	74	83	97	41	61	52	59	65	81	69	85	59	51	80	86	48	47	100	87		69
49	84	83	62	40	65	62	89	66	59	58	80	58	56	55	72	42	51	72	58		68
83	55	61	48	42	96	55	107	71	62	60	59	59	51	60	40	50	52	53	61		94
82	37	40	72	44	90	50	74	76	58	62	60	100	61	60	40	41	89	49	61		75
80	43	41	63	44	92	49	60	62	91	86	70	118	92	94	44	90	66	54	61		69
57	48	46	50	64	85	88	71	59	76	63	77	110	72	76	60	62	40	71	71		66
51	67	76	57	60	60	83	66	62	60	58	63	111	58	59	79	45	45	69	57		107
67	86	83	82	37	62	63	61	81	57	70	58	67	76	78	49	49	73	56	61		72
89	76	64	95	48	67	54	72	76	78	62	84	53	63	107	93	76	63	75	82		64
84	66	42	58	40	97	65	94	74	68	80	104	58	109	103	101	58	93	101	94		67
88	88	56	83	70	91	105	119	80	94	75	99	67	109	109	78	74	98	104	57		76
75	60	87	102	71	91	100	82	104	86	103	103	98	109	96	100	60	70	97	90		109
77	58	87	95	56	98	102	96	101	89	107	104	106	108	85	95	74	63	93	97		106
66	99	84	88	39	97	100	112	99	93	104	99	74	82	89	97	88	92	91	108		75
49	92	87	97	38	100	101	102	103	108	109	104	60	55	85	89	92	101	87	108		104
49	84	75	72	69	104	99	76	96	102	102	94	119	54	65	80	84	110	89	94		82
81	91	48	47	46	90	98	100	94	121	100	83	125	90	72	77	85	89	95	98		60
79	83	55	83	26	84	71	107	85	74	102	98	123	95	66	84	73	100	90	108		99
85	72	84	91	44	59	64	105	81	94	94	99	85	79	93	99	78	108	86	108		106
83	67	88	97	38	65	100	101	69	99	69	70	103	57	93	69	65	85	86	101		105
82	39	88	90	68	89	81	94	75	79	78	60	112	55	71	67	73	48	80	102		107
84	37	82	59	64	114	61	74	74	93	73	68	88	88	72	90	90	39	81	62		75
87	63	77	41	46	116	80	61	98	74	81	64	65	75	96	101	88	80	77	55		74
84	82	71	36	37	99	102	70	115	67	98	90	59	81	100	73	84	78	49	96		103
71	49	41	39	31	106	106	94	112	62	105	97	88	95	84	49	80	48	51	113		103
57	43	41	44	29	118	112	73	103	68	114	91	85	106	54	87	81	43	80	91		98
45	47	43	97	56	105	105	99	102	92	106	60	71	99	84	69	72	53	79	90		73
61	77	79	102	60	115	103	86	98	73	69	63	88	92	80	48	57	48	67	100		57
49	75	66	104	34	110	104	81	98	62	69	80	106	59	75	70	54	84	54	80		98
47	47	42	63	27	102	93	81	70	89	79	68	75	65	62	72	54	89	49	111		74
59	45	36	41	16	100	58	64	81	72	63	63	64	83	80	71	86	63	55	110		68
57	48	78	43	19	94	52	64	98	68	61	97	88	64	64	54	61	43	79	79		67
44	43	70	50	66	69	57	65	82	62	77	72	104	78	67	83	59	38	70	96		64
55	81	49	91	50	70	84	65	79	79	86	62	75	65	72	72	74	78	56	92		69
81	75	74	54	25	82	87	66	76	68	68	62	64	57	66	53	72	76	64	64		83
74	87	82	42	16	87	59	95	103	65	76	94	63	53	85	44	72	52	94	56		75
50	77	94	43	27	79	49	108	76	65	98	87	71	66	89	52	55	49	75	51		80
52	69	74	71	13	69	88	109	97	73	68	60	103	61	63	83	76	53	52	83		79
63	45	47	107	9	70	86	89	78	110	67	77	99	77	73	80	61	45	53	78		115

49	41	37	89	45	93	61	100	72	81	88	66	103	66	80	52	84	44	49	59	106
49	46	39	79	70	106	48	92	96	95	74	102	75	110	91	38	66	74	85	57	103
62	71	46	84	51	111	92	68	98	101	97	89	64	81	61	33	46	90	59	57	96
92	78	39	64	26	103	71	59	85	88	77	69	64	58	57	99	49	105	51	89	73
75	59	79	40	18	88	56	75	73	59	52	60	115	89	81	80	62	85	53	82	57
50	44	83	32	14	64	70	58	64	58	55	60	121	82	63	44	47	66	97	53	58
44	45	55	42	17	63	91	90	72	74	88	76	101	60	64	39	67	47	67	52	94
58	44	41	93	10	71	85	87	68	72	84	58	71	53	69	40	51	46	49	92	73
51	44	50	74	11	90	58	75	62	61	58	62	56	71	58	38	53	45	54	96	63
45	66	75	49	35	93	51	58	74	64	51	68	80	88	73	40	71	63	76	74	91
72	50	49	47	75	64	51	60	62	57	57	57	69	69	59	91	77	93	81	51	65
49	46	44	88	61	67	94	76	81	73	75	61	59	53	64	79	47	68	54	50	63
47	55	76	68	55	69	69	81	69	63	64	57	59	54	83	47	44	52	49	62	61
52	88	78	69	70	93	58	57	70	64	62	58	89	53	65	42	56	75	58	55	70
48	67	61	65	81	71	52	58	61	85	54	59	88	84	62	54	43	52	54	58	62
53	44	46	53	83	69	88	58	87	86	93	80	59	55	56	43	49	80	99	78	81
70	44	44	47	75	70	69	61	88	60	78	75	58	71	68			68	81	61	66
63	58	47	77	48	106	60	77	66	62	58	58	63	97	61			49	51	59	59
52	70	70	84	48	100	57	98	61	61	54	59	103	72	65			53	52	54	69
	48	44	53	70	70	80	60	58	76	53	76	89	56				53	58	70	83
	48	46	47	82	66	79	63	60	70	70	57	60	56				79	78	85	85
	47	65	47	66	79	59	56	69	63	57	58	59	62				64	83	58	63
	47	84	54	43	70	58	60	65	66	53	56	96	73				76	53	57	64
	49	77	51	45	70	69	59	63	87	53	53	72	60				55	55	54	64
	72	49	89	44	66	87	82	60	62	53	76	60	60				68	55	62	64
	49	43	76	63	99	60	90	85	64	63	56	73	74				95	71	91	81
	43	47	50	78	92	52	64	100	78	96	70	63	97				72	52	72	92
	47	66	46	54	64	56	64	66	70	70	58	96	74				51	66	49	64
	55	49	52	49	64	49	82	70	67	53	61	87	60				51	53	57	70
	73	48	78	63	61	87	86	62	64	73	67	72	75				58	59	59	69
	49	81	88	46	105	93	61	94	61	63	69	71	57				50	56	97	66
	48	71	55	61	93	95	60	69	67	93	59	67	78				50	82	62	81
	68	75	50	88	63	72	84	69	91	93	60	83	90				90	59	59	79
	81	81	66	83	67	62	93	73	81	62	83	110	68				102	54	71	70
	54	77	93	49	88	60	69	74	62	59	89	79	92				70	70	66	78
	78	44	71	49	92	88	69	67	89	82	89	67	105				52	110	89	78
	69	72	54	49	110	102	110	98	92	85	68	102	92				84	113	88	69
	59	60	54	82	88	113	116	103	90	91	93	116	104				98	108	82	103
	81	52	71	86	105	93	101	101	84	109	102	120	105				98	94	100	102
	75	81	108	57	81	107	120	77	81	108	89	117	98				64	94	105	108
	69	70	97	43	74	106	120	102	94	103	84	92	87				105	77	75	107
	85	59	78	68	110	104	118	76	99	106	97	93	66				99	63	102	112
	78	87	59	47	113	80	110	98	85	86	79	95	74				96	55	84	97
	87	78	87	43	81	61	116	88	72	79	78	82	67				96	81	100	95
	51	86	104	75	113	79	117	89	105	79	68	118	69				80	67	88	96
	49	56	98	59	93	107	111	107	108	96	70	100	90				53	61	81	67
	79	37	82	59	96	104	107	76	93	89	66	117	86				59	69	102	67
	90	64	91	67	123	108	100	88	65	115	92	101	89				80	102	100	68
	56	75	87	70	117	104	65	82	68	102	100	73	94				53	105	87	72
	59	53	92	64	116	103	86	101	93	75	89	61	76				68	87	75	68
	48	41	61	64	101	105	87	107	83	90	76	94	73				101	85	100	109
	52	40	42	46	87	104	63	109	80	103	70	80	75				100	81	93	74
	84	77	43	56	73	99	92	96	77	101	62	82	64				78	47	97	81
	92	82	46	40	65	70	118	95	77	72	91	77	63				51	51	81	97
	52	77	64	45	67	68	117	63	75	70	65	106	79				53	79	91	72
	53	50	52	43	98	61	116	70	103	80	69	93	88				75	96	100	74
	86	47	50	57	107	92	110	103	96	86	68	71	65				53	-1	90	81
	58	59	45	46	113	73	93	74	72	68	84	61	58				52	1	65	68
	53	75	78	45	115	81	86	73	79	80	80	65	53				51	75	84	68
	75	49	103	46	100	62	62	97	84	81	84	64	82				48	56	92	86
	57	43	87	44	72	61	98	73	78	69	72	58	68				48	57	71	85
	80	71	91	60	65	57	87	99	81	86	73	77	65				85	55	67	99
	67	54	71	73	93	57	62	86	82	77	67	89	63				97	58	88	96
	58	69	45	50	69	99	89	78	85	87	90	78	63				58	53	89	74
	69	47	42	45	64	110	94	85	74	74	68	75	77				68	60	61	113
	53	45	41	51	68	92	70	79	96	91	86	59	60				78	66	51	106
	66	86	90	40	67	94	72	77	75	102	78	89	74				64	71	47	97
	48	85	97	43	106	72	69	89	59	87	77	110	102				44	52	80	85
	42	77	70	51	85	59	66	97	88	56	63	113	78				65	102	72	61
	45	72	42	74	63	54	91	63	72	45	67	89	89				81	96	52	59
	47	51	42	59	62	84	62	69	67	49	66	62	84				60	76	69	57
	49	45	83	40	70	63	86	63	63	81	62	69	60				38	63	105	82
	79	62	79	76	68	70	69	63	57	59	64	68	57				45	47	87	68
	61	50	51	60	95	86	62	64	72	63	63	57	53				80	54	52	63
	49	46	44	76	94	75	72	59	64	66	63	65	77				86	98	48	92
	43	72	50	67	64	57	90	81	59	63	61	102	71				71	65	49	91
	49	46	84	43	76	58	64	66	63	61	63	78	55				47	51	54	63
	44	40	83	47	66	64	57	63	70	62	59	54	73				43	47	90	59
	62	46	48	48	69	87	70	65	69	69	75	50	85				75	56	60	87
	73	75	49	65	83	57	58	73	59	85	59	59	57				48	83	48	63
	49	72	56	59	100	51	88	91	82	57	62	60	60				65	62	56	62
	50	62	48	40	94	56	88	65	70	58	71	72	95				52	53	58	70
71	84	62	107	93	104	94	74	103	92	98	99	85	103	75	69	45	53	101	67	107
63	66	73	95	81	99	105	68	103	89	91	101	82	72	81	61	91	86	94	106	97
69	60	78	71	77	85	80	83	90	66	81	96	107	71	71	91	101	91	66	71	97
61	76	66	77	78	81	69	64	67	96	84	90	88	89	93	65	64	62	89	72	76
73	63	81	65	75	90	100	96	98	83	71	73	70	66	69	51	62	50	99	70	81
64	83	60	87	77	84	74	106	78	88	72	80	66	67	62	69	85	80	70	93	106
58	68	81	70	79	82	68	70	87	92	70	72	99	97	89	75	82	59	94	91	94
68	59	87	64	81	87	70	93	76	63	80	84	84	102	67	50	47	49	75	76	77
76	59	63	60	81	81	73	77	77	83	75	79	94	78	82	49	83	53	70	76	93
67	82	60	97	86	77	99	68	82	72	88	70	72	76	86	47	72	81	85	87	78
65	66	59	93	74	86	80	87	72	64	77	73	72	82	73	66	53	69	70	72	88
58	76	83	81	54	83	69	68	74	69	72	85	71	90	69	67	94	62	99	89	87
79	112	72	65	57	89	71	75	82	66	82	76	93	84	86	87	72	95	100	84	79
74	96	75	82	70	84	77	98	96	92	89	91	83	95	89	104	85	84	74	83	103
99	81	91	89	58	86	97	91	89	99	88	80	103	109	89	72	70	106	76	90	107
90	84	90	78	56	73	76	114	100	89	100	94	112	108	82	89	71	105	94	88	108
92	95	84	85	63	109	97	123	108	83	88	81	111	89	88	94	92	74	88	99	92
84	66	83	88	80	112	110	110	94	89	95	94	104	98	77	84	103	83	102	113	111
81	81	84	104	56	98	105	109	100	95	100	95	101	106	85	85	71	78	106	101	106
55	88	83	105	80	98	99	99	106	117	86	76	101	94	88	68	77	87	85	107	107
90	84	84	75	70	93	101	86	97	97	88	87	98	81	99	84	69	66	85	114	88
84	71	74	63	86	94	107	76	89	94	95	97	110	99	88	81	91	80	95	93	92

84	59	57	92	78	115	98	86	104	93	99	82	118	88	93	69	71	79	87	88	93
72	78	77	90	68	99	104	83	122	93	116	81	121	86	86	64	69	60	78	113	87
90	70	63	76	70	112	100	92	104	89	90	84	110	94	83	87	87	71	86	105	87
84	63	55	69	52	103	98	84	93	89	85	75	96	110	94	100	71	96	100	91	110
63	59	92	60	48	85	95	86	87	80	86	80	93	101	94	89	72	89	105	113	100
88	78	87	84	65	97	77	124	90	86	98	95	110	108	82	66	90	81	78	100	86
82	85	86	98	52	116	77	118	100	97	104	104	123	100	92	78	73	70	94	103	92
66	83	84	97	47	110	74	111	111	109	88	74	99	89	87	67	68	74	79	95	106
62	67	72	92	60	114	73	110	101	101	87	83	103	86	86	84	78	92	80	111	110
62	61	58	108	50	115	73	109	87	93	90	86	108	84	88	65	94	70	101	105	93
66	53	67	94	50	109	100	105	93	82	83	72	116	81	95	69	74	71	96	93	82
66	59	56	75	73	115	101	96	109	82	87	70	98	81	95	66	81	74	73	94	105
84	53	72	66	51	108	102	79	90	87	87	72	94	88	69	79	75	74	85	93	81
84	52	62	61	82	103	91	78	93	80	81	80	97	87	76	63	68	70	75	103	89
73	73	52	62	73	106	73	96	92	96	106	78	98	93	82	83	67	83	79	94	87
67	59	47	108	50	90	71	78	111	92	82	81	95	82	81	71	91	77	85	112	83
69	58	87	91	47	82	69	88	84	85	78	77	107	80	82	64	72	65	77	107	88
62	87	81	71	47	84	93	80	83	91	82	87	98	83	82	65	74	71	80	105	87
73	86	73	66	50	93	98	79	89	92	97	84	92	84	84	62	69	72	72	94	89
77	82	60	66	52	85	79	79	92	84	88	78	89	91	98	65	71	67	84	112	89
62	59	52	64	77	77	72	90	88	84	79	73	99	86	69	77	69	74	87	109	83
58	58	78	66	60	78	80	97	82	84	93	98	92	80	64	87	66	74	72	86	94
72	77	84	60	48	84	71	102	89	78	76	70	119	75	69	58	87	68	67	79	100
65	91	50	57	43	105	101	91	86	74	69	70	121	86	98	48	63	77	76	74	99
55	79	50	97	49	82	102	72	81	72	65	62	84	105	77	59	60	61	106	96	77
51	58	78	90	82	77	78	66	88	76	72	76	98	76	54	60	63	57	88	69	82
67	53	52	81	73	94	67	78	74	88	63	65	92	69	84	43	47	63	62	90	91
60	69	59	74	49	77	73	91	90	77	66	80	84	64	59	46	42	62	70	89	78
54	60	61	59	50	82	68	78	77	72	66	67	94	87	55	62	56	87	70	89	71
64	83	96	76	47	110	66	80	76	67	74	62	79	74	75	50	66	58	75	71	73
89	59	80	69	50	102	66	85	73	79	78	66	71	64	64	71	50	52	88	71	78
68	55	51	64	51	85	104	75	72	68	66	68	92	71	93	49	56	83	64	76	81
57	58	51	58	59	80	88	97	63	78	79	77	75	70	84	50	57	77	63	77	95
56	84	56	77	74	92	70	78	68	78	68	64	79	80	70	48	57	66	70	64	81
59	76	78	86	72	78	74	74	100	68	72	72	77	82	63	42	50	60	72	70	78
61	61	60	62	47	74	94	75	72	73	77	62	69	76	68	95	56	64	72	87	70
72	58	73	66	48	78	79	77	74	74	73	66	63	70	69	76	80	63	70	73	83
81	56	60	68	53	103	74	76	78	84	73	81	93	67	74	45	46	67	88	69	78
67	47	59	62	68	85	68	94	69	70	71	67	81	68	71	49	55	53	76	82	84
57	56	83	60	52	78	66	80	89	67	68	64	69	96	70	52	55	52	70	59	84
60	78	64	65	50	87	98	75	68	72	66	78	61	78	58	53	61	60	64	65	67
55	74	65	65	50	99	78	74	66	67	84	61	77	76	68	56	47	81	75	67	77
72	52	79	63	55	82	70	81	72	66	70	65	74	87	87	65	57	70	70	63	77
57	51	62	92	88	85	67	93	88	60	64	58	75	67	70	40	83	51	65	65	91
54	76	58	86	63	77	72	102	71	67	67	59	69	66	63	37	76	80	69	88	80
65	75	76	64	49	77	68	95	72	67	59	83	63	76	66	43	52	63	72	68	76
55	50	69	62	51	97	62	79	66	83	82	71	75	69	80	61	59	58	68	107	70
55	51	63	93	52	106	96	100	77	66	70	74	105	82	61	69	47	52	66	92	73
55	81	57	69	58	79	90	86	71	69	71	69	79	78	60	46	52	52	84	64	87
83	87	61	67	50	78	72	74	66	82	80	69	94	72	88	65	92	70	90	83	77
81	65	61	65	49	74	66	78	81	72	74	68	103	84	87	68	71	79	94	70	75
76	55	82	98	91	85	90	74	99	69	64	85	75	100	68	70	62	69	71	63	90
55	87	89	95	81	84	79	78	66	63	85	80	73	71	97	51	51	69	69	101	76
53	65	64	68	86	77	68	90	66	70	71	68	76	89	73	56	88	52	63	64	93
79	62	60	67	62	82	69	100	93	65	67	63	82	82	69	76	99	53	81	108	93
91	82	89	92	57	111	68	84	75	84	70	93	74	81	70	91	65	84	75	93	82
81	101	96	76	52	86	95	91	87	87	102	96	100	112	103	81	69	102	70	72	92
91	94	86	80	69	98	104	125	106	104	117	85	110	114	104	89	79	86	80	82	122
81	90	84	96	88	108	104	125	114	83	99	102	127	111	76	75	59	87	104	112	109
78	86	86	107	91	114	103	119	107	84	102	87	117	104	100	77	84	104	110	104	93
87	73	91	101	79	92	94	105	92	103	106	92	125	96	92	64	72	85	111	86	110
77	66	65	83	71	103	109	127	90	104	103	69	122	98	88	83	79	79	106	100	92
53	52	74	100	72	115	102	109	84	107	91	83	108	78	97	70	67	70	106	94	86
50	85	67	106	47	123	102	109	96	102	84	74	113	100	77	66	87	77	113	111	109
82	88	78	91	49	105	96	116	90	91	81	91	103	90	70	76	88	74	91	119	98
75	79	62	79	79	108	112	110	89	94	86	102	113	87	83	85	57	79	65	94	79
51	59	86	101	72	106	108	105	113	84	96	87	105	96	89	98	81	68	99	89	81
74	54	84	102	50	113	83	102	115	80	79	80	103	111	84	90	89	77	64	112	72
61	50	75	94	50	108	68	89	110	77	80	82	124	96	111	86	82	82	91	118	81
54	54	59	96	51	110	95	90	83	86	99	82	110	79	103	80	64	62	76	116	84
57	76	79	91	51	108	91	94	89	103	115	85	101	86	90	84	75	89	65	109	104
61	89	92	67	83	99	104	119	81	85	97	81	107	82	66	71	70	91	99	87	108
82	86	78	93	75	114	103	118	81	79	78	92	119	88	93	66	66	69	103	104	87
89	83	69	95	54	111	87	110	118	101	89	83	115	104	77	65	68	59	91	91	85
76	78	62	82	73	109	77	102	109	103	78	83	107	89	79	88	68	79	60	94	88
76	73	69	74	75	110	96	95	94	88	90	73	102	84	100	65	88	95	64	87	74
74	54	67	81	71	97	75	72	86	81	76	80	105	82	76	66	87	65	66	103	95
75	48	65	77	60	93	84	81	75	81	81	73	110	89	100	71	68	77	88	93	112
78	49	74	82	60	93	94	99	81	76	78	79	108	78	82	70	73	75	89	105	90
74	50	63	81	51	84	70	91	81	82	78	86	104	80	83	67	67	67	67	82	77
67	49	74	91	47	95	77	98	81	93	108	77	101	96	76	69	67	65	70	111	79
60	77	74	83	47	85	96	86	103	100	105	70	103	87	86	58	66	65	76	89	79
46	59	85	88	47	96	89	94	69	85	86	76	95	80	98	70	88	78	84	102	65
46	73	69	81	67	94	71	103	96	85	87	88	96	68	76	64	85	79	73	97	80
66	83	65	86	48	82	74	81	87	78	88	69	80	85	84	71	66	77	68	90	104
81	90	58	88	71	83	73	82	81	94	92	75	84	72	77	85	49	81	87	80	74
55	74	86	84	82	97	94	81	93	100	69	87	83	77	71	59	84	51	78	84	81
47	88	64	74	81	89	70	88	100	75	74	86	117	107	90	60	80	73	94	113	76
90	74	49	86	72	98	70	85	83	59	93	88	94	104	101	43	57	59	72	109	110
81	59	74	88	66	82	98	77	82	96	79	69	89	77	65	45	52	55	83	80	97
47	51	50	68	52	84	85	92	70	73	59	86	103	65	64	71	47	92	102	64	92
51	49	51	64	52	88	65	74	78	68	72	72	71	77	70	49	55	51	65	66	60
52	51	72	76	46	81	69	74	82	63	84	68	94	69	70	43	50	46	63	66	77
78	75	51	81	47	79	65	86	74	68	79	62	66	62	62	38	80	87	74	83	73
76	71	47	68	48	98	66	80	92	63	70	67	73	82	60	67	49	64	61	82	90
55	56	50	91	65	84	84	80	70	69	68	76	110	75	72	50	54	52	64	68	75
51	53	95	68	44	73	64	91	107	104	100	92	91	85	61	85	53	52	71	80	113
57	51	78	76	86	75	62	69	83	74	73	69	69	80	66	88	74	80	64	75	74
60	61	55	69	84	70	65	71	76	64	64	71	59	63	68	62	49	56	67	70	80
49	51	56	78	70	75	97	72	78	69	63	74	66	85	84	47	53	47	83	71	80
52	52	53	65	50	76	75	104	77	75	78	78	77	73	59	49	58	74	62	89	80
68	72	48	58	51	91	64	92	70	69	68	63	58	67	61	52	44	94	80	62	78
51	61	79	63	46	76	67	66	93	80	76	74	71	86	74	75	71	67	63	59	73
54	57	61	55	48	75	66	76	75	68	68	68	63	73	59			58	64	99	88
52	52	52	54	59	81	66	81	69	62	68	64	60	74	87			50	98	76	68

	77	52	74	72	50	78	64	73	73	67	80	83	64	73	59			50	79	60	81
	65	87	69	94	56	74	91	67	77	68	72	65	61	97	56			53	61	67	76
	27	71	60	59	45	85	68	73	70	73	70	64	64	68	36			82	65	74	76
		60	81	56	89	72	64	66	83	68	65	69	66	63				60	69	71	76
		62	86	54	79	99	65	89	71	76	65	73	79	69				55	63	69	74
		51	61	55	64	100	65	78	72	75	89	69	107	65				47	77	69	64
		54	54	55	44	79	61	70	87	71	73	86	87	80				48	64	72	88
		86	55	60	44	70	66	75	78	85	74	85	62	101				72	71	67	75
		67	96	107	49	66	94	85	69	93	69	68	110	85				64	80	64	74
		52	61	77	70	74	74	72	72	67	76	69	72	72				57	65	73	92
		67	64	82	89	70	66	68	91	66	71	82	90	73				54	88	61	74
		59	60	78	81	87	64	78	86	67	87	88	88	91				79	66	109	64
		80	58	62	58	106	61	101	74	71	70	74	82	80				61	95	99	89
		81	58	96	49	77	88	80	71	82	64	73	85	71				55	64	59	79
		71	92	71	58	73	94	71	77	65	74	78	76	71				84	64	71	86
		104	101	58	69	75	74	71	93	73	75	67	112	88				62	70	71	80
		104	93	58	57	80	75	107	85	73	87	92	91	106				105	65	67	104
		94	91	91	79	102	83	108	97	93	91	111	111	95				101	101	84	108
		90	82	113	89	99	97	127	110	100	113	83	107	104				100	81	107	116
		82	88	104	83	105	101	108	109	101	100	93	138	112				97	111	79	93
		75	85	85	79	91	101	97	104	101	98	97	108	110				98	102	111	102
		61	79	93	76	104	100	90	78	95	85	84	106	97				85	100	118	80
		54	70	111	51	87	75	100	92	98	97	86	109	102				86	76	106	90
		50	58	92	75	85	83	117	88	90	86	91	98	83				89	89	86	102
		50	54	64	87	94	77	110	79	79	103	85	132	88				64	70	102	103
		69	85	56	78	82	77	100	96	93	91	89	122	94				71	78	119	92
		78	69	57	75	81	88	86	114	88	114	109	97	82				77	71	103	81
		85	77	49	54	114	96	92	112	86	102	91	96	87				90	89	97	78
		84	64	105	53	104	93	116	90	86	98	81	87	117				82	105	82	87
		68	51	110	51	99	95	109	86	88	76	86	93	94				65	91	80	86
		49	56	100	48	90	101	92	96	76	86	78	126	76				76	99	113	84
		44	65	97	96	75	90	81	107	99	81	74	117	78				70	75	118	94
		46	54	105	61	72	81	88	82	101	77	82	106	90				61	69	102	95
		49	58	86	50	76	73	91	99	94	84	70	92	101				66	76	82	111
		48	53	85	49	70	89	91	76	79	88	73	98	78				62	71	95	104
		75	53	81	47	71	91	96	94	73	78	86	107	77				61	70	84	97
		82	81	67	49	69	99	105	80	88	78	85	104	93				70	93	90	70
		87	79	62	49	72	77	87	91	76	103	70	89	72				68	112	108	88
		69	85	57	48	75	70	93	78	93	102	86	108	83				73	89	89	84
		48	74	52	78	75	76	105	96	75	95	80	99	85				72	79	88	84
		46	80	56	66	102	75	75	92	76	89	72	99	85				59	78	78	78
		52	69	51	54	92	81	76	79	84	88	66	94	78				56	92	100	70
		75	51	59	50	74	72	89	81	81	79	81	103	77				55	90	83	76
		89	45	57	50	74	87	76	80	67	77	83	88	75				49	83	99	95
		70	45	91	48	82	85	89	84	88	90	74	101	76				83	65	97	92
		60	44	106	50	76	65	80	79	77	73	69	97	76				81	85	72	88
		44	74	75	62	75	78	80	77	102	103	78	72	82				58	76	85	68
		44	72	55	80	95	72	93	83	87	76	70	89	84				52	76	77	78
		46	79	55	85	114	66	100	117	65	88	84	96	105				81	79	64	86
		47	68	71	83	98	66	99	110	84	70	78	112	88				56	101	86	72
		47	46	111	72	103	70	74	77	65	59	70	82	65				47	90	97	85
		51	47	89	42	67	84	68	70	57	61	68	70	55				48	67	65	68
		76	46	80	47	74	64	77	68	76	68	65	67	66				50	85	59	72
		71	51	57	46	68	61	66	63	64	88	70	72	75				58	61	104	104
		74	57	49	47	68	58	89	100	64	65	72	81	58				87	62	66	86
		66	57	48	47	66	65	67	70	69	65	77	66	71				55	92	63	65
		52	51	56	50	74	59	62	74	59	67	74	95	60				46	57	78	71
		50	55	81	81	73	64	69	64	86	78	63	74	94				48	86	60	66
		49	53	53	76	73	63	75	76	67	65	67	77	75				52	70	62	75
		75	66	54	56	96	65	61	68	63	65	66	64	58				56	65	101	79
		60	47	54	49	74	90	65	71	63	70	76	72	61				54	65	97	70
		57	50	55	48	69	81	99	66	66	58	64	70	70				53	91	64	71
		52	83	74	47	75	62	98	73	60	65	78	68	63				81	60	57	63
.		92	74	101	97	101	94	98	103	105	115	99	115	106 .		78	55	61	92	75	110
.		83	78	90	92	105	90	92	103	101	109	98	104	101 .		76	55	86	88	66	110
.		75	77	88	84	88	77	72	86	92	97	93	84	96 .		60	65	62	78	83	104
.		80	67	80	72	92	96	79	87	84	99	75	97	85 .		55	62	60	95	68	94
.		78	64	81	83	81	93	76	83	75	102	71	96	95 .		78	56	66	75	92	98
.		71	81	75	75	79	94	100	102	98	75	80	81	75 .		53	60	64	75	66	78
.		72	62	65	66	102	73	85	80	85	95	68	79	77 .		55	53	62	71	76	72
.		73	70	68	60	81	73	73	81	80	83	68	83	75 .		50	78	61	84	85	80
.		68	65	90	57	78	85	79	73	75	73	63	102	71 .		78	64	70	77	90	96
.		65	69	64	82	78	78	96	81	83	79	69	85	79 .		64	60	72	85	73	82
.		86	63	68	73	84	81	77	81	86	81	63	88	83 .		59	71	59	74	88	96
.		76	76	69	70	101	95	83	82	76	74	77	94	80 .		71	60	76	78	76	86
.		92	86	67	57	85	77	79	80	79	85	92	89	78 .		87	66	106	79	103	91
.		84	91	90	80	81	80	110	100	87	77	109	102	113 .		71	72	101	84	94	92
.		85	86	96	83	88	77	121	109	102	100	104	116	114 .		79	79	94	110	109	112
.		79	76	78	67	123	105	122	86	91	104	104	126	103 .		86	79	87	83	94	108
.		78	79	84	73	116	111	121	88	90	102	93	118	97 .		73	74	75	86	108	82
.		68	78	67	85	108	107	98	110	87	107	76	114	95 .		81	89	67	93	97	105
.		76	68	67	83	98	103	110	91	99	89	88	92	89 .		86	74	69	88	97	104
.		62	75	70	74	85	102	97	98	120	94	92	90	83 .		78	77	91	91	98	88
.		69	82	62	79	86	108	73	94	97	95	73	113	88 .		74	78	93	85	89	80
.		75	83	90	82	83	99	78	95	90	97	74	114	93 .		73	97	69	96	115	87
.		67	95	90	90	85	97	72	97	97	91	89	87	81 .		73	97	72	93	115	97
.		93	68	73	84	78	95	90	91	93	110	97	83	77 .		72	71	86	110	95	86
.		105	79	68	68	111	96	83	97	86	108	91	85	94 .		89	73	93	82	84	86
.		86	75	66	79	104	77	85	118	109	96	70	90	84 .		96	72	92	86	93	97
.		82	74	90	65	84	84	78	104	109	89	83	83	83 .		74	84	63	92	98	90
.		73	69	90	66	85	82	78	109	97	99	80	92	111 .		71	77	65	88	89	119
.		71	68	90	58	78	69	120	77	85	88	96	89	95 .		95	71	72	89	103	92
.		75	53	66	58	79	73	117	91	84	93	99	89	79 .		85	88	87	107	92	102
.		92	70	62	61	118	72	107	103	93	89	78	117	77 .		74	95	99	107	117	112
.		80	62	79	75	121	100	108	102	83	91	90	88	78 .		73	84	71	89	87	104
.		80	81	96	80	115	102	102	91	108	102	81	99	77 .		83	75	83	87	93	87
.		77	79	97	63	114	77	103	91	103	99	80	112	88 .		72	75	66	86	98	97
.		87	70	91	62	114	66	92	91	99	103	98	84	89 .		70	79	83	92	91	100
.		73	55	84	60	107	69	76	90	82	77	90	85	83 .		72	79	86	83	85	87
.		74	51	83	64	95	65	71	109	98	84	68	97	91 .		68	73	72	81	110	78
.		75	52	61	64	82	100	68	100	82	86	69	86	110 .		72	89	74	93	106	96
.		70	77	73	78	78	101	78	93	85	87	91	113	79 .		73	79	69	100	96	88
.		79	79	61	81	83	92	89	91	94	91	79	111	82 .		75	79	60	86	84	83
.		68	69	77	74	84	88	87	105	91	86	91	83	78 .		80	71	80	92	88	95

.	72	62	69	58	82	76	70	93	110	93	75	91	78	.	68	68	72	84	93	97
.	63	53	62	63	78	69	71	82	94	97	96	105	85	.	67	78	72	103	84	114
.	73	67	65	59	77	70	103	94	78	82	79	107	88	.	79	78	83	92	106	98
.	73	59	93	73	77	68	108	83	104	93	67	73	107	.	58	86	51	67	106	75
.	57	53	81	79	105	93	94	77	99	87	89	110	106	.	65	84	56	65	77	107
.	66	60	83	75	83	92	71	77	73	71	71	102	72	.	55	55	54	70	75	94
.	69	53	58	62	81	77	68	93	83	71	76	89	78	.	58	57	56	67	69	87
.	70	71	55	60	78	70	65	81	77	80	65	75	78	.	60	54	69	79	75	83
.	60	54	66	60	99	70	84	87	81	72	71	73	76	.	68	58	53	73	69	76
.	64	66	80	66	104	65	68	87	76	81	75	77	73	.	58	51	78	66	81	90
.	60	60	59	59	94	92	71	93	70	70	70	72	80	.	62	51	56	68	86	83
.	61	66	63	69	78	83	72	80	78	77	82	101	75	.	56	54	73	77	76	79
.	73	73	61	66	74	76	90	78	93	65	82	72	90	.	55	79	58	65	68	86
.	61	66	72	59	86	69	89	77	76	80	71	71	81	.	55	54	61	68	76	80
.	62	58	74	55	72	95	71	97	72	69	70	107	84	.	55	55	60	75	92	88
.	74	76	81	58	90	72	72	70	70	67	66	77	79	.	73	59	74	70	83	76
.	58	65	61	67	102	66	72	75	83	85	75	73	75	.	56	50	60	89	68	80
.	55	81	77	55	83	84	77	76	86	69	78	99	98	.	63	58	69	66	68	80
.	56	74	66	56	81	77	73	92	75	90	75	77	73	.	62	62	85	68	71	85
.	65	74	60	54	77	74	71	75	70	70	71	82	72	.	57	79	54	79	72	81
.	62	61	64	58	78	85	88	75	80	79	58	107	102	.	72	65	58	86	79	82
.	65	63	62	52	73	91	103	82	75	85	75	81	84	.	89	59	63	73	67	82
.	61	68	67	86	91	77	87	87	79	70	69	75	74	.	61	56	60	79	71	79
.	86	60	84	80	101	75	72	80	74	76	69	86	80	.	54	62	52	80	80	92
.	80	63	61	65	88	75	68	74	74	68	75	78	75	.	62	58	75	73	67	77
.	72	62	66	69	77	83	73	78	73	69	67	87	72	.	59	71	53	66	67	101
.	64	59	87	63	78	71	67	80	80	71	69	82	77	.	53	52	77	72	67	84
.	56	54	66	62	78	78	91	80	95	90	90	84	78	.	65	70	84	70	73	76
.	59	75	63	78	83	92	74	102	77	78	67	82	79	.	60	62	52	93	67	80
.	61	70	65	76	78	90	68	80	70	77	66	108	101	.	57	53	55	73	78	94
.	69	57	77	63	77	74	72	75	82	81	81	86	83	.	70	60	95	86	90	80
.	55	56	68	67	96	72	103	88	72	81	80	84	77	.	77	61	67	73	73	90
.	64	77	73	62	100	68	108	84	91	69	92	82	77	.	63	57	60	69	80	82
.	78	62	81	76	88	95	109	82	80	105	73	78	78	.	62	67	65	90	84	92
.	64	58	74	66	77	77	101	109	94	109	99	89	83	.	56	62	67	67	77	99
.	62	65	83	67	85	70	92	85	80	90	87	120	101	.	76	63	104	73	77	92
.	87	92	73	83	90	83	80	82	81	76	86	85	78	.	67	81	106	89	84	91
.	94	94	84	95	89	85	96	83	86	94	80	99	97	.	93	67	97	78	115	100
.	88	91	113	96	108	110	115	99	116	105	88	108	132	.	94	87	93	110	114	105
.	119	91	109	89	114	109	115	116	97	99	101	128	130	.	88	93	81	121	106	112
.	81	87	104	90	115	105	109	87	105	115	104	138	102	.	67	97	75	103	102	92
.	79	81	104	89	84	105	109	100	92	92	90	122	99	.	74	73	75	96	99	109
.	74	84	90	71	100	103	108	102	88	99	85	93	113	.	70	71	86	89	115	95
.	66	80	103	84	120	107	101	92	79	85	92	111	88	.	73	80	98	79	92	93
.	62	91	101	81	113	105	85	101	90	103	75	117	83	.	86	90	81	80	94	85
.	55	68	95	81	106	92	85	85	103	107	80	107	89	.	100	101	76	106	119	97
.	61	74	78	85	96	97	84	90	99	83	106	91	77	.	91	90	82	109	112	95
.	57	58	67	71	97	78	88	91	96	81	89	101	76	.	67	73	78	96	105	95
.	95	69	64	64	89	71	91	89	89	95	68	107	85	.	79	68	63	81	106	97
.	90	61	65	61	123	73	120	106	90	91	85	108	99	.	93	79	86	93	112	84
.	84	67	105	60	118	88	109	111	90	93	77	107	102	.	86	91	74	100	112	86
.	87	79	103	71	116	116	102	97	96	106	79	106	94	.	71	96	97	113	107	101
.	75	81	97	91	125	107	113	92	98	80	80	101	104	.	70	71	64	107	99	114
.	62	78	79	82	106	100	95	78	90	79	80	102	106	.	70	71	58	75	103	112
.	55	71	73	76	109	96	100	92	85	86	76	95	102	.	75	72	80	82	107	101
.	55	57	82	65	90	99	80	109	86	93	79	114	87	.	84	71	65	87	95	81
.	55	67	107	65	79	81	73	77	102	95	86	128	75	.	70	75	88	76	121	91
.	61	58	101	61	90	78	68	90	97	101	101	110	94	.	60	71	81	82	89	87
.	73	58	90	77	89	74	83	83	99	78	106	94	77	.	73	79	80	82	100	96
.	73	56	99	67	108	77	84	83	94	92	78	112	94	.	69	74	86	100	88	87
.	74	75	97	66	99	72	70	90	86	90	96	106	88	.	65	71	59	82	95	87
.	58	79	85	76	88	94	72	79	90	79	92	95	87	.	65	74	60	90	114	84
.	61	87	71	59	113	77	106	86	85	90	86	98	87	.	73	89	60	76	120	93
.	82	88	79	68	109	74	105	84	95	96	78	115	89	.	68	77	64	88	101	91
.	89	89	72	92	95	98	95	84	84	73	92	96	100	.	66	60	89	105	115	72
.	95	76	100	85	77	100	95	81	80	85	83	106	111	.	68	81	64	87	120	96
.	84	67	93	68	76	95	69	102	90	80	101	96	101	.	66	85	59	93	107	98
.	74	52	73	71	83	88	71	106	77	90	91	105	70	.	63	54	74	78	84	101
.	93	58	72	59	100	94	66	100	84	90	70	114	75	.	92	61	54	81	101	119
.	73	54	66	79	76	98	94	78	96	78	95	119	83	.	93	59	51	69	82	113
.	66	55	85	64	77	96	68	102	74	73	66	90	82	.	86	60	58	104	98	95
.	62	72	66	56	76	90	71	72	83	81	74	104	79	.	56	59	90	104	76	96
.	55	85	67	60	101	76	70	74	76	66	74	89	99	.	57	77	76	80	84	80
.	56	71	83	56	97	84	80	80	78	65	82	70	78	.	51	54	52	91	81	71
.	67	51	72	62	82	72	72	79	67	72	68	81	83	.	62	62	54	62	79	84
.	61	56	69	65	78	71	69	83	76	76	79	89	85	.	54	64	77	66	82	89
.	56	49	83	67	86	84	76	78	73	92	79	83	74	.	69	58	78	86	96	75
.	80	54	91	73	77	83	84	83	71	69	78	89	78	.	52	76	51	65	81	72
.	77	76	74	59	80	72	71	85	69	68	72	89	79	.	49	61	55	69	74	95
.	65	60	70	68	106	77	66	73	95	84	83	78	83	.	60	65	63	85	83	81
.	61	61	76	55	94	85	66	78	89	69	69	83	99	.	63	55	53	71	74	73
.	61	59	92	57	84	88	77	78	76	84	73	77	79	.	57	62	67	73	79	80
.	69	57	93	69	83	72	108	74	74	74	74	81	83	.	57	56	50	84	81	91
.	62	53	73	66	97	75	96	92	80	69	76	111	77	.	62	75	87	72	84	84
.	61	75	75	76	83	73	86	78	68	74	84	82	77	.	53	56	75	83	79	102
.	60	79	71	56	83	83	72	73	71	71	75	78	76	.	61	58	60	70	78	76
.	58	64	80	62	79	77	72	74	87	103	93	82	82	.	56	69	56	88	83	98
.	55	64	67	57	80	69	72	79	80	74	71	72	78	.	62	65	55	77	93	76
.	67	61	68	73	106	78	100	82	74	73	75	78	99	.	69	59	60	99	91	91
.	60	60	68	67	108	79	72	77	74	84	72	107	83	.	57	64	59	71	75	83
.	57	58	80	61	85	72	75	75	86	73	76	103	69	.	48	60	80	72	73	78
.	56	69	73	66	85	78	71	85	79	69	88	78	71	.	46	78	52	76	84	86
.	74	64	68	60	85	83	76	76	74	74	72	82	84	.	75	72	56	77	72	87
.	63	79	76	62	102	78	78	75	78	81	73	77	73	.	63	56	76	73	76	81
.	61	78	72	67	92	91	83	92	76	79	68	90	85	.	55	63	58	91	89	85
.	67	63	77	61	82	91	89	85	80	76	74	79	77	.	61	75	58	74	84	76
.	71	66	90	73	83	82	74	77	75	85	94	77	87	.	62	57	74	75	82	93
.	59	65	81	72	85	77	118	81	81	74	74	89	83	.	58	72	82	83	97	82
.	58	59	74	83	107	97	95	80	79	79	93	113	120	.	58	63	56	89	103	102
.	82	70	75	80	90	80	84	84	74	73	73	88	95	.	76	60	61	92	103	86
.	66	64	78	62	86	91	84	80	78	83	75	95	89	.	62	64	63	79	85	79
.	70	97	79	63	90	83	103	92	84	86	78	95	82	.	74	83	105	91	79	96
.	92	105	80	90	92	72	89	86	110	84	80	101	94	.	86	68	105	91	91	98
.	86	97	96	87	93	79	106	106	102	118	112	107	103	.	84	96	99	105	109	103
.	83	93	118	85	95	102	116	100	90	93	108	107	128	.	107	99	91	122	110	112

.	79	90	112	78	103	103	117	103	116	107	92	126	110	.	100	100	70	106	116	113
.	86	84	97	85	103	102	105	112	112	100	83	106	95	.	88	78	61	90	97	81
.	73	89	92	78	111	97	113	103	98	88	84	112	94	.	80	79	86	86	106	110
.	73	74	96	86	111	105	102	100	76	106	112	102	95	.	74	74	88	90	104	117
.	66	80	104	82	101	80	105	87	86	104	97	96	83	.	86	72	71	88	128	83
.	62	78	103	89	116	80	90	119	90	78	84	120	83	.	97	73	58	86	111	101
.	55	84	102	80	99	91	83	91	116	94	81	119	93	.	86	92	57	88	110	86
.	62	85	100	68	110	99	74	97	80	96	72	100	99	.	76	104	61	88	104	79
.	62	80	98	76	112	96	96	91	92	97	88	96	83	.	81	85	79	123	97	79
.	86	74	90	84	110	103	116	94	89	72	87	107	101	.	72	86	73	105	109	92
.	81	72	98	80	104	97	115	125	104	95	102	120	118	.	74	75	54	96	110	90
.	78	65	98	81	114	85	108	114	99	105	90	100	95	.	83	86	70	92	103	90
.	77	90	86	69	110	92	104	90	85	108	80	90	91	.	99	84	83	106	101	90
.	69	85	88	78	96	71	102	93	96	92	79	88	94	.	96	73	70	104	113	93
.	69	83	98	80	102	105	110	102	79	86	91	89	88	.	73	75	65	80	121	90
.	70	84	98	79	96	108	108	97	85	85	79	127	102	.	71	74	75	87	103	110
.	56	66	92	68	103	104	80	89	85	83	77	116	91	.	70	86	59	69	106	90
.	65	76	81	75	91	100	90	94	90	96	96	103	99	.	70	93	65	81	107	88
.	67	69	93	69	97	104	99	89	89	78	89	92	108	.	69	67	68	92	121	88
.	55	77	87	79	109	96	112	94	92	89	77	95	113	.	88	74	63	87	126	90
.	52	75	84	70	103	91	96	86	90	96	78	92	78	.	77	77	74	75	116	96
.	56	79	93	76	96	90	95	127	89	73	83	102	87	.	69	69	67	70	102	106
.	51	81	88	78	93	79	115	113	102	92	85	110	87	.	80	79	90	94	116	83
.	59	78	92	70	105	90	82	104	95	89	81	106	97	.	61	87	63	94	97	97
.	54	80	92	88	104	103	83	95	96	84	96	92	85	.	67	69	64	93	110	96
.	97	81	89	72	99	86	77	97	96	77	100	103	93	.	77	81	60	87	116	88
.	86	73	92	71	106	72	78	84	84	84	89	99	90	.	69	67	66	85	127	95
.	74	71	86	72	93	77	102	90	101	80	87	84	103	.	82	69	99	94	95	114
.	68	82	84	72	91	75	83	110	101	97	85	121	98	.	68	71	76	76	86	105
.	73	78	99	71	104	91	72	83	75	71	73	124	87	.	57	93	63	78	110	94
.	64	72	87	68	104	96	85	113	71	94	76	117	106	.	56	86	49	82	92	97
.	55	67	85	67	97	90	72	92	79	67	75	77	76	.	58	55	62	101	84	75
.	54	65	82	74	97	75	79	79	86	66	72	87	88	.	77	66	60	70	106	96
.	66	61	81	74	90	78	78	73	67	85	78	82	78	.	94	72	69	64	75	74
.	60	55	75	71	86	73	90	85	72	70	79	83	89	.	60	66	52	71	78	74
.	52	54	85	78	80	72	76	83	77	77	73	77	80	.	60	60	47	89	92	76
.	57	54	74	73	88	82	98	73	73	78	71	82	73	.	59	55	83	67	82	72
.	79	68	90	59	93	73	92	71	73	95	66	83	85	.	66	56	79	66	89	72
.	68	59	69	68	85	79	84	79	71	76	74	96	80	.	63	55	58	69	85	75
.	61	65	75	61	86	89	83	83	65	74	68	84	83	.	59	72	62	72	79	87
.	57	54	66	51	80	68	84	82	82	71	73	93	76	.	53	58	51	73	92	75
.	68	53	61	62	77	79	81	75	77	83	66	96	76	.	61	62	58	67	110	78
.	62	78	64	56	83	72	77	79	70	72	77	89	77	.	62	60	72	79	85	68
.	61	73	66	59	84	96	85	74	76	76	72	82	71	.	58	54	62	83	74	68
.	55	57	63	50	86	78	80	79	67	80	72	89	73	.	61	59	59	76	73	101
.	57	63	82	50	87	80	82	87	73	73	79	101	77	.	57	65	69	74	78	88
	70	73	72	59	97	78	84	72	75	84	70	83	72				72	71	73	74
	62	60	63	51	79	76	77	72	87	75	72	81	90				69	84	72	78
	60	58	60	57	84	85	104	72	73	72	76	82	105				60	69	85	83
	72	60	63	61	97	76	109	92	70	82	85	102	77				54	79	95	74
	77	58	63	57	83	72	97	74	74	75	79	82	73				55	83	73	88
	66	57	57	82	83	84	78	73	82	82	77	82	71				75	84	76	81
	73	77	71	75	91	78	72	77	78	73	74	94	84				80	74	83	78
	61	66	87	66	82	84	73	83	72	77	79	107	77				57	85	73	82
	66	60	82	64	82	92	82	71	67	75	76	82	77				52	70	97	84
	62	67	69	59	84	78	96	84	74	72	79	95	75				64	74	77	77
	83	76	70	69	104	84	83	79	71	82	79	106	74				59	71	73	85
	74	76	65	63	84	80	75	72	89	92	78	87	79				82	85	77	96
	89	71	64	59	79	96	86	96	77	79	69	84	72				64	106	96	85
	72	78	70	58	74	95	84	87	72	69	84	86	83				64	97	79	93
	101	76	74	79	98	84	96	78	90	92	76	83	106				66	73	74	85
	78	75	103	63	83	82	88	90	89	92	78	100	87				78	94	78	81
	97	78	98	77	79	87	83	87	73	96	77	88	84				110	83	90	88
	103	92	71	82	93	102	106	94	80	93	102	104	120				108	80	111	92
	97	89	88	85	110	103	114	113	94	113	104	102	116				103	122	107	120
	81	82	105	80	110	109	104	115	123	97	95	138	97				89	115	94	110
	76	79	92	81	99	110	109	111	106	97	96	99	95				84	92	99	89
	80	66	92	71	102	107	91	98	92	105	102	100	85				70	97	115	107
	69	87	85	79	107	96	103	97	89	108	98	101	102				65	84	122	108
	86	85	86	68	120	97	117	108	90	95	86	102	130				65	76	101	106
	79	77	79	56	109	98	97	110	92	96	83	131	116				93	89	103	90
	75	64	89	75	108	87	120	116	88	94	91	125	98				88	88	98	89
	74	59	71	74	101	90	97	114	83	95	100	113	94				78	88	98	90
	62	63	68	79	95	105	95	95	105	100	94	115	85				57	116	101	88
	56	58	67	76	122	109	100	89	115	89	89	123	90				72	110	103	84
	67	71	99	79	121	110	115	112	94	96	93	105	99				72	88	121	97
	69	71	104	67	109	103	111	108	82	89	83	102	100				65	95	122	88
	101	73	122	61	96	98	110	95	86	112	83	95	93				95	77	105	98
	99	74	117	53	94	101	115	91	84	84	109	99	91				85	96	95	91
	79	78	93	60	91	108	104	90	98	89	90	107	91				67	97	96	81
	72	84	72	58	91	92	96	100	90	88	82	113	86				78	96	100	88
	71	81	61	60	120	96	91	94	89	82	83	103	101				75	114	115	82
	67	79	62	55	108	98	91	92	93	82	99	106	120				78	98	109	100
	63	86	69	77	102	88	103	111	86	85	87	96	89				63	101	95	88
	56	69	72	76	83	101	110	88	94	105	88	98	87				70	93	104	91
	54	60	68	58	84	99	107	95	88	84	89	97	92				81	95	97	99
	60	61	78	61	88	104	103	86	100	83	88	97	84				65	95	101	116
	88	50	65	73	85	85	96	99	93	88	88	111	90				72	89	102	101
	90	60	76	84	114	92	91	103	99	99	74	102	79				89	95	98	79
	86	76	65	74	111	100	90	100	103	86	75	97	84				80	105	122	87
	89	83	71	79	83	92	83	92	99	86	95	90	84				81	93	118	88
	80	76	94	79	87	85	89	86	89	84	78	100	88				54	88	98	81
	70	63	97	66	76	82	78	94	83	79	77	101	85				67	82	86	112
	63	53	117	61	90	82	99	85	74	74	92	127	81				76	80	102	97
	69	68	115	63	102	86	94	110	100	81	81	97	110				68	81	87	76
	65	55	97	64	123	94	96	100	95	98	93	108	115				50	83	104	111
	64	71	92	61	98	90	79	87	80	97	73	100	103				55	107	79	98
	62	54	79	78	90	84	76	80	74	74	79	74	73				56	100	79	83
	58	49	91	73	83	88	79	87	71	78	83	77	75				79	75	78	84
	73	65	77	68	79	79	78	88	77	75	84	77	79				55	72	75	80
	79	57	66	65	84	73	76	77	70	71	67	74	90				95	70	67	82
	65	59	60	61	93	84	75	73	71	76	76	92	68				66	76	75	70
	64	71	67	60	100	93	96	94	70	71	76	81	80				56	77	82	89
	61	65	67	60	93	76	84	75	80	70	77	87	96				71	80	82	81
	95	57	67	61	84	89	77	82	71	77	73	84	73				85	73	77	76

76	56	64	71	84	84	79	85	69	87	71	90	75				66	72	77		75
61	79	63	66	78	80	78	78	77	81	67	78	69				56	76	92		77
63	67	74	62	92	77	73	78	72	79	76	80	80				62	65	77		83
66	61	66	67	86	95	78	71	73	87	69	81	74				61	80	78		89
64	61	67	68	83	84	77	84	71	74	75	81	75				58	93	103		80
		62	68	80	86	74												74		
		71	51	83	78	85												85		
		72	77	79	75	77														

2	2	6	6	8	9	9	9	11	11	11	11	1	1	1	2	2	5	6	6	8	9
Box-6	Box-8	Box-8	Box-6	Box-5	Box-1	Box-2	Box-6	Box-1	Box-2	Box-4	Box-6	Box-7	Box-8	Box-2	Box-4	Box-7	Box-3	Box-1	Box-4	Box-7	Box-3
23.3	27.5	37	46	50.1	62	63	69	74	76	81	83	1.1	2.2	5.5	19.1	25.1	34.2	38	43	52.2	64
M	M	M	M	M	M	M	M	M	M	M	M	M	M	M	M	M	M	M	M	M	M
H2O	H2O	H2O	H2O	H2O	H2O	H2O	H2O	H2O	H2O	H2O	H2O	Malto	Malto	Malto	Malto	Malto	MD	MD	MD	MD	MD
MH	MH	MH	MH	MH	MH	MH	MH	MH	MH	MH	MH	MM	MM	MM	MM	MM	MM	MM	MM	MM	MM
58	86	88	77	52	51	76	53	125	109	104	111	.	101	86	60	103	38	55	52	44	84
54	80	63	53	87	60	68	92	116	85	88	103	.	105	94	66	104	41	98	55	65	65
59	75	76	59	54	66	59	71	118	72	61	71	.	103	101	95	115	89	85	87	90	65
68	106	56	94	75	96	67	88	123	109	62	98	.	106	71	66	87	87	80	88	79	103
102	102	79	96	69	101	94	106	105	110	112	68	.	98	66	66	96	87	85	64	42	106
92	74	78	95	55	68	70	84	76	73	112	67	.	100	111	106	107	88	87	76	43	103
57	72	78	89	87	91	113	62	117	107	99	63	.	103	105	102	77	89	66	93	92	103
77	80	75	76	53	73	98	86	117	87	96	92	.	103	93	60	96	91	87	96	88	101
84	102	89	85	61	60	95	106	123	69	71	112	.	103	112	58	89	89	93	66	112	81
61	97	89	94	96	110	107	126	123	112	125	106	.	110	102	105	70	93	88	58	98	83
56	74	66	100	79	113	111	111	74	98	111	88	.	105	108	110	63	89	101	75	95	116
105	65	71	74	77	113	107	111	93	62	107	105	.	104	117	114	64	94	95	74	104	109
110	106	79	82	69	115	104	114	129	99	114	130	.	102	110	72	93	91	94	64	96	104
102	79	59	59	60	111	107	114	132	63	103	103	.	74	119	78	69	90	101	84	100	110
107	65	82	66	73	111	105	117	115	97	107	77	.	78	107	52	64	91	97	76	95	69
113	73	89	73	77	101	105	109	81	112	114	81	.	98	115	108	101	84	98	99	90	96
101	72	90	55	83	111	104	109	141	85	111	88	.	67	112	124	105	82	97	70	97	116
66	74	90	48	76	112	105	108	140	58	114	85	.	82	106	119	71	54	94	105	88	102
54	114	78	94	77	82	90	104	134	57	116	129	.	108	103	107	56	43	99	104	77	80
78	113	78	99	62	56	87	84	132	103	119	133	.	110	96	79	57	74	90	99	78	48
75	90	72	88	52	60	97	49	135	100	117	123	.	112	68	54	58	82	61	99	73	51
87	65	79	53	82	91	97	47	132	130	119	125	.	104	62	95	80	83	51	93	46	87
96	66	48	45	61	72	81	61	124	118	103	106	.	97	79	82	93	78	51	89	39	95
66	67	61	68	76	56	50	54	128	104	109	121	.	100	91	60	105	53	89	95	44	65
50	92	54	60	74	71	47	56	125	113	104	120	.	78	73	48	100	45	54	87	83	49
57	72	53	77	60	103	44	90	122	67	105	114	.	57	78	48	100	45	50	70	60	73
71	79	49	58	75	104	59	107	123	56	103	124	.	54	74	93	72	45	82	60	48	50
58	62	46	47	61	92	86	102	110	112	92	123	.	56	86	66	62	49	84	57	53	75
56	96	59	99	82	99	59	56	92	111	85	123	.	80	73	55	54	69	57	86	49	58
65	94	50	94	66	98	88	40	67	71	60	123	.	61	84	51	59	57	50	77	48	90
69	72	51	58	58	91	73	84	69	59	60	118	.	56	89	54	64	46	68	81	87	66
84	66	46	48	78	56	89	61	99	94	66	97	.	91	60	67	72	47	77	67	92	46
95	60	84	62	81	52	82	70	70	72	68	96	.	67	70	55	59	55	54	59	77	83
84	99	101	48	82	110	46	58	70	120	92	118	.	62	68	62	60	83	82	72	45	101
55	86	76	68	77	91	42	99	98	137	72	96	.	57	92	108	68	62	95	54	41	91
71	101	44	78	69	62	45	88	78	124	62	100	.	96	87	109	96	46	81	64	38	78
65	95	39	56	47	57	49	48	64	99	62	89	.	104	60	68	106	59	48	86	88	53
59	78	53	44	56	86	96	46	67	57	95	62	.	91	63	80	88	50	41	77	66	50
57	53	72	64	65	61	92	49	71	73	98	70	.	53	72	118	53	76	46	50	43	51
55	63	47	48	51	62	70	49	116	63	89	68	.	70	79	104	55	80	56	49	40	54
88	96	47	76	52	100	48	77	118	100	56	76	.	55	69	82	78	58	100	51	49	65
59	96	48	72	66	89	52	66	102	102	88	113	.	56	57	84	94	45	84	55	84	89
76	60	79	61	70	67	50	56	63	64	57	95	.	59	63	60	65	46	47	75	71	82
97	62	60	52	52	55	49	52	58	63	57	64	.	55	85	76	60	44	48	69	45	53
79	63	48	51	56	62	65	54	65	61	72	67	.	89	69	88	55	49	49	46	45	48
56	93	52	71	81	60	45	59	68	64	76	63	.	63	64	62	60	48	59	53	52	56
50	85	71	55	70	88	51	55	99	73	55	61	.	58	58	56	61	47	84	63	86	49
56	60	60	62	51	60	85	107	106	88	62	79	.	57	69	52	61	71	60	54	56	87
61	67	48	51	58	59	55	77	87	70	62	64	.	54	67	57	72	82	48	59	48	79
61	67	60	49	53	58	43	47	62	64	61	69	.	60	82	104	55	47	50	74	50	54
89	71	65	65	54	81	44	49	64	66	62	69	.	58	63	102	51	49	52	49	75	55
67	100	48	48	73	62	48	50	61	65	93	66	.	53	60	67	97	46	73	52	76	57
61	73	70	50	74	77	55	60	67	74	59	64	.	55	53	55	96	66	56	54	69	45
58	72	67	71	55	84	81	55	68	61	60	99	.	92	63	56	67	72	44	54	49	54
58	67	54	46	47	61	48	54	79	68	62	68	.	78	60	60	64	50	50	68	45	86
72	86	48	49	48	62	45	89	66	66	61	65	.	54	63	55	61	46	49	53	57	61
61	65	50	78	70	60	44	88	62	64	55	67	.	56	71	100	71	71	77	41	80	53
59	70	69	61	75	94	48	52	61	68	66	60	.	54	83	74	61	58	62	49	77	55
57	64	48	57	58	87	44	53	62	64	67	56	.	50	61	62	60	47	47	65	48	48
90	79	58	51	52	53	49	49	75	61	70	61	.	51	64	56	53	46	50	51	46	62
99	106	62	77	74	60	47	49	94	86	96	68	.	88	62	93	86	44	73	43	57	91
61	94	50	83	57	59	74	78	70	104	73	86	.	72	99	73	102	49	49	65	55	60
51	66	43	49	50	87	88	93	67	80	61	69	.	54	79	75	61	73	48	42	77	48
98	72	75	50	77	74	78	62	66	71	61	62	.	57	59	99	62	61	61	59	66	55
95	74	68	78	49	58	46	52	62	66	87	58	.	58	60	69	67	47	69	66	53	89
62	71	52	52	52	61	48	85	80	73	67	76	.	54	63	66	95	56	62	46	51	60
75	83	61	53	75	85	76	82	68	75	60	62	.	92	88	84	79	65	55	60	51	56
67	91	78	60	77	83	52	62	73	73	66	70	.	93	72	117	66	83	90	72	56	70
67	108	66	95	59	62	78	79	96	114	76	102	.	65	87	120	83	85	81	80	82	114
90	78	72	94	62	97	95	103	70	121	104	91	.	84	109	88	97	79	66	81	88	110
103	71	59	76	74	104	92	106	92	125	89	72	.	99	104	115	101	85	93	67	75	113
97	91	68	65	62	105	65	83	112	118	98	97	.	99	104	120	115	89	63	71	56	104
89	117	66	77	69	74	87	54	113	128	101	105	.	111	89	124	99	72	60	89	77	100
105	112	74	57	79	92	112	55	79	114	100	71	.	116	92	119	103	89	79	78	92	98
106	94	83	67	63	68	98	81	68	125	72	109	.	109	97	108	111	85	78	87	81	93
113	105	74	71	61	83	76	87	123	118	76	115	.	111	63	116	100	89	80	64	55	50
104	100	95	91	89	75	86	56	106	108	83	120	.	105	81	103	89	78	91	83	65	81
72	77	78	114	65	70	86	78	98	83	66	120	.	99	93	72	73	83	66	75	80	83
59	72	78	93	67	77	80	106	65	66	95	108	.	66	70	60	62	81	83	86	86	65
58	104	88	82	87	106	89	102	99	69	104	115	.	93	69	57	71	81	96	90	70	91
89	77	94	60	81	89	76	66	119	104	102	106	.	65	92	70	62	82	71	96	82	97
91	76	56	67	74	91	55	55	114	116	72	110	.	62	64	113	65	82	86	87	87	89
65	79	80	55	70	93	52	67	118	112	111	77	.	103	97	107	81	83	61	82	93	96
76	71	61	72	69	82	72	78	127	83	103	61	.	84	100	71	90	74	86	97	82	90
73	108	59	55	66	99	86	60	104	103	102	71	.	103	93	59	97	54	102	81	60	84
68	106	86	90	64	104	63	85	114	97	104	103	.	97	62	84	72	83	92	70	43	84
99	82	90	91	91	109	68	101	76	97	90	72	.	104	52	113	65	57	93	68	68	48
100	67	73	82	86	98	84	97	61	73	101	78	.	104	59	84	59	48	55	81	61	45
104	67	67	47	57	97	68	73	110	72	90	107	.	84	64	63	57	60	49	62	56	84
86	82	75	56	47	103	70	55	114	76	65	108	.	55	91	59	76	59	78	59	64	66
65	77	71	49	54	98	101	52	122	107	56	75	.	55	64	61	71	53	70	92	61	63
68	73	56	66	82	57	92	69	87	108	61	108	.	57	63	61	62	58	52	87	58	52
88	70	63	78	83	63	61	61	65	78	61	112	.	60	75	65	69	73	56	56	70	74
82	68	51	56	79	76	50	56	68	75	63	105	.	56	87	71	67	52	53	56	88	62
71	98	71	78	49	66	52	81	59	72	58	74	.	74	92	66	91	77	42	55	63	52
65	84	49	54	62	70	68	97	61	71	77	59	.	58	63	67	96	59	46	60	46	69
63	69	59	67	84	60	47	87	70	88	101	59	.	101	66	108	81	61	67	84	44	61
63	65	48	51	65	65	48	52	69	90	68	91	.	96	92	104	65	78	56	78	50	47

101	114	38	71	61	97	82	47	120	122	72	102	.	61	71	72	61	60	45	55	65	60
120	96	69	48	60	97	93	83	134	103	94	90	.	54	74	60	79	78	52	36	86	100
69	99	63	79	76	90	72	69	121	102	68	59	.	46	54	62	100	46	81	57	81	70
53	88	56	59	50	56	46	52	108	76	52	59	.	50	59	92	100	43	72	50	72	47
85	62	53	73	43	53	41	48	93	66	44	89	.	89	65	117	83	52	46	44	45	48
82	57	67	55	50	60	41	77	60	87	51	67	.	98	81	100	52	61	50	37	43	94
58	56	46	48	59	98	54	54	51	107	52	60	.	68	55	85	58	49	84	43	46	63
53	58	48	66	52	64	59	53	78	84	90	64	.	53	56	54	54	50	80	90	55	47
59	86	80	49	52	54	87	54	61	77	85	83	.	51	57	48	56	45	44	62	51	53
62	89	70	49	79	95	76	97	99	69	53	62	.	51	93	102	88	54	42	40	79	67
65	61	48	71	75	89	48	84	79	74	55	84	.	55	78	106	49	47	48	38	74	58
59	61	52	49	63	56	48	52	62	77	54	70	.	64	55	68	55	46	81	49	56	88
63	60	50	51	58	54	59	55	63	68	66	64	.	79	58	53	67	64	84	75	59	90
60	79	61	73	49	60	46	54	63	82	52	63	.	57	51	55	90	77	50	45	52	64
60	66	71	45	63	91	51	84	65	101	61	64	.	51	93	59	66	50	47	43	53	45
91	73	61	67	57	66	83	63	68	71	59	94	.	52	60	60	54	50	48	41	59	49
82	56			72	52	78	50	67	69	54	89	.	51	53	57	65	48			67	59
59	65			60	59	57	53	119	73	89	59	.	52	58	108	58	57			45	89
56	62			49	55	46	84	111	91	78	65	.	90	54	79	61	69			47	87
88	95			49	82	48	59	89	74	53	64	.	81	82	60	62				66	53
71	95			59	59	48	54	94	73	53	64	.	48	53	56	70				54	48
62	61			58	55	53	45	69	73	52	98	.	51	52	57	64				52	55
62	76			76	53	47	49	68	96	53	88	.	54	55	63	63				87	54
60	62			55	90	77	90	68	90	55	62	.	53	55	62	61				84	73
72	67			56	63	75	68	68	73	66	68	.	54	69	97	101				55	61
67	64			59	60	44	51	67	75	55	64	.	90	80	81	80				67	55
62	93			88	52	46	49	59	64	56	91	.	88	59	62	62				60	55
86	103			50	61	50	55	66	72	53	68	.	64	58	80	61				54	76
80	72			51	89	48	90	79	76	76	63	.	53	83	83	76				68	54
58	67			76	85	80	84	102	75	81	72	.	59	65	104	59				80	62
58	68			58	59	66	47	68	70	57	101	.	56	59	80	62				57	93
92	81			55	51	51	53	64	84	57	95	.	57	59	65	75				47	67
81	95			53	57	48	65	64	109	57	68	.	62	52	92	92				68	54
66	98			81	77	65	81	69	72	61	65	.	90	58	80	107				62	75
67	77			77	91	75	77	106	83	69	70	.	68	93	78	69				56	102
77	74			79	68	82	59	107	113	81	80	.	92	90	97	65				84	89
75	115			57	101	84	95	71	94	108	74	.	106	97	121	103				88	86
100	85			68	80	94	100	102	120	101	97	.	76	78	116	92				95	109
87	95			80	104	72	67	106	125	90	83	.	94	89	75	86				64	71
92	107			59	79	92	61	94	119	91	101	.	102	80	104	92				85	107
107	80			72	82	89	86	96	100	101	110	.	96	89	79	115				83	99
94	115			65	93	70	73	89	71	81	113	.	73	67	101	89				53	81
67	104			65	104	74	80	80	101	69	96	.	98	87	96	90				68	92
100	102			84	75	84	102	111	131	73	77	.	100	78	93	71				83	92
101	72			85	96	76	85	89	86	105	101	.	87	77	119	102				62	80
66	73			61	86	100	61	106	107	107	101	.	91	104	100	106				52	53
65	82			86	81	96	85	118	89	105	108	.	104	96	77	79				52	80
90	103			89	91	74	73	94	85	101	89	.	101	78	71	72				76	74
81	76			79	101	99	74	118	88	96	108	.	108	68	85	70				84	61
76	65			59	97	71	107	95	116	84	116	.	101	96	119	80				96	81
82	81			84	98	73	68	111	114	71	97	.	66	73	107	86				83	84
111	85			68	70	91	63	111	118	67	111	.	62	69	84	76				55	93
101	70			87	74	70	56	108	107	76	118	.	83	66	70	100				45	98
86	67			83	89	66	70	89	96	104	105	.	73	69	93	108				56	88
60	101			57	98	79	89	118	97	94	101	.	81	64	70	84				48	56
84	85			63	98	97	96	113	96	72	93	.	72	92	100	67				46	96
85	73			79	0	0	-1	122	66	83	77	.	61	96	102	90				65	-1
65	63			63	0	0	1	86	77	65	100	.	62	88	75	66				52	0
66	84			60	97	81	104	82	80	60	71	.	96	57	65	84				75	40
70	71			64	67	56	81	124	99	60	83	.	79	83	59	77				66	51
84	96			79	69	62	55	114	73	59	90	.	82	70	58	97				51	68
72	106			78	89	81	48	75	68	110	75	.	90	69	62	84				48	53
69	95			58	87	64	52	64	62	108	109	.	84	73	59	77				75	54
101	61			64	69	74	54	73	86	96	85	.	57	67	72	76				60	55
78	69			54	88	72	64	65	120	82	76	.	47	65	66	72				44	71
55	87			79	89	81	56	62	86	55	91	.	72	90	66	67				40	95
53	65			88	98	64	96	96	67	47	106	.	101	107	107	84				84	65
60	61			50	87	59	61	105	63	56	83	.	88	64	105	92				88	50
57	62			46	59	48	81	103	92	95	86	.	56	55	88	86				85	40
66	67			49	78	90	85	68	67	74	75	.	51	71	60	53				72	50
54	91			69	59	74	61	51	66	51	73	.	56	62	86	52				60	61
96	101			55	60	41	47	61	109	53	111	.	69	56	91	63				44	108
88	94			50	85	42	48	63	102	54	67	.	55	62	72	56				42	65
60	72			58	90	43	56	61	63	55	60	.	53	75	54	62				47	42
53	70			57	67	67	55	93	61	73	76	.	79	58	55	57				71	51
57	66			56	54	48	52	61	65	60	67	.	60	58	61	57				73	53
54	74			69	60	44	55	61	92	57	67	.	59	79	78	69				57	81
56	66			62	54	47	90	69	70	58	92	.	58	63	102	59				56	48
82	70			60	61	77	67	90	72	60	89	.	59	63	66	62				57	54
102	91			60	85	82	54	122	80	94	60	.	58	61	53	61				52	53
73	96			71	61	53	53	97	85	61	59	.	70	57	55	56				56	55
61	70			74	55	49	55	60	60	55	61	.	77	85	90	73				57	97
71	106	82	88	59	84	87	107	101	106	95	77	.	99	85	94	96	88	91	87	91	103
99	71	83	81	84	102	70	95	71	92	71	101	.	97	81	79	73	72	72	57	63	96
77	96	61	49	78	97	81	109	124	101	65	82	.	90	62	89	97	89	82	74	84	100
69	76	75	84	67	69	63	83	116	102	89	97	.	99	60	74	81	61	65	56	61	77
99	72	59	75	76	106	82	76	121	82	69	87	.	74	64	74	101	71	98	85	64	92
80	113	72	43	61	78	82	86	111	77	94	74	.	62	81	101	80	92	62	91	76	90
71	83	56	46	74	71	58	83	76	88	71	84	.	75	62	118	88	65	57	60	57	94
72	108	78	80	59	95	78	94	118	100	70	101	.	72	92	88	98	56	69	63	64	85
96	89	62	49	61	68	72	70	85	72	95	74	.	91	91	85	75	63	66	80	82	65
73	83	60	77	60	91	50	90	82	76	76	100	.	71	63	96	74	71	70	57	74	80
79	89	61	59	63	68	93	100	109	108	72	87	.	95	71	115	90	56	61	58	73	68
90	84	73	56	76	93	66	94	85	82	93	83	.	75	80	90	79	57	67	76	76	90
79	113	75	76	74	77	58	71	89	84	73	76	.	78	82	89	73	86	79	93	83	80
101	100	66	81	71	66	70	83	95	119	101	99	.	106	102	124	111	85	82	98	92	77
97	107	71	60	85	73	74	70	95	125	100	107	.	90	106	126	97	89	79	78	69	77
96	96	83	88	75	94	100	92	101	105	104	94	.	94	93	103	95	76	90	79	74	93
77	90	86	85	73	94	76	84	107	104	90	109	.	97	88	86	99	93	96	92	96	99
100	108	69	69	74	105	71	112	112	105	99	100	.	100	74	100	112	72	98	80	89	97
132	114	76	75	81	98	71	92	108	101	107	109	.	108	79	123	111	86	73	79	69	99
93	101	71	92	89	102	94	98	118	111	91	89	.	88	89	129	89	76	93	90	87	110
77	93	77	92	68	92	118	93	87	137	100	101	.	112	86	94	86	88	95	83	86	80
94	81	76	76	64	91	86	110	99	125	101	94	.	111	102	96	108	74	82	79	67	80

90	88	74	63	75	87	73	110	82	101	89	94 .	103	100	82	90	70	68	90	84	86
108	102	77	66	76	97	81	81	99	94	88	112 .	100	77	82	105	98	92	84	69	108
105	103	84	54	80	106	98	96	106	100	94	113 .	88	76	93	89	88	77	80	78	101
92	90	78	65	77	96	104	93	95	116	110	101 .	103	86	106	99	76	82	90	92	77
106	90	77	76	103	97	92	98	98	99	100	107 .	99	83	88	92	80	78	97	76	104
86	102	69	94	93	79	83	103	113	111	101	95 .	86	97	89	96	80	98	101	65	79
87	117	82	72	92	98	75	111	123	99	100	118 .	89	94	105	112	82	98	94	96	86
85	82	88	76	90	115	75	85	119	95	104	111 .	103	75	118	89	81	80	89	94	90
83	94	77	56	89	90	72	91	119	90	86	106 .	98	75	105	94	76	65	72	79	87
95	109	82	52	86	83	79	104	94	122	86	94 .	77	70	78	98	71	67	69	63	84
99	96	75	73	93	105	96	89	112	100	87	111 .	93	85	71	84	82	63	71	71	91
86	88	82	52	85	89	74	85	100	82	94	112 .	86	71	77	80	76	68	74	75	74
81	87	80	72	75	93	84	84	93	94	83	94 .	82	88	121	105	77	82	79	83	85
83	99	70	63	72	83	104	91	92	122	99	96 .	80	83	83	98	81	69	79	77	98
81	85	70	63	70	80	105	93	116	85	82	104 .	88	66	75	81	85	70	80	77	88
95	80	71	55	82	103	74	85	106	94	82	89 .	82	78	81	87	80	82	96	78	96
88	92	70	89	81	85	74	80	93	95	91 .	.	96	71	96	87	67	96	79	75	81
89	86	77	87	68	80	84	84	82	99	87 .	.	98	71	87	87	73	65	66	71	94
100	79	75	56	78	80	70	80	92	117	74 .	.	78	77	86	81	83	60	70	65	75
77	97	68	80	94	80	76	88	88	95	76 .	.	90	89	86	84	69	62	68	67	93
76	102	61	70	85	93	98	90	86	85	70 .	.	88	72	80	80	87	58	74	73	78
86	80	78	58	63	105	90	81	108	86	66 .	.	86	68	68	91	73	56	81	88	89
112	68	77	51	57	97	64	75	118	114	83 .	.	99	66	88	83	83	49	87	77	67
80	67	64	51	80	94	65	88	105	106	100 .	.	76	62	121	60	78	53	69	57	59
83	80	63	71	62	66	70	93	80	79	68 .	.	59	58	75	63	68	55	51	59	58
102	68	54	45	63	74	77	85	77	84	69 .	.	91	67	76	67	67	86	55	56	65
83	70	80	45	60	88	57	64	108	80	69 .	.	75	64	83	78	71	70	75	68	60
66	83	60	46	65	75	65	89	79	80	69 .	.	72	91	72	75	65	53	63	64	72
87	71	86	59	84	68	66	69	77	75	94 .	.	82	75	72	67	54	63	58	62	58
70	74	56	47	70	74	52	65	83	71	76 .	.	75	58	75	77	68	56	60	58	64
82	85	66	51	58	65	55	88	78	91	65 .	.	71	70	94	63	57	81	66	56	58
65	107	62	48	66	76	90	70	99	75	61 .	.	77	57	97	69	55	61	59	84	82
63	83	73	73	59	77	64	70	96	76	89 .	.	72	62	70	99	54	81	71	62	72
91	75	61	55	71	65	53	74	70	79	72 .	.	72	57	77	73	67	74	65	61	61
72	76	62	50	63	94	69	88	92	93	66 .	.	84	76	65	72	66	57	72	66	60
81	69	60	49	58	69	71	73	87	70	65 .	.	72	59	73	65	65	58	64	62	62
71	93	63	55	67	68	55	77	88	76	73 .	.	72	64	70	64	68	57	55	58	60
60	74	63	74	58	68	59	86	83	72	66 .	.	67	77	74	77	78	57	57	73	61
89	69	53	50	50	68	78	70	75	80	79 .	.	74	59	66	71	64	57	67	70	82
97	76	58	80	53	81	53	70	75	72	63 .	.	71	59	90	63	58	53	64	57	59
77	80	74	55	55	76	53	71	82	65	77 .	.	82	72	88	65	58	71	74	57	61
65	95	63	55	52	87	54	91	81	63	66 .	.	77	55	62	64	59	60	61	51	71
64	79	66	51	69	66	66	70	74	75	68 .	.	70	57	62	90	70	55	70	60	89
72	75	75	60	55	66	53	69	103	67	71 .	.	75	91	84	76	55	54	57	73	77
65	81	62	51	68	63	83	90	73	73	64 .	.	70	87	85	65	57	84	64	58	58
69	94	58	60	61	82	89	72	103	112	63 .	.	70	60	88	77	73	65	51	61	55
85	78	57	66	51	69	58	73	113	84	84 .	.	63	60	91	66	56	53	77	65	60
80	77	59	49	92	72	54	71	112	69	77 .	.	81	60	68	67	82	75	78	83	55
67	83	59	54	76	71	61	77	72	109	65 .	.	84	88	101	89	58	58	56	68	53
104	95	82	85	59	69	88	68	95	105	64 .	.	88	87	106	71	59	98	67	56	85
82	76	70	76	61	66	89	87	83	71	83	94 .	75	64	74	66	77	76	72	67	85
71	76	90	88	58	90	63	88	73	75	82	87 .	69	60	108	81	92	95	96	64	58
70	114	67	60	77	100	64	77	73	75	73	71 .	89	58	106	66	67	80	65	80	89
70	110	65	60	70	69	59	83	111	75	82	78 .	76	68	79	89	63	60	65	73	69
75	76	71	81	60	73	58	74	73	112	101	71 .	69	61	82	77	57	81	76	73	54
115	110	97	83	66	71	85	87	79	117	76	104 .	86	74	112	78	69	66	82	79	59
111	120	88	122	87	76	66	75	78	112	87	106 .	115	109	118	113	86	93	93	95	89
88	109	67	88	73	86	80	98	115	120	116	106 .	114	107	133	113	88	98	89	88	69
86	114	84	89	93	99	81	78	110	91	88	111 .	98	97	120	111	89	107	81	74	91
88	116	64	72	98	111	104	104	104	123	91	100 .	99	86	119	88	81	87	81	87	114
91	106	77	98	92	98	79	80	111	129	98	100 .	98	91	111	75	81	95	103	65	124
88	87	84	87	90	80	85	88	98	99	95	117 .	80	63	115	110	97	94	86	78	118
94	111	83	75	67	108	97	116	110	117	101	111 .	101	86	117	106	80	76	74	81	99
122	98	86	87	79	106	105	94	99	97	110	93 .	97	98	87	88	82	66	81	89	109
88	92	71	82	79	110	95	79	80	108	87	115 .	71	76	94	87	82	99	88	80	95
82	95	89	80	79	101	68	88	94	90	96	108 .	95	64	115	102	70	76	80	74	90
98	99	86	71	70	94	91	104	127	105	84	117 .	100	58	101	90	83	74	75	68	92
86	89	69	71	70	112	107	95	97	106	87	113 .	103	80	105	91	87	62	100	72	74
100	102	64	96	78	112	73	78	105	96	87	97 .	98	105	78	87	76	94	95	66	85
88	112	93	81	82	83	84	92	130	91	105	100 .	75	92	112	109	68	98	97	67	89
94	88	77	71	68	88	73	96	124	91	114	96 .	97	63	101	105	87	87	92	88	85
83	88	64	58	80	108	90	106	121	128	104	93 .	93	68	89	78	80	59	77	80	82
80	89	82	64	86	112	91	82	96	123	94	91 .	83	68	102	81	73	81	76	73	99
86	92	65	58	90	108	87	87	106	93	112	107 .	98	101	107	96	85	69	65	65	108
77	91	63	94	82	72	83	99	94	92	96	117 .	80	97	108	90	69	61	83	69	98
106	91	64	87	67	83	83	84	87	114	77	102 .	73	65	91	108	75	76	79	74	58
88	87	88	75	70	104	71	100	117	122	106	99 .	101	64	89	92	72	65	72	83	83
96	88	77	76	67	77	80	104	122	88	78	92 .	74	75	92	100	72	78	70	79	89
82	99	64	56	86	76	71	84	93	82	86	99 .	76	78	106	78	66	71	73	68	77
87	106	77	57	81	81	97	70	85	82	81	104 .	103	65	82	83	80	88	83	71	80
85	95	69	81	71	80	98	77	82	96	77	96 .	98	64	86	84	65	64	83	68	72
84	88	82	55	73	105	73	92	102	81	84	89 .	79	86	89	93	74	60	64	57	102
101	88	61	59	80	95	80	86	114	89	88	96 .	79	72	121	81	66	56	72	70	73
99	78	60	83	63	69	68	89	96	96	97	115 .	99	80	92	69	65	89	84	64	77
84	88	54	76	61	90	101	90	124	89	76	109 .	75	67	92	87	80	68	82	63	96
73	94	91	49	66	95	71	78	121	99	61	90 .	94	64	89	105	78	67	71	61	70
66	76	81	48	55	77	63	88	103	78	68	88 .	104	65	73	103	65	62	49	66	54
97	70	82	81	72	78	104	78	81	82	93	85 .	88	88	75	80	73	88	47	55	53
97	71	58	59	76	77	67	79	88	108	85	72 .	68	67	73	63	54	66	88	56	59
73	76	51	49	58	104	60	70	75	114	84	82 .	65	66	76	74	54	44	52	77	66
85	72	45	50	60	103	85	110	79	78	80	108 .	65	57	97	62	59	53	42	51	64
79	68	73	72	61	71	62	78	72	70	74	90 .	76	58	75	85	69	77	78	51	52
83	68	59	50	62	62	61	65	69	76	82	89 .	80	84	61	61	76	54	79	52	57
58	74	65	45	61	72	79	63	120	104	71	73 .	66	64	64	61	54	53	58	53	96
69	71	59	46	65	73	67	70	110	74	64	86 .	69	65	59	77	48	54	63	54	95
63	70	55	83	63	79	67	65	83	67	87	74 .	93	81	106	59	69	69	76	53	62
92	95	56	79	71	67	56	61	77	74	69	68 .	108	93	92	68	58	51	59	73	57
87	92	72	71	64	68	62	65	84	84	62	91 .	67	57	74	58	57	55	90	75	57
68	69	59	48	59	65	68	74	94	69	65	109 .	63	84	88	74	54	66	87	57	53
74	69	79	51	55	68	79	88	79	72	69	76 .	74	66	79	63	78	56	64	54	65
74	64	63	49	62	67	63	59	86	77	57	74 .	72	69	66	84	62	55	58	51	88
66	72	57	62	58	68	57	81	83	95	89	60 .	66	63	68	69	53	61	86	76	54
72	64	89	70	63	66	53	71	75	71	78	69 .	66	84	105	83	52	56	79	65	53
65	78			75	93	63	65	104	71	61	86 .	65	53	75	71	58			59	64
66	64			62	66	63	72	85	73	66	71 .	62	58	101	64	56			47	59

80	64			58	63	82	59	72	71	84	73 .	64	64	73	93	78			79	57
89	64			58	67	63	73	71	82	65	90 .	90	60	70	77	57			57	91
63	70			62	67	60	78	72	74	61	78 .	74	66	79	70	30			52	75
64	64			60	76	70	68	74	71	77	72 .	67	63	62	60				58	60
106	108			79	66	62	69	107	74	65	72 .	69	59	114	59				83	60
67	101			62	71	64	64	85	87	85	74 .	65	63	96	64				70	65
65	89			69	65	79	64	74	73	73	97 .	94	62	79	96				55	63
107	116			58	93	80	89	78	79	63	109 .	69	58	85	109				67	75
105	90			59	67	65	72	77	68	61	70 .	69	86	103	98				56	72
75	76			83	65	60	71	101	75	76	65 .	90	75	80	77				51	95
97	78			57	98	55	84	82	69	61	78 .	69	57	96	81				82	74
74	108			78	95	65	66	75	108	60	102 .	68	60	78	100				62	64
72	99			63	66	75	67	88	85	74	102 .	69	59	109	83				52	60
88	87			59	65	87	99	101	77	99	86 .	68	71	86	71				65	63
88	82			69	95	68	72	73	96	68	79 .	74	60	78	73				55	87
100	95			72	82	58	83	92	88	73	83 .	76	76	97	101				95	66
94	93			83	69	65	79	87	109	98	85 .	101	118	84	114				92	63
95	98			84	75	70	68	127	119	89	118 .	105	105	118	89				86	94
90	122			96	89	81	83	122	134	98	112 .	93	75	106	92				84	68
97	100			94	109	93	111	122	130	95	108 .	96	96	89	106				65	115
106	86			71	101	93	104	106	94	89	101 .	104	92	119	88				75	105
106	115			87	116	100	79	127	110	110	91 .	85	61	123	107				90	96
91	98			83	111	91	109	102	129	106	93 .	84	64	113	108				85	103
87	112			83	93	82	87	110	115	93	98 .	86	90	116	94				70	96
93	104			91	115	90	91	99	83	103	117 .	95	101	97	98				62	80
105	85			79	90	70	90	87	103	99	100 .	109	82	82	99				63	105
103	101			86	68	60	83	90	87	89	85 .	98	75	111	100				76	88
111	80			65	74	79	97	117	111	110	108 .	90	71	92	85				63	109
86	74			58	95	96	91	101	93	111	96 .	76	96	78	100				89	71
92	69			85	101	66	89	94	111	117	105 .	83	91	109	105				89	96
80	88			81	114	74	115	99	111	88	94 .	77	63	87	105				78	112
83	80			55	106	95	103	131	86	92	107 .	78	59	75	86				62	86
77	75			92	74	98	67	136	90	80	94 .	104	94	75	101				68	91
110	86			90	77	104	83	135	84	99	92 .	92	99	115	105				63	83
93	100			82	76	76	96	120	85	86	101 .	75	64	87	93				63	93
93	83			69	82	65	97	119	95	82	77 .	67	73	63	95				67	82
85	72			77	108	64	73	84	87	94	85 .	88	61	73	88				91	75
84	75			67	112	97	74	86	87	89	112 .	79	94	79	96				72	88
78	69			58	96	95	100	117	83	80	105 .	74	96	98	91				75	107
86	97			70	58	72	73	119	111	82	90 .	95	65	76	86				63	103
97	78			63	98	68	82	89	129	87	90 .	100	66	78	91				57	90
85	79			80	89	72	73	83	110	87	84 .	80	76	108	88				69	74
82	86			84	83	76	84	102	88	104	85 .	71	65	110	89				60	80
79	109			61	101	97	79	113	88	117	105 .	71	84	78	98				59	73
79	88			74	75	103	79	72	106	104	107 .	82	69	74	84				84	85
80	75			84	61	89	75	77	93	81	85 .	59	64	73	96				61	74
90	87			65	91	82	100	114	94	71	94 .	82	71	73	74				69	62
78	108			64	95	72	78	88	101	81	99 .	97	71	67	86				69	104
73	103			83	70	86	79	74	102	98	94 .	94	57	109	85				91	83
63	70			75	59	61	74	104	71	106	59 .	78	62	94	75				81	57
78	67			56	63	72	98	91	71	70	79 .	57	87	76	64				63	54
74	73			64	104	64	90	80	127	79	104 .	96	69	64	78				45	51
62	70			56	72	73	56	80	89	89	83 .	77	58	85	68				45	69
55	96			59	61	56	69	73	73	68	79 .	68	66	112	67				80	58
73	105			58	70	61	64	76	69	66	79 .	66	57	97	72				59	62
99	75			57	64	61	83	91	95	67	86 .	63	63	69	61				59	57
69	75			68	64	62	69	106	76	84	75 .	62	78	67	78				56	70
57	73			55	66	71	70	106	80	70	71 .	66	64	69	65				66	96
59	70			55	68	54	67	65	81	60	74 .	68	58	74	54				60	64
75	65			56	61	61	71	105	77	60	64 .	88	78	70	73				61	58
67	72			76	65	71	93	74	108	79	73 .	75	59	66	83				68	63
63	70			53	71	91	69	68	96	63	84 .	64	60	82	66				66	54
73	76			52	85	70	63	68	71	67	78 .	64	57	69	62				57	70
103	99	63	62	90	99	87	104	78	85	75	83 .	102	89	112	105 .		68	64	62	92
96	93	71	62	67	91	76	78	80	77	95	84 .	98	89	107	95 .		68	72	58	89
78	83	55	59	71	71	60	78	92	82	72	85 .	91	89	108	80 .		69	72	62	84
77	99	67	79	58	73	67	91	79	102	76	84 .	89	79	103	91 .		68	83	87	86
77	91	65	61	77	84	56	70	91	95	101	104 .	73	90	89	82 .		67	65	74	75
77	73	71	60	75	68	64	73	102	102	74	86 .	86	71	109	84 .		68	65	67	81
80	74	57	71	65	68	91	75	84	73	81	97 .	79	73	85	84 .		68	64	63	70
96	74	77	83	66	72	79	67	84	98	85	82 .	69	73	82	89 .		63	75	65	72
73	96	80	62	74	74	75	68	90	87	95	90 .	70	90	79	72 .		62	68	69	78
80	78	71	62	69	67	64	79	90	85	78	98 .	74	75	99	85 .		79	70	67	67
88	78	66	83	70	86	55	77	85	88	85	85 .	86	72	84	78 .		80	64	66	67
113	93	68	88	72	68	78	74	84	83	78	78 .	77	89	84	89 .		59	82	80	72
85	81	79	100	79	67	65	72	115	113	86	90 .	90	92	87	103 .		67	93	77	70
102	98	92	97	88	77	66	92	93	109	107	110 .	105	81	121	101 .		67	86	73	83
116	102	67	86	83	78	80	105	128	109	111	106 .	98	103	128	107 .		57	105	76	91
102	105	67	72	71	108	80	90	125	97	123	97 .	99	85	103	107 .		57	95	71	104
101	93	75	67	99	84	97	91	109	105	92	110 .	109	103	91	95 .		97	81	79	91
112	94	76	84	77	82	82	91	103	108	101	126 .	81	95	111	103 .		74	80	77	92
103	107	91	89	78	95	85	113	92	135	93	95 .	92	76	120	94 .		68	91	71	90
102	107	92	74	72	84	79	81	104	125	98	105 .	89	92	112	96 .		71	83	86	97
89	95	71	79	67	82	102	92	98	96	98	90 .	85	109	87	95 .		85	103	75	99
93	89	74	80	74	90	108	96	114	116	103	97 .	90	84	119	101 .		72	108	64	102
89	88	84	68	73	82	84	88	112	102	96	98 .	108	90	96	99 .		91	109	79	87
97	107	60	60	71	75	72	90	130	103	91	109 .	103	84	97	108 .		96	80	83	87
97	102	72	72	83	85	70	82	102	100	103	109 .	86	78	96	106 .		88	64	75	89
96	82	72	72	87	107	84	89	104	98	96	109 .	86	83	103	90 .		101	78	73	95
114	96	84	98	67	94	108	118	103	105	103	98 .	88	96	112	97 .		86	84	74	103
97	88	84	89	77	77	102	83	108	97	116	104 .	119	101	115	103 .		84	87	92	81
97	83	72	55	89	110	94	84	102	110	125	102 .	109	76	107	102 .		90	93	88	111
109	96	79	53	78	106	70	100	139	128	113	100 .	95	78	102	107 .		72	95	92	91
124	102	54	62	72	82	69	76	123	97	102	124 .	78	104	85	114 .		55	101	79	92
78	113	64	55	74	76	89	80	94	90	98	98 .	67	72	82	101 .		74	91	64	82
96	86	67	59	72	79	76	92	114	116	85	95 .	89	91	83	89 .		67	71	70	84
92	102	63	67	72	93	76	83	98	87	87	103 .	90	83	109	84 .		60	72	59	91
103	102	69	79	77	73	75	77	87	98	110	92 .	76	86	84	104 .		59	74	55	80
100	108	77	90	78	92	81	85	94	84	98	97 .	83	86	80	90 .		62	80	58	102
97	105	62	83	82	105	81	93	99	99	87	99 .	79	80	94	91 .		62	83	70	116
91	79	59	51	77	80	85	87	93	97	95	87 .	80	85	90	93 .		75	77	64	98
91	94	64	77	88	101	84	78	117	109	88	99 .	78	82	83	85 .		74	83	54	81
90	89	67	56	60	117	108	74	124	88	85	99 .	75	77	110	73 .		64	95	79	80
84	96	85	79	78	104	92	79	91	94	87	87 .	89	88	115	99 .		72	88	81	98

96	89	62	85	82	66	79	106	118	91	117	100 .	84	81	102	108 .	66	84	61	90
90	83	68	67	77	63	91	105	111	124	111	86 .	96	75	84	99 .	57	71	69	98
86	83	59	52	61	80	74	92	95	119	84	96 .	100	98	101	71 .	74	79	74	86
78	105	62	53	84	117	74	75	100	81	74	123 .	110	71	72	88 .	59	97	86	72
66	108	61	49	73	100	66	70	86	81	68	93 .	76	86	101	70 .	61	88	81	80
97	99	60	92	66	101	67	100	75	88	69	88 .	72	71	84	71 .	57	62	54	94
95	76	74	82	64	74	59	62	92	86	86	82 .	75	63	75	70 .	81	50	55	63
71	78	90	49	66	65	85	67	81	100	75	93 .	75	70	94	78 .	91	69	69	56
71	90	60	52	76	89	58	66	80	86	74	87 .	75	88	77	77 .	95	60	55	65
72	83	56	50	67	90	63	81	77	99	71	99 .	90	75	78	88 .	77	74	60	68
70	90	72	57	67	62	63	58	104	80	87	84 .	102	76	110	74 .	56	59	63	61
94	82	63	69	74	68	69	86	94	87	75	98 .	73	81	102	81 .	57	58	65	64
72	77	68	52	74	72	84	74	74	86	72	79 .	76	70	95	84 .	68	63	62	70
73	82	80	78	65	69	57	68	78	93	75	77 .	76	72	78	95 .	77	60	77	64
73	98	58	55	78	85	65	71	99	75	80	87 .	70	76	94	85 .	56	71	86	63
78	79	62	56	68	66	65	67	106	109	77	96 .	69	64	79	86 .	56	58	75	69
79	73	70	75	67	89	63	70	80	82	75	80 .	75	77	74	75 .	63	62	58	63
100	77	59	54	69	71	59	86	81	78	76	84 .	75	70	85	92 .	76	59	59	69
83	76	57	60	68	97	89	74	115	78	74	88 .	90	71	110	91 .	62	66	67	91
77	78	62	58	67	76	81	76	78	85	85	86 .	81	70	83	81 .	62	65	57	66
81	94	61	80	63	75	69	78	89	76	77	87 .	75	80	85	90 .	76	77	74	72
71	83	72	62	80	93	73	79	100	84	80	84 .	79	76	72	80 .	61	68	67	66
74	79	60	56	71	71	57	77	82	88	79	85 .	74	73	78	83 .	72	65	61	67
95	96	54	50	71	73	63	80	92	92	100	93 .	79	72	78	97 .	59	89	61	72
71	82	64	67	70	73	79	67	80	77	67	104 .	79	82	72	83 .	81	65	72	79
77	85	62	53	74	66	62	72	84	80	85	98 .	74	74	118	84 .	57	64	60	68
88	84	55	65	69	71	61	86	91	80	83	81 .	69	75	89	78 .	59	62	62	60
71	78	54	80	73	89	63	75	90	91	79	79 .	74	89	84	78 .	59	77	70	67
75	78	65	55	67	73	57	68	95	76	78	95 .	69	74	76	82 .	71	61	58	78
77	78	69	77	72	83	79	82	83	85	73	83 .	101	75	83	89 .	57	61	80	65
97	95	54	72	95	73	68	69	99	109	69	107 .	75	72	81	81 .	54	63	73	82
102	95	61	55	73	104	92	93	85	79	92	85 .	85	75	101	101 .	80	63	70	75
81	78	60	63	71	69	66	93	85	81	85	91 .	78	70	83	85 .	84	62	85	69
80	92	58	69	76	72	57	68	116	79	85	94 .	68	86	108	91 .	58	68	74	84
78	108	69	60	81	79	64	74	118	97	91	83 .	101	91	84	89 .	54	84	86	89
96	87	69	76	86	109	60	81	83	96	79	103 .	85	97	87	101 .	66	89	93	73
93	98	91	74	96	75	78	86	104	109	93	103 .	81	91	85	84 .	76	62	110	74
96	116	83	104	92	86	65	79	103	103	96	97 .	81	107	124	112 .	86	86	93	80
121	115	75	109	83	81	71	93	114	117	114	115 .	110	88	132	108 .	77	106	95	95
120	113	73	88	90	96	76	103	120	122	123	107 .	114	92	125	101 .	76	100	75	112
85	104	97	74	102	111	92	87	101	113	114	127 .	116	106	117	118 .	72	97	83	101
111	105	86	94	80	111	91	85	100	102	106	110 .	105	105	133	120 .	75	92	72	97
85	84	77	85	85	97	73	94	135	108	91	122 .	107	89	113	114 .	85	65	80	78
97	83	81	61	86	83	118	109	108	112	128	110 .	107	91	115	107 .	104	77	94	104
85	101	91	62	97	115	110	88	105	143	121	104 .	91	85	113	113 .	93	77	68	106
88	112	73	90	77	115	74	81	114	130	107	111 .	103	91	97	96 .	72	76	78	80
109	91	102	95	79	115	82	110	109	93	97	106 .	84	78	115	125 .	72	72	74	91
114	84	96	100	80	96	76	109	105	126	99	117 .	95	99	93	119 .	74	99	87	95
112	95	75	84	82	89	84	98	106	100	100	133 .	88	102	92	106 .	90	101	70	86
77	101	77	60	101	97	99	90	117	98	100	105 .	109	77	99	99 .	96	91	76	76
93	104	77	61	95	122	90	87	131	103	94	106 .	91	97	128	91 .	104	78	96	103
83	100	84	62	87	114	116	87	109	120	95	100 .	90	89	104	101 .	85	70	100	105
114	102	71	65	86	91	105	84	122	130	94	118 .	105	76	97	112 .	66	77	86	86
89	101	84	85	80	98	93	78	113	94	119	119 .	89	94	118	100 .	81	77	62	77
94	101	80	93	79	108	76	86	149	97	121	113 .	78	88	83	91 .	74	94	67	82
91	101	83	62	83	101	76	107	132	111	114	89 .	90	114	101	102 .	63	100	70	83
109	114	80	55	73	88	71	112	102	96	99	125 .	102	83	95	99 .	77	81	74	96
91	99	77	69	74	83	96	77	120	99	107	121 .	95	90	83	102 .	75	76	77	98
89	89	80	80	70	89	71	92	98	108	101	99 .	84	85	88	107 .	66	75	76	82
100	91	78	62	100	107	76	93	119	97	102	107 .	97	97	87	94 .	64	89	67	75
93	100	85	68	72	84	90	83	118	109	101	98 .	95	86	96	93 .	71	92	76	95
93	95	77	94	76	73	105	81	100	109	102	98 .	89	78	91	91 .	79	77	80	74
92	89	67	88	76	93	76	85	97	100	93	106 .	94	78	99	100 .	74	79	60	97
86	84	70	59	77	81	74	83	99	127	98	107 .	84	90	101	90 .	67	77	85	100
106	85	79	73	81	93	81	95	112	117	90	94 .	87	86	117	97 .	80	70	88	85
87	95	74	69	71	79	88	83	112	101	89	93 .	80	89	118	101 .	62	78	68	81
82	106	79	62	69	85	106	81	97	120	121	104 .	101	87	105	102 .	98	89	73	81
88	96	70	80	68	120	76	104	115	129	111	93 .	101	99	83	82 .	96	66	74	81
100	87	94	78	85	89	69	114	101	95	113	113 .	91	95	84	78 .	86	73	101	74
97	76	88	60	63	75	68	97	100	93	102	82 .	106	90	117	86 .	53	92	80	77
84	83	67	51	59	65	65	99	109	88	85	86 .	71	73	102	86 .	52	65	69	93
87	89	60	50	64	99	86	95	92	93	101	88 .	72	90	75	93 .	82	67	69	94
87	95	80	58	66	74	90	69	96	112	89	96 .	66	75	72	85 .	55	72	61	56
89	92	57	48	65	66	66	90	114	86	92	89 .	79	79	73	81 .	55	95	64	65
83	78	55	50	70	91	61	72	125	81	81	80 .	78	78	73	89 .	80	61	62	71
73	79	64	61	65	67	79	84	107	82	82	94 .	71	82	84	83 .	58	78	63	69
85	79	49	48	64	71	72	84	76	86	78	95 .	72	75	78	84 .	52	60	70	64
84	86	66	53	72	90	66	82	80	84	82	84 .	83	75	89	84 .	56	56	60	67
92	82	79	51	71	70	56	72	82	103	75	82 .	72	83	83	95 .	56	59	58	89
83	82	57	55	67	75	55	90	80	79	77	87 .	72	90	72	77 .	69	71	80	70
91	82	64	63	64	74	71	91	104	85	74	105 .	74	68	102	90 .	60	60	63	74
83	82	61	52	76	88	63	74	78	78	91	75 .	79	77	77	96 .	63	73	58	70
88	101	79	56	65	69	60	76	87	89	74	78 .	80	69	95	83 .	84	58	67	71
83	94	73	79	61	78	74	75	80	90	73	88 .	74	85	89	89 .	64	63	60	65
77	77	59	59	66	90	89	80	91	84	70	85 .	73	77	84	95 .	58	71	74	67
81	84	61	62	73	72	63	71	73	80	67	92 .	67	92	77	88 .	66	64	69	68
75	82	66	55	68	79	67	67	108	95	96	85 .	110	93	81	82 .	75	57	65	94
94	101	54	61	72	78	78	73	75	88	85	80 .	74	83	85	92 .	68	74	69	63
91	80	64	56	70	82	67	97	79	92	75	73 .	78	76	104	81 .	79	59	56	77
76	83	55	55	73	73	66	81	98	98	78	98 .	73	72	87	79 .	60	75	61	67
83	95	61	58	66	78	75	87	77	81	79	88 .	74	72	83	102 .	63	64	75	76
73	82	60	62	64	83	66	79	84	79	79	89 .	95	79	95	76 .	62	58	64	67
75	80	56	76	61	66	61	76	80	95	73	72 .	94	78	85	80 .	63	65	69	67
99	82	64	51	66	73	61	84	85	90	76	84 .	78	86	99	83 .	61	64	63	66
107	84	63	51	70	90	67	80	72	85	73	85 .	77	91	98	82 .	65	70	77	72
79	102	65	76	68	72	67	85	86	84	84	109 .	82	76	90	73 .	64	58	76	60
78	79	60	55	71	79	64	80	79	82	94	92 .	77	77	88	90 .	64	61	66	72
115	120	59	53	75	114	104	85	100	93	85	83 .	74	74	143	121 .	63	69	71	110
101	113	72	58	82	112	65	93	85	90	74	82 .	80	78	127	94 .	84	68	68	77
78	82	83	67	75	83	70	83	79	96	79	86 .	77	97	98	79 .	67	75	81	80
90	90	74	82	80	83	80	86	81	96	83	97 .	98	91	84	86 .	61	72	75	77
97	99	85	66	86	85	74	90	108	117	98	108 .	73	80	102	86 .	68	65	96	68
101	89	97	79	92	98	74	89	91	97	113	93 .	84	114	99	102 .	96	83	96	85
120	115	92	110	82	87	87	89	125	115	116	115 .	113	103	128	112 .	90	87	91	83
103	91	73	95	96	109	98	96	140	129	122	104 .	116	100	139	112 .	102	91	87	98

94	119	71	93	89	112	97	109	120	95	116	109 .	97	90	114	124 .	113	100	69	107
113	100	86	73	97	113	100	126	101	124	124	125 .	101	89	91	107 .	96	90	85	90
98	106	86	71	78	95	96	107	101	101	116	99 .	104	104	131	102 .	75	73	77	88
98	120	80	67	83	113	88	81	123	99	111	125 .	102	84	108	96 .	73	78	101	85
100	116	79	68	85	107	83	86	134	134	122	121 .	90	97	92	118 .	91	92	101	84
90	93	73	83	71	87	107	83	129	109	128	105 .	96	106	85	90 .	71	97	92	88
97	102	86	94	81	100	106	97	123	99	112	93 .	97	83	127	83 .	89	96	86	95
106	88	93	79	77	89	97	87	103	109	107	97 .	90	85	120	100 .	94	89	86	115
93	106	86	70	95	124	83	103	103	103	113	91 .	109	97	98	90 .	89	75	80	101
105	100	60	65	105	94	83	106	105	108	125	124 .	100	100	101	99 .	71	78	79	98
95	94	71	90	87	94	97	78	108	102	121	92 .	81	90	103	106 .	73	82	69	86
87	110	82	99	66	104	115	90	111	96	115	97 .	90	93	110	114 .	86	78	87	96
106	123	68	87	70	120	87	103	127	100	103	107 .	90	113	106	118 .	96	111	91	82
105	106	84	55	77	111	79	87	136	129	92	102 .	88	83	102	88 .	77	93	70	78
94	93	91	56	92	91	83	91	108	134	103	105 .	91	96	110	101 .	88	73	72	106
104	90	82	71	86	113	89	79	109	107	114	121 .	87	91	128	89 .	93	69	74	114
97	95	76	61	72	112	87	100	98	126	97	114 .	97	84	110	89 .	70	76	70	106
98	94	71	58	80	88	106	82	109	110	107	91 .	115	79	90	95 .	71	76	80	89
96	91	72	83	76	96	82	92	122	102	133	108 .	100	94	96	102 .	70	85	80	97
92	97	76	88	75	87	80	105	100	116	123	114 .	85	81	111	96 .	64	83	80	85
96	101	74	65	79	88	87	103	98	122	109	95 .	102	84	106	107 .	76	69	79	94
90	106	71	53	73	104	86	70	121	103	98	95 .	95	86	94	100 .	69	76	78	78
106	89	72	51	76	107	85	80	100	109	105	91 .	98	85	108	109 .	98	86	76	79
110	89	70	64	72	77	99	77	98	108	95	102 .	81	83	101	103 .	93	75	77	93
90	91	70	66	91	94	84	80	98	128	104	98 .	86	89	96	88 .	69	74	69	96
94	90	85	89	87	90	78	122	96	101	97	104 .	75	96	102	92 .	68	86	74	97
96	109	72	65	92	83	83	109	95	90	92	111 .	89	78	89	98 .	81	68	89	72
83	100	60	58	67	80	97	87	125	119	97	117 .	90	73	113	75 .	68	69	90	87
91	82	67	77	93	126	108	80	122	132	86	105 .	83	79	116	71 .	71	76	89	75
106	84	82	69	84	106	91	100	108	116	108	114 .	81	114	102	78 .	70	83	80	75
75	83	72	49	66	69	75	102	88	87	97	113 .	77	91	110	79 .	63	70	63	76
82	104	55	50	76	95	73	109	96	92	84	98 .	72	77	83	70 .	69	69	55	97
81	86	75	53	67	100	67	98	89	103	94	74 .	72	90	86	77 .	59	61	58	93
71	85	61	85	67	70	87	85	113	82	79	79 .	75	79	106	95 .	67	63	64	72
81	88	73	60	65	73	87	72	97	97	75	83 .	95	79	80	81 .	67	71	58	66
84	84	59	61	74	74	77	70	83	106	84	90 .	80	79	77	74 .	71	57	62	59
79	84	71	61	68	73	62	71	83	84	90	90 .	73	73	81	77 .	62	60	76	63
78	107	61	52	75	103	68	90	90	91	86	86 .	73	76	88	95 .	97	64	63	77
82	90	78	75	74	67	66	70	111	91	85	87 .	88	89	83	77 .	84	66	70	73
90	97	62	75	83	71	64	89	97	89	89	102 .	72	79	84	77 .	80	78	70	66
84	99	60	51	81	79	70	68	82	79	83	90 .	72	72	94	71 .	73	61	75	67
82	83	68	56	75	79	73	68	138	118	111	102 .	75	105	84	82 .	69	55	69	72
87	84	75	54	73	77	67	86	120	89	79	90 .	71	80	91	78 .	61	63	69	79
88	77	62	50	70	75	67	76	86	86	95	94 .	79	72	90	70 .	62	66	92	80
95	90	57	56	73	84	67	73	85	83	95	91 .	73	77	84	78 .	55	61	73	67
92	81	60	56	65	73	63	85	110	98	101	93 .	75	73	84	83 .	66	60	68	67
87	72			74	73	72	72	81	90	87	99 .	72	77	85	90			62	68
94	84			68	72	85	76	78	91	78	84 .	82	68	86	79			62	67
89	90			64	86	89	74	98	78	87	88 .	74	79	87	76			60	66
82	79			70	75	69	70	102	91	88	97 .	72	84	85	72			73	66
93	89			73	73	64	86	86	84	80	80 .	78	78	84	83			65	63
101	93			68	78	79	77	92	81	86	84 .	73	79	84	78			60	75
83	101			75	93	69	78	89	80	84	106 .	83	72	100	78			68	91
82	92			74	76	70	87	83	98	85	101 .	78	75	107	78			67	67
79	95			70	75	66	100	96	85	78	85 .	73	83	85	88			68	63
85	86			79	74	58	74	99	81	97	98 .	73	80	86	80			67	67
77	84			79	77	73	75	102	85	87	92 .	72	80	86	82			69	67
79	83			68	91	62	80	88	97	96	91 .	72	90	89	75			67	73
84	91			70	77	68	84	85	82	91	91 .	72	75	102	95			72	74
92	84			71	74	66	77	96	96	81	82 .	78	84	95	83			89	70
90	98			85	98	73	81	89	94	94	96 .	83	90	90	83			75	78
94	81			73	72	67	73	108	95	107	86 .	73	82	92	81			67	71
95	111			83	95	65	91	99	115	102	94 .	88	97	105	107			73	67
114	103			100	81	72	83	115	102	104	112 .	123	116	132	114			86	99
117	86			99	86	85	79	124	108	121	96 .	108	109	129	106			90	99
96	121			82	126	89	119	128	148	124	124 .	96	102	108	107			88	123
103	124			78	108	96	85	114	127	118	115 .	90	96	101	117			80	103
112	108			83	96	107	108	97	98	110	113 .	103	89	127	96			71	96
107	99			83	84	107	114	97	120	106	106 .	106	105	119	101			76	101
93	92			84	101	95	91	103	109	97	114 .	101	107	97	99			69	99
90	103			89	120	85	90	109	109	114	92 .	96	106	91	107			78	95
86	111			86	111	83	101	104	124	102	128 .	94	102	90	105			93	88
94	100			82	88	100	102	103	117	121	105 .	92	95	134	116			88	103
99	96			78	94	90	88	109	120	113	116 .	93	89	124	110			94	112
95	102			86	95	113	120	145	107	108	126 .	117	87	118	103			94	100
109	100			82	99	105	102	135	112	104	108 .	95	89	94	95			79	84
89	110			84	119	107	91	128	121	109	108 .	95	111	101	103			76	88
88	110			87	103	82	95	104	121	98	101 .	89	103	115	104			90	99
104	98			101	96	89	83	105	134	95	115 .	79	84	112	117			74	94
86	96			91	83	96	82	113	115	93	121 .	96	86	103	96			70	88
93	97			75	117	88	85	112	102	97	106 .	95	87	102	104			70	107
97	103			74	121	96	87	102	108	109	103 .	95	94	118	113			76	115
95	102			81	83	87	99	134	115	99	108 .	85	83	115	97			80	98
86	114			69	90	95	101	130	114	90	101 .	83	89	92	96			67	94
86	116			78	98	76	81	114	99	103	115 .	92	85	100	92			78	81
86	84			101	103	82	88	98	103	95	102 .	83	95	98	91			73	82
89	84			88	84	88	89	102	112	91	101 .	77	107	103	88			77	81
83	95			88	82	97	83	97	103	101	116 .	107	86	102	97			78	82
108	92			89	81	84	89	133	109	91	118 .	90	88	98	98			69	91
106	92			100	87	75	92	109	117	99	98 .	83	91	110	101			76	86
82	97			70	83	78	105	122	98	111	94 .	77	100	103	103			90	82
84	91			71	88	91	106	132	98	102	112 .	87	86	102	113			89	106
94	115			82	82	114	76	97	100	98	86 .	113	117	104	76			85	89
90	86			65	106	83	92	102	95	87	93 .	104	103	109	97			88	80
90	90			65	113	84	82	97	91	104	104 .	97	85	79	81			90	89
87	103			72	101	71	73	98	134	103	94 .	75	98	80	77			64	66
80	80			68	65	60	104	88	115	79	68 .	71	75	92	92			64	80
85	76			64	75	76	106	101	80	86	75 .	69	75	83	80			69	68
85	85			65	83	76	72	116	85	98	86 .	70	77	75	72			61	65
75	82			65	85	70	79	104	82	81	77 .	69	93	78	72			73	64
93	95			62	71	66	73	76	97	84	91 .	89	75	94	67			64	66
71	85			66	77	73	85	74	74	80	84 .	69	83	78	72			71	67
79	80			70	77	67	84	79	79	85	98 .	74	88	80	81			68	70
97	88			68	79	77	77	103	74	79	80 .	74	87	78	83			84	72
111	86			69	71	67	78	79	93	73	80 .	72	82	97	73			64	72

80	80	63	68	67	80	84	80	89	85 .	81	79	78	70	70	67
84	101	67	97	71	76	92	87	82	108 .	76	75	80	87	68	66
75	85	83	94	70	73	79	70	97	84 .	76	88	75	76	68	70
81	78	69	72	60	95	99	74	96	89 .	75	76	92	77	69	65
80	77	75	91	64	71	84	78	78	101 .	74	74	82	81	62	69
						76	93	79	90						
						90	75	75	85						

9	2	2	5	8	6	6	9	9	11	11	12	11	3	10
Box-8	Box-1	Box-5	Box-2	Box-6	Box-5	Box-7	Box-4	Box-5	Box-3	Box-7	Box-8	Box-8	Box-8	Box-8
75	13.7	20.2	32.3	44.5	45	47	65	68	78	84	Z	Z	Z	Z
M	M	M	M	M	M	M	M	M	M	M	Z	Z	Z	Z
Malto	MCT	MCT	MCT	MCT	MCT	MCT	MCT	MCT	MCT	MCT	Z	Z	Z	Z
MM	MT	MT	MT	MT	MT	MT	MT	MT	MT	MT	Z	Z	Z	Z
52	102	62	62	62	68	66	63	64	96	111	11	4	.	.
57	94	76	83	62	54	86	41	73	82	102	12	2	.	.
84	47	110	62	97	49	87	41	98	72	81	13	2	.	.
54	43	77	91	68	90	83	66	111	55	117	12	3	.	.
54	95	89	56	58	77	69	46	78	62	100	11	2	.	.
63	100	55	57	73	88	81	92	69	98	79	11	2	.	.
53	94	101	99	103	55	99	89	94	66	92	13	2	.	.
92	112	107	71	105	85	114	76	109	55	61	13	1	.	.
69	105	132	73	60	63	98	62	112	96	94	13	3	.	.
65	106	116	96	92	68	102	44	108	96	67	15	4	.	.
68	113	119	72	63	89	88	88	111	119	111	13	3	.	.
87	118	121	72	86	75	90	94	117	118	99	13	3	.	.
50	123	127	97	108	63	66	91	101	106	123	15	3	.	.
69	109	129	95	73	53	74	97	105	107	115	14	4	.	.
86	117	125	96	80	47	74	97	67	114	120	13	5	.	.
87	111	115	102	100	51	55	94	63	78	113	14	4	.	.
100	108	72	59	118	60	52	100	111	114	119	13	6	.	.
91	109	58	72	116	51	77	89	103	115	111	14	3	.	.
96	101	57	51	118	86	88	96	80	112	118	14	4	.	.
91	69	93	88	114	77	88	80	55	122	116	13	3	.	.
69	65	65	96	119	45	56	78	99	92	129	13	3	.	.
54	66	53	110	109	44	47	47	98	71	114	13	5	.	.
70	51	95	81	98	47	81	38	74	79	111	14	4	.	.
64	63	96	51	59	36	59	73	89	71	98	13	4	.	.
104	60	62	48	52	44	53	52	59	70	93	14	5	.	.
76	65	107	60	70	67	53	47	62	66	86	14	3	.	.
55	82	109	51	83	75	74	46	70	102	83	14	1	.	.
50	62	81	57	69	46	79	61	61	107	56	13	3	.	.
50	54	57	52	104	40	92	76	92	113	63	12	1	.	.
96	99	49	55	94	35	72	53	67	121	62	13	2	.	.
92	101	77	43	76	51	54	44	90	115	56	13	0	.	.
50	96	73	52	54	52	51	53	102	104	108	13	1	.	.
92	70	95	55	48	69	70	49	95	106	113	13	1	.	.
88	40	61	80	113	81	96	95	84	106	80	13	3	.	.
80	91	53	44	108	48	79	91	59	96	56	13	2	.	.
55	93	59	43	96	36	52	70	58	85	59	13	1	.	.
45	86	117	50	59	42	49	44	93	81	63	13	1	.	.
62	44	124	81	53	65	59	44	64	59	63	13	1	.	.
100	45	122	51	61	75	74	45	59	58	66	12	1	.	.
95	47	116	44	54	59	75	46	71	62	107	10	3	.	.
71	93	105	69	69	38	51	70	79	69	88	12	1	.	.
53	72	81	58	91	39	54	93	94	99	56	10	2	.	.
54	46	56	42	75	49	65	86	69	68	56	10	-6	.	.
54	49	54	49	56	44	71	79	69	62	52	11	0	.	.
55	56	54	50	58	44	46	48	63	76	56	11	3	.	.
94	96	74	67	90	61	50	47	103	61	57	10	4	.	.
67	83	87	54	71	71	84	52	91	68	56	11	4	.	.
43	50	57	50	56	49	78	42	67	90	96	12	3	.	.
50	60	58	62	58	39	51	71	63	69	87	11	3	.	.
85	64	62	51	52	44	47	78	61	61	55	10	3	.	.
87	50	92	53	59	44	96	74	68	60	53	11	1	.	.
47	50	97	56	61	55	83	47	67	95	60	10	4	.	.
41	60	69	54	50	55	54	53	79	69	55	11	4	.	.
47	57	58	53	56	42	53	62	89	60	99	11	3	.	.
50	56	57	90	72	50	51	70	64	64	61	10	2	.	.
89	57	69	83	88	60	89	52	61	76	61	10	4	.	.
71	54	96	50	47	45	71	53	67	69	58	10	3	.	.
47	69	74	52	47	46	49	42	66	66	61	11	4	.	.
49	93	61	61	54	39	55	47	95	84	60	11	3	.	.
51	91	55	57	57	70	84	73	89	66	91	10	4	.	.
63	63	58	54	85	46	77	48	61	66	95	11	4	.	.
77	54	61	53	57	41	62	50	72	67	69	12	2	.	.
46	55	91	75	51	34	79	81	67	65	56	12	5	.	.
60	82	91	53	55	84	59	63	83	99	56	10	3	.	.
78	76	62	69	84	61	82	43	64	94	60	10	3	.	.
58	53	66	55	67	50	88	47	68	61	87	10	5	.	.
52	54	67	99	55	64	58	46	88	61	61	11	2	.	.
47	78	100	86	94	50	81	58	105	61	67	12	3	.	.
92	61	69	66	104	82	73	81	85	59	73	12	1	.	.
91	77	115	74	109	71	104	88	93	73	80	14	2	.	.
60	93	109	58	106	78	99	86	105	77	110	13	3	.	.
54	91	93	92	95	97	82	56	101	73	104	14	3	.	.
94	107	116	59	88	73	95	84	68	107	110	14	2	.	.
93	106	119	73	67	55	91	61	70	108	109	8	3	.	.
103	94	114	58	91	91	82	64	103	110	107	9	3	.	.
62	96	105	89	60	72	65	65	101	113	114	10	2	.	.
90	107	96	78	83	67	68	75	99	120	97	10	3	.	.
62	106	77	113	118	60	70	57	86	119	104	10	3	.	.
88	125	105	106	111	92	92	69	87	116	68	14	2	.	.
93	107	85	96	113	76	104	88	92	119	54	14	4	.	.
92	100	73	89	116	51	83	90	57	112	74	14	3	.	.
95	94	66	92	83	68	57	96	55	116	101	14	3	.	.
65	75	103	91	65	100	87	95	79	120	102	14	5	.	.
85	65	89	106	88	86	86	89	91	120	65	13	4	.	.
79	87	118	96	65	72	58	87	103	113	49	13	4	.	.
71	104	88	65	90	42	52	89	82	121	57	14	5	.	.
55	106	72	100	95	40	64	73	68	107	72	15	6	.	.
77	71	104	112	101	43	62	44	86	67	110	14	6	.	.
72	56	88	77	100	46	53	62	67	60	101	14	5	.	.
58	71	69	57	79	75	83	74	75	58	110	14	3	.	.
58	71	88	59	89	50	83	51	87	101	97	13	3	.	.
82	60	71	59	70	44	73	54	94	118	104	13	2	.	.
61	54	97	73	61	40	48	45	63	123	65	14	2	.	.
56	90	106	82	59	41	58	51	69	116	53	13	3	.	.
77	110	81	94	55	67	78	59	70	80	53	14	1	.	.
88	81	78	83	83	67	79	79	66	59	60	13	1	.	.
58	55	80	80	66	42	55	53	86	48	72	13	2	.	.
63	54	75	59	95	36	43	66	64	53	58	13	1	.	.

88	53	87	57	72	35	77	48	59	61	58	13	1 .	.
62	87	69	100	61	55	72	51	64	57	109	14	3 .	.
54	109	112	98	55	40	48	85	65	101	102	13	3 .	.
53	74	111	58	87	43	43	65	80	109	72	12	2 .	.
99	44	71	53	92	42	61	41	107	78	50	11	1 .	.
86	45	52	53	79	78	86	53	95	51	53	12	0 .	.
53	58	90	52	53	78	63	48	55	49	56	11	0 .	.
75	77	93	58	63	46	42	43	54	60	76	10	1 .	.
71	75	83	58	58	35	40	60	60	63	60	7	2 .	.
56	53	60	82	73	36	54	88	60	62	62	9	-1 .	.
53	56	54	76	64	44	88	75	84	99	81	7	1 .	.
71	105	71	51	56	44	54	48	79	72	60	9	-1 .	.
48	96	56	51	64	46	45	37	59	56	56	8	-1 .	.
92	58	65	92	89	35	73	65	59	54	55	8	1 .	.
80	60	56	71	72	40	73	71	56	56	59	10	2 .	.
53	58	60	51	61	68	49	46	74	57	106	11	6 .	.
56	90	91	54	61			41	58	93	93	9	4 .	.
76	89	72	68	68			42	85	76	74	8	5 .	.
53	53	53	54	84			71	74	60	57	10	4 .	.
43	51	61	55				52	59	58	63	10	5 .	.
51	59	97	52				40	60	57	56	10	5 .	.
78	54	90	46				43	66	50	73	11	4 .	.
81	102	56	62				41	66	52	59	10	4 .	.
56	102	62	56				73	87	56	63	10	4 .	.
56	65	60	56				51	83	91	88	10	5 .	.
55	60	74	63				49	60	69	80	10	4 .	.
48	74	85	100				49	73	60	53	10	4 .	.
93	92	69	99				40	67	53	55	10	3 .	.
65	71	61	68				77	81	54	55	11	3 .	.
49	53	68	74				81	93	82	62	9	2 .	.
72	55	63	51				77	65	93	62	11	2 .	.
86	60	82	54				72	65	61	101	12	2 .	.
58	62	103	67				48	69	56	94	11	2 .	.
70	94	73	81				46	81	67	66	12	1 .	.
69	77	64	62				55	72	61	59	12	1 .	.
60	99	85	91				73	101	56	69	14	2 .	.
65	80	103	80				89	72	79	68	13	2 .	.
92	103	92	92				89	107	94	111	14	1 .	.
96	94	114	110				92	72	101	99	14	4 .	.
67	93	114	78				71	96	112	66	14	4 .	.
80	81	97	98				62	80	119	102	14	4 .	.
92	104	92	96				86	75	123	108	13	4 .	.
85	84	103	84				65	69	113	78	14	5 .	.
71	103	120	112				46	86	103	88	13	5 .	.
54	103	106	81				79	91	100	95	13	6 .	.
92	95	93	100				62	68	110	83	14	4 .	.
83	117	104	122				69	75	119	60	14	5 .	.
90	112	110	76				88	78	121	81	15	1 .	.
69	105	88	95				103	111	128	99	15	3 .	.
68	118	80	102				94	124	122	100	14	3 .	.
72	118	74	102				67	98	111	112	15	4 .	.
82	98	105	79				55	56	124	102	13	4 .	.
104	64	115	94				49	70	117	82	13	1 .	.
107	51	82	66				63	76	82	58	13	4 .	.
85	56	78	53				71	111	112	66	15	4 .	.
96	103	107	54				97	96	91	60	14	3 .	.
-1	118	83	70				-1	-1	77	94	14	4 .	.
1	83	71	86				1	1	61	79	14	3 .	.
46	65	88	82				80	89	58	65	13	4 .	.
54	65	85	59				87	63	61	64	13	3 .	.
50	94	83	53				66	70	60	84	13	3 .	.
74	73	81	55				66	64	102	61	13	4 .	.
84	64	89	54				65	83	94	64	13	3 .	.
52	75	72	84				87	70	66	107	13	5 .	.
52	60	70	91				69	69	58	98	12	4 .	.
57	52	84	65				59	82	61	96	13	4 .	.
80	110	81	48				52	65	66	67	14	4 .	.
63	95	61	65				63	78	111	58	14	5 .	.
83	60	79	55				52	99	107	63	13	4 .	.
88	43	69	84				78	103	70	60	10	5 .	.
59	65	65	80				53	70	55	65	12	4 .	.
42	56	60	52				44	55	63	77	11	4 .	.
44	68	55	48				49	59	101	66	12	-1 .	.
68	53	96	66				47	65	105	66	10	0 .	.
88	59	64	66				86	85	71	60	4	1 .	.
54	60	83	49				61	54	60	55	5	2 .	.
45	56	84	68				43	59	52	84	7	1 .	.
44	71	64	83				42	61	72	94	9	3 .	.
86	76	62	52				51	72	59	68	9	5 .	.
74	55	79	55				53	62	65	60	9	5 .	.
42	51	74	80				78	73	94	60	8	4 .	.
51	53	65	71				61	85	87	74	10	5 .	.
97	69	94	56	107	72	85	80	110	101	87	6 .	.	.
95	99	100	59	101	60	88	74	108	99	106	10 .	.	.
95	94	79	103	102	76	81	76	97	70	75	9 .	.	.
78	65	108	84	74	58	91	60	80	100	107	10 .	.	.
59	88	107	60	77	52	74	77	89	73	88	11 .	.	.
94	103	77	96	71	75	88	77	103	69	75	11 .	.	.
64	67	76	72	97	74	74	56	82	104	71	12 .	.	.
62	70	76	62	68	58	60	73	82	95	95	12 .	.	.
85	67	90	110	64	66	85	76	101	71	107	13 .	.	.
64	69	81	79	90	67	64	53	71	89	72	13 .	.	.
63	76	76	83	77	72	64	50	88	111	72	13 .	.	.
71	63	90	83	74	84	67	60	100	78	84	15 .	.	.
68	89	78	83	93	73	111	58	105	77	122	14 .	.	.
76	78	93	107	107	86	116	71	94	75	94	14 .	.	.
91	94	110	97	79	88	83	63	88	99	108	16 .	.	.
101	83	112	100	99	88	106	81	102	109	95	14 .	.	.
87	104	105	96	87	102	88	77	94	100	102	15 .	.	.
80	113	111	98	99	76	81	86	94	91	107	14 .	.	.
80	122	93	102	88	71	88	97	105	92	94	16 .	.	.
85	105	111	87	87	70	96	86	99	95	86	14 .	.	.
75	81	118	82	90	82	105	82	89	99	88	14 .	.	.
91	99	99	105	92	78	69	88	99	106	98	17 .	.	.

96	82	103	117	106	80	75	70	107	121	82	15	.	.	.
97	81	105	103	108	64	87	71	98	118	106	14	.	.	.
102	94	94	109	101	68	66	70	96	112	98	12	.	.	.
80	79	103	90	98	71	91	82	110	108	92	14	.	.	.
98	79	99	83	91	83	92	75	85	100	87	14	.	.	.
96	76	110	102	97	96	80	85	85	100	105	13	.	.	.
91	100	104	105	87	82	69	91	109	111	119	13	.	.	.
79	104	122	81	86	69	71	84	108	106	83	14	.	.	.
72	89	126	84	92	77	73	85	93	93	88	14	.	.	.
81	73	123	89	85	67	85	76	101	96	105	13	.	.	.
76	62	108	76	93	69	91	70	93	126	121	13	.	.	.
86	74	98	100	92	62	68	74	109	112	111	13	.	.	.
86	71	98	93	82	64	74	78	97	104	89	12	.	.	.
73	78	102	70	100	88	64	85	88	101	83	13	.	.	.
72	83	99	76	87	95	74	90	83	102	82	15	.	.	.
74	65	102	95	76	68	67	84	105	94	97	13	.	.	.
98	71	108	74	85	66	88	72	78	94	76	14	.	.	.
79	71	103	74	78	67	72	77	87	88	90	12	.	.	.
89	107	92	71	91	71	85	78	103	99	104	14	.	.	.
76	89	84	75	92	79	81	76	98	92	102	12	.	.	.
68	66	91	70	99	81	69	83	87	81	76	13	.	.	.
90	76	87	76	86	58	57	77	87	98	85	13	.	.	.
70	105	97	62	71	61	53	67	75	102	90	12	.	.	.
78	70	74	87	89	59	59	71	83	93	74	12	.	.	.
98	59	80	70	87	54	47	64	76	101	76	13	.	.	.
70	102	80	77	61	62	87	78	75	86	73	11	.	.	.
76	75	72	58	59	75	80	73	81	76	64	12	.	.	.
88	63	68	59	77	68	63	57	100	87	75	12	.	.	.
66	93	103	64	71	62	56	64	63	73	95	12	.	.	.
56	75	80	72	66	57	60	61	67	75	74	11	.	.	.
57	69	65	70	56	53	81	59	71	87	73	7	.	.	.
75	86	82	64	80	68	55	72	84	81	95	8	.	.	.
66	77	74	62	77	52	61	57	71	88	94	7	.	.	.
64	76	76	61	67	61	61	65	89	75	70	9	.	.	.
93	67	104	95	62	62	62	63	64	73	75	10	.	.	.
69	95	70	82	71	59	55	57	69	71	76	12	.	.	.
68	77	70	57	66	58	99	71	94	84	106	11	.	.	.
72	72	98	61	70	56	68	61	69	87	101	11	.	.	.
93	71	81	59	66	68	56	58	75	89	72	12	.	.	.
64	86	69	55	66	76	73	67	71	80	70	13	.	.	.
70	66	67	56	59	58	71	61	76	64	73	12	.	.	.
83	76	88	62	89	60	59	52	75	67	91	13	.	.	.
64	65	72	60	67	65	84	50	74	71	70	14	.	.	.
68	69	73	60	80	55	86	61	64	90	66	12	.	.	.
69	80	93	58	65	52	62	60	87	75	104	12	.	.	.
64	75	105	88	88	55	57	78	66	70	88	12	.	.	.
81	69	76	85	70	77	58	72	80	105	69	13	.	.	.
93	69	71	60	59	90	88	53	67	71	70	11	.	.	.
59	62	85	54	73	78	65	54	101	65	64	13	.	.	.
63	91	78	54	64	77	94	71	95	96	86	13	.	.	.
66	70	78	60	59	71	104	53	101	99	78	12	.	.	.
85	68	75	58	83	69	106	63	83	59	69	14	.	.	.
99	95	80	55	71	64	91	51	76	95	81	13	.	.	.
74	75	90	94	57	70	75	84	70	109	83	13	.	.	.
71	62	80	79	93	65	73	89	84	77	100	13	.	.	.
63	81	107	67	70	91	109	67	76	69	82	14	.	.	.
75	81	107	85	93	77	87	64	99	68	95	15	.	.	.
82	89	129	103	97	89	79	76	74	104	113	14	.	.	.
98	113	127	111	99	73	100	91	104	115	80	16	.	.	.
82	111	123	106	100	66	96	81	108	100	124	14	.	.	.
100	90	105	100	92	93	93	75	90	99	94	17	.	.	.
97	70	96	106	87	90	101	85	93	92	107	16	.	.	.
92	100	116	105	96	88	90	88	85	122	115	17	.	.	.
85	76	107	92	86	68	77	94	99	98	82	16	.	.	.
96	94	104	99	104	72	79	85	123	81	86	17	.	.	.
91	82	107	95	108	66	75	76	104	81	88	16	.	.	.
70	71	107	72	92	66	84	69	109	117	80	17	.	.	.
69	70	100	71	83	77	85	64	107	121	88	15	.	.	.
99	81	117	78	92	89	86	82	109	104	127	17	.	.	.
99	76	92	80	76	82	76	71	80	81	123	16	.	.	.
79	75	118	103	94	65	70	66	106	96	106	15	.	.	.
78	67	113	112	77	67	75	62	98	127	98	16	.	.	.
70	102	81	109	82	68	77	72	101	110	114	15	.	.	.
69	103	86	108	89	60	74	90	77	90	119	14	.	.	.
94	79	.	103	78	68	86	99	86	105	93	13	.	.	.
80	61	.	86	102	66	94	97	96	97	95	12	.	.	.
87	67	.	73	98	60	83	91	84	86	103	13	.	.	.
94	89	.	82	79	62	75	85	78	86	109	12	.	.	.
69	58	.	74	78	87	56	66	83	90	86	13	.	.	.
70	97	.	72	87	78	79	66	106	101	98	14	.	.	.
63	69	.	78	71	61	60	83	108	95	85	15	.	.	.
92	66	.	97	73	51	63	64	84	95	88	12	.	.	.
95	78	.	93	71	61	87	59	81	90	85	14	.	.	.
67	56	.	77	82	67	85	70	100	78	95	17	.	.	.
59	69	.	78	67	81	59	76	85	91	81	15	.	.	.
62	95	.	74	98	63	49	68	95	85	78	14	.	.	.
77	83	.	73	81	65	87	76	101	103	78	15	.	.	.
57	56	.	74	71	55	87	73	96	121	94	13	.	.	.
69	56	.	90	101	50	78	53	102	108	75	13	.	.	.
92	95	.	60	78	63	54	59	78	85	91	13	.	.	.
101	87	.	50	63	53	48	51	66	100	87	13	.	.	.
66	50	.	52	63	59	49	60	65	96	67	13	.	.	.
58	63	.	68	90	82	51	51	87	97	66	13	.	.	.
63	72	.	54	71	72	87	77	93	97	68	13	.	.	.
67	64	.	52	69	51	59	.	79	77	101	12	.	.	.
58	68	.	98	65	58	58	.	67	96	94	10	.	.	.
57	50	.	79	53	56	80	.	72	74	70	10	.	.	.
58	61	.	67	90	49	54	.	92	92	87	10	.	.	.
90	57	.	53	64	55	82	.	67	91	75	11	.	.	.
70	57	.	57	59	56	70	.	77	71	71	10	.	.	.
57	58	.	56	79	50	55	.	69	65	75	12	.	.	.
58	57	.	55	63	47	88	.	75	66	68	11	.	.	.
61	56	.	52	63	75	98	.	80	63	68	3	.	.	.
89	73	.	55	69			.	72	103	67	6	.	.	.
63	55	.	51	64			.	93	78	105	6	.	.	.

75	57	.	60	80			.	87	66	78	7	.	.	.
66	55	.	61	65			.	74	66	69	7	.	.	.
62	49	.	79	36			.	70	64	76	8	.	.	.
75	74	.	57				.	64	69	71	9	.	.	.
57	64	.	54				.	79	85	64	9	.	.	.
72	56	.	56				.	73	79	75	10	.	.	.
81	50	.	54				.	78	64	95	11	.	.	.
87	51	.	103				.	77	69	72	10	.	.	.
83	60	.	93				.	83	74	72	12	.	.	.
66	101	.	57				.	95	106	83	3	.	.	.
72	74	.	66				.	71	72	106	7	.	.	.
80	63	.	77				.	70	71	94	8	.	.	.
87	94	.	74				.	102	67	77	7	.	.	.
87	63	.	100				.	81	68	76	8	.	.	.
71	65	.	96				64	81	82	81	9	.	.	.
64	89	.	66				63	96	68	87	10	.	.	.
71	78	.	95				56	73	96	120	11	.	.	.
97	111	.	105				56	105	106	119	12	.	.	.
96	111	.	96				66	92	105	113	12	.	.	.
86	126	.	111				70	88	85	85	14	.	.	.
98	107	.	84				102	109	107	93	15	.	.	.
90	90	.	87				95	111	119	89	16	.	.	.
100	99	.	98				82	107	101	94	16	.	.	.
82	117	.	105				65	83	86	109	16	.	.	.
87	110	.	111				69	72	110	122	15	.	.	.
82	74	.	103				73	112	107	111	16	.	.	.
90	75	.	85				79	106	107	97	16	.	.	.
109	89	.	81				62	72	102	97	16	.	.	.
74	72	.	95				65	102	91	87	16	.	.	.
72	74	.	106				65	98	84	103	17	.	.	.
82	108	.	76				85	89	85	122	15	.	.	.
71	82	.	74				81	79	90	128	15	.	.	.
75	54	.	78				78	92	113	107	15	.	.	.
84	69	.	98				74	88	112	81	14	.	.	.
100	87	.	97				91	81	85	110	14	.	.	.
84	93	.	94				77	77	75	118	15	.	.	.
58	62	.	72				68	98	88	85	15	.	.	.
99	71	.	72				71	127	96	83	15	.	.	.
64	72	.	72				55	104	81	87	17	.	.	.
83	67	.	76				85	82	83	81	17	.	.	.
68	76	.	89				64	95	99	96	15	.	.	.
84	89	.	95				85	78	84	95	16	.	.	.
77	80	.	71				86	84	83	81	17	.	.	.
69	55	.	74				62	80	110	84	15	.	.	.
68	67	.	66				55	112	108	91	14	.	.	.
58	75	.	67				77	107	93	71	15	.	.	.
75	56	.	68				70	78	89	107	15	.	.	.
86	62	.	61				75	81	61	71	17	.	.	.
62	89	.	80				76	72	72	78	15	.	.	.
57	45	.	55				50	72	94	60	15	.	.	.
95	51	.	55				69	69	68	66	14	.	.	.
61	59	.	87				74	74	55	68	15	.	.	.
56	53	.	98				50	63	58	68	13	.	.	.
88	58	.	81				49	108	96	117	13	.	.	.
60	62	.	54				67	105	91	100	14	.	.	.
52	62	.	51				56	84	61	69	13	.	.	.
52	56	.	78				58	57	57	64	11	.	.	.
59	62	.	56				62	74	63	73	12	.	.	.
89	61	.	52				50	73	79	74	12	.	.	.
63	71	.	51				63	62	55	71	12	.	.	.
51	93	.	54				85	82	55	69	12	.	.	.
86	56	.	69				81	73	50	69	11	.	.	.
56	58	.	57				51	63	60	93	10	.	.	.
94	71	124	91	.	51	75	98	99	85	73	14	.	.	.
85	68	117	74	.	70	68	73	81	77	82	12	.	.	.
89	71	112	83	.	51	90	77	80	74	78	14	.	.	.
82	65	111	71	.	73	68	67	72	89	82	15	.	.	.
70	88	86	75	.	59	72	64	79	79	83	14	.	.	.
69	87	113	71	.	76	86	63	113	81	76	14	.	.	.
70	67	96	72	.	63	69	61	68	97	81	15	.	.	.
90	79	96	84	.	67	95	66	94	74	98	13	.	.	.
71	78	84	74	.	56	71	67	63	78	111	14	.	.	.
92	75	83	72	.	59	71	61	69	79	103	14	.	.	.
72	84	94	71	.	77	78	73	104	84	85	15	.	.	.
71	88	95	83	.	69	77	67	116	90	83	13	.	.	.
74	101	96	88	.	78	107	68	80	94	96	15	.	.	.
90	88	95	92	.	91	101	66	69	91	89	16	.	.	.
104	99	109	89	.	81	101	85	80	123	97	17	.	.	.
74	102	123	96	.	92	87	79	87	97	92	16	.	.	.
98	97	110	103	.	80	79	91	74	102	110	16	.	.	.
82	78	124	94	.	76	78	82	86	115	84	16	.	.	.
84	85	119	103	.	80	68	83	77	106	109	15	.	.	.
93	86	119	102	.	57	73	84	74	101	96	16	.	.	.
97	108	97	84	.	68	79	86	92	113	101	15	.	.	.
106	107	100	94	.	58	85	97	91	102	94	14	.	.	.
79	104	91	88	.	69	94	85	82	105	102	16	.	.	.
72	96	122	94	.	58	112	81	66	97	117	15	.	.	.
81	73	127	91	.	89	73	75	71	101	97	16	.	.	.
76	97	108	101	.	91	85	76	84	115	126	15	.	.	.
82	85	103	93	.	77	87	96	69	101	132	16	.	.	.
77	85	105	109	.	55	64	83	69	96	103	16	.	.	.
73	79	123	89	.	61	67	80	75	123	102	16	.	.	.
83	82	107	83	.	85	84	81	69	123	99	17	.	.	.
81	91	104	94	.	67	66	78	71	107	94	17	.	.	.
106	110	111	108	.	61	68	75	66	99	120	15	.	.	.
100	72	121	96	.	63	90	81	76	92	109	17	.	.	.
71	80	112	84	.	94	73	100	75	105	97	16	.	.	.
91	81	113	95	.	82	75	91	71	96	98	16	.	.	.
86	73	91	95	.	62	72	87	92	110	97	15	.	.	.
72	77	111	82	.	74	94	85	100	104	98	16	.	.	.
80	93	94	77	.	57	93	75	88	98	119	15	.	.	.
78	74	95	97	.	57	71	82	62	92	94	15	.	.	.
67	75	102	80	.	57	67	73	68	92	95	15	.	.	.
73	69	95	86	.	68	71	72	62	100	100	16	.	.	.

85	83	87	76	.	58	79	69	73	117	111	16	.	.	.
91	83	95	79	.	60	62	87	80	103	113	16	.	.	.
68	92	83	78	.	55	70	88	74	87	79	15	.	.	.
62	86	94	70	.	70	58	75	68	94	72	16	.	.	.
69	60	122	90	.	49	61	79	62	87	93	16	.	.	.
102	67	102	96	.	72	55	97	66	88	75	15	.	.	.
61	70	79	70	.	59	60	81	102	94	83	13	.	.	.
60	63	77	65	.	58	60	62	100	93	92	12	.	.	.
61	99	106	73	.	54	62	60	79	81	75	12	.	.	.
92	62	94	84	.	53	58	69	57	81	87	11	.	.	.
60	62	77	71	.	63	85	75	63	70	79	12	.	.	.
68	68	78	66	.	68	81	58	69	77	76	11	.	.	.
83	68	87	70	.	94	57	53	72	98	64	11	.	.	.
79	77	83	76	.	68	49	72	63	71	93	12	.	.	.
66	85	117	69	.	48	68	58	75	98	87	11	.	.	.
83	67	91	72	.	58	89	70	66	74	81	11	.	.	.
72	73	85	69	.	52	59	66	67	74	75	14	.	.	.
59	66	83	65	.	56	66	69	84	77	78	12	.	.	.
81	69	85	83	.	69	65	85	91	72	90	11	.	.	.
74	84	89	93	.	61	66	62	64	85	73	12	.	.	.
66	77	84	71	.	60	77	65	77	67	75	11	.	.	.
68	80	82	70	.	56	66	73	66	96	72	11	.	.	.
63	66	101	65	.	65	61	61	73	73	73	11	.	.	.
85	77	84	70	.	61	60	69	68	72	73	11	.	.	.
79	77	82	74	.	55	91	71	68	90	69	10	.	.	.
67	78	83	83	.	58	86	60	64	85	78	7	.	.	.
72	73	101	71	.	59	59	68	73	79	77	8	.	.	.
79	66	86	77	.	55	64	67	59	109	84	9	.	.	.
74	68	89	68	.	55	68	67	67	89	96	10	.	.	.
68	94	117	75	.	52	86	62	91	89	73	12	.	.	.
82	84	113	83	.	73	71	77	93	100	79	10	.	.	.
86	71	86	71	.	53	94	60	65	78	84	13	.	.	.
81	78	84	80	.	60	98	62	64	89	78	12	.	.	.
74	80	99	78	.	73	89	85	69	74	97	11	.	.	.
79	87	88	79	.	57	60	71	69	82	79	12	.	.	.
77	78	109	78	.	59	76	63	79	92	90	12	.	.	.
89	104	91	96	.	87	94	81	113	90	97	14	.	.	.
78	112	119	95	.	97	74	73	93	110	89	17	.	.	.
109	104	126	112	.	92	96	74	71	124	131	16	.	.	.
97	79	108	95	.	92	97	90	85	113	99	16	.	.	.
87	118	137	90	.	73	80	106	93	103	114	17	.	.	.
91	86	127	109	.	81	74	98	109	116	97	17	.	.	.
106	72	132	113	.	77	89	98	105	119	93	17	.	.	.
100	79	96	83	.	71	74	87	84	116	104	17	.	.	.
92	75	114	97	.	68	95	79	79	103	131	17	.	.	.
90	83	106	106	.	68	103	68	84	109	123	16	.	.	.
90	83	97	107	.	66	93	71	78	100	111	17	.	.	.
75	77	91	99	.	73	74	70	79	103	99	18	.	.	.
84	105	99	89	.	72	77	67	93	107	99	15	.	.	.
97	103	113	93	.	80	75	75	95	118	119	17	.	.	.
96	79	106	113	.	90	79	78	76	119	101	16	.	.	.
101	55	101	107	.	90	89	77	103	106	100	16	.	.	.
90	61	125	81	.	68	71	87	88	117	98	16	.	.	.
88	59	117	81	.	60	77	106	105	98	101	16	.	.	.
82	73	100	94	.	73	91	92	105	114	125	16	.	.	.
96	62	111	101	.	65	83	96	74	102	128	17	.	.	.
82	74	96	79	.	64	70	88	88	101	102	17	.	.	.
79	100	113	88	.	73	68	76	88	107	106	15	.	.	.
79	86	89	86	.	91	84	83	81	106	90	15	.	.	.
96	85	106	80	.	74	78	88	82	112	115	15	.	.	.
90	59	113	90	.	69	70	88	73	107	128	14	.	.	.
75	81	90	94	.	68	96	77	72	113	89	15	.	.	.
71	69	108	76	.	63	72	83	93	108	104	14	.	.	.
70	82	96	88	.	62	70	73	85	99	104	16	.	.	.
93	77	108	70	.	69	69	81	70	99	93	16	.	.	.
94	98	83	75	.	93	73	94	76	108	87	16	.	.	.
78	90	95	89	.	53	60	86	71	107	105	19	.	.	.
87	56	115	103	.	62	66	76	68	95	77	14	.	.	.
72	95	84	80	.	70	90	81	76	108	83	15	.	.	.
70	55	108	71	.	90	83	89	97	106	94	15	.	.	.
63	65	114	94	.	72	55	76	98	83	85	14	.	.	.
84	58	106	100	.	69	55	87	65	90	73	14	.	.	.
64	67	90	94	.	51	47	65	63	81	71	12	.	.	.
68	63	103	74	.	52	72	59	64	88	83	12	.	.	.
92	69	85	77	.	51	62	60	86	87	88	11	.	.	.
76	56	88	71	.	49	52	66	68	91	79	14	.	.	.
65	64	96	70	.	62	64	69	65	86	85	11	.	.	.
66	61	85	72	.	81	66	65	63	85	84	7	.	.	.
61	80	84	70	.	52	61	72	64	96	77	8	.	.	.
90	62	102	82	.	51	61	65	67	98	76	8	.	.	.
88	65	96	68	.	61	64	71	78	80	75	10	.	.	.
66	65	84	71	.	73	58	66	87	89	95	11	.	.	.
68	80	101	65	.	58	62	68	69	85	84	11	.	.	.
67	68	89	71	.	69	80	60	67	104	74	11	.	.	.
67	58	84	83	.	54	71	64	70	82	74	11	.	.	.
79	64	95	88	.	54	70	71	74	83	94	10	.	.	.
76	87	88	71	.	63	65	63	71	78	73	11	.	.	.
75	63	93	69	.	49	66	61	69	78	67	12	.	.	.
87	88	89	62	.	45	69	85	108	83	73	13	.	.	.
81	69	100	65	.	50	60	81	92	83	75	10	.	.	.
69	63	112	89	.	68	66	65	70	74	95	11	.	.	.
73	51	96	82	.	61	82	61	73	83	73	11	.	.	.
76	86	102	67	.	61	65	66	68	97	73	11	.	.	.
73	66	94	67	.	56	68	67	72	79	84	10	.	.	.
81	81	83	75	.	58	82	68	102	108	91	12	.	.	.
83	65	89	70	.	75	68	85	90	102	83	11	.	.	.
71	64	113	74	.	59	74	68	67	87	78	10	.	.	.
73	64	94	116	.	61	90	96	85	82	85	10	.	.	.
82	71	118	101	.	62	73	77	77	86	72	11	.	.	.
88	69	90	86	.	57	73	74	83	84	82	12	.	.	.
91	69	89	78	.	65	95	71	80	86	90	11	.	.	.
74	103	100	79	.	68	114	67	87	104	86	12	.	.	.
83	114	94	95	.	96	96	64	82	128	115	13	.	.	.
86	117	118	94	.	71	88	82	86	106	119	15	.	.	.
83	113	146	107	.	67	96	80	81	111	92	17	.	.	.

119	108	115	101	.	71	82	81	93	97	101	16	.	.	.
101	89	134	87	.	96	87	73	122	108	104	17	.	.	.
101	73	102	83	.	84	86	71	103	118	117	17	.	.	.
74	91	102	109	.	59	71	96	101	121	119	17	.	.	.
88	69	101	102	.	58	63	70	91	114	114	16	.	.	.
88	74	107	89	.	69	79	77	90	99	100	18	.	.	.
95	72	115	107	.	65	78	105	89	93	98	17	.	.	.
89	70	97	80	.	57	101	99	86	109	95	17	.	.	.
83	69	105	107	.	78	96	96	82	114	104	15	.	.	.
81	95	123	96	.	99	88	76	107	99	103	17	.	.	.
82	110	132	90	.	75	74	78	122	97	123	17	.	.	.
96	94	119	94	.	52	74	97	113	108	116	16	.	.	.
84	67	103	101	.	62	79	84	100	96	100	16	.	.	.
102	64	105	88	.	62	72	79	83	97	105	16	.	.	.
86	78	101	85	.	61	89	75	123	97	99	16	.	.	.
73	66	105	84	.	60	84	76	74	121	115	16	.	.	.
86	80	119	101	.	65	67	89	87	115	128	15	.	.	.
83	59	101	111	.	65	66	83	83	108	103	17	.	.	.
68	78	106	83	.	72	71	86	92	103	97	16	.	.	.
64	66	102	77	.	78	62	87	114	93	96	16	.	.	.
72	80	110	86	.	72	90	71	82	98	115	16	.	.	.
91	87	105	108	.	64	70	61	90	100	115	15	.	.	.
83	69	96	70	.	62	64	65	93	108	107	16	.	.	.
78	62	106	82	.	64	66	78	87	105	105	15	.	.	.
81	75	114	72	.	59	94	80	86	92	117	15	.	.	.
93	63	88	84	.	62	77	86	83	103	97	15	.	.	.
65	80	97	96	.	54	73	96	79	97	98	14	.	.	.
69	90	104	107	.	63	65	70	80	98	103	14	.	.	.
107	90	119	95	.	66	73	68	86	99	129	15	.	.	.
65	75	97	77	.	50	59	60	101	83	109	14	.	.	.
88	62	116	70	.	56	58	69	80	80	79	15	.	.	.
90	64	79	76	.	60	52	96	98	80	81	13	.	.	.
69	59	99	68	.	55	63	86	76	81	75	14	.	.	.
59	60	97	70	.	83	60	62	70	97	78	11	.	.	.
65	67	90	73	.	77	68	72	66	74	84	11	.	.	.
93	63	79	87	.	64	62	65	71	84	96	10	.	.	.
72	66	89	76	.	56	82	67	63	85	72	7	.	.	.
65	61	84	71	.	52	90	66	68	79	87	9	.	.	.
66	77	97	84	.	52	61	69	77	80	74	10	.	.	.
64	79	82	71	.	46	73	67	77	72	78	11	.	.	.
68	70	95	70	.	62	60	73	73	74	91	11	.	.	.
71	87	80	79	.	51	60	72	77	122	89	11	.	.	.
62	68	109	72	.	66	61	66	79	99	74	12	.	.	.
82	59	85	91	.	51	62	61	63	78	81	12	.	.	.
66	68	82	70	.	54	73	61	89	79	76	12	.	.	.
84	84	88	67	.	50	67	61	68	96	82	12	.	.	.
68	71	84	72				61	75	86	75	12	.	.	.
68	62	81	73				61	93	78	84	13	.	.	.
80	62	107	89				81	89	77	74	12	.	.	.
67	81	87	75				82	69	84	90	13	.	.	.
69	61	103	77				68	67	79	85	13	.	.	.
72	70	94	64				68	73	79	94	6	.	.	.
82	68	102	81				68	71	77	81	7	.	.	.
80	68	83	77				67	84	85	86	9	.	.	.
74	87	86	84				64	79	76	79	11	.	.	.
96	67	88	84				63	84	81	99	11	.	.	.
79	70	107	82				73	78	105	99	11	.	.	.
73	70	107	72				72	96	75	72	10	.	.	.
97	85	91	101				67	72	72	84	13	.	.	.
98	70	90	73				72	80	89	84	11	.	.	.
79	75	81	93				81	80	81	75	12	.	.	.
70	87	92	77				67	82	83	103	13	.	.	.
73	105	98	90				78	85	85	96	14	.	.	.
74	117	104	84				68	98	114	123	16	.	.	.
90	97	119	88				69	78	114	114	17	.	.	.
109	94	127	115				109	91	122	98	16	.	.	.
87	62	120	95				108	125	121	102	17	.	.	.
91	78	102	84				107	105	96	96	16	.	.	.
89	79	108	83				96	76	99	107	16	.	.	.
94	82	119	86				88	105	106	105	16	.	.	.
90	80	102	109				71	102	120	95	16	.	.	.
70	88	102	107				75	88	100	115	16	.	.	.
71	105	102	99				83	82	100	120	16	.	.	.
93	91	116	90				97	78	102	101	16	.	.	.
92	79	93	87				78	92	100	95	16	.	.	.
88	72	98	93				79	82	104	105	16	.	.	.
104	77	94	82				76	98	120	107	16	.	.	.
104	73	117	87				92	91	113	128	14	.	.	.
90	71	93	92				71	114	102	135	14	.	.	.
89	70	89	84				70	115	108	96	16	.	.	.
83	76	98	111				82	106	112	103	15	.	.	.
87	102	104	110				81	107	116	96	14	.	.	.
87	80	106	91				81	80	110	99	16	.	.	.
76	79	92	85				98	80	108	93	15	.	.	.
95	80	92	83				96	92	106	96	16	.	.	.
97	82	99	89				93	105	110	108	14	.	.	.
119	69	104	89				71	83	95	106	15	.	.	.
82	77	125	92				68	75	103	95	15	.	.	.
88	76	112	84				71	77	116	96	14	.	.	.
91	58	104	106				79	100	90	84	14	.	.	.
88	69	91	102				76	77	97	106	15	.	.	.
78	83	96	73				78	85	91	81	14	.	.	.
93	75	94	69				69	77	92	92	13	.	.	.
69	73	94	73				93	102	96	86	13	.	.	.
70	76	97	84				96	104	110	82	14	.	.	.
89	62	128	103				85	71	104	73	14	.	.	.
95	73	115	85				94	104	78	79	15	.	.	.
84	72	105	68				64	112	82	67	16	.	.	.
74	62	81	67				66	95	85	91	15	.	.	.
84	69	81	98				67	67	74	74	13	.	.	.
67	81	80	67				64	72	85	80	15	.	.	.
90	76	79	74				73	73	73	75	14	.	.	.
72	68	92	74				65	72	93	95	9	.	.	.
84	72	82	78				66	78	74	74	9	.	.	.
72	87	86	70				65	67	83	72	10	.	.	.

76	73	95	67	63	73	80	75	11 .	.	.
100	77	84	83	68	94	86	103	11 .	.	.
78	68	83	79	66	79	81	73	12 .	.	.
73	75	92	64	72	73	72	73	11 .	.	.
70	67	82	70	66	74	97	89	11 .	.	.
						81	76	.	.	.
						76	78	.	.	.
								.		.
