# Supplementary material for: Does prenatal alcohol exposure cause a metabolic syndrome? (Non-)evidence from a mouse model of fetal alcohol spectrum disorder
Source: PLoS One. 2018 Jun 28;13(6):e0199213. doi: 10.1371/journal.pone.0199213 (PMC6023152; doi:10.1371/journal.pone.0199213)
Supplement: S1 Dataset — (ZIP) [file pone.0199213.s010.zip › New folder/Water intake.pdf]

Blk	3	3	3	3	4	4	5	7	7	8	3	4	4	7	7	8	10	10	10	12	12	12
Box	Box-3	Box-4	Box-5	Box-6	Box-2	Box-3	Box-4	Box-3	Box-4	Box-4	Box-7	Box-5	Box-8	Box-1	Box-7	Box-2	Box-6	Box-5	Box-4	Box-1	Box-2	Box-5
ID	6.4	9.6	10.6	11.1	17.8	18.7	28.7	41	42	53.5	12.4	23.1	27.7	37	46	50.6	62	63	69	74	76	81
Sex	F	F	F	F	F	F	F	F	F	F	F	F	F	F	F	F	F	F	F	F	F	F
Exposure	EtOH	EtOH	EtOH	EtOH	EtOH	EtOH	EtOH	EtOH	EtOH	EtOH	H2O	H2O	H2O	H2O	H2O	H2O	H2O	H2O	H2O	H2O	H2O	H2O
Tx	FE	FE	FE	FE	FE	FE	FE	FE	FE	FE	FH	FH	FH	FH	FH	FH	FH	FH	FH	FH	FH	FH
	0	0	0	0	0	0	0	0	0	0	0	0	0	0	0	0	0	0	0	0	0	0
	0	0	0	0.05	0	0.18	0	0	0	0.08	0	0.01	0	0	0	0.03	0	0.06	0.01	0.01	0	0
	0	0	0	0.05	0	0.18	0	0	0	0.08	0	0.02	0	0.02	0	0.03	0	0.06	0.02	0.01	0	0
	0	0	0.01	0.06	0.03	0.18	0	0	0	0.08	0	0.02	0	0.02	0	0.04	0.01	0.06	0.02	0.02	0	0
	0	0	0.01	0.06	0.03	0.18	0	0	0	0.08	0	0.02	0	0.03	0	0.04	0.02	0.06	0.02	0.02	0	0
	0	0	0.01	0.06	0.03	0.18	0	0	0	0.08	0.01	0.03	0	0.03	0	0.04	0.02	0.06	0.03	0.03	0	0
	0	0	0.01	0.29	0.04	0.18	0	0.06	0	0.08	0.01	0.03	0	0.03	0	0.04	0.02	0.06	0.03	0.03	0	0
	0	0	0.02	0.48	0.04	0.18	0	0.27	0	0.32	0.01	0.03	0	0.04	0	0.04	0.02	0.06	0.04	0.04	0	0
	0	0.01	0.02	0.49	0.04	0.39	0	0.36	0	0.4	0.01	0.03	0	0.06	0	0.04	0.03	0.18	0.05	0.04	0	0
	0	0.01	0.02	0.69	0.04	0.69	0	0.36	0.4	0.68	0.01	0.03	0	0.06	0	0.04	0.03	0.87	0.05	0.05	0	0
	0	0.01	0.27	0.96	0.04	0.96	0	0.36	0.47	0.82	0.01	0.2	0	0.06	0	0.04	0.04	1.02	0.06	0.05	0.12	0
	0	0.01	0.36	1.02	0.04	1.13	0	0.41	0.54	0.82	0.01	0.8	0	0.06	0.35	0.04	0.04	1.08	0.06	0.05	0.13	0
	0	0.01	0.73	1.21	0.04	1.13	0	0.55	0.85	0.89	0.03	0.81	0	0.06	0.35	0.04	0.04	1.16	0.06	0.06	0.13	0
	0	0.01	1	1.31	0.04	1.15	0	0.55	1.1	0.98	0.32	0.96	0.98	0.06	0.76	0.09	0.05	1.33	0.06	0.07	0.6	0.33
	0	0.29	1.01	1.31	0.04	1.35	0	0.55	1.1	1.1	0.67	1.37	1.49	0.06	0.96	0.44	0.05	1.63	0.06	0.07	0.62	0.95
	0	0.9	1.11	1.32	0.04	1.79	0	0.56	1.25	1.19	0.68	1.63	1.49	0.62	0.98	0.94	0.05	1.76	0.07	0.48	0.8	1.17
	0.09	1.01	1.49	1.63	0.04	1.85	0.77	0.56	1.55	1.63	0.68	1.63	1.49	1.07	1.35	0.95	0.05	2.05	0.07	0.74	0.89	1.4
	0.35	1.4	1.9	1.63	0.04	2.16	1.18	0.62	1.69	1.69	0.8	2.17	1.57	1.24	1.64	0.95	0.06	2.26	0.08	1	1	1.94
	0.88	1.4	1.91	1.63	0.05	2.2	1.58	0.85	1.76	1.74	1.13	2.28	1.77	1.61	2	0.95	0.14	2.41	0.08	1.28	1.03	1.94
	0.88	1.6	1.91	1.63	0.05	2.53	1.61	0.88	1.76	1.74	1.33	2.28	2.1	1.8	2	1.27	0.18	2.41	0.08	1.36	1.22	2.59
	0.88	1.86	2.01	1.64	0.05	2.53	1.62	0.91	1.76	1.74	1.4	2.29	2.51	2.02	2.01	1.61	0.27	2.43	0.08	1.61	1.28	2.59
	0.93	2.15	2.25	1.64	0.1	2.53	1.62	0.91	1.76	1.79	1.84	2.29	2.73	2.23	2.01	2.14	0.28	2.51	0.08	1.69	1.28	2.59
	1.21	2.27	2.39	1.64	0.14	2.53	1.62	0.96	1.76	2.14	1.9	2.29	2.74	2.47	2.29	2.2	0.93	2.51	0.08	2.09	1.28	3.13
	1.21	2.46	2.39	1.9	0.22	2.53	1.62	0.96	1.76	2.17	1.95	2.58	2.79	2.67	2.58	2.24	1.03	2.51	0.19	2.1	1.28	3.51
	1.38	2.73	2.39	2.11	0.22	2.83	1.62	1.04	1.76	2.23	2.38	3.05	2.79	2.78	2.73	2.24	1.14	2.51	0.19	2.28	1.28	3.51
	1.73	2.84	2.76	2.11	0.22	2.84	1.62	1.04	1.8	2.34	2.4	3.14	2.79	2.78	2.84	2.25	1.3	2.57	0.2	2.29	1.28	3.51
	1.95	2.85	2.76	2.11	0.22	2.84	1.67	1.04	1.8	2.34	2.45	3.14	2.79	2.78	2.85	2.26	1.36	2.78	0.2	2.29	1.28	3.51
	2.02	2.95	2.77	2.12	0.22	2.85	1.94	1.04	1.8	2.34	2.65	3.15	2.79	2.78	2.85	2.26	1.36	2.91	0.2	2.29	1.28	3.51
	2.02	2.96	2.95	2.12	0.22	2.99	2.29	1.04	1.8	2.34	2.65	3.15	3.27	2.78	2.86	2.26	1.36	2.91	0.26	2.3	1.29	3.52
	2.17	2.96	3.1	2.12	0.22	2.99	2.31	1.04	1.8	2.35	2.66	3.15	3.27	2.78	2.86	2.67	1.36	2.97	0.31	2.3	1.44	3.52
	2.18	2.97	3.27	2.12	0.22	2.99	2.33	1.04	1.8	2.37	2.66	3.16	3.27	2.78	3.21	3.02	1.37	2.99	0.32	2.3	1.44	3.52
	2.2	3.48	3.27	2.12	0.24	2.99	2.35	1.3	1.8	2.37	2.67	3.16	3.28	2.78	3.21	3.02	1.37	2.99	0.33	2.74	1.44	3.52
	2.2	3.58	3.28	2.12	0.43	2.99	2.36	1.6	1.8	2.39	2.67	3.23	3.28	2.78	3.22	3.02	1.38	3.11	0.33	2.76	1.45	3.52
	2.28	3.59	3.41	2.12	0.67	2.99	2.36	1.68	1.8	2.73	2.67	3.54	3.52	2.78	3.4	3.06	1.38	3.11	0.33	2.99	1.45	3.52
	2.29	3.6	3.41	2.12	0.77	3.68	2.47	1.93	1.8	2.98	2.86	3.96	3.52	2.83	3.71	3.06	1.38	3.28	0.33	2.99	1.45	3.52
	2.29	3.6	3.42	2.13	1.18	3.78	2.59	2.1	1.81	3.23	2.91	4.12	3.52	3.26	3.9	3.06	1.39	3.28	0.33	2.99	1.45	4.48
	2.29	3.6	3.42	2.13	1.29	3.78	2.62	2.1	1.81	3.23	3.23	4.12	3.62	3.54	3.9	3.51	1.39	3.33	0.33	2.99	1.45	4.56
	2.29	3.9	3.42	2.31	1.66	3.78	2.66	2.1	1.81	3.23	3.23	4.13	3.78	3.54	3.91	3.51	1.4	3.6	0.34	3	1.45	4.69
	2.29	3.92	3.55	2.61	1.84	3.78	2.68	2.1	1.81	3.23	3.23	4.13	3.82	3.54	3.91	3.51	1.4	3.6	0.34	3	1.45	4.82
	2.29	3.92	3.56	2.62	1.97	4.41	2.73	2.1	1.82	3.23	3.23	4.13	3.82	3.68	3.92	3.51	1.62	3.6	0.35	3.02	1.72	4.82
	2.29	3.92	3.56	2.62	1.98	4.41	3.19	2.1	1.82	3.23	3.24	4.38	4.01	3.92	3.92	3.55	1.88	3.62	0.35	3.4	1.72	4.82
	2.41	4.08	3.56	2.62	1.98	4.41	3.25	2.1	1.85	3.23	3.24	4.38	4.01	4.08	4.19	3.55	1.96	3.62	0.35	3.46	2.19	4.82
	2.69	4.08	3.75	2.62	1.98	4.41	3.25	2.1	1.85	3.23	3.38	4.39	4.01	4.08	4.19	3.55	1.97	3.62	0.37	3.47	2.19	4.82
	2.69	4.09	3.75	2.63	2	4.43	3.26	2.1	1.85	3.23	3.5	4.39	4.06	4.08	4.19	3.55	1.97	3.67	0.38	3.47	2.19	4.82
	2.69	4.09	3.75	3.05	2.02	4.43	3.28	2.1	1.85	3.44	3.5	4.69	4.1	4.08	4.35	3.56	1.97	3.88	0.38	3.47	2.19	4.82
	2.69	4.23	3.76	3.05	2.02	4.76	3.28	2.1	1.85	3.44	3.5	4.69	4.24	4.08	4.36	3.58	1.98	3.88	0.38	3.47	2.38	4.82
	2.84	4.24	3.88	3.05	2.15	4.78	3.3	2.1	1.85	3.46	3.5	4.7	4.24	4.38	4.36	3.58	1.98	3.88	0.39	3.47	2.38	4.82
	2.84	4.24	3.89	3.05	2.15	4.81	3.62	2.1	2.09	3.56	3.51	4.7	4.24	4.44	4.36	3.58	1.98	3.88	0.39	3.74	2.38	4.82
	2.93	4.27	3.89	3.05	2.15	4.82	3.62	2.1	2.21	3.56	3.83	4.82	4.24	4.44	4.37	3.58	1.99	3.88	0.39	3.74	2.38	4.82
	2.93	4.29	3.89	3.06	2.15	4.83	3.62	2.1	2.23	3.62	3.84	4.82	4.24	4.55	4.71	3.58	1.99	3.88	0.39	3.74	2.78	4.82
	2.93	4.29	3.89	3.06	2.43	5.12	3.62	2.1	2.23	3.62	3.84	4.82	4.48	4.55	4.71	3.58	2	3.88	0.4	3.74	2.78	4.82
	3.29	4.29	3.9	3.06	2.8	5.12	3.62	2.1	2.25	3.62	3.85	4.83	4.48	4.57	4.72	3.6	2.06	4	0.4	3.74	2.78	4.82
	3.59	4.29	3.9	3.06	3	5.12	3.62	2.1	2.25	3.62	3.85	4.83	4.48	4.68	4.72	4.08	2.14	4.16	0.41	3.74	2.78	4.82
	3.63	4.3	4.08	3.07	3.05	5.13	3.64	2.1	2.27	3.74	3.86	4.83	4.7	4.7	4.73	4.09	2.14	4.16	0.41	3.74	2.89	5.18
	3.63	4.3	4.09	3.51	3.13	5.15	3.83	2.2	2.32	3.96	3.86	4.84	4.7	4.89	4.85	4.1	2.14	4.16	0.42	3.74	2.89	5.41
	3.67	4.3	4.09	3.52	3.14	5.15	3.87	2.2	2.64	4	4.22	4.84	4.7	5.04	5.09	4.1	2.15	4.16	0.42	3.75	2.89	5.41
	3.78	4.3	4.09	3.52	3.14	5.15	3.92	2.25	2.64	4	4.28	5.09	4.85	5.04	5.09	4.1	2.3	4.16	0.43	3.75	2.91	5.41
	3.81	4.3	4.1	3.52	3.23	5.15	3.93	2.25	2.64	4.12	4.52	5.09	4.85	5.04	5.1	4.15	2.3	4.16	0.43	3.82	2.91	5.44
	3.95	4.3	4.1	3.52	3.34	5.52	4.04	2.27	2.64	4.28	4.52	5.1	4.85	5.04	5.17	4.15	2.31	4.16	0.44	3.95	2.91	5.67
	4.38	4.31	4.11	3.52	3.34	5.6	4.11	2.28	2.67	4.32	4.52	5.1	4.86	5.04	5.36	4.15	2.31	4.16	0.44	4.08	3.11	5.67
	4.38	4.31	4.23	3.79	3.35	5.7	4.13	2.28	2.89	4.32	4.53	5.36	5.21	5.04	5.36	4.15	2.31	4.16	0.45	4.08	3.11	5.76
	4.38	4.32	4.48	3.79	3.61	5.7	4.4	2.29	2.89	4.32	4.54	5.49	5.22	5.11	5.36	4.16	2.32	4.16	0.45	4.09	3.11	5.96
	4.38	4.32	4.48	3.8	3.77	5.7	4.4	2.29	2.89	4.32	4.54	5.71	5.22	5.11	5.36	4.57	2.32	4.16	0.45	4.09	3.12	6.04
	4.38	4.32	4.49	3.8	3.77	6.17	4.4	2.59	2.89	4.56	4.55	5.77	5.22	5.14	5.36	4.57	2.33	4.16	0.46	4.1	3.16	6.04
	4.38	4.48	4.49	3.8	3.77	6.2	4.48	2.7	2.89	4.7	4.55	5.86	5.22	5.14	5.37	4.57	2.41	4.16	0.46	4.33	3.17	6.06
	4.66	4.49	4.63	3.8	3.77	6.2	4.54	2.84	3.14	4.77	4.81	5.86	5.22	5.14	5.64	4.59	2.52	4.16	0.47	4.33	3.18	6.22
	4.72	4.49	4.63	3.8	3.77	6.25	4.56	2.84	3.14	4.77	4.87	5.87	5.23	5.14	5.65	4.6	2.56	4.16	0.47	4.34	3.19	6.22
	4.73	4.52	4.63	4.04	4.04	6.25	4.62	2.84	3.47	4.77	4.87	5.87	5.23	5.3	5.73	4.79	2.56	4.19	0.47	4.34	3.22	6.22
	4.73	4.52	4.67	4.04	4.21	6.29	4.7	2.84	3.5	4.8	4.87	5.87	5.4	5.43	5.74	4.79	2.56	4.3	0.48	4.34	3.22	6.27
	4.73	4.55	4.96	4.05	4.23	6.59	4.7	2.92	3.7	4.9	4.88	5.87	5.52	5.58	5.74	5.11	2.56	4.38	0.48	4.43	3.22	6.37
	4.73	4.7	5.03	4.05	4.23	6.74	4.89	3.08	3.71	4.97	4.92	6.08	5.55	5.6	5.74	5.11	2.57	4.48	0.49	4.51	3.52	6.41
	4.98	4.81	5.03	4.21	4.23	6.74	5.21	3.13	3.98	5.01	5.01	6.14	5.55	5.6	5.74	5.12	2.66	4.66	0.49	4.51	3.52	6.41
	5.02	4.81	5.28	4.35	4.24	6.83	5.21	3.42	4.2	5.26	5.07	6.21	5.75	5.64	5.85	5.18	2.81	4.78	0.5	4.79	3.8	6.41
	5.07	4.94	5.29	4.4	4.56	6.83	5.21	3.54	4.37	5.27	5.07	6.33	5.85	5.79	6.1	5.57	2.97	4.78	0.5	4.8	3.93	6.61
	5.12	5.23	5.52	4.41	4.66	6.83	5.44	3.55	4.37	5.27	5.13	6.4	5.85	6	6.1	5.58	2.99	5.09	0.51	4.8	4.02	6.63
	5.12	5.23	5.77	4.41	4.66	6.84	5.46	3.97	4.37	5.46	5.46	6.75	6	6.03	6.1	5.59	3.19	5.4	0.52	4.95	4.16	6.63
	5.18	5.39	5.77	4.71	4.73	6.84	5.51	4.08	4.76	5.58	5.55	6.88	6.02	6.03	6.31	5.85	3.48	5.43	0.52	4.95	4.41	6.88
	5.18	5.4	5.78	4.8	4.74	6.94	5.55	4.14	4.79	5.71	5.56	6.98	6.26	6.05	6.31	5.86	3.48	5.43	0.52	4.95	4.47	6.88
	5.47	5.4	5.89	4.8	4.75	7.23	5.58	4.14	4.79	5.88	5.56	7.03	6.26	6.11	6.45	5.94	3.48	5.43	0.52	5.08	4.75	7.11
	5.76	5.4	5.89	4.8	4.98	7.24	5.63	4.43	5.2	5.98	5.8	7.04	6.4	6.11	6.52	6.16	4	5.6	0.52	5.09	4.96	7.19
	6.03	5.58	5.9	4.8	5	7.57	5.67	4.7	5.2	6.03	5.8	7.08	6.67	6.11	6.55	6.26	4.01	5.87	0.52	5.27	4.96	7.19
	6.15	5.62	6.05	4.8	5	7.76	6.01	4.7	5.2	6.05	5.81	7.08	6.94	6.5	6.74	6.26	4.01	6.2	0.53	5.39	5.34	7.19
	6.41	5.91	6.21	5.16	5	7.96	6.15	4.7	5.21	6.05	6.01	7.08	6.98	6.6	6.81	6.67	4.02	6.35	1.37	5.39	5.54	7.53
	6.58	5.92	6.21	5.17	5.01	8.24	6.32	4.7	5.21	6.07	6.09	7.08	6.99	6.6	6.94	6.96	4.44	6.35	1.96	5.64	5.78	8.21
	6.91	6.21	6.21	5.5	5.17	8.35	6.34	4.7	5.23	6.15	6.43	7.09	6.99	6.8	6.94	7.14	4.74	6.35	2.25	5.81	5.78	8.27
	6.94	6.24	6.57	5.61	5.17	8.41	6.34	5.12	5.63	6.38	6.56	7.09	6.99	6.83	6.94	7.14	5.01	6.54	2.72	5.86	5.78	8.35
	7.11	6.35	6.75	5.62	5.17	8.41	6.36	5.21	5.63	6.55	6.61	7.09	7.29	7.19	6.95	7.38	5.01	6.54	2.84	6.26	5.82	8.35
	7.3	6.65	6.76	5.62	5.17	8.54	6.38	5.22	5.63	6.74	6.61	7.1	7.37	7.23	6.97	7.47	5.01	6.54	2.84	6.35	5.95	8.42
	7.4	6.87	6.76	5.75	5.17	8.54	6.38	5.5	5.71	6.82	7.02	7.1	7.37	7.23	6.97	7.47	5.02	6.56	2.91	6.46	6.08	8.68
	7.42	6.89	6.84	5.88	5.17	8.54	6.38	5.5	5.92	6.82	7.02	7.49	7.37	7.23	7.16	7.53	5.02	6.56	3.14	6.53	6.29	8.95
	7.42	6.89	6.85	5.88	5.17	8.54	6.45	5.5	5.92	6.82	7.03	7.49	7.43	7.23	7.57	7.55	5.02	6.72	3.15	6.8	6.29	9.17
	7.42	6.89	6.93	5.89	5.17	8.54	6.49	5.5	5.92	6.82	7.03	7.56	7.56	7.23	7.57	7.55	5.03	6.89	3.16	6.89	6.37	9.17
	7.52	6.89	6.93	5.89	5.17	8.54	6.5	5.5	5.92	6.82	7.26	7.56	7.56	7.23	7.61	7.55	5.03	6.89	3.16	6.89	6.59	9.23
	7.52	7.19	6.93	5.93	5.17	8.54	6.5	5.58	5.92	6.82	7.26	7.56	7.57	7.23	7.61	7.55	5.03	6.89	3.47	6.89	6.59	9.32
	7.52	7.23	7.23	5.93	5.17	8.7	6.51	5.58	5.92	6.94	7.26	7.56	7.69	7.39	7.91	7.56	5.04	6.89	3.8	6.89	6.69	9.49
	7.53	7.23	7.23	5.93	5.17	8.79	6.51	5.67	5.92	6.94	7.35	7.57	7.78	7.49	7.91	7.63	5.04	6.93	3.8	6.9	6.97	9.49
	7.56	7.23	7.29	5.93	5.34	8.83	6.76	5.74	5.92	6.98	7.77	7.97	7.94	7.49	7.91	7.65	5.05	7.01	3.8	7.04	6.98	9.49
	7.56	7.23	7.43	6.23	5.34	8.85	6.76	5.98	5.92	7.3	7.8	8.09	7.94	7.49	7.93	7.66	5.45	7.4	3.81	7.05	6.98	9.49
	7.56	7.23	7.43	6.23	5.38	8.96	6.76	6.05	5.92	7.34	7.8	8.09	7.94	7.49	8.08	7.67	5.68	7.4	3.83	7.05	6.98	9.49
	7.56	7.23	7.63	6.24	5.38	8.96	6.76	6.05	5.92	7.5	7.92	8.1	8.03	7.49	8.09	7.67	5.74	7.4	4.17	7.05	7	9.74
	7.59	7.29	7.72	6.24	5.53	8.96	6.76	6.05	5.95	7.5	7.92	8.1	8.24	7.49	8.09	7.67	5.77	7.4	4.27	7.24	7.34	9.9
	7.62	7.29	7.72	6.24	5.58	9.16	6.82	6.05	6.47	7.5	7.92	8.58	8.24	7.82	8.09	7.67	5.78	7.4	4.27	7.24	7.36	9.93
	7.93	7.29	7.72	6.24	5.62	9.72	6.82	6.06	6.8	7.53	7.93	8.84	8.33	7.82	8.09	7.67	5.78	7.4	4.3	7.24	7.36	10.32
	7.93	7.46	7.72	6.24	5.95	9.82	6.84	6.06	6.94	7.68	8.17	8.84	8.33	7.82	8.3	7.89	5.79	7.4	4.53	7.24	7.36	10.32
	7.93	7.56	7.73	6.24	5.96	9.82	6.9	6.06	6.95	7.68	8.17	8.85	8.33	8.02	8.43	7.89	5.79	7.44	4.54	7.24	7.42	10.32
	7.93	7.56	7.82	6.24	5.96	9.82	6.99	6.37	6.96	7.71	8.18	8.85	8.42	8.1	8.71	7.89	5.79	7.45	4.54	7.37	7.42	10.37
	7.93	7.56	7.82	6.24	5.96	9.92	6.99	6.56	6.98	7.77	8.23	8.85	8.55	8.1	8.71	7.89	5.79	7.45	4.54	7.66	7.42	10.49
	7.93	7.56	7.99	6.24	5.96	10.11	6.99	6.65	6.99	7.77	8.23	8.85	8.55	8.1	8.71	7.89	5.8	7.68	4.55	7.66	7.42	10.49
	8.26	7.56	7.99	6.24	5.96	10.24	7.07	6.74	7.26	7.81	8.24	8.86	8.55	8.35	8.71	7.89	5.8	7.68	4.57	7.66	7.42	10.49
	8.36	7.56	8.1	6.51	5.96	10.31	7.07	6.74	7.45	7.81	8.24	8.96	8.62	8.35	8.97	7.89	5.85	7.68	4.59	7.67	7.43	10.68
	8.36	7.57	8.1	6.51	5.96	10.31	7.07	6.74	7.45	7.93	8.74	8.96	8.74	8.35	8.97	8.23	5.85	7.7	4.64	7.67	7.43	10.77
	8.36	7.57	8.11	6.51	5.96	10.38	7.07	6.74	7.45	7.93	8.79	8.96	8.74	8.35	8.98	8.23	5.94	7.7	4.64	7.67	7.43	10.77
	8.64	7.84	8.11	6.51	5.96	10.38	7.07	6.74	7.45	7.93	8.79	8.97	8.74	8.57	9.17	8.23	6.13	7.7	4.64	7.67	7.43	10.92
	8.67	7.85	8.11	6.77	5.96	10.38	7.07	6.74	7.45	8.06	8.79	9.23	8.9	8.65	9.17	8.42	6.31	7.7	4.64	7.86	7.43	10.92
	9.44	7.85	8.36	6.77	5.96	10.38	7.39	6.74	7.45	8.07	8.8	9.23	8.91	8.79	9.17	8.42	6.31	7.7	4.64	7.96	7.43	10.92
	9.44	7.85	8.36	6.77	5.96	10.38	7.39	6.76	7.92	8.1	8.85	9.23	8.91	8.79	9.44	8.42	6.31	7.7	4.64	7.96	7.43	11.21
	9.44	7.94	8.36	6.78	5.96	10.38	7.39	6.76	7.92	8.1	8.85	9.23	9.14	8.79	9.46	8.42	6.32	7.7	4.64	7.96	7.43	11.21
	9.44	7.97	8.37	6.78	5.96	10.38		6.76	7.92	8.1	8.85	9.24	9.21	8.79	9.46	8.52	6.32	7.7	4.64	7.96	7.68	11.21
	9.44	7.97	8.37	6.78	5.96	10.51		6.89	7.92	8.1	8.86	9.51	9.21	8.79	9.7	8.52	6.43	7.7	4.68	7.96	7.78	11.21
	9.44	7.98	8.37	7	5.96	10.68		6.89	7.92	8.1	8.86	9.61	9.21	8.79	9.7	8.52	6.52	7.7	4.68	7.96	8.05	11.21
	9.44	7.98	8.38	7.01	5.96	10.69		6.98	7.93	8.19	9.03	9.61	9.53	8.84	9.7	8.55	6.53	7.9	4.68	7.96	8.05	11.21
	9.44	7.98	8.38	7.01	5.96	10.69		6.98	8.07	8.24	9.17	9.62	9.58	9.03	9.84	8.55	6.53	7.91	4.68	8.44	8.05	11.21
	9.44	7.98	8.38	7.01	5.96	10.94		7.06	8.16	8.35	9.17	9.62	9.58	9.03	9.84	8.55	6.53	7.91	4.68	8.45	8.05	11.21
	9.44	8.2	8.6	7.01	5.96	10.94		7.06	8.16	8.4	9.18	9.62	9.59	9.03	9.85	8.75	6.53	7.91	4.68	8.45	8.05	11.21
	9.44	8.2	8.71	7.02	5.96	10.94		7.06	8.16	8.4	9.18	9.62	9.59	9.03	9.85	8.81	7	8.14	4.68	8.45	8.18	11.21

9.44	8.2	8.71	7.02	5.96	10.94		7.38	8.16	8.43	9.58	10.07	9.59	9.36	10.14	8.81	7	8.14	4.68	8.46	8.44	11.21
9.44	8.2	8.71	7.02	5.96	10.97		7.38	8.16	8.47	9.58	10.07	9.85	9.43	10.31	8.81	7.01	8.14	4.68	8.46	8.57	11.21
9.44	8.2	8.72	7.36	5.96	11.21		7.45	8.27	8.5	9.58	10.07	9.95	9.43	10.31	8.81	7.01	8.14	4.68	8.46	8.66	11.21
9.44	8.48	8.78	7.36	5.96	11.24		7.66	8.36	8.5	9.58	10.07	10.05	9.43	10.31	9.09	7.21	8.14	4.68	8.46	8.66	11.27
9.58	8.49	8.79	7.36	5.96	11.32		7.66	8.72	8.5	9.8	10.27	10.05	9.43	10.32	9.09	7.22	8.14	4.84	8.64	8.66	11.33
9.6	8.49	8.79	7.36	5.97	11.32		7.66	8.72	8.64	9.8	10.27	10.05	9.43	10.32	9.09	7.22	8.26	4.84	8.64	8.66	11.79
9.6	8.49	8.94	7.62	6.01	11.32		7.66	8.72	8.7	9.81	10.27	10.05	9.43	10.33	9.25	7.22	8.34	4.84	8.65	8.71	11.81
9.6	8.52	8.94	7.63	6.01	11.54		7.66	8.72	9.12	9.81	10.27	10.09	9.5	10.66	9.45	7.23	8.51	5.13	8.65	8.71	11.88
9.6	8.73	9.08	7.64	6.17	11.59		7.66	8.84	9.12	9.82	10.27	10.1	9.5	10.73	9.55	7.24	8.71	5.35	8.65	8.71	12.02
9.7	8.75	9.21	7.97	6.21	11.83		7.78	8.84	9.12	10.11	10.4	10.1	9.79	10.74	9.57	7.46	8.71	5.44	8.65	9.03	12.38
9.77	8.93	9.4	8.06	6.26	12.04		7.95	9.45	9.12	10.12	10.46	10.25	9.86	11.04	9.78	7.51	8.92	5.78	8.77	9.03	12.47
9.77	8.93	9.41	8.06	6.26	12.08		8.07	9.51	9.15	10.13	10.7	10.25	9.86	11.04	9.82	7.86	9.02	5.97	8.77	9.19	12.47
10.25	9.17	9.53	8.07	6.4	12.08		8.49	9.51	9.15	10.25	10.79	10.25	9.86	11.04	9.88	7.86	9.2	5.97	9.07	9.37	12.47
10.25	9.36	9.76	8.07	6.52	12.08		8.66	9.86	9.19	10.44	10.79	10.39	10.22	11.26	10.05	8.14	9.31	6.23	9.07	9.41	12.57
10.25	9.37	9.77	8.07	6.65	12.28		8.66	9.93	9.22	10.44	11.22	10.56	10.24	11.33	10.37	8.14	9.31	6.23	9.08	9.58	12.85
10.25	9.53	10.04	8.34	6.98	12.63		8.66	10.12	9.31	10.45	11.36	10.62	10.37	11.34	10.4	8.38	9.37	6.24	9.3	9.76	13.15
11.09	9.53	10.18	8.4	6.98	12.7		8.66	10.25	9.59	10.63	11.62	10.79	10.37	11.49	10.4	8.39	9.51	6.53	9.31	9.76	13.2
11.09	9.53	10.19	8.4	6.98	12.72		8.92	10.25	9.59	10.85	11.83	10.85	10.44	11.5	10.59	8.39	9.52	6.54	9.31	10.09	13.27
11.09	9.69	10.39	8.69	7.14	12.72		9.23	10.27	9.91	10.94	11.99	10.98	10.44	11.66	10.67	8.4	9.8	6.55	9.48	10.18	13.37
11.09	9.7	10.39	8.83	7.21	13		9.58	10.27	10.1	10.99	12.02	11.02	10.44	11.66	10.78	8.83	9.94	6.61	9.61	10.34	13.37
11.1	9.83	10.39	8.84	7.26	13.24		9.58	10.27	10.1	10.99	12.02	11.15	10.81	11.93	10.86	9.02	10.17	6.93	9.72	10.35	13.38
11.44	9.92	10.6	8.84	7.26	13.48		9.58	10.27	10.1	11.18	12.02	11.28	10.99	11.93	10.93	9.02	10.34	7.03	9.72	10.54	13.55
11.44	10.17	10.72	8.84	7.26	13.67		9.58	10.43	10.14	11.18	12.02	11.28	10.99	12.17	10.97	9.03	10.39	7.23	10.07	10.6	13.67
11.48	10.17	10.73	9.2	7.26	13.72		9.94	10.69	10.14	11.18	12.16	11.29	10.99	12.23	11.19	9.03	10.48	7.51	10.11	10.6	14.22
11.7	10.46	10.86	9.35	7.26	13.72		10.2	10.84	10.26	11.18	12.17	11.5	10.99	12.23	11.37	9.03	10.48	7.69	10.15	10.71	14.77
12	10.51	10.86	9.36	7.45	13.72		10.2	10.88	10.42	11.33	12.2	11.5	11	12.23	11.43	9.03	10.48	7.81	10.18	10.71	14.78
12.07	10.62	10.86	9.36	7.49	13.72		10.2	10.88	10.42	11.34	12.29	11.74	11.31	12.23	11.47	9.14	10.51	7.91	10.66	10.89	15.03
12.07	10.65	11.07	9.36	7.52	13.77		10.2	10.88	10.76	11.34	12.32	11.87	11.31	12.23	11.58	9.49	10.67	7.92	10.8	10.9	15.08
12.22	10.65	11.08	9.36	7.52	13.77		10.78	10.88	10.87	11.35	12.74	11.87	11.34	12.36	11.62	9.62	10.84	8.08	10.8	11.16	15.17
12.22	10.65	11.08	9.39	7.52	13.77		10.78	10.88	10.93	11.56	12.81	11.87	11.34	12.36	11.63	10.07	10.84	8.1	10.8	11.26	15.3
12.85	10.71	11.08	9.39	7.53	13.77		10.78	11.03	10.93	11.77	12.82	11.87	11.35	12.37	11.63	10.07	10.84	8.11	10.8	11.29	15.38
12.85	10.85	11.08	9.4	7.53	13.78		10.78	11.15	10.93	11.8	12.96	12.17	11.37	12.37	11.63	10.17	10.84	8.12	10.8	11.29	15.77
12.85	11	11.08	9.4	7.71	13.78		10.78	11.15	10.93	11.8	12.96	12.21	11.41	12.38	11.71	10.28	10.95	8.22	10.8	11.29	15.89
12.85	11.08	11.27	9.4	7.71	13.78		10.78	11.27	10.93	12.14	12.96	12.21	11.41	12.38	11.96	10.28	10.95	8.33	10.8	11.29	15.89
12.85	11.09	11.28	9.4	7.73	13.9		10.78	11.28	10.93	12.26	12.96	12.27	11.61	12.71	12.03	10.29	10.97	8.35	10.8	11.29	15.89
12.85	11.28	11.28	9.78	7.81	13.9		10.82	11.28	11.03	12.26	12.96	12.27	11.61	12.81	12.03	10.29	11.18	8.56	10.85	11.29	15.91
12.85	11.39	11.51	9.79	7.81	13.9		11.04	11.28	11.05	12.27	12.97	12.6	11.61	12.82	12.03	10.29	11.2	8.69	10.86	11.4	15.93
12.85	11.39	11.51	9.79	7.81	14.21		11.07	11.28	11.08	12.27	12.97	12.68	11.61	12.82	12.03	10.29	11.2	8.74	10.86	11.42	15.93
12.85	11.39	11.51	9.79	7.81	14.49		11.07	11.28	11.15	12.28	12.97	12.68	11.61	13.01	12.22	10.3	11.21	8.92	10.86	11.42	15.93
12.85	11.42	11.51	9.93	7.9	14.84		11.07	11.53	11.29	12.28	13.03	12.73	11.64	13.1	12.48	10.3	11.21	8.94	10.86	11.42	15.93
12.99	11.73	11.52	9.97	8.22	14.91		11.07	11.72	11.44	12.66	13.03	12.73	11.98	13.1	12.51	10.3	11.22	8.97	10.86	11.74	15.93
13.04	11.73	11.72	9.97	8.36	14.91		11.12	11.72	11.44	12.79	13.04	12.73	11.98	13.11	12.52	10.31	11.37	8.97	11.26	12.04	16.25
13.04	11.73	11.72	9.97	8.4	14.91		11.16	11.72	11.45	12.79	13.04	12.89	11.98	13.13	12.53	10.31	11.56	8.97	11.38	12.15	16.25
13.04	11.73	11.72	9.97	8.4	14.91		11.16	12.01	11.52	12.8	13.36	12.96	12.07	13.52	12.54	10.86	11.56	8.97	11.38	12.15	16.28
13.04	11.74	11.85	10.07	8.4	14.96		11.33	12.04	11.52	12.8	13.37	12.96	12.36	13.52	12.64	11.02	11.57	9.26	11.38	12.15	16.35
13.04	11.87	12.05	10.38	8.44	15.05		11.48	12.04	11.62	12.8	13.37	12.97	12.43	13.52	12.64	11.02	11.57	9.28	11.38	12.15	16.57
13.04	11.87	12.05	10.38	8.68	15.34		11.48	12.04	11.62	12.82	13.37	12.97	12.43	13.53	12.65	11.03	11.58	9.28	11.6	12.35	16.75
13.04	11.96	12.06	10.38	8.8	15.48		11.48	12.05	11.62	12.91	13.38	12.97	12.43	13.53	12.65	11.03	11.58	9.28	11.61	12.39	16.75
13.09	11.96	12.06	10.38	8.94	15.51		11.48	12.44	11.82	12.91	13.57	13.19	12.46	13.78	12.81	11.03	11.59	9.28	11.61	12.39	16.82
13.09	11.96	12.06	10.38	8.94	15.51		11.48	12.55	11.82	12.91	13.58	13.19	12.66	13.88	12.84	11.04	11.59	9.4	11.61	12.39	16.82
13.17	11.96	12.07	10.39	8.94	15.51		11.48	12.55	11.82	12.92	13.58	13.19	12.66	13.88	12.84	11.04	11.59	9.41	11.61	12.55	16.85
13.27	12	12.07	10.39	8.94	15.51		11.48	12.55	11.82	13.05	13.58	13.19	12.68	13.89	12.86	11.04	11.76	9.42	11.61	12.64	16.85
13.27	12	12.18	10.39	8.95	15.51		11.48	12.61	11.82	13.05	13.76	13.25	12.71	13.89	12.92	11.04	11.93	9.42	11.83	12.7	16.85
13.27	12	12.28	10.39	8.95	15.71		11.48	12.72	11.82	13.06	13.76	13.43	12.71	13.89	12.93	11.05	11.93	9.42	11.83	12.81	17.21
13.27	12.01	12.29	10.39	8.95	15.84		11.63	12.72	11.86	13.06	13.77	13.43	12.71	14.04	12.96	11.38	11.93	9.43	**11.83**	**12.84**	**17.36**

0	0	0	0	0	0	0	0	0	0	0	0	0	0	0	0	0.16	0	0	0	0	0
0.29	0	0	0.01	0	0	0	0	0.28	0	0.17	0.28	0.01	0.17	0.01	0	0.27	0	0	0	0.1	0.27
0.44	0.15	0	0.29	0	0	0	0.18	0.28	0	0.44	0.32	0.18	0.2	0.14	0	0.39	0	0	0.05	0.1	0.27
0.47	0.33	0.17	0.3	0	0.19	0.29	0.21	0.28	0	0.48	0.32	0.18	0.21	0.14	0	0.39	0	0.15	0.06	0.1	0.54
0.47	0.33	0.17	0.39	0.01	0.19	0.3	0.21	0.51	0	0.48	0.38	0.25	0.21	0.15	0	0.4	0.18	0.3	0.06	0.12	0.6
0.52	0.34	0.18	0.45	0.01	0.19	0.3	0.42	0.51	0	0.48	0.38	0.34	0.22	0.16	0	0.41	0.3	0.37	0.17	0.17	0.73
0.7	0.61	0.32	0.46	0.01	0.26	0.46	0.42	0.51	0	0.68	0.38	0.34	0.33	0.16	0	0.41	0.31	0.38	0.17	0.21	0.75
0.71	0.61	0.32	0.46	0.04	0.34	0.46	0.42	0.51	0	0.68	0.38	0.34	0.33	0.16	0	0.42	0.46	0.38	0.17	0.29	0.81
0.71	0.67	0.32	0.46	0.06	0.34	0.46	0.72	0.51	0	0.68	0.44	0.38	0.34	0.29	0	0.42	0.55	0.38	0.25	0.29	0.81
0.77	0.67	0.33	0.47	0.11	0.41	0.54	0.72	0.63	0	0.82	0.44	0.46	0.35	0.29	0	0.47	0.58	0.4	0.26	0.37	1.03
0.87	0.68	0.54	0.59	0.21	0.41	0.54	1.11	0.63	0	0.82	0.44	0.46	0.42	0.3	0	0.47	0.65	0.41	0.34	0.47	1.03
0.94	0.68	0.54	0.59	0.21	0.52	0.55	1.11	0.63	0.79	0.82	0.44	0.56	0.46	0.3	0.9	0.48	0.76	0.41	0.34	0.47	1.03
0.98	0.68	0.55	0.69	0.21	0.75	0.55	1.11	0.63	0.79	0.86	0.44	0.57	0.46	0.31	0.97	0.57	0.78	0.43	0.34	0.47	1.13
0.98	0.88	0.55	0.69	0.31	0.83	0.6	1.11	0.71	0.79	0.89	0.51	0.57	0.46	0.32	1.08	0.58	0.8	0.43	0.49	0.47	1.13
0.98	0.93	0.55	0.69	0.36	0.83	0.66	1.11	0.78	0.81	0.91	0.56	0.78	0.54	0.41	1.08	0.58	0.85	0.43	0.5	0.51	1.13
0.98	0.93	0.64	0.85	0.36	0.88	0.67	1.11	0.86	0.81	0.91	0.7	0.78	0.54	0.41	1.42	0.59	0.88	0.43	0.5	0.55	1.21
0.99	0.93	0.65	0.97	0.36	1.08	0.68	1.29	0.9	0.81	1.02	0.74	0.78	0.54	0.41	1.42	0.59	0.97	0.57	0.59	0.7	1.21
1.22	1	0.65	0.98	0.36	1.1	0.68	1.3	0.9	0.96	1.03	0.95	0.79	0.56	0.57	1.42	0.59	1.16	0.62	0.69	0.73	1.27
1.3	1.17	0.84	0.98	0.36	1.1	0.79	1.3	0.95	0.97	1.04	1	0.91	0.56	0.57	1.48	0.6	1.22	0.68	0.73	0.76	1.35
1.44	1.17	0.97	1.15	0.36	1.1	0.93	1.34	0.95	0.97	1.13	1	1.09	0.72	0.57	1.48	1.05	1.22	0.76	0.75	0.83	1.35
1.45	1.18	0.99	1.15	0.39	1.24	0.96	1.34	0.95	0.97	1.2	1	1.14	0.72	0.74	1.49	1.07	1.35	0.76	0.92	0.86	1.46
1.47	1.18	1	1.16	0.43	1.25	1.07	1.51	0.95	1.01	1.29	1.26	1.2	0.8	0.78	1.62	1.18	1.37	0.76	1.01	1.12	1.46
1.47	1.18	1	1.16	0.51	1.27	1.1	1.51	0.95	1.05	1.31	1.33	1.26	0.8	0.83	1.63	1.42	1.56	0.83	1.01	1.14	1.51
1.47	1.25	1	1.33	0.62	1.55	1.1	1.7	1.05	1.05	1.31	1.33	1.4	0.85	1	1.63	1.51	1.58	0.96	1.01	1.26	1.72
1.47	1.26	1.1	1.39	0.71	1.68	1.31	1.81	1.23	1.05	1.34	1.33	1.45	0.91	1.02	1.63	1.55	1.59	0.96	1.01	1.36	1.9
1.53	1.45	1.18	1.39	0.85	1.94	1.33	1.99	1.26	1.11	1.34	1.33	1.45	0.98	1.09	1.63	1.56	1.74	0.96	1.15	1.4	1.9
1.53	1.56	1.24	1.4	0.85	2.03	1.33	1.99	1.26	1.27	1.45	1.43	1.46	1.05	1.09	1.76	1.56	1.82	0.96	1.16	1.57	1.9
1.84	1.58	1.24	1.45	0.85	2.14	1.33	1.99	1.26	1.28	1.46	1.52	1.46	1.11	1.09	1.92	1.56	1.85	1.04	1.16	1.6	2.02
1.85	1.59	1.3	1.62	0.85	2.14	1.61	1.99	1.26	1.39	1.48	1.52	1.6	1.21	1.09	1.96	1.57	1.88	1.21	1.23	1.67	2.44
1.97	1.68	1.3	1.63	0.85	2.19	1.62	1.99	1.26	1.39	1.48	1.52	1.7	1.21	1.19	2.09	1.57	2	1.36	1.32	1.67	2.48
2.07	1.79	1.3	1.68	0.85	2.58	1.62	2.12	1.3	1.39	1.51	1.71	1.7	1.37	1.32	2.09	1.57	2.07	1.46	1.36	1.69	2.58
2.21	1.79	1.3	1.68	0.85	2.58	1.68	2.12	1.6	1.39	1.51	1.71	1.71	1.37	1.33	2.21	1.64	2.08	1.54	1.52	1.71	2.86
2.36	1.8	1.3	1.69	0.85	2.58	1.68	2.12	1.61	1.5	1.51	1.76	1.71	1.52	1.33	2.21	1.65	2.08	1.58	1.63	2.05	2.9
2.41	1.98	1.3	1.69	1.07	2.58	1.68	2.3	1.61	1.7	1.64	1.79	1.97	1.52	1.34	2.27	1.65	2.13	1.58	1.8	2.22	2.9
2.41	2.03	1.31	1.9	1.28	2.68	1.9	2.3	1.61	1.75	1.85	1.8	2.04	1.52	1.65	2.47	1.65	2.14	1.58	1.8	2.39	2.9
2.41	2.04	1.62	2.07	1.28	2.68	1.9	2.3	1.61	1.78	1.89	1.9	2.04	1.65	1.66	2.61	1.65	2.19	1.97	1.97	2.49	2.9
2.47	2.04	1.63	2.08	1.28	2.68	1.9	2.3	1.61	1.78	2.02	1.92	2.04	1.65	1.66	2.61	1.98	2.19	1.97	1.97	2.49	3.14
2.54	2.15	1.63	2.09	1.28	2.68	1.9	2.6	1.61	1.78	2.17	1.92	2.05	1.65	1.66	2.61	2.07	2.34	1.97	1.98	2.49	3.15
2.66	2.16	1.71	2.09	1.28	2.86	1.91	2.67	1.79	1.78	2.17	1.99	2.3	1.65	1.66	2.61	2.07	2.34	1.98	1.98	2.56	3.29
2.72	2.16	1.71	2.09	1.28	3.14	1.91	2.67	1.8	2.06	2.18	1.99	2.31	1.65	1.67	2.88	2.07	2.35	1.98	2.07	2.58	3.45
2.74	2.16	1.72	2.09	1.28	3.14	1.91	2.67	1.8	2.07	2.32	2.04	2.31	1.65	1.67	3.02	2.08	2.36	1.98	2.07	2.65	3.45
2.79	2.16	1.82	2.28	1.39	3.14	2.13	2.67	1.8	2.08	2.4	2.04	2.31	1.65	1.75	3.02	2.08	2.36	2.06	2.34	2.73	3.82
2.79	2.16	1.82	2.28	1.39	3.14	2.13	2.75	1.8	2.2	2.42	2.04	2.64	1.65	1.76	3.02	2.08	2.36	2.07	2.34	2.74	3.82
2.79	2.17	1.82	2.28	1.39	3.14	2.13	2.88	1.8	2.27	2.42	2.13	2.71	1.65	1.81	3.02	2.08	2.36	2.1	2.34	2.74	3.94
2.91	2.37	1.83	2.28	1.39	3.14	2.14	2.88	2.14	2.27	2.42	2.28	2.78	1.7	1.98	3.02	2.08	2.43	2.11	2.34	2.78	4.13
3.12	2.52	1.83	2.28	1.5	3.18	2.14	2.88	2.14	2.3	2.46	2.28	2.78	1.7	1.98	3.34	2.41	2.52	2.2	2.35	2.91	4.28
3.12	2.52	1.83	2.28	1.58	3.53	2.14	2.99	2.14	2.41	2.49	2.28	2.78	1.72	1.98	3.43	2.52	2.52	2.24	2.35	2.98	4.28
3.39	2.52	1.83	2.28	1.58	3.65	2.3	2.99	2.14	2.43	2.83	2.28	2.89	1.86	1.99	3.43	2.52	2.53	2.3	2.35	3.16	4.28
3.42	2.8	2.02	2.59	1.58	3.65	2.42	3.25	2.25	2.49	2.83	2.38	2.9	1.88	2.18	3.43	2.52	2.73	2.39	2.35	3.25	4.28
3.45	2.8	2.02	2.6	1.58	3.65	2.43	3.25	2.35	2.52	2.84	2.51	2.9	2.08	2.19	3.43	2.53	2.74	2.41	2.61	3.25	4.36
3.62	2.8	2.18	2.6	1.58	3.65	2.43	3.25	2.35	2.63	2.84	2.59	3.08	2.08	2.19	3.43	2.55	2.75	2.47	2.7	3.34	4.59
3.65	3.04	2.19	2.6	1.7	3.71	2.61	3.31	2.35	2.69	2.86	2.61	3.21	2.14	2.19	3.43	2.67	2.75	2.48	2.7	3.34	4.67
3.69	3.11	2.19	2.6	1.7	3.71	2.62	3.31	2.74	2.69	2.87	2.63	3.21	2.14	2.19	3.49	2.68	2.76	2.75	2.7	3.58	4.67
3.79	3.11	2.32	2.69	1.76	4.14	2.63	3.31	2.74	2.71	2.93	2.63	3.21	2.35	2.31	3.5	2.68	2.87	2.76	2.7	3.6	4.67
3.79	3.11	2.32	2.8	1.76	4.21	2.63	3.31	2.74	2.71	2.95	2.83	3.21	2.36	2.32	3.5	2.97	2.88	2.77	2.98	3.71	4.67
3.84	3.12	2.33	2.81	1.82	4.21	2.63	3.42	2.74	2.71	2.95	2.97	3.28	2.36	2.32	3.58	2.98	2.88	2.77	2.98	3.78	4.67
3.84	3.12	2.61	2.97	1.82	4.21	2.7	3.42	2.74	2.71	2.97	3.04	3.28	2.44	2.32	3.58	2.98	3.12	2.77	2.98	3.82	4.67
3.89	3.28	2.61	2.98	1.82	4.47	2.79	3.42	2.74	2.72	2.97	3.04	3.52	2.52	2.33	3.58	2.98	3.13	2.78	2.98	3.82	4.67
3.95	3.28	2.62	2.98	1.93	4.51	2.79	3.42	2.74	2.74	3.18	3.14	3.52	2.52	2.34	3.69	2.99	3.13	2.78	3.15	3.93	4.67
3.95	3.28	2.62	2.98	1.95	4.55	2.8	3.42	3.04	2.78	3.3	3.21	3.53	2.52	2.34	3.69	2.99	3.13	2.78	3.15	3.94	4.78
4.22	3.28	2.84	2.98	1.96	4.55	2.8	3.47	3.06	2.82	3.3	3.37	3.53	2.56	2.53	3.69	2.99	3.14	2.79	3.2	3.94	4.78
4.39	3.29	2.91	2.98	1.96	4.69	2.8	3.47	3.06	2.82	3.3	3.37	3.53	2.56	2.53	3.69	2.99	3.14	2.79	3.2	4.05	4.79
4.39	3.29	2.91	2.98	2.21	4.69	2.81	3.5	3.06	2.89	3.32	3.37	3.72	2.56	2.53	3.69	3.32	3.24	2.8	3.27	4.13	4.86
4.39	3.29	2.92	2.99	2.36	5	2.81	3.5	3.06	3	3.33	3.37	3.81	2.66	2.54	3.69	3.33	3.3	2.8	3.27	4.14	4.86
4.39	3.44	2.92	3.17	2.38	5.11	2.81	3.5	3.06	3.01	3.54	3.37	3.81	2.79	2.58	3.7	3.33	3.31	2.88	3.27	4.15	5.49
4.39	3.44	3.06	3.17	2.48	5.11	2.83	3.5	3.06	3.09	3.55	3.53	3.82	2.79	2.59	3.82	3.33	3.31	2.89	3.28	4.15	5.82
4.39	3.44	3.06	3.17	2.49	5.12	2.83	4.16	3.06	3.09	3.55	3.65	3.82	2.83	2.6	3.82	3.33	3.5	2.89	3.59	4.15	5.82
4.52	3.65	3.06	3.18	2.49	5.15	2.84	4.2	3.2	3.09	3.56	3.65	3.94	2.97	2.82	3.82	3.33	3.5	2.89	3.59	4.36	5.82
4.52	3.65	3.07	3.18	2.49	5.15	2.95	4.2	3.2	3.15	3.92	3.65	3.94	2.97	2.9	3.85	3.34	3.5	2.9	3.59	4.44	5.82
4.52	3.65	3.07	3.18	2.5	5.15	2.96	4.2	3.2	3.19	3.94	3.65	3.98	2.97	2.9	4.06	3.34	3.5	3.05	3.59	4.51	5.82
4.61	3.66	3.15	3.43	2.5	5.6	2.96	4.2	3.2	3.19	3.95	3.9	4.13	2.97	2.9	4.15	3.76	3.63	3.19	3.6	4.54	5.82
4.81	3.86	3.16	3.43	2.5	5.6	3.12	4.2	3.2	3.2	3.95	3.97	4.22	3.02	2.9	4.15	3.81	3.64	3.19	3.6	4.62	5.82
4.81	3.86	3.16	3.43	2.59	5.62	3.12	4.2	3.3	3.2	3.95	3.97	4.23	3.12	2.91	4.15	3.82	3.64	3.19	3.6	4.72	5.82
4.81	3.87	3.17	3.44	2.7	5.62	3.12	4.2	3.3	3.3	4.16	3.99	4.23	3.14	3.07	4.16	3.82	3.77	3.19	3.6	4.74	5.82
4.92	3.87	3.31	3.57	2.73	5.62	3.13	4.2	3.3	3.3	4.28	3.99	4.23	3.14	3.15	4.23	4.02	3.77	3.33	3.61	4.91	5.99
4.94	4.08	3.31	3.57	2.73	5.75	3.26	4.2	3.3	3.36	4.29	4.11	4.23	3.14	3.16	4.23	4.02	3.78	3.34	3.91	4.91	5.99

5.12	4.08	3.32	3.57	2.73	5.94	3.26	4.3	3.3	3.36	4.3	4.11	4.24	3.14	3.16	4.33	4.02	3.79	3.34	3.91	4.95	5.99
5.12	4.08	3.32	3.57	2.77	5.96	3.27	4.46	3.41	3.41	4.3	4.13	4.46	3.21	3.17	4.39	4.03	3.79	3.34	3.92	4.95	6.22
5.16	4.12	3.35	3.74	2.8	5.99	3.33	4.88	3.43	3.46	4.38	4.19	4.58	3.22	3.17	4.46	4.03	3.79	3.39	3.98	4.98	6.22
5.16	4.13	3.36	3.86	2.82	6.07	3.35	4.88	3.43	3.46	4.45	4.34	4.65	3.34	3.17	4.64	4.03	3.86	3.48	3.99	5.1	6.66
5.3	4.14	3.36	3.89	2.82	6.12	3.35	4.88	3.45	3.46	4.46	4.41	4.7	3.34	3.17	4.8	4.03	3.88	3.51	4.17	5.25	6.66
5.43	4.32	3.46	3.89	2.82	6.22	3.42	4.88	3.45	3.46	4.5	4.44	4.8	3.34	3.29	4.97	4.23	3.88	3.52	4.21	5.39	6.73
5.68	4.33	3.46	4.17	2.82	6.29	3.48	4.88	3.69	3.46	4.5	4.46	4.85	3.34	3.36	5.05	4.41	3.89	3.78	4.21	5.6	6.82
5.73	4.33	3.46	4.26	2.82	6.29	3.8	4.99	3.78	3.46	4.5	4.69	4.93	3.34	3.36	5.08	4.44	3.89	3.78	4.36	5.81	6.84
5.73	4.34	3.53	4.36	2.82	6.29	3.99	5.11	3.78	3.46	4.5	4.78	4.93	3.34	3.37	5.18	4.56	4.16	3.82	4.42	5.82	6.84
5.92	4.45	3.53	4.44	2.82	6.54	4.13	5.15	3.78	3.66	4.7	4.82	4.97	3.37	3.37	5.18	4.95	4.18	4	4.48	5.87	6.93
6.11	4.45	3.61	4.71	2.89	6.89	4.13	5.16	3.83	3.67	4.75	4.92	5.07	3.79	3.38	5.39	5.04	4.18	4.05	4.55	5.95	6.96
6.3	4.68	3.66	4.72	3.17	6.89	4.14	5.16	3.83	3.67	4.75	4.95	5.31	3.79	3.67	5.39	5.12	4.18	4.06	4.56	5.97	6.96
6.49	4.68	3.66	4.72	3.17	6.89	4.2	5.58	3.91	3.77	5.01	4.95	5.39	3.8	3.68	5.46	5.12	4.33	4.06	4.73	6.24	6.96
6.6	4.68	3.66	4.72	3.2	6.89	4.21	5.58	3.98	3.77	5.22	4.95	5.4	3.81	3.68	5.56	5.12	4.34	4.06	4.73	6.33	7.27
6.75	4.75	3.81	4.96	3.2	6.89	4.21	5.63	3.99	3.77	5.34	4.98	5.4	3.82	3.68	5.58	5.12	4.38	4.22	5.04	6.53	7.68
6.79	4.76	3.81	5.02	3.2	6.89	4.22	5.63	4.01	3.93	5.34	4.98	5.4	3.83	3.75	5.58	5.12	4.45	4.31	5.05	6.63	7.68
6.99	4.77	3.87	5.1	3.2	6.89	4.22	5.72	4.04	3.96	5.34	5.17	5.41	3.84	3.75	5.58	5.12	4.64	4.34	5.13	6.72	7.68
7.04	5.05	3.98	5.1	3.2	7.08	4.56	5.76	4.07	3.97	5.34	5.17	5.77	4.05	3.75	5.75	5.18	4.66	4.34	5.21	6.82	7.74
7.04	5.12	3.99	5.11	3.2	7.08	4.57	5.95	4.4	4.07	5.44	5.31	5.82	4.11	3.76	5.82	5.38	4.67	4.35	5.21	7.05	8.05
7.21	5.15	3.99	5.11	3.32	7.29	4.57	5.95	4.63	4.12	5.46	5.32	6 .		3.98	5.82	5.5	4.67	4.47	5.32	7.08	8.06
7.24	5.15	4.06	5.11	3.32	7.57	4.57	5.95	4.63	4.13	5.53	5.32	6.09 .		4	6.06	5.59	4.67	4.56	5.55	7.19	8.06
7.24	5.15	4.21	5.11	3.32	7.76	4.66	5.95	4.64	4.3	5.55	5.32	6.12 .		4.01	6.18	5.7	4.67	4.62	5.55	7.2	8.06
7.38	5.15	4.22	5.39	3.32	7.76	4.87	5.95	4.67	4.31	5.55	5.33	6.12 .		4.04	6.18	5.87	4.67	4.7	5.56	7.3	8.22
7.46	5.28	4.22	5.39	3.45	7.82	4.87	6.53	4.78	4.31	5.55	5.33	6.12 .		4.04	6.28	6.01	4.71	4.79	5.57	7.3	8.3
7.46	5.29	4.22	5.39	**3.45**	**7.82**	4.88	6.53	4.78	4.4	5.55	**5.35**	**6.12** .		4.04	6.28	6.07	4.85	5.02	5.6	7.3	8.52
7.46	5.29	4.22	5.39	**3.45**	**7.82**	4.88	6.53	4.78	4.62	5.73	**5.44**	**6.12** .		4.04	6.33	6.07	4.93	5.05	5.6	7.32	8.53
7.55	5.29	4.23	5.59	**3.45**	**7.83**	4.89	6.53	4.78	4.63	5.73	**5.45**	**6.27** .		4.05	6.33	6.08	4.94	5.07	5.79	7.32	8.53
7.61	5.29	4.23	5.68	**3.45**	**7.83**	4.89	6.53	4.81	4.64	5.88	**5.45**	**6.55** .		4.05	6.33	6.08	4.94	5.42	5.8	7.34	8.53
7.96	5.29	4.68	5.69	**3.45**	**7.83**	4.9	6.53	4.85	4.84	5.94	**5.46**	**6.56** .		4.26	6.49	6.36	5.02	5.43	5.8	7.43	8.53
7.96	5.46	4.69	5.69	**3.45**	**7.83**	4.92	6.53	4.85	4.85	5.99	**5.56**	**6.56** .		4.35	6.53	6.46	5.12	5.43	5.82	7.43	8.53
8.05	5.61	4.69	5.69	**3.45**	**8.1**	4.92	6.87	4.85	4.88	6.02	**5.6**	**6.56** .		4.35	6.56	6.46	5.12	5.43	5.96	7.51	8.53
8.05	5.64	4.69	5.69	**3.46**	**8.33**	4.92	6.87	4.9	4.91	6.08	**5.67**	**6.56** .		4.35	6.57	6.47	5.19	5.46	6.02	7.6	8.53
8.05	5.64	4.69	6.15	3.55	8.33	4.92	6.87	4.93	4.93	6.08	5.9	6.65 .		4.36	6.57	6.47	5.2	5.59	6.07	7.6	8.53
8.05	5.65	4.69	6.15	3.67	8.33	4.94	6.99	4.93	4.99	6.43	5.98	6.93 .		4.37	6.58	6.47	5.25	5.75	6.07	7.61	8.65
8.09	5.96	4.7	6.15	3.8	8.49	4.94	6.99	5.23	4.99	6.45	6.03	6.94 .		4.48	6.96	6.59	5.25	5.77	6.23	7.65	8.95
8.3	6.09	4.7	6.16	3.8	8.49	4.94	6.99	5.31	4.99	6.45	6.03	6.94 .		4.66	6.96	6.59	5.25	5.77	6.26	7.69	9.03
8.3	6.09	4.7	6.16	3.8	8.49	5.07	6.99	5.31	4.99	6.45	6.03	6.94 .		4.83	6.96	6.6	5.25	5.77	6.26	7.69	9.07
8.3	6.18	4.7	6.16	3.8	8.5	5.17	6.99	5.32	5	6.45	6.2	6.94 .		4.83	6.96	6.6	5.25	5.77	6.44	7.82	9.11
8.55	6.22	4.88	6.16	3.8	8.75	5.18	6.99	5.32	5.18	6.62	6.38	6.94 .		4.83	7.18	7.01	5.26	5.77	6.44	7.85	9.11
8.6	6.23	4.88	6.17	3.83	8.82	5.18	6.99	5.33	5.19	6.63	6.38	7.15 .		4.83	7.18	7.03	5.54	5.77	6.44	7.85	9.11
8.74	6.23	4.89	6.17	3.83	8.82	5.19	6.99	5.33	5.19	6.65	6.38	7.15 .		4.84	7.18	7.03	5.57	5.77	6.6	7.96	9.3
8.81	6.26	4.89	6.26	3.89	8.82	5.32	6.99	5.37	5.19	6.76	6.38	7.16 .		4.84	7.18	7.03	5.66	5.77	6.6	8.09	9.37
8.97	6.26	4.89	6.36	3.89	8.82	5.36	6.99	5.37	5.21	6.76	6.39	7.16 .		4.84	7.18	7.03	5.66	5.78	6.77	8.1	9.44
9.13	6.26	4.9	6.36	3.9	8.84	5.36	6.99	5.37	5.33	6.76	6.39	7.16 .		4.93	7.25	7.07	5.67	5.78	6.77	8.11	9.44
9.19	6.38	4.96	6.36	3.92	8.84	5.37	6.99	5.37	5.33	6.92	6.4	7.42 .		5.1	7.25	7.07	5.75	5.95	6.77	8.11	9.44
9.19	6.39	4.96	6.36	4.07	8.84	5.37	7.36	5.37	5.33	6.99	6.4	7.56 .		5.11	7.25	7.18	5.76	6	6.77	8.46	9.44
9.24	6.39	4.97	6.45	4.09	9.01	5.49	7.36	5.5	5.34	6.99	6.63	7.56 .		5.11	7.52	7.18	5.76	6.01	6.9	8.52	9.44
9.3	6.39	4.97	6.45	4.09	9.39	5.5	7.36	5.89	5.36	6.99	6.63	7.57 .		5.12	7.52	7.18	5.76	6.01	6.9	8.62	9.53
9.3	6.44	5.22	6.45	4.09	9.39	5.51	7.36	5.95	5.64	7.02	6.63	7.57 .		5.12	7.52	7.19	5.77	6.01	7.04	8.71	9.53
9.3	6.44	5.22	6.45	4.09	9.43	5.56	7.54	5.95	5.65	7.1	6.63	7.57 .		5.13	7.52	7.33	5.77	6.02	7.04	8.71	9.53
9.3	6.45	5.23	6.46	4.2	9.43	5.56	7.54	5.96	5.65	7.28	6.63	7.57 .		5.13	7.52	7.34	5.77	6.02	7.12	8.71	9.54
9.35	6.53	5.23	6.46	4.2	9.43	5.56	7.54	5.96	5.65	7.28	6.63	7.57 .		5.13	7.52	7.34	5.88	6.02	7.12	8.71	9.54
9.4	6.54	5.23	6.46	4.38	9.43	5.57	7.71	5.96	5.66	7.28	6.63	7.58 .		5.14	7.58	7.44	5.88	6.12	7.13	8.79	9.54
9.43	6.54	5.24	6.46	4.38	9.43	5.59	7.71	5.96	5.67	7.28	6.64	7.58 .		5.48	7.58	7.64	5.89	6.12	7.14	8.83	9.54
9.49	6.54	5.54	6.46	4.38	9.43		7.71	5.96	5.85	7.39	6.66	7.59 .		5.56	7.59	7.64	5.89	6.12	7.14	8.85	9.54
9.49	6.55	5.54	6.47	4.38	9.72		7.71	5.96	5.88	7.4	6.85	7.93 .		5.56	7.59	7.65	5.89	6.12	7.14	9.06	9.54
9.51	6.83	5.54	6.64	4.38	9.72		7.71	5.96	5.88	7.4	6.85	7.93 .		5.57	7.66	7.65	5.89	6.18	7.14	9.08	9.54
9.56	6.88	5.54	6.64	4.38	9.76		7.71	5.99	5.88	7.42	6.85	7.93 .		5.58	7.66	7.65	6.03	6.32	7.34	9.14	9.54
9.56	6.89	5.55	6.65	4.38	9.76		7.71	5.99	5.88	7.75	6.85	7.94 .		5.58	7.66	7.65	6.11	6.37	7.49	9.17	9.54
10	6.89	5.55	6.65	4.38	9.8		7.71	6.02	5.91	7.78	6.85	7.94 .		5.59	7.66	7.66	6.12	6.37	7.56	9.27	9.54
10.18	6.98	5.56	6.65	4.4	9.8		7.78	6.02	5.91	7.78	7.03	7.94 .		5.59	7.66	7.66	6.12	6.38	7.56	9.31	9.54
10.37	6.98	5.8	6.78	4.45	10.06		7.78	6.03	5.91	7.78	7.03	7.95 .		5.81	7.66	7.76	6.12	6.38	7.57	9.32	9.54
10.37	6.99	5.8	6.78	4.45	10.12		7.78	6.04	5.92	7.78	7.21	7.95 .		5.81	7.86	7.76	6.26	6.38	7.57	9.47	9.58
10.42	7.11	5.81	6.78	4.45	10.13		7.78	6.04	5.93	7.91	7.28	7.95 .		5.82	7.86	7.76	6.27	6.58	7.57	9.53	9.58
10.44	7.15	5.93	6.79	4.6	10.38		7.78	6.19	5.93	7.93	7.38	8.09 .		5.82	7.86	7.94	6.27	6.6	7.67	9.54	9.69
10.44	7.15	5.93	6.88	4.6	10.41		7.78	6.49	5.93	7.93	7.51	8.1 .		5.83	7.99	8	6.28	6.65	7.67	9.58	9.75
10.44	7.22	5.94	6.88	4.6	10.44		7.78	6.49	5.99	7.94	7.52	8.15	5.57	5.84	7.99	8	6.29	6.66	7.67	9.58	9.75
10.51	7.22	5.94	6.89	4.79	10.44		7.78	6.49	6	8	7.52	8.15	5.67	5.85	8.01	8	6.29	6.74	7.67	9.58	9.76
10.54	7.22	5.99	7.03	4.79	10.52		8.01	6.56	6.01	8.08	7.74	8.15	5.75	5.85	8.06	8	6.29	6.8	7.74	9.61	9.79
10.64	7.23	5.99	7.05	4.8	10.54		8.01	6.6	6.02	8.14	7.97	8.16	5.77	5.86	8.06	8.01	6.29	6.91	7.75	9.77	10.14
10.65	7.23	5.99	7.13	4.8	10.76		8.01	6.7	6.03	8.28	8.02	8.3	5.77	5.86	8.06	8.02	6.41	6.97	7.75	9.78	10.14
11	7.23	5.99	7.18	4.8	10.8		8.01	6.73	6.05	8.31	8.03	8.35	5.77	6.03	8.28	8.05	6.43	6.97	7.75	9.88	10.14
11	7.23	6.38	7.29	4.92	10.91		8.01	6.73	6.18	8.35	8.27	8.35	5.77	6.03	8.34	8.05	6.44	7.07	7.9	9.89	10.14
11.09	7.27	6.38	7.62	4.93	11.12		8.01	6.73	6.18	8.35	8.3	8.39	5.77	6.03	8.35	8.27	6.67	7.34	8.1	9.9	10.14
11.09	7.27	6.38	7.63	5	11.36		8.43	6.73	6.22	8.4	8.39	8.47	5.77	6.03	8.35	8.27	6.73	7.36	8.1	9.98	10.14
11.14	7.27	6.38	7.76	5.09	11.44		8.43	6.8	6.24	8.49	8.4	8.74	6.05	6.03	8.35	8.36	6.73	7.4	8.25	10.15	10.14
11.25	7.27	6.38	7.81	5.09	11.51		8.43	6.86	6.3	8.51	8.47	8.76	6.11	6.03	8.58	8.36	6.74	7.43	8.25	10.26	10.2
11.73	7.48	6.47	7.81	5.21	11.51		8.43	6.86	6.37	8.53	8.48	8.76	6.15	6.04	8.6	8.69	6.74	7.43	8.26	10.28	10.31
11.73	7.49	6.47	7.81	5.43	11.82		8.43	6.93	6.38	8.53	8.48	8.76	6.15	6.11	8.66	8.79	6.74	7.52	8.44	10.28	10.34
11.73	7.5	6.48	7.81	5.43	11.83		8.43	6.96	6.38	8.58	8.48	8.76	6.15	6.12	8.68	8.79	6.9	7.69	8.5	10.34	10.4
11.96	7.5	6.48	8.13	5.45	11.83		8.46	7	6.52	8.66	8.49	8.76	6.15	6.16	8.73	8.79	6.98	7.69	8.5	10.45	10.4
12.17	7.5	6.78	8.18	5.45	11.84		8.46	7.02	6.59	8.71	8.49	8.76	6.55	6.44	8.77	8.84	7.02	7.73	8.62	10.61	10.4
12.54	7.58	6.78	8.18	5.45	12		8.46	7.02	6.59	8.73	8.52	8.76	6.6	6.49	8.77	8.84	7.07	7.84	8.83	10.62	10.4
12.54	7.58	6.78	8.18	5.49	12.03		8.46	7.04	6.6	8.74	8.53	9.12	6.65	6.51	8.77	8.85	7.07	7.85	8.91	10.62	10.4
12.54	7.58	6.78	8.18	5.49	12.03		8.94	7.04	6.69	8.74	8.7	9.13	6.65	6.51	8.79	8.85	7.12	8.11	8.91	10.62	10.4
12.58	7.58	6.78	8.19	5.49	12.13		8.94	7.05	6.7	9.08	8.79	9.14	6.65	6.51	8.8	8.85	7.14	8.17	9	10.62	10.7
13	7.75	7.09	8.19	5.49	12.28		8.94	7.06	6.78	9.09	8.84	9.15	6.65	6.51	8.8	8.85	7.14	8.28	9.01	10.62	10.82
13.55	7.79	7.2	8.38	5.49	12.32		8.94	7.07	7.03	9.21	8.92	9.15	6.65	6.51	8.8	8.95	7.18	8.33	9.02	10.62	10.82
13.62	7.8	7.21	8.59	5.49	12.44		8.94	7.07	7.15	9.39	8.93	9.19	6.74	6.66	8.8	8.95	7.18	8.33	9.02	10.62	10.84
13.62	7.8	7.21	8.67	5.64	12.44		8.94	7.15	7.16	9.42	8.93	9.27	6.74	6.66	9.11	9.08	7.24	8.4	9.02	10.71	11.09
13.62	7.8	7.21	8.67	5.64	12.44		8.94	7.17	7.16	9.46	8.93	9.29	6.74	6.67	9.18	9.08	7.24	8.48	9.02	10.72	11.14
13.62	7.8	7.21	8.71	5.64	12.44		8.95	7.18	7.2	9.76	9.11	9.31	6.74	6.76	9.18	9.09	7.25	8.58	9.14	10.72	11.15
13.62	7.96	7.21	8.91	5.64	12.6		8.95	7.2	7.2	9.78	9.17	9.53	6.74	6.89	9.19	9.15	7.25	8.58	9.14	10.74	11.15
13.62	7.96	7.22	8.91	5.64	12.63		8.95	7.2	7.2	9.79	9.17	9.53	6.74	6.89	9.3	9.52	7.35	8.67	9.32	10.74	11.16
13.62	7.96	7.22	9.01	5.64	12.63		9.32	7.23	7.21	9.79	9.18	9.53	6.74	6.9	9.34	9.53	7.36	8.71	9.47	10.81	11.59
13.8	8.24	7.35	9.01	5.64	12.64		9.32	7.24	7.21	9.79	9.18	9.53	6.76	6.9	9.4	9.53	7.46	8.71	9.48	10.82	11.6
14	8.29	7.44	9.01	5.64	12.64		9.32	7.26	7.35	9.8	9.32	9.53	6.77	6.91	9.41	9.53	7.56	9.07	9.48	10.82	11.6
14.01	8.29	7.44	9.01	5.75	12.84		9.32	7.31	7.43	9.86	9.34	9.53	6.77	7.12	9.53	9.53	7.56	9.13	9.48	10.88	11.6
14.2	8.3	7.44	9.01	5.75	12.86		9.69	7.41	7.45	9.9	9.34	9.68	6.83	7.21	9.53	9.66	7.56	9.13	9.48	10.88	11.6
14.26	8.3	7.44	9.04	5.75	12.88		10.12	7.52	7.46	9.91	9.34	9.68	6.83	7.22	9.55	9.66	7.57	9.13	9.9	10.98	11.6
14.31	8.3	7.44	9.04	5.87	12.88		10.21	7.56	7.46	9.95	9.34	9.68	6.83	7.22	9.55	9.76	7.57	9.37	9.9	11.21	11.6
14.31	8.3	7.44	9.04	5.97	12.9		10.21	7.6	7.48	9.99	9.58	9.68	6.83	7.23	9.55	9.76	7.58	9.4	9.9	11.23	11.6
14.31	8.3	7.44	9.04	5.97	12.9		10.21	7.62	7.49	9.99	9.58	9.68	6.83	7.23	9.55	9.76	7.58	9.4	9.9	11.37	11.6
14.53	8.3	7.53	9.05	5.97	13.28		10.21	7.62	7.52	9.99	9.58	9.74	6.83	7.24	10.04	10	7.58	9.4	9.9	11.39	11.6
14.68	8.53	7.53	9.05	6.01	13.32		10.21	7.62	7.79	9.99	9.59	9.87	6.83	7.34	10.04	10	7.58	9.41	10	11.5	11.6
15.35	8.53	7.6	9.05	6.01	13.32		10.21	7.72	7.94	10.23	9.59	9.88	6.84	7.34	10.04	10	7.76	9.41	10	11.51	11.6
15.49	8.69	7.6	9.26	6.01	13.32		10.21	8.18	7.95	10.25	9.96	9.88	6.84	7.34	10.04	10.01	7.76	9.41	10.19	11.53	11.6
15.49	8.69	7.61	9.26	6.01	13.32		10.21	8.24	7.95	10.25	9.96	9.88	6.84	7.56	10.04	10.01	7.76	9.5	10.19	11.65	11.6
15.49	8.7	7.61	9.27	6.28	13.36		10.21	8.24	7.97	10.3	9.96	9.91	6.86	7.56	10.04	10.01	7.76	9.5	10.19	11.71	11.6
15.49	8.7	7.62	9.27	6.28	13.5		10.21	8.24	8.07	10.42	9.96	9.91	6.86	7.67	10.04	10.01	7.77	9.51	10.19	11.71	11.74
15.49	8.7	7.62	9.27	6.28	13.66		10.21	8.24	8.23	10.43	9.96	9.91	6.86	7.68	10.05	10.02	7.77	9.58	10.19	11.71	11.84
15.49	8.7	7.62	9.27	6.28	13.66		10.21	8.24	8.24	10.44	9.96	9.92	6.86	7.69	10.05	10.02	7.78	9.65	10.19	11.71	11.85
15.49	8.7	7.62	9.27	6.3	13.66		10.21	8.36	8.26	10.71	9.97	10.11	6.86	7.69	10.11	10.28	7.78	9.66	10.19	11.71	11.87
15.49	8.71	7.63	9.27	6.31	13.72		10.21	8.36	8.31	10.71	9.97	10.18	6.88	7.7	10.11	10.28	7.78	9.66	10.2	11.71	11.92
0	0	0	0	0	0	0	0	0	0	0	0	0	0	0	0	0.25	0.08	0	0	0	0
0	0.01	0.07	0.04	0	0.09	0	0.7	0	0	0.02	0.02	0.15	0.23	0	0.16	0.33	0.18	0	0.06	0.17	0
0.17	0.01	0.07	0.09	0	0.15	0	0.7	0.1	0.09	0.06	0.13	0.16	0.29	0	0.24	0.39	0.19	0	0.17	0.17	0.18
0.26	0.02	0.07	0.09	0	0.17	0.19	0.7	0.33	0.09	0.07	0.13	0.24	0.29	0.04	0.24	0.39	0.19	0	0.21	0.17	0.38
0.33	0.02	0.07	0.09	0.16	0.27	0.19	0.7	0.35	0.2	0.13	0.14	0.25	0.38	0.14	0.24	0.49	0.19	0	0.3	0.26	0.38
0.39	0.17	0.08	0.18	0.16	0.29	0.19	0.7	0.35	0.2	0.13	0.19	0.26	0.42	0.25	0.24	0.5	0.26	0.05	0.32	0.26	0.38
0.41	0.18	0.08	0.19	0.16	0.33	0.19	0.7	0.35	0.2	0.19	0.23	0.3	0.43	0.25	0.25	0.5	0.27	0.18	0.35	0.43	0.38
0.48	0.29	0.23	0.19	0.19	0.5	0.19	0.7	0.35	0.23	0.23	0.24	0.44	0.43	0.35	0.34	0.5	0.27	0.18	0.47	0.46	0.38
0.52	0.29	0.24	0.19	0.19	0.5	0.26	0.7	0.35	0.25	0.38	0.24	0.45	0.5	0.35	0.45	0.64	0.27	0.18	0.47	0.46	0.38
0.52	0.29	0.24	0.2	0.3	0.54	0.41	0.7	0.35	0.25	0.38	0.34	0.5	0.56	0.36	0.45	0.64	0.27	0.19	0.47	0.53	0.4
0.52	0.3	0.35	0.2	0.53	0.55	0.46	0.7	0.35	0.31	0.51	0.34	0.54	0.56	0.44	0.45	0.65	0.28	0.19	0.57	0.53	0.68
0.57	0.4	0.36	0.27	0.53	0.56	0.46	0.7	0.35	0.38	0.51	0.34	0.66	0.56	0.45	0.45	0.65	0.28	0.25	0.57	0.7	0.68
0.57	0.4	0.37	0.39	0.73	0.68	0.53	0.7	0.35	0.38	0.62	0.47	0.67	0.67	0.46	0.51	0.65	0.33	0.26	0.57	0.7	0.7
0.73	0.41	0.42	0.44	0.73	0.84	0.53	0.7	0.44	0.41	0.69	0.48	0.74	0.67	0.52	0.52	0.66	0.34	0.29	0.57	0.7	0.74
0.73	0.43	0.55	0.45	0.73	0.94	0.73	0.7	0.56	0.41	0.69	0.5	0.82	0.67	0.52	0.53	0.74	0.42	0.38	0.57	0.8	0.89
0.78	0.44	0.57	0.45	0.73	0.94	0.73	1.04	0.64	0.49	0.69	0.5	0.83	0.88	0.6	0.53	0.99	0.42	0.48	0.74	0.96	0.9
0.83	0.48	0.62	0.52	0.73	0.97	0.73	1.04	0.69	0.49	0.69	0.55	0.93	0.88	0.65	0.63	1.06	0.56	0.59	0.78	0.99	0.9

0.9	0.52	0.7	0.53	0.73	1.12	0.85	1.04	0.77	0.63	0.83	0.68	0.98	0.88	0.76	0.79	1.07	0.6	0.6	0.81	1.08	1.02
1.06	0.55	0.75	0.69	0.73	1.17	0.93	1.04	0.9	0.63	0.88	0.77	1.04	0.91	0.76	0.79	1.07	0.67	0.72	0.83	1.18	1.21
1.09	0.59	0.79	0.71	0.73	1.23	0.93	1.04	0.91	0.63	0.89	0.77	1.07	1.04	0.78	0.79	1.07	0.67	0.72	0.94	1.29	1.21
1.09	0.6	0.9	0.72	0.73	1.23	0.94	1.04	0.92	0.85	0.94	0.82	1.15	1.16	0.91	0.94	1.08	0.78	0.73	0.99	1.33	1.29
1.09	0.6	0.9	0.72	0.73	1.23	0.98	1.04	0.93	0.94	1.05	0.99	1.18	1.16	0.92	0.96	1.27	0.87	0.86	1.1	1.42	1.34
1.09	0.88	0.9	0.72	0.73	1.32	1.08	1.05	0.94	0.94	1.06	1	1.25	1.16	0.92	1.2	1.27	0.91	0.9	1.11	1.42	1.34
1.22	0.88	0.91	0.72	0.73	1.4	1.24	1.05	0.99	0.94	1.15	1	1.3	1.26	0.97	1.22	1.41	0.96	0.92	1.11	1.46	1.34
1.49	0.93	0.91	0.72	0.73	1.56	1.24	1.05	1.15	0.94	1.25	1	1.31	1.26	1.03	1.22	1.5	1.07	0.99	1.29	1.56	1.34
1.65	0.94	0.92	0.86	0.74	1.69	1.27	1.19	1.15	1.06	1.3	1.12	1.31	1.4	1.03	1.22	1.5	1.08	1.04	1.33	1.56	1.44
1.65	1.05	1.09	0.97	0.79	1.76	1.27	1.4	1.15	1.12	1.38	1.12	1.41	1.4	1.1	1.31	1.51	1.18	1.08	1.41	1.56	1.44
1.76	1.11	1.17	1.03	0.83	1.87	1.27	1.63	1.15	1.16	1.44	1.21	1.42	1.41	1.12	1.55	1.51	1.2	1.15	1.48	1.77	1.47
2.04	1.13	1.28	1.06	0.83	1.89	1.28	1.63	1.3	1.44	1.5	1.22	1.42	1.41	1.12	1.55	1.63	1.3	1.19	1.5	1.77	1.47
2.28	1.13	1.28	1.22	0.83	1.89	1.35	1.79	1.3	1.44	1.56	1.38	1.42	1.57	1.14	1.55	1.69	1.3	1.24	1.61	1.77	1.57
2.78	1.31	1.29	1.23	0.83	1.89	1.41	1.79	1.39	1.44	1.63	1.38	1.5	1.62	1.24	1.68	1.69	1.36	1.35	1.65	1.89	1.59
3.03	1.32	1.44	1.33	0.83	1.94	1.41	1.79	1.52	1.44	1.63	1.39	1.65	1.7	1.27	1.8	1.69	1.36	1.36	1.77	2.03	1.6
3.18	1.36	1.45	1.34	0.83	2.1	1.46	1.79	1.52	1.44	1.64	1.42	1.7	1.7	1.28	1.9	1.7	1.4	1.42	1.83	2.13	1.61
3.24	1.36	1.59	1.41	0.83	2.2	1.47	1.79	1.6	1.46	1.64	1.42	1.76	1.7	1.28	1.99	1.7	1.4	1.42	1.83	2.27	1.9
3.24	1.36	1.65	1.41	1.03	2.28	1.47	1.79	1.67	1.53	1.8	1.59	1.77	1.8	1.31	1.99	1.71	1.58	1.45	1.83	2.35	2.11
3.25	1.37	1.66	1.42	1.29	2.28	1.56	1.94	1.67	1.69	1.86	1.68	1.81	1.8	1.47	1.99	1.79	1.59	1.46	1.83	2.5	2.12
3.25	1.52	1.66	1.42	1.29	2.28	1.62	1.94	1.67	1.74	1.88	1.76	1.81	1.8	1.56	1.99	1.8	1.6	1.57	1.85	2.51	2.24
3.45	1.53	1.66	1.42	1.29	2.35	1.62	1.94	1.67	1.74	1.99	1.76	1.91	1.92	1.57	1.99	1.8	1.66	1.59	1.85	2.54	2.33
3.45	1.53	1.75	1.42	1.29	2.35	1.68	1.94	1.67	1.79	2.02	1.88	1.95	1.92	1.62	1.99	1.96	1.74	1.63	1.85	2.54	2.42
3.45	1.64	1.75	1.54	1.29	2.5	1.68	1.96	1.73	1.79	2.06	1.89	2.01	2.03	1.67	2.06	1.96	1.82	1.72	1.91	2.54	2.42
3.45	1.73	1.99	1.72	1.29	2.54	1.68	2.1	1.8	1.96	2.1	1.89	2.02	2.09	1.68	2.23	2.1	1.82	1.77	2.04	2.59	2.58
3.88	1.74	2.12	1.72	1.29	2.54	1.77	2.36	1.87	2.11	2.14	2.05	2.02	2.21	1.71	2.27	2.19	1.86	1.83	2.1	2.59	2.58
3.88	1.75	2.12	1.72	1.3	2.59	1.77	2.4	1.98	2.32	2.15	2.05	2.04	2.22	1.72	2.27	2.19	1.99	1.83	2.1	2.6	2.58
3.88	1.92	2.13	1.81	1.38	2.61	1.77	2.59	2.08	2.32	2.15	2.05	2.07	2.24	1.77	2.38	2.3	1.99	1.85	2.11	2.69	2.64
3.88	1.95	2.28	1.81	1.44	2.73	1.77	2.81	2.14	2.35	2.17	2.05	2.13	2.24	1.86	2.4	2.31	1.99	1.92	2.19	2.69	2.64
3.95	1.96	2.28	1.81	1.52	2.8	1.91	2.87	2.14	2.35	2.5	2.22	2.13	2.24	1.89	2.4	2.46	2	1.92	2.32	2.69	2.82
3.95	1.96	2.28	1.81	1.52	2.8	1.91	2.87	2.38	2.35	2.5	2.33	2.14	2.24	1.89	2.41	2.47	2	1.92	2.45	2.85	2.83
3.95	1.96	2.28	1.81	1.57	2.8	2.19	2.87	2.46	2.35	2.51	2.33	2.14	2.31	1.89	2.42	2.56	2.01	2.04	2.54	2.85	2.84
3.99	1.97	2.28	1.82	1.57	2.8	2.19	2.87	2.46	2.35	2.52	2.33	2.14	2.32	2.04	2.43	2.56	2.01	2.06	2.67	2.86	2.85
4.02	2.13	2.29	1.82	1.88	2.8	2.19	2.87	2.46	2.35	2.6	2.33	2.22	2.38	2.05	2.5	2.57	2.02	2.09	2.67	2.87	2.92
4.17	2.13	2.29	1.88	1.88	2.8	2.19	2.87	2.46	2.4	2.6	2.34	2.28	2.4	2.15	2.62	2.57	2.02	2.09	2.67	3.08	2.93
4.17	2.13	2.49	2	1.88	2.9	2.19	2.87	2.46	2.4	2.6	2.34	2.28	2.45	2.15	2.66	2.64	2.07	2.17	2.67	3.14	2.93
4.17	2.13	2.49	2.01	1.88	3	2.3	2.87	2.46	2.4	2.65	2.34	2.38	2.45	2.16	2.71	2.65	2.07	2.17	2.67	3.14	2.93
4.17	2.22	2.5	2.01	1.88	3.1	2.31	2.87	2.46	2.45	2.65	2.35	2.41	2.54	2.24	2.73	2.65	2.22	2.22	2.68	3.27	3.07
4.17	2.23	2.5	2.01	1.9	3.1	2.32	2.87	2.47	2.45	2.77	2.35	2.45	2.59	2.31	2.73	2.66	2.22	2.28	2.75	3.27	3.08
4.17	2.23	2.51	2.02	1.9	3.25	2.32	2.87	2.47	2.49	2.91	2.36	2.45	2.64	2.43	2.89	2.85	2.23	2.3	2.77	3.29	3.08
4.26	2.23	2.68	2.02	1.9	3.25	2.33	2.87	2.47	2.52	2.91	2.36	2.45	2.64	2.43	2.89	2.86	2.23	2.3	2.77	3.29	3.1
4.26	2.24	2.68	2.03	2.08	3.26	2.52	2.87	2.47	2.52	2.92	2.36	2.46	2.64	2.43	2.91	2.86	2.24	2.3	2.94	3.47	3.13
4.26	2.39	2.68	2.03	2.11	3.28	2.52	2.87	2.47	2.61	2.94	2.65	2.52	2.64	2.44	2.93	2.87	2.24	2.3	2.94	3.47	3.14
4.26	2.48	2.69	2.03	2.26	3.28	2.52	2.87	2.63	2.61	2.94	2.65	2.6	2.64	2.44	2.99	2.87	2.24	2.31	3.01	3.47	3.18
4.3	2.48	2.69	2.04	2.26	3.48	2.52	2.87	2.63	2.72	3.04	2.66	2.6	2.72	2.44	3.01	2.87	2.37	2.31	3.01	3.47	3.18
4.3	2.49	2.7	2.04	2.26	3.49	2.52	2.87	2.63	2.9	3.04	2.73	2.6	2.72	2.44	3.02	2.88	2.37	2.43	3.01	3.47	3.19
4.3	2.49	2.7	2.04	2.26	3.55	2.57	2.87	2.67	2.9	3.04	2.74	2.6	2.72	2.57	3.04	3.01	2.37	2.5	3.03	3.47	3.19
4.3	2.5	2.82	2.05	2.26	3.55	2.57	3	2.67	2.9	3.04	2.82	2.64	2.81	2.57	3.07	3.15	2.53	2.5	3.14	3.64	3.19
4.37	2.5	2.82	2.27	2.26	3.64	2.57	3	2.67	2.9	3.15	2.84	2.66	2.81	2.58	3.09	3.16	2.54	2.53	3.16	3.65	3.19
4.39	2.57	2.83	2.28	2.26	3.75	2.57	3.01	2.7	2.9	3.15	2.84	2.66	2.81	2.58	3.1	3.16	2.54	2.54	3.17	3.65	3.19
4.62	2.62	2.83	2.28	2.26	3.76	2.72	3.11	2.78	2.9	3.18	2.91	2.75	2.81	2.59	3.1	3.17	2.54	2.55	3.18	3.71	3.42
4.62	2.62	2.91	2.28	2.26	3.76	2.72	3.11	2.78	2.9	3.18	3.04	2.76	2.81	2.84	3.37	3.24	2.55	2.67	3.27	3.77	3.45
4.62	2.63	2.92	2.29	2.26	3.76	2.73	3.11	2.83	2.9	3.18	3.04	2.79	2.81	2.84	3.43	3.34	2.64	2.7	3.31	3.84	3.45
4.63	2.66	2.92	2.29	2.29	3.84	2.9	3.23	3	2.9	3.34	3.05	2.89	2.87	2.84	3.48	3.34	2.72	2.7	3.34	3.93	3.54
4.63	2.66	2.92	2.37	2.34	3.9	2.9	3.23	3.07	2.94	3.36	3.05	2.89	2.92	2.84	3.48	3.35	2.72	2.75	3.43	4.14	3.59
4.65	2.72	3.13	2.37	2.41	3.93	2.9	3.23	3.07	2.97	3.36	3.05	2.9	2.97	2.92	3.48	3.35	2.73	2.76	3.48	4.14	3.59
4.9	2.76	3.13	2.38	2.45	3.93	2.9	3.23	3.07	3.05	3.53	3.1	2.99	2.97	2.92	3.48	3.35	2.73	2.83	3.63	4.14	3.59
5.04	2.77	3.14	2.38	2.45	3.93	3.07	3.25	3.07	3.31	3.64	3.15	3.1	3.1	2.98	3.53	3.58	2.89	2.92	3.64	4.14	3.71
5.04	2.8	3.14	2.38	2.45	4.09	3.07	3.41	3.14	3.31	3.64	3.28	3.1	3.1	3.01	3.58	3.58	2.89	2.92	3.64	4.14	3.86
5.04	2.8	3.14	2.39	2.45	4.13	3.07	3.41	3.18	3.31	3.64	3.29	3.1	3.1	3.01	3.62	3.59	2.9	2.93	3.66	4.14	3.89
5.04	2.81	3.26	2.48	2.45	4.13	3.07	3.41	3.28	3.31	3.81	3.29	3.1	3.11	3.01	3.77	3.59	2.9	2.99	3.77	4.32	3.89
5.04	2.84	3.27	2.68	2.45	4.21	3.07	3.41	3.28	3.31	3.82	3.35	3.2	3.15	3.1	3.8	3.59	3	3.05	3.77	4.32	3.89
5.23	2.84	3.44	2.69	2.46	4.3	3.3	3.41	3.28	3.31	3.83	3.42	3.2	3.44	3.11	3.8	3.66	3	3.11	3.77	4.32	3.92
5.23	2.85	3.44	2.76	2.49	4.49	3.3	3.45	3.28	3.31	3.84	3.51	3.37	3.44	3.23	3.86	3.67	3.07	3.19	3.77	4.39	4.02
5.33	2.88	3.47	2.82	2.49	4.58	3.3	3.48	3.28	3.31	3.95	3.65	3.41	3.44	3.25	3.93	3.72	3.08	3.23	3.85	4.52	4.04
5.33	2.88	3.53	2.91	2.56	4.61	3.34	3.73	3.29	3.31	4.02	3.82	3.48	3.44	3.31	3.97	3.75	3.13	3.27	4	4.61	4.04
5.55	2.94	3.59	2.92	2.56	4.73	3.47	3.73	3.42	3.31	4.02	3.87	3.51	3.44	3.32	4.03	3.85	3.13	3.34	4.1	4.74	4.16
5.78	2.94	3.65	2.92	2.56	4.85	3.5	3.85	3.48	3.31	4.09	3.9	3.57	3.44	3.34	4.05	3.85	3.2	3.4	4.1	4.82	4.21
5.86	3.05	3.66	2.92	2.63	4.9	3.58	3.93	3.55	3.31	4.13	3.97	3.57	3.44	3.34	4.21	4.09	3.33	3.46	4.1	4.84	4.21
6.24	3.08	3.77	3.02	2.69	5	3.67	4.04	3.64	3.33	4.19	4.03	3.7	3.44	3.34	4.35	4.11	3.38	3.46	4.1	4.95	4.34
6.5	3.08	3.78	3.13	2.69	5.05	3.71	4.16	3.67	3.35	4.27	4.09	3.7	3.5	3.44	4.35	4.26	3.38	3.59	4.12	4.95	4.47
6.5	3.12	3.78	3.13	2.71	5.05	3.71	4.16	3.67	3.38	4.32	4.21	3.7	3.58	3.55	4.38	4.39	3.48	3.6	4.26	5.13	4.51
6.5	3.27	3.78	3.13	2.74	5.07	3.71	4.16	3.67	3.41	4.35	4.22	3.84	3.67	3.63	4.41	4.52	3.51	3.67	4.26	5.19	4.51
6.5	3.28	3.89	3.14	2.84	5.19	3.71	4.16	3.67	3.41	4.35	4.29	3.84	3.7	3.64	4.51	4.53	3.56	3.69	4.28	5.19	4.51
6.56	3.28	4.08	3.24	2.84	5.33	4.01	4.27	3.77	3.62	4.35	4.36	3.88	3.79	3.7	4.54	4.53	3.57	3.72	4.5	5.19	4.51
6.98	3.41	4.16	3.24	2.93	5.37	4.01	4.65	3.83	3.62	4.35	4.41	3.88	3.9	3.75	4.57	4.53	3.74	3.73	4.5	5.25	4.54
7.26	3.5	4.2	3.35	2.99	5.37	4.01	4.65	3.93	3.93	4.41	4.51	3.96	3.97	3.81	4.64	4.62	3.81	3.8	4.58	5.33	4.81
8.36	3.5	4.31	3.43	2.99	5.37	4.14	4.65	4.04	3.93	4.59	4.6	4.07	3.97	3.81	4.81	4.79	3.84	3.8	4.7	5.33	4.81
8.79	3.5	4.31	3.54	3.03	5.41	4.14	4.65	4.04	3.93	4.59	4.64	4.1	3.97	3.98	4.82	4.87	3.88	3.86	4.7	5.36	5.1
8.82	3.54	4.32	3.55	3.13	5.58	4.14	4.65	4.05	4.04	4.6	4.67	4.11	3.97	4.04	4.82	4.87	3.88	4.01	4.7	5.43	5.15
8.82	3.6	4.42	3.55	3.28	5.69	4.14	4.65	4.06	4.05	4.73	4.67	4.11	3.97	4.07	4.82	4.87	3.96	4.08	4.71	5.56	5.15
8.82	3.63	4.43	3.71	3.28	5.75	4.3	4.65	4.09	4.05	4.74	4.78	4.11	3.98	4.09	4.91	4.87	4.02	4.19	4.72	5.65	5.15
8.82	3.68	4.43	3.72	3.28	5.81	4.3	4.68	4.11	4.1	4.79	4.82	4.22	4.04	4.1	5.04	4.88	4.1	4.2	4.82	5.69	5.15
9.07	3.68	4.48	3.76	3.28	5.88	4.42	4.97	4.27	4.1	4.93	4.82	4.25	4.04	4.14	5.27	5.01	4.1	4.25	4.92	5.74	5.18
9.1	3.68	4.48	3.76	3.28	5.92	4.44	4.97	4.27	4.31	5.05	4.83	4.25	4.12	4.14	5.35	5.07	4.11	4.36	4.93	5.8	5.21
9.23	3.82	4.49	3.81	3.29	5.92	4.44	4.98	4.27	4.31	5.1	4.83	4.25	4.12	4.14	5.42	5.22	4.11	4.36	4.94	5.88	5.21
9.73	3.83	4.62	3.82	3.46	5.92	4.76	5.37	4.31	4.31	5.26	4.88	4.26	4.12	4.14	5.42	5.37	4.24	4.37	5.01	5.94	5.34
9.73	3.9	4.63	3.96	3.56	6.03	4.85	5.37	4.32	4.61	5.27	4.9	4.38	4.14	4.28	5.49	5.46	4.29	4.37	5.14	5.94	5.34
9.73	4.06	4.63	3.97	3.56	6.06	4.85	5.37	4.32	4.61	5.35	4.9	4.38	4.14	4.29	5.62	5.49	4.29	4.37	5.33	6.04	5.53
9.73	4.06	4.81	3.97	3.56	6.1	4.85	5.37	4.32	4.74	5.44	4.95	4.43	4.24	4.5	5.66	5.52	4.32	4.44	5.36	6.23	5.65
9.73	4.15	4.82	4.1	3.61	6.23	4.85	5.37	4.4	4.74	5.45	5.01	4.49	4.28	4.53	5.68	5.58	4.36	4.63	5.46	6.36	5.65
9.79	4.25	4.82	4.1	3.73	6.3	4.88	5.37	4.47	4.74	5.54	5.03	4.5	4.29	4.53	5.68	5.58	4.42	4.68	5.48	6.36	5.66
9.79	4.26	4.82	4.1	3.73	6.36	5.06	5.37	4.57	4.74	5.6	5.04	4.5	4.3	4.53	5.7	5.69	4.42	4.68	5.48	6.36	5.66
9.79	4.32	4.89	4.1	3.73	6.41	5.11	5.37	4.79	4.74	5.67	5.04	4.5	4.33	4.54	5.87	5.77	4.43	4.81	5.48	6.36	5.83
9.79	4.32	5.03	4.1	3.9	6.41	5.27	5.37	4.79	4.94	5.77	5.12	4.56	4.33	4.68	5.87	5.77	4.44	4.81	5.49	6.52	5.96
9.79	4.32	5.04	4.1	3.9	6.41	5.27	5.37	4.79	5.15	5.85	5.21	4.56	4.33	4.68	5.87	5.78	4.44	4.85	5.53	6.54	5.96
9.8	4.32	5.04	4.1	3.9	6.41	5.27	5.58	4.79	5.15	5.88	5.21	4.56	4.33	4.69	5.98	5.78	4.6	4.86	5.58	6.63	6.1
9.8	4.32	5.04	4.1	3.9	6.46	5.29	5.61	4.79	5.15	5.89	5.21	4.71	4.33	4.69	5.99	5.79	4.6	4.86	5.58	6.69	6.16
10.05	4.33	5.05	4.19	3.9	6.54	5.29	5.61	4.79	5.15	5.9	5.21	4.72	4.33	4.87	5.99	5.92	4.63	4.86	5.75	6.69	6.16
10.05	4.54	5.12	4.19	3.9	6.54	5.31	5.97	4.79	5.15	6.02	5.3	4.72	4.33	4.98	6.13	5.92	4.7	4.87	5.75	6.69	6.16
10.05	4.54	5.32	4.2	3.9	6.54	5.37	6.05	4.81	5.15	6.02	5.44	4.84	4.34	4.98	6.14	5.93	4.76	4.92	6	6.69	6.16
10.05	4.68	5.33	4.33	4	6.59	5.37	6.05	4.94	5.15	6.02	5.45	4.85	4.43	4.98	6.16	5.93	4.82	4.93	6	6.82	6.16
10.05	4.69	5.33	4.33	4	6.6	5.37	6.1	4.94	5.15	6.07	5.45	4.91	4.52	4.99	6.16	5.94	4.9	4.94	6	6.82	6.16
10.05	4.7	5.33	4.33	4.17	6.83	5.37	6.1	4.98	5.15	6.07	5.45	5.02	4.53	5	6.21	5.94	4.9	4.94	6	6.83	6.16
10.18	4.7	5.34	4.33	4.17	6.83	5.4	6.1	5.06	5.15	6.07	5.51	5.03	4.66	5	6.26	6.15	4.98	4.94	6	6.83	6.16
10.32	4.7	5.34	4.34	4.17	6.83	5.55	6.1	5.15	5.15	6.16	5.61	5.03	4.66	5.18	6.31	6.23	4.98	4.94	6	6.93	6.17
10.32	4.71	5.35	4.34	4.17	6.86	5.55	6.31	5.15	5.15	6.36	5.66	5.03	4.66	5.18	6.31	6.24	4.99	5.02	6	6.93	6.27
10.32	4.8	5.5	4.34	4.21	6.88	5.65	6.31	5.22	5.15	6.36	5.66	5.03	4.75	5.18	6.31	6.24	5.07	5.03	6	6.93	6.29
10.32	4.84	5.5	4.35	4.32	7.03	5.65	6.31	5.22	5.23	6.36	5.77	5.13	4.79	5.18	6.31	6.24	5.07	5.04	6.04	6.93	6.36
10.32	4.84	5.51	4.47	4.36	7.03	5.65	6.31	5.27	5.23	6.36	5.77	5.14	4.79	5.18	6.35	6.25	5.08	5.04	6.04	6.93	6.49
10.32	4.84	5.51	4.48	4.36	7.13	5.65	6.31	5.33	5.23	6.36	5.77	5.17	4.79	5.19	6.36	6.25	5.08	5.05	6.05	7.03	6.53
10.32	4.85	5.73	4.48	4.38	7.16	5.65	6.31	5.37	5.23	6.36	5.78	5.17	4.94	5.19	6.36	6.25	5.09	5.05	6.11	7.03	6.53
10.32	4.85	5.73	4.65	4.51	7.2	5.65	6.31	5.37	5.23	6.44	5.84	5.18	4.94	5.29	6.45	6.26	5.18	5.05	6.15	7.1	6.53
10.32	4.85	5.73	4.66	4.51	7.31	5.69	6.31	5.37	5.23	6.44	6.02	5.18	4.94	5.29	6.45	6.38	5.18	5.16	6.16	7.1	6.53
10.32	4.96	5.74	4.66	4.51	7.31	5.69	6.36	5.37	5.48	6.46	6.02	5.33	4.94	5.3	6.47	6.45	5.21	5.16	6.19	7.23	6.57
10.32	4.96	5.74	4.67	4.51	7.43	5.69	6.63	5.37	5.48	6.47	6.02	5.33	4.97	5.3	6.47	6.45	5.24	5.16	6.19	7.23	6.57
10.32	4.96	5.75	4.67	4.51	7.43	5.69	6.63	5.37	5.48	6.49	6.03	5.48	4.97	5.38	6.71	6.46	5.25	5.35	6.24	7.23	6.66
10.32	4.97	5.96	4.68	4.57	7.43	5.69	6.63	5.43	5.48	6.62	6.03	5.48	5.04	5.39	6.8	6.46	5.25	5.36	6.26	7.32	6.73
10.32	5.04	5.96	4.68	4.57	7.43	5.77	6.63	5.43	5.89	6.65	6.19	5.48	5.04	5.5	6.81	6.46	5.32	5.37	6.4	7.32	6.75
10.36	5.11	5.96	4.68	4.57	7.57	5.77	6.63	5.43	5.89	6.78	6.19	5.65	5.04	5.5	6.81	6.59	5.32	5.37	6.42	7.32	6.76
10.45	5.11	6.01	4.69	4.57	7.57	5.77	6.63	5.43	5.89	6.78	6.2	5.65	5.24	5.5	6.81	6.8	5.49	5.37	6.46	7.43	6.88
10.45	5.11	6.02	4.69	4.67	7.66	5.77	6.63	5.43	5.89	6.79	6.26	5.65	5.24	5.6	6.81	6.91	5.49	5.57	6.7	7.57	6.89
10.45	5.12	6.02	4.69	4.74	7.66	6.17	6.63	5.44	5.89	7.01	6.27	5.65	5.24	5.61	6.81	6.91	5.49	5.58	6.73	8	6.89
10.5	5.12	6.06	4.81	4.74	7.83	6.32	6.63	5.57	5.89	7.01	6.38	5.72	5.24	5.61	6.82	6.92	5.5	5.58	6.73	8.13	6.92
10.56	5.16	6.07	4.81	4.76	7.83	6.34	6.67	5.61	5.89	7.02	6.38	5.8	5.4	5.71	7.04	6.92	5.5	5.59	6.73	8.21	6.92
10.56	5.27	6.15	4.82	4.86	7.87	6.34	6.71	5.68	5.89	7.03	6.52	5.85	5.44	5.71	7.05	6.92	5.53	5.6	6.73	8.29	6.92
10.61	5.31	6.26	4.89	4.92	7.9	6.34	6.71	5.68	5.9	7.03	6.52	5.87	5.46	5.71	7.05	6.92	5.6	5.61	6.79	8.29	6.92
10.61	5.34	6.34	5.01	4.94	7.97	6.34	6.71	5.68	5.9	7.09	6.67	5.87	5.6	5.8	7.05	6.92	5.65	5.72	6.8	8.29	6.92
11.3	5.34	6.43	5.15	4.96	8.13	6.38	6.71	5.69	5.9	7.16	6.76	5.88	5.6	5.81	7.09	7.02	5.65	5.82	6.9	8.29	7.06
11.34	5.4	6.49	5.16	5.05	8.24	6.38	6.81	5.82	5.9	7.3	6.84	5.9	5.6	5.82	7.09	7.07	5.66	5.84	7.05	8.29	7.12
11.58	5.45	6.56	5.16	5.05	8.35	6.38	6.88	5.82	5.94	7.41	6.86	6.01	5.76	5.86	7.18	7.13	5.73	5.92	7.05	8.29	7.14
11.58	5.49	6.6	5.18	5.05	8.38	6.38	7.2	5.85	5.94	7.42	6.86	6.04	5.86	5.91	7.18	7.18	5.76	5.94	7.16	8.34	7.19
11.58	5.49	6.65	5.19	5.05	8.51	6.38	7.2	5.85	5.94	7.42	6.97	6.05	5.86	5.91	7.18	7.2	5.76	5.94	7.16	8.43	7.32

11.72	5.6	6.69	5.23	5.08	8.53	6.38	7.2	5.85	5.96
11.89	5.69	6.69	5.23	5.14	8.65	6.38	7.2	5.85	5.96
11.9	5.69	6.69	5.24	5.14	8.7	6.38	7.2	5.93	5.96
11.9	5.74	6.69	5.24	5.32	8.7	6.38	7.2	5.97	6.02
11.9	5.74	6.7	5.53	5.47	8.7	6.44	7.2	5.99	6.1
11.9	5.74	6.9	5.58	5.47	8.7	6.51	7.2	6.1	6.29
12.07	5.74	6.94	5.58	5.47	8.77	6.51	7.28	6.15	6.29
12.15	5.83	6.94	5.58	5.47	8.9	6.6	7.37	6.25	6.29
12.15	5.93	7.06	5.58	5.49	8.9	6.6	7.37	6.36	6.48
12.27	5.93	7.06	5.58	5.49	9.06	6.6	7.37	6.52	6.48
12.43	5.93	7.24	5.58	5.56	9.24	6.96	7.37	6.65	6.48
12.43	6	7.31	5.78	5.56	9.24	7.04	7.37	6.65	6.48
12.52	6.01	7.41	5.79	5.56	9.39	7.05	7.68	6.76	6.48
12.52	6.06	7.42	5.92	5.56	9.47	7.06	8.02	6.76	6.48
12.52	6.06	7.46	6	5.61	9.55	7.06	8.02	6.76	6.48
12.75	6.1	7.54	6.11	5.61	9.61	7.12	8.02	6.76	6.48
12.75	6.11	7.54	6.11	5.63	9.63	7.12	8.02	6.77	6.52
12.75	6.21	7.54	6.12	5.83	9.63	7.29	8.02	6.95	6.52
12.76	6.29	7.54	6.22	5.87	9.68	7.29	8.02	6.95	6.52
12.91	6.3	7.55	6.22	5.87	9.69	7.3	8.02	6.95	6.87
12.91	6.4	7.69	6.33	5.97	9.72	7.37	8.02	6.95	6.87
12.93	6.56	7.86	6.34	5.97	9.72	7.48	8.41	6.97	6.87
12.93	6.57	8.02	6.34	6.03	9.81	7.48	8.41	7	6.87
13	6.57	8.06	6.34	6.03	9.86	7.66	8.41	7.03	6.87
13	6.57	8.06	6.46	6.03	10.05	7.76	8.41	7.18	6.87
13.1	6.57	8.11	6.46	6.15	10.25	7.76	8.41	7.18	6.87
13.37	6.73	8.29	6.46	6.16	10.25	7.81	8.41	7.18	6.87
13.38	6.73	8.29	6.46	6.16	10.28	7.81	8.41	7.38	6.87
13.38	6.74	8.29	6.47	6.18	10.28	7.89	8.41	7.38	6.87
13.42	6.74	8.3	6.47	6.18	10.28	7.89	8.41	7.38	6.96
13.42	6.74	8.3	6.58	6.18	10.28	7.99	8.52	7.38	7.07
13.63	6.9	8.3	6.58	6.18	10.28	7.99	8.53	7.38	7.07
13.68	6.9	8.31	6.58	6.19	10.43	8	8.73	7.38	7.07
13.81	6.91	8.31	6.58	6.29	10.43	8	8.75	7.38	7.36
13.81	7.04	8.32	6.59	6.29	10.43	8.11	8.77	7.38	7.36
13.81	7.04	8.32	6.59	6.42	10.43	8.11	8.77	7.46	7.36
13.81	7.04	8.53	6.59	6.48	10.43	8.11	8.77	7.63	7.55
13.81	7.05	8.62	6.6	6.48	10.52	8.11	8.77	7.69	7.55
13.81	7.11	8.62	6.6	6.48	10.68	8.11	8.77	7.72	7.55
13.81	7.12	8.63	6.6	6.48	10.68	8.11	8.77	7.74	7.55
13.81	7.12	8.63	6.6	6.48	10.71	8.11	8.77	7.76	7.55
13.81	7.12	8.63	6.61	6.48	10.71	8.11	8.77	7.78	7.61
13.81	7.12	8.72	6.75	6.48	10.71	8.11	8.77	7.78	7.61
14	7.31	8.73	6.75	6.48	10.78		8.81	7.88	7.61
14	7.32	8.73	6.75	6.5	10.78		8.81	7.88	7.61
14	7.32	8.74	6.76	6.5	10.78		8.87	7.9	7.61
14	7.32	8.74	6.76	6.5	10.95		9.01	7.9	7.61
14.05	7.33	8.75	6.87	6.5	10.95		9.1	7.9	7.61
14.08	7.41	8.78	6.87	6.54	10.97		9.13	7.9	7.61
14.08	7.41	8.78	6.88	6.54	11.08		9.15	7.94	7.66
14.08	7.55	8.79	6.88	6.57	11.08		9.15	7.94	7.69
14.14	7.56	8.94	6.88	6.57	11.13		9.18	7.96	7.69
14.14	7.56	8.94	6.89	6.57	11.13		9.39	8.01	7.77
14.14	7.56	8.95	6.89	6.57	11.13		9.64	8.42	7.81
14.14	7.57	9.14	6.89	6.57	11.24		9.64	8.42	7.92
14.14	7.62	9.14	6.99	6.6	11.26		9.64	8.56	8.14
14.14	7.62	9.15	6.99	6.64	11.26		9.64	8.66	8.14
14.14	7.62	9.28	6.99	6.64	11.35		9.64	8.71	8.14
14.16	7.72	9.28	7	6.65	11.37		9.74	8.74	8.14
14.16	7.72	9.28	7.12	6.66	11.46		9.74	8.82	8.14
14.2	7.73	9.29	7.32	6.69	11.49		9.74	8.82	8.14
14.36	7.78	9.38	7.43	6.81	11.49		9.85	8.82	8.29
14.39	7.78	9.5	7.44	6.91	11.58		9.85	8.82	8.29
14.39	7.86	9.62	7.52	6.91	11.69		9.85	8.82	8.29
14.49	7.94	9.67	7.6	6.95	11.93		9.91	8.82	8.29
14.49	7.96	9.67	7.61	7.02	11.93		9.91	8.82	8.29
14.83	8.01	9.8	7.61	7.12	12.15		9.91	8.89	8.29
14.92	8.01	9.84	7.61	7.2	12.17		9.96	9.01	8.29
14.92	8.12	9.85	7.62	7.34	12.24		9.96	9.01	8.29
14.92	8.2	9.94	7.89	7.34	12.28		10.19	9.01	8.29
14.92	8.21	9.95	7.9	7.34	12.28		10.19	9.01	8.36
15.14	8.21	9.96	7.9	7.34	12.28		10.19	9.01	8.36
15.21	8.28	9.96	7.9	7.35	12.28		10.19	9.1	8.53
15.38	8.35	10.14	7.98	7.35	12.35		10.36	9.1	8.53
15.38	8.38	10.28	8.1	7.35	12.68		10.36	9.28	8.8
15.38	8.53	10.28	8.21	7.43	12.7		10.44	9.28	8.8
15.38	8.53	10.29	8.22	7.43	12.72		10.71	9.28	8.8
15.38	8.53	10.4	8.22	7.53	12.74		10.82	9.28	8.8
15.43	8.53	10.4	8.37	7.53	12.74		10.82	9.37	8.8
15.43	8.54	10.48	8.37	7.53	12.85		10.9	9.38	8.8
15.64	8.63	10.48	8.37	7.65	12.89		10.9	9.38	8.8
15.75	8.71	10.48	8.53	7.65	12.95		10.93	9.51	8.8
16	8.87	10.61	8.53	7.65	12.95		10.93	9.59	8.8
16.11	8.88	10.62	8.54	7.65	13.18		11.08	9.59	8.8
16.11	8.88	10.85	8.54	7.65	13.23		11.1	9.59	8.8
16.27	8.88	10.94	8.63	7.65	13.23		11.1	9.59	8.8
16.27	8.88	11.07	8.63	7.65	13.23		11.1	9.59	9.05
16.27	8.99	11.14	8.63	7.72	13.23		11.41	9.59	9.05
16.83	9.13	11.16	8.64	7.72	13.41		11.47	9.86	9.05
16.83	9.18	11.17	8.75	7.73	13.48		11.64	9.86	9.09
16.83	9.19	11.3	8.75	7.75	13.68		11.64	9.88	9.09
16.83	9.19	11.31	8.76	7.83	14.08		11.64	10.19	9.14
16.83	9.19	11.31	8.76	7.83	14.18		11.72	10.19	9.14
16.83	9.22	11.31	8.76	7.83	14.18		11.72	10.52	9.29
16.83	9.22	11.32	8.77	7.83	14.18		11.72	10.54	9.34
16.83	9.23	11.32	8.77	7.83	14.18		11.72	10.54	9.34
16.83	9.23	11.32	8.77	7.83	14.18		11.87	10.54	9.34
16.83	9.23	11.45	8.9	7.86	14.22		11.87	10.54	9.34
16.83	9.38	11.46	8.91	7.86	14.22		11.87	10.54	9.34
16.83	9.38	11.46	8.91	7.86	14.25		11.87	10.54	9.34
16.86	9.5	11.63	8.91	7.89	14.25		11.87	10.54	9.34
16.86	9.5	11.63	8.91	7.91	14.28		11.87	10.54	9.34
16.86	9.5	11.64	8.92	7.98	14.29		11.9	10.54	9.55
16.86	9.5	11.72	8.92	7.99	14.29		11.98	10.54	9.71
16.86	9.65	11.73	8.92	7.99	14.43		12.02	10.54	9.71
16.86	9.65	11.73	8.93	7.99	14.47		12.02	10.54	9.72
16.86	9.66	11.78	8.93	7.99	14.5		12.02	10.54	9.72

7.43	6.98	6.11	5.86	5.97	7.21	7.28	5.79	6.06	7.16	8.5	7.43
7.51	7.06	6.17	5.86	6.01	7.29	7.3	5.87	6.16	7.17	8.62	7.66
7.51	7.06	6.2	5.86	6.02	7.35	7.41	5.87	6.29	7.17	8.73	7.66
7.51	7.09	6.22	5.86	6.11	7.35	7.5	5.88	6.32	7.4	8.82	7.66
7.51	7.1	6.23	6.11	6.12	7.35	7.62	5.94	6.32	7.5	8.87	7.66
7.6	7.1	6.23	6.13	6.2	7.46	7.62	5.94	6.45	7.51	8.89	7.71
7.78	7.2	6.23	6.15	6.21	7.46	7.62	5.95	6.47	7.56	9.08	7.94
7.78	7.34	6.52	6.15	6.29	7.51	7.63	6.06	6.47	7.68	9.16	8.04
7.9	7.43	6.52	6.15	6.47	7.62	7.63	6.06	6.59	7.76	9.27	8.11
8.06	7.49	6.52	6.25	6.55	7.63	7.63	6.12	6.68	7.84	9.33	8.13
8.08	7.51	6.66	6.29	6.55	7.7	7.84	6.13	6.78	7.95	9.39	8.17
8.16	7.54	6.69	6.37	6.64	7.76	7.96	6.28	6.88	8.07	9.51	8.27
8.23	7.54	6.71	6.42	6.69	7.9	7.97	6.35	6.91	8.15	9.61	8.45
8.23	7.54	6.72	6.44	6.69	7.93	7.97	6.42	6.99	8.16	9.68	8.46
8.23	7.67	6.72	6.44	6.69	7.95	7.98	6.49	7.05	8.33	9.74	8.46
8.23	7.67	6.72	6.44	6.69	7.99	7.98	6.5	7.05	8.33	9.83	8.54
8.23	7.77	6.86	6.44	6.7	8.06	7.98	6.53	7.05	8.4	9.84	8.6
8.25	7.77	6.95	6.66	6.82	8.1	8.23	6.53	7.05	8.4	9.84	8.68
8.48	7.78	6.96	6.66	6.82	8.14	8.27	6.53	7.22	8.44	9.95	8.68
8.53	7.92	6.96	6.66	6.93	8.27	8.32	6.64	7.31	8.45	10.04	8.71
8.54	7.92	7.06	6.66	7.14	8.35	8.4	6.71	7.32	8.45	10.04	8.71
8.77	7.97	7.29	6.67	7.14	8.35	8.45	6.71	7.37	8.6	10.16	8.99
8.81	8.13	7.31	6.73	7.15	8.35	8.45	6.86	7.44	8.74	10.16	9.04
8.91	8.21	7.37	6.96	7.33	8.35	8.45	6.94	7.59	8.74	10.19	9.04
8.99	8.24	7.37	6.98	7.43	8.42	8.56	6.95	7.65	8.74	10.26	9.05
9.02	8.27	7.37	7	7.43	8.46	8.57	6.95	7.66	8.75	10.28	9.05
9.13	8.37	7.38	7.03	7.51	8.48	8.74	6.98	7.66	8.79	10.46	9.25
9.15	8.41	7.38	7.03	7.58	8.49	8.74	6.98	7.8	8.97	10.47	9.26
9.15	8.41	7.51	7.03	7.58	8.49	8.74	7.02	7.8	8.97	10.47	9.3
9.15	8.41	7.52	7.03	7.58	8.5	8.74	7.02	7.8	8.97	10.57	9.37
9.15	8.42	7.52	7.03	7.58	8.5	8.75	7.2	7.8	9.12	10.57	9.37
9.15	8.42	7.52	7.14	7.72	8.63	8.85	7.2	7.8	9.12	10.58	9.37
9.4	8.43	7.52	7.15	7.73	8.64	8.85	7.2	7.8	9.12	10.66	9.38
9.4	8.43	7.58	7.16	7.73	8.64	8.85	7.2	7.89	9.26	10.74	9.42
9.41	8.43	7.73	7.28	7.79	8.78	8.91	7.2	7.9	9.27	10.75	9.47
9.42	8.64	7.74	7.3	7.8	8.78	9.04	7.21	7.91	9.27	10.84	9.47
9.51	8.64	7.74	7.3	7.86	8.9	9.04	7.33	7.91	9.27	10.86	9.47
9.51	8.74	7.74	7.3	7.86	8.93	9.04	7.38	7.91	9.27	10.94	9.47
9.52	8.74	7.84	7.37	7.86	8.94	9.05	7.38	7.98	9.43	10.94	9.47
9.61	8.74	7.84	7.47	7.86	8.95	9.05	7.39	7.98	9.43	10.95	9.47
9.61	8.75	7.85	7.47	7.87	8.96	9.05	7.4	7.99	9.43	11.05	9.57
9.71	8.75	7.85	7.47	7.87	8.96	9.19	7.4	8.04	9.59	11.05	9.58
9.71	8.75	7.98	7.47	7.88	9.07	9.19	7.42	8.04	9.6	11.14	9.58
9.71	8.82	7.99	7.47	8.17	9.08	9.19	7.42	8.05	9.6	11.14	9.58
9.81	8.91	7.99	7.47	8.17	9.08	9.19	7.42	8.05	9.63	11.16	9.58
9.81	8.91	8	7.57	8.18	9.09	9.19	7.49	8.06	9.63	11.16	9.58
9.81	8.92	8	7.61	8.18	9.22	9.19	7.5	8.12	9.63	11.16	9.58
9.81	8.92	8.09	7.62	8.21	9.27	9.19	7.5	8.18	9.75	11.25	9.58
9.89	8.95	8.1	7.62	8.21	9.27	9.28	7.5	8.19	9.75	11.26	9.58
9.9	8.96	8.1	7.76	8.22	9.27	9.29	7.51	8.2	9.87	11.37	9.58
9.91	9.14	8.15	7.76	8.3	9.27	9.29	7.7	8.2	9.87	11.37	9.58
10.01	9.14	8.25	7.76	8.34	9.27	9.29	7.77	8.28	9.87	11.37	9.58
10.11	9.14	8.47	7.76	8.34	9.27	9.38	7.77	8.29	9.88	11.41	9.58
10.11	9.14	8.47	8.3	8.44	9.43	9.39	7.78	8.29	9.99	11.5	9.58
10.12	9.25	8.47	8.3	8.44	9.49	9.39	7.78	8.29	10.14	11.54	9.64
10.13	9.26	8.47	8.3	8.45	9.54	9.39	7.82	8.45	10.15	11.54	9.74
10.28	9.32	8.6	8.3	8.45	9.54	9.39	7.85	8.45	10.15	11.56	9.74
10.29	9.33	8.6	8.3	8.5	9.59	9.61	7.85	8.45	10.2	11.64	9.91
10.36	9.33	8.6	8.3	8.56	9.62	9.61	7.94	8.45	10.26	11.73	9.97
10.37	9.4	8.67	8.3	8.57	9.65	9.61	7.95	8.57	10.27	11.73	10.13
10.45	9.47	8.71	8.38	8.57	9.71	9.62	8.01	8.65	10.27	11.77	10.26
10.59	9.55	8.74	8.38	8.57	9.78	9.72	8.04	8.75	10.46	11.99	10.58
10.59	9.59	8.76	8.65	8.62	9.91	9.74	8.05	8.76	10.53	12.16	10.64
10.63	9.64	8.82	8.65	8.62	9.93	9.82	8.14	8.8	10.53	12.27	10.75
10.72	9.7	8.88	8.65	8.63	9.95	9.94	8.14	8.84	10.65	12.4	10.78
10.75	9.76	8.95	8.65	8.64	9.95	9.99	8.18	8.93	10.66	12.41	10.88
10.83	9.83	9.03	8.65	8.7	9.96	10.08	8.19	8.97	10.66	12.47	10.88
10.83	9.83	9.03	8.86	8.71	10.1	10.08	8.22	9.06	10.67	12.58	10.91
10.86	9.94	9.08	9.29	8.91	10.14	10.18	8.31	9.16	10.72	12.71	11.19
10.87	10.03	9.08	9.29	9	10.17	10.32	8.31	9.16	10.81	12.71	11.19
10.88	10.03	9.15	9.29	9.01	10.19	10.33	8.36	9.24	10.9	12.77	11.2
10.95	10.03	9.24	9.29	9.01	10.28	10.33	8.37	9.27	11	12.77	11.2
11.05	10.04	9.24	9.29	9.01	10.38	10.33	8.43	9.3	11.01	12.82	11.34
11.06	10.04	9.33	9.29	9.01	10.42	10.44	8.43	9.36	11.18	13.01	11.51
11.2	10.05	9.4	9.29	9.21	10.44	10.45	8.52	9.47	11.18	13.05	11.51
11.41	10.15	9.48	9.29	9.25	10.52	10.5	8.54	9.47	11.27	13.12	11.58
11.41	10.27	9.48	9.29	9.28	10.54	10.59	8.67	9.51	11.35	13.22	11.6
11.42	10.31	9.52	9.29	9.3	10.6	10.65	8.86	9.67	11.36	13.22	11.6
11.42	10.31	9.52	9.29	9.3	10.62	10.78	8.9	9.76	11.36	13.3	11.96
11.59	10.31	9.52	9.29	9.31	10.82	10.78	8.94	9.82	11.58	13.38	11.96
11.75	10.32	9.7	9.29	9.31	10.88	10.78	8.99	9.87	11.66	13.39	12.08
11.84	10.32	9.76	9.29	9.32	10.93	10.87	9.01	9.87	11.71	13.56	12.08
11.86	10.32	9.82	9.29	9.41	11.01	10.88	9.04	9.89	11.75	13.64	12.08
11.91	10.39	9.82	9.31	9.41	11.09	10.88	9.07	9.89	11.85	13.64	12.16
11.91	10.47	9.82	9.43	9.65	11.16	10.92	9.15	9.9	11.86	13.7	12.25
11.92	10.52	9.82	9.43	9.66	11.16	11	9.16	10.15	11.94	13.74	12.41
12.08	10.58	9.9	9.43	9.67	11.18	11.01	9.16	10.27	12.1	13.74	12.43
12.22	10.59	9.91	9.63	9.75	11.23	11.01	9.17	10.28	12.18	13.83	12.44
12.27	10.59	9.99	9.63	9.75	11.23	11.01	9.38	10.29	12.18	13.9	12.44
12.31	10.59	10.1	9.63	9.75	11.23	11.11	9.42	10.29	12.27	13.9	12.59
12.31	10.59	10.14	9.63	9.76	11.23	11.12	9.42	10.41	12.28	13.95	12.59
12.45	10.6	10.17	9.66	9.93	11.33	11.12	9.42	10.47	12.28	13.95	12.59
12.47	10.71	10.18	9.98	10.02	11.34	11.24	9.43	10.47	12.45	14.11	12.81
12.47	10.78	10.3	10.03	10.02	11.39	11.24	9.43	10.47	12.45	14.11	12.87
12.56	10.78	10.3	10.03	10.02	11.51	11.25	9.5	10.47	12.45	14.11	12.87
12.56	10.78	10.3	10.03	10.02	11.61	11.31	9.56	10.48	12.46	14.24	12.87
12.56	10.78	10.3	10.03	10.12	11.69	11.31	9.56	10.64	12.54	14.29	12.87
12.65	10.78	10.42	10.03	10.13	11.7	11.43	9.57	10.64	12.54	14.29	12.87
12.66	10.79	10.42	10.03	10.13	11.7	11.43	9.57	10.64	12.64	14.29	12.87
12.67	10.89	10.42	10.03	10.14	11.7	11.44	9.73	10.7	12.64	14.4	12.87
12.68	10.89	10.43	10.03	10.27	11.74	11.44	9.77	10.7	12.65	14.43	12.87
12.79	10.89	10.48	10.03	10.27	11.74	11.54	9.77	10.71	12.65	14.43	12.87
12.79	10.9	10.48	10.03	10.27	11.76	11.66	9.78	10.71	12.77	14.43	13.15
12.8	10.9	10.49	10.03	10.27	11.8	11.67	9.78	10.83	12.78	14.65	13.15
12.81	10.91	10.57	10.03	10.27	11.8	11.67	9.78	10.84	12.78	14.65	13.15
12.82	10.91	10.57	10.03	10.27	11.91	11.67	9.79	10.85	12.78	14.65	13.15
12.96	10.91	10.75	10.03	10.46	12.05	11.79	9.85	10.85	12.78	14.65	13.15
									12.85	14.8	13.2
									12.95	14.8	13.2
									12.95	14.8	13.2

12	3	3	4	4	5	7	7	8	10	10	4	4	5	8	7	7	10	10	12	12	12
Box-6	Box-1	Box-2	Box-6	Box-7	Box-6	Box-2	Box-5	Box-3	Box-1	Box-3	Box-1	Box-4	Box-5	Box-1	Box-6	Box-8	Box-7	Box-2	Box-3	Box-4	Box-7
83	1.7	2.3	25.3	26.6	34.6	38	43.6	52.6	64	75	13.3	21.5	32.6	44.6	45	47	65	68	77	78	84
F	F	F	F	F	F	F	F	F	F	F	F	F	F	F	F	F	F	F	F	F	F
H2O	Malto	Malto	Malto	Malto	Malto	Malto	Malto	Malto	Malto	Malto	MCT	MCT	MCT	MCT	MCT	MCT	MCT	MCT	MCT	MCT	MCT
FH	FM	FM	FM	FM	FM	FM	FM	FM	FM	FM	FT	FT	FT	FT	FT	FT	FT	FT	FT	FT	FT
0	0	0	0	0	0	0		0	0	0	0	0	0	0	0	0	0	0	0	0	0
0	0	0.09	0	0.02	0	0		0.03	0.01	0	0.06	0.01	0	0.08	0	0.02	0	0	0	0	0
0	0	0.09	0	0.02	0	0.07		0.03	0.01	0	0.19	0.02	0	0.23	0	0.03	0	0	0	0	0
0.01	0	0.09	0	0.03	0	0.09		0.03	0.02	0	0.26	0.02	0.01	0.23	0	0.03	0.01	0.01	0	0	0
0.01	0	0.09	0	0.03	0	0.09		0.03	0.02	0.01	0.26	0.02	0.02	0.4	0	0.03	0.03	0.02	0	0	0
0.02	0.01	0.09	0	0.04	0	0.15		0.04	0.02	0.02	0.35	0.03	0.02	0.4	0	0.04	0.03	0.03	0	0	0
0.02	0.16	0.09	0	0.06	0.16	0.15		0.07	0.03	0.02	0.46	0.05	0.02	0.4	0	0.04	0.03	0.03	0	0.01	0
0.03	0.33	0.33	0	0.06	0.23	0.15		0.07	0.03	0.02	0.54	0.05	0.02	0.45	0	0.04	0.03	0.03	0	0.01	0
0.03	0.49	0.94	0	0.06	0.32	0.15		0.08	0.03	0.03	0.54	0.06	0.02	0.45	0	0.04	0.21	0.03	0	0.01	0
0.03	0.49	1.18	0	0.06	0.32	0.15		0.08	0.04	0.03	0.54	0.07	0.02	0.62	0	0.05	0.32	0.03	0	0.01	0
0.03	0.49	1.54	0	0.06	0.58	0.15		0.1	0.04	0.04	0.58	0.07	0.03	0.81	0	0.05	0.34	0.03	0	0.01	0
0.03	0.58	1.72	0	0.06	0.98	0.15		0.1	0.05	0.04	0.64	0.07	0.03	0.98	0	0.05	0.34	0.03	0	0.29	0
0.03	0.7	1.72	0	0.06	1.19	0.25		0.1	0.32	0.04	0.77	0.07	0.03	1.29	0	0.06	0.52	0.03	0	0.29	0
0.03	0.87	1.9	0	0.07	1.3	0.59		0.1	1.23	0.04	0.84	0.07	0.03	1.41	0.06	0.06	0.7	0.03	0	0.29	0
0.03	0.88	2.07	0	0.07	1.32	0.67		0.1	1.47	0.04	0.88	0.07	0.04	1.66	0.2	0.07	0.73	0.03	0	0.36	0
0.03	0.88	2.08	0	0.07	1.32	0.67		0.1	1.62	0.68	0.88	0.07	0.33	1.88	0.37	0.07	0.95	0.03	0	0.71	0
0.05	0.89	2.14	0	0.49	1.32	2.16		0.1	1.62	0.97	0.88	0.07	0.33	1.91	0.49	0.08	1.15	0.03	0	1.24	0
0.43	0.89	2.3	0.31	0.97	1.32	2.17		0.1	1.62	1.19	0.88	0.28	0.69	1.91	0.59	0.08	1.15	0.03	0	1.37	0
0.7	1.08	2.32	0.38	1.24	1.59	2.17		0.17	1.71	1.45	0.88	0.45	0.76	1.93	0.62	0.08	1.15	0.04	0	1.65	0
1.37	1.31	2.86	0.38	1.26	1.6	2.17		0.17	2.25	1.78	0.88	0.75	1.31	2.09	0.62	0.08	1.15	0.05	0	1.83	0
1.46	1.31	3.13	0.38	1.43	1.72	2.17		0.17	2.46	2.24	0.88	1.13	1.32	2.19	0.62	0.08	1.48	0.05	0	2.22	0
1.64	1.47	3.22	0.49	1.44	1.96	2.17		0.17	2.7	2.25	0.88	1.33	1.32	2.21	0.62	0.58	1.62	0.05	0	2.34	0
1.97	1.51	3.35	0.73	1.44	2.28	2.17		0.17	2.71	2.25	0.88	1.33	1.32	2.32	0.62	0.58	1.76	0.06	0	2.34	0
2.3	1.99	3.35	1.03	1.48	2.32	2.17		0.17	2.71	2.25	0.88	1.33	1.32	2.53	0.62	0.93	1.95	0.07	0	2.34	0
2.3	2.27	3.4	1.04	1.67	2.34	2.17		0.17	2.71	2.25	0.88	1.34	1.84	2.61	0.62	1.47	1.96	0.07	0	2.34	0
2.3	2.43	3.4	1.04	1.67	2.34	2.17		0.17	2.72	2.25	0.88	1.35	2.32	2.75	1.12	1.56	1.97	0.07	0	2.34	0
2.3	2.58	3.64	1.04	1.67	2.48	2.17		0.75	3.09	2.25	0.88	1.35	2.38	2.97	1.15	1.56	1.98	0.09	0	2.34	0.37
2.3	2.58	3.64	1.05	2.04	2.48	2.17		0.98	3.09	2.61	0.88	1.57	2.39	3.02	1.28	1.56	1.98	0.1	0	2.34	0.52
2.3	2.59	3.64	1.05	2.24	2.48	2.17		1.2	3.09	2.87	0.88	1.77	2.39	3.02	1.28	1.56	1.98	0.11	0	2.51	0.53
2.3	2.59	3.72	1.44	2.24	2.49	2.17		1.55	3.1	2.87	0.88	1.85	2.39	3.07	1.28	1.56	2.17	0.11	0	2.87	0.54
2.3	2.6	3.91	1.45	2.26	2.5	2.17		1.55	3.22	2.87	0.88	1.85	2.39	3.18	1.28	1.56	2.17	0.13	0	3.34	0.54
2.6	2.6	4.02	1.45	2.51	2.5	2.17		1.55	3.22	2.88	0.88	1.86	2.39	3.18	1.28	1.71	2.17	0.13	0	3.34	0.55
2.95	2.68	4.02	1.45	2.64	2.68	2.17		1.55	3.23	2.88	0.88	1.87	2.45	3.18	1.3	1.71	2.37	0.14	0	3.34	0.56
3.28	2.7	4.02	1.46	2.64	2.7	2.17		1.55	3.23	3.09	0.88	1.87	2.53	3.3	1.3	1.71	2.51	0.14	0	3.35	0.56
3.29	2.7	4.11	1.93	2.73	2.7	2.17		1.55	3.23	3.17	0.88	2.14	2.82	3.3	1.3	1.71	2.63	0.15	0	3.35	0.56
3.29	2.7	4.35	1.93	2.74	2.71	2.17		1.55	3.23	3.17	0.88	2.45	2.82	3.3	1.3	1.74	2.64	0.15	0	3.35	0.56
3.29	2.71	4.41	1.93	2.74	3.01	2.41		1.55	3.23	3.17	0.88	2.45	2.82	3.3	1.67	1.99	2.66	0.16	0	3.35	0.56
3.29	2.71	4.41	1.94	2.76	3.16	2.41		1.55	3.24	3.17	0.88	2.46	3.03	3.66	1.83	2.08	2.67	0.17	0	3.35	0.56
3.44	2.71	4.41	2.18	2.76	3.16	2.41		1.55	3.24	3.17	0.88	2.46	3.2	3.87	1.98	2.09	2.68	0.17	0	3.35	0.56
3.6	2.72	4.41	2.19	2.78	3.41	2.41		1.55	3.24	3.25	0.88	2.46	3.2	3.87	1.98	2.09	2.9	0.17	0	3.57	0.56
3.6	2.73	4.41	2.19	3.12	3.42	2.41		1.55	3.25	3.63	0.88	2.68	3.2	3.87	1.98	2.09	2.92	0.17	0	4.02	0.56
3.61	2.73	4.46	2.19	3.13	3.44	2.54		1.55	3.25	3.85	0.88	2.82	3.2	3.87	1.98	2.09	2.92	0.17	0	4.02	0.58
3.61	2.73	4.46	2.19	3.26	3.44	2.8		1.55	3.25	3.85	0.88	2.82	3.21	3.87	2.02	2.09	2.92	0.18	0	4.02	0.58
3.77	2.74	4.46	2.2	3.26	3.44	2.94		1.55	3.26	3.85	0.88	2.82	3.21	3.87	2.31	2.09	2.94	0.19	0	4.02	0.58
3.78	2.74	4.51	2.53	3.28	3.44	2.94		1.55	3.26	3.85	0.88	2.83	3.21	4	2.31	2.09	2.95	0.19	0	4.02	0.58
3.78	2.74	4.51	2.73	3.3	3.69	2.94		1.55	3.26	3.85	0.88	2.83	3.38	4	2.31	2.3	2.96	0.19	0	4.02	0.58
3.78	2.74	4.51	2.74	3.3	3.73	3		1.55	3.26	3.85	0.88	2.84	3.39	4	2.31	2.76	2.96	0.19	0	4.03	0.59
3.78	2.75	4.51	2.74	3.33	3.73	3		1.55	3.26	3.86	0.88	2.92	3.39	4.14	2.31	2.98	3.25	0.19	0	4.03	0.59
3.78	2.75	4.52	2.74	3.67	3.73	3		1.55	3.26	3.86	0.88	2.92	3.39	4.16	2.31	2.98	3.25	0.19	0	4.03	0.59
3.78	2.76	4.52	2.75	3.71	3.73	3		1.55	3.26	4.26	0.88	2.93	3.4	4.2	2.37	2.98	3.26	0.2	0	4.03	0.63
3.78	2.76	4.57	3.09	3.71	3.92	3		1.55	3.26	4.26	0.88	2.93	3.4	4.35	2.4	2.98	3.26	0.2	0	4.03	0.63
3.88	2.76	4.63	3.09	3.77	3.92	5		1.55	3.26	4.26	0.88	2.94	3.45	4.35	2.4	2.98	3.27	0.21	0	4.42	0.65
3.9	2.76	4.63	3.1	3.77	3.92	5.07		1.55	3.27	4.27	0.88	2.94	3.77	4.35	2.4	2.98	3.28	0.22	0	4.58	0.66
3.9	2.77	4.63	3.1	4.01	3.92	5.07		1.56	3.27	4.5	0.88	2.95	3.77	4.66	2.41	2.98	3.28	0.22	0	4.58	0.66
3.9	2.77	4.63	3.1	4.04	3.92	5.07		1.6	3.28	4.83	0.88	2.95	3.77	4.82	2.89	3.17	3.48	0.23	0	4.58	0.97
3.9	2.77	4.68	3.1	4.04	3.92	5.07		1.6	3.28	4.94	0.88	2.97	3.78	4.85	2.89	3.17	3.48	0.23	0	4.58	0.97
3.9	2.78	4.68	3.38	4.05	3.92	5.07		1.6	3.28	4.95	0.88	2.97	3.88	4.85	2.89	3.17	3.48	0.23	0	4.58	1.02
3.9	2.78	4.68	3.52	4.05	4.07	5.07		1.6	3.28	4.95	0.88	2.97	4	4.85	2.89	3.17	3.48	0.24	0	4.59	1.03
3.9	2.79	4.68	3.53	4.47	4.1	5.07		1.65	3.28	4.95	0.88	3.03	4	4.85	2.89	3.19	3.7	0.25	0	4.59	1.04
3.9	2.79	4.68	3.53	4.48	4.13	5.07		1.65	3.29	5.13	0.88	3.11	4	4.85	2.89	3.38	3.77	0.25	0	4.59	1.04
3.9	2.79	4.68	3.53	4.5	4.13	5.07		1.65	3.29	5.31	0.88	3.11	4.01	4.85	3.56	3.46	3.78	0.26	0	4.59	1.05
3.9	3.31	4.68	3.7	4.5	4.26	5.07		1.89	3.29	5.31	0.88	3.22	4.01	5.05	3.56	3.7	3.8	0.26	0	4.59	1.95
3.9	3.67	4.85	3.7	4.5	4.26	5.07		1.89	3.29	5.32	0.88	3.23	4.01	5.18	3.56	3.7	3.88	0.26	0.06	4.59	1.95
3.9	3.67	4.99	3.71	4.67	4.26	5.07		1.89	3.29	5.32	0.88	3.23	4.23	5.32	3.56	3.7	3.88	0.27	0.06	4.6	1.95
3.9	3.67	4.99	3.71	4.67	4.26	5.07		1.98	3.3	5.69	0.88	3.23	4.24	5.36	3.62	3.7	3.89	0.27	0.06	4.85	1.96
3.9	3.67	4.99	3.71	4.67	4.26	5.07		1.98	3.3	5.69	0.88	3.24	4.24	5.36	3.62	3.7	3.89	0.27	0.06	5.07	1.96
3.91	3.68	4.99	3.85	4.87	4.28	5.08		1.98	3.3	5.7	0.88	3.24	4.25	5.43	3.62	3.81	4.01	0.27	0.06	5.08	1.97
4.34	3.69	5.23	4.02	4.88	4.45	5.08		2.23	3.3	5.7	1.52	3.48	4.3	5.52	3.71	3.82	4.08	0.27	0.06	5.08	1.97
4.34	3.69	5.4	4.09	4.92	4.65	5.08		2.34	4.06	6.06	1.95	3.48	4.3	5.55	3.73	3.82	4.08	0.28	0.06	5.27	2.15
4.35	3.8	5.4	4.1	4.97	4.74	5.08		2.36	4.06	6.07	2.05	3.71	4.55	5.63	4.23	3.82	4.16	0.28	0.06	5.4	2.63
4.59	4.03	5.44	4.36	5.12	4.88	5.08		2.66	4.2	6.07	2.51	3.71	4.7	5.68	4.37	3.93	4.18	0.28	0.07	5.47	2.88
4.59	4.21	5.46	4.42	5.22	4.91	5.08		2.76	4.37	6.41	2.99	3.72	4.7	5.81	4.37	4.14	4.18	0.28	0.07	5.62	3.08
4.59	4.22	5.61	4.5	5.47	4.97	5.08		3.07	4.37	6.42	2.99	3.97	4.85	5.98	4.37	4.17	4.21	0.28	0.07	5.7	3.32
4.81	4.4	5.77	4.79	5.47	5.07	5.08		3.42	4.38	6.42	3.11	4.08	5.13	6.03	4.4	4.23	4.33	0.29	0.07	5.89	3.57
4.95	4.51	5.92	4.83	5.48	5.1	5.08		3.51	4.52	6.72	4.38	4.23	5.14	6.03	4.54	4.4	4.33	0.3	0.09	6.08	3.57
4.95	4.51	6.05	4.96	5.68	5.12	5.08		3.89	4.84	6.86	4.38	4.23	5.14	6.33	4.73	4.4	4.33	0.3	0.09	6.11	3.76
5.55	4.63	6.14	5.02	5.87	5.12	5.08		4.08	4.92	6.87	4.38	4.23	5.14	6.33	4.81	4.4	4.43	0.3	0.11	6.11	4.01
5.79	4.93	6.35	5.18	5.92	5.3	5.08		4.32	4.93	7.11	4.38	4.55	5.58	6.59	4.9	4.5	4.43	0.3	0.11	6.11	4.21
5.92	4.93	6.41	5.26	6.04	5.36	5.08		4.5	4.93	7.24	4.38	4.56	5.71	6.83	4.9	4.91	4.74	0.3	0.11	6.32	4.35
5.92	5.14	6.45	5.29	6.28	5.47	5.41		4.63	5.24	7.25	4.38	4.56	5.9	7.03	4.9	4.96	4.77	0.31	1.12	6.32	4.51
5.92	5.14	6.49	5.29	6.34	5.6	5.68		4.73	5.54	7.25	4.38	4.83	5.91	7.28	4.9	5.23	4.78	0.31	1.95	6.33	4.51
5.93	5.15	6.54	5.56	6.34	5.6	5.68		4.73	5.86	7.42	4.38	4.83	6.14	7.44	4.9	5.45	4.78	0.31	2.51	6.42	4.51
6.06	5.15	6.78	5.64	6.34	5.6	5.68		5.07	6.09	7.63	4.55	4.83	6.15	7.63	4.91	5.56	4.78	0.32	2.78	6.71	4.51
6.26	5.51	6.96	5.64	6.74	5.7	5.68		5.07	6.23	7.72	4.89	5.06	6.18	7.84	5.2	5.56	4.78	0.33	3.19	6.93	4.65
6.27	5.77	7	5.64	6.92	5.78	8.91		5.07	6.35	7.72	4.96	5.06	6.18	7.86	5.2	5.56	4.79	0.34	3.63	7.12	4.65
6.5	5.77	7.11	5.64	6.96	6.01	8.95		5.07	6.42	8.28	5.06	5.06	6.18	7.86	5.3	5.56	5.11	0.35	3.68	7.3	4.82
6.72	5.78	7.22	5.64	6.96	6.14	8.96		5.07	6.67	8.35	5.06	5.31	6.18	7.89	5.36	5.8	5.48	0.35	3.79	7.3	4.99
6.9	5.78	7.33	5.64	7.07	6.26	8.96		5.07	6.68	8.35	5.11	5.47	6.18	8.05	5.76	6	5.56	0.35	4.06	7.5	5.04
7.13	5.78	7.42	5.64	7.45	6.26	8.96		5.07	6.68	8.35	5.12	5.66	6.19	8.05	5.93	6	5.56	0.36	4.34	7.5	5.22
7.28	5.86	7.69	5.64	7.46	6.26	8.96		5.34	6.68	8.35	5.12	5.76	6.31	8.12	5.93	6	5.57	0.36	4.34	7.5	5.22
7.28	6.1	7.73	5.64	7.47	6.5	8.96		5.48	6.69	8.35	5.57	5.84	6.47	8.19	5.93	6	5.58	0.36	4.41	7.5	5.22
7.32	6.38	7.73	5.65	7.48	6.5	8.96		5.48	6.69	8.35	5.57	5.98	6.76	8.19	5.93	6.25	5.71	0.37	4.41	7.5	5.23
7.32	6.48	7.73	5.65	7.48	6.51	8.96		5.48	6.7	8.35	5.57	5.98	6.76	8.33	5.93	6.25	5.76	0.37	4.41	7.61	5.23
7.49	6.53	7.73	5.92	7.49	6.62	8.96		5.48	6.7	8.35	5.57	5.98	6.76	8.83	5.96	6.55	5.77	0.38	4.62	7.79	5.23
7.49	6.85	7.73	6.14	7.49	6.72	8.96		5.48	6.7	8.35	5.57	5.98	6.76	8.87	6.06	6.77	5.77	0.38	4.93	7.79	5.34
7.49	6.91	8.01	6.15	7.49	6.72	8.96		5.63	7	8.67	5.57	5.99	7.06	8.87	6.37	7	5.78	0.39	5.1	7.8	5.56
7.49	6.91	8.05	6.18	7.49	6.91	8.96		6.05	7.17	8.83	5.57	6	7.06	8.87	6.46	7.08	6.13	0.4	5.1	7.95	5.71
7.49	6.91	8.33	6.18	8.07	6.96	8.96		6.05	7.18	8.83	5.76	6	7.07	8.96	6.46	7.08	6.4	0.41	5.1	8.02	5.78
7.49	6.91	8.44	6.19	8.13	6.96	8.96		6.05	7.18	8.84	5.91	6	7.07	9	6.46	7.08	6.46	0.41	5.49	8.03	5.78
7.49	6.91	8.44	6.57	8.16	6.97	8.96		6.06	7.18	8.84	5.91	6.32	7.07	9	6.46	7.08	6.46	0.41	5.91	8.03	5.78
7.49	6.91	8.44	6.64	8.48	7.04	8.96		6.06	7.18	8.84	5.91	6.32	7.37	9.15	6.46	7.08	6.64	0.42	6.33	8.3	5.78
7.49	6.91	8.44	6.64	8.51	7.04	8.96		6.06	7.32	8.84	5.91	6.33	7.37	9.49	6.76	7.08	6.64	0.42	6.33	8.31	5.78
7.49	6.91	8.44	6.65	8.51	7.04	8.96		6.06	7.33	9.33	5.91	6.73	7.43	9.52	6.85	7.16	6.64	0.43	6.33	8.31	5.78
7.49	6.91	8.44	6.65	8.53	7.07	8.96		6.06	7.33	9.41	5.91	6.73	7.43	9.52	6.85	7.61	6.65	0.44	6.33	8.31	5.78
7.49	6.92	8.44	6.97	8.53	7.07	8.96		6.06	7.33	9.42	5.91	6.73	7.44	9.52	6.85	7.61	6.65	0.44	6.33	8.57	5.79
7.49	6.92	8.44	7.11	8.54	7.33	8.96		6.06	7.33	9.42	5.91	6.73	7.75	9.52	6.87	7.61	6.89	0.45	6.33	8.57	5.79
7.77	6.92	8.47	7.11	8.81	7.38	8.96		6.06	7.33	9.42	5.91	6.81	7.87	9.52	6.87	7.61	6.89	0.45	6.33	8.57	5.79
7.77	6.95	8.47	7.11	8.86	7.39	8.96		6.06	7.51	9.57	6.5	6.81	7.87	9.52	6.87	7.79	6.91	0.46	6.33	8.57	5.8
7.77	6.95	8.5	7.11	8.88	7.4	8.96		6.09	7.51	9.57	6.5	6.81	7.87	9.57	6.99	7.79	6.93	0.46	6.35	8.57	5.8
7.77	6.95	8.57	7.33	8.98	7.58	8.96		6.09	7.52	9.57	6.5	6.81	7.87	9.58	6.99	7.79	7.16	0.46	6.35	8.92	5.8
7.77	6.95	8.57	7.33	9.28	7.59	8.96		6.09	7.54	9.57	6.5	6.91	8.04	9.61	6.99	7.79	7.26	0.52	6.37	9.01	5.88
7.77	6.96	8.57	7.33	9.34	7.59	8.96		6.09	7.54	9.57	6.5	6.91	8.04	9.64	7.02	7.94	7.26	0.52	6.51	9.02	5.88
7.77	7.1	8.66	7.33	9.34	7.59	8.96		6.09	7.54	9.57	6.68	6.92	8.04	9.64	7.25	7.96	7.26	1.14	6.54	9.02	5.88
7.77	7.12	8.66	7.33	9.34	7.59	8.96		6.09	7.54	9.9	6.79	6.92	8.04	9.64	7.25	7.96	7.26	1.64	6.54	9.26	5.88
7.77	7.12	8.76	7.34	9.34	7.59	8.96		6.09	7.54	9.9	6.8	6.92	8.43	9.67	7.25	7.96	7.26	1.92	6.54	9.26	5.89
7.77	7.12	8.76	7.44	9.62	7.76	8.96		6.09	7.54	9.9	6.8	6.92	8.43	9.75	7.25	7.96	7.54	1.93	6.9	9.26	5.9
7.77	7.15	8.76	7.44	9.65	7.77	8.96		6.09	7.54	10.02	6.8	6.93	8.44	9.75	7.85	8.19	7.54	1.96	6.9	9.26	5.9
7.77	7.2	8.76	7.44	9.66		8.96		6.09	7.54	10.03	7.5	6.93	8.44		8.04	8.2	7.54	2.15	6.9	9.64	5.9
7.77	7.2	8.76	7.44	9.66		8.96		6.28	7.54	10.03	7.5	6.99	8.44		8.04	8.2	7.55	2.5	6.9	9.65	5.9
7.77	7.2	8.76	7.45	9.66		8.96		6.28	7.54	10.03	7.5	7	8.44		8.04	8.2	7.55	2.67	6.9	9.65	5.9
7.78	7.2	8.76	7.45	9.71		8.96		6.28	7.54	10.04	7.5	7	8.44		8.04	8.4	7.55	2.67	6.9	9.65	5.9
7.78	7.21	8.88	7.71	10		8.96		6.28	7.54	10.36	7.5	7.01	8.44		8.04	8.69	7.55	2.68	6.9	9.74	5.9
7.78	7.21	9.07	7.8	10.11		8.96		6.28	7.54	10.36	7.5	7.01	8.58		8.04	8.7	7.87	2.68	6.9	9.89	5.92
7.78	7.21	9.11	8.1	10.14		8.96		6.28	7.55	10.36	7.5	7.17	8.58		8.06	8.7	7.87	2.68	7.43	9.89	6.23
7.78	7.54	9.11	8.11	10.16		10.55		6.28	7.55	10.37	7.5	7.18	8.58		8.08	8.7	7.88	2.89	7.44	9.89	6.24

7.79	7.54	9.11	8.11	10.16		10.55	6.62	7.55	10.38	7.5	7.18	8.59		8.08	8.78	7.89	2.98	7.44	9.89	6.24
7.79	7.54	9.11	8.11	10.16		10.55	6.62	7.55	10.38	7.5	7.32	8.59		8.08	8.79	7.89	2.99	7.44	9.89	6.24
7.98	7.55	9.35	8.11	10.16		10.55	6.62	7.55	10.38	7.5	7.46	8.8		8.13	8.79	8.26	2.99	7.44	10.05	6.47
7.98	7.84	9.35	8.11	10.24		10.55	6.62	7.55	10.74	7.5	7.47	8.8		8.2	8.89	8.28	2.99	7.54	10.17	6.47
7.98	7.84	9.37	8.11	10.43		10.55	6.62	7.55	10.74	7.5	7.47	8.8		8.2	8.89	8.28	2.99	7.65	10.31	6.49
7.98	7.85	9.38	8.18	10.57		10.55	6.77	7.97	10.75	7.5	7.47	8.8		8.32	8.96	8.28	3.28	7.65	10.32	6.49
7.99	7.99	9.53	8.24	10.57		10.55	6.77	7.98	10.75	7.67	7.65	8.8		8.64	9.02	8.28	3.54	7.89	10.32	6.62
8.11	7.99	9.62	8.27	10.6		10.55	6.77	7.98	10.75	7.67	7.73	8.87		8.77	9.12	8.48	3.55	7.93	10.32	6.62
8.12	8	9.62	8.27	10.61		10.55	6.77	7.98	11.03	7.94	7.73	8.98		8.77	9.12	8.49	3.59	7.96	10.49	6.62
8.12	8	9.74	8.48	10.75		10.55	7.06	8.21	11.17	8.46	7.87	9.02		8.79	9.12	8.49	3.6	8.13	10.49	6.72
8.4	8.01	9.74	8.48	10.84		10.55	7.14	8.21	11.17	8.46	7.93	9.02		8.85	9.29	8.5	3.73	8.14	10.53	7.07
8.73	8.42	9.94	8.49	10.85		10.55	7.4	8.21	11.56	8.61	8.14	9.18		9.21	9.3	8.51	3.92	8.33	10.53	7.2
9.23	8.62	10.04	8.77	11.11		10.55	7.4	8.21	11.58	8.79	8.39	9.33		9.21	9.3	8.54	4.07	8.33	10.7	7.39
9.28	8.79	10.23	8.78	11.41		10.55	7.6	8.4	11.58	9.03	8.39	9.33		9.21	9.36	8.68	4.08	8.61	10.82	7.59
9.62	8.85	10.35	8.83	11.41		10.55	7.89	8.41	12.04	9.15	8.46	9.38		9.21	9.36	8.68	4.22	8.94	10.96	7.93
9.7	8.85	10.35	8.9	11.66		10.55	7.89	8.41	12.18	9.34	8.46	9.54		9.47	9.57	8.73	4.22	9.04	10.96	8.11
9.94	8.95	10.35	8.9	11.68		10.55	8.16	8.74	12.18	9.41	8.88	9.68		10.24	9.6	9.1	4.23	9.04	10.97	8.23
9.95	9.18	10.35	8.9	11.7		10.55	8.45	8.74	12.19	9.42	8.95	9.78		10.24	9.73	9.34	4.23	9.36	11.12	8.43
9.95	9.4	10.6	8.9	11.7		10.55	8.45	8.74	12.63	9.43	9.2	9.86		10.24	9.82	9.34	4.41	9.36	11.13	8.62
10.01	9.4	10.8	9.27	11.92		10.55	8.59	8.96	12.69	9.52	9.21	9.86		10.24	9.82	9.44	4.52	9.36	11.22	8.72
10.01	9.41	10.85	9.39	12.02		11.56	8.78	9.09	12.83	9.52	9.25	9.86		10.4	9.86	9.73	4.7	9.57	11.33	8.78
10.01	9.47	10.86	9.39	12.04		11.56	8.83	9.1	12.84	9.53	9.26	9.86		10.47	10.11	9.81	4.78	10.05	11.54	8.78
10.27	9.47	11.13	9.47	12.2		11.56	9.32	9.1	12.95	9.69	9.26	9.87		10.51	10.12	9.82	4.83	10.3	11.63	8.78
10.61	9.48	11.16	9.47	12.24		11.56	9.32	9.51	13.34	9.85	9.56	10.03		10.51	10.13	9.82	4.92	10.3	11.84	8.79
10.73	9.53	11.16	9.47	12.24		11.56	9.4	9.7	13.39	10.15	9.56	10.34		10.51	10.13	9.83	4.93	10.3	11.98	8.82
10.99	9.78	11.2	9.47	12.24		11.56	9.47	9.71	13.4	10.19	9.57	10.4		10.51	10.13	9.83	5.03	10.3	12.07	8.86
11.08	10.07	11.33	9.72	12.25		11.56	9.47	9.98	13.4	10.19	9.57	10.56		10.92	10.33	9.84	5.04	10.3	12.07	9.2
11.29	10.16	11.44	9.79	12.3		11.56	9.58	10.13	13.4	10.19	9.57	10.57		10.97	10.51	9.84	5.05	10.3	12.07	9.47
11.39	10.28	11.44	10.2	12.3		11.56	9.58	10.14	13.4	10.19	9.58	10.57		11.03	10.51	9.85	5.06	10.79	12.07	9.6
11.39	10.46	11.44	10.2	12.67		11.56	9.58	10.14	13.4	10.19	9.7	10.57		11.03	10.51	9.85	5.06	10.96	12.36	9.63
11.39	10.46	11.54	10.2	12.72		11.56	9.58	10.14	13.4	10.45	9.7	10.58		11.1	10.51	9.86	5.06	11.13	12.38	9.7
11.39	10.47	11.54	10.2	12.73		11.56	9.58	10.15	13.89	10.68	9.75	10.58		11.1	10.63	10.1	5.07	11.13	12.59	9.71
11.39	10.47	11.57	10.2	12.73		11.56	9.91	10.15	13.9	10.79	9.75	10.58		11.1	10.71	10.24	5.08	11.17	12.59	9.79
11.39	10.47	11.82	10.2	12.74		11.56	9.91	10.15	13.9	10.89	9.86	10.58		11.1	10.72	10.32	5.08	11.17	12.59	9.8
11.77	10.72	11.91	10.2	12.75		11.56	9.93	10.15	13.9	10.89	10.04	11		11.1	10.88	10.33	5.08	11.17	12.59	9.92
11.77	10.77	11.91	10.42	12.75		11.56	10.33	10.32	13.9	10.95	10.04	11		11.22	10.89	10.33	5.26	11.63	12.59	9.95
11.78	10.77	12.03	10.42	12.76		11.56	10.33	10.32	14.15	11.13	10.04	11.01		11.22	10.89	10.33	5.27	11.63	12.77	9.95
11.78	10.77	12.13	10.43	12.77		11.56	10.33	10.32	14.44	11.18	10.05	11.01		11.4	10.89	10.33	5.28	11.63	13.05	10.11
12.01	10.8	12.21	10.43	12.77		11.56	10.43	10.34	14.53	11.23	10.05	11.28		11.5	10.89	10.4	5.29	11.63	13.05	10.13
12.01	10.83	12.23	10.73	12.82		11.56	10.44	10.34	14.54	11.24	10.1	11.52		11.68	10.9	10.73	5.29	11.63	13.3	10.13
12.01	10.83	12.54	10.73	13.44		11.56	10.45	10.34	14.56	11.37	10.42	11.52		11.73	11.17	10.83	5.3	11.63	13.3	10.13
12.01	10.84	12.54	10.77	13.74		11.56	10.57	10.34	14.56	11.37	10.43	11.53		11.73	11.36	10.83	5.3	11.63	13.3	10.13
12.01	10.84	12.54	10.78	13.84		11.56	10.57	10.48	14.57	11.39	10.43	11.53		11.73	11.43	10.83	5.49	11.63	13.31	10.36
12.01	10.84	12.62	10.78	13.97		11.56	10.69	10.49	14.57	11.53	10.43	11.53		11.73	11.43	10.84	5.6	11.63	13.53	10.39
12.01	10.85	12.67	10.78	13.97		11.56	10.69	10.49	14.57	11.54	10.7	11.79		11.73	11.44	10.96	5.6	11.63	13.53	10.48
12.02	10.94	12.67	11.07	13.97		11.56	10.69	10.5	14.57	11.62	10.7	11.79		11.73	11.44	10.99	5.6	11.64	13.53	10.49
12.02	11.01	12.67	11.07	13.99		11.56	10.78	10.7	14.9	11.62	10.71	11.79		12.08	11.5	10.99	5.76	12.21	13.72	10.54
12.02	11.01	12.67	11.08	14.24		11.56	10.78	10.7	14.91	11.86	10.71	11.8		12.08	11.5	11	5.78	12.22	13.72	10.56
12.02	11.01	12.76	11.08	14.24		12.21	10.78	10.71	14.91	11.86	10.77	11.8		12.08	11.58	11	5.86	12.22	13.78	10.71
12.02	11.03	12.76	11.08	14.26		12.21	10.78	10.71	14.91	11.88	10.77	11.8		12.08	11.59	11.22	5.86	12.22	13.79	10.71
12.02	11.04	12.79	11.08	14.26		12.22	10.78	10.71	14.92	11.89	10.77	11.8		12.08	11.59	11.22	5.86	12.22	13.79	10.72
12.1	11.04	12.84	11.26	14.58		12.22	10.78	10.71	14.92	11.9	10.91	11.8		12.1	11.59	11.22	5.86	12.22	13.94	10.72
12.11	11.04	12.86	11.26	14.6		12.22	10.78	10.97	15.15	11.99	10.98	12.24		12.1	11.75	11.23	5.87	12.22	13.94	10.72
12.11	11.04	12.95	11.27	14.6		12.22	10.78	10.98	15.3	12.11	10.98	12.24		12.13	11.87	11.23	5.93	12.78	13.94	10.72
12.11	11.05	13.02	11.27	14.6		12.22	10.78	10.98	15.31	12.2	10.98	12.24		12.21	11.89	11.23	5.93	12.78	13.94	10.92

0	0	0	0	0	0	0	0	0	0.31	0	0	0	0	0	0	0.42	0.04	0	0	0
0	0.01	0	0	0.14	0.05	0.04	0	0	0.33	0	0.12	0.14	0.11	0.17	0	0.57	0.04	0	0.16	0
0	0.21	0.16	0.38	0.3	0.13	0.04	0	0	0.33	0	0.12	0.25	0.23	0.17	0	0.58	0.15	0	0.16	0.09
0.16	0.21	0.3	0.39	0.31	0.17	0.04	0	0	0.51	0	0.12	0.25	0.25	0.17	0	0.65	0.23	0	0.16	0.1
0.16	0.35	0.43	0.39	0.36	0.27	0.04	0	0.01	0.54	0	0.43	0.25	0.42	0.35	0.17	0.7	0.23	0	0.28	0.1
0.16	0.36	0.45	0.63	0.48	0.34	0.25	0	0.64	0.62	0	0.53	0.35	0.51	0.35	0.17	0.84	0.3	0	0.29	0.22
0.28	0.36	0.57	0.63	0.49	0.34	0.27	0	0.73	0.8	0	0.53	0.44	0.51	0.4	0.25	0.84	0.35	0	0.29	0.28
0.29	0.37	0.57	0.63	0.5	0.45	0.27	0	0.74	0.83	0	0.69	0.44	0.51	0.51	0.37	0.84	0.38	0	0.29	0.28
0.29	0.52	0.7	0.63	0.5	0.47	0.3	0	0.74	0.83	0	0.69	0.44	0.72	0.51	0.49	0.84	0.38	0	0.29	0.38
0.29	0.53	0.7	0.63	0.5	0.47	0.35	0	0.75	0.83	0	0.69	0.44	0.76	0.51	0.49	1.1	0.46	0	0.38	0.39
0.29	0.54	0.7	0.63	0.51	0.47	0.36	0	0.98	0.83	0	0.69	0.53	0.76	0.82	0.59	1.13	0.51	0	0.38	0.48
0.3	0.55	0.81	0.64	0.64	0.47	0.36	1.32	0.99	0.83	1.21	0.69	0.53	0.77	0.82	0.65	1.13	0.51	0	0.39	0.49
0.3	0.58	0.89	0.64	0.64	0.47	0.38	1.49	0.99	0.96	1.21	0.69	0.53	0.83	0.82	0.65	1.14	0.58	0	0.39	0.49
0.3	0.62	0.98	0.66	0.64	0.61	0.39	1.49	0.99	0.96	1.33	0.7	0.56	0.96	0.82	0.65	1.14	0.58	0	0.44	0.5
0.31	0.68	0.98	0.67	0.76	0.63	0.48	1.57	0.99	0.96	1.42	0.84	0.56	0.96	0.82	0.65	1.19	0.58	0	0.51	0.51
0.45	0.82	0.98	0.67	0.87	0.63	0.48	1.57	1.07	0.96	1.51	0.84	0.7	0.99	0.82	0.73	1.23	0.58	0	0.51	0.55
0.46	0.89	1.04	1.03	0.87	0.72	0.48	1.57	1.24	1.14	1.7	1.11	0.81	1	0.82	0.88	1.25	0.63	0	0.52	0.66
0.63	0.97	1.08	1.04	0.88	0.84	0.78	1.67	1.24	1.25	1.76	1.13	0.92	1	0.82	0.88	1.38	0.63	0.28	0.68	0.7
0.63	1.08	1.08	1.1	1.11	0.9	0.8	1.74	1.25	1.29	1.85	1.18	0.98	1.02	0.82	0.88	1.59	0.63	0.28	0.68	0.72
0.74	1.09	1.34	1.1	1.18	0.94	0.8	2.02	1.25	1.48	1.99	1.18	1.02	1.02	0.84	1.08	1.66	0.69	0.28	0.68	0.78
0.86	1.19	1.58	1.33	1.18	0.95	0.83	2.04	1.34	1.55	2.02	1.18	1.11	1.38	0.89	1.08	1.67	0.69	0.3	0.8	0.78
1.01	1.19	1.58	1.38	1.22	1.14	0.86	2.18	1.35	1.62	2.13	1.18	1.2	1.38	0.97	1.08	1.68	0.75	0.45	0.87	0.79
1.09	1.2	1.58	1.39	1.22	1.17	0.91	2.18	1.47	1.62	2.16	1.23	1.2	1.39	1.08	1.08	1.94	0.89	0.51	0.88	0.87
1.22	1.2	1.58	1.53	1.23	1.17	0.91	2.27	1.47	1.62	2.4	1.26	1.2	1.44	1.08	1.11	1.94	0.92	0.51	0.93	0.88
1.23	1.2	1.65	1.71	1.23	1.18	1.11	2.27	1.47	1.62	2.49	1.26	1.44	1.53	1.09	1.11	1.94	0.98	0.91	0.99	1.04
1.37	1.35	1.66	1.71	1.4	1.21	1.21	2.27	1.48	1.67	2.52	1.47	1.44	1.55	1.18	1.19	1.94	0.99	0.91	1.1	1.1
1.43	1.43	1.66	1.96	1.4	1.6	1.27	2.3	1.56	1.67	2.66	1.73	1.44	1.59	1.2	1.22	1.94	1.02	0.91	1.11	1.11
1.52	1.48	1.66	2.03	1.4	1.6	1.29	2.55	1.56	1.79	2.9	1.73	1.64	1.77	1.2	1.39	2.04	1.05	0.98	1.11	1.31
1.57	1.48	1.68	2.03	1.41	1.6	1.52	2.55	1.7	1.95	2.94	1.73	1.76	1.83	1.4	1.39	2.14	1.08	1.83	1.11	1.48
1.57	1.65	1.93	2.2	1.41	1.86	1.64	2.59	1.7	2.2	2.94	1.73	1.76	1.83	1.43	1.41	2.3	1.12	1.83	1.12	1.62
1.57	1.73	1.93	2.21	1.61	1.86	1.69	2.91	1.75	2.2	3.14	1.73	1.77	1.93	1.43	1.6	2.38	1.12	1.83	1.12	1.7
1.57	1.82	1.93	2.22	1.73	1.86	1.88	2.91	1.75	2.46	3.15	1.73	1.77	2.06	1.45	1.6	2.61	1.14	1.89	1.12	1.88
1.77	1.82	1.94	2.22	1.74	1.86	1.89	2.91	1.81	2.67	3.15	1.73	1.77	2.16	1.46	1.63	2.61	1.5	1.89	1.32	1.88
1.77	1.93	2.12	2.4	1.74	1.86	1.93	2.91	1.82	3	3.28	1.75	1.77	2.16	1.48	1.89	2.64	1.5	1.91	1.62	1.94
2	1.93	2.12	2.72	1.74	1.9	1.93	2.91	1.83	3	3.28	1.75	1.77	2.23	1.48	1.89	2.64	1.5	2.1	1.72	1.95
2	2	2.3	2.72	1.74	1.9	1.93	3.16	1.83	3	3.29	1.87	1.77	2.32	1.48	1.94	2.64	1.5	2.1	1.73	1.95
2.06	2.12	2.3	2.72	1.92	1.91	1.93	3.16	1.9	3.03	3.32	1.87	1.77	2.4	1.48	1.97	2.68	1.5	2.46	1.73	2.15
2.35	2.21	2.3	2.72	1.92	2.1	2.01	3.32	1.9	3.07	3.41	1.87	1.77	2.4	1.49	2.04	2.79	1.5	2.46	1.73	2.15
2.36	2.22	2.34	2.88	1.93	2.18	2.21	3.56	2	3.11	3.54	2.02	2.2	2.45	1.49	2.08	2.94	1.5	2.46	1.74	2.15
2.36	2.28	2.34	2.94	1.93	2.25	2.21	3.56	2.08	3.18	3.65	2.1	2.21	2.45	1.67	2.08	2.94	1.5	2.46	1.92	2.16
2.36	2.34	2.36	2.94	2.04	2.25	2.21	3.56	2.09	3.18	3.69	2.1	2.35	2.71	1.81	2.11	2.94	1.5	2.56	2.01	2.32
2.36	2.38	2.42	2.94	2.05	2.41	2.39	3.56	2.25	3.22	3.69	2.14	2.38	2.72	1.9	2.11	2.99	1.5	2.56	2.01	2.32
2.64	2.38	2.73	3.03	2.05	2.41	2.51	3.56	2.25	3.22	3.71	2.14	2.43	2.72	2.07	2.19	3	1.5	2.56	2.01	2.39
2.72	2.38	2.73	3.03	2.06	2.48	2.59	3.56	2.25	3.22	3.82	2.42	2.47	2.72	2.07	2.46	3.06	1.5	2.56	2.14	2.48
2.72	2.45	2.73	3.14	2.37	2.48	2.68	3.56	2.26	3.56	3.91	2.6	2.72	2.85	2.07	2.46	3.09	1.5	2.56	2.14	2.61
2.73	2.59	2.73	3.26	2.41	2.48	2.68	3.56	2.26	3.56	3.93	2.6	2.72	2.95	2.16	2.62	3.14	1.5	2.82	2.41	2.65
2.73	2.59	2.73	3.37	2.58	2.5	2.68	3.56	2.34	3.56	3.94	2.6	2.72	2.95	2.37	2.75	3.39	1.5	2.82	2.46	2.66
2.96	2.6	2.73	3.37	2.71	2.74	2.68	3.56	2.35	3.66	3.96	2.6	2.72	2.96	2.44	2.75	3.5	1.51	2.82	2.46	2.67
2.96	2.76	2.73	3.37	2.71	2.74	2.68	3.56	2.35	3.66	4.19	2.6	2.73	3.18	2.44	2.75	3.51	1.63	2.82	2.46	2.78
2.96	2.86	2.73	3.38	2.72	2.75	2.79	3.56	2.35	3.66	4.19	2.6	2.87	3.21	2.6	2.75	3.52	1.67	2.82	2.46	2.79
2.96	2.86	2.82	3.61	2.72	2.8	2.88	3.69	2.35	3.78	4.19	2.6	2.97	3.22	2.6	2.75	3.54	1.68	2.82	2.46	2.98
3.07	2.91	2.82	3.62	2.8	2.87	2.88	3.72	2.35	4.02	4.3	2.71	2.97	3.22	2.6	2.75	3.67	1.77	3.1	2.46	2.99
3.08	2.91	2.82	3.62	2.8	2.89	2.88	4	2.36	4.02	4.36	2.74	2.98	3.69	2.6	2.75	3.67	1.77	3.12	2.46	3.07
3.08	2.91	2.82	3.62	2.8	2.89	2.88	4.03	2.36	4.06	4.41	2.74	2.98	3.69	2.6	2.75	3.7	1.77	3.12	2.72	3.08
3.08	2.92	2.97	3.62	2.92	2.92	2.88	4.1	2.36	4.06	4.41	2.75	2.98	3.69	2.6	2.75	3.73	1.77	3.14	2.72	3.23
3.21	3.1	3.17	3.62	2.92	2.92	2.88	4.1	2.4	4.07	4.43	2.96	2.98	3.7	2.64	2.75	3.91	1.92	3.22	2.72	3.23
3.21	3.15	3.17	3.71	2.93	2.93	2.89	4.1	2.41	4.07	4.57	3.05	3.33	3.7	2.74	2.76	3.94	1.95	3.24	2.72	3.23
3.22	3.26	3.17	3.72	2.93	2.97	2.89	4.1	2.5	4.07	4.65	3.05	3.34	3.71	2.74	2.81	3.94	1.95	3.24	2.91	3.33
3.22	3.26	3.17	3.72	3.13	2.97	2.89	4.1	2.5	4.27	4.65	3.05	3.34	3.71	2.74	2.81	3.96	1.95	3.24	2.91	3.34
3.23	3.26	3.17	3.72	3.13	2.99	2.89	4.1	2.51	4.45	4.65	3.28	3.34	3.84	2.74	2.81	3.96	1.95	3.7	2.92	3.34
3.23	3.36	3.17	4	3.33	2.99	2.96	4.1	2.51	4.45	4.79	3.28	3.34	3.84	2.91	2.94	3.97	2.03	3.7	2.92	3.42
3.24	3.36	3.19	4.01	3.33	3.09	3.02	4.1	2.51	4.45	4.95	3.28	3.38	3.84	2.91	2.94	3.97	2.03	3.7	2.92	3.43
3.47	3.37	3.26	4.01	3.34	3.2	3.16	4.1	2.51	4.55	4.97	3.28	3.4	3.84	2.91	2.95	3.98	2.03	3.71	3.13	3.45
3.47	3.37	3.26	4.02	3.62	3.2	3.16	4.1	2.52	4.56	4.97	3.28	3.4	3.84	3.34	3.15	4.03	2.03	3.71	3.13	3.46
3.47	3.58	3.34	4.02	3.62	3.2	3.16	4.12	2.52	4.76	5.02	3.28	3.4	3.85	3.34	3.15	4.05	2.03	3.71	3.14	3.47
3.48	3.58	3.38	4.18	3.62	3.2	3.16	4.12	2.52	4.76	5.08	3.37	3.41	3.85	3.34	3.15	4.14	2.15	3.71	3.23	3.61
3.48	3.63	3.45	4.19	3.63	3.2	3.16	4.12	2.53	4.81	5.15	3.45	3.41	3.86	3.34	3.15	4.18	2.17	3.71	3.24	3.69
3.48	3.73	3.54	4.19	3.64	3.29	3.26	4.12	2.53	4.81	5.18	3.45	3.58	3.86	3.34	3.17	4.18	2.19	3.71	3.24	3.7
3.48	3.73	3.54	4.19	3.64	3.37	3.36	4.12	2.53	4.93	5.18	3.45	3.58	3.87	3.34	3.25	4.31	2.19	3.91	3.25	3.71
3.6	3.74	3.54	4.19	3.8	3.37	3.4	4.16	2.74	4.98	5.55	3.45	3.58	3.87	3.34	3.33	4.43	2.19	4.12	3.44	3.75
3.6	3.87	3.55	4.36	3.95	3.37	3.43	4.16	2.75	4.98	5.55	3.56	3.58	3.88	3.34	3.33	4.47	2.19	4.12	3.45	3.79
3.6	4	3.59	4.36	3.96	3.37	3.43	4.16	2.75	5.08	5.55	3.56	3.58	3.96	3.51	3.38	4.5	2.19	4.12	3.45	3.8
3.62	4.01	3.59	4.36	3.96	3.37	3.43	4.33	2.9	5.23	5.55	3.71	3.59	4.07	3.59	3.38	4.5	2.19	4.12	3.45	3.91
3.62	4.11	3.62	4.36	4.13	3.41	3.71	4.57	2.99	5.39	5.68	3.71	3.59	4.07	3.59	3.45	4.64	2.25	4.12	3.62	3.92
3.85	4.11	3.71	4.59	4.14	3.43	3.73	4.57	2.99	5.4	5.75	3.71	3.86	4.08	3.59	3.61	4.69	2.44	4.83	3.62	3.93
3.85	4.12	3.71	4.62	4.14	3.44	3.73	4.57	2.99	5.4	5.81	3.94	3.87	4.08	3.61	3.61	4.69	2.49	4.83	3.62	4.03

3.85	4.12	3.74	4.63	4.14	3.52	3.73	4.57	3.06	5.4	5.81	3.94	3.87	4.14	3.7	3.61	4.69	2.66	4.83	3.62	4.03
3.86	4.2	3.82	4.63	4.16	3.58	3.96	4.57	3.13	5.44	5.89	3.98	3.87	4.33	3.7	3.64	4.78	2.66	4.83	3.63	4.03
3.93	4.36	4.02	4.64	4.16	3.58	4.03	4.57	3.14	5.44	6.12	3.99	3.93	4.33	3.93	3.77	4.79	2.68	4.83	3.71	4.04
4.08	4.43	4.02	4.71	4.25	3.58	4.14	4.73	3.18	5.54	6.24	4.02	4.01	4.36	3.93	3.77	4.81	2.68	4.83	3.71	4.08
4.2	4.62	4.27	4.78	4.28	3.61	4.2	4.73	3.33	5.59	6.4	4.11	4.06	4.36	3.94	3.82	5.01	2.71	4.83	3.71	4.11
4.38	4.63	4.27	4.98	4.6	3.65	4.2	4.8	3.33	5.87	6.61	4.15	4.11	4.52	3.94	3.82	5.05	2.85	5.17	3.78	4.21
4.38	4.69	4.27	5.12	4.66	3.69	4.49	5.11	3.33	6	6.85	4.18	4.24	4.67	4.2	3.82	5.08	2.91	5.17	3.78	4.3
4.6	4.7	4.33	5.13	4.73	3.8	4.49	5.11	3.4	6.12	7	4.31	4.35	4.69	4.2	3.88	5.08	2.95	5.53	3.88	4.48
4.61	4.83	4.33	5.2	4.73	3.82	4.56	5.18	3.4	6.12	7.02	4.33	4.35	4.7	4.31	3.98	5.32	2.95	5.53	3.88	4.5
4.61	4.91	4.38	5.29	4.95	3.87	4.78	5.18	3.54	6.18	7.4	4.44	4.35	4.7	4.31	4.06	5.32	2.95	5.53	4.05	4.6
4.66	4.92	4.61	5.3	5.02	3.87	4.84	5.18	3.54	6.4	7.42	4.55	4.57	4.78	4.49	4.21	5.34	2.95	5.53	4.13	4.76
4.8	5.06	4.81	5.69	5.02	3.87	4.87	5.31	3.67	6.96	7.72	4.67	4.57	4.78	4.52	4.21	5.35	3	5.53	4.22	4.84
4.88	5.06	4.81	5.77	5.03	3.94	4.87	5.31	3.79	7.19	7.77	4.68	4.57	4.79	4.54	4.39	5.52	3.19	5.53	4.31	4.96
5.02	5.07	4.81	5.83	5.33	3.99	4.9	5.31	3.79	7.43	7.83	4.68	4.57	5.04	4.54	4.47	5.58	3.19	5.8	4.31	5.14
5.04	5.07	4.81	5.83	5.44	4.01	4.9	5.46	3.8	7.48	7.95	4.85	4.57	5.15	4.54	4.47	5.65	3.19	5.8	4.32	5.17
5.3	5.08	5.07	.	5.44	4.3	4.92	5.52	3.8	7.7	8.15	4.9	4.81	5.16	4.54	4.47	5.68	3.19	6.11	4.61	5.2
5.45	5.19	5.13	.	5.45	4.35	5.06	5.52	3.86	7.86	8.28	5.09	4.94	5.17	4.54	4.51	5.75	3.19	6.13	4.62	5.2
5.52	5.25	5.13	.	5.45	4.43	5.11	5.56	3.86	7.86	8.37	5.17	5.02	5.2	4.54	4.86	5.94	3.19	6.27	4.93	5.3
5.61	5.28	5.13	.	5.74	4.43	5.11	5.56	4.03	8.01	8.5	5.26	5.03	5.24	4.66	4.91	5.99	3.23	6.58	4.93	5.35
5.66	5.28	5.13	.	5.88	4.45	5.24	5.65	4.03	8.15	8.5	5.26	5.17	5.3	4.66	4.91	6	3.29	6.6	4.93	5.47
5.74	5.28	5.13	.	5.96	4.51	5.28	5.65	4.03	8.15	8.82	5.28	5.17	5.36	4.78	4.91	6.02	3.43	6.6	4.94	5.51
5.93	5.28	5.16	.	5.96	4.6	5.28	5.82	4.03	8.15	8.93	5.52	5.17	5.38	4.78	4.91	6.07	3.48	6.7	5.12	5.69
5.97	5.49	5.16	.	5.96	4.7	5.28	5.82	4.04	8.17	8.93	5.52	5.18	5.4	4.82	4.93	6.09	3.5	6.7	5.12	5.69
6.02	5.55	5.17	.	5.96	4.72	5.39	5.82	4.04	8.45	9.04	5.52	5.18	5.6	4.82	4.93	6.18	3.5	6.71	5.19	5.91
6.14	5.69	5.23	.	**5.96**	4.73	5.4	5.82	4.04	8.5	9.14	**5.52**	**5.27**	5.72	4.9	4.93	6.18	3.55	6.88	5.23	6
6.15	5.69	5.23	.	**5.97**	4.76	5.4	5.82	4.08	8.96	9.14	**5.52**	**5.29**	5.78	4.99	4.93	6.24	3.59	6.9	5.23	6.14
6.31	5.69	5.23	.	**5.97**	4.87	5.41	6.07	4.09	8.96	9.41	**5.56**	**5.29**	5.79	4.99	4.95	6.24	3.59	6.9	5.24	6.26
6.38	5.75	5.33	.	**5.98**	4.87	5.41	6.07	4.09	8.96	9.51	**5.58**	**5.29**	5.79	4.99	5.2	6.24	3.63	7.11	5.41	6.26
6.39	5.95	5.48	.	**5.98**	5.24	5.49	6.07	4.1	8.96	9.51	**5.63**	**5.35**	5.8	5.09	5.47	6.31	3.64	7.17	5.41	6.47
6.39	6.08	5.51	.	**6.21**	5.34	5.51	6.07	4.1	9.51	9.68	**5.66**	**5.36**	5.8	5.09	5.58	6.31	3.65	7.17	5.42	6.51
6.39	6.12	5.51	.	**6.29**	5.46	5.51	6.07	4.1	9.51	9.68	**5.77**	**5.36**	5.93	5.34	5.63	6.34	3.65	7.34	5.47	6.54
6.39	6.12	5.51	.	**6.29**	5.46	5.51	6.07	4.11	9.51	9.88	**5.89**	**5.48**	6.14	5.34	5.73	6.35	3.65	7.34	5.54	6.54
6.39	6.13	5.51	.	6.3	5.46	5.53	6.38	4.11	9.51	9.9	5.89	5.52	6.14	5.34	5.73	6.35	3.81	7.38	5.63	6.66
6.5	6.13	5.51	.	6.3	5.46	5.53	6.38	4.11	9.51	9.95	6.15	5.8	6.14	5.45	5.93	6.45	3.88	7.39	5.63	6.75
6.67	6.26	5.51	.	6.31	5.46	5.71	6.38	4.11	9.55	9.96	6.3	5.81	6.27	5.45	5.95	6.61	3.9	7.52	5.63	6.79
6.67	6.4	5.95	.	6.31	5.46	5.8	6.38	4.12	9.55	9.96	6.3	5.81	6.33	5.45	5.95	6.61	3.92	7.52	5.94	6.79
6.67	6.4	6.02	.	6.53	5.46	5.91	6.38	4.33	9.55	10.24	6.3	5.81	6.34	5.45	6.16	6.62	3.97	7.52	5.94	6.79
6.68	6.41	6.02	.	6.88	5.46	5.92	6.38	4.39	9.59	10.25	6.43	5.81	6.34	5.46	6.28	6.66	4.03	7.52	5.94	6.9
6.8	6.41	6.02	.	6.88	5.52	5.92	6.38	4.39	9.69	10.29	6.45	5.81	6.35	5.5	6.28	6.7	4.03	7.52	5.94	6.91
6.8	6.41	6.02	.	6.88	5.52	5.94	6.38	4.4	9.69	10.37	6.45	5.99	6.56	5.71	6.28	6.79	4.07	7.52	5.94	6.91
6.8	6.74	6.02	.	6.88	5.54	5.97	6.5	4.5	9.69	10.42	6.68	6.01	6.62	5.82	6.28	6.79	4.08	8.08	5.94	7.05
6.96	6.83	6.02	.	6.88	5.54	6.16	6.64	4.5	9.69	10.59	6.73	6.02	6.62	5.82	6.28	6.88	4.08	8.08	6.04	7.05
7.09	6.83	6.05	.	6.88	5.54	6.25	6.64	4.51	9.82	10.65	6.73	6.02	6.62	5.82	6.38	6.88	4.08	8.08	6.04	7.19
7.09	6.84	6.05	.	6.88	5.54	6.25	6.64	4.59	9.92	10.69	6.73	6.24	6.63	5.82	6.47	6.88	4.1	8.08	6.04	7.19
7.19	6.84	6.3	.	6.89	5.54	6.25	6.93	4.59	10.02	10.73	6.82	6.25	6.64	5.82	6.54	6.88	4.2	8.08	6.04	7.2
7.19	6.84	6.32	.	6.99	5.63	6.36	6.93	4.63	10.17	10.88	6.82	6.25	6.65	5.84	6.73	6.97	4.2	8.09	6.3	7.2
7.29	6.93	6.32	.	6.99	5.67	6.44	6.93	4.63	10.17	10.95	6.83	6.49	6.65	5.91	6.73	7.05	4.21	8.09	6.3	7.4
7.29	6.93	6.38	.	6.99	5.67	6.44	7.11	4.63	10.17	10.95	6.83	6.5	6.79	5.91	6.73	7.05	4.21	8.18	6.3	7.4
7.29	6.93	6.39	.	6.99	5.67	6.44	7.2	4.63	10.17	10.95	6.83	6.5	6.8	6.02	6.73	7.05	4.27	8.2	6.31	7.4
7.29	6.93	6.39	.	7	5.67	6.44	7.2	4.63	10.22	10.95	6.84	6.5	6.8	6.24	6.84	7.05	4.29	8.2	6.31	7.41
7.39	6.93	6.39	.	7.1	5.75	6.46	7.2	4.84	10.28	10.95	6.84	6.5	6.8	6.3	6.84	7.06	4.36	8.2	6.31	7.41
7.52	6.93	6.43	.	7.21	5.77	6.46	7.2	4.84	10.46	11.14	6.86	6.51	7.02	6.3	6.84	7.18	4.36	8.2	6.31	7.42
7.52	6.93	6.43	.	7.29	5.78	6.46	7.2	4.85	10.5	11.31	6.87	6.51	7.03	6.3	6.84	7.19	4.36	8.2	6.53	7.42
7.52	6.94	6.43	.	7.29	5.78	6.61	7.2	4.85	10.5	11.34	6.87	6.51	7.04	6.3	6.91	7.24	4.36	8.2	6.53	7.42
7.52	7.05	6.43	.	7.3		6.61	7.2	4.99	10.5	11.35	6.88	6.51		6.3	6.91	7.24	4.36	8.2	6.53	7.43
7.52	7.05	6.43	.	7.3		6.61	7.39	4.99	10.5	11.36	6.88	6.81		6.3	6.94	7.24	4.5	8.74	6.53	7.61
7.6	7.25	6.46	.	7.47		6.61	7.39	4.99	10.6	11.49	6.94	6.82		6.3	7.11	7.24	4.5	9.2	6.53	7.61
7.6	7.32	6.79	.	7.48		6.63	7.39	4.99	10.6	11.49	6.95	6.82		6.3	7.11	7.24	4.51	9.2	6.53	7.61
7.6	7.32	6.82	.	7.49		6.84	7.64	5	10.6	11.49	6.95	6.82		6.42	7.11	7.28	4.7	9.2	6.54	7.62
7.78	7.32	6.82	.	7.62		6.85	7.64	5	10.6	11.74	7.21	6.82		6.59	7.11	7.5	4.73	9.2	6.8	7.62
7.79	7.52	6.98	.	7.84		6.85	7.64	5.09	10.69	11.84	7.23	6.91		6.64	7.11	7.5	4.78	9.2	6.8	7.63
7.79	7.52	7.01	.	7.95		6.85	7.64	5.09	10.92	12.01	7.23	6.91		6.64	7.18	7.5	4.78	9.2	6.8	7.63
7.79	7.52	7.09	.	7.95		7.17	7.64	5.16	10.92	12.05	7.24	6.91		6.73	7.3	7.5	5.04	9.2	6.8	7.63
7.85	7.53	7.09	.	7.96		7.23	7.64	5.35	10.92	12.11	7.49	6.91		6.88	7.31	7.5	5.04	9.42	6.8	7.8
7.86	7.53	7.14	.	7.96		7.23	7.64	5.35	10.97	12.11	7.49	6.91		6.88	7.31	7.77	5.04	9.42	7.02	7.81
7.86	7.63	7.14	.	7.97		7.27	7.64	5.36	10.97	12.11	7.49	7.06		6.88	7.31	7.77	5.04	9.42	7.03	7.81
7.86	7.63	7.14	.	8.14		7.29	7.64	5.36	11.17	12.11	7.66	7.06		6.88	7.31	7.77	5.04	9.42	7.04	7.81
7.86	7.63	7.15	.	8.16		7.29	7.64	5.46	11.49	12.41	7.69	7.06		6.88	7.38	7.77	5.04	9.42	7.04	7.88
7.86	7.72	7.15	.	8.21		7.35	7.64	5.47	11.49	12.43	7.74	7.12		6.88	7.38	7.92	5.04	9.61	7.11	7.88
7.86	7.76	7.15	.	8.21		7.36	7.64	5.74	11.49	12.59	7.85	7.13		6.88	7.41	7.99	5.04	9.61	7.12	7.93
7.93	7.81	7.16	.	8.47		7.43	7.64	5.75	11.49	12.59	7.92	7.13		6.88	8	8.01	5.16	9.61	7.12	8
8.16	7.88	7.2	.	8.47		7.45	7.82	5.75	12.1	12.7	7.92	7.14		6.88	8.34	8.04	5.21	9.64	7.31	8.07
8.16	8.02	7.2	.	8.54		7.45	7.9	5.86	12.67	12.82	8.13	7.16		6.88	8.35	8.27	5.21	9.75	7.32	8.17
8.16	8.02	7.2	.	8.76		7.45	8.04	5.99	12.67	12.95	8.13	7.16		6.88	8.35	8.32	5.26	9.75	7.32	8.37
8.56	8.13	7.34	.	8.81		7.61	8.04	5.99	12.67	13.11	8.32	7.26		6.94	8.35	8.34	5.26	9.75	7.32	8.46
8.56	8.2	7.37	.	8.9		7.83	8.05	5.99	12.81	13.14	8.32	7.27		6.94	8.35	8.34	5.47	9.83	7.32	8.54
8.56	8.2	7.4	.	8.9		7.83	8.05	6	12.81	13.31	8.34	7.33		6.94	8.52	8.39	5.5	9.83	7.33	8.68
8.66	8.2	7.6	.	8.94		7.86	8.26	6.1	12.85	13.5	8.48	7.36		6.94	8.83	8.39	5.53	9.84	7.39	8.76
8.67	8.46	7.6	.	9.02		7.86	8.26	6.19	12.85	13.55	8.48	7.36		6.98	8.83	8.42	5.63	9.95	7.39	8.89
8.67	8.62	7.6	.	9.02		7.88	8.26	6.27	13.76	13.56	8.54	7.47		7.13	8.83	8.65	5.64	10.18	7.4	8.98
8.67	8.63	7.6	.	9.1		8.04	8.26	6.32	14.12	13.64	8.54	7.8		7.16	8.83	8.77	5.67	10.18	7.4	9.05
8.67	8.63	7.6	.	9.1		8.04	8.34	6.33	14.23	14.06	8.66	7.84		7.25	8.83	8.92	5.67	10.5	7.74	9.1
8.8	8.8	7.6	.	9.11		8.05	8.35	6.36	14.28	14.06	8.71	7.84		7.25	8.97	8.92	5.7	10.5	7.74	9.1
9.03	8.82	7.72	.	9.56		8.08	8.67	6.37	14.29	14.07	8.71	7.84		7.42	8.97	8.92	5.75	10.5	7.84	9.11
9.11	8.82	7.72	9.72	9.56		8.08	8.67	6.37	14.29	14.08	8.71	7.84		7.42	8.97	8.92	5.75	10.5	7.84	9.22
9.11	8.83	7.72	9.72	9.56		8.11	8.67	6.37	14.29	14.08	9.16	7.84		7.42	8.97	8.92	5.75	10.5	8	9.29
9.29	8.83	7.82	9.72	9.62		8.12	8.67	6.38	14.39	14.19	9.16	7.84		7.42	8.97	8.92	5.77	10.5	8	9.48
9.32	8.97	7.87	9.77	9.63		8.18	8.67	6.38	14.39	14.19	9.16	7.84		7.46	8.97	8.93	5.79	10.5	8	9.7
9.67	9	7.87	9.82	9.67		8.18	8.67	6.38	14.58	14.35	9.18	8.18		7.55	9.46	8.93	5.79	10.5	8.01	9.7
9.67	9.02	7.93	9.95	9.74		8.18	8.73	6.55	14.58	14.4	9.38	8.28		7.55	9.46	8.93	5.85	10.5	8.01	9.7
9.67	9.41	7.93	10.2	9.74		8.18	8.83	6.56	14.58	14.4	9.38	8.28		7.65	9.61	8.93	5.9	10.5	8.07	9.79
9.67	9.43	7.93	10.34	9.74		8.18	9.1	6.56	14.59	14.55	9.38	8.28		7.69	9.61	8.93	5.96	10.74	8.08	9.8
9.73	9.44	7.93	10.35	9.74		8.31	9.1	6.56	14.64	14.58	9.38	8.29		7.69	9.65	8.93	5.96	10.8	8.08	9.8
9.92	9.5	7.95	10.35	9.75		8.43	9.1	6.66	14.64	14.58	9.38	8.29		7.69	9.65	8.93	5.96	10.8	8.09	9.87
9.92	9.5	7.95	10.35	9.75		8.54	9.1	6.67	14.64	14.64	9.38	8.34		7.74	9.65	8.94	5.96	10.8	8.19	9.87
9.92	9.51	7.95	10.67	9.83		8.56	9.1	6.67	14.75	14.71	9.38	8.38		7.89	9.72	9.11	5.96	10.8	8.19	10.03
10.02	9.6	7.95	10.76	9.9		8.59	9.25	6.68	14.8	14.83	9.39	8.41		7.9	9.79	9.2	5.96	10.8	8.19	10.03
10.02	9.61	8.23	10.76	9.97		8.64	9.25	6.71	14.8	14.83	9.55	8.41		7.9	9.79	9.22	5.96	10.8	8.19	10.04
10.02	9.62	8.31	10.79	9.97		8.75	9.25	6.72	14.8	14.83	9.55	8.41		7.9	9.79	9.22	5.96	10.8	8.19	10.12
10.03	9.62	8.31	10.79	10.16		8.79	9.25	6.72	14.8	15.01	9.55	8.57		7.9	9.79	9.23	6.11	10.8	8.19	10.21
10.03	9.62	8.37	10.8	10.17		8.85	9.5	7.02	14.8	15.01	9.55	8.62		7.9	9.98	9.45	6.22	11.12	8.19	10.34
10.2	9.94	8.37	10.8	10.29		8.88	9.71	7.03	15.17	15.01	9.55	8.62		7.9	10.04	9.56	6.37	11.12	8.19	10.34
10.2	9.94	8.37	10.8	10.29		8.88	9.71	7.03	15.17	15.18	9.74	8.62		7.9	10.24	9.61	6.37	11.12	8.41	10.34
10.2	9.94	8.37	10.8	10.3		9.05	9.71	7.03	15.17	15.18	9.82	8.62		8.01	10.24	9.61	6.45	11.12	8.41	10.35
10.28	9.94	8.37	10.8	10.3		9.21	9.82	7.03	15.18	15.18	9.82	8.62		8.26	10.24	9.61	6.48	11.12	8.41	10.35
10.3	9.94	8.37	10.81	10.31		9.21	9.83	7.03	15.29	15.18	9.82	8.62		8.39	10.24	9.61	6.48	11.12	8.41	10.49
10.3	10.04	8.37	11.11	10.31		9.21	9.83	7.1	15.31	15.18	9.93	8.63		8.39	10.24	9.61	6.48	11.12	8.41	10.49
10.3	10.06	8.37	11.11	10.32		9.21	9.83	7.11	15.31	15.18	10.02	8.74		8.41	10.24	9.61	6.48	11.12	8.51	10.56
10.3	10.06	8.48	11.11	10.54		9.21	9.83	7.11	15.34	15.32	10.03	8.82		8.41	10.24	9.61	6.5	11.12	8.52	10.56
10.3	10.35	8.6	11.11	10.83		9.21	9.83	7.11	15.36	15.34	10.03	8.82		8.41	10.37	9.61	6.51	11.12	8.52	10.57
10.3	10.35	8.6	11.11	10.83		9.21	9.83	7.28	15.36	15.34	10.03	8.82		8.41	10.37	9.61	6.51	11.12	8.52	10.57
10.3	10.36	8.66	11.25	10.84		9.21	9.83	7.28	15.36	15.47	10.14	8.82		8.41	10.37	9.61	6.51	11.12	8.73	10.57
10.31	10.36	8.66	11.25	10.84		9.21	9.86	7.29	15.55	15.67	10.14	8.82		8.41	10.37	9.61	6.51	11.12	8.73	10.58
10.31	10.36	8.66	11.25	10.85		9.39	9.86	7.29	15.56	15.71	10.14	8.96		8.41	10.47	9.63	6.54	11.12	8.74	10.88
0	0	0	0	0	0	0	0	0	0.02	0	0	0	0	0	0	0.13	0	0	0	0
0.01	0.05	0.01	0	0	0.06	0.09	0	0	0.13	0.04	0.08	0.07	0.14	0	0	0.27	0.08	0	0	0
0.01	0.07	0.14	0	0	0.24	0.14	0	0	0.13	0.18	0.2	0.08	0.14	0.16	0.08	0.39	0.08	0	0	0.01
0.02	0.13	0.14	0.08	0.18	0.24	0.18	0	0	0.13	0.18	0.2	0.08	0.25	0.16	0.09	0.39	0.08	0	0.12	0.02
0.16	0.13	0.23	0.16	0.25	0.3	0.18	0	0	0.6	0.25	0.21	0.23	0.25	0.19	0.09	0.41	0.08	0	0.21	0.03
0.16	0.13	0.23	0.19	0.26	0.38	0.23	0	0	0.93	0.25	0.22	0.23	0.25	0.44	0.16	0.41	0.08	0	0.29	0.22
0.16	0.46	0.23	0.2	0.38	0.49	0.23	0	0.21	0.93	0.25	0.23	0.23	0.25	0.53	0.16	0.42	0.08	0	0.3	0.36
0.29	0.48	0.23	0.31	0.4	0.55	0.24	0	0.34	0.93	0.25	0.28	0.24	0.25	0.53	0.17	0.43	0.08	0	0.3	0.36
0.3	0.48	0.28	0.32	0.42	0.55	0.36	0	0.34	0.93	0.38	0.28	0.34	0.32	0.53	0.26	0.54	0.08	0	0.43	0.37
0.3	0.5	0.32	0.32	0.5	0.55	0.36	0.12	0.34	0.93	0.4	0.4	0.35	0.45	0.53	0.3	0.56	0.1	0	0.43	0.5
0.4	0.5	0.32	0.44	0.5	0.55	0.36	0.6	0.34	0.98	0.4	0.41	0.35	0.45	0.53	0.3	0.6	0.17	0	0.43	0.5
0.41	0.5	0.37	0.52	0.5	0.55	0.46	0.79	0.35	1.25	0.44	0.59	0.36	0.45	0.53	0.34	0.63	0.21	0.13	0.62	0.51
0.42	0.65	0.37	0.52	0.5	0.55	0.56	0.79	0.35	1.25	0.44	0.59	0.42	0.56	0.62	0.42	0.7	0.22	0.16	0.63	0.51
0.58	0.69	0.44	0.62	0.58	0.55	0.56	0.79	0.35	1.25	0.51	0.6	0.49	0.64	0.83	0.49	0.7	0.25	0.16	0.63	0.64
0.62	0.69	0.58	0.7	0.58	0.55	0.57	0.79	0.42	1.25	0.56	0.66	0.57	0.69	1.04	0.51	0.8	0.38	0.26	0.64	0.67
0.63	0.69	0.58	0.72	0.73	0.55	0.72	0.79	0.42	1.25	0.7	0.68	0.66	0.79	1.14	0.51	0.97	0.38	0.41	0.64	0.67
0.63	0.74	0.58	0.74	0.8	0.55	0.79	0.79	0.45	1.25	0.8	0.71	0.67	0.94	1.14	0.59	0.97	0.44	0.68	0.64	0.73

0.73	0.75	0.63	0.93	0.83	0.55	0.79	0.79	0.45	1.25	0.84	0.76	0.7	1.12	1.14	0.59	1.06	0.47	0.8	0.74	0.73
1.05	0.75	0.71	0.97	0.86	0.63	0.79	0.79	0.45	1.25	0.87	0.8	0.81	1.17	1.25	0.69	1.11	0.6	0.84	0.87	0.87
1.07	0.77	0.75	1.02	0.99	0.65	1	0.79	0.45	1.25	0.97	0.82	0.89	1.23	1.25	0.69	1.11	0.6	0.84	0.89	0.9
1.07	0.9	0.83	1.02	1.01	0.65	1.01	0.79	0.45	1.25	0.97	0.82	0.9	1.47	1.25	0.8	1.11	0.61	0.84	0.95	0.91
1.25	0.97	0.91	1.04	1.01	1.01	1.01	0.79	0.54	1.25	0.98	0.82	0.93	1.47	1.31	0.92	1.66	0.7	0.84	1.03	0.92
1.3	0.97	1.07	1.04	1.15	1.01	1.01	0.79	0.54	1.25	1.07	0.91	0.96	1.47	1.43	0.96	1.66	0.7	0.84	1.1	0.92
1.31	0.98	1.07	1.21	1.24	1.01	1.08	0.79	0.67	1.25	1.13	0.92	0.96	1.55	1.56	0.98	1.66	0.77	0.84	1.1	1.11
1.31	0.99	1.07	1.35	1.25	1.01	1.18	0.79	0.74	1.25	1.13	0.92	0.97	1.55	1.56	1.09	1.66	0.94	0.84	1.1	1.11
1.51	1.2	1.13	1.46	1.25	1.01	1.18	0.95	0.83	1.25	1.38	1.03	0.97	1.55	1.56	1.09	1.66	0.94	0.84	1.11	1.22
1.51	1.25	1.13	1.53	1.25	1.01	1.24	0.98	0.88	1.25	1.52	1.03	1.06	1.55	1.56	1.14	1.69	0.94	0.9	1.11	1.23
1.67	1.25	1.16	1.64	1.28	1.06	1.35	1.04	0.91	1.25	1.6	1.12	1.17	1.56	1.56	1.14	1.79	1.04	0.9	1.15	1.23
1.73	1.25	1.17	1.66	1.28	1.06	1.48	1.04	0.92	1.25	1.66	1.12	1.27	1.82	1.56	1.14	2.09	1.1	0.9	1.15	1.44
1.82	1.25	1.19	1.73	1.47	1.09	1.54	1.11	0.99	1.3	1.66	1.12	1.39	1.96	1.56	1.21	2.26	1.1	0.93	1.15	1.54
1.82	1.26	1.23	1.96	1.68	1.3	1.54	1.15	1	1.38	1.72	1.15	1.46	1.96	1.56	1.31	2.28	1.12	1.18	1.16	1.57
1.83	1.42	1.23	1.97	1.68	1.3	1.54	1.34	1	1.38	1.75	1.32	1.53	1.96	1.79	1.31	2.28	1.12	1.27	1.5	1.64
1.92	1.63	1.3	1.99	1.78	1.32	1.6	1.36	1	1.6	1.75	1.34	1.56	1.96	1.79	1.42	2.28	1.12	1.43	1.52	1.69
2.02	1.7	1.38	2.16	1.78	1.35	1.74	1.36	1.03	1.81	1.86	1.43	1.56	2.22	1.79	1.55	2.28	1.15	1.6	1.52	1.72
2.03	1.72	1.41	2.19	1.78	1.43	1.81	1.44	1.11	1.81	1.96	1.48	1.56	2.34	1.82	1.55	2.28	1.28	1.6	1.52	1.75
2.03	1.74	1.5	2.19	1.78	1.45	1.83	1.5	1.11	1.81	2.01	1.53	1.57	2.34	1.82	1.56	2.28	1.3	1.6	1.52	1.75
2.26	1.74	1.5	2.21	1.8	1.45	1.83	1.5	1.11	1.81	2.01	1.63	1.58	2.34	1.83	1.56	2.28	1.32	1.62	1.52	1.75
2.38	1.75	1.5	2.21	1.8	1.54	1.88	1.5	1.11	1.81	2.04	1.63	1.58	2.34	1.83	1.56	2.28	1.32	1.62	1.68	1.75
2.4	1.92	1.71	2.21	1.81	1.54	1.94	1.6	1.14	1.81	2.13	1.73	1.58	2.34	1.94	1.7	2.42	1.32	1.62	1.82	1.81
2.47	2.05	1.71	2.41	1.86	1.54	1.94	1.66	1.14	1.81	2.13	1.73	1.68	2.34	1.98	1.96	2.42	1.33	1.62	1.82	1.98
2.54	2.18	1.71	2.42	2.12	1.86	1.99	1.85	1.22	1.81	2.13	1.74	1.69	2.34	2.02	1.97	2.42	1.38	1.83	1.82	2.07
2.59	2.23	1.71	2.43	2.13	1.86	2.05	1.9	1.35	1.81	2.17	1.75	1.69	2.43	2.08	1.97	2.42	1.5	1.83	1.82	2.07
2.66	2.29	1.71	2.48	2.13	1.86	2.06	1.9	1.41	1.81	2.43	1.77	1.86	2.43	2.14	1.97	2.42	1.79	1.83	1.82	2.08
2.66	2.29	1.71	2.54	2.13	1.95	2.06	2	1.48	1.81	2.5	1.85	2.06	2.43	2.14	1.98	2.42	1.79	1.83	1.82	2.2
2.66	2.29	1.74	2.7	2.22	1.95	2.11	2.25	1.55	2.04	2.52	1.85	2.1	2.59	2.14	2.13	2.42	1.91	1.83	2.04	2.26
2.66	2.34	1.74	2.7	2.23	1.98	2.13	2.25	1.55	2.04	2.57	1.99	2.14	2.73	2.18	2.13	2.52	1.91	2.1	2.14	2.39
2.67	2.34	1.74	2.7	2.31	2.08	2.13	2.25	1.55	2.06	2.57	1.99	2.14	2.86	2.2	2.13	2.57	1.91	2.1	2.14	2.39
2.69	2.36	1.74	2.83	2.33	2.11	2.21	2.25	1.55	2.06	2.57	1.99	2.14	2.86	2.29	2.13	2.68	1.91	2.1	2.14	2.39
2.79	2.36	1.88	2.88	2.34	2.11	2.21	2.25	1.6	2.16	2.57	2	2.15	2.86	2.29	2.22	2.68	1.91	2.1	2.14	2.39
2.8	2.4	1.88	2.9	2.49	2.16	2.41	2.25	1.6	2.16	2.69	2.17	2.15	2.86	2.3	2.23	2.68	1.91	2.1	2.15	2.39
2.8	2.4	1.88	2.9	2.56	2.16	2.43	2.29	1.6	2.16	2.71	2.22	2.16	2.86	2.3	2.23	2.68	1.91	2.13	2.28	2.42
2.8	2.43	1.88	3	2.56	2.16	2.43	2.29	1.6	2.29	2.71	2.22	2.16	2.86	2.83	2.3	2.68	1.91	2.13	2.37	2.42
2.8	2.43	1.88	3.09	2.62	2.16	2.43	2.29	1.68	2.6	2.76	2.22	2.26	2.86	2.83	2.3	2.69	1.91	2.13	2.37	2.43
2.8	2.59	2.08	3.09	2.64	2.25	2.44	2.34	1.68	2.6	2.76	2.28	2.26	2.86	2.87	2.41	2.99	2.06	2.13	2.37	2.6
2.81	2.61	2.08	3.21	2.64	2.27	2.6	2.34	1.68	2.6	2.94	2.29	2.27	3.08	2.87	2.41	2.99	2.17	2.13	2.38	2.6
2.81	2.61	2.1	3.3	2.66	2.27	2.61	2.45	1.68	2.6	2.94	2.3	2.38	3.08	2.87	2.41	2.99	2.23	2.13	2.38	2.61
3.06	2.62	2.1	3.3	2.75	2.27	2.65	2.65	1.68	2.6	2.94	2.39	2.46	3.08	2.87	2.51	2.99	2.26	2.33	2.38	2.61
3.06	2.63	2.18	3.3	2.83	2.31	2.65	2.65	1.68	2.69	2.94	2.39	2.47	3.09	2.87	2.52	3.06	2.33	2.33	2.39	2.62
3.07	2.64	2.18	3.37	2.86	2.41	2.65	2.65	1.78	2.78	3.01	2.39	2.47	3.09	2.87	2.59	3.06	2.33	2.33	2.48	2.75
3.07	2.64	2.18	3.37	2.87	2.44	2.75	2.73	1.85	2.86	3.06	2.54	2.48	3.09	2.87	2.61	3.29	2.33	2.33	2.48	2.76
3.07	2.64	2.33	3.37	2.87	2.44	2.82	2.75	1.85	2.86	3.08	2.55	2.48	3.09	2.87	2.61	3.29	2.33	2.33	2.48	2.77
3.19	2.72	2.33	3.42	2.9	2.46	2.92	3.07	1.85	2.86	3.11	2.55	2.57	3.21	2.87	2.61	3.29	2.33	2.33	2.61	2.85
3.21	2.72	2.33	3.44	2.99	2.48	2.92	3.07	1.85	2.86	3.32	2.55	2.57	3.25	2.87	2.61	3.29	2.33	2.33	2.61	3
3.21	2.72	2.33	3.47	3	2.6	3.04	3.07	1.92	2.93	3.32	2.56	2.58	3.25	2.87	2.61	3.29	2.56	2.49	2.62	3
3.21	2.82	2.33	3.47	3.1	2.6	3.27	3.07	1.93	2.93	3.32	2.65	2.64	3.25	2.87	2.64	3.29	2.68	2.66	2.62	3
3.22	2.82	2.33	3.58	3.14	2.6	3.27	3.07	1.93	2.94	3.32	2.73	2.64	3.25	2.99	2.65	3.29	2.68	2.66	2.62	3
3.34	2.82	2.43	3.59	3.17	2.72	3.27	3.07	1.93	3.07	3.32	2.73	2.65	3.26	2.99	2.65	3.29	2.68	2.66	2.63	3
3.35	2.82	2.43	3.71	3.29	2.73	3.27	3.07	1.94	3.23	3.37	2.74	2.65	3.3	2.99	2.65	3.3	2.68	2.66	2.7	3.01
3.35	3.01	2.43	3.71	3.29	2.73	3.5	3.07	2.06	3.23	3.38	2.75	2.66	3.4	3	2.71	3.3	2.68	2.66	2.81	3.09
3.48	3.07	2.43	3.77	3.29	2.73	3.51	3.07	2.06	3.23	3.53	2.87	2.67	3.4	3	2.79	3.3	2.73	2.66	2.89	3.17
3.54	3.07	2.46	3.93	3.31	2.73	3.51	3.07	2.06	3.23	3.55	2.92	2.67	3.4	3.13	2.86	3.3	2.84	2.66	2.89	3.18
3.55	3.07	2.67	3.94	3.44	2.73	3.51	3.07	2.07	3.23	3.55	2.92	2.77	3.54	3.13	2.88	3.32	2.84	2.66	2.89	3.19
3.55	3.07	2.76	3.94	3.45	2.73	3.51	3.07	2.35	3.23	3.55	2.99	2.77	3.54	3.24	2.88	3.33	2.9	2.88	2.89	3.29
3.55	3.19	2.76	3.95	3.47	2.74	3.51	3.07	2.36	3.23	3.78	3.1	2.77	3.62	3.24	2.88	3.49	2.95	3.05	2.93	3.29
3.55	3.27	2.76	3.95	3.48	2.74	3.51	3.07	2.37	3.23	3.86	3.1	2.78	3.63	3.24	2.94	3.49	3	3.05	3.03	3.39
3.55	3.36	2.76	4.07	3.53	2.81	3.57	3.07	2.37	3.23	3.86	3.1	2.78	3.64	3.24	2.94	3.49	3.02	3.05	3.04	3.39
3.7	3.36	2.76	4.07	3.65	2.81	3.58	3.07	2.61	3.23	3.86	3.12	2.78	3.64	3.24	2.94	3.52	3.05	3.05	3.04	3.39
3.77	3.36	2.86	4.13	3.65	2.86	3.58	3.07	2.61	3.26	3.86	3.2	3.01	3.75	3.24	3.08	3.98	3.07	3.07	3.12	3.39
3.78	3.38	2.87	4.21	3.65	2.95	3.76	3.15	2.62	3.26	3.95	3.2	3.01	3.85	3.24	3.13	3.98	3.23	3.08	3.19	3.4
3.91	3.39	2.99	4.34	3.75	2.95	3.82	3.35	2.66	3.26	3.96	3.37	3.07	3.94	3.32	3.15	3.98	3.29	3.22	3.2	3.49
4.01	3.5	3	4.42	3.8	3.01	3.82	3.4	2.74	3.36	3.98	3.44	3.08	4.08	3.5	3.19	3.98	3.29	3.28	3.2	3.58
4.09	3.56	3.08	4.46	3.85	3.03	3.98	3.4	2.78	3.37	4.06	3.45	3.15	4.08	3.5	3.2	3.98	3.37	3.38	3.28	3.64
4.14	3.67	3.08	4.51	3.96	3.06	4	3.44	2.81	3.37	4.1	3.53	3.17	4.15	3.5	3.2	4.15	3.45	3.4	3.31	3.7
4.2	3.67	3.08	4.54	4.05	3.06	4.08	3.44	2.88	3.45	4.28	3.58	3.18	4.2	3.5	3.21	4.25	3.46	3.48	3.43	3.77
4.3	3.67	3.08	4.54	4.07	3.06	4.08	3.44	3.04	3.52	4.32	3.63	3.31	4.23	3.5	3.24	4.34	3.53	3.48	3.6	3.83
4.35	3.68	3.1	4.57	4.15	3.13	4.24	3.58	3.06	3.53	4.32	3.63	3.32	4.26	3.5	3.29	4.34	3.54	3.6	3.61	3.83
4.4	3.83	3.18	4.73	4.18	3.13	4.32	3.58	3.06	3.73	4.32	3.68	3.42	4.28	3.58	3.34	4.48	3.64	3.61	3.61	3.91
4.48	3.87	3.23	4.88	4.32	3.17	4.37	3.58	3.14	3.73	4.41	3.68	3.51	4.34	3.58	3.4	4.5	3.66	3.62	3.68	3.96
4.54	3.89	3.23	5	4.37	3.18	4.37	3.58	3.18	3.99	4.41	3.8	3.51	4.37	3.8	3.41	4.54	3.69	3.68	3.68	4.01
4.6	3.89	3.44	5.1	4.39	3.18	4.38	3.76	3.24	3.99	4.5	3.92	3.52	4.42	3.9	3.46	4.56	3.71	3.68	3.68	4.12
4.74	3.99	3.44	5.2	4.4	3.28	4.48	3.76	3.32	4.02	4.56	4.09	3.6	4.42	3.9	3.46	4.56	3.71	3.96	3.68	4.24
4.74	4.06	3.45	5.23	4.4	3.39	4.49	3.76	3.41	4.03	4.62	4.1	3.65	4.64	4.3	3.55	4.63	3.73	4.06	3.87	4.27
4.74	4.06	3.51	5.33	4.4	3.39	4.69	3.91	3.41	4.03	4.91	4.11	3.71	4.78	4.3	3.6	5.05	3.78	4.06	3.98	4.27
4.81	4.13	3.6	5.34	4.54	3.39	4.85	3.97	3.47	4.36	5.06	4.28	3.82	4.81	4.3	3.81	5.15	3.89	4.06	4.07	4.38
4.81	4.2	3.65	5.36	4.67	3.39	4.94	4.25	3.47	4.36	5.06	4.31	3.9	4.95	4.3	3.87	5.15	4	4.14	4.2	4.38
4.94	4.3	3.68	5.36	4.74	3.39	5.02	4.25	3.54	4.38	5.06	4.32	4	4.95	4.3	3.93	5.15	4	4.15	4.25	4.48
4.94	4.3	3.7	5.54	4.75	3.39	5.11	4.27	3.58	4.6	5.06	4.32	4	5.04	4.3	3.93	5.17	4	4.15	4.29	4.5
5.13	4.3	3.92	5.55	4.77	3.39	5.11	4.28	3.73	4.7	5.06	4.59	4.04	5.04	4.36	3.93	5.29	4	4.15	4.29	4.5
5.13	4.38	3.92	5.62	4.91	3.39	5.11	4.32	3.82	4.7	5.13	4.61	4.08	5.05	4.36	4.04	5.46	4.02	4.15	4.29	4.55
5.14	4.44	3.92	5.82	5.04	3.4	5.12	4.38	3.82	4.7	5.42	4.66	4.08	5.13	4.51	4.04	5.46	4.07	4.15	4.3	4.68
5.26	4.44	3.92	5.94	5.04	3.49	5.24	4.41	3.83	4.7	5.46	4.66	4.09	5.31	4.51	4.04	5.54	4.23	4.44	4.3	4.68
5.48	4.51	3.92	5.95	5.04	3.49	5.25	4.41	3.83	4.7	5.49	4.66	4.17	5.31	4.51	4.27	5.54	4.25	4.44	4.3	4.69
5.54	4.54	3.92	5.97	5.04	3.49	5.36	4.51	3.83	4.7	5.61	4.67	4.3	5.31	4.51	4.42	5.54	4.32	4.44	4.47	4.7
5.66	4.6	4.04	6.05	5.04	3.54	5.36	4.56	3.84	4.7	5.73	4.73	4.31	5.31	4.51	4.45	5.56	4.32	4.66	4.48	4.92
5.69	4.62	4.22	6.07	5.16	3.54	5.36	4.79	3.84	4.7	5.73	4.74	4.31	5.39	4.72	4.45	5.61	4.32	4.74	4.48	5.08
5.75	4.65	4.22	6.17	5.33	3.57	5.43	4.9	4.05	4.93	5.73	4.74	4.31	5.41	4.96	4.57	5.66	4.47	4.74	4.71	5.09
5.75	4.7	4.29	6.23	5.37	3.62	5.51	4.9	4.13	4.93	5.73	4.88	4.42	5.66	4.96	4.64	5.91	4.51	4.74	4.79	5.16
5.82	4.71	4.45	6.3	5.4	3.66	5.58	4.9	4.13	4.93	5.73	4.97	4.55	5.71	4.96	4.64	5.91	4.52	4.74	4.85	5.16
5.82	4.71	4.45	6.35	5.4	3.67	5.66	4.9	4.19	4.93	5.73	4.97	4.56	5.74	4.96	4.65	5.91	4.52	4.74	4.85	5.16
5.82	4.72	4.45	6.35	5.41	3.86	5.69	5.02	4.22	4.93	5.91	4.97	4.63	5.82	5.05	4.65	5.93	4.52	4.86	4.94	5.16
6	4.72	4.51	6.49	5.56	3.86	5.73	5.03	4.37	4.93	6.01	5.04	4.63	5.85	5.07	4.65	5.99	4.52	4.86	4.95	5.29
6	4.75	4.54	6.49	5.69	3.86	5.73	5.03	4.42	4.93	6.17	5.12	4.64	5.92	5.07	4.65	6.04	4.52	4.86	4.95	5.29
6	4.75	4.61	6.55	5.69	3.86	5.73	5.03	4.42	4.93	6.24	5.13	4.64	5.92	5.07	4.79	6.07	4.52	4.95	4.95	5.3
6	4.77	4.62	6.55	5.76	3.86	5.73	5.03	4.42	5	6.27	5.16	4.64	5.92	5.07	4.92	6.09	4.52	5.15	4.95	5.3
6	4.86	4.67	6.56	5.79	3.96	5.8	5.03	4.42	5	6.27	5.16	4.64	5.98	5.07	4.99	6.13	4.52	5.24	5.11	5.31
6.16	4.86	4.67	6.57	5.79	4.21	5.8	5.03	4.42	5.06	6.42	5.16	4.8	5.98	5.14	4.99	6.66	4.52	5.24	5.11	5.38
6.16	4.86	4.67	6.57	5.79	4.32	5.99	5.13	4.42	5.06	6.49	5.26	4.8	5.99	5.5	5	6.66	4.67	5.24	5.11	5.38
6.16	4.87	4.84	6.78	5.8	4.32	5.99	5.28	4.43	5.06	6.49	5.27	4.81	5.99	5.5	5	6.66	4.7	5.24	5.26	5.38
6.17	4.87	4.84	6.92	5.81	4.32	5.99	5.29	4.43	5.24	6.49	5.27	4.81	6.05	5.5	5	6.66	4.8	5.24	5.36	5.47
6.17	4.87	4.84	6.92	5.9	4.32	5.99	5.29	4.61	5.24	6.49	5.28	4.81	6.05	5.74	5.11	6.75	4.84	5.24	5.36	5.47
6.17	4.87	4.84	6.92	5.92	4.32	6.08	5.32	4.61	5.24	6.49	5.41	4.99	6.05	5.91	5.12	6.75	4.84	5.44	5.36	5.48
6.25	5	4.85	6.92	6.02	4.32	6.22	5.34	4.61	5.24	6.53	5.49	4.99	6.23	5.96	5.12	6.85	4.84	5.44	5.5	5.49
6.25	5	4.85	6.96	6.02	4.32	6.22	5.34	4.61	5.24	6.64	5.49	5	6.23	6.2	5.13	6.85	4.84	5.44	5.5	5.52
6.25	5	4.87	6.96	6.02	4.37	6.34	5.34	4.62	5.24	6.82	5.5	5	6.29	6.2	5.13	6.85	4.85	5.44	5.51	5.67
6.39	5	4.87	7.11	6.24	4.44	6.34	5.34	4.62	5.24	6.83	5.64	5.01	6.29	6.2	5.14	6.85	4.89	5.57	5.51	5.7
6.39	5.12	4.87	7.17	6.24	4.44	6.34	5.34	4.62	5.24	6.86	5.75	5.07	6.29	6.2	5.17	6.85	4.9	5.57	5.51	5.7
6.39	5.13	4.87	7.17	6.24	4.45	6.34	5.37	4.63	5.34	6.87	5.75	5.07	6.29	6.2	5.29	6.85	4.9	5.57	5.6	5.7
6.39	5.13	4.87	7.2	6.25	4.45	6.34	5.45	4.67	5.34	6.87	5.75	5.07	6.31	6.2	5.29	6.85	4.9	5.57	5.6	5.7
6.49	5.25	4.94	7.23	6.25	4.54	6.34	5.47	4.67	5.34	6.87	5.77	5.07	6.31	6.2	5.3	6.85	4.9	5.57	5.67	5.7
6.5	5.36	4.94	7.23	6.34	4.54	6.34	5.58	4.67	5.52	6.87	5.77	5.08	6.36	6.2	5.3	6.85	5.01	5.79	5.67	5.88
6.5	5.36	4.94	7.23	6.41	4.55	6.34	5.62	4.67	5.58	6.92	5.77	5.2	6.5	6.2	5.31	6.85	5.05	5.89	5.67	5.88
6.5	5.36	5.08	7.24	6.42	4.84	6.34	5.62	4.67	6.36	6.95	5.92	5.2	6.5	6.2	5.31	7.13	5.05	5.89	5.68	5.88
6.58	5.36	5.08	7.24	6.42	4.91	6.34	5.62	4.67	6.36	6.95	5.92	5.21	6.5	6.2	5.38	7.13	5.05	5.89	5.68	5.88
6.58	5.39	5.08	7.37	6.43	4.91	6.34	5.71	4.73	6.36	6.98	5.92	5.21	6.5	6.2	5.38	7.13	5.19	5.89	5.8	5.89
6.58	5.61	5.08	7.37	6.43	4.91	6.35	5.77	4.81	6.42	7.09	5.92	5.3	6.56	6.2	5.39	7.13	5.19	5.95	5.8	5.9
6.64	5.61	5.12	7.37	6.53	4.91	6.37	5.77	4.91	6.42	7.13	5.93	5.3	6.67	6.2	5.48	7.13	5.19	5.95	5.93	5.91
6.72	5.63	5.12	7.43	6.54	4.91	6.37	5.77	4.91	6.42	7.28	5.95	5.3	6.67	6.2	5.48	7.13	5.2	5.96	5.94	6.03
6.72	5.63	5.27	7.54	6.54	4.92	6.52	5.77	5.06	6.42	7.31	6.01	5.31	6.68	6.2	5.52	7.65	5.26	5.96	5.94	6.04
6.79	5.63	5.32	7.54	6.55	4.92	6.61	5.77	5.06	6.42	7.33	6.07	5.45	6.69	6.2	5.53	7.65	5.42	6.09	5.99	6.14
6.88	5.63	5.32	7.54	6.69	5.04	6.62	5.79	5.06	6.42	7.33	6.07	5.45	6.75	6.2	5.53	7.65	5.49	6.09	6.02	6.16
6.88	5.78	5.32	7.9	6.7	5.09	6.62	5.86	5.07	6.42	7.44	6.1	5.45	6.87	6.2	5.57	7.65	5.49	6.09	6.02	6.16
6.88	5.8	5.34	7.9	6.7	5.13	6.66	5.87	5.21	6.42	7.44	6.12	5.45	6.88	6.2	5.64	7.65	5.49	6.09	6.17	6.16
7.03	5.8	5.34	7.9	6.78	5.13	6.77	5.87	5.22	6.49	7.44	6.13	5.45	6.88	6.2	5.77	7.65	5.49	6.09	6.17	6.23
7.03	5.83	5.34	7.9	6.78	5.18	6.81	6.02	5.22	6.55	7.57	6.2	5.48	6.93	6.2	5.82	7.65	5.51	6.09	6.17	6.24
7.03	5.88	5.4	7.9	6.85	5.18	6.86	6.03	5.22	6.55	7.66	6.26	5.49	7.21	6.2	5.83	7.65	5.65	6.09	6.17	6.31
7.08	5.96	5.49	7.9	6.95	5.18	6.94	6.23	5.37	6.55	7.89	6.29	5.51	7.26	6.2	5.86	7.65	5.75	6.09	6.25	6.34
7.21	5.98	5.68	7.98	7.04	5.2	6.98	6.37	5.38	6.55	7.97	6.49	5.53	7.27	6.2	5.88	7.65	5.75	6.09	6.31	6.48
7.31	6.05	5.68	7.98	7.05	5.23	7	6.37	5.46	6.55	8.08	6.58	5.56	7.35	6.2	5.89	7.65	5.75	6.5	6.42	6.48
7.39	6.1	5.68	8.02	7.13	5.23	7.05	6.37	5.47	6.58	8.13	6.59	5.59	7.44	6.28	5.89	7.73	6.05	6.5	6.46	6.54

7.39	6.11	5.68	8.23	7.13	5.23	7.15	6.37	5.55	6.9	8.14	6.7	5.64	7.52	6.28	6.03	7.86	6.14	6.74	6.46	6.61
7.5	6.11	5.68	8.23	7.13	5.23	7.3	6.37	5.62	6.9	8.26	6.89	5.72	7.65	6.7	6.11	7.88	6.14	6.8	6.46	6.75
7.68	6.25	5.68	8.23	7.31	5.23	7.32	6.37	5.62	6.9	8.32	6.97	5.72	7.65	6.7	6.11	7.88	6.14	6.82	6.46	6.75
7.71	6.3	5.68	8.23	7.32	5.4	7.32	6.37	5.63	7.04	8.42	7.05	5.92	7.65	6.7	6.19	8.13	6.14	6.82	6.6	6.76
7.71	6.39	5.92	8.31	7.32	5.46	7.32	6.37	5.65	7.07	8.47	7.05	5.97	7.77	6.7	6.25	8.13	6.29	6.82	6.74	6.76
7.78	6.4	5.92	8.4	7.32	5.52	7.55	6.41	5.69	7.17	8.55	7.05	6.01	7.96	6.7	6.27	8.13	6.29	6.82	6.75	6.8
7.86	6.47	6.2	8.47	7.32	5.52	7.55	6.41	5.71	7.17	8.72	7.17	6.01	7.96	6.7	6.28	8.13	6.48	6.82	6.85	6.96
7.89	6.47	6.2	8.53	7.56	5.79	7.55	6.48	5.72	7.17	8.74	7.23	6.07	7.96	6.74	6.34	8.16	6.48	6.91	6.85	7.11
7.98	6.47	6.2	8.63	7.56	5.79	7.59	6.58	5.74	7.28	8.76	7.25	6.07	7.96	6.75	6.35	8.31	6.48	7.05	6.85	7.16
8.11	6.6	6.22	8.78	7.56	5.79	7.84	6.58	5.81	7.43	8.77	7.3	6.17	8.01	6.78	6.45	8.31	6.48	7.23	6.85	7.26
8.21	6.6	6.22	8.78	7.57	5.79	7.84	6.58	5.85	7.43	8.94	7.3	6.26	8.29	6.79	6.45	8.47	6.48	7.23	6.85	7.28
8.21	6.6	6.22	8.78	7.66	5.79	7.84	6.58	5.97	7.43	8.99	7.3	6.3	8.37	6.98	6.45	8.78	6.48	7.23	7.21	7.35
8.23	6.6	6.22	8.9	7.85	5.79	8.03	6.58	5.97	7.43	9	7.3	6.35	8.53	7.09	6.49	8.78	6.48	7.23	7.29	7.43
8.23	6.68	6.29	9.02	7.93	5.79	8.04	6.58	5.98	7.43	9	7.5	6.35	8.53	7.09	6.56	8.78	6.48	7.23	7.36	7.43
8.42	6.81	6.31	9.04	7.93	5.79	8.06	6.58	5.98	7.43	9	7.62	6.4	8.53	7.09	6.56	8.78	6.48	7.23	7.42	7.43
8.49	6.9	6.31	9.06	7.93	6.06	8.06	6.7	5.98	7.43	9.14	7.64	6.4	8.7	7.36	6.6	8.78	6.48	7.5	7.42	7.56
8.56	6.96	6.31	9.08	7.94	6.06	8.2	6.7	5.99	7.43	9.24	7.64	6.41	8.7	7.36	6.6	8.78	6.48	7.53	7.42	7.71
8.59	6.99	6.39	9.28	7.94	6.06	8.31	6.7	5.99	7.48	9.28	7.64	6.41	8.7	7.36	6.61	8.78	6.48	7.53	7.49	7.8
8.7	7.01	6.41	9.28	8.04	6.06	8.47	6.77	6.03	7.92	9.32	7.68	6.41	8.91	7.36	6.66	8.78	6.48	7.53	7.5	7.81
8.72	7.12	6.6	9.28	8.05	6.13	8.51	6.8	6.11	7.92	9.48	7.69	6.41	9	7.36	6.69	8.78	6.48	7.57	7.5	7.87
8.85	7.17	6.71	9.34	8.22	6.19	8.51	7.06	6.11	7.92	9.48	7.76	6.49	9.02	7.36	6.79	8.78	6.48	8.11	7.61	7.87
8.98	7.22	6.71	9.38	8.22	6.4	8.51	7.09	6.12	8.12	9.61	7.83	6.53	9.09	7.36	6.85	8.78	6.48	8.16	7.69	7.97
8.98	7.23	6.97	9.47	8.22	6.4	8.6	7.09	6.22	8.54	9.65	7.99	6.61	9.1	7.47	6.86	8.78	6.48	8.16	7.69	7.98
8.98	7.23	6.97	9.61	8.22	6.4	8.61	7.09	6.29	8.54	9.65	7.99	6.63	9.1	7.47	6.86	8.82	6.48	8.16	7.69	7.99
9.05	7.23	6.97	9.61	8.23	6.4	8.7	7.15	6.3	8.54	9.76	7.99	6.64	9.1	7.53	6.93	8.82	6.48	8.16	7.69	8.16
9.05	7.23	6.97	9.61	8.23	6.4	8.89	7.15	6.3	8.54	9.76	7.99	6.64	9.28	7.59	6.98	9.15	6.48	8.2	7.69	8.22
9.21	7.23	6.97	9.61	8.38	6.42	8.89	7.15	6.3	8.54	9.76	8.08	6.71	9.34	7.61	7.06	9.15	6.48	8.2	7.93	8.22
9.21	7.24	6.97	9.61	8.62	6.42	8.94	7.15	6.3	8.54	9.76	8.14	6.79	9.34	7.75	7.06	9.15	6.48	8.27	7.94	8.23
9.25	7.42	7.03	9.64	8.62	6.49	8.94	7.15	6.3	8.54	9.88	8.15	6.81	9.34	7.75	7.06	9.15	6.48	8.29	8.09	8.23
9.25	7.44	7.05	9.67	8.62	6.49	8.94	7.32	6.3	8.54	9.92	8.16	6.81	9.34	7.79	7.07	9.15	6.48	8.29	8.09	8.24
9.26	7.44	7.05	9.7	8.62	6.49	8.94	7.32	6.3	9.12	9.96	8.25	6.82	9.34	7.79	7.07	9.15	6.48	8.29	8.09	8.25
9.29	7.44	7.05	9.7	8.63	6.49	9.13	7.32	6.3	9.12	9.99	8.26	6.82	9.34	7.79	7.07	9.15	6.52	8.29	8.09	8.29
9.3	7.45	7.05	9.78	8.63	6.49	9.13	7.32	6.3	9.12	10	8.26	6.82	9.34	7.91	7.07	9.15	6.52	8.29	8.09	8.29
9.43	7.54	7.22	9.78	8.75	6.59	9.17	7.42	6.35	9.12	10.01	8.26	7	9.53	7.91	7.08	9.15	6.52	8.29	8.09	8.49
9.43	7.6	7.22	9.91	8.77	6.79	9.17	7.46	6.35	9.12	10.01	8.4	7	9.53	7.91	7.2	9.48	6.55	8.29	8.09	8.57
9.43	7.61	7.22	9.91	8.77	6.79	9.17	7.46	6.36	9.12	10.01	8.5	7	9.53	7.95	7.32	9.48	6.55	8.29	8.22	8.57
9.43	7.61	7.22	9.91	8.77	6.83	9.21	7.46	6.42	9.12	10.14	8.5	7.01	9.53	8	7.32	9.48	6.64	8.29	8.22	8.58
9.53	7.68	7.22	9.91	8.88	6.83	9.23	7.46	6.42	9.12	10.14	8.5	7.01	9.53	8.17	7.32	9.48	6.66	8.29	8.22	8.58
9.53	7.68	7.22	9.91	8.89	6.83	9.23	7.46	6.52	9.12	10.14	8.5	7.16	9.56	8.27	7.33	9.48	6.68	8.3	8.22	8.69
9.53	7.68	7.22	9.91	9.03	6.83	9.23	7.46	6.52	9.12	10.14	8.5	7.17	9.58	8.36	7.49	9.48	6.79	8.3	8.22	8.7
9.63	7.69	7.23	10.09	9.03	6.83	9.34	7.49	6.61	9.12	10.14	8.55	7.17	9.66	8.38	7.52	9.48	6.82	8.53	8.43	8.71
9.63	7.69	7.25	10.1	9.03	6.83	9.38	7.49	6.61	9.12	10.24	8.57	7.25	9.67	8.39	7.53	9.48	6.89	8.53	8.43	8.71
9.63	7.76	7.26	10.12	9.03	6.83	9.38	7.49	6.61	9.12	10.24	8.57	7.25	9.7	8.59	7.54	9.48	6.89	8.58	8.43	8.76
9.63	7.76	7.26	10.12	9.03		9.41	7.49	6.61	9.16	10.25	8.58	7.25		8.62	7.54	9.5	6.89	8.65	8.43	8.76
9.63	7.76	7.36	10.12	9.03		9.45	7.55	6.76	9.16	10.27	8.7	7.25		8.62	7.54	9.5	6.9	8.72	8.43	8.77
9.69	7.88	7.36	10.15	9.03		9.47	7.68	6.77	9.16	10.27	8.7	7.26		8.62	7.55	9.5	6.9	8.72	8.43	8.93
9.69	7.89	7.36	10.15	9.04		9.47	7.74	6.77	9.34	10.27	8.7	7.42		8.62	7.55	9.5	6.9	8.73	8.56	9.01
9.69	7.89	7.69	10.19	9.11		9.47	7.74	6.77	9.47	10.48	8.7	7.43		8.62	7.55	9.5	7.02	8.73	8.56	9.01
9.69	7.89	7.69	10.26	9.12		9.7	7.74	6.77	9.91	10.61	8.83	7.43		8.62	7.55	9.5	7.02	8.73	8.72	9.01
9.8	7.99	7.69	10.28	9.12		9.7	7.74	6.85	9.93	10.68	8.89	7.44		8.62	7.67	9.51	7.04	8.92	8.73	9.01
9.8	8.04	7.69	10.28	9.13		9.7	7.74	6.85	10.12	10.68	8.89	7.44		8.62	7.76	9.65	7.07	9.13	8.73	9.01
9.8	8.04	7.69	10.28	9.27		9.7	7.85	6.85	10.12	10.68	8.89	7.56		8.62	7.76	9.78	7.07	9.13	8.8	9.01
9.8	8.05	7.69	10.28	9.33		9.87	7.85	7.06	10.12	10.68	8.89	7.57		8.62	7.79	9.78	7.07	9.16	8.8	9.08
9.93	8.05	7.69	10.49	9.34		9.87	7.85	7.06	10.12	10.68	8.9	7.57		9.04	8.22	9.78	7.09	9.16	8.8	9.1
9.93	8.18	7.69	10.49	9.34		9.87	7.85	7.07	10.12	10.68	8.91	7.57		9.04	8.31	9.78	7.15	9.16	8.91	9.1
9.94	8.18	7.69	10.51	9.35		9.87	7.85	7.25	10.12	10.69	9.12	7.58		9.08	8.31	9.78	7.15	9.16	9.05	9.1
9.94	8.19	7.75	10.52	9.36		9.87	7.92	7.26	10.12	10.87	9.16	7.58		9.08	8.31	9.91	7.17	9.29	9.05	9.11
10.13	8.25	7.77	10.52	9.36		9.87	7.93	7.26	10.12	10.97	9.16	7.59		9.08	8.31	9.91	7.21	9.44	9.09	9.26
10.14	8.27	7.81	10.52	9.47		10.02	7.93	7.35	10.12	10.97	9.19	7.59		9.14	8.31	10.14	7.27	9.44	9.11	9.26
10.23	8.29	7.9	10.54	9.48		10.08	8.06	7.35	10.12	10.97	9.19	7.62		9.14	8.31	10.17	7.27	9.44	9.2	9.26
10.34	8.29	7.91	10.68	9.49		10.09	8.16	7.35	10.12	11.08	9.25	7.74		9.2	8.31	10.19	7.37	9.44	9.26	9.32
10.38	8.36	7.98	10.69	9.58		10.13	8.16	7.46	10.12	11.11	9.35	7.77		9.47	8.31	10.23	7.45	9.63	9.28	9.41
10.5	8.36	8	10.95	9.59		10.18	8.16	7.58	10.12	11.21	9.36	7.8		9.47	8.31	10.61	7.47	9.72	9.37	9.52
10.62	8.43	8	10.95	9.6		10.19	8.23	7.58	10.12	11.29	9.45	7.83		9.54	8.31	10.61	7.49	9.72	9.37	9.52
10.62	8.49	8	10.95	9.6		10.25	8.27	7.66	10.12	11.56	9.56	7.83		9.54	8.33	10.61	7.58	9.72	9.44	9.62
10.74	8.54	8.12	10.95	9.75		10.27	8.63	7.72	10.12	11.69	9.6	7.83		9.54	8.36	10.61	7.67	9.9	9.48	9.63
10.81	8.59	8.2	10.97	9.76		10.27	8.63	7.76	10.12	11.74	9.71	7.96		9.54	8.53	10.61	7.67	10.07	9.59	9.78
10.93	8.7	8.28	11.05	9.76		10.47	8.63	7.87	10.12	11.8	9.71	7.97		9.67	8.53	10.61	7.67	10.07	9.65	9.78
10.93	8.71	8.28	11.13	9.83		10.5	8.63	7.87	10.12	11.8	9.8	7.97		9.82	8.53	10.61	7.68	10.07	9.65	9.9
11.08	8.77	8.28	11.21	9.9		10.5	8.63	7.87	10.12	11.82	9.81	7.97		9.82	8.53	10.65	8.25	10.07	9.65	9.91
11.23	8.77	8.28	11.31	9.9		10.74	8.63	7.94	10.12	11.88	9.85	7.97		9.82	8.68	10.65	8.25	10.07	9.76	10.05
11.31	8.77	8.35	11.46	9.98		10.78	8.63	8	10.6	11.93	9.85	7.98		9.82	8.68	10.65	8.25	10.21	9.82	10.09
11.38	8.77	8.5	11.53	10.13		10.85	8.63	8.05	10.6	12.06	9.95	8.1		9.82	8.68	10.9	8.25	10.48	9.99	10.17
11.42	8.77	8.54	11.6	10.13		10.95	8.72	8.07	10.62	12.17	10.04	8.24		9.82	8.7	11.05	8.25	10.5	10.02	10.22
11.45	8.85	8.54	11.65	10.17		11.07	8.84	8.11	10.62	12.27	10.14	8.33		9.89	8.75	11.05	8.25	10.51	10.09	10.3
11.5	8.94	8.57	11.8	10.18		11.07	8.92	8.11	10.68	12.3	10.26	8.36		10.03	8.87	11.06	8.25	10.52	10.26	10.36
11.53	9.02	8.59	11.8	10.18		11.07	8.97	8.17	10.68	12.3	10.26	8.41		10.14	8.87	11.2	8.25	10.52	10.26	10.39
11.6	9.14	8.62	11.81	10.3		11.07	9.13	8.29	10.97	12.32	10.32	8.41		10.19	8.9	11.37	8.25	10.72	10.26	10.47
11.65	9.22	8.73	12.03	10.41		11.07	9.13	8.29	11.16	12.45	10.38	8.41		10.35	9.08	11.41	8.25	11.01	10.26	10.49
11.65	9.27	8.73	12.03	10.41		11.17	9.17	8.55	11.18	12.48	10.38	8.42		10.35	9.08	11.41	8.25	11.28	10.34	10.52
11.78	9.28	8.73	12.03	10.46		11.17	9.21	8.65	11.4	12.48	10.38	8.42		10.35	9.08	11.41	8.25	11.28	10.34	10.62
11.81	9.28	8.73	12.03	10.5		11.22	9.27	8.65	11.4	12.51	10.42	8.51		10.35	9.15	11.41	8.51	11.28	10.46	10.7
11.86	9.28	8.73	12.03	10.51		11.44	9.27	8.65	11.4	12.56	10.44	8.61		10.35	9.22	11.41	8.51	11.28	10.51	10.82
11.98	9.29	8.96	12.03	10.6		11.47	9.27	8.66	11.4	12.59	10.44	8.67		10.35	9.24	11.41	8.51	11.28	10.52	10.87
12.09	9.47	9.01	12.03	10.61		11.57	9.27	8.66	11.4	12.6	10.57	8.68		10.4	9.3	11.41	8.51	11.28	10.63	10.87
12.1	9.55	9.08	12.25	10.72		11.57	9.37	8.73	11.65	12.64	10.63	8.68		10.63	9.3	11.41	8.51	11.28	10.64	11
12.17	9.61	9.23	12.36	10.73		11.57	9.41	8.79	11.69	12.74	10.63	8.68		10.87	9.3	11.41	8.51	11.28	10.74	11
12.17	9.69	9.23	12.36	10.77		11.57	9.41	8.86	11.69	12.78	10.64	8.68		10.87	9.4	11.41	8.56	11.28	10.86	11
12.24	9.75	9.23	12.36	10.78		11.76	9.41	8.86	11.69	12.87	10.71	8.8		10.87	9.51	11.57	8.62	11.28	10.86	11.07
12.28	9.75	9.23	12.51	10.78		11.91	9.41	8.86	11.69	12.9	10.77	8.86		10.87	9.51	11.59	8.62	11.28	10.86	11.08
12.29	9.75	9.23	12.59	10.79		11.95	9.56	8.87	11.69	13.05	10.78	8.86		10.94	9.51	11.67	8.62	11.28	10.86	11.22
12.29	9.75	9.23	12.62	10.93		12.04	10.14	8.87	11.69	13.08	10.78	8.87		11	9.68	11.83	8.65	11.44	10.89	11.25
12.35	9.9	9.26	12.62	11		12.04	10.14	8.95	11.69	13.08	10.92	8.9		11	9.73	11.83	8.65	11.56	11.11	11.25
12.35	9.91	9.33	12.62	11		12.04	10.14	8.95	11.69	13.13	11.01	8.96		11	9.74	11.89	8.68	11.56	11.11	11.25
12.35	9.91	9.33	12.65	11.01		12.04	10.14	8.95	11.69	13.18	11.01	8.96		11	9.74	11.89	8.84	11.56	11.11	11.25
12.35	9.92	9.33	12.65	11.01		12.04	10.14	9.05	11.69	13.18	11.01	8.96		11	9.74	11.89	8.86	11.61	11.11	11.26
12.5	10.03	9.33	12.65	11.08		12.17	10.14	9.05	11.69	13.18	11.01	8.97		11.1	9.8	11.89	8.86	11.61	11.11	11.36
12.5	10.04	9.38	12.79	11.17		12.17	10.14	9.05	11.78	13.25	11.12	8.97		11.2	9.84	12	8.86	11.61	11.11	11.36
12.5	10.04	9.45	12.85	11.17		12.22	10.14	9.12	11.78	13.25	11.12	9.03		11.2	9.84	12	8.86	11.69	11.11	11.36
12.5	10.04	9.5	12.94	11.17		12.24	10.14	9.13	11.78	13.3	11.12	9.05		11.2	9.84	12	8.86	11.76	11.15	11.47
12.5	10.04	9.5	12.94	11.18		12.24	10.14	9.19	12.07	13.31	11.12	9.05		11.2	9.95	12	8.9	11.76	11.16	11.47
12.61	10.05	9.6	12.94	11.24		12.24	10.14	9.19	12.09	13.4	11.15	9.05		11.32	9.96	12	8.9	11.76	11.17	11.48
12.61	10.1	9.6	12.94	11.24		12.24	10.14	9.19	12.22	13.41	11.15	9.06		11.35	9.99	12	8.92	11.76	11.2	11.49
12.65	10.14	9.6	12.94	11.25		12.25	10.14	9.2	12.22	13.47	11.21	9.29		11.39	10.22	12	8.97	11.76	11.21	11.5
12.78	10.14	9.6	12.97	11.36		12.45	10.14	9.2	12.35	13.47	11.21	9.29		11.39	10.32	12	8.97	11.9	11.21	11.5
12.78	10.14	9.6	12.99	11.37		12.45	10.14	9.2	12.39	13.49	11.23	9.29		11.5	10.45	12.04	8.97	11.9	11.38	11.62
12.78	10.14	9.6	13.18	11.37		12.45	10.14	9.25	12.39	13.55	11.23	9.29		11.5	10.45	12.04	9.01	11.93	11.42	11.63
12.93																		11.93	11.42	11.63
12.93																		11.93	11.42	11.69
12.93																		11.93	11.42	11.69

Box-1	Box-3	Box-4	Box-5	Box-2	Box-3	Box-1	Box-2	Box-3	Box-8	Box-7	Box-5	Box-6	Box-6	Box-8	Box-8	Box-6	Box-5	Box-1	Box-2	Box-6	Box-1	Box-2	Box-4	Box-6	Box-7
1	1	1	1	2	2	5	6	6	8	9	11	1	2	2	6	6	8	9	9	9	11	11	11	11	1
6.1	9.4	10.1	11.6	16.4	17.4	28.1	41	42	53.2	71	82	12.1	23.3	27.5	37	46	50.1	62	63	69	74	76	81	83	1.1
M	M	M	M	M	M	M	M	M	M	M	M	M	M	M	M	M	M	M	M	M	M	M	M	M	M
EtOH	EtOH	EtOH	EtOH	EtOH	EtOH	EtOH	EtOH	EtOH	EtOH	EtOH	EtOH	H2O	H2O	H2O	H2O	H2O	H2O	H2O	H2O	H2O	H2O	H2O	H2O	H2O	Malto
ME	ME	ME	ME	ME	ME	ME	ME	ME	ME	ME	ME	MH	MH	MH	MH	MH	MH	MH	MH	MH	MH	MH	MH	MH	MM
0	0	0	0	0	0	0	0	0	0	0	0	0	0	0	0	0	0	0	0	0	0	0	0	0	0
0	0	0	0	0.03	0	0.01	0	0	0	0	0	0	0	0.01	0	0.03	0.02	0	0	0	0	0	0.19	0	0.21
0.05	0.02	0.01	0	0.04	0	0.02	0.19	0.08	0	0	0	0	0	0.01	0.17	0.05	0.02	0	0	0	0.01	0.01	0.4	0	0.21
0.12	0.08	0.01	0	0.05	0	0.21	0.19	0.13	0	0	0	0	0	0.01	0.25	0.06	0.02	0	0	0	0.05	0.02	0.55	0	0.34
0.17	0.18	0.01	0	0.06	0.07	0.35	0.3	0.22	0	0	0	0	0.15	0.23	0.25	0.1	0.02	0	0	0	0.06	0.02	0.62	0	0.67
0.28	0.34	0.01	0	0.07	0.09	0.47	0.44	0.22	0	0	0	0.08	0.41	0.52	0.25	0.3	0.02	0	0	0	0.14	0.02	0.77	0	0.98
0.37	0.56	0.02	0	0.08	0.1	0.65	0.47	0.5	0	0	0	0.18	0.41	0.53	0.32	0.41	0.02	0	0.07	0.01	0.14	0.02	0.78	0	1.14
0.64	0.57	0.23	0	0.09	0.1	0.75	0.47	0.51	0	0	0	0.55	0.42	0.55	0.44	0.64	0.03	0	0.2	0.01	0.14	0.02	0.87	0	1.6
0.66	0.92	0.6	0	0.1	0.1	0.77	0.74	0.69	0	0	0	0.8	0.5	0.63	0.58	0.78	0.03	0.22	0.35	0.03	0.14	0.02	0.87	0	1.94
0.96	1.15	0.91	0	0.14	0.1	1	0.74	0.81	0	0	0	0.81	0.55	0.63	0.63	0.85	0.03	0.74	0.56	0.06	0.14	0.02	0.87	0	2.07
0.99	1.27	1.11	0	0.14	0.11	1	0.92	0.89	0	0	0	1.08	0.6	0.64	0.88	1.03	0.56	1.17	0.84	0.35	0.14	0.02	0.87	0	2.34
1.08	1.52	1.4	0	0.2	0.11	1.2	1.07	0.89	0	0	0	1.23	0.81	0.64	0.93	1.22	0.56	1.24	1.08	0.54	0.21	0.02	0.87	0	2.68
1.31	1.68	1.56	0	0.24	0.11	1.29	1.22	1.15	0	0	0	1.4	0.94	0.91	1.04	1.58	0.56	1.35	1.27	0.69	0.22	0.02	0.87	0	2.88
1.31	1.7	1.72	0.07	0.25	0.27	1.3	1.43	1.15	0.22	0	0	1.54	1.18	1.09	1.09	1.61	0.77	1.55	1.36	0.91	0.37	0.02	0.87	0	2.88
1.32	1.91	1.94	0.07	0.34	0.45	1.45	1.5	1.55	0.48	0	0	1.54	1.31	1.09	1.2	1.84	0.77	1.71	1.54	1.05	0.4	0.02	0.87	0	2.89
1.34	1.91	2.18	0.07	0.45	0.57	1.45	1.65	1.75	0.5	0	0	1.63	1.4	1.09	1.4	2.13	0.96	1.86	1.61	1.25	0.65	0.02	0.87	0	2.94
1.53	1.93	2.18	0.07	0.49	0.61	1.54	1.68	1.75	0.85	0	0.1	1.83	1.74	1.09	1.6	2.13	1.24	2.13	1.73	1.47	0.83	0.02	0.87	0	3.27
1.56	2.08	2.35	0.07	0.49	0.61	1.7	1.68	1.75	0.89	0	0.11	1.83	1.74	1.09	1.61	2.14	1.51	2.39	2.04	1.68	1.24	0.02	0.87	0	3.51
1.56	2.32	2.36	0.07	0.49	0.61	1.71	1.83	1.76	0.95	0	0.17	1.83	1.74	1.09	1.67	2.2	1.76	2.39	2.04	1.73	1.24	0.02	0.87	0.03	3.59
1.85	2.32	2.36	0.81	0.49	0.61	1.76	2.01	1.82	0.95	0	0.3	1.95	1.79	1.36	1.72	2.46	1.86	2.39	2.04	1.95	1.24	0.02	1.02	0.7	3.6
2.05	2.46	2.56	1.11	0.49	0.64	2.1	2.08	1.97	1.41	0.57	0.43	2.27	1.89	1.79	1.76	2.62	1.99	2.39	2.1	1.95	1.24	0.02	1.46	1.07	3.6
2.12	2.48	2.89	1.13	0.49	0.64	2.1	2.08	1.97	1.41	1.06	0.64	2.49	2.13	1.79	2.1	2.73	2.25	2.46	2.42	1.95	1.24	0.3	2.87	1.32	3.73
2.42	2.5	3.15	1.13	0.64	0.65	2.27	2.17	2.2	1.5	1.07	0.95	2.49	2.39	1.8	2.18	2.73	2.28	2.49	2.42	1.99	1.39	0.47	2.87	1.58	3.74
2.51	2.51	3.19	1.13	0.64	0.65	2.29	2.37	2.2	1.5	1.23	0.95	2.53	2.39	1.81	2.18	2.89	2.77	2.5	2.42	1.99	1.68	0.49	2.87	1.75	3.82
2.58	2.51	3.31	1.13	0.65	0.65	2.29	2.47	2.21	1.5	1.23	0.98	2.53	2.39	1.81	2.18	3.12	2.77	2.59	2.42	1.99	1.73	0.49	2.87	2.09	3.83
2.59	2.52	3.31	1.13	0.65	0.65	2.3	2.48	2.36	1.5	1.24	0.98	2.61	2.39	1.92	2.18	3.34	2.77	2.94	2.43	2.04	1.82	0.49	2.87	2.13	3.83
2.59	2.63	3.31	1.13	0.65	0.65	2.39	2.48	2.36	1.5	1.24	1.08	2.73	2.39	1.92	2.29	3.4	2.78	2.94	2.43	2.26	1.95	0.49	2.87	2.19	3.83
2.71	2.91	3.39	1.13	0.65	0.65	2.39	2.49	2.37	1.68	1.24	1.15	2.74	2.39	1.93	2.31	3.5	2.9	3.26	2.73	2.53	2.1	0.7	2.87	2.31	3.99
2.71	2.94	3.47	1.13	0.65	0.65	2.39	2.51	2.37	1.88	1.31	1.16	2.74	2.39	1.93	2.31	3.63	3.09	3.42	2.73	2.53	2.1	0.74	2.87	2.55	4.09
2.71	3.12	3.63	1.13	0.65	0.65	2.39	2.51	2.37	1.88	1.32	1.21	3.02	2.53	2.37	2.31	3.71	3.18	3.42	2.8	2.53	2.1	0.74	2.87	2.85	4.37
2.71	3.12	3.64	1.13	0.65	0.97	2.39	2.51	2.37	1.93	1.33	1.3	3.26	2.53	2.73	2.31	3.74	3.28	3.43	2.91	2.62	2.1	0.74	4.61	3.03	4.37
2.71	3.17	3.64	1.13	0.65	1.15	2.58	2.74	2.55	1.95	1.33	1.3	3.26	2.53	2.73	2.31	3.89	3.44	3.43	3.02	2.64	2.1	0.74	4.61	3.07	4.38
2.96	3.17	3.65	1.13	0.65	1.19	2.58	2.75	2.55	2.24	1.33	1.3	3.26	3.04	2.74	2.31	3.89	3.44	3.49	3.02	2.66	2.1	0.74	4.61	3.13	4.38
2.96	3.17	3.65	1.8	0.65	1.21	2.58	2.75	2.86	2.53	1.45	1.3	3.26	3.05	2.75	2.6	3.89	3.44	3.9	3.04	2.66	2.1	0.86	4.61	3.22	4.53
3.09	3.19	3.78	3.35	0.74	1.31	2.87	2.75	2.91	2.67	1.45	1.35	3.35	3.05	2.92	2.6	3.89	3.44	3.91	3.04	3.01	2.1	1.14	4.61	3.43	4.91
3.14	3.19	3.97	3.35	0.75	1.47	2.87	2.75	2.91	2.67	1.46	1.57	3.43	3.1	3.17	2.6	4.06	3.44	3.91	3.04	3.03	2.1	1.31	4.61	3.46	5.01
3.14	3.29	3.97	3.35	0.94	1.63	2.87	2.79	2.91	2.67	1.46	1.62	3.43	3.1	3.36	2.6	4.16	3.44	3.91	3.04	3.03	2.1	1.31	4.61	3.46	5.01
3.15	3.29	3.98	3.35	1.25	1.67	2.87	2.99	2.91	2.67	1.93	1.64	3.43	3.32	3.36	2.6	4.17	3.44	3.91	3.17	3.05	2.1	1.31	4.61	3.46	5.01
3.29	3.29	4.01	3.35	1.52	1.67	2.87	2.99	2.92	2.87	1.93	1.65	3.52	3.32	3.37	2.63	4.18	3.44	3.91	3.41	3.05	2.1	1.31	4.61	3.46	5.14
3.3	3.31	4.01	3.35	1.79	1.67	2.88	2.99	2.92	3.12	1.93	1.65	3.57	3.32	3.37	2.63	4.19	3.44	3.91	3.41	3.05	2.1	1.31	4.61	3.46	5.15
3.38	3.61	4.05	3.35	2.16	1.91	3	2.99	3.08	3.16	1.93	1.65	3.58	3.37	3.37	2.63	4.2	3.44	4.08	3.41	3.05	2.13	1.38	4.61	3.46	5.15
3.38	3.77	4.23	3.35	2.21	1.98	3	3.08	3.08	3.16	1.94	1.65	3.7	3.37	3.37	2.63	4.25	3.44	4.12	3.43	3.06	2.16	1.45	4.61	3.53	5.32
3.39	3.77	4.23	4.46	2.21	1.99	3.01	3.18	3.08	3.16	1.94	1.65	3.7	3.37	3.37	2.7	4.44	3.44	4.12	3.43	3.06	2.16	1.45	4.61	3.64	5.32
3.39	3.77	4.23	4.46	2.21	2	3.03	3.18	3.08	3.26	1.94	1.65	3.7	3.73	3.37	2.91	4.72	3.44	4.12	3.43	3.06	2.21	1.46	4.61	3.64	5.32
3.57	3.77	4.23	4.46	2.21	2	3.03	3.18	3.08	3.3	1.99	1.67	3.77	3.73	3.37	2.91	4.72	3.44	4.15	3.44	3.06	2.21	1.5	4.61	3.65	5.33
3.57	3.78	4.23	4.46	2.21	2.05	3.04	3.18	3.08	3.36	2	1.76	3.78	3.73	3.38	2.94	4.72	3.44	4.21	3.45	3.06	2.76	1.5	4.61	3.65	5.33
3.58	3.81	4.24	4.46	2.21	2.18	3.04	3.18	3.2	3.36	2	1.85	3.82	3.73	3.62	3.02	4.72	3.66	4.27	3.45	3.06	2.76	1.67	4.61	3.67	5.33
3.58	3.83	4.24	4.46	2.21	2.18	3.1	3.18	3.21	3.36	2.01	1.85	3.83	3.73	3.67	3.11	4.75	3.94	4.28	3.5	3.14	2.76	1.68	4.61	3.71	5.4
3.59	3.83	4.3	4.46	2.21	2.19	3.1	3.18	3.21	3.36	2.01	1.85	3.95	3.73	3.67	3.45	4.75	3.94	4.31	3.51	3.23	2.76	1.68	4.61	3.78	5.4
3.81	3.84	4.46	4.46	2.21	2.19	3.11	3.24	3.21	3.36	2.02	1.85	3.96	3.73	3.67	3.59	4.75	3.96	4.31	3.51	3.24	2.76	1.68	4.61	3.78	5.41
3.81	3.97	4.46	4.46	2.21	2.2	3.12	3.26	3.21	3.36	2.2	1.85	3.96	3.94	3.67	3.59	4.75	4.04	4.31	3.52	3.24	2.76	1.68	4.61	3.78	5.46
3.9	3.97	4.47	4.46	2.21	2.2	3.12	3.26	3.22	3.36	2.21	1.85	3.96	3.94	3.86	3.59	4.86	4.12	4.31	3.52	3.24	2.76	1.68	4.61	3.78	5.47
3.91	4.01	4.47	4.46	2.21	2.2	3.13	3.26	3.25	3.42	2.21	1.85	3.96	4.05	3.9	3.59	4.86	4.12	4.5	3.57	3.24	2.76	1.68	4.61	3.82	5.47
3.91	4.01	4.55	4.46	2.24	2.2	3.13	3.26	3.4	3.56	2.22	1.85	3.96	4.05	4.06	3.59	4.86	4.12	4.5	3.62	3.24	2.76	1.68	4.61	3.82	5.47
3.92	4.01	4.55	5.21	2.24	2.2	3.3	3.36	3.41	3.71	2.56	1.85	3.96	4.05	4.06	3.59	4.86	4.12	4.5	3.62	3.24	2.76	1.68	4.61	3.82	5.72
3.92	4.05	4.55	5.51	2.24	2.2	3.3	3.36	3.41	3.71	2.56	1.85	4.04	4.06	4.06	3.59	4.86	4.12	4.5	3.62	3.24	2.76	1.68	4.61	3.82	5.72
4.02	4.06	4.56	5.51	2.24	2.4	3.31	3.36	3.41	3.71	2.57	1.85	4.09	4.06	4.12	3.59	4.87	4.15	4.5	3.62	3.46	2.76	1.68	4.61	3.86	5.72
4.03	4.06	4.56	5.67	2.24	2.5	3.55	3.36	3.41	3.71	2.57	1.85	4.1	4.06	4.12	3.59	5.05	4.15	4.84	3.64	3.47	2.76	1.68	4.61	3.86	5.93
4.03	4.06	4.72	5.67	2.24	2.51	3.56	3.36	3.42	3.71	2.58	1.89	4.11	4.06	4.12	3.59	5.18	4.25	4.84	3.64	3.47	2.76	1.68	4.61	3.88	5.93
4.04	4.06	4.73	5.67	2.27	2.51	3.57	3.46	3.42	3.72	2.75	2.33	4.15	4.06	4.14	3.59	5.19	4.42	4.84	3.64	3.47	2.76	1.68	4.61	4.01	5.93
4.04	4.07	4.73	5.67	2.27	2.52	3.57	3.48	3.42	3.72	2.88	2.33	4.22	4.39	4.47	3.59	5.19	4.42	4.85	3.65	3.47	2.76	1.69	4.61	4.01	5.93
4.12	4.09	4.73	5.67	2.27	2.53	3.57	3.49	3.42	3.72	2.89	2.43	4.22	4.39	4.75	3.59	5.23	4.42	4.86	3.93	3.48	2.76	1.78	4.61	4.01	6.14
4.12	4.09	4.89	5.67	2.28	2.53	3.6	3.49	3.42	3.72	2.89	2.43	4.22	4.4	4.75	3.59	5.23	4.42	5.07	3.98	3.74	2.76	1.8	4.61	4.01	6.24
4.12	4.14	4.96	5.67	2.44	2.94	3.76	3.49	3.6	3.77	2.9	2.43	4.24	4.58	4.75	3.59	5.27	4.42	5.07	3.98	3.75	2.76	1.85	4.61	4.18	6.24
4.13	4.15	4.96	5.99	2.69	2.95	3.76	3.62	3.6	3.84	2.91	2.43	4.36	4.62	4.75	3.64	5.29	4.93	5.07	3.98	3.75	2.76	1.88	4.61	4.18	6.25
4.13	4.15	4.96	6.07	2.78	2.95	3.77	3.64	3.61	4.01	3.07	2.43	4.36	4.62	4.75	3.75	5.29	5.1	5.07	3.99	3.75	2.76	1.9	4.61	4.18	6.25
4.13	4.15	5.11	6.07	2.8	2.95	3.78	3.65	3.61	4.01	3.07	2.5	4.36	4.62	4.75	3.75	5.65	5.23	5.17	4.05	3.91	2.76	1.9	4.61	4.18	6.25
4.19	4.15	5.11	6.07	2.81	2.97	3.85	3.82	4.01	4.08	3.08	2.5	4.38	4.62	4.75	4.05	5.65	5.23	5.17	4.05	3.96	2.76	1.9	4.61	4.18	6.51
4.29	4.15	5.18	6.4	2.81	3.11	3.95	3.87	4.02	4.08	3.31	2.5	4.42	4.62	5.02	4.05	5.65	5.23	5.24	4.05	4	2.76	2.12	4.61	4.18	6.69
4.35	4.44	5.26	6.4	2.81	3.16	4.18	3.98	4.15	4.08	3.44	2.5	4.52	4.62	5.11	4.05	5.65	5.23	5.4	4.37	4.14	2.76	2.44	4.61	4.18	6.69
4.46	4.7	5.42	6.4	2.87	3.39	4.21	4.2	4.15	4.31	3.48	2.5	4.65	4.75	5.13	4.17	5.71	5.23	5.47	4.45	4.28	2.76	2.74	4.61	4.18	6.95
4.62	4.86	5.53	6.4	2.99	3.53	4.42	4.23	4.31	4.31	3.54	2.5	4.77	4.89	5.14	4.17	5.71	5.23	5.62	4.45	4.57	2.76	2.94	4.61	4.18	7.13
4.83	5.16	5.56	6.4	3.08	3.59	4.49	4.52	4.31	4.31	3.54	2.5	4.89	5.01	5.54	4.17	5.88	5.23	5.68	4.74	4.58	2.76	3.22	4.61	4.18	7.45
4.83	5.33	5.75	6.4	3.11	3.59	4.6	4.52	4.55	4.31	3.56	2.5	5.12	5.23	5.88	4.17	5.95	5.23	5.86	4.91	4.58	2.76	3.35	4.61	4.21	7.76
4.9	5.5	5.77	6.4	3.27	3.6	4.72	4.73	4.59	4.82	3.61	2.52	5.2	5.31	5.96	4.17	6.17	5.23	5.86	5.1	4.69	2.76	3.46	4.61	4.43	8.16
4.95	5.57	5.97	6.4	3.66	3.85	4.72	4.95	4.79	4.91	3.61	2.69	5.22	5.61	6.03	4.41	6.51	5.23	5.99	5.1	4.89	2.89	3.6	4.61	4.64	8.17
5.05	5.57	6.11	6.4	3.9	3.97	4.72	5.05	4.79	5	3.7	2.98	5.53	5.82	6.27	4.47	6.51	5.28	5.99	5.73	4.89	3.2	3.75	4.61	4.76	8.32
5.05	6.02	6.3	6.4	3.91	4.03	4.79	5.33	4.92	5.42	3.84	2.98	5.64	5.82	6.27	4.64	6.52	5.32	5.99	5.73	4.9	3.2	3.75	4.61	4.86	8.65
5.12	6.06	6.37	6.4	4.16	4.03	4.97	5.37	4.92	5.47	3.98	3.02	5.66	5.82	6.35	4.67	6.52	5.51	6.07	5.73	5.21	3.2	3.75	4.61	4.91	8.93
5.27	6.08	6.49	6.4	4.43	4.03	5.06	5.38	5.06	5.54	4.04	3.03	5.68	5.82	6.65	5.13	6.69	5.6	6.31	6.19	5.49	3.38	3.75	4.61	5.07	9.04
5.27	6.44	6.55	6.4	4.48	4.21	5.2	5.6	5.06	5.54	4.05	3.08	5.81	6	6.71	5.16	6.84	5.69	6.31	6.4	5.57	3.44	3.97	4.61	5.21	9.04
5.44	6.44	6.6	6.4	4.48	4.34	5.3	5.73	5.06	5.57	4.05	3.09	5.81	6.14	6.72	5.17	6.97	5.8	6.45	6.4	5.57	3.49	4.28	4.61	5.25	9.05
5.48	6.44	6.86	6.4	4.48	4.36	5.31	5.77	5.06	5.69	4.05	3.16	5.98	6.14	6.72	5.37	7	5.94	6.71	6.4	5.57	3.52	4.3	4.84	5.51	9.26
5.74	6.44	6.98	6.4	4.6	4.67	5.31	5.77	5.32	5.69	4.05	3.43	6.14	6.14	6.72	5.37	7.18	6.2	6.75	6.4	5.83	3.73	4.4	4.87	5.8	9.5
5.81	6.44	7.14	6.4	4.71	4.91	5.31	5.97	5.48	5.69	4.05	3.62	6.31	6.27	6.72	5.37	7.24	6.2	6.95	6.4	5.83	4.12	4.61	5.1	5.8	9.59
6.03	6.66	7.25	6.41	4.71	4.95	5.4	5.97	5.48	5.77	4.2	3.62	6.31	6.28	7.19	5.56	7.4	6.34	7.01	6.4	6.03	4.12	4.63	5.1	5.8	9.59
6.16	6.66	7.25	6.51	4.92	4.96	5.58	5.97	5.48	5.79	4.46	3.95	6.31	6.57	7.33	5.57	7.46	6.44	7.19	6.4	6.25	4.12	4.72	6.44	5.8	9.59
6.16	6.66	7.34	6.56	5.06	4.96	5.58	6.09	5.48	5.84	4.56	4.19	6.49	6.85	7.34	5.65	7.56	6.49	7.21	6.4	6.41	4.12	4.72	6.44	5.84	9.94
6.17	6.97	7.4	6.56	5.07	5.17	5.67	6.2	5.48	6.28	4.56	4.29	6.5	6.97	7.34	5.81	7.62	6.54	7.55	6.48	6.42	4.45	4.72	6.44	5.84	9.94
6.47	7.06	7.52	6.56	5.12	5.18	5.89	6.21	5.6	6.7	4.57	4.53	6.51	6.97	7.34	5.9	7.65	6.6	7.75	6.65	6.42	4.88	4.72	6.44	6.01	10.06
6.47	7.07	7.52	6.56	5.2	5.22	5.89	6.22	5.6	6.86	4.57	4.59	6.83	6.97	7.34	5.9	7.89	6.6	7.75	6.92	6.42	4.88	4.95	6.44	6.16	10.06
6.58	7.07	7.53	6.56	5.41	5.28	5.89	6.45	5.74	6.9	4.7	4.73	6.87	6.97	7.35	5.9	7.89	6.62	7.75	7.08	6.42	4.89	4.99	6.44	6.36	10.32
6.58	7.23	7.63	6.56	5.41	5.3	6.04	6.46	5.93	6.9	4.71	4.93	6.89	7.19	7.35	5.9	7.99	7.09	7.75	7.13	6.42	4.91	4.99	6.44	6.7	10.53
6.76	7.23	7.7	6.56	5.41	5.3	6.05	6.47	5.94	6.9	4.72	4.93	6.91	7.19	7.35	5.9	8.07	7.19	7.75	7.13	6.43	4.91	4.99	6.44	6.77	10.53
6.85	7.25	7.75	6.56	5.42	5.3	6.45	6.47	6.19	6.9	5.09	4.94	7.15	7.31	7.69	5.9	8.21	7.19	7.75	7.14	6.51	4.92	5.01	6.44	6.77	10.54
7.03	7.28	7.85	6.56	5.42	5.3	6.48	6.5	6.19	6.9	5.09	5.13	7.27	7.31	7.79	5.9	8.25	7.19	7.75	7.15	6.88	4.96	5.03	6.44	6.77	10.54
7.11	7.28	7.85	6.56	5.71	5.3	6.49	6.61	6.3	6.9	5.09	5.13	7.27	7.32	7.83	5.9	8.38	7.19	7.75	7.15	6.88	4.96	5.03	6.44	6.77	10.66
7.12	7.65	7.95	6.77	5.97	5.33	6.49	6.67	6.3	6.9	5.09	5.13	7.34	7.32	7.84	5.9	8.42	7.21	7.75	7.15	6.88	4.96	5.03	6.44	6.77	10.66
7.12	7.66	8	6.77	5.99	5.35	6.6	6.68	6.51	6.9	5.1	5.13	7.34	7.38	8	5.9	8.52	7.24	7.98	7.17	6.89	5.19	5.57	6.44	7.05	10.79
7.28	8.01	8.01	6.77	6	5.66	6.67	6.68	6.51	6.95	5.41	5.13	7.66	7.65	8.27	6.04	8.58	7.24	8.18	7.54	6.89	5.59	5.63	6.44	7.65	10.95
7.32	8.11	8.15	6.77	6	5.76	6.67	6.69	6.51	6.95	5.42	5.13	7.67	7.65	8.29	6.04	8.65	7.41	8.18	7.54	6.95	5.74	6.07	6.44	7.74	11.01
7.32	8.18	8.15	6.77	6.03	5.77	6.69	6.84	6.51	7.08	5.42	5.13	7.68	7.65	8.29	6.04	8.77	7.44	8.18	7.54	6.95	5.74	6.25	6.44	7.93	11.01
7.33	8.18	8.16	6.77	6.43	6.21	6.69	6.84	6.52	7.44	5.43	5.13	7.7	7.65	8.29	6.12	8.83	7.51	8.18	7.54	6.95	5.76	6.25	6.44	7.93	11.01
7.34	8.18	8.16	6.77	6.8	6.23	6.69	6.86	6.52	7.44	5.67	5.13	7.71	7.82	8.29	6.16	8.83	7.51	8.18	7.54	6.97	5.76	6.25	6.44	7.93	11.11
7.34	8.21	8.16	6.77	6.87	6.24	6.71	6.87	6.52	7.76	5.67	5.13	7.72	7.82	8.29	6.21	8.83	7.51	8.28	7.54	6.97	5.82	6.37	6.44	7.93	11.11
7.34	8.32	8.29	6.77	6.87	6.26	6.71	6.87	6.52	7.84	5.68	5.13	7.72	7.82	8.29	6.28	8.83	7.51	8.29	7.54	6.97	5.9	6.4	6.44	7.93	11.11
7.35	8.32	8.29	6.77	6.87	6.53	6.71	6.87	6.52	7.98	5.69	5.26	7.72	7.82	8.29	6.38	8.83	7.51	8.29	7.72	6.97	5.95	6.4	6.44	7.93	11.2
7.35	8.32	8.29	6.77	6.87	6.62	6.71	6.88	6.64	7.98	5.69	5.31	7.72	7.82	8.47	6.89	8.83	7.51	8.53	7.75	7.06	5.95	6.4	6.44	7.93	11.2
7.36	8.32	8.35	6.77	6.87	6.63	6.71	7.02	6.72	7.98	5.81	5.37	7.72	7.82	8.5	6.89	8.83	7.54	8.53	7.75	7.07	5.95	6.4	6.44	7.93	11.2
7.44	8.32	8.43	6.77	6.87	6.64	6.73	7.02	6.72	7.98	5.82	5.37	7.76	7.82	8.5	6.89	9.09	7.54	8.53	7.75	7.08	5.95	6.4	6.44	7.93	11.33
7.55	8.32	8.43	6.77	6.87	6.64	6.8	7.02	6.72	7.98	5.82	5.37	7.77	7.82	8.51	6.89	9.09	7.57	8.53	7.75	7.08	5.95	6.4	6.44	7.93	11.34
7.55	8.32	8.43	6.77	6.97	6.64	6.8	7.03	6.73	7.98	5.83	5.38	7.77	7.82	8.52	6.89	9.09	7.57	8.6	7.75	7.08	5.95	6.59	6.44	7.93	11.34
7.56	8.46	8.43	6.77	6.99	6.64	6.8	7.03	6.73	7.98	5.83	5.4	7.77	7.83	8.74	6.89	9.09	7.57	8.63	7.75	7.09	5.95	6.62	6.44	7.93	11.5
7.6	8.46	8.58	7.08	6.99	6.64	6.8	7.03	6.73	8.05	5.84	5.43	7.92	7.91	8.74	6.89	9.09	7.57	8.63	7.85	7.24	5.95	6.64	6.44	7.93	11.5
7.63	8.46	8.58	7.16	6.99	6.88	6.8			8.41	6.05	5.47	7.96	7.95	8.75			7.68	8.63	7.86	7.24	5.95	6.64	6.44	7.93	11.5
7.63	8.46	8.58	7.21	6.99	7.02	6.8			8.41	6.05	5.47	7.96	7.95	8.76			7.78	8.64	7.88	7.24	6.08	6.66	6.44	7.93	11.5
7.64	8.46	8.59	7.21	7.24	7.03	6.8			8.41	6.06	5.52	7.96	7.95	8.77			7.78	8.66	7.88	7.45	6.21	6.66	6.44	7.93	11.5
7.64	8.5	8.59	7.21	7.26	7.03				8.41	6.07	5.52	8.03	8.09	9.06			7.78	8.68	7.88	7.45	6.35	6.74	6.44	7.93	11.5
7.65	8.51	8.62	7.21	7.26	7.03				8.41	6.27	5.53	8.05	8.09	9.23			7.78	8.68	7.88	7.45	6.35	6.82	6.44	7.93	11.5
7.73	8.51	8.62	7.21	7.26	7.03				8.41	6.43	5.53	8.05	8.09	9.29			7.78	8.68	7.88	7.45	6.35	6.86	6.44	7.93	11.67
7.75	8.51	8.62	7.21	7.52	7.04				8.41	6.44	5.53	8.05	8.18	9.3			7.78	8.68	7.88	7.46	6.35	6.9	6.44	7.95	11.67
7.75	8.58	8.62	7.29	7.53	7.04				8.41	6.45	5.53	8.05	8.18	9.3			7.78	8.7	7.94	7.52	6.35	6.9	6.44	8.15	11.67
8.12	8.58	8.62	7.29	7.55	7.04				8.41	6.45	5.53	8.23	8.19	9.3			7.78	8.7	7.94	7.6	6.35	6.9	6.44	8.26	11.67
8.16	8.58	8.64	7.29	7.55	7.32				8.61	6.65	5.58	8.26	8.19	9.3			7.78	8.7	7.94	7.6	6.35	6.9	6.44	8.32	11.68
8.18	8.7	8.8	7.29	7.55	7.51				8.68	6.66	5.62	8.26	8.19	9.31			7.78	8.7	7.94	7.6	6.35	6.9	6.44	8.32	11.99

8.18	8.88	8.9	7.29	7.82	7.52				8.68	6.8	5.67	8.26	8.36	9.65			7.78	8.7	7.95	7.6	6.35	6.9	6.44	8.32	12
8.19	8.88	8.93	7.29	7.95	7.52				8.68	6.81	5.67	8.26	8.45	9.8			7.78	8.97	7.95	7.84	6.48	6.9	6.44	8.32	12
8.2	8.88	8.95	7.29	7.96	7.55				8.68	6.81	5.72	8.26	8.45	9.81			7.84	8.97	8.13	7.89	6.54	6.9	6.44	8.32	12
8.22	8.88	8.96	7.29	8.02	7.56				8.68	6.82	5.72	8.52	8.45	9.81			7.85	8.97	8.17	7.9	6.54	6.9	6.44	8.32	12.1
8.23	8.89	8.97	7.29	8.02	7.56				8.68	7.02	5.72	8.53	8.46	9.81			7.85	8.97	8.18	7.9	6.54	6.9	6.44	8.32	12.1
8.24	9.02	9.07	7.29	8.05	7.61				8.68	7.02	5.72	8.53	8.68	9.82			7.85	8.97	8.18	7.9	6.54	6.9	6.44	8.32	12.1
8.34	9.02	9.08	7.29	8.09	7.62				9.03	7.03	5.72	8.68	8.69	10.12			8.11	8.98	8.18	7.98	6.54	6.9	6.44	8.32	12.1
8.43	9.02	9.08	7.29	8.13	7.63				9.13	7.03	6.07	8.74	8.69	10.24			8.11	9.06	8.18	8.16	6.73	6.9	6.44	8.32	12.27
8.48	9.17	9.14	7.29	8.14	7.93				9.14	7.1	6.07	8.82	8.69	10.26			8.27	9.3	8.18	8.17	6.75	7.03	7.45	8.54	12.49
8.66	9.19	9.28	7.29	8.2	8.13				9.15	7.17	6.07	8.82	8.69	10.5			8.42	9.34	8.49	8.18	6.87	7.03	7.45	8.6	12.66
8.8	9.39	9.4	7.29	8.5	8.18				9.25	7.45	6.07	9	8.93	10.54			8.57	9.35	8.5	8.43	6.96	7.26	7.45	8.6	12.89
8.94	9.44	9.52	7.29	8.74	8.5				9.52	7.59	6.11	9.14	9.01	10.56			8.67	9.57	8.5	8.43	7.22	7.43	7.45	8.67	13.03
9.07	9.53	9.68	7.29	8.9	8.62				9.64	7.91	6.23	9.23	9.01	10.74			8.68	9.57	9.09	8.43	7.47	7.6	7.45	8.7	13.38
9.08	9.75	9.84	8.05	8.94	8.68				9.91	8.03	6.24	9.47	9.27	10.75			8.81	9.62	9.14	8.66	7.91	7.6	7.45	8.7	13.51
9.17	9.75	10.09	8.05	9.08	8.68				9.98	8.04	6.53	9.65	9.37	11.29			8.82	9.89	9.14	8.78	7.91	7.6	7.45	8.7	13.8
9.34	9.95	10.17	8.05	9.24	8.68				10	8.05	6.64	9.65	9.37	11.49			8.82	9.91	9.21	8.83	7.91	7.79	7.45	8.73	13.92
9.34	9.98	10.23	8.05	9.33	8.69				10.35	8.13	6.75	9.78	9.56	11.61			8.97	9.92	9.59	9.17	7.91	7.99	7.45	8.75	14.04
9.58	10.43	10.28	9.2	9.5	8.69				10.54	8.13	6.91	9.81	9.65	11.61			9.17	10.2	9.59	9.17	7.91	7.99	7.45	8.84	14.25
9.65	10.56	10.39	9.33	9.72	8.94				10.54	8.13	6.91	9.81	9.66	11.64			9.35	10.2	9.59	9.17	8.02	8.13	7.45	8.96	14.37
9.65	10.57	10.48	9.33	10	9.06				10.55	8.14	7.09	9.9	9.66	11.64			9.49	10.38	9.59	9.33	8.74	8.16	7.45	8.96	14.38
9.9	10.58	10.68	9.33	10.05	9.31				10.73	8.32	7.22	10.19	9.74	12.18			9.65	10.52	9.72	9.41	8.74	8.16	7.45	9.09	14.39
10.04	10.64	10.82	9.33	10.05	9.31				10.73	8.39	7.39	10.29	9.88	12.21			9.76	10.79	10	9.44	8.74	8.19	7.45	9.17	14.64
10.24	10.69	10.92	9.33	10.05	9.31				10.73	8.39	7.4	10.59	9.88	12.22			9.76	10.81	10.1	9.79	8.74	8.43	7.45	9.25	14.7
10.36	10.69	11.09	9.33	10.14	9.44				11.17	8.45	7.48	10.59	9.88	12.23			9.87	10.98	10.1	9.79	8.74	8.57	7.45	9.28	14.7
10.52	10.7	11.22	9.33	10.16	9.53				11.52	8.45	7.65	10.59	10.07	12.23			9.98	10.98	10.29	9.91	8.74	8.67	7.45	9.42	15.03
10.59	10.7	11.24	9.33	10.25	9.54				11.53	8.45	7.66	10.6	10.09	12.25			10	10.98	10.29	9.92	8.74	8.87	7.45	9.48	15.03
10.62	10.75	11.41	9.33	10.25	9.54				11.53	8.45	7.67	10.84	10.09	12.25			10.27	11.47	10.38	9.92	8.74	8.87	8.51	9.75	15.03
10.62	10.75	11.42	9.33	10.55	9.59				11.53	8.45	7.67	10.84	10.09	12.27			10.27	11.52	10.71	9.98	9.03	8.87	8.51	10.11	15.27
10.79	11.09	11.54	9.33	10.55	9.6				11.71	8.45	7.92	10.84	10.31	12.75			10.27	11.55	10.91	10.06	9.24	8.87	8.51	10.24	15.34
10.85	11.2	11.54	9.33	10.6	9.61				11.72	8.45	7.92	10.89	10.34	12.92			10.49	11.55	10.91	10.06	9.41	8.87	8.51	10.24	15.34
10.86	11.2	11.56	9.33	10.64	9.61				11.72	8.46	7.92	10.89	10.34	13.02			10.5	11.55	10.91	10.06	9.41	8.87	8.51	10.24	15.34
10.87	11.2	11.57	9.45	10.64	9.64				11.72	8.68	7.92	10.89	10.34	13.03			10.5	11.55	10.91	10.43	9.41	8.87	8.51	10.24	15.34
10.97	11.44	11.68	9.45	10.64	9.79				11.72	8.68	8.07	11.08	10.35	13.03			10.51	11.55	10.91	10.48	9.54	9.01	8.51	10.24	15.34
10.98	11.59	11.78	9.45	10.64	9.9				11.73	8.68	8.15	11.09	10.35	13.03			10.69	11.55	10.91	10.48	9.65	9.01	8.51	10.33	15.47
11.28	11.59	11.85	9.45	10.64	9.91				11.77	8.69	8.26	11.09	10.35	13.51			10.8	11.81	10.98	10.48	9.69	9.01	8.51	10.33	15.47
11.29	11.59	12.01	9.45	10.64	9.92				12.21	8.69	8.33	11.09	10.4	13.59			10.8	11.81	10.99	10.48	9.69	9.01	8.85	10.33	15.47
11.29	11.79	12.12	9.85	10.64	9.92				12.21	8.7	8.34	11.25	10.63	13.59			10.8	11.99	10.99	10.48	9.69	9.01	8.85	10.39	15.47
11.29	11.83	12.13	9.85	10.64	9.92				12.21	8.7	8.36	11.26	10.76	13.59			10.8	11.99	11.05	10.48	9.69	9.32	9.56	10.39	15.55
11.31	12.16	12.26	9.85	10.64	9.92				12.39	8.71	8.36	11.4	10.76	13.59			11.05	12.21	11.26	10.48	9.69	9.32	9.58	10.39	15.56
11.31	12.16	12.4	9.85	10.64	10.04				12.53	8.82	8.37	11.47	10.76	13.65			11.17	12.26	11.28	10.57	9.69	9.32	9.58	10.59	15.56
11.55	12.18	12.43	11.18	10.93	10.38				12.53	8.83	8.37	11.49	10.77	13.65			11.17	12.26	11.28	10.57	9.69	9.38	9.58	10.59	15.56
11.59	12.34	12.43	11.32	11.1	10.39				12.54	8.88	8.45	11.49	10.77	13.66			11.17	12.3	11.38	10.69	10.21	9.53	9.58	10.59	15.56
11.59	12.52	12.44	11.32	11.12	10.63				12.82	9.2	8.45	11.49	10.77	13.66			11.17	12.3	11.53	10.69	10.21	9.57	9.58	10.59	15.94
11.59	12.52	12.44	11.32	11.13	10.72				12.82	9.2	8.45	11.56	10.77	13.66			11.27	12.3	11.53	10.69	10.21	9.57	9.58	10.59	15.94
11.59	12.53	12.5	11.32	11.13	10.72				12.82	9.2	8.66	11.56	10.96	13.8			11.28	12.3	11.53	10.69	10.21	9.82	9.58	10.63	15.94
11.59	12.56	12.6	11.32	11.16	10.74				12.82	9.21	8.67	11.56	11.08	14.03			11.28	12.35	11.53	10.7	10.21	9.84	9.58	10.96	15.96
11.6	12.56	12.66	11.32	11.18	10.75				12.83	9.22	8.68	11.67	11.08	14.14			11.28	12.52	11.56	10.71	10.21	10.1	9.58	10.96	15.96
11.6	12.56	12.72	11.32	11.35	10.76				13.14	9.45	8.83	11.68	11.08	14.19			11.28	12.52	11.57	10.72	10.21	10.1	9.58	10.96	15.96
11.64	12.56	12.72	11.32	11.35	10.76				13.18	9.56	8.95	11.68	11.08	14.25			11.28	12.52	11.57	10.72	10.21	10.14	9.58	10.96	15.96
11.72	12.56	12.72	11.32	11.35	10.76				13.18	9.57	8.96	11.68	11.08	14.25			11.48	12.52	11.64	10.72	10.21	10.14	9.58	10.96	16.17
11.72	12.56	12.72	11.32	11.35	10.85				13.18	9.57	8.96	11.69	11.09	14.25			11.48	12.52	11.64	10.89	10.21	10.14	9.58	10.96	16.17
11.73	12.56	12.78	11.32	11.35	10.86				13.28	9.58	8.96	11.81	11.09	14.25			11.48	12.52	11.73	10.98	10.21	10.14	9.58	10.96	16.17
11.84	12.56	12.87	11.32	11.35	10.89				13.28	9.73	8.96	11.81	11.36	14.3			11.48	12.55	11.8	10.98	10.21	10.14	9.58	10.96	16.23
11.97	12.56	12.89	12.21	11.35	10.89				13.29	9.73	8.96	11.82	11.37	14.59			11.57	12.55	11.84	10.98	10.21	10.14	9.58	10.96	16.24
11.97	12.56	12.89	12.21	11.35	10.93				13.29	9.73	8.96	11.95	11.38	14.66			11.57	12.55	11.93	10.98	10.21	10.14	9.58	10.96	16.24

0	0	0	0	0	0	0	0	0	0	0	0	0	0	0	0	0	0	0	0	0	0.58	0.77	0.48	0.68	0
0.05	0.12	0.17	0	0	0	0.02	0	0.23	0	0.11	0.04	0.13	0.02	0.02	0.07	0.27	0	0.12	0.07	0.17	0.59	0.77	0.48	0.68	0.34
0.08	0.13	0.2	0	0.01	0	0.17	0	0.39	0	0.11	0.04	0.31	0.02	0.22	0.13	0.27	0	0.13	0.16	0.35	0.74	0.77	0.49	0.86	0.34
0.13	0.31	0.29	0	0.03	0.29	0.38	0	0.39	0	0.18	0.07	0.39	0.02	0.25	0.13	0.27	0	0.13	0.18	0.35	0.89	0.77	0.62	0.86	0.34
0.2	0.31	0.32	0	0.03	0.29	0.4	0	0.39	0	0.34	0.07	0.41	0.02	0.25	0.15	0.39	0	0.31	0.27	0.36	0.98	0.77	0.62	1.07	0.34
0.34	0.5	0.32	0	0.03	0.29	0.46	0	0.39	0	0.34	0.24	0.51	0.19	0.25	0.28	0.39	0	0.31	0.28	0.36	0.98	0.77	0.79	1.07	0.34
0.35	0.61	0.32	0	0.17	0.48	0.48	0.07	0.45	0	0.35	0.24	0.51	0.2	0.49	0.28	0.39	0	0.32	0.28	0.52	0.99	0.77	0.8	1.07	0.51
0.35	0.61	0.42	0	0.18	0.48	0.48	0.07	0.53	0	0.52	0.24	0.58	0.2	0.49	0.3	0.57	0	0.36	0.34	0.56	1.19	1.05	0.81	1.07	0.51
0.44	0.61	0.5	0	0.2	0.49	0.5	0.07	0.54	0	0.52	0.24	0.66	0.23	0.69	0.37	0.57	0	0.37	0.36	0.57	1.19	1.05	0.93	1.07	0.6
0.44	0.61	0.53	0.12	0.21	0.49	0.52	0.07	0.54	0	0.52	0.28	0.66	0.24	0.69	0.44	0.58	0	0.37	0.37	0.57	1.19	1.25	0.94	1.11	0.6
0.44	0.61	0.62	0.12	0.21	0.5	0.53	0.07	0.61	0	0.53	0.35	0.67	0.24	0.69	0.44	0.58	0	0.37	0.65	0.78	1.26	1.25	0.94	1.19	0.61
0.45	0.61	0.62	0.12	0.21	0.57	0.53	0.07	0.69	0.65	0.54	0.39	0.74	0.34	0.69	0.44	0.58	0.58	0.46	0.65	0.85	1.26	1.25	0.98	1.2	0.72
0.54	0.62	0.64	0.12	0.21	0.58	0.61	0.07	0.7	0.76	0.82	0.39	0.74	0.34	0.77	0.54	0.58	0.66	0.46	0.65	0.87	1.26	1.25	0.99	1.2	0.72
0.54	0.62	0.64	0.12	0.21	0.61	0.64	0.07	0.7	0.78	0.82	0.43	0.78	0.42	0.79	0.92	0.71	0.66	0.47	0.65	0.87	1.26	1.25	1.05	1.2	0.78
0.55	0.7	0.75	0.12	0.3	0.78	0.65	0.19	0.7	0.91	0.82	0.47	0.82	0.47	0.79	0.92	0.71	0.7	0.47	0.65	0.87	1.27	1.25	1.06	1.42	0.79
0.65	0.73	0.79	0.12	0.43	0.79	0.67	0.48	0.72	1.01	0.83	0.49	0.87	0.62	0.79	0.92	0.73	0.83	0.53	0.98	0.87	1.32	1.25	1.21	1.42	0.79
0.66	0.87	0.85	0.12	0.53	1.1	0.71	0.48	0.81	1.02	0.9	0.49	0.87	0.63	0.81	0.92	0.74	0.83	0.53	0.98	0.89	1.32	1.25	1.22	1.42	0.79
0.67	0.87	0.87	0.12	0.59	1.19	0.73	0.56	0.96	1.06	0.9	0.55	0.92	0.63	0.81	0.92	0.85	0.83	0.53	0.98	1.02	1.32	1.25	1.22	1.44	0.97
0.78	0.87	1.05	0.12	0.59	1.29	0.73	0.56	0.96	1.11	1.05	0.58	1	0.89	0.93	0.92	0.85	0.93	0.53	0.98	1.05	1.47	1.25	1.38	1.55	0.98
0.84	1	1.05	0.12	0.59	1.31	0.8	0.56	0.96	1.2	1.1	0.68	1.05	0.89	0.94	0.92	0.97	0.98	0.67	1.13	1.11	1.47	1.25	1.39	1.55	1.16
0.84	1	1.12	0.46	0.63	1.36	1.06	0.56	0.96	1.2	1.1	0.81	1.09	0.9	0.99	1.11	1.13	0.98	0.67	1.25	1.11	1.6	1.35	1.4	1.66	1.16
0.94	1.17	1.21	0.79	0.76	1.53	1.06	0.91	1.1	1.2	1.19	0.83	1.21	0.9	0.99	1.17	1.18	0.98	0.67	1.26	1.21	1.62	1.35	1.49	1.66	1.16
1.08	1.23	1.31	0.79	0.87	1.53	1.11	0.91	1.1	1.28	1.29	0.84	1.25	0.94	1	1.17	1.26	1.05	0.68	1.27	1.36	1.62	1.35	1.5	1.69	1.22
1.09	1.25	1.55	0.79	0.94	1.58	1.35	0.91	1.1	1.28	1.29	0.9	1.28	1	1.23	1.17	1.26	1.06	0.94	1.27	1.36	1.79	1.49	1.57	1.7	1.22
1.19	1.25	1.59	0.79	0.94	1.69	1.35	0.91	1.16	1.28	1.29	0.96	1.36	1.12	1.38	1.17	1.34	1.15	0.94	1.49	1.44	1.79	1.49	1.58	1.94	1.23
1.29	1.25	1.62	0.79	1.06	1.82	1.52	0.91	1.22	1.42	1.29	1.35	1.36	1.13	1.38	1.63	1.35	1.28	0.94	1.57	1.54	1.79	1.51	1.8	2.03	1.23
1.3	1.25	1.72	0.93	1.09	1.95	1.61	1.07	1.22	1.48	1.49	1.35	1.36	1.34	1.38	1.63	1.36	1.43	0.94	1.57	1.54	1.79	1.59	1.8	2.09	1.23
1.31	1.33	1.87	0.93	1.19	2.01	1.61	1.07	1.79	1.52	1.49	1.35	1.48	1.35	1.48	1.63	1.46	1.53	0.94	1.57	1.82	1.87	2.05	1.89	2.09	1.23
1.46	1.55	1.97	0.93	1.32	2.05	1.75	1.07	1.79	1.63	1.64	1.35	1.59	1.35	1.66	1.63	1.55	1.54	1.1	1.57	1.84	1.92	2.05	1.9	2.09	1.23
1.49	1.8	2.1	0.93	1.32	2.13	1.79	1.07	1.79	1.63	1.65	1.39	1.59	1.35	1.66	1.63	1.68	1.85	1.26	1.61	1.85	2.05	2.05	2.03	2.25	1.23
1.49	1.84	2.16	1.68	1.37	2.15	1.79	1.26	1.79	1.82	1.66	1.46	1.59	1.36	1.67	1.63	1.69	1.85	1.26	1.62	2	2.06	2.12	2.06	2.39	1.51
1.49	1.9	2.27	1.68	1.56	2.16	1.79	1.68	1.79	1.91	1.7	1.62	1.77	1.4	2.05	1.63	1.74	1.86	1.42	1.79	2.11	2.06	2.12	2.06	2.39	1.52
1.56	1.9	2.34	1.68	1.57	2.21	2.07	1.68	2.24	1.91	1.97	1.93	1.77	1.49	2.07	1.63	1.83	1.89	1.65	2.02	2.11	2.22	2.12	2.14	2.48	1.52
1.68	1.93	2.39	1.68	1.57	2.26	2.1	1.68	2.24	2	1.97	1.93	1.77	1.49	2.07	1.66	1.83	1.89	1.65	2.02	2.11	2.29	2.12	2.15	2.69	1.52
1.75	1.93	2.43	2.09	1.57	2.31	2.11	1.68	2.24	2.06	1.98	1.93	1.86	1.5	2.08	1.66	1.92	1.89	1.65	2.02	2.17	2.29	2.12	2.15	2.73	1.52
1.75	2.06	2.52	2.09	1.57	2.31	2.11	1.68	2.43	2.08	2.06	1.93	1.88	1.59	2.37	1.66	2	1.89	1.66	2.35	2.21	2.38	2.23	2.26	2.85	1.52
1.96	2.06	2.64	2.09	1.65	2.32	2.11	1.68	2.43	2.08	2.16	1.93	1.96	1.59	2.39	1.66	2.01	1.89	1.71	2.47	2.42	2.53	2.23	2.26	2.98	1.52
1.96	2.33	2.64	2.09	1.66	2.32	2.11	1.68	2.43	2.2	2.17	1.99	2	1.73	2.47	1.68	2.02	2.12	1.83	2.5	2.45	2.63	2.23	2.27	3.01	1.67
1.99	2.34	2.68	2.09	1.87	2.41	2.11	1.68	2.43	2.2	2.17	2.09	2.01	1.74	2.48	1.77	2.26	2.21	1.83	2.56	2.45	2.63	2.35	2.37	#VALUE!	1.67
1.99	2.34	2.68	2.09	1.88	2.47	2.13	1.68	2.43	2.25	2.18	2.45	2.15	1.75	2.63	1.77	2.32	2.21	1.88	2.58	2.45	2.63	2.38	2.38	#VALUE!	1.67
2.17	2.63	2.93	2.09	1.91	2.51	2.15	1.68	2.43	2.31	2.26	2.57	2.22	1.97	2.64	1.97	2.33	2.39	1.88	2.6	2.49	2.69	2.47	2.38	#VALUE!	1.67
2.17	2.64	2.93	2.23	2.04	2.53	2.26	1.68	2.86	2.31	2.34	2.57	2.28	1.98	2.68	2.11	2.45	2.47	1.88	2.73	2.54	2.69	2.54	2.5	#VALUE!	1.85
2.23	2.85	2.99	2.23	2.04	2.68	2.26	1.88	2.86	2.32	2.38	2.57	2.33	1.98	2.89	2.11	2.45	2.5	2.05	2.77	2.6	2.69	2.54	2.5	#VALUE!	1.85
2.3	2.88	3.04	2.23	2.04	2.69	2.27	2.37	2.86	2.32	2.38	2.57	2.49	2.12	2.96	2.11	2.46	2.5	2.18	2.87	2.61	2.95	2.7	2.51	#VALUE!	1.85
2.31	2.88	3.14	2.23	2.19	2.7	2.38	2.37	2.86	2.55	2.39	2.57	2.49	2.25	2.96	2.11	2.46	2.5	2.18	2.89	2.61	2.98	2.72	2.61	#VALUE!	1.85
2.31	2.92	3.14	2.23	2.34	2.96	2.47	2.37	2.86	2.64	2.39	2.63	2.52	2.29	2.96	2.33	2.46	2.73	2.18	2.9	2.61	2.99	2.87	2.74	#VALUE!	1.85
2.38	2.92	3.14	2.23	2.34	3.18	2.47	2.37	2.86	2.69	2.5	2.64	2.52	2.29	2.96	2.33	2.52	2.73	2.25	2.9	2.66	2.99	2.87	2.74	#VALUE!	1.86
2.38	2.92	3.31	2.23	2.39	3.18	2.47	2.37	2.86	2.7	2.66	2.64	2.57	2.38	2.96	2.33	2.53	2.73	2.31	3.03	2.77	2.99	2.87	2.75	#VALUE!	1.86
2.39	3.11	3.31	2.23	2.47	3.2	2.57	2.37	2.86	2.71	2.67	2.74	2.58	2.39	2.96	2.33	2.53	2.73	2.32	3.04	2.77	3.13	2.87	2.75	#VALUE!	1.86
2.51	3.11	3.41	2.23	2.47	3.23	2.58	2.37	2.86	2.71	2.76	3.12	2.7	2.39	2.96	2.33	2.54	2.73	2.32	3.04	2.78	3.13	2.87	2.75	#VALUE!	1.86
2.51	3.12	3.42	2.23	2.51	3.31	2.59	2.73	2.86	2.89	2.77	3.12	2.7	2.39	2.97	2.33	2.54	2.83	2.37	3.07	2.92	3.2	2.87	3	#VALUE!	1.86
2.58	3.22	3.49	2.23	2.53	3.33	2.6	2.73	2.86	2.91	2.78	3.12	2.81	2.47	2.97	2.33	2.55	2.86	2.37	3.08	2.92	3.2	2.87	3	#VALUE!	1.86
2.58	3.22	3.49	2.23	2.53	3.34	2.6	2.73	2.86	2.91	2.88	3.12	2.81	2.47	2.98	2.33	2.56	2.86	2.5	3.08	2.99	3.25	2.98	3	#VALUE!	1.86
2.58	3.24	3.49	2.23	2.63	3.34	2.74	2.73	2.86	2.92	2.88	3.12	2.81	2.47	3.22	2.43	2.56	2.86	2.53	3.3	2.99	3.33	3.05	3	#VALUE!	1.86
2.59	3.24	3.61	2.23	2.64	3.34	2.74	2.73	2.86	3.04	2.89	3.12	2.88	2.47	3.32	2.43	2.63	2.86	2.53	3.31	3.03	3.33	3.19	3	#VALUE!	1.9
2.59	3.25	3.71	2.23	2.64	3.36	2.74	2.73	2.86	3.04	2.89	3.12	2.88	2.54	3.32	2.43	2.67	2.86	2.53	3.31	3.1	3.33	3.54	3	#VALUE!	2.08
2.68	3.25	3.76	2.23	2.64	3.42	2.8	2.73	2.86	3.04	3.01	3.12	2.89	2.55	3.32	2.43	2.68	2.86	2.64	3.31	3.23	3.41	3.54	3	#VALUE!	2.08
2.68	3.25	3.76	2.23	2.64	3.42	2.8	2.73	2.86	3.04	3.01	3.34	2.89	2.69	3.32	2.63	2.68	2.86	2.65	3.31	3.24	3.41	3.54	3	#VALUE!	2.08
2.68	3.25	3.76	2.23	2.64	3.43	2.8	2.73	2.86	3.04	3.02	3.34	2.89	2.7	3.44	2.63	2.69	2.86	2.65	3.31	3.28	3.41	3.54	3	#VALUE!	2.08
2.78	3.35	3.76	2.23	2.76	3.46	2.8	2.73	2.86	3.05	3.14	3.34	2.97	2.7	3.44	2.63	2.8	2.86	2.66	3.32	3.29	3.41	3.54	3	#VALUE!	2.08
2.82	3.35	3.83	2.23	2.83	3.48	2.8	2.73	2.86	3.05	3.14	3.34	2.97	2.73	3.45	2.63	2.81	2.86	2.66	3.32	3.41	3.42	3.54	3.02	#VALUE!	2.08
2.82	3.37	3.83	2.23	2.85	3.55	2.81	2.73	2.86	3.05	3.15	3.34	2.97	2.89	3.45	2.63	2.81	2.86	2.66	3.33	3.41	3.5	3.54	3.02	#VALUE!	2.08
2.82	3.37	3.87	2.23	2.85	3.58	2.81	2.73	2.9	3.05	3.15	3.34	3.02	2.89	3.45	2.63	2.94	2.86	2.73	3.34	3.41	3.5	3.54	3.02	#VALUE!	2.08
2.82	3.37	3.96	2.23	2.86	3.58	2.81	2.73	2.9	3.19	3.21	3.34	3.02	2.9	3.74	2.63	2.95	2.86	2.73	3.34	3.56	3.5	3.54	3.02	#VALUE!	2.08
2.82	3.37	3.96	2.23	2.87	3.66	3	2.73	2.9	3.31	3.21	3.34	3.02	2.9	3.88	2.73	2.95	2.86	2.73	3.34	3.56	3.5	3.54	3.02	#VALUE!	2.09
2.85	3.37	3.97	2.23	2.89	3.66	3	2.73	2.9	3.31	3.22	3.34	3.05	2.94	3.89	2.76	2.96	2.86	2.74	3.34	3.56	3.5	3.54	3.02	#VALUE!	2.09
2.86	3.37	3.97	2.23	2.9	3.66	3.01	2.73	2.99	3.39	3.23	3.44	3.05	2.94	3.89	2.78	3.04	2.86	2.81	3.48	3.62	3.5	3.54	3.02	#VALUE!	2.09
2.89	3.39	3.97	2.23	2.9	3.76	3.06	2.73	2.99	3.39	3.23	3.5	3.17	2.94	3.89	2.94	3.04	2.86	2.82	3.48	3.75	3.69	3.54	3.04	#VALUE!	2.09
2.89	3.47	4.06	2.23	3.01	3.78	3.06	2.73	2.99	3.39	3.23	3.51	3.17	3.02	4.04	2.94	3.1	2.86	2.82	3.48	3.75	3.69	3.54	3.18	#VALUE!	2.09
2.93	3.47	4.07	2.23	3.2	3.88	3.06	2.73	3.01	3.39	3.24	3.81	3.25	3.14	4.04	2.94	3.11	2.86	2.93	3.48	3.75	3.69	3.54	3.18	#VALUE!	2.09
2.94	3.54	4.07	2.23	3.2	3.99	3.08	2.73	3.01	3.4	3.32	3.85	3.25	3.14	4.04	2.94	3.11	2.93	2.98	3.48	3.81	3.7	3.54	3.18	#VALUE!	2.09
2.94	3.54	4.12	2.23	3.24	3.99	3.23	2.73	3.12	3.42	3.39	3.85	3.25	3.29	4.05	2.94	3.32	2.93	2.98	3.7	3.82	3.74	3.54	3.18	#VALUE!	2.2
3.07	3.54	4.2	2.23	3.38	4.06	3.24	2.73	3.12	3.6	3.41	3.85	3.25	3.29	4.05	2.94	3.33	2.94	3.08	3.7	3.82	3.75	3.54	3.29	4.13	2.21
3.07	3.55	4.21	2.23	3.44	4.06	3.25	3.06	3.14	3.6	3.42	3.85	3.48	3.3	4.05	2.94	3.42	2.94	3.11	3.71	3.83	3.75	3.54	3.29	4.15	2.21
3.08	3.56	4.28	2.23	3.44	4.06	3.43	3.06	3.14	3.67	3.42	3.85	3.51	3.3	4.22	3.02	3.43	2.99	3.18	3.78	3.89	3.83	3.54	3.29	4.15	2.21
3.08	3.56	4.28	2.23	3.45	4.23	3.44	3.06	3.26	3.67	3.47	3.85	3.52	3.3	4.33	3.1	3.51	3	3.18	3.8	3.99	3.88	3.54	3.4	4.19	2.21

3.19	3.56	4.33	2.23	3.45	4.25	3.44	3.06	3.44	3.67	3.48	3.85	3.52	3.3	4.34	3.25	3.52	3	3.19	3.81	4	3.88	3.54	3.61	4.2	2.21
3.23	3.62	4.33	2.8	3.45	4.27	3.44	3.06	3.44	3.67	3.48	3.86	3.66	3.36	4.34	3.25	3.57	3	3.19	3.95	4	3.88	3.58	3.62	4.2	2.73
3.24	3.64	4.41	2.8	3.45	4.33	3.58	3.06	3.44	3.83	3.48	3.9	3.7	3.52	4.52	3.25	3.7	3.2	3.19	3.95	4.01	3.88	3.67	3.63	4.28	2.73
3.36	3.75	4.53	2.8	3.55	4.45	3.69	3.06	3.44	3.91	3.49	3.9	3.73	3.6	4.6	3.25	3.76	3.28	3.28	4.1	4.07	3.91	3.86	3.63	4.36	2.8
3.41	3.75	4.53	2.84	3.75	4.51	3.78	3.06	3.44	3.93	3.49	3.9	3.85	3.67	4.6	3.25	3.82	3.37	3.29	4.29	4.09	4.01	3.97	3.76	4.41	3.09
3.46	3.8	4.59	2.84	3.75	4.61	3.79	3.14	3.45	4.05	3.54	3.9	3.88	3.68	4.77	3.4	3.82	3.48	3.34	4.31	4.14	4.01	3.97	3.76	4.41	3.09
3.5	3.8	4.73	2.84	3.8	4.71	3.79	3.18	3.64	4.09	3.55	3.9	3.99	3.68	4.95	3.4	3.95	3.48	3.34	4.32	4.14	4.07	4.17	3.76	4.44	3.35
3.57	3.9	4.84	3.06	3.94	4.71	3.94	3.18	3.66	4.28	3.6	3.9	4.03	3.68	4.95	3.4	4.02	3.65	3.35	4.32	4.32	4.07	4.17	3.83	4.46	3.35
3.65	4.01	4.84	3.78	3.99	4.71	4.08	3.18	3.83	4.28	3.67	3.91	4.03	3.69	5.17	3.4	4.02	3.65	3.38	4.41	4.48	4.14	4.38	4.02	4.61	3.36
3.66	4.22	4.94	3.78	4.08	4.74	4.08	3.26	3.87	4.36	3.81	4.01	4.03	3.92	5.28	3.4	4.21	3.76	3.45	4.51	4.48	4.15	4.61	4.09	4.68	3.46
3.66	4.25	5.02	3.78	4.15	4.83	4.09	3.26	3.91	4.36	3.81	4.16	4.1	3.93	5.28	3.4	4.25	3.84	3.54	4.67	4.49	4.15	4.68	4.09	4.77	3.62
3.71	4.42	5.05	3.78	4.21	4.85	4.09	3.3	3.91	4.55	3.82	4.16	4.16	3.93	5.28	3.4	4.35	3.86	3.56	4.68	4.49	4.23	4.68	4.18	4.82	3.62
3.85	4.52	5.09	3.78	4.23	4.99	4.16	3.31	4.06	4.62	3.98	4.21	4.18	4.04	5.28	3.59	4.41	3.86	3.56	4.82	4.64	4.24	4.68	4.2	5.01	3.62
3.85	4.53	5.14	3.78	4.33	5.1	4.24	3.64	4.3	4.7	3.98	4.35	4.36	4.08	5.45	3.7	4.53	3.9	3.73	4.98	4.65	4.25	4.69	4.2	5.12	3.8
3.89	4.53	5.17	3.78	4.41	5.1	4.28	3.82	4.3	4.82	4.06	4.4	4.36	4.2	5.5	3.7	4.71	3.9	3.74	4.98	4.65	4.3	4.69	4.2	5.2	3.82
4.02	4.55	5.3	3.78	4.44	5.11	4.42	3.82	4.3	4.82	4.15	4.46	4.41	4.29	5.69	3.7	4.77	4.1	3.74	4.98	4.82	4.45	4.7	4.27	5.22	4.01
4.02	4.71	5.45	3.78	4.46	5.11	4.42	3.82	4.3	4.84	4.15	4.61	4.41	4.34	5.69	3.7	4.83	4.1	3.78	4.98	4.94	4.67	4.73	4.28	5.27	4.01
4.06	4.71	5.45	3.78	4.65	5.18	4.42	4.02	4.34	5.03	4.21	4.64	4.5	4.34	5.75	3.7	4.83	4.21	3.94	5.02	5.06	4.68	4.73	4.38	5.27	4.01
4.18	4.75	5.48	3.78	4.66	5.32	4.46	4.02	4.34	5.09	4.33	4.68	4.53	4.34	5.78	3.7	4.87	4.31	4	5.05	5.06	4.79	5.59	4.54	5.27	4.01
4.25	4.79	5.53	3.78	4.89	5.36	4.46	4.02	4.34	5.11	4.4	4.84	4.54	4.34	5.84	3.85	4.94	4.4	4.14	5.16	5.06	4.8	5.7	4.62	5.28	4.01
4.33	4.99	5.53	3.78	4.9	5.38	4.46	4.02	4.34	5.23	4.41	4.86	4.6	4.35	5.84	3.85	5.07	4.4	4.14	5.19	5.21	4.83	5.77	4.63	5.61	4.02
4.33	5.09	5.63	3.78	4.98	5.45	4.69	4.02	4.67	5.36	4.42	4.86	4.64	4.69	5.84	3.85	5.23	4.41	4.29	5.26	5.21	4.84	5.77	4.64	5.61	4.02
4.33	5.09	5.68	3.78	4.98	5.52	4.7	4.02	4.89	5.37	4.49	4.89	4.64	4.7	5.91	4.01	5.27	4.42	4.39	5.3	5.28	5.05	5.77	4.85	5.71	4.02
4.44	5.09	5.68	3.78	5.11	5.59	4.84	4.21	5.26	5.48	4.61	5.08	4.67	4.83	5.91	4.27	5.36	4.42	4.39	5.31	5.52	5.05	5.77	4.85	5.71	4.19
4.44	5.09	5.68	4.62	5.15	5.64	4.84	4.21	5.26	5.58	4.67	5.08	4.67	4.84	6.05	4.27	5.36	4.68	4.48	5.31	5.56	5.08	5.77	4.85	6.27	4.19
4.44	5.09	5.69	4.62	5.35	5.64	4.84	4.34	5.26	5.58	4.67	5.08	4.67	4.91	6.46	4.27	5.37	4.68	4.48	5.46	5.56	5.09	5.77	4.87	6.27	4.36
4.54	5.32	6	4.62	5.39	5.71	4.91	4.38	5.26	5.64	4.67	5.2	4.78	4.91	6.6	4.28	5.58	4.68	4.49	5.66	5.63	5.09	5.94	4.87	6.27	4.36
4.54	5.54	6.14	4.62	5.42	5.73	5.11	4.42	5.26	5.74	4.67	5.2	4.78	4.91	6.6	4.28	5.58	4.83	4.62	5.71	5.63	5.31	6.12	4.87	6.27	4.36
4.55	5.54	6.14	4.62	5.43	5.73	5.18	4.42	5.26	5.85	4.82	5.22	4.83	5.12	6.6	4.28	5.58	4.93	4.62	5.77	5.98	5.36	6.26	4.99	6.27	4.36
4.75	5.59	6.23	4.62	5.46	5.73	5.18	4.42	5.26	6.09	4.82	5.51	4.97	5.23	6.6	4.28	5.7	4.93	4.63	5.77	5.98	5.39	6.26	5.1	6.27	4.36
4.75	5.67	6.34	4.62	5.46	5.81	5.19	4.42	5.26	6.15	4.83	5.64	4.97	5.24	6.6	4.28	5.75	4.98	4.82	5.84	5.98	5.6	6.26	5.1	6.27	4.37
4.76	5.77	6.42	4.62	5.47	5.83	5.19	4.42	5.26	6.29	4.96	5.64	5	5.24	6.77	4.35	5.76	5.01	4.95	5.87	5.98	5.67	6.26	5.11	6.33	4.37
4.93	5.83	6.42	4.62	5.47	6.01	5.2	4.42	5.26	6.29	4.97	5.82	5.17	5.24	6.85	4.35	5.77	5.01	4.95	5.89	6.01	5.67	6.29	5.11	6.33	4.37
5.02	5.83	6.42	5.11	5.81	6.31	5.38	4.62	5.65	6.33	5.21	5.82	5.22	5.48	6.86	4.35	5.77	5.23	5.05	6.26	6.01	5.67	6.29	5.22	6.36	4.37
5.02	5.83	6.54	5.22	5.88	6.45	5.38	4.62	5.65	6.34	5.21	5.82	5.26	5.55	6.86	4.47	5.81	5.33	5.09	6.26	6.16	5.67	6.29	5.24	6.36	4.37
5.02	5.95	6.58	5.22	5.93	6.47	5.38	4.62	5.78	6.35	5.29	5.82	5.27	5.55	6.86	4.47	5.81	5.33	5.31	6.26	6.17	5.67	7.1	5.42	6.36	4.37
5.03	5.96	6.58	5.22	6.14	6.54	5.38	4.62	5.78	6.56	5.51	5.82	5.35	5.55	6.86	4.47	5.82	5.33	5.31	6.26	6.65	5.68	7.1	5.43	6.5	4.37
5.09	5.99	6.58	5.22	6.14	6.65	5.38	4.62	5.78	6.62	5.51	5.92	5.53	5.55	6.87	4.48	5.89	5.33	5.31	6.26	6.71	5.68	7.1	5.43	6.55	4.37
5.09	6.03	6.7	5.22	6.14	6.67	5.39	4.62	5.78	6.62	5.52	5.92	5.54	5.55	6.87	4.51	5.89	5.34	5.31	6.29	6.85	5.86	7.1	5.55	6.55	4.37
5.1	6.03	6.72	5.22	6.14	6.67	5.39	4.62	5.78	6.76	5.52	5.92	5.54	5.56	6.87	4.54	5.89	5.34	5.31	6.43	6.85	5.98	7.1	5.55	6.55	4.37
5.11	6.04	6.72	5.22	6.14	6.67	5.39	5.09	5.78	6.76	5.58	5.92	5.54	5.56	6.87	4.57	5.9	5.34	5.39	6.59	6.85	5.99	7.1	5.55	6.56	4.37
5.12	6.04	6.72	5.22	6.22	6.79	5.4	5.09	5.78	6.76	5.58	5.92	5.54	5.56	6.87	4.59	5.9	5.34	5.39	6.64	6.85	5.99	7.1	5.58	6.56	4.47
5.51	6.14	6.84	5.97	6.22	6.81	5.4	5.09	5.78	6.76	5.59	5.95	5.62	5.57	6.88	4.64	6.08	5.35	5.4	6.64	6.88	5.99	7.1	5.58	6.56	4.55
5.51	6.14	6.89	5.97	6.22	6.87	5.4	5.09	5.78	6.76	5.59	5.95	5.62	5.58	6.94	4.68	6.15	5.35	5.4	6.65	6.88	6.07	7.1	5.58	6.56	4.58
5.57	6.15	6.89	5.97	6.24	6.87	5.46	5.09	5.78	6.87	5.6	6.01	5.62	5.58	6.95	4.95	6.15	5.35	5.4	6.66	6.88	6.07	7.1	5.58	6.98	4.58
5.58	6.16	6.9	5.97	6.24	6.96	5.46	5.09	5.78	6.87	5.6	6.01	5.62	5.58	6.95	4.95	6.15	5.35	5.4	6.66	6.88	6.14	7.1	5.58	6.98	4.58
5.58	6.16	7.02	5.97	6.24	7.02	5.46	5.09	5.78	6.87	5.61	6.08	5.73	5.59	6.95	4.95	6.16	5.35	5.4	6.66	6.88	6.14	7.1	5.58	6.98	4.58
5.58	6.16	7.03	7.77	6.24	7.03	5.46	5.09	5.78	7.14	5.61	6.08	5.74	5.59	6.96	4.95	6.16	5.36	5.41	6.66	6.88	6.14	7.1	5.67	6.98	4.58
5.58	6.25	7.03	7.77	6.24	7.06	5.47	5.15	5.96	7.14	5.61	6.08	5.81	5.59	6.96	4.95	6.2	5.36	5.56	6.66	6.88	6.24	7.1	5.67	6.98	4.58
5.62	6.38	7.11	7.77	6.24	7.06	5.47			7.14	5.62	6.08	5.81	5.59	6.97			5.45	5.56	6.66	6.88	6.24	7.1	5.68	6.98	4.58
5.63	6.38	7.11	7.77	6.27	7.06	5.48			7.15	5.62	6.29	5.82	5.6	6.97			5.45	5.57	6.66	6.88	6.24	7.1	5.68	6.98	4.58
5.69	6.39	7.12	7.77	6.27	7.13	5.48			7.15	5.73	6.29	5.82	5.6	6.97			5.45	5.57	6.69	6.88	6.24	7.1	5.76	6.98	4.58
5.73	6.39	7.12	7.77	6.3	7.18	5.49			7.15	5.73	6.29	5.82	5.74	6.97			5.45	5.57	6.72	6.88	6.25	7.1	5.77	6.98	4.79
5.73	6.4	7.13	7.77	6.3	7.18	5.5			7.2	5.73	6.3	5.83	5.74	6.97			5.45	5.57	6.72	6.88	6.25	7.1	5.77	7.01	4.81
5.77	6.4	7.13	7.77	6.3	7.19				7.27	5.79	6.3	5.84	5.74	6.97			5.45	5.57	6.73	6.88	6.35	7.1	5.83	7.01	4.81
5.77	6.41	7.14	7.77	6.3	7.19				7.27	5.79	6.31	5.9	5.81	6.98			5.58	5.57	6.73	6.88	6.35	7.1	5.84	7.02	4.82
5.77	6.41	7.27	7.77	6.48	7.19				7.27	5.8	6.35	5.9	5.82	7.28			5.58	5.58	6.73	6.88	6.35	7.1	5.91	7.07	4.84
5.81	6.41	7.28	7.77	6.49	7.21				7.27	5.8	6.35	5.9	5.82	7.47			5.59	5.58	6.9	6.88	6.35	7.1	5.92	7.07	4.84
5.93	6.64	7.28	7.77	6.68	7.46				7.27	5.8	6.35	6.06	5.92	7.48			5.6	5.58	6.93	6.88	6.36	7.1	5.92	7.21	4.84
5.93	6.65	7.29	7.92	6.83	7.53				7.3	5.86	6.35	6.06	6.11	7.7			5.6	5.58	6.95	6.88	6.4	7.1	5.92	7.32	4.84
5.93	6.65	7.35	8.3	6.83	7.53				7.31	5.87	6.35	6.07	6.12	7.7			5.67	5.59	6.98	6.88	6.4	7.1	5.93	7.32	4.84
6.16	6.65	7.4	8.3	6.96	7.6				7.32	5.88	6.35	6.25	6.42	7.71			5.67	5.67	6.98	6.96	6.4	7.1	5.93	7.32	4.95
6.16	6.66	7.46	8.3	6.99	7.74				7.39	5.88	6.35	6.26	6.43	7.95			5.78	5.67	6.98	6.99	6.4	7.1	5.93	7.32	4.95
6.16	6.66	7.46	8.3	7.06	7.8				7.42	5.95	6.51	6.26	6.44	7.99			5.78	5.68	6.98	6.99	6.44	7.1	5.94	7.32	5.22
6.16	6.67	7.47	8.3	7.07	7.8				7.42	6.03	6.51	6.26	6.44	7.99			5.78	5.68	7.03	7.03	6.45	7.1	6.03	7.37	5.22
6.16	6.67	7.5	8.3	7.07	7.9				7.46	6.03	6.51	6.27	6.57	7.99			5.78	5.82	7.03	7.03	6.45	7.1	6.03	7.4	5.26
6.16	6.67	7.61	8.3	7.18	7.96				7.46	6.04	6.51	6.27	6.62	8.05			5.78	5.82	7.03	7.09	6.45	7.1	6.04	7.4	5.26
6.16	6.68	7.67	8.3	7.22	8.04				7.48	6.04	6.54	6.27	6.62	8.05			5.87	5.83	7.04	7.1	6.46	7.1	6.3	7.4	5.46
6.27	6.72	7.75	8.3	7.3	8.08				7.55	6.11	6.54	6.41	6.71	8.05			5.88	5.83	7.04	7.1	6.46	7.1	6.38	7.4	5.46
6.27	6.77	7.96	8.3	7.39	8.17				7.63	6.18	6.67	6.41	6.71	8.31			5.99	5.83	7.04	7.13	6.46	7.1	6.39	7.43	5.85
6.41	6.77	7.98	8.3	7.68	8.32				7.82	6.23	6.73	6.53	6.71	8.32			6.12	5.87	7.06	7.38	6.64	7.1	6.45	7.6	5.94
6.57	6.91	7.98	8.3	7.68	8.34				7.93	6.33	6.73	6.67	6.88	8.32			6.12	5.88	7.21	7.52	6.66	7.1	6.45	7.62	6.12
6.57	7.01	8.05	8.3	7.68	8.4				8	6.38	6.82	6.68	6.93	8.63			6.23	6.02	7.21	7.52	6.72	7.36	6.58	7.64	6.22
6.61	7.06	8.23	8.3	7.72	8.63				8.21	6.38	7.18	6.78	6.94	8.63			6.23	6.02	7.23	7.63	6.83	7.49	6.68	7.66	6.38
6.63	7.06	8.23	8.3	7.73	8.66				8.21	6.39	7.18	6.79	6.94	8.77			6.4	6.23	7.33	7.79	6.84	7.51	6.69	7.7	6.46
6.68	7.07	8.32	8.3	7.84	8.69				8.27	6.5	7.18	6.79	6.94	8.77			6.45	6.24	7.33	7.79	6.84	7.57	6.69	7.73	6.46
6.68	7.19	8.38	8.3	7.9	8.73				8.31	6.56	7.18	6.82	6.99	8.77			6.45	6.24	7.37	7.82	6.84	7.77	6.69	7.87	6.6
6.8	7.19	8.54	8.3	7.93	8.73				8.32	6.56	7.18	6.9	6.99	8.89			6.48	6.25	7.37	7.82	6.85	7.79	6.69	7.92	6.6
6.91	7.19	8.65	9.51	7.95	8.73				8.56	6.58	7.18	6.97	7.13	8.89			6.48	6.25	7.37	7.82	6.92	7.79	6.88	8.02	6.6
6.97	7.32	8.65	9.74	7.95	8.95				8.57	6.75	7.18	6.97	7.13	8.96			6.48	6.34	7.44	7.98	6.92	7.79	6.88	8.36	6.6
7.06	7.32	8.65	10.08	8.09	8.95				8.58	6.82	7.18	7.16	7.14	8.96			6.65	6.62	7.45	8.02	6.93	8.11	7.14	8.38	6.6
7.13	7.32	8.71	10.08	8.22	8.95				8.61	6.98	7.24	7.16	7.14	8.96			6.66	6.64	7.51	8.19	6.93	8.11	7.14	8.43	6.77
7.13	7.48	8.71	10.08	8.27	8.97				8.74	6.99	7.43	7.17	7.14	8.96			6.66	6.64	7.73	8.22	7.08	8.11	7.16	8.43	6.77
7.15	7.55	8.77	10.08	8.31	9.03				8.74	7.06	7.62	7.25	7.16	8.97			7.01	6.64	7.73	8.22	7.09	8.23	7.16	8.43	6.77
7.15	7.57	8.83	10.08	8.34	9.09				8.74	7.14	7.62	7.25	7.3	8.97			7.04	6.67	7.93	8.22	7.25	8.23	7.23	8.48	6.77
7.17	7.57	8.83	10.08	8.39	9.09				8.77	7.15	7.62	7.25	7.3	9.08			7.04	6.67	7.94	8.25	7.34	8.23	7.23	8.48	6.77
7.36	7.58	8.84	10.08	8.47	9.19				8.77	7.15	7.62	7.38	7.34	9.31			7.04	6.76	7.96	8.63	7.34	8.25	7.24	8.48	7.06
7.36	7.88	8.92	10.08	8.68	9.39				8.92	7.21	7.62	7.51	7.35	9.31			7.2	6.84	8.13	8.63	7.34	8.25	7.4	8.48	7.06
7.54	7.88	9.15	10.08	8.75	9.46				8.94	7.37	7.62	7.53	7.44	9.32			7.28	6.85	8.32	8.63	7.34	8.34	7.4	8.71	7.06
7.55	7.89	9.31	10.08	8.86	9.47				8.95	7.46	7.62	7.54	7.44	9.32			7.28	6.85	8.32	8.73	7.46	8.34	7.41	8.8	7.06
7.64	7.99	9.39	10.08	8.87	9.54				8.96	7.51	7.62	7.72	7.52	9.56			7.28	6.85	8.32	8.73	7.58	8.41	7.41	8.82	7.06
7.64	7.99	9.43	10.08	8.88	9.58				8.96	7.58	7.9	7.72	7.61	9.6			7.28	7.08	8.32	8.73	7.59	8.41	7.55	8.82	7.06
7.68	8.16	9.43	10.08	9	9.62				8.96	7.73	7.9	7.72	7.61	9.61			7.42	7.21	8.32	8.73	7.6	8.41	7.55	8.85	7.06
7.68	8.17	9.52	10.08	9	9.62				8.96	7.74	7.9	7.78	7.61	9.61			7.54	7.31	8.32	8.73	7.73	8.41	7.65	8.85	7.06
7.68	8.17	9.54	10.08	9	9.79				8.96	7.82	7.9	7.97	7.61	9.8			7.54	7.37	8.55	8.79	7.73	8.41	7.86	9.02	7.06
7.68	8.3	9.63	10.08	9.09	9.98				9.06	7.82	7.9	8.02	7.61	9.95			7.55	7.37	8.84	8.8	7.73	8.6	7.86	9.2	7.28
7.68	8.32	9.66	10.08	9.09	10.03				9.36	7.83	7.9	8.02	7.62	9.96			7.7	7.38	8.86	8.8	7.73	8.62	7.88	9.22	7.42
7.68	8.5	9.73	10.08	9.11	10.03				9.38	7.91	7.9	8.08	7.77	9.96			7.7	7.4	8.86	9.02	8.02	8.78	7.88	9.23	7.43
7.74	8.51	9.76	10.08	9.36	10.11				9.38	7.99	7.9	8.14	7.77	10.17			7.71	7.42	8.98	9.05	8.03	8.78	7.88	9.29	7.58
8.06	8.51	9.87	10.08	9.55	10.17				9.67	7.99	7.9	8.22	7.78	10.24			7.86	7.42	9	9.2	8.03	8.78	7.99	9.29	7.59
8.06	8.58	9.88	10.08	9.62	10.19				9.67	8.13	7.9	8.3	7.78	10.25			7.86	7.42	9.01	9.24	8.2	9.05	8	9.29	7.59
8.06	8.58	9.88	10.08	9.62	10.2				9.67	8.13	7.9	8.31	7.78	10.25			7.86	7.65	9.02	9.38	8.2	9.05	8	9.3	7.59
8.06	8.71	9.88	10.08	9.62	10.2				9.68	8.13	7.9	8.31	7.78	10.25			7.86	7.66	9.02	9.54	8.2	9.83	8.18	9.3	7.59
8.06	8.72	9.89	10.08	9.62	10.2				9.68	8.24	7.9	8.31	7.78	10.25			7.86	7.66	9.02	9.54	8.2	9.92	8.18	9.77	7.76
8.06	8.72	10.05	10.08	9.68	10.32				9.69	8.25	7.9	8.32	7.79	10.26			7.86	7.66	9.02	9.62	8.2	9.92	8.18	9.77	7.76
8.19	8.72	10.05	10.08	9.69	10.33				9.79	8.25	7.9	8.43	7.79	10.6			7.86	7.66	9.02	9.71	8.2	9.92	8.18	9.86	7.77
8.19	8.72	10.05	10.08	9.74	10.47				9.82	8.26	7.9	8.43	8.06	10.61			7.86	7.66	9.04	9.71	8.27	9.92	8.19	9.86	7.77
8.19	8.73	10.17	10.08	9.87	10.47				9.85	8.26	7.9	8.43	8.07	10.61			7.86	7.67	9.06	9.71	8.35	9.92	8.27	9.86	7.77
8.19	8.73	10.17	10.08	9.91	10.51				9.87	8.27	7.9	8.43	8.07	10.61			7.86	7.67	9.06	9.71	8.35	9.92	8.27	9.89	7.85
8.28	8.74	10.26	10.08	9.91	10.51				9.88	8.39	8.03	8.44	8.07	10.61			7.86	7.74	9.06	9.71	8.35	9.92	8.27	9.92	8.02
8.28	8.74	10.37	10.08	9.91	10.51				9.89	8.4	8.03	8.44	8.08	10.62			7.86	7.75	9.06	9.71	8.36	9.92	8.28	9.93	8.03
8.28	8.74	10.37	10.08	9.91	10.51				9.89	8.4	8.03	8.45	8.08	10.62			8.01	7.75	9.16	9.73	8.36	9.92	8.28	9.93	8.03
8.28	8.75	10.37	10.08	9.91	10.54				9.9	8.57	8.03	8.45	8.08	10.63			8.02	7.75	9.16	9.73	8.36	9.92	8.28	9.93	8.03
8.28	8.75	10.38	10.08	9.91	10.54				9.9	8.58	8.03	8.51	8.09	10.63			8.02	7.75	9.16	9.73	8.36	9.92	8.29	9.93	8.03

0	0	0	0	0	0	0	0	0	0	0	0	0	0	0	0	0	0	0	0	0	0	0	0	0	0
0.02	0.07	0.13	0	0.01	0.01	0	0	0	0.1	0	0.13	0.01	0	0	0	0	0	0.02	0	0.12	0	0	0.02	0	0.01
0.03	0.14	0.17	0	0.03	0.02	0.08	0	0	0.15	0	0.13	0.02	0	0.02	0	0	0	0.03	0	0.12	0.01	0	0.02	0	0.01
0.1	0.16	0.17	0	0.08	0.02	0.09	0	0	0.16	0.08	0.13	0.03	0.07	0.04	0	0.06	0	0.12	0	0.12	0.01	0	0.02	0.01	0.02
0.18	0.18	0.2	0.85	0.1	0.09	0.09	0	0	0.16	0.08	0.13	0.04	0.25	0.04	0	0.06	0.18	0.13	0	0.15	0.07	0.11	0.13	0.03	0.11
0.23	0.38	0.2	1.11	0.1	0.3	0.09	0	0	0.16	0.08	0.13	0.05	0.25	0.05	0	0.07	0.18	0.13	0	0.15	0.16	0.14	0.13	0.03	0.12
0.23	0.38	0.3	1.11	0.1	0.3	0.09	0	0	0.16	0.12	0.13	0.1	0.26	0.05	0	0.11	0.21	0.17	0.1	0.15	0.16	0.14	0.14	0.12	0.12
0.24	0.46	0.42	1.11	0.15	0.37	0.17	0	0	0.19	0.13	0.13	0.1	0.37	0.06	0	0.26	0.24	0.18	0.1	0.15	0.16	0.14	0.15	0.13	0.2
0.28	0.48	0.42	1.11	0.19	0.44	0.18	0	0.02	0.2	0.13	0.15	0.11	0.49	0.06	0	0.26	0.28	0.18	0.1	0.15	0.25	0.2	0.28	0.13	0.31
0.28	0.48	0.42	1.11	0.2	0.44	0.18	0	0.05	0.26	0.29	0.15	0.15	0.5	0.25	0.11	0.26	0.28	0.3	0.11	0.15	0.26	0.24	0.28	0.22	0.32
0.28	0.58	0.45	1.11	0.29	0.44	0.19	0	0.05	0.26	0.3	0.15	0.15	0.54	0.25	0.2	0.31	0.28	0.3	0.11	0.18	0.26	0.24	0.29	0.23	0.41
0.34	0.59	0.46	1.11	0.29	0.53	0.35	0	0.09	0.26	0.3	0.15	0.15	0.62	0.44	0.2	0.31	0.4	0.3	0.24	0.22	0.26	0.31	0.3	0.23	0.41
0.43	0.59	0.51	1.11	0.37	0.55	0.4	0	0.09	0.41	0.38	0.15	0.21	0.66	0.45	0.2	0.37	0.53	0.38	0.27	0.23	0.34	0.38	0.3	0.31	0.49
0.43	0.64	0.52	1.11	0.39	0.64	0.55	0	0.14	0.49	0.38	0.15	0.29	0.76	0.45	0.2	0.39	0.55	0.44	0.28	0.27	0.39	0.6	0.4	0.42	0.67
0.49	0.75	0.61	1.11	0.48	0.75	0.56	0	0.21	0.52	0.48	0.15	0.41	0.76	0.63	0.6	0.43	0.65	0.47	0.28	0.28	0.59	0.61	0.53	0.47	0.84
0.57	0.76	0.78	1.11	0.63	0.77	0.56	0	0.35	0.63	0.49	0.54	0.52	0.78	0.81	0.6	0.5	0.66	0.56	0.34	0.28	0.59	0.61	0.63	0.51	0.85
0.67	0.76	0.92	1.11	0.63	0.9	0.64	0	0.35	0.64	0.55	0.54	0.55	0.95	0.83	0.6	0.51	0.8	0.56	0.46	0.34	0.66	0.61	0.63	0.51	1.01

0.67	0.83	1.01	1.11	0.75	0.91	0.73	0	0.54	0.74	0.65	0.54	0.61	0.95	0.83	0.6	0.69	0.8	0.56	0.46	0.4	0.67	0.72	0.7	0.66	1.12
0.73	0.97	1.01	1.11	0.77	0.97	0.74	0	0.54	0.74	0.66	0.54	0.61	0.95	0.83	0.6	0.76	0.96	0.69	0.52	0.65	0.74	0.82	0.71	0.67	1.27
0.74	1.07	1.11	1.11	0.89	0.97	0.8	0	0.54	0.95	0.73	0.56	0.69	1.07	1.05	0.6	0.76	0.97	0.69	0.52	0.65	0.74	0.87	0.71	0.81	1.28
0.89	1.08	1.16	1.11	1.02	1.12	0.81	0	0.56	1.01	0.73	0.76	0.73	1.08	1.16	0.6	0.76	0.97	0.78	0.72	0.69	0.79	0.87	0.82	0.81	1.42
0.9	1.17	1.16	1.44	1.03	1.27	0.89	0	0.71	1.02	0.74	0.78	0.73	1.17	1.17	0.6	0.94	1.11	0.78	0.88	0.78	0.88	1.27	0.83	0.86	1.43
0.94	1.25	1.22	1.44	1.06	1.29	0.89	0	0.85	1.02	0.79	0.78	0.74	1.22	1.18	0.6	0.95	1.2	0.83	0.88	0.83	1.02	1.27	0.83	0.97	1.53
0.96	1.25	1.37	1.44	1.07	1.29	0.89	0	0.85	1.17	0.92	0.78	0.84	1.23	1.3	0.6	0.95	1.23	0.83	0.88	0.85	1.02	1.37	0.84	1	1.68
1.06	1.3	1.46	1.44	1.07	1.29	0.92	0	0.85	1.29	0.98	0.78	0.84	1.29	1.31	0.67	1.01	1.36	0.89	0.96	0.97	1.02	1.41	0.87	1.07	1.68
1.12	1.37	1.54	1.44	1.11	1.29	0.93	0	0.86	1.29	1.06	0.78	0.84	1.3	1.31	0.77	1.01	1.39	1.02	1.09	1.1	1.12	1.52	0.98	1.16	1.69
1.14	1.51	1.54	1.44	1.17	1.29	0.93	0	0.89	1.3	1.06	0.78	0.84	1.47	1.31	0.86	1.2	1.41	1.02	1.21	1.11	1.17	1.52	1	1.2	1.69
1.18	1.51	1.55	1.44	1.17	1.59	1.01	0	0.89	1.3	1.13	0.78	0.91	1.47	1.39	0.86	1.25	1.6	1.09	1.29	1.23	1.3	1.52	1.14	1.3	1.75
1.2	1.53	1.55	1.44	1.18	1.63	1.19	0	0.89	1.42	1.21	0.78	1.04	1.47	1.4	0.88	1.25	1.66	1.17	1.3	1.34	1.45	1.52	1.33	1.31	1.76
1.27	1.54	1.68	1.44	1.37	1.74	1.28	0	1.15	1.46	1.26	0.78	1.2	1.51	1.51	0.88	1.26	1.68	1.17	1.3	1.35	1.49	1.52	1.34	1.38	1.93
1.52	1.6	1.73	1.44	1.46	1.74	1.28	0	1.27	1.65	1.4	0.78	1.2	1.73	1.68	0.88	1.38	1.81	1.26	1.3	1.44	1.63	1.6	1.35	1.53	1.94
1.55	1.69	1.74	1.44	1.48	1.81	1.36	0	1.38	1.68	1.48	0.78	1.21	1.73	1.71	0.88	1.38	1.86	1.26	1.39	1.44	1.63	1.77	1.42	1.59	2.11
1.56	1.74	1.84	1.44	1.63	1.83	1.37	0	1.38	1.72	1.58	0.78	1.36	1.74	1.71	0.94	1.39	1.93	1.34	1.39	1.46	1.63	1.79	1.51	1.6	2.12
1.61	1.79	1.95	1.44	1.66	1.96	1.43	0	1.42	1.75	1.68	0.78	1.36	1.78	1.71	0.94	1.39	1.95	1.34	1.47	1.55	1.64	1.79	1.66	1.7	2.12
1.68	1.81	2.06	1.44	1.66	1.96	1.43	0	1.42	1.85	1.69	0.86	1.36	1.93	1.72	1.12	1.47	1.95	1.46	1.48	1.6	1.64	1.88	1.72	1.7	2.21
1.68	1.81	2.06	1.44	1.67	1.97	1.52	0	1.48	1.85	1.79	1.61	1.45	1.93	1.82	1.13	1.64	2.06	1.6	1.67	1.6	1.73	1.93	1.72	1.79	2.22
1.81	1.99	2.07	1.44	1.71	2.13	1.53	0	1.5	1.89	1.79	1.79	1.48	2.05	2.04	1.28	1.65	2.1	1.6	1.68	1.66	1.73	2.02	1.72	1.87	2.26
1.92	1.99	2.12	1.44	1.79	2.3	1.66	0	1.68	1.96	1.92	1.82	1.49	2.05	2.04	1.3	1.65	2.17	1.61	1.68	1.66	1.79	2.2	1.72	1.87	2.26
1.92	2.07	2.14	1.44	1.93	2.3	1.66	0	1.68	2.01	1.92	1.83	1.59	2.21	2.04	1.3	1.65	2.29	1.72	1.99	1.66	1.95	2.2	1.82	1.94	2.27
1.93	2.1	2.22	1.44	1.93	2.35	1.67	0	1.68	2.01	1.98	1.83	1.63	2.28	2.04	1.3	1.8	2.29	1.8	2.08	1.77	1.96	2.2	1.82	2.05	2.42
2.14	2.13	2.22	1.44	1.93	2.42	1.67	0	1.68	2.16	2.04	1.97	1.69	2.28	2.04	1.3	1.87	2.38	1.8	2.08	1.83	2.01	2.37	1.88	2.05	2.57
2.14	2.19	2.27	1.44	2.07	2.42	1.73	0	1.69	2.18	2.14	1.97	1.69	2.28	2.16	1.3	2.07	2.48	1.81	2.09	1.97	2.09	2.67	1.97	2.16	2.57
2.16	2.19	2.43	1.44	2.27	2.47	1.73	0	1.69	2.19	2.3	1.97	1.74	2.35	2.16	1.3	2.07	2.51	1.81	2.19	2.07	2.14	2.67	1.97	2.16	2.58
2.25	2.26	2.5	1.44	2.31	2.58	1.73	0	1.77	2.39	2.3	2.21	1.74	2.46	2.32	1.43	2.07	2.54	1.98	2.2	2.15	2.14	2.67	2.02	2.23	2.69
2.26	2.39	2.63	1.44	2.34	2.61	1.8	0	2.02	2.39	2.3	2.21	1.83	2.46	2.39	1.43	2.07	2.59	2.11	2.2	2.15	2.15	2.67	2.02	2.39	2.81
2.26	2.42	2.63	1.44	2.45	2.66	1.8	0	2.26	2.39	2.32	2.21	1.96	2.46	2.45	1.43	2.07	2.64	2.12	2.21	2.15	2.15	2.67	2.02	2.4	2.89
2.26	2.44	2.63	1.44	2.46	2.68	1.81	0	2.26	2.4	2.37	2.21	1.96	2.61	2.53	1.43	2.16	2.69	2.18	2.21	2.41	2.15	2.67	2.09	2.4	2.89
2.41	2.44	2.63	1.44	2.46	2.68	1.93	0	2.31	2.4	2.38	2.21	1.97	2.61	2.53	1.43	2.32	2.71	2.18	2.32	2.41	2.27	2.67	2.17	2.4	2.9
2.42	2.44	2.7	1.44	2.46	2.8	1.93	0	2.31	2.49	2.51	2.28	1.97	2.62	2.53	1.59	2.32	2.81	2.18	2.32	2.41	2.27	2.67	2.17	2.46	2.96
2.45	2.44	2.7	1.44	2.49	2.8	1.94	0	2.31	2.49	2.57	2.28	1.98	2.62	2.53	1.59	2.33	2.83	2.26	2.33	2.41	2.27	2.67	2.17	2.46	2.96
2.45	2.55	2.76	1.44	2.54	2.8	2.03	0	2.31	2.5	2.58	2.28	1.98	2.63	2.53	1.59	2.33	2.83	2.26	2.33	2.41	2.36	2.69	2.18	2.52	2.97
2.56	2.55	2.84	1.44	2.6	2.83	2.03	0	2.31	2.56	2.58	2.28	1.98	2.63	2.53	1.64	2.33	2.83	2.26	2.34	2.41	2.4	2.69	2.34	2.52	3.1
2.56	2.55	2.84	1.44	2.65	2.83	2.04	0	2.36	2.56	2.69	2.28	1.99	2.72	2.62	1.64	2.37	2.88	2.26	2.46	2.51	2.41	2.69	2.34	2.6	3.11
2.57	2.63	2.85	1.44	2.68	3	2.04	0	2.36	2.61	2.69	2.28	2.09	2.79	2.63	1.85	2.37	2.88	2.27	2.47	2.56	2.41	2.69	2.35	2.6	3.11
2.63	2.63	2.9	1.44	2.68	3	2.14	0	2.36	2.61	2.7	2.28	2.09	2.79	2.63	1.85	2.38	2.88	2.27	2.47	2.62	2.41	2.69	2.36	2.6	3.19
2.63	2.63	2.91	1.44	2.76	3	2.14	0	2.36	2.62	2.71	2.28	2.1	2.79	2.79	1.85	2.38	3.07	2.43	2.47	2.64	2.55	2.94	2.36	2.61	3.19
2.63	2.65	2.91	1.44	2.76	3	2.14	0	2.36	2.62	2.71	2.28	2.11	2.79	2.8	1.85	2.38	3.11	2.43	2.47	2.64	2.56	3.04	2.47	2.7	3.32
2.64	2.73	3.04	1.44	2.76	3	2.14	0	2.46	2.63	2.92	2.28	2.11	2.8	2.8	1.85	2.6	3.13	2.5	2.47	2.64	2.56	3.18	2.51	2.7	3.33
2.69	2.74	3.05	1.44	2.76	3.09	2.15	0	2.46	2.63	2.92	2.3	2.19	2.91	2.8	1.85	2.61	3.13	2.5	2.48	2.64	2.65	3.18	2.52	2.77	3.33
2.69	2.74	3.05	1.44	2.78	3.1	2.31	0	2.46	2.88	2.92	2.3	2.19	2.92	2.81	1.85	2.61	3.14	2.56	2.48	2.64	2.66	3.18	2.52	2.78	3.33
2.7	2.75	3.15	1.44	2.81	3.11	2.32	0	2.46	2.88	2.93	2.54	2.19	2.92	2.81	1.85	2.61	3.14	2.56	2.49	2.64	2.66	3.28	2.65	2.79	3.33
2.71	2.75	3.18	1.44	2.93	3.27	2.32	0	2.46	2.89	2.93	2.54	2.19	2.93	2.83	1.85	2.71	3.14	2.56	2.49	2.64	2.66	3.28	2.66	2.83	3.34
2.76	2.87	3.19	1.44	2.93	3.27	2.37	0	2.46	2.89	2.98	2.54	2.19	2.93	2.84	2.01	2.72	3.24	2.56	2.49	2.66	2.66	3.49	2.66	2.83	3.34
2.77	2.97	3.19	1.44	2.93	3.28	2.37	0	2.46	2.9	2.99	2.54	2.19	2.93	2.85	2.37	2.72	3.24	2.56	2.5	2.71	2.67	3.49	2.66	2.88	3.54
2.85	2.97	3.24	1.44	2.93	3.28	2.38	0	2.46	2.9	2.99	2.54	2.2	3.08	3.01	2.37	2.72	3.24	2.57	2.5	2.73	2.76	3.49	2.79	2.88	3.54
2.86	2.99	3.24	1.44	2.93	3.28	2.38	0	2.46	2.91	3	2.54	2.2	3.09	3.01	2.4	2.73	3.24	2.66	2.65	2.73	2.78	3.49	2.79	3.01	3.55
2.89	2.99	3.24	1.44	2.95	3.31	2.38	0	2.46	2.91	3.09	2.54	2.2	3.09	3.02	2.6	2.73	3.36	2.66	2.66	2.79	2.78	3.49	2.79	3.02	3.78
2.97	3.17	3.24	1.44	3	3.35	2.52	0	2.46	3.04	3.1	2.54	2.21	3.14	3.02	2.6	2.73	3.43	2.74	2.66	2.87	2.78	3.49	2.79	3.02	3.78
3.08	3.19	3.35	1.44	3.07	3.35	2.53	0	2.55	3.09	3.14	2.54	2.43	3.14	3.02	2.6	2.91	3.44	2.75	2.67	2.87	2.88	3.49	2.79	3.02	3.79
3.13	3.19	3.35	1.44	3.13	3.44	2.53	0	2.56	3.1	3.2	2.54	2.44	3.15	3.02	2.6	2.91	3.44	2.83	2.67	2.87	2.89	3.49	2.81	3.15	3.79
3.14	3.19	3.36	2.25	3.19	3.48	2.64	0	2.56	3.1	3.21	2.54	2.44	3.15	3.03	2.6	2.91	3.44	2.83	2.67	2.91	2.89	3.49	2.81	3.15	3.79
3.15	3.19	3.37	2.27	3.19	3.49	2.72	0	2.56	3.14	3.21	2.54	2.45	3.29	3.04	2.6	3.01	3.71	2.83	2.9	2.97	2.97	3.49	2.82	3.16	3.79
3.23	3.19	3.45	2.27	3.19	3.49	2.73	0	2.56	3.14	3.29	2.54	2.45	3.41	3.3	2.6	3.01	3.71	2.9	2.9	3.11	2.97	3.49	3.03	3.25	3.86
3.23	3.3	3.45	2.27	3.19	3.49	2.82	0	2.62	3.15	3.3	2.54	2.67	3.43	3.3	2.6	3.01	3.71	2.9	2.91	3.22	2.97	3.49	3.04	3.25	3.87
3.24	3.31	3.68	2.27	3.19	3.56	2.83	0	2.78	3.15	3.45	2.99	2.68	3.46	3.31	2.6	3.02	3.71	2.9	2.91	3.25	3.15	3.57	3.04	3.31	4.02
3.3	3.42	3.72	2.33	3.22	3.59	2.97	0	2.78	3.19	3.46	3.08	2.74	3.47	3.42	2.6	3.02	3.81	3.16	2.92	3.26	3.16	3.85	3.04	3.31	4.12
3.34	3.43	3.72	5.2	3.38	3.71	2.98	0	2.86	3.24	3.5	3.08	2.74	3.52	3.42	2.6	3.13	3.84	3.19	2.92	3.26	3.16	3.85	3.09	3.41	4.12
3.38	3.48	3.72	5.8	3.39	3.8	3.14	0	2.98	3.38	3.56	3.09	2.74	3.61	3.43	2.6	3.14	4	3.2	3.09	3.34	3.21	3.85	3.19	3.42	4.12
3.38	3.55	3.76	5.8	3.39	3.83	3.2	0	2.98	3.5	3.56	3.15	2.75	3.62	3.5	2.6	3.19	4.05	3.2	3.17	3.36	3.22	3.85	3.2	3.47	4.12
3.46	3.6	3.82	8.29	3.47	3.96	3.2	2.47	3.03	3.55	3.56	3.26	2.86	3.82	3.53	2.6	3.29	4.09	3.29	3.17	3.41	3.36	3.86	3.2	3.55	4.26
3.46	3.68	3.89	8.29	3.52	4.12	3.24	2.72	3.25	3.62	3.77	3.26	2.96	3.83	3.63	2.6	3.35	4.17	3.3	3.18	3.52	3.36	4.34	3.31	3.55	4.34
3.52	3.7	4	8.29	3.63	4.15	3.38	2.85	3.44	3.67	3.87	3.36	3.03	3.83	3.78	2.6	3.35	4.29	3.49	3.34	3.52	3.44	4.35	3.44	3.7	4.51
3.63	3.85	4.03	8.29	3.66	4.32	3.43	2.85	3.44	3.67	3.88	3.36	3.03	4.01	3.79	2.66	3.57	4.29	3.5	3.34	3.59	3.51	4.46	3.45	3.71	4.51
3.63	3.86	4.07	8.29	3.75	4.32	3.51	2.85	3.44	3.67	3.98	3.36	3.03	4.01	3.94	2.84	3.63	4.31	3.51	3.44	3.6	3.66	4.46	3.45	3.83	4.61
3.74	3.86	4.09	9.36	3.84	4.32	3.51	2.85	3.5	3.94	4.06	3.36	3.11	4.01	3.94	2.84	3.63	4.46	3.51	3.6	3.75	3.69	4.46	3.68	3.94	4.62
3.76	3.91	4.19	9.36	3.96	4.39	3.64	2.85	3.67	3.99	4.06	3.36	3.11	4.19	3.95	2.87	3.63	4.46	3.68	3.75	3.8	3.78	4.46	3.69	3.95	4.75
3.81	3.96	4.32	9.36	3.96	4.57	3.65	2.85	3.95	3.99	4.17	3.36	3.19	4.19	4.12	3.23	3.82	4.46	3.76	3.78	3.8	3.91	4.46	3.76	4.03	4.93
3.83	3.96	4.32	9.36	3.97	4.57	3.65	2.85	3.95	4.04	4.35	3.36	3.33	4.3	4.12	3.23	3.95	4.54	3.78	3.78	4	3.92	4.66	3.8	4.08	4.97
3.88	4.05	4.38	9.36	3.97	4.57	3.8	2.85	3.95	4.05	4.41	3.36	3.4	4.39	4.14	3.24	4.03	4.59	3.84	3.79	4.16	3.97	4.66	3.8	4.19	4.97
4.05	4.11	4.42	9.36	4.07	4.57	4.02	2.85	3.95	4.05	4.42	3.67	3.4	4.39	4.14	3.24	4.03	4.69	3.91	3.79	4.24	4.02	4.66	3.89	4.28	4.97
4.05	4.17	4.42	9.36	4.17	4.69	4.09	2.85	3.95	4.05	4.43	3.94	3.48	4.39	4.3	3.28	4.11	4.83	4.02	3.89	4.39	4.06	4.66	3.96	4.29	4.97
4.19	4.18	4.52	9.36	4.17	4.75	4.09	2.85	4.27	4.14	4.57	3.95	3.49	4.48	4.37	3.3	4.11	4.91	4.1	3.98	4.39	4.12	4.66	3.97	4.48	5.1
4.19	4.23	4.66	9.36	4.25	4.79	4.09	2.85	4.36	4.33	4.68	3.97	3.58	4.49	4.37	3.42	4.13	4.91	4.11	4.17	4.44	4.12	5.03	4.05	4.51	5.11
4.26	4.25	4.66	9.36	4.37	5.06	4.19	2.85	4.36	4.33	4.84	3.97	3.59	4.72	4.37	3.42	4.25	4.91	4.21	4.28	4.48	4.22	5.09	4.06	4.64	5.34
4.37	4.31	4.66	9.36	4.46	5.12	4.2	2.85	4.36	4.33	4.84	4.18	3.6	4.75	4.5	3.43	4.36	5.04	4.32	4.28	4.55	4.39	5.51	4.24	4.66	5.34
4.48	4.4	4.76	10.6	4.49	5.12	4.31	2.85	4.36	4.54	4.85	4.2	3.64	4.83	4.5	3.52	4.51	5.08	4.4	4.29	4.6	4.44	5.57	4.25	4.75	5.41
4.52	4.48	4.82	10.6	4.65	5.28	4.48	2.85	4.37	4.58	4.93	4.28	3.64	4.93	4.59	3.78	4.51	5.14	4.47	4.37	4.75	4.45	5.57	4.35	4.75	5.5
4.52	4.51	4.88	10.6	4.73	5.35	4.48	2.85	4.49	4.6	4.99	4.36	3.74	5.03	4.83	3.78	4.51	5.25	4.47	4.56	4.91	4.45	5.57	4.35	4.87	5.5
4.54	4.51	5.02	10.6	4.73	5.35	4.48	2.85	4.49	4.71	5.07	4.36	3.95	5.05	4.93	3.78	4.57	5.29	4.53	4.57	4.94	4.55	5.66	4.48	4.98	5.5
4.66	4.53	5.02	10.6	4.73	5.35	4.63	2.85	4.49	4.76	5.17	4.41	4.03	5.05	4.93	3.8	4.68	5.3	4.62	4.67	4.94	4.7	5.66	4.49	4.98	5.51
4.66	4.55	5.02	10.6	4.82	5.42	4.63	2.85	4.57	4.76	5.29	4.48	4.03	5.15	4.93	4.07	4.79	5.54	4.66	4.67	5.13	4.7	5.66	4.61	5.07	5.51
4.68	4.57	5.1	10.6	4.88	5.53	4.69	2.85	4.57	4.76	5.3	4.53	4.08	5.16	5.06	4.13	4.79	5.54	4.66	4.67	5.13	4.7	5.66	4.62	5.07	5.51
4.72	4.61	5.1	10.6	4.99	5.55	4.7	2.85	4.57	4.76	5.41	4.61	4.17	5.21	5.06	4.3	4.97	5.58	4.72	4.79	5.27	4.7	5.66	4.62	5.15	5.59
4.79	4.61	5.19	10.6	5.04	5.72	4.76	2.85	5.1	4.76	5.41	4.61	4.22	5.32	5.07	4.3	5.05	5.69	4.78	4.91	5.32	4.7	5.66	4.62	5.23	5.75
4.8	4.68	5.27	10.6	5.09	5.8	4.82	2.85	5.1	4.82	5.47	4.75	4.24	5.32	5.07	4.3	5.09	5.77	4.79	4.92	5.35	4.84	5.73	4.63	5.23	5.75
4.87	4.71	5.28	10.6	5.09	5.96	4.84	2.85	5.1	4.91	5.59	4.94	4.24	5.42	5.07	4.3	5.1	5.83	4.94	5	5.58	4.86	5.84	4.77	5.32	5.86
4.97	4.83	5.28	10.6	5.27	5.96	4.88	2.85	5.12	5.09	5.68	4.94	4.33	5.42	5.17	4.3	5.18	5.83	4.99	5.24	5.58	5.01	5.87	4.77	5.39	5.99
5.07	5.03	5.39	10.6	5.27	5.96	4.89	2.85	5.36	5.09	5.68	5.04	4.43	5.43	5.28	4.3	5.18	5.85	5.21	5.31	5.59	5.01	6.16	4.93	5.48	6.04
5.09	5.05	5.55	10.6	5.29	5.96	5.06	2.85	5.52	5.09	5.69	5.04	4.56	5.49	5.39	4.3	5.4	6.02	5.27	5.32	5.59	5.1	6.17	4.93	5.48	6.04
5.11	5.05	5.6	10.6	5.56	5.96	5.06	2.85	5.52	5.09	5.81	5.2	4.56	5.67	5.39	4.32	5.41	6.13	5.27	5.32	5.86	5.22	6.18	4.93	5.66	6.04
5.15	5.1	5.6	10.6	5.56	5.97	5.13	3.36	5.52	5.14	5.91	5.21	4.64	5.72	5.51	4.32	5.41	6.13	5.27	5.32	5.86	5.23	6.19	4.93	5.68	6.2
5.2	5.11	5.6	10.6	5.56	6.13	5.13	3.36	5.52	5.14	6.08	5.27	4.64	5.73	5.51	4.35	5.41	6.13	5.37	5.49	5.9	5.3	6.2	4.93	5.68	6.2
5.21	5.2	5.65	10.62	5.56	6.19	5.13	3.36	5.52	5.34	6.19	5.27	4.64	5.77	5.51	4.35	5.41	6.13	5.37	5.5	5.97	5.3	6.23	5.03	5.68	6.2
5.22	5.2	5.65	10.62	5.56	6.28	5.19	3.36	5.52	5.43	6.19	5.33	4.64	5.77	5.51	4.35	5.43	6.13	5.37	5.5	6	5.31	6.29	5.08	5.69	6.21
5.22	5.33	5.65	10.62	5.56	6.29	5.19	3.38	5.92	5.43	6.24	5.41	4.65	5.84	5.76	4.35	5.43	6.13	5.37	5.5	6.09	5.44	6.57	5.09	5.79	6.21
5.23	5.33	5.73	10.62	5.6	6.32	5.32	3.38	5.92	5.44	6.24	5.41	4.65	5.9	5.76	4.35	5.44	6.13	5.42	5.5	6.13	5.44	6.59	5.09	5.8	6.27
5.35	5.34	5.73	10.62	5.68	6.32	5.33	3.38	5.98	5.44	6.25	5.46	4.66	5.9	5.77	4.38	5.51	6.13	5.42	5.51	6.13	5.44	6.65	5.09	5.8	6.27
5.36	5.35	5.89	10.62	5.68	6.32	5.33	3.38	5.98	5.54	6.26	5.58	4.66	5.9	5.77	4.53	5.51	6.13	5.43	5.51	6.22	5.45	6.65	5.09	5.85	6.41
5.36	5.35	5.89	10.62	5.78	6.36	5.34	3.38	5.98	5.54	6.26	5.58	4.74	5.99	5.77	4.53	5.52	6.3	5.55	5.63	6.24	5.45	6.65	5.25	5.85	6.41
5.42	5.45	5.89	10.62	5.82	6.36	5.4	3.38	6.01	5.54	6.27	5.58	4.75	5.99	5.77	4.62	5.52	6.3	5.55	5.63	6.24	5.45	6.65	5.25	5.85	6.41
5.42	5.57	5.96	10.62	5.93	6.53	5.48	3.38	6.01	5.54	6.36	5.58	4.8	6.05	5.82	4.74	5.52	6.3	5.55	5.64	6.24	5.54	6.65	5.26	5.85	6.5
5.43	5.57	5.96	10.62	5.94	6.58	5.48	3.38	6.19	5.55	6.36	5.62	4.8	6.11	5.89	4.83	5.64	6.3	5.56	5.64	6.4	5.61	6.65	5.26	6	6.63
5.44	5.58	6.03	10.62	5.94	6.58	5.6	3.38	6.19	5.55	6.37	5.63	4.81	6.11	5.89	4.83	5.64	6.42	5.68	5.65	6.53	5.61	6.65	5.43	6	6.63
5.54	5.7	6.03	10.62	5.94	6.61	5.6	3.38	6.19	5.55	6.43	5.73	4.81	6.18	6.06	4.9	5.65	6.43	5.68	5.65	6.53	5.62	6.65	5.43	6	6.63
5.55	5.7	6.03	10.62	6.1	6.67	5.6	3.38	6.19	5.68	6.44	5.73	4.81	6.18	6.06	4.9	5.84	6.43	5.68	5.78	6.54	5.62	6.65	5.43	6	6.63
5.55	5.7	6.04	10.62	6.1	6.67	5.6	3.38	6.19	5.74	6.53	5.73	4.98	6.18	6.07	4.9	5.85	6.43	5.69	5.79	6.54	5.62	6.65	5.44	6.08	6.69
5.56	5.71	6.11	10.62	6.1	6.67	5.67	3.38	6.19	5.74	6.53	5.76	4.98	6.18	6.07	4.9	5.85	6.58	5.8	5.79	6.54	5.75	6.65	5.44	6.08	6.7
5.67	5.81	6.22	10.62	6.11	6.69	5.68	3.38	6.19	5.74	6.54	5.76	5.06	6.24	6.07	4.9	5.86	6.59	5.8	5.8	6.64	5.75	6.87	5.58	6.08	6.88
5.67	5.81	6.22	10.62	6.13	6.69	5.68	3.38	6.19	5.75	6.54	5.91	5.06	6.35	6.16	4.9	5.86	6.59	5.8	5.8	6.66	5.75	6.87	5.58	6.08	6.88
5.67	5.81	6.22	10.62	6.13	6.72	5.74	3.79	6.19	5.75	6.71	5.91	5.07	6.35	6.16	4.9	5.86	6.59	5.94	5.81	6.78	5.79	6.87	5.59	6.08	6.88
5.68	5.83	6.23	10.62	6.25	6.73	5.74	3.82	6.19	5.75	6.71	5.91	5.07	6.36	6.17	4.9	5.86	6.72	5.95	5.81	6.78	5.79	6.87	5.59	6.12	6.88
5.68	5.93	6.23	10.74	6.33	6.73	5.75	3.82	6.19	5.75	6.72	5.91	5.07	6.37	6.25	4.9	5.87	6.74	5.95	5.81	6.79	5.8	6.87	5.59	6.12	6.88
5.72	5.96	6.23	10.74	6.33	6.73	5.8	3.93	6.19	5.85	6.73	5.91	5.08	6.37	6.25	4.92	5.87	6.75	6.04	5.81	6.87	5.8	6.87	5.59	6.12	6.88
5.73	5.96	6.24	10.74	6.33	6.79	5.82	3.98	6.33	5.86	6.79	5.94	5.08	6.37	6.38	4.92	6.16	6.77	6.04	5.82	6.92	5.8	6.87	5.6	6.13	6.98
5.75	5.96	6.36	10.74	6.38	6.89	6	4.03	6.33	5.86	6.86	6.01	5.09	6.57	6.38	4.92	6.17	6.86	6.05	5.82	6.92	5.8	7.17	5.6	6.13	6.99
5.8	5.96	6.36	10.74	6.4	6.96	6	4.03	6.33	5.86	6.87	6.01	5.19	6.65	6.39	4.96	6.17	6.88	6.15	5.83	6.94	5.9	7.17	5.6	6.18	6.99
5.84	6.07	6.37	10.74	6.4	6.96	6	4.03	6.33	5.86	6.91	6.02	5.19	6.65	6.55	4.96	6.18	6.88	6.15	5.95	6.96	5.91	7.17	5.75	6.29	7.07
5.88	6.07	6.44	10.74	6.4	6.97	6.01	4.03	6.33	5.98	6.91	6.02	5.37	6.67	6.56	4.96	6.18	6.88	6.16	5.96	7.04	5.91	7.17	5.83	6.33	7.07
5.88	6.18	6.44	10.74	6.66	7.3	6.07	4.03	6.33	6.12	6.92	6.14	5.38	6.69	6.64	4.96	6.19	7.05	6.29	6.08	7.06	5.99	7.17	5.83	6.33	7.07
5.94	6.19	6.51	10.74	6.69	7.3	6.09	4.03	6.46	6.13	6.99	6.24	5.38	6.76	6.93	4.96	6.19	7.05	6.3	6.19	7.16	5.99	7.17	5.83	6.33	7.19
5.94	6.19	6.51	10.74	6.7	7.3	6.09	4.03	6.63	6.13	7.01	6.24	5.38	6.76	6.93	4.96	6.32	7.05	6.33	6.2	7.16	5.99	7.17	5.84	6.34	7.43
5.95	6.2	6.52	10.74	6.73	7.38	6.14	4.03	6.63	6.13	7.02	6.24	5.39	6.77	6.94	5.06	6.41	7.11	6.34	6.2	7.16	5.99	7.17	5.84	6.38	7.43
5.95	6.22	6.59	10.74	6.73	7.44	6.14	4.07	6.63	6.24	7.09	6.24	5.4	6.85	6.94	5.13	6.42	7.11	6.44	6.21	7.21	6	7.17	5.9	6.48	7.43
6.09	6.35	6.59	10.74	6.73	7.44	6.32	4.2	6.66	6.39	7.1	6.34	5.46	6.86	6.95	5.31	6.42	7.26	6.45	6.21	7.28	6.01	7.31	6.08	6.48	7.43
6.24	6.36	6.73	10.74	6.81	7.72	6.4	4.3	6.74	6.44	7.19	6.4	5.67	7.02	7.04	5.33	6.54	7.29	6.45	6.37	7.28	6.15	7.31	6.1	6.55	7.43
6.24	6.36	6.78	10.74	6.96	7.74	6.45	4.3	6.74	6.54	7.29	6.4	5.75	7.02	7.04	5.51	6.58	7.42	6.48	6.47	7.37	6.16	7.44	6.1	6.65	7.56
6.26	6.45	6.94	10.74	7.08	7.76	7.09	4.3	6.81	6.54	7.33	6.5	5.8	7.02	7.22	5.66	6.65	7.48	6.62	6.6	7.45	6.27	7.44	6.1	6.73	7.6
6.34	6.48	6.94	10.74	7.1	7.78	7.2	4.3	6.81	6.55	7.44	6.5	5.85	7.13	7.27	5.66	6.7	7.55	6.63	6.6	7.76	6.31	7.5	6.19	6.8	7.71
6.43	6.54	6.99	10.74	7.21	7.86	7.28	4.3	6.81	6.63	7.44	6.5	5.85	7.14	7.27	5.76	6.71	7.55	6.69	6.66	7.77	6.31	7.5	6.27	6.8	7.78

6.57	6.54	7.14	10.74	7.21	7.95	7.33	4.3	6.92	6.82	7.44	6.5	6.02	7.22	7.42	5.76	6.8	7.55	6.77	6.67	7.77	6.46	7.5	6.28	6.88	7.94
6.6	6.61	7.2	10.74	7.21	7.95	7.4	5.25	6.92	6.82	7.56	6.62	6.1	7.26	7.56	5.76	6.88	7.66	6.85	6.82	7.85	6.58	7.55	6.41	7	7.98
6.79	6.64	7.21	10.74	7.48	8.01	7.47	5.25	7.05	6.83	7.57	6.62	6.1	7.29	7.62	6	6.94	7.67	6.92	6.83	7.93	6.69	7.55	6.48	7.05	8.06
6.79	6.7	7.21	10.74	7.58	8.01	7.51	5.25	7.21	6.83	7.67	6.62	6.1	7.34	7.66	6	7.02	7.67	6.93	6.95	7.97	6.69	7.56	6.51	7.14	8.15
6.86	6.7	7.25	10.74	7.58	8.06	7.52	5.25	7.41	6.83	7.77	6.67	6.2	7.41	7.66	6	7.07	7.67	6.97	6.95	8.05	6.69	7.84	6.52	7.19	8.15
6.88	6.7	7.38	10.74	7.6	8.23	7.62	5.25	7.41	6.95	7.95	6.74	6.29	7.43	7.76	6	7.12	7.88	7.06	7	8.21	6.75	7.92	6.65	7.19	8.15
6.94	6.7	7.38	10.74	7.84	8.3	7.62	5.25	7.49	7	8.05	6.85	6.38	7.59	7.76	6.77	7.24	8.04	7.07	7.17	8.28	6.76	7.92	6.67	7.3	8.26
7	6.92	7.38	10.74	7.88	8.41	8.2	5.25	7.49	7	8.11	6.91	6.47	7.59	7.87	6.84	7.32	8.04	7.18	7.34	8.28	6.76	7.92	6.87	7.3	8.27
7	6.95	7.56	10.74	7.96	8.43	9	5.25	7.68	7	8.19	6.91	6.51	7.6	7.95	6.84	7.42	8.04	7.24	7.37	8.36	6.88	7.92	6.87	7.42	8.27
7.07	7.01	7.76	10.74	7.96	8.49	9	5.25	7.68	7.26	8.26	6.91	6.51	7.8	8.11	6.84	7.53	8.04	7.29	7.37	8.52	7	7.92	6.94	7.48	8.4
7.2	7.05	7.8	10.74	7.96	8.55	9	5.25	7.74	7.27	8.35	6.91	6.52	7.84	8.29	6.84	7.53	8.18	7.39	7.43	8.53	7.12	8.08	6.96	7.56	8.4
7.37	7.05	7.84	10.74	7.96	8.55	9.1	5.61	7.74	7.3	8.36	7.01	6.7	7.84	8.29	6.84	7.59	8.36	7.5	7.43	8.59	7.12	8.46	6.96	7.66	8.4
7.37	7.06	7.84	10.74	8.27	8.64	9.15	5.61	7.74	7.35	8.43	7.2	6.71	7.85	8.29	6.84	7.64	8.45	7.56	7.44	8.59	7.17	8.48	7.05	7.8	8.6
7.45	7.06	7.87	10.74	8.33	8.64	9.17	5.61	7.84	7.35	8.44	7.2	6.71	7.92	8.29	6.84	7.64	8.45	7.56	7.61	8.68	7.17	8.48	7.05	7.86	8.6
7.45	7.16	7.97	10.75	8.39	8.73	9.23	5.61	7.84	7.48	8.54	7.2	6.84	7.96	8.29	6.84	7.73	8.47	7.61	7.67	8.69	7.26	8.52	7.15	7.86	8.6
7.46	7.16	8.02	11.08	8.39	8.9	9.29	5.61	7.84	7.52	8.63	7.2	6.92	8.03	8.29	6.84	7.86	8.56	7.71	7.77	8.77	7.26	8.52	7.25	7.99	8.61
7.51	7.21	8.11	11.22	8.39	8.92	9.74	5.61	7.84	7.56	8.68	7.52	6.92	8.06	8.41	6.84	8.11	8.64	7.71	7.88	9.05	7.4	8.7	7.34	8.11	8.69
7.57	7.25	8.22	12.11	8.41	8.95	9.84	5.61	8.05	7.56	8.69	7.52	6.95	8.17	8.53	6.84	8.11	8.64	7.72	7.88	9.12	7.4	9	7.35	8.11	8.69
7.69	7.32	8.22	12.11	8.41	8.99	9.84	5.61	8.14	7.57	8.78	7.52	7.02	8.18	8.57	6.84	8.11	8.68	7.87	7.95	9.13	7.45	9	7.35	8.11	8.7
7.81	7.4	8.22	12.11	8.49	9.07	9.85	5.61	8.2	7.75	8.86	7.66	7.13	8.31	8.68	6.99	8.21	8.75	7.9	8.08	9.15	7.58	9	7.36	8.2	8.82
7.82	7.46	8.25	12.11	8.7	9.07	9.89	5.61	8.35	7.8	8.95	7.75	7.14	8.43	8.68	6.99	8.22	8.82	7.9	8.09	9.23	7.59	9	7.5	8.26	8.92
7.87	7.46	8.3	12.11	8.71	9.15	9.89	5.61	8.44	7.84	9.02	7.75	7.32	8.44	8.68	6.99	8.22	8.94	8.01	8.09	9.23	7.73	9.08	7.5	8.32	8.93
7.88	7.51	8.31	12.11	8.87	9.15	9.92	5.61	8.62	7.84	9.1	7.75	7.45	8.53	8.74	6.99	8.43	8.98	8.02	8.12	9.46	7.73	9.32	7.51	8.46	8.99
7.9	7.54	8.36	12.11	8.87	9.28	10.56	5.61	8.62	7.89	9.1	7.75	7.45	8.58	8.89	6.99	8.43	8.98	8.08	8.19	9.57	7.88	9.32	7.6	8.57	9.06
8	7.65	8.41	12.11	8.96	9.33	10.56	5.61	8.62	8.1	9.2	7.95	7.51	8.58	8.94	6.99	8.43	8.98	8.13	8.25	9.57	7.97	9.32	7.6	8.8	9.23
8.01	7.66	8.57	12.11	9.06	9.39	10.56	5.61	8.63	8.13	9.2	7.95	7.63	8.58	8.94	6.99	8.51	9.19	8.29	8.4	9.57	7.97	9.71	7.6	8.8	9.23
8.01	7.66	8.62	12.11	9.44	9.44	10.61	5.61	8.78	8.13	9.2	7.99	7.63	8.74	8.94	6.99	8.62	9.21	8.29	8.41	9.83	8.06	9.71	7.75	8.81	9.24
8.11	7.66	8.73	12.11	9.44	9.56	10.61	5.61	8.87	8.13	9.2	8.01	7.63	8.74	8.94	6.99	8.62	9.21	8.3	8.41	9.84	8.06	9.71	7.88	8.88	9.24
8.11	7.66	8.74	12.11	9.44	9.56	10.71	5.61	8.94	8.13	9.5	8.01	7.64	8.81	9.05	6.99	8.62	9.21	8.43	8.41	9.88	8.08	9.71	7.88	8.92	9.24
8.12	7.66	8.74	12.11	9.44	9.56	10.71	5.61	8.94	8.14	9.51	8.01	7.64	8.81	9.25	7.04	8.62	9.21	8.43	8.58	9.95	8.09	9.71	7.88	8.93	9.35
8.13	7.66	8.77	12.11	9.44	9.56	10.71	5.61	8.94	8.15	9.52	8.01	7.65	8.86	9.25	7.09	8.62	9.21	8.44	8.59	9.95	8.09	9.71	7.88	8.93	9.35
8.14	7.77	8.77	12.11	9.44	9.7	10.72	5.61	8.94	8.23	9.53	8.18	7.74	8.98	9.25	7.09	8.73	9.22	8.44	8.71	9.95	8.17	9.71	7.88	8.93	9.51
8.14	7.91	8.77	12.11	9.44	9.72	10.96	5.61	8.94	8.24	9.59	8.18	7.74	8.98	9.25	7.19	8.73	9.37	8.44	8.72	9.96	8.17	9.71	7.97	8.93	9.51
8.15	7.91	8.85	12.11	9.44	9.72	10.97	5.61	9.06	8.34	9.59	8.2	7.75	9.05	9.25	7.22	8.73	9.37	8.52	8.72	9.96	8.17	9.71	7.97	8.99	9.51
8.25	7.91	8.92	12.11	9.59	9.72	10.97	5.61	9.17	8.45	9.6	8.28	7.75	9.16	9.45	7.22	8.73	9.38	8.57	8.73	10.02	8.23	9.71	7.97	8.99	9.63
8.25	7.91	8.92	12.11	9.59	9.92	10.97	5.61	9.17	8.46	9.61	8.3	7.86	9.16	9.45	7.22	8.75	9.39	8.57	8.74	10.02	8.24	9.71	8.09	8.99	9.63
8.26	7.91	8.92	12.11	9.59	9.94	10.98	5.61	9.3	8.46	9.61	8.33	7.87	9.16	9.46	7.25	8.94	9.4	8.68	8.74	10.02	8.24	9.71	8.09	9.13	9.63
8.26	8.02	8.92	12.11	9.59	10.06	10.98	5.61	9.53	8.46	9.71	8.33	7.87	9.24	9.57	7.25	8.94	9.41	8.69	8.9	10.03	8.24	9.87	8.09	9.16	9.63
8.31	8.02	9.04	12.11	9.62	10.06	10.98	5.61	9.53	8.46	9.72	8.55	7.87	9.29	9.58	7.3	8.95	9.54	8.69	8.9	10.03	8.43	11	8.25	9.24	9.64
8.32	8.03	9.05	12.11	9.62	10.06	11.02	5.61	9.53	8.46	9.73	8.55	7.88	9.29	9.58	7.3	8.98	9.63	8.69	8.91	10.2	8.44	11	8.28	9.24	9.64
8.37	8.03	9.08	12.11	9.67	10.2	11.12	5.61	9.53	8.49	9.81	8.55	7.88	9.37	9.58	7.3	8.98	9.63	8.75	8.91	10.21	8.44	11	8.28	9.32	9.84
8.37	8.03	9.08	12.11	9.67	10.2	11.12	5.61	9.53	8.5	9.82	8.55	7.89	9.38	9.66	7.36	8.98	9.63	8.75	8.92	10.21	8.44	11	8.29	9.32	9.84
8.45	8.03	9.08	12.11	9.7	10.2	11.12	5.61	9.53	8.5	9.82	8.55	7.93	9.48	9.75	7.36	8.99	9.63	8.76	8.92	10.34	8.48	11	8.36	9.33	9.84
8.45	8.03	9.08	12.11	9.7	10.2				8.64	9.83	8.55	7.94	9.48	9.75			9.71	8.76	9	10.34	8.48	11	8.36	9.34	9.84
8.46	8.04	9.09	12.11	9.72	10.24				8.65	9.83	8.55	7.94	9.48	9.76			9.72	8.76	9.18	10.35	8.48	11	8.42	9.35	9.95
8.55	8.04	9.09	12.11	9.78	10.28				8.72	9.83	8.55	7.96	9.49	9.76			9.74	8.92	9.18	10.35	8.52	11	8.47	9.38	9.96
8.55	8.05	9.09	12.11	9.91	10.29				8.72	9.93	8.55	8.1	9.49	9.76			9.75	8.93	9.18	10.35	8.52	11	8.47	9.4	9.96
8.56	8.13	9.17	12.11	9.91	10.3				8.72	9.93	8.55	8.1	9.49	9.78			9.75	8.93	9.19	10.35	8.52	11	8.47	9.43	9.96
8.56	8.32	9.26	12.11	9.91	10.32				8.79	10.01	8.55	8.11	9.66	9.78			9.87	9.06	9.27	10.36	8.53	11	8.53	9.43	9.96
8.57	8.33	9.26	12.11	9.98	10.32				8.89	10.01	8.55	8.11	9.72	9.94			9.87	9.07	9.27	10.36	8.53	11	8.53	9.49	10.2
8.61	8.34	9.26	12.11	10.06	10.34				8.9	10.01	8.55	8.11	9.73	9.95			9.87	9.11	9.28	10.36	8.53	11	8.63	9.67	10.2
8.62	8.35	9.27	12.11	10.07	10.47				8.9	10.02	8.55	8.12	9.73	10.03			9.89	9.12	9.28	10.57	8.53	11	8.63	9.67	10.21
8.62	8.35	9.27	12.11	10.08	10.7				8.9	10.03	8.55	8.2	9.79	10.03			10.12	9.12	9.29	10.58	8.62	11	8.69	9.67	10.21
8.7	8.35	9.48	12.11	10.27	10.7				8.9	10.03	8.89	8.21	9.79	10.03			10.14	9.18	9.29	10.59	8.71	11	8.7	9.67	10.21
8.78	8.49	9.51	12.43	10.28	10.7				9.09	10.04	8.89	8.21	9.8	10.03			10.17	9.18	9.29	10.59	8.71	11	8.7	9.76	10.21
8.82	8.5	9.51	12.43	10.28	10.7				9.1	10.28	8.96	8.22	9.89	10.04			10.18	9.18	9.3	10.59	8.72	11	8.71	9.76	10.28
8.84	8.51	9.51	12.43	10.28	10.89				9.1	10.28	8.96	8.31	9.9	10.12			10.19	9.18	9.41	10.59	8.81	11	8.71	9.76	10.36
8.84	8.6	9.58	12.43	10.28	10.96				9.1	10.29	8.96	8.31	9.9	10.23			10.25	9.31	9.58	10.71	8.81	11	8.71	9.76	10.36
9.07	8.6	9.58	12.43	10.28	10.98				9.1	10.29	8.96	8.37	9.97	10.32			10.27	9.38	9.58	10.71	8.81	11	8.88	9.87	10.36
9.08	8.61	9.62	12.43	10.34	10.98				9.26	10.3	8.96	8.37	9.98	10.34			10.29	9.39	9.59	10.76	8.87	11	8.88	9.9	10.49
9.16	8.61	9.74	12.43	10.41	10.98				9.45	10.31	8.96	8.59	10.11	10.46			10.47	9.39	9.59	10.87	8.91	11	8.88	9.9	10.61
9.2	8.67	9.87	12.43	10.63	11.14				9.47	10.4	8.96	8.6	10.21	10.56			10.48	9.5	9.73	10.91	9.1	11	8.97	9.99	10.68
9.22	8.7	9.91	12.43	10.63	11.19				9.52	10.45	9	8.6	10.21	10.67			10.52	9.51	9.87	11.02	9.1	11	9.06	10.08	10.76
9.42	8.78	9.91	12.44	10.72	11.2				9.52	10.49	9.28	8.72	10.3	10.78			10.52	9.58	9.87	11.03	9.2	11	9.08	10.13	10.86
9.42	8.78	10.05	12.54	10.72	11.23				9.55	10.57	9.31	8.74	10.43	10.78			10.63	9.64	10.02	11.11	9.2	11	9.08	10.22	10.96
9.47	8.78	10.14	12.99	10.77	11.3				9.59	10.58	9.41	8.77	10.45	10.79			10.64	9.65	10.06	11.26	9.2	11.08	9.18	10.28	11.05
9.55	8.79	10.14	12.99	10.89	11.37				9.59	10.68	9.47	8.82	10.48	10.85			10.68	9.78	10.06	11.33	9.21	11.22	9.22	10.38	11.05
9.63	8.8	10.14	12.99	10.89	11.46				9.72	10.72	9.6	8.86	10.55	10.9			10.8	9.82	10.06	11.33	9.31	11.22	9.23	10.39	11.05
9.63	8.8	10.24	12.99	10.89	11.46				9.8	10.72	9.6	8.91	10.58	10.99			10.83	9.9	10.06	11.45	9.31	11.22	9.28	10.52	11.05
9.67	8.8	10.4	12.99	10.89	11.63				9.85	10.82	9.7	8.96	10.6	11.13			10.84	9.9	10.27	11.45	9.31	11.22	9.38	10.59	11.29
9.72	8.92	10.44	12.99	11.19	11.63				9.85	10.85	9.7	9.04	10.7	11.13			10.91	10.04	10.28	11.46	9.45	11.22	9.48	10.67	11.35
9.72	9	10.44	12.99	11.3	11.69				9.85	11	9.72	9.1	10.71	11.17			10.92	10.11	10.52	11.67	9.58	11.53	9.5	10.85	11.35
9.99	9	10.48	12.99	11.3	11.81				9.98	11	10	9.13	10.8	11.29			11.07	10.21	10.6	11.72	9.62	11.53	9.59	10.86	11.44
9.99	9	10.5	12.99	11.31	11.93				10	11.01	10	9.19	10.86	11.31			11.07	10.24	10.6	11.72	9.63	11.53	9.66	10.96	11.44
10.04	9	10.7	12.99	11.48	11.96				10.1	11.15	10.1	9.2	10.86	11.57			11.17	10.3	10.74	11.9	9.69	11.53	9.69	11.03	11.53
10.05	9	10.7	12.99	11.48	11.96				10.15	11.19	10.1	9.3	10.98	11.57			11.21	10.43	10.78	11.91	9.76	11.53	9.78	11.1	11.68
10.11	9.19	10.8	12.99	11.55	11.96				10.24	11.31	10.1	9.41	10.99	11.62			11.21	10.43	10.84	11.91	9.77	11.53	9.78	11.19	11.75
10.16	9.19	10.8	12.99	11.64	12.08				10.3	11.37	10.17	9.41	11.02	11.63			11.26	10.53	10.97	12	9.88	11.64	9.81	11.26	11.75
10.16	9.19	10.86	12.99	11.64	12.16				10.3	11.55	10.23	9.47	11.22	11.7			11.3	10.67	11.05	12.1	9.88	11.69	9.93	11.29	11.76
10.22	9.29	10.86	12.99	11.8	12.32				10.3	11.61	10.23	9.62	11.25	11.79			11.31	10.68	11.18	12.36	10.05	11.69	9.97	11.37	11.8
10.28	9.34	10.93	12.99	11.88	12.32				10.31	11.61	10.46	9.68	11.33	11.95			11.4	10.68	11.18	12.36	10.06	11.8	10.03	11.44	11.8
10.28	9.41	11.16	12.99	11.88	12.32				10.34	11.62	10.48	9.68	11.34	12.11			11.4	10.81	11.18	12.36	10.06	11.85	10.04	11.55	11.8
10.35	9.52	11.16	12.99	11.93	12.32				10.4	11.7	10.55	9.79	11.41	12.12			11.59	10.81	11.3	12.46	10.06	12.1	10.2	11.66	11.81
10.35	9.65	11.17	12.99	12	12.44				10.4	11.8	10.59	9.79	11.44	12.12			11.6	10.88	11.45	12.54	10.12	12.1	10.2	11.78	11.81
10.54	9.76	11.17	12.99	12.02	12.44				10.49	11.81	10.59	9.87	11.44	12.12			11.64	10.98	11.55	12.57	10.18	12.18	10.31	11.91	11.81
10.83	9.77	11.27	12.99	12.17	12.51				10.59	11.95	10.59	9.88	11.61	12.29			11.74	10.98	11.55	12.66	10.28	12.18	10.31	12.03	11.92
10.83	9.77	11.27	14.29	12.21	12.55				10.72	12.1	10.59	9.96	11.68	12.29			11.81	11.07	11.64	12.77	10.41	12.18	10.48	12.04	11.92
10.87	9.85	11.35	14.29	12.32	12.79				10.8	12.1	10.59	10.02	11.69	12.42			11.81	11.07	11.71	12.96	10.47	12.23	10.49	12.08	11.92
10.93	9.92	11.43	14.29	12.36	12.79				10.8	12.11	10.59	10.02	11.77	12.42			11.87	11.33	11.84	13.09	10.59	12.43	10.49	12.18	12.18
10.94	9.92	11.51	14.29	12.47	12.79				10.8	12.11	10.59	10.02	11.89	12.43			11.87	11.33	12	13.09	10.59	12.66	10.49	12.19	12.27
11.01	9.92	11.52	14.62	12.47	12.8				10.88	12.2	10.59	10.12	11.9	12.58			11.87	11.45	12.01	13.19	10.7	12.66	10.49	12.2	12.27
11.1	10.09	11.63	14.62	12.47	12.9				10.88	12.36	10.59	10.18	11.9	12.58			11.87	11.62	12.01	13.27	10.7	12.77	10.75	12.34	12.27
11.1	10.1	11.78	14.62	12.81	13.01				10.88	12.37	11.15	10.25	11.9	12.72			11.87	11.63	12.01	13.27	10.71	12.83	10.84	12.36	12.43
11.1	10.1	11.84	14.62	12.81	13.01				10.88	12.64	11.16	10.25	11.9	12.72			12.03	11.63	12.02	13.54	10.71	12.83	10.84	12.37	12.43
11.18	10.1	11.84	14.62	12.81	13.09				10.96	12.69	11.16	10.26	12.02	12.72			12.04	11.64	12.14	13.58	10.85	13.13	10.84	12.37	12.43
11.18	10.1	11.84	14.62	12.92	13.17				11.06	12.7	11.16	10.26	12.03	12.86			12.04	11.64	12.26	13.58	10.85	13.28	10.84	12.42	12.43
11.19	10.1	11.84	14.62	12.92	13.17				11.06	12.71	11.22	10.26	12.12	12.86			12.2	11.64	12.27	13.59	10.93	13.28	10.85	12.49	12.43
11.2	10.1	11.84	14.62	12.92	13.36				11.24	12.77	11.24	10.27	12.21	12.86			12.21	11.64	12.27	13.59	10.93	13.28	10.96	12.53	12.43
11.2	10.1	11.85	14.62	12.92	13.36				11.26	12.78	11.24	10.27	12.22	12.86			12.21	11.78	12.46	13.59	10.93	13.28	10.97	12.59	12.61
11.27	10.16	11.85	14.62	12.92	13.36				11.26	12.78	11.25	10.37	12.22	12.87			12.21	11.78	12.46	13.7	10.93	13.28	11.12	12.62	12.61
11.27	10.17	11.98	14.62	12.92	13.42				11.27	12.88	11.36	10.38	12.26	12.87			12.21	11.79	12.47	13.7	10.94	13.28	11.12	12.76	12.67
11.28	10.3	11.99	14.62	12.94	13.42				11.29	12.89	11.44	10.38	12.36	12.87			12.23	11.9	12.47	13.7	10.94	13.28	11.12	12.76	12.67
11.4	10.3	12.03	14.62	13.16	13.42				11.3	12.89	11.45	10.39	12.36	12.87			12.23	11.9	12.58	13.71	10.94	13.28	11.12	12.76	12.67
11.4	10.3	12.1	14.62	13.27	13.45				11.3	12.9	11.45	10.39	12.36	12.97			12.35	11.99	12.59	13.71	10.94	13.28	11.12	12.76	12.67
11.41	10.3	12.1	14.62	13.27	13.6				11.3	12.95	11.45	10.53	12.36	12.97			12.54	11.99	12.59	13.71	11.15	13.28	11.31	12.79	12.78
11.42	10.3	12.1	14.62	13.27	13.6				11.31	12.96	11.45	10.54	12.36	12.98			12.55	12	12.6	13.82	11.15	13.28	11.31	12.79	12.78
11.48	10.3	12.11	14.62	13.27	13.65				11.31	13.11	11.45	10.54	12.37	12.98			12.55	12	12.6	13.82	11.15	13.28	11.33	13.04	12.78

1	1	2	2	5	6	6	8	9	9	2	2	5	8	6	6	9	9	11	11
Box-8	Box-2	Box-4	Box-7	Box-3	Box-1	Box-4	Box-7	Box-3	Box-8	Box-1	Box-5	Box-2	Box-6	Box-5	Box-7	Box-4	Box-5	Box-3	Box-7
2.2	5.5	19.1	25.1	34.2	38	43	52.2	64	75	13.7	20.2	32.3	44.5	45	47	65	68	78	84
M	M	M	M	M	M	M	M	M	M	M	M	M	M	M	M	M	M	M	M
Malto	Malto	Malto	Malto	MD	MD	MD	MD	MD	Malto	MCT	MCT	MCT	MCT	MCT	MCT	MCT	MCT	MCT	MCT
MM	MM	MM	MM	MM	MM	MM	MM	MM	MM	MT	MT	MT	MT	MT	MT	MT	MT	MT	MT
0	0	0	0	0	0	0	0	0	0	0	0	0	0	0	0	0	0	0	0
0.02	0.01	0	0.2	0	0.1	0.02	0	0	0.01	0	0	0	0	0	0.04	0	0	0.01	0.06
0.09	0.01	0	0.22	0	0.1	0.06	0	0	0.01	0	0	0	0	0	0.23	0	0	0.01	0.2
0.1	0.03	0	0.42	0.32	0.1	0.33	0	0	0.01	0	0	0	0.02	0.22	0.23	0	0	0.02	0.24
0.16	0.04	0	0.48	0.61	0.36	0.37	0	0	0.01	0	0	0	0.02	0.44	0.32	0	0	0.02	0.25
0.27	0.04	0.03	0.86	0.86	0.36	0.57	0	0	0.01	0	0	0	0.02	0.59	0.45	0	0	0.02	0.25
0.29	0.17	0.04	0.93	0.98	0.41	0.78	0	0	0.02	0	0	0	0.02	0.59	0.55	0	0	0.02	0.32
0.46	0.36	0.04	1.21	1.26	0.54	0.81	0	0	0.02	0	0.06	0	0.03	0.81	0.78	0	0	0.03	0.32
0.69	0.69	0.05	1.51	1.32	0.54	0.99	0	0	0.02	0.28	0.31	0	0.03	0.81	1.1	0	0	0.03	0.32
0.77	0.95	0.15	1.51	1.62	0.8	1.01	0.42	0	0.02	0.61	0.92	0	0.03	1.02	1.25	0	0	0.03	0.42
1.01	1.24	0.24	1.51	1.94	0.9	1.12	0.66	0	0.02	0.91	1.09	0	0.03	1.21	1.42	0	0	0.03	0.42
1.11	1.42	0.37	1.52	2.22	0.9	1.37	0.93	0	0.02	1.12	1.26	0	0.03	1.31	1.53	0.42	0	0.44	0.55
1.28	1.67	0.55	1.71	2.36	1.07	1.37	1.06	0	0.02	1.59	1.79	0	0.05	1.39	1.58	0.58	0	0.68	0.55
1.28	1.93	0.55	1.74	2.41	1.08	1.76	1.46	0	0.02	1.81	1.94	0	0.05	1.45	1.74	0.83	0	1.19	0.55
1.3	2.04	0.57	1.8	2.64	1.45	1.89	1.59	0	0.02	1.9	2.27	0	0.05	1.45	1.83	0.92	0	1.36	0.55
1.46	2.22	0.78	1.8	2.71	1.66	2.06	1.75	0	0.03	2.43	2.39	0	0.5	1.45	1.83	1.14	0	1.42	0.55
1.47	2.46	0.78	2.1	2.72	1.78	2.17	1.84	0	0.03	2.69	2.39	0	0.95	1.53	1.84	1.43	0	1.88	0.55
1.48	2.6	1.16	2.1	2.74	1.88	2.33	2.19	0	0.05	3.06	2.39	0	1.32	1.61	2.03	1.66	0	2.3	0.55
1.72	2.86	1.38	2.19	2.78	1.95	2.6	2.33	0	0.69	3.11	2.39	0	1.47	1.87	2.18	1.87	0.09	2.47	1.13
2.03	3.04	1.38	2.25	2.78	2.11	2.87	2.44	0	0.9	3.23	2.45	0.01	1.69	1.9	2.35	1.92	0.67	2.71	1.32
2.13	3.04	1.4	2.25	3.02	2.12	3.06	2.54	0	1.01	3.23	2.45	0.02	2.18	1.91	2.36	2.02	0.82	2.88	1.32
2.21	3.04	1.46	2.25	3.09	2.12	3.16	2.54	0	1.01	3.54	2.45	0.03	2.4	1.91	2.37	2.02	1	2.94	1.32
2.21	3.04	1.59	2.25	3.26	2.18	3.21	2.55	0	1.05	3.54	2.64	0.04	2.55	1.91	2.51	2.03	1.05	3.02	1.32
2.41	3.04	1.62	2.38	3.33	2.34	3.43	2.56	0	1.05	3.54	2.65	0.05	2.66	1.92	2.52	2.12	1.05	3.02	1.32
2.42	3.17	1.62	2.49	3.33	2.34	3.43	2.56	0	1.28	3.54	2.69	0.06	2.66	1.92	2.52	2.12	1.05	3.02	1.35
2.43	3.32	1.62	3.02	3.33	2.4	3.43	2.73	0	1.66	3.54	3.12	0.07	2.66	2.26	2.56	2.13	1.05	3.02	1.35
2.44	3.32	1.8	3.1	3.33	2.59	3.54	2.74	0	1.67	3.82	3.28	0.09	2.66	2.33	2.56	2.13	1.05	3.15	1.47
2.45	3.35	1.81	3.1	3.33	2.61	3.54	2.74	0	1.68	4.02	3.28	0.09	2.66	2.33	2.57	2.13	1.05	3.26	1.47
2.46	3.41	1.86	3.1	3.44	2.61	3.55	2.74	0	1.68	4.02	3.28	0.11	2.99	2.34	2.83	2.47	1.05	3.66	1.47
2.47	3.46	1.92	3.1	3.59	2.61	3.73	2.75	0	1.7	4.11	3.28	0.11	3.06	2.34	2.89	2.47	1.05	3.9	1.47
2.48	3.66	1.92	3.1	3.59	2.81	3.91	2.75	0	1.88	4.22	3.28	0.13	3.28	2.34	2.89	2.47	1.05	3.99	1.47
2.7	3.7	1.92	3.11	3.59	2.86	4.03	3.28	0	1.88	4.33	3.35	0.14	3.45	2.34	2.9	2.47	1.05	4.02	1.47
2.71	3.7	1.92	3.12	3.71	2.86	4.19	3.34	0	1.89	4.47	3.41	0.15	3.47	2.43	2.91	2.47	1.05	4.25	1.47
2.71	3.73	1.92	3.12	3.83	3.27	4.39	3.35	0	2.12	4.58	3.41	0.16	3.47	2.72	3.19	2.78	1.05	4.35	1.47
2.73	3.83	1.92	3.12	3.87	3.38	4.4	3.35	0	2.26	4.58	3.42	0.17	3.57	2.73	3.33	3.14	1.05	4.38	1.47
2.74	4.21	2.27	3.12	3.87	3.38	4.42	3.36	0	2.31	4.58	3.42	0.18	3.61	2.73	3.33	3.14	1.05	4.53	1.47
3.06	4.21	2.27	3.64	3.87	3.38	4.55	3.48	0	2.37	4.68	3.59	0.19	3.61	2.73	3.33	3.15	1.05	4.53	1.91
3.06	4.26	2.27	3.64	3.87	3.38	4.61	3.63	0	2.37	4.68	4.01	0.2	3.64	2.84	3.33	3.15	1.52	4.53	1.99
3.06	4.26	2.88	3.64	4.22	3.38	4.61	3.64	0	2.4	4.75	4.36	0.21	3.65	3.13	3.34	3.15	1.52	4.53	2.03
3.07	4.32	2.95	3.65	4.25	3.66	4.66	3.64	0	2.93	4.75	4.49	0.22	3.72	3.13	3.39	3.15	1.52	4.53	2.03
3.07	4.33	2.97	3.67	4.25	3.78	4.66	3.65	0.16	3.01	4.75	4.55	0.23	3.72	3.13	3.39	3.15	1.52	4.73	2.03
3.08	4.33	2.97	3.83	4.25	3.79	4.66	3.71	0.18	3.01	4.75	4.56	0.24	3.72	3.13	3.4	3.56	1.52	4.94	2.03
3.09	4.33	2.97	3.92	4.25	3.79	4.68	3.82	0.18	3.01	4.75	4.56	0.25	3.72	3.14	3.41	3.61	1.52	4.94	2.03
3.09	4.56	2.98	3.92	4.25	3.79	4.87	3.83	0.18	3.01	4.75	4.56	0.26	3.9	3.14	3.41	3.61	1.64	4.94	2.03
3.1	4.67	3.25	3.92	4.25	3.79	4.87	3.83	0.18	3.05	4.82	4.61	0.27	3.91	3.14	3.41	3.61	1.64	4.94	2.03
3.1	4.67	3.25	3.92	4.25	3.87	4.87	3.83	0.18	3.07	4.82	4.61	0.28	3.91	3.14	3.41	3.62	1.64	4.94	2.03
3.1	4.67	3.25	3.93	4.26	3.95	4.87	3.84	0.18	3.21	4.82	4.65	0.29	3.91	3.41	3.42	3.62	1.64	4.94	2.03
3.11	4.67	3.25	3.93	4.4	3.95	4.87	4	0.22	3.21	4.92	4.65	0.3	3.91	3.41	3.53	3.62	1.64	4.99	2.03
3.11	4.67	3.25	4.07	4.4	3.95	4.87	4	0.23	3.21	4.92	4.67	0.31	3.91	3.41	3.53	3.72	1.64	4.99	2.77
3.12	4.67	3.45	4.07	4.4	3.95	4.87	4.01	0.23	3.23	4.92	4.67	0.32	3.91	3.42	3.53	3.9	1.64	4.99	3.57
3.12	4.88	3.73	4.07	4.4	3.95	4.87	4.02	0.23	3.36	4.92	4.84	0.33	3.91	3.42	3.54	3.9	1.64	4.99	3.57
3.13	4.88	3.73	4.08	4.42	3.95	4.98	4.23	0.23	3.37	4.92	4.95	0.34	3.91	3.42	3.78	3.9	1.64	5.1	3.57
3.14	4.88	3.73	4.52	4.45	3.95	4.98	4.23	0.24	3.41	4.92	5.02	0.34	3.91	3.42	3.78	3.91	1.64	5.1	3.57
3.16	4.88	3.73	4.54	4.46	3.95	4.98	4.23	0.24	3.43	4.92	5.02	0.35	3.91	3.43	3.78	4.01	2.17	5.1	3.57
3.16	4.88	3.73	4.65	4.47	3.95	4.98	4.24	0.24	3.46	4.92	5.02	0.36	3.91	3.43	3.78	4.02	2.17	5.11	3.75
3.16	4.88	3.73	4.66	4.48	3.95	4.98	4.24	0.25	3.75	4.92	5.04	0.36	3.91	3.48	3.78	4.02	2.18	5.11	3.81
3.16	4.88	3.88	4.67	4.61	4.15	4.99	4.38	0.25	3.76	4.92	5.34	0.37	3.91	3.48	3.95	4.02	2.18	5.12	3.85
3.17	4.89	4.05	4.67	4.61	4.17	5.03	4.56	0.25	3.76	4.92	5.34	0.38	3.91	3.48	3.95	4.02	2.19	5.12	3.85
3.17	4.97	4.07	4.68	4.61	4.17	5.03	4.57	0.26	3.76	4.92	5.37	0.38	3.91	3.48	3.96	4.08	2.33	5.13	3.85
3.18	4.97	4.08	4.68	4.61	4.22	5.03	4.57	0.39	3.76	5.07	5.37	0.39	3.91	3.69	4.1	4.19	2.33	5.13	3.85
3.18	4.97	4.2	4.77	4.62	4.22	5.03	4.58	0.46	3.76	5.44	5.37	0.39	3.93	3.69	4.2	4.19	2.33	5.13	3.85
3.22	4.97	4.36	5.05	4.63	4.22	5.23	4.58	0.47	3.81	5.44	5.37	0.4	3.96	3.69	4.34	4.2	2.33	5.13	3.85
3.22	5.02	4.37	5.05	4.77	4.22	5.23	4.7	0.48	3.83	5.51	5.54	0.4	3.96	3.69	4.52	4.55	2.33	5.14	3.85
3.22	5.02	4.55	5.05	4.77	4.27	5.23	4.86	0.48	3.85	5.51	5.54	0.41	3.96	3.75	4.52	4.55	2.33	5.15	3.85
3.22	5.02	4.57	5.07	4.78	4.33	5.23	4.87	0.49	4.03	5.51	5.54	0.41	4.04	3.86	4.53	4.56	2.33	5.17	3.85
3.23	5.02	4.57	5.33	4.79	4.33	5.23	4.87	0.49	4.09	5.51	5.54	0.42	4.07	3.86	4.83	4.56	2.33	5.17	3.85
3.23	5.03	4.57	5.34	4.9	4.52	5.23	4.88	0.49	4.09	5.51	5.74	0.42	4.08	3.91	4.83	4.56	2.33	5.17	3.85
3.54	5.26	4.81	5.35	5.08	4.52	5.4	4.88	0.5	4.11	5.51	5.74	0.42	4.31	3.92	4.93	4.64	2.33	5.18	3.85
3.54	5.26	4.86	5.36	5.14	4.65	5.57	4.89	0.52	4.12	5.51	6.13	0.43	4.52	4.15	4.93	4.86	2.33	5.18	3.85
3.55	5.47	4.97	5.37	5.38	4.9	5.74	5.21	0.69	4.42	5.51	6.21	0.43	4.73	4.15	5.18	5.07	2.33	5.18	3.85
3.69	5.47	5.24	5.37	5.5	5.01	5.83	5.21	0.7	4.43	5.69	6.21	0.44	4.82	4.5	5.29	5.12	2.33	5.22	3.85
3.7	5.48	6.63	5.63	5.56	5.01	5.9	5.25	0.79	4.46	5.9	6.58	0.44	4.88	4.59	5.37	5.22	2.33	5.22	3.85
3.74	5.58	6.8	5.63	5.72	5.01	6.18	5.25	0.79	4.53	6.07	6.72	0.44	5.06	4.59	5.69	5.3	2.34	5.22	3.85
3.78	5.61	6.96	5.87	5.98	5.34	6.21	5.54	0.98	4.57	6.39	6.95	0.45	5.06	4.59	5.85	5.3	2.34	5.22	3.85
4.31	6.02	7.13	6.12	6.02	5.34	6.29	5.83	0.99	4.82	6.4	7.14	0.45	5.06	4.95	5.94	5.46	2.34	5.37	3.85
4.49	6.05	7.5	6.2	6.21	5.58	6.29	5.83	0.99	4.84	6.77	7.37	0.45	5.07	4.96	5.94	5.46	2.34	5.53	3.85
4.58	6.11	7.5	6.34	6.43	5.64	6.37	5.88	1.05	5.22	7.11	7.37	1.16	5.26	4.96	6.02	5.47	2.34	5.59	3.85
4.74	6.34	7.5	6.35	6.51	5.7	6.54	6.12	1.29	5.22	7.11	7.47	1.97	5.5	4.96	6.1	5.47	2.34	5.6	3.85
4.74	6.34	7.5	6.47	6.68	6.03	6.72	6.25	1.3	5.34	7.67	7.63	2.54	5.81	5.35	6.16	5.47	2.34	5.84	3.85
4.91	6.38	7.5	6.49	6.98	6.08	6.83	6.34	1.54	5.44	7.72	7.64	2.73	6.02	5.4	6.43	5.72	2.34	6.08	3.85
4.92	6.41	7.5	6.49	6.99	6.29	7.06	6.5	1.65	5.62	7.95	7.64	2.98	6.19	5.4	6.43	5.79	2.34	6.22	3.85
4.92	6.45	7.62	6.49	7.2	6.31	7.27	6.56	1.93	5.86	8.14	7.68	3.27	6.2	5.4	6.44	6.03	2.34	6.4	3.85
4.93	6.48	7.88	6.49	7.24	6.31	7.44	6.7	2	5.87	8.33	8.16	3.31	6.31	5.77	6.44	6.13	2.34	6.66	3.85
5.16	6.98	7.88	6.61	7.24	6.4	7.62	6.82	2	6.12	8.34	8.34	3.7	6.34	5.93	6.72	6.14	2.34	6.76	3.85
5.33	6.98	7.88	6.65	7.37	6.67	7.65	6.82	2	6.25	8.48	8.45	3.82	6.34	5.93	6.72	6.24	2.34	6.88	3.85
5.52	7.02	7.88	6.72	7.4	6.67	7.78	6.83	2	6.3	8.84	8.49	3.83	6.51	5.93	6.73	6.27	2.34	7.21	3.85
5.52	7.02	8.13	6.74	7.4	6.67	7.86	6.83	2.01	6.35	8.98	8.63	3.93	6.82	5.93	6.75	6.27	2.34	7.22	3.85
5.89	7.02	8.14	6.76	7.4	6.69	7.87	6.98	2.01	6.38	9.05	8.82	4.34	7.02	5.93	6.75	6.27	2.83	7.22	3.85
5.89	7.02	8.14	6.77	7.5	6.69	7.87	6.98	2.15	6.43	9.11	8.82	4.34	7.14	5.94	6.75	6.28	2.83	7.23	3.85
5.89	7.06	8.16	6.78	7.6	6.69	7.95	6.98	2.23	6.56	9.12	8.92	4.35	7.15	6.14	6.99	6.28	2.83	7.23	4.22
5.9	7.07	8.16	6.78	7.6	6.69	8.17	6.99	2.24	6.6	9.15	8.92	4.35	7.26	6.14	7.04	6.28	2.83	7.27	4.22
5.91	7.19	8.16	6.78	7.62	6.69	8.19	6.99	2.24	6.76	9.24	8.92	4.35	7.27	6.14	7.05	6.28	2.83	7.47	5.34
5.91	7.19	8.16	6.78	7.78	6.69	8.19	7.03	2.44	6.77	9.27	8.92	4.39	7.27	6.15	7.05	6.28	2.83	7.78	5.41
5.92	7.19	8.16	6.78	7.78	6.69	8.2	7.28	2.44	6.87	9.33	9.12	4.4	7.27	6.15	7.05	6.28	2.83	7.93	5.41
5.92	7.48	8.16	6.78	7.93	6.69	8.2	7.31	2.44	7.03	9.62	9.22	4.64	7.27	6.31	7.05	6.28	2.83	7.93	5.41
5.92	7.53	8.17	7.24	7.99	6.69	8.2	7.31	2.49	7.19	10.16	9.24	4.75	7.27	6.38	7.18	6.4	2.83	7.95	5.41
5.96	7.56	8.18	7.36	8.1	6.72	8.38	7.32	2.5	7.2	10.16	9.24	4.82	7.27	6.38	7.19	6.47	2.83	7.95	5.41
6.39	7.56	8.46	7.44	8.32	6.73	8.45	7.32	2.5	7.24	10.16	9.24	4.82	7.49	6.39	7.19	6.47	2.83	7.95	5.41
6.39	7.59	8.46	7.44	8.32	6.76	8.5	7.32	2.5	7.39	10.16	9.25	4.83	7.51	6.39	7.2	6.47	4.2	7.95	5.41
6.4	7.64	8.46	7.53	8.32	6.98	8.51	7.54	2.76	7.39	10.16	9.36	4.83	7.52	6.39	7.3	6.48	4.2	7.95	5.41
6.41	7.69	8.46	7.64	8.32	7.26	8.56	7.63	2.77	7.39	10.16	9.72	5.1	7.52	6.39	7.3	6.8	4.2	8.04	5.41
6.42	7.7	8.46	7.9	8.32	7.27	8.56	7.7	2.77	7.39	10.16	9.72	5.1	7.69	6.39	7.31	6.8	4.2	8.17	5.76
6.47	7.71	8.73	7.9	8.32	7.27	8.56	7.71	2.77	7.62	10.17	9.72	5.1	7.78	6.39	7.31	6.81	4.2	8.17	5.76
6.68	7.79	9.01	7.9	8.32	7.27	8.56	7.72	2.93	7.76	10.17	9.72	5.11	7.78	6.61	7.42	6.81	4.2	8.17	5.76
6.68	7.83	9.01	7.9	8.32	7.53	8.56	7.72	2.93	7.79	10.17	9.86	5.11	7.78	6.87	7.43	6.82	4.2	8.18	5.76
6.68	7.83	9.01	7.9	8.32	7.53	8.56	7.72	2.94	7.81	10.17	9.99	5.11	7.78	6.87	7.44	6.82	4.2	8.18	5.76
6.68	7.83	9.01	7.9	8.32	7.53	8.59	7.73	2.94	7.81	10.17	9.99	5.12	7.78	6.87	7.44	6.82	4.2	8.18	5.76
6.69	7.83	9.01	7.92	8.32	7.53	8.59	7.73	2.94	7.81	10.17	9.99	5.13	7.78	6.87	7.44	7.07	4.2	8.19	5.76
6.69	7.91	9.16	7.94	8.32	7.53	8.59	7.93	2.94	7.83	10.17	9.99	5.13	7.78	6.87	7.49	7.1	4.2	8.23	6.51
6.7	7.91	9.16	7.94	8.33	7.78	8.59	7.93	3.24	7.83	10.17	9.99	5.13	7.78	6.88	7.57	7.11	4.2	8.23	6.51
6.7	7.95	9.16	7.95	8.33	7.8	8.7	7.93	3.35	7.84	10.6	9.99	5.14	7.81	6.88	7.57	7.11	4.2	8.24	6.51
6.7	7.95	9.16	8.24	8.45	7.8	8.72	7.99	3.35	7.84	10.6	9.99	5.28	7.97	6.88	7.58	7.15	4.2	8.24	6.51
6.71	7.95	9.16	8.26	8.45	7.8	8.72	7.99	3.35	8.06	10.77	10.01	5.28	7.97	6.88	7.74	7.27	4.2	8.25	6.51
6.71	8.05	9.17	8.27	8.45	7.8	8.73	7.99	3.35	8.08	10.77	10.01	5.28	7.97	7.06	7.74	7.27	4.49	8.25	6.51
6.72	8.12	9.17	8.27	8.45			8	3.36	8.1	10.77	10.15	5.29	7.97			7.28	4.75	8.26	6.51
6.73	8.14	9.55	8.28	8.46			8	3.59	8.18	10.77	10.15	5.29	7.99			7.28	4.93	8.26	7.16
6.73	8.14	9.56	8.29	8.46			8	3.59	8.18	10.77	10.15	5.29	7.99			7.29	4.93	8.26	7.16
6.82	8.14	9.69	8.29				8.01	3.6	8.19	10.77	10.15	5.3				7.29	4.93	8.27	7.16
6.83	8.27	9.69	8.29				8.01	3.6	8.19	10.77	10.37	5.3				7.29	4.93	8.27	7.16
6.83	8.27	9.69	8.29				8.01	3.6	8.2	10.77	10.39	5.31				7.29	4.93	8.27	7.16
6.84	8.3	9.69	8.3				8.04	3.61	8.42	10.82	10.42	5.31				7.3	4.93	8.27	7.16
6.85	8.3	9.69	8.32				8.29	3.78	8.42	11.32	10.42	5.31				7.49	4.93	8.27	7.16
6.85	8.3	9.96	8.72				8.3	3.79	8.45	11.32	10.42	5.32				7.54	4.93	8.27	7.16
6.86	8.31	9.97	8.83				8.31	3.79	8.45	11.32	10.42	5.32				7.54	4.93	8.28	7.16
7.12	8.35	9.97	8.84				8.44	3.79	8.49	11.32	10.59	5.32				7.54	4.93	8.29	7.16

7.13	8.35	10.12	8.84	8.5	3.8	8.56	11.67	10.69	5.8	7.55	4.93	8.29	7.16
7.14	8.4	10.3	8.84	8.5	3.8	8.77	11.68	10.69	5.8	7.66	4.93	8.29	7.16
7.15	8.44	10.43	8.84	8.58	3.8	8.79	11.73	10.71	5.8	7.97	4.93	8.34	7.16
7.15	8.59	10.43	8.84	8.6	4	8.79	11.73	10.73	5.81	8.05	5.43	8.34	7.16
7.16	8.6	10.43	8.84	8.61	4	9.04	11.73	10.97	5.81	8.09	5.43	8.34	7.16
7.16	8.64	10.49	8.87	8.67	4.01	9.07	11.73	11.03	5.81	8.09	5.7	8.34	7.16
7.24	8.64	10.56	9.18	8.67	4.01	9.07	11.81	11.03	5.82	8.09	5.7	8.36	7.16
7.25	8.64	10.57	9.21	8.68	4.13	9.07	11.93	11.03	5.82	8.09	5.7	8.36	7.16
7.26	8.84	10.68	9.21	8.68	4.22	9.07	12.04	11.32	5.87	8.1	5.7	8.36	7.16
7.6	9.02	10.79	9.48	8.91	4.43	9.07	12.17	11.36	5.87	8.17	5.7	8.37	7.16
7.61	9.13	10.8	9.51	9.06	4.49	9.1	12.18	11.6	6.12	8.48	5.7	8.4	7.16
7.86	9.29	10.8	9.6	9.13	4.62	9.27	12.52	11.72	6.27	8.63	5.7	8.4	7.16
8.25	9.62	11.03	9.62	9.32	4.9	9.27	12.86	12.09	6.35	8.64	5.7	8.71	8.83
8.35	9.62	11.08	9.63	9.44	4.91	9.29	12.88	12.09	6.35	8.64	6.01	9.02	9.13
8.36	9.74	11.33	9.7	9.45	4.91	9.78	13.02	12.09	6.52	8.97	6.39	9.33	9.13
8.44	9.76	11.33	9.76	9.45	5.09	9.81	13.04	12.14	6.52	8.98	6.4	9.58	9.13
8.72	9.88	11.51	9.78	9.83	5.09	9.85	13.44	12.31	6.91	8.98	6.63	9.58	9.13
8.86	9.99	11.97	10.08	9.83	5.09	9.95	13.69	12.31	7	9.12	6.74	9.74	9.13
8.86	10.05	12	10.33	9.83	5.09	10.07	13.74	12.31	7.15	9.12	6.74	9.85	9.13
9.23	10.37	12	10.33	9.91	5.09	10.3	13.85	12.9	7.39	9.31	6.74	9.85	9.13
9.24	10.48	12	10.33	10.08	5.09	10.55	14.06	12.9	7.47	9.47	6.74	10.25	9.13
9.66	10.58	12.22	10.33	10.24	5.1	10.61	14.08	12.9	7.58	9.56	6.91	10.53	9.13
9.8	10.63	12.43	10.33	10.44	5.1	10.61	14.27	12.9	7.66	9.76	6.91	10.71	9.13
9.81	10.92	12.46	10.33	10.55	5.31	10.62	14.39	12.9	7.81	9.77	6.97	10.78	9.13
9.82	10.97	12.47	10.35	10.55	5.31	10.68	14.71	13.32	7.87	9.77	7.12	11.03	9.13
9.98	10.97	12.48	10.73	10.55	5.6	10.79	14.78	13.37	7.97	9.77	7.12	11.24	9.13
10.11	11	12.48	11	10.55	5.6	11.13	14.78	13.38	7.97	9.87	7.12	11.25	10.38
10.14	11	12.48	11.07	10.55	5.6	11.21	14.78	13.6	7.98	9.87	7.12	11.52	10.38
10.14	11	12.74	11.07	10.56	5.6	11.21	14.78	13.74	7.98	9.87	7.12	11.6	10.38
10.15	11.29	12.74	11.09	10.56	5.6	11.21	15.24	13.77	7.99	9.87	7.12	11.61	10.38
10.15	11.49	12.74	11.1	10.56	5.6	11.21	15.48	13.8	8.08	9.87	7.12	11.61	10.38
10.4	11.55	12.74	11.1	10.73	5.6	11.21	15.6	13.97	8.11	10.18	7.23	11.61	10.38
10.4	11.58	12.74	11.11	10.73	5.6	11.24	15.66	14.02	8.11	10.24	7.43	11.61	10.38
10.41	11.63	12.74	11.23	10.82	5.6	11.24	15.75	14.05	8.12	10.24	7.43	11.61	10.38
10.47	11.65	12.74	11.62	10.82	5.61	11.24	16.03	14.08	8.12	10.34	7.43	11.84	10.38
10.47	11.65	12.74	11.78	11.09	5.61	11.36	16.06	14.17	8.12	10.5	7.43	11.93	10.38
10.47	11.65	12.74	11.79	11.1	5.61	11.38	16.14	14.17	8.12	10.64	7.43	11.94	10.38
10.47	11.65	12.74	11.82	11.1	5.61	11.45	16.17	14.17	8.35	10.64	7.43	11.94	10.38
10.47	11.65	12.74	11.82	11.1	5.96	11.45	16.22	14.35	8.36	10.65	7.43	11.94	10.58
10.5	11.88	12.78	11.94	11.11	5.97	11.59	16.35	14.35	8.36	10.65	7.43	11.94	10.58
10.5	11.99	12.96	11.99	11.18	5.97	11.59	16.6	14.35	8.36	10.65	7.43	12.29	10.58
10.51	11.99	12.96	11.99	11.39	5.97	11.85	16.62	14.45	8.37	10.65	7.43	12.32	10.58
10.51	12.04	12.96	11.99	11.39	5.97	11.88	16.62	14.52	8.57	10.79	7.43	12.32	10.58
10.52	12.04	13.06	11.99	11.57	5.97	11.89	16.64	14.54	8.67	10.8	7.43	12.33	10.58
10.53	12.04	13.06	11.99	11.57	6.1	11.9	16.65	14.56	8.67	10.8	8.21	12.33	10.58
10.53	12.04	13.06	12	11.58	6.1	11.91	16.68	14.57	8.67	10.8	8.21	12.33	10.58
10.53	12.06	13.06	12.01	11.58	6.11	11.93	16.68	14.69	8.68	10.8	8.26	12.63	10.58
10.54	12.1	13.06	12.02	11.59	6.11	11.96	16.68	14.69	8.68	10.97	8.27	12.66	10.58
10.54	12.12	13.06	12.02	11.7	6.11	11.98	16.68	14.79	8.68	10.97	8.27	12.66	10.58
10.54	12.17	13.06	12.02	11.71	6.23	11.98	16.69	14.9	8.75	10.98	8.29	12.66	10.58
10.55	12.25	13.23	12.02	11.82	6.24	11.98	16.74	14.9	8.84	10.98	8.29	12.66	10.58
10.55	12.26	13.23	12.02	11.83	6.24	11.98	16.74	14.91	8.84	10.98	8.29	12.66	10.58
10.56	12.26	13.27	12.03	11.83	6.24	12.05	16.74	15.01	8.85	10.99	8.31	12.66	10.58
10.56	12.26	13.27	12.04	11.84	6.25	12.07	16.74	15.01	8.94	11.16	8.31	13.08	10.58
10.57	12.26	13.27	12.04	11.84	6.38	12.07	16.8	15.01	9	11.17	8.32	13.08	10.58

0	0	0	0	0	0	0	0	0	0	0	0	0	0	0	0	0	0	0.52	0.12
0.35	0.05	0	0.01	0.01	0	0.05	0	0.01	0.26	0	0.08	0	0.02	0	0	0.04	0	0.64	0.32
0.36	0.06	0	0.06	0.11	0.08	0.21	0	0.01	0.32	0	0.21	0.11	0.04	0.12	0.03	0.08	0	0.64	0.32
0.36	0.09	0.01	0.06	0.11	0.32	0.24	0	0.01	0.34	0	0.4	0.17	0.07	0.13	0.1	0.09	0	0.7	0.32
0.53	0.09	0.01	0.26	0.11	0.36	0.27	0	0.14	0.42	0	0.4	0.17	0.1	0.13	0.11	0.18	0	0.71	0.32
0.54	0.09	0.01	0.27	0.2	0.37	0.38	0	0.14	0.48	0	0.4	0.27	0.2	0.16	0.16	0.32	0	0.72	0.32
0.65	0.09	0.04	0.31	0.21	0.37	0.39	0	0.15	0.6	0	0.4	0.27	0.26	0.26	0.18	0.34	0	0.85	0.32
0.66	0.14	0.11	0.31	0.22	0.37	0.44	0	0.36	0.62	0	0.4	0.27	0.26	0.26	0.18	0.35	0	0.91	0.33
0.66	0.22	0.11	0.36	0.22	0.37	0.52	0	0.36	0.62	0	0.4	0.43	0.28	0.26	0.25	0.47	0	0.91	0.48
0.67	0.33	0.11	0.43	0.33	0.37	0.52	0	0.44	0.74	0	0.4	0.43	0.28	0.28	0.25	0.5	0	0.91	0.48
0.75	0.36	0.11	0.55	0.34	0.37	0.52	0	0.44	0.77	0	0.4	0.44	0.28	0.31	0.27	0.52	0	1.05	0.48
0.75	0.36	0.11	0.57	0.39	0.37	0.52	0.7	0.53	0.84	1.36	0.4	0.44	0.34	0.31	0.32	0.52	0	1.06	0.48
0.76	0.36	0.11	0.57	0.43	0.37	0.6	0.7	0.64	0.86	1.37	0.46	0.44	0.38	0.31	0.36	0.52	0	1.07	0.67
0.94	0.37	0.13	0.6	0.44	0.44	0.65	0.84	0.64	0.86	1.42	0.52	0.44	0.55	0.38	0.38	0.52	0	1.12	0.67
0.94	0.42	0.16	0.79	0.56	0.48	0.67	0.84	0.64	0.87	1.42	0.76	0.45	0.56	0.38	0.39	0.53	0	1.12	0.75
1.01	0.82	0.16	0.8	0.57	0.48	0.74	0.84	0.65	0.97	1.46	0.76	0.47	0.71	0.5	0.39	0.53	0	1.21	0.77
1.07	0.82	0.16	0.81	0.73	0.78	0.8	0.84	0.74	0.97	1.61	0.76	0.51	0.71	0.67	0.39	0.54	0	1.21	0.77
1.08	0.96	0.16	0.96	0.74	0.78	0.85	1.06	0.75	1.09	1.61	0.88	0.56	0.8	0.7	0.39	0.55	0	1.22	0.77
1.23	0.96	0.18	1.01	0.92	0.78	0.86	1.06	0.75	1.1	1.91	0.88	0.7	0.83	0.75	0.39	0.74	0	1.22	0.78
1.24	1.03	0.51	1.01	1.07	0.92	0.95	1.06	0.94	1.1	1.98	1.28	0.7	0.83	0.75	0.39	0.85	0	1.22	0.8
1.24	1.03	0.56	1.03	1.08	0.92	1	1.28	0.94	1.15	1.99	1.28	0.71	0.83	0.95	0.39	0.98	0	1.22	0.8
1.45	1.08	0.56	1.18	1.24	0.94	1	1.28	0.94	1.21	2.32	1.38	0.71	0.98	0.99	0.39	1.05	1.53	1.29	0.91
1.53	1.19	0.56	1.18	1.3	1.03	1	1.39	0.95	1.24	2.38	1.41	0.85	1.08	0.99	0.39	1.09	1.53	1.49	0.91
1.54	1.31	0.56	1.21	1.5	1.34	1	1.39	1.2	1.41	2.38	1.59	1.09	1.21	0.99	0.39	1.1	1.53	1.57	1.1
1.65	1.35	0.56	1.28	1.5	1.34	1.05	1.41	1.3	1.42	2.44	1.6	1.13	1.21	1.2	0.39	1.11	1.53	1.57	1.26
1.77	1.35	0.75	1.28	1.5	1.35	1.14	1.54	1.3	1.56	2.45	1.74	1.21	1.39	1.2	0.39	1.12	1.53	1.62	1.26
1.77	1.57	0.75	1.33	1.56	1.35	1.22	1.62	1.49	1.73	2.49	1.74	1.21	1.39	1.23	0.39	1.12	1.95	1.65	1.26
1.78	1.57	0.82	1.4	1.57	1.62	1.36	1.62	1.5	1.82	2.49	1.74	1.37	1.39	1.36	0.39	1.27	1.95	1.78	1.26
1.81	1.57	0.82	1.5	1.67	1.64	1.43	1.7	1.5	1.87	2.53	1.83	1.52	1.39	1.36	0.39	1.73	2.07	1.79	1.52
1.96	1.57	0.99	1.61	1.67	1.72	1.43	1.88	1.55	1.89	2.6	1.91	1.52	1.41	1.36	0.39	1.86	2.18	1.79	1.52
1.99	1.57	0.99	1.66	1.73	1.72	1.52	1.9	1.67	1.97	2.69	1.98	1.52	1.41	1.36	0.39	1.86	2.19	1.84	1.52
1.99	1.57	0.99	1.7	1.73	1.75	1.52	1.9	1.67	1.97	2.7	2.05	1.57	1.46	1.44	0.39	1.86	2.19	1.89	1.68
2.06	1.97	0.99	1.73	1.8	1.82	1.52	1.95	1.81	1.98	2.7	2.09	1.58	1.51	1.45	0.39	1.87	2.19	2.18	1.78
2.06	2.02	0.99	1.81	1.8	1.85	1.67	1.95	1.81	1.98	2.7	2.29	1.63	1.51	1.45	0.39	1.87	2.19	2.29	2.03
2.15	2.02	1.19	1.83	1.89	2.04	1.76	2.22	1.82	2	2.7	2.29	1.75	1.57	1.45	0.39	1.87	2.19	2.32	2.03
2.15	2.09	1.19	1.91	1.89	2.04	1.76	2.22	1.95	2.02	2.75	2.5	1.75	1.65	1.58	0.39	2.03	2.48	2.32	2.03
2.16	2.09	1.19	1.91	2.02	2.04	1.76	2.26	1.95	2.06	2.8	2.5	1.75	1.66	1.89	0.39	2.06	2.5	2.34	2.03
2.2	2.09	1.19	2.01	2.08	2.28	1.94	2.37	1.95	2.08	2.83	2.5	2.02	1.7	1.89	0.39	2.06	2.5	2.48	2.03
2.31	2.09	1.37	2.02	2.09	2.34	1.94	2.47	1.95	2.21	2.83	2.58	2.02	1.71	1.89	0.39	2.06	2.68	2.48	2.03
2.42	2.11	1.37	2.05	2.09	2.34	1.94	2.51	2.09	2.21	2.84	2.79	2.02	1.71	1.89	0.39	2.07	2.68	2.49	2.2
2.42	2.11	1.37	2.19	2.24	2.37	2.26	2.56	2.09	2.32	3.13	2.8	2.02	1.83	1.89	0.39	2.17	2.68	2.54	2.36
2.67	2.11	1.4	2.2	2.24	2.37	2.26	2.59	2.22	2.36	3.35	2.9	2.03	1.84	1.97	0.39	2.23	2.68	2.57	2.4
2.78	2.11	1.59	2.2	2.48	2.37	2.41	2.6	2.22	2.39	3.43	2.96	2.03	1.91	2.09	0.39	2.39	2.68	2.58	2.4
2.79	2.11	1.6	2.3	2.48	2.37	2.43	2.84	2.3	2.56	3.44	2.97	2.08	1.91	2.09	0.39	2.4	2.76	2.78	2.4
2.85	2.11	1.6	2.38	2.62	2.37	2.58	2.9	2.31	2.6	3.61	3.03	2.08	2.14	2.09	0.39	2.4	2.76	2.78	2.56
2.85	2.11	1.61	2.38	2.62	2.37	2.58	2.9	2.31	2.67	3.75	3.07	2.18	2.24	2.2	0.39	2.4	2.76	2.9	2.71
2.86	2.11	1.62	2.38	2.63	2.37	2.58	2.91	2.4	2.73	3.8	3.27	2.18	2.25	2.25	0.39	2.4	2.91	3.06	2.72
2.86	2.11	1.62	2.39	2.63	2.44	2.61	2.91	2.4	2.81	3.81	3.27	2.28	2.25	2.25	0.39	2.66	2.91	3.09	2.72
2.98	2.12	1.62	2.41	2.63	2.45	2.65	3.15	2.4	2.81	3.92	3.27	2.28	2.25	2.41	0.39	2.66	2.91	3.09	2.73
2.98	2.12	1.62	2.58	2.71	2.55	2.65	3.16	2.41	2.94	3.97	3.49	2.28	2.35	2.41	0.39	2.66	2.91	3.15	2.73
3.05	2.32	1.62	2.58	2.72	2.61	2.77	3.16	2.41	2.97	4.23	3.52	2.29	2.35	2.41	0.39	2.76	2.91	3.16	2.77
3.17	2.37	1.62	2.77	2.81	2.63	2.91	3.16	2.41	2.99	4.36	3.52	2.29	2.35	2.42	0.39	2.76	2.91	3.16	2.77
3.18	2.37	1.93	2.77	2.81	2.64	2.91	3.17	2.41	3.01	4.4	3.61	2.3	2.35	2.43	0.39	2.76	2.91	3.16	2.77
3.25	2.38	2.06	2.8	2.82	2.64	2.92	3.29	2.42	3.07	4.46	3.61	2.3	2.39	2.59	0.39	2.86	2.91	3.24	2.77
3.25	2.47	2.06	2.83	2.82	2.84	2.92	3.29	2.42	3.11	4.46	3.61	2.31	2.4	2.6	0.39	2.86	2.91	3.24	3.09
3.26	2.47	2.06	2.95	2.88	2.85	3.05	3.3	2.42	3.12	4.53	3.64	2.32	2.4	2.6	0.39	2.93	2.91	3.24	3.09
3.35	2.47	2.06	2.96	2.89	2.85	3.13	3.3	2.43	3.23	4.53	3.64	2.59	2.41	2.6	0.39	2.94	2.91	3.24	3.09
3.36	2.47	2.06	2.96	2.9	2.85	3.19	3.31	2.43	3.28	4.68	3.64	2.59	2.41	2.6	0.39	2.94	2.91	3.24	3.1
3.45	2.5	2.06	2.97	3.05	2.85	3.19	3.35	2.44	3.31	4.73	3.64	2.59	2.41	2.66	0.39	2.94	2.91	3.34	3.1
3.46	2.5	2.06	3.04	3.11	2.85	3.37	3.48	2.54	3.35	4.8	3.66	2.6	2.41	2.7	0.39	2.95	2.91	3.35	3.22
3.46	2.5	2.06	3.07	3.12	2.85	3.37	3.51	2.54	3.46	4.8	3.68	2.6	2.41	2.7	0.39	3.05	2.91	3.45	3.22
3.46	2.5	2.13	3.1	3.12	2.85	3.37	3.51	2.54	3.51	4.9	3.73	2.61	2.41	2.89	0.39	3.06	2.91	3.45	3.22
3.54	2.5	2.16	3.11	3.13	2.85	3.37	3.52	2.55	3.52	4.9	3.73	2.61	2.42	2.89	0.39	3.06	2.91	3.45	3.22
3.54	2.5	2.17	3.13	3.13	2.85	3.4	3.52	2.55	3.58	4.92	3.8	2.61	2.42	2.89	0.39	3.06	2.91	3.45	3.23
3.59	2.5	2.17	3.19	3.14	2.85	3.4	3.53	2.67	3.58	4.92	3.87	2.62	2.42	2.89	0.39	3.07	2.91	3.45	3.23
3.64	2.5	2.18	3.19	3.14	2.85	3.4	3.67	2.68	3.58	4.97	3.91	2.62	2.44	2.89	0.39	3.07	2.91	3.45	3.23
3.64	2.6	2.41	3.19	3.16	3.03	3.4	3.67	2.68	3.59	5.06	4.02	2.63	2.44	2.89	0.39	3.07	2.91	3.45	3.25
3.64	2.6	2.41	3.25	3.16	3.03	3.45	3.68	2.69	3.59	5.12	4.02	2.68	2.55	2.91	0.39	3.18	2.91	3.46	3.55
3.65	2.6	2.46	3.27	3.16	3.03	3.55	3.68	2.69	3.59	5.12	4.09	2.76	2.55	3.04	0.39	3.18	2.91	3.61	3.55
3.66	2.6	2.46	3.27	3.22	3.07	3.76	3.79	2.7	3.66	5.15	4.15	2.76	2.55	3.08	0.39	3.19	2.91	3.62	3.55
3.66	2.6	2.7	3.29	3.23	3.07	3.76	3.79	2.7	3.68	5.15	4.17	2.76	2.55	3.1	0.39	3.19	2.91	3.62	3.56
3.66	2.96	2.83	3.29	3.23	3.07	3.92	3.8	2.83	3.69	5.22	4.18	2.77	2.55	3.1	0.39	3.23	2.91	3.74	3.62
3.79	3.04	2.84	3.31	3.37	3.07	3.92	3.81	2.84	3.7	5.22	4.18	2.77	2.55	3.24	0.39	3.23	2.91	3.8	3.64
3.8	3.04	2.99	3.42	3.37	3.31	3.92	3.81	2.85	3.7	5.22	4.18	2.78	2.57	3.35	0.43	3.23	2.91	3.8	3.65
3.87	3.04	3.08	3.43	3.37	3.32	3.98	3.87	2.97	3.99	5.44	4.26	2.78	2.57	3.38	0.43	3.24	2.91	3.83	3.66
3.87	3.04	3.18	3.49	3.38	3.33	4.09	3.88	2.98	4.06	5.44	4.3	2.81	2.57	3.41	0.45	3.44	2.91	4.12	3.67

3.88	3.06	3.18	3.49	3.38	3.33	4.09	3.9	2.98	4.07
3.88	3.07	3.18	3.52	3.45	3.36	4.09	3.9	2.98	4.08
3.94	3.09	3.43	3.6	3.53	3.36	4.09	4.06	2.99	4.09
3.98	3.41	3.43	3.69	3.54	3.49	4.09	4.06	2.99	4.11
3.98	3.41	3.57	3.81	3.69	3.63	4.23	4.12	3	4.11
4.04	3.45	3.76	3.85	3.69	3.63	4.23	4.28	3	4.11
4.12	3.62	3.82	3.89	3.74	3.8	4.47	4.28	3.01	4.11
4.23	3.62	3.88	4.02	3.87	3.8	4.47	4.39	3.17	4.11
4.33	3.62	4.12	4.11	3.88	3.82	4.47	4.52	3.18	4.11
4.33	3.78	4.12	4.11	3.97	3.92	4.47	4.66	3.4	4.11
4.33	3.79	4.12	4.17	4.02	4.07	4.47	4.67	3.4	4.19
4.33	3.83	4.35	4.33	4.03	4.13	4.5	4.78	3.47	4.32
4.33	3.86	4.37	4.33	4.16	4.2	4.5	4.78	3.48	4.32
4.65	3.86	4.37	4.41	4.2	4.23	4.62	4.87	3.48	4.37
4.92	4.06	4.37	4.49	4.21	4.46	4.66	4.91	3.56	4.49
4.93	4.23	4.73	4.5	4.21	4.57	4.8	4.95	3.66	4.62
4.96	4.26	4.73	4.73	4.28	4.61	4.85	5.1	3.66	4.62
5.11	4.26	4.73	4.73	4.33	4.62	4.89	5.18	3.67	4.62
5.11	4.26	5.09	4.73	4.33	4.68	5.04	5.18	3.67	4.84
5.11	4.51	5.09	4.86	4.42	4.71	5.04	5.24	3.83	4.85
5.23	4.51	5.09	4.92	4.44	4.74	5.22	5.3	3.83	5.06
5.24	4.51	5.09	4.93	4.45	4.76	5.27	5.3	3.83	5.12
5.24	4.51	5.12	5	4.48	4.76	5.33	5.47	3.98	5.13
5.24	4.51	5.2	5.17	4.57	4.76	5.38	5.48	4.11	5.19
5.24	4.51	5.5	5.24	4.57	4.76	5.41	5.61	4.11	5.22
5.24	4.51	5.5	5.26	4.67	4.84	5.5	5.65	4.12	5.34
5.53	4.59	5.5	5.26	4.68	4.84	5.54	5.72	4.12	5.35
5.53	4.67	5.54	5.26	4.78	4.84	5.54	5.72	4.19	5.42
5.58	4.95	5.82	5.27	4.79	4.84	5.6	5.72	4.19	5.55
5.71	4.97	5.82	5.27	4.79	5.14	5.69	5.79	4.33	5.58
5.71	4.97	5.9	5.27	4.91	5.14	5.74	5.79	4.49	5.62
5.71	4.97	5.96	5.45	4.97	5.14	5.75	5.81	4.49	5.62
6.08	4.97	5.96	5.49	5.01	5.15	5.75	5.82	4.49	5.63
6.09	5.08	5.96	5.49	5.06	5.54	5.87	5.92	4.49	5.64
6.1	5.21	5.96	5.49	5.06	5.54	6.29	5.93	4.5	5.65
6.11	5.28	5.96	5.49	5.06	5.54	6.5	6.13	4.5	5.65
6.11	5.28	6.18	5.49	5.06	5.54	6.67	6.13	4.5	5.79
6.12	5.29	6.18	5.63	5.23	5.58	6.67	6.14	4.5	5.84
6.25	5.4	6.18	5.76	5.24	5.58	6.67	6.15	4.51	5.86
6.26	5.4	6.18	5.77	5.24	5.58	6.67	6.16	4.88	5.86
6.26	5.4	6.18	5.78	5.25	5.58	6.67	6.16	4.88	5.87
6.37	5.43	6.46	5.78	5.33	5.58	6.67	6.17	4.89	5.87
6.51	5.49	6.56	5.78	5.33	5.58	6.67	6.18	4.89	5.87
6.51	5.5	6.56	5.79	5.34	5.64	6.67	6.34	4.89	5.88
6.51	5.51	6.56	5.8	5.34	5.64	6.67	6.34	4.89	5.91
6.51	5.62	6.93	5.81	5.55	5.64	6.74	6.35	4.89	5.91
6.51	5.63	6.93	5.96	5.56	5.64	6.88	6.35	4.9	5.91
6.52	5.63	6.93	5.96	5.56	5.64	7.13	6.41	4.9	5.91
6.55	5.68	7.04	5.99	5.56	5.77	7.37	6.48	4.9	5.91
6.56	5.68	7.04	6.01	5.57			6.48	4.91	6.15
6.57	5.68	7.17	6.01	5.57			6.49	4.91	6.18
6.57	5.68	7.17	6.05	5.62			6.54	4.91	6.19
6.58	5.69	7.18	6.07	5.62			6.54	5.07	6.28
6.58	5.69	7.18	6.08	5.63			6.55	5.07	6.32
6.59	5.69	7.18	6.08				6.55	5.08	6.32
6.59	5.7	7.32	6.09				6.55	5.08	6.36
6.6	5.7	7.33	6.09				6.63	5.08	6.37
6.83	5.7	7.6	6.25				6.63	5.09	6.37
6.84	5.71	7.72	6.31				6.66	5.09	6.37
6.85	5.74	7.72	6.37				6.69	5.09	6.62
6.85	5.84	7.72	6.38				6.69	5.22	6.63
6.91	5.85	7.73	6.49				6.77	5.29	6.63
6.92	5.85	7.73	6.58				6.77	5.36	6.63
6.93	5.85	7.91	6.63				6.78	5.36	6.67
6.93	5.91	7.91	6.71				6.82	5.36	6.72
6.94	5.92	7.99	6.74				6.82	5.37	6.76
6.95	5.92	8.08	6.75				6.94	5.37	6.76
6.97	6	8.15	7				7.05	5.37	6.77
7.16	6.35	8.21	7.01				7.11	5.38	6.8
7.16	6.36	8.21	7.1				7.21	5.38	6.87
7.16	6.47	8.21	7.2				7.21	5.39	6.87
7.44	6.58	8.46	7.22				7.22	5.39	6.88
7.45	6.59	8.53	7.31				7.37	5.4	6.88
7.47	6.6	8.63	7.39				7.5	5.4	7.03
7.54	6.79	8.8	7.39				7.51	5.4	7.09
7.55	6.91	8.8	7.42				7.55	5.57	7.2
7.72	7.02	8.8	7.5				7.6	5.57	7.22
8.09	7.02	8.91	7.5				7.64	5.66	7.31
8.09	7.02	8.91	7.54				7.7	5.79	7.33
8.09	7.02	8.93	7.57				7.9	5.79	7.37
8.1	7.16	9.16	7.7				8.04	5.98	7.39
8.11	7.17	9.27	7.75				8.06	6.11	7.51
8.11	7.17	9.28	7.76				8.06	6.12	7.51
8.52	7.17	9.29	7.83				8.1	6.12	7.51
8.52	7.56	9.56	8.07				8.11	6.13	7.6
8.53	7.57	9.56	8.09				8.12	6.13	7.7
8.54	7.57	9.56	8.1				8.14	6.21	7.75
8.6	7.58	9.56	8.11				8.32	6.22	7.75
8.6	7.58	9.56	8.11				8.43	6.31	7.84
8.61	7.71	9.56	8.28				8.43	6.48	8.01
9.16	7.71	9.56	8.29				8.59	6.56	8.01
9.74	7.72	9.56	8.35				8.59	6.57	8.01
9.9	7.72	9.8	8.39				8.63	6.57	8.13
9.91	7.72	9.93	8.47				8.63	6.58	8.23
9.92	7.86	9.93	8.58				8.63	6.58	8.23
9.93	7.86	10.05	8.63				8.86	6.69	8.28
9.93	7.98	10.05	8.65				8.86	6.7	8.28
9.94	7.99	10.05	8.72				8.87	6.7	8.28
10.15	8.06	10.05	8.77				8.87	7.14	8.32
10.33	8.06	10.22	8.77				9.11	7.14	8.43
10.34	8.06	10.33	8.77				9.15	7.14	8.43
10.34	8.26	10.33	8.79				9.15	7.15	8.65
10.35	8.38	10.33	8.79				9.15	7.15	8.69
10.67	8.39	10.38	8.87				9.15	7.15	8.72
10.68	8.39	10.8	8.87				9.29	7.15	8.82
10.68	8.39	10.8	8.93				9.29	7.15	8.84
10.69	8.4	10.8	8.93				9.35	7.16	8.86
10.69	8.47	10.8	8.98				9.35	7.27	8.92
10.7	8.47	10.8	8.99				9.36	7.28	8.95
10.71	8.47	10.8	8.99				9.43	7.28	9.01
10.71	8.47	10.8	9				9.44	7.29	9.01
10.81	8.48	10.82	9.12				9.48	7.29	9.06
10.82	8.48	10.87	9.13				9.57	7.29	9.15
10.83	8.49	10.87	9.13				9.58	7.31	9.17

5.44	4.31	2.82	2.62	3.41	0.51	3.5	2.91	4.12	3.87
5.5	4.31	2.82	2.63	3.41	0.54	3.5	3.27	4.12	3.87
5.5	4.42	2.83	2.69	3.42	0.54	3.51	3.27	4.12	3.87
5.61	4.42	2.83	2.69	3.48	0.54	3.56	3.27	4.12	3.93
5.61	4.61	2.93	2.91	3.58	0.58	3.65	3.44	4.19	3.93
5.88	4.73	3.05	2.91	3.58	0.58	3.65	3.62	4.22	4.07
5.88	4.93	3.08	3.1	3.71	0.58	3.66	3.62	4.22	4.13
5.88	4.93	3.19	3.1	3.86	0.58	3.66	3.62	4.22	4.14
6.07	5.08	3.26	3.15	3.87	0.58	3.77	3.62	4.43	4.48
6.07	5.08	3.32	3.21	3.87	0.58	3.88	3.87	4.43	4.48
6.19	5.1	3.47	3.32	3.87	0.58	3.88	3.87	4.43	4.48
6.31	5.37	3.51	3.47	3.87	0.58	3.88	3.87	4.43	4.48
6.31	5.45	3.54	3.47	4.01	0.58	3.89	3.99	4.56	4.48
6.38	5.45	3.54	3.59	4.04	0.58	3.89	3.99	4.7	4.48
6.42	5.75	3.54	3.64	4.25	0.58	4.08	3.99	4.79	4.65
6.42	5.76	3.55	3.65	4.27	0.58	4.09	3.99	4.79	4.74
6.42	5.88	3.63	3.65	4.27	0.58	4.15	3.99	4.79	4.76
6.42	5.91	3.75	3.65	4.3	0.58	4.15	3.99	5.13	4.81
6.44	5.95	3.86	3.68	4.42	0.58	4.16	3.99	5.13	4.93
6.77	5.95	3.96	3.68	4.42	0.58	4.25	3.99	5.13	5.13
6.77 .		4.04	3.69	4.57	0.58	4.37	3.99	5.21	5.16
6.77 .		4.05	3.98	4.57	0.58	4.47	3.99	5.56	5.17
6.77 .		4.05	3.98	4.57	0.59	4.56	3.99	5.66	5.29
6.77 .		4.08	3.98	4.57	0.59	4.56	3.99	5.66	5.45
6.9 .		4.1	4.03	4.79	0.59	4.56	3.99	5.66	5.46
7 .		4.11	4.04	4.79	0.59	4.56	4.45	5.66	5.46
7 .		4.17	4.06	4.79	0.59	4.83	4.51	5.66	5.46
7.12 .		4.44	4.06	4.79	0.59	4.84	4.51	5.69	5.55
7.13 .		4.5	4.06	4.79	0.59	4.89	4.51	5.69	5.55
7.25 .		4.52	4.06	5.03	0.59	4.93	4.64	5.69	5.7
7.32 .		4.56	4.06	5.08	0.59	4.97	4.64	5.69	5.83
7.32 .		4.57	4.15	5.08	0.59	5.01	4.8	5.69	5.84
7.36 .		4.57	4.36	5.16	0.59	5.05	4.8	5.69	5.84
7.41 .		4.57	4.39	5.2	0.62	5.09	4.8	5.69	5.99
7.41 .		4.74	4.55	5.2	0.62	5.13	4.8	5.87	6
7.41 .		4.74	4.55	5.4	0.62	5.17	4.8	5.93	6
7.71 .		4.74	4.55	5.4	0.62	5.21	4.8	6	6.17
7.81 .		4.74	4.55	5.43	0.62	5.25	4.8	6	6.18
7.87 .		4.75	4.63	5.85	0.62	5.29	4.8	6	6.18
7.87 .		4.75	4.63	5.85	0.62	5.33	4.8	6.07	6.19
7.87 .		4.75	4.63	5.85	0.62	5.37	4.8	6.08	6.26
7.87 .		4.95	4.64	5.85	0.62	5.41	4.8	6.08	6.31
7.87 .		4.95	4.87	5.85	0.62	5.45	4.8	6.23	6.31
7.87 .		5.02	4.87	5.85	0.62	5.49	4.91	6.23	6.31
7.87 .		5.02	4.87	5.85	0.62	5.53	4.91	6.23	6.35
7.87 .		5.03	4.96	5.85	0.62	5.57	5.05	6.23	6.36
7.87 .		5.03	4.97	5.85	0.62	5.61	5.21	6.23	6.37
7.87 .		5.03	4.97	5.85	0.62	5.65	5.3	6.23	6.37
7.87 .		5.03	4.97	5.85	0.62	5.69	5.3	6.23	6.38
7.87 .		5.04	4.98			5.73	5.71	6.48	6.38
7.87 .		5.04	4.99			5.77	5.76	6.48	6.56
7.87 .		5.05	4.99			5.81	5.76	6.48	6.56
7.87 .		5.05	4.99			5.85	5.76	6.48	6.57
7.89 .		5.13	5.15			5.89	5.76	6.48	6.58
7.89 .		5.13				5.92	5.76	6.48	6.58
7.97 .		5.13				5.94	5.76	6.49	6.58
7.97 .		5.14				5.97	5.76	6.49	6.59
7.98 .		5.14				5.99	5.76	6.51	6.74
7.98 .		5.15				6.02	5.76	6.52	6.75
7.98 .		5.19				6.04	5.76	6.54	6.76
8.3 .		5.2				6.07	5.76	6.64	6.76
8.31 .		5.2				6.09	5.76	6.64	6.88
8.33 .		5.2				6.12	5.76	6.65	6.96
8.48 .		5.2				6.14	5.76	6.65	6.97
8.57 .		5.33				6.17	5.76	6.65	6.97
8.63 .		5.47				6.17	5.76	6.65	6.97
8.65 .		5.47				6.17	5.76	6.65	6.98
8.65 .		5.48				6.17	5.76	6.65	7.05
8.65 .		5.59				6.17	5.76	6.75	7.15
8.77 .		5.6				6.18	5.76	6.82	7.29
8.91 .		5.73				6.18	5.76	6.82	7.3
8.96 .		5.74				6.37	5.77	6.84	7.33
9.1 .		5.78				6.42	5.9	7.16	7.47
9.12 .		5.79				6.48	6.04	7.18	7.47
9.63 .		5.97				6.48	6.05	7.2	7.5
9.64 .		6.14				6.48	6.23	7.23	7.75
9.64 .		6.17				6.6	6.91	7.3	7.84
9.64 .		6.17				6.61	6.91	7.36	7.84
9.76 .		6.17				6.61	6.91	7.36	7.93
9.89 .		6.42				6.61	6.91	7.37	7.93
9.95 .		6.59				6.62	6.91	7.38	7.93
9.96 .		6.59				6.62	6.91	7.4	8.13
10.15 .		6.59				6.73	6.91	7.41	8.31
10.15 .		6.61				6.74	6.91	7.42	8.39
10.15 .		6.69				6.88	6.91	7.42	8.4
10.15 .		6.82				6.96	6.91	7.42	8.58
10.4 .		6.84				6.98	7.25	7.42	8.59
10.4 .		6.85				6.99	7.28	7.42	8.62
10.42 .		6.86				6.99	7.65	7.44	8.63
10.43 .		6.86				6.99	7.85	7.44	8.8
10.43 .		6.87				7.16	7.88	7.44	8.85
10.47 .		6.95				7.17	7.88	7.45	8.95
10.65 .		7.13				7.23	7.88	7.46	9.02
10.65 .		7.13				7.29	7.91	7.5	9.02
10.65 .		7.13				7.29	7.93	7.75	9.11
10.65 .		7.14				7.3	8.26	7.76	9.2
10.65 .		7.14				7.36	8.3	7.76	9.23
10.65 .		7.15				7.43	8.3	7.76	9.23
10.65 .		7.17				7.48	8.3	7.76	9.29
10.91 .		7.17				7.48	8.3	7.76	9.3
10.91 .		7.18				7.48	8.3	7.79	9.31
10.91 .		7.18				7.48	8.3	7.82	9.32
10.91 .		7.25				7.66	8.3	7.82	9.33
10.91 .		7.54				7.67	8.3	7.83	9.33
10.94 .		7.54				7.67	8.37	8.02	9.49
10.94 .		7.54				7.74	8.43	8.13	9.78
10.94 .		7.54				7.74	8.43	8.13	9.78
10.94 .		7.54				7.74	8.44	8.13	9.78
10.94 .		7.55				7.74	8.44	8.13	9.78
10.94 .		7.55				7.74	8.44	8.13	9.78
11.1 .		7.56				7.75	8.45	8.13	9.78
11.11 .		7.56				7.95	8.45	8.13	9.78
11.12 .		7.6				8.01	8.45	8.13	9.78
11.12 .		7.6				8.02	8.46	8.13	

0	0	0	0	0	0	0	0	0	0
0.11	0.04	0.13	0.02	0	0	0	0	0	0
0.13	0.05	0.14	0.02	0.01	0	0	0	0.02	0
0.13	0.06	0.23	0.06	0.01	0	0	0.03	0.02	0
0.13	0.13	0.23	0.18	0.02	0	0	0.13	0.02	0
0.19	0.14	0.35	0.2	0.02	0	0	0.15	0.21	0
0.3	0.15	0.35	0.3	0.03	0	0	0.15	0.21	0
0.38	0.19	0.35	0.31	0.03	0	0.03	0.26	0.21	0.12
0.38	0.25	0.35	0.33	0.04	0.01	0.46	0.32	0.29	0.13
0.44	0.26	0.47	0.41	0.04	0.01	0.46	0.38	0.29	0.14
0.45	0.26	0.48	0.41	0.05	0.01	0.46	0.39	0.38	0.23
0.45	0.27	0.48	0.58	0.06	0.04	0.46	0.4	0.4	0.31
0.47	0.45	0.58	0.61	0.2	0.06	0.46	0.55	0.41	0.31
0.64	0.46	0.67	0.61	0.21	0.07	0.46	0.59	0.43	0.31
0.66	0.47	0.67	0.75	0.26	0.08	0.46	0.62	0.45	0.32
0.71	0.53	0.75	0.8	0.36	0.13	0.47	0.62	0.61	0.33
0.89	0.71	0.77	0.8	0.4	0.33	0.62	0.78	0.65	0.42

0	0	0	0	0	0	0	0	0	0
0	0.1	0.01	0	0.01	0.01	0.01	0.02	0	0
0	0.1	0.06	0	0.01	0.15	0.01	0.02	0	0
0.05	0.1	0.11	0	0.11	0.15	0.02	0.02	0	0
0.09	0.21	0.16	0	0.11	0.15	0.02	0.02	0.02	0
0.29	0.23	0.16	0	0.2	0.21	0.03	0.02	0.02	0
0.29	0.23	0.24	0.07	0.21	0.23	0.03	0.02	0.22	0
0.3	0.29	0.28	0.07	0.27	0.3	0.04	0.02	0.22	0.04
0.42	0.3	0.33	0.07	0.3	0.33	0.11	0.02	0.29	0.04
0.45	0.47	0.38	0.07	0.31	0.35	0.11	0.02	0.29	0.04
0.53	0.47	0.38	0.07	0.35	0.39	0.24	0.02	0.39	0.04
0.64	0.58	0.48	0.07	0.36	0.42	0.24	0.02	0.43	0.04
0.69	0.65	0.52	0.19	0.41	0.42	0.24	0.31	0.5	0.04
0.74	0.65	0.58	0.19	0.59	0.43	0.32	0.4	0.59	0.04
0.77	0.65	0.71	0.26	0.79	0.54	0.42	0.4	0.6	0.04
0.94	0.72	0.71	0.33	0.94	0.99	0.43	0.4	0.65	0.04
0.98	0.8	0.73	0.33	1.02	0.99	0.49	0.4	0.76	0.04

0.95	0.82	0.86	0.8	0.61	0.33	0.64	0.8	0.65	0.43
0.95	0.83	0.93	1.04	0.62	0.34	0.64	0.83	0.65	0.51
0.99	0.93	0.99	1.04	0.66	0.34	0.64	0.95	0.67	0.52
1	1.15	1	1.04	0.75	0.52	0.77	0.99	0.91	0.57
1	1.16	1.11	1.08	0.77	0.71	0.82	1.01	0.94	0.65
1.28	1.16	1.16	1.1	0.78	0.78	1.05	1.12	0.95	0.66
1.54	1.16	1.23	1.19	0.88	0.82	1.05	1.23	0.95	0.67
1.54	1.16	1.23	1.22	0.91	0.94	1.05	1.24	1.07	0.73
1.57	1.28	1.29	1.22	0.91	1.09	1.05	1.25	1.13	0.79
1.57	1.29	1.43	1.31	0.97	1.12	1.09	1.29	1.14	0.88
1.79	1.45	1.54	1.46	1.08	1.24	1.17	1.44	1.24	0.94
1.81	1.46	1.63	1.55	1.13	1.24	1.17	1.46	1.38	1.02
2.3	1.46	1.63	1.61	1.15	1.24	1.28	1.58	1.47	1.08
2.4	1.62	1.73	1.79	1.15	1.24	1.35	1.62	1.52	1.08
2.43	1.62	1.75	1.8	1.15	1.24	1.49	1.62	1.52	1.19
2.44	1.62	1.75	1.8	1.16	1.24	1.61	1.64	1.54	1.29
2.49	1.62	1.93	1.88	1.32	1.24	1.64	2.23	1.67	1.3
2.52	1.64	1.95	2.07	1.32	1.44	1.77	2.23	1.67	1.36
2.56	1.65	1.95	2.07	1.32	1.44	1.77	2.23	1.76	1.41
2.56	1.78	2.04	2.11	1.42	1.44	1.82	2.23	1.78	1.42
2.58	1.79	2.04	2.24	1.43	1.47	1.88	2.23	1.82	1.59
2.61	1.85	2.04	2.25	1.43	1.47	1.9	2.23	1.82	1.65
2.61	1.86	2.21	2.33	1.56	1.47	1.97	2.23	1.82	1.65
2.79	2.01	2.3	2.33	1.59	1.47	2.06	2.23	1.94	1.75
2.8	2.01	2.39	2.48	1.62	1.47	2.06	2.23	2.14	1.76
2.93	2.01	2.4	2.68	1.73	1.49	2.15	2.23	2.15	1.96
3.05	2.19	2.4	2.68	1.73	1.5	2.23	2.29	2.15	1.96
3.05	2.2	2.4	2.73	1.73	1.53	2.35	2.34	2.25	1.97
3.05	2.2	2.53	2.73	1.73	1.53	2.72	2.44	2.3	1.97
3.05	2.2	2.54	2.73	1.73	1.53	2.72	2.46	2.38	2.1
3.05	2.2	2.54	2.73	1.73	1.66	2.72	2.46	2.39	2.1
3.11	2.2	2.72	2.89	1.73	1.86	2.72	2.64	2.39	2.1
3.11	2.34	2.72	2.91	1.83	1.98	2.72	2.65	2.39	2.11
3.18	2.34	2.73	2.99	1.84	1.98	2.72	2.72	2.44	2.32
3.3	2.34	2.94	2.99	1.95	1.98	3.15	2.72	2.44	2.33
3.3	2.44	3	2.99	1.95	1.98	3.15	2.72	2.44	2.33
3.32	2.44	3.01	2.99	1.96	2.11	3.17	2.72	2.56	2.34
3.32	2.44	3.01	3.13	1.96	2.11	3.17	2.75	2.6	2.41
3.32	2.46	3.19	3.24	1.96	2.11	3.17	2.84	2.6	2.41
3.46	2.46	3.19	3.24	2.02	2.11	3.17	2.84	2.71	2.58
3.47	2.47	3.19	3.24	2.03	2.11	3.17	2.84	2.71	2.59
3.47	2.47	3.31	3.24	2.03	2.11	3.17	2.85	2.71	2.59
3.57	2.55	3.35	3.29	2.04	2.11	3.17	2.86	2.71	2.6
3.6	2.55	3.37	3.3	2.08	2.11	3.28	2.86	2.71	2.6
3.62	2.56	3.37	3.38	2.09	2.11	3.28	2.98	2.72	2.6
3.62	2.56	3.37	3.38	2.18	2.11	3.28	3	2.72	2.61
3.72	2.57	3.37	3.38	2.18	2.11	3.28	3	2.72	2.61
3.74	2.57	3.37	3.51	2.19	2.16	3.28	3.08	2.82	2.62
3.74	2.73	3.37	3.55	2.19	2.18	3.54	3.09	2.87	2.62
3.75	2.73	3.55	3.55	2.31	2.25	3.79	3.09	2.87	2.8
3.76	2.73	3.55	3.57	2.41	2.25	3.79	3.12	2.88	2.8
3.88	2.8	3.55	3.6	2.42	2.25	3.79	3.25	2.88	2.8
3.9	2.88	3.65	3.68	2.42	2.25	3.79	3.28	3.05	2.81
3.97	2.88	3.75	3.68	2.43	2.25	3.79	3.36	3.05	2.86
3.99	2.9	3.76	3.78	2.43	2.35	3.79	3.36	3.06	2.95
3.99	2.97	3.76	3.84	2.44	2.47	3.79	3.36	3.15	3.04
4.01	2.97	3.77	3.84	2.44	2.48	3.79	3.47	3.15	3.12
4.22	2.98	3.86	3.95	2.44	2.48	3.79	3.53	3.22	3.13
4.33	2.99	3.87	3.95	2.51	2.5	3.79	3.53	3.4	3.13
4.33	2.99	3.99	3.95	2.7	2.5	3.79	3.67	3.42	3.2
4.33	3.12	4	3.98	2.77	2.6	3.79	3.77	3.44	3.21
4.43	3.35	4.05	4.11	2.81	2.6	3.79	3.8	3.44	3.24
4.55	3.35	4.05	4.25	2.89	2.7	3.85	3.88	3.49	3.25
4.59	3.43	4.05	4.35	2.95	2.74	3.85	3.97	3.56	3.26
4.72	3.43	4.08	4.45	3.02	2.77	4.03	4.08	3.71	3.38
4.73	3.5	4.11	4.56	3.08	2.79	4.03	4.09	3.72	3.39
4.8	3.5	4.11	4.64	3.13	2.91	4.03	4.18	3.75	3.53
4.83	3.6	4.33	4.64	3.14	3.15	4.03	4.25	3.85	3.64
4.83	3.6	4.34	4.64	3.26	3.18	4.12	4.31	3.9	3.66
4.95	3.68	4.34	4.71	3.33	3.18	4.21	4.45	3.9	3.82
4.97	3.72	4.43	4.82	3.41	3.18	4.21	4.49	4	3.9
5	3.86	4.65	4.93	3.45	3.27	4.28	4.64	4.04	3.91
5.06	4.05	4.76	4.93	3.46	3.42	4.4	4.66	4.32	3.91
5.26	4.05	4.87	5	3.56	3.44	4.4	4.71	4.55	4.13
5.26	4.05	4.96	5.12	3.57	3.58	4.85	4.9	4.55	4.13
5.27	4.14	4.98	5.17	3.6	3.58	4.87	4.94	4.55	4.14
5.69	4.15	5.16	5.39	3.71	3.69	4.87	5	4.55	4.34
5.72	4.22	5.24	5.43	3.75	3.74	4.87	5	4.55	4.34
5.72	4.22	5.24	5.43	3.75	3.75	4.87	5.1	4.55	4.34
5.73	4.46	5.37	5.63	3.81	3.78	4.87	5.12	4.55	4.44
6.15	4.46	5.37	5.63	3.9	3.8	5.09	5.22	4.58	4.5
6.17	4.51	5.43	5.64	3.9	3.8	5.09	5.31	4.58	4.5
6.19	4.51	5.44	5.83	4.06	3.88	5.12	5.36	4.68	4.61
6.49	4.51	5.44	5.86	4.07	3.92	5.12	5.44	5.09	4.72
6.51	4.56	5.44	6.02	4.11	3.97	5.34	5.51	5.09	4.86
6.51	4.56	5.51	6.02	4.18	4.07	5.4	5.52	5.09	4.87
6.56	4.56	5.54	6.02	4.19	4.07	5.42	5.53	5.09	4.87
6.61	4.58	5.54	6.1	4.22	4.1	5.42	5.71	5.09	4.96
6.61	4.72	5.88	6.1	4.22	4.17	5.42	5.75	5.09	5.01
6.79	4.81	5.88	6.38	4.38	4.28	5.52	5.83	5.09	5.11
6.86	4.82	5.88	6.38	4.46	4.4	5.52	5.9	5.09	5.15
6.93	4.87	5.88	6.44	4.47	4.56	5.52	5.91	5.15	5.23
6.93	5.08	5.88	6.48	4.47	4.58	5.77	6.07	5.15	5.24
7.09	5.14	6.1	6.52	4.47	4.58	5.77	6.2	5.17	5.34
7.15	5.15	6.1	6.61	4.47	4.58	5.77	6.2	5.41	5.34
7.15	5.15	6.11	6.69	4.58	4.65	5.77	6.21	5.42	5.34
7.15	5.15	6.11	6.78	4.67	4.65	5.98	6.23	5.42	5.44
7.15	5.15	6.11	6.78	4.67	4.65	6.22	6.23	5.42	5.44
7.15	5.15	6.11	6.78	4.75	4.8	6.22	6.23	5.43	5.59
7.2	5.16	6.31	6.92	4.76	4.8	6.66	6.23	5.52	5.66
7.21	5.26	6.31	6.93	4.83	4.8	6.75	6.23	5.52	5.66
7.23	5.26	6.38	6.95	4.83	4.8	6.87	6.24	5.52	5.67
7.23	5.26	6.38	7.02	4.84	4.8	6.96	6.28	5.59	5.67
7.25	5.3	6.38	7.02	4.87	5.03	6.96	6.36	5.6	5.67
7.25	5.3	6.67	7.03	4.89	5.03	6.96	6.43	5.66	5.94
7.25	5.31	6.67	7.13	4.9	5.03	6.96	6.43	5.66	5.94
7.34	5.39	6.81	7.13	4.9	5.17	6.96	6.44	5.79	5.94
7.36	5.39	6.83	7.26	4.9	5.17	6.96	6.44	5.79	5.94
7.38	5.39	6.9	7.27	4.91	5.17	6.96	6.52	5.79	5.95
7.38	5.4	6.91	7.37	4.91	5.17	6.96	6.52	5.79	5.95
7.51	5.46	6.91	7.4	4.92	5.17	6.96	6.53	5.84	6.05
7.51	5.46	6.95	7.4	4.92	5.17	6.96	6.53	5.87	6.05
7.51	5.47	7.02	7.4	5.05	5.19	6.96	6.6	5.96	6.2
7.51	5.47	7.03	7.4	5.05	5.19	6.96	6.61	5.97	6.24
7.51	5.47	7.03	7.49	5.05	5.2	6.96	6.61	5.98	6.24
7.52	5.47	7.03	7.49	5.12	5.2	6.96	6.61	5.99	6.24
7.52	5.48	7.03	7.5	5.13	5.4	6.96	6.67	6.01	6.26
7.54	5.6	7.31	7.55	5.19	5.4	6.96	6.67	6.01	6.29
7.54	5.61	7.33	7.55	5.19	5.4	6.96	6.74	6.09	6.3
7.67	5.61	7.33	7.63	5.3	5.54	6.96	6.74	6.09	6.58
7.74	5.61	7.34	7.67	5.38	5.54	6.96	6.8	6.1	6.62
7.75	5.62	7.46	7.76	5.38	5.54	6.98	6.81	6.19	6.62
7.75	5.76	7.46	7.79	5.38	5.54	7.06	6.94	6.19	6.63
7.76	5.84	7.47	7.79	5.38	5.54	7.06	6.95	6.24	6.68
7.78	5.93	7.5	7.92	5.39	5.54	7.11	6.96	6.24	6.76
7.78	5.93	7.5	7.95	5.5	5.62	7.11	7.02	6.25	6.78
7.88	6.05	7.58	8.01	5.65	5.68	7.12	7.12	6.26	6.78
8	6.16	7.58	8.02	5.68	5.78	8.1	7.21	6.4	6.78
8.12	6.37	7.72	8.25	5.76	5.95	8.1	7.34	6.49	6.78
8.12	6.38	7.72	8.39	5.85	6.12	8.1	7.4	6.53	6.85
8.16	6.42	7.73	8.41	5.89	6.12	8.1	7.45	6.53	6.99
8.67	6.47	7.95	8.45	5.98	6.17	8.1	7.51	6.53	6.99

0.98	0.82	0.75	0.37	1.05	0.99	0.5	0.4	0.76	0.04
1.06	0.82	0.89	0.57	1.17	0.99	0.52	0.41	0.86	0.04
1.13	0.95	0.97	0.6	1.18	0.99	0.55	0.41	0.87	0.04
1.32	0.96	0.97	0.71	1.19	0.99	0.64	0.46	0.99	0.04
1.38	1.03	1.07	0.71	1.19	0.99	0.72	0.5	1.05	0.04
1.57	1.09	1.11	0.8	1.24	0.99	0.79	0.63	1.05	0.04
1.57	1.26	1.16	0.8	1.25	1.1	0.8	0.63	1.2	0.04
1.57	1.31	1.19	0.8	1.43	1.1	0.8	0.63	1.29	0.04
1.75	1.31	1.27	0.89	1.48	1.12	0.92	0.69	1.36	0.04
1.78	1.36	1.29	0.89	1.51	1.17	0.99	0.75	1.36	0.04
1.79	1.41	1.42	0.91	1.52	1.17	0.99	0.75	1.46	0.04
1.94	1.65	1.52	0.91	1.52	1.17	1	0.75	1.55	0.04
1.94	1.65	1.53	1.12	1.58	1.18	1.08	0.75	1.67	0.04
1.97	1.71	1.57	1.21	1.59	1.26	1.13	0.75	1.67	0.04
2.17	1.8	1.74	1.21	1.59	1.26	1.13	0.75	1.68	0.04
2.18	1.98	1.82	1.21	1.59	1.46	1.17	0.75	1.74	0.04
2.28	2.05	1.82	1.29	1.8	1.46	1.37	0.83	1.75	0.04
2.3	2.05	1.82	1.33	1.95	1.46	1.4	0.92	1.88	0.04
2.3	2.05	1.99	1.33	1.95	1.49	1.41	0.92	2	0.04
2.3	2.14	2.03	1.4	2.1	1.62	1.47	0.98	2	0.04
2.41	2.28	2.07	1.6	2.11	1.78	1.54	0.98	2.03	0.04
2.42	2.28	2.09	1.6	2.11	1.78	1.6	1.05	2.06	0.04
2.42	2.45	2.14	1.61	2.11	1.78	1.61	1.11	2.08	0.04
2.43	2.45	2.24	1.61	2.11	1.78	1.61	1.12	2.18	0.04
2.54	2.45	2.24	1.7	2.26	1.92	1.75	1.14	2.29	0.04
2.71	2.46	2.24	1.86	2.32	1.92	1.82	1.21	2.39	0.04
2.81	2.46	2.35	1.86	2.32	1.92	1.83	1.21	2.39	0.04
2.81	2.65	2.35	1.87	2.33	2.05	1.83	1.21	2.39	0.04
2.83	2.65	2.52	1.92	2.33	2.07	1.9	1.21	2.39	0.04
2.84	2.65	2.57	1.92	2.4	2.08	2.04	1.21	2.39	0.04
2.84	2.65	2.57	1.92	2.4	2.08	2.05	1.21	2.39	0.04
2.84	2.66	2.57	1.92	2.43	2.12	2.05	1.46	2.43	0.04
2.85	2.78	2.57	2.02	2.52	2.2	2.06	1.46	2.45	0.04
3.14	2.78	2.7	2.02	2.53	2.2	2.06	1.46	2.45	0.04
3.14	2.79	2.73	2.02	2.53	2.4	2.13	1.46	2.45	0.04
3.14	2.79	2.73	2.03	2.63	2.45	2.14	1.46	2.53	0.04
3.22	2.86	2.73	2.03	2.75	2.47	2.14	1.46	2.64	0.04
3.22	2.86	2.73	2.1	2.75	2.5	2.21	1.46	2.71	0.04
3.39	3.03	2.73	2.1	2.75	2.5	2.22	1.46	2.93	0.04
3.44	3.03	2.77	2.1	2.76	2.7	2.22	1.46	2.93	0.15
3.45	3.06	2.77	2.15	2.76	3.11	2.22	1.46	2.93	0.16
3.45	3.06	2.79	2.18	2.76	3.11	2.23	1.55	2.93	0.24
3.45	3.19	2.79	2.18	2.82	3.11	2.38	1.55	2.93	0.31
3.6	3.2	3.01	2.18	2.82	3.11	2.38	1.55	3.06	0.32
3.62	3.2	3.01	2.3	2.82	3.11	2.38	1.55	3.06	0.33
3.73	3.27	3.01	2.3	2.92	3.11	2.39	1.55	3.06	0.34
3.73	3.27	3.01	2.3	2.93	3.11	2.39	1.55	3.06	0.39
3.75	3.27	3.01	2.4	2.93	3.11	2.47	1.55	3.06	0.4
3.75	3.28	3.02	2.4	2.94	3.11	2.47	1.57	3.06	0.4
3.9	3.39	3.13	2.42	3.09	3.11	2.48	1.57	3.06	0.49
3.93	3.39	3.13	2.43	3.09	3.11	2.55	1.57	3.06	0.54
3.93	3.39	3.17	2.53	3.1	3.11	2.56	1.59	3.15	0.55
3.94	3.52	3.17	2.56	3.1	3.11	2.56	1.59	3.15	0.67
4.09	3.68	3.18	2.56	3.1	3.11	2.68	1.7	3.15	0.68
4.23	3.68	3.38	2.56	3.29	3.11	2.69	1.7	3.15	0.69
4.25	3.68	3.38	2.56	3.3	3.15	2.69	1.7	3.17	0.75
4.25	3.69	3.52	2.62	3.3	3.3	2.81	1.7	3.29	0.81
4.27	3.69	3.52	2.62	3.33	3.51	2.81	1.7	3.29	0.87
4.33	3.69	3.52	2.62	3.34	3.87	2.82	1.7	3.29	0.88
4.33	3.77	3.52	2.68	3.34	4.02	2.82	1.7	3.33	0.93
4.42	3.87	3.6	2.7	3.42	4.02	3.08	1.79	3.35	1.01
4.57	3.95	3.6	2.9	3.62	4.02	3.08	1.89	3.48	1.02
4.57	3.95	3.66	3.01	3.83	4.02	3.14	1.94	3.67	1.21
4.58	4	3.68	3.01	4.09	4.02	3.14	2.11	3.69	1.32
4.81	4.08	3.7	3.07	4.58	4.02	3.21	2.13	3.72	1.33
4.82	4.15	3.91	3.07	4.58	4.02	3.3	2.18	3.81	1.4
4.85	4.15	4	3.08	4.7	4.02	3.48	2.27	3.91	1.47
4.87	4.32	4	3.08	4.79	4.02	3.48	2.38	3.91	1.5
4.87	4.39	4.08	3.28	4.79	4.02	3.57	2.42	3.93	1.63
4.99	4.39	4.22	3.4	4.83	4.02	3.62	2.48	3.99	1.78
5	4.39	4.32	3.42	4.84	4.02	3.63	2.48	4.04	1.86
5	4.39	4.32	3.42	4.94	4.02	3.63	2.54	4.1	1.87
5.21	4.5	4.4	3.52	4.94	4.26	3.68	2.66	4.16	1.94
5.25	4.59	4.44	3.67	4.94	4.26	3.74	2.68	4.26	2.06
5.34	4.7	4.56	3.72	5.34	4.26	3.79	2.72	4.35	2.06
5.34	4.7	4.73	3.72	5.37	4.26	3.83	2.82	4.35	2.12
5.34	4.78	4.73	3.8	5.38	4.26	3.96	2.82	4.46	2.16
5.4	4.85	4.74	3.8	5.38	4.26	4.11	2.94	4.49	2.21
5.64	4.99	4.89	3.8	5.46	4.29	4.17	3.05	4.58	2.35
5.65	5	4.96	3.83	5.46	4.43	4.22	3.05	4.72	2.61
5.65	5	4.96	3.84	5.46	4.43	4.27	3.1	4.72	2.62
5.76	5.15	4.97	3.84	5.61	4.56	4.28	3.1	4.73	2.62
5.85	5.16	4.97	3.94	5.68	4.6	4.38	3.19	4.78	2.66
5.96	5.16	5.01	4	5.68	4.67	4.49	3.21	4.84	2.75
5.96	5.21	5.14	4	5.68	4.67	4.49	3.21	4.9	2.87
6.11	5.21	5.28	4	5.72	4.73	4.56	3.21	4.94	2.95
6.16	5.33	5.28	4.1	5.78	4.77	4.56	3.28	4.97	2.96
6.16	5.33	5.31	4.15	5.79	4.77	4.63	3.3	5.06	3.1
6.16	5.34	5.31	4.21	5.79	4.77	4.63	3.32	5.18	3.1
6.34	5.34	5.31	4.21	6.25	4.77	4.77	3.34	5.21	3.24
6.39	5.48	5.56	4.3	6.25	4.9	4.78	3.34	5.33	3.34
6.43	5.48	5.67	4.47	6.26	5.03	4.78	3.48	5.33	3.34
6.48	5.48	5.67	4.52	6.42	5.27	4.88	3.48	5.41	3.35
6.49	5.6	5.67	4.52	6.6	5.27	5	3.56	5.43	3.46
6.55	5.63	5.87	4.52	6.61	5.27	5.08	3.64	5.43	3.46
6.59	5.72	5.92	4.6	6.61	5.27	5.12	3.64	5.43	3.54
6.59	5.72	5.93	4.61	6.61	5.27	5.12	3.64	5.43	3.55
6.59	5.78	5.93	4.61	6.61	5.31	5.12	3.64	5.43	3.55
6.63	5.79	5.93	4.74	6.61	5.38	5.13	3.78	5.5	3.55
6.72	5.91	5.93	4.76	6.62	5.43	5.19	3.78	5.73	3.68
6.73	5.92	5.94	4.76	6.62	5.59	5.26	3.78	5.73	3.68
6.74	5.92	5.94	4.81	6.91	5.59	5.27	3.78	5.73	3.69
7	5.93	6	4.92	6.91	5.98	5.28	3.78	5.74	3.69
7.01	6.02	6	4.92	6.92	5.98	5.28	3.78	5.74	3.7
7.01	6.03	6	4.94	6.92	5.98	5.4	3.82	5.74	3.7
7.01	6.03	6	4.94	6.92	5.98	5.4	3.84	5.75	3.91
7.02	6.03	6.12	4.94	6.92	5.98	5.41	3.84	5.79	3.92
7.02	6.04	6.12	5.06	7.03	5.98	5.41	3.84	5.92	3.92
7.02	6.16	6.16	5.07	7.03	6.16	5.42	3.84	5.92	3.93
7.02	6.16	6.16	5.07	7.04	6.16	5.42	3.84	5.92	4
7.02	6.17	6.16	5.07	7.04	6.16	5.42	3.84	5.96	4.01
7.04	6.17	6.16	5.09	7.05	6.16	5.43	3.84	5.96	4.01
7.14	6.18	6.16	5.1	7.05	6.16	5.56	4.03	5.96	4.02
7.14	6.38	6.16	5.1	7.05	6.16	5.57	4.08	5.96	4.03
7.14	6.38	6.29	5.1	7.05	6.24	5.57	4.08	5.96	4.03
7.14	6.44	6.29	5.11	7.06	6.3	5.57	4.08	5.96	4.04
7.14	6.44	6.29	5.11	7.06	6.3	5.57	4.08	5.96	4.04
7.45	6.44	6.29	5.18	7.18	6.3	5.58	4.08	5.96	4.05
7.5	6.45	6.32	5.18	7.18	6.43	5.58	4.25	6.06	4.2
7.5	6.55	6.32	5.34	7.33	6.43	5.83	4.25	6.06	4.21
7.51	6.58	6.32	5.36	7.33	6.66	5.84	4.25	6.06	4.21
7.51	6.67	6.4	5.4	7.33	6.66	5.85	4.3	6.06	4.29
7.65	6.67	6.51	5.41	7.34	6.66	5.86	4.3	6.06	4.3
7.78	6.67	6.58	5.47	7.42	6.66	5.96	4.3	6.06	4.3
7.79	6.67	6.58	5.47	7.42	6.66	5.97	4.38	6.06	4.31
7.85	6.71	6.58	5.55	7.54	6.66	5.97	4.5	6.25	4.41
8.13	6.94	6.61	5.67	7.71	6.66	6.06	4.5	6.34	4.42
8.29	6.94	6.62	5.73	7.71	6.66	6.13	4.61	6.47	4.68
8.29	6.94	6.8	5.82	7.75	6.7	6.18	4.68	6.6	4.68
8.54	6.94	6.87	5.9	7.81	6.7	6.22	4.68	6.6	4.8
8.62	7.1	6.87	5.92	7.92	6.94	6.31	4.83	6.6	4.81
8.62	7.18	6.87	5.98	8.02	7	6.36	4.9	6.62	4.91

8.7	6.63	8.05	8.45	6.06	6.18	8.1	7.7	6.63	7.1	8.68	7.19	6.97	6.15	8.02	7	6.41	5.02	6.81	4.99
8.7	6.7	8.05	8.64	6.13	6.3	8.1	7.76	6.74	7.1	8.68	7.19	7.02	6.19	8.03	7	6.48	5.02	6.89	5.09
8.83	7.04	8.23	8.65	6.18	6.32	8.1	7.86	6.74	7.33	8.68	7.26	7.02	6.33	8.13	7.09	6.54	5.08	6.89	5.09
8.9	7.04	8.31	8.65	6.19	6.55	8.1	8	6.81	7.38	8.68	7.26	7.11	6.36	8.16	7.14	6.61	5.13	6.89	5.18
8.91	7.08	8.32	8.69	6.23	6.78	8.1	8.08	6.93	7.52	8.82	7.26	7.11	6.36	8.22	7.23	6.67	5.15	6.89	5.18
9.12	7.09	8.4	8.82	6.27	6.78	8.1	8.15	7.25	7.53	8.86	7.44	7.2	6.4	8.45	7.32	6.82	5.15	7.03	5.26
9.28	7.2	8.46	8.84	6.28	6.78	8.1	8.21	7.25	7.59	8.98	7.6	7.26	6.45	8.47	7.34	6.82	5.32	7.08	5.3
9.28	7.2	8.6	8.88	6.43	6.78	8.1	8.21	7.25	7.63	9.15	7.6	7.29	6.52	8.61	7.36	6.92	5.48	7.15	5.53
9.28	7.2	8.61	8.94	6.53	6.94	8.15	8.36	7.33	7.7	9.34	7.69	7.34	6.55	8.62	7.37	6.99	5.61	7.28	5.61
9.34	7.41	8.74	9.07	6.53	7.02	8.36	8.47	7.33	7.71	9.36	7.69	7.48	6.57	8.62	7.37	6.99	5.64	7.28	5.62
9.34	7.42	8.74	9.17	6.58	7.09	8.45	8.47	7.33	7.82	9.36	7.8	7.49	6.61	8.63	7.41	6.99	5.72	7.28	5.68
9.35	7.42	8.87	9.24	6.59	7.22	8.5	8.51	7.35	7.91	9.45	7.8	7.5	6.63	8.66	7.46	7.07	5.88	7.33	5.74
9.36	7.42	9.05	9.35	6.72	7.26	8.51	8.62	7.58	7.92	9.6	7.9	7.55	6.78	8.66	7.57	7.11	5.88	7.55	5.88
9.42	7.49	9.05	9.44	6.75	7.26	8.55	8.71	7.6	7.92	9.72	8.02	7.76	6.78	8.66	7.57	7.15	5.88	7.55	6.02
9.54	7.49	9.05	9.53	6.75	7.42	8.61	8.79	7.64	8.01	9.72	8.02	7.93	6.84	8.67	7.59	7.19	5.96	7.55	6.02
9.82	7.49	9.05	9.59	6.92	7.44	8.62	8.87	7.72	8.24	9.8	8.11	7.93	6.87	8.81	7.6	7.33	6.06	7.55	6.09
9.83	7.63	9.19	9.65	6.92	7.44	8.69	8.94	7.75	8.24	10.06	8.12	7.93	6.89	9.04	7.62	7.33	6.25	7.64	6.15
9.94	7.63	9.34	9.78	6.92	7.44	8.8	8.99	7.95	8.32	10.06	8.17	7.93	6.98	9.08	7.83	7.33	6.25	7.89	6.28
10.02	7.63	9.37	9.79	7.01	7.58	8.87	9.11	7.95	8.42	10.06	8.18	8.03	7	9.08	7.86	7.34	6.36	7.89	6.39
10.05	7.75	9.43	9.96	7.02	7.75	9.04	9.18	7.95	8.42	10.06	8.19	8.05	7.02	9.13	7.88	7.56	6.41	7.89	6.4
10.13	7.75	9.43	9.96	7.05	7.82	9.17	9.18	7.95	8.49	10.09	8.49	8.05	7.04	9.14	7.9	7.57	6.41	7.89	6.46
10.14	7.76	9.58	9.97	7.05	7.82	9.26	9.19	8.11	8.51	10.26	8.49	8.14	7.09	9.21	8.1	7.64	6.43	7.89	6.59
10.14	7.76	9.64	9.97	7.07	7.82	9.26	9.26	8.11	8.52	10.43	8.49	8.18	7.15	9.21	8.16	7.73	6.59	8.01	6.62
10.17	7.92	9.66	10.05	7.28	7.95	9.26	9.44	8.11	8.54	10.45	8.54	8.35	7.17	9.22	8.17	7.83	6.59	8.01	6.67
10.6	7.92	9.84	10.05	7.28	7.95	9.26	9.51	8.11	8.62	10.72	8.7	8.38	7.38	9.22	8.18	7.84	6.59	8.01	6.74
10.65	7.93	10.08	10.07	7.31	7.95	9.26	9.51	8.11	8.74	10.73	8.7	8.48	7.41	9.3	8.24	7.89	6.59	8.01	6.88
10.65	8.17	10.16	10.08	7.39	8	9.63	9.54	8.12	8.75	10.73	8.75	8.48	7.43	9.3	8.33	7.93	6.69	8.01	6.9
10.66	8.27	10.16	10.18	7.39	8	9.82	9.54	8.24	8.8	10.73	8.85	8.48	7.49	9.3	8.33	8	6.83	8.01	6.9
10.73	8.27	10.16	10.18	7.39	8	9.97	9.54	8.3	9.03	10.73	8.88	8.48	7.51	9.38	8.33	8.25	7.03	8.01	6.9
10.73	8.27	10.16	10.19	7.39	8	10.15	9.63	8.33	9.08	10.73	8.89	8.49	7.52	9.38	8.37	8.34	7.03	8.16	6.91
10.83	8.27	10.36	10.4	7.39	8	10.15	9.64	8.36	9.08	10.79	8.89	8.5	7.53	9.58	8.5	8.34	7.03	8.31	6.95
10.85	8.27	10.37	10.44	7.39	8	10.15	9.7	8.39	9.11	10.79	9.09	8.5	7.62	9.59	8.51	8.35	7.03	8.31	6.95
10.85	8.28	10.37	10.45	7.39	8	10.15	9.83	8.39	9.11	10.79	9.1	8.67	7.7	9.59	8.6	8.35	7.03	8.31	7.04
10.86	8.29	10.46	10.46	7.5	8	10.15	9.84	8.39	9.21	10.84	9.1	8.67	7.75	9.6	8.6	8.42	7.03	8.31	7.04
10.86	8.29	10.46	10.54	7.51	8.23	10.23	9.89	8.48	9.22	10.84	9.1	8.67	7.83	9.6	8.92	8.42	7.03	8.31	7.12
10.86	8.38	10.46	10.64	7.51	8.23	10.28	9.9	8.48	9.22	10.84	9.11	8.73	7.88	9.61	8.92	8.43	7.1	8.4	7.13
10.94	8.38	10.61	10.64	7.51	8.23	10.67	9.98	8.48	9.22	10.93	9.11	8.73	7.98	9.61	8.92	8.43	7.1	8.4	7.13
10.96	8.38	10.61	10.64	7.61	8.23	10.87	10.02	8.48	9.22	10.99	9.22	8.73	7.98	9.61	8.92	8.55	7.19	8.4	7.26
10.96	8.39	10.67	10.72	7.71	8.23	10.92	10.02	8.56	9.22	11	9.23	8.73	8.11	9.61	8.92	8.55	7.2	8.68	7.31
10.96	8.49	10.8	10.82	7.71	8.23	10.93	10.02	8.64	9.22	11	9.23	8.73	8.11	9.7	8.95	8.55	7.25	8.68	7.31
10.96	8.49	10.8	10.82	7.81	8.23	10.93	10.16	8.64	9.29	11.15	9.24	8.86	8.11	9.7	8.96	8.56	7.25	8.69	7.31
11.03	8.49	10.8	10.82	7.81	8.23	11.01	10.16	8.64	9.3	11.15	9.25	8.86	8.11	9.7	8.97	8.56	7.25	8.69	7.31
11.05	8.49	10.95	10.82	7.81	8.25	11.01	10.16	8.64	9.3	11.27	9.33	8.87	8.11	9.71	9.15	8.57	7.25	8.8	7.36
11.05	8.5	11.05	10.87				10.17	8.64	9.3	11.27	9.34	8.87				8.57	7.25	8.8	7.37
11.05	8.5	11.06	10.87				10.23	8.64	9.49	11.27	9.34	8.87				8.58	7.29	8.81	7.38
11.05	8.51	11.07	10.96				10.23	8.64	9.49	11.27	9.34	8.94				8.81	7.37	8.82	7.39
11.05	8.51	11.07	10.96				10.33	8.71	9.5	11.27	9.35	8.94				8.82	7.37	8.82	7.49
11.16	8.51	11.13	10.99				10.33	8.71	9.66	11.27	9.44	8.94				8.82	7.37	8.94	7.5
11.17	8.52	11.14	10.99				10.34	8.88	9.66	11.3	9.44	8.94				8.89	7.37	8.98	7.51
11.17	8.62	11.22	11.08				10.38	8.91	9.66	11.33	9.51	8.94				8.9	7.37	8.98	7.52
11.19	8.62	11.41	11.09				10.47	8.91	9.66	11.36	9.52	8.95				8.9	7.37	8.98	7.52
11.19	8.62	11.42	11.24				10.47	8.91	9.66	11.38	9.52	8.95				8.91	7.5	8.98	7.53
11.2	8.63	11.42	11.24				10.48	8.91	9.66	11.41	9.53	9.05				8.91	7.5	8.98	7.62
11.24	8.63	11.43	11.25				10.54	9.04	9.75	11.43	9.76	9.06				9.03	7.5	9.06	7.73
11.27	8.64	11.43	11.27				10.62	9.13	9.88	11.53	9.77	9.06				9.03	7.61	9.06	7.74
11.27	8.77	11.59	11.31				10.62	9.17	9.88	11.53	9.77	9.14				9.03	7.66	9.06	7.75
11.28	8.77	11.61	11.41				10.84	9.17	10.07	11.69	9.77	9.15				9.04	7.66	9.06	7.75
11.37	8.88	11.75	11.43				10.84	9.32	10.08	11.69	9.78	9.3				9.15	7.66	9.07	7.76
11.42	8.89	11.75	11.52				10.84	9.32	10.08	11.69	9.78	9.3				9.15	7.7	9.07	7.77
11.43	8.89	11.89	11.69				10.91	9.32	10.24	11.82	9.9	9.3				9.16	7.7	9.07	7.78
11.61	9.16	11.89	11.76				11.03	9.39	10.32	12.03	9.95	9.38				9.24	7.84	9.18	7.97
11.67	9.28	11.94	11.76				11.13	9.47	10.32	12.13	10.07	9.38				9.26	7.96	9.33	8.05
11.67	9.35	12.09	11.83				11.18	9.55	10.38	12.24	10.21	9.44				9.44	7.96	9.39	8.05
11.67	9.43	12.19	11.95				11.24	9.57	10.38	12.24	10.24	9.47				9.55	8.14	9.39	8.09
11.69	9.44	12.38	11.95				11.24	9.64	10.38	12.42	10.24	9.54				9.66	8.2	9.39	8.09
11.79	9.64	12.41	11.95				11.33	9.73	10.42	12.45	10.38	9.59				9.7	8.2	9.43	8.16
11.96	9.88	12.41	11.99				11.43	9.8	10.44	12.45	10.48	9.59				9.81	8.23	9.6	8.26
11.97	9.88	12.42	12.1				11.43	9.88	10.58	12.53	10.48	9.68				9.81	8.4	9.76	8.28
12.01	9.95	12.57	12.12				11.65	9.88	10.58	12.56	10.48	9.88				9.89	8.4	9.79	8.34
12.02	9.95	12.84	12.26				11.8	10.02	10.66	12.73	10.65	9.93				9.99	8.49	9.79	8.47
12.13	9.95	13.02	12.29				11.94	10.05	10.89	12.87	10.65	10.01				10.06	8.5	9.79	8.49
12.28	10.07	13.15	12.38				11.99	10.09	10.98	12.87	10.65	10.01				10.07	8.58	9.79	8.58
12.5	10.08	13.16	12.38				12.03	10.14	10.98	12.96	10.65	10.06				10.17	8.61	9.9	8.64
12.5	10.26	13.16	12.42				12.12	10.22	11.05	12.97	10.74	10.12				10.26	8.61	10.13	8.73
12.52	10.33	13.39	12.56				12.21	10.25	11.15	12.98	10.85	10.26				10.32	8.72	10.13	8.79
12.64	10.33	13.44	12.68				12.21	10.41	11.26	12.98	10.95	10.29				10.39	8.85	10.17	8.94
12.66	10.39	13.5	12.68				12.23	10.43	11.3	12.98	10.96	10.34				10.39	8.98	10.2	8.97
12.86	10.47	13.6	12.74				12.27	10.51	11.36	13.17	10.96	10.59				10.6	9.01	10.32	9.04
12.94	10.47	13.83	12.9				12.27	10.51	11.38	13.43	11.09	10.67				10.6	9.15	10.41	9.13
12.94	10.57	14.03	12.95				12.46	10.53	11.4	13.45	11.09	10.67				10.7	9.15	10.44	9.14
12.94	10.57	14.03	12.95				12.46	10.67	11.4	13.5	11.09	10.74				10.84	9.15	10.49	9.22
12.99	10.67	14.09	12.97				12.51	10.73	11.47	13.56	11.21	10.75				10.86	9.22	10.49	9.28
13.04	10.82	14.15	12.98				12.63	10.76	11.47	13.59	11.21	10.82				10.92	9.3	10.49	9.43
13.06	10.82	14.27	12.98				12.74	11.04	11.72	13.59	11.33	10.88				10.92	9.3	10.5	9.53
13.4	10.82	14.47	13.12				12.77	11.1	11.74	13.67	11.46	10.88				11.07	9.37	10.59	9.62
13.41	10.96	14.47	13.12				12.79	11.1	11.91	13.68	11.52	10.94				11.15	9.46	10.66	9.62
13.44	10.96	14.47	13.25				12.9	11.19	11.97	13.7	11.6	11.11				11.24	9.61	10.86	9.72
13.44	10.96	14.47	13.25				12.98	11.19	11.98	13.73	11.61	11.21				11.33	9.68	10.86	9.82
13.44	10.96	14.56	13.46				13.07	11.28	12.05	13.9	11.68	11.22				11.33	9.68	10.86	9.82
13.57	11.24	14.69	13.46				13.17	11.41	12.23	14.04	11.75	11.22				11.41	9.68	10.87	9.83
13.78	11.24	14.73	13.6				13.21	11.41	12.25	14.24	11.75	11.22				11.56	9.85	10.87	9.83
13.97	11.24	14.89	13.64				13.21	11.41	12.28	14.27	11.79	11.34				11.66	9.93	10.92	9.88
14.01	11.35	14.9	13.64				13.21	11.41	12.28	14.47	11.96	11.56				11.67	9.93	10.98	9.89
14.01	11.35	15.05	13.65				13.21	11.48	12.47	14.5	12.01	11.56				11.67	10.02	10.99	9.89
14.01	11.35	15.06	13.65				13.29	11.49	12.53	14.5	12.01	11.56				11.67	10.2	10.99	9.89
14.01	11.35	15.06	13.73				13.3	11.74	12.65	14.5	12.02	11.56				11.79	10.2	11.02	10.06
14.01	11.35	15.06	13.73				13.36	11.74	12.65	14.55	12.02	11.56				11.79	10.2	11.04	10.07
14.01	11.35	15.18	13.75				13.54	11.75	12.65	14.61	12.02	11.56				11.87	10.2	11.04	10.1
14.17	11.35	15.18	13.76				13.54	11.85	12.67	14.63	12.02	11.56				11.91	10.2	11.06	10.1
14.17	11.35	15.18	13.8				13.59	11.87	12.79	14.63	12.03	11.68				11.94	10.2	11.16	10.25
14.17	11.35	15.18	13.96				13.64	11.9	12.79	14.63	12.03	11.69				12.01	10.24	11.22	10.26
14.18	11.35	15.28	13.97				13.67	11.91	12.79	14.65	12.22	11.82				12.02	10.24	11.22	10.26
14.18	11.35	15.29	13.98				13.68	11.91	12.79	14.69	12.22	11.82				12.02	10.25	11.22	10.27
14.18	11.35	15.29	14.04				13.68	11.91	12.79	14.87	12.23	11.84				12.08	10.31	11.22	10.37
14.19	11.36	15.29	14.06				13.7	11.97	12.89	14.89	12.23	11.92				12.09	10.31	11.22	10.38
14.34	11.54	15.52	14.08				13.84	11.97	12.89	14.89	12.41	11.93				12.26	10.31	11.22	10.38
14.34	11.54	15.52	14.2				13.84	11.98	12.89	14.89	12.41	11.93				12.26	10.31	11.22	10.39
